# Genome-wide association study identifies genetic variants underlying footrot in Portuguese Merino sheep

**DOI:** 10.1186/s12864-023-09844-x

**Published:** 2024-01-23

**Authors:** Daniel Gaspar, Catarina Ginja, Nuno Carolino, Célia Leão, Helena Monteiro, Lino Tábuas, Sandra Branco, Ludovina Padre, Pedro Caetano, Ricardo Romão, Claudino Matos, António Marcos Ramos, Elisa Bettencourt, Ana Usié

**Affiliations:** 1 Centro de Biotecnologia Agrícola E Agro-Alimentar Do Alentejo (CEBAL)/ Instituto Politécnico de Beja (IPBeja), 7801-908 Beja, Portugal; 2 CIBIO, Centro de Investigação em Biodiversidade e Recursos Genéticos, InBIO Laboratório Associado, Universidade do Porto, Campus de Vairão, R. Padre Armando Quintas 7, 4485-661 Vairão, Portugal; 3 BIOPOLIS Program in Genomics, Biodiversity and Land Planning, Campus do Varão, Campus de Vairão, R. Padre Armando Quintas 7, 4485-661 Vairão, Portugal; 4 CIISA, Centro de Investigação Interdisciplinar em Sanidade Animal, Faculdade de Medicina Veterinária, Universidade de Lisboa, Av. Universidade Técnica, 1300-477 Lisboa, Portugal; 5 Instituto Nacional de Investigação Agrária E Veterinária, I.P. (INIAV, I.P.), Avenida da República, Quinta Do Marquês, 2780-157 Oeiras, Portugal; 6 Escola Universitária Vasco da Gama, Av. José R. Sousa Fernandes 197, 3020-210 Lordemão, Coimbra Portugal; 7 MED – Mediterranean Institute for Agriculture, Environment and Development and CHANGE – Global Change and Sustainability Institute, CEBAL – Centro de Biotecnologia Agrícola e Agro-Alimentar do Alentejo, 7801-908 Beja, Portugal; 8 ACOS – Agricultores do Sul, Beja, Portugal; 9 MED—Mediterranean Institute for Agriculture, Environment and Development and CHANGE – Global Change and Sustainability Institute, University of Évora, Polo da Mitra, Ap. 94, 7006-554 Évora, Portugal; 10 Departamento de Medicina Veterinária, Escola de Ciências E Tecnologia, Évora University, Pólo da Mitra Ap. 94, 7002-554 Évora, Portugal

**Keywords:** *Ovis aries*, Portuguese Merino, Footrot, Genome-wide association study

## Abstract

**Background:**

Ovine footrot caused by *Dichelobacter nodosus* (*D. nodosus*) is a contagious disease with serious economic and welfare impacts in sheep production systems worldwide. A better understanding of the host genetic architecture regarding footrot resistance/susceptibility is crucial to develop disease control strategies that efficiently reduce infection and its severity. A genome-wide association study was performed using a customized SNP array (47,779 SNPs in total) to identify genetic variants associated to footrot resistance/susceptibility in two Portuguese native breeds, i.e. Merino Branco and Merino Preto, and a population of crossbred animals. A cohort of 1375 sheep sampled across 17 flocks, located in the Alentejo region (southern Portugal), was included in the analyses.

**Results:**

Phenotypes were scored from 0 (healthy) to 5 (severe footrot) based on visual inspection of feet lesions, following the Modified Egerton System. Using a linear mixed model approach, three SNPs located on chromosome 24 reached genome-wide significance after a Bonferroni correction (*p* < 0.05). Additionally, six genome-wide suggestive SNPs were identified each on chromosomes 2, 4, 7, 8, 9 and 15. The annotation and KEGG pathway analyses showed that these SNPs are located within regions of candidate genes such as the nonsense mediated mRNA decay associated PI3K related kinase (SMG1) (chromosome 24) and the RALY RNA binding protein like (RALYL) (chromosome 9), both involved in immunity, and the heparan sulfate proteoglycan 2 (HSPG2) (chromosome 2) and the Thrombospodin 1 (THBS1) (chromosome 7) implicated in tissue repair and wound healing processes.

**Conclusion:**

This is the first attempt to identify molecular markers associated with footrot in Portuguese Merino sheep. These findings provide relevant information on a likely genetic association underlying footrot resistance/susceptibility and the potential candidate genes affecting this trait. Genetic selection strategies assisted on the information obtained from this study could enhance Merino sheep-breeding programs, in combination with farm management strategies, for a more effective and sustainable long-term solution for footrot control.

**Supplementary Information:**

The online version contains supplementary material available at 10.1186/s12864-023-09844-x.

## Introduction

Ovine footrot is a highly contagious disease caused by *Dichelobacter nodosus* (*D. nodosus*), a gram-negative anaerobic bacterium [1], which, depending on its virulence, could result in an acute necrotic disease. Footrot affects the epidermal tissues of the interdigital skin and horn of the hooves, being the main cause of lameness, decreased animal welfare and economical concern for sheep farming worldwide [2]. Affected sheep display a wide spectrum of clinical manifestations that vary from a mild interdigital dermatitis to under-running and separation of the hard horn of the hoof in severe stages of footrot. These clinical manifestations usually lead to poor feed intake, production losses, low fertility and reduction in milk yield [3–7]. New strategies for prevention of footrot are required. Treatment and control methods implemented so far are costly and rely mainly on the use of antibiotics and chemicals in footbaths which induce bacterial resistance, being a major challenge to profitable sheep farming [8–11].

Footrot incidence and severity are essentially modulated by three key factors: i) environmental conditions; ii) virulence of *D. nodosus* strains; and iii) host genetics [2, 12, 13]. Reports indicate that footrot innate resistance is a heritable trait and that some breeds, such as Merino sheep, are particularly susceptible to mild footrot stages showing low recovery rates [14]. Moreover, under the same environmental conditions, the innate resistance varies considerably both between and within breeds. Understanding the genetic basis of footrot resistance would facilitate the selection of more resilient animals using molecular techniques [15–17]. The finding of molecular markers associated with footrot resistance/susceptibility would be a powerful aid in the development of new methods for disease control based on breeding strategies [6, 18].

The genetic architecture underlying host resistance traits is usually complex and generally determined by multiple gene interactions. With the advent of cost-effective high-throughput genotyping methods, genome-wide association studies (GWAS) have been widely used to identify disease related variants in livestock. Such studies have contributed to a better understanding of the complex biological processes of disease pathogenesis, leading to the identification of molecular markers tightly linked to resistance genes [19, 20]. Extensive efforts have been made to investigate the pathogenesis and aetiology of footrot in sheep [21–25], but few studies were focused to understand innate genetic resistance. The first attempt to investigate the genetic association to footrot resistance/susceptibility in sheep resulted in the identification of genetic variants involved in the major histocompatibility complex (MHC) class II genes [26]. More recently, a first GWAS used the Illumina OvineSNP50 array to identify seven SNPs significantly associated with footrot resistance/susceptibility in Texel sheep on a chromosome-wide level [27]. Subsequently, Niggeler et al*.* [28] genotyped Swiss White Alpine sheep with the Illumina Ovine SNP600K array and reported one SNP located on the Multiple PDZ Domain Crumbs Cell Polarity Complex Component (MPDZ) gene showing significant genome-wide association with footrot resistance/susceptibility. The function of this gene, located on chromosome 2, is not well established but it has been reported to be involved in the maintenance of tight junctions integrity [29]. In general, these studies highlighted that footrot resistance/susceptibility is a complex trait determined by the interplay of multiple genetic mechanisms. This reinforces further research is needed for a more comprehensive understanding of footrot.

Previous GWAS studies relied on the use of commercially available SNP arrays. However, these markers are susceptible to ascertainment bias due to the limited number of individuals and breeds used in the array design [30–32]. As a result, the use of commercial SNP arrays could miss informative variants segregating specifically in local breeds.

The objective of this study was to identify genomic regions and candidate genes with variants significantly associated with footrot resistance/susceptibility in Portuguese Merino breeds and crossbred animals reared in the Alentejo region (south of Portugal). A customized SNP array from whole-genome sequence data collected in these native breeds was developed to carry out a GWAS for footrot resistance/susceptibility.

## Materials and methods

### Sampling and phenotypic data

Sampling was carried out during clinical diagnostics of footrot infection in 17 flocks distributed throughout the Alentejo region in southern Portugal. These flocks were identified based on epidemiologic inquiries to sheep breeders from the Alentejo region conducted by the project team to evaluate the prevalence of footrot (described in more detail in Albuquerque et al. [33] and Supplementary Note). The flocks were visited twice and each sheep was scored at least once. The set of animals sampled in the two visits per flock was not exactly the same due to unforeseen situations such as death, animals being sold or participation in fairs. A total of 1436 animals, including Merino Branco (*N* = 356), Merino Preto (*N* = 142) and crossbreds (*N* = 938) were sampled and information regarding their breed, flock, sampling month and age was also registered. No control measures or footrot treatment were applied prior to animal inspection and sampling to avoid biased results. Briefly, whole blood (10 ml) was collected from the jugular vein, using vacuum EDTA collection tubes and stored at -20ºC for subsequent DNA extraction. Foot lesions were scored following the Modified Egerton System [34], ranging from 0 (healthy) to 5 (severe footrot), based on visual inspection of each hoof (left and right hindlimb, left and right forelimb). Hence, each animal had a potential highest footrot score (HFS) per hoof of 5 and a potential maximum global footrot score (GFS) of 20, per scoring event. In addition, a weighting factor was defined to better account for the overall impact of the injury on the animal. Scores of three, four and five were given a weighting factor of 2.5, 3 and 3.25, respectively. The original individual foot score was converted into an index by multiplying it by the respective weighting factor, resulting in individual foot index scores of 0, 1, 4, 7.5, 12 and 16.25 (Table S1). This strategy was adopted to avoid an animal with no signs of infection from having a phenotype worse than an animal with a mild or severe footrot score. Hence, each animal had a potential maximum index footrot score (IFS) of 65, per scoring event, which was the phenotypic value considered for the association analyses. In addition, two alternative scenarios were used for comparison purposes: one considering HFS as the phenotype of interest, classified in three categories (i – no signs of footrot infection, scores 0 and 1; ii – mild signs of footrot infection, scores 2 and 3; iii – severe signs of footrot infection, scores 4 and 5); and the other based on GFS. As mentioned above, some of the animals had only a single score, so a uniform criteria was used by considering the highest global score between the two visits. As a complementary diagnostic method, the presence of the main causal agent, i.e. *D. nodosus*, was monitored in ~ 18% and 15% of the animals included in our study by qPCR and metagenomic analyses, respectively [25, 33]. Details on flock locations, number of animals sampled per flock and breed(s) are shown in Table S2.

Heritability estimates were based on footrot records (score 0—not affected vs score > 0—affected) collected in 437 ewes (239 Merino Branco and 198 Merino Preto) registered in the Flockbook from three flocks, between 2016 and 2018. The pedigree matrix included genealogical information on a total of 1229 animals (136 rams and 1093 ewes) comprising ~ 75% of animals with known parents. We used two methods (Frequentist—REM with MTDFREML software and Bayesian—Gibbs sampling with TM software). In both methods, the model used included, in addition to the random effects (genetic and permanent environmental effects), the fixed effects of the farm, the season of the score evaluation and the age of the animal [35].

### SNP panel design

For the SNP panel design, whole-genome resequencing data obtained by Gaspar et al. [36] for 39 sheep, including: 10 Merino Branco; 10 Merino Preto; and 19 crossbreds, was used. The criteria used for SNP selection and probe design were the following: i) minor allele frequency (MAF) > 1%; ii) call rate per SNP > 90%; iii) flanking region of 150 base pairs (bp) upstream and downstream of the SNP position; and iv) no variable positions allowed (SNPs or INDELs) within these flanking regions. SNPs in coding regions were prioritized and an even distribution of the SNPs across all chromosomes was assured. The SNP panel included a total of 47,779 SNPs with known position across the 26 ovine autosomes of the sheep reference genome Rambouillet version 1.0 (GCA_002742125.1 Oar_rambouillet_v1.0). The genomic locations of the SNPs used for genotyping are shown in Table S3.

### Genotyping, quality control and imputation

Extraction of genomic DNA was done by service acquisition (LGC science Group, Teddington, UK). Samples were genotyped using our customized SNP panel at IGATech (IGA Technology Services, Udine, Italy), following a targeted genotyping system based on single primer enrichment technology (Allegro, Tecan Genomics). The PLINK software v.1.90b5.2 [37] was used to assess SNP quality based on the following criteria: i) Minor allele frequency > 1%; ii) call rate per SNP > 90%; and (iii) no extreme deviation from Hardy–Weinberg equilibrium (*P* < 10^–6^). Additionally, individuals with more than 20% missing data were excluded from the analysis. After quality control, 29,716 SNPs and 1375 samples were retained and used in the GWAS. Finally, missing genotypes were imputed using Beagle software v.5.4 [38].

### Genome-wide association study

Prior to the GWAS, a molecular kinship matrix between individuals was obtained with the VanRaden kinship algorithm [39]. Then, the GWAS was conducted using the function “GWAS” implemented in the R package rrBLUP v.4.6.2 [40] and a mixed linear model (MLM) approach [41], as follows:

$$\mathrm{y }=\mathrm{ X\beta }+\mathrm{ Zg }+\mathrm{ S\tau }+\upvarepsilon$$

where **y** is a vector of phenotypic values; **X** is a matrix of fixed effects; **β** is a vector containing fixed effects, such as breed, flock, sampling month, age class (lambs < 12 months; hoggets 12–24 months; and adults > 24 months) and the kinship matrix (**K**); **Z** and** S** are incidence matrices of the model; **g** models the genetic background as a random effect with Var[g] = Kσ^2^, where **σ**^**2**^ is the total genetic variance; **τ** models the additive SNP effect as a fixed effect; **ε** is a vector of residual variance. The family-wise error rate was controlled by using an adjusted Bonferroni *p*-value based on the estimated number of independent SNPs. The adjusted genome-wide significance threshold was -log10 (p = 0.05 / N) (2.06 × 10^−6^), where N is the number of independent SNPs that was determined using a variant pruning estimator (-indep-pairwise 50 10 0.2) in the PLINK software (*N* = 24,211). In addition, a genome-wide suggestive threshold of -log10 (p = 1 / N) (4.13 ×  × 10^–5^) was considered. The R package “qqman” [42] was used for the graphical visualization of the results. A quantile–quantile (QQ) plot was generated to represent the deviation of the observed *p*-values from the null hypothesis, which is useful to determine potential population stratification or analytical approach biases based on the comparison of the observed and expected (null) distributions of the data. Candidate genes within the range of genome-wide significant and suggestive SNP regions were identified using the Ensembl database (http://www.ensembl.org) and the gene annotation information of the Oar_rambouillet_v1.0 reference genome. Moreover, the biological processes, cell components and molecular function of the associated genes were inferred through the Gene Ontology terms [43] and Kyoto Encyclopedia of Genes and Genomes (KEGG) [44] using the Database for Annotation, Visualization and Integrated Discovery v.2022q4 (DAVID) [45, 46].

## Results

### SNP panel selection and distribution

The selection of SNPs to be used in the assay was based on whole-genome resequencing data from 39 Merino Branco, Merino Preto and crossbred animals sampled in the south of Portugal [36]. A total of 47,779 autosomal SNPs were used for genotyping (Figure S1) of which 10,053 (21.04%) were exonic, 12,655 (26.49%) intronic, 15,616 (32.68%) intergenic, 6959 (14.56%) were in 3’ untranslated regions (UTR) and 2496 (5.22%) in 5’ UTRs (Figure S1). The SNPs located in coding regions included 6636 (66.01%) and 3291 (32.74%) associated with synonymous and non-synonymous effects, respectively. On average, the distance between SNPs was 53.54 kb. The maximum distance between neighbouring SNPs was found on chromosome 12, where no SNPs were detected in a gap of approximately 5.57 Mb. A graphical visualization of the number of selected SNPs per chromosome is provided in Figure S2.

### Footrot statistics

Descriptive statistics for the footrot scores of the animals included in this study are shown in Table S4. A total of 1436 sheep from 17 different flocks were phenotyped using the Modified Egerton scoring scale system (scores 0 to 5) based on clinical signs of footrot. The distribution of animals per flock is presented in Figure S3. The IFS, GFS and HFS varied from 0 to 39 (mean = 2.87 ± 5.14), 0 to 14 (mean = 1.62 ± 2.31) and 0 to 5 (mean 0.89 ± 1.09), respectively (Table S4), with most of the animals exhibiting no clinical signs of disease (score 0). The distribution of phenotypic scores per breed is plotted in Figure S4. The complementary diagnostic methods showed that for three flocks the causal agent was not detected, namely: I (*n* = 6), J (*n* = 14) and Q (*n* = 7) which represented ~ 6%, 32% and 7.5% of the animals sampled, respectively. However, sheep in flocks I and J had feet scores that varied from 0 to 2, and those in flock Q varied from 0 to 4, thus the animals of these flocks were also included in the GWAS. Indeed, it has been shown that about 43% and 59% of the animals with score 2 feet show under-running of the hoof (score 3) within 5 or 10 days, respectively [47]. Estimates of footrot heritabilities from the frequentist and Bayesian approaches were 0.127 and 0.130, respectively.

### Genome-wide association analysis

**Fig. 1:**
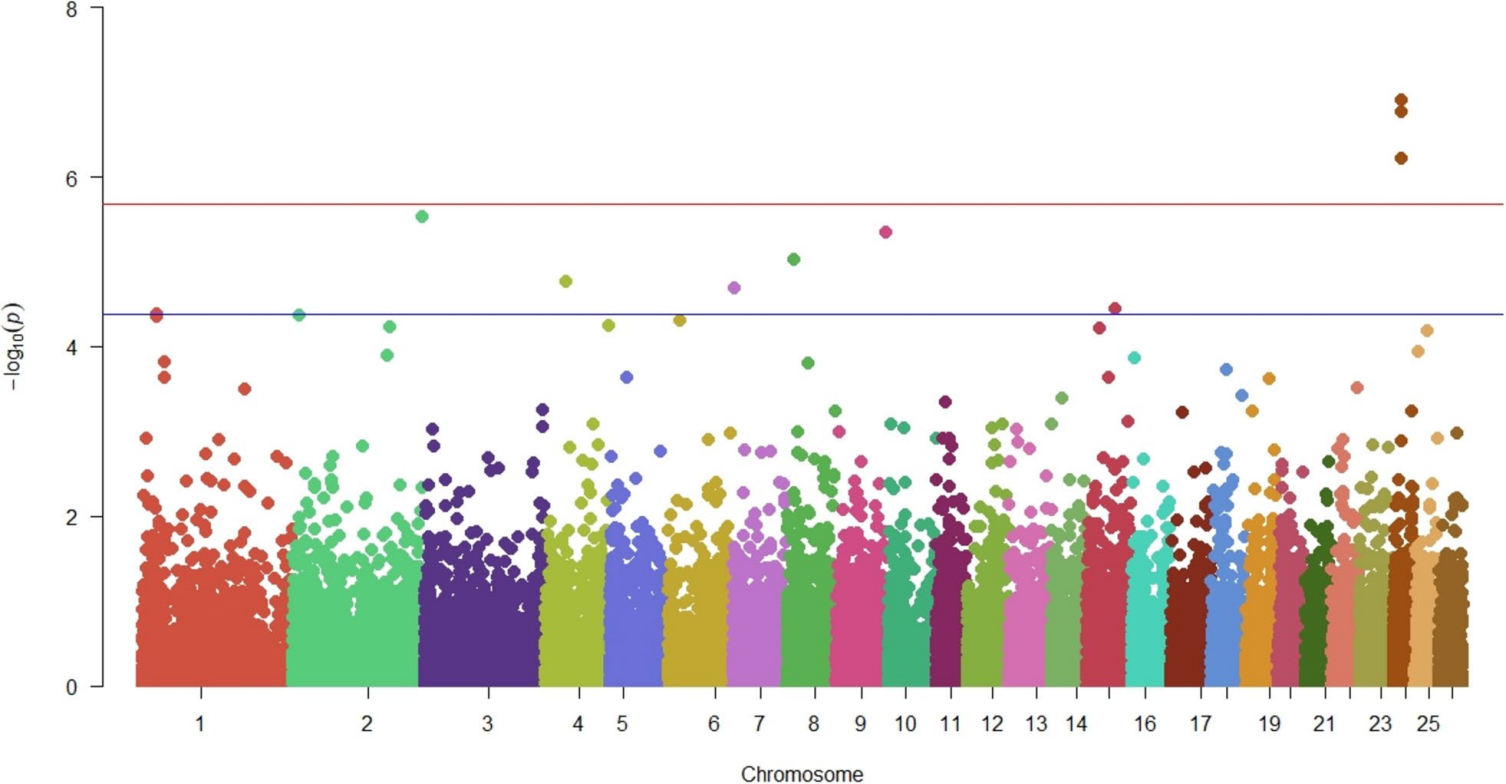
Manhattan plot displaying the results of the genome-wide association analysis for footrot. The left vertical axis indicates the –log10 of *p*-values, while the horizontal axis indicates chromosomes and physical map positions of the SNPs. Red and blue lines indicate the thresholds for Bonferroni-adjusted genome-wide significant and suggestive level, respectively

Following quality control procedures, a filtered set of 29,716 autosomal SNPs and 1375 animals were used for the association analysis to identify variants and genes linked to footrot resistance/susceptibility in Portuguese Merino and crossbred animals. Considering the adjusted Bonferroni genome-wide significance threshold of p = 2.06 × 10^–6^, the GWAS results based on the IFS phenotypes revealed three genome-wide significant SNPs located on chromosome 24. The proportion of phenotypic variance explained by the significant SNPs was between 1.33% and 1.37%. Additionally, six genome-wide suggestive SNPs were detected each on chromosomes 2, 4, 7, 8, 9 and 15 (Fig. 1). Further details on the location, significance level and annotation of these SNPs are summarized in Table 1.

**Table 1:** Summary of genome-wide significant and suggestive SNPs associated with footrot resistance/susceptibility in Portuguese Merino and crossbred animals

Chr	Position (bp)	A1	A2	*p*-value	Genomic Region	Functional effect	Gene
**Genome-wide significant SNPs**							
24	16,664,127	G	T	1.23E-07	UTR3	-	SMG1
24	16,683,234	G	A	1.69E-07	exon	synonymous	SMG1
24	16,688,212	T	C	6.16E-07	exon	synonymous	SMG1
**Genome-wide suggestive SNPs**							
2	260,442,170	C	T	2.93E-06	exon	synonymous	HSPG2
9	100,456,022	T	G	4.49E-06	intron	-	RALYL
8	13,714,405	G	A	9.38E-06	UTR5	-	CENPW
4	42,346,212	T	G	2.08E-05	intron	-	PCLO
7	3,506,174	C	G	2.06E-05	Intergenic	-	LOC106991156 (dist = 553,763); THBS1 (dist = 161,183)
15	57,714,827	G	A	3.66E-5	intron	-	KLHL35

*Chr *chromosome, *A1 *allele 1, *A2 *allele 2

**Fig. 2:**
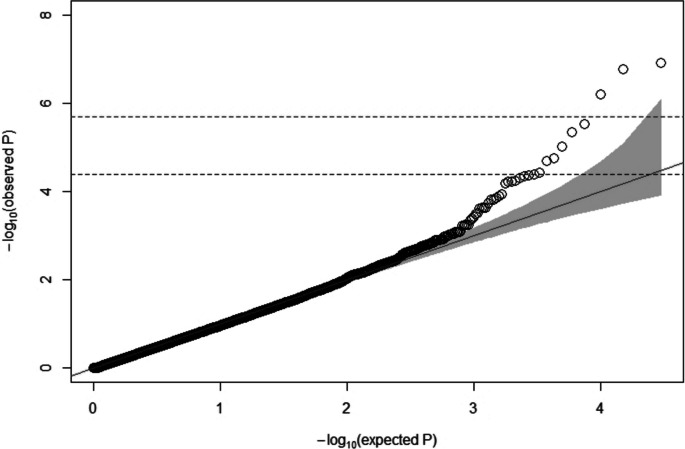
Quantile–quantile (QQ) plot of genome-wide association results for footrot. Top and bottom horizontal dashed lines indicate the thresholds for Bonferroni-adjusted genome-wide significant and suggestive level, respectively

A major critical problem in GWAS is the false positive findings that arise from population structure and family relatedness. Thus, a QQ plot was obtained to verify the validity of the *p*-values and identify population structure that might not have been considered in the statistical model. Homogeneity among populations was also inferred by kinship analysis, which indicated slight population stratification (Figure S5). The QQ plot obtained from the GWAS shows that the data follow a normal distribution, without evidence for systematic bias from population structure (Fig. 2). Furthermore, the deviation of the SNP *p*-values observed from their expected probability at the tail of the distribution suggests a strong association effect on footrot resistance/susceptibility, which is in accordance with the results shown in the Manhattan plot (Fig. 2).

When the alternative GFS scores were used as the main phenotype, two genome-wide significant SNPs were identified on chromosome 24 (16,664,127 bp; 16,683,234 bp), which were in common with the IFS approach. In addition, six genome-wide suggestive SNPs were located on chromosomes 2 (13,704,688 bp; 260,442,170 bp; 194,217,323 bp), 4 (42,346,212 bp), 9 (100,456,022 bp) and 24 (16,688,212 bp) (Figure S6A), of which one was identified as genome-wide significant and three as genome-wide suggestive in the IFS approach.

When the HFS scores were used, no significant genome-wide SNPs were identified. However, two genome-wide suggestive SNPs were found on chromosomes 19 (45,832,644 bp) and 24 (38,953,050 bp) (Figure S6B).

### Gene annotation and pathway analyses

The SNPs associated with footrot resistance/susceptibility were found within regions of known genes in the GCA_002742125.1 Oar_rambouillet_v1.0 genome reference (Table 1). Three genome-wide significant SNPs were found on chromosome 24 within the nonsense mediated mRNA decay region of the PI3K related kinase (SMG1) gene. Genome-wide suggestive SNPs were located within an exonic region of heparan sulfate proteoglycan 2 (HSPG2) coding gene on chromosome 2, the UTR5 region of Centromere protein W (CENPW) coding gene on chromosome 8 and an intronic region of RALY RNA binding protein like (RALYL), Piccolo Presynaptic Cytomatrix (PCLO) and Kelch like family member 35 (KLHL35) coding genes on chromosomes 9, 4 and 15, respectively. Furthermore, one genome-wide suggestive SNP was located in the intergenic region, 161 kb away from the Thrombospodin 1 (THBS1) coding gene, on chromosome 7.

For a better understanding of their function, the candidate genes were functionally annotated with DAVID web server (Table S5) to retrieve information on gene ontologies and KEGG pathways. The results of the functional annotation showed that SMG1 and THBS1 have ontologies related to known immunological processes and wound healing, including nonsense-mediated mRNA decay (GO:0000184), cell adhesion (GO:0007155) and heparin-binding (GO:0008201). Additionally, HSPG2 and PCLO are involved in the reinforcement of the physical cellular structures, namely the extracellular matrix (GO:0031012), basement membrane (GO:0005604) and cell projection (GO:0042995). Regarding the KEGG pathways, the results show that SMG1, HSPG2 and THBS1 are linked to seventeen pathways (Table S5). These pathways were mainly associated with the activation and regulation of innate immune cells, such as the Rap1, p53, PI3K-Akt and TGF-beta signalling pathways, necroptosis, mRNA surveillance, ECM-receptor interaction and proteoglycans in cancer.

## Discussion

The GWAS analysis to investigate the genetic basis of footrot resistance/susceptibility in the Portuguese native breeds Merino Branco and Merino Preto, and a population of crossbred sheep, resulted in the identification of three and six genome-wide significant and suggestive SNPs, respectively (Table 1). The three significant SNPs were located within the SMG1 coding gene on chromosome 24, which could indicate a promising candidate region affecting footrot resistance/susceptibility. In a study of Texel sheep [27], a chromosome-wise significant SNP associated with footrot had already been identified in chromosome 24 (Oar_v3.1, 24:962,868). SMG1 is a member of the PIKK (phosphoinositide 3-kinase related kinases) family and plays a critical role in the DNA damage response, resistance to oxidative stress, apoptosis as well as in the nonsense-mediated mRNA decay (NMD). The latter is an essential surveillance mechanism that modulates cellular homeostasis, response to stress, inflammation and immune regulation in reaction to pathogen infections [48–51]. In mice, decreased SMG1 expression levels led to increased basal inflammation and subsequent development of either cancer or chronic inflammatory disorders [52].

The immune response is triggered by a complex biological network system as a reaction to pathogens. It can be classified as either innate, which is non-specific, or adaptive which is highly specific [53]. While the innate immune system constitutes the first line of defence, representing an essential mechanism to prevent the spread of infection and maintain homeostasis, the adaptive immune system is the basis for the development of an immunologic memory that can lead e.g. to an effective immunization against infectious diseases [53–55]. A genome-wide suggestive SNP was identified on chromosome 9 within the intronic region of the RALYL coding gene, indicating a possible involvement in the response to footrot infection. However, additional research is needed to uncover its specific role. Recent evidence suggests that post-transcriptional regulatory machinery, controlled by RNA-binding proteins, is essential for the maintenance and modulation of immune responses [56, 57]. RALY RNA binding protein is a member of the heterogeneous nuclear ribonucleoproteins involved in mRNA splicing and metabolism processes [58]. A recent study described the role of RALY in the expression regulation of immunity and inflammatory response-related genes, by modulating the splicing of regulatory factors and alternative splicing, despite it remains poorly characterized in mammals [59].

Interdigital skin ulcerations and necrotic lesions are among the most frequent clinical manifestations of severe footrot infections [4, 60]. When a cutaneous injury occurs, a range of complex molecular processes is triggered towards wound healing and reestablishment of tissue structure and function [61, 62]. The results suggest a potential association of HSPG2 with footrot infection, as a genome-wide suggestive SNP was identified within its exonic region on chromosome 2. The HSPG2 protein, also known as Perlecan, is a basement membrane of the extracellular matrix (ECM) that plays a key role in both structural and regulatory mechanisms of wound repair activity through all phases of the healing process. HSPG2 mediates both scaffold support and signal transduction events by mainly controlling a range of growth factors activity to promote cell proliferation and differentiation, ECM organization and metabolism, as well as tissue remodelling [63–66]. It is worth highlighting the findings of a study that identified changes in HSPG2 patterns significantly associated with delayed wound repair and malformation of connective tissues rich in fibrillar collagens [67]. In addition, HSPG2-deficient keratinocytes have been reported to form a strikingly thin and poorly organized epidermis [68, 69]. HSPG2 has a crucial role in the reinforcement of physical barriers through which pathogens must penetrate, as it mediates both the survival and terminal differentiation events of keratinocytes, the major cell type of epidermis, being essential to epidermal integrity. The role of HSPG2 in relation to footrot could be split in two distinct stages: it could be involved in the reinforcement of structural defences as a primary physical barrier against pathogens; following infection, it could act in wound healing and tissue recovery. Recently, Niggeler et al. [28] reported one SNP significantly associated with footrot resistance in Swiss White Alpine sheep [70–72] located on chromosome 2 (Oar_v3.1, 2:81205092), upstream of the multi-PDZ domain protein 1 coding gene (MUPP1). Likewise, this gene seems to be associated with the reinforcement of the physical barrier function and integrity.

On chromosome 7, a genome-wide suggestive SNP was identified in an intergenic region 161 kb away from the THBS1 coding gene. THBS1 is an extracellular calcium-binding multifunctional protein, secreted by endothelial cells and fibroblasts, involved in a broad spectrum of cellular processes, such as the initial inflammatory events throughout the chronic inflammatory processes and tissue repair including cell adhesion, migration, proliferation and extracellular matrix expression and organization [70–72]. THBS1 is known to be a major activator of transforming growth factor-Beta (TGF-β) that mediates multiple physiological processes such as wound healing, cell proliferation, extracellular matrix formation and T-cells immune responses [73–75]. TGF-β plays a crucial role indistinct phases of the wound healing process, including epithelialization and tissue regeneration [76, 77]. TGFβ1 may have a therapeutic potential for atopic dermatitis as it is an important fibrogenic and immunomodulatory factor that regulates cellular processes implicated in the suppression of atopic dermatitis skin lesions [78, 79].

A genome-wide suggestive SNP was located on chromosome 15, within the intronic region of Kelch-like family gene KLHL35. An important paralog of this gene is KLHL24, which is involved in maintenance of mechanical stability of keratinocytes, the major cell type found in the epidermis, and turnover of intermediate filaments, in particular of keratin 14 [80, 81]. KLHL35 was associated with pyroptosis regulation in cell inflammatory necrosis despite being poorly studied [82].

A genome-wide suggestive SNP was found on chromosome 8 within UTR5 of CENPW coding gene. However, its function in footrot remains unclear. CENPW (centromere protein W), also known as cancer-upregulated gene 2 is suggested to have oncogenic activity as it is frequently upregulated in various cancer tissues and is known to play an important role in tumorigenesis [83, 84]. A recent study pointed out the role of CENPW on the regulation of gene expression in Treg cells [85].

In this study we identified genetic variants significantly associated with footrot resistance/susceptibility in two Portuguese native Merino sheep breeds and a population of crossbred animals. The candidate genes identified have specific roles in immune response against infection and wound healing processes. These findings contribute to a better understanding of the mechanisms underlying footrot disease in Merino sheep. Nonetheless, footrot resistance/susceptibility results from the interaction of many genes and is determined by multiple factors other than the genetic make-up of the animals. This is reflected in the heritability estimates obtained which were slightly lower than those previously reported for other breeds, i.e. below 13% of the variation between animals is due to genetic factors [15, 16, 27]. A limitation of this study is a low prevalence of severe footrot lesions in the flocks analysed which could overestimate the SNP effects observed. A major challenge inherent to field sampling conditions results from the limitation to carry out repeated scorings to monitor disease progression. This is due to the breeders’ need to treat animals before their welfare is compromised and could affect comparisons between individuals for their susceptibility [47]. As future work, it seems important to investigate how host genetic variation interplays with the footrot microbiome. Hence, examining the impact of host genetics on the composition of the foot-skin microbiome could contribute to further clarify the processes involved in footrot resistance/susceptibility. Identifying likely candidate genes that influence the relative abundance of certain taxa in the ovine foot-skin microbial communities may offer new perspectives to employ in breeding programs aiming to prevent footrot.

## Conclusion

This study represents a first attempt to infer the genetic basis of resistance/susceptibility to footrot in Portuguese Merino Branco and Merino Preto breeds and crossbreds. The GWAS analysis revealed three genome-wide significant SNPs located on chromosome 24 and six genome-wide suggestive SNPs each located on chromosomes 2, 4, 7, 8, 9, and 15. We disclosed novel information on the candidate genes involved in footrot resistance/susceptibility, contributing to a better understanding of the genetic architecture of this condition in Portuguese native sheep. The pratical use of the significant SNPs may be limited by the fact that no specific genomics regions with a high controbution to the genetic variance were identified. Nonetheless, our results could have a positive impact on future breeding programs in these breeds, by providing additional information aiming to select animals with the potential to be less susceptible to footrot.

### Supplementary Information

**Additional file 1:** **Supplementary Note.** **Figure S1.** Distribution of genotyped SNPs across genomic regions. **Figure S2.** Distribution of genotyped SNPs per chromosome. **Figure S3.** Distribution of sampled animals per farm. **Figure S4.** Distribution of footrot scores per breed. (A) Highest footrot score. (B) Global footrot score. (C) Index footrot score. Mean values are represented by green circles. **Figure S5.** Genomic kinship matrix heatmap of analysed animals. **Figure S6.** Manhattan plot and QQ-plot displaying the results of genome-wide association analysis considering the global footrot score (A) and the highest footrot score (B). Red and blue lines indicate the thresholds for Bonferroni-adjusted genome-wide significant and suggestive level, respectively.

**Additional file 2:** **Table S1.** Index footrot score weighting factors per individual score. **Table S2.** Details on farm locations, breeds and number of sampled animals per farm. Breed acronyms are as follows: Merino Branco -MB; Merino Preto -MP; and crossbreds –CR. **Table S3.** Genomic location of the SNPs used for genotyping and respective alleles. Chr - Chromosome; Pos. **Table S4.** Descriptive statistics for footrot score. Number of analysed animals (N), minimum (Min) and maximum (Max) footrot score, mean and standard deviation (SD). **Table S5.** Functional annotation of the candidate genes identified in the GWAS. Distribution of KEGG pathways and gene ontology categories are depicted, namely the cellular component, biological process, and molecular function.

## Data Availability

The dataset generated and analysed during the current study is available in the European Variation Archive (EVA) repository under the accession number PRJEB64885.
