# Correction: Genome-wide association study identifies genetic variants underlying footrot in Portuguese Merino sheep

**DOI:** 10.1186/s12864-024-10130-7

**Published:** 2024-03-04

**Authors:** Daniel Gaspar, Catarina Ginja, Nuno Carolino, Célia Leão, Helena Monteiro, Lino Tábuas, Sandra Branco, Ludovina Padre, Pedro Caetano, Ricardo Romão, Claudino Matos, António Marcos Ramos, Elisa Bettencourt, Ana Usié

**Affiliations:** 1 Centro de Biotecnologia Agrícola E Agro-Alimentar Do Alentejo (CEBAL)/Instituto Politécnico de Beja (IPBeja), 7801-908 Beja, Portugal; 2 CIBIO, Centro de Investigação em Bioaffiliationersidade e Recursos Genéticos, InBIO Laboratório Associado, Universidade do Porto, Campus de Vairão, R. Padre Armando Quintas 7, 4485-661 Vairão, Portugal; 3 BIOPOLIS Program in Genomics, Bioaffiliationersity and Land Planning, Campus do Varão, Campus de Vairão, R. Padre Armando Quintas 7, 4485-661 Vairão, Portugal; 4 CIISA, Centro de Investigação Interdisciplinar em Sanidade Animal, Faculdade de Medicina Veterinária, Universidade de Lisboa, Av. Universidade Técnica, 1300-477 Lisboa, Portugal; 5 Instituto Nacional de Investigação Agrária E Veterinária, I.P. (INIAV, I.P.), Avenida da República, Quinta Do Marquês, 2780-157 Oeiras, Portugal; 6 Escola Universitária Vasco da Gama, Av. José R. Sousa Fernandes 197, 3020-210 Lordemão, Coimbra, Portugal; 7 MED– Mediterranean Institute for Agriculture, Environment and Development, CHANGE– Global Change and Sustainability Institute, CEBAL– Centro de Biotecnologia Agrícola e Agro-Alimentar do Alentejo, 7801-908 Beja, Portugal; 8 ACOS– Agricultores do Sul, Beja, Portugal; 9 MED—Mediterranean Institute for Agriculture, Environment and Development and CHANGE– Global Change and Sustainability Institute, University of Évora, Polo da Mitra, Ap. 94, 7006-554 Évora, Portugal; 10 Departamento de Medicina Veterinária, Escola de Ciências E Tecnologia, Évora University, Pólo da Mitra Ap. 94, 7002-554 Évora, Portugal

**Correction**: ***BMC Genomics *25, 100 (2024)**

10.1186/s12864-023-09844-x

Following publication of the original article an error in the affiliation listing was reported. The incorrect affiliation distribution is shown below:

Daniel Gaspar1,2,3, Catarina Ginja2,3,4, Nuno Carolino4,5,6, Célia Leão1,5,7, Helena Monteiro7, Lino Tábuas7, Sandra Branco8,9, Ludovina Padre8, Pedro Caetano8, Ricardo Romão8, Claudino Matos7, António Marcos Ramos1,7, Elisa Bettencourt8 and Ana Usié1,7*

1 Centro de Biotecnologia Agrícola E Agro-Alimentar Do Alentejo (CEBAL)/ Instituto Politécnico de Beja (IPBeja), 7801 − 908 Beja, Portugal

2 CIBIO, Centro de Investigação em Biodiversidade e Recursos Genéticos, InBIO Laboratório Associado, Universidade do Porto, Campus de Vairão, R. Padre Armando Quintas 7, 4485 − 661 Vairão, Portugal

3 BIOPOLIS Program in Genomics, Biodiversity and Land Planning, Campus do Varão, Campus de Vairão, R. Padre Armando Quintas 7, 4485 − 661 Vairão, Portugal

4 Instituto Nacional de Investigação Agrária E Veterinária, I.P. (INIAV, I.P.), Avenida da República, Quinta Do Marquês, 2780 − 157 Oeiras, Portugal

5 CIISA, Centro de Investigação Interdisciplinar em Sanidade Animal, Faculdade de Medicina Veterinária, Universidade de Lisboa, Av. Universidade Técnica, 1300 − 477 Lisboa, Portugal

6 Escola Universitária Vasco da Gama, Av. José R. Sousa Fernandes 197, 3020 − 210 Lordemão, Coimbra, Portugal

7 MED– Mediterranean Institute for Agriculture, Environment and Development and CHANGE– Global Change and Sustainability Institute, CEBAL– Centro de Biotecnologia Agrícola e Agro-Alimentar do Alentejo, 7801 − 908 Beja, Portugal

8 MED—Mediterranean Institute for Agriculture, Environment and Development and CHANGE– Global Change and Sustainability Institute, University of Évora, Polo da Mitra, Ap. 94, 7006 − 554 Évora, Portugal

9 Departamento de Medicina Veterinária, Escola de Ciências E Tecnologia, Évora University, Pólo da Mitra Ap. 94, 7002 − 554 Évora, Portugal

The correct affiliations are given in the authorship list of this Correction article and the original article has been updated.

