# Supplementary material for: Genome-wide association study identifies genetic variants underlying footrot in Portuguese Merino sheep
Source: BMC Genomics. 2024 Jan 23;25:100. doi: 10.1186/s12864-023-09844-x (PMC10804546; doi:10.1186/s12864-023-09844-x)
Supplement: Supplementary file 1 — Additional file 1: Supplementary Note. Figure S1. Distribution of genotyped SNPs across genomic regions. Figure S2. Distribution of genotyped SNPs per chromosome. Figure S3. Distribution of sampled animals per farm. Figure S4. Distribution of footrot scores per breed. (A) Highest footrot score. (B) Global footrot score. (C) Index footrot score. Mean values are represented by green circles. Figure S5. Genomic kinship matrix heatmap of analysed animals. Figure S6. Manhattan plot and QQ-plot displaying the results of genome-wide association analysis considering the global footrot score (A) and the highest footrot score (B). Red and blue lines indicate the thresholds for Bonferroni-adjusted genome-wide significant and suggestive level, respectively. [file 12864_2023_9844_MOESM1_ESM.zip › FigureS6.pdf]

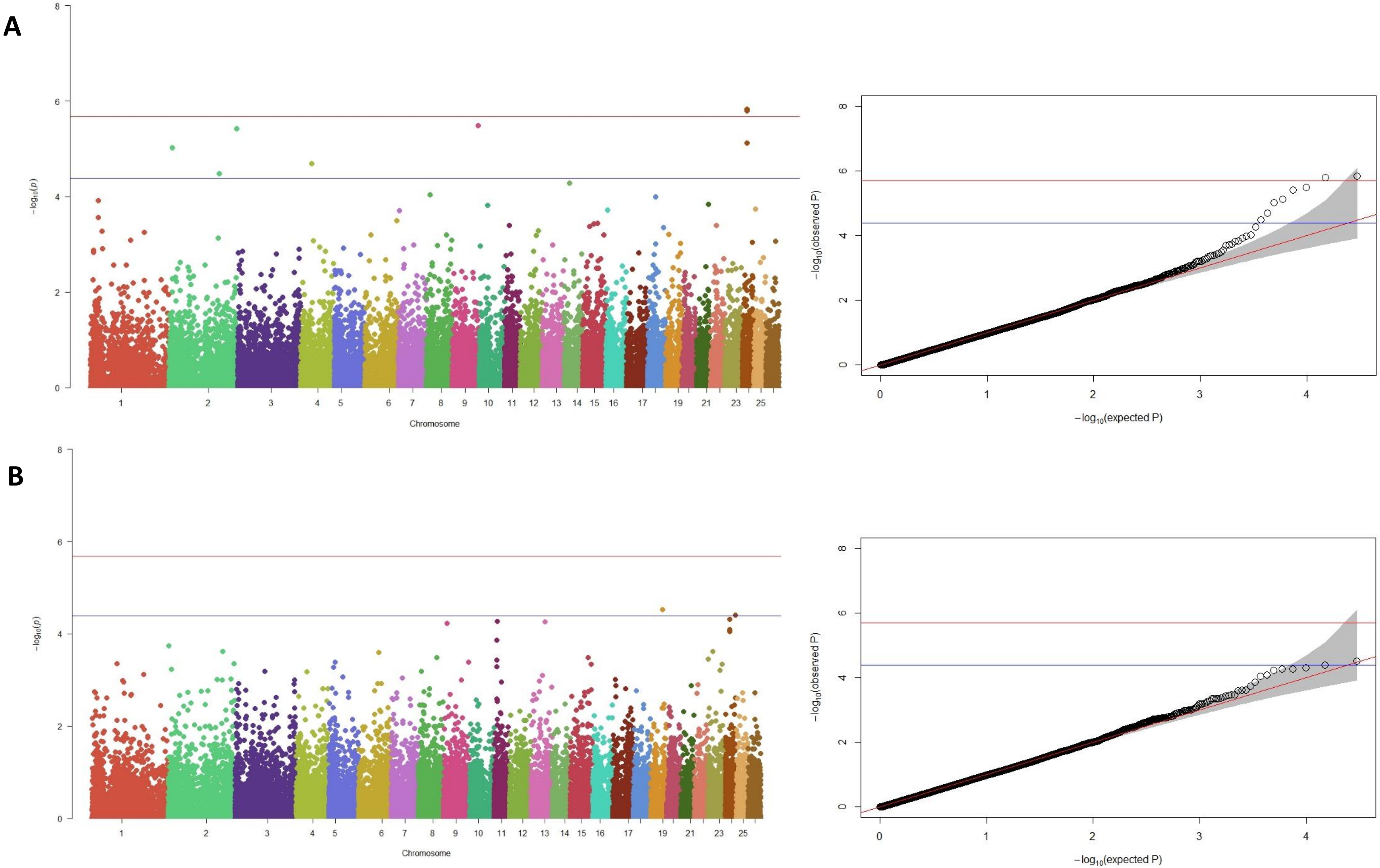

Figure S6 Manhattan plot and QQ-plot displaying the results of genome-wide association analysis considering the global footrot score **(A)** and the highest footrot score **(B)**. Red and blue lines indicate the thresholds for Bonferroni-adjusted genome-wide significant and suggestive level, respectively.
