# Supplementary material for: Genome-wide association study identifies genetic variants underlying footrot in Portuguese Merino sheep
Source: BMC Genomics. 2024 Jan 23;25:100. doi: 10.1186/s12864-023-09844-x (PMC10804546; doi:10.1186/s12864-023-09844-x)
Supplement: Supplementary file 2 — Additional file 2: Table S1. Index footrot score weighting factors per individual score. Table S2. Details on farm locations, breeds and number of sampled animals per farm. Breed acronyms are as follows: Merino Branco -MB; Merino Preto -MP; and crossbreds –CR. Table S3. Genomic location of the SNPs used for genotyping and respective alleles. Chr - Chromosome; Pos. Table S4. Descriptive statistics for footrot score. Number of analysed animals (N), minimum (Min) and maximum (Max) footrot score, mean and standard deviation (SD). Table S5. Functional annotation of the candidate genes identified in the GWAS. Distribution of KEGG pathways and gene ontology categories are depicted, namely the cellular component, biological process, and molecular function. [file 12864_2023_9844_MOESM2_ESM.zip › Table S2.pdf]

Table S2: Details on farm locations, breeds and number of sampled animals per farm. Breed acronyms are as follows: Merino Branco - MB; Merino Preto - MP; and crossbreds - CR

Farm	Location	Breed	Number of sampled animals
A	Castro Verde	CR	92
B	Évora	MB	49
C	Serpa	MB	89
D	Serpa	MP	74
E	Almodôvar	CR	94
F	Portel	CR	103
G	Ponte de Sor	CR	51
H	Montemor-o-Novo	CR	73
I	Alandroal	CR	102
J	Portalegre	MB / MP	44
K	Ferreira do Alentejo	CR	55
L	Alvito	CR	94
M	Fronteira	MB	93
N	Évora	MB / CR	63
O	Odemira	CR	103
P	Ponte de Sor	CR	103
Q	Moura	MB / MP	93
