# Supplementary material for: Genome-wide association study identifies genetic variants underlying footrot in Portuguese Merino sheep
Source: BMC Genomics. 2024 Jan 23;25:100. doi: 10.1186/s12864-023-09844-x (PMC10804546; doi:10.1186/s12864-023-09844-x)
Supplement: Supplementary file 2 — Additional file 2: Table S1. Index footrot score weighting factors per individual score. Table S2. Details on farm locations, breeds and number of sampled animals per farm. Breed acronyms are as follows: Merino Branco -MB; Merino Preto -MP; and crossbreds –CR. Table S3. Genomic location of the SNPs used for genotyping and respective alleles. Chr - Chromosome; Pos. Table S4. Descriptive statistics for footrot score. Number of analysed animals (N), minimum (Min) and maximum (Max) footrot score, mean and standard deviation (SD). Table S5. Functional annotation of the candidate genes identified in the GWAS. Distribution of KEGG pathways and gene ontology categories are depicted, namely the cellular component, biological process, and molecular function. [file 12864_2023_9844_MOESM2_ESM.zip › Table S3.pdf]

Table S3: Genomic location of the SNPs used for genotyping and respective alleles. Chr - Chromosome; Pos

Chr	Pos	REF	ALT
NC_040252.1	19107	T	C
NC_040252.1	56232	G	A
NC_040252.1	75407	C	T
NC_040252.1	121414	A	G
NC_040252.1	127102	A	G
NC_040252.1	136832	C	G
NC_040252.1	137026	G	C
NC_040252.1	138370	A	G
NC_040252.1	144783	T	C
NC_040252.1	145035	T	G
NC_040252.1	185403	A	G
NC_040252.1	193632	C	G
NC_040252.1	199057	A	G
NC_040252.1	199935	G	T
NC_040252.1	207300	T	C
NC_040252.1	232025	A	G
NC_040252.1	249916	G	A
NC_040252.1	262253	A	G
NC_040252.1	303453	T	G
NC_040252.1	336641	T	C
NC_040252.1	347452	A	C
NC_040252.1	399977	C	T
NC_040252.1	456143	C	T
NC_040252.1	502964	A	G
NC_040252.1	509061	T	C
NC_040252.1	526090	C	T
NC_040252.1	582542	T	C
NC_040252.1	589687	T	C
NC_040252.1	607779	G	A
NC_040252.1	607991	T	C
NC_040252.1	608160	A	G
NC_040252.1	620737	A	G
NC_040252.1	622263	A	G
NC_040252.1	622901	T	C
NC_040252.1	636233	T	C
NC_040252.1	638541	A	G
NC_040252.1	694473	A	G
NC_040252.1	702352	A	T
NC_040252.1	723637	T	C
NC_040252.1	727774	T	C
NC_040252.1	779664	A	G
NC_040252.1	830017	A	G
NC_040252.1	856868	T	G
NC_040252.1	912814	T	C
NC_040252.1	976711	T	C

NC_040252.1	993768	T	C
NC_040252.1	1005800	C	A
NC_040252.1	1006830	C	G
NC_040252.1	1066706	T	C
NC_040252.1	1067662	T	C
NC_040252.1	1093886	A	G
NC_040252.1	1144896	A	G
NC_040252.1	1171925	A	G
NC_040252.1	1201447	T	C
NC_040252.1	1233422	A	G
NC_040252.1	1354242	G	A
NC_040252.1	1435149	C	G
NC_040252.1	1483504	C	T
NC_040252.1	1508551	T	C
NC_040252.1	1550243	C	T
NC_040252.1	1569712	T	C
NC_040252.1	1656745	T	C
NC_040252.1	1703512	G	A
NC_040252.1	1817740	A	G
NC_040252.1	1864302	T	C
NC_040252.1	1918000	C	T
NC_040252.1	1971032	A	G
NC_040252.1	1996595	A	G
NC_040252.1	2051181	C	A
NC_040252.1	2106763	G	A
NC_040252.1	2160122	C	T
NC_040252.1	2212944	G	A
NC_040252.1	2230481	A	G
NC_040252.1	2285438	A	T
NC_040252.1	2331228	T	C
NC_040252.1	2355978	T	C
NC_040252.1	2399320	G	T
NC_040252.1	2456864	A	C
NC_040252.1	2509190	G	A
NC_040252.1	2575190	C	T
NC_040252.1	2609852	A	C
NC_040252.1	2633785	G	A
NC_040252.1	2684746	G	A
NC_040252.1	2687557	G	T
NC_040252.1	2720003	T	C
NC_040252.1	2721373	C	T
NC_040252.1	2721557	A	G
NC_040252.1	2722300	T	C
NC_040252.1	2723231	A	G
NC_040252.1	2724075	G	A
NC_040252.1	2784722	T	C
NC_040252.1	2816585	T	C

NC_040252.1	2843546	T	C
NC_040252.1	2868051	A	G
NC_040252.1	2910804	A	G
NC_040252.1	2912460	A	G
NC_040252.1	2964058	G	T
NC_040252.1	2989317	C	G
NC_040252.1	3109378	T	C
NC_040252.1	3165534	G	A
NC_040252.1	3187847	C	T
NC_040252.1	3242948	A	T
NC_040252.1	3277487	T	C
NC_040252.1	3291867	G	A
NC_040252.1	3295862	A	G
NC_040252.1	3296664	C	T
NC_040252.1	3309508	A	G
NC_040252.1	3310051	C	T
NC_040252.1	3312082	A	G
NC_040252.1	3324333	G	A
NC_040252.1	3324621	T	C
NC_040252.1	3324849	C	T
NC_040252.1	3380810	T	C
NC_040252.1	3401593	C	T
NC_040252.1	3464483	A	G
NC_040252.1	3497257	A	C
NC_040252.1	3531933	C	G
NC_040252.1	3580636	G	A
NC_040252.1	3631168	C	T
NC_040252.1	3678263	G	A
NC_040252.1	3692888	G	A
NC_040252.1	3698285	T	C
NC_040252.1	3705635	T	C
NC_040252.1	3713391	T	C
NC_040252.1	3718448	C	T
NC_040252.1	3742813	T	C
NC_040252.1	3745019	G	A
NC_040252.1	3787728	T	G
NC_040252.1	3844787	G	C
NC_040252.1	3889112	A	G
NC_040252.1	3906479	A	C
NC_040252.1	3967428	G	A
NC_040252.1	4026293	C	G
NC_040252.1	4081832	A	G
NC_040252.1	4138431	G	T
NC_040252.1	4192346	T	C
NC_040252.1	4247426	T	C
NC_040252.1	4265186	A	G
NC_040252.1	4329244	T	C

NC_040252.1	4384858	G	T
NC_040252.1	4428266	T	C
NC_040252.1	4489495	A	G
NC_040252.1	4531732	T	C
NC_040252.1	4584820	A	G
NC_040252.1	4644583	G	C
NC_040252.1	4662360	A	G
NC_040252.1	4718757	G	T
NC_040252.1	4774064	A	G
NC_040252.1	4820032	T	G
NC_040252.1	4883688	T	G
NC_040252.1	4931038	C	G
NC_040252.1	4969183	A	G
NC_040252.1	4969546	G	C
NC_040252.1	5011617	C	A
NC_040252.1	5028946	T	C
NC_040252.1	5080247	T	G
NC_040252.1	5136128	T	C
NC_040252.1	5187984	G	C
NC_040252.1	5245084	A	G
NC_040252.1	5300395	G	T
NC_040252.1	5358629	T	G
NC_040252.1	5416377	C	T
NC_040252.1	5464733	G	A
NC_040252.1	5517024	T	C
NC_040252.1	5572245	G	C
NC_040252.1	5582201	A	C
NC_040252.1	5641898	G	A
NC_040252.1	5693606	C	T
NC_040252.1	5748888	G	A
NC_040252.1	5781543	A	G
NC_040252.1	5802053	A	T
NC_040252.1	5846171	C	T
NC_040252.1	5885558	T	C
NC_040252.1	5910846	T	C
NC_040252.1	5963862	T	C
NC_040252.1	6007150	A	G
NC_040252.1	6014576	T	C
NC_040252.1	6015545	G	A
NC_040252.1	6070062	A	G
NC_040252.1	6098226	G	A
NC_040252.1	6156365	T	G
NC_040252.1	6208322	T	C
NC_040252.1	6235207	T	C
NC_040252.1	6265359	A	C
NC_040252.1	6319686	C	A
NC_040252.1	6361157	T	C

NC_040252.1	6481244	A	G
NC_040252.1	6532675	C	A
NC_040252.1	6566085	G	C
NC_040252.1	6566241	C	T
NC_040252.1	6619120	T	C
NC_040252.1	6665092	A	G
NC_040252.1	6703860	T	C
NC_040252.1	6769197	T	C
NC_040252.1	6814640	T	C
NC_040252.1	6850776	A	G
NC_040252.1	6867496	C	T
NC_040252.1	6913632	T	C
NC_040252.1	6939060	G	A
NC_040252.1	7084803	T	C
NC_040252.1	7103282	G	T
NC_040252.1	7159845	C	G
NC_040252.1	7185974	G	A
NC_040252.1	7251417	A	G
NC_040252.1	7260745	A	G
NC_040252.1	7260900	A	G
NC_040252.1	7298941	T	C
NC_040252.1	7323020	A	G
NC_040252.1	7352060	A	G
NC_040252.1	7364523	T	C
NC_040252.1	7369653	A	G
NC_040252.1	7369989	T	C
NC_040252.1	7371538	C	T
NC_040252.1	7425146	T	C
NC_040252.1	7473604	A	G
NC_040252.1	7486119	T	C
NC_040252.1	7526708	T	A
NC_040252.1	7544472	A	G
NC_040252.1	7573863	T	C
NC_040252.1	7584588	A	G
NC_040252.1	7599706	G	A
NC_040252.1	7604845	A	G
NC_040252.1	7610055	T	C
NC_040252.1	7655032	C	A
NC_040252.1	7698645	0	C
NC_040252.1	7742179	C	T
NC_040252.1	7782070	G	A
NC_040252.1	7790272	T	C
NC_040252.1	7795012	A	G
NC_040252.1	7797117	T	C
NC_040252.1	7822355	G	A
NC_040252.1	7839019	G	A
NC_040252.1	7895078	C	T

NC_040252.1	7897012	A	C
NC_040252.1	7922406	G	C
NC_040252.1	7953637	G	A
NC_040252.1	8006654	A	G
NC_040252.1	8064655	A	G
NC_040252.1	8114047	A	G
NC_040252.1	8136240	A	G
NC_040252.1	8136435	A	C
NC_040252.1	8185727	A	G
NC_040252.1	8256500	C	G
NC_040252.1	8284312	G	A
NC_040252.1	8295296	T	C
NC_040252.1	8338937	G	A
NC_040252.1	8355772	T	C
NC_040252.1	8410539	A	C
NC_040252.1	8429527	T	C
NC_040252.1	8469448	G	A
NC_040252.1	8490005	T	C
NC_040252.1	8532425	A	G
NC_040252.1	8567728	A	G
NC_040252.1	8572217	T	C
NC_040252.1	8581952	T	G
NC_040252.1	8585383	A	G
NC_040252.1	8587679	G	A
NC_040252.1	8643055	T	C
NC_040252.1	8699446	A	G
NC_040252.1	8715791	A	G
NC_040252.1	8768173	T	C
NC_040252.1	8823155	T	C
NC_040252.1	8879929	A	C
NC_040252.1	8933154	A	C
NC_040252.1	8980085	T	C
NC_040252.1	9038178	T	C
NC_040252.1	9090905	A	G
NC_040252.1	9143751	T	C
NC_040252.1	9171563	T	C
NC_040252.1	9226296	A	G
NC_040252.1	9282884	T	C
NC_040252.1	9301220	C	T
NC_040252.1	9339554	C	A
NC_040252.1	9353229	G	A
NC_040252.1	9385860	T	C
NC_040252.1	9416370	T	C
NC_040252.1	9443269	T	C
NC_040252.1	9503123	A	C
NC_040252.1	9524681	A	G
NC_040252.1	9707041	C	A

NC_040252.1	9761589	G	A
NC_040252.1	9800311	C	T
NC_040252.1	9881441	C	T
NC_040252.1	9937278	G	A
NC_040252.1	9984598	T	G
NC_040252.1	9992754	A	G
NC_040252.1	10045721	C	A
NC_040252.1	10075395	T	C
NC_040252.1	10077812	G	C
NC_040252.1	10077987	A	G
NC_040252.1	10104133	C	T
NC_040252.1	10119733	A	G
NC_040252.1	10170468	T	C
NC_040252.1	10181919	G	A
NC_040252.1	10182309	A	G
NC_040252.1	10222520	T	C
NC_040252.1	10222797	C	A
NC_040252.1	10223586	G	A
NC_040252.1	10258493	A	G
NC_040252.1	10301230	C	T
NC_040252.1	10304153	C	T
NC_040252.1	10309066	C	T
NC_040252.1	10314020	C	T
NC_040252.1	10315241	A	T
NC_040252.1	10335501	C	T
NC_040252.1	10383920	G	A
NC_040252.1	10397013	A	G
NC_040252.1	10399030	C	T
NC_040252.1	10399474	C	T
NC_040252.1	10400017	G	A
NC_040252.1	10410186	G	A
NC_040252.1	10411308	C	A
NC_040252.1	10411500	C	T
NC_040252.1	10432328	A	G
NC_040252.1	10450964	A	G
NC_040252.1	10453095	T	G
NC_040252.1	10453943	C	T
NC_040252.1	10454391	C	T
NC_040252.1	10455214	T	A
NC_040252.1	10461951	A	T
NC_040252.1	10517865	C	A
NC_040252.1	10551505	A	G
NC_040252.1	10580812	C	G
NC_040252.1	10641301	C	T
NC_040252.1	10687231	G	A
NC_040252.1	10758651	A	T
NC_040252.1	10786487	T	C

NC_040252.1	10786790	C	T
NC_040252.1	10787462	C	T
NC_040252.1	10787817	C	T
NC_040252.1	10788353	T	C
NC_040252.1	10788528	C	G
NC_040252.1	10809385	G	T
NC_040252.1	10810295	A	G
NC_040252.1	10866971	T	C
NC_040252.1	10875606	T	C
NC_040252.1	10911885	A	C
NC_040252.1	10915651	0	C
NC_040252.1	10928759	G	A
NC_040252.1	10947579	C	T
NC_040252.1	10984323	T	A
NC_040252.1	11019021	A	C
NC_040252.1	11048201	T	C
NC_040252.1	11065555	T	C
NC_040252.1	11065827	G	A
NC_040252.1	11072419	T	C
NC_040252.1	11077104	T	C
NC_040252.1	11087147	C	T
NC_040252.1	11089510	T	C
NC_040252.1	11093057	C	T
NC_040252.1	11146313	T	C
NC_040252.1	11162724	C	T
NC_040252.1	11162885	A	G
NC_040252.1	11163446	T	C
NC_040252.1	11163598	A	G
NC_040252.1	11164376	C	T
NC_040252.1	11165107	C	A
NC_040252.1	11165392	T	C
NC_040252.1	11165596	G	C
NC_040252.1	11225841	C	T
NC_040252.1	11227891	G	A
NC_040252.1	11229103	T	C
NC_040252.1	11229379	A	T
NC_040252.1	11229929	A	C
NC_040252.1	11230093	G	C
NC_040252.1	11230491	G	C
NC_040252.1	11264635	C	T
NC_040252.1	11275939	A	T
NC_040252.1	11295485	A	T
NC_040252.1	11338087	G	T
NC_040252.1	11342530	G	T
NC_040252.1	11351999	G	T
NC_040252.1	11358136	T	C
NC_040252.1	11359050	C	T

NC_040252.1	11360467	G	A
NC_040252.1	11360617	A	G
NC_040252.1	11361554	A	G
NC_040252.1	11362118	G	A
NC_040252.1	11362981	C	T
NC_040252.1	11363191	G	A
NC_040252.1	11378883	A	C
NC_040252.1	11382599	A	G
NC_040252.1	11382826	A	G
NC_040252.1	11384606	G	C
NC_040252.1	11384771	G	A
NC_040252.1	11386267	T	C
NC_040252.1	11386815	A	G
NC_040252.1	11387283	C	T
NC_040252.1	11387724	A	G
NC_040252.1	11442954	A	G
NC_040252.1	11451095	G	C
NC_040252.1	11451247	T	C
NC_040252.1	11453068	A	G
NC_040252.1	11455084	A	G
NC_040252.1	11482552	T	A
NC_040252.1	11539043	C	T
NC_040252.1	11546948	T	C
NC_040252.1	11550554	C	A
NC_040252.1	11550731	G	C
NC_040252.1	11551281	A	C
NC_040252.1	11551607	A	G
NC_040252.1	11564830	T	C
NC_040252.1	11590627	T	C
NC_040252.1	11611515	T	C
NC_040252.1	11658749	C	G
NC_040252.1	11667326	T	G
NC_040252.1	11673758	A	G
NC_040252.1	11676237	T	G
NC_040252.1	11682835	T	C
NC_040252.1	11734870	G	A
NC_040252.1	11760687	T	C
NC_040252.1	11789927	A	G
NC_040252.1	11824343	A	G
NC_040252.1	11847802	C	A
NC_040252.1	11903565	G	A
NC_040252.1	11939384	A	G
NC_040252.1	12003098	G	A
NC_040252.1	12018071	G	A
NC_040252.1	12023491	T	C
NC_040252.1	12077981	C	T
NC_040252.1	12093407	A	G

NC_040252.1	12146933	T	G
NC_040252.1	12202390	C	G
NC_040252.1	12257071	T	G
NC_040252.1	12313558	G	A
NC_040252.1	12371279	A	G
NC_040252.1	12426151	C	T
NC_040252.1	12467184	C	T
NC_040252.1	12509183	A	C
NC_040252.1	12525445	G	A
NC_040252.1	12674943	A	G
NC_040252.1	12728450	A	G
NC_040252.1	12765752	T	C
NC_040252.1	12765937	C	T
NC_040252.1	12772893	A	T
NC_040252.1	12783777	G	A
NC_040252.1	12820057	G	A
NC_040252.1	12820255	T	C
NC_040252.1	12820645	T	C
NC_040252.1	12822877	A	G
NC_040252.1	12841885	A	G
NC_040252.1	12845366	C	T
NC_040252.1	12846067	T	C
NC_040252.1	12848869	T	C
NC_040252.1	12853168	C	G
NC_040252.1	12870122	A	G
NC_040252.1	12886819	A	G
NC_040252.1	12888450	T	C
NC_040252.1	12901628	T	G
NC_040252.1	12964804	A	G
NC_040252.1	12979688	A	G
NC_040252.1	12982530	A	G
NC_040252.1	12983222	T	C
NC_040252.1	13021784	T	G
NC_040252.1	13057475	C	T
NC_040252.1	13058611	C	T
NC_040252.1	13058957	A	G
NC_040252.1	13065310	G	A
NC_040252.1	13065999	C	A
NC_040252.1	13066426	T	C
NC_040252.1	13067614	A	G
NC_040252.1	13070001	T	C
NC_040252.1	13075160	T	C
NC_040252.1	13112899	T	C
NC_040252.1	13113065	T	G
NC_040252.1	13119159	A	G
NC_040252.1	13129481	C	T
NC_040252.1	13154115	T	C

NC_040252.1	13162063	T	C
NC_040252.1	13163481	T	C
NC_040252.1	13208130	A	G
NC_040252.1	13297441	G	A
NC_040252.1	13335933	T	C
NC_040252.1	13368250	C	A
NC_040252.1	13392619	T	C
NC_040252.1	13448913	T	C
NC_040252.1	13482629	C	T
NC_040252.1	13509487	T	C
NC_040252.1	13566663	T	G
NC_040252.1	13626658	A	T
NC_040252.1	13680631	C	T
NC_040252.1	13726006	C	T
NC_040252.1	13737545	C	G
NC_040252.1	13795203	A	T
NC_040252.1	13852153	T	C
NC_040252.1	13908080	T	C
NC_040252.1	13958751	A	G
NC_040252.1	14014451	T	C
NC_040252.1	14073133	A	G
NC_040252.1	14130863	C	T
NC_040252.1	14143253	A	G
NC_040252.1	14143433	T	C
NC_040252.1	14143638	T	C
NC_040252.1	14201137	C	G
NC_040252.1	14244714	G	A
NC_040252.1	14266125	A	G
NC_040252.1	14297654	T	C
NC_040252.1	14335211	G	C
NC_040252.1	14390768	C	T
NC_040252.1	14446189	A	G
NC_040252.1	14497962	C	G
NC_040252.1	14514904	T	C
NC_040252.1	14524976	A	G
NC_040252.1	14543932	A	G
NC_040252.1	14550363	A	G
NC_040252.1	14551102	C	T
NC_040252.1	14582416	A	G
NC_040252.1	14589263	T	C
NC_040252.1	14607237	G	A
NC_040252.1	14608622	C	A
NC_040252.1	14609157	T	C
NC_040252.1	14609396	A	G
NC_040252.1	14609628	T	C
NC_040252.1	14613162	A	G
NC_040252.1	14619817	T	C

NC_040252.1	14625169	T	C
NC_040252.1	14650033	T	C
NC_040252.1	14656701	T	C
NC_040252.1	14676139	C	G
NC_040252.1	14720822	A	T
NC_040252.1	14721500	C	T
NC_040252.1	14767964	G	A
NC_040252.1	14807789	T	C
NC_040252.1	14809146	G	A
NC_040252.1	14809986	G	C
NC_040252.1	14864609	A	G
NC_040252.1	14878622	A	G
NC_040252.1	14926012	G	A
NC_040252.1	14940685	A	G
NC_040252.1	14978395	C	T
NC_040252.1	15016790	G	A
NC_040252.1	15017300	T	C
NC_040252.1	15020220	T	C
NC_040252.1	15060737	G	A
NC_040252.1	15071320	A	G
NC_040252.1	15073324	A	G
NC_040252.1	15077241	A	C
NC_040252.1	15077469	A	G
NC_040252.1	15134280	A	G
NC_040252.1	15167787	T	G
NC_040252.1	15199914	A	T
NC_040252.1	15223088	T	C
NC_040252.1	15226880	G	A
NC_040252.1	15227155	G	A
NC_040252.1	15229356	A	G
NC_040252.1	15239284	C	G
NC_040252.1	15253844	A	G
NC_040252.1	15335288	C	G
NC_040252.1	15393000	A	G
NC_040252.1	15415937	A	G
NC_040252.1	15417149	G	A
NC_040252.1	15426942	C	T
NC_040252.1	15488686	T	C
NC_040252.1	15492319	C	T
NC_040252.1	15503323	T	C
NC_040252.1	15553511	C	A
NC_040252.1	15553785	G	C
NC_040252.1	15589054	T	C
NC_040252.1	15621813	T	C
NC_040252.1	15662423	T	C
NC_040252.1	15662766	G	A
NC_040252.1	15687599	C	T

NC_040252.1	15702454	A	T
NC_040252.1	15752379	T	C
NC_040252.1	15778169	T	C
NC_040252.1	15833806	G	A
NC_040252.1	15880467	C	G
NC_040252.1	15931347	C	A
NC_040252.1	15951438	A	C
NC_040252.1	15975722	C	G
NC_040252.1	16031996	A	G
NC_040252.1	16095835	C	G
NC_040252.1	16097027	T	C
NC_040252.1	16123513	G	A
NC_040252.1	16129938	A	G
NC_040252.1	16184750	C	T
NC_040252.1	16239932	C	T
NC_040252.1	16264098	A	T
NC_040252.1	16320384	T	C
NC_040252.1	16351193	C	A
NC_040252.1	16410342	C	T
NC_040252.1	16463056	A	G
NC_040252.1	16480282	A	G
NC_040252.1	16499104	T	G
NC_040252.1	16499401	C	T
NC_040252.1	16556077	C	T
NC_040252.1	16604311	A	G
NC_040252.1	16639983	A	G
NC_040252.1	16643176	A	G
NC_040252.1	16643858	G	C
NC_040252.1	16646092	G	T
NC_040252.1	16693961	A	G
NC_040252.1	16694541	T	C
NC_040252.1	16697081	C	T
NC_040252.1	16742336	A	G
NC_040252.1	16778151	A	G
NC_040252.1	16802332	T	C
NC_040252.1	16834422	A	G
NC_040252.1	16890565	C	G
NC_040252.1	16945278	A	G
NC_040252.1	16996436	T	C
NC_040252.1	17050748	C	T
NC_040252.1	17107369	A	G
NC_040252.1	17137493	A	T
NC_040252.1	17164229	A	G
NC_040252.1	17222767	C	A
NC_040252.1	17254638	C	T
NC_040252.1	17275650	A	G
NC_040252.1	17295515	T	C

NC_040252.1	17296269	T	C
NC_040252.1	17297132	C	G
NC_040252.1	17297389	C	A
NC_040252.1	17311074	C	A
NC_040252.1	17311249	T	C
NC_040252.1	17365875	T	C
NC_040252.1	17423260	C	T
NC_040252.1	17442124	T	G
NC_040252.1	17470954	C	T
NC_040252.1	17501742	T	A
NC_040252.1	17520948	T	A
NC_040252.1	17539611	0	C
NC_040252.1	17549353	T	C
NC_040252.1	17606462	C	A
NC_040252.1	17668926	C	T
NC_040252.1	17722463	T	G
NC_040252.1	17747431	C	T
NC_040252.1	17792647	T	C
NC_040252.1	17810779	G	A
NC_040252.1	17824015	T	C
NC_040252.1	17869088	T	C
NC_040252.1	17869277	A	C
NC_040252.1	17907554	G	T
NC_040252.1	17907718	A	C
NC_040252.1	17912566	G	A
NC_040252.1	17916032	C	T
NC_040252.1	17962406	C	T
NC_040252.1	17962856	A	T
NC_040252.1	17963545	A	G
NC_040252.1	17965892	A	C
NC_040252.1	17967313	A	G
NC_040252.1	17982738	A	G
NC_040252.1	18035421	C	T
NC_040252.1	18078048	A	C
NC_040252.1	18100007	C	T
NC_040252.1	18120730	C	G
NC_040252.1	18171895	G	T
NC_040252.1	18215735	C	G
NC_040252.1	18254037	A	C
NC_040252.1	18282842	C	T
NC_040252.1	18321004	C	T
NC_040252.1	18348436	C	T
NC_040252.1	18370429	T	C
NC_040252.1	18398806	A	G
NC_040252.1	18399101	A	G
NC_040252.1	18399501	G	A
NC_040252.1	18433924	T	C

NC_040252.1	18446340	C	G
NC_040252.1	18502580	G	A
NC_040252.1	18525999	C	T
NC_040252.1	18528650	A	G
NC_040252.1	18528867	G	A
NC_040252.1	18539061	A	G
NC_040252.1	18539236	G	A
NC_040252.1	18540986	A	T
NC_040252.1	18575893	T	C
NC_040252.1	18610548	T	C
NC_040252.1	18616077	C	T
NC_040252.1	18616290	T	C
NC_040252.1	18619184	C	G
NC_040252.1	18632276	T	C
NC_040252.1	18632579	G	A
NC_040252.1	18635061	T	C
NC_040252.1	18657403	T	C
NC_040252.1	18657657	T	C
NC_040252.1	18680845	A	G
NC_040252.1	18685305	T	C
NC_040252.1	18685845	T	C
NC_040252.1	18686148	T	C
NC_040252.1	18686467	A	G
NC_040252.1	18695244	A	G
NC_040252.1	18700304	T	C
NC_040252.1	18706906	T	C
NC_040252.1	18710211	G	A
NC_040252.1	18710384	C	T
NC_040252.1	18710544	C	G
NC_040252.1	18712089	A	G
NC_040252.1	18741792	A	G
NC_040252.1	18783891	G	A
NC_040252.1	18849136	G	C
NC_040252.1	18866318	A	G
NC_040252.1	18866529	A	G
NC_040252.1	18904541	T	C
NC_040252.1	18936714	G	C
NC_040252.1	19001043	A	G
NC_040252.1	19059853	T	C
NC_040252.1	19112747	A	G
NC_040252.1	19160347	G	C
NC_040252.1	19160877	T	C
NC_040252.1	19163617	T	G
NC_040252.1	19166343	G	C
NC_040252.1	19177022	T	C
NC_040252.1	19198266	C	T
NC_040252.1	19199669	C	T

NC_040252.1	19200548	T	G
NC_040252.1	19207118	T	C
NC_040252.1	19210578	G	A
NC_040252.1	19217304	C	T
NC_040252.1	19222104	A	G
NC_040252.1	19253134	A	G
NC_040252.1	19277359	T	C
NC_040252.1	19345841	C	T
NC_040252.1	19356317	G	A
NC_040252.1	19422799	T	C
NC_040252.1	19458171	A	G
NC_040252.1	19459495	G	A
NC_040252.1	19465608	G	A
NC_040252.1	19467252	A	G
NC_040252.1	19520911	C	A
NC_040252.1	19573843	T	C
NC_040252.1	19600032	A	G
NC_040252.1	19654651	T	C
NC_040252.1	19707201	A	G
NC_040252.1	19763360	T	C
NC_040252.1	19821815	T	C
NC_040252.1	19877812	A	G
NC_040252.1	19901637	T	C
NC_040252.1	19912067	G	A
NC_040252.1	19913074	G	T
NC_040252.1	19920053	A	G
NC_040252.1	19966296	C	T
NC_040252.1	20087387	C	T
NC_040252.1	20091507	T	C
NC_040252.1	20096618	C	T
NC_040252.1	20128091	T	A
NC_040252.1	20128324	A	G
NC_040252.1	20129739	A	G
NC_040252.1	20145456	A	C
NC_040252.1	20148934	C	T
NC_040252.1	20149184	T	C
NC_040252.1	20149779	T	C
NC_040252.1	20150021	A	C
NC_040252.1	20154296	A	G
NC_040252.1	20163732	A	G
NC_040252.1	20166673	T	C
NC_040252.1	20167764	T	C
NC_040252.1	20184627	C	T
NC_040252.1	20185260	T	C
NC_040252.1	20237728	G	T
NC_040252.1	20285200	A	G
NC_040252.1	20316212	A	G

NC_040252.1	20322293	G	A
NC_040252.1	20323681	A	G
NC_040252.1	20329183	T	C
NC_040252.1	20329707	A	G
NC_040252.1	20330812	A	C
NC_040252.1	20331015	A	G
NC_040252.1	20332041	T	C
NC_040252.1	20332838	T	G
NC_040252.1	20359429	T	C
NC_040252.1	20388335	T	A
NC_040252.1	20447169	C	T
NC_040252.1	20504494	A	G
NC_040252.1	20530922	A	G
NC_040252.1	20561694	T	C
NC_040252.1	20592823	T	C
NC_040252.1	20628675	G	A
NC_040252.1	20632533	C	T
NC_040252.1	20634376	C	G
NC_040252.1	20641931	G	A
NC_040252.1	20642275	T	C
NC_040252.1	20642973	T	C
NC_040252.1	20643989	A	G
NC_040252.1	20645088	C	A
NC_040252.1	20698422	A	G
NC_040252.1	20737351	T	C
NC_040252.1	20775362	T	C
NC_040252.1	20788035	C	T
NC_040252.1	20789793	C	T
NC_040252.1	20794064	C	T
NC_040252.1	20796597	G	A
NC_040252.1	20807956	G	T
NC_040252.1	20846683	A	C
NC_040252.1	20847068	A	G
NC_040252.1	20855247	T	C
NC_040252.1	20899788	T	C
NC_040252.1	20899967	A	G
NC_040252.1	20903346	A	C
NC_040252.1	20903632	A	G
NC_040252.1	20962596	G	A
NC_040252.1	20982525	A	G
NC_040252.1	20991925	C	G
NC_040252.1	21001288	T	C
NC_040252.1	21010517	C	T
NC_040252.1	21012706	T	C
NC_040252.1	21016149	G	C
NC_040252.1	21072663	A	T
NC_040252.1	21128650	A	G

NC_040252.1	21188204	G	A
NC_040252.1	21243150	C	A
NC_040252.1	21253587	C	G
NC_040252.1	21266132	T	C
NC_040252.1	21267843	A	G
NC_040252.1	21268617	G	A
NC_040252.1	21270700	T	C
NC_040252.1	21271878	A	G
NC_040252.1	21272070	G	T
NC_040252.1	21277474	A	G
NC_040252.1	21278294	T	C
NC_040252.1	21278940	C	G
NC_040252.1	21279463	C	T
NC_040252.1	21288359	T	C
NC_040252.1	21307844	A	G
NC_040252.1	21380134	A	G
NC_040252.1	21439148	C	A
NC_040252.1	21467699	T	C
NC_040252.1	21469201	T	C
NC_040252.1	21469966	G	T
NC_040252.1	21472029	A	G
NC_040252.1	21472495	T	C
NC_040252.1	21472780	A	G
NC_040252.1	21523913	C	T
NC_040252.1	21529015	C	T
NC_040252.1	21535604	T	C
NC_040252.1	21544068	C	T
NC_040252.1	21545110	T	C
NC_040252.1	21545308	T	C
NC_040252.1	21546356	T	C
NC_040252.1	21583000	C	T
NC_040252.1	21594199	A	G
NC_040252.1	21595183	T	C
NC_040252.1	21622265	A	G
NC_040252.1	21655486	A	C
NC_040252.1	21692353	G	A
NC_040252.1	21726325	G	A
NC_040252.1	21776193	C	T
NC_040252.1	21824728	T	C
NC_040252.1	21825576	C	T
NC_040252.1	21826435	A	C
NC_040252.1	21878797	C	T
NC_040252.1	21881356	A	G
NC_040252.1	21881554	G	T
NC_040252.1	21888823	T	C
NC_040252.1	21889244	A	G
NC_040252.1	21889693	C	A

NC_040252.1	21889971	G	A
NC_040252.1	21890476	T	C
NC_040252.1	21891304	T	C
NC_040252.1	21896460	C	T
NC_040252.1	21904178	C	T
NC_040252.1	21926470	C	A
NC_040252.1	21926781	C	A
NC_040252.1	21972899	G	T
NC_040252.1	22001137	T	C
NC_040252.1	22014244	T	C
NC_040252.1	22015951	T	C
NC_040252.1	22053485	G	A
NC_040252.1	22079386	A	G
NC_040252.1	22121900	T	C
NC_040252.1	22148677	T	C
NC_040252.1	22208138	A	C
NC_040252.1	22271251	T	A
NC_040252.1	22295322	C	T
NC_040252.1	22337199	A	C
NC_040252.1	22380935	A	G
NC_040252.1	22398184	C	T
NC_040252.1	22424797	C	T
NC_040252.1	22426025	T	C
NC_040252.1	22426542	A	G
NC_040252.1	22427128	A	G
NC_040252.1	22427428	T	C
NC_040252.1	22433773	G	C
NC_040252.1	22439062	T	C
NC_040252.1	22439273	A	G
NC_040252.1	22439806	A	G
NC_040252.1	22440092	T	C
NC_040252.1	22458246	C	T
NC_040252.1	22458703	A	G
NC_040252.1	22473278	T	C
NC_040252.1	22480123	T	C
NC_040252.1	22515006	C	T
NC_040252.1	22540751	T	G
NC_040252.1	22594809	G	C
NC_040252.1	22611937	C	T
NC_040252.1	22613600	T	G
NC_040252.1	22669367	T	C
NC_040252.1	22700487	A	G
NC_040252.1	22727203	G	C
NC_040252.1	22784613	T	G
NC_040252.1	22835744	T	C
NC_040252.1	22892619	A	G
NC_040252.1	22919111	T	C

NC_040252.1	22952551	A	G
NC_040252.1	23008865	C	T
NC_040252.1	23064165	A	G
NC_040252.1	23121750	T	C
NC_040252.1	23166010	C	G
NC_040252.1	23222276	A	T
NC_040252.1	23272813	C	T
NC_040252.1	23320065	A	G
NC_040252.1	23377468	A	G
NC_040252.1	23401397	T	C
NC_040252.1	23449719	G	T
NC_040252.1	23455687	C	T
NC_040252.1	23455850	A	G
NC_040252.1	23463749	T	C
NC_040252.1	23482640	T	C
NC_040252.1	23537643	T	C
NC_040252.1	23588846	T	A
NC_040252.1	23640986	T	C
NC_040252.1	23666768	G	T
NC_040252.1	23697116	C	A
NC_040252.1	23697426	A	G
NC_040252.1	23733602	C	G
NC_040252.1	23770067	C	A
NC_040252.1	23808260	G	A
NC_040252.1	23865244	C	G
NC_040252.1	23909458	C	A
NC_040252.1	23925301	T	C
NC_040252.1	23957259	T	G
NC_040252.1	24012310	T	C
NC_040252.1	24068889	G	A
NC_040252.1	24125609	G	A
NC_040252.1	24181747	A	G
NC_040252.1	24232166	T	C
NC_040252.1	24270157	C	T
NC_040252.1	24314025	G	A
NC_040252.1	24370489	T	C
NC_040252.1	24429690	C	T
NC_040252.1	24561481	C	T
NC_040252.1	24695991	C	A
NC_040252.1	24751253	G	A
NC_040252.1	24804199	C	T
NC_040252.1	24893754	T	G
NC_040252.1	24950015	G	A
NC_040252.1	25172908	A	G
NC_040252.1	25242389	C	T
NC_040252.1	25266380	A	G
NC_040252.1	25372596	T	C

NC_040252.1	25436229	C	T
NC_040252.1	25685066	C	A
NC_040252.1	25769638	G	A
NC_040252.1	25801489	T	C
NC_040252.1	25830785	G	A
NC_040252.1	25858258	A	G
NC_040252.1	25908350	A	G
NC_040252.1	25959677	C	T
NC_040252.1	26073045	T	C
NC_040252.1	26110157	T	C
NC_040252.1	26166944	T	G
NC_040252.1	26224377	A	G
NC_040252.1	26275264	G	A
NC_040252.1	26302998	T	C
NC_040252.1	26311411	C	T
NC_040252.1	26357805	T	G
NC_040252.1	26397798	C	T
NC_040252.1	26444249	G	A
NC_040252.1	26456423	G	A
NC_040252.1	26488160	A	G
NC_040252.1	26517054	A	G
NC_040252.1	26536773	T	C
NC_040252.1	26537097	G	A
NC_040252.1	26537273	T	G
NC_040252.1	26537909	A	C
NC_040252.1	26581532	T	C
NC_040252.1	26638947	A	C
NC_040252.1	26695689	C	G
NC_040252.1	26751302	T	C
NC_040252.1	26810318	T	C
NC_040252.1	26865206	A	G
NC_040252.1	26920443	T	C
NC_040252.1	26973403	C	T
NC_040252.1	27033779	A	G
NC_040252.1	27034113	A	G
NC_040252.1	27091584	T	C
NC_040252.1	27141375	A	G
NC_040252.1	27185062	C	A
NC_040252.1	27220541	T	C
NC_040252.1	27241695	A	G
NC_040252.1	27289974	T	A
NC_040252.1	27325761	A	T
NC_040252.1	27334925	C	A
NC_040252.1	27335460	A	G
NC_040252.1	27386730	G	A
NC_040252.1	27394353	T	C
NC_040252.1	27395813	A	G

NC_040252.1	27399186	A	T
NC_040252.1	27456658	A	C
NC_040252.1	27512224	A	G
NC_040252.1	27542021	A	G
NC_040252.1	27596810	T	C
NC_040252.1	27643281	T	C
NC_040252.1	27699158	C	T
NC_040252.1	27755901	T	A
NC_040252.1	27807295	G	A
NC_040252.1	27820446	G	A
NC_040252.1	27877153	G	T
NC_040252.1	27883501	G	C
NC_040252.1	27887426	G	T
NC_040252.1	27887604	C	T
NC_040252.1	27929973	C	G
NC_040252.1	27962695	T	G
NC_040252.1	27963042	T	G
NC_040252.1	27964138	T	C
NC_040252.1	27965272	A	G
NC_040252.1	28021311	G	A
NC_040252.1	28065209	G	A
NC_040252.1	28086117	G	A
NC_040252.1	28089423	T	G
NC_040252.1	28105897	C	T
NC_040252.1	28107025	T	C
NC_040252.1	28111953	G	C
NC_040252.1	28161787	G	A
NC_040252.1	28215548	A	G
NC_040252.1	28251037	C	T
NC_040252.1	28303295	T	A
NC_040252.1	28345652	A	C
NC_040252.1	28348158	C	G
NC_040252.1	28350503	T	A
NC_040252.1	28355149	T	C
NC_040252.1	28356204	G	T
NC_040252.1	28360811	A	G
NC_040252.1	28367681	G	A
NC_040252.1	28388345	C	T
NC_040252.1	28390851	T	A
NC_040252.1	28409926	T	C
NC_040252.1	28427590	G	A
NC_040252.1	28427787	T	C
NC_040252.1	28428092	G	A
NC_040252.1	28429539	G	A
NC_040252.1	28438508	C	T
NC_040252.1	28442410	G	A
NC_040252.1	28443500	T	C

NC_040252.1	28499896	A	T
NC_040252.1	28555521	A	G
NC_040252.1	28593514	T	C
NC_040252.1	28629814	A	G
NC_040252.1	28679793	G	C
NC_040252.1	28711401	A	G
NC_040252.1	28760004	A	G
NC_040252.1	28829479	G	A
NC_040252.1	28830412	A	G
NC_040252.1	28831106	T	C
NC_040252.1	28832028	A	T
NC_040252.1	28833419	G	A
NC_040252.1	28883983	A	G
NC_040252.1	28940673	A	G
NC_040252.1	28981875	C	T
NC_040252.1	29019125	T	A
NC_040252.1	29069215	T	C
NC_040252.1	29107184	C	A
NC_040252.1	29158092	T	G
NC_040252.1	29192100	C	G
NC_040252.1	29211824	C	G
NC_040252.1	29227319	T	C
NC_040252.1	29267317	A	G
NC_040252.1	29325312	T	A
NC_040252.1	29345451	A	T
NC_040252.1	29345872	T	G
NC_040252.1	29347094	T	C
NC_040252.1	29347798	T	C
NC_040252.1	29361575	A	G
NC_040252.1	29381895	C	T
NC_040252.1	29382346	T	G
NC_040252.1	29383042	A	G
NC_040252.1	29384692	C	T
NC_040252.1	29385150	T	C
NC_040252.1	29398023	A	G
NC_040252.1	29402313	A	G
NC_040252.1	29406982	G	A
NC_040252.1	29462280	T	C
NC_040252.1	29521267	G	T
NC_040252.1	29584415	A	C
NC_040252.1	29632700	T	C
NC_040252.1	29689655	C	T
NC_040252.1	29719082	A	G
NC_040252.1	29777113	A	G
NC_040252.1	29838649	T	C
NC_040252.1	29879294	G	A
NC_040252.1	29903770	C	A

NC_040252.1	29931050	A		G
NC_040252.1	29966714	C		T
NC_040252.1	29976940	A		G
NC_040252.1	30027118	A		G
NC_040252.1	30046492	A		G
NC_040252.1	30046821	A		G
NC_040252.1	30102587	C		T
NC_040252.1	30155956	C		T
NC_040252.1	30180643	A		G
NC_040252.1	30181069	C		A
NC_040252.1	30223299	T		C
NC_040252.1	30293699	G		C
NC_040252.1	30293850	A		G
NC_040252.1	30295090	A		G
NC_040252.1	30299450	A		C
NC_040252.1	30339037	A		G
NC_040252.1	30346962	A		C
NC_040252.1	30400464	C		T
NC_040252.1	30455644	C		A
NC_040252.1	30510675	C		G
NC_040252.1	30550192	G		A
NC_040252.1	30583881	T		C
NC_040252.1	30641560	A		G
NC_040252.1	30712264	C		T
NC_040252.1	30712492	G		A
NC_040252.1	30737311	G		C
NC_040252.1	30745324	G		A
NC_040252.1	30786613	G		A
NC_040252.1	30789204	G		A
NC_040252.1	30789371	A		G
NC_040252.1	30808383	T		G
NC_040252.1	30811822	A		G
NC_040252.1	30869468	A		G
NC_040252.1	30869737	A		T
NC_040252.1	30879150	A		G
NC_040252.1	30879477	T		C
NC_040252.1	30879928	C		A
NC_040252.1	30880093	A		G
NC_040252.1	30911280	A		G
NC_040252.1	30961480	T		C
NC_040252.1	30963401	T		C
NC_040252.1	30996917	A		G
NC_040252.1	31028650	A		C
NC_040252.1	31086447	A		G
NC_040252.1	31122089	A		G
NC_040252.1	31189841		0	C
NC_040252.1	31240418	T		A

NC_040252.1	31269291	G	A
NC_040252.1	31302039	A	G
NC_040252.1	31357194	T	C
NC_040252.1	31386375	A	C
NC_040252.1	31416253	T	C
NC_040252.1	31474671	G	A
NC_040252.1	31530168	T	C
NC_040252.1	31586623	T	C
NC_040252.1	31644533	A	G
NC_040252.1	31700067	G	A
NC_040252.1	31732181	G	A
NC_040252.1	31763630	C	G
NC_040252.1	31820305	T	G
NC_040252.1	31879195	A	C
NC_040252.1	31919106	A	G
NC_040252.1	31981735	T	C
NC_040252.1	32037875	A	G
NC_040252.1	32096459	A	G
NC_040252.1	32143691	C	T
NC_040252.1	32178665	A	G
NC_040252.1	32205457	G	A
NC_040252.1	32261100	T	C
NC_040252.1	32294031	A	G
NC_040252.1	32326260	G	A
NC_040252.1	32391048	T	C
NC_040252.1	32446991	C	A
NC_040252.1	32503061	C	G
NC_040252.1	32556044	C	T
NC_040252.1	32599240	A	G
NC_040252.1	32641401	C	T
NC_040252.1	32694859	C	T
NC_040252.1	32726122	A	G
NC_040252.1	32766473	G	C
NC_040252.1	32788887	T	C
NC_040252.1	32842728	T	A
NC_040252.1	32872141	G	T
NC_040252.1	32873980	C	T
NC_040252.1	32874651	C	T
NC_040252.1	32914283	A	G
NC_040252.1	32969555	C	T
NC_040252.1	33023794	G	A
NC_040252.1	33077107	A	G
NC_040252.1	33117368	A	G
NC_040252.1	33144278	T	C
NC_040252.1	33192855	C	A
NC_040252.1	33210961	A	G
NC_040252.1	33211210	A	G

NC_040252.1	33211418	A	G
NC_040252.1	33231184	C	T
NC_040252.1	33287152	A	G
NC_040252.1	33340655	T	C
NC_040252.1	33397376	A	C
NC_040252.1	33468897	C	T
NC_040252.1	33538553	C	G
NC_040252.1	33573867	A	G
NC_040252.1	33603516	C	T
NC_040252.1	33663381	A	G
NC_040252.1	33714660	G	A
NC_040252.1	33765377	A	G
NC_040252.1	33822848	T	G
NC_040252.1	33877420	A	G
NC_040252.1	33931335	C	T
NC_040252.1	33962632	C	T
NC_040252.1	33995553	T	C
NC_040252.1	34050116	A	G
NC_040252.1	34082013	A	G
NC_040252.1	34110306	T	G
NC_040252.1	34166875	C	T
NC_040252.1	34228694	A	G
NC_040252.1	34281321	G	A
NC_040252.1	34332926	A	G
NC_040252.1	34387441	0	T
NC_040252.1	34445727	T	A
NC_040252.1	34498946	A	G
NC_040252.1	34550293	A	G
NC_040252.1	34605852	G	A
NC_040252.1	34659049	T	C
NC_040252.1	34694628	T	A
NC_040252.1	34731264	G	A
NC_040252.1	34787954	T	C
NC_040252.1	34825563	G	C
NC_040252.1	34885783	T	G
NC_040252.1	34929128	G	A
NC_040252.1	34976195	C	T
NC_040252.1	34976778	G	A
NC_040252.1	34977885	C	T
NC_040252.1	34978565	A	G
NC_040252.1	34978739	G	A
NC_040252.1	34979554	C	T
NC_040252.1	35002870	G	A
NC_040252.1	35003021	A	G
NC_040252.1	35038850	T	C
NC_040252.1	35064497	C	T
NC_040252.1	35098146	C	G

NC_040252.1	35098830	A	G
NC_040252.1	35099421	A	G
NC_040252.1	35156128	A	G
NC_040252.1	35211456	A	G
NC_040252.1	35265302	T	C
NC_040252.1	35321531	T	C
NC_040252.1	35369048	C	T
NC_040252.1	35416629	T	C
NC_040252.1	35470637	C	T
NC_040252.1	35523526	C	T
NC_040252.1	35572503	C	T
NC_040252.1	35634150	T	G
NC_040252.1	35747262	A	G
NC_040252.1	35857571	G	A
NC_040252.1	35898895	G	A
NC_040252.1	35926657	A	G
NC_040252.1	35930658	C	T
NC_040252.1	35984437	T	A
NC_040252.1	36042899	G	C
NC_040252.1	36100335	T	C
NC_040252.1	36157428	T	C
NC_040252.1	36208641	T	C
NC_040252.1	36252800	G	A
NC_040252.1	36309958	T	A
NC_040252.1	36354949	C	T
NC_040252.1	36431836	G	A
NC_040252.1	36490960	A	G
NC_040252.1	36503129	G	A
NC_040252.1	36514543	T	C
NC_040252.1	36581418	T	C
NC_040252.1	36643385	G	A
NC_040252.1	36683481	C	T
NC_040252.1	36693827	C	T
NC_040252.1	36724070	C	T
NC_040252.1	36783489	A	G
NC_040252.1	36788104	G	A
NC_040252.1	36802606	A	G
NC_040252.1	36859834	C	T
NC_040252.1	36860050	T	C
NC_040252.1	36907940	C	T
NC_040252.1	36943936	G	T
NC_040252.1	36979583	G	A
NC_040252.1	37007594	A	T
NC_040252.1	37061490	A	G
NC_040252.1	37119079	A	G
NC_040252.1	37172044	G	T
NC_040252.1	37204455	A	G

NC_040252.1	37233988	G	A
NC_040252.1	37261636	A	T
NC_040252.1	37301432	A	G
NC_040252.1	37349696	T	C
NC_040252.1	37400564	T	C
NC_040252.1	37456772	T	C
NC_040252.1	37506003	A	G
NC_040252.1	37511759	G	A
NC_040252.1	37570140	T	C
NC_040252.1	37620327	A	G
NC_040252.1	37672164	C	G
NC_040252.1	37726640	C	G
NC_040252.1	37780939	T	C
NC_040252.1	37837371	T	A
NC_040252.1	37892645	T	C
NC_040252.1	37951132	A	G
NC_040252.1	38003690	A	G
NC_040252.1	38049704	A	G
NC_040252.1	38106495	G	A
NC_040252.1	38158217	T	G
NC_040252.1	38201086	G	C
NC_040252.1	38257489	C	G
NC_040252.1	38312285	G	A
NC_040252.1	38368683	C	T
NC_040252.1	38424500	G	C
NC_040252.1	38480034	T	C
NC_040252.1	38539467	G	A
NC_040252.1	38596671	G	T
NC_040252.1	38597834	T	A
NC_040252.1	38598689	A	G
NC_040252.1	38598863	T	G
NC_040252.1	38602022	A	G
NC_040252.1	38603224	C	G
NC_040252.1	38603387	C	T
NC_040252.1	38658374	G	A
NC_040252.1	38713120	C	T
NC_040252.1	38763484	A	G
NC_040252.1	38798254	C	T
NC_040252.1	38848019	G	T
NC_040252.1	38857425	T	A
NC_040252.1	38901581	G	T
NC_040252.1	38958082	C	T
NC_040252.1	39012626	T	A
NC_040252.1	39062190	G	A
NC_040252.1	39120109	C	G
NC_040252.1	39160722	C	A
NC_040252.1	39207073	A	C

NC_040252.1	39305776	G	A
NC_040252.1	39312857	T	C
NC_040252.1	39313040	T	G
NC_040252.1	39313346	A	G
NC_040252.1	39350745	A	G
NC_040252.1	39405109	C	A
NC_040252.1	39418074	T	G
NC_040252.1	39522616	G	A
NC_040252.1	39557590	A	C
NC_040252.1	39598202	G	A
NC_040252.1	39619761	G	A
NC_040252.1	39635031	C	T
NC_040252.1	39639227	C	T
NC_040252.1	39644312	A	C
NC_040252.1	39645033	A	G
NC_040252.1	39645225	C	T
NC_040252.1	39704540	G	C
NC_040252.1	39759615	C	A
NC_040252.1	39819014	T	C
NC_040252.1	39824531	G	A
NC_040252.1	39832023	A	G
NC_040252.1	39845263	G	A
NC_040252.1	39861869	T	C
NC_040252.1	39870396	C	T
NC_040252.1	39885237	A	G
NC_040252.1	39942365	T	C
NC_040252.1	39995226	G	A
NC_040252.1	40021454	T	G
NC_040252.1	40068420	A	G
NC_040252.1	40097079	C	T
NC_040252.1	40147174	C	T
NC_040252.1	40192597	T	C
NC_040252.1	40249105	G	T
NC_040252.1	40305310	G	A
NC_040252.1	40359714	T	G
NC_040252.1	40413926	G	A
NC_040252.1	40470281	C	T
NC_040252.1	40522646	G	C
NC_040252.1	40570568	C	T
NC_040252.1	40628428	C	G
NC_040252.1	40673620	A	G
NC_040252.1	40704992	T	C
NC_040252.1	40705343	A	G
NC_040252.1	40760359	C	T
NC_040252.1	40811472	G	C
NC_040252.1	40875854	G	A
NC_040252.1	40937317	C	T

NC_040252.1	40991905	G	A
NC_040252.1	41018965	T	A
NC_040252.1	41074478	T	G
NC_040252.1	41131068	T	G
NC_040252.1	41189000	T	C
NC_040252.1	41239538	A	C
NC_040252.1	41295886	T	C
NC_040252.1	41341323	G	A
NC_040252.1	41394669	C	T
NC_040252.1	41450864	A	G
NC_040252.1	41502213	T	C
NC_040252.1	41556616	G	A
NC_040252.1	41585074	C	T
NC_040252.1	41585903	A	G
NC_040252.1	41586235	A	C
NC_040252.1	41607116	A	C
NC_040252.1	41668420	G	A
NC_040252.1	41668790	A	G
NC_040252.1	41710540	A	G
NC_040252.1	41754881	T	C
NC_040252.1	41763208	T	C
NC_040252.1	41818721	G	T
NC_040252.1	41877123	G	A
NC_040252.1	41916228	T	C
NC_040252.1	41971076	T	G
NC_040252.1	42026134	A	G
NC_040252.1	42073686	C	T
NC_040252.1	42098146	G	A
NC_040252.1	42153739	G	A
NC_040252.1	42153955	G	C
NC_040252.1	42154154	A	G
NC_040252.1	42215332	A	G
NC_040252.1	42273616	T	C
NC_040252.1	42324169	A	C
NC_040252.1	42364788	G	A
NC_040252.1	42375178	A	G
NC_040252.1	42431271	A	G
NC_040252.1	42493053	G	T
NC_040252.1	42547198	T	G
NC_040252.1	42595962	T	C
NC_040252.1	42629709	G	A
NC_040252.1	42670851	T	C
NC_040252.1	42692172	G	C
NC_040252.1	42748603	A	G
NC_040252.1	42781932	T	C
NC_040252.1	42782330	C	T
NC_040252.1	42825331	T	C

NC_040252.1	42833753	A	G
NC_040252.1	42889793	T	C
NC_040252.1	42964458	A	G
NC_040252.1	42966277	T	C
NC_040252.1	43001364	T	C
NC_040252.1	43004530	T	C
NC_040252.1	43005062	C	T
NC_040252.1	43057096	A	C
NC_040252.1	43103041	G	A
NC_040252.1	43179498	G	A
NC_040252.1	43197353	G	A
NC_040252.1	43267100	A	G
NC_040252.1	43276947	T	C
NC_040252.1	43545820	G	C
NC_040252.1	43576217	T	C
NC_040252.1	43597416	G	A
NC_040252.1	43648406	T	C
NC_040252.1	43677745	A	G
NC_040252.1	43716864	A	G
NC_040252.1	43773433	T	A
NC_040252.1	43831655	G	C
NC_040252.1	43903215	T	C
NC_040252.1	43956214	T	C
NC_040252.1	44005395	C	G
NC_040252.1	44065108	T	C
NC_040252.1	44123127	C	T
NC_040252.1	44184519	T	C
NC_040252.1	44219121	A	G
NC_040252.1	44262710	C	T
NC_040252.1	44269015	C	A
NC_040252.1	44316146	A	G
NC_040252.1	44363417	T	C
NC_040252.1	44373951	A	G
NC_040252.1	44430678	C	T
NC_040252.1	44474700	C	T
NC_040252.1	44526330	C	G
NC_040252.1	44588944	T	C
NC_040252.1	44603523	0	G
NC_040252.1	44632937	C	T
NC_040252.1	44687050	0	C
NC_040252.1	44818721	T	A
NC_040252.1	44853318	C	T
NC_040252.1	44854056	A	G
NC_040252.1	44855060	A	G
NC_040252.1	44913183	C	A
NC_040252.1	44954213	T	C
NC_040252.1	45003875	A	G

NC_040252.1	45053331	G	A
NC_040252.1	45081984	A	G
NC_040252.1	45123366	C	T
NC_040252.1	45161624	A	G
NC_040252.1	45214415	T	A
NC_040252.1	45234976	C	T
NC_040252.1	45244850	C	T
NC_040252.1	45259532	A	G
NC_040252.1	45313495	A	G
NC_040252.1	45363100	G	A
NC_040252.1	45412566	G	A
NC_040252.1	45473090	C	A
NC_040252.1	45512565	A	C
NC_040252.1	45559484	C	T
NC_040252.1	45619141	G	C
NC_040252.1	45660079	T	C
NC_040252.1	45698953	G	A
NC_040252.1	45739737	A	G
NC_040252.1	45803226	T	G
NC_040252.1	45835770	A	G
NC_040252.1	45893952	A	T
NC_040252.1	45908148	C	T
NC_040252.1	45951254	A	G
NC_040252.1	45974134	A	T
NC_040252.1	46022309	T	C
NC_040252.1	46082753	A	G
NC_040252.1	46115321	A	G
NC_040252.1	46142836	C	A
NC_040252.1	46175473	A	G
NC_040252.1	46223358	G	C
NC_040252.1	46266308	T	G
NC_040252.1	46302612	T	C
NC_040252.1	46366441	G	A
NC_040252.1	46373200	T	C
NC_040252.1	46397128	C	T
NC_040252.1	46418148	A	G
NC_040252.1	46880228	C	T
NC_040252.1	47008852	A	G
NC_040252.1	47052226	C	T
NC_040252.1	47090260	G	C
NC_040252.1	47131300	G	C
NC_040252.1	47209955	G	A
NC_040252.1	47259359	A	C
NC_040252.1	47350633	C	T
NC_040252.1	47613743	C	G
NC_040252.1	47739033	G	A
NC_040252.1	47887650	G	A

NC_040252.1	47993640	G	T
NC_040252.1	48119819	G	A
NC_040252.1	48194540	T	C
NC_040252.1	48252663	G	A
NC_040252.1	48341145	C	T
NC_040252.1	48392119	T	C
NC_040252.1	48438934	T	G
NC_040252.1	48458177	A	G
NC_040252.1	48507539	C	G
NC_040252.1	48573250	A	C
NC_040252.1	48658573	G	A
NC_040252.1	48701103	C	A
NC_040252.1	48731331	C	T
NC_040252.1	48766220	A	C
NC_040252.1	48874146	A	G
NC_040252.1	48901472	C	T
NC_040252.1	48990691	T	C
NC_040252.1	49058199	C	T
NC_040252.1	49067759	C	G
NC_040252.1	49104678	C	T
NC_040252.1	49147708	A	G
NC_040252.1	49259429	A	C
NC_040252.1	49326444	T	A
NC_040252.1	49510364	G	A
NC_040252.1	49527390	T	C
NC_040252.1	49693215	C	T
NC_040252.1	49712492	A	G
NC_040252.1	49832577	A	G
NC_040252.1	49906607	T	C
NC_040252.1	49995372	A	G
NC_040252.1	50061282	A	G
NC_040252.1	50136453	T	C
NC_040252.1	50204086	G	A
NC_040252.1	50286453	C	T
NC_040252.1	50340833	C	T
NC_040252.1	50470799	G	A
NC_040252.1	50567994	A	G
NC_040252.1	50624374	T	C
NC_040252.1	51172436	A	T
NC_040252.1	51692020	T	G
NC_040252.1	51977145	C	T
NC_040252.1	52033156	C	T
NC_040252.1	52272560	T	C
NC_040252.1	52370101	T	C
NC_040252.1	52683756	A	G
NC_040252.1	52856092	A	C
NC_040252.1	52989533	A	G

NC_040252.1	53089511	T	A
NC_040252.1	53268509	G	A
NC_040252.1	53393067	T	C
NC_040252.1	53443646	A	G
NC_040252.1	53834091	A	G
NC_040252.1	54077869	C	T
NC_040252.1	54138910	T	C
NC_040252.1	54211891	C	T
NC_040252.1	54250732	G	A
NC_040252.1	54307239	T	G
NC_040252.1	54351725	C	T
NC_040252.1	54425760	G	C
NC_040252.1	54488335	G	A
NC_040252.1	54654365	A	T
NC_040252.1	55098337	C	T
NC_040252.1	55184492	A	G
NC_040252.1	55295524	T	G
NC_040252.1	55339250	T	C
NC_040252.1	55415828	A	T
NC_040252.1	55555399	G	T
NC_040252.1	56107567	T	G
NC_040252.1	56300960	A	G
NC_040252.1	56468106	G	C
NC_040252.1	56612668	A	T
NC_040252.1	56730408	G	A
NC_040252.1	56823751	A	C
NC_040252.1	56994331	G	T
NC_040252.1	57209335	T	C
NC_040252.1	57424791	G	A
NC_040252.1	57546658	C	T
NC_040252.1	57740734	A	G
NC_040252.1	57867808	G	T
NC_040252.1	58238662	T	C
NC_040252.1	58297704	C	T
NC_040252.1	58335605	G	A
NC_040252.1	58400886	C	T
NC_040252.1	58532798	G	A
NC_040252.1	58754900	C	T
NC_040252.1	58817152	C	T
NC_040252.1	58946967	A	G
NC_040252.1	58975620	C	G
NC_040252.1	59077757	G	T
NC_040252.1	59223296	A	G
NC_040252.1	59736727	A	G
NC_040252.1	59997875	A	G
NC_040252.1	60973352	A	G
NC_040252.1	61576474	G	A

NC_040252.1	62247067	T	C
NC_040252.1	62486308	C	A
NC_040252.1	62649347	T	C
NC_040252.1	63012868	C	T
NC_040252.1	63254740	C	A
NC_040252.1	64164477	C	T
NC_040252.1	64217800	G	A
NC_040252.1	64280206	C	T
NC_040252.1	64350102	T	C
NC_040252.1	64410952	A	G
NC_040252.1	64469525	T	C
NC_040252.1	64493897	T	C
NC_040252.1	64564316	A	G
NC_040252.1	64788477	T	C
NC_040252.1	64850895	A	G
NC_040252.1	64915964	G	A
NC_040252.1	64975827	A	T
NC_040252.1	65001132	T	C
NC_040252.1	65058014	T	C
NC_040252.1	65110412	C	G
NC_040252.1	65175741	T	C
NC_040252.1	65256610	T	C
NC_040252.1	65264553	C	T
NC_040252.1	65291791	A	G
NC_040252.1	65810521	T	C
NC_040252.1	66100935	T	C
NC_040252.1	66718307	G	C
NC_040252.1	66799813	T	C
NC_040252.1	66931592	T	C
NC_040252.1	67042324	C	T
NC_040252.1	67523619	T	C
NC_040252.1	67995581	T	A
NC_040252.1	68759785	T	C
NC_040252.1	68871492	T	C
NC_040252.1	68929201	A	G
NC_040252.1	68993669	C	G
NC_040252.1	69052988	G	C
NC_040252.1	69115981	T	C
NC_040252.1	69296536	G	A
NC_040252.1	69355126	G	A
NC_040252.1	69411647	G	C
NC_040252.1	69474906	T	C
NC_040252.1	69535055	A	G
NC_040252.1	69597429	A	C
NC_040252.1	69666581	T	C
NC_040252.1	69724998	A	G
NC_040252.1	69779717	C	T

NC_040252.1	69843276	C	T
NC_040252.1	69899761	A	C
NC_040252.1	69941840	T	C
NC_040252.1	70749340	C	T
NC_040252.1	71019584	T	C
NC_040252.1	71281422	A	G
NC_040252.1	71572695	T	C
NC_040252.1	71773731	C	T
NC_040252.1	72174960	A	G
NC_040252.1	72332054	T	C
NC_040252.1	73134487	C	T
NC_040252.1	73567092	G	A
NC_040252.1	73722330	A	G
NC_040252.1	73857429	T	C
NC_040252.1	73999355	T	C
NC_040252.1	74165080	T	G
NC_040252.1	74859657	G	A
NC_040252.1	75088243	G	C
NC_040252.1	75325668	T	C
NC_040252.1	76273588	G	C
NC_040252.1	76476254	T	C
NC_040252.1	76657934	A	G
NC_040252.1	76963172	C	G
NC_040252.1	77709119	T	C
NC_040252.1	78169156	G	A
NC_040252.1	78806097	T	C
NC_040252.1	78872742	G	T
NC_040252.1	78943193	G	A
NC_040252.1	79287372	T	G
NC_040252.1	79356825	T	C
NC_040252.1	79622845	G	A
NC_040252.1	79689463	T	C
NC_040252.1	79754772	C	T
NC_040252.1	79821607	A	G
NC_040252.1	80029845	T	G
NC_040252.1	80089972	A	G
NC_040252.1	80160929	C	T
NC_040252.1	80276264	C	T
NC_040252.1	80304324	G	A
NC_040252.1	80358576	T	C
NC_040252.1	80411011	A	G
NC_040252.1	80484273	A	G
NC_040252.1	80545324	G	A
NC_040252.1	80810278	G	A
NC_040252.1	80861178	G	A
NC_040252.1	80920342	C	T
NC_040252.1	80989175	T	C

NC_040252.1	81057866	C	T
NC_040252.1	81133844	C	T
NC_040252.1	81207187	C	A
NC_040252.1	81264892	A	G
NC_040252.1	81316862	T	C
NC_040252.1	81369834	A	C
NC_040252.1	81438478	A	G
NC_040252.1	81510444	C	T
NC_040252.1	81577527	G	T
NC_040252.1	81639441	T	C
NC_040252.1	81703441	G	T
NC_040252.1	81759900	G	A
NC_040252.1	81822999	G	A
NC_040252.1	81884603	C	T
NC_040252.1	82001646	G	A
NC_040252.1	82054519	A	G
NC_040252.1	82120661	G	A
NC_040252.1	82180993	G	A
NC_040252.1	82243780	G	A
NC_040252.1	82309900	A	G
NC_040252.1	82368835	C	T
NC_040252.1	82429692	C	A
NC_040252.1	82489194	C	T
NC_040252.1	82556147	G	A
NC_040252.1	82622035	T	G
NC_040252.1	82681078	T	G
NC_040252.1	82738650	A	G
NC_040252.1	82805145	G	A
NC_040252.1	82867046	A	G
NC_040252.1	82926439	T	C
NC_040252.1	82990076	G	A
NC_040252.1	83056015	G	A
NC_040252.1	83114045	T	C
NC_040252.1	83172248	C	A
NC_040252.1	83238625	T	C
NC_040252.1	83298372	T	C
NC_040252.1	83362636	G	C
NC_040252.1	83416601	G	A
NC_040252.1	83481273	C	A
NC_040252.1	83540225	C	T
NC_040252.1	83604870	A	G
NC_040252.1	83661068	C	T
NC_040252.1	83731431	G	A
NC_040252.1	83789298	A	C
NC_040252.1	83843718	T	G
NC_040252.1	83906003	G	T
NC_040252.1	83975701	T	C

NC_040252.1	84052604	A	G
NC_040252.1	84107893	T	C
NC_040252.1	84160008	A	C
NC_040252.1	84217524	G	C
NC_040252.1	84271808	T	C
NC_040252.1	84298514	A	G
NC_040252.1	84345570	T	G
NC_040252.1	84377629	A	G
NC_040252.1	84417267	T	C
NC_040252.1	84482340	T	C
NC_040252.1	84542541	G	A
NC_040252.1	84667116	C	T
NC_040252.1	84726181	A	G
NC_040252.1	84807180	G	A
NC_040252.1	84861558	G	A
NC_040252.1	84926699	T	A
NC_040252.1	84986608	T	C
NC_040252.1	85048244	A	G
NC_040252.1	85112070	A	C
NC_040252.1	85169074	C	T
NC_040252.1	85283870	T	C
NC_040252.1	85478381	G	A
NC_040252.1	85534349	A	C
NC_040252.1	85601306	T	A
NC_040252.1	85709104	A	G
NC_040252.1	85771359	G	A
NC_040252.1	85892038	G	A
NC_040252.1	85952149	A	G
NC_040252.1	86010829	G	A
NC_040252.1	86072189	A	G
NC_040252.1	86149398	A	G
NC_040252.1	86229058	C	T
NC_040252.1	86268676	A	G
NC_040252.1	86313204	A	G
NC_040252.1	86374658	T	C
NC_040252.1	86438362	G	A
NC_040252.1	86554342	T	C
NC_040252.1	86615786	G	T
NC_040252.1	86657491	A	G
NC_040252.1	86759239	G	A
NC_040252.1	86816780	G	A
NC_040252.1	86865860	C	T
NC_040252.1	86890099	A	G
NC_040252.1	87004398	A	G
NC_040252.1	87026873	G	T
NC_040252.1	87077371	G	C
NC_040252.1	87137198	G	T

NC_040252.1	87195406	G	A
NC_040252.1	87250832	C	T
NC_040252.1	87328575	A	G
NC_040252.1	87389379	C	A
NC_040252.1	87542442	T	C
NC_040252.1	87595703	C	T
NC_040252.1	87655965	C	T
NC_040252.1	87720345	C	T
NC_040252.1	87848498	T	C
NC_040252.1	87917096	G	C
NC_040252.1	87982470	C	T
NC_040252.1	88020104	C	T
NC_040252.1	88074293	G	C
NC_040252.1	88134942	T	C
NC_040252.1	88193927	T	C
NC_040252.1	88249501	G	A
NC_040252.1	88320948	A	G
NC_040252.1	88424161	A	G
NC_040252.1	88452760	C	T
NC_040252.1	88501758	A	G
NC_040252.1	88552889	C	T
NC_040252.1	88748447	C	T
NC_040252.1	88803762	G	A
NC_040252.1	88852791	A	G
NC_040252.1	88912335	T	C
NC_040252.1	88963650	T	C
NC_040252.1	89034429	G	A
NC_040252.1	89096688	G	A
NC_040252.1	89153687	G	T
NC_040252.1	89225144	T	C
NC_040252.1	89283641	C	G
NC_040252.1	89336300	T	C
NC_040252.1	89398908	A	G
NC_040252.1	89446014	A	G
NC_040252.1	89517352	T	C
NC_040252.1	89574187	C	T
NC_040252.1	89637422	C	T
NC_040252.1	89695453	C	G
NC_040252.1	89749601	C	T
NC_040252.1	89819991	A	G
NC_040252.1	89883205	A	G
NC_040252.1	89949039	G	T
NC_040252.1	90003041	T	C
NC_040252.1	90065960	A	T
NC_040252.1	90138004	C	T
NC_040252.1	90248045	A	G
NC_040252.1	90313803	A	G

NC_040252.1	90374070	C	T
NC_040252.1	90425317	T	C
NC_040252.1	90485982	C	A
NC_040252.1	90552545	A	C
NC_040252.1	90611377	G	C
NC_040252.1	90668541	A	C
NC_040252.1	90736355	C	G
NC_040252.1	90788613	T	C
NC_040252.1	90855756	C	T
NC_040252.1	90922982	A	G
NC_040252.1	90986988	C	T
NC_040252.1	91051685	C	T
NC_040252.1	91108356	C	G
NC_040252.1	91164028	T	C
NC_040252.1	91223250	G	C
NC_040252.1	91278324	C	G
NC_040252.1	91296633	G	A
NC_040252.1	91353890	C	T
NC_040252.1	91412177	T	G
NC_040252.1	91475718	T	C
NC_040252.1	91663040	G	T
NC_040252.1	91721172	C	T
NC_040252.1	91779545	A	C
NC_040252.1	91836335	T	C
NC_040252.1	91895063	A	G
NC_040252.1	91947691	A	G
NC_040252.1	92006195	T	G
NC_040252.1	92069863	A	G
NC_040252.1	92135051	G	A
NC_040252.1	92202071	G	A
NC_040252.1	92262713	G	C
NC_040252.1	92313801	G	T
NC_040252.1	92372763	C	T
NC_040252.1	92440748	C	T
NC_040252.1	92498001	T	C
NC_040252.1	92551067	T	C
NC_040252.1	92615440	G	A
NC_040252.1	92681707	T	C
NC_040252.1	92754709	T	G
NC_040252.1	92913971	G	C
NC_040252.1	93095876	A	C
NC_040252.1	93159263	T	C
NC_040252.1	93223929	G	A
NC_040252.1	93334954	T	G
NC_040252.1	93381533	C	T
NC_040252.1	93629782	A	C
NC_040252.1	93688008	T	C

NC_040252.1	93758865	G	A
NC_040252.1	93812105	A	C
NC_040252.1	93866770	G	T
NC_040252.1	93924212	C	T
NC_040252.1	93987287	A	G
NC_040252.1	94048882	T	C
NC_040252.1	94110446	A	G
NC_040252.1	94167152	A	G
NC_040252.1	94223604	G	A
NC_040252.1	94298471	A	G
NC_040252.1	94354233	A	G
NC_040252.1	94412098	T	C
NC_040252.1	94480619	A	C
NC_040252.1	94541334	A	G
NC_040252.1	94603269	T	G
NC_040252.1	94665919	C	T
NC_040252.1	94724513	A	G
NC_040252.1	94779614	T	C
NC_040252.1	94841540	G	T
NC_040252.1	94908482	G	T
NC_040252.1	95031466	A	T
NC_040252.1	95082377	C	T
NC_040252.1	95134916	0	T
NC_040252.1	95200182	T	C
NC_040252.1	95261797	T	C
NC_040252.1	95380325	A	G
NC_040252.1	95437099	T	C
NC_040252.1	95495688	A	G
NC_040252.1	95562907	G	A
NC_040252.1	95623020	A	G
NC_040252.1	95692858	G	A
NC_040252.1	95751576	C	T
NC_040252.1	95805338	T	C
NC_040252.1	95865556	A	G
NC_040252.1	95926510	A	T
NC_040252.1	95985124	A	G
NC_040252.1	96048381	T	C
NC_040252.1	96108340	T	C
NC_040252.1	96169912	C	T
NC_040252.1	96226083	T	C
NC_040252.1	96294567	A	G
NC_040252.1	96351494	T	C
NC_040252.1	96410627	A	T
NC_040252.1	96487800	A	G
NC_040252.1	96576764	T	C
NC_040252.1	96631424	A	G
NC_040252.1	96692912	G	C

NC_040252.1	96747551	C	A
NC_040252.1	96806342	T	C
NC_040252.1	96868637	C	T
NC_040252.1	96921195	G	A
NC_040252.1	96980271	C	A
NC_040252.1	97039537	A	G
NC_040252.1	97099631	A	T
NC_040252.1	97156682	C	T
NC_040252.1	97215994	A	G
NC_040252.1	97273338	T	C
NC_040252.1	97343074	C	T
NC_040252.1	97454602	C	T
NC_040252.1	97515162	C	T
NC_040252.1	97580213	G	A
NC_040252.1	97636858	G	T
NC_040252.1	97694562	C	A
NC_040252.1	97765465	A	T
NC_040252.1	97816855	G	A
NC_040252.1	97874282	C	T
NC_040252.1	97935313	T	C
NC_040252.1	97991354	T	C
NC_040252.1	98052792	G	A
NC_040252.1	98114521	A	G
NC_040252.1	98169442	A	G
NC_040252.1	98191690	T	C
NC_040252.1	98218556	A	G
NC_040252.1	98276637	A	G
NC_040252.1	98332865	A	G
NC_040252.1	98394372	C	A
NC_040252.1	98414248	T	C
NC_040252.1	98517714	A	G
NC_040252.1	98577237	A	G
NC_040252.1	98640925	G	A
NC_040252.1	98696943	C	T
NC_040252.1	98757478	A	G
NC_040252.1	98818769	G	T
NC_040252.1	98878019	T	C
NC_040252.1	98939048	A	C
NC_040252.1	99002088	G	T
NC_040252.1	99060336	G	A
NC_040252.1	99121095	C	T
NC_040252.1	99189652	T	C
NC_040252.1	99240209	A	G
NC_040252.1	99297509	A	G
NC_040252.1	99352889	T	C
NC_040252.1	99416439	T	C
NC_040252.1	99476547	C	G

NC_040252.1	99530776	A	G
NC_040252.1	99599998	A	G
NC_040252.1	99623341	C	T
NC_040252.1	99731395	A	G
NC_040252.1	99849735	A	G
NC_040252.1	99913498	A	C
NC_040252.1	99981981	C	G
NC_040252.1	100037415	C	T
NC_040252.1	100097452	A	G
NC_040252.1	100160999	C	T
NC_040252.1	100214259	A	G
NC_040252.1	100281863	T	C
NC_040252.1	100345313	A	G
NC_040252.1	100396437	C	G
NC_040252.1	100518508	G	A
NC_040252.1	100575672	T	C
NC_040252.1	100640936	T	C
NC_040252.1	100763315	T	A
NC_040252.1	100828270	A	C
NC_040252.1	100880541	G	A
NC_040252.1	101001791	A	C
NC_040252.1	101056136	G	A
NC_040252.1	101114031	G	A
NC_040252.1	101180667	G	A
NC_040252.1	101237608	T	C
NC_040252.1	101295080	G	C
NC_040252.1	101354478	T	G
NC_040252.1	101415680	T	C
NC_040252.1	101473921	C	A
NC_040252.1	101532491	T	C
NC_040252.1	101584977	C	T
NC_040252.1	101649283	T	C
NC_040252.1	101716580	C	T
NC_040252.1	101775988	G	A
NC_040252.1	101895970	T	G
NC_040252.1	101963956	A	G
NC_040252.1	102022398	A	G
NC_040252.1	102087531	G	C
NC_040252.1	102149212	A	G
NC_040252.1	102214543	A	G
NC_040252.1	102272553	A	G
NC_040252.1	102329580	C	T
NC_040252.1	102385105	A	G
NC_040252.1	102444893	G	T
NC_040252.1	102502577	A	T
NC_040252.1	102569044	T	C
NC_040252.1	102626479	T	G

NC_040252.1	102688865	A	G
NC_040252.1	102746930	T	G
NC_040252.1	102759175	C	T
NC_040252.1	102939120	G	C
NC_040252.1	103765073	C	A
NC_040252.1	103827042	C	G
NC_040252.1	103881328	T	A
NC_040252.1	103941308	C	A
NC_040252.1	104001689	T	G
NC_040252.1	104070815	T	A
NC_040252.1	104241540	A	G
NC_040252.1	104299301	A	G
NC_040252.1	104360094	C	G
NC_040252.1	104422774	A	C
NC_040252.1	104475798	A	G
NC_040252.1	104544100	A	G
NC_040252.1	104604931	A	T
NC_040252.1	104663635	A	C
NC_040252.1	104730982	A	G
NC_040252.1	104779992	A	G
NC_040252.1	104906339	A	C
NC_040252.1	104968390	C	T
NC_040252.1	105026110	A	G
NC_040252.1	105227037	G	A
NC_040252.1	105348385	A	G
NC_040252.1	105481909	C	G
NC_040252.1	105539556	T	C
NC_040252.1	105599560	G	A
NC_040252.1	105659312	C	T
NC_040252.1	105721047	A	G
NC_040252.1	105780847	T	C
NC_040252.1	105838777	G	A
NC_040252.1	105896528	T	A
NC_040252.1	105951385	G	A
NC_040252.1	106012118	C	G
NC_040252.1	106070821	T	C
NC_040252.1	106129146	C	T
NC_040252.1	106192209	C	T
NC_040252.1	106254669	G	C
NC_040252.1	106309261	A	G
NC_040252.1	106367449	C	G
NC_040252.1	106423699	A	G
NC_040252.1	106488711	A	G
NC_040252.1	106547432	G	T
NC_040252.1	106607115	C	A
NC_040252.1	106665986	G	A
NC_040252.1	106725562	C	T

NC_040252.1	106786215	T	C
NC_040252.1	106849111	G	A
NC_040252.1	106907884	G	A
NC_040252.1	106967384	G	A
NC_040252.1	107030929	T	C
NC_040252.1	107082362	G	C
NC_040252.1	107145486	T	G
NC_040252.1	107207408	C	T
NC_040252.1	107262493	T	A
NC_040252.1	107313989	G	A
NC_040252.1	107386211	T	G
NC_040252.1	107443734	A	T
NC_040252.1	107502712	G	A
NC_040252.1	107574531	C	A
NC_040252.1	107631752	A	G
NC_040252.1	107816752	T	C
NC_040252.1	107876218	G	C
NC_040252.1	107939733	A	C
NC_040252.1	108000872	A	C
NC_040252.1	108057999	A	G
NC_040252.1	108114100	C	A
NC_040252.1	108292155	T	C
NC_040252.1	108369949	A	G
NC_040252.1	108602909	A	G
NC_040252.1	108663636	C	T
NC_040252.1	108789592	A	G
NC_040252.1	108846929	T	C
NC_040252.1	108910126	C	G
NC_040252.1	109084435	A	G
NC_040252.1	109242078	G	A
NC_040252.1	109676257	A	G
NC_040252.1	109744652	C	T
NC_040252.1	109803375	G	A
NC_040252.1	109864911	A	G
NC_040252.1	109926876	T	C
NC_040252.1	109989869	A	T
NC_040252.1	110165393	A	G
NC_040252.1	110226040	G	A
NC_040252.1	110282838	A	G
NC_040252.1	110340764	A	G
NC_040252.1	110401087	T	C
NC_040252.1	110461309	T	A
NC_040252.1	110514087	T	G
NC_040252.1	110579334	T	A
NC_040252.1	110639324	A	G
NC_040252.1	110698160	A	G
NC_040252.1	110760416	C	T

NC_040252.1	110811487	T	C
NC_040252.1	110877262	C	T
NC_040252.1	110934113	A	T
NC_040252.1	111003129	G	A
NC_040252.1	111065562	C	T
NC_040252.1	111126245	C	T
NC_040252.1	111188213	A	G
NC_040252.1	111247906	C	T
NC_040252.1	111310143	T	C
NC_040252.1	111366182	A	G
NC_040252.1	111425765	C	G
NC_040252.1	111487962	T	C
NC_040252.1	111546370	T	C
NC_040252.1	111604583	A	G
NC_040252.1	111667369	A	G
NC_040252.1	111726653	G	A
NC_040252.1	111782330	T	C
NC_040252.1	111842262	C	G
NC_040252.1	111897501	C	A
NC_040252.1	111958931	T	C
NC_040252.1	112021003	C	T
NC_040252.1	112039703	T	A
NC_040252.1	112135847	C	G
NC_040252.1	112197786	T	C
NC_040252.1	112259277	C	A
NC_040252.1	112322587	G	A
NC_040252.1	112380977	G	C
NC_040252.1	112440596	C	T
NC_040252.1	112497473	T	C
NC_040252.1	112555122	A	G
NC_040252.1	112616121	0	G
NC_040252.1	112678789	T	C
NC_040252.1	112744411	A	G
NC_040252.1	112795695	T	C
NC_040252.1	112849426	G	A
NC_040252.1	112909974	T	C
NC_040252.1	112966419	T	C
NC_040252.1	113025494	A	G
NC_040252.1	113089516	T	C
NC_040252.1	113142380	G	A
NC_040252.1	113201258	A	G
NC_040252.1	113259173	C	T
NC_040252.1	113320562	G	A
NC_040252.1	113620785	G	A
NC_040252.1	113857844	C	G
NC_040252.1	113927265	G	A
NC_040252.1	113982141	G	A

NC_040252.1	114034980	C	T
NC_040252.1	114090357	C	T
NC_040252.1	114160459	C	T
NC_040252.1	114211418	T	A
NC_040252.1	114272598	T	C
NC_040252.1	114329797	G	C
NC_040252.1	114396284	T	C
NC_040252.1	114504853	G	C
NC_040252.1	114567648	G	A
NC_040252.1	114631817	A	T
NC_040252.1	114690949	T	C
NC_040252.1	114751074	A	G
NC_040252.1	114808656	A	G
NC_040252.1	114865957	T	C
NC_040252.1	114909295	T	C
NC_040252.1	115231193	T	C
NC_040252.1	115296066	T	G
NC_040252.1	115353400	A	G
NC_040252.1	115410318	A	T
NC_040252.1	115528008	G	A
NC_040252.1	115546896	T	G
NC_040252.1	115719778	T	C
NC_040252.1	115831170	C	A
NC_040252.1	116055241	T	C
NC_040252.1	116097979	C	T
NC_040252.1	116164848	A	G
NC_040252.1	116201526	C	T
NC_040252.1	116350549	A	G
NC_040252.1	116435276	A	T
NC_040252.1	116599514	C	T
NC_040252.1	116656804	G	A
NC_040252.1	116715031	A	G
NC_040252.1	116776197	C	T
NC_040252.1	116899137	C	T
NC_040252.1	116962516	T	C
NC_040252.1	117036260	C	A
NC_040252.1	117093996	T	C
NC_040252.1	117196892	C	T
NC_040252.1	117556473	G	C
NC_040252.1	117613051	G	C
NC_040252.1	117668385	T	C
NC_040252.1	117740084	T	C
NC_040252.1	117808859	T	C
NC_040252.1	117867807	A	G
NC_040252.1	117931664	A	G
NC_040252.1	117993199	C	T
NC_040252.1	118043582	T	C

NC_040252.1	118099892	C	A
NC_040252.1	118156221	C	T
NC_040252.1	118217753	G	A
NC_040252.1	118276449	T	C
NC_040252.1	118391449	T	C
NC_040252.1	118514110	A	T
NC_040252.1	118568032	C	T
NC_040252.1	118865063	T	C
NC_040252.1	118925203	C	T
NC_040252.1	119521849	T	C
NC_040252.1	119581461	A	G
NC_040252.1	119636699	G	A
NC_040252.1	119691326	C	T
NC_040252.1	119752200	C	A
NC_040252.1	119807990	A	G
NC_040252.1	119881766	G	C
NC_040252.1	120052859	C	T
NC_040252.1	120170668	A	G
NC_040252.1	120231624	T	C
NC_040252.1	120294197	G	A
NC_040252.1	120352090	T	G
NC_040252.1	120466722	G	A
NC_040252.1	120526151	A	G
NC_040252.1	120650154	G	A
NC_040252.1	120705465	G	A
NC_040252.1	120763368	A	G
NC_040252.1	120825095	G	A
NC_040252.1	120881249	C	T
NC_040252.1	120939640	G	A
NC_040252.1	121008883	A	G
NC_040252.1	121065092	A	C
NC_040252.1	121122078	A	G
NC_040252.1	121190563	C	T
NC_040252.1	121372440	A	G
NC_040252.1	121438589	G	C
NC_040252.1	121494902	T	C
NC_040252.1	121558724	C	G
NC_040252.1	121739682	A	C
NC_040252.1	121797325	T	C
NC_040252.1	121853730	T	C
NC_040252.1	121908737	C	T
NC_040252.1	121967463	T	G
NC_040252.1	122031946	T	C
NC_040252.1	122261327	A	G
NC_040252.1	122381448	C	G
NC_040252.1	122438271	T	C
NC_040252.1	122507786	G	A

NC_040252.1	122567826	C	T
NC_040252.1	122627487	T	C
NC_040252.1	122688412	T	C
NC_040252.1	122748702	C	T
NC_040252.1	122819807	G	T
NC_040252.1	122980204	A	G
NC_040252.1	123048570	G	A
NC_040252.1	123104018	A	G
NC_040252.1	123165962	A	T
NC_040252.1	123326995	T	A
NC_040252.1	123450781	G	A
NC_040252.1	123512573	A	T
NC_040252.1	123570801	A	G
NC_040252.1	123631748	A	T
NC_040252.1	123680184	T	A
NC_040252.1	123736540	T	C
NC_040252.1	123798687	C	T
NC_040252.1	123864391	A	G
NC_040252.1	123919779	A	G
NC_040252.1	123978511	A	G
NC_040252.1	124039106	A	G
NC_040252.1	124095739	T	C
NC_040252.1	124152816	A	G
NC_040252.1	124208750	C	T
NC_040252.1	124274737	C	T
NC_040252.1	124451990	A	C
NC_040252.1	124507089	T	C
NC_040252.1	124572112	T	C
NC_040252.1	124624211	A	C
NC_040252.1	124682289	A	C
NC_040252.1	124740048	C	G
NC_040252.1	124801871	C	G
NC_040252.1	124859666	A	G
NC_040252.1	124915891	G	A
NC_040252.1	124977349	A	G
NC_040252.1	125037213	A	G
NC_040252.1	125097598	C	T
NC_040252.1	125150924	A	G
NC_040252.1	125203122	G	T
NC_040252.1	125250028	A	C
NC_040252.1	125282318	C	T
NC_040252.1	125380783	G	A
NC_040252.1	125441623	G	A
NC_040252.1	125557328	T	C
NC_040252.1	125614481	A	G
NC_040252.1	125670493	A	G
NC_040252.1	125724618	C	A

NC_040252.1	125868003	T	C
NC_040252.1	125925534	T	G
NC_040252.1	125981742	T	C
NC_040252.1	126311671	A	G
NC_040252.1	126344095	G	C
NC_040252.1	126436567	A	T
NC_040252.1	126488827	A	G
NC_040252.1	126609556	T	C
NC_040252.1	126668373	G	C
NC_040252.1	126724815	A	G
NC_040252.1	126762290	G	A
NC_040252.1	126800785	C	T
NC_040252.1	126850471	A	G
NC_040252.1	126905155	T	G
NC_040252.1	126963995	T	C
NC_040252.1	127025416	C	G
NC_040252.1	127138320	A	G
NC_040252.1	127196351	G	A
NC_040252.1	127255234	G	A
NC_040252.1	127275698	G	A
NC_040252.1	127331117	C	T
NC_040252.1	127389924	G	T
NC_040252.1	127460951	T	C
NC_040252.1	127517400	G	A
NC_040252.1	127626313	T	G
NC_040252.1	127684106	A	G
NC_040252.1	127746531	G	A
NC_040252.1	127798870	T	C
NC_040252.1	127967598	G	A
NC_040252.1	128025282	T	C
NC_040252.1	128139512	A	G
NC_040252.1	128200352	C	T
NC_040252.1	128259367	G	T
NC_040252.1	128317615	C	T
NC_040252.1	128435632	A	G
NC_040252.1	128557029	A	G
NC_040252.1	128755318	T	C
NC_040252.1	128810636	A	C
NC_040252.1	128870879	G	A
NC_040252.1	128926181	A	G
NC_040252.1	128986248	G	A
NC_040252.1	129042259	T	C
NC_040252.1	129115700	C	T
NC_040252.1	129352396	C	T
NC_040252.1	129405560	G	C
NC_040252.1	129464154	C	T
NC_040252.1	129515755	C	T

NC_040252.1	129570430	C	G
NC_040252.1	129631887	G	A
NC_040252.1	129694510	G	A
NC_040252.1	129746893	A	G
NC_040252.1	129805319	A	G
NC_040252.1	129864107	A	G
NC_040252.1	129901617	T	C
NC_040252.1	129959857	A	C
NC_040252.1	130017481	C	T
NC_040252.1	130131822	T	G
NC_040252.1	130188431	C	G
NC_040252.1	130246231	G	T
NC_040252.1	130302161	T	C
NC_040252.1	130359215	G	C
NC_040252.1	130421707	C	T
NC_040252.1	130485104	A	G
NC_040252.1	130540341	T	C
NC_040252.1	130602823	G	C
NC_040252.1	130659077	A	G
NC_040252.1	130721754	A	G
NC_040252.1	130775109	T	C
NC_040252.1	130839921	T	G
NC_040252.1	130899024	C	T
NC_040252.1	130955205	G	A
NC_040252.1	131014660	T	C
NC_040252.1	131082364	C	G
NC_040252.1	131134496	T	C
NC_040252.1	131192537	A	T
NC_040252.1	131359994	A	G
NC_040252.1	131422779	T	G
NC_040252.1	131491820	T	C
NC_040252.1	131525332	A	G
NC_040252.1	131605444	C	G
NC_040252.1	131666274	G	A
NC_040252.1	131725253	G	A
NC_040252.1	131784054	T	C
NC_040252.1	131841657	T	C
NC_040252.1	131904546	A	G
NC_040252.1	131959285	T	C
NC_040252.1	132017834	C	A
NC_040252.1	132075500	C	A
NC_040252.1	132136027	T	G
NC_040252.1	132194816	A	G
NC_040252.1	132247803	C	T
NC_040252.1	132303961	C	T
NC_040252.1	132372904	T	C
NC_040252.1	132432489	C	T

NC_040252.1	132493040	A	G
NC_040252.1	132549985	G	C
NC_040252.1	132621133	G	A
NC_040252.1	132673328	T	C
NC_040252.1	132729179	T	C
NC_040252.1	132782437	A	G
NC_040252.1	132839666	T	C
NC_040252.1	132857944	G	A
NC_040252.1	133042199	C	G
NC_040252.1	133103845	G	A
NC_040252.1	133151274	A	C
NC_040252.1	133213089	G	C
NC_040252.1	133271626	G	A
NC_040252.1	133335742	A	G
NC_040252.1	133389836	T	G
NC_040252.1	133458676	T	G
NC_040252.1	133517944	G	A
NC_040252.1	133575369	C	T
NC_040252.1	133633858	G	A
NC_040252.1	133738920	T	G
NC_040252.1	133808036	A	G
NC_040252.1	133990820	C	T
NC_040252.1	134057061	T	C
NC_040252.1	134106542	G	A
NC_040252.1	134168314	C	T
NC_040252.1	134222376	G	A
NC_040252.1	134281747	G	A
NC_040252.1	134341185	C	G
NC_040252.1	134399179	A	G
NC_040252.1	134452295	T	C
NC_040252.1	134508091	G	A
NC_040252.1	134564206	A	G
NC_040252.1	134624113	T	C
NC_040252.1	134676772	A	G
NC_040252.1	134738609	A	C
NC_040252.1	134793176	G	A
NC_040252.1	134850810	A	G
NC_040252.1	134915538	T	C
NC_040252.1	134971986	T	C
NC_040252.1	135022202	A	G
NC_040252.1	135084289	C	G
NC_040252.1	135143022	G	A
NC_040252.1	135313423	A	G
NC_040252.1	135378566	A	C
NC_040252.1	135436669	C	T
NC_040252.1	135491953	C	T
NC_040252.1	135558378	T	G

NC_040252.1	135615178	C	A
NC_040252.1	135684521	T	G
NC_040252.1	135795969	C	A
NC_040252.1	135847764	A	G
NC_040252.1	135914904	T	C
NC_040252.1	135976649	C	T
NC_040252.1	136029167	A	G
NC_040252.1	136083984	C	T
NC_040252.1	136155926	G	A
NC_040252.1	136204993	T	C
NC_040252.1	136254560	T	C
NC_040252.1	136307292	G	A
NC_040252.1	136376564	C	T
NC_040252.1	136495110	T	C
NC_040252.1	136560835	C	A
NC_040252.1	136630661	A	T
NC_040252.1	136853307	C	T
NC_040252.1	136905454	G	A
NC_040252.1	136966636	G	A
NC_040252.1	137017337	C	A
NC_040252.1	137082548	T	A
NC_040252.1	137286625	T	C
NC_040252.1	137336096	C	A
NC_040252.1	137393918	T	C
NC_040252.1	137450327	A	G
NC_040252.1	137508240	C	G
NC_040252.1	137573673	A	T
NC_040252.1	137627715	G	A
NC_040252.1	137674612	C	T
NC_040252.1	137738784	T	C
NC_040252.1	137813291	G	A
NC_040252.1	137870024	C	T
NC_040252.1	137926413	G	A
NC_040252.1	137982626	T	C
NC_040252.1	138039205	A	C
NC_040252.1	138093106	C	T
NC_040252.1	138153984	A	G
NC_040252.1	138227594	T	C
NC_040252.1	138286317	C	T
NC_040252.1	138339985	A	G
NC_040252.1	138453863	T	C
NC_040252.1	138525935	A	G
NC_040252.1	138688904	T	C
NC_040252.1	138713876	T	C
NC_040252.1	138773517	A	C
NC_040252.1	138830063	A	G
NC_040252.1	138887227	T	A

NC_040252.1	138943244	C	T
NC_040252.1	139002864	T	C
NC_040252.1	139069665	G	T
NC_040252.1	139127902	A	T
NC_040252.1	139176184	C	T
NC_040252.1	139257685	A	C
NC_040252.1	139322769	C	T
NC_040252.1	139372779	C	G
NC_040252.1	139438896	G	A
NC_040252.1	139500007	G	T
NC_040252.1	139557718	A	G
NC_040252.1	139616663	C	T
NC_040252.1	139688878	T	A
NC_040252.1	139743250	T	G
NC_040252.1	139804107	G	A
NC_040252.1	139860716	A	G
NC_040252.1	139925150	T	C
NC_040252.1	139972246	T	C
NC_040252.1	140033084	0	T
NC_040252.1	140154513	T	C
NC_040252.1	140313625	T	G
NC_040252.1	140358548	T	C
NC_040252.1	140401074	A	G
NC_040252.1	140684593	G	C
NC_040252.1	140748552	A	C
NC_040252.1	140816710	T	C
NC_040252.1	140871719	G	C
NC_040252.1	141096915	T	C
NC_040252.1	141158281	G	A
NC_040252.1	141212171	G	A
NC_040252.1	141269802	G	T
NC_040252.1	141333663	A	G
NC_040252.1	141401135	C	T
NC_040252.1	141512902	T	G
NC_040252.1	141572439	A	G
NC_040252.1	141681792	C	T
NC_040252.1	142041228	G	A
NC_040252.1	142118719	A	G
NC_040252.1	142321634	T	C
NC_040252.1	142356666	C	T
NC_040252.1	142482235	A	C
NC_040252.1	142657129	T	A
NC_040252.1	142710688	C	T
NC_040252.1	142772822	A	T
NC_040252.1	142993686	T	C
NC_040252.1	143048968	G	A
NC_040252.1	143562110	G	A

NC_040252.1	143620307	G	A
NC_040252.1	143680648	C	T
NC_040252.1	143903832	T	C
NC_040252.1	144101000	T	C
NC_040252.1	144154903	A	G
NC_040252.1	144441048	T	C
NC_040252.1	144487631	G	A
NC_040252.1	144530599	G	A
NC_040252.1	144584259	T	C
NC_040252.1	144629857	G	A
NC_040252.1	144692500	A	C
NC_040252.1	144761313	C	T
NC_040252.1	144860952	A	C
NC_040252.1	144939192	T	C
NC_040252.1	145047861	C	T
NC_040252.1	145163085	G	A
NC_040252.1	145212969	G	A
NC_040252.1	145280384	T	G
NC_040252.1	145413305	G	A
NC_040252.1	145603788	A	G
NC_040252.1	145783113	C	T
NC_040252.1	145838322	G	A
NC_040252.1	145900533	C	T
NC_040252.1	145970321	A	G
NC_040252.1	146100138	T	C
NC_040252.1	146159938	C	T
NC_040252.1	146221450	A	T
NC_040252.1	146281414	C	T
NC_040252.1	146351456	A	T
NC_040252.1	146407226	T	A
NC_040252.1	146473292	A	G
NC_040252.1	146508737	G	A
NC_040252.1	146572853	A	G
NC_040252.1	146651477	A	G
NC_040252.1	146700672	G	C
NC_040252.1	146765458	T	C
NC_040252.1	146830353	G	T
NC_040252.1	146890195	T	C
NC_040252.1	147121192	A	T
NC_040252.1	147294764	G	C
NC_040252.1	147423988	T	C
NC_040252.1	147529639	C	A
NC_040252.1	147726715	A	T
NC_040252.1	147762294	G	A
NC_040252.1	147810172	A	G
NC_040252.1	147967288	C	G
NC_040252.1	148023490	A	C

NC_040252.1	148083061	A		G
NC_040252.1	148156665	A		G
NC_040252.1	148212054	T		C
NC_040252.1	148293625	C		T
NC_040252.1	148356737	C		T
NC_040252.1	148437849	A		T
NC_040252.1	148576090	C		T
NC_040252.1	148619536	T		A
NC_040252.1	148671850	T		G
NC_040252.1	148957351	C		G
NC_040252.1	148974631	T		C
NC_040252.1	149055444	G		T
NC_040252.1	149109517	A		G
NC_040252.1	149200598	T		A
NC_040252.1	149263887	C		T
NC_040252.1	149325396	G		A
NC_040252.1	149351394	T		C
NC_040252.1	149437043	C		T
NC_040252.1	149636075	T		C
NC_040252.1	149690673	G		A
NC_040252.1	149753977	A		G
NC_040252.1	149814958	G		A
NC_040252.1	149936709	C		G
NC_040252.1	150003526	C		T
NC_040252.1	150248541	C		T
NC_040252.1	150323283	C		T
NC_040252.1	150380312	G		A
NC_040252.1	150443952	G		A
NC_040252.1	150508547	G		T
NC_040252.1	150564035	C		T
NC_040252.1	150619845	T		C
NC_040252.1	150678995	A		G
NC_040252.1	150734864		0	G
NC_040252.1	150795547	C		G
NC_040252.1	150874390	C		T
NC_040252.1	151043493	C		T
NC_040252.1	151110384	T		G
NC_040252.1	151167671	G		C
NC_040252.1	151227892	T		C
NC_040252.1	151291295	C		A
NC_040252.1	151415885	C		G
NC_040252.1	151475209	A		G
NC_040252.1	151540019	T		A
NC_040252.1	151595176	T		G
NC_040252.1	151659982	G		A
NC_040252.1	151733236	A		C
NC_040252.1	151847267	A		G

NC_040252.1	151907012	G	T
NC_040252.1	151964100	C	T
NC_040252.1	151982524	A	G
NC_040252.1	152037693	C	T
NC_040252.1	152099609	T	C
NC_040252.1	152154615	A	G
NC_040252.1	152270625	C	A
NC_040252.1	152331815	T	C
NC_040252.1	152386695	G	C
NC_040252.1	152442004	A	G
NC_040252.1	152500523	T	G
NC_040252.1	152565767	G	C
NC_040252.1	152626628	T	C
NC_040252.1	152693688	C	T
NC_040252.1	152753139	C	T
NC_040252.1	152809277	C	G
NC_040252.1	152864122	G	A
NC_040252.1	152926222	C	T
NC_040252.1	152987991	A	G
NC_040252.1	153044440	C	T
NC_040252.1	153100544	G	A
NC_040252.1	153165893	A	G
NC_040252.1	153229087	C	A
NC_040252.1	153285330	C	A
NC_040252.1	153346213	T	G
NC_040252.1	153403097	C	G
NC_040252.1	153461896	G	C
NC_040252.1	153520633	A	G
NC_040252.1	153576292	C	G
NC_040252.1	153643639	A	G
NC_040252.1	153758385	A	G
NC_040252.1	153826635	A	G
NC_040252.1	153882805	G	A
NC_040252.1	153943532	C	A
NC_040252.1	154010274	A	G
NC_040252.1	154408938	A	G
NC_040252.1	154488904	T	C
NC_040252.1	154561469	C	T
NC_040252.1	154617705	A	G
NC_040252.1	154660921	A	C
NC_040252.1	154739140	A	G
NC_040252.1	154824156	C	T
NC_040252.1	154885724	C	T
NC_040252.1	154940572	T	A
NC_040252.1	155000884	C	T
NC_040252.1	155064278	A	C
NC_040252.1	155127809	T	C

NC_040252.1	155180272	T	G
NC_040252.1	155256387	T	C
NC_040252.1	155308689	G	A
NC_040252.1	155374134	T	C
NC_040252.1	155437839	G	A
NC_040252.1	155492292	A	G
NC_040252.1	155560007	A	G
NC_040252.1	155635212	T	G
NC_040252.1	155691210	T	C
NC_040252.1	155746651	T	C
NC_040252.1	155804230	T	C
NC_040252.1	155855869	T	A
NC_040252.1	155890533	C	T
NC_040252.1	155995980	G	A
NC_040252.1	156064788	A	G
NC_040252.1	156111959	T	C
NC_040252.1	156182526	G	A
NC_040252.1	156207046	T	G
NC_040252.1	156269623	A	T
NC_040252.1	156333563	T	C
NC_040252.1	156396129	G	T
NC_040252.1	156461228	C	T
NC_040252.1	156523914	A	G
NC_040252.1	156578038	G	A
NC_040252.1	156646798	A	G
NC_040252.1	156752024	T	C
NC_040252.1	156828348	G	T
NC_040252.1	156887294	C	T
NC_040252.1	156957159	T	C
NC_040252.1	157009599	T	A
NC_040252.1	157060642	G	A
NC_040252.1	157116382	G	A
NC_040252.1	157176889	T	C
NC_040252.1	157483320	A	C
NC_040252.1	157540780	C	G
NC_040252.1	157678620	T	A
NC_040252.1	157733685	T	A
NC_040252.1	157804410	G	A
NC_040252.1	157862814	G	A
NC_040252.1	157926916	A	G
NC_040252.1	157975015	A	G
NC_040252.1	158032510	C	A
NC_040252.1	158372981	A	T
NC_040252.1	158431844	A	G
NC_040252.1	158485659	G	A
NC_040252.1	158540158	C	T
NC_040252.1	158594848	T	G

NC_040252.1	158657745	T	C
NC_040252.1	158713684	G	A
NC_040252.1	158774949	T	C
NC_040252.1	158832345	C	T
NC_040252.1	158893784	T	C
NC_040252.1	158948669	A	G
NC_040252.1	159007928	G	T
NC_040252.1	159067456	G	A
NC_040252.1	159121055	C	G
NC_040252.1	159247498	G	T
NC_040252.1	159580747	T	C
NC_040252.1	159597978	C	T
NC_040252.1	159713165	A	G
NC_040252.1	159772345	A	G
NC_040252.1	159831986	A	C
NC_040252.1	159895043	G	A
NC_040252.1	159923742	G	C
NC_040252.1	159993573	A	C
NC_040252.1	160039451	T	G
NC_040252.1	160349322	A	G
NC_040252.1	160385236	A	G
NC_040252.1	160462385	C	T
NC_040252.1	160500773	A	G
NC_040252.1	160584364	T	C
NC_040252.1	160663660	C	T
NC_040252.1	160723198	A	G
NC_040252.1	160925122	C	G
NC_040252.1	160992783	T	C
NC_040252.1	161048000	G	A
NC_040252.1	161101483	G	A
NC_040252.1	161159301	A	G
NC_040252.1	161214578	C	G
NC_040252.1	161277023	T	C
NC_040252.1	161337300	T	C
NC_040252.1	161401005	T	G
NC_040252.1	161460312	A	G
NC_040252.1	161511981	A	T
NC_040252.1	161569776	T	C
NC_040252.1	161636658	C	T
NC_040252.1	161695379	G	A
NC_040252.1	161761276	G	T
NC_040252.1	161810967	T	C
NC_040252.1	161868157	G	A
NC_040252.1	161925182	G	T
NC_040252.1	161977778	G	A
NC_040252.1	162033786	T	C
NC_040252.1	162087345	C	A

NC_040252.1	162140788	T	G
NC_040252.1	162197070	G	T
NC_040252.1	162316071	A	G
NC_040252.1	162381222	C	A
NC_040252.1	162435442	C	T
NC_040252.1	162534433	G	C
NC_040252.1	162586868	G	A
NC_040252.1	162656891	T	A
NC_040252.1	162705019	A	C
NC_040252.1	162770293	T	C
NC_040252.1	162835298	G	A
NC_040252.1	162889874	A	T
NC_040252.1	162942365	A	G
NC_040252.1	163066083	A	C
NC_040252.1	163120704	G	A
NC_040252.1	163172019	T	C
NC_040252.1	163233073	A	T
NC_040252.1	163302755	C	A
NC_040252.1	163356305	C	T
NC_040252.1	163543965	T	A
NC_040252.1	163660732	T	C
NC_040252.1	163717908	G	A
NC_040252.1	163769901	G	A
NC_040252.1	163822150	T	A
NC_040252.1	163876979	A	C
NC_040252.1	163929213	C	A
NC_040252.1	163984385	T	C
NC_040252.1	164038558	C	G
NC_040252.1	164096996	A	T
NC_040252.1	164256592	T	C
NC_040252.1	164494698	C	A
NC_040252.1	164553218	T	G
NC_040252.1	164613423	G	A
NC_040252.1	164894739	A	T
NC_040252.1	164942442	G	C
NC_040252.1	164969094	A	T
NC_040252.1	165246243	T	C
NC_040252.1	165436160	A	G
NC_040252.1	165483615	G	T
NC_040252.1	165541172	G	A
NC_040252.1	165590283	C	A
NC_040252.1	165646332	T	A
NC_040252.1	165718767	A	G
NC_040252.1	165788609	C	T
NC_040252.1	165832063	T	A
NC_040252.1	165888290	G	A
NC_040252.1	165998801	G	A

NC_040252.1	166052911	C	T
NC_040252.1	166112412	A	G
NC_040252.1	166168219	G	T
NC_040252.1	166284009	C	T
NC_040252.1	166307240	T	G
NC_040252.1	166425758	A	G
NC_040252.1	166479880	T	C
NC_040252.1	166549453	G	C
NC_040252.1	166609304	T	C
NC_040252.1	166665331	G	A
NC_040252.1	166719518	C	A
NC_040252.1	166769436	G	A
NC_040252.1	166850038	C	T
NC_040252.1	166915773	C	T
NC_040252.1	167352808	C	T
NC_040252.1	167465713	G	A
NC_040252.1	167535981	G	T
NC_040252.1	167598662	G	A
NC_040252.1	167666405	C	T
NC_040252.1	167731253	T	C
NC_040252.1	168018450	C	T
NC_040252.1	168084636	T	C
NC_040252.1	168151704	G	A
NC_040252.1	168208084	A	T
NC_040252.1	168263617	C	T
NC_040252.1	168326875	G	A
NC_040252.1	168390822	G	C
NC_040252.1	168509740	A	G
NC_040252.1	168558996	T	C
NC_040252.1	168616120	A	G
NC_040252.1	168676172	C	A
NC_040252.1	168738587	G	T
NC_040252.1	168797606	C	T
NC_040252.1	168866107	G	A
NC_040252.1	169037781	T	C
NC_040252.1	169150689	G	A
NC_040252.1	169209334	T	A
NC_040252.1	169502203	G	A
NC_040252.1	169561045	G	A
NC_040252.1	169617359	A	G
NC_040252.1	169668243	A	G
NC_040252.1	169736590	A	G
NC_040252.1	169793227	T	C
NC_040252.1	169847886	C	T
NC_040252.1	169902752	C	T
NC_040252.1	169963053	A	G
NC_040252.1	170017510	C	T

NC_040252.1	170084180	G	A
NC_040252.1	170137379	T	A
NC_040252.1	170200001	A	G
NC_040252.1	170260511	T	C
NC_040252.1	170318981	T	C
NC_040252.1	170382134	G	A
NC_040252.1	170442176	T	C
NC_040252.1	170510039	C	T
NC_040252.1	170731967	T	C
NC_040252.1	170792342	G	A
NC_040252.1	170903558	A	G
NC_040252.1	170960821	T	C
NC_040252.1	171019045	A	G
NC_040252.1	171075712	A	G
NC_040252.1	171142696	A	G
NC_040252.1	171321843	G	A
NC_040252.1	171382704	C	T
NC_040252.1	171441490	G	A
NC_040252.1	171505571	T	C
NC_040252.1	171557742	C	T
NC_040252.1	171677631	T	C
NC_040252.1	171735271	T	C
NC_040252.1	171799985	G	C
NC_040252.1	171855458	C	T
NC_040252.1	171908958	A	G
NC_040252.1	171976252	G	A
NC_040252.1	172031621	T	G
NC_040252.1	172084869	A	G
NC_040252.1	172160669	T	C
NC_040252.1	172223276	C	T
NC_040252.1	172279395	T	G
NC_040252.1	172334205	A	G
NC_040252.1	172387516	C	T
NC_040252.1	172466945	T	C
NC_040252.1	172574326	T	A
NC_040252.1	172635690	A	C
NC_040252.1	172688870	G	A
NC_040252.1	172871723	T	C
NC_040252.1	172940387	G	A
NC_040252.1	173000621	A	G
NC_040252.1	173067208	C	T
NC_040252.1	173251427	T	C
NC_040252.1	173304729	C	T
NC_040252.1	173354649	C	T
NC_040252.1	173426649	A	T
NC_040252.1	173564990	G	A
NC_040252.1	173632322	A	G

NC_040252.1	173709942	A	G
NC_040252.1	173827329	G	A
NC_040252.1	173973985	C	T
NC_040252.1	174045281	T	G
NC_040252.1	174163085	C	T
NC_040252.1	174243726	T	A
NC_040252.1	174316377	G	T
NC_040252.1	174390126	T	C
NC_040252.1	174845870	A	G
NC_040252.1	174900071	G	A
NC_040252.1	174953343	C	G
NC_040252.1	175013700	A	G
NC_040252.1	175071522	T	C
NC_040252.1	175135980	T	C
NC_040252.1	175189287	T	A
NC_040252.1	175243065	G	A
NC_040252.1	175300776	G	C
NC_040252.1	175356867	G	A
NC_040252.1	175412985	C	T
NC_040252.1	175469111	A	G
NC_040252.1	175534585	A	C
NC_040252.1	175593489	A	G
NC_040252.1	175654591	A	G
NC_040252.1	175710701	A	G
NC_040252.1	175773401	A	G
NC_040252.1	175887510	C	G
NC_040252.1	176012931	C	T
NC_040252.1	176078990	T	G
NC_040252.1	176146122	C	T
NC_040252.1	176810076	A	C
NC_040252.1	176884231	A	G
NC_040252.1	176923488	A	C
NC_040252.1	177003932	A	G
NC_040252.1	177025596	A	G
NC_040252.1	177080678	T	G
NC_040252.1	177147277	G	A
NC_040252.1	177203258	C	T
NC_040252.1	177230510	G	A
NC_040252.1	177336265	G	A
NC_040252.1	177396172	C	T
NC_040252.1	177463470	G	C
NC_040252.1	177491483	T	C
NC_040252.1	177546258	C	T
NC_040252.1	177613578	A	T
NC_040252.1	177671329	A	G
NC_040252.1	177781636	G	A
NC_040252.1	177838843	G	T

NC_040252.1	177902936	A	G
NC_040252.1	177955354	A	G
NC_040252.1	178012599	T	G
NC_040252.1	178075470	G	A
NC_040252.1	178126511	A	G
NC_040252.1	178149465	G	A
NC_040252.1	178212576	C	A
NC_040252.1	178330599	T	C
NC_040252.1	178392146	T	C
NC_040252.1	178458677	G	A
NC_040252.1	178518334	G	A
NC_040252.1	178582801	C	T
NC_040252.1	178647037	G	A
NC_040252.1	178706017	C	T
NC_040252.1	178758393	G	A
NC_040252.1	178815462	T	C
NC_040252.1	178871300	T	A
NC_040252.1	178922755	A	G
NC_040252.1	178978548	A	G
NC_040252.1	179041044	T	C
NC_040252.1	179106603	C	A
NC_040252.1	179175789	T	C
NC_040252.1	179309413	C	A
NC_040252.1	179534298	G	A
NC_040252.1	179589924	G	A
NC_040252.1	179711034	A	G
NC_040252.1	179775838	T	C
NC_040252.1	179832560	G	A
NC_040252.1	179889432	A	G
NC_040252.1	179951884	C	T
NC_040252.1	180012999	A	G
NC_040252.1	180069695	A	G
NC_040252.1	180127897	A	C
NC_040252.1	180190701	G	A
NC_040252.1	180248528	A	G
NC_040252.1	180305044	G	A
NC_040252.1	180371542	C	T
NC_040252.1	180477256	T	C
NC_040252.1	180532796	A	T
NC_040252.1	180585986	G	A
NC_040252.1	180655356	T	C
NC_040252.1	180713570	G	T
NC_040252.1	180762880	C	T
NC_040252.1	180840095	T	G
NC_040252.1	180895968	G	C
NC_040252.1	180949648	T	C
NC_040252.1	181005839	G	A

NC_040252.1	181083719	G	T
NC_040252.1	181138960	A	G
NC_040252.1	181193072	C	T
NC_040252.1	181309988	A	T
NC_040252.1	181365091	C	A
NC_040252.1	181433508	T	C
NC_040252.1	181530381	A	G
NC_040252.1	181612072	G	A
NC_040252.1	181663106	C	T
NC_040252.1	181715214	C	G
NC_040252.1	181772448	T	C
NC_040252.1	181843888	C	T
NC_040252.1	181899321	C	T
NC_040252.1	181955877	C	A
NC_040252.1	182008305	C	T
NC_040252.1	182065823	G	A
NC_040252.1	182124690	A	G
NC_040252.1	182178179	T	C
NC_040252.1	182239642	T	C
NC_040252.1	182575598	C	T
NC_040252.1	182626408	T	C
NC_040252.1	182686040	G	A
NC_040252.1	182743882	T	C
NC_040252.1	182813683	G	A
NC_040252.1	183026995	T	G
NC_040252.1	183097708	G	T
NC_040252.1	183167266	A	G
NC_040252.1	183231312	A	G
NC_040252.1	183300909	G	C
NC_040252.1	183359411	T	C
NC_040252.1	183462049	C	T
NC_040252.1	183500965	A	G
NC_040252.1	183562031	C	T
NC_040252.1	183622853	C	T
NC_040252.1	183706642	G	T
NC_040252.1	183768430	T	C
NC_040252.1	183825302	G	T
NC_040252.1	183886808	A	G
NC_040252.1	184065012	C	T
NC_040252.1	184119235	G	A
NC_040252.1	184150870	T	C
NC_040252.1	184212281	T	G
NC_040252.1	184276184	C	T
NC_040252.1	184330552	A	G
NC_040252.1	184402802	G	A
NC_040252.1	184459613	C	A
NC_040252.1	184516148	A	G

NC_040252.1	184583202	A	G
NC_040252.1	184819744	C	T
NC_040252.1	184879806	C	A
NC_040252.1	184993660	C	T
NC_040252.1	185132378	T	C
NC_040252.1	185177537	T	C
NC_040252.1	185261417	C	A
NC_040252.1	185552204	A	T
NC_040252.1	185683075	C	T
NC_040252.1	185792394	C	T
NC_040252.1	185850952	T	A
NC_040252.1	185904322	G	T
NC_040252.1	185972883	T	G
NC_040252.1	186026583	C	T
NC_040252.1	186138317	C	T
NC_040252.1	186192750	C	T
NC_040252.1	186242254	G	A
NC_040252.1	186309749	G	T
NC_040252.1	186368631	C	T
NC_040252.1	186424666	G	A
NC_040252.1	186492258	G	A
NC_040252.1	186549848	G	T
NC_040252.1	186604022	A	G
NC_040252.1	186664838	A	G
NC_040252.1	186727861	C	A
NC_040252.1	186783534	T	C
NC_040252.1	186839258	A	G
NC_040252.1	186901235	C	G
NC_040252.1	186961465	T	A
NC_040252.1	187021263	G	A
NC_040252.1	187131744	T	C
NC_040252.1	187352255	A	G
NC_040252.1	187412204	A	G
NC_040252.1	187475070	G	T
NC_040252.1	187600512	A	C
NC_040252.1	187658520	G	A
NC_040252.1	187715020	T	G
NC_040252.1	187772495	C	T
NC_040252.1	187827804	T	C
NC_040252.1	187883768	T	C
NC_040252.1	187946256	G	A
NC_040252.1	187965687	C	T
NC_040252.1	188133251	A	G
NC_040252.1	188208661	T	C
NC_040252.1	188357854	C	T
NC_040252.1	188481047	T	C
NC_040252.1	188535015	G	T

NC_040252.1	188593544	T	A
NC_040252.1	188662229	A	G
NC_040252.1	188718724	C	T
NC_040252.1	188777162	G	A
NC_040252.1	188834278	G	A
NC_040252.1	188898995	T	G
NC_040252.1	188963474	G	A
NC_040252.1	189023038	C	T
NC_040252.1	189077439	G	A
NC_040252.1	189131509	A	C
NC_040252.1	189191464	A	G
NC_040252.1	189218233	G	A
NC_040252.1	189315444	T	C
NC_040252.1	189380823	A	T
NC_040252.1	189430871	A	C
NC_040252.1	189488969	C	T
NC_040252.1	189550653	G	A
NC_040252.1	189601069	C	T
NC_040252.1	189672329	G	A
NC_040252.1	189728702	C	T
NC_040252.1	189786480	G	A
NC_040252.1	189843922	T	C
NC_040252.1	189900946	T	C
NC_040252.1	189968596	C	T
NC_040252.1	190032327	A	G
NC_040252.1	190088434	C	T
NC_040252.1	190143439	G	T
NC_040252.1	190210997	C	G
NC_040252.1	190269265	T	G
NC_040252.1	190328608	T	G
NC_040252.1	190386681	T	A
NC_040252.1	190447676	T	C
NC_040252.1	190561198	C	T
NC_040252.1	190619719	C	T
NC_040252.1	190676644	A	G
NC_040252.1	190736253	T	C
NC_040252.1	190797083	T	C
NC_040252.1	190860561	G	A
NC_040252.1	190917229	A	G
NC_040252.1	190972875	G	A
NC_040252.1	190999511	T	G
NC_040252.1	191215515	C	A
NC_040252.1	191273846	A	G
NC_040252.1	191336199	A	G
NC_040252.1	191387124	T	C
NC_040252.1	191404979	T	C
NC_040252.1	191468896	G	A

NC_040252.1	191525218	A	G
NC_040252.1	191545394	G	T
NC_040252.1	191646628	G	C
NC_040252.1	191715522	G	A
NC_040252.1	191766095	C	G
NC_040252.1	191851447	A	G
NC_040252.1	191994440	A	G
NC_040252.1	192005834	T	C
NC_040252.1	192091694	A	C
NC_040252.1	192197237	C	T
NC_040252.1	192250618	G	C
NC_040252.1	192307300	T	G
NC_040252.1	192373130	A	C
NC_040252.1	192442592	C	G
NC_040252.1	192508414	C	T
NC_040252.1	192617444	A	G
NC_040252.1	192734557	C	G
NC_040252.1	192789204	A	G
NC_040252.1	192848761	A	T
NC_040252.1	192918784	T	C
NC_040252.1	192976500	G	T
NC_040252.1	193031700	G	A
NC_040252.1	193098920	A	T
NC_040252.1	193163815	C	T
NC_040252.1	193184456	A	C
NC_040252.1	193613859	C	T
NC_040252.1	193674638	C	T
NC_040252.1	193729972	0	T
NC_040252.1	193795000	G	C
NC_040252.1	193909014	A	G
NC_040252.1	193929584	A	G
NC_040252.1	193982347	T	A
NC_040252.1	194097744	C	G
NC_040252.1	194158719	T	G
NC_040252.1	194219788	T	C
NC_040252.1	194271008	C	T
NC_040252.1	194292783	A	G
NC_040252.1	194342994	G	A
NC_040252.1	194408782	G	A
NC_040252.1	194465463	C	T
NC_040252.1	194519812	A	G
NC_040252.1	194580608	T	G
NC_040252.1	194632560	C	T
NC_040252.1	194697325	A	G
NC_040252.1	194817270	T	C
NC_040252.1	194883405	A	G
NC_040252.1	194995017	G	A

NC_040252.1	195056284	G	C
NC_040252.1	195114517	A	G
NC_040252.1	195175970	C	A
NC_040252.1	195347032	A	G
NC_040252.1	195413379	A	G
NC_040252.1	195474557	T	C
NC_040252.1	195525914	C	T
NC_040252.1	195584342	A	G
NC_040252.1	195646861	C	T
NC_040252.1	195703122	T	C
NC_040252.1	195761626	T	G
NC_040252.1	195819294	C	T
NC_040252.1	195885291	T	G
NC_040252.1	195943147	T	C
NC_040252.1	195966644	T	G
NC_040252.1	196007886	A	G
NC_040252.1	196065008	C	T
NC_040252.1	196115765	T	C
NC_040252.1	196173090	G	A
NC_040252.1	196228311	A	G
NC_040252.1	196283301	A	G
NC_040252.1	196343909	T	C
NC_040252.1	196403918	T	C
NC_040252.1	196467516	A	T
NC_040252.1	196528322	C	G
NC_040252.1	196583744	A	G
NC_040252.1	196640363	C	G
NC_040252.1	196700498	T	C
NC_040252.1	196763829	G	A
NC_040252.1	196816800	T	C
NC_040252.1	196873416	C	G
NC_040252.1	196930113	A	T
NC_040252.1	196990131	T	C
NC_040252.1	197066774	T	C
NC_040252.1	197253310	A	G
NC_040252.1	197380549	A	G
NC_040252.1	197440010	A	T
NC_040252.1	197494258	T	C
NC_040252.1	197553918	A	T
NC_040252.1	197663420	C	T
NC_040252.1	197719370	A	G
NC_040252.1	197794018	A	G
NC_040252.1	197864291	A	G
NC_040252.1	197928277	T	C
NC_040252.1	197989066	A	T
NC_040252.1	198051882	T	C
NC_040252.1	198115957	G	A

NC_040252.1	198176670	A	T
NC_040252.1	198250647	A	G
NC_040252.1	198363533	G	C
NC_040252.1	198422166	A	G
NC_040252.1	198473779	G	A
NC_040252.1	198507059	T	C
NC_040252.1	198564858	C	T
NC_040252.1	198615346	T	C
NC_040252.1	198644115	T	C
NC_040252.1	198706756	A	C
NC_040252.1	198769686	A	G
NC_040252.1	198829316	T	G
NC_040252.1	198887176	A	C
NC_040252.1	198965891	A	G
NC_040252.1	199032873	C	G
NC_040252.1	199094616	T	G
NC_040252.1	199168349	T	C
NC_040252.1	199331293	A	G
NC_040252.1	199392315	G	C
NC_040252.1	199451262	C	T
NC_040252.1	199515567	G	A
NC_040252.1	199581732	C	T
NC_040252.1	199643324	A	G
NC_040252.1	199713836	C	T
NC_040252.1	199774183	G	C
NC_040252.1	200006806	T	C
NC_040252.1	200072276	A	G
NC_040252.1	200134917	T	C
NC_040252.1	200201221	G	A
NC_040252.1	200257409	C	T
NC_040252.1	200319999	T	C
NC_040252.1	200385703	T	C
NC_040252.1	200453530	A	G
NC_040252.1	200514756	T	C
NC_040252.1	200575003	C	T
NC_040252.1	200633363	C	T
NC_040252.1	200689044	A	G
NC_040252.1	200748428	A	G
NC_040252.1	200805591	T	A
NC_040252.1	200859948	C	T
NC_040252.1	200920491	C	G
NC_040252.1	200988196	A	G
NC_040252.1	201058327	A	G
NC_040252.1	201114687	G	A
NC_040252.1	201176444	C	G
NC_040252.1	201233398	A	G
NC_040252.1	201291351	C	A

NC_040252.1	201351677	C	T
NC_040252.1	201406372	G	A
NC_040252.1	201479145	G	T
NC_040252.1	201537835	T	C
NC_040252.1	201593869	T	C
NC_040252.1	201654104	T	C
NC_040252.1	201736921	A	G
NC_040252.1	201898424	A	G
NC_040252.1	201954154	C	T
NC_040252.1	202015572	T	C
NC_040252.1	202081108	A	G
NC_040252.1	202134046	G	T
NC_040252.1	202179672	T	C
NC_040252.1	202245879	A	G
NC_040252.1	202306346	T	G
NC_040252.1	202372905	C	T
NC_040252.1	202434374	T	C
NC_040252.1	202494404	T	C
NC_040252.1	202549185	C	A
NC_040252.1	202617333	C	A
NC_040252.1	202672211	G	T
NC_040252.1	202729515	C	T
NC_040252.1	202785537	G	A
NC_040252.1	202844421	C	T
NC_040252.1	202903464	A	G
NC_040252.1	202960396	G	C
NC_040252.1	203020564	C	T
NC_040252.1	203078736	C	T
NC_040252.1	203135160	A	G
NC_040252.1	203193112	C	G
NC_040252.1	203249885	A	C
NC_040252.1	203313664	T	C
NC_040252.1	203369905	T	G
NC_040252.1	203428338	A	G
NC_040252.1	203494534	T	A
NC_040252.1	203560429	C	T
NC_040252.1	203616194	T	C
NC_040252.1	203796310	A	G
NC_040252.1	203847608	C	T
NC_040252.1	203966333	C	T
NC_040252.1	204134549	A	G
NC_040252.1	204193117	A	G
NC_040252.1	204255626	T	G
NC_040252.1	204314567	G	A
NC_040252.1	204369974	G	C
NC_040252.1	204423049	G	A
NC_040252.1	204479457	C	T

NC_040252.1	204545739	C	G
NC_040252.1	204596017	T	A
NC_040252.1	204655936	C	G
NC_040252.1	204714761	C	T
NC_040252.1	204772640	G	T
NC_040252.1	204827182	T	C
NC_040252.1	204885832	G	C
NC_040252.1	204940993	A	G
NC_040252.1	204994071	G	A
NC_040252.1	205049069	G	T
NC_040252.1	205109737	G	C
NC_040252.1	205222324	C	T
NC_040252.1	205276995	G	A
NC_040252.1	205339038	C	G
NC_040252.1	205399479	G	A
NC_040252.1	205457639	T	G
NC_040252.1	205513767	G	A
NC_040252.1	205569514	T	C
NC_040252.1	205644389	A	G
NC_040252.1	205740161	T	C
NC_040252.1	205798265	T	C
NC_040252.1	205860496	C	T
NC_040252.1	205916355	T	C
NC_040252.1	205967379	G	A
NC_040252.1	206028894	C	T
NC_040252.1	206092299	T	C
NC_040252.1	206148614	A	G
NC_040252.1	206205564	C	T
NC_040252.1	206259199	C	T
NC_040252.1	206319523	C	T
NC_040252.1	206372764	C	T
NC_040252.1	206440046	A	G
NC_040252.1	206491182	T	A
NC_040252.1	206547925	T	C
NC_040252.1	206605437	T	C
NC_040252.1	206663185	T	C
NC_040252.1	206718915	G	T
NC_040252.1	206776740	G	A
NC_040252.1	206834838	G	T
NC_040252.1	206899723	C	T
NC_040252.1	206935409	C	T
NC_040252.1	206986838	C	T
NC_040252.1	207046469	A	T
NC_040252.1	207103816	T	C
NC_040252.1	207158300	A	G
NC_040252.1	207217249	T	C
NC_040252.1	207276718	G	A

NC_040252.1	207341187	C	G
NC_040252.1	207400338	G	C
NC_040252.1	207455386	A	G
NC_040252.1	207514356	A	G
NC_040252.1	207571140	C	T
NC_040252.1	207631011	C	T
NC_040252.1	207685746	A	C
NC_040252.1	207740415	G	A
NC_040252.1	207798489	T	G
NC_040252.1	207859510	T	G
NC_040252.1	207926448	G	C
NC_040252.1	207985591	G	C
NC_040252.1	208039206	T	C
NC_040252.1	208098025	A	T
NC_040252.1	208153576	T	C
NC_040252.1	208215390	G	A
NC_040252.1	208282115	T	C
NC_040252.1	208406125	T	C
NC_040252.1	208547181	T	C
NC_040252.1	208743665	T	C
NC_040252.1	209433019	T	C
NC_040252.1	209971399	T	C
NC_040252.1	210174629	C	G
NC_040252.1	211049914	A	G
NC_040252.1	211669441	T	C
NC_040252.1	211808108	G	A
NC_040252.1	212093020	T	C
NC_040252.1	212465171	A	C
NC_040252.1	212785880	T	C
NC_040252.1	213433328	A	C
NC_040252.1	213497041	T	C
NC_040252.1	213558301	0	C
NC_040252.1	213684173	A	G
NC_040252.1	213788756	C	T
NC_040252.1	213847649	A	G
NC_040252.1	213909005	T	C
NC_040252.1	213964893	A	G
NC_040252.1	214268166	G	T
NC_040252.1	214331802	A	G
NC_040252.1	214453815	G	A
NC_040252.1	214518247	A	G
NC_040252.1	214592362	G	A
NC_040252.1	214649490	C	T
NC_040252.1	214713042	A	G
NC_040252.1	214789289	G	A
NC_040252.1	214866009	A	G
NC_040252.1	214914657	C	A

NC_040252.1	215230250	A	G
NC_040252.1	215281661	A	G
NC_040252.1	215356756	A	G
NC_040252.1	215420766	C	T
NC_040252.1	215481409	A	G
NC_040252.1	215589499	G	T
NC_040252.1	215649507	G	A
NC_040252.1	215721016	G	A
NC_040252.1	215779148	G	A
NC_040252.1	215843420	T	C
NC_040252.1	215900668	A	C
NC_040252.1	215955722	G	A
NC_040252.1	216016340	A	G
NC_040252.1	216071735	T	C
NC_040252.1	216127252	A	G
NC_040252.1	216186654	G	A
NC_040252.1	216239705	C	T
NC_040252.1	216301619	A	G
NC_040252.1	216380769	T	G
NC_040252.1	216400951	G	A
NC_040252.1	216684151	T	G
NC_040252.1	216885573	T	C
NC_040252.1	217511439	A	C
NC_040252.1	217909870	A	G
NC_040252.1	218604024	G	T
NC_040252.1	219012068	A	G
NC_040252.1	219172602	A	G
NC_040252.1	219378969	G	A
NC_040252.1	219545210	T	C
NC_040252.1	219683566	T	C
NC_040252.1	219870678	A	G
NC_040252.1	219928046	G	T
NC_040252.1	219981542	A	G
NC_040252.1	220047698	A	G
NC_040252.1	220094840	C	G
NC_040252.1	220151395	T	C
NC_040252.1	220208052	T	C
NC_040252.1	220264076	A	G
NC_040252.1	220325972	T	C
NC_040252.1	220387143	A	G
NC_040252.1	220438149	T	C
NC_040252.1	220499101	A	G
NC_040252.1	220561364	T	C
NC_040252.1	220619376	G	T
NC_040252.1	220681084	T	C
NC_040252.1	220750882	T	C
NC_040252.1	220805086	A	G

NC_040252.1	220867988	A	G
NC_040252.1	220912106	C	T
NC_040252.1	221059929	C	G
NC_040252.1	221592777	A	T
NC_040252.1	221866234	C	A
NC_040252.1	221974202	T	C
NC_040252.1	222096037	T	A
NC_040252.1	222701211	G	A
NC_040252.1	222893543	C	T
NC_040252.1	223335090	A	G
NC_040252.1	223743132	A	C
NC_040252.1	223866316	C	G
NC_040252.1	224200607	A	G
NC_040252.1	224532361	A	G
NC_040252.1	224674190	C	T
NC_040252.1	225290326	A	G
NC_040252.1	225345055	C	A
NC_040252.1	225399530	T	A
NC_040252.1	225455478	C	T
NC_040252.1	225527312	A	G
NC_040252.1	225585076	T	C
NC_040252.1	225640122	T	C
NC_040252.1	225701753	C	T
NC_040252.1	225756899	A	G
NC_040252.1	225834673	C	T
NC_040252.1	225891230	C	G
NC_040252.1	225949183	C	A
NC_040252.1	226017444	C	T
NC_040252.1	226085450	G	A
NC_040252.1	226145822	G	A
NC_040252.1	226262649	G	A
NC_040252.1	226304266	A	G
NC_040252.1	227219756	A	G
NC_040252.1	227960994	G	A
NC_040252.1	228528017	T	C
NC_040252.1	229372058	A	G
NC_040252.1	230161739	C	A
NC_040252.1	230857445	C	A
NC_040252.1	230932536	A	G
NC_040252.1	231005515	C	G
NC_040252.1	231072056	T	G
NC_040252.1	231127839	C	T
NC_040252.1	231186806	T	C
NC_040252.1	231246533	T	C
NC_040252.1	231320772	A	G
NC_040252.1	231400668	A	C
NC_040252.1	231454836	G	C

NC_040252.1	231517201	A	G
NC_040252.1	231565452	C	A
NC_040252.1	231600054	G	A
NC_040252.1	231650865	C	T
NC_040252.1	231755983	A	G
NC_040252.1	231811935	G	C
NC_040252.1	231904248	G	T
NC_040252.1	231965122	A	G
NC_040252.1	232001942	A	C
NC_040252.1	232643642	T	C
NC_040252.1	232895073	T	C
NC_040252.1	233183323	A	G
NC_040252.1	233473087	C	A
NC_040252.1	234058348	C	T
NC_040252.1	234276071	C	T
NC_040252.1	234460774	A	G
NC_040252.1	234600031	C	T
NC_040252.1	234742442	G	A
NC_040252.1	235082887	A	G
NC_040252.1	235312560	G	A
NC_040252.1	235585391	A	G
NC_040252.1	235829255	A	G
NC_040252.1	236645440	C	G
NC_040252.1	236813593	C	G
NC_040252.1	236976868	A	G
NC_040252.1	237109110	G	A
NC_040252.1	237256076	C	T
NC_040252.1	237307806	A	G
NC_040252.1	237366217	A	G
NC_040252.1	237429805	C	T
NC_040252.1	237541791	C	A
NC_040252.1	237595512	A	T
NC_040252.1	237651938	T	C
NC_040252.1	237712777	A	G
NC_040252.1	237786486	G	A
NC_040252.1	237840963	G	A
NC_040252.1	237963292	C	T
NC_040252.1	238030034	A	G
NC_040252.1	238099131	A	T
NC_040252.1	238158901	C	T
NC_040252.1	238213919	G	T
NC_040252.1	238283976	T	C
NC_040252.1	238346561	T	C
NC_040252.1	239016976	G	A
NC_040252.1	239936174	T	A
NC_040252.1	240214771	G	A
NC_040252.1	240279086	A	T

NC_040252.1	240334278	A	C
NC_040252.1	240401095	A	G
NC_040252.1	240467927	A	C
NC_040252.1	240534185	C	T
NC_040252.1	240600161	T	G
NC_040252.1	240642040	T	G
NC_040252.1	240698327	G	A
NC_040252.1	240777598	A	G
NC_040252.1	240901813	C	G
NC_040252.1	240961974	A	G
NC_040252.1	241019142	T	C
NC_040252.1	241087008	A	G
NC_040252.1	241386536	A	G
NC_040252.1	241428764	G	C
NC_040252.1	241634034	T	C
NC_040252.1	242175682	A	G
NC_040252.1	242741793	T	C
NC_040252.1	242861092	C	T
NC_040252.1	243023395	A	T
NC_040252.1	243114250	G	C
NC_040252.1	243132619	T	C
NC_040252.1	243373006	C	T
NC_040252.1	243629023	A	G
NC_040252.1	243682022	T	C
NC_040252.1	243767400	G	T
NC_040252.1	243783633	C	T
NC_040252.1	243834116	A	C
NC_040252.1	244282267	A	T
NC_040252.1	245033564	T	C
NC_040252.1	245467034	A	G
NC_040252.1	245732804	T	G
NC_040252.1	246099410	T	C
NC_040252.1	246734949	G	A
NC_040252.1	247014410	A	G
NC_040252.1	247210384	C	T
NC_040252.1	247628991	G	T
NC_040252.1	247718163	A	G
NC_040252.1	247926694	G	A
NC_040252.1	248393576	G	A
NC_040252.1	248915552	A	G
NC_040252.1	249245571	T	C
NC_040252.1	249634487	0	G
NC_040252.1	250386248	C	A
NC_040252.1	250460709	G	C
NC_040252.1	250682091	A	T
NC_040252.1	251184440	C	T
NC_040252.1	251353200	A	G

NC_040252.1	251488813	G	A
NC_040252.1	251549328	G	A
NC_040252.1	251885167	G	T
NC_040252.1	252209941	G	A
NC_040252.1	252746589	G	C
NC_040252.1	253024012	G	A
NC_040252.1	253228226	C	T
NC_040252.1	253397302	G	A
NC_040252.1	253882819	G	A
NC_040252.1	254088797	C	T
NC_040252.1	254425231	G	A
NC_040252.1	254799522	G	T
NC_040252.1	254888191	G	A
NC_040252.1	255311298	A	G
NC_040252.1	255665537	T	C
NC_040252.1	255837112	A	G
NC_040252.1	256155934	C	T
NC_040252.1	256443162	T	C
NC_040252.1	256493694	T	A
NC_040252.1	256892188	C	T
NC_040252.1	257949091	G	A
NC_040252.1	258466900	T	C
NC_040252.1	258617016	A	T
NC_040252.1	258711362	A	G
NC_040252.1	258958381	G	A
NC_040252.1	259380095	G	T
NC_040252.1	259744661	A	G
NC_040252.1	259810215	C	T
NC_040252.1	260362143	G	A
NC_040252.1	260422366	C	G
NC_040252.1	260595098	T	C
NC_040252.1	261004001	G	A
NC_040252.1	261413828	T	A
NC_040252.1	261787645	C	T
NC_040252.1	262481350	C	T
NC_040252.1	262731254	G	A
NC_040252.1	263438492	T	C
NC_040252.1	264107179	G	A
NC_040252.1	264526473	G	A
NC_040252.1	264583909	A	G
NC_040252.1	264643682	C	T
NC_040252.1	264737188	T	C
NC_040252.1	264998077	G	A
NC_040252.1	265124493	C	T
NC_040252.1	265333027	A	C
NC_040252.1	265404068	C	T
NC_040252.1	265427881	A	G

NC_040252.1	265526550	C	T
NC_040252.1	265700999	C	A
NC_040252.1	265765488	T	C
NC_040252.1	265937097	G	A
NC_040252.1	265991732	A	G
NC_040252.1	266066419	T	C
NC_040252.1	266113959	G	A
NC_040252.1	266297708	G	T
NC_040252.1	266348382	T	A
NC_040252.1	266411131	A	G
NC_040252.1	266470833	T	G
NC_040252.1	266580431	G	C
NC_040252.1	266655639	T	G
NC_040252.1	266717979	T	C
NC_040252.1	266826894	A	T
NC_040252.1	266896092	T	A
NC_040252.1	266954582	T	C
NC_040252.1	267017942	C	T
NC_040252.1	267073706	T	C
NC_040252.1	267127437	C	G
NC_040252.1	267193758	C	G
NC_040252.1	267247483	A	G
NC_040252.1	267309882	A	G
NC_040252.1	267499519	G	A
NC_040252.1	268206268	A	G
NC_040252.1	268335227	C	T
NC_040252.1	268503544	T	C
NC_040252.1	268830320	G	T
NC_040252.1	268944187	G	A
NC_040252.1	269052329	A	G
NC_040252.1	269272901	T	C
NC_040252.1	269507318	G	T
NC_040252.1	269610829	G	A
NC_040252.1	270131003	C	G
NC_040252.1	270340116	T	C
NC_040252.1	270424505	G	A
NC_040252.1	270545181	G	A
NC_040252.1	270847348	C	T
NC_040252.1	271051262	T	C
NC_040252.1	271459974	A	C
NC_040252.1	271658096	T	C
NC_040252.1	272381341	T	C
NC_040252.1	272614063	A	G
NC_040252.1	273375464	A	C
NC_040252.1	274315307	T	C
NC_040252.1	274648447	T	A
NC_040252.1	274911111	C	T

NC_040252.1	275001348	T		A
NC_040252.1	275118144		0	G
NC_040252.1	275233360	T		A
NC_040252.1	275434778	G		A
NC_040252.1	275472139	C		A
NC_040252.1	275537126	A		C
NC_040252.1	275684515	C		T
NC_040252.1	275821824	T		C
NC_040252.1	275953081	A		G
NC_040252.1	276457575	C		T
NC_040252.1	276640217	G		T
NC_040252.1	276888369	C		T
NC_040252.1	276968718	C		A
NC_040252.1	277089561	A		G
NC_040252.1	277188730	A		G
NC_040252.1	277451850	T		G
NC_040252.1	277595303	C		G
NC_040252.1	277719410	G		C
NC_040252.1	278121815	T		C
NC_040252.1	278237134	A		G
NC_040252.1	278412610	A		G
NC_040252.1	278530257	A		G
NC_040252.1	279181144	T		C
NC_040252.1	279214714	G		A
NC_040252.1	279398995	C		T
NC_040252.1	279547704	C		T
NC_040252.1	279646210	G		A
NC_040252.1	279700533	C		A
NC_040252.1	279729204	G		A
NC_040252.1	279848877	C		T
NC_040252.1	279963390	G		A
NC_040252.1	280036014	T		G
NC_040252.1	280114040	T		C
NC_040252.1	280131021	G		T
NC_040252.1	280487960	G		A
NC_040252.1	280542794	C		G
NC_040252.1	280563936	G		A
NC_040252.1	280844952	A		G
NC_040252.1	280985369	G		A
NC_040252.1	281028479	T		G
NC_040252.1	281056859	C		T
NC_040252.1	281407519	A		G
NC_040252.1	281469435	G		C
NC_040252.1	281679373	A		G
NC_040252.1	281704804	C		T
NC_040252.1	281766925	A		C
NC_040252.1	281873802	A		G

NC_040252.1	281917635	T	C
NC_040252.1	281941774	C	A
NC_040252.1	281992731	G	A
NC_040252.1	282089413	T	C
NC_040252.1	282230424	A	T
NC_040252.1	282271560	G	A
NC_040252.1	282354598	T	C
NC_040252.1	282370119	T	A
NC_040252.1	282418961	T	G
NC_040252.1	282531582	T	C
NC_040252.1	282552415	A	G
NC_040252.1	282836565	G	A
NC_040252.1	282852959	C	T
NC_040252.1	282863577	G	A
NC_040252.1	283088110	T	C
NC_040252.1	283161993	G	C
NC_040252.1	283250983	C	T
NC_040252.1	283616961	C	T
NC_040252.1	283748727	A	C
NC_040252.1	283797751	C	T
NC_040252.1	283903471	A	G
NC_040252.1	283992599	A	G
NC_040252.1	284071280	G	T
NC_040252.1	284185605	A	C
NC_040252.1	284750390	A	G
NC_040252.1	284921751	A	G
NC_040252.1	284963032	T	C
NC_040252.1	284992414	A	G
NC_040252.1	285110814	G	C
NC_040252.1	285156136	T	C
NC_040252.1	285236632	G	A
NC_040252.1	285471451	A	G
NC_040252.1	285528775	A	G
NC_040252.1	285602975	C	G
NC_040252.1	285793863	T	C
NC_040252.1	285810346	C	G
NC_040252.1	285857200	T	C
NC_040252.1	285941534	A	G
NC_040252.1	286096999	G	A
NC_040252.1	286130433	T	C
NC_040252.1	286183843	A	G
NC_040252.1	286239002	G	A
NC_040252.1	286321126	A	G
NC_040252.1	286482537	G	A
NC_040252.1	286548793	A	T
NC_040252.1	286572854	T	C
NC_040252.1	286612008	T	G

NC_040252.1	286657919	A	T
NC_040252.1	286682448	A	G
NC_040252.1	286792980	G	A
NC_040252.1	286844537	G	A
NC_040252.1	286925886	A	G
NC_040252.1	286972924	C	T
NC_040252.1	286996179	G	A
NC_040252.1	287033885	T	G
NC_040252.1	287045533	A	G
NC_040252.1	287068945	G	C
NC_040252.1	287114196	A	G
NC_040252.1	287179807	T	C
NC_040252.1	287209181	T	A
NC_040252.1	287230233	C	T
NC_040252.1	287264333	T	C
NC_040252.1	287338610	G	A
NC_040252.1	287344956	A	G
NC_040252.1	287347643	T	C
NC_040252.1	287374877	G	A
NC_040252.1	287403677	C	T
NC_040252.1	287452699	A	G
NC_040252.1	287482665	A	G
NC_040252.1	287521576	C	A
NC_040252.1	287551992	A	G
NC_040252.1	287588299	G	A
NC_040252.1	287605814	T	C
NC_040252.1	287663054	T	C
NC_040252.1	287704187	T	C
NC_040252.1	287708122	T	C
NC_040252.1	287749254	A	G
NC_040252.1	287776230	A	G
NC_040252.1	287811212	A	G
NC_040252.1	287860484	T	C
NC_040252.1	287952590	A	G
NC_040252.1	287971109	T	G
NC_040252.1	287998990	C	T
NC_040252.1	288043259	A	G
NC_040252.1	288058181	T	C
NC_040252.1	288108974	T	C
NC_040252.1	288145131	G	A
NC_040252.1	288149812	A	G
NC_040252.1	288167693	T	C
NC_040252.1	288184278	A	G
NC_040252.1	288230616	G	C
NC_040252.1	288252352	T	C
NC_040252.1	288278472	T	C
NC_040252.1	288305316	C	G

NC_040252.1	288360797	C		T
NC_040252.1	288391717		0	T
NC_040252.1	288443468	C		T
NC_040252.1	288526884	A		G
NC_040252.1	288566678	C		G
NC_040252.1	288613218	A		G
NC_040252.1	288679003	G		A
NC_040252.1	288710437	G		C
NC_040252.1	288739885	G		C
NC_040252.1	288764731	T		C
NC_040252.1	288793664	G		A
NC_040252.1	288834912	T		C
NC_040252.1	288860159	T		C
NC_040252.1	288922282	A		G
NC_040252.1	288986335	T		C
NC_040252.1	289032789	T		G
NC_040252.1	289039583	A		G
NC_040252.1	289052110	T		C
NC_040252.1	289097006	A		G
NC_040252.1	289125610	T		G
NC_040252.1	289158973	C		T
NC_040252.1	289181308	A		G
NC_040252.1	289201609	T		A
NC_040252.1	289260221	C		T
NC_040252.1	289294764	G		C
NC_040252.1	289313765	T		C
NC_040252.1	289317504	A		G
NC_040252.1	289368091	A		C
NC_040252.1	289375408	T		C
NC_040252.1	289432146	A		C
NC_040252.1	289491727	T		C
NC_040252.1	289550247	T		C
NC_040252.1	289578780	T		C
NC_040252.1	289609739	C		T
NC_040252.1	289632618	A		G
NC_040252.1	289651336	C		G
NC_040252.1	289699592	C		G
NC_040252.1	289772527	C		T
NC_040252.1	289790017	A		G
NC_040252.1	289831331	C		T
NC_040252.1	289854802	T		C
NC_040252.1	289893180	A		T
NC_040252.1	289920685	A		G
NC_040252.1	289963503	T		C
NC_040252.1	290018203	A		G
NC_040252.1	290067979	T		C
NC_040252.1	290103186	C		T

NC_040252.1	290147599	G	A
NC_040252.1	290174290	T	C
NC_040252.1	290202671	C	T
NC_040252.1	290239444	G	C
NC_040252.1	290294201	G	A
NC_040252.1	290355603	T	A
NC_040252.1	290413506	T	C
NC_040252.1	290448856	A	G
NC_040252.1	290503900	T	C
NC_040252.1	290569078	A	G
NC_040252.1	290604707	C	T
NC_040252.1	290621907	T	A
NC_040252.1	290679779	T	C
NC_040252.1	290738485	A	G
NC_040252.1	290798221	A	G
NC_040252.1	290830923	T	A
NC_040252.1	290919968	G	A
NC_040252.1	290942443	C	T
NC_040252.1	290956872	T	C
NC_040252.1	290990421	G	A
NC_040252.1	291011639	T	C
NC_040252.1	291044614	T	C
NC_040252.1	291067179	A	G
NC_040252.1	291119783	G	T
NC_040252.1	291151565	G	T
NC_040252.1	291192321	A	T
NC_040252.1	291242472	A	G
NC_040252.1	291299185	T	C
NC_040252.1	291364212	A	G
NC_040252.1	291435237	T	C
NC_040252.1	291484034	T	G
NC_040252.1	291548169	A	G
NC_040252.1	291641429	C	T
NC_040252.1	291699212	A	G
NC_040252.1	291721256	A	G
NC_040252.1	291747405	A	G
NC_040252.1	291796429	T	C
NC_040252.1	291832131	G	T
NC_040252.1	291885487	A	C
NC_040252.1	291913714	C	T
NC_040252.1	291926460	G	C
NC_040252.1	291963877	G	A
NC_040252.1	291990930	A	G
NC_040252.1	292042110	G	C
NC_040252.1	292095199	G	A
NC_040252.1	292149635	T	C
NC_040252.1	292199650	A	C

NC_040252.1	292226885	G	A
NC_040252.1	292273522	A	G
NC_040252.1	292282276	C	T
NC_040252.1	292333842	T	C
NC_040252.1	292399061	T	A
NC_040252.1	292454187	G	A
NC_040252.1	292489754	G	A
NC_040252.1	292506230	T	C
NC_040252.1	292560827	C	T
NC_040252.1	292608866	G	A
NC_040252.1	292685473	G	T
NC_040252.1	292726022	T	C
NC_040252.1	292777831	C	T
NC_040252.1	292810655	A	G
NC_040252.1	292858111	G	A
NC_040252.1	292911471	A	G
NC_040252.1	292943387	T	C
NC_040252.1	292981133	T	G
NC_040252.1	293258827	G	A
NC_040252.1	293294058	G	A
NC_040252.1	293358237	A	G
NC_040252.1	293362213	G	A
NC_040252.1	293411029	G	A
NC_040252.1	293444316	T	C
NC_040252.1	293515306	T	C
NC_040252.1	293535398	C	T
NC_040252.1	293600903	T	C
NC_040252.1	293644958	T	G
NC_040252.1	293719141	C	G
NC_040252.1	293825253	C	A
NC_040252.1	293882207	A	G
NC_040252.1	293916914	G	A
NC_040252.1	293969435	G	A
NC_040252.1	294022183	T	G
NC_040252.1	294054386	G	T
NC_040252.1	294128384	A	C
NC_040252.1	294133780	T	C
NC_040252.1	294209110	G	C
NC_040252.1	294291963	T	C
NC_040252.1	294362895	A	C
NC_040252.1	294403822	G	A
NC_040252.1	294435354	A	G
NC_040252.1	294455136	C	A
NC_040252.1	294517120	G	A
NC_040252.1	294585109	G	T
NC_040252.1	294628569	G	T
NC_040252.1	294677999	G	C

NC_040252.1	294721883	G	A
NC_040252.1	294738792	G	C
NC_040252.1	294792559	A	G
NC_040252.1	294859046	T	C
NC_040252.1	295109879	T	C
NC_040252.1	295161866	T	C
NC_040252.1	295207674	A	C
NC_040252.1	295245410	G	A
NC_040252.1	295299534	C	G
NC_040252.1	295419922	A	G
NC_040252.1	295472577	T	C
NC_040252.1	295516036	A	G
NC_040252.1	295522289	T	C
NC_040252.1	295636661	A	G
NC_040252.1	295711880	G	A
NC_040252.1	295812922	A	G
NC_040252.1	295841424	T	C
NC_040252.1	295896420	A	G
NC_040252.1	295950247	A	G
NC_040252.1	295979444	T	C
NC_040252.1	296044032	A	C
NC_040252.1	296068885	C	T
NC_040252.1	296105894	C	T
NC_040252.1	296143293	C	T
NC_040252.1	296182763	C	T
NC_040252.1	296205444	G	A
NC_040252.1	296264059	G	T
NC_040252.1	296294281	A	G
NC_040252.1	296329041	C	T
NC_040252.1	296375158	A	G
NC_040252.1	296443689	G	A
NC_040252.1	296444425	G	A
NC_040252.1	296512258	T	C
NC_040252.1	296554828	G	A
NC_040252.1	296599101	T	C
NC_040252.1	296663295	G	A
NC_040252.1	296718131	C	T
NC_040252.1	296779635	C	G
NC_040252.1	296804387	G	A
NC_040252.1	296820481	G	A
NC_040252.1	296872651	C	T
NC_040252.1	296920824	A	G
NC_040252.1	296939519	A	G
NC_040252.1	296992281	T	C
NC_040252.1	297049888	G	A
NC_040252.1	297110821	G	A
NC_040252.1	297168423	G	C

NC_040252.1	297220683	T	C
NC_040252.1	297263305	A	G
NC_040252.1	297271635	0	G
NC_040252.1	297326787	G	A
NC_040252.1	297375001	C	G
NC_040252.1	297420801	C	T
NC_040252.1	297466385	T	C
NC_040252.1	297497403	A	G
NC_040252.1	297550928	G	A
NC_040252.1	297600433	C	T
NC_040252.1	297655482	G	A
NC_040252.1	297700428	A	G
NC_040252.1	297756079	A	C
NC_040252.1	297810472	G	A
NC_040252.1	297867882	C	T
NC_040252.1	297933098	C	T
NC_040252.1	297989178	A	G
NC_040252.1	298044846	G	A
NC_040252.1	298101601	T	C
NC_040252.1	298167078	C	T
NC_040252.1	298247122	C	T
NC_040252.1	298310613	A	G
NC_040252.1	298359047	0	G
NC_040252.1	298381994	G	C
NC_040252.1	298456164	G	A
NC_040252.1	298489960	A	G
NC_040252.1	298534605	G	A
NC_040252.1	298592385	T	G
NC_040252.1	298634150	T	C
NC_040252.1	298649133	T	C
NC_040252.1	298713252	C	A
NC_040252.1	298745089	A	T
NC_040252.1	298838074	A	G
NC_040252.1	298899189	C	T
NC_040252.1	298956106	G	A
NC_040252.1	298990266	G	A
NC_040252.1	299009318	C	T
NC_040252.1	299057061	A	C
NC_040252.1	299111085	T	C
NC_040252.1	299163923	G	T
NC_040252.1	299183545	T	C
NC_040252.1	299236312	C	G
NC_040252.1	299271632	A	G
NC_040252.1	299310822	G	A
NC_040252.1	299342892	G	C
NC_040252.1	299383033	T	G
NC_040252.1	299415222	T	C

NC_040252.1	299438415	A	G
NC_040252.1	299480218	T	C
NC_040252.1	299495256	G	T
NC_040252.1	299524515	A	G
NC_040252.1	299556928	G	A
NC_040252.1	299581271	G	A
NC_040252.1	299605069	T	C
NC_040252.1	299646385	T	C
NC_040252.1	299662470	A	C
NC_040252.1	299698647	G	A
NC_040252.1	299728314	A	G
NC_040252.1	299776551	A	G
NC_040252.1	299826095	G	C
NC_040252.1	299895718	G	A
NC_040252.1	299937844	A	G
NC_040252.1	299997690	T	C
NC_040252.1	300031049	A	G
NC_040252.1	300098517	C	T
NC_040252.1	300133756	T	C
NC_040252.1	300225853	G	C
NC_040252.1	300233379	A	G
NC_040252.1	300293924	C	T
NC_040252.1	300353070	G	A
NC_040252.1	300402606	A	G
NC_040252.1	300457677	C	A
NC_040252.1	300470821	G	C
NC_040252.1	300530710	T	C
NC_040252.1	300589826	G	A
NC_040252.1	300654223	G	A
NC_040252.1	300719729	T	C
NC_040252.1	300768636	A	G
NC_040252.1	300830335	T	A
NC_040252.1	300860690	T	C
NC_040252.1	300904398	T	C
NC_040252.1	300913406	T	C
NC_040252.1	300956384	T	C
NC_040252.1	301013236	G	C
NC_040252.1	301069752	A	G
NC_040252.1	301081788	A	C
NC_040252.1	301083867	T	C
NC_040252.1	301104229	C	A
NC_040252.1	301104469	T	C
NC_040252.1	301137418	A	G
NC_040252.1	301166347	C	T
NC_040252.1	301192034	T	C
NC_040252.1	301202146	T	C
NC_040252.1	301202607	A	T

NC_040252.1	301233800	G	A
NC_040252.1	301253302	C	T
NC_040253.1	36405	C	A
NC_040253.1	80755	C	T
NC_040253.1	85043	C	G
NC_040253.1	85275	C	G
NC_040253.1	135612	G	A
NC_040253.1	167841	G	A
NC_040253.1	168529	A	T
NC_040253.1	216775	A	G
NC_040253.1	216956	G	A
NC_040253.1	230093	C	A
NC_040253.1	253132	G	C
NC_040253.1	308300	A	G
NC_040253.1	356172	T	C
NC_040253.1	412924	G	C
NC_040253.1	466062	A	T
NC_040253.1	516587	T	C
NC_040253.1	564007	A	G
NC_040253.1	611283	G	A
NC_040253.1	615142	C	A
NC_040253.1	659655	C	T
NC_040253.1	746988	G	A
NC_040253.1	836839	G	C
NC_040253.1	846351	A	G
NC_040253.1	902289	A	G
NC_040253.1	903659	T	C
NC_040253.1	903933	G	A
NC_040253.1	904171	A	G
NC_040253.1	904758	G	C
NC_040253.1	961401	T	C
NC_040253.1	1020676	T	C
NC_040253.1	1063452	C	T
NC_040253.1	1099129	C	T
NC_040253.1	1146159	G	C
NC_040253.1	1202355	T	C
NC_040253.1	1252205	G	A
NC_040253.1	1303907	G	A
NC_040253.1	1320559	A	G
NC_040253.1	1376076	G	A
NC_040253.1	1421995	A	G
NC_040253.1	1453336	T	C
NC_040253.1	1476807	A	G
NC_040253.1	1525549	A	G
NC_040253.1	1531833	C	T
NC_040253.1	1581234	G	A
NC_040253.1	1586133	G	A

NC_040253.1	1621617	C	T
NC_040253.1	1657560	A	G
NC_040253.1	1747145	A	G
NC_040253.1	1773611	T	C
NC_040253.1	1793303	T	C
NC_040253.1	1829153	T	C
NC_040253.1	1853476	T	C
NC_040253.1	1897203	T	G
NC_040253.1	1929341	A	G
NC_040253.1	1978002	A	G
NC_040253.1	1994824	C	T
NC_040253.1	1995264	C	G
NC_040253.1	2047072	G	A
NC_040253.1	2058066	T	C
NC_040253.1	2135774	C	G
NC_040253.1	2196081	T	C
NC_040253.1	2212040	A	G
NC_040253.1	2260215	A	C
NC_040253.1	2312762	G	C
NC_040253.1	2378647	A	G
NC_040253.1	2391668	A	G
NC_040253.1	2393398	A	C
NC_040253.1	2393717	A	G
NC_040253.1	2421538	T	G
NC_040253.1	2455112	A	G
NC_040253.1	2468002	A	G
NC_040253.1	2484150	C	A
NC_040253.1	2513305	T	C
NC_040253.1	2555126	G	T
NC_040253.1	2577637	T	C
NC_040253.1	2586472	A	G
NC_040253.1	2591974	A	G
NC_040253.1	2635636	C	T
NC_040253.1	2659020	G	T
NC_040253.1	2659250	A	G
NC_040253.1	2659481	T	C
NC_040253.1	2661494	C	A
NC_040253.1	2687183	G	A
NC_040253.1	2718540	T	C
NC_040253.1	2740210	A	G
NC_040253.1	2758294	T	G
NC_040253.1	2768074	C	T
NC_040253.1	2820777	A	C
NC_040253.1	2871191	G	T
NC_040253.1	2926358	C	A
NC_040253.1	2947262	T	C
NC_040253.1	2950004	G	A

NC_040253.1	2950240	T	A
NC_040253.1	2950444	G	A
NC_040253.1	2950677	T	C
NC_040253.1	2951517	A	G
NC_040253.1	2953682	G	A
NC_040253.1	3007488	T	C
NC_040253.1	3050592	C	T
NC_040253.1	3106448	T	C
NC_040253.1	3147788	G	T
NC_040253.1	3213505	C	T
NC_040253.1	3266790	T	C
NC_040253.1	3314607	A	C
NC_040253.1	3367140	T	C
NC_040253.1	3423679	T	C
NC_040253.1	3476054	G	A
NC_040253.1	3535090	T	C
NC_040253.1	3589427	A	G
NC_040253.1	3649886	A	G
NC_040253.1	3705746	C	T
NC_040253.1	3750422	A	G
NC_040253.1	3793664	C	T
NC_040253.1	3805637	A	G
NC_040253.1	3864728	A	G
NC_040253.1	3927304	A	C
NC_040253.1	3979819	T	C
NC_040253.1	4054398	G	A
NC_040253.1	4112414	T	C
NC_040253.1	4161824	T	C
NC_040253.1	4209517	C	A
NC_040253.1	4253833	G	A
NC_040253.1	4311689	G	A
NC_040253.1	4354889	C	T
NC_040253.1	4355594	T	C
NC_040253.1	4415436	C	T
NC_040253.1	4475025	G	T
NC_040253.1	4511738	G	A
NC_040253.1	4530167	G	A
NC_040253.1	4590692	T	C
NC_040253.1	4641462	A	T
NC_040253.1	4644794	C	A
NC_040253.1	4699969	C	T
NC_040253.1	4768015	C	T
NC_040253.1	4829410	A	C
NC_040253.1	4872784	T	G
NC_040253.1	4929307	C	T
NC_040253.1	4977939	G	A
NC_040253.1	5028326	C	T

NC_040253.1	5082999	A	G	
NC_040253.1	5131429	T	C	
NC_040253.1	5133303	T	A	
NC_040253.1	5226505	G	A	
NC_040253.1	5282955	C	T	
NC_040253.1	5318993	G	T	
NC_040253.1	5339130	T	C	
NC_040253.1	5374939	G	A	
NC_040253.1	5391174	A	G	
NC_040253.1	5450035	T	A	
NC_040253.1	5501972	C	T	
NC_040253.1	5518372	G	T	
NC_040253.1	5578997	A	G	
NC_040253.1	5619292	C	A	
NC_040253.1	5655118	T	C	
NC_040253.1	5705880	A	G	
NC_040253.1	5744897	C	T	
NC_040253.1	5790504	G	A	
NC_040253.1	5802716	G	A	
NC_040253.1	5843033	T	C	
NC_040253.1	5918404	G	A	
NC_040253.1	5981310	G	A	
NC_040253.1	6017723	0	T	
NC_040253.1	6045353	T	C	
NC_040253.1	6068501	T	G	
NC_040253.1	6074140	A	G	
NC_040253.1	6127675	A	G	
NC_040253.1	6181529	T	C	
NC_040253.1	6230213	C	T	
NC_040253.1	6230651	G	A	
NC_040253.1	6281901	T	C	
NC_040253.1	6324769	A	G	
NC_040253.1	6333026	A	G	
NC_040253.1	6379613	C	G	
NC_040253.1	6417100	0		0
NC_040253.1	6473449	T	C	
NC_040253.1	6523731	C	T	
NC_040253.1	6590814	A	G	
NC_040253.1	6648795	T	C	
NC_040253.1	6696999	A	G	
NC_040253.1	6778606	G	C	
NC_040253.1	6799374	C	T	
NC_040253.1	6957548	C	T	
NC_040253.1	7015548	G	C	
NC_040253.1	7046544	G	A	
NC_040253.1	7076821	G	A	
NC_040253.1	7124761	T	G	

NC_040253.1	7176904	G	A
NC_040253.1	7229352	A	G
NC_040253.1	7248271	G	A
NC_040253.1	7290477	A	G
NC_040253.1	7326400	A	G
NC_040253.1	7331862	T	G
NC_040253.1	7368977	A	G
NC_040253.1	7422197	G	A
NC_040253.1	7478024	T	C
NC_040253.1	7534565	T	C
NC_040253.1	7569725	G	A
NC_040253.1	7592447	A	G
NC_040253.1	7594744	G	T
NC_040253.1	7645302	C	T
NC_040253.1	7698351	C	T
NC_040253.1	7752914	T	C
NC_040253.1	7806636	C	T
NC_040253.1	7851221	C	G
NC_040253.1	7908938	T	C
NC_040253.1	7969341	C	A
NC_040253.1	8020975	C	T
NC_040253.1	8084548	C	T
NC_040253.1	8142574	T	C
NC_040253.1	8195407	C	A
NC_040253.1	8255912	A	T
NC_040253.1	8315503	A	G
NC_040253.1	8369457	T	C
NC_040253.1	8425299	T	C
NC_040253.1	8462693	G	A
NC_040253.1	8497756	T	C
NC_040253.1	8518015	G	A
NC_040253.1	8591871	G	A
NC_040253.1	8622873	G	A
NC_040253.1	8688224	A	G
NC_040253.1	8726364	G	T
NC_040253.1	8740820	T	A
NC_040253.1	8849878	C	T
NC_040253.1	8901856	G	T
NC_040253.1	8931561	T	C
NC_040253.1	8948954	C	G
NC_040253.1	8997670	C	A
NC_040253.1	9043936	T	C
NC_040253.1	9054968	T	C
NC_040253.1	9088043	C	T
NC_040253.1	9142044	A	T
NC_040253.1	9190590	G	A
NC_040253.1	9214901	A	G

NC_040253.1	9269793	C	A
NC_040253.1	9323767	G	C
NC_040253.1	9337633	C	T
NC_040253.1	9394504	T	C
NC_040253.1	9433241	C	T
NC_040253.1	9464850	T	G
NC_040253.1	9478297	G	C
NC_040253.1	9506680	A	G
NC_040253.1	9522208	A	G
NC_040253.1	9588867	T	C
NC_040253.1	9625146	G	C
NC_040253.1	9657598	C	T
NC_040253.1	9698397	T	C
NC_040253.1	9702225	A	G
NC_040253.1	9729541	A	G
NC_040253.1	9762575	G	A
NC_040253.1	9816897	G	C
NC_040253.1	9871868	G	A
NC_040253.1	9897057	A	G
NC_040253.1	9906888	A	G
NC_040253.1	9961638	A	G
NC_040253.1	10006817	T	C
NC_040253.1	10010194	T	C
NC_040253.1	10060665	C	A
NC_040253.1	10104470	T	C
NC_040253.1	10162522	A	G
NC_040253.1	10210756	A	T
NC_040253.1	10236462	A	G
NC_040253.1	10260850	C	T
NC_040253.1	10317639	T	C
NC_040253.1	10370503	T	C
NC_040253.1	10422155	G	A
NC_040253.1	10473433	T	C
NC_040253.1	10485160	G	A
NC_040253.1	10506757	A	G
NC_040253.1	10525483	A	G
NC_040253.1	10568393	T	C
NC_040253.1	10572898	G	A
NC_040253.1	10603163	T	A
NC_040253.1	10634790	G	A
NC_040253.1	10637009	G	T
NC_040253.1	10654820	T	C
NC_040253.1	10655617	A	G
NC_040253.1	10668610	C	T
NC_040253.1	10696830	A	C
NC_040253.1	10708402	A	G
NC_040253.1	10723039	A	G

NC_040253.1	10755950	A	G
NC_040253.1	10800572	T	C
NC_040253.1	10802121	T	C
NC_040253.1	10858549	A	G
NC_040253.1	10895805	T	C
NC_040253.1	10905510	A	G
NC_040253.1	10989536	T	G
NC_040253.1	11063292	G	C
NC_040253.1	11116977	T	A
NC_040253.1	11154678	T	C
NC_040253.1	11156439	T	C
NC_040253.1	11173516	T	C
NC_040253.1	11179527	A	G
NC_040253.1	11230796	A	C
NC_040253.1	11278495	T	G
NC_040253.1	11335452	G	A
NC_040253.1	11389229	T	C
NC_040253.1	11404698	A	G
NC_040253.1	11455961	C	A
NC_040253.1	11506562	T	A
NC_040253.1	11511502	T	C
NC_040253.1	11533538	T	C
NC_040253.1	11599044	T	A
NC_040253.1	11634156	T	C
NC_040253.1	11644647	G	A
NC_040253.1	11645935	G	A
NC_040253.1	11690156	A	G
NC_040253.1	11738916	A	G
NC_040253.1	11794797	C	T
NC_040253.1	11809932	A	G
NC_040253.1	11866580	T	C
NC_040253.1	11923295	T	C
NC_040253.1	11976139	G	A
NC_040253.1	11986316	G	T
NC_040253.1	12048419	A	T
NC_040253.1	12103238	A	G
NC_040253.1	12138898	A	G
NC_040253.1	12174699	G	A
NC_040253.1	12176501	T	C
NC_040253.1	12177659	T	C
NC_040253.1	12180399	T	C
NC_040253.1	12198963	C	T
NC_040253.1	12214757	T	C
NC_040253.1	12258168	A	G
NC_040253.1	12293292	G	A
NC_040253.1	12335641	T	C
NC_040253.1	12346944	0	C

NC_040253.1	12392658	G	A
NC_040253.1	12423699	A	G
NC_040253.1	12441331	G	A
NC_040253.1	12456950	A	C
NC_040253.1	12518295	G	C
NC_040253.1	12585643	A	G
NC_040253.1	12692129	T	C
NC_040253.1	12723857	T	C
NC_040253.1	12778771	T	C
NC_040253.1	12827959	A	G
NC_040253.1	12872212	T	C
NC_040253.1	12884531	C	G
NC_040253.1	12936582	C	T
NC_040253.1	12938536	C	T
NC_040253.1	13060564	T	A
NC_040253.1	13108125	T	G
NC_040253.1	13160033	T	C
NC_040253.1	13213269	C	T
NC_040253.1	13241907	T	C
NC_040253.1	13279708	G	A
NC_040253.1	13337365	G	A
NC_040253.1	13350212	T	C
NC_040253.1	13405422	T	C
NC_040253.1	13436675	C	T
NC_040253.1	13487727	T	C
NC_040253.1	13539073	C	T
NC_040253.1	13567540	G	A
NC_040253.1	13583196	A	G
NC_040253.1	13613399	G	A
NC_040253.1	13618845	A	G
NC_040253.1	13619388	G	A
NC_040253.1	13621550	T	C
NC_040253.1	13651742	A	G
NC_040253.1	13662220	C	A
NC_040253.1	13704688	A	G
NC_040253.1	13759420	A	G
NC_040253.1	13809752	T	C
NC_040253.1	13833025	A	G
NC_040253.1	13866658	T	C
NC_040253.1	13915007	C	T
NC_040253.1	13952748	A	C
NC_040253.1	14009806	A	G
NC_040253.1	14041961	A	G
NC_040253.1	14091045	G	T
NC_040253.1	14129203	C	T
NC_040253.1	14144680	T	C
NC_040253.1	14207535	A	G

NC_040253.1	14250111	T	C
NC_040253.1	14298718	A	G
NC_040253.1	14352020	A	G
NC_040253.1	14396495	C	G
NC_040253.1	14461994	T	C
NC_040253.1	14513315	T	C
NC_040253.1	14568843	G	A
NC_040253.1	14602814	G	A
NC_040253.1	14603298	A	G
NC_040253.1	14630385	T	C
NC_040253.1	14635418	A	C
NC_040253.1	14637234	A	G
NC_040253.1	14638505	C	G
NC_040253.1	14690906	A	T
NC_040253.1	14723135	A	G
NC_040253.1	14724075	G	A
NC_040253.1	14725104	G	A
NC_040253.1	14776140	G	A
NC_040253.1	14826621	A	G
NC_040253.1	14847890	G	C
NC_040253.1	14855218	A	G
NC_040253.1	14855788	A	G
NC_040253.1	14858835	A	G
NC_040253.1	14914228	T	C
NC_040253.1	14959786	C	T
NC_040253.1	14993491	G	A
NC_040253.1	15011913	G	C
NC_040253.1	15068051	G	A
NC_040253.1	15125132	A	G
NC_040253.1	15175155	C	A
NC_040253.1	15231494	G	A
NC_040253.1	15286391	T	C
NC_040253.1	15344522	C	T
NC_040253.1	15404072	C	T
NC_040253.1	15473795	A	G
NC_040253.1	15520706	A	G
NC_040253.1	15558672	A	G
NC_040253.1	15659416	T	A
NC_040253.1	15712841	A	T
NC_040253.1	15764312	A	G
NC_040253.1	15816261	C	T
NC_040253.1	15876542	G	A
NC_040253.1	15931626	T	C
NC_040253.1	15980124	A	G
NC_040253.1	16007603	C	T
NC_040253.1	16041370	T	C
NC_040253.1	16097922	G	A

NC_040253.1	16155223	G	A
NC_040253.1	16196565	A	G
NC_040253.1	16250519	T	C
NC_040253.1	16301718	C	T
NC_040253.1	16349178	C	T
NC_040253.1	16357412	C	T
NC_040253.1	16413949	G	A
NC_040253.1	16462325	G	C
NC_040253.1	16519493	T	C
NC_040253.1	16556533	T	C
NC_040253.1	16576566	C	A
NC_040253.1	16617563	G	A
NC_040253.1	16664760	G	T
NC_040253.1	16699619	T	C
NC_040253.1	16703330	A	G
NC_040253.1	16758473	A	T
NC_040253.1	16766992	A	G
NC_040253.1	16824437	G	A
NC_040253.1	16873840	T	A
NC_040253.1	16940446	A	G
NC_040253.1	16986164	A	G
NC_040253.1	16994501	G	A
NC_040253.1	17050796	A	G
NC_040253.1	17103934	A	G
NC_040253.1	17138726	T	C
NC_040253.1	17160502	T	A
NC_040253.1	17201006	C	G
NC_040253.1	17272947	C	T
NC_040253.1	17300727	A	C
NC_040253.1	17313622	A	G
NC_040253.1	17342193	G	A
NC_040253.1	17381503	A	G
NC_040253.1	17442287	G	A
NC_040253.1	17494132	G	C
NC_040253.1	17510880	C	G
NC_040253.1	17598133	A	T
NC_040253.1	17641730	C	T
NC_040253.1	17668185	T	C
NC_040253.1	17675174	C	T
NC_040253.1	17871282	A	G
NC_040253.1	17892841	A	G
NC_040253.1	17975561	T	C
NC_040253.1	18018609	A	G
NC_040253.1	18081600	T	C
NC_040253.1	18116383	T	A
NC_040253.1	18150208	G	A
NC_040253.1	18181963	T	C

NC_040253.1	18183670	A	G
NC_040253.1	18233538	G	A
NC_040253.1	18248967	T	C
NC_040253.1	18310432	C	T
NC_040253.1	18313512	C	T
NC_040253.1	18339857	A	G
NC_040253.1	18389650	C	T
NC_040253.1	18433362	C	T
NC_040253.1	18491092	T	C
NC_040253.1	18550799	A	T
NC_040253.1	18609181	T	C
NC_040253.1	18668817	A	G
NC_040253.1	18719565	T	C
NC_040253.1	18726020	C	T
NC_040253.1	18776229	T	C
NC_040253.1	18795502	G	A
NC_040253.1	18843322	G	T
NC_040253.1	18859357	A	G
NC_040253.1	18874578	T	C
NC_040253.1	18903666	T	C
NC_040253.1	18918085	G	A
NC_040253.1	18990960	G	A
NC_040253.1	19041498	T	C
NC_040253.1	19046629	T	C
NC_040253.1	19113099	C	T
NC_040253.1	19159385	T	C
NC_040253.1	19291066	A	G
NC_040253.1	19328997	T	C
NC_040253.1	19372811	T	C
NC_040253.1	19419601	T	A
NC_040253.1	19463674	A	G
NC_040253.1	19489681	A	G
NC_040253.1	19563573	C	T
NC_040253.1	19629969	T	A
NC_040253.1	19678600	T	C
NC_040253.1	19721872	C	T
NC_040253.1	19728901	G	C
NC_040253.1	19761806	C	T
NC_040253.1	19790747	C	T
NC_040253.1	19941225	G	A
NC_040253.1	20057943	C	A
NC_040253.1	20141205	A	G
NC_040253.1	20187312	T	C
NC_040253.1	20242818	A	G
NC_040253.1	20260063	T	C
NC_040253.1	20333496	T	C
NC_040253.1	20366028	G	T

NC_040253.1	20614533	A	G
NC_040253.1	20675848	A	T
NC_040253.1	20734922	G	T
NC_040253.1	20775323	G	C
NC_040253.1	20836020	A	G
NC_040253.1	20872465	G	A
NC_040253.1	20910198	A	C
NC_040253.1	20959458	A	C
NC_040253.1	21020876	T	C
NC_040253.1	21147681	G	A
NC_040253.1	21211967	G	A
NC_040253.1	21315099	G	A
NC_040253.1	21337638	A	G
NC_040253.1	21779363	A	G
NC_040253.1	21806614	G	T
NC_040253.1	21879109	T	C
NC_040253.1	21919459	A	G
NC_040253.1	21934094	T	C
NC_040253.1	21991733	A	C
NC_040253.1	21996594	T	G
NC_040253.1	22087661	C	A
NC_040253.1	22116772	C	T
NC_040253.1	22162960	G	T
NC_040253.1	22174424	T	C
NC_040253.1	22284741	A	G
NC_040253.1	22297312	G	C
NC_040253.1	22301626	C	T
NC_040253.1	22375719	A	C
NC_040253.1	22527209	T	C
NC_040253.1	22567116	A	G
NC_040253.1	22590095	T	G
NC_040253.1	22620925	A	C
NC_040253.1	22682014	G	A
NC_040253.1	22727569	C	T
NC_040253.1	22736244	C	T
NC_040253.1	22783334	A	G
NC_040253.1	22805244	A	T
NC_040253.1	22856635	T	G
NC_040253.1	22890477	T	C
NC_040253.1	22946308	A	G
NC_040253.1	22988748	A	G
NC_040253.1	23108560	G	A
NC_040253.1	23314905	G	T
NC_040253.1	23373782	G	A
NC_040253.1	23412830	G	A
NC_040253.1	23451762	C	G
NC_040253.1	23567692	G	A

NC_040253.1	23627352	G	A
NC_040253.1	23723713	C	T
NC_040253.1	23875786	A	G
NC_040253.1	23888431	G	A
NC_040253.1	23937176	G	T
NC_040253.1	23986040	A	G
NC_040253.1	24005695	T	C
NC_040253.1	24042671	C	T
NC_040253.1	24072248	A	G
NC_040253.1	24126984	A	C
NC_040253.1	24170444	A	C
NC_040253.1	24176416	C	T
NC_040253.1	24205708	G	A
NC_040253.1	24251056	C	A
NC_040253.1	24306245	T	C
NC_040253.1	24344655	T	C
NC_040253.1	24402239	C	T
NC_040253.1	24455167	A	C
NC_040253.1	24507830	C	T
NC_040253.1	24514087	T	C
NC_040253.1	24568381	T	G
NC_040253.1	24592377	T	C
NC_040253.1	24638092	T	C
NC_040253.1	24711039	A	G
NC_040253.1	24759605	C	T
NC_040253.1	24769502	C	T
NC_040253.1	24867434	T	C
NC_040253.1	24896254	T	C
NC_040253.1	24934778	T	C
NC_040253.1	25074323	A	G
NC_040253.1	25083160	T	C
NC_040253.1	25104382	T	G
NC_040253.1	25157489	G	A
NC_040253.1	25178369	A	C
NC_040253.1	25225015	T	C
NC_040253.1	25282319	A	G
NC_040253.1	25326463	G	T
NC_040253.1	25346475	A	G
NC_040253.1	25407271	T	C
NC_040253.1	25438372	A	C
NC_040253.1	25632311	T	C
NC_040253.1	25639259	T	C
NC_040253.1	25674183	C	T
NC_040253.1	25715938	C	T
NC_040253.1	25750745	T	C
NC_040253.1	25807281	C	T
NC_040253.1	25857988	T	C

NC_040253.1	25868073	T	C
NC_040253.1	25916237	C	A
NC_040253.1	25963062	T	C
NC_040253.1	26022073	C	T
NC_040253.1	26057138	G	A
NC_040253.1	26083316	C	G
NC_040253.1	26137973	C	G
NC_040253.1	26216559	A	G
NC_040253.1	26276689	G	A
NC_040253.1	26321351	T	C
NC_040253.1	26337235	T	C
NC_040253.1	26389483	G	A
NC_040253.1	26451227	T	A
NC_040253.1	26516381	C	G
NC_040253.1	26520468	G	A
NC_040253.1	26672166	C	T
NC_040253.1	26750857	C	T
NC_040253.1	26883621	A	G
NC_040253.1	26965306	T	C
NC_040253.1	27075844	A	G
NC_040253.1	27106640	T	C
NC_040253.1	27160744	A	G
NC_040253.1	27204956	G	A
NC_040253.1	27279024	A	T
NC_040253.1	27313039	A	G
NC_040253.1	27351975	C	T
NC_040253.1	27451251	G	A
NC_040253.1	27499176	G	A
NC_040253.1	27526713	T	C
NC_040253.1	27554367	T	C
NC_040253.1	27609922	T	C
NC_040253.1	27658058	G	T
NC_040253.1	27667058	A	C
NC_040253.1	27717803	T	C
NC_040253.1	27733622	T	C
NC_040253.1	27787596	A	T
NC_040253.1	27840105	C	T
NC_040253.1	27853689	G	A
NC_040253.1	27919875	A	C
NC_040253.1	27973329	A	G
NC_040253.1	27983287	T	A
NC_040253.1	28018076	A	T
NC_040253.1	28029217	T	C
NC_040253.1	28064126	T	C
NC_040253.1	28098839	G	T
NC_040253.1	28152205	C	T
NC_040253.1	28197414	A	G

NC_040253.1	28235462	A	G
NC_040253.1	28268857	A	T
NC_040253.1	28277793	C	T
NC_040253.1	28327450	A	G
NC_040253.1	28382652	A	G
NC_040253.1	28430654	T	G
NC_040253.1	28471670	C	G
NC_040253.1	28521041	G	T
NC_040253.1	28523252	C	G
NC_040253.1	28551707	C	T
NC_040253.1	28579660	A	G
NC_040253.1	28636051	G	C
NC_040253.1	28668939	C	T
NC_040253.1	28713069	C	G
NC_040253.1	28733742	C	T
NC_040253.1	28785664	C	A
NC_040253.1	28828597	G	A
NC_040253.1	28857575	C	T
NC_040253.1	28870147	C	T
NC_040253.1	28925583	T	C
NC_040253.1	28969431	G	C
NC_040253.1	28974594	C	T
NC_040253.1	29036325	C	T
NC_040253.1	29088241	T	G
NC_040253.1	29127824	G	C
NC_040253.1	29156687	G	A
NC_040253.1	29203753	C	A
NC_040253.1	29266875	G	A
NC_040253.1	29326892	T	C
NC_040253.1	29453817	G	A
NC_040253.1	29474672	C	T
NC_040253.1	29526845	C	T
NC_040253.1	29562578	T	C
NC_040253.1	29584395	A	G
NC_040253.1	29637742	A	C
NC_040253.1	29690949	G	T
NC_040253.1	29721879	T	C
NC_040253.1	29770844	T	G
NC_040253.1	29802224	A	G
NC_040253.1	29876055	C	T
NC_040253.1	29933497	C	T
NC_040253.1	29977346	A	G
NC_040253.1	29993855	T	C
NC_040253.1	30033028	T	C
NC_040253.1	30052171	T	C
NC_040253.1	30072263	0	T
NC_040253.1	30128007	C	A

NC_040253.1	30167390	A	G
NC_040253.1	30191512	C	T
NC_040253.1	30240367	T	C
NC_040253.1	30283109	A	G
NC_040253.1	30309661	G	A
NC_040253.1	30311239	A	G
NC_040253.1	30313824	A	G
NC_040253.1	30362751	G	A
NC_040253.1	30382251	T	C
NC_040253.1	30389320	G	A
NC_040253.1	30427521	G	A
NC_040253.1	30449414	T	C
NC_040253.1	30497970	T	C
NC_040253.1	30549862	A	G
NC_040253.1	30566684	G	A
NC_040253.1	30643157	G	A
NC_040253.1	30693998	T	C
NC_040253.1	30703175	C	T
NC_040253.1	30716265	A	C
NC_040253.1	30769905	C	T
NC_040253.1	30786615	T	C
NC_040253.1	30790391	T	C
NC_040253.1	30819099	T	C
NC_040253.1	30863466	C	T
NC_040253.1	30919372	C	T
NC_040253.1	30972666	A	G
NC_040253.1	31019810	G	C
NC_040253.1	31065687	T	G
NC_040253.1	31106039	C	T
NC_040253.1	31150300	G	T
NC_040253.1	31260869	G	A
NC_040253.1	31298023	A	G
NC_040253.1	31341823	T	C
NC_040253.1	31343366	T	C
NC_040253.1	31372573	C	T
NC_040253.1	31406578	T	C
NC_040253.1	31460871	T	G
NC_040253.1	31505867	A	G
NC_040253.1	31534362	C	T
NC_040253.1	31578270	A	C
NC_040253.1	31613958	T	C
NC_040253.1	31631883	T	C
NC_040253.1	31676025	A	G
NC_040253.1	31718117	T	C
NC_040253.1	31780709	A	G
NC_040253.1	31820158	T	C
NC_040253.1	31851915	T	C

NC_040253.1	31890243	C	T
NC_040253.1	31927028	A	G
NC_040253.1	31966073	A	G
NC_040253.1	32037267	T	C
NC_040253.1	32086612	C	T
NC_040253.1	32113037	C	T
NC_040253.1	32137986	T	C
NC_040253.1	32192970	T	C
NC_040253.1	32240017	A	G
NC_040253.1	32297921	G	A
NC_040253.1	32351804	T	C
NC_040253.1	32392271	G	T
NC_040253.1	32415629	T	C
NC_040253.1	32417733	A	C
NC_040253.1	32422008	A	G
NC_040253.1	32450764	T	C
NC_040253.1	32502539	T	A
NC_040253.1	32567188	T	C
NC_040253.1	32625947	T	C
NC_040253.1	32651517	C	T
NC_040253.1	32696023	C	T
NC_040253.1	32715435	C	T
NC_040253.1	32765924	T	C
NC_040253.1	32805835	C	T
NC_040253.1	32857131	C	T
NC_040253.1	32891405	T	C
NC_040253.1	32905536	C	T
NC_040253.1	32963704	T	C
NC_040253.1	33009408	C	T
NC_040253.1	33028446	G	A
NC_040253.1	33062729	T	C
NC_040253.1	33096292	A	G
NC_040253.1	33143273	C	G
NC_040253.1	33238859	A	G
NC_040253.1	33294804	T	C
NC_040253.1	33343769	T	C
NC_040253.1	33372368	C	T
NC_040253.1	33395546	C	T
NC_040253.1	33444781	C	T
NC_040253.1	33580410	C	T
NC_040253.1	33700766	T	G
NC_040253.1	33746096	A	G
NC_040253.1	33787474	C	T
NC_040253.1	33819800	T	C
NC_040253.1	33884550	A	G
NC_040253.1	33905657	G	C
NC_040253.1	33946611	C	A

NC_040253.1	34065979	A	G
NC_040253.1	34121457	A	G
NC_040253.1	34177611	T	C
NC_040253.1	34229291	C	A
NC_040253.1	34263286	A	T
NC_040253.1	34308826	T	G
NC_040253.1	34360030	C	T
NC_040253.1	34414596	T	A
NC_040253.1	34452847	C	T
NC_040253.1	34465081	G	A
NC_040253.1	34533700	A	G
NC_040253.1	34587940	G	T
NC_040253.1	34636017	T	C
NC_040253.1	34684603	T	C
NC_040253.1	34696718	C	A
NC_040253.1	34736173	C	T
NC_040253.1	34745596	T	C
NC_040253.1	34776154	C	A
NC_040253.1	34788464	A	G
NC_040253.1	34834033	T	C
NC_040253.1	34890779	A	G
NC_040253.1	34921064	T	C
NC_040253.1	35019742	C	G
NC_040253.1	35075564	C	T
NC_040253.1	35113136	A	G
NC_040253.1	35159335	T	C
NC_040253.1	35163865	A	G
NC_040253.1	35218401	G	T
NC_040253.1	35236545	C	T
NC_040253.1	35290082	T	G
NC_040253.1	35346274	T	C
NC_040253.1	35427315	C	T
NC_040253.1	35473612	T	C
NC_040253.1	35529780	A	G
NC_040253.1	35604925	C	T
NC_040253.1	35634647	C	T
NC_040253.1	35684045	G	A
NC_040253.1	35726124	G	A
NC_040253.1	35776661	C	T
NC_040253.1	35791560	T	C
NC_040253.1	35824498	A	G
NC_040253.1	35829822	T	C
NC_040253.1	35889582	C	T
NC_040253.1	35922188	G	A
NC_040253.1	35960736	G	A
NC_040253.1	35990488	T	C
NC_040253.1	36046726	C	T

NC_040253.1	36086944	C	A
NC_040253.1	36146371	T	A
NC_040253.1	36182288	G	A
NC_040253.1	36217739	G	A
NC_040253.1	36248233	G	A
NC_040253.1	36317733	G	A
NC_040253.1	36356891	T	A
NC_040253.1	36407092	A	T
NC_040253.1	36463262	A	G
NC_040253.1	36520612	C	T
NC_040253.1	36575465	C	A
NC_040253.1	36609446	A	T
NC_040253.1	36876922	G	T
NC_040253.1	36912203	C	T
NC_040253.1	36940704	G	C
NC_040253.1	37006830	A	G
NC_040253.1	37059492	A	T
NC_040253.1	37127992	A	C
NC_040253.1	37153718	T	C
NC_040253.1	37193201	G	A
NC_040253.1	37241809	G	C
NC_040253.1	37297180	G	A
NC_040253.1	37334080	C	G
NC_040253.1	37344210	C	T
NC_040253.1	37364427	G	A
NC_040253.1	37421496	T	G
NC_040253.1	37447744	T	C
NC_040253.1	37497475	C	A
NC_040253.1	37597327	T	C
NC_040253.1	37623083	A	G
NC_040253.1	37630714	A	G
NC_040253.1	37677716	A	G
NC_040253.1	37732202	T	G
NC_040253.1	37743364	G	C
NC_040253.1	37801619	A	G
NC_040253.1	37851185	A	T
NC_040253.1	37895675	A	G
NC_040253.1	37932810	C	T
NC_040253.1	37996922	A	G
NC_040253.1	38046030	C	A
NC_040253.1	38062399	A	G
NC_040253.1	38114319	G	A
NC_040253.1	38149020	C	T
NC_040253.1	38194474	T	C
NC_040253.1	38226103	T	C
NC_040253.1	38272349	C	T
NC_040253.1	38331379	C	T

NC_040253.1	38364052	T	C
NC_040253.1	38405034	T	C
NC_040253.1	38455080	C	G
NC_040253.1	38547841	C	T
NC_040253.1	38774775	T	C
NC_040253.1	38796514	A	G
NC_040253.1	38854926	T	G
NC_040253.1	38874196	C	T
NC_040253.1	38911322	A	T
NC_040253.1	38949662	A	G
NC_040253.1	38967570	A	G
NC_040253.1	39015146	G	A
NC_040253.1	39060041	T	C
NC_040253.1	39094798	A	G
NC_040253.1	39139760	A	C
NC_040253.1	39167837	C	T
NC_040253.1	39175098	T	C
NC_040253.1	39228064	T	G
NC_040253.1	39251895	C	T
NC_040253.1	39263907	A	G
NC_040253.1	39293862	A	G
NC_040253.1	39324632	G	A
NC_040253.1	39430182	T	C
NC_040253.1	39452041	A	G
NC_040253.1	39480323	C	T
NC_040253.1	39514038	C	A
NC_040253.1	39568869	C	A
NC_040253.1	39609341	T	C
NC_040253.1	39638477	T	C
NC_040253.1	39692895	T	C
NC_040253.1	39736397	T	A
NC_040253.1	39752767	C	T
NC_040253.1	39783981	A	G
NC_040253.1	39803465	C	G
NC_040253.1	39850557	T	C
NC_040253.1	39888784	C	T
NC_040253.1	39899999	T	C
NC_040253.1	39921392	A	G
NC_040253.1	39952199	T	C
NC_040253.1	39975177	G	T
NC_040253.1	40007543	T	C
NC_040253.1	40025108	C	T
NC_040253.1	40071259	C	T
NC_040253.1	40138183	G	C
NC_040253.1	40165554	A	G
NC_040253.1	40181306	T	C
NC_040253.1	40218731	T	C

NC_040253.1	40265414	A	G
NC_040253.1	40365752	C	T
NC_040253.1	40379887	T	C
NC_040253.1	40437777	G	A
NC_040253.1	40454610	C	T
NC_040253.1	40503405	A	G
NC_040253.1	40518002	G	A
NC_040253.1	40588187	T	A
NC_040253.1	40646131	T	C
NC_040253.1	40697158	T	A
NC_040253.1	40757104	A	C
NC_040253.1	40837180	G	C
NC_040253.1	40868297	T	G
NC_040253.1	40893906	G	A
NC_040253.1	40982865	G	A
NC_040253.1	41041197	T	G
NC_040253.1	41053759	C	T
NC_040253.1	41169689	A	G
NC_040253.1	41217819	C	T
NC_040253.1	41254468	G	A
NC_040253.1	41265286	T	C
NC_040253.1	41308803	G	T
NC_040253.1	41327108	A	T
NC_040253.1	41368242	G	A
NC_040253.1	41411949	T	C
NC_040253.1	41451419	C	T
NC_040253.1	41497971	A	C
NC_040253.1	41509115	A	G
NC_040253.1	41565925	G	A
NC_040253.1	41581824	T	G
NC_040253.1	41650479	C	T
NC_040253.1	41660843	A	C
NC_040253.1	41700432	T	C
NC_040253.1	41740502	T	G
NC_040253.1	41797918	C	G
NC_040253.1	41847183	T	C
NC_040253.1	41863314	T	C
NC_040253.1	41877416	T	C
NC_040253.1	41913677	A	G
NC_040253.1	41930750	T	C
NC_040253.1	41988583	C	T
NC_040253.1	42039923	A	G
NC_040253.1	42092139	G	A
NC_040253.1	42147548	A	G
NC_040253.1	42225513	C	T
NC_040253.1	42246053	T	C
NC_040253.1	42290253	C	A

NC_040253.1	42405623	A	G
NC_040253.1	42479289	G	A
NC_040253.1	42516425	A	G
NC_040253.1	42564952	C	T
NC_040253.1	42593386	A	G
NC_040253.1	42674460	G	C
NC_040253.1	42704691	G	T
NC_040253.1	42795373	C	T
NC_040253.1	42854676	C	T
NC_040253.1	42871288	C	G
NC_040253.1	42913145	G	C
NC_040253.1	42973447	T	G
NC_040253.1	43023861	T	C
NC_040253.1	43070224	A	G
NC_040253.1	43105555	A	G
NC_040253.1	43282798	T	C
NC_040253.1	43396816	T	G
NC_040253.1	43449876	G	C
NC_040253.1	43466557	0	G
NC_040253.1	43618558	C	T
NC_040253.1	43638765	T	C
NC_040253.1	43697195	C	T
NC_040253.1	43735834	G	A
NC_040253.1	43770389	A	T
NC_040253.1	43799536	A	G
NC_040253.1	43845894	T	G
NC_040253.1	43910498	G	T
NC_040253.1	43943949	C	G
NC_040253.1	43963235	T	G
NC_040253.1	44004334	G	A
NC_040253.1	44036164	G	A
NC_040253.1	44097239	C	T
NC_040253.1	44136944	C	T
NC_040253.1	44183063	G	A
NC_040253.1	44220534	A	G
NC_040253.1	44257186	A	G
NC_040253.1	44295926	G	A
NC_040253.1	44324567	G	A
NC_040253.1	44384972	G	T
NC_040253.1	44427020	T	C
NC_040253.1	44458238	T	C
NC_040253.1	44594684	A	G
NC_040253.1	44629230	G	A
NC_040253.1	44755518	T	C
NC_040253.1	44863419	G	A
NC_040253.1	44891086	A	G
NC_040253.1	44924886	G	C

NC_040253.1	44961078	T	A
NC_040253.1	45007079	A	G
NC_040253.1	45051961	G	C
NC_040253.1	45095495	T	C
NC_040253.1	45114479	A	G
NC_040253.1	45175162	G	T
NC_040253.1	45215416	T	C
NC_040253.1	45235598	C	T
NC_040253.1	45289195	T	C
NC_040253.1	45337091	T	G
NC_040253.1	45366370	T	C
NC_040253.1	45397221	T	C
NC_040253.1	45408320	A	G
NC_040253.1	45436284	A	G
NC_040253.1	45469754	T	C
NC_040253.1	45498712	T	C
NC_040253.1	45546488	G	A
NC_040253.1	45554310	A	G
NC_040253.1	45605984	T	C
NC_040253.1	45634267	A	G
NC_040253.1	45674197	G	A
NC_040253.1	45679529	A	G
NC_040253.1	45699194	T	C
NC_040253.1	45723721	A	G
NC_040253.1	45780259	T	G
NC_040253.1	45812248	G	A
NC_040253.1	45834350	T	G
NC_040253.1	45846603	A	G
NC_040253.1	45875984	T	C
NC_040253.1	45915916	A	G
NC_040253.1	45947518	T	C
NC_040253.1	45983588	T	C
NC_040253.1	46031409	G	T
NC_040253.1	46054281	G	A
NC_040253.1	46108480	T	C
NC_040253.1	46128696	A	G
NC_040253.1	46191155	G	T
NC_040253.1	46224046	A	G
NC_040253.1	46265523	T	A
NC_040253.1	46341198	T	C
NC_040253.1	46426181	T	G
NC_040253.1	46433878	T	C
NC_040253.1	46514194	T	C
NC_040253.1	46730285	T	C
NC_040253.1	46792856	A	G
NC_040253.1	46845857	G	A
NC_040253.1	46867439	T	C

NC_040253.1	46968289	T	C
NC_040253.1	47033133	G	A
NC_040253.1	47058843	G	T
NC_040253.1	47093860	A	G
NC_040253.1	47168676	T	C
NC_040253.1	47253752	T	C
NC_040253.1	47337357	T	C
NC_040253.1	47482800	A	G
NC_040253.1	47551769	G	A
NC_040253.1	47600303	G	T
NC_040253.1	47644129	A	G
NC_040253.1	47692154	C	T
NC_040253.1	47711133	C	G
NC_040253.1	47822583	T	A
NC_040253.1	47913969	C	G
NC_040253.1	47960196	G	A
NC_040253.1	47977164	T	C
NC_040253.1	48022045	C	T
NC_040253.1	48034325	A	G
NC_040253.1	48191096	T	C
NC_040253.1	48221290	A	C
NC_040253.1	48326740	G	A
NC_040253.1	48343743	C	T
NC_040253.1	48376486	G	A
NC_040253.1	48492939	C	T
NC_040253.1	48561114	G	A
NC_040253.1	48587946	G	A
NC_040253.1	48769740	A	T
NC_040253.1	48864998	G	A
NC_040253.1	49011170	G	C
NC_040253.1	49072104	C	T
NC_040253.1	49216834	G	A
NC_040253.1	49318515	C	T
NC_040253.1	49383201	A	T
NC_040253.1	49604548	G	T
NC_040253.1	49777565	C	T
NC_040253.1	50244157	A	G
NC_040253.1	50265761	G	A
NC_040253.1	50303043	C	T
NC_040253.1	50345058	A	G
NC_040253.1	50385234	A	G
NC_040253.1	50432920	G	C
NC_040253.1	50479469	A	T
NC_040253.1	50501470	A	G
NC_040253.1	50528999	A	T
NC_040253.1	50555267	A	G
NC_040253.1	50619530	A	G

NC_040253.1	50674221	A	G
NC_040253.1	50687058	T	C
NC_040253.1	50730022	C	A
NC_040253.1	50793105	C	A
NC_040253.1	50851748	G	A
NC_040253.1	50910142	A	C
NC_040253.1	50937257	T	C
NC_040253.1	50960716	G	A
NC_040253.1	51001514	A	G
NC_040253.1	51036815	G	A
NC_040253.1	51058629	G	T
NC_040253.1	51091251	T	C
NC_040253.1	51127448	C	T
NC_040253.1	51157317	G	C
NC_040253.1	51205869	C	A
NC_040253.1	51271476	G	A
NC_040253.1	51328827	C	A
NC_040253.1	51353920	A	G
NC_040253.1	51383336	A	G
NC_040253.1	51448626	G	T
NC_040253.1	51490104	C	G
NC_040253.1	51545005	G	A
NC_040253.1	51590283	T	C
NC_040253.1	51659353	C	T
NC_040253.1	51714467	G	A
NC_040253.1	51730238	T	G
NC_040253.1	51764412	G	T
NC_040253.1	51802625	T	C
NC_040253.1	51851309	G	A
NC_040253.1	51893400	A	G
NC_040253.1	51946936	G	A
NC_040253.1	52002473	A	G
NC_040253.1	52017393	T	C
NC_040253.1	52083198	C	A
NC_040253.1	52134828	A	G
NC_040253.1	52156410	C	T
NC_040253.1	52201728	G	A
NC_040253.1	52238407	T	C
NC_040253.1	52274703	A	G
NC_040253.1	52300544	G	A
NC_040253.1	52343719	G	A
NC_040253.1	52371332	T	C
NC_040253.1	52431640	G	C
NC_040253.1	52544857	A	G
NC_040253.1	52584207	G	C
NC_040253.1	52610646	A	G
NC_040253.1	52658814	A	G

NC_040253.1	52711413	C	T
NC_040253.1	52721423	A	G
NC_040253.1	52767360	T	C
NC_040253.1	52809860	C	T
NC_040253.1	52854076	A	G
NC_040253.1	52878123	A	G
NC_040253.1	52962841	A	G
NC_040253.1	52988255	A	G
NC_040253.1	53013894	T	C
NC_040253.1	53052439	C	A
NC_040253.1	53082521	C	T
NC_040253.1	53121277	T	C
NC_040253.1	53189012	C	T
NC_040253.1	53225305	T	C
NC_040253.1	53236815	A	G
NC_040253.1	53300598	T	G
NC_040253.1	53339339	T	C
NC_040253.1	53462272	T	C
NC_040253.1	53520910	T	C
NC_040253.1	53572241	A	T
NC_040253.1	53605065	C	T
NC_040253.1	53619612	T	A
NC_040253.1	53650918	T	C
NC_040253.1	53671624	A	G
NC_040253.1	53717406	T	C
NC_040253.1	53743557	G	T
NC_040253.1	53854364	T	C
NC_040253.1	53910744	A	G
NC_040253.1	53964624	G	T
NC_040253.1	54022845	C	T
NC_040253.1	54056606	T	C
NC_040253.1	54086649	C	A
NC_040253.1	54098469	A	G
NC_040253.1	54125921	A	T
NC_040253.1	54181202	G	A
NC_040253.1	54225385	A	T
NC_040253.1	54299800	A	G
NC_040253.1	54319389	T	C
NC_040253.1	54364252	C	T
NC_040253.1	54409278	T	C
NC_040253.1	54440195	A	G
NC_040253.1	54478286	T	C
NC_040253.1	54530317	G	A
NC_040253.1	54587394	A	C
NC_040253.1	54641375	A	G
NC_040253.1	54672479	T	C
NC_040253.1	54775801	T	G

NC_040253.1	54859611	T	A
NC_040253.1	54977993	G	T
NC_040253.1	55061343	A	G
NC_040253.1	55072141	A	G
NC_040253.1	55100975	C	T
NC_040253.1	55117237	A	G
NC_040253.1	55175312	A	G
NC_040253.1	55233295	C	T
NC_040253.1	55288913	A	G
NC_040253.1	55309728	A	G
NC_040253.1	55415412	A	G
NC_040253.1	55458006	G	A
NC_040253.1	55471610	A	G
NC_040253.1	55512991	T	C
NC_040253.1	55573807	A	G
NC_040253.1	55584362	C	A
NC_040253.1	55634905	T	C
NC_040253.1	55689308	T	C
NC_040253.1	55737401	T	C
NC_040253.1	55775571	C	T
NC_040253.1	55827168	G	A
NC_040253.1	55886917	C	T
NC_040253.1	55939453	T	C
NC_040253.1	55985502	T	C
NC_040253.1	56064978	G	A
NC_040253.1	56086640	C	G
NC_040253.1	56130516	T	C
NC_040253.1	56194726	T	C
NC_040253.1	56651754	T	C
NC_040253.1	56703319	A	T
NC_040253.1	56770532	C	T
NC_040253.1	56797933	T	C
NC_040253.1	57185349	A	G
NC_040253.1	57253569	G	A
NC_040253.1	57563805	A	G
NC_040253.1	57668762	C	T
NC_040253.1	57714366	G	A
NC_040253.1	57800816	G	T
NC_040253.1	57997351	G	A
NC_040253.1	58020691	C	A
NC_040253.1	58103148	A	C
NC_040253.1	58177656	T	C
NC_040253.1	58250645	C	G
NC_040253.1	58271844	G	T
NC_040253.1	58329035	G	A
NC_040253.1	58390170	A	G
NC_040253.1	58450260	T	C

NC_040253.1	58464215	A	G
NC_040253.1	58485640	A	G
NC_040253.1	58538075	T	C
NC_040253.1	58573196	G	A
NC_040253.1	58598490	T	A
NC_040253.1	58625139	T	C
NC_040253.1	58671036	A	G
NC_040253.1	58936934	A	G
NC_040253.1	59007792	A	G
NC_040253.1	59102299	A	T
NC_040253.1	59508952	C	T
NC_040253.1	59568870	T	C
NC_040253.1	59634934	C	T
NC_040253.1	59961695	G	T
NC_040253.1	60102982	C	A
NC_040253.1	60134178	T	G
NC_040253.1	60436580	C	T
NC_040253.1	60522980	C	A
NC_040253.1	60567363	G	A
NC_040253.1	60598940	T	C
NC_040253.1	60649052	T	A
NC_040253.1	60669516	G	A
NC_040253.1	60703970	C	T
NC_040253.1	60755116	G	A
NC_040253.1	60786897	T	C
NC_040253.1	60804957	T	A
NC_040253.1	60835013	T	C
NC_040253.1	60878611	0	A
NC_040253.1	60896091	T	A
NC_040253.1	60921112	G	A
NC_040253.1	60961772	G	A
NC_040253.1	60987878	T	C
NC_040253.1	61031475	C	T
NC_040253.1	61053042	A	G
NC_040253.1	61113017	C	T
NC_040253.1	61144135	T	A
NC_040253.1	61169737	G	A
NC_040253.1	61245504	G	C
NC_040253.1	61433007	C	G
NC_040253.1	61735385	A	C
NC_040253.1	61784440	A	G
NC_040253.1	61855851	G	A
NC_040253.1	61940902	A	C
NC_040253.1	61984775	C	G
NC_040253.1	62011424	A	G
NC_040253.1	62039354	T	C
NC_040253.1	62108493	C	G

NC_040253.1	62271378	C	T
NC_040253.1	62295314	T	C
NC_040253.1	62451015	G	A
NC_040253.1	62499085	A	G
NC_040253.1	62541806	A	G
NC_040253.1	62741817	T	C
NC_040253.1	62866961	G	A
NC_040253.1	62916738	G	A
NC_040253.1	63004131	C	T
NC_040253.1	63060430	C	T
NC_040253.1	63098296	G	A
NC_040253.1	63367869	G	A
NC_040253.1	63427713	G	A
NC_040253.1	63555076	T	C
NC_040253.1	63591412	G	A
NC_040253.1	63714781	T	C
NC_040253.1	63738584	T	C
NC_040253.1	63820865	T	C
NC_040253.1	63878673	T	C
NC_040253.1	63907078	C	T
NC_040253.1	63945646	G	A
NC_040253.1	64044803	G	A
NC_040253.1	64079057	T	C
NC_040253.1	64097114	T	C
NC_040253.1	64276695	A	G
NC_040253.1	64321975	G	A
NC_040253.1	64360061	T	C
NC_040253.1	64411951	C	A
NC_040253.1	64425174	C	A
NC_040253.1	64509329	T	C
NC_040253.1	64548489	C	G
NC_040253.1	64632501	T	C
NC_040253.1	64925791	G	C
NC_040253.1	65167878	C	T
NC_040253.1	65213203	T	C
NC_040253.1	65267743	T	C
NC_040253.1	65330737	T	G
NC_040253.1	65379519	C	T
NC_040253.1	65448377	C	T
NC_040253.1	65535859	G	A
NC_040253.1	65617201	T	C
NC_040253.1	65882099	C	T
NC_040253.1	65943850	A	G
NC_040253.1	66028316	C	T
NC_040253.1	66092838	C	G
NC_040253.1	66135700	A	G
NC_040253.1	66152508	G	T

NC_040253.1	66214063	C	T
NC_040253.1	66314969	T	C
NC_040253.1	66349040	T	C
NC_040253.1	66379373	C	T
NC_040253.1	66423932	A	G
NC_040253.1	66458216	C	T
NC_040253.1	66488693	A	G
NC_040253.1	66555732	G	A
NC_040253.1	66771108	C	A
NC_040253.1	66812650	T	G
NC_040253.1	66840301	C	T
NC_040253.1	66897110	A	G
NC_040253.1	66965853	G	A
NC_040253.1	67131522	G	A
NC_040253.1	67194845	A	T
NC_040253.1	67404443	C	T
NC_040253.1	67424797	G	C
NC_040253.1	67706446	C	A
NC_040253.1	67780547	T	C
NC_040253.1	67842166	C	G
NC_040253.1	67894819	C	T
NC_040253.1	68002534	A	G
NC_040253.1	68049597	G	A
NC_040253.1	68066727	C	T
NC_040253.1	68125673	C	T
NC_040253.1	68247029	A	G
NC_040253.1	68306174	A	G
NC_040253.1	68365676	A	G
NC_040253.1	68408058	G	A
NC_040253.1	68437218	T	G
NC_040253.1	68460538	A	G
NC_040253.1	68491322	A	G
NC_040253.1	68748037	G	A
NC_040253.1	68794807	G	C
NC_040253.1	68851774	C	A
NC_040253.1	69054992	A	T
NC_040253.1	69204277	A	G
NC_040253.1	69231963	G	A
NC_040253.1	69257683	T	C
NC_040253.1	69287227	T	G
NC_040253.1	69306443	G	C
NC_040253.1	69345675	C	A
NC_040253.1	69361455	T	C
NC_040253.1	69416742	A	G
NC_040253.1	69450347	G	T
NC_040253.1	69507506	T	G
NC_040253.1	69549915	T	C

NC_040253.1	69579863	T	C
NC_040253.1	69588423	C	T
NC_040253.1	69795258	G	A
NC_040253.1	69901062	G	T
NC_040253.1	69939440	G	A
NC_040253.1	69980122	C	T
NC_040253.1	70141652	C	T
NC_040253.1	70187327	T	G
NC_040253.1	70213557	T	G
NC_040253.1	70312844	G	C
NC_040253.1	70521382	T	C
NC_040253.1	70643653	T	G
NC_040253.1	71089903	A	G
NC_040253.1	71131746	A	C
NC_040253.1	71166529	G	A
NC_040253.1	71194252	G	A
NC_040253.1	71288364	A	G
NC_040253.1	71433698	C	T
NC_040253.1	71601599	G	C
NC_040253.1	71694402	T	C
NC_040253.1	72013835	A	G
NC_040253.1	72084789	C	A
NC_040253.1	72106637	T	C
NC_040253.1	72190053	A	G
NC_040253.1	72254501	C	A
NC_040253.1	72284632	A	G
NC_040253.1	72320456	A	G
NC_040253.1	72449985	T	C
NC_040253.1	72586558	A	C
NC_040253.1	72801323	A	G
NC_040253.1	73066342	A	G
NC_040253.1	73089093	T	C
NC_040253.1	73140677	T	C
NC_040253.1	73199999	T	G
NC_040253.1	73235946	C	G
NC_040253.1	73360024	A	C
NC_040253.1	73452772	T	C
NC_040253.1	73483902	A	G
NC_040253.1	73541122	A	G
NC_040253.1	73573264	T	C
NC_040253.1	73610487	A	G
NC_040253.1	73663959	T	A
NC_040253.1	73708521	G	A
NC_040253.1	73805092	G	A
NC_040253.1	73831949	G	A
NC_040253.1	73869163	C	G
NC_040253.1	73946142	C	T

NC_040253.1	73985648	C	T
NC_040253.1	74126250	C	A
NC_040253.1	74140700	G	T
NC_040253.1	74206166	0	C
NC_040253.1	74249137	C	A
NC_040253.1	74323852	T	A
NC_040253.1	74364222	T	A
NC_040253.1	74411445	G	A
NC_040253.1	74444012	G	A
NC_040253.1	74478209	T	C
NC_040253.1	74573579	T	A
NC_040253.1	74631035	T	G
NC_040253.1	74698924	A	C
NC_040253.1	74748455	C	T
NC_040253.1	74775413	G	A
NC_040253.1	74808706	C	T
NC_040253.1	74854310	G	A
NC_040253.1	74868287	A	T
NC_040253.1	75132761	C	T
NC_040253.1	75204521	C	T
NC_040253.1	75238227	C	T
NC_040253.1	75253943	C	T
NC_040253.1	75309057	A	T
NC_040253.1	75355252	C	A
NC_040253.1	75382874	A	C
NC_040253.1	75433678	G	A
NC_040253.1	75460675	T	C
NC_040253.1	75479713	T	C
NC_040253.1	75539932	C	T
NC_040253.1	75619577	A	G
NC_040253.1	75637364	C	T
NC_040253.1	75700694	T	C
NC_040253.1	75714334	T	C
NC_040253.1	75998275	T	C
NC_040253.1	76194998	C	T
NC_040253.1	76222786	C	T
NC_040253.1	76241962	A	G
NC_040253.1	76257089	T	C
NC_040253.1	76266635	A	G
NC_040253.1	76302514	C	T
NC_040253.1	76364174	A	G
NC_040253.1	76418844	T	C
NC_040253.1	76483699	A	T
NC_040253.1	76493727	C	A
NC_040253.1	76620220	G	A
NC_040253.1	76661171	T	C
NC_040253.1	76685598	G	T

NC_040253.1	76722882	T	C
NC_040253.1	76777112	A	C
NC_040253.1	76813187	T	C
NC_040253.1	76866950	G	A
NC_040253.1	76896897	A	G
NC_040253.1	77061409	C	T
NC_040253.1	77117609	G	A
NC_040253.1	77142662	A	T
NC_040253.1	77195033	A	G
NC_040253.1	77237497	T	C
NC_040253.1	77303839	C	T
NC_040253.1	77350624	C	T
NC_040253.1	77378962	G	A
NC_040253.1	77412133	T	C
NC_040253.1	77440430	T	C
NC_040253.1	77464670	T	C
NC_040253.1	77553272	G	C
NC_040253.1	77600018	G	A
NC_040253.1	77636190	C	G
NC_040253.1	77669646	A	G
NC_040253.1	77698483	T	C
NC_040253.1	77788421	T	C
NC_040253.1	77796680	A	G
NC_040253.1	77827345	A	G
NC_040253.1	77853851	G	C
NC_040253.1	77879010	A	G
NC_040253.1	77893729	A	C
NC_040253.1	78012129	A	C
NC_040253.1	78106408	C	A
NC_040253.1	78161873	A	G
NC_040253.1	78279824	G	A
NC_040253.1	78309615	T	C
NC_040253.1	78331321	A	G
NC_040253.1	78372829	G	C
NC_040253.1	78434033	C	T
NC_040253.1	78450188	T	A
NC_040253.1	78476463	G	A
NC_040253.1	78495982	A	G
NC_040253.1	78532357	T	C
NC_040253.1	78572967	A	C
NC_040253.1	78628709	G	A
NC_040253.1	78664555	A	G
NC_040253.1	78697302	A	G
NC_040253.1	78722429	G	T
NC_040253.1	78761656	C	A
NC_040253.1	78798323	A	G
NC_040253.1	78877729	G	A

NC_040253.1	78925789	T	C
NC_040253.1	78951283	T	C
NC_040253.1	78967845	A	C
NC_040253.1	79036639	A	G
NC_040253.1	79057383	G	T
NC_040253.1	79173491	A	G
NC_040253.1	79180201	G	C
NC_040253.1	79249912	C	A
NC_040253.1	79303333	A	G
NC_040253.1	79316498	A	T
NC_040253.1	79387628	G	A
NC_040253.1	79441100	G	A
NC_040253.1	79448769	A	G
NC_040253.1	79506729	T	C
NC_040253.1	79589899	T	C
NC_040253.1	79642727	G	T
NC_040253.1	79706566	G	A
NC_040253.1	79713538	G	T
NC_040253.1	79818245	T	C
NC_040253.1	79845160	T	C
NC_040253.1	79867135	C	T
NC_040253.1	79885956	G	T
NC_040253.1	79935847	T	C
NC_040253.1	79986654	C	A
NC_040253.1	80051984	A	G
NC_040253.1	80118302	G	C
NC_040253.1	80160745	G	A
NC_040253.1	80218514	C	T
NC_040253.1	80261677	C	T
NC_040253.1	80312757	C	T
NC_040253.1	80358346	C	A
NC_040253.1	80364749	T	C
NC_040253.1	80443352	C	T
NC_040253.1	80471398	A	T
NC_040253.1	80513585	A	G
NC_040253.1	80539384	G	C
NC_040253.1	80567129	A	G
NC_040253.1	80625419	T	C
NC_040253.1	80650376	G	C
NC_040253.1	80767342	C	G
NC_040253.1	80796432	C	A
NC_040253.1	80801855	T	C
NC_040253.1	80844851	T	C
NC_040253.1	80877593	A	G
NC_040253.1	80906536	G	T
NC_040253.1	80955355	A	T
NC_040253.1	80998336	T	G

NC_040253.1	81091325	G	A
NC_040253.1	81120690	G	A
NC_040253.1	81145539	A	G
NC_040253.1	81195999	T	C
NC_040253.1	81254351	T	C
NC_040253.1	81312015	C	G
NC_040253.1	81331433	C	G
NC_040253.1	81427476	C	G
NC_040253.1	81436429	C	G
NC_040253.1	81641150	G	A
NC_040253.1	81717357	G	C
NC_040253.1	81762650	T	C
NC_040253.1	81793079	A	C
NC_040253.1	81840930	G	T
NC_040253.1	81882031	C	A
NC_040253.1	81919301	T	C
NC_040253.1	81957961	T	C
NC_040253.1	81970457	T	A
NC_040253.1	82015260	T	C
NC_040253.1	82018472	G	T
NC_040253.1	82058522	A	G
NC_040253.1	82080781	A	G
NC_040253.1	82141754	C	T
NC_040253.1	82176235	C	T
NC_040253.1	82199134	A	G
NC_040253.1	82251403	C	T
NC_040253.1	82297705	T	C
NC_040253.1	82363903	G	A
NC_040253.1	82434208	G	A
NC_040253.1	82479331	T	G
NC_040253.1	82509846	C	T
NC_040253.1	82551721	G	T
NC_040253.1	82615991	A	G
NC_040253.1	82671159	G	A
NC_040253.1	82724642	A	T
NC_040253.1	82768849	T	C
NC_040253.1	82807391	C	T
NC_040253.1	82888328	G	A
NC_040253.1	83015210	C	T
NC_040253.1	83180925	C	T
NC_040253.1	83203800	C	T
NC_040253.1	83243535	A	G
NC_040253.1	83257294	C	T
NC_040253.1	83357709	T	C
NC_040253.1	83473385	A	C
NC_040253.1	83510671	C	T
NC_040253.1	83738989	C	T

NC_040253.1	83796067	C	T
NC_040253.1	83832752	A	G
NC_040253.1	83903158	A	G
NC_040253.1	84294628	G	C
NC_040253.1	84326634	0	C
NC_040253.1	84365100	A	C
NC_040253.1	84433006	G	A
NC_040253.1	84571927	C	A
NC_040253.1	84683924	G	T
NC_040253.1	84788682	A	C
NC_040253.1	84838445	T	A
NC_040253.1	84868386	0	G
NC_040253.1	84941344	C	G
NC_040253.1	85065220	C	T
NC_040253.1	85521385	A	G
NC_040253.1	85552684	G	A
NC_040253.1	85595912	T	A
NC_040253.1	85660780	A	C
NC_040253.1	85690557	T	A
NC_040253.1	85873469	T	C
NC_040253.1	86147120	T	C
NC_040253.1	86249497	G	A
NC_040253.1	86306364	A	G
NC_040253.1	86359794	C	T
NC_040253.1	86517875	T	G
NC_040253.1	86583774	T	C
NC_040253.1	86679822	T	A
NC_040253.1	86778572	T	C
NC_040253.1	86906234	G	A
NC_040253.1	87356349	A	G
NC_040253.1	87532401	T	C
NC_040253.1	87718128	A	C
NC_040253.1	87762039	A	G
NC_040253.1	87824937	G	A
NC_040253.1	87903661	C	T
NC_040253.1	87952260	T	C
NC_040253.1	88047771	G	A
NC_040253.1	88054326	A	G
NC_040253.1	88185726	T	C
NC_040253.1	88233453	A	G
NC_040253.1	88254713	T	C
NC_040253.1	88444359	A	G
NC_040253.1	88472484	T	C
NC_040253.1	88494613	A	C
NC_040253.1	88540730	C	T
NC_040253.1	88545723	T	C
NC_040253.1	88605353	G	A

NC_040253.1	88644522	A	G
NC_040253.1	88684768	C	G
NC_040253.1	88736646	G	C
NC_040253.1	88764334	T	C
NC_040253.1	88804778	T	C
NC_040253.1	88824413	C	T
NC_040253.1	88856013	T	G
NC_040253.1	88871841	A	C
NC_040253.1	88919291	A	G
NC_040253.1	88952012	T	C
NC_040253.1	89051033	A	T
NC_040253.1	89106508	A	G
NC_040253.1	89162978	T	C
NC_040253.1	89190953	T	C
NC_040253.1	89212925	A	G
NC_040253.1	89280648	G	A
NC_040253.1	89320320	C	T
NC_040253.1	89367356	T	C
NC_040253.1	89378599	G	A
NC_040253.1	89423218	C	T
NC_040253.1	89431135	C	T
NC_040253.1	89483700	T	C
NC_040253.1	89491178	A	G
NC_040253.1	89546545	T	C
NC_040253.1	89585799	A	G
NC_040253.1	89602593	T	A
NC_040253.1	89635055	T	C
NC_040253.1	89723143	C	T
NC_040253.1	89768184	T	C
NC_040253.1	89796761	C	T
NC_040253.1	89825798	A	G
NC_040253.1	89854198	T	C
NC_040253.1	89933090	G	A
NC_040253.1	89955589	G	T
NC_040253.1	89998615	C	T
NC_040253.1	90035772	C	T
NC_040253.1	90077681	G	A
NC_040253.1	90110978	A	G
NC_040253.1	90132228	C	G
NC_040253.1	90188623	A	G
NC_040253.1	90214986	G	A
NC_040253.1	90244382	A	T
NC_040253.1	90469795	C	T
NC_040253.1	90513943	A	G
NC_040253.1	90538138	C	G
NC_040253.1	90629686	T	C
NC_040253.1	90696915	T	C

NC_040253.1	90793489	G	A
NC_040253.1	90853451	T	C
NC_040253.1	90918464	T	G
NC_040253.1	90964245	A	G
NC_040253.1	91023379	G	A
NC_040253.1	91050280	T	A
NC_040253.1	91121751	G	A
NC_040253.1	91161356	C	G
NC_040253.1	91175074	A	C
NC_040253.1	91237629	C	A
NC_040253.1	91295615	C	A
NC_040253.1	91351177	A	G
NC_040253.1	91385885	T	C
NC_040253.1	91436302	C	T
NC_040253.1	91457996	T	G
NC_040253.1	91546531	A	G
NC_040253.1	91560169	T	C
NC_040253.1	91592373	T	C
NC_040253.1	91625813	C	G
NC_040253.1	91651331	G	C
NC_040253.1	91696056	T	C
NC_040253.1	91739672	A	C
NC_040253.1	91801733	T	C
NC_040253.1	91868990	A	G
NC_040253.1	91876288	C	G
NC_040253.1	91904157	A	G
NC_040253.1	91928557	C	T
NC_040253.1	91966436	A	G
NC_040253.1	91992333	T	C
NC_040253.1	92030700	A	G
NC_040253.1	92096751	C	T
NC_040253.1	92149090	A	G
NC_040253.1	92301017	G	A
NC_040253.1	92372319	C	G
NC_040253.1	92441474	C	T
NC_040253.1	92468154	G	A
NC_040253.1	92521447	C	T
NC_040253.1	92647201	A	G
NC_040253.1	92680594	A	G
NC_040253.1	92774663	T	G
NC_040253.1	92876709	T	C
NC_040253.1	92909332	T	C
NC_040253.1	93083536	C	T
NC_040253.1	93138504	C	G
NC_040253.1	93173187	A	T
NC_040253.1	93223692	G	A
NC_040253.1	93258846	T	C

NC_040253.1	93272255	A	G
NC_040253.1	93304552	A	G
NC_040253.1	93394736	A	T
NC_040253.1	93435404	T	G
NC_040253.1	93496069	G	T
NC_040253.1	93535483	T	A
NC_040253.1	93574784	G	T
NC_040253.1	93583098	G	A
NC_040253.1	93614342	A	G
NC_040253.1	93636598	G	A
NC_040253.1	93663358	T	A
NC_040253.1	93706346	A	T
NC_040253.1	93713138	G	T
NC_040253.1	93878984	C	A
NC_040253.1	93918077	A	G
NC_040253.1	93944069	A	G
NC_040253.1	93987824	A	G
NC_040253.1	94014743	G	A
NC_040253.1	94034352	T	C
NC_040253.1	94132010	G	A
NC_040253.1	94143254	T	G
NC_040253.1	94159068	T	C
NC_040253.1	94218793	G	A
NC_040253.1	94276017	A	C
NC_040253.1	94309948	T	A
NC_040253.1	94330973	A	G
NC_040253.1	94387773	G	C
NC_040253.1	94406797	T	C
NC_040253.1	94465959	T	C
NC_040253.1	94512312	T	C
NC_040253.1	94538505	A	G
NC_040253.1	94564546	G	A
NC_040253.1	94572639	T	G
NC_040253.1	94642091	G	A
NC_040253.1	94667605	G	A
NC_040253.1	94693199	C	T
NC_040253.1	94715416	T	C
NC_040253.1	94768768	C	T
NC_040253.1	94821738	C	T
NC_040253.1	94852494	A	G
NC_040253.1	94863348	A	G
NC_040253.1	94889507	T	C
NC_040253.1	94923475	C	T
NC_040253.1	94956176	A	G
NC_040253.1	94976407	T	C
NC_040253.1	95003778	A	G
NC_040253.1	95022593	C	T

NC_040253.1	95084225	C	T
NC_040253.1	95126536	T	C
NC_040253.1	95141616	T	C
NC_040253.1	95223056	G	C
NC_040253.1	95269856	A	G
NC_040253.1	95323820	A	C
NC_040253.1	95405775	T	C
NC_040253.1	95430932	G	A
NC_040253.1	95513199	T	C
NC_040253.1	95539151	C	T
NC_040253.1	95594718	T	G
NC_040253.1	95665884	G	A
NC_040253.1	95704279	T	C
NC_040253.1	95729643	T	C
NC_040253.1	95755724	C	T
NC_040253.1	95771323	A	G
NC_040253.1	95819789	A	G
NC_040253.1	95830635	A	G
NC_040253.1	95867187	A	G
NC_040253.1	95900240	T	C
NC_040253.1	95941569	A	G
NC_040253.1	96026781	C	T
NC_040253.1	96102632	T	C
NC_040253.1	96141467	T	C
NC_040253.1	96174296	T	A
NC_040253.1	96227764	T	C
NC_040253.1	96241034	G	A
NC_040253.1	96281763	A	G
NC_040253.1	96303117	A	C
NC_040253.1	96355742	G	A
NC_040253.1	96395589	T	C
NC_040253.1	96403495	C	T
NC_040253.1	96440650	T	G
NC_040253.1	96486169	T	C
NC_040253.1	96520888	C	T
NC_040253.1	96532537	C	T
NC_040253.1	96576432	A	G
NC_040253.1	96622518	T	C
NC_040253.1	96630550	A	G
NC_040253.1	97104272	T	A
NC_040253.1	97260036	A	G
NC_040253.1	97289567	A	G
NC_040253.1	97354439	C	T
NC_040253.1	97646913	T	C
NC_040253.1	97679126	T	C
NC_040253.1	97699938	A	G
NC_040253.1	97753240	T	C

NC_040253.1	97815360	G	C
NC_040253.1	97882115	A	G
NC_040253.1	97914790	T	C
NC_040253.1	97941565	G	A
NC_040253.1	97998773	T	A
NC_040253.1	98035729	C	T
NC_040253.1	98048964	C	T
NC_040253.1	98089622	T	C
NC_040253.1	98109668	G	A
NC_040253.1	98135444	C	T
NC_040253.1	98147309	G	C
NC_040253.1	98189264	A	G
NC_040253.1	98210054	G	A
NC_040253.1	98264022	A	G
NC_040253.1	98307340	A	G
NC_040253.1	98354004	T	C
NC_040253.1	98376286	C	T
NC_040253.1	98417169	A	G
NC_040253.1	98437083	G	C
NC_040253.1	98476769	A	G
NC_040253.1	98489002	C	T
NC_040253.1	98558296	T	C
NC_040253.1	98619502	C	T
NC_040253.1	98672683	G	A
NC_040253.1	98681708	A	C
NC_040253.1	98737845	A	C
NC_040253.1	98810580	G	C
NC_040253.1	98983763	A	G
NC_040253.1	99036642	T	C
NC_040253.1	99139048	A	G
NC_040253.1	99217959	C	T
NC_040253.1	99284446	A	G
NC_040253.1	99362102	T	C
NC_040253.1	99404567	A	G
NC_040253.1	99420159	T	G
NC_040253.1	99467235	G	A
NC_040253.1	99539705	G	A
NC_040253.1	99614234	C	T
NC_040253.1	99646950	A	G
NC_040253.1	99703542	A	G
NC_040253.1	99730251	T	A
NC_040253.1	99765285	A	C
NC_040253.1	99771512	A	G
NC_040253.1	99850421	A	T
NC_040253.1	99899299	C	G
NC_040253.1	99907958	C	G
NC_040253.1	99934732	T	C

NC_040253.1	100004731	C	A
NC_040253.1	100122522	T	C
NC_040253.1	100171158	G	A
NC_040253.1	100279996	C	T
NC_040253.1	100325918	T	G
NC_040253.1	100385070	C	T
NC_040253.1	100466154	C	T
NC_040253.1	100505832	A	G
NC_040253.1	100536081	C	T
NC_040253.1	100610776	T	G
NC_040253.1	100667329	T	C
NC_040253.1	100751742	A	G
NC_040253.1	100815222	G	A
NC_040253.1	100846748	T	C
NC_040253.1	100925860	T	A
NC_040253.1	100963221	T	C
NC_040253.1	101016035	G	A
NC_040253.1	101068420	C	T
NC_040253.1	101143636	T	C
NC_040253.1	101197567	A	T
NC_040253.1	101248931	C	T
NC_040253.1	101265896	T	C
NC_040253.1	101363156	T	C
NC_040253.1	101548373	A	G
NC_040253.1	101660453	T	C
NC_040253.1	101735059	C	T
NC_040253.1	101761432	A	G
NC_040253.1	101881026	T	G
NC_040253.1	101937391	T	C
NC_040253.1	101985328	C	T
NC_040253.1	102029470	C	T
NC_040253.1	102079578	T	C
NC_040253.1	102130658	G	A
NC_040253.1	102193992	G	T
NC_040253.1	102223842	C	T
NC_040253.1	102260520	T	A
NC_040253.1	102318227	C	T
NC_040253.1	102352692	A	T
NC_040253.1	102401569	G	T
NC_040253.1	102435594	G	A
NC_040253.1	102475770	A	G
NC_040253.1	102495198	T	C
NC_040253.1	102549585	A	C
NC_040253.1	102593422	C	T
NC_040253.1	102647104	C	A
NC_040253.1	102676307	G	C
NC_040253.1	102733041	C	T

NC_040253.1	102783642	C	T
NC_040253.1	102850492	C	T
NC_040253.1	102895997	A	G
NC_040253.1	102927062	G	A
NC_040253.1	102985708	T	G
NC_040253.1	103043821	T	G
NC_040253.1	103082695	T	C
NC_040253.1	103123805	C	A
NC_040253.1	103161373	C	T
NC_040253.1	103213091	T	G
NC_040253.1	103269904	A	G
NC_040253.1	103339541	A	G
NC_040253.1	103345798	T	C
NC_040253.1	103408371	G	A
NC_040253.1	103446725	G	A
NC_040253.1	103477955	C	T
NC_040253.1	103544204	A	C
NC_040253.1	103566269	G	T
NC_040253.1	103620501	T	C
NC_040253.1	103630900	T	C
NC_040253.1	103708595	T	C
NC_040253.1	103734105	G	A
NC_040253.1	103831024	C	T
NC_040253.1	103872296	A	C
NC_040253.1	103912238	T	A
NC_040253.1	104041741	G	A
NC_040253.1	104107264	G	T
NC_040253.1	104141226	C	T
NC_040253.1	104158903	G	T
NC_040253.1	104220496	T	C
NC_040253.1	104328096	A	G
NC_040253.1	104358844	C	T
NC_040253.1	104379947	G	A
NC_040253.1	104442359	C	T
NC_040253.1	104469853	T	C
NC_040253.1	104513605	A	C
NC_040253.1	104520965	T	C
NC_040253.1	104545763	C	T
NC_040253.1	104595675	C	G
NC_040253.1	104646718	T	G
NC_040253.1	104686961	C	T
NC_040253.1	104780381	A	G
NC_040253.1	104839145	A	G
NC_040253.1	104870905	A	T
NC_040253.1	104896645	C	A
NC_040253.1	104962187	C	T
NC_040253.1	104970628	G	A

NC_040253.1	105088765	A	G
NC_040253.1	105135183	G	T
NC_040253.1	105333415	C	T
NC_040253.1	105421895	A	G
NC_040253.1	105455497	A	G
NC_040253.1	105485196	A	G
NC_040253.1	105537479	A	T
NC_040253.1	105563255	C	A
NC_040253.1	105573238	T	G
NC_040253.1	105633826	G	A
NC_040253.1	105786637	G	T
NC_040253.1	105872382	G	A
NC_040253.1	105925082	C	T
NC_040253.1	105957582	G	A
NC_040253.1	105972921	T	C
NC_040253.1	105998070	A	G
NC_040253.1	106046490	G	A
NC_040253.1	106096280	C	T
NC_040253.1	106122502	C	T
NC_040253.1	106128182	G	A
NC_040253.1	106207885	T	C
NC_040253.1	106232017	G	C
NC_040253.1	106322707	A	G
NC_040253.1	106420772	A	T
NC_040253.1	106425608	G	A
NC_040253.1	106525185	C	T
NC_040253.1	106546119	G	A
NC_040253.1	106798160	C	T
NC_040253.1	106925598	T	C
NC_040253.1	107001321	T	G
NC_040253.1	107266814	G	A
NC_040253.1	107376891	G	A
NC_040253.1	107646010	C	T
NC_040253.1	107729364	T	C
NC_040253.1	107779864	T	C
NC_040253.1	107880624	G	A
NC_040253.1	108194949	A	T
NC_040253.1	108223167	T	C
NC_040253.1	108496900	C	T
NC_040253.1	108548433	A	G
NC_040253.1	108666542	T	C
NC_040253.1	108716004	C	T
NC_040253.1	108746187	T	C
NC_040253.1	108778027	G	A
NC_040253.1	108850715	T	A
NC_040253.1	108977152	T	C
NC_040253.1	109060973	T	C

NC_040253.1	109124585	C	T
NC_040253.1	109179009	G	A
NC_040253.1	109573483	A	G
NC_040253.1	109631817	G	A
NC_040253.1	109783423	T	C
NC_040253.1	109823750	C	T
NC_040253.1	109970749	G	T
NC_040253.1	110166719	C	T
NC_040253.1	110288093	T	C
NC_040253.1	110335438	G	C
NC_040253.1	110775953	C	G
NC_040253.1	110969149	G	A
NC_040253.1	111018244	A	G
NC_040253.1	111062665	G	A
NC_040253.1	111949178	A	G
NC_040253.1	112669388	T	C
NC_040253.1	113090127	T	G
NC_040253.1	113319042	T	G
NC_040253.1	113509478	A	G
NC_040253.1	113728289	C	A
NC_040253.1	113884556	T	C
NC_040253.1	114168284	T	G
NC_040253.1	114218368	G	A
NC_040253.1	114276405	A	C
NC_040253.1	114312538	T	A
NC_040253.1	114460685	T	C
NC_040253.1	114538706	A	T
NC_040253.1	114609176	A	C
NC_040253.1	114723273	T	G
NC_040253.1	114760617	C	T
NC_040253.1	114911482	A	G
NC_040253.1	115151117	G	A
NC_040253.1	115223401	T	C
NC_040253.1	115437830	A	G
NC_040253.1	115751538	A	G
NC_040253.1	115800498	G	C
NC_040253.1	115902114	C	T
NC_040253.1	115961339	G	A
NC_040253.1	116158474	T	G
NC_040253.1	116235751	T	C
NC_040253.1	116323523	T	C
NC_040253.1	116480458	G	A
NC_040253.1	116841230	T	C
NC_040253.1	117355776	C	T
NC_040253.1	117583275	C	G
NC_040253.1	118014065	C	T
NC_040253.1	118486269	A	G

NC_040253.1	119148650	C	T
NC_040253.1	119235433	A	C
NC_040253.1	119843596	G	A
NC_040253.1	119996206	C	T
NC_040253.1	120207618	G	A
NC_040253.1	120345593	T	A
NC_040253.1	120409412	G	A
NC_040253.1	120453394	T	C
NC_040253.1	120711950	G	A
NC_040253.1	120746998	A	G
NC_040253.1	120806456	G	T
NC_040253.1	120836737	T	G
NC_040253.1	121036863	T	C
NC_040253.1	121068306	T	C
NC_040253.1	121194076	T	A
NC_040253.1	121331459	T	C
NC_040253.1	121365654	A	G
NC_040253.1	121463967	T	C
NC_040253.1	121509866	T	C
NC_040253.1	121549127	T	G
NC_040253.1	121587410	C	T
NC_040253.1	121685769	A	G
NC_040253.1	121768999	T	A
NC_040253.1	121802468	G	A
NC_040253.1	121836723	T	C
NC_040253.1	121978206	T	A
NC_040253.1	122178434	A	G
NC_040253.1	122200881	C	T
NC_040253.1	122660304	T	G
NC_040253.1	122788522	T	C
NC_040253.1	123228133	G	A
NC_040253.1	124112422	C	T
NC_040253.1	124412033	T	C
NC_040253.1	124517974	T	G
NC_040253.1	124594569	T	C
NC_040253.1	124722110	A	G
NC_040253.1	124911392	T	G
NC_040253.1	125582839	T	C
NC_040253.1	125728296	C	T
NC_040253.1	125810572	G	C
NC_040253.1	125843219	T	C
NC_040253.1	126410612	C	T
NC_040253.1	126797427	T	C
NC_040253.1	126884635	A	G
NC_040253.1	127280299	G	A
NC_040253.1	127330413	C	T
NC_040253.1	127410791	G	A

NC_040253.1	127442711	A	G
NC_040253.1	127515037	A	C
NC_040253.1	127594845	A	G
NC_040253.1	127729930	G	C
NC_040253.1	127885970	A	G
NC_040253.1	128150180	A	G
NC_040253.1	128176202	G	C
NC_040253.1	128254073	C	T
NC_040253.1	128468785	G	A
NC_040253.1	128672520	T	G
NC_040253.1	128751228	T	C
NC_040253.1	128877907	G	A
NC_040253.1	128927852	C	A
NC_040253.1	129027397	A	G
NC_040253.1	129267631	T	C
NC_040253.1	129419370	A	G
NC_040253.1	129734857	G	C
NC_040253.1	129877123	A	T
NC_040253.1	130191096	0	C
NC_040253.1	130239525	C	T
NC_040253.1	130449437	T	C
NC_040253.1	130578353	C	T
NC_040253.1	130691219	T	C
NC_040253.1	131008674	T	C
NC_040253.1	131070341	G	A
NC_040253.1	131135935	A	G
NC_040253.1	131204593	A	G
NC_040253.1	131469258	C	T
NC_040253.1	131713322	C	T
NC_040253.1	131774074	A	T
NC_040253.1	131832603	A	G
NC_040253.1	131887263	T	C
NC_040253.1	131943184	G	T
NC_040253.1	132005583	T	C
NC_040253.1	132059145	A	C
NC_040253.1	132117000	C	A
NC_040253.1	132144952	C	T
NC_040253.1	132482504	C	T
NC_040253.1	132757729	T	C
NC_040253.1	132993087	T	C
NC_040253.1	133430395	C	T
NC_040253.1	133559100	G	A
NC_040253.1	133628945	A	G
NC_040253.1	133786766	C	T
NC_040253.1	133995179	A	G
NC_040253.1	134384616	0	C
NC_040253.1	134547571	G	T

NC_040253.1	134704039	T	C
NC_040253.1	135238870	G	T
NC_040253.1	135306613	C	T
NC_040253.1	135518108	T	G
NC_040253.1	135996383	C	T
NC_040253.1	136124932	C	T
NC_040253.1	136419031	A	G
NC_040253.1	136501296	T	C
NC_040253.1	136633363	A	G
NC_040253.1	137430349	G	A
NC_040253.1	137834124	G	A
NC_040253.1	137976408	A	G
NC_040253.1	138028497	A	G
NC_040253.1	138097910	T	C
NC_040253.1	138153624	A	C
NC_040253.1	138216277	T	C
NC_040253.1	138274939	G	A
NC_040253.1	138337796	G	A
NC_040253.1	138394025	T	C
NC_040253.1	138449999	G	T
NC_040253.1	138517596	G	A
NC_040253.1	138578857	G	T
NC_040253.1	138641945	T	C
NC_040253.1	138699221	G	A
NC_040253.1	138763823	A	G
NC_040253.1	138826323	T	C
NC_040253.1	138936055	A	G
NC_040253.1	138999788	A	G
NC_040253.1	139062890	A	C
NC_040253.1	139121885	C	T
NC_040253.1	139186470	T	C
NC_040253.1	139260518	G	A
NC_040253.1	139362757	T	C
NC_040253.1	139427840	A	C
NC_040253.1	139483995	A	C
NC_040253.1	139536584	T	C
NC_040253.1	139591379	C	T
NC_040253.1	139620667	T	C
NC_040253.1	139672630	C	T
NC_040253.1	139742229	A	T
NC_040253.1	139800344	C	T
NC_040253.1	139863600	C	T
NC_040253.1	140213938	G	A
NC_040253.1	140271667	G	A
NC_040253.1	140333372	A	C
NC_040253.1	140396662	C	T
NC_040253.1	140454218	G	A

NC_040253.1	140513282	A	T
NC_040253.1	140572021	A	G
NC_040253.1	140628123	A	C
NC_040253.1	140686028	C	T
NC_040253.1	140759618	T	C
NC_040253.1	140862844	A	G
NC_040253.1	140894091	G	A
NC_040253.1	140996714	C	T
NC_040253.1	141054965	A	G
NC_040253.1	141118297	C	A
NC_040253.1	141176348	A	G
NC_040253.1	141242517	C	T
NC_040253.1	141292787	A	G
NC_040253.1	141351850	G	C
NC_040253.1	141401402	C	G
NC_040253.1	141453415	T	C
NC_040253.1	141515966	T	A
NC_040253.1	141579413	T	C
NC_040253.1	141633636	A	G
NC_040253.1	141710676	C	A
NC_040253.1	141766622	G	C
NC_040253.1	141821995	T	C
NC_040253.1	141878810	G	A
NC_040253.1	141932854	A	C
NC_040253.1	141993945	A	G
NC_040253.1	142117448	C	G
NC_040253.1	142173973	G	C
NC_040253.1	142232154	T	G
NC_040253.1	142286644	G	T
NC_040253.1	142348306	T	C
NC_040253.1	142411144	T	C
NC_040253.1	142470574	G	A
NC_040253.1	142525698	A	G
NC_040253.1	142587314	T	G
NC_040253.1	142650818	C	T
NC_040253.1	142704231	C	T
NC_040253.1	142781853	A	G
NC_040253.1	142838528	T	G
NC_040253.1	142885274	G	T
NC_040253.1	142945030	C	T
NC_040253.1	143007240	G	A
NC_040253.1	143064152	A	G
NC_040253.1	143123913	A	G
NC_040253.1	143183315	T	C
NC_040253.1	143237569	T	C
NC_040253.1	143298735	A	G
NC_040253.1	143350294	C	T

NC_040253.1	143403575	A	T
NC_040253.1	143461132	G	T
NC_040253.1	143525427	T	A
NC_040253.1	143582993	C	T
NC_040253.1	143645538	T	C
NC_040253.1	143697933	A	G
NC_040253.1	143760928	C	A
NC_040253.1	143832423	T	C
NC_040253.1	143891410	C	A
NC_040253.1	143956475	T	C
NC_040253.1	144010595	C	T
NC_040253.1	144067974	A	G
NC_040253.1	144121460	G	T
NC_040253.1	144190261	C	T
NC_040253.1	144245146	C	A
NC_040253.1	144305437	A	G
NC_040253.1	144365598	C	T
NC_040253.1	144477114	T	A
NC_040253.1	144545202	A	G
NC_040253.1	144601661	A	G
NC_040253.1	144661545	C	G
NC_040253.1	144717076	T	A
NC_040253.1	144837819	A	G
NC_040253.1	144899547	A	G
NC_040253.1	144957861	C	A
NC_040253.1	145018811	T	C
NC_040253.1	145081269	T	C
NC_040253.1	145141935	G	C
NC_040253.1	145210576	A	G
NC_040253.1	145270914	T	C
NC_040253.1	145326750	G	T
NC_040253.1	145379190	A	G
NC_040253.1	145446521	C	T
NC_040253.1	145562832	G	A
NC_040253.1	145618764	A	C
NC_040253.1	145681129	C	T
NC_040253.1	145742586	G	C
NC_040253.1	145854484	A	G
NC_040253.1	145920806	A	G
NC_040253.1	145977480	C	T
NC_040253.1	146033572	A	G
NC_040253.1	146089275	A	G
NC_040253.1	146146959	T	G
NC_040253.1	146210968	G	C
NC_040253.1	146271409	A	G
NC_040253.1	146325192	C	G
NC_040253.1	146384684	G	C

NC_040253.1	146438715	C	A
NC_040253.1	146497033	G	T
NC_040253.1	146558609	C	G
NC_040253.1	146615983	G	A
NC_040253.1	146673506	C	A
NC_040253.1	146730017	C	T
NC_040253.1	146788661	A	G
NC_040253.1	146845801	G	T
NC_040253.1	146901415	A	G
NC_040253.1	146958567	A	G
NC_040253.1	147016536	C	T
NC_040253.1	147076420	A	G
NC_040253.1	147135686	G	A
NC_040253.1	147191256	A	C
NC_040253.1	147253321	C	T
NC_040253.1	147433431	T	A
NC_040253.1	147491094	G	C
NC_040253.1	147547528	C	G
NC_040253.1	147605936	G	A
NC_040253.1	147663248	A	G
NC_040253.1	147717042	T	C
NC_040253.1	147781944	C	T
NC_040253.1	147840264	C	A
NC_040253.1	147899135	C	T
NC_040253.1	147959091	A	G
NC_040253.1	148023682	G	A
NC_040253.1	148147503	A	C
NC_040253.1	148201248	G	A
NC_040253.1	148275204	C	A
NC_040253.1	148329891	A	C
NC_040253.1	148515960	T	C
NC_040253.1	148575432	T	C
NC_040253.1	148628318	A	G
NC_040253.1	148690185	G	T
NC_040253.1	148748824	A	G
NC_040253.1	148863132	T	G
NC_040253.1	148921280	C	A
NC_040253.1	148978054	T	A
NC_040253.1	149035801	A	G
NC_040253.1	149092172	T	C
NC_040253.1	149151042	G	A
NC_040253.1	149210919	C	T
NC_040253.1	149273082	T	C
NC_040253.1	149333822	G	A
NC_040253.1	149396139	A	G
NC_040253.1	149622735	G	A
NC_040253.1	149685623	A	C

NC_040253.1	149746825	T	C
NC_040253.1	149918060	A	C
NC_040253.1	149978600	T	C
NC_040253.1	150039675	A	G
NC_040253.1	150098536	T	C
NC_040253.1	150161582	T	G
NC_040253.1	150273794	A	G
NC_040253.1	150336369	T	C
NC_040253.1	150357372	C	T
NC_040253.1	150416535	C	G
NC_040253.1	150483247	T	A
NC_040253.1	150541489	A	G
NC_040253.1	150605555	T	C
NC_040253.1	150668852	T	C
NC_040253.1	150780733	T	C
NC_040253.1	150838558	A	G
NC_040253.1	150900049	A	G
NC_040253.1	150956884	G	A
NC_040253.1	151010443	A	G
NC_040253.1	151072512	T	C
NC_040253.1	151128239	C	T
NC_040253.1	151185324	A	G
NC_040253.1	151251289	G	A
NC_040253.1	151318701	A	G
NC_040253.1	151386787	T	C
NC_040253.1	151455624	G	T
NC_040253.1	151507831	G	A
NC_040253.1	151562798	A	G
NC_040253.1	151628007	C	T
NC_040253.1	151689703	G	T
NC_040253.1	151749695	A	G
NC_040253.1	151810981	C	T
NC_040253.1	151879474	C	T
NC_040253.1	151994594	C	G
NC_040253.1	152050965	G	A
NC_040253.1	152109092	T	C
NC_040253.1	152167482	T	C
NC_040253.1	152231535	C	A
NC_040253.1	152293156	T	C
NC_040253.1	152357153	C	T
NC_040253.1	152413444	A	G
NC_040253.1	152474038	A	C
NC_040253.1	152555137	G	A
NC_040253.1	152609615	A	T
NC_040253.1	152668940	T	C
NC_040253.1	152729611	T	C
NC_040253.1	152803618	A	G

NC_040253.1	152858689	G	T
NC_040253.1	152925085	T	C
NC_040253.1	153047555	A	C
NC_040253.1	153108803	A	G
NC_040253.1	153169012	A	G
NC_040253.1	153469906	G	T
NC_040253.1	153550000	G	A
NC_040253.1	153596167	C	G
NC_040253.1	153650229	T	C
NC_040253.1	153831514	T	C
NC_040253.1	153891672	G	A
NC_040253.1	153950979	C	A
NC_040253.1	154101752	T	C
NC_040253.1	154427728	G	T
NC_040253.1	154485967	T	A
NC_040253.1	154548372	G	A
NC_040253.1	154614694	T	C
NC_040253.1	154683658	A	G
NC_040253.1	154742580	G	C
NC_040253.1	154813604	C	A
NC_040253.1	154877516	C	G
NC_040253.1	155058315	G	A
NC_040253.1	155233319	T	C
NC_040253.1	155354655	G	A
NC_040253.1	155422662	C	T
NC_040253.1	155492678	T	A
NC_040253.1	155508459	A	T
NC_040253.1	155563390	T	C
NC_040253.1	155620304	G	A
NC_040253.1	155677399	G	A
NC_040253.1	155736445	G	A
NC_040253.1	155797725	T	C
NC_040253.1	155860580	T	A
NC_040253.1	155919848	C	T
NC_040253.1	155980444	C	T
NC_040253.1	156040801	A	G
NC_040253.1	156095900	T	G
NC_040253.1	156124700	A	C
NC_040253.1	156185423	A	G
NC_040253.1	156251633	T	G
NC_040253.1	156314694	A	G
NC_040253.1	156377509	A	G
NC_040253.1	156447984	T	C
NC_040253.1	156566451	A	T
NC_040253.1	156626036	T	A
NC_040253.1	156696303	A	C
NC_040253.1	156761258	G	A

NC_040253.1	156817796	G	A
NC_040253.1	156878771	T	C
NC_040253.1	156947536	C	T
NC_040253.1	157086391	A	G
NC_040253.1	157247472	T	C
NC_040253.1	157371724	C	T
NC_040253.1	157395064	A	G
NC_040253.1	157516170	T	C
NC_040253.1	157574378	A	G
NC_040253.1	157632370	A	G
NC_040253.1	157692453	C	A
NC_040253.1	157751065	G	A
NC_040253.1	157810695	G	A
NC_040253.1	157869413	T	G
NC_040253.1	157923326	C	T
NC_040253.1	157991310	G	A
NC_040253.1	158058621	G	A
NC_040253.1	158120992	A	G
NC_040253.1	158179280	T	C
NC_040253.1	158237551	T	G
NC_040253.1	158369138	C	A
NC_040253.1	158431135	T	A
NC_040253.1	158498580	C	T
NC_040253.1	158555985	A	G
NC_040253.1	158614135	T	C
NC_040253.1	158671791	C	T
NC_040253.1	158731702	T	C
NC_040253.1	158800859	G	A
NC_040253.1	158858600	A	G
NC_040253.1	158916010	G	A
NC_040253.1	158977118	G	A
NC_040253.1	159037675	A	T
NC_040253.1	159214792	T	C
NC_040253.1	159268805	A	G
NC_040253.1	159335849	C	T
NC_040253.1	159394149	C	T
NC_040253.1	159460000	C	T
NC_040253.1	159524711	A	T
NC_040253.1	159637787	T	C
NC_040253.1	159697491	A	G
NC_040253.1	159757145	A	G
NC_040253.1	159815705	A	C
NC_040253.1	159873346	T	A
NC_040253.1	159989215	C	T
NC_040253.1	160054176	A	C
NC_040253.1	160119727	G	C
NC_040253.1	160171380	C	T

NC_040253.1	160283948	A	G
NC_040253.1	160343668	C	T
NC_040253.1	160402289	C	G
NC_040253.1	160462558	C	G
NC_040253.1	160519610	T	C
NC_040253.1	160574118	A	G
NC_040253.1	160638807	T	C
NC_040253.1	160696834	A	G
NC_040253.1	160759833	A	T
NC_040253.1	160817576	G	A
NC_040253.1	160874152	T	C
NC_040253.1	161537683	C	T
NC_040253.1	161589399	C	T
NC_040253.1	161654436	G	A
NC_040253.1	161712299	C	G
NC_040253.1	161769905	T	C
NC_040253.1	161827524	A	G
NC_040253.1	161884844	A	T
NC_040253.1	161944048	A	G
NC_040253.1	161999993	G	A
NC_040253.1	162055726	A	C
NC_040253.1	162118229	T	C
NC_040253.1	162173693	C	G
NC_040253.1	162231004	A	G
NC_040253.1	162289021	C	G
NC_040253.1	162347792	G	C
NC_040253.1	162402646	A	G
NC_040253.1	162470094	G	T
NC_040253.1	162541574	A	G
NC_040253.1	162597034	G	A
NC_040253.1	162657243	G	A
NC_040253.1	162724049	T	G
NC_040253.1	162782839	G	T
NC_040253.1	162845603	C	T
NC_040253.1	162913749	A	G
NC_040253.1	162979261	T	C
NC_040253.1	163037522	A	G
NC_040253.1	163091076	C	T
NC_040253.1	163156735	A	G
NC_040253.1	163218701	T	C
NC_040253.1	163280532	G	T
NC_040253.1	163361247	C	T
NC_040253.1	163537189	A	G
NC_040253.1	163597279	G	A
NC_040253.1	163654401	T	C
NC_040253.1	163724134	T	C
NC_040253.1	163776348	A	G

NC_040253.1	163836048	T	C
NC_040253.1	163897650	G	A
NC_040253.1	163952956	T	A
NC_040253.1	164017718	C	T
NC_040253.1	164091952	C	T
NC_040253.1	164812310	C	T
NC_040253.1	165352786	A	G
NC_040253.1	165670719	C	T
NC_040253.1	166444961	G	A
NC_040253.1	166734176	C	T
NC_040253.1	167361416	C	A
NC_040253.1	167420104	A	G
NC_040253.1	167611580	A	G
NC_040253.1	167668289	A	G
NC_040253.1	167731321	G	A
NC_040253.1	167804778	A	G
NC_040253.1	167970394	C	T
NC_040253.1	168031026	A	G
NC_040253.1	168091437	G	A
NC_040253.1	168294708	A	C
NC_040253.1	168360051	G	A
NC_040253.1	168428126	C	A
NC_040253.1	168488616	G	A
NC_040253.1	168550447	T	C
NC_040253.1	168591712	T	C
NC_040253.1	169578651	A	G
NC_040253.1	170338665	G	A
NC_040253.1	170397797	A	G
NC_040253.1	170453936	A	C
NC_040253.1	170512247	C	T
NC_040253.1	170573676	A	G
NC_040253.1	170632640	T	C
NC_040253.1	170694196	C	A
NC_040253.1	170761323	T	G
NC_040253.1	170818106	T	C
NC_040253.1	170876072	T	G
NC_040253.1	170956752	T	C
NC_040253.1	171023828	C	T
NC_040253.1	171096366	G	A
NC_040253.1	171150945	A	T
NC_040253.1	171217715	A	G
NC_040253.1	171280790	G	A
NC_040253.1	171354354	A	G
NC_040253.1	171419980	C	T
NC_040253.1	171434513	T	C
NC_040253.1	171673979	T	C
NC_040253.1	171893627	A	T

NC_040253.1	172353520	T	C
NC_040253.1	172501406	G	A
NC_040253.1	173318011	T	C
NC_040253.1	173484172	T	C
NC_040253.1	173677028	T	C
NC_040253.1	173894919	T	C
NC_040253.1	173950887	C	T
NC_040253.1	174016017	A	G
NC_040253.1	174079422	A	G
NC_040253.1	174139378	G	A
NC_040253.1	174204860	A	G
NC_040253.1	174278531	C	A
NC_040253.1	174352109	G	A
NC_040253.1	174408935	C	G
NC_040253.1	174462013	G	A
NC_040253.1	174529545	A	G
NC_040253.1	174601371	T	C
NC_040253.1	174665297	A	G
NC_040253.1	174732867	G	T
NC_040253.1	174791550	C	T
NC_040253.1	174852476	T	G
NC_040253.1	174912448	G	T
NC_040253.1	174946139	C	T
NC_040253.1	175201975	C	T
NC_040253.1	175447512	C	G
NC_040253.1	175873507	C	T
NC_040253.1	176185274	A	G
NC_040253.1	176962179	G	A
NC_040253.1	177780516	T	C
NC_040253.1	177943549	A	C
NC_040253.1	178112810	C	A
NC_040253.1	178170280	G	T
NC_040253.1	178234693	G	A
NC_040253.1	178354544	G	A
NC_040253.1	178523901	A	G
NC_040253.1	178595210	G	A
NC_040253.1	178652215	T	C
NC_040253.1	178715312	A	G
NC_040253.1	178775546	C	G
NC_040253.1	178836927	G	A
NC_040253.1	178993153	C	T
NC_040253.1	179058772	G	A
NC_040253.1	179100169	A	G
NC_040253.1	179265870	A	G
NC_040253.1	179580365	A	C
NC_040253.1	179642910	C	T
NC_040253.1	179708238	G	A

NC_040253.1	179769886	G	A
NC_040253.1	179829262	T	G
NC_040253.1	179949116	G	A
NC_040253.1	180023510	A	G
NC_040253.1	180080303	0	C
NC_040253.1	180133953	C	T
NC_040253.1	180207037	C	T
NC_040253.1	180384677	A	G
NC_040253.1	180440975	T	G
NC_040253.1	180704720	G	A
NC_040253.1	180753674	A	G
NC_040253.1	180820314	T	C
NC_040253.1	180887750	G	T
NC_040253.1	180962836	G	T
NC_040253.1	181025630	A	G
NC_040253.1	181135187	A	G
NC_040253.1	181211630	C	A
NC_040253.1	181269029	C	T
NC_040253.1	181334041	C	A
NC_040253.1	181355419	C	T
NC_040253.1	181544691	A	G
NC_040253.1	181607747	C	T
NC_040253.1	181682542	T	C
NC_040253.1	181855596	T	A
NC_040253.1	181914602	T	A
NC_040253.1	182156190	A	C
NC_040253.1	182214926	G	A
NC_040253.1	182275861	A	G
NC_040253.1	182335702	A	T
NC_040253.1	182392330	T	G
NC_040253.1	182469733	G	A
NC_040253.1	182495996	G	A
NC_040253.1	182579331	G	A
NC_040253.1	182875671	C	A
NC_040253.1	182929676	A	G
NC_040253.1	183005085	G	T
NC_040253.1	183066419	T	C
NC_040253.1	183130531	T	C
NC_040253.1	183151038	T	C
NC_040253.1	183219414	C	T
NC_040253.1	183280500	G	A
NC_040253.1	183354275	C	T
NC_040253.1	183416121	C	T
NC_040253.1	183466509	T	C
NC_040253.1	183532973	A	G
NC_040253.1	183599429	G	C
NC_040253.1	183652024	A	G

NC_040253.1	183714147	C	T
NC_040253.1	183746179	A	G
NC_040253.1	183807701	G	T
NC_040253.1	183874412	T	C
NC_040253.1	183942465	G	A
NC_040253.1	184001399	T	G
NC_040253.1	184063689	A	G
NC_040253.1	184126238	A	G
NC_040253.1	184181755	T	C
NC_040253.1	184242715	G	A
NC_040253.1	184304153	A	C
NC_040253.1	184364926	C	T
NC_040253.1	184430264	T	C
NC_040253.1	184500310	C	T
NC_040253.1	184560012	C	A
NC_040253.1	184622501	T	C
NC_040253.1	184660233	T	C
NC_040253.1	184719523	T	C
NC_040253.1	185071507	G	T
NC_040253.1	185139786	A	G
NC_040253.1	185208387	A	G
NC_040253.1	185265761	C	T
NC_040253.1	185331467	T	C
NC_040253.1	185392000	A	G
NC_040253.1	185454412	C	T
NC_040253.1	185510743	A	T
NC_040253.1	185580071	C	T
NC_040253.1	185649930	G	A
NC_040253.1	185721927	C	T
NC_040253.1	185764086	A	G
NC_040253.1	185823905	A	C
NC_040253.1	185882934	C	T
NC_040253.1	185939273	A	G
NC_040253.1	186000594	C	A
NC_040253.1	186064577	A	G
NC_040253.1	186096075	A	G
NC_040253.1	186150199	C	T
NC_040253.1	186207047	A	G
NC_040253.1	186279399	A	G
NC_040253.1	186337372	C	T
NC_040253.1	186402448	A	T
NC_040253.1	186462153	0	G
NC_040253.1	186521175	G	A
NC_040253.1	186576818	A	C
NC_040253.1	186634906	A	G
NC_040253.1	186708587	G	A
NC_040253.1	186773408	C	T

NC_040253.1	186836052	A	G
NC_040253.1	186902231	T	C
NC_040253.1	186964398	A	C
NC_040253.1	187022850	A	G
NC_040253.1	187078915	C	T
NC_040253.1	187140084	A	G
NC_040253.1	187201789	A	C
NC_040253.1	187256656	C	T
NC_040253.1	187316198	C	T
NC_040253.1	187373421	A	G
NC_040253.1	187438046	T	C
NC_040253.1	187552704	C	T
NC_040253.1	187611318	A	G
NC_040253.1	187665218	A	T
NC_040253.1	187719041	A	G
NC_040253.1	187783221	T	C
NC_040253.1	187854498	C	T
NC_040253.1	187974418	A	G
NC_040253.1	188037852	T	C
NC_040253.1	188090252	G	T
NC_040253.1	188148790	T	C
NC_040253.1	188220041	A	C
NC_040253.1	188324338	T	C
NC_040253.1	188383014	A	C
NC_040253.1	188441201	G	C
NC_040253.1	188500202	G	C
NC_040253.1	188678536	A	G
NC_040253.1	188737234	G	A
NC_040253.1	188795155	C	T
NC_040253.1	188858236	T	G
NC_040253.1	188920044	G	A
NC_040253.1	188980435	A	G
NC_040253.1	189041512	T	C
NC_040253.1	189098868	A	G
NC_040253.1	189124041	C	T
NC_040253.1	189173972	T	C
NC_040253.1	189230207	A	T
NC_040253.1	189289955	T	C
NC_040253.1	189347038	A	G
NC_040253.1	189403332	G	A
NC_040253.1	189464579	T	C
NC_040253.1	189523674	T	C
NC_040253.1	189585632	G	A
NC_040253.1	189642003	T	C
NC_040253.1	189703918	A	G
NC_040253.1	189761079	A	G
NC_040253.1	189818838	G	A

NC_040253.1	189876495	T	C
NC_040253.1	189934214	A	G
NC_040253.1	190001582	A	G
NC_040253.1	190061254	T	C
NC_040253.1	190114993	A	G
NC_040253.1	190179232	T	C
NC_040253.1	190254877	T	C
NC_040253.1	190330699	C	A
NC_040253.1	190381357	T	A
NC_040253.1	190442772	C	T
NC_040253.1	190500971	T	C
NC_040253.1	190558864	T	C
NC_040253.1	190615252	C	A
NC_040253.1	190674312	A	G
NC_040253.1	190733627	A	G
NC_040253.1	190789488	T	C
NC_040253.1	190846567	G	A
NC_040253.1	190879910	T	A
NC_040253.1	190930850	C	T
NC_040253.1	190989623	T	C
NC_040253.1	191054244	G	T
NC_040253.1	191181072	G	A
NC_040253.1	191237759	G	A
NC_040253.1	191309058	G	A
NC_040253.1	191364034	T	C
NC_040253.1	191416906	A	G
NC_040253.1	191592554	G	A
NC_040253.1	191654155	G	C
NC_040253.1	191711812	T	C
NC_040253.1	191777703	A	G
NC_040253.1	191831322	C	G
NC_040253.1	191898201	G	T
NC_040253.1	192015959	G	A
NC_040253.1	192065137	A	G
NC_040253.1	192103000	T	C
NC_040253.1	192170236	G	A
NC_040253.1	192223052	G	A
NC_040253.1	192292537	C	T
NC_040253.1	192352597	G	A
NC_040253.1	192528927	G	A
NC_040253.1	192587553	G	A
NC_040253.1	192644085	T	C
NC_040253.1	192706615	G	C
NC_040253.1	192767482	A	G
NC_040253.1	192896951	C	G
NC_040253.1	193018832	A	G
NC_040253.1	193092389	T	C

NC_040253.1	193418974	A	G
NC_040253.1	193481189	T	C
NC_040253.1	193552774	C	T
NC_040253.1	193584292	A	G
NC_040253.1	193654800	C	A
NC_040253.1	193713848	A	G
NC_040253.1	193789959	C	T
NC_040253.1	193847522	A	C
NC_040253.1	193965874	C	T
NC_040253.1	194022040	A	G
NC_040253.1	194217323	A	G
NC_040253.1	194229060	T	C
NC_040253.1	194471431	G	T
NC_040253.1	194524906	C	T
NC_040253.1	194574058	T	C
NC_040253.1	194627552	A	G
NC_040253.1	194722748	C	T
NC_040253.1	194785363	C	T
NC_040253.1	194844108	G	A
NC_040253.1	195143697	G	A
NC_040253.1	195200512	A	G
NC_040253.1	195221097	T	C
NC_040253.1	195348393	G	A
NC_040253.1	195413741	T	C
NC_040253.1	195483789	T	C
NC_040253.1	195743153	G	A
NC_040253.1	195801010	G	A
NC_040253.1	195982625	A	G
NC_040253.1	196045718	G	A
NC_040253.1	196114664	A	G
NC_040253.1	196170077	C	A
NC_040253.1	196230062	G	A
NC_040253.1	196318866	T	C
NC_040253.1	196416187	C	T
NC_040253.1	196501856	G	A
NC_040253.1	196556951	C	T
NC_040253.1	196616292	C	T
NC_040253.1	196675375	C	T
NC_040253.1	196730951	C	T
NC_040253.1	196800025	G	A
NC_040253.1	196852721	A	G
NC_040253.1	196911725	A	G
NC_040253.1	196969632	C	T
NC_040253.1	197031660	T	G
NC_040253.1	197074635	A	C
NC_040253.1	197863672	G	C
NC_040253.1	198684521	T	C

NC_040253.1	200167045	A	G
NC_040253.1	200834259	T	C
NC_040253.1	201100453	A	G
NC_040253.1	201395204	A	G
NC_040253.1	201457583	G	A
NC_040253.1	201521432	G	A
NC_040253.1	201578158	C	T
NC_040253.1	201719250	T	C
NC_040253.1	201781720	A	G
NC_040253.1	201848860	C	G
NC_040253.1	201905008	A	C
NC_040253.1	201973075	G	A
NC_040253.1	202032045	G	A
NC_040253.1	202063688	A	G
NC_040253.1	202157274	C	T
NC_040253.1	202463445	C	A
NC_040253.1	202540695	T	C
NC_040253.1	202719298	A	C
NC_040253.1	202768968	C	T
NC_040253.1	202971733	C	T
NC_040253.1	203207844	A	T
NC_040253.1	203270596	A	T
NC_040253.1	203315463	C	A
NC_040253.1	203407558	C	T
NC_040253.1	203472976	T	C
NC_040253.1	203546111	A	G
NC_040253.1	203604633	A	T
NC_040253.1	203659871	G	T
NC_040253.1	203730571	G	A
NC_040253.1	203790531	C	T
NC_040253.1	203851406	T	C
NC_040253.1	203870773	C	T
NC_040253.1	203924267	T	C
NC_040253.1	203970490	T	C
NC_040253.1	204018510	A	G
NC_040253.1	204138542	A	G
NC_040253.1	204157618	T	C
NC_040253.1	204188569	G	T
NC_040253.1	204879674	G	A
NC_040253.1	205063212	T	C
NC_040253.1	205173374	T	C
NC_040253.1	205248723	A	G
NC_040253.1	205305471	G	A
NC_040253.1	205357897	C	T
NC_040253.1	205472397	T	G
NC_040253.1	205533051	T	C
NC_040253.1	205598750	C	T

NC_040253.1	205671649	T	G
NC_040253.1	205731681	T	A
NC_040253.1	205872417	C	T
NC_040253.1	206118128	G	A
NC_040253.1	206186209	T	G
NC_040253.1	206263914	A	G
NC_040253.1	206857667	A	G
NC_040253.1	207133606	G	A
NC_040253.1	207622248	C	T
NC_040253.1	207824764	A	G
NC_040253.1	208045491	G	C
NC_040253.1	208102922	T	C
NC_040253.1	208212960	A	G
NC_040253.1	208335599	C	A
NC_040253.1	208831028	A	T
NC_040253.1	209021678	A	G
NC_040253.1	209076906	A	G
NC_040253.1	209200510	T	G
NC_040253.1	209497421	A	C
NC_040253.1	209517788	A	G
NC_040253.1	209564716	A	G
NC_040253.1	209738947	A	C
NC_040253.1	210075415	C	T
NC_040253.1	210178983	G	A
NC_040253.1	210467555	A	C
NC_040253.1	210932004	A	G
NC_040253.1	211052118	G	T
NC_040253.1	211093937	G	C
NC_040253.1	211270144	C	T
NC_040253.1	211372113	G	C
NC_040253.1	211471556	C	T
NC_040253.1	211717898	G	A
NC_040253.1	211751014	A	G
NC_040253.1	211860767	T	C
NC_040253.1	212146034	C	G
NC_040253.1	212241501	T	C
NC_040253.1	212320099	G	A
NC_040253.1	212522045	A	G
NC_040253.1	212643384	T	C
NC_040253.1	212681532	A	G
NC_040253.1	212849074	A	G
NC_040253.1	212892289	A	G
NC_040253.1	212922048	G	T
NC_040253.1	213090242	A	G
NC_040253.1	213114415	T	C
NC_040253.1	213130865	T	C
NC_040253.1	213224102	T	C

NC_040253.1	213263117	G	C
NC_040253.1	213316988	A	G
NC_040253.1	213342188	T	A
NC_040253.1	213374616	T	C
NC_040253.1	213420090	A	G
NC_040253.1	213478748	T	A
NC_040253.1	213504559	G	C
NC_040253.1	213526153	T	C
NC_040253.1	213562814	C	T
NC_040253.1	213603866	A	G
NC_040253.1	213630724	A	G
NC_040253.1	213643494	A	G
NC_040253.1	213675712	A	C
NC_040253.1	213691271	G	A
NC_040253.1	213734075	T	C
NC_040253.1	213846379	C	T
NC_040253.1	213891900	C	T
NC_040253.1	213954578	A	G
NC_040253.1	214015959	G	A
NC_040253.1	214049588	G	C
NC_040253.1	214164617	A	G
NC_040253.1	214175512	C	G
NC_040253.1	214175867	C	T
NC_040253.1	214228007	T	C
NC_040253.1	214255078	T	G
NC_040253.1	214313688	C	T
NC_040253.1	214359934	C	G
NC_040253.1	214413720	T	C
NC_040253.1	214470288	A	T
NC_040253.1	214525305	G	A
NC_040253.1	214578480	C	T
NC_040253.1	214635990	G	A
NC_040253.1	214698937	T	A
NC_040253.1	214754057	T	C
NC_040253.1	214810683	C	A
NC_040253.1	214875723	C	A
NC_040253.1	214931123	T	C
NC_040253.1	214984388	A	G
NC_040253.1	215027807	C	T
NC_040253.1	215053660	T	C
NC_040253.1	215077037	G	A
NC_040253.1	215148842	A	T
NC_040253.1	215155756	A	T
NC_040253.1	215370177	C	T
NC_040253.1	215424827	T	C
NC_040253.1	215453450	T	C
NC_040253.1	215475107	A	G

NC_040253.1	215506219	G	T
NC_040253.1	215531825	T	C
NC_040253.1	215592459	A	G
NC_040253.1	215646558	C	G
NC_040253.1	215702811	A	G
NC_040253.1	215759879	T	C
NC_040253.1	215783942	T	G
NC_040253.1	215841270	G	A
NC_040253.1	215899508	T	C
NC_040253.1	215956610	G	A
NC_040253.1	216012618	T	C
NC_040253.1	216069900	C	T
NC_040253.1	216124800	C	G
NC_040253.1	216185725	A	G
NC_040253.1	216245963	A	G
NC_040253.1	216256442	A	G
NC_040253.1	216280692	C	T
NC_040253.1	216338157	A	C
NC_040253.1	216386890	A	G
NC_040253.1	216432610	G	A
NC_040253.1	216443767	T	C
NC_040253.1	216500490	G	T
NC_040253.1	216533864	A	G
NC_040253.1	216583614	A	G
NC_040253.1	216641902	T	A
NC_040253.1	216697965	C	A
NC_040253.1	216753698	A	G
NC_040253.1	216766834	T	C
NC_040253.1	216778342	A	G
NC_040253.1	216799891	A	G
NC_040253.1	216834699	G	A
NC_040253.1	216835314	A	G
NC_040253.1	216843889	A	G
NC_040253.1	216858110	G	A
NC_040253.1	216913363	T	G
NC_040253.1	216961561	T	C
NC_040253.1	216989103	C	A
NC_040253.1	217001282	A	G
NC_040253.1	217045221	T	C
NC_040253.1	217092839	A	G
NC_040253.1	217120489	T	C
NC_040253.1	217165812	A	G
NC_040253.1	217201844	T	C
NC_040253.1	217202189	T	C
NC_040253.1	217257407	A	G
NC_040253.1	217287287	A	G
NC_040253.1	217302992	A	G

NC_040253.1	217321127	C	T
NC_040253.1	217322424	C	T
NC_040253.1	217326023	C	T
NC_040253.1	217326707	T	C
NC_040253.1	217370089	G	A
NC_040253.1	217516508	C	T
NC_040253.1	217520821	T	C
NC_040253.1	217559562	C	G
NC_040253.1	217595483	A	T
NC_040253.1	217649951	T	C
NC_040253.1	217676090	C	T
NC_040253.1	217698185	A	G
NC_040253.1	217735372	T	C
NC_040253.1	217747611	G	C
NC_040253.1	217747993	C	G
NC_040253.1	217788213	G	A
NC_040253.1	217838919	T	G
NC_040253.1	217862597	A	G
NC_040253.1	217874902	G	A
NC_040253.1	217875590	G	T
NC_040253.1	217912989	T	C
NC_040253.1	217950663	G	A
NC_040253.1	217964424	T	C
NC_040253.1	218021241	C	T
NC_040253.1	218033683	T	C
NC_040253.1	218070773	C	T
NC_040253.1	218114763	C	T
NC_040253.1	218119068	A	G
NC_040253.1	218158590	A	C
NC_040253.1	218170125	A	G
NC_040253.1	218234794	A	G
NC_040253.1	218237572	A	G
NC_040253.1	218250233	C	T
NC_040253.1	218307990	A	G
NC_040253.1	218362925	G	A
NC_040253.1	218408606	T	C
NC_040253.1	218409410	G	T
NC_040253.1	218410302	G	A
NC_040253.1	218410701	T	A
NC_040253.1	218448557	G	A
NC_040253.1	218502629	T	C
NC_040253.1	218559492	T	C
NC_040253.1	218579905	A	T
NC_040253.1	218636681	G	C
NC_040253.1	218652734	G	A
NC_040253.1	218711947	T	C
NC_040253.1	218767326	G	T

NC_040253.1	218823930	G	A
NC_040253.1	218854675	G	C
NC_040253.1	218920811	C	T
NC_040253.1	219037627	T	C
NC_040253.1	219049162	A	G
NC_040253.1	219066491	T	C
NC_040253.1	219125244	C	T
NC_040253.1	219165410	C	A
NC_040253.1	219197807	A	G
NC_040253.1	219198812	C	T
NC_040253.1	219199470	G	A
NC_040253.1	219234644	C	T
NC_040253.1	219285441	T	A
NC_040253.1	219308771	C	T
NC_040253.1	219320513	T	C
NC_040253.1	219373157	A	G
NC_040253.1	219422077	G	A
NC_040253.1	219483964	A	G
NC_040253.1	219534321	G	A
NC_040253.1	219560514	A	G
NC_040253.1	219560993	A	G
NC_040253.1	219567623	C	T
NC_040253.1	219576487	G	A
NC_040253.1	219629720	C	T
NC_040253.1	219663461	A	G
NC_040253.1	219714698	T	C
NC_040253.1	219772535	G	T
NC_040253.1	219825343	T	C
NC_040253.1	219854204	T	C
NC_040253.1	219912409	G	A
NC_040253.1	219966447	G	T
NC_040253.1	220021743	C	A
NC_040253.1	220063653	T	G
NC_040253.1	220083065	G	A
NC_040253.1	220137202	A	G
NC_040253.1	220194387	A	G
NC_040253.1	220251037	T	G
NC_040253.1	220303660	T	C
NC_040253.1	220339801	T	C
NC_040253.1	220387403	G	A
NC_040253.1	220394721	T	C
NC_040253.1	220454751	G	A
NC_040253.1	220512835	A	C
NC_040253.1	220579850	A	C
NC_040253.1	220634236	G	A
NC_040253.1	220688623	C	T
NC_040253.1	220719427	T	G

NC_040253.1	220756613	A	G
NC_040253.1	220781727	C	T
NC_040253.1	220837837	C	T
NC_040253.1	220888970	T	C
NC_040253.1	220914384	A	G
NC_040253.1	220947414	A	G
NC_040253.1	221002629	T	C
NC_040253.1	221058452	C	T
NC_040253.1	221115882	G	T
NC_040253.1	221167905	T	C
NC_040253.1	221234050	A	G
NC_040253.1	221281545	G	A
NC_040253.1	221285243	C	G
NC_040253.1	221338570	G	A
NC_040253.1	221389329	G	A
NC_040253.1	221435796	C	T
NC_040253.1	221494137	G	A
NC_040253.1	221547268	A	G
NC_040253.1	221618101	C	T
NC_040253.1	221652764	G	A
NC_040253.1	221671702	A	T
NC_040253.1	221722938	T	C
NC_040253.1	221756286	T	C
NC_040253.1	221812968	A	C
NC_040253.1	221877917	T	A
NC_040253.1	221936717	A	G
NC_040253.1	221987946	T	C
NC_040253.1	222020297	A	G
NC_040253.1	222054313	G	T
NC_040253.1	222056278	A	C
NC_040253.1	222069808	A	G
NC_040253.1	222130798	G	T
NC_040253.1	222187384	G	C
NC_040253.1	222253497	T	A
NC_040253.1	222282763	G	C
NC_040253.1	222338907	A	G
NC_040253.1	222391314	T	C
NC_040253.1	222420999	G	A
NC_040253.1	222470227	G	A
NC_040253.1	222525523	A	G
NC_040253.1	222526684	A	G
NC_040253.1	222527764	T	C
NC_040253.1	222529739	T	C
NC_040253.1	222530340	T	C
NC_040253.1	222531580	T	C
NC_040253.1	222532369	T	A
NC_040253.1	222593256	T	C

NC_040253.1	222630842	C	G
NC_040253.1	222650569	G	A
NC_040253.1	222688475	T	C
NC_040253.1	222694289	T	C
NC_040253.1	222725802	C	G
NC_040253.1	222751302	T	G
NC_040253.1	222808469	T	C
NC_040253.1	222824245	G	A
NC_040253.1	222852869	T	G
NC_040253.1	222907074	C	A
NC_040253.1	222969926	T	C
NC_040253.1	222978136	C	T
NC_040253.1	222987100	A	G
NC_040253.1	223045472	A	G
NC_040253.1	223104227	T	C
NC_040253.1	223168157	C	T
NC_040253.1	223185807	G	A
NC_040253.1	223238749	C	T
NC_040253.1	223281536	T	C
NC_040253.1	223284989	A	T
NC_040253.1	223343435	T	G
NC_040253.1	223382548	T	C
NC_040253.1	223441018	G	A
NC_040253.1	223497289	T	C
NC_040253.1	223551740	A	G
NC_040253.1	223613724	G	C
NC_040253.1	223671502	T	C
NC_040253.1	223731309	T	C
NC_040253.1	223785520	C	T
NC_040253.1	223816857	G	A
NC_040253.1	223820461	A	G
NC_040253.1	223829868	T	C
NC_040253.1	223831259	A	G
NC_040253.1	223838186	A	C
NC_040253.1	223893147	T	C
NC_040253.1	223941170	A	G
NC_040253.1	223950378	C	T
NC_040253.1	223957279	A	G
NC_040253.1	223959506	C	G
NC_040253.1	223960964	A	G
NC_040253.1	223961922	T	C
NC_040253.1	224021104	A	G
NC_040253.1	224021796	T	G
NC_040253.1	224022267	T	C
NC_040253.1	224022794	G	A
NC_040253.1	224023212	G	T
NC_040253.1	224024512	A	C

NC_040253.1	224080706	C	T
NC_040253.1	224137505	C	T
NC_040253.1	224195037	T	C
NC_040253.1	224233334	G	C
NC_040253.1	224274111	G	T
NC_040253.1	224312770	T	G
NC_040253.1	224327700	G	A
NC_040253.1	224349864	A	G
NC_040253.1	224449921	T	C
NC_040253.1	224494144	A	G
NC_040253.1	224508758	A	C
NC_040253.1	224528237	C	T
NC_040253.1	224559112	C	T
NC_040253.1	224615784	A	T
NC_040253.1	224662428	G	A
NC_040253.1	224708276	T	C
NC_040253.1	224756247	A	G
NC_040253.1	224787721	A	G
NC_040253.1	224812911	T	C
NC_040253.1	224918363	C	T
NC_040253.1	224976092	C	A
NC_040253.1	225029387	C	T
NC_040253.1	225091101	T	C
NC_040253.1	225268769	G	A
NC_040253.1	225325645	G	A
NC_040253.1	225379737	C	G
NC_040253.1	225436978	G	C
NC_040253.1	225550095	A	C
NC_040253.1	225604351	C	T
NC_040253.1	225620464	A	G
NC_040253.1	225660835	G	A
NC_040253.1	225661352	T	C
NC_040253.1	225662534	A	G
NC_040253.1	225663328	C	T
NC_040253.1	225663532	T	C
NC_040253.1	225663865	T	C
NC_040253.1	225664018	C	G
NC_040253.1	225664209	A	G
NC_040253.1	225695751	G	A
NC_040253.1	225736660	T	C
NC_040253.1	225770403	T	C
NC_040253.1	225825928	T	A
NC_040253.1	225882603	A	T
NC_040253.1	225910063	A	T
NC_040253.1	225944097	T	C
NC_040253.1	225944511	G	A
NC_040253.1	225945129	0	A

NC_040253.1	225945819	G	A
NC_040253.1	225946369	G	A
NC_040253.1	225946532	T	C
NC_040253.1	225946686	T	C
NC_040253.1	225971618	G	C
NC_040253.1	225977553	G	A
NC_040253.1	226029882	T	C
NC_040253.1	226082516	T	C
NC_040253.1	226083385	C	T
NC_040253.1	226119935	T	C
NC_040253.1	226152255	A	G
NC_040253.1	226162660	C	T
NC_040253.1	226213714	C	A
NC_040253.1	226254268	A	G
NC_040253.1	226266749	A	G
NC_040253.1	226314692	G	A
NC_040253.1	226346381	C	T
NC_040253.1	226372537	A	G
NC_040253.1	226420093	A	G
NC_040253.1	226427264	A	T
NC_040253.1	226474303	T	C
NC_040253.1	226501063	T	C
NC_040253.1	226533897	G	T
NC_040253.1	226567456	C	T
NC_040253.1	226606194	T	A
NC_040253.1	226652298	A	C
NC_040253.1	226692581	G	A
NC_040253.1	226704128	G	A
NC_040253.1	226757202	T	C
NC_040253.1	226793093	G	C
NC_040253.1	226810438	C	T
NC_040253.1	226863698	C	A
NC_040253.1	226913010	A	G
NC_040253.1	226925483	C	T
NC_040253.1	226969080	G	A
NC_040253.1	226994832	0	T
NC_040253.1	227026957	T	C
NC_040253.1	227080694	A	G
NC_040253.1	227133186	A	G
NC_040253.1	227169620	T	G
NC_040253.1	227229438	T	C
NC_040253.1	227274857	T	C
NC_040253.1	227287752	A	C
NC_040253.1	227352038	C	G
NC_040253.1	227399465	C	A
NC_040253.1	227401753	A	G
NC_040253.1	227405609	A	G

NC_040253.1	227460201	T	C
NC_040253.1	227517941	G	A
NC_040253.1	227572495	A	G
NC_040253.1	227625928	T	C
NC_040253.1	227672647	C	T
NC_040253.1	227713696	G	A
NC_040253.1	227853666	T	C
NC_040253.1	227925644	T	C
NC_040253.1	227980068	A	G
NC_040253.1	228044060	T	C
NC_040253.1	228094135	C	T
NC_040253.1	228101415	A	G
NC_040253.1	228153149	C	T
NC_040253.1	228211194	G	A
NC_040253.1	228264301	G	A
NC_040253.1	228321338	A	G
NC_040253.1	228383008	T	C
NC_040253.1	228455670	G	C
NC_040253.1	228515524	T	C
NC_040253.1	228579324	C	T
NC_040253.1	228634119	A	G
NC_040253.1	228659620	A	G
NC_040253.1	228688106	A	G
NC_040253.1	228753830	A	G
NC_040253.1	228808649	A	G
NC_040253.1	228834414	C	T
NC_040253.1	228889296	T	C
NC_040253.1	228945836	A	G
NC_040253.1	229001691	G	A
NC_040253.1	229059085	T	C
NC_040253.1	229082107	C	T
NC_040253.1	229129788	G	A
NC_040253.1	229138491	C	T
NC_040253.1	229194858	G	A
NC_040253.1	229254133	C	G
NC_040253.1	229310768	C	T
NC_040253.1	229352601	G	A
NC_040253.1	229353303	T	A
NC_040253.1	229356196	A	C
NC_040253.1	229357429	C	T
NC_040253.1	229358160	T	C
NC_040253.1	229358325	T	A
NC_040253.1	229413992	T	C
NC_040253.1	229470944	C	T
NC_040253.1	229529233	A	G
NC_040253.1	229585756	A	G
NC_040253.1	229776719	G	A

NC_040253.1	229821672	C	T
NC_040253.1	229877116	C	T
NC_040253.1	229944104	G	A
NC_040253.1	229982499	G	T
NC_040253.1	229993867	A	G
NC_040253.1	230038688	C	T
NC_040253.1	230074627	G	A
NC_040253.1	230095776	T	C
NC_040253.1	230148948	T	C
NC_040253.1	230196920	A	G
NC_040253.1	230222729	G	A
NC_040253.1	230259461	T	G
NC_040253.1	230316078	T	A
NC_040253.1	230319389	T	A
NC_040253.1	230375632	C	T
NC_040253.1	230456023	G	A
NC_040253.1	230677690	C	T
NC_040253.1	230725246	0	G
NC_040253.1	230730845	G	A
NC_040253.1	230799449	A	G
NC_040253.1	230855428	G	A
NC_040253.1	230885121	T	C
NC_040253.1	230909383	T	A
NC_040253.1	230969536	G	A
NC_040253.1	231038372	G	A
NC_040253.1	231102666	G	T
NC_040253.1	231146943	A	G
NC_040253.1	231173047	T	C
NC_040253.1	231203804	C	A
NC_040253.1	231261100	C	A
NC_040253.1	231297605	A	G
NC_040253.1	231354450	G	A
NC_040253.1	231408610	A	C
NC_040253.1	231462555	C	T
NC_040253.1	231516746	C	T
NC_040253.1	231565287	A	G
NC_040253.1	231629856	G	A
NC_040253.1	231671365	A	G
NC_040253.1	231679767	T	G
NC_040253.1	231709868	G	T
NC_040253.1	231750321	A	G
NC_040253.1	231804227	C	T
NC_040253.1	231856432	T	C
NC_040253.1	231900832	T	A
NC_040253.1	231908368	T	C
NC_040253.1	231956118	G	A
NC_040253.1	232016396	G	A

NC_040253.1	232050292	C	A
NC_040253.1	232060117	A	G
NC_040253.1	232118123	C	G
NC_040253.1	232176104	T	C
NC_040253.1	232232696	C	A
NC_040253.1	232287204	C	A
NC_040253.1	232349915	G	A
NC_040253.1	232396454	A	C
NC_040253.1	232425748	G	C
NC_040253.1	232452066	T	C
NC_040253.1	232507528	C	G
NC_040253.1	232536515	T	C
NC_040253.1	232643208	A	G
NC_040253.1	232698352	G	A
NC_040253.1	232765384	A	C
NC_040253.1	232766396	T	C
NC_040253.1	232801200	C	A
NC_040253.1	232823272	A	G
NC_040253.1	232876732	A	C
NC_040253.1	232917403	T	C
NC_040253.1	232921503	C	T
NC_040253.1	232938780	A	G
NC_040253.1	232993724	A	G
NC_040253.1	233014232	T	G
NC_040253.1	233068231	T	C
NC_040253.1	233125741	A	G
NC_040253.1	233173724	T	C
NC_040253.1	233237362	G	T
NC_040253.1	233278407	A	T
NC_040253.1	233330517	G	A
NC_040253.1	233392859	T	C
NC_040253.1	233448155	T	C
NC_040253.1	233462327	T	C
NC_040253.1	233480106	T	G
NC_040253.1	233512006	T	C
NC_040253.1	233540718	A	G
NC_040253.1	233588565	A	G
NC_040253.1	233645546	C	T
NC_040253.1	233701511	T	C
NC_040253.1	233755462	T	G
NC_040253.1	233812251	C	T
NC_040253.1	233874445	C	A
NC_040253.1	233934474	T	G
NC_040253.1	233989077	A	C
NC_040253.1	234046466	A	G
NC_040253.1	234100565	G	A
NC_040253.1	234157129	T	C

NC_040253.1	234211597	G	A
NC_040253.1	234263932	C	T
NC_040253.1	234286349	A	G
NC_040253.1	234329002	T	C
NC_040253.1	234342529	A	T
NC_040253.1	234395699	A	G
NC_040253.1	234456733	G	A
NC_040253.1	234499064	A	T
NC_040253.1	234532717	C	G
NC_040253.1	234555966	T	C
NC_040253.1	234610934	A	C
NC_040253.1	234664849	G	A
NC_040253.1	234698200	G	A
NC_040253.1	234730308	A	C
NC_040253.1	234731028	G	A
NC_040253.1	234732845	A	G
NC_040253.1	234779235	C	T
NC_040253.1	234779483	A	G
NC_040253.1	234779653	T	C
NC_040253.1	234780600	C	T
NC_040253.1	234834605	G	C
NC_040253.1	234891691	G	A
NC_040253.1	234939899	T	G
NC_040253.1	234982186	G	T
NC_040253.1	234982537	T	C
NC_040253.1	235016177	G	A
NC_040253.1	235016340	G	T
NC_040253.1	235071522	G	A
NC_040253.1	235113776	G	A
NC_040253.1	235116715	G	A
NC_040253.1	235116961	A	G
NC_040253.1	235117150	A	G
NC_040253.1	235117361	T	C
NC_040253.1	235123977	T	C
NC_040253.1	235174739	C	T
NC_040253.1	235189872	G	A
NC_040253.1	235190395	T	C
NC_040253.1	235190732	C	T
NC_040253.1	235190894	T	C
NC_040253.1	235200401	A	G
NC_040253.1	235227053	C	A
NC_040253.1	235234926	T	C
NC_040253.1	235254257	T	C
NC_040253.1	235257266	T	G
NC_040253.1	235262104	C	A
NC_040253.1	235265241	C	T
NC_040253.1	235268189	T	C

NC_040253.1	235268801	C	A
NC_040253.1	235269580	C	T
NC_040253.1	235271076	G	T
NC_040253.1	235271761	C	A
NC_040253.1	235272246	C	T
NC_040253.1	235294975	A	G
NC_040253.1	235297153	G	C
NC_040253.1	235351317	G	T
NC_040253.1	235351630	T	A
NC_040253.1	235352631	G	T
NC_040253.1	235354074	A	C
NC_040253.1	235407558	T	C
NC_040253.1	235409648	A	G
NC_040253.1	235423680	T	C
NC_040253.1	235424520	C	G
NC_040253.1	235428832	T	C
NC_040253.1	235430041	A	G
NC_040253.1	235430203	A	G
NC_040253.1	235435393	A	G
NC_040253.1	235445166	A	G
NC_040253.1	235448422	G	C
NC_040253.1	235451554	C	T
NC_040253.1	235457442	A	C
NC_040253.1	235481949	T	C
NC_040253.1	235488823	G	A
NC_040253.1	235489098	T	G
NC_040253.1	235527619	T	C
NC_040253.1	235528660	T	C
NC_040253.1	235529327	T	C
NC_040253.1	235587170	C	T
NC_040253.1	235615106	A	C
NC_040253.1	235616187	G	A
NC_040253.1	235622217	C	G
NC_040253.1	235622695	T	C
NC_040253.1	235630124	T	C
NC_040253.1	235659207	G	T
NC_040253.1	235660911	G	C
NC_040253.1	235724143	G	A
NC_040253.1	235724498	T	C
NC_040253.1	235770487	C	T
NC_040253.1	235782975	A	G
NC_040253.1	235785117	G	A
NC_040253.1	235791825	G	A
NC_040253.1	235792040	A	G
NC_040253.1	235794438	T	C
NC_040253.1	235807020	G	C
NC_040253.1	235822995	A	G

NC_040253.1	235876159	G	C
NC_040253.1	235929912	C	T
NC_040253.1	235930113	T	G
NC_040253.1	235931483	C	A
NC_040253.1	235932565	T	C
NC_040253.1	235938751	T	C
NC_040253.1	235945266	A	G
NC_040253.1	235947771	A	G
NC_040253.1	235972975	A	C
NC_040253.1	235975953	C	T
NC_040253.1	235980493	A	G
NC_040253.1	235982116	T	C
NC_040253.1	235983339	A	G
NC_040253.1	235985998	C	T
NC_040253.1	235988708	A	G
NC_040253.1	235993174	T	C
NC_040253.1	235993442	A	G
NC_040253.1	235996046	A	G
NC_040253.1	235999334	T	C
NC_040253.1	236000263	C	T
NC_040253.1	236000534	T	C
NC_040253.1	236000880	A	G
NC_040253.1	236002085	A	G
NC_040253.1	236002468	G	A
NC_040253.1	236003645	G	A
NC_040253.1	236008425	G	A
NC_040253.1	236009645	T	C
NC_040253.1	236011629	A	G
NC_040253.1	236014979	G	A
NC_040253.1	236042531	T	C
NC_040253.1	236062931	A	G
NC_040253.1	236119060	G	A
NC_040253.1	236159332	T	C
NC_040253.1	236182827	G	C
NC_040253.1	236183159	T	C
NC_040253.1	236186007	A	C
NC_040253.1	236186442	A	G
NC_040253.1	236186992	A	C
NC_040253.1	236189361	A	T
NC_040253.1	236203234	A	C
NC_040253.1	236203461	T	C
NC_040253.1	236219878	G	A
NC_040253.1	236220228	C	T
NC_040253.1	236220710	T	C
NC_040253.1	236221491	A	G
NC_040253.1	236228504	G	C
NC_040253.1	236229735	T	C

NC_040253.1	236250809	A	G
NC_040253.1	236251044	C	G
NC_040253.1	236267556	A	G
NC_040253.1	236276885	A	C
NC_040253.1	236284881	A	G
NC_040253.1	236294022	T	C
NC_040253.1	236303665	A	G
NC_040253.1	236306659	A	G
NC_040253.1	236306935	A	G
NC_040253.1	236307268	T	C
NC_040253.1	236311367	A	G
NC_040253.1	236326660	A	G
NC_040253.1	236340684	T	C
NC_040253.1	236364242	C	T
NC_040253.1	236366181	C	T
NC_040253.1	236413247	A	G
NC_040253.1	236471171	T	C
NC_040253.1	236527858	A	G
NC_040253.1	236582840	G	T
NC_040253.1	236638284	C	G
NC_040253.1	236695567	G	A
NC_040253.1	236748799	T	A
NC_040253.1	236801603	G	A
NC_040253.1	236833622	A	G
NC_040253.1	236857229	G	A
NC_040253.1	236913963	A	G
NC_040253.1	236955420	A	G
NC_040253.1	236971933	T	A
NC_040253.1	237027903	A	G
NC_040253.1	237088254	C	T
NC_040253.1	237141415	C	T
NC_040253.1	237197547	A	G
NC_040253.1	237258374	C	T
NC_040253.1	237316335	T	A
NC_040253.1	237371973	A	G
NC_040253.1	237405307	C	T
NC_040253.1	237428137	G	A
NC_040253.1	237481337	T	A
NC_040253.1	237539368	T	C
NC_040253.1	237604310	G	A
NC_040253.1	237660838	A	G
NC_040253.1	237707671	A	G
NC_040253.1	237754759	C	T
NC_040253.1	237782221	C	T
NC_040253.1	237947365	A	G
NC_040253.1	238000811	C	T
NC_040253.1	238053665	G	A

NC_040253.1	238108119	C	A
NC_040253.1	238140491	G	T
NC_040253.1	238180247	A	C
NC_040253.1	238188175	T	C
NC_040253.1	238250353	T	C
NC_040253.1	238306009	G	A
NC_040253.1	238360121	C	A
NC_040253.1	238418801	G	A
NC_040253.1	238469053	C	G
NC_040253.1	238494032	T	G
NC_040253.1	238548094	C	T
NC_040253.1	238597559	G	A
NC_040253.1	238642141	T	A
NC_040253.1	238699461	T	C
NC_040253.1	238813640	T	C
NC_040253.1	238870270	C	G
NC_040253.1	238921019	G	T
NC_040253.1	238921797	T	G
NC_040253.1	238982422	T	C
NC_040253.1	239026527	G	A
NC_040253.1	239039219	T	C
NC_040253.1	239105624	G	A
NC_040253.1	239158966	T	G
NC_040253.1	239213939	A	G
NC_040253.1	239272494	G	A
NC_040253.1	239338878	G	A
NC_040253.1	239396347	A	G
NC_040253.1	239431572	G	C
NC_040253.1	239472938	A	T
NC_040253.1	239514015	C	T
NC_040253.1	239535555	T	C
NC_040253.1	239592202	C	A
NC_040253.1	239656884	A	G
NC_040253.1	239712560	C	T
NC_040253.1	239712726	C	G
NC_040253.1	239715772	A	G
NC_040253.1	239766768	C	T
NC_040253.1	239775040	G	A
NC_040253.1	239833700	T	C
NC_040253.1	239886901	A	C
NC_040253.1	239936439	G	T
NC_040253.1	239991496	G	A
NC_040253.1	240022021	C	T
NC_040253.1	240061058	A	C
NC_040253.1	240072075	A	G
NC_040253.1	240101998	A	G
NC_040253.1	240128101	T	G

NC_040253.1	240179612	C	T
NC_040253.1	240179914	T	C
NC_040253.1	240181814	A	T
NC_040253.1	240231443	G	A
NC_040253.1	240292256	A	G
NC_040253.1	240346715	T	C
NC_040253.1	240402293	C	T
NC_040253.1	240457222	G	A
NC_040253.1	240493810	C	T
NC_040253.1	240537244	A	G
NC_040253.1	240550029	G	A
NC_040253.1	240604364	G	A
NC_040253.1	240660009	C	T
NC_040253.1	240716240	T	C
NC_040253.1	240775600	C	T
NC_040253.1	240826400	C	T
NC_040253.1	240890590	T	C
NC_040253.1	240942924	T	C
NC_040253.1	240943326	T	C
NC_040253.1	240998376	A	G
NC_040253.1	241055876	A	G
NC_040253.1	241093532	G	A
NC_040253.1	241111207	C	T
NC_040253.1	241127687	G	C
NC_040253.1	241184343	G	A
NC_040253.1	241195836	G	T
NC_040253.1	241249947	C	T
NC_040253.1	241299343	A	G
NC_040253.1	241352524	A	G
NC_040253.1	241412442	C	A
NC_040253.1	241452423	C	T
NC_040253.1	241495286	G	A
NC_040253.1	241549488	T	A
NC_040253.1	241606162	C	T
NC_040253.1	241631138	C	T
NC_040253.1	241631477	A	C
NC_040253.1	241644310	G	A
NC_040253.1	241697843	T	G
NC_040253.1	241725519	T	G
NC_040253.1	241783748	C	T
NC_040253.1	241828558	A	G
NC_040253.1	241881205	A	G
NC_040253.1	241929586	C	T
NC_040253.1	241945847	C	A
NC_040253.1	241995523	A	G
NC_040253.1	242058226	C	T
NC_040253.1	242074595	A	G

NC_040253.1	242104145	C	A
NC_040253.1	242159793	T	A
NC_040253.1	242191831	T	C
NC_040253.1	242231143	T	C
NC_040253.1	242283480	G	C
NC_040253.1	242338484	C	T
NC_040253.1	242389524	T	C
NC_040253.1	242444280	A	G
NC_040253.1	242498892	A	G
NC_040253.1	242553363	C	T
NC_040253.1	242586952	T	C
NC_040253.1	242630833	A	T
NC_040253.1	242670821	C	T
NC_040253.1	242680174	G	A
NC_040253.1	242736001	G	C
NC_040253.1	242791008	A	C
NC_040253.1	242813715	A	G
NC_040253.1	242873274	C	T
NC_040253.1	242914252	C	T
NC_040253.1	242935362	G	A
NC_040253.1	242992851	A	C
NC_040253.1	243042434	A	G
NC_040253.1	243099107	G	A
NC_040253.1	243154829	C	T
NC_040253.1	243208166	T	G
NC_040253.1	243246345	A	G
NC_040253.1	243335512	T	A
NC_040253.1	243407863	G	T
NC_040253.1	243466273	T	C
NC_040253.1	243521874	G	T
NC_040253.1	243575225	C	T
NC_040253.1	243604593	C	T
NC_040253.1	243636379	G	T
NC_040253.1	243690386	A	G
NC_040253.1	243745078	T	C
NC_040253.1	243803336	G	A
NC_040253.1	243856825	A	G
NC_040253.1	243892120	G	A
NC_040253.1	243908813	A	G
NC_040253.1	243965410	A	G
NC_040253.1	244002576	T	C
NC_040253.1	244018859	G	A
NC_040253.1	244072302	C	T
NC_040253.1	244124852	C	A
NC_040253.1	244182046	A	T
NC_040253.1	244237552	A	G
NC_040253.1	244237789	G	A

NC_040253.1	244239251	A	G
NC_040253.1	244239539	A	G
NC_040253.1	244240320	T	G
NC_040253.1	244298021	T	C
NC_040253.1	244298979	T	G
NC_040253.1	244299144	T	C
NC_040253.1	244299842	A	G
NC_040253.1	244300448	A	G
NC_040253.1	244300946	A	G
NC_040253.1	244355803	G	A
NC_040253.1	244409126	A	G
NC_040253.1	244460738	C	G
NC_040253.1	244489636	A	G
NC_040253.1	244513618	T	C
NC_040253.1	244562944	A	T
NC_040253.1	244618323	G	A
NC_040253.1	244676435	C	T
NC_040253.1	244740407	A	G
NC_040253.1	244779936	C	A
NC_040253.1	244783209	T	G
NC_040253.1	244783374	A	G
NC_040253.1	244784190	A	G
NC_040253.1	244808865	A	G
NC_040253.1	244823218	A	G
NC_040253.1	244883810	T	C
NC_040253.1	244925907	C	A
NC_040253.1	244954580	C	T
NC_040253.1	244982052	A	G
NC_040253.1	245001183	T	C
NC_040253.1	245004560	G	A
NC_040253.1	245030073	C	T
NC_040253.1	245177991	C	T
NC_040253.1	245180287	C	T
NC_040253.1	245182274	T	C
NC_040253.1	245197224	T	C
NC_040253.1	245381787	G	A
NC_040253.1	245444644	A	C
NC_040253.1	245505331	T	C
NC_040253.1	245570613	A	G
NC_040253.1	245600485	G	A
NC_040253.1	245638045	T	C
NC_040253.1	245656621	A	G
NC_040253.1	245739470	A	G
NC_040253.1	245752225	A	G
NC_040253.1	245807847	G	A
NC_040253.1	245861580	A	G
NC_040253.1	245900300	T	C

NC_040253.1	245986059	A	G
NC_040253.1	246020296	C	T
NC_040253.1	246021341	C	T
NC_040253.1	246151032	C	T
NC_040253.1	246185979	C	T
NC_040253.1	246206759	T	C
NC_040253.1	246251662	G	A
NC_040253.1	246264954	T	C
NC_040253.1	246314709	G	A
NC_040253.1	246367345	C	T
NC_040253.1	246422597	C	T
NC_040253.1	246478345	T	G
NC_040253.1	246534875	A	G
NC_040253.1	246567061	G	C
NC_040253.1	246604244	A	G
NC_040253.1	246659252	T	C
NC_040253.1	246716645	C	T
NC_040253.1	246817147	A	C
NC_040253.1	246874743	G	C
NC_040253.1	246933056	T	A
NC_040253.1	246988511	T	C
NC_040253.1	247038014	G	A
NC_040253.1	247069952	G	A
NC_040253.1	247090580	C	T
NC_040253.1	247135088	G	A
NC_040253.1	247168608	G	C
NC_040253.1	247224331	T	C
NC_040253.1	247277953	G	A
NC_040253.1	247335319	A	G
NC_040253.1	247393915	C	A
NC_040253.1	247430355	C	T
NC_040253.1	247430656	G	C
NC_040253.1	247430818	G	A
NC_040253.1	247430979	C	T
NC_040253.1	247431207	T	C
NC_040253.1	247455572	A	T
NC_040253.1	247468049	A	G
NC_040253.1	247520646	A	G
NC_040253.1	247576531	G	A
NC_040253.1	247627705	G	C
NC_040253.1	247695925	A	G
NC_040253.1	247696638	T	C
NC_040253.1	247708109	G	C
NC_040253.1	247758264	G	A
NC_040253.1	247777540	A	G
NC_040253.1	247789902	C	T
NC_040253.1	247790140	T	C

NC_040253.1	247790804	C	T
NC_040253.1	247791170	T	A
NC_040253.1	247791406	C	T
NC_040253.1	247791822	T	C
NC_040253.1	247803192	T	C
NC_040253.1	247856360	A	T
NC_040253.1	247907847	A	G
NC_040253.1	247963392	A	T
NC_040253.1	248009510	A	G
NC_040253.1	248055557	G	A
NC_040253.1	248085550	T	C
NC_040253.1	248140997	C	T
NC_040253.1	248201686	G	A
NC_040253.1	248257855	T	G
NC_040253.1	248303165	A	G
NC_040253.1	248338243	T	C
NC_040253.1	248338470	T	C
NC_040253.1	248392883	T	C
NC_040253.1	248434282	T	C
NC_040253.1	248468597	C	G
NC_040253.1	248475395	C	T
NC_040253.1	248476126	A	G
NC_040253.1	248476593	G	C
NC_040253.1	248494766	G	A
NC_040253.1	248515715	A	G
NC_040253.1	248528515	T	C
NC_040253.1	248528702	C	T
NC_040253.1	248528873	A	G
NC_040253.1	248590510	G	C
NC_040253.1	248640062	T	G
NC_040253.1	248694623	G	A
NC_040253.1	248749256	G	T
NC_040253.1	248762734	A	C
NC_040253.1	248808083	C	T
NC_040253.1	248808844	T	C
NC_040253.1	248859778	C	T
NC_040253.1	248916132	T	C
NC_040253.1	248916854	T	C
NC_040253.1	248917258	C	T
NC_040253.1	248956574	G	T
NC_040253.1	248957260	T	C
NC_040253.1	249010987	A	G
NC_040253.1	249039923	A	T
NC_040253.1	249058249	T	C
NC_040253.1	249061120	T	A
NC_040253.1	249118104	T	C
NC_040253.1	249137438	A	G

NC_040253.1	249138123	G	A
NC_040253.1	249199126	T	G
NC_040253.1	249199379	T	C
NC_040253.1	249200005	A	G
NC_040253.1	249200910	C	T
NC_040253.1	249201146	T	C
NC_040253.1	249251722	A	G
NC_040253.1	249302670	T	C
NC_040253.1	249357579	A	C
NC_040253.1	249412232	C	A
NC_040253.1	249469531	T	C
NC_040253.1	249522278	G	A
NC_040253.1	249566851	G	A
NC_040253.1	249591670	T	C
NC_040253.1	249593725	T	C
NC_040253.1	249593937	A	G
NC_040253.1	249644789	C	G
NC_040253.1	249655232	G	C
NC_040253.1	249695662	A	G
NC_040253.1	249720194	C	A
NC_040253.1	249727278	T	C
NC_040253.1	249766726	T	C
NC_040253.1	249767351	T	C
NC_040253.1	249767516	G	C
NC_040253.1	249785989	T	C
NC_040253.1	249815738	T	G
NC_040253.1	249868341	G	A
NC_040253.1	249921027	G	A
NC_040253.1	249945549	T	C
NC_040253.1	249987632	G	C
NC_040253.1	249997370	G	C
NC_040253.1	250004798	C	T
NC_040253.1	250031642	G	T
NC_040253.1	250085775	A	G
NC_040253.1	250147231	A	C
NC_040253.1	250148747	A	C
NC_040253.1	250169896	A	C
NC_040253.1	250209181	G	A
NC_040253.1	250263610	A	G
NC_040253.1	250282763	A	G
NC_040253.1	250325862	T	C
NC_040253.1	250388149	T	C
NC_040253.1	250425095	G	A
NC_040253.1	250429956	A	G
NC_040253.1	250430346	A	G
NC_040253.1	250430510	G	A
NC_040253.1	250430846	A	G

NC_040253.1	250431234	C	T
NC_040253.1	250455881	G	T
NC_040253.1	250495874	A	G
NC_040253.1	250496054	C	A
NC_040253.1	250496227	T	C
NC_040253.1	250497077	A	G
NC_040253.1	250497927	T	C
NC_040253.1	250498368	C	G
NC_040253.1	250498696	T	C
NC_040253.1	250503930	A	G
NC_040253.1	250504560	G	A
NC_040253.1	250523350	T	G
NC_040253.1	250574206	C	T
NC_040253.1	250616202	G	A
NC_040253.1	250638183	G	A
NC_040253.1	250640604	A	G
NC_040253.1	250648846	T	C
NC_040253.1	250696357	A	C
NC_040253.1	250756498	T	C
NC_040253.1	250810117	G	A
NC_040253.1	250831397	T	C
NC_040253.1	250832171	T	G
NC_040253.1	250834430	G	A
NC_040253.1	250862179	A	G
NC_040253.1	250890728	T	C
NC_040253.1	250898681	G	A
NC_040253.1	250899126	A	G
NC_040253.1	250918293	G	A
NC_040253.1	250919188	C	T
NC_040253.1	250944578	A	G
NC_040253.1	250949672	A	G
NC_040253.1	250960904	G	C
NC_040253.1	250961082	G	C
NC_040253.1	250962572	T	C
NC_040253.1	250964275	G	A
NC_040253.1	250970403	C	T
NC_040253.1	250970586	T	C
NC_040253.1	250972325	A	G
NC_040253.1	250978997	G	T
NC_040253.1	250979819	G	C
NC_040253.1	250984697	C	T
NC_040253.1	251044129	G	C
NC_040253.1	251052795	T	C
NC_040253.1	251053844	G	A
NC_040253.1	251054060	C	T
NC_040253.1	251099226	T	G
NC_040253.1	251155390	G	A

NC_040253.1	251193900	G	A
NC_040253.1	251247373	C	T
NC_040253.1	251291720	G	A
NC_040253.1	251338396	T	C
NC_040253.1	251363342	T	C
NC_040253.1	251391814	T	A
NC_040253.1	251394229	A	G
NC_040253.1	251394423	G	A
NC_040253.1	251425151	T	C
NC_040253.1	251442302	G	A
NC_040253.1	251453636	T	A
NC_040253.1	251463418	T	C
NC_040253.1	251484167	T	C
NC_040253.1	251527106	T	C
NC_040253.1	251569469	A	G
NC_040253.1	251615155	A	G
NC_040253.1	251622578	T	C
NC_040253.1	251671226	G	T
NC_040253.1	251717243	T	C
NC_040253.1	251739000	T	C
NC_040253.1	251747239	A	G
NC_040253.1	251765443	0	G
NC_040253.1	251811398	G	C
NC_040253.1	251848257	T	C
NC_040253.1	251933628	A	G
NC_040253.1	251983827	G	A
NC_040253.1	252026427	T	C
NC_040253.1	252048954	A	G
NC_040253.1	252056969	A	G
NC_040253.1	252114147	T	C
NC_040253.1	252116734	A	G
NC_040253.1	252117248	T	G
NC_040253.1	252117581	T	A
NC_040253.1	252117879	G	A
NC_040253.1	252118232	C	T
NC_040253.1	252175385	G	A
NC_040253.1	252230011	T	C
NC_040253.1	252254427	T	C
NC_040253.1	252273450	A	G
NC_040253.1	252275985	T	C
NC_040253.1	252332948	T	C
NC_040253.1	252389744	A	G
NC_040253.1	252442427	A	G
NC_040253.1	252500843	G	A
NC_040253.1	252555730	C	T
NC_040253.1	252608909	A	G
NC_040253.1	252666866	A	G

NC_040253.1	252727357	A	C
NC_040253.1	252785918	T	G
NC_040253.1	252833988	T	C
NC_040253.1	252843978	T	C
NC_040253.1	252898110	T	C
NC_040253.1	253037701	C	T
NC_040253.1	253091900	T	C
NC_040253.1	253142608	A	G
NC_040253.1	253176037	C	A
NC_040253.1	253200680	A	T
NC_040253.1	253240963	T	G
NC_040253.1	253282417	T	C
NC_040253.1	253310857	A	C
NC_040253.1	253312657	T	C
NC_040253.1	253351562	A	G
NC_040253.1	253370596	A	G
NC_040253.1	253427421	C	A
NC_040253.1	253483024	C	T
NC_040253.1	253538403	T	C
NC_040253.1	253564575	C	A
NC_040253.1	253599458	G	A
NC_040253.1	253655777	C	T
NC_040253.1	253710875	G	T
NC_040253.1	253738239	C	T
NC_040253.1	253754530	C	T
NC_040253.1	253801550	T	C
NC_040253.1	253818776	A	G
NC_040253.1	253849222	A	G
NC_040253.1	253865792	A	G
NC_040253.1	253875085	T	C
NC_040253.1	253875657	C	G
NC_040253.1	253890680	G	C
NC_040253.1	253893227	T	G
NC_040253.1	253899367	A	G
NC_040253.1	253900167	A	G
NC_040253.1	253900486	T	G
NC_040253.1	253902390	T	A
NC_040253.1	253904207	G	A
NC_040253.1	253904438	T	A
NC_040253.1	253948736	C	T
NC_040253.1	253949800	T	A
NC_040253.1	253966882	A	G
NC_040253.1	254021762	A	G
NC_040253.1	254053131	G	C
NC_040253.1	254083285	C	T
NC_040253.1	254109004	G	T
NC_040253.1	254131695	T	C

NC_040253.1	254134643	C	G
NC_040253.1	254154789	T	C
NC_040253.1	254157838	C	T
NC_040253.1	254191906	C	T
NC_040253.1	254234468	C	T
NC_040253.1	254256611	T	C
NC_040253.1	254257183	C	T
NC_040253.1	254257448	T	A
NC_040253.1	254262435	A	G
NC_040253.1	254277898	G	A
NC_040253.1	254278117	T	C
NC_040253.1	254278731	A	C
NC_040253.1	254279242	G	A
NC_040253.1	254305248	T	C
NC_040253.1	254306465	G	T
NC_040253.1	254307165	T	C
NC_040253.1	254307847	A	G
NC_040253.1	254310401	C	T
NC_040253.1	254364230	A	C
NC_040253.1	254375883	C	T
NC_040253.1	254402171	T	C
NC_040253.1	254405553	C	T
NC_040253.1	254437847	C	G
NC_040253.1	254439417	C	T
NC_040253.1	254447766	C	T
NC_040253.1	254460237	T	C
NC_040253.1	254505178	A	T
NC_040253.1	254522111	C	G
NC_040253.1	254580898	A	G
NC_040253.1	254585130	A	C
NC_040253.1	254622729	C	T
NC_040253.1	254622911	T	C
NC_040253.1	254623146	C	T
NC_040253.1	254625970	G	A
NC_040253.1	254628497	T	C
NC_040253.1	254628905	T	C
NC_040253.1	254639486	C	G
NC_040253.1	254639676	T	G
NC_040253.1	254672853	G	A
NC_040253.1	254732071	T	A
NC_040253.1	254792376	A	T
NC_040253.1	254846058	G	A
NC_040253.1	254900279	C	T
NC_040253.1	254901785	G	C
NC_040253.1	254903059	A	C
NC_040253.1	254903598	A	G
NC_040253.1	254945209	T	C

NC_040253.1	254961112	G	A
NC_040253.1	254972895	A	G
NC_040253.1	254989444	G	A
NC_040253.1	254989846	G	T
NC_040253.1	255042107	A	G
NC_040253.1	255042396	A	G
NC_040253.1	255052108	T	C
NC_040253.1	255063104	A	G
NC_040253.1	255063649	A	G
NC_040253.1	255064200	A	G
NC_040253.1	255065651	G	T
NC_040253.1	255074793	A	G
NC_040253.1	255100656	T	C
NC_040253.1	255116525	C	T
NC_040253.1	255116709	C	T
NC_040253.1	255174347	T	G
NC_040253.1	255196918	A	G
NC_040253.1	255203130	A	G
NC_040253.1	255203962	A	G
NC_040253.1	255205750	G	T
NC_040253.1	255206328	T	C
NC_040253.1	255211571	G	A
NC_040253.1	255215335	T	C
NC_040253.1	255267802	A	G
NC_040253.1	255274042	A	C
NC_040253.1	255274767	T	C
NC_040253.1	255276390	T	C
NC_040253.1	255288419	T	C
NC_040253.1	255323070	G	C
NC_040253.1	255388071	A	G
NC_040253.1	255401936	T	C
NC_040253.1	255416444	T	C
NC_040253.1	255420442	A	G
NC_040253.1	255426358	T	C
NC_040253.1	255426524	T	C
NC_040253.1	255427040	G	A
NC_040253.1	255435914	T	C
NC_040253.1	255444534	T	G
NC_040253.1	255446229	T	C
NC_040253.1	255453526	A	G
NC_040253.1	255453832	G	C
NC_040253.1	255454053	A	G
NC_040253.1	255477663	A	G
NC_040253.1	255477903	T	C
NC_040253.1	255478356	A	G
NC_040253.1	255479754	T	C
NC_040253.1	255486186	C	T

NC_040253.1	255489647	G	A
NC_040253.1	255548911	T	A
NC_040253.1	255557187	G	A
NC_040253.1	255611146	G	T
NC_040253.1	255611855	T	G
NC_040253.1	255634637	C	T
NC_040253.1	255656427	A	G
NC_040253.1	255663409	T	C
NC_040253.1	255708994	A	C
NC_040253.1	255734981	G	C
NC_040253.1	255740952	G	C
NC_040253.1	255746341	G	A
NC_040253.1	255792366	G	C
NC_040253.1	255823714	A	G
NC_040253.1	255824068	A	G
NC_040253.1	255825366	T	C
NC_040253.1	255862901	T	C
NC_040253.1	255871052	A	G
NC_040253.1	255871647	T	C
NC_040253.1	255871968	A	G
NC_040253.1	255873121	A	G
NC_040253.1	255915838	C	T
NC_040253.1	255934886	C	T
NC_040253.1	255935039	T	C
NC_040253.1	255936390	A	G
NC_040253.1	255941304	T	C
NC_040253.1	255943019	T	C
NC_040253.1	255996828	A	C
NC_040253.1	256053303	T	G
NC_040253.1	256100488	C	G
NC_040253.1	256105497	G	A
NC_040253.1	256159709	C	A
NC_040253.1	256188672	G	A
NC_040253.1	256221998	A	G
NC_040253.1	256284782	A	G
NC_040253.1	256285719	T	C
NC_040253.1	256290421	C	T
NC_040253.1	256304221	C	G
NC_040253.1	256304818	G	A
NC_040253.1	256305157	C	A
NC_040253.1	256305329	C	T
NC_040253.1	256318390	G	A
NC_040253.1	256344796	A	G
NC_040253.1	256357462	T	C
NC_040253.1	256366105	G	C
NC_040253.1	256382052	A	C
NC_040253.1	256394969	T	C

NC_040253.1	256425010	A	G
NC_040253.1	256442265	A	G
NC_040253.1	256451139	T	C
NC_040253.1	256467208	G	C
NC_040253.1	256522514	A	G
NC_040253.1	256555022	G	A
NC_040253.1	256558843	A	C
NC_040253.1	256568432	T	G
NC_040253.1	256584678	T	C
NC_040253.1	256584836	G	A
NC_040253.1	256587529	A	C
NC_040253.1	256627912	A	C
NC_040253.1	256689539	T	C
NC_040253.1	256733411	A	C
NC_040253.1	256760594	A	G
NC_040253.1	256775062	A	G
NC_040253.1	256775364	A	T
NC_040253.1	256783476	A	G
NC_040253.1	256842024	A	G
NC_040253.1	256894069	T	C
NC_040253.1	256950767	G	T
NC_040253.1	256977229	T	G
NC_040253.1	257030676	T	C
NC_040253.1	257075825	T	C
NC_040253.1	257076470	G	A
NC_040253.1	257076890	A	G
NC_040253.1	257092127	G	A
NC_040253.1	257119259	T	C
NC_040253.1	257126655	C	T
NC_040253.1	257174119	C	T
NC_040253.1	257241632	T	C
NC_040253.1	257294955	T	C
NC_040253.1	257346055	G	A
NC_040253.1	257400481	T	G
NC_040253.1	257450746	C	T
NC_040253.1	257507305	C	T
NC_040253.1	257549425	A	G
NC_040253.1	257583030	A	G
NC_040253.1	257599872	T	C
NC_040253.1	257655089	T	A
NC_040253.1	257701733	T	G
NC_040253.1	257733706	A	T
NC_040253.1	257789860	G	A
NC_040253.1	257807146	G	A
NC_040253.1	257819623	C	T
NC_040253.1	257871971	G	A
NC_040253.1	257917088	T	C

NC_040253.1	257938270	A	G
NC_040253.1	257938543	A	G
NC_040253.1	257965890	A	C
NC_040253.1	257969906	C	A
NC_040253.1	257989544	C	T
NC_040253.1	257990582	C	A
NC_040253.1	257991129	T	C
NC_040253.1	257992053	G	C
NC_040253.1	258018342	G	A
NC_040253.1	258058554	C	T
NC_040253.1	258076305	A	G
NC_040253.1	258131488	T	C
NC_040253.1	258188060	C	T
NC_040253.1	258238939	A	C
NC_040253.1	258282541	C	T
NC_040253.1	258282938	G	A
NC_040253.1	258284148	A	G
NC_040253.1	258335997	A	G
NC_040253.1	258349578	A	G
NC_040253.1	258374413	T	C
NC_040253.1	258386018	A	G
NC_040253.1	258435326	G	A
NC_040253.1	258474378	C	T
NC_040253.1	258482382	G	A
NC_040253.1	258483333	T	C
NC_040253.1	258483533	T	A
NC_040253.1	258531061	C	A
NC_040253.1	258535726	T	C
NC_040253.1	258582420	A	G
NC_040253.1	258582826	C	G
NC_040253.1	258628149	T	A
NC_040253.1	258647499	T	C
NC_040253.1	258651800	T	C
NC_040253.1	258652164	G	A
NC_040253.1	258652333	T	C
NC_040253.1	258676733	G	T
NC_040253.1	258677414	A	G
NC_040253.1	258679748	G	A
NC_040253.1	258682729	A	G
NC_040253.1	258727701	A	G
NC_040253.1	258782835	T	G
NC_040253.1	258834339	G	A
NC_040253.1	258855337	G	A
NC_040253.1	258865279	T	C
NC_040253.1	258874958	G	A
NC_040253.1	258875230	T	C
NC_040253.1	258875466	G	A

NC_040253.1	258923577	T	C
NC_040253.1	258950551	A	G
NC_040253.1	258961503	G	A
NC_040253.1	259010261	C	T
NC_040253.1	259012701	T	C
NC_040253.1	259015375	T	C
NC_040253.1	259017352	G	A
NC_040253.1	259017674	C	A
NC_040253.1	259073204	G	C
NC_040253.1	259130660	C	T
NC_040253.1	259183435	C	G
NC_040253.1	259233511	T	C
NC_040253.1	259275905	T	C
NC_040253.1	259297318	T	G
NC_040253.1	259299620	A	G
NC_040253.1	259302323	A	G
NC_040253.1	259302529	C	T
NC_040253.1	259303565	G	A
NC_040253.1	259305230	A	G
NC_040253.1	259339586	T	G
NC_040253.1	259363479	A	G
NC_040253.1	259396828	T	C
NC_040253.1	259410351	T	C
NC_040253.1	259432586	A	C
NC_040253.1	259468410	A	G
NC_040253.1	259524224	C	T
NC_040253.1	259579796	G	A
NC_040253.1	259635219	A	G
NC_040253.1	259678419	T	G
NC_040253.1	259709378	G	A
NC_040253.1	259713295	C	T
NC_040253.1	259755519	A	T
NC_040253.1	259777073	T	C
NC_040253.1	259832106	A	G
NC_040253.1	259860272	G	C
NC_040253.1	259899132	A	C
NC_040253.1	259954953	C	T
NC_040253.1	260015491	T	C
NC_040253.1	260073404	G	A
NC_040253.1	260134096	A	G
NC_040253.1	260188483	A	G
NC_040253.1	260243035	T	C
NC_040253.1	260243688	T	C
NC_040253.1	260269301	C	T
NC_040253.1	260269691	A	G
NC_040253.1	260269916	G	C
NC_040253.1	260314326	A	G

NC_040253.1	260316466	C	G
NC_040253.1	260345770	T	C
NC_040253.1	260379019	T	C
NC_040253.1	260379329	A	G
NC_040253.1	260427070	A	G
NC_040253.1	260442170	T	C
NC_040253.1	260444101	T	C
NC_040253.1	260452021	T	C
NC_040253.1	260461954	A	G
NC_040253.1	260467720	T	G
NC_040253.1	260482331	C	T
NC_040253.1	260483495	T	C
NC_040253.1	260495129	G	C
NC_040253.1	260495638	A	G
NC_040253.1	260496714	T	C
NC_040253.1	260496895	T	C
NC_040253.1	260501427	T	C
NC_040253.1	260502063	A	G
NC_040253.1	260503019	T	C
NC_040253.1	260504886	G	A
NC_040253.1	260535710	T	C
NC_040253.1	260566724	T	C
NC_040253.1	260619490	C	A
NC_040253.1	260684939	T	C
NC_040253.1	260697359	T	C
NC_040253.1	260754006	A	G
NC_040253.1	260805596	A	G
NC_040253.1	260860564	A	G
NC_040253.1	260913462	G	T
NC_040253.1	260974363	T	C
NC_040253.1	261029059	C	T
NC_040253.1	261081725	G	A
NC_040253.1	261115868	C	T
NC_040253.1	261161695	G	A
NC_040253.1	261169990	G	C
NC_040253.1	261224174	C	G
NC_040253.1	261268051	A	G
NC_040253.1	261310743	A	G
NC_040253.1	261353948	A	G
NC_040253.1	261408027	G	C
NC_040253.1	261421504	C	T
NC_040253.1	261443591	T	C
NC_040253.1	261443748	C	A
NC_040253.1	261462097	A	G
NC_040253.1	261462484	G	C
NC_040253.1	261462930	A	G
NC_040253.1	261463667	C	G

NC_040253.1	261480372	C	G
NC_040253.1	261483619	T	C
NC_040253.1	261514907	T	C
NC_040253.1	261518702	A	G
NC_040253.1	261542793	A	G
NC_040253.1	261553646	C	A
NC_040253.1	261591019	A	G
NC_040253.1	261626247	T	A
NC_040253.1	261627127	C	G
NC_040253.1	261627307	A	G
NC_040253.1	261628070	C	T
NC_040253.1	261628274	A	G
NC_040253.1	261670476	C	T
NC_040253.1	261726229	A	G
NC_040253.1	261779558	T	C
NC_040253.1	261819189	A	T
NC_040253.1	261832964	G	A
NC_040253.1	261889276	G	A
NC_040253.1	261954272	C	T
NC_040253.1	262006832	T	C
NC_040253.1	262011566	G	A
NC_040253.1	262016118	G	T
NC_040253.1	262062242	T	C
NC_040253.1	262104113	T	C
NC_040253.1	262134392	T	C
NC_040253.1	262159362	G	C
NC_040253.1	262161916	C	T
NC_040253.1	262200669	C	A
NC_040253.1	262208383	G	A
NC_040253.1	262258037	G	T
NC_040253.1	262292704	A	G
NC_040253.1	262306106	G	A
NC_040253.1	262309762	C	T
NC_040253.1	262316906	C	T
NC_040253.1	262320270	A	G
NC_040253.1	262377204	G	A
NC_040253.1	262437239	A	G
NC_040253.1	262475927	G	A
NC_040253.1	262494430	A	T
NC_040253.1	262538735	G	T
NC_040253.1	262540209	T	C
NC_040253.1	262541512	T	G
NC_040253.1	262592258	T	C
NC_040253.1	262642522	A	G
NC_040253.1	262672679	G	T
NC_040253.1	262695758	A	G
NC_040253.1	262756516	G	A

NC_040253.1	262762437	A	G
NC_040253.1	262818864	A	G
NC_040253.1	262849639	G	C
NC_040253.1	262859619	A	C
NC_040253.1	262894648	T	C
NC_040253.1	262916128	A	C
NC_040253.1	262916674	G	A
NC_040253.1	262918684	T	C
NC_040253.1	262931696	G	A
NC_040253.1	262937669	A	G
NC_040253.1	262978771	T	C
NC_040253.1	262982316	A	G
NC_040253.1	262994180	T	C
NC_040253.1	263015349	T	C
NC_040253.1	263018882	T	C
NC_040253.1	263024669	A	G
NC_040253.1	263046973	G	C
NC_040253.1	263057885	A	G
NC_040253.1	263108623	G	C
NC_040253.1	263169033	A	G
NC_040253.1	263208172	A	C
NC_040253.1	263253463	T	C
NC_040253.1	263253773	C	T
NC_040253.1	263279764	G	A
NC_040253.1	263305575	A	G
NC_040253.1	263378273	A	G
NC_040253.1	263421978	A	C
NC_040253.1	263475904	A	G
NC_040253.1	263526856	C	G
NC_040253.1	263578745	G	T
NC_040253.1	263633077	A	G
NC_040253.1	263681943	C	G
NC_040253.1	263682183	G	C
NC_040253.1	263683233	C	T
NC_040253.1	263685000	T	C
NC_040253.1	263685660	A	G
NC_040253.1	263751146	T	C
NC_040253.1	263783840	A	G
NC_040253.1	263806238	A	G
NC_040253.1	263836864	G	A
NC_040253.1	263854089	T	C
NC_040253.1	263904864	T	C
NC_040253.1	263911611	A	T
NC_040253.1	263965124	A	G
NC_040253.1	264015694	A	G
NC_040253.1	264062598	A	G
NC_040253.1	264100419	A	G

NC_040253.1	264133129	C	T
NC_040253.1	264194453	C	G
NC_040253.1	264245212	C	A
NC_040253.1	264303719	T	C
NC_040253.1	264329304	C	G
NC_040253.1	264329487	A	G
NC_040253.1	264369773	G	A
NC_040253.1	264387924	A	G
NC_040253.1	264449487	C	T
NC_040253.1	264485058	G	A
NC_040253.1	264486871	A	G
NC_040253.1	264519068	A	G
NC_040253.1	264539981	C	T
NC_040253.1	264585965	C	G
NC_040253.1	264649064	C	G
NC_040253.1	264691387	A	G
NC_040253.1	264706749	T	C
NC_040253.1	264712713	T	C
NC_040253.1	264715521	A	G
NC_040253.1	264724286	T	A
NC_040253.1	264740380	T	C
NC_040253.1	264773141	T	G
NC_040253.1	264802076	T	G
NC_040253.1	264802306	A	G
NC_040253.1	264861151	C	T
NC_040253.1	264914614	A	G
NC_040253.1	264964810	C	G
NC_040253.1	265011584	A	G
NC_040253.1	265044107	T	C
NC_040253.1	265085422	A	G
NC_040253.1	265088262	T	C
NC_040253.1	265093234	T	A
NC_040253.1	265098955	T	C
NC_040253.1	265099274	A	G
NC_040253.1	265110431	T	C
NC_040253.1	265129614	A	G
NC_040253.1	265150312	T	C
NC_040253.1	265164072	T	C
NC_040253.1	265167822	T	C
NC_040253.1	265176458	G	C
NC_040253.1	265228185	A	G
NC_040253.1	265272198	A	G
NC_040253.1	265302812	T	C
NC_040253.1	265330229	G	C
NC_040253.1	265368534	A	C
NC_040253.1	265369264	T	C
NC_040253.1	265370468	T	C

NC_040253.1	265370669	C	A
NC_040253.1	265371429	A	G
NC_040253.1	265381817	G	A
NC_040253.1	265387179	G	A
NC_040253.1	265387874	T	C
NC_040253.1	265412440	A	G
NC_040253.1	265415838	G	C
NC_040253.1	265457953	A	G
NC_040253.1	265464671	T	C
NC_040253.1	265464839	A	G
NC_040253.1	265484996	G	T
NC_040253.1	265541888	C	G
NC_040253.1	265572822	A	G
NC_040253.1	265630179	A	G
NC_040254.1	3037	C	T
NC_040254.1	3948	T	C
NC_040254.1	5836	A	G
NC_040254.1	54712	T	C
NC_040254.1	89343	0	T
NC_040254.1	124424	T	C
NC_040254.1	128472	T	C
NC_040254.1	182957	C	T
NC_040254.1	233945	T	C
NC_040254.1	255548	A	G
NC_040254.1	282468	A	G
NC_040254.1	328971	G	A
NC_040254.1	357145	T	C
NC_040254.1	358457	A	G
NC_040254.1	391131	A	G
NC_040254.1	440860	T	C
NC_040254.1	485842	A	G
NC_040254.1	499878	T	C
NC_040254.1	507416	T	C
NC_040254.1	507601	G	C
NC_040254.1	528640	A	G
NC_040254.1	535133	G	A
NC_040254.1	567440	G	T
NC_040254.1	585200	T	C
NC_040254.1	586758	T	C
NC_040254.1	614670	A	G
NC_040254.1	665509	0	C
NC_040254.1	668653	A	G
NC_040254.1	677490	T	C
NC_040254.1	679607	A	G
NC_040254.1	683890	A	G
NC_040254.1	697124	T	C
NC_040254.1	702498	T	C

NC_040254.1	705362	A	C
NC_040254.1	706275	A	G
NC_040254.1	706528	T	C
NC_040254.1	708135	0	C
NC_040254.1	708291	C	G
NC_040254.1	713184	A	G
NC_040254.1	721083	0	C
NC_040254.1	729838	A	T
NC_040254.1	732187	T	C
NC_040254.1	744978	G	C
NC_040254.1	745165	T	G
NC_040254.1	745937	T	C
NC_040254.1	746237	A	G
NC_040254.1	766772	T	C
NC_040254.1	771187	T	C
NC_040254.1	807589	C	T
NC_040254.1	825108	T	C
NC_040254.1	828735	A	C
NC_040254.1	836285	G	A
NC_040254.1	841595	A	G
NC_040254.1	841855	T	C
NC_040254.1	842867	T	C
NC_040254.1	843025	G	T
NC_040254.1	853622	C	T
NC_040254.1	855679	T	C
NC_040254.1	863509	A	G
NC_040254.1	864787	G	A
NC_040254.1	864958	A	C
NC_040254.1	869310	T	C
NC_040254.1	870299	T	C
NC_040254.1	870492	C	G
NC_040254.1	878149	T	C
NC_040254.1	883769	G	A
NC_040254.1	883926	A	G
NC_040254.1	889668	T	C
NC_040254.1	891399	C	T
NC_040254.1	895945	C	T
NC_040254.1	896273	T	C
NC_040254.1	947639	T	C
NC_040254.1	985188	G	A
NC_040254.1	995406	T	C
NC_040254.1	995668	C	T
NC_040254.1	996534	T	G
NC_040254.1	1007130	T	C
NC_040254.1	1008759	T	C
NC_040254.1	1010649	A	G
NC_040254.1	1011327	A	G

NC_040254.1	1011553	A	C
NC_040254.1	1011810	T	G
NC_040254.1	1028432	G	C
NC_040254.1	1114456	T	C
NC_040254.1	1161564	T	A
NC_040254.1	1163064	T	C
NC_040254.1	1193856	T	A
NC_040254.1	1198989	A	G
NC_040254.1	1200518	G	A
NC_040254.1	1201037	A	G
NC_040254.1	1203479	G	A
NC_040254.1	1255939	C	T
NC_040254.1	1303831	A	G
NC_040254.1	1340379	T	C
NC_040254.1	1350475	T	G
NC_040254.1	1380088	G	C
NC_040254.1	1411027	T	C
NC_040254.1	1418285	T	C
NC_040254.1	1433626	C	T
NC_040254.1	1482760	A	G
NC_040254.1	1490450	T	C
NC_040254.1	1541777	T	C
NC_040254.1	1561740	A	G
NC_040254.1	1568886	A	G
NC_040254.1	1591916	A	G
NC_040254.1	1647811	A	G
NC_040254.1	1669713	G	C
NC_040254.1	1698359	A	G
NC_040254.1	1703072	T	C
NC_040254.1	1756168	C	G
NC_040254.1	1800142	C	T
NC_040254.1	1805349	C	T
NC_040254.1	1860418	A	G
NC_040254.1	1900471	T	C
NC_040254.1	1952526	T	C
NC_040254.1	2015080	A	G
NC_040254.1	2071409	T	C
NC_040254.1	2126909	T	C
NC_040254.1	2178818	A	G
NC_040254.1	2204310	T	C
NC_040254.1	2235423	A	G
NC_040254.1	2299101	G	T
NC_040254.1	2322475	A	G
NC_040254.1	2347189	T	C
NC_040254.1	2351480	T	C
NC_040254.1	2399118	T	C
NC_040254.1	2409738	T	C

NC_040254.1	2412495	T	C
NC_040254.1	2452728	C	T
NC_040254.1	2497869	A	G
NC_040254.1	2505639	A	G
NC_040254.1	2539643	A	C
NC_040254.1	2544291	A	G
NC_040254.1	2581980	A	G
NC_040254.1	2590404	T	C
NC_040254.1	2591747	T	C
NC_040254.1	2592420	C	T
NC_040254.1	2593092	A	G
NC_040254.1	2621791	A	G
NC_040254.1	2627872	T	C
NC_040254.1	2629063	C	A
NC_040254.1	2638736	T	A
NC_040254.1	2664665	A	G
NC_040254.1	2715782	T	C
NC_040254.1	2719820	T	C
NC_040254.1	2720392	A	G
NC_040254.1	2724226	C	G
NC_040254.1	2780829	G	A
NC_040254.1	2834579	A	G
NC_040254.1	2836883	A	G
NC_040254.1	2884286	G	A
NC_040254.1	2884468	T	C
NC_040254.1	2888948	T	C
NC_040254.1	2897509	C	T
NC_040254.1	2951227	T	C
NC_040254.1	3084068	C	T
NC_040254.1	3143647	G	C
NC_040254.1	3159822	A	G
NC_040254.1	3163736	T	C
NC_040254.1	3167481	A	G
NC_040254.1	3168236	T	C
NC_040254.1	3184775	G	C
NC_040254.1	3189044	A	G
NC_040254.1	3190160	A	G
NC_040254.1	3190678	A	G
NC_040254.1	3190923	A	G
NC_040254.1	3191835	A	G
NC_040254.1	3214484	A	G
NC_040254.1	3214682	C	A
NC_040254.1	3215105	T	C
NC_040254.1	3217500	A	G
NC_040254.1	3217934	T	C
NC_040254.1	3225704	G	C
NC_040254.1	3231889	T	C

NC_040254.1	3232394	A	G
NC_040254.1	3237334	C	T
NC_040254.1	3239868	A	G
NC_040254.1	3243332	C	T
NC_040254.1	3246625	G	A
NC_040254.1	3262913	T	C
NC_040254.1	3265756	T	C
NC_040254.1	3283201	A	G
NC_040254.1	3285971	A	G
NC_040254.1	3330240	A	G
NC_040254.1	3340346	G	A
NC_040254.1	3369122	C	T
NC_040254.1	3397241	C	G
NC_040254.1	3410557	T	C
NC_040254.1	3455831	A	T
NC_040254.1	3480332	G	C
NC_040254.1	3483384	A	G
NC_040254.1	3538771	A	G
NC_040254.1	3578346	G	A
NC_040254.1	3627991	T	C
NC_040254.1	3669356	G	A
NC_040254.1	3686566	T	C
NC_040254.1	3692350	A	C
NC_040254.1	3698852	T	C
NC_040254.1	3756269	C	A
NC_040254.1	3815194	G	A
NC_040254.1	3853592	T	C
NC_040254.1	3890708	C	T
NC_040254.1	3914821	G	A
NC_040254.1	3942250	C	T
NC_040254.1	3948095	G	C
NC_040254.1	3955356	T	C
NC_040254.1	3982312	A	G
NC_040254.1	4006998	T	C
NC_040254.1	4011156	G	A
NC_040254.1	4011321	A	G
NC_040254.1	4064575	C	T
NC_040254.1	4161290	T	C
NC_040254.1	4163283	A	G
NC_040254.1	4172267	T	C
NC_040254.1	4174622	T	C
NC_040254.1	4175249	C	T
NC_040254.1	4187272	T	C
NC_040254.1	4240485	A	G
NC_040254.1	4295876	C	T
NC_040254.1	4348995	T	C
NC_040254.1	4357839	T	C

NC_040254.1	4358133	A	G
NC_040254.1	4370281	T	C
NC_040254.1	4386852	C	T
NC_040254.1	4438343	T	C
NC_040254.1	4459490	A	G
NC_040254.1	4512031	T	C
NC_040254.1	4515326	T	C
NC_040254.1	4556513	T	C
NC_040254.1	4558778	G	A
NC_040254.1	4592174	T	C
NC_040254.1	4611626	G	A
NC_040254.1	4660904	A	C
NC_040254.1	4680653	T	C
NC_040254.1	4702149	T	C
NC_040254.1	4703120	G	A
NC_040254.1	4703714	A	G
NC_040254.1	4721777	T	C
NC_040254.1	4744242	T	C
NC_040254.1	4746027	T	C
NC_040254.1	4787136	C	G
NC_040254.1	4826403	A	G
NC_040254.1	4870169	A	G
NC_040254.1	4913271	T	C
NC_040254.1	4966726	C	G
NC_040254.1	5022144	T	C
NC_040254.1	5077650	A	T
NC_040254.1	5129501	G	C
NC_040254.1	5183791	A	G
NC_040254.1	5239882	T	C
NC_040254.1	5285507	C	T
NC_040254.1	5311994	T	C
NC_040254.1	5316912	T	C
NC_040254.1	5332348	A	G
NC_040254.1	5332555	T	C
NC_040254.1	5337002	T	C
NC_040254.1	5351755	A	G
NC_040254.1	5397301	G	T
NC_040254.1	5423495	G	C
NC_040254.1	5426095	T	C
NC_040254.1	5426869	G	A
NC_040254.1	5427278	G	A
NC_040254.1	5430775	T	C
NC_040254.1	5443634	C	A
NC_040254.1	5445893	T	C
NC_040254.1	5446961	A	G
NC_040254.1	5449611	T	C
NC_040254.1	5456976	T	C

NC_040254.1	5459985	A	G
NC_040254.1	5468684	T	C
NC_040254.1	5502786	T	C
NC_040254.1	5502987	T	C
NC_040254.1	5572334	T	C
NC_040254.1	5585943	T	C
NC_040254.1	5598894	A	G
NC_040254.1	5599237	T	C
NC_040254.1	5611278	C	T
NC_040254.1	5648829	A	G
NC_040254.1	5678985	T	C
NC_040254.1	5679341	T	C
NC_040254.1	5683210	T	C
NC_040254.1	5730730	T	C
NC_040254.1	5741884	A	G
NC_040254.1	5801430	A	G
NC_040254.1	5828116	A	G
NC_040254.1	5852710	T	G
NC_040254.1	5892637	T	C
NC_040254.1	5931954	T	C
NC_040254.1	5932797	T	C
NC_040254.1	5933911	A	G
NC_040254.1	5934360	T	C
NC_040254.1	5934580	T	C
NC_040254.1	5982742	T	G
NC_040254.1	6029053	G	T
NC_040254.1	6068439	A	G
NC_040254.1	6068882	G	A
NC_040254.1	6069082	A	G
NC_040254.1	6072215	C	G
NC_040254.1	6091604	A	C
NC_040254.1	6141057	C	T
NC_040254.1	6170453	T	C
NC_040254.1	6226571	G	A
NC_040254.1	6277099	T	C
NC_040254.1	6326389	C	T
NC_040254.1	6332963	A	C
NC_040254.1	6389381	A	G
NC_040254.1	6437773	A	G
NC_040254.1	6504360	A	G
NC_040254.1	6509310	A	G
NC_040254.1	6520476	G	A
NC_040254.1	6588376	C	G
NC_040254.1	6646411	C	G
NC_040254.1	6699391	G	A
NC_040254.1	6745600	A	G
NC_040254.1	6776697	A	G

NC_040254.1	6779397	T	C
NC_040254.1	6783623	G	C
NC_040254.1	6785203	T	C
NC_040254.1	6785387	C	T
NC_040254.1	6785707	G	A
NC_040254.1	6786097	G	T
NC_040254.1	6833403	C	T
NC_040254.1	6848302	G	T
NC_040254.1	6908693	A	G
NC_040254.1	6965276	C	T
NC_040254.1	7026753	A	G
NC_040254.1	7027258	A	G
NC_040254.1	7027534	T	C
NC_040254.1	7029347	A	G
NC_040254.1	7029539	A	G
NC_040254.1	7030737	T	C
NC_040254.1	7041598	A	G
NC_040254.1	7045725	A	G
NC_040254.1	7099299	A	G
NC_040254.1	7132414	T	G
NC_040254.1	7152467	T	G
NC_040254.1	7208705	A	G
NC_040254.1	7263955	A	G
NC_040254.1	7315281	T	C
NC_040254.1	7371847	G	A
NC_040254.1	7435763	G	C
NC_040254.1	7437287	A	C
NC_040254.1	7461231	G	A
NC_040254.1	7494321	T	A
NC_040254.1	7497837	G	C
NC_040254.1	7508210	T	C
NC_040254.1	7508395	T	C
NC_040254.1	7566225	T	C
NC_040254.1	7580749	A	G
NC_040254.1	7581045	C	G
NC_040254.1	7584327	T	C
NC_040254.1	7594912	T	C
NC_040254.1	7622626	T	C
NC_040254.1	7627109	A	G
NC_040254.1	7639904	T	C
NC_040254.1	7640109	C	T
NC_040254.1	7643222	T	C
NC_040254.1	7649681	T	C
NC_040254.1	7650005	T	C
NC_040254.1	7711609	C	T
NC_040254.1	7861987	C	T
NC_040254.1	7872570	T	C

NC_040254.1	7873767	C	G
NC_040254.1	7877248	A	G
NC_040254.1	7877449	G	A
NC_040254.1	7878601	A	G
NC_040254.1	7880473	T	C
NC_040254.1	7905064	T	C
NC_040254.1	7917735	A	G
NC_040254.1	7924695	T	G
NC_040254.1	7925998	T	C
NC_040254.1	7933100	T	C
NC_040254.1	7934453	C	A
NC_040254.1	7942238	T	C
NC_040254.1	7945745	G	A
NC_040254.1	7947420	C	T
NC_040254.1	7951760	A	C
NC_040254.1	7959892	A	G
NC_040254.1	7960353	A	G
NC_040254.1	7996794	T	C
NC_040254.1	8005524	A	G
NC_040254.1	8005880	A	G
NC_040254.1	8019062	A	G
NC_040254.1	8027043	A	G
NC_040254.1	8040194	A	G
NC_040254.1	8048499	C	T
NC_040254.1	8073365	T	C
NC_040254.1	8073909	C	T
NC_040254.1	8074283	G	A
NC_040254.1	8103888	C	G
NC_040254.1	8104208	A	G
NC_040254.1	8105281	T	C
NC_040254.1	8129071	G	A
NC_040254.1	8149496	A	G
NC_040254.1	8149890	A	G
NC_040254.1	8157597	A	G
NC_040254.1	8160971	C	G
NC_040254.1	8161196	T	C
NC_040254.1	8209768	G	C
NC_040254.1	8218453	A	G
NC_040254.1	8242736	G	C
NC_040254.1	8273339	G	A
NC_040254.1	8287960	A	G
NC_040254.1	8289344	T	C
NC_040254.1	8298001	T	C
NC_040254.1	8303982	A	G
NC_040254.1	8318005	C	T
NC_040254.1	8344654	T	G
NC_040254.1	8351603	T	C

NC_040254.1	8360727	T	C
NC_040254.1	8361661	C	G
NC_040254.1	8391469	C	G
NC_040254.1	8393035	T	C
NC_040254.1	8416881	A	G
NC_040254.1	8420721	A	C
NC_040254.1	8424883	T	G
NC_040254.1	8442438	A	G
NC_040254.1	8445213	T	C
NC_040254.1	8445508	C	T
NC_040254.1	8476355	G	T
NC_040254.1	8499973	G	A
NC_040254.1	8549478	T	C
NC_040254.1	8552412	A	G
NC_040254.1	8552672	A	G
NC_040254.1	8552891	T	C
NC_040254.1	8560186	G	A
NC_040254.1	8569685	G	A
NC_040254.1	8570398	T	C
NC_040254.1	8577088	T	C
NC_040254.1	8587662	C	T
NC_040254.1	8595054	T	G
NC_040254.1	8595361	T	C
NC_040254.1	8613388	T	C
NC_040254.1	8624507	C	T
NC_040254.1	8654494	A	G
NC_040254.1	8656049	T	C
NC_040254.1	8658554	T	C
NC_040254.1	8693028	T	C
NC_040254.1	8725504	C	T
NC_040254.1	8728654	G	A
NC_040254.1	8732029	A	G
NC_040254.1	8740546	A	G
NC_040254.1	8742236	T	C
NC_040254.1	8748601	A	C
NC_040254.1	8751637	A	G
NC_040254.1	8756198	C	T
NC_040254.1	8759499	G	A
NC_040254.1	8761792	C	T
NC_040254.1	8772903	A	G
NC_040254.1	8773053	T	C
NC_040254.1	8819522	C	G
NC_040254.1	8851720	T	C
NC_040254.1	8876404	G	T
NC_040254.1	8936501	T	C
NC_040254.1	8936968	T	C
NC_040254.1	8997582	G	C

NC_040254.1	9005710	A	G
NC_040254.1	9013531	G	A
NC_040254.1	9071180	C	A
NC_040254.1	9121525	C	T
NC_040254.1	9156437	C	T
NC_040254.1	9180557	G	A
NC_040254.1	9180863	G	A
NC_040254.1	9181252	T	C
NC_040254.1	9182633	T	C
NC_040254.1	9188578	A	G
NC_040254.1	9207410	A	G
NC_040254.1	9263619	T	C
NC_040254.1	9302798	T	C
NC_040254.1	9323671	T	C
NC_040254.1	9324800	T	C
NC_040254.1	9381350	A	G
NC_040254.1	9436256	C	A
NC_040254.1	9500464	G	A
NC_040254.1	9501094	C	A
NC_040254.1	9502494	A	T
NC_040254.1	9502644	G	T
NC_040254.1	9503875	C	G
NC_040254.1	9546627	G	A
NC_040254.1	9549546	A	G
NC_040254.1	9549840	T	C
NC_040254.1	9550756	A	G
NC_040254.1	9602872	G	A
NC_040254.1	9658346	T	C
NC_040254.1	9714808	T	C
NC_040254.1	9771517	A	G
NC_040254.1	9832252	A	G
NC_040254.1	9895990	A	G
NC_040254.1	9949819	G	A
NC_040254.1	9987788	G	A
NC_040254.1	10046314	T	C
NC_040254.1	10099434	T	C
NC_040254.1	10154947	T	C
NC_040254.1	10218189	G	A
NC_040254.1	10270239	G	T
NC_040254.1	10298878	C	T
NC_040254.1	10326226	T	G
NC_040254.1	10383224	T	C
NC_040254.1	10436933	A	G
NC_040254.1	10491988	A	G
NC_040254.1	10548933	A	G
NC_040254.1	10601148	C	T
NC_040254.1	10605591	G	A

NC_040254.1	10646473	T	C
NC_040254.1	10682832	A	G
NC_040254.1	10732012	C	T
NC_040254.1	10785617	T	C
NC_040254.1	10799794	C	T
NC_040254.1	10857652	A	G
NC_040254.1	10915742	T	C
NC_040254.1	10965484	T	C
NC_040254.1	10965718	T	A
NC_040254.1	10968352	A	C
NC_040254.1	10968545	C	T
NC_040254.1	11016304	T	C
NC_040254.1	11052384	A	G
NC_040254.1	11072892	A	G
NC_040254.1	11082258	A	G
NC_040254.1	11118939	A	C
NC_040254.1	11120672	A	G
NC_040254.1	11124568	T	C
NC_040254.1	11177004	T	G
NC_040254.1	11201005	T	C
NC_040254.1	11271649	G	C
NC_040254.1	11286121	T	C
NC_040254.1	11287832	A	G
NC_040254.1	11288103	C	T
NC_040254.1	11346800	T	C
NC_040254.1	11359816	G	A
NC_040254.1	11398892	A	C
NC_040254.1	11432890	T	C
NC_040254.1	11467832	G	A
NC_040254.1	11523527	A	G
NC_040254.1	11567463	C	T
NC_040254.1	11611948	C	G
NC_040254.1	11626269	G	C
NC_040254.1	11691565	T	C
NC_040254.1	11714831	G	T
NC_040254.1	11738108	A	G
NC_040254.1	11754239	C	T
NC_040254.1	11819236	T	C
NC_040254.1	11906351	C	T
NC_040254.1	11952538	A	G
NC_040254.1	12006578	T	C
NC_040254.1	12017038	T	C
NC_040254.1	12203711	C	T
NC_040254.1	12243037	G	C
NC_040254.1	12295064	A	G
NC_040254.1	12328025	T	C
NC_040254.1	12402342	T	C

NC_040254.1	12447892	C	G
NC_040254.1	12574931	A	G
NC_040254.1	12616758	G	A
NC_040254.1	12643885	T	C
NC_040254.1	12702150	A	G
NC_040254.1	12758782	G	C
NC_040254.1	12787287	G	A
NC_040254.1	12814402	C	T
NC_040254.1	12891176	A	C
NC_040254.1	12899573	T	C
NC_040254.1	12950830	G	A
NC_040254.1	12990818	C	G
NC_040254.1	13049566	G	A
NC_040254.1	13076253	A	C
NC_040254.1	13105605	C	T
NC_040254.1	13153785	G	A
NC_040254.1	13185272	T	C
NC_040254.1	13198419	A	G
NC_040254.1	13239053	A	G
NC_040254.1	13283511	T	C
NC_040254.1	13313173	G	C
NC_040254.1	13340070	G	A
NC_040254.1	13378227	G	C
NC_040254.1	13407351	A	G
NC_040254.1	13449431	C	T
NC_040254.1	13528460	T	C
NC_040254.1	13584007	T	C
NC_040254.1	13863355	C	T
NC_040254.1	13938519	T	A
NC_040254.1	14081348	A	G
NC_040254.1	14122703	G	A
NC_040254.1	14184400	C	T
NC_040254.1	14286452	A	G
NC_040254.1	14477962	G	T
NC_040254.1	14532416	C	T
NC_040254.1	14574845	A	G
NC_040254.1	14627723	A	G
NC_040254.1	14919874	G	A
NC_040254.1	15056669	A	G
NC_040254.1	15610190	A	G
NC_040254.1	16133667	C	T
NC_040254.1	16196751	A	G
NC_040254.1	16266724	T	C
NC_040254.1	16354299	T	C
NC_040254.1	16416305	T	C
NC_040254.1	16663839	T	C
NC_040254.1	16702111	G	A

NC_040254.1	16827671	G	C
NC_040254.1	16880526	T	C
NC_040254.1	16963369	T	C
NC_040254.1	17047553	G	A
NC_040254.1	17184298	T	A
NC_040254.1	17274191	G	A
NC_040254.1	17490130	A	G
NC_040254.1	17562564	A	T
NC_040254.1	17667172	T	C
NC_040254.1	18019640	A	G
NC_040254.1	18489487	A	G
NC_040254.1	18575780	A	C
NC_040254.1	18622249	A	G
NC_040254.1	18699379	G	A
NC_040254.1	18761322	A	G
NC_040254.1	18792519	G	A
NC_040254.1	18859107	T	C
NC_040254.1	18905701	G	A
NC_040254.1	19012972	A	G
NC_040254.1	19048044	A	G
NC_040254.1	19091031	G	A
NC_040254.1	19157051	A	G
NC_040254.1	19223319	C	T
NC_040254.1	19293946	G	A
NC_040254.1	19366714	T	A
NC_040254.1	19434333	C	T
NC_040254.1	19675903	C	A
NC_040254.1	19875697	T	G
NC_040254.1	19929334	A	C
NC_040254.1	20017012	C	A
NC_040254.1	20071423	T	C
NC_040254.1	20190882	G	A
NC_040254.1	20247193	G	A
NC_040254.1	20578552	A	C
NC_040254.1	20635672	T	C
NC_040254.1	20702241	G	T
NC_040254.1	20765586	A	C
NC_040254.1	20843940	C	T
NC_040254.1	20902791	A	T
NC_040254.1	20958745	C	G
NC_040254.1	21017032	A	G
NC_040254.1	21084761	T	C
NC_040254.1	21135866	C	T
NC_040254.1	21163572	C	T
NC_040254.1	21217333	A	T
NC_040254.1	21274042	A	G
NC_040254.1	21341564	T	C

NC_040254.1	21414564	A	G
NC_040254.1	21487911	C	T
NC_040254.1	21548790	C	T
NC_040254.1	21605218	A	G
NC_040254.1	21661243	A	G
NC_040254.1	21725796	G	T
NC_040254.1	21781878	A	C
NC_040254.1	21837098	A	C
NC_040254.1	21901141	A	G
NC_040254.1	21956719	G	A
NC_040254.1	22012989	C	T
NC_040254.1	22150706	A	G
NC_040254.1	22207127	A	G
NC_040254.1	22266488	T	C
NC_040254.1	22327968	A	G
NC_040254.1	22388957	C	T
NC_040254.1	22446300	A	G
NC_040254.1	22509603	T	C
NC_040254.1	22572799	T	C
NC_040254.1	22783125	C	T
NC_040254.1	23004312	G	A
NC_040254.1	23054142	C	T
NC_040254.1	23106055	A	T
NC_040254.1	23459777	T	C
NC_040254.1	23479727	A	G
NC_040254.1	23626381	C	T
NC_040254.1	23825061	T	C
NC_040254.1	23885267	T	C
NC_040254.1	23960356	C	G
NC_040254.1	23978459	A	C
NC_040254.1	24144548	T	C
NC_040254.1	24206775	C	G
NC_040254.1	24389251	G	A
NC_040254.1	24523867	G	T
NC_040254.1	24586868	A	T
NC_040254.1	24640463	G	A
NC_040254.1	24690253	C	T
NC_040254.1	24734914	C	T
NC_040254.1	24790342	T	C
NC_040254.1	24846032	C	A
NC_040254.1	24900046	A	T
NC_040254.1	24960725	C	T
NC_040254.1	25022353	T	G
NC_040254.1	25086507	A	G
NC_040254.1	25162198	G	A
NC_040254.1	25186482	A	G
NC_040254.1	25433670	G	C

NC_040254.1	26351155	T	G
NC_040254.1	26970455	G	C
NC_040254.1	27757290	A	G
NC_040254.1	28165283	A	C
NC_040254.1	28623228	A	T
NC_040254.1	28812090	A	G
NC_040254.1	29462938	C	A
NC_040254.1	29519354	C	T
NC_040254.1	29574147	A	G
NC_040254.1	29636729	A	C
NC_040254.1	29691089	A	G
NC_040254.1	29760349	G	A
NC_040254.1	29821875	T	C
NC_040254.1	29884731	A	C
NC_040254.1	29951261	T	C
NC_040254.1	30061353	A	G
NC_040254.1	30253130	T	C
NC_040254.1	30313544	C	G
NC_040254.1	30376567	T	C
NC_040254.1	30557427	T	A
NC_040254.1	30594388	A	C
NC_040254.1	30649674	G	A
NC_040254.1	30716823	G	A
NC_040254.1	30772612	A	G
NC_040254.1	30837494	G	A
NC_040254.1	30971048	G	A
NC_040254.1	31028362	G	A
NC_040254.1	31096456	A	G
NC_040254.1	31270674	C	A
NC_040254.1	31338918	G	T
NC_040254.1	31410378	T	A
NC_040254.1	31465666	A	G
NC_040254.1	31521310	C	T
NC_040254.1	31585402	A	G
NC_040254.1	31647417	G	A
NC_040254.1	31743410	C	T
NC_040254.1	31769072	G	A
NC_040254.1	31784178	T	C
NC_040254.1	32120168	T	C
NC_040254.1	32601546	T	C
NC_040254.1	33062454	C	T
NC_040254.1	33725944	T	C
NC_040254.1	33858792	A	T
NC_040254.1	34279352	A	G
NC_040254.1	34897463	C	A
NC_040254.1	35208359	C	T
NC_040254.1	35579556	A	G

NC_040254.1	35746733	G	C
NC_040254.1	35992654	G	A
NC_040254.1	36093155	G	T
NC_040254.1	36218847	C	A
NC_040254.1	36653680	T	C
NC_040254.1	36773815	G	T
NC_040254.1	37092483	A	G
NC_040254.1	37740422	T	G
NC_040254.1	38008076	C	T
NC_040254.1	38398579	T	C
NC_040254.1	38787954	A	G
NC_040254.1	39169130	G	A
NC_040254.1	40145450	T	A
NC_040254.1	40598247	G	A
NC_040254.1	40711261	T	C
NC_040254.1	40775074	C	A
NC_040254.1	40831206	G	A
NC_040254.1	40888239	C	G
NC_040254.1	40947432	C	T
NC_040254.1	41002899	G	A
NC_040254.1	41062063	G	A
NC_040254.1	41121633	A	G
NC_040254.1	41188582	A	C
NC_040254.1	41241396	A	C
NC_040254.1	41309946	T	G
NC_040254.1	41378507	G	A
NC_040254.1	41436798	A	G
NC_040254.1	41494757	A	G
NC_040254.1	41617424	G	A
NC_040254.1	41675164	G	A
NC_040254.1	41733002	G	A
NC_040254.1	41791616	A	C
NC_040254.1	41850082	C	T
NC_040254.1	41907454	T	C
NC_040254.1	41967334	T	C
NC_040254.1	42032178	G	A
NC_040254.1	42090986	C	T
NC_040254.1	42207792	C	T
NC_040254.1	42268250	G	C
NC_040254.1	42329371	T	G
NC_040254.1	42382763	T	C
NC_040254.1	42402234	A	G
NC_040254.1	42461794	C	G
NC_040254.1	42523033	A	T
NC_040254.1	42586072	G	A
NC_040254.1	42643583	T	C
NC_040254.1	42707343	A	G

NC_040254.1	42773441	T	C
NC_040254.1	42841488	A	C
NC_040254.1	43043589	C	T
NC_040254.1	43265780	A	G
NC_040254.1	43477931	A	G
NC_040254.1	43858254	A	G
NC_040254.1	44070335	G	C
NC_040254.1	44368216	A	G
NC_040254.1	44676270	A	G
NC_040254.1	44922709	T	C
NC_040254.1	45141620	A	G
NC_040254.1	45742541	A	G
NC_040254.1	46729818	T	C
NC_040254.1	47288078	A	G
NC_040254.1	47950406	C	T
NC_040254.1	48403794	A	T
NC_040254.1	48527484	C	G
NC_040254.1	48586936	T	C
NC_040254.1	48701921	G	A
NC_040254.1	48761589	G	A
NC_040254.1	48818178	C	T
NC_040254.1	48872734	G	A
NC_040254.1	48939937	G	A
NC_040254.1	49003168	G	T
NC_040254.1	49242126	G	C
NC_040254.1	49410742	G	A
NC_040254.1	49476698	G	T
NC_040254.1	49546553	T	C
NC_040254.1	49595447	A	G
NC_040254.1	49654931	G	A
NC_040254.1	49720218	C	A
NC_040254.1	50086525	C	T
NC_040254.1	50153398	A	G
NC_040254.1	50223918	C	T
NC_040254.1	50340172	A	T
NC_040254.1	50411783	A	G
NC_040254.1	50469261	T	C
NC_040254.1	50542169	G	A
NC_040254.1	50708364	G	A
NC_040254.1	50776151	C	A
NC_040254.1	50839115	G	A
NC_040254.1	50960660	T	C
NC_040254.1	51077425	T	C
NC_040254.1	51146928	A	G
NC_040254.1	51375384	T	G
NC_040254.1	51559879	A	G
NC_040254.1	51821771	T	G

NC_040254.1	51904545	C	G
NC_040254.1	51967398	A	G
NC_040254.1	52084235	T	C
NC_040254.1	52156199	G	A
NC_040254.1	52218132	G	T
NC_040254.1	52284544	A	C
NC_040254.1	52359127	T	C
NC_040254.1	52435354	C	T
NC_040254.1	52578755	T	C
NC_040254.1	52700653	T	G
NC_040254.1	52732243	C	A
NC_040254.1	52796557	A	C
NC_040254.1	52873725	G	A
NC_040254.1	52941944	G	A
NC_040254.1	53007021	G	A
NC_040254.1	53067843	A	G
NC_040254.1	53127056	A	G
NC_040254.1	53190985	T	G
NC_040254.1	53255573	G	C
NC_040254.1	53315014	T	C
NC_040254.1	53625200	T	C
NC_040254.1	53702323	C	T
NC_040254.1	53782129	A	C
NC_040254.1	53861603	A	G
NC_040254.1	53933492	T	C
NC_040254.1	54059455	C	T
NC_040254.1	54116815	A	G
NC_040254.1	54178852	A	G
NC_040254.1	54239924	C	G
NC_040254.1	54303332	C	T
NC_040254.1	54375029	G	A
NC_040254.1	54469213	A	G
NC_040254.1	55133735	A	G
NC_040254.1	55194618	G	A
NC_040254.1	55250854	C	G
NC_040254.1	55374931	A	G
NC_040254.1	55504784	G	A
NC_040254.1	55556556	G	A
NC_040254.1	55768769	T	G
NC_040254.1	55834301	T	C
NC_040254.1	55950250	G	C
NC_040254.1	56072968	T	A
NC_040254.1	56148382	T	G
NC_040254.1	56182727	T	C
NC_040254.1	56236506	C	T
NC_040254.1	56291612	A	T
NC_040254.1	56760532	C	A

NC_040254.1	56991364	T	G
NC_040254.1	57019150	G	T
NC_040254.1	57082992	G	A
NC_040254.1	57157365	A	T
NC_040254.1	57223200	A	T
NC_040254.1	57344554	A	G
NC_040254.1	57411673	A	G
NC_040254.1	57470377	A	G
NC_040254.1	57529282	G	A
NC_040254.1	57584574	T	C
NC_040254.1	57719432	T	C
NC_040254.1	57769272	G	C
NC_040254.1	57958249	G	A
NC_040254.1	57977317	C	T
NC_040254.1	58079406	T	C
NC_040254.1	58375908	G	A
NC_040254.1	58446765	C	T
NC_040254.1	58516099	A	G
NC_040254.1	58532944	G	A
NC_040254.1	58632371	T	C
NC_040254.1	58685242	T	C
NC_040254.1	58747201	T	G
NC_040254.1	58817662	C	T
NC_040254.1	58893194	C	T
NC_040254.1	59098008	C	T
NC_040254.1	59312176	C	T
NC_040254.1	59510304	A	G
NC_040254.1	59710310	G	A
NC_040254.1	59832342	A	G
NC_040254.1	59888935	A	G
NC_040254.1	59951696	A	G
NC_040254.1	60027020	A	G
NC_040254.1	60314809	A	G
NC_040254.1	60376094	A	G
NC_040254.1	60445732	G	A
NC_040254.1	60568238	C	G
NC_040254.1	60683225	A	C
NC_040254.1	60756564	T	C
NC_040254.1	60814352	A	G
NC_040254.1	60871282	T	G
NC_040254.1	60928468	A	G
NC_040254.1	60989645	T	C
NC_040254.1	61047729	C	T
NC_040254.1	61107998	G	A
NC_040254.1	61170641	C	T
NC_040254.1	61224177	T	C
NC_040254.1	61284881	G	A

NC_040254.1	61346633	G	A
NC_040254.1	61404367	G	C
NC_040254.1	61465081	A	G
NC_040254.1	61521019	G	A
NC_040254.1	61600594	T	C
NC_040254.1	61643137	A	G
NC_040254.1	61758533	A	G
NC_040254.1	61811912	T	C
NC_040254.1	61941837	A	G
NC_040254.1	62001940	G	A
NC_040254.1	62058046	C	T
NC_040254.1	62171316	A	G
NC_040254.1	62239411	A	G
NC_040254.1	62307850	G	A
NC_040254.1	62359221	G	C
NC_040254.1	62416453	C	T
NC_040254.1	62476968	G	A
NC_040254.1	62601544	G	C
NC_040254.1	62657532	T	G
NC_040254.1	62716744	T	C
NC_040254.1	62784926	T	C
NC_040254.1	62836984	C	G
NC_040254.1	62903542	C	T
NC_040254.1	62961343	A	G
NC_040254.1	63031413	G	A
NC_040254.1	63091024	T	C
NC_040254.1	63150794	G	A
NC_040254.1	63210576	G	T
NC_040254.1	63276948	T	C
NC_040254.1	63335453	T	C
NC_040254.1	63391935	A	G
NC_040254.1	63569329	C	T
NC_040254.1	63641728	T	C
NC_040254.1	63697924	C	T
NC_040254.1	63759984	C	A
NC_040254.1	63820469	G	A
NC_040254.1	63879474	T	C
NC_040254.1	63935952	C	T
NC_040254.1	64001770	T	C
NC_040254.1	64109698	T	C
NC_040254.1	64168839	C	T
NC_040254.1	64230532	T	C
NC_040254.1	64286522	T	C
NC_040254.1	64343060	T	A
NC_040254.1	64402964	A	G
NC_040254.1	64460432	C	T
NC_040254.1	64586664	A	C

NC_040254.1	64657462	A	G
NC_040254.1	64955425	A	G
NC_040254.1	65011274	G	A
NC_040254.1	65068092	T	C
NC_040254.1	65128424	T	C
NC_040254.1	65188004	A	G
NC_040254.1	65252594	C	A
NC_040254.1	65309151	C	G
NC_040254.1	65367742	C	G
NC_040254.1	65430992	A	G
NC_040254.1	65492999	T	C
NC_040254.1	65552351	A	G
NC_040254.1	65607585	G	C
NC_040254.1	65688126	A	G
NC_040254.1	65742873	C	T
NC_040254.1	65802144	A	T
NC_040254.1	65862779	T	C
NC_040254.1	65922887	T	G
NC_040254.1	65979236	T	G
NC_040254.1	66034351	G	A
NC_040254.1	66092360	T	C
NC_040254.1	66159711	G	A
NC_040254.1	66217024	G	T
NC_040254.1	66273706	A	G
NC_040254.1	66331171	A	G
NC_040254.1	66399731	C	G
NC_040254.1	66460389	C	T
NC_040254.1	66517448	T	A
NC_040254.1	66578424	C	T
NC_040254.1	66645629	A	G
NC_040254.1	66701382	C	G
NC_040254.1	66760303	T	C
NC_040254.1	66891832	C	T
NC_040254.1	67121543	G	A
NC_040254.1	67185011	A	G
NC_040254.1	67246722	G	A
NC_040254.1	67303970	C	T
NC_040254.1	67363944	C	T
NC_040254.1	67421320	T	C
NC_040254.1	67476497	G	A
NC_040254.1	67532898	C	T
NC_040254.1	67589280	C	T
NC_040254.1	67714544	C	T
NC_040254.1	67768496	C	T
NC_040254.1	67825200	T	C
NC_040254.1	67882483	A	T
NC_040254.1	67939598	C	A

NC_040254.1	67998248	T		C
NC_040254.1	68057480	C		T
NC_040254.1	68114421	G		A
NC_040254.1	68172435	A		T
NC_040254.1	68231759	A		G
NC_040254.1	68289375	T		C
NC_040254.1	68345876	G		A
NC_040254.1	68407780	T		A
NC_040254.1	68465590	C		T
NC_040254.1	68526411	G		C
NC_040254.1	68592256	T		C
NC_040254.1	68697466	T		C
NC_040254.1	68766707	G		A
NC_040254.1	68822890	A		G
NC_040254.1	68897357	G		A
NC_040254.1	68959050	T		G
NC_040254.1	69037653	G		A
NC_040254.1	69092965	G		C
NC_040254.1	69149956	C		T
NC_040254.1	69210450	A		G
NC_040254.1	69265699	A		G
NC_040254.1	69331390	G		C
NC_040254.1	69391204	A		G
NC_040254.1	69754456	G		A
NC_040254.1	69832707	T		G
NC_040254.1	69897863	C		T
NC_040254.1	70082070	T		G
NC_040254.1	70151891	A		G
NC_040254.1	70208988	C		T
NC_040254.1	70276579	C		T
NC_040254.1	70339115	G		A
NC_040254.1	70404001	A		T
NC_040254.1	70463374	T		G
NC_040254.1	70524994	G		A
NC_040254.1	70581235	C		T
NC_040254.1	70648774	C		T
NC_040254.1	70713472	A		G
NC_040254.1	70770197	T		C
NC_040254.1	70844574	T		C
NC_040254.1	70905910	G		A
NC_040254.1	70972338	C		T
NC_040254.1	71030992	A		G
NC_040254.1	71086175	A		G
NC_040254.1	71149439	A		G
NC_040254.1	71206797		0	C
NC_040254.1	71263911	A		G
NC_040254.1	71322820	C		T

NC_040254.1	71392650	T	A
NC_040254.1	71448465	C	T
NC_040254.1	71506222	C	A
NC_040254.1	71563677	A	T
NC_040254.1	71620613	A	G
NC_040254.1	71680788	A	G
NC_040254.1	71737635	A	G
NC_040254.1	71795488	A	G
NC_040254.1	71858979	C	G
NC_040254.1	72037776	T	C
NC_040254.1	72091541	A	G
NC_040254.1	72175693	C	T
NC_040254.1	72405141	G	A
NC_040254.1	72490246	A	G
NC_040254.1	72548985	T	C
NC_040254.1	72600160	C	T
NC_040254.1	72659771	A	C
NC_040254.1	72718694	A	T
NC_040254.1	72779690	A	C
NC_040254.1	72848716	G	T
NC_040254.1	72898453	G	A
NC_040254.1	72953704	G	A
NC_040254.1	73012512	C	T
NC_040254.1	73069574	T	A
NC_040254.1	73127277	T	C
NC_040254.1	73188618	C	T
NC_040254.1	73294628	G	A
NC_040254.1	73357229	A	G
NC_040254.1	73475460	C	A
NC_040254.1	73531991	G	C
NC_040254.1	73590592	G	A
NC_040254.1	73644263	C	T
NC_040254.1	73707798	A	G
NC_040254.1	73762648	T	C
NC_040254.1	73794349	G	A
NC_040254.1	73849730	T	C
NC_040254.1	73902986	A	G
NC_040254.1	73964582	T	C
NC_040254.1	74045013	C	T
NC_040254.1	74102798	C	T
NC_040254.1	74171064	A	G
NC_040254.1	74229294	G	A
NC_040254.1	74285831	T	C
NC_040254.1	74342125	A	G
NC_040254.1	74403080	C	A
NC_040254.1	74519371	C	T
NC_040254.1	74599497	T	C

NC_040254.1	74668955	A	T
NC_040254.1	74730897	T	A
NC_040254.1	74793679	T	G
NC_040254.1	74908691	A	G
NC_040254.1	74963359	G	A
NC_040254.1	75023690	G	A
NC_040254.1	75210204	G	C
NC_040254.1	75276833	G	T
NC_040254.1	75350057	G	A
NC_040254.1	75414178	G	A
NC_040254.1	75472796	G	A
NC_040254.1	75532994	A	G
NC_040254.1	75598271	G	A
NC_040254.1	75672134	C	A
NC_040254.1	75735200	T	C
NC_040254.1	75796891	C	T
NC_040254.1	75859298	C	T
NC_040254.1	75928955	G	A
NC_040254.1	75993479	G	A
NC_040254.1	76048997	G	A
NC_040254.1	76114196	C	T
NC_040254.1	76184211	A	G
NC_040254.1	76251458	G	A
NC_040254.1	76335282	G	A
NC_040254.1	76403710	T	C
NC_040254.1	76466205	T	C
NC_040254.1	76535660	T	C
NC_040254.1	76651656	A	G
NC_040254.1	76725449	T	C
NC_040254.1	76792081	C	G
NC_040254.1	76863609	T	G
NC_040254.1	76991000	G	A
NC_040254.1	77310947	C	T
NC_040254.1	77432187	G	A
NC_040254.1	77507670	T	C
NC_040254.1	77630326	T	C
NC_040254.1	77751971	C	T
NC_040254.1	78019271	C	T
NC_040254.1	78093084	G	C
NC_040254.1	78156193	G	A
NC_040254.1	78240253	A	G
NC_040254.1	78305380	T	C
NC_040254.1	78414187	C	A
NC_040254.1	78480779	T	C
NC_040254.1	78539086	T	C
NC_040254.1	78615841	T	A
NC_040254.1	78674927	G	C

NC_040254.1	78731311	A	G
NC_040254.1	78796234	A	G
NC_040254.1	78853858	A	G
NC_040254.1	78910545	C	T
NC_040254.1	78971459	A	G
NC_040254.1	79087559	C	T
NC_040254.1	79148422	A	T
NC_040254.1	79218518	A	G
NC_040254.1	79272069	G	A
NC_040254.1	79338189	T	C
NC_040254.1	79405066	G	A
NC_040254.1	79472226	C	A
NC_040254.1	79539178	C	A
NC_040254.1	79600167	G	A
NC_040254.1	79657362	A	G
NC_040254.1	79721479	T	G
NC_040254.1	80026610	A	G
NC_040254.1	80094010	C	T
NC_040254.1	80158407	T	C
NC_040254.1	80221066	G	A
NC_040254.1	80272899	A	C
NC_040254.1	80338529	C	G
NC_040254.1	80409557	A	G
NC_040254.1	80527489	G	T
NC_040254.1	80593555	A	G
NC_040254.1	80655958	C	A
NC_040254.1	80686113	A	G
NC_040254.1	80742409	C	A
NC_040254.1	80799667	T	C
NC_040254.1	80861246	T	A
NC_040254.1	80942110	A	G
NC_040254.1	80995627	C	T
NC_040254.1	81068154	A	G
NC_040254.1	81134270	C	T
NC_040254.1	81190808	A	G
NC_040254.1	81278210	T	G
NC_040254.1	81339227	T	C
NC_040254.1	81450752	A	G
NC_040254.1	81518165	A	G
NC_040254.1	81584107	G	C
NC_040254.1	81640214	G	C
NC_040254.1	81702769	A	G
NC_040254.1	81761626	G	T
NC_040254.1	81827137	T	C
NC_040254.1	81880583	A	G
NC_040254.1	81932846	G	T
NC_040254.1	81996081	C	A

NC_040254.1	82065143	G	A
NC_040254.1	82123579	T	C
NC_040254.1	82182347	C	G
NC_040254.1	82251387	C	T
NC_040254.1	82311282	G	T
NC_040254.1	82373676	G	A
NC_040254.1	82433729	T	C
NC_040254.1	82490850	A	G
NC_040254.1	82547742	A	G
NC_040254.1	82607517	T	C
NC_040254.1	82669278	T	A
NC_040254.1	82732963	C	T
NC_040254.1	82799849	A	G
NC_040254.1	82854577	C	T
NC_040254.1	82924624	G	A
NC_040254.1	82987960	G	A
NC_040254.1	83043266	A	G
NC_040254.1	83066250	C	G
NC_040254.1	83186255	G	A
NC_040254.1	83249016	G	C
NC_040254.1	83306996	A	G
NC_040254.1	83365145	A	G
NC_040254.1	83419320	C	T
NC_040254.1	83483867	T	C
NC_040254.1	83544617	G	C
NC_040254.1	83608974	A	G
NC_040254.1	83675315	G	T
NC_040254.1	83731520	G	C
NC_040254.1	83788835	G	A
NC_040254.1	83848279	C	T
NC_040254.1	83905660	T	C
NC_040254.1	83970653	C	G
NC_040254.1	84038511	G	A
NC_040254.1	84102842	C	A
NC_040254.1	84158272	A	G
NC_040254.1	84219550	G	A
NC_040254.1	84281019	T	C
NC_040254.1	84344753	A	G
NC_040254.1	84415205	A	G
NC_040254.1	84469799	T	C
NC_040254.1	84537115	T	C
NC_040254.1	84593385	T	C
NC_040254.1	84653124	A	C
NC_040254.1	84720537	G	T
NC_040254.1	84781379	T	C
NC_040254.1	84841799	T	C
NC_040254.1	84898774	C	G

NC_040254.1	84956145	T	C
NC_040254.1	85076921	T	C
NC_040254.1	85133380	A	G
NC_040254.1	85198384	A	G
NC_040254.1	85257220	T	C
NC_040254.1	85314151	T	C
NC_040254.1	85375043	G	A
NC_040254.1	85443011	G	T
NC_040254.1	85548746	G	A
NC_040254.1	85607298	T	G
NC_040254.1	85668551	T	C
NC_040254.1	85728116	T	C
NC_040254.1	85785531	C	T
NC_040254.1	85842659	G	A
NC_040254.1	85904670	T	C
NC_040254.1	85962943	G	A
NC_040254.1	86020945	C	T
NC_040254.1	86079178	A	C
NC_040254.1	86197353	T	C
NC_040254.1	86255002	T	C
NC_040254.1	86314675	A	G
NC_040254.1	86378349	T	C
NC_040254.1	86503425	G	T
NC_040254.1	86562033	G	A
NC_040254.1	86620732	T	C
NC_040254.1	86673007	A	G
NC_040254.1	87439575	T	C
NC_040254.1	87496698	A	T
NC_040254.1	87560102	C	T
NC_040254.1	87617609	C	A
NC_040254.1	87678304	T	G
NC_040254.1	87739739	C	T
NC_040254.1	87828493	A	G
NC_040254.1	87942330	A	T
NC_040254.1	88314359	C	T
NC_040254.1	88387484	T	C
NC_040254.1	88452531	G	A
NC_040254.1	88516215	T	C
NC_040254.1	88569566	C	T
NC_040254.1	88666592	A	T
NC_040254.1	88727253	A	G
NC_040254.1	88775516	A	C
NC_040254.1	88839879	G	A
NC_040254.1	88897118	A	G
NC_040254.1	88957687	T	C
NC_040254.1	89023989	C	T
NC_040254.1	89128827	T	A

NC_040254.1	89204619	G	A
NC_040254.1	89263660	G	A
NC_040254.1	89324130	C	G
NC_040254.1	89397736	T	C
NC_040254.1	89454694	G	A
NC_040254.1	89514951	G	C
NC_040254.1	89574657	A	G
NC_040254.1	89661906	T	C
NC_040254.1	89719907	A	G
NC_040254.1	89754476	C	T
NC_040254.1	89819774	T	C
NC_040254.1	89885720	A	G
NC_040254.1	89952136	C	T
NC_040254.1	90016958	T	C
NC_040254.1	90074679	C	T
NC_040254.1	90131998	G	A
NC_040254.1	90189229	T	C
NC_040254.1	90250383	A	G
NC_040254.1	90314063	T	C
NC_040254.1	90366985	G	A
NC_040254.1	90423203	G	T
NC_040254.1	90493323	A	G
NC_040254.1	90555225	T	C
NC_040254.1	90628644	T	A
NC_040254.1	90685569	G	T
NC_040254.1	90745489	C	T
NC_040254.1	90807932	G	A
NC_040254.1	90922230	C	T
NC_040254.1	90981412	C	T
NC_040254.1	91043863	A	G
NC_040254.1	91111407	T	A
NC_040254.1	91167570	G	A
NC_040254.1	91233882	A	C
NC_040254.1	91296933	A	G
NC_040254.1	91357706	G	T
NC_040254.1	91419658	G	T
NC_040254.1	91478691	T	G
NC_040254.1	91535709	C	T
NC_040254.1	91594276	T	C
NC_040254.1	91658468	C	T
NC_040254.1	91728094	T	A
NC_040254.1	91793229	A	C
NC_040254.1	91843707	G	A
NC_040254.1	91915204	G	A
NC_040254.1	91969838	T	G
NC_040254.1	91984042	C	T
NC_040254.1	92164096	A	C

NC_040254.1	92222205	T	G
NC_040254.1	92297699	T	A
NC_040254.1	92360346	A	G
NC_040254.1	92425731	G	A
NC_040254.1	92489456	T	C
NC_040254.1	92554474	G	A
NC_040254.1	92605222	T	C
NC_040254.1	92665904	T	C
NC_040254.1	92725519	G	C
NC_040254.1	92784993	C	T
NC_040254.1	92843294	A	G
NC_040254.1	92901480	C	T
NC_040254.1	92964002	G	A
NC_040254.1	93016308	T	C
NC_040254.1	93074322	A	T
NC_040254.1	93131463	C	T
NC_040254.1	93193260	G	T
NC_040254.1	93257024	C	T
NC_040254.1	93321311	C	A
NC_040254.1	93499073	A	G
NC_040254.1	93560870	A	G
NC_040254.1	93625791	A	G
NC_040254.1	93689926	A	C
NC_040254.1	93884068	A	C
NC_040254.1	93897785	T	C
NC_040254.1	94137672	G	C
NC_040254.1	94195523	G	T
NC_040254.1	94236151	T	A
NC_040254.1	94401400	G	C
NC_040254.1	94463071	G	A
NC_040254.1	94534192	C	A
NC_040254.1	94562148	T	G
NC_040254.1	94692500	A	G
NC_040254.1	94881178	T	C
NC_040254.1	94944893	C	G
NC_040254.1	94998874	T	C
NC_040254.1	95132191	C	T
NC_040254.1	95303791	A	G
NC_040254.1	95461047	C	T
NC_040254.1	95505950	A	G
NC_040254.1	95559551	A	G
NC_040254.1	95624422	C	T
NC_040254.1	95781001	A	G
NC_040254.1	95978500	A	G
NC_040254.1	96003439	C	T
NC_040254.1	96279022	A	C
NC_040254.1	96335291	T	C

NC_040254.1	96398834	C	T
NC_040254.1	96516845	A	T
NC_040254.1	96579699	A	C
NC_040254.1	96637606	A	G
NC_040254.1	96697363	T	A
NC_040254.1	96756735	G	A
NC_040254.1	96818286	T	G
NC_040254.1	96879005	G	T
NC_040254.1	96939175	C	T
NC_040254.1	96997978	A	C
NC_040254.1	97059526	A	G
NC_040254.1	97120416	G	A
NC_040254.1	97235283	A	G
NC_040254.1	97291596	A	T
NC_040254.1	97352905	C	T
NC_040254.1	97416083	C	T
NC_040254.1	97479535	C	A
NC_040254.1	97535160	G	T
NC_040254.1	97590727	C	G
NC_040254.1	97658133	T	C
NC_040254.1	97715616	A	C
NC_040254.1	97772089	T	C
NC_040254.1	97836685	T	C
NC_040254.1	97893040	A	G
NC_040254.1	97948888	T	G
NC_040254.1	98174324	C	G
NC_040254.1	98233172	A	G
NC_040254.1	98289722	C	T
NC_040254.1	98348524	G	C
NC_040254.1	98406486	A	T
NC_040254.1	98462423	A	C
NC_040254.1	98518073	C	G
NC_040254.1	98577470	C	A
NC_040254.1	98643515	G	A
NC_040254.1	98702569	A	G
NC_040254.1	98768427	T	C
NC_040254.1	98825286	G	A
NC_040254.1	98901018	C	T
NC_040254.1	98960696	T	G
NC_040254.1	99019672	G	A
NC_040254.1	99076589	A	G
NC_040254.1	99135264	A	G
NC_040254.1	99200970	A	G
NC_040254.1	99263004	T	A
NC_040254.1	99320803	A	G
NC_040254.1	99384147	G	A
NC_040254.1	99440271	A	G

NC_040254.1	99497447	A	G
NC_040254.1	99554466	A	C
NC_040254.1	99611098	G	A
NC_040254.1	99670080	C	A
NC_040254.1	99731807	T	G
NC_040254.1	99793192	G	A
NC_040254.1	99849006	T	C
NC_040254.1	99911058	T	C
NC_040254.1	99971253	G	C
NC_040254.1	100030816	T	C
NC_040254.1	100084785	T	C
NC_040254.1	100140529	C	T
NC_040254.1	100196192	G	C
NC_040254.1	100258045	A	G
NC_040254.1	100322831	T	G
NC_040254.1	100379723	A	G
NC_040254.1	100434771	T	C
NC_040254.1	100491839	G	C
NC_040254.1	100549368	C	A
NC_040254.1	100609230	A	C
NC_040254.1	100670717	C	T
NC_040254.1	100793166	T	C
NC_040254.1	100861008	T	C
NC_040254.1	100922395	G	A
NC_040254.1	101094666	T	A
NC_040254.1	101212051	G	C
NC_040254.1	101268138	C	T
NC_040254.1	101326690	A	G
NC_040254.1	101385008	A	G
NC_040254.1	101440339	T	G
NC_040254.1	101500958	G	A
NC_040254.1	101565446	T	C
NC_040254.1	101631334	T	A
NC_040254.1	101691151	C	T
NC_040254.1	101747358	G	A
NC_040254.1	101803075	C	T
NC_040254.1	101864769	C	A
NC_040254.1	101926187	A	G
NC_040254.1	101986175	G	A
NC_040254.1	102050083	G	A
NC_040254.1	102111850	A	C
NC_040254.1	102172592	T	C
NC_040254.1	102236635	T	C
NC_040254.1	102294000	C	T
NC_040254.1	102347333	A	T
NC_040254.1	102414550	T	C
NC_040254.1	102489362	G	A

NC_040254.1	102545498	T	C
NC_040254.1	102602613	A	G
NC_040254.1	102658287	T	C
NC_040254.1	102717807	T	C
NC_040254.1	102785693	A	G
NC_040254.1	102850243	A	G
NC_040254.1	103086216	A	T
NC_040254.1	103113885	G	C
NC_040254.1	103291306	T	C
NC_040254.1	103308608	C	T
NC_040254.1	103413776	A	G
NC_040254.1	103478488	A	G
NC_040254.1	103544685	T	G
NC_040254.1	103606343	T	G
NC_040254.1	103664731	T	C
NC_040254.1	103775126	G	C
NC_040254.1	103833370	C	T
NC_040254.1	103893860	G	A
NC_040254.1	103953414	G	A
NC_040254.1	104010382	C	T
NC_040254.1	104069026	G	A
NC_040254.1	104125143	A	G
NC_040254.1	104183321	G	A
NC_040254.1	104239490	T	C
NC_040254.1	104299314	A	G
NC_040254.1	104361041	C	T
NC_040254.1	104422127	G	A
NC_040254.1	104486321	A	G
NC_040254.1	104541242	C	T
NC_040254.1	104606210	T	G
NC_040254.1	104666189	T	C
NC_040254.1	104729145	A	G
NC_040254.1	104797373	A	T
NC_040254.1	104860298	A	G
NC_040254.1	104928641	G	A
NC_040254.1	105052264	T	C
NC_040254.1	105082502	C	T
NC_040254.1	105147362	T	G
NC_040254.1	105218457	A	G
NC_040254.1	105282579	A	G
NC_040254.1	105344120	A	T
NC_040254.1	105407088	T	C
NC_040254.1	105467201	C	G
NC_040254.1	105529619	T	C
NC_040254.1	105592629	T	C
NC_040254.1	105649714	C	T
NC_040254.1	105677392	G	C

NC_040254.1	105727243	C	A
NC_040254.1	105795121	A	G
NC_040254.1	105873193	G	A
NC_040254.1	105992308	A	G
NC_040254.1	106050079	T	C
NC_040254.1	106111331	T	G
NC_040254.1	106167451	C	G
NC_040254.1	106229368	T	C
NC_040254.1	106285627	A	G
NC_040254.1	106344324	A	G
NC_040254.1	106404175	T	C
NC_040254.1	106462397	T	C
NC_040254.1	106519971	T	C
NC_040254.1	106582293	T	A
NC_040254.1	106639688	G	A
NC_040254.1	106700151	A	G
NC_040254.1	106760815	C	G
NC_040254.1	106822470	C	T
NC_040254.1	106897095	A	C
NC_040254.1	106959214	A	G
NC_040254.1	107082106	T	C
NC_040254.1	107137420	C	G
NC_040254.1	107190635	A	G
NC_040254.1	107266868	C	T
NC_040254.1	107336814	G	T
NC_040254.1	107407096	G	A
NC_040254.1	107423799	G	A
NC_040254.1	107487049	A	G
NC_040254.1	107541433	T	C
NC_040254.1	107600698	C	T
NC_040254.1	107660632	G	C
NC_040254.1	107715475	G	T
NC_040254.1	107769671	C	T
NC_040254.1	107832317	T	C
NC_040254.1	107892920	T	C
NC_040254.1	107955173	G	C
NC_040254.1	108021307	A	G
NC_040254.1	108100883	T	C
NC_040254.1	108178077	G	A
NC_040254.1	108239305	C	G
NC_040254.1	108305607	A	G
NC_040254.1	108368579	G	A
NC_040254.1	108499313	A	G
NC_040254.1	108566549	A	G
NC_040254.1	108627040	T	C
NC_040254.1	108685338	C	T
NC_040254.1	108741293	C	T

NC_040254.1	108798972	A	C
NC_040254.1	108855886	G	A
NC_040254.1	108919893	C	T
NC_040254.1	109100873	G	A
NC_040254.1	109166758	T	C
NC_040254.1	109223769	G	A
NC_040254.1	109295310	C	T
NC_040254.1	109340164	T	C
NC_040254.1	109396720	T	C
NC_040254.1	109454028	T	C
NC_040254.1	109518931	A	G
NC_040254.1	109573792	G	A
NC_040254.1	109631624	C	T
NC_040254.1	109686241	T	G
NC_040254.1	109749226	A	C
NC_040254.1	109807761	A	G
NC_040254.1	109869576	T	C
NC_040254.1	109984222	T	C
NC_040254.1	110038978	A	C
NC_040254.1	110097694	T	C
NC_040254.1	110156493	C	T
NC_040254.1	110222761	A	G
NC_040254.1	110276404	C	T
NC_040254.1	110344465	T	G
NC_040254.1	110404045	T	C
NC_040254.1	110467482	T	C
NC_040254.1	110539558	C	A
NC_040254.1	110595724	T	C
NC_040254.1	110654085	T	C
NC_040254.1	110711223	G	A
NC_040254.1	110777318	T	C
NC_040254.1	110835859	A	G
NC_040254.1	110892788	G	A
NC_040254.1	110952113	T	C
NC_040254.1	111008424	T	G
NC_040254.1	111067410	A	G
NC_040254.1	111140149	C	T
NC_040254.1	111270561	T	C
NC_040254.1	111304013	A	G
NC_040254.1	111348729	A	G
NC_040254.1	111429749	G	A
NC_040254.1	111484652	C	A
NC_040254.1	111550442	C	T
NC_040254.1	111660447	G	A
NC_040254.1	111771520	T	C
NC_040254.1	111830174	T	C
NC_040254.1	111887670	T	C

NC_040254.1	111943852	T	C
NC_040254.1	112004090	G	A
NC_040254.1	112066150	G	T
NC_040254.1	112123723	A	G
NC_040254.1	112179832	G	A
NC_040254.1	112239482	A	G
NC_040254.1	112299116	T	C
NC_040254.1	112356348	A	T
NC_040254.1	112411820	G	A
NC_040254.1	112587633	C	A
NC_040254.1	112644061	T	C
NC_040254.1	112699696	G	C
NC_040254.1	113000799	G	A
NC_040254.1	113057853	T	C
NC_040254.1	113118314	T	G
NC_040254.1	113180071	A	G
NC_040254.1	113225475	A	G
NC_040254.1	113813444	A	G
NC_040254.1	113868368	T	C
NC_040254.1	113932630	G	T
NC_040254.1	113994394	C	A
NC_040254.1	114057564	G	A
NC_040254.1	114111140	A	G
NC_040254.1	114165256	G	C
NC_040254.1	114227555	T	C
NC_040254.1	114288654	T	G
NC_040254.1	114348696	A	G
NC_040254.1	114410958	C	T
NC_040254.1	114468726	T	C
NC_040254.1	114533415	T	C
NC_040254.1	114773207	C	G
NC_040254.1	114833256	C	T
NC_040254.1	114894280	C	T
NC_040254.1	114956352	G	A
NC_040254.1	115011765	A	T
NC_040254.1	115138509	A	G
NC_040254.1	115230794	A	G
NC_040254.1	115312033	G	A
NC_040254.1	115427045	G	C
NC_040254.1	115671712	T	C
NC_040254.1	115801286	C	A
NC_040254.1	115867396	C	G
NC_040254.1	115996788	G	A
NC_040254.1	116054660	T	C
NC_040254.1	116126520	A	C
NC_040254.1	116183254	A	G
NC_040254.1	116276274	A	G

NC_040254.1	116492963	G	A
NC_040254.1	116995736	A	G
NC_040254.1	117065086	A	G
NC_040254.1	117230880	T	A
NC_040254.1	117258294	A	T
NC_040254.1	117313005	A	G
NC_040254.1	117375968	T	G
NC_040254.1	117442864	C	T
NC_040254.1	117508562	C	T
NC_040254.1	117573931	G	A
NC_040254.1	117637207	A	G
NC_040254.1	117693770	T	C
NC_040254.1	117816016	C	T
NC_040254.1	117936945	G	T
NC_040254.1	118034721	A	G
NC_040254.1	118146192	T	A
NC_040254.1	118228172	C	T
NC_040254.1	118275769	T	C
NC_040254.1	118307600	A	T
NC_040254.1	118372690	A	G
NC_040254.1	118433333	C	T
NC_040254.1	118489883	A	G
NC_040254.1	118552916	T	G
NC_040254.1	118675782	A	C
NC_040254.1	118737552	C	T
NC_040254.1	118799783	C	T
NC_040254.1	118855188	C	T
NC_040254.1	118919773	C	T
NC_040254.1	119042794	T	G
NC_040254.1	119101858	C	T
NC_040254.1	119163036	A	G
NC_040254.1	119216823	T	C
NC_040254.1	119274799	A	G
NC_040254.1	119334993	A	C
NC_040254.1	119431232	C	T
NC_040254.1	119462171	A	G
NC_040254.1	119513386	G	A
NC_040254.1	119581633	A	G
NC_040254.1	119642069	A	C
NC_040254.1	119704701	C	A
NC_040254.1	119820399	T	G
NC_040254.1	119879249	G	A
NC_040254.1	119935639	C	T
NC_040254.1	120001663	T	A
NC_040254.1	120068131	T	C
NC_040254.1	120134911	T	C
NC_040254.1	120205538	T	C

NC_040254.1	120274983	A	C
NC_040254.1	120337390	T	G
NC_040254.1	120384589	A	G
NC_040254.1	120426004	C	T
NC_040254.1	120476419	G	A
NC_040254.1	120535656	C	T
NC_040254.1	120584984	G	A
NC_040254.1	120710211	A	G
NC_040254.1	120774615	G	A
NC_040254.1	120956693	A	G
NC_040254.1	120977999	T	C
NC_040254.1	121032152	T	C
NC_040254.1	121085717	C	T
NC_040254.1	121142099	G	A
NC_040254.1	121198965	G	A
NC_040254.1	121255723	T	C
NC_040254.1	121318491	C	A
NC_040254.1	121381551	G	A
NC_040254.1	121439209	C	A
NC_040254.1	121505350	C	T
NC_040254.1	121580922	C	T
NC_040254.1	121643492	G	T
NC_040254.1	121943569	C	T
NC_040254.1	121961434	A	T
NC_040254.1	122169533	T	C
NC_040254.1	122227361	G	A
NC_040254.1	122287551	G	A
NC_040254.1	122347533	G	A
NC_040254.1	122479232	G	T
NC_040254.1	122532557	T	C
NC_040254.1	122610024	T	A
NC_040254.1	122682610	G	A
NC_040254.1	122743647	T	C
NC_040254.1	122804390	T	C
NC_040254.1	122866182	A	G
NC_040254.1	122926266	G	A
NC_040254.1	122988384	G	A
NC_040254.1	123116689	T	C
NC_040254.1	123907808	G	C
NC_040254.1	123962525	T	C
NC_040254.1	124023578	C	T
NC_040254.1	124077800	A	G
NC_040254.1	124137972	A	C
NC_040254.1	124197725	T	G
NC_040254.1	124262614	T	C
NC_040254.1	124279703	A	T
NC_040254.1	124553493	C	A

NC_040254.1	124615119	A	G
NC_040254.1	124677495	T	C
NC_040254.1	124736873	C	T
NC_040254.1	124804984	T	C
NC_040254.1	124873325	C	T
NC_040254.1	124935840	T	G
NC_040254.1	124999994	T	C
NC_040254.1	125054113	A	G
NC_040254.1	125117835	C	G
NC_040254.1	125191430	T	G
NC_040254.1	125251244	T	C
NC_040254.1	125312440	T	C
NC_040254.1	125371895	G	A
NC_040254.1	125431969	C	T
NC_040254.1	125509864	G	A
NC_040254.1	125571719	A	G
NC_040254.1	125633664	G	A
NC_040254.1	125689818	C	T
NC_040254.1	125749941	T	C
NC_040254.1	125817663	A	G
NC_040254.1	125893035	A	C
NC_040254.1	125976112	A	G
NC_040254.1	126028867	A	G
NC_040254.1	126098148	C	T
NC_040254.1	126152730	G	C
NC_040254.1	126226343	G	C
NC_040254.1	126274132	T	A
NC_040254.1	126337026	G	A
NC_040254.1	126404988	C	T
NC_040254.1	126471026	T	C
NC_040254.1	126532518	A	G
NC_040254.1	126653424	T	C
NC_040254.1	126707532	C	T
NC_040254.1	126778437	A	T
NC_040254.1	126843943	C	G
NC_040254.1	126902151	G	T
NC_040254.1	126927447	T	G
NC_040254.1	126978855	T	C
NC_040254.1	127159928	T	C
NC_040254.1	127217852	G	A
NC_040254.1	127333621	C	G
NC_040254.1	127393166	C	T
NC_040254.1	127513983	G	T
NC_040254.1	127574527	A	C
NC_040254.1	127899206	G	C
NC_040254.1	128229683	G	C
NC_040254.1	128292858	T	C

NC_040254.1	128464983	C	G
NC_040254.1	128557010	C	T
NC_040254.1	128743091	A	G
NC_040254.1	128822137	C	A
NC_040254.1	128883432	G	A
NC_040254.1	129000551	A	G
NC_040254.1	129061578	T	C
NC_040254.1	129206981	C	G
NC_040254.1	129596860	A	G
NC_040254.1	129655575	T	C
NC_040254.1	129720385	T	G
NC_040254.1	129778098	T	C
NC_040254.1	129842212	C	T
NC_040254.1	129928381	T	G
NC_040254.1	129985916	A	C
NC_040254.1	130046207	T	C
NC_040254.1	130105542	T	C
NC_040254.1	130172790	T	C
NC_040254.1	130235973	T	C
NC_040254.1	130301830	G	A
NC_040254.1	130428217	C	T
NC_040254.1	130705539	G	A
NC_040254.1	130773175	A	G
NC_040254.1	130849449	T	C
NC_040254.1	130906818	A	C
NC_040254.1	130982757	G	T
NC_040254.1	131102294	C	G
NC_040254.1	131226245	G	A
NC_040254.1	131284564	C	A
NC_040254.1	131459990	C	T
NC_040254.1	131537239	C	T
NC_040254.1	131606688	A	T
NC_040254.1	131668284	C	A
NC_040254.1	131802028	T	C
NC_040254.1	131987278	A	T
NC_040254.1	132276704	C	T
NC_040254.1	132649618	T	C
NC_040254.1	132751260	A	C
NC_040254.1	132811454	G	C
NC_040254.1	132870999	G	A
NC_040254.1	132927963	C	T
NC_040254.1	132980271	T	G
NC_040254.1	133070309	A	G
NC_040254.1	133127681	A	G
NC_040254.1	133194735	C	T
NC_040254.1	133254902	C	T
NC_040254.1	133325840	T	C

NC_040254.1	133383315	C	A
NC_040254.1	133456737	C	T
NC_040254.1	133519194	T	G
NC_040254.1	133585209	A	G
NC_040254.1	133649798	T	C
NC_040254.1	133703734	T	C
NC_040254.1	133761951	C	A
NC_040254.1	133830174	T	C
NC_040254.1	133894391	C	T
NC_040254.1	133966910	A	G
NC_040254.1	133979604	T	A
NC_040254.1	134096189	0	G
NC_040254.1	134148250	A	G
NC_040254.1	134210805	C	T
NC_040254.1	134268021	T	C
NC_040254.1	134335753	A	G
NC_040254.1	134393224	T	G
NC_040254.1	134459451	C	T
NC_040254.1	134517530	G	A
NC_040254.1	134632092	C	T
NC_040254.1	134696297	G	A
NC_040254.1	134752088	T	C
NC_040254.1	134810118	C	A
NC_040254.1	134865804	T	G
NC_040254.1	134922019	G	A
NC_040254.1	134981115	G	A
NC_040254.1	135040748	T	C
NC_040254.1	135100725	A	G
NC_040254.1	135159958	G	A
NC_040254.1	135225345	A	G
NC_040254.1	135294778	T	G
NC_040254.1	135360836	T	G
NC_040254.1	135417518	C	T
NC_040254.1	135483867	G	T
NC_040254.1	135541084	T	C
NC_040254.1	135598324	C	T
NC_040254.1	135658555	C	A
NC_040254.1	135718889	C	T
NC_040254.1	135783544	G	A
NC_040254.1	135848989	C	T
NC_040254.1	135911408	A	G
NC_040254.1	135968758	T	C
NC_040254.1	136037599	T	C
NC_040254.1	136098941	A	G
NC_040254.1	136220966	T	C
NC_040254.1	136274751	G	A
NC_040254.1	136341653	T	G

NC_040254.1	136397694	C	T
NC_040254.1	136464148	C	T
NC_040254.1	136516262	T	C
NC_040254.1	136574468	T	C
NC_040254.1	136665828	C	G
NC_040254.1	136683778	C	G
NC_040254.1	136739331	T	C
NC_040254.1	136809414	C	G
NC_040254.1	136866726	G	A
NC_040254.1	136926406	A	G
NC_040254.1	136991271	G	A
NC_040254.1	137046562	A	C
NC_040254.1	137103092	G	A
NC_040254.1	137166921	G	A
NC_040254.1	137223184	A	G
NC_040254.1	137278623	C	T
NC_040254.1	137341215	C	T
NC_040254.1	137396869	A	G
NC_040254.1	137461491	T	C
NC_040254.1	137519831	A	G
NC_040254.1	137587232	C	T
NC_040254.1	137642533	T	G
NC_040254.1	137702113	A	T
NC_040254.1	137756058	A	G
NC_040254.1	137826295	A	G
NC_040254.1	137891193	T	G
NC_040254.1	137949235	T	C
NC_040254.1	138008893	G	A
NC_040254.1	138063380	C	T
NC_040254.1	138127725	A	G
NC_040254.1	138202641	A	G
NC_040254.1	138258291	A	G
NC_040254.1	138323795	G	A
NC_040254.1	138438157	T	A
NC_040254.1	138502722	T	C
NC_040254.1	138612679	C	T
NC_040254.1	138673380	A	C
NC_040254.1	138730089	C	T
NC_040254.1	138788646	G	T
NC_040254.1	138851455	A	G
NC_040254.1	138906680	A	G
NC_040254.1	138968396	T	G
NC_040254.1	139026290	T	C
NC_040254.1	139088655	G	T
NC_040254.1	139148782	C	T
NC_040254.1	139260568	T	C
NC_040254.1	139341361	A	T

NC_040254.1	139416520	T	A
NC_040254.1	139526973	T	C
NC_040254.1	139583102	T	C
NC_040254.1	139639251	T	C
NC_040254.1	139695956	G	A
NC_040254.1	139815284	G	A
NC_040254.1	139882626	T	C
NC_040254.1	139941015	T	C
NC_040254.1	139999110	G	A
NC_040254.1	140056642	T	C
NC_040254.1	140116976	A	G
NC_040254.1	140175550	C	T
NC_040254.1	140243285	T	C
NC_040254.1	140301038	T	C
NC_040254.1	140358308	A	C
NC_040254.1	140423399	G	T
NC_040254.1	140539476	A	G
NC_040254.1	140598117	G	A
NC_040254.1	140661938	T	C
NC_040254.1	140729939	T	C
NC_040254.1	140785982	G	T
NC_040254.1	140842769	A	G
NC_040254.1	140898291	A	G
NC_040254.1	140959510	A	G
NC_040254.1	141076299	T	C
NC_040254.1	141135744	T	C
NC_040254.1	141201949	A	G
NC_040254.1	141325105	T	C
NC_040254.1	141452805	T	C
NC_040254.1	141569936	C	G
NC_040254.1	141630610	C	G
NC_040254.1	141689015	T	C
NC_040254.1	141747979	T	C
NC_040254.1	141805865	T	G
NC_040254.1	141859706	A	G
NC_040254.1	141919328	A	C
NC_040254.1	141977080	A	G
NC_040254.1	142035410	A	G
NC_040254.1	142090792	T	C
NC_040254.1	142150612	T	C
NC_040254.1	142213627	C	T
NC_040254.1	142270293	C	T
NC_040254.1	142329660	T	C
NC_040254.1	142391546	G	T
NC_040254.1	142449248	G	A
NC_040254.1	142510738	A	C
NC_040254.1	142566542	C	T

NC_040254.1	142622643	A	G
NC_040254.1	142682118	G	C
NC_040254.1	142739189	T	C
NC_040254.1	142793127	T	C
NC_040254.1	142850086	T	C
NC_040254.1	142911572	T	C
NC_040254.1	142967042	G	A
NC_040254.1	143029214	T	A
NC_040254.1	143096491	A	T
NC_040254.1	143150980	A	G
NC_040254.1	143210938	C	A
NC_040254.1	143268491	C	A
NC_040254.1	143323316	A	G
NC_040254.1	143385831	T	C
NC_040254.1	143449263	T	C
NC_040254.1	143500935	C	T
NC_040254.1	143559501	C	G
NC_040254.1	143620799	T	C
NC_040254.1	143683501	A	G
NC_040254.1	143767367	G	A
NC_040254.1	143824299	A	G
NC_040254.1	143879401	A	G
NC_040254.1	143937970	A	G
NC_040254.1	143996135	C	T
NC_040254.1	144056864	A	G
NC_040254.1	144118028	A	G
NC_040254.1	144234083	A	T
NC_040254.1	144294842	T	C
NC_040254.1	144357525	A	G
NC_040254.1	144470131	A	G
NC_040254.1	144524862	C	A
NC_040254.1	144587039	G	T
NC_040254.1	144644056	A	T
NC_040254.1	144703709	G	A
NC_040254.1	144820205	T	C
NC_040254.1	144935469	G	T
NC_040254.1	145008151	C	A
NC_040254.1	145065899	T	A
NC_040254.1	145124883	T	C
NC_040254.1	145187729	T	C
NC_040254.1	145249687	C	T
NC_040254.1	145330386	A	T
NC_040254.1	145386708	T	G
NC_040254.1	145454880	T	C
NC_040254.1	145625464	T	C
NC_040254.1	145681638	C	T
NC_040254.1	145753237	G	A

NC_040254.1	145931590	T	G
NC_040254.1	145996118	G	T
NC_040254.1	146054117	G	A
NC_040254.1	146113847	A	G
NC_040254.1	146168372	C	G
NC_040254.1	146226084	A	G
NC_040254.1	146289698	T	C
NC_040254.1	146352864	G	A
NC_040254.1	146410158	C	G
NC_040254.1	146466240	A	C
NC_040254.1	146523140	T	C
NC_040254.1	146580527	T	C
NC_040254.1	146639577	A	C
NC_040254.1	146701799	T	A
NC_040254.1	146757654	C	T
NC_040254.1	146817806	T	C
NC_040254.1	146884271	T	C
NC_040254.1	146942777	T	C
NC_040254.1	147000309	G	A
NC_040254.1	147058040	T	C
NC_040254.1	147116025	A	G
NC_040254.1	147174724	G	A
NC_040254.1	147233991	C	A
NC_040254.1	147297812	T	C
NC_040254.1	147352605	C	T
NC_040254.1	147405361	T	C
NC_040254.1	147464789	C	T
NC_040254.1	147530410	T	C
NC_040254.1	147621814	C	T
NC_040254.1	147692210	G	A
NC_040254.1	147810388	A	G
NC_040254.1	147835296	G	C
NC_040254.1	148194033	C	T
NC_040254.1	148499134	T	A
NC_040254.1	148559965	G	A
NC_040254.1	148617952	C	T
NC_040254.1	148673745	T	G
NC_040254.1	148728828	A	C
NC_040254.1	148785845	A	G
NC_040254.1	148847623	T	C
NC_040254.1	148901289	A	G
NC_040254.1	148960668	A	G
NC_040254.1	149024374	A	G
NC_040254.1	149153961	T	C
NC_040254.1	149212306	A	G
NC_040254.1	149272336	G	C
NC_040254.1	149331018	C	G

NC_040254.1	149395598	T	G
NC_040254.1	149449490	T	C
NC_040254.1	149514150	A	G
NC_040254.1	149553316	C	T
NC_040254.1	149610823	A	G
NC_040254.1	149668312	T	G
NC_040254.1	149713630	T	A
NC_040254.1	149751679	T	A
NC_040254.1	149933972	C	T
NC_040254.1	149988052	A	G
NC_040254.1	150049482	A	G
NC_040254.1	150105211	T	C
NC_040254.1	150157865	A	G
NC_040254.1	150225417	G	C
NC_040254.1	150284297	G	A
NC_040254.1	150340828	T	G
NC_040254.1	150399661	T	C
NC_040254.1	150510151	T	G
NC_040254.1	150571526	G	A
NC_040254.1	150642878	C	T
NC_040254.1	150695340	C	A
NC_040254.1	150751959	A	T
NC_040254.1	150811931	G	A
NC_040254.1	150866787	C	G
NC_040254.1	150930757	G	T
NC_040254.1	150995413	T	C
NC_040254.1	151055582	G	A
NC_040254.1	151115372	G	A
NC_040254.1	151182512	C	T
NC_040254.1	151234093	A	G
NC_040254.1	151306763	G	A
NC_040254.1	151405841	T	C
NC_040254.1	151483228	A	G
NC_040254.1	151546196	C	T
NC_040254.1	151602446	T	C
NC_040254.1	151666197	C	T
NC_040254.1	151782909	A	G
NC_040254.1	151903828	G	A
NC_040254.1	151961916	G	A
NC_040254.1	152030861	C	T
NC_040254.1	152099887	C	A
NC_040254.1	152162843	T	C
NC_040254.1	152226314	A	G
NC_040254.1	152278225	G	A
NC_040254.1	152338306	G	A
NC_040254.1	152393460	A	G
NC_040254.1	152457400	A	G

NC_040254.1	152519450	T	C
NC_040254.1	152582696	T	C
NC_040254.1	152645835	A	G
NC_040254.1	152706916	A	G
NC_040254.1	152763121	C	T
NC_040254.1	152824495	T	C
NC_040254.1	152883348	C	T
NC_040254.1	152953366	T	A
NC_040254.1	153017734	T	C
NC_040254.1	153079716	T	C
NC_040254.1	153136883	G	A
NC_040254.1	153201921	C	T
NC_040254.1	153270642	A	T
NC_040254.1	153335122	T	C
NC_040254.1	153390485	G	T
NC_040254.1	153451287	C	T
NC_040254.1	153518350	T	C
NC_040254.1	153577979	G	A
NC_040254.1	153642045	C	G
NC_040254.1	153704801	A	G
NC_040254.1	153768655	A	T
NC_040254.1	153837335	G	A
NC_040254.1	153955271	T	C
NC_040254.1	154019445	T	C
NC_040254.1	154074404	A	G
NC_040254.1	154137311	T	G
NC_040254.1	154213399	T	G
NC_040254.1	154376872	G	A
NC_040254.1	154444622	G	A
NC_040254.1	154519747	C	T
NC_040254.1	154582080	T	G
NC_040254.1	154651682	A	C
NC_040254.1	154762084	T	C
NC_040254.1	154823173	T	C
NC_040254.1	154879734	A	C
NC_040254.1	154940192	T	A
NC_040254.1	154998422	T	C
NC_040254.1	155062356	G	C
NC_040254.1	155117878	A	G
NC_040254.1	155181504	T	A
NC_040254.1	155437852	G	T
NC_040254.1	155733224	A	G
NC_040254.1	155808169	A	C
NC_040254.1	155868470	T	C
NC_040254.1	155935771	T	C
NC_040254.1	155963184	C	T
NC_040254.1	156024441	T	A

NC_040254.1	156095442	G		A
NC_040254.1	156157737	A		G
NC_040254.1	156231445	G		A
NC_040254.1	156283389	T		G
NC_040254.1	156338978	T		C
NC_040254.1	156400175	C		G
NC_040254.1	156456900	C		T
NC_040254.1	156530009	A		C
NC_040254.1	156594653	A		T
NC_040254.1	156667292	A		G
NC_040254.1	156688209	G		A
NC_040254.1	156743662	G		A
NC_040254.1	156804292	C		T
NC_040254.1	156864727	G		C
NC_040254.1	156979557	G		A
NC_040254.1	157046813	A		C
NC_040254.1	157099235	G		T
NC_040254.1	157123805	T		G
NC_040254.1	157194645	A		G
NC_040254.1	157384444	C		T
NC_040254.1	157457446	G		A
NC_040254.1	157518614	C		G
NC_040254.1	157585830	A		T
NC_040254.1	157649607	G		A
NC_040254.1	157762348	A		T
NC_040254.1	157828658	C		A
NC_040254.1	157886146		0	C
NC_040254.1	157943866	T		A
NC_040254.1	158004589	C		T
NC_040254.1	158060984	C		A
NC_040254.1	158123596	G		A
NC_040254.1	158173461	A		G
NC_040254.1	158217638	T		A
NC_040254.1	158285377	A		G
NC_040254.1	158384919	A		C
NC_040254.1	158567844	A		C
NC_040254.1	158622420	C		T
NC_040254.1	158683596	T		C
NC_040254.1	158745907	T		C
NC_040254.1	158806838	A		G
NC_040254.1	158870252	G		A
NC_040254.1	158939405	A		G
NC_040254.1	159011056	A		G
NC_040254.1	159078905	A		G
NC_040254.1	159244233	T		C
NC_040254.1	159429546	A		G
NC_040254.1	159518950	T		C

NC_040254.1	159581605	A	C
NC_040254.1	159646195	G	A
NC_040254.1	159798590	C	T
NC_040254.1	159866163	T	C
NC_040254.1	159900371	A	G
NC_040254.1	159963206	G	T
NC_040254.1	159999182	G	A
NC_040254.1	160054357	T	C
NC_040254.1	160118639	A	G
NC_040254.1	160180908	A	C
NC_040254.1	160240074	C	T
NC_040254.1	160296495	G	C
NC_040254.1	160357705	C	T
NC_040254.1	160417556	T	A
NC_040254.1	160473813	A	G
NC_040254.1	160534263	T	C
NC_040254.1	160594277	T	A
NC_040254.1	160620889	C	A
NC_040254.1	160682509	T	C
NC_040254.1	160750843	T	C
NC_040254.1	160807725	T	C
NC_040254.1	160937810	T	C
NC_040254.1	161014401	C	T
NC_040254.1	161071656	C	T
NC_040254.1	161133661	C	T
NC_040254.1	161194740	A	T
NC_040254.1	161258757	A	C
NC_040254.1	161315191	A	C
NC_040254.1	161378216	C	A
NC_040254.1	161441585	C	T
NC_040254.1	161547585	T	C
NC_040254.1	161624610	C	T
NC_040254.1	161682394	G	A
NC_040254.1	161736958	A	G
NC_040254.1	161799475	T	C
NC_040254.1	161866213	G	T
NC_040254.1	161916861	G	A
NC_040254.1	161944396	G	A
NC_040254.1	161998031	T	C
NC_040254.1	162060962	G	A
NC_040254.1	162121991	G	A
NC_040254.1	162257022	C	T
NC_040254.1	162314217	T	C
NC_040254.1	162384177	C	T
NC_040254.1	162446616	G	A
NC_040254.1	162502932	G	A
NC_040254.1	162566443	G	A

NC_040254.1	162628410	T	C
NC_040254.1	162698048	A	G
NC_040254.1	162756735	G	C
NC_040254.1	162817655	G	A
NC_040254.1	162879766	A	G
NC_040254.1	162942097	C	T
NC_040254.1	163009671	T	C
NC_040254.1	163128143	A	C
NC_040254.1	163187853	A	G
NC_040254.1	163250103	A	G
NC_040254.1	163316718	T	C
NC_040254.1	163386130	A	G
NC_040254.1	163578782	G	A
NC_040254.1	163706243	C	G
NC_040254.1	163764274	C	T
NC_040254.1	163822009	C	T
NC_040254.1	163881568	C	T
NC_040254.1	163953986	A	T
NC_040254.1	164074263	C	A
NC_040254.1	164133287	G	A
NC_040254.1	164194463	C	T
NC_040254.1	164273394	A	G
NC_040254.1	164337978	G	A
NC_040254.1	164396715	T	C
NC_040254.1	164463913	G	A
NC_040254.1	164528811	A	T
NC_040254.1	164604133	C	G
NC_040254.1	164660459	G	C
NC_040254.1	164717499	C	T
NC_040254.1	164776378	A	G
NC_040254.1	164837087	G	A
NC_040254.1	164894092	A	T
NC_040254.1	164952031	C	T
NC_040254.1	165013768	G	A
NC_040254.1	165069447	A	G
NC_040254.1	165131914	T	A
NC_040254.1	165191859	A	C
NC_040254.1	165255184	C	T
NC_040254.1	165312942	A	G
NC_040254.1	165369452	A	G
NC_040254.1	165426932	G	A
NC_040254.1	165486196	A	G
NC_040254.1	165546539	C	T
NC_040254.1	165608897	T	C
NC_040254.1	165665776	T	C
NC_040254.1	165725656	C	T
NC_040254.1	165786298	C	T

NC_040254.1	165842192	A	T
NC_040254.1	165899191	G	A
NC_040254.1	165955449	G	C
NC_040254.1	166024408	G	A
NC_040254.1	166085760	G	A
NC_040254.1	166156063	A	G
NC_040254.1	166215774	T	A
NC_040254.1	166346242	A	G
NC_040254.1	166406645	G	A
NC_040254.1	166478688	C	T
NC_040254.1	166536594	C	G
NC_040254.1	166595212	A	G
NC_040254.1	166655997	A	G
NC_040254.1	166717028	A	G
NC_040254.1	166779329	G	A
NC_040254.1	166837980	G	A
NC_040254.1	166897121	G	A
NC_040254.1	166953772	G	A
NC_040254.1	167014436	T	C
NC_040254.1	167072678	T	C
NC_040254.1	167131244	T	C
NC_040254.1	167195080	T	C
NC_040254.1	167372535	T	C
NC_040254.1	167437669	A	G
NC_040254.1	167515417	G	T
NC_040254.1	167637994	A	G
NC_040254.1	167706334	A	C
NC_040254.1	167779427	T	G
NC_040254.1	167847868	C	G
NC_040254.1	167910396	T	C
NC_040254.1	167971807	C	T
NC_040254.1	168032202	A	G
NC_040254.1	168087110	G	A
NC_040254.1	168143273	A	G
NC_040254.1	168204295	A	G
NC_040254.1	168263987	T	A
NC_040254.1	168323625	T	A
NC_040254.1	168389883	T	A
NC_040254.1	168466254	G	A
NC_040254.1	168513467	G	T
NC_040254.1	168568939	T	C
NC_040254.1	168813542	T	G
NC_040254.1	168898801	G	A
NC_040254.1	169072175	A	G
NC_040254.1	169127679	A	G
NC_040254.1	169196052	G	A
NC_040254.1	169255289	A	G

NC_040254.1	169339318	A	G
NC_040254.1	169403498	C	A
NC_040254.1	169474253	A	G
NC_040254.1	169535032	G	T
NC_040254.1	169622648	G	A
NC_040254.1	169674190	A	G
NC_040254.1	169749240	A	T
NC_040254.1	169825041	A	G
NC_040254.1	169956227	C	T
NC_040254.1	170098534	G	A
NC_040254.1	170212514	G	T
NC_040254.1	170283146	A	G
NC_040254.1	170353827	C	T
NC_040254.1	170421636	C	T
NC_040254.1	170485153	A	G
NC_040254.1	170682060	A	G
NC_040254.1	170809010	A	G
NC_040254.1	170828143	C	T
NC_040254.1	170943863	G	A
NC_040254.1	171004091	T	C
NC_040254.1	171062939	T	A
NC_040254.1	171139669	T	A
NC_040254.1	171199413	G	A
NC_040254.1	171267168	T	G
NC_040254.1	171325736	G	A
NC_040254.1	171392163	G	T
NC_040254.1	171461511	T	C
NC_040254.1	171594966	C	T
NC_040254.1	171648335	A	G
NC_040254.1	171723192	G	T
NC_040254.1	171829909	T	C
NC_040254.1	171850883	T	A
NC_040254.1	171958211	C	T
NC_040254.1	172017236	T	C
NC_040254.1	172073253	C	T
NC_040254.1	172133704	C	A
NC_040254.1	172204726	A	G
NC_040254.1	172324004	C	T
NC_040254.1	172444324	A	G
NC_040254.1	172509012	T	C
NC_040254.1	172575256	T	C
NC_040254.1	172661535	G	A
NC_040254.1	172721863	C	T
NC_040254.1	172793915	T	C
NC_040254.1	172849308	C	G
NC_040254.1	172962781	G	A
NC_040254.1	173088916	C	T

NC_040254.1	173173573	A	G
NC_040254.1	173420637	A	G
NC_040254.1	173477455	T	C
NC_040254.1	173533077	G	A
NC_040254.1	173596931	A	G
NC_040254.1	173654742	A	G
NC_040254.1	173709604	T	C
NC_040254.1	173763944	G	A
NC_040254.1	173823592	G	T
NC_040254.1	173882924	G	C
NC_040254.1	173939959	T	C
NC_040254.1	173995848	A	G
NC_040254.1	174053551	A	G
NC_040254.1	174113153	G	A
NC_040254.1	174174161	T	G
NC_040254.1	174231374	T	C
NC_040254.1	174288133	T	G
NC_040254.1	174344761	A	G
NC_040254.1	174400904	G	A
NC_040254.1	174461691	G	A
NC_040254.1	174520865	A	G
NC_040254.1	174579573	G	A
NC_040254.1	174639333	T	C
NC_040254.1	174701420	G	T
NC_040254.1	174762151	C	G
NC_040254.1	174817203	G	A
NC_040254.1	174878942	A	C
NC_040254.1	174937108	T	C
NC_040254.1	174992368	A	G
NC_040254.1	175056666	C	T
NC_040254.1	175199959	C	T
NC_040254.1	175256985	T	C
NC_040254.1	175317209	A	G
NC_040254.1	175373510	A	G
NC_040254.1	175432480	T	C
NC_040254.1	175637681	C	G
NC_040254.1	175694122	T	G
NC_040254.1	175750166	A	G
NC_040254.1	175807410	T	A
NC_040254.1	175935693	C	T
NC_040254.1	176513133	G	A
NC_040254.1	176629461	A	G
NC_040254.1	176690240	A	G
NC_040254.1	177011645	T	A
NC_040254.1	177158772	G	A
NC_040254.1	177208047	G	C
NC_040254.1	177263583	C	T

NC_040254.1	177340023	G	A
NC_040254.1	177417607	T	C
NC_040254.1	177482978	A	G
NC_040254.1	177542809	G	A
NC_040254.1	177604663	A	G
NC_040254.1	177664462	A	G
NC_040254.1	177720550	C	T
NC_040254.1	177780493	T	C
NC_040254.1	177840818	T	C
NC_040254.1	177896806	T	C
NC_040254.1	177953513	C	T
NC_040254.1	177980483	G	A
NC_040254.1	178036624	T	C
NC_040254.1	178093269	T	C
NC_040254.1	178151454	T	C
NC_040254.1	178209052	T	G
NC_040254.1	178274838	G	T
NC_040254.1	178337842	A	C
NC_040254.1	178398115	T	C
NC_040254.1	178461707	T	C
NC_040254.1	178527144	C	A
NC_040254.1	178581418	A	C
NC_040254.1	178648642	T	A
NC_040254.1	178709593	A	C
NC_040254.1	178767985	G	A
NC_040254.1	178831627	T	C
NC_040254.1	178887090	C	T
NC_040254.1	178947839	A	G
NC_040254.1	179003340	C	T
NC_040254.1	179065171	A	C
NC_040254.1	179123510	T	C
NC_040254.1	179179178	C	G
NC_040254.1	179243022	A	G
NC_040254.1	179296059	T	C
NC_040254.1	179318821	C	T
NC_040254.1	179372558	C	T
NC_040254.1	179439446	T	C
NC_040254.1	179458721	T	C
NC_040254.1	179525499	T	C
NC_040254.1	179592196	G	A
NC_040254.1	179708820	T	C
NC_040254.1	179774213	G	A
NC_040254.1	179834112	A	G
NC_040254.1	179892747	C	T
NC_040254.1	179947003	A	G
NC_040254.1	180008847	G	A
NC_040254.1	180071013	G	A

NC_040254.1	180132766	T	C
NC_040254.1	180189701	G	A
NC_040254.1	180253841	A	G
NC_040254.1	180319321	A	G
NC_040254.1	180387937	G	A
NC_040254.1	180451708	C	G
NC_040254.1	180507504	A	G
NC_040254.1	180572007	C	G
NC_040254.1	180686565	T	C
NC_040254.1	180748417	C	T
NC_040254.1	180809439	A	G
NC_040254.1	180995492	T	C
NC_040254.1	181073911	G	T
NC_040254.1	181321081	C	T
NC_040254.1	181392410	C	A
NC_040254.1	181449914	A	G
NC_040254.1	181505320	A	G
NC_040254.1	181563827	C	G
NC_040254.1	181621652	T	C
NC_040254.1	181638088	A	G
NC_040254.1	181701110	A	C
NC_040254.1	181763680	T	C
NC_040254.1	181937826	A	C
NC_040254.1	182133653	G	T
NC_040254.1	182190807	C	T
NC_040254.1	182246857	A	C
NC_040254.1	182304009	A	G
NC_040254.1	182374365	T	C
NC_040254.1	182439220	A	G
NC_040254.1	182506711	T	A
NC_040254.1	182562537	C	T
NC_040254.1	182619263	G	C
NC_040254.1	182696204	T	C
NC_040254.1	182749615	T	G
NC_040254.1	182806829	T	C
NC_040254.1	182870232	A	G
NC_040254.1	182933329	C	T
NC_040254.1	182952956	A	T
NC_040254.1	183743322	A	G
NC_040254.1	184453108	C	A
NC_040254.1	185002174	T	G
NC_040254.1	185481902	C	T
NC_040254.1	185866150	C	G
NC_040254.1	186242618	G	A
NC_040254.1	186299147	A	C
NC_040254.1	186352593	T	C
NC_040254.1	186412270	C	T

NC_040254.1	186468289	T	C
NC_040254.1	186531855	T	C
NC_040254.1	186587731	A	G
NC_040254.1	186649891	A	G
NC_040254.1	186713839	A	G
NC_040254.1	186769765	A	T
NC_040254.1	186831520	A	G
NC_040254.1	186892681	A	G
NC_040254.1	186955742	G	A
NC_040254.1	187008351	A	G
NC_040254.1	187066847	A	T
NC_040254.1	187122548	A	G
NC_040254.1	187189381	C	T
NC_040254.1	187259807	T	G
NC_040254.1	187601631	A	G
NC_040254.1	187788061	A	T
NC_040254.1	187910363	G	T
NC_040254.1	188049465	C	T
NC_040254.1	188442186	A	C
NC_040254.1	188718141	T	C
NC_040254.1	188918448	T	C
NC_040254.1	189044062	T	C
NC_040254.1	189302472	G	A
NC_040254.1	189918741	T	C
NC_040254.1	190255549	T	C
NC_040254.1	190563769	A	G
NC_040254.1	191248370	A	C
NC_040254.1	191428081	T	G
NC_040254.1	192185988	C	G
NC_040254.1	192841096	G	A
NC_040254.1	193028810	T	G
NC_040254.1	193244391	C	T
NC_040254.1	193481419	G	A
NC_040254.1	193517037	C	T
NC_040254.1	193615065	T	C
NC_040254.1	193653879	A	G
NC_040254.1	193721293	T	C
NC_040254.1	193767680	A	G
NC_040254.1	193838758	A	G
NC_040254.1	193870195	C	T
NC_040254.1	193906653	G	A
NC_040254.1	193944747	G	A
NC_040254.1	194027218	C	T
NC_040254.1	194049840	A	G
NC_040254.1	194089608	A	C
NC_040254.1	194147242	T	C
NC_040254.1	194247485	A	C

NC_040254.1	194347583	T	C
NC_040254.1	194383946	T	G
NC_040254.1	194425585	A	C
NC_040254.1	194565246	A	C
NC_040254.1	195240349	T	C
NC_040254.1	195505965	T	C
NC_040254.1	195537692	G	C
NC_040254.1	195591357	C	T
NC_040254.1	195682962	G	A
NC_040254.1	195720956	T	C
NC_040254.1	195761547	T	C
NC_040254.1	195966766	T	C
NC_040254.1	196695411	A	T
NC_040254.1	196739493	G	A
NC_040254.1	196770694	A	C
NC_040254.1	196789645	G	A
NC_040254.1	196828409	T	G
NC_040254.1	196860283	A	G
NC_040254.1	196899491	G	A
NC_040254.1	196926466	A	G
NC_040254.1	197087628	T	C
NC_040254.1	197123116	C	T
NC_040254.1	197255093	T	A
NC_040254.1	197382999	C	T
NC_040254.1	197571009	T	G
NC_040254.1	197644392	G	A
NC_040254.1	197753451	A	G
NC_040254.1	197857425	A	G
NC_040254.1	198166582	T	C
NC_040254.1	198222490	T	C
NC_040254.1	198284287	A	G
NC_040254.1	198342497	G	A
NC_040254.1	198407149	A	G
NC_040254.1	198478148	G	A
NC_040254.1	198549314	A	C
NC_040254.1	198666129	T	A
NC_040254.1	198798960	C	T
NC_040254.1	199186996	T	C
NC_040254.1	199323178	T	C
NC_040254.1	199521701	T	C
NC_040254.1	199574323	C	T
NC_040254.1	199648063	T	C
NC_040254.1	199827364	G	A
NC_040254.1	199911227	G	A
NC_040254.1	200081303	A	G
NC_040254.1	200169124	T	C
NC_040254.1	200260970	A	G

NC_040254.1	200304033	C	T
NC_040254.1	200350538	T	G
NC_040254.1	200411188	T	A
NC_040254.1	200461191	C	T
NC_040254.1	200748322	G	A
NC_040254.1	200813631	A	G
NC_040254.1	201013117	A	G
NC_040254.1	201092088	C	T
NC_040254.1	201179166	C	T
NC_040254.1	201223762	C	T
NC_040254.1	201287522	T	C
NC_040254.1	202056502	A	G
NC_040254.1	202140602	G	A
NC_040254.1	202454537	C	T
NC_040254.1	202498358	A	C
NC_040254.1	202604342	T	C
NC_040254.1	202703174	A	G
NC_040254.1	203615884	T	C
NC_040254.1	203746825	C	T
NC_040254.1	203877518	T	C
NC_040254.1	203997113	G	A
NC_040254.1	204108256	G	A
NC_040254.1	204398831	G	A
NC_040254.1	204435060	A	G
NC_040254.1	204473454	G	A
NC_040254.1	204509236	T	C
NC_040254.1	204608558	T	G
NC_040254.1	204731797	G	A
NC_040254.1	204851066	A	G
NC_040254.1	204906961	G	A
NC_040254.1	204936250	G	C
NC_040254.1	204969760	A	G
NC_040254.1	205006032	T	A
NC_040254.1	205120410	T	C
NC_040254.1	205151857	C	T
NC_040254.1	205187562	A	T
NC_040254.1	205266243	C	T
NC_040254.1	205307778	A	C
NC_040254.1	205342287	G	A
NC_040254.1	205459502	G	T
NC_040254.1	205572614	G	A
NC_040254.1	205754981	T	C
NC_040254.1	205817549	C	T
NC_040254.1	205876327	C	T
NC_040254.1	205927501	A	G
NC_040254.1	206018843	T	G
NC_040254.1	206177216	C	A

NC_040254.1	206388309	C		T
NC_040254.1	206587926	G		T
NC_040254.1	206723314	A		G
NC_040254.1	206754695	G		A
NC_040254.1	206875693	G		A
NC_040254.1	206986942	G		C
NC_040254.1	207182032	C		G
NC_040254.1	207295582	A		G
NC_040254.1	207607701	A		G
NC_040254.1	207789915	G		C
NC_040254.1	207898206	G		T
NC_040254.1	207999344	C		T
NC_040254.1	208661383	G		A
NC_040254.1	208695366	C		T
NC_040254.1	208743807	T		C
NC_040254.1	208785934	T		C
NC_040254.1	208933055	C		T
NC_040254.1	209610628	A		G
NC_040254.1	209698487	C		A
NC_040254.1	209815817	C		G
NC_040254.1	209951772	G		A
NC_040254.1	209981218	G		A
NC_040254.1	210048359	T		C
NC_040254.1	210441561	C		T
NC_040254.1	210769237	T		C
NC_040254.1	211068733	C		T
NC_040254.1	211229379	G		A
NC_040254.1	211353536	G		A
NC_040254.1	211703973	A		G
NC_040254.1	211864595	C		G
NC_040254.1	211978052	A		G
NC_040254.1	212168966	T		G
NC_040254.1	212280279	G		A
NC_040254.1	212571616	G		A
NC_040254.1	212715126	C		T
NC_040254.1	213127190	C		T
NC_040254.1	213313797		0	T
NC_040254.1	213376895	G		C
NC_040254.1	213425985	T		C
NC_040254.1	213515699	T		C
NC_040254.1	213562896	T		C
NC_040254.1	213633846	T		C
NC_040254.1	214092354		0	T
NC_040254.1	214237582	G		A
NC_040254.1	214323086	T		C
NC_040254.1	214423541	T		C
NC_040254.1	214650102	C		T

NC_040254.1	214772008	C	G
NC_040254.1	215049012	A	G
NC_040254.1	215090314	A	G
NC_040254.1	215143341	A	G
NC_040254.1	215214885	C	T
NC_040254.1	215393498	G	A
NC_040254.1	215549104	T	C
NC_040254.1	215636834	C	T
NC_040254.1	215689034	A	T
NC_040254.1	215774885	C	A
NC_040254.1	215928588	G	A
NC_040254.1	216124672	T	C
NC_040254.1	216172274	C	T
NC_040254.1	216205205	T	G
NC_040254.1	216233107	A	G
NC_040254.1	216330086	T	C
NC_040254.1	216354575	C	T
NC_040254.1	216380627	A	G
NC_040254.1	216410962	A	G
NC_040254.1	216436221	A	G
NC_040254.1	216467268	G	A
NC_040254.1	216509331	T	C
NC_040254.1	216604501	A	C
NC_040254.1	216689267	G	A
NC_040254.1	216756296	T	G
NC_040254.1	216805673	T	C
NC_040254.1	216872066	A	G
NC_040254.1	216960234	A	G
NC_040254.1	217015545	T	C
NC_040254.1	217182292	G	A
NC_040254.1	217219568	G	A
NC_040254.1	217390688	T	C
NC_040254.1	217414695	G	C
NC_040254.1	217443523	T	C
NC_040254.1	217504270	G	A
NC_040254.1	217519924	C	T
NC_040254.1	217589347	A	G
NC_040254.1	217610201	C	A
NC_040254.1	217634196	T	C
NC_040254.1	217670765	A	G
NC_040254.1	217696285	C	T
NC_040254.1	217732527	T	C
NC_040254.1	217831124	T	C
NC_040254.1	217901341	C	G
NC_040254.1	217935563	T	C
NC_040254.1	217959101	G	A
NC_040254.1	218007924	G	C

NC_040254.1	218040388	C	A
NC_040254.1	218069543	T	C
NC_040254.1	218131635	C	A
NC_040254.1	218227981	C	T
NC_040254.1	218275105	G	A
NC_040254.1	218309419	G	C
NC_040254.1	218332585	C	T
NC_040254.1	218357420	T	C
NC_040254.1	218434488	T	C
NC_040254.1	218502953	C	T
NC_040254.1	218513473	C	T
NC_040254.1	218594438	G	T
NC_040254.1	218772879	T	C
NC_040254.1	218913620	G	A
NC_040254.1	219026840	A	T
NC_040254.1	219121321	T	C
NC_040254.1	219218620	A	T
NC_040254.1	219256826	T	C
NC_040254.1	219314101	T	C
NC_040254.1	219769847	0	G
NC_040254.1	219875598	C	T
NC_040254.1	219971175	T	A
NC_040254.1	220095621	G	A
NC_040254.1	220262329	A	T
NC_040254.1	220319248	C	A
NC_040254.1	220365488	A	C
NC_040254.1	220417285	A	G
NC_040254.1	220721300	T	C
NC_040254.1	220747595	G	T
NC_040254.1	220773116	C	A
NC_040254.1	220798413	C	T
NC_040254.1	220867676	C	T
NC_040254.1	220928524	C	T
NC_040254.1	220944910	T	C
NC_040254.1	221175722	T	C
NC_040254.1	221200405	A	C
NC_040254.1	221242870	G	A
NC_040254.1	221264167	C	T
NC_040254.1	221287287	T	C
NC_040254.1	221329429	A	G
NC_040254.1	221347844	C	T
NC_040254.1	221827514	A	G
NC_040254.1	221856076	T	C
NC_040254.1	221900977	G	T
NC_040254.1	221944227	T	C
NC_040254.1	221972000	A	C
NC_040254.1	221993681	C	T

NC_040254.1	222014788	C	T
NC_040254.1	222021283	C	G
NC_040254.1	222049766	C	T
NC_040254.1	222272619	C	A
NC_040254.1	222600355	G	A
NC_040254.1	222630746	G	A
NC_040254.1	222676461	C	A
NC_040254.1	222738219	T	C
NC_040254.1	222765355	G	C
NC_040254.1	222802980	T	C
NC_040254.1	222875631	G	T
NC_040254.1	222913318	G	A
NC_040254.1	222926069	G	A
NC_040254.1	222963823	G	A
NC_040254.1	222978130	G	A
NC_040254.1	223932024	C	A
NC_040254.1	224001506	A	G
NC_040254.1	224015845	C	T
NC_040254.1	224042473	C	T
NC_040254.1	224101446	C	T
NC_040254.1	224150655	A	G
NC_040254.1	224203372	A	G
NC_040254.1	224224372	T	C
NC_040254.1	224260427	A	C
NC_040254.1	224292293	0	G
NC_040254.1	224296508	C	T
NC_040254.1	224346524	T	C
NC_040254.1	224356397	A	G
NC_040254.1	224367513	T	C
NC_040254.1	224438597	G	A
NC_040254.1	224486234	A	G
NC_040254.1	224536039	G	A
NC_040254.1	224539153	T	C
NC_040254.1	224563526	T	C
NC_040254.1	224617020	A	G
NC_040254.1	224666817	C	A
NC_040254.1	224684461	A	G
NC_040254.1	224686387	G	A
NC_040254.1	224700331	C	T
NC_040254.1	224747361	A	G
NC_040254.1	224768439	A	G
NC_040254.1	224792994	G	C
NC_040254.1	224830990	T	C
NC_040254.1	224887246	G	A
NC_040254.1	224928484	G	A
NC_040254.1	225003034	T	C
NC_040254.1	225051884	G	T

NC_040254.1	225108036	C	G
NC_040254.1	225133521	T	C
NC_040254.1	225171804	T	C
NC_040254.1	225229419	A	G
NC_040254.1	225250623	G	A
NC_040254.1	225276257	T	C
NC_040254.1	225328201	T	C
NC_040254.1	225383051	A	G
NC_040254.1	225472507	T	C
NC_040254.1	225486616	T	C
NC_040254.1	225543362	C	G
NC_040254.1	225582093	T	A
NC_040254.1	225614217	C	T
NC_040254.1	225694175	G	A
NC_040254.1	225719906	A	G
NC_040254.1	225764278	C	T
NC_040254.1	225826543	A	G
NC_040254.1	225878994	A	G
NC_040254.1	225919174	A	G
NC_040254.1	225939675	A	G
NC_040254.1	226007277	T	G
NC_040254.1	226057091	C	A
NC_040254.1	226115597	A	T
NC_040254.1	226172277	T	C
NC_040254.1	226176859	G	A
NC_040254.1	226219195	A	G
NC_040254.1	226265898	A	G
NC_040254.1	226312676	C	A
NC_040254.1	226397226	G	A
NC_040254.1	226461171	A	G
NC_040254.1	226476892	A	G
NC_040254.1	226515412	A	G
NC_040254.1	226529940	C	T
NC_040254.1	226566912	T	G
NC_040254.1	226585278	G	A
NC_040254.1	226642175	G	A
NC_040254.1	226703113	T	C
NC_040254.1	226759049	G	A
NC_040254.1	226808017	A	G
NC_040254.1	226836780	G	A
NC_040254.1	226855358	T	C
NC_040254.1	226898362	T	C
NC_040254.1	226945195	T	C
NC_040254.1	226980269	C	T
NC_040254.1	226997815	T	G
NC_040254.1	227019101	A	G
NC_040254.1	227037998	T	C

NC_040254.1	227058000	A	C
NC_040254.1	227082844	C	T
NC_040254.1	227108480	T	G
NC_040254.1	227142970	A	G
NC_040254.1	227202026	A	G
NC_040254.1	227262079	T	C
NC_040254.1	227311437	C	G
NC_040254.1	227358302	T	C
NC_040254.1	227380516	C	G
NC_040254.1	227419186	A	G
NC_040254.1	227455731	C	G
NC_040254.1	227458093	T	A
NC_040254.1	227497166	A	G
NC_040254.1	227515962	A	G
NC_040254.1	227560919	G	A
NC_040254.1	227569303	G	C
NC_040254.1	227630176	C	G
NC_040254.1	227666848	A	G
NC_040254.1	227716489	A	G
NC_040254.1	227765830	G	A
NC_040254.1	227771043	G	C
NC_040254.1	227826463	A	G
NC_040254.1	227869513	T	C
NC_040254.1	227880500	A	G
NC_040254.1	227952324	A	G
NC_040254.1	227959747	T	G
NC_040254.1	227961529	G	A
NC_040254.1	228023868	C	T
NC_040254.1	228044938	A	G
NC_040254.1	228080414	T	C
NC_040254.1	228136245	T	C
NC_040254.1	228188843	T	C
NC_040254.1	228211686	T	A
NC_040254.1	228252251	C	T
NC_040254.1	228256703	T	C
NC_040254.1	228313196	C	A
NC_040254.1	228347436	T	G
NC_040254.1	228368611	T	C
NC_040254.1	228418043	G	T
NC_040254.1	228443026	A	G
NC_040254.1	228475624	T	C
NC_040254.1	228521125	A	G
NC_040254.1	228585120	T	G
NC_040254.1	228615172	T	C
NC_040254.1	228637174	T	C
NC_040254.1	228699464	C	T
NC_040254.1	228754904	A	G

NC_040254.1	228814187	T	G
NC_040254.1	228874983	A	G
NC_040254.1	228948545	G	A
NC_040254.1	228998875	A	G
NC_040254.1	229042526	T	C
NC_040254.1	229074973	T	C
NC_040254.1	229133854	A	G
NC_040254.1	229162802	C	T
NC_040254.1	229254278	A	C
NC_040254.1	229297606	T	C
NC_040254.1	229308905	A	G
NC_040254.1	229359833	T	C
NC_040254.1	229363342	T	G
NC_040254.1	229387300	T	C
NC_040254.1	229420909	T	C
NC_040254.1	229442774	G	A
NC_040254.1	229471964	G	T
NC_040254.1	229478704	A	G
NC_040254.1	229510130	C	T
NC_040254.1	229532958	A	G
NC_040254.1	229577331	A	G
NC_040254.1	229582598	T	C
NC_040254.1	229616463	C	T
NC_040254.1	229635682	T	C
NC_040254.1	229638655	A	G
NC_040254.1	229650711	T	C
NC_040254.1	229672138	A	G
NC_040254.1	229682176	A	G
NC_040254.1	229737624	C	T
NC_040254.1	229768846	A	G
NC_040254.1	229796616	G	A
NC_040254.1	229824467	G	A
NC_040254.1	229846951	A	G
NC_040254.1	229888301	A	G
NC_040254.1	229897265	C	T
NC_040254.1	229955307	A	G
NC_040254.1	229973027	A	G
NC_040254.1	230002978	A	C
NC_040254.1	230025318	T	C
NC_040254.1	230079054	A	G
NC_040254.1	230123516	A	C
NC_040254.1	230143838	A	G
NC_040254.1	230173049	A	G
NC_040254.1	230194426	T	C
NC_040254.1	230226831	T	C
NC_040254.1	230254898	T	C
NC_040254.1	230270241	A	G

NC_040254.1	230299665	A	G
NC_040254.1	230322510	C	G
NC_040254.1	230351184	G	A
NC_040254.1	230358635	G	A
NC_040254.1	230380618	A	G
NC_040254.1	230387471	C	T
NC_040254.1	230450387	A	G
NC_040254.1	230462843	T	C
NC_040254.1	230466959	C	T
NC_040254.1	230507901	C	T
NC_040254.1	230528088	A	G
NC_040254.1	230548851	G	T
NC_040254.1	230591070	G	C
NC_040254.1	230598844	T	C
NC_040254.1	230651990	G	A
NC_040254.1	230689131	A	G
NC_040254.1	230696580	T	G
NC_040254.1	230729875	A	G
NC_040254.1	230733228	A	G
NC_040254.1	230766549	G	A
NC_040254.1	230818759	A	G
NC_040254.1	230882843	T	C
NC_040254.1	230885402	G	A
NC_040254.1	230889480	A	G
NC_040254.1	230950508	A	G
NC_040254.1	230950738	G	C
NC_040254.1	230951712	C	T
NC_040254.1	230952239	C	T
NC_040254.1	230964745	A	G
NC_040254.1	230965066	G	C
NC_040254.1	230998790	T	C
NC_040254.1	230999326	G	T
NC_040254.1	230999570	C	G
NC_040254.1	231000126	T	A
NC_040254.1	231053489	T	C
NC_040254.1	231115282	T	C
NC_040254.1	231115745	A	G
NC_040254.1	231148203	T	C
NC_040254.1	231157383	C	T
NC_040254.1	231160929	A	G
NC_040254.1	231161174	C	T
NC_040254.1	231162217	T	C
NC_040254.1	231167646	A	G
NC_040254.1	231168977	T	G
NC_040254.1	231169236	G	A
NC_040254.1	231171778	G	A
NC_040254.1	231181231	A	G

NC_040254.1	231182360	A	C
NC_040254.1	231187260	T	C
NC_040254.1	231200492	A	C
NC_040254.1	231200685	C	T
NC_040254.1	231200915	G	A
NC_040254.1	231201067	A	G
NC_040254.1	231229589	T	C
NC_040254.1	231229841	A	G
NC_040254.1	231254181	T	C
NC_040254.1	231291282	C	T
NC_040254.1	231292547	C	T
NC_040254.1	231293120	T	C
NC_040254.1	231345553	A	G
NC_040254.1	231402065	T	C
NC_040254.1	231419581	T	C
NC_040254.1	231419877	T	C
NC_040254.1	231420581	G	C
NC_040254.1	231473588	A	G
NC_040254.1	231500360	C	A
NC_040254.1	231557264	A	C
NC_040254.1	231604557	A	G
NC_040254.1	231629189	C	T
NC_040254.1	231632197	A	G
NC_040254.1	231632823	A	G
NC_040254.1	231638083	G	C
NC_040254.1	231694883	T	G
NC_040254.1	231735846	T	C
NC_040254.1	231736407	A	G
NC_040254.1	231737085	A	G
NC_040254.1	231739763	A	G
NC_040254.1	231764195	A	G
NC_040254.1	231765865	G	A
NC_040254.1	231766706	G	A
NC_040254.1	231766873	A	G
NC_040254.1	231767914	C	T
NC_040254.1	231770009	T	C
NC_040254.1	231771712	T	G
NC_040254.1	231778424	T	C
NC_040254.1	231826514	A	G
NC_040254.1	231865171	A	G
NC_040254.1	231891903	A	C
NC_040254.1	231909530	T	C
NC_040254.1	231914070	A	C
NC_040254.1	231970931	C	A
NC_040254.1	232014070	G	A
NC_040254.1	232045695	C	T
NC_040254.1	232101241	T	C

NC_040254.1	232156579	T	C
NC_040254.1	232183040	G	A
NC_040254.1	232218069	C	T
NC_040254.1	232232588	C	T
NC_040254.1	232232930	A	G
NC_040254.1	232288333	G	A
NC_040254.1	232341672	G	A
NC_040254.1	232398235	T	C
NC_040254.1	232431879	T	C
NC_040254.1	232432366	A	G
NC_040254.1	232482585	A	G
NC_040254.1	232489647	G	A
NC_040254.1	232490874	A	C
NC_040254.1	232492673	A	C
NC_040254.1	232493999	T	C
NC_040254.1	232512882	A	G
NC_040254.1	232584619	C	T
NC_040254.1	232586061	G	T
NC_040254.1	232586791	A	G
NC_040254.1	232591218	T	C
NC_040254.1	232647717	C	T
NC_040254.1	232648106	G	A
NC_040254.1	232691726	T	A
NC_040254.1	232693434	T	C
NC_040254.1	232693741	G	T
NC_040254.1	232694170	C	T
NC_040254.1	232694402	C	A
NC_040254.1	232745098	C	G
NC_040254.1	232796199	C	T
NC_040254.1	232834435	T	C
NC_040254.1	232884486	A	G
NC_040254.1	232946199	A	T
NC_040254.1	232998242	C	T
NC_040254.1	233051930	T	C
NC_040254.1	233093250	G	A
NC_040254.1	233121635	G	A
NC_040254.1	233142017	T	C
NC_040254.1	233168986	A	G
NC_040254.1	233197364	G	A
NC_040254.1	233203881	T	C
NC_040254.1	233205603	G	A
NC_040254.1	233229426	G	C
NC_040254.1	233231543	T	C
NC_040254.1	233246027	T	C
NC_040254.1	233246620	A	G
NC_040254.1	233247285	T	G
NC_040254.1	233253414	T	C

NC_040254.1	233253629	T	G
NC_040254.1	233269952	A	G
NC_040254.1	233286897	T	C
NC_040254.1	233352020	A	G
NC_040254.1	233353442	G	C
NC_040254.1	233353658	A	G
NC_040254.1	233379120	T	C
NC_040254.1	233379337	A	C
NC_040254.1	233380460	A	G
NC_040254.1	233380668	C	T
NC_040254.1	233382068	T	C
NC_040254.1	233403101	A	G
NC_040254.1	233403862	A	G
NC_040254.1	233404133	T	C
NC_040254.1	233404681	C	T
NC_040254.1	233404911	T	C
NC_040254.1	233405554	T	C
NC_040254.1	233420418	T	C
NC_040254.1	233486860	A	G
NC_040254.1	233508732	T	C
NC_040254.1	233541907	T	C
NC_040254.1	233562138	T	C
NC_040254.1	233591161	C	T
NC_040254.1	233625387	G	A
NC_040254.1	233625570	A	C
NC_040254.1	233631619	C	T
NC_040254.1	233671396	T	C
NC_040254.1	233677753	G	A
NC_040254.1	233685513	T	C
NC_040254.1	233696352	T	C
NC_040254.1	233746450	T	C
NC_040254.1	233748018	G	T
NC_040254.1	233781588	G	A
NC_040254.1	233785426	T	C
NC_040254.1	233800500	T	C
NC_040254.1	233867211	C	T
NC_040254.1	233868077	G	A
NC_040254.1	233868774	C	T
NC_040254.1	233931006	A	G
NC_040254.1	233936620	T	C
NC_040254.1	233978004	A	G
NC_040254.1	234064625	A	G
NC_040254.1	234108165	A	G
NC_040254.1	234108885	A	G
NC_040254.1	234109911	A	G
NC_040254.1	234110775	T	C
NC_040254.1	234111314	T	C

NC_040254.1	234112000	T	C
NC_040254.1	234112179	C	T
NC_040254.1	234112518	T	C
NC_040254.1	234168371	G	A
NC_040254.1	234226216	A	G
NC_040254.1	234282028	T	C
NC_040254.1	234308339	C	A
NC_040254.1	234378770	T	C
NC_040254.1	234380334	T	C
NC_040254.1	234395909	T	C
NC_040254.1	234396942	G	A
NC_040254.1	234397148	A	G
NC_040254.1	234397705	A	G
NC_040254.1	234398153	T	C
NC_040254.1	234409556	T	C
NC_040254.1	234409821	A	G
NC_040254.1	234409990	G	A
NC_040254.1	234452602	T	C
NC_040254.1	234493493	A	G
NC_040254.1	234496087	C	T
NC_040254.1	234533883	T	G
NC_040254.1	234555445	G	A
NC_040254.1	234578610	T	C
NC_040254.1	234585850	T	C
NC_040254.1	234608628	A	G
NC_040254.1	234646348	A	G
NC_040254.1	234652300	A	G
NC_040254.1	234662095	A	G
NC_040254.1	234718612	T	C
NC_040254.1	234778528	G	A
NC_040254.1	234834143	C	A
NC_040254.1	234885696	A	G
NC_040254.1	234904906	A	G
NC_040254.1	234968897	C	T
NC_040254.1	235027462	T	C
NC_040254.1	235065254	0	G
NC_040254.1	235107600	G	A
NC_040254.1	235141375	C	T
NC_040254.1	235141547	A	G
NC_040254.1	235151124	A	G
NC_040254.1	235183543	T	C
NC_040254.1	235195478	A	G
NC_040254.1	235225839	G	A
NC_040254.1	235228356	C	A
NC_040254.1	235255333	G	A
NC_040254.1	235310683	A	G
NC_040254.1	235370945	A	G

NC_040254.1	235421100	G	A
NC_040254.1	235456571	G	A
NC_040254.1	235504317	C	A
NC_040254.1	235565585	G	A
NC_040254.1	235589457	A	G
NC_040254.1	235623033	T	C
NC_040254.1	235645116	T	C
NC_040254.1	235667620	G	C
NC_040254.1	235709394	A	G
NC_040254.1	235715057	C	A
NC_040254.1	235756389	A	G
NC_040254.1	235786427	T	C
NC_040254.1	235787449	A	G
NC_040254.1	235824669	T	G
NC_040254.1	235857084	C	A
NC_040254.1	235901667	T	A
NC_040254.1	235957203	C	G
NC_040254.1	235992620	T	C
NC_040254.1	236022850	A	G
NC_040254.1	236052915	C	T
NC_040254.1	236053067	T	C
NC_040254.1	236053906	A	T
NC_040254.1	236055151	A	G
NC_040254.1	236056868	C	T
NC_040254.1	236057405	C	T
NC_040254.1	236111492	T	C
NC_040254.1	236133664	A	G
NC_040254.1	236163576	G	A
NC_040254.1	236220273	C	T
NC_040254.1	236274793	A	G
NC_040254.1	236324868	A	G
NC_040254.1	236325077	A	G
NC_040254.1	236354169	A	G
NC_040254.1	236368869	T	C
NC_040254.1	236416588	G	A
NC_040254.1	236467892	A	G
NC_040254.1	236491219	A	G
NC_040254.1	236531617	C	T
NC_040254.1	236531926	G	A
NC_040254.1	236537221	T	C
NC_040254.1	236538010	G	A
NC_040254.1	236543974	G	A
NC_040254.1	236544133	T	C
NC_040254.1	236544726	A	G
NC_040254.1	236547756	T	C
NC_040254.1	236602078	G	T
NC_040254.1	236664169	G	A

NC_040254.1	236669932	T	C
NC_040254.1	236672033	A	G
NC_040254.1	236672354	C	T
NC_040254.1	236717961	C	T
NC_040254.1	236740544	A	G
NC_040254.1	236757132	A	G
NC_040254.1	236805265	T	G
NC_040254.1	236825567	A	G
NC_040254.1	236856394	G	A
NC_040254.1	236898125	T	C
NC_040254.1	236954711	G	C
NC_040254.1	237010836	T	G
NC_040254.1	237064724	G	A
NC_040254.1	237119890	A	G
NC_040254.1	237120399	A	G
NC_040254.1	237159601	C	T
NC_040254.1	237184570	A	G
NC_040254.1	237237714	A	C
NC_040254.1	237294301	G	A
NC_040254.1	237343197	A	G
NC_040254.1	237393398	A	C
NC_040254.1	237442874	T	C
NC_040254.1	237454149	G	A
NC_040254.1	237455926	A	G
NC_040254.1	237456128	A	G
NC_040254.1	237456877	T	C
NC_040254.1	237457054	T	C
NC_040254.1	237457220	T	C
NC_040254.1	237459591	A	G
NC_040254.1	237459842	G	C
NC_040254.1	237460090	G	T
NC_040254.1	237493828	A	G
NC_040254.1	237496627	C	G
NC_040254.1	237515865	T	C
NC_040254.1	237537313	G	A
NC_040254.1	237552811	T	C
NC_040254.1	237596124	G	A
NC_040254.1	237600524	G	C
NC_040254.1	237602931	T	C
NC_040254.1	237633129	T	C
NC_040254.1	237633492	A	C
NC_040254.1	237643135	T	C
NC_040254.1	237671020	A	G
NC_040254.1	237695729	T	C
NC_040254.1	237740081	T	C
NC_040254.1	237741074	T	C
NC_040254.1	237741320	A	G

NC_040254.1	237741515	G	C
NC_040254.1	237742615	G	A
NC_040254.1	237789802	A	C
NC_040254.1	237829846	A	G
NC_040254.1	237839087	T	G
NC_040254.1	237840021	T	C
NC_040254.1	237863591	A	G
NC_040254.1	237898509	A	G
NC_040254.1	237946613	C	T
NC_040254.1	238003147	T	C
NC_040254.1	238043245	T	C
NC_040254.1	238074279	T	C
NC_040254.1	238093700	G	A
NC_040254.1	238136565	C	T
NC_040254.1	238193809	T	C
NC_040254.1	238225229	A	G
NC_040254.1	238334221	T	C
NC_040254.1	238426059	T	C
NC_040254.1	238510934	A	G
NC_040254.1	238552803	C	T
NC_040254.1	238611694	C	T
NC_040254.1	238701160	A	C
NC_040254.1	238756315	T	C
NC_040254.1	238819093	G	T
NC_040254.1	238821663	C	T
NC_040254.1	238869695	T	G
NC_040254.1	238892908	C	T
NC_040254.1	238953167	A	G
NC_040254.1	239009935	T	C
NC_040254.1	239065555	G	T
NC_040254.1	239100484	A	G
NC_040254.1	239125396	C	T
NC_040254.1	239181525	T	G
NC_040254.1	239257379	T	C
NC_040254.1	239306930	G	C
NC_040254.1	239307764	A	G
NC_040254.1	239333267	G	A
NC_040254.1	239422924	T	C
NC_040254.1	239475517	A	G
NC_040254.1	239507710	T	C
NC_040254.1	239569612	A	G
NC_040254.1	239647606	A	G
NC_040254.1	239687032	G	A
NC_040254.1	239708734	G	A
NC_040254.1	239774742	G	A
NC_040254.1	239811276	T	C
NC_040254.1	239826396	C	T

NC_040254.1	239868479	G	A
NC_040254.1	239875743	A	G
NC_040254.1	239933870	T	C
NC_040254.1	240001589	A	G
NC_040254.1	240054891	T	C
NC_040254.1	240115625	G	A
NC_040254.1	240169800	A	G
NC_040254.1	240215560	T	C
NC_040254.1	240248524	T	C
NC_040254.1	240267004	C	G
NC_040254.1	240336779	T	C
NC_040254.1	240338556	T	C
NC_040254.1	240339549	A	G
NC_040254.1	240356261	T	C
NC_040254.1	240396861	C	G
NC_040254.1	240397313	T	C
NC_040254.1	240398456	C	T
NC_040254.1	240467613	T	C
NC_040254.1	240499103	A	G
NC_040254.1	240499263	T	C
NC_040254.1	240547632	G	A
NC_040254.1	240579203	A	G
NC_040254.1	240584548	T	C
NC_040254.1	240594196	T	C
NC_040254.1	240599084	T	C
NC_040254.1	240599318	T	C
NC_040254.1	240599765	C	G
NC_040254.1	240599985	T	G
NC_040254.1	240600772	G	A
NC_040254.1	240635306	T	C
NC_040254.1	240635609	G	A
NC_040254.1	240637893	G	A
NC_040254.1	240644574	A	G
NC_040254.1	240653644	T	C
NC_040254.1	240658665	A	G
NC_040254.1	240661699	A	G
NC_040254.1	240664403	C	G
NC_040254.1	240664630	C	T
NC_040254.1	240666696	G	C
NC_040254.1	240666981	T	C
NC_040254.1	240677894	G	A
NC_040254.1	240679755	T	C
NC_040254.1	240686648	A	G
NC_040254.1	240687178	A	G
NC_040254.1	240694239	T	C
NC_040254.1	240699417	T	C
NC_040254.1	240699606	A	G

NC_040254.1	240753214	T	G
NC_040254.1	240801573	T	C
NC_040254.1	240803157	T	C
NC_040254.1	240803512	A	G
NC_040254.1	240814757	T	C
NC_040254.1	240815147	C	T
NC_040254.1	240815688	A	G
NC_040254.1	240818789	G	A
NC_040254.1	240819537	T	C
NC_040254.1	240821669	A	G
NC_040254.1	240857615	T	C
NC_040254.1	240859231	T	C
NC_040254.1	240867492	A	G
NC_040254.1	240878167	A	G
NC_040254.1	240901776	C	T
NC_040254.1	240903217	C	A
NC_040254.1	240904421	T	C
NC_040254.1	240904743	T	C
NC_040254.1	240941003	G	A
NC_040254.1	240952520	A	C
NC_040254.1	240952958	T	C
NC_040254.1	240978026	A	G
NC_040254.1	240978244	A	T
NC_040254.1	240984092	A	G
NC_040254.1	240997105	C	G
NC_040254.1	241008720	G	A
NC_040254.1	241032337	T	C
NC_040254.1	241047056	C	G
NC_040254.1	241047536	A	G
NC_040254.1	241047834	A	G
NC_040254.1	241066422	T	C
NC_040254.1	241072107	C	T
NC_040254.1	241072298	T	C
NC_040254.1	241072621	T	C
NC_040254.1	241108088	C	A
NC_040255.1	25235	C	T
NC_040255.1	74103	C	A
NC_040255.1	134425	T	C
NC_040255.1	188516	G	C
NC_040255.1	235025	A	G
NC_040255.1	303517	G	A
NC_040255.1	372270	G	A
NC_040255.1	432005	C	A
NC_040255.1	501640	A	T
NC_040255.1	566431	T	A
NC_040255.1	615850	T	A
NC_040255.1	617565	A	G

NC_040255.1	670170	A	T
NC_040255.1	732248	A	G
NC_040255.1	789858	G	T
NC_040255.1	839207	G	A
NC_040255.1	923405	T	C
NC_040255.1	956692	G	A
NC_040255.1	1136537	A	G
NC_040255.1	1174856	A	G
NC_040255.1	1237377	T	G
NC_040255.1	1291324	T	A
NC_040255.1	1377969	C	T
NC_040255.1	1438717	T	C
NC_040255.1	1492026	A	G
NC_040255.1	1547373	G	A
NC_040255.1	1604542	A	G
NC_040255.1	1670204	T	C
NC_040255.1	1742386	T	C
NC_040255.1	1800684	G	A
NC_040255.1	1850828	A	T
NC_040255.1	1918181	C	T
NC_040255.1	1972874	T	C
NC_040255.1	2032285	G	A
NC_040255.1	2076013	C	T
NC_040255.1	2119847	A	C
NC_040255.1	2253019	T	G
NC_040255.1	2498153	T	C
NC_040255.1	2635354	T	G
NC_040255.1	2728079	G	C
NC_040255.1	2777719	G	A
NC_040255.1	2833873	C	T
NC_040255.1	2878092	C	T
NC_040255.1	2940339	G	A
NC_040255.1	3090982	G	A
NC_040255.1	3163208	A	C
NC_040255.1	3198262	0	0
NC_040255.1	3249235	G	A
NC_040255.1	3312507	C	T
NC_040255.1	3376383	C	T
NC_040255.1	3445030	T	G
NC_040255.1	3488199	A	G
NC_040255.1	3640147	A	T
NC_040255.1	3689720	T	G
NC_040255.1	3724716	C	G
NC_040255.1	3802321	T	A
NC_040255.1	3843791	G	A
NC_040255.1	3897616	T	G
NC_040255.1	4042383	G	T

NC_040255.1	4115076	C	T
NC_040255.1	4209634	A	G
NC_040255.1	4265645	C	T
NC_040255.1	4309813	G	C
NC_040255.1	4397961	C	A
NC_040255.1	4589112	A	G
NC_040255.1	4665730	C	A
NC_040255.1	4734808	C	T
NC_040255.1	4778973	G	A
NC_040255.1	4836892	A	T
NC_040255.1	4893699	A	G
NC_040255.1	4959023	T	G
NC_040255.1	4981831	T	C
NC_040255.1	5247651	C	T
NC_040255.1	5304285	T	C
NC_040255.1	5367061	G	C
NC_040255.1	5437452	C	G
NC_040255.1	5492439	G	T
NC_040255.1	5542059	T	C
NC_040255.1	5643599	C	T
NC_040255.1	5692864	T	C
NC_040255.1	5740199	C	T
NC_040255.1	5792100	A	G
NC_040255.1	5918177	A	G
NC_040255.1	5959748	A	G
NC_040255.1	6014527	G	A
NC_040255.1	6066852	T	G
NC_040255.1	6118104	A	G
NC_040255.1	6160122	T	C
NC_040255.1	6247552	C	T
NC_040255.1	6290982	A	G
NC_040255.1	6330141	C	G
NC_040255.1	6387838	A	G
NC_040255.1	6444799	G	A
NC_040255.1	6509459	T	C
NC_040255.1	6511464	C	G
NC_040255.1	6512087	C	T
NC_040255.1	6512254	C	A
NC_040255.1	6555364	A	G
NC_040255.1	6606633	T	G
NC_040255.1	6653171	C	T
NC_040255.1	6679341	C	A
NC_040255.1	6680018	C	T
NC_040255.1	6680794	C	T
NC_040255.1	6681580	T	C
NC_040255.1	6681740	A	G
NC_040255.1	6705318	G	T

NC_040255.1	6758416	C	T
NC_040255.1	6815285	C	G
NC_040255.1	6860195	T	C
NC_040255.1	6914825	T	A
NC_040255.1	6968050	A	G
NC_040255.1	7024933	A	C
NC_040255.1	7060725	A	G
NC_040255.1	7157500	A	G
NC_040255.1	7228269	T	C
NC_040255.1	7298329	A	G
NC_040255.1	7341201	G	A
NC_040255.1	7392548	G	A
NC_040255.1	7461097	A	G
NC_040255.1	7537714	T	C
NC_040255.1	7906442	T	G
NC_040255.1	7977244	A	G
NC_040255.1	8059484	T	C
NC_040255.1	8121056	T	G
NC_040255.1	8155723	C	T
NC_040255.1	8264356	T	C
NC_040255.1	8305034	C	T
NC_040255.1	8423350	A	C
NC_040255.1	8455209	C	A
NC_040255.1	8522402	C	T
NC_040255.1	8599232	G	C
NC_040255.1	8654383	C	T
NC_040255.1	8709373	T	C
NC_040255.1	8765784	C	G
NC_040255.1	8834754	A	C
NC_040255.1	8894108	G	A
NC_040255.1	8959914	A	G
NC_040255.1	9019687	G	A
NC_040255.1	9082634	A	G
NC_040255.1	9140228	A	C
NC_040255.1	9193012	A	G
NC_040255.1	9220001	G	C
NC_040255.1	9254636	A	G
NC_040255.1	9316621	T	C
NC_040255.1	9365211	T	C
NC_040255.1	9413606	A	G
NC_040255.1	9455254	0	T
NC_040255.1	9507296	T	G
NC_040255.1	9560003	A	G
NC_040255.1	9612673	G	A
NC_040255.1	9644445	G	A
NC_040255.1	9694968	T	C
NC_040255.1	9731360	A	G

NC_040255.1	9788670	G	A
NC_040255.1	9843793	G	C
NC_040255.1	9897345	G	A
NC_040255.1	9960093	C	T
NC_040255.1	10009165	C	T
NC_040255.1	10065963	T	A
NC_040255.1	10129361	A	G
NC_040255.1	10130215	C	A
NC_040255.1	10183086	T	C
NC_040255.1	10183253	G	A
NC_040255.1	10183472	A	G
NC_040255.1	10185336	G	T
NC_040255.1	10185640	C	T
NC_040255.1	10186534	A	G
NC_040255.1	10241254	C	T
NC_040255.1	10298759	T	C
NC_040255.1	10352593	G	A
NC_040255.1	10410856	T	C
NC_040255.1	10449651	G	A
NC_040255.1	10503522	T	C
NC_040255.1	10540616	T	C
NC_040255.1	10585560	A	G
NC_040255.1	10682232	T	C
NC_040255.1	10737620	C	G
NC_040255.1	10796254	C	A
NC_040255.1	10796768	T	C
NC_040255.1	10801876	A	G
NC_040255.1	10844774	C	G
NC_040255.1	10894051	A	G
NC_040255.1	10945078	G	A
NC_040255.1	10972544	A	G
NC_040255.1	10986558	C	G
NC_040255.1	10987506	C	A
NC_040255.1	10999639	G	A
NC_040255.1	11113329	T	C
NC_040255.1	11136845	G	T
NC_040255.1	11152788	T	C
NC_040255.1	11153562	G	C
NC_040255.1	11175166	T	C
NC_040255.1	11175574	C	T
NC_040255.1	11200516	C	T
NC_040255.1	11214401	T	C
NC_040255.1	11268112	G	A
NC_040255.1	11321228	T	C
NC_040255.1	11367905	C	T
NC_040255.1	11368104	A	T
NC_040255.1	11368381	T	C

NC_040255.1	11369427	G	A
NC_040255.1	11404361	A	G
NC_040255.1	11444274	T	C
NC_040255.1	11507403	G	C
NC_040255.1	11518872	T	G
NC_040255.1	11530843	A	G
NC_040255.1	11531049	G	A
NC_040255.1	11531269	G	A
NC_040255.1	11532742	T	C
NC_040255.1	11533988	A	T
NC_040255.1	11566290	G	A
NC_040255.1	11566524	A	G
NC_040255.1	11566716	A	G
NC_040255.1	11566885	C	A
NC_040255.1	11567993	G	C
NC_040255.1	11568305	C	A
NC_040255.1	11569810	T	C
NC_040255.1	11570153	T	C
NC_040255.1	11571232	C	A
NC_040255.1	11572831	T	A
NC_040255.1	11573619	C	T
NC_040255.1	11574789	A	G
NC_040255.1	11575038	C	T
NC_040255.1	11629665	T	G
NC_040255.1	11681507	G	A
NC_040255.1	11736258	A	C
NC_040255.1	11787950	T	C
NC_040255.1	11824863	T	C
NC_040255.1	11825796	A	G
NC_040255.1	11881268	T	C
NC_040255.1	11934343	C	T
NC_040255.1	12003586	C	T
NC_040255.1	12028958	T	C
NC_040255.1	12309458	G	A
NC_040255.1	12364936	A	G
NC_040255.1	12403102	T	A
NC_040255.1	12407880	T	C
NC_040255.1	12408071	G	A
NC_040255.1	12464449	T	C
NC_040255.1	12509825	A	G
NC_040255.1	12560169	G	A
NC_040255.1	12613304	G	A
NC_040255.1	12661648	G	A
NC_040255.1	12715763	C	T
NC_040255.1	12771513	C	T
NC_040255.1	12825055	C	T
NC_040255.1	12880406	C	G

NC_040255.1	12933668	T	C
NC_040255.1	12992528	T	C
NC_040255.1	13035870	G	A
NC_040255.1	13072461	A	G
NC_040255.1	13123790	G	T
NC_040255.1	13180214	A	G
NC_040255.1	13234670	A	G
NC_040255.1	13292197	A	G
NC_040255.1	13348921	A	T
NC_040255.1	13408595	G	A
NC_040255.1	13464680	T	G
NC_040255.1	13520312	C	G
NC_040255.1	13576861	T	C
NC_040255.1	13614723	G	T
NC_040255.1	13664616	T	A
NC_040255.1	13738753	C	A
NC_040255.1	13763421	A	G
NC_040255.1	13763593	G	A
NC_040255.1	13763833	T	C
NC_040255.1	13830610	T	C
NC_040255.1	13886575	A	G
NC_040255.1	13887717	T	C
NC_040255.1	13948122	C	T
NC_040255.1	14011896	T	C
NC_040255.1	14013941	A	C
NC_040255.1	14014091	T	C
NC_040255.1	14072778	G	T
NC_040255.1	14128073	G	A
NC_040255.1	14200977	T	G
NC_040255.1	14256336	T	C
NC_040255.1	14309912	A	T
NC_040255.1	14349965	A	C
NC_040255.1	14350670	C	G
NC_040255.1	14350877	T	G
NC_040255.1	14352313	G	C
NC_040255.1	14354736	G	A
NC_040255.1	14355631	A	G
NC_040255.1	14402703	G	A
NC_040255.1	14465594	T	C
NC_040255.1	14526054	T	A
NC_040255.1	14570762	A	G
NC_040255.1	14620149	T	G
NC_040255.1	14669500	A	T
NC_040255.1	14724875	A	G
NC_040255.1	14766274	A	C
NC_040255.1	14832506	T	C
NC_040255.1	14886336	G	A

NC_040255.1	14940996	C	G
NC_040255.1	14988600	A	G
NC_040255.1	15042183	A	G
NC_040255.1	15096889	A	G
NC_040255.1	15148900	G	A
NC_040255.1	15199928	G	A
NC_040255.1	15254786	A	C
NC_040255.1	15275422	A	G
NC_040255.1	15275704	A	G
NC_040255.1	15276050	A	G
NC_040255.1	15319302	A	G
NC_040255.1	15361365	C	G
NC_040255.1	15363770	C	G
NC_040255.1	15419758	G	A
NC_040255.1	15475191	T	C
NC_040255.1	15529970	A	G
NC_040255.1	15584921	A	C
NC_040255.1	15646331	T	A
NC_040255.1	15702849	A	G
NC_040255.1	15764044	C	T
NC_040255.1	15825729	C	T
NC_040255.1	15875266	G	A
NC_040255.1	15930170	C	A
NC_040255.1	15987143	C	T
NC_040255.1	16044674	T	C
NC_040255.1	16104475	T	C
NC_040255.1	16158160	T	C
NC_040255.1	16187784	T	C
NC_040255.1	16187939	A	C
NC_040255.1	16206250	G	A
NC_040255.1	16257999	A	G
NC_040255.1	16320875	A	G
NC_040255.1	16396476	T	C
NC_040255.1	16454753	G	A
NC_040255.1	16495778	A	C
NC_040255.1	16549132	A	G
NC_040255.1	16590777	A	G
NC_040255.1	16661397	T	C
NC_040255.1	16801664	G	T
NC_040255.1	16857116	C	G
NC_040255.1	16914072	T	G
NC_040255.1	16977801	G	T
NC_040255.1	17032882	A	G
NC_040255.1	17088300	C	T
NC_040255.1	17141058	T	C
NC_040255.1	17196771	G	C
NC_040255.1	17229870	G	T

NC_040255.1	17269207	C	T
NC_040255.1	17269657	G	A
NC_040255.1	17276598	A	G
NC_040255.1	17320681	G	C
NC_040255.1	17395105	T	C
NC_040255.1	17432883	T	A
NC_040255.1	17487198	C	T
NC_040255.1	17538193	T	G
NC_040255.1	17572209	G	A
NC_040255.1	17628104	C	G
NC_040255.1	17680434	T	C
NC_040255.1	17688691	C	T
NC_040255.1	17689906	T	C
NC_040255.1	17690103	C	G
NC_040255.1	17751300	A	G
NC_040255.1	17807626	T	A
NC_040255.1	17864183	G	T
NC_040255.1	17911194	G	A
NC_040255.1	18177877	T	C
NC_040255.1	18212689	A	G
NC_040255.1	18213004	C	G
NC_040255.1	18267963	A	G
NC_040255.1	18315994	G	C
NC_040255.1	18368988	A	G
NC_040255.1	18424934	A	G
NC_040255.1	18425154	G	A
NC_040255.1	18425491	T	C
NC_040255.1	18476647	G	T
NC_040255.1	18535325	G	A
NC_040255.1	18589338	T	G
NC_040255.1	18630068	T	A
NC_040255.1	18689565	A	C
NC_040255.1	18746912	T	G
NC_040255.1	18799624	G	A
NC_040255.1	18856441	T	C
NC_040255.1	18909927	A	T
NC_040255.1	18973189	T	C
NC_040255.1	19029595	T	C
NC_040255.1	19080953	A	T
NC_040255.1	19130273	A	G
NC_040255.1	19169495	G	A
NC_040255.1	19243469	T	G
NC_040255.1	19295562	A	G
NC_040255.1	19336240	T	C
NC_040255.1	19411579	G	A
NC_040255.1	19457121	T	C
NC_040255.1	19497171	C	T

NC_040255.1	19567378	A	G
NC_040255.1	19623251	C	T
NC_040255.1	19691726	T	G
NC_040255.1	19766488	C	T
NC_040255.1	19826054	0	G
NC_040255.1	19943697	C	T
NC_040255.1	20015650	T	G
NC_040255.1	20068709	0	T
NC_040255.1	20122159	A	C
NC_040255.1	20156692	G	A
NC_040255.1	20211506	T	C
NC_040255.1	20290775	T	C
NC_040255.1	20469937	G	A
NC_040255.1	20521510	C	T
NC_040255.1	20554398	T	C
NC_040255.1	20609348	A	C
NC_040255.1	20672046	T	C
NC_040255.1	20740658	G	A
NC_040255.1	20801722	T	G
NC_040255.1	20850806	A	G
NC_040255.1	20898090	T	C
NC_040255.1	20954941	G	A
NC_040255.1	21006849	C	T
NC_040255.1	21063501	A	T
NC_040255.1	21117679	C	T
NC_040255.1	21147285	G	A
NC_040255.1	21203307	A	G
NC_040255.1	21252461	T	C
NC_040255.1	21312424	A	G
NC_040255.1	21367388	A	G
NC_040255.1	21418934	T	C
NC_040255.1	21475896	T	C
NC_040255.1	21531593	A	G
NC_040255.1	21586669	T	A
NC_040255.1	21633163	A	G
NC_040255.1	21659360	T	C
NC_040255.1	21705823	T	G
NC_040255.1	21759117	C	T
NC_040255.1	21801640	G	C
NC_040255.1	21838314	G	A
NC_040255.1	21892548	C	G
NC_040255.1	21949101	C	A
NC_040255.1	22006287	G	A
NC_040255.1	22047102	T	C
NC_040255.1	22099013	T	A
NC_040255.1	22100151	T	C
NC_040255.1	22100665	G	A

NC_040255.1	22100857	C	T
NC_040255.1	22153948	G	A
NC_040255.1	22209598	T	C
NC_040255.1	22265320	T	A
NC_040255.1	22307894	T	C
NC_040255.1	22347481	A	G
NC_040255.1	22403512	A	G
NC_040255.1	22461963	T	C
NC_040255.1	22517427	T	C
NC_040255.1	22576738	T	A
NC_040255.1	22630350	T	C
NC_040255.1	22689331	G	A
NC_040255.1	22744356	T	G
NC_040255.1	22794591	A	G
NC_040255.1	22865170	T	C
NC_040255.1	22932716	A	C
NC_040255.1	22994050	G	T
NC_040255.1	23060574	C	T
NC_040255.1	23081741	C	T
NC_040255.1	23141287	G	A
NC_040255.1	23202231	T	C
NC_040255.1	23258264	C	T
NC_040255.1	23318321	A	G
NC_040255.1	23382353	C	T
NC_040255.1	23383719	T	C
NC_040255.1	23384314	A	G
NC_040255.1	23384649	G	A
NC_040255.1	23425946	C	A
NC_040255.1	23481707	C	T
NC_040255.1	23533574	A	G
NC_040255.1	23585631	C	T
NC_040255.1	23645895	T	C
NC_040255.1	23691965	G	C
NC_040255.1	23753662	A	G
NC_040255.1	23808026	G	T
NC_040255.1	23874887	A	G
NC_040255.1	24168607	T	C
NC_040255.1	24228546	G	T
NC_040255.1	24280511	A	T
NC_040255.1	24330032	A	G
NC_040255.1	24385534	G	A
NC_040255.1	24441976	A	T
NC_040255.1	24500267	A	G
NC_040255.1	24554298	G	T
NC_040255.1	24595964	T	C
NC_040255.1	24637567	T	C
NC_040255.1	24680610	A	G

NC_040255.1	24751384	G	A
NC_040255.1	24809882	A	G
NC_040255.1	24864703	A	G
NC_040255.1	24915033	A	G
NC_040255.1	24960678	C	T
NC_040255.1	24960860	T	A
NC_040255.1	24961091	G	T
NC_040255.1	24961537	G	T
NC_040255.1	24962426	C	A
NC_040255.1	24962610	C	T
NC_040255.1	25019479	T	C
NC_040255.1	25057334	T	C
NC_040255.1	25079968	C	T
NC_040255.1	25234185	C	T
NC_040255.1	25288411	A	G
NC_040255.1	25345135	T	C
NC_040255.1	25403649	G	T
NC_040255.1	25456864	G	A
NC_040255.1	25524699	G	A
NC_040255.1	25654392	A	G
NC_040255.1	25704687	C	T
NC_040255.1	25757640	C	T
NC_040255.1	25809387	G	T
NC_040255.1	25865399	C	T
NC_040255.1	25928052	C	T
NC_040255.1	25987336	A	G
NC_040255.1	26044360	G	A
NC_040255.1	26164140	T	C
NC_040255.1	26217273	A	C
NC_040255.1	26256661	C	T
NC_040255.1	26320230	G	A
NC_040255.1	26376878	A	G
NC_040255.1	26418343	T	C
NC_040255.1	26470991	A	G
NC_040255.1	26625758	T	C
NC_040255.1	26680379	G	T
NC_040255.1	26737169	C	T
NC_040255.1	26792986	C	T
NC_040255.1	26848785	G	T
NC_040255.1	26909220	G	A
NC_040255.1	26962462	C	T
NC_040255.1	27018200	C	T
NC_040255.1	27075410	G	A
NC_040255.1	27124753	T	C
NC_040255.1	27172346	C	T
NC_040255.1	27208143	C	T
NC_040255.1	27262562	A	G

NC_040255.1	27299226	T	C
NC_040255.1	27299403	A	T
NC_040255.1	27299638	A	G
NC_040255.1	27356304	G	C
NC_040255.1	27412251	T	C
NC_040255.1	27470709	A	G
NC_040255.1	27525810	A	C
NC_040255.1	27586614	A	G
NC_040255.1	27643017	C	G
NC_040255.1	27696096	A	G
NC_040255.1	27752652	C	T
NC_040255.1	27792637	T	C
NC_040255.1	27827101	T	C
NC_040255.1	27885395	T	C
NC_040255.1	27948402	A	T
NC_040255.1	28016330	C	A
NC_040255.1	28074051	T	C
NC_040255.1	28134443	A	G
NC_040255.1	28175461	C	T
NC_040255.1	28217013	G	A
NC_040255.1	28410344	A	G
NC_040255.1	28461092	G	C
NC_040255.1	28500002	G	A
NC_040255.1	28567867	C	T
NC_040255.1	28569393	G	A
NC_040255.1	28576173	C	G
NC_040255.1	28605078	G	A
NC_040255.1	28631825	T	C
NC_040255.1	28653702	C	T
NC_040255.1	28717312	A	G
NC_040255.1	28742522	T	C
NC_040255.1	28799360	C	A
NC_040255.1	28853902	T	C
NC_040255.1	28912415	C	T
NC_040255.1	28974123	T	C
NC_040255.1	29033710	G	A
NC_040255.1	29091165	A	G
NC_040255.1	29143943	T	C
NC_040255.1	29196808	A	G
NC_040255.1	29252038	A	G
NC_040255.1	29318516	G	A
NC_040255.1	29319040	A	T
NC_040255.1	29319289	T	C
NC_040255.1	29319736	G	T
NC_040255.1	29320349	A	T
NC_040255.1	29320645	G	A
NC_040255.1	29377215	C	T

NC_040255.1	29440568	A	C
NC_040255.1	29508602	C	T
NC_040255.1	29561749	C	T
NC_040255.1	29608359	G	C
NC_040255.1	29660069	C	T
NC_040255.1	29707332	C	T
NC_040255.1	29750747	C	T
NC_040255.1	29766335	A	G
NC_040255.1	29767528	C	T
NC_040255.1	29768059	A	G
NC_040255.1	29818247	G	A
NC_040255.1	29872418	A	G
NC_040255.1	29920974	T	C
NC_040255.1	29961161	C	A
NC_040255.1	30004682	G	C
NC_040255.1	30060836	C	T
NC_040255.1	30115379	G	A
NC_040255.1	30172017	A	G
NC_040255.1	30226737	A	G
NC_040255.1	30282047	A	G
NC_040255.1	30337721	T	C
NC_040255.1	30393859	A	G
NC_040255.1	30449663	A	G
NC_040255.1	30506291	G	A
NC_040255.1	30563379	G	T
NC_040255.1	30616591	G	A
NC_040255.1	30655505	A	C
NC_040255.1	30712062	G	A
NC_040255.1	30769225	G	A
NC_040255.1	30825451	A	G
NC_040255.1	30882370	T	C
NC_040255.1	30938922	G	A
NC_040255.1	30992155	A	G
NC_040255.1	31112063	G	A
NC_040255.1	31166943	A	G
NC_040255.1	31227025	C	T
NC_040255.1	31283943	T	G
NC_040255.1	31337021	G	T
NC_040255.1	31387397	C	G
NC_040255.1	31444047	T	C
NC_040255.1	31505210	A	G
NC_040255.1	31561165	C	A
NC_040255.1	31613322	T	G
NC_040255.1	31670868	T	C
NC_040255.1	31726307	A	T
NC_040255.1	31776165	G	C
NC_040255.1	31843171	A	T

NC_040255.1	31898732	C	T
NC_040255.1	31947004	T	C
NC_040255.1	31997863	T	C
NC_040255.1	32053370	C	A
NC_040255.1	32110097	G	A
NC_040255.1	32165571	G	T
NC_040255.1	32216151	G	A
NC_040255.1	32256275	T	A
NC_040255.1	32256713	G	C
NC_040255.1	32281466	T	C
NC_040255.1	32322119	A	G
NC_040255.1	32378275	T	C
NC_040255.1	32433476	C	T
NC_040255.1	32484290	T	C
NC_040255.1	32533835	A	G
NC_040255.1	32575021	A	T
NC_040255.1	32632138	G	T
NC_040255.1	32688362	C	G
NC_040255.1	32734764	A	T
NC_040255.1	32801178	T	C
NC_040255.1	32863557	G	A
NC_040255.1	32910830	C	T
NC_040255.1	32924093	A	G
NC_040255.1	32947885	T	C
NC_040255.1	33001473	G	A
NC_040255.1	33056923	A	G
NC_040255.1	33112149	G	A
NC_040255.1	33167794	T	C
NC_040255.1	33220433	C	A
NC_040255.1	33276238	G	A
NC_040255.1	33330942	C	T
NC_040255.1	33387276	G	A
NC_040255.1	33445842	T	C
NC_040255.1	33490652	A	G
NC_040255.1	33546618	A	G
NC_040255.1	33600826	T	C
NC_040255.1	33653254	C	T
NC_040255.1	33653573	C	T
NC_040255.1	33698592	C	T
NC_040255.1	33728622	T	C
NC_040255.1	33775080	T	C
NC_040255.1	33807268	T	C
NC_040255.1	33845585	A	G
NC_040255.1	33889755	G	A
NC_040255.1	33918212	A	G
NC_040255.1	33977312	C	T
NC_040255.1	34014450	A	G

NC_040255.1	34057135	G	A
NC_040255.1	34103315	T	C
NC_040255.1	34104025	C	G
NC_040255.1	34122391	C	G
NC_040255.1	34291489	C	T
NC_040255.1	34346852	T	C
NC_040255.1	34404579	T	C
NC_040255.1	34456527	T	G
NC_040255.1	34495149	G	C
NC_040255.1	34533462	A	G
NC_040255.1	34587864	C	A
NC_040255.1	34642901	A	G
NC_040255.1	34699486	G	T
NC_040255.1	34755442	G	A
NC_040255.1	34807198	C	G
NC_040255.1	34863147	A	G
NC_040255.1	34916843	C	A
NC_040255.1	34989990	A	C
NC_040255.1	35045090	C	A
NC_040255.1	35098860	T	A
NC_040255.1	35142485	T	C
NC_040255.1	35145795	A	T
NC_040255.1	35201860	G	A
NC_040255.1	35255437	G	A
NC_040255.1	35297277	C	G
NC_040255.1	35309532	G	A
NC_040255.1	35344857	A	G
NC_040255.1	35370684	A	G
NC_040255.1	35372379	C	T
NC_040255.1	35372534	T	C
NC_040255.1	35375615	G	T
NC_040255.1	35376849	G	C
NC_040255.1	35377273	T	C
NC_040255.1	35377650	T	C
NC_040255.1	35411596	A	G
NC_040255.1	35480020	C	T
NC_040255.1	35481521	C	T
NC_040255.1	35550216	T	C
NC_040255.1	35606225	A	T
NC_040255.1	35668404	T	C
NC_040255.1	35668878	G	T
NC_040255.1	35722998	G	T
NC_040255.1	35792177	G	A
NC_040255.1	35839102	C	T
NC_040255.1	35892558	C	T
NC_040255.1	36262449	C	A
NC_040255.1	36304801	T	A

NC_040255.1	36344691	A	G
NC_040255.1	36396526	A	G
NC_040255.1	36438505	G	A
NC_040255.1	36473095	T	C
NC_040255.1	36533709	G	C
NC_040255.1	36681612	T	A
NC_040255.1	36704633	C	T
NC_040255.1	36899961	G	T
NC_040255.1	36944045	C	A
NC_040255.1	37017066	T	C
NC_040255.1	37100281	T	C
NC_040255.1	37301690	C	T
NC_040255.1	37302464	G	T
NC_040255.1	37369099	C	A
NC_040255.1	37420962	G	A
NC_040255.1	37478572	G	T
NC_040255.1	37523862	C	T
NC_040255.1	37548442	T	C
NC_040255.1	37605369	G	C
NC_040255.1	37658040	A	G
NC_040255.1	37716030	C	A
NC_040255.1	37777726	A	C
NC_040255.1	37849829	C	T
NC_040255.1	37900641	C	G
NC_040255.1	37900853	A	G
NC_040255.1	37909747	G	A
NC_040255.1	37910240	A	C
NC_040255.1	37910464	G	A
NC_040255.1	37941657	A	G
NC_040255.1	38000293	A	G
NC_040255.1	38023747	G	A
NC_040255.1	38083209	A	G
NC_040255.1	38135284	G	A
NC_040255.1	38191283	T	C
NC_040255.1	38244032	C	T
NC_040255.1	38304584	C	A
NC_040255.1	38357772	A	G
NC_040255.1	38426690	T	A
NC_040255.1	38495213	A	G
NC_040255.1	38552821	T	G
NC_040255.1	38597387	T	C
NC_040255.1	38641102	A	G
NC_040255.1	38692542	A	G
NC_040255.1	38735962	A	G
NC_040255.1	38782812	A	G
NC_040255.1	38972997	G	T
NC_040255.1	39033542	A	G

NC_040255.1	39086970	G	T
NC_040255.1	39142320	A	G
NC_040255.1	39203931	C	T
NC_040255.1	39263461	C	T
NC_040255.1	39334009	A	G
NC_040255.1	39391154	A	C
NC_040255.1	39449601	C	G
NC_040255.1	39508908	C	T
NC_040255.1	39574693	T	C
NC_040255.1	39629119	A	T
NC_040255.1	39691233	A	G
NC_040255.1	39745941	T	C
NC_040255.1	39812155	T	G
NC_040255.1	39865778	A	G
NC_040255.1	39922534	T	C
NC_040255.1	39978407	A	C
NC_040255.1	40036212	T	C
NC_040255.1	40107946	G	A
NC_040255.1	40154260	G	A
NC_040255.1	40154857	G	A
NC_040255.1	40155862	C	T
NC_040255.1	40156360	T	C
NC_040255.1	40156912	C	T
NC_040255.1	40210304	A	G
NC_040255.1	40260786	A	G
NC_040255.1	40319919	T	C
NC_040255.1	40392314	G	A
NC_040255.1	40456819	T	C
NC_040255.1	40524508	G	A
NC_040255.1	40577275	C	T
NC_040255.1	40632374	G	T
NC_040255.1	40692888	G	A
NC_040255.1	40747328	C	G
NC_040255.1	40803299	C	T
NC_040255.1	40871980	A	G
NC_040255.1	40932797	G	T
NC_040255.1	40992414	A	G
NC_040255.1	41056611	C	G
NC_040255.1	41114296	A	G
NC_040255.1	41171559	T	C
NC_040255.1	41215717	T	C
NC_040255.1	41268506	G	A
NC_040255.1	41517577	G	A
NC_040255.1	41582008	G	A
NC_040255.1	41644208	G	A
NC_040255.1	41714201	T	G
NC_040255.1	41755007	G	A

NC_040255.1	41811419	C	G
NC_040255.1	41870785	A	G
NC_040255.1	41914363	C	T
NC_040255.1	41970231	T	C
NC_040255.1	42041241	A	G
NC_040255.1	42097694	C	T
NC_040255.1	42152658	A	T
NC_040255.1	42185822	G	C
NC_040255.1	42241675	G	A
NC_040255.1	42297524	A	G
NC_040255.1	42346212	T	G
NC_040255.1	42385802	C	G
NC_040255.1	42386011	G	A
NC_040255.1	42386209	A	G
NC_040255.1	42386830	A	C
NC_040255.1	42389316	G	A
NC_040255.1	42389668	A	G
NC_040255.1	42415345	A	G
NC_040255.1	42415630	T	C
NC_040255.1	42415804	T	C
NC_040255.1	42423052	T	A
NC_040255.1	42465976	C	T
NC_040255.1	42502328	T	A
NC_040255.1	42508407	T	C
NC_040255.1	42561781	C	G
NC_040255.1	42561994	T	C
NC_040255.1	42562279	T	C
NC_040255.1	42562622	T	C
NC_040255.1	42562818	G	A
NC_040255.1	42629545	G	T
NC_040255.1	42675911	G	T
NC_040255.1	42737586	T	C
NC_040255.1	42790367	T	C
NC_040255.1	42848884	G	T
NC_040255.1	42893625	G	A
NC_040255.1	42945348	T	G
NC_040255.1	42998094	C	T
NC_040255.1	43055221	G	A
NC_040255.1	43102618	T	G
NC_040255.1	43215821	A	G
NC_040255.1	43277956	G	A
NC_040255.1	43335214	T	C
NC_040255.1	43387920	T	A
NC_040255.1	43422082	C	T
NC_040255.1	43433266	C	T
NC_040255.1	43479541	G	A
NC_040255.1	43518575	G	C

NC_040255.1	43566905	G	T
NC_040255.1	43625193	G	A
NC_040255.1	43685250	A	G
NC_040255.1	43741227	A	C
NC_040255.1	43785375	G	A
NC_040255.1	43787616	T	C
NC_040255.1	43788446	C	T
NC_040255.1	43841466	C	T
NC_040255.1	43899790	C	T
NC_040255.1	43955910	A	G
NC_040255.1	44024039	C	T
NC_040255.1	44051403	T	C
NC_040255.1	44289191	G	A
NC_040255.1	44329588	C	T
NC_040255.1	44422085	T	A
NC_040255.1	44471277	T	C
NC_040255.1	44526805	C	T
NC_040255.1	44583855	G	A
NC_040255.1	44645029	T	C
NC_040255.1	44701708	A	C
NC_040255.1	44756469	C	T
NC_040255.1	44810743	C	T
NC_040255.1	44865204	C	T
NC_040255.1	44920935	G	A
NC_040255.1	44964862	G	T
NC_040255.1	45015439	G	C
NC_040255.1	45065230	A	G
NC_040255.1	45115345	A	G
NC_040255.1	45162863	T	C
NC_040255.1	45218382	T	C
NC_040255.1	45257163	G	C
NC_040255.1	45322075	G	A
NC_040255.1	45499358	T	C
NC_040255.1	45508504	G	T
NC_040255.1	45568971	T	C
NC_040255.1	45637545	G	A
NC_040255.1	45751962	T	C
NC_040255.1	45792007	A	G
NC_040255.1	45841300	G	T
NC_040255.1	45899552	C	T
NC_040255.1	45956828	C	A
NC_040255.1	46019902	A	G
NC_040255.1	46078146	G	T
NC_040255.1	46133646	C	T
NC_040255.1	46184778	C	A
NC_040255.1	46217987	A	G
NC_040255.1	46219008	A	G

NC_040255.1	46274682	T	C
NC_040255.1	46331681	T	G
NC_040255.1	46389660	C	T
NC_040255.1	46444827	C	T
NC_040255.1	46498208	T	A
NC_040255.1	46595662	G	C
NC_040255.1	46651630	G	A
NC_040255.1	46707815	G	C
NC_040255.1	46763263	G	A
NC_040255.1	46811431	G	T
NC_040255.1	46921831	T	A
NC_040255.1	46981835	G	A
NC_040255.1	47037954	G	C
NC_040255.1	47090244	G	C
NC_040255.1	47145301	G	A
NC_040255.1	47264044	A	C
NC_040255.1	47320564	T	G
NC_040255.1	47377125	T	C
NC_040255.1	47432333	G	C
NC_040255.1	47482805	T	C
NC_040255.1	47514554	G	A
NC_040255.1	47766179	A	C
NC_040255.1	47821129	A	G
NC_040255.1	47876188	C	T
NC_040255.1	47930055	C	T
NC_040255.1	47983512	A	G
NC_040255.1	48037916	C	A
NC_040255.1	48124634	T	C
NC_040255.1	48176894	C	G
NC_040255.1	48245885	A	G
NC_040255.1	48374334	A	G
NC_040255.1	48431360	G	A
NC_040255.1	48487495	T	C
NC_040255.1	48543628	G	A
NC_040255.1	48596692	T	C
NC_040255.1	48652943	0	A
NC_040255.1	48713078	C	T
NC_040255.1	48764680	A	G
NC_040255.1	48810624	A	T
NC_040255.1	48851744	G	A
NC_040255.1	48852124	0	0
NC_040255.1	48904310	G	A
NC_040255.1	48904559	G	A
NC_040255.1	48905217	G	A
NC_040255.1	48905646	T	C
NC_040255.1	48961438	C	T
NC_040255.1	49012221	A	G

NC_040255.1	49061608	C	T
NC_040255.1	49062343	T	A
NC_040255.1	49118926	C	T
NC_040255.1	49190078	A	G
NC_040255.1	49190316	A	G
NC_040255.1	49265681	T	C
NC_040255.1	49319900	C	G
NC_040255.1	49377619	A	T
NC_040255.1	49428321	G	A
NC_040255.1	49474063	A	G
NC_040255.1	49474589	A	C
NC_040255.1	49485243	G	A
NC_040255.1	49536176	C	T
NC_040255.1	49594390	G	A
NC_040255.1	49650314	A	C
NC_040255.1	49709895	C	T
NC_040255.1	49774167	T	A
NC_040255.1	49825258	T	C
NC_040255.1	49831736	T	C
NC_040255.1	49880657	T	C
NC_040255.1	49935178	C	G
NC_040255.1	49990647	T	C
NC_040255.1	50050202	C	A
NC_040255.1	50096062	G	A
NC_040255.1	50155841	A	G
NC_040255.1	50222335	G	A
NC_040255.1	50262455	G	C
NC_040255.1	50319900	A	G
NC_040255.1	50365666	T	C
NC_040255.1	50419786	C	A
NC_040255.1	50464356	C	T
NC_040255.1	50505316	G	T
NC_040255.1	50729378	G	C
NC_040255.1	50791943	G	A
NC_040255.1	50848345	T	C
NC_040255.1	50909859	G	C
NC_040255.1	50981809	A	T
NC_040255.1	51144633	T	G
NC_040255.1	51178335	T	C
NC_040255.1	51228134	C	T
NC_040255.1	51274301	A	G
NC_040255.1	51328179	C	A
NC_040255.1	51390450	T	C
NC_040255.1	51460053	C	T
NC_040255.1	51507816	A	G
NC_040255.1	51716972	A	G
NC_040255.1	51777869	A	T

NC_040255.1	51829286	A	G
NC_040255.1	51901306	T	G
NC_040255.1	51962638	T	G
NC_040255.1	52034248	C	T
NC_040255.1	52090664	A	C
NC_040255.1	52145313	A	G
NC_040255.1	52201036	A	G
NC_040255.1	52241315	C	G
NC_040255.1	52270967	G	A
NC_040255.1	52324234	A	G
NC_040255.1	52378691	A	G
NC_040255.1	52437750	C	T
NC_040255.1	52499783	G	A
NC_040255.1	52575740	T	C
NC_040255.1	52622390	T	C
NC_040255.1	52680184	G	A
NC_040255.1	52733843	A	G
NC_040255.1	52787193	G	A
NC_040255.1	52826767	T	C
NC_040255.1	52893816	G	C
NC_040255.1	52951782	G	A
NC_040255.1	53010656	T	C
NC_040255.1	53071385	A	G
NC_040255.1	53141388	A	G
NC_040255.1	53200115	A	G
NC_040255.1	53260163	C	T
NC_040255.1	53319549	T	A
NC_040255.1	53371805	T	C
NC_040255.1	53422375	C	A
NC_040255.1	53469433	T	C
NC_040255.1	53555220	G	A
NC_040255.1	53592721	G	A
NC_040255.1	53642248	T	C
NC_040255.1	53706219	G	A
NC_040255.1	53774444	C	T
NC_040255.1	53841711	T	C
NC_040255.1	53905038	A	G
NC_040255.1	53952490	C	T
NC_040255.1	54002753	C	T
NC_040255.1	54083648	T	C
NC_040255.1	54151087	A	G
NC_040255.1	54248305	C	T
NC_040255.1	54294291	T	G
NC_040255.1	54341579	T	A
NC_040255.1	54400581	C	T
NC_040255.1	54446718	A	C
NC_040255.1	54509086	C	T

NC_040255.1	54577435	G	A
NC_040255.1	54620092	T	C
NC_040255.1	54671427	T	C
NC_040255.1	54700297	A	G
NC_040255.1	54759163	A	G
NC_040255.1	54765819	C	T
NC_040255.1	54849875	A	G
NC_040255.1	54913558	C	T
NC_040255.1	54970439	G	A
NC_040255.1	55033241	A	T
NC_040255.1	55330640	C	A
NC_040255.1	55374465	C	G
NC_040255.1	55443451	A	G
NC_040255.1	55503889	T	C
NC_040255.1	55562300	A	G
NC_040255.1	55695765	T	C
NC_040255.1	55747643	A	G
NC_040255.1	55782629	T	C
NC_040255.1	55866142	A	G
NC_040255.1	55917546	T	C
NC_040255.1	55980453	A	C
NC_040255.1	56033709	A	G
NC_040255.1	56110306	T	C
NC_040255.1	56170147	T	G
NC_040255.1	56213959	C	G
NC_040255.1	56251081	T	C
NC_040255.1	56291323	G	A
NC_040255.1	56377477	T	C
NC_040255.1	56441387	T	G
NC_040255.1	56753662	G	A
NC_040255.1	56843952	G	C
NC_040255.1	56876112	A	G
NC_040255.1	56934232	C	T
NC_040255.1	56987677	C	T
NC_040255.1	57016292	T	C
NC_040255.1	57063492	C	T
NC_040255.1	57110960	T	C
NC_040255.1	57150788	T	C
NC_040255.1	57217455	T	C
NC_040255.1	57265693	T	C
NC_040255.1	57293238	C	T
NC_040255.1	57378680	G	T
NC_040255.1	57420658	G	C
NC_040255.1	57468091	C	T
NC_040255.1	57534752	G	A
NC_040255.1	57592006	G	A
NC_040255.1	57621015	C	A

NC_040255.1	57688171	T	G
NC_040255.1	57735377	C	T
NC_040255.1	57793798	G	A
NC_040255.1	57854166	T	C
NC_040255.1	57914947	A	G
NC_040255.1	57971871	C	T
NC_040255.1	58041084	G	A
NC_040255.1	58094297	A	G
NC_040255.1	58130957	T	C
NC_040255.1	58177883	G	C
NC_040255.1	58228171	A	C
NC_040255.1	58280880	T	A
NC_040255.1	58347243	A	G
NC_040255.1	58418193	A	T
NC_040255.1	58502525	G	A
NC_040255.1	58616862	T	A
NC_040255.1	58685914	T	C
NC_040255.1	58737966	A	G
NC_040255.1	58778584	A	C
NC_040255.1	58935599	T	A
NC_040255.1	59005916	C	T
NC_040255.1	59032984	T	C
NC_040255.1	59118630	T	C
NC_040255.1	59178571	C	A
NC_040255.1	59231231	C	G
NC_040255.1	59312797	T	C
NC_040255.1	59402013	A	G
NC_040255.1	59668308	G	A
NC_040255.1	59736129	T	C
NC_040255.1	59810779	T	A
NC_040255.1	59866720	C	T
NC_040255.1	59926942	T	C
NC_040255.1	59971938	C	G
NC_040255.1	60010665	C	G
NC_040255.1	60300305	G	A
NC_040255.1	60351158	G	A
NC_040255.1	60385582	A	T
NC_040255.1	60501555	G	A
NC_040255.1	60563314	G	A
NC_040255.1	60612284	C	T
NC_040255.1	60662559	T	C
NC_040255.1	60737965	G	A
NC_040255.1	60780328	A	T
NC_040255.1	60833854	C	T
NC_040255.1	60928681	A	G
NC_040255.1	60974342	G	A
NC_040255.1	61098289	G	A

NC_040255.1	61375263	T	C
NC_040255.1	61573213	G	T
NC_040255.1	61944240	T	C
NC_040255.1	62009950	T	C
NC_040255.1	62060785	A	G
NC_040255.1	62106204	C	G
NC_040255.1	62128905	G	C
NC_040255.1	62323753	G	T
NC_040255.1	62555073	T	C
NC_040255.1	62768551	A	G
NC_040255.1	62880576	G	A
NC_040255.1	62976483	T	A
NC_040255.1	63039509	C	T
NC_040255.1	63103939	G	A
NC_040255.1	63256834	A	G
NC_040255.1	63378371	A	G
NC_040255.1	63444448	G	C
NC_040255.1	63589440	C	T
NC_040255.1	63874064	G	A
NC_040255.1	63954564	G	A
NC_040255.1	64036163	G	A
NC_040255.1	64145018	C	T
NC_040255.1	64433765	G	A
NC_040255.1	64664945	T	C
NC_040255.1	64872716	A	G
NC_040255.1	64898165	A	G
NC_040255.1	65139992	G	C
NC_040255.1	65686351	A	G
NC_040255.1	65924325	A	G
NC_040255.1	66043030	A	G
NC_040255.1	66111141	G	A
NC_040255.1	66170274	C	T
NC_040255.1	66214032	G	T
NC_040255.1	66397891	G	A
NC_040255.1	66435486	G	A
NC_040255.1	66515690	A	G
NC_040255.1	66579511	G	A
NC_040255.1	66667253	A	G
NC_040255.1	66727598	T	A
NC_040255.1	66867889	A	G
NC_040255.1	66938142	A	C
NC_040255.1	66981290	A	G
NC_040255.1	67033136	A	C
NC_040255.1	67083859	T	C
NC_040255.1	67563988	T	C
NC_040255.1	67613896	G	A
NC_040255.1	67684186	C	G

NC_040255.1	67757635	A	G
NC_040255.1	68034938	C	A
NC_040255.1	68098992	G	A
NC_040255.1	68192223	G	T
NC_040255.1	68383720	T	C
NC_040255.1	68489057	C	T
NC_040255.1	68550775	T	G
NC_040255.1	68620951	A	G
NC_040255.1	68662597	C	A
NC_040255.1	68717401	T	G
NC_040255.1	68757957	A	G
NC_040255.1	68792830	C	T
NC_040255.1	68974605	T	C
NC_040255.1	69201547	G	A
NC_040255.1	69267177	G	A
NC_040255.1	69368919	T	C
NC_040255.1	69472748	T	C
NC_040255.1	69574994	T	C
NC_040255.1	69627941	G	C
NC_040255.1	69961103	A	G
NC_040255.1	69999016	G	T
NC_040255.1	70122049	A	G
NC_040255.1	70166305	C	T
NC_040255.1	70287824	T	C
NC_040255.1	70698580	A	T
NC_040255.1	70751886	A	G
NC_040255.1	70833981	T	A
NC_040255.1	70976130	A	G
NC_040255.1	71077006	T	C
NC_040255.1	71128995	C	T
NC_040255.1	71188832	T	C
NC_040255.1	71246601	C	T
NC_040255.1	71277632	C	G
NC_040255.1	71328854	G	A
NC_040255.1	71394184	T	C
NC_040255.1	71469346	T	C
NC_040255.1	71538789	T	C
NC_040255.1	71623726	G	A
NC_040255.1	71702840	A	T
NC_040255.1	71757081	T	G
NC_040255.1	71806548	A	T
NC_040255.1	71854335	A	C
NC_040255.1	71898565	C	T
NC_040255.1	71942004	C	T
NC_040255.1	72014974	A	G
NC_040255.1	72129587	C	T
NC_040255.1	72214293	T	C

NC_040255.1	72270838	C	T
NC_040255.1	72334516	A	G
NC_040255.1	72536285	T	G
NC_040255.1	72590532	T	G
NC_040255.1	72655159	A	C
NC_040255.1	72744615	G	A
NC_040255.1	72857990	C	T
NC_040255.1	72958268	C	T
NC_040255.1	73011468	G	A
NC_040255.1	73200345	C	A
NC_040255.1	73341096	G	T
NC_040255.1	73446911	C	T
NC_040255.1	73735524	C	T
NC_040255.1	73867549	G	C
NC_040255.1	73921806	G	T
NC_040255.1	74024860	A	G
NC_040255.1	74080842	A	G
NC_040255.1	74121371	A	C
NC_040255.1	74189754	C	T
NC_040255.1	74238385	C	G
NC_040255.1	74282362	T	G
NC_040255.1	74370735	C	T
NC_040255.1	74495120	T	C
NC_040255.1	74574782	T	C
NC_040255.1	74643440	A	G
NC_040255.1	74700455	A	G
NC_040255.1	74756553	G	A
NC_040255.1	74822544	G	A
NC_040255.1	74865368	C	G
NC_040255.1	74929546	T	C
NC_040255.1	74992635	A	G
NC_040255.1	75091356	C	G
NC_040255.1	75165662	G	C
NC_040255.1	75290692	C	T
NC_040255.1	75339984	A	C
NC_040255.1	75395861	G	A
NC_040255.1	75452765	G	A
NC_040255.1	75639280	T	A
NC_040255.1	75686020	G	A
NC_040255.1	75759254	C	G
NC_040255.1	75852113	C	A
NC_040255.1	75933909	T	C
NC_040255.1	76134715	T	C
NC_040255.1	76166921	T	C
NC_040255.1	76238749	A	C
NC_040255.1	76344452	T	C
NC_040255.1	76409558	G	C

NC_040255.1	76487590	G	A
NC_040255.1	76636739	C	T
NC_040255.1	76683132	C	T
NC_040255.1	76737651	A	T
NC_040255.1	76778033	A	G
NC_040255.1	76847894	C	T
NC_040255.1	76915476	C	A
NC_040255.1	76977638	A	G
NC_040255.1	77055143	A	C
NC_040255.1	77091095	T	C
NC_040255.1	77140397	C	A
NC_040255.1	77175362	G	C
NC_040255.1	77371304	C	A
NC_040255.1	77435550	C	A
NC_040255.1	77574953	A	G
NC_040255.1	77702616	T	C
NC_040255.1	77820327	T	A
NC_040255.1	77879904	C	T
NC_040255.1	78048232	A	C
NC_040255.1	78531236	G	A
NC_040255.1	78760079	C	T
NC_040255.1	78952097	A	G
NC_040255.1	78983224	A	T
NC_040255.1	79130344	T	A
NC_040255.1	79228052	G	A
NC_040255.1	79326537	C	T
NC_040255.1	79391484	A	T
NC_040255.1	79520152	A	G
NC_040255.1	79600885	A	G
NC_040255.1	79678296	C	T
NC_040255.1	79739733	T	A
NC_040255.1	79774557	A	C
NC_040255.1	79876930	G	A
NC_040255.1	79926830	G	A
NC_040255.1	80092806	T	C
NC_040255.1	80153525	C	T
NC_040255.1	80208279	A	G
NC_040255.1	80303729	G	T
NC_040255.1	80458190	A	G
NC_040255.1	80507041	C	T
NC_040255.1	80572765	C	T
NC_040255.1	80605849	G	A
NC_040255.1	80734455	C	T
NC_040255.1	80891363	C	T
NC_040255.1	80959310	T	C
NC_040255.1	81564063	G	C
NC_040255.1	81833291	T	C

NC_040255.1	81923767	G	A
NC_040255.1	81974023	A	C
NC_040255.1	82253257	G	C
NC_040255.1	82495992	C	G
NC_040255.1	82566246	C	A
NC_040255.1	82602850	T	C
NC_040255.1	82689588	T	C
NC_040255.1	82810057	T	C
NC_040255.1	83112256	A	G
NC_040255.1	83355938	A	G
NC_040255.1	83429002	G	T
NC_040255.1	83534413	G	T
NC_040255.1	83601901	T	C
NC_040255.1	83644303	T	C
NC_040255.1	83709134	A	G
NC_040255.1	83746351	C	T
NC_040255.1	83810311	T	C
NC_040255.1	83864215	G	A
NC_040255.1	83905816	C	T
NC_040255.1	84029768	T	C
NC_040255.1	84079575	A	G
NC_040255.1	84137288	T	C
NC_040255.1	84192780	C	T
NC_040255.1	84249854	T	C
NC_040255.1	84322682	T	C
NC_040255.1	84381776	A	G
NC_040255.1	84433807	T	C
NC_040255.1	84475291	C	A
NC_040255.1	84549392	A	G
NC_040255.1	84603683	A	G
NC_040255.1	84654686	G	A
NC_040255.1	84676351	A	G
NC_040255.1	84734306	G	T
NC_040255.1	84803852	A	G
NC_040255.1	84849119	T	C
NC_040255.1	84965638	C	T
NC_040255.1	84997299	T	C
NC_040255.1	85038976	A	G
NC_040255.1	85079529	G	A
NC_040255.1	85117691	A	C
NC_040255.1	85169395	A	C
NC_040255.1	85213966	T	G
NC_040255.1	85310302	A	C
NC_040255.1	85340298	C	G
NC_040255.1	85470289	G	A
NC_040255.1	85527080	T	A
NC_040255.1	85584940	A	G

NC_040255.1	85624360	C	T
NC_040255.1	85672823	A	T
NC_040255.1	85719581	T	G
NC_040255.1	85759607	T	C
NC_040255.1	85805928	T	G
NC_040255.1	85859332	G	C
NC_040255.1	85903709	T	A
NC_040255.1	85956290	G	C
NC_040255.1	86007902	C	T
NC_040255.1	86067968	G	A
NC_040255.1	86120641	T	C
NC_040255.1	86194723	G	A
NC_040255.1	86242861	A	G
NC_040255.1	86273394	C	A
NC_040255.1	86330175	A	G
NC_040255.1	86379400	A	G
NC_040255.1	86435544	T	C
NC_040255.1	86495954	C	T
NC_040255.1	86544622	T	C
NC_040255.1	86605037	A	G
NC_040255.1	86652269	T	C
NC_040255.1	86717305	T	G
NC_040255.1	86793064	G	A
NC_040255.1	86872780	T	C
NC_040255.1	86918129	A	C
NC_040255.1	86980128	A	G
NC_040255.1	87032934	T	C
NC_040255.1	87071262	T	A
NC_040255.1	87138242	G	A
NC_040255.1	87195853	A	G
NC_040255.1	87246205	A	G
NC_040255.1	87291078	A	T
NC_040255.1	87346341	T	C
NC_040255.1	87426815	G	A
NC_040255.1	87514743	G	A
NC_040255.1	87567978	A	C
NC_040255.1	87615344	C	G
NC_040255.1	87678849	T	C
NC_040255.1	87751985	T	C
NC_040255.1	87816367	T	A
NC_040255.1	87872415	A	G
NC_040255.1	87932049	A	G
NC_040255.1	87987048	C	T
NC_040255.1	88052194	T	A
NC_040255.1	88108563	A	G
NC_040255.1	88173280	C	T
NC_040255.1	88228146	T	C

NC_040255.1	88289700	C	T
NC_040255.1	88348293	T	C
NC_040255.1	88450310	C	T
NC_040255.1	88514589	A	G
NC_040255.1	88575768	A	G
NC_040255.1	88601409	A	G
NC_040255.1	88646726	A	G
NC_040255.1	88725394	C	T
NC_040255.1	88844899	G	C
NC_040255.1	88908770	T	C
NC_040255.1	88968310	G	C
NC_040255.1	89023688	T	C
NC_040255.1	89074482	C	A
NC_040255.1	89114897	G	A
NC_040255.1	89188848	T	C
NC_040255.1	89298866	G	A
NC_040255.1	89355690	G	C
NC_040255.1	89412288	T	C
NC_040255.1	89469907	T	C
NC_040255.1	89521090	T	C
NC_040255.1	89615553	C	T
NC_040255.1	89667699	G	A
NC_040255.1	89770666	G	A
NC_040255.1	89832458	T	C
NC_040255.1	89977518	G	C
NC_040255.1	90042166	G	A
NC_040255.1	90087041	A	G
NC_040255.1	90319982	A	G
NC_040255.1	90399250	G	A
NC_040255.1	90526043	G	T
NC_040255.1	90553892	C	T
NC_040255.1	90589233	C	T
NC_040255.1	90634697	A	G
NC_040255.1	90694982	C	A
NC_040255.1	90912442	C	T
NC_040255.1	91106957	G	C
NC_040255.1	91234584	A	C
NC_040255.1	91311088	T	C
NC_040255.1	91359200	C	A
NC_040255.1	91686861	T	G
NC_040255.1	91770443	T	C
NC_040255.1	91835977	G	A
NC_040255.1	91936653	T	A
NC_040255.1	92095541	A	T
NC_040255.1	92164689	G	A
NC_040255.1	92245429	C	T
NC_040255.1	92337855	G	C

NC_040255.1	92409221	T	C
NC_040255.1	92485534	C	T
NC_040255.1	92541633	T	G
NC_040255.1	92681952	A	G
NC_040255.1	92712717	C	G
NC_040255.1	92802665	G	T
NC_040255.1	92853901	G	A
NC_040255.1	92893841	T	C
NC_040255.1	92947703	T	C
NC_040255.1	92987578	T	G
NC_040255.1	93044029	G	C
NC_040255.1	93078829	A	G
NC_040255.1	93128707	T	C
NC_040255.1	93175762	A	G
NC_040255.1	93245068	C	G
NC_040255.1	93329765	G	A
NC_040255.1	93421821	A	C
NC_040255.1	93495209	A	G
NC_040255.1	93537338	T	C
NC_040255.1	93597385	T	C
NC_040255.1	93681932	T	C
NC_040255.1	93736746	C	T
NC_040255.1	93797568	C	T
NC_040255.1	93865073	G	A
NC_040255.1	93920753	G	C
NC_040255.1	93970575	G	T
NC_040255.1	94028960	A	G
NC_040255.1	94093787	C	T
NC_040255.1	94150064	C	T
NC_040255.1	94219168	T	C
NC_040255.1	94270138	C	T
NC_040255.1	94318323	T	C
NC_040255.1	94382942	A	C
NC_040255.1	94455560	T	A
NC_040255.1	94523304	A	G
NC_040255.1	94649530	T	C
NC_040255.1	94708702	A	G
NC_040255.1	94778290	G	A
NC_040255.1	94804919	A	G
NC_040255.1	94862877	A	C
NC_040255.1	94925687	T	G
NC_040255.1	94988783	A	G
NC_040255.1	95060233	C	G
NC_040255.1	95121239	T	C
NC_040255.1	95206812	C	G
NC_040255.1	95216817	A	G
NC_040255.1	95242086	T	C

NC_040255.1	95342954	C	T
NC_040255.1	95393024	T	C
NC_040255.1	95436426	C	T
NC_040255.1	95486387	A	G
NC_040255.1	95577079	C	G
NC_040255.1	95655081	C	T
NC_040255.1	95874393	G	C
NC_040255.1	95926660	G	A
NC_040255.1	95995636	A	C
NC_040255.1	96096844	G	T
NC_040255.1	96143573	C	G
NC_040255.1	96183938	T	C
NC_040255.1	96230247	G	A
NC_040255.1	96293461	C	T
NC_040255.1	96367699	T	C
NC_040255.1	96446725	T	C
NC_040255.1	96479064	T	C
NC_040255.1	96597869	T	C
NC_040255.1	96664738	A	T
NC_040255.1	96735711	A	G
NC_040255.1	96814401	T	C
NC_040255.1	96870101	C	G
NC_040255.1	97156844	A	G
NC_040255.1	97224266	C	T
NC_040255.1	97320689	A	C
NC_040255.1	97340257	T	C
NC_040255.1	97414993	C	T
NC_040255.1	97490066	A	G
NC_040255.1	97616484	T	A
NC_040255.1	97744339	T	C
NC_040255.1	97803310	T	C
NC_040255.1	97865957	T	A
NC_040255.1	98010885	A	G
NC_040255.1	98040717	T	A
NC_040255.1	98186020	C	T
NC_040255.1	98247700	A	G
NC_040255.1	98314197	T	C
NC_040255.1	98573474	C	T
NC_040255.1	98603670	C	G
NC_040255.1	98697503	G	A
NC_040255.1	98736241	A	G
NC_040255.1	99176403	C	A
NC_040255.1	99249478	A	G
NC_040255.1	99352041	C	G
NC_040255.1	99372932	A	G
NC_040255.1	99462936	A	T
NC_040255.1	99508050	G	A

NC_040255.1	99597353	A	C
NC_040255.1	99663371	G	A
NC_040255.1	100080174	G	T
NC_040255.1	100131381	C	A
NC_040255.1	100192897	C	T
NC_040255.1	100260736	A	G
NC_040255.1	100311339	T	C
NC_040255.1	100365625	T	C
NC_040255.1	100408737	G	A
NC_040255.1	100481463	T	C
NC_040255.1	100587008	A	T
NC_040255.1	100652414	G	A
NC_040255.1	100664259	A	G
NC_040255.1	100681554	C	T
NC_040255.1	100728086	C	T
NC_040255.1	100784404	A	G
NC_040255.1	100843474	G	A
NC_040255.1	100897781	T	C
NC_040255.1	100946632	T	C
NC_040255.1	101004892	T	A
NC_040255.1	101052260	G	A
NC_040255.1	101113556	A	G
NC_040255.1	101171566	A	T
NC_040255.1	101227724	T	G
NC_040255.1	101301104	T	C
NC_040255.1	101364879	A	G
NC_040255.1	101390846	A	G
NC_040255.1	101460324	C	T
NC_040255.1	101508043	T	C
NC_040255.1	101545557	C	T
NC_040255.1	101600645	T	C
NC_040255.1	101619142	A	G
NC_040255.1	101651636	C	T
NC_040255.1	101691464	T	G
NC_040255.1	101739106	T	C
NC_040255.1	101809163	T	G
NC_040255.1	101900719	A	G
NC_040255.1	101953535	A	G
NC_040255.1	101997744	A	C
NC_040255.1	102055311	A	G
NC_040255.1	102113265	C	A
NC_040255.1	102158148	T	C
NC_040255.1	102211104	G	A
NC_040255.1	102256058	C	G
NC_040255.1	102318055	C	T
NC_040255.1	102373373	T	C
NC_040255.1	102428095	A	C

NC_040255.1	102478842	T	A
NC_040255.1	102549976	A	G
NC_040255.1	102601106	T	C
NC_040255.1	102651361	T	C
NC_040255.1	102706502	A	G
NC_040255.1	102735386	G	C
NC_040255.1	102763865	C	T
NC_040255.1	102823679	T	G
NC_040255.1	102880115	A	G
NC_040255.1	102914949	C	G
NC_040255.1	102921403	T	C
NC_040255.1	102939425	A	G
NC_040255.1	102968118	T	C
NC_040255.1	103039632	C	T
NC_040255.1	103079072	A	G
NC_040255.1	103138097	G	A
NC_040255.1	103188168	C	T
NC_040255.1	103259374	A	G
NC_040255.1	103342555	A	G
NC_040255.1	103426869	A	G
NC_040255.1	103494278	A	G
NC_040255.1	103558254	C	A
NC_040255.1	103610233	C	A
NC_040255.1	103668564	G	A
NC_040255.1	103719846	A	G
NC_040255.1	103790630	T	C
NC_040255.1	103860721	C	T
NC_040255.1	103908284	A	C
NC_040255.1	103968322	G	A
NC_040255.1	104024547	G	A
NC_040255.1	104031190	G	T
NC_040255.1	104038947	C	G
NC_040255.1	104087388	C	A
NC_040255.1	104150151	T	C
NC_040255.1	104208064	A	T
NC_040255.1	104283148	T	C
NC_040255.1	104334834	A	T
NC_040255.1	104392574	A	G
NC_040255.1	104450308	A	G
NC_040255.1	104503585	T	C
NC_040255.1	104551842	T	C
NC_040255.1	104604206	A	G
NC_040255.1	104662674	T	C
NC_040255.1	104712114	T	C
NC_040255.1	104768355	C	T
NC_040255.1	104834882	G	A
NC_040255.1	104904454	A	C

NC_040255.1	104968272	A	G
NC_040255.1	105024279	T	C
NC_040255.1	105078416	A	G
NC_040255.1	105132841	T	C
NC_040255.1	105190875	T	C
NC_040255.1	105247507	C	A
NC_040255.1	105299759	T	C
NC_040255.1	105355936	T	C
NC_040255.1	105410671	T	C
NC_040255.1	105470514	G	A
NC_040255.1	105527955	A	G
NC_040255.1	105586331	C	T
NC_040255.1	105622300	C	A
NC_040255.1	105680785	A	G
NC_040255.1	105744299	T	A
NC_040255.1	105806450	T	G
NC_040255.1	105849189	T	C
NC_040255.1	105906227	C	T
NC_040255.1	105975907	T	C
NC_040255.1	106030312	G	T
NC_040255.1	106088805	G	T
NC_040255.1	106141461	C	G
NC_040255.1	106198370	T	C
NC_040255.1	106259097	G	T
NC_040255.1	106307704	G	A
NC_040255.1	106363738	G	A
NC_040255.1	106397542	C	T
NC_040255.1	106455148	G	A
NC_040255.1	106498298	G	A
NC_040255.1	106539086	A	G
NC_040255.1	106594312	G	A
NC_040255.1	106633273	T	C
NC_040255.1	106694131	G	A
NC_040255.1	106751153	A	G
NC_040255.1	106806188	G	A
NC_040255.1	106866366	A	G
NC_040255.1	106916485	T	C
NC_040255.1	106963276	G	A
NC_040255.1	107017281	G	A
NC_040255.1	107086893	A	G
NC_040255.1	107132946	G	A
NC_040255.1	107190620	G	C
NC_040255.1	107247626	C	G
NC_040255.1	107304622	G	A
NC_040255.1	107359857	A	G
NC_040255.1	107414888	G	A
NC_040255.1	107459863	G	A

NC_040255.1	107471873	G	A
NC_040255.1	107644933	A	G
NC_040255.1	107692489	T	A
NC_040255.1	107693523	T	C
NC_040255.1	107715566	G	A
NC_040255.1	107729103	A	G
NC_040255.1	107788595	T	G
NC_040255.1	107841057	G	C
NC_040255.1	107891774	G	T
NC_040255.1	107924069	G	A
NC_040255.1	107976297	C	A
NC_040255.1	108030663	C	A
NC_040255.1	108086455	C	T
NC_040255.1	108124541	A	T
NC_040255.1	108176411	G	A
NC_040255.1	108199322	A	G
NC_040255.1	108241656	C	A
NC_040255.1	108298904	G	C
NC_040255.1	108356391	T	C
NC_040255.1	108404263	G	T
NC_040255.1	108451934	C	T
NC_040255.1	108516808	G	A
NC_040255.1	108583769	C	A
NC_040255.1	108642148	C	T
NC_040255.1	108713865	G	A
NC_040255.1	108791730	T	C
NC_040255.1	108847731	A	C
NC_040255.1	108901354	C	T
NC_040255.1	108959781	C	T
NC_040255.1	109018621	A	G
NC_040255.1	109072085	T	C
NC_040255.1	109117316	A	G
NC_040255.1	109154904	T	A
NC_040255.1	109247251	C	G
NC_040255.1	109324162	A	G
NC_040255.1	109422085	0	0
NC_040255.1	109478353	T	C
NC_040255.1	109530257	T	C
NC_040255.1	109581748	C	T
NC_040255.1	109633896	A	G
NC_040255.1	109673513	T	C
NC_040255.1	109675613	G	C
NC_040255.1	109678121	C	A
NC_040255.1	109736306	A	G
NC_040255.1	109790543	G	A
NC_040255.1	109812119	G	A
NC_040255.1	109857226	T	A

NC_040255.1	109917240	T	C
NC_040255.1	109988751	G	T
NC_040255.1	110037511	C	G
NC_040255.1	110088379	C	T
NC_040255.1	110155427	G	A
NC_040255.1	110232969	G	A
NC_040255.1	110288686	G	T
NC_040255.1	110344512	T	C
NC_040255.1	110398925	A	G
NC_040255.1	110455893	A	G
NC_040255.1	110518093	T	C
NC_040255.1	110519742	A	G
NC_040255.1	110520573	T	A
NC_040255.1	110574018	A	G
NC_040255.1	110627312	T	C
NC_040255.1	110683426	C	G
NC_040255.1	110751937	C	A
NC_040255.1	110810217	C	G
NC_040255.1	110854848	T	A
NC_040255.1	110910473	G	A
NC_040255.1	111025312	A	G
NC_040255.1	111084140	G	A
NC_040255.1	111142053	G	A
NC_040255.1	111200847	C	T
NC_040255.1	111261484	C	G
NC_040255.1	111321996	A	C
NC_040255.1	111368685	G	C
NC_040255.1	111422146	T	C
NC_040255.1	111423161	T	C
NC_040255.1	111424532	T	C
NC_040255.1	111425815	T	A
NC_040255.1	111426418	A	G
NC_040255.1	111439070	T	C
NC_040255.1	111491963	T	C
NC_040255.1	111544486	C	T
NC_040255.1	111590247	T	A
NC_040255.1	111602915	T	C
NC_040255.1	111669735	A	G
NC_040255.1	111702631	C	T
NC_040255.1	111762863	A	G
NC_040255.1	111772840	G	T
NC_040255.1	111773931	G	A
NC_040255.1	111775150	G	A
NC_040255.1	111775932	T	C
NC_040255.1	111777976	A	G
NC_040255.1	111780063	A	G
NC_040255.1	111802823	A	C

NC_040255.1	111846242	C	T
NC_040255.1	111883385	T	C
NC_040255.1	111945934	C	G
NC_040255.1	111947808	G	C
NC_040255.1	111957527	A	G
NC_040255.1	111958809	A	G
NC_040255.1	111962026	G	A
NC_040255.1	111962910	T	C
NC_040255.1	111964197	A	G
NC_040255.1	111965339	G	A
NC_040255.1	111965807	T	C
NC_040255.1	111968026	A	G
NC_040255.1	112019139	G	A
NC_040255.1	112059655	A	G
NC_040255.1	112097216	A	G
NC_040255.1	112150739	A	G
NC_040255.1	112202786	T	G
NC_040255.1	112253987	T	C
NC_040255.1	112308049	G	C
NC_040255.1	112355129	A	G
NC_040255.1	112402893	G	A
NC_040255.1	112438705	T	C
NC_040255.1	112440505	C	T
NC_040255.1	112441458	G	A
NC_040255.1	112450456	T	C
NC_040255.1	112503976	C	T
NC_040255.1	112560827	C	T
NC_040255.1	112607790	C	A
NC_040255.1	112649372	A	G
NC_040255.1	112694754	A	T
NC_040255.1	112752220	A	G
NC_040255.1	112792259	T	C
NC_040255.1	112855192	A	C
NC_040255.1	112905883	T	C
NC_040255.1	112934543	C	T
NC_040255.1	112935090	A	G
NC_040255.1	112937964	G	C
NC_040255.1	112940085	A	G
NC_040255.1	112994643	G	A
NC_040255.1	113047316	T	A
NC_040255.1	113101275	G	C
NC_040255.1	113150950	C	T
NC_040255.1	113208154	A	C
NC_040255.1	113260997	G	A
NC_040255.1	113308961	T	C
NC_040255.1	113363690	T	C
NC_040255.1	113418379	C	T

NC_040255.1	113475070	A	G
NC_040255.1	113533721	T	C
NC_040255.1	113575729	C	T
NC_040255.1	113621285	A	G
NC_040255.1	113673665	T	C
NC_040255.1	113716812	A	G
NC_040255.1	113771834	T	C
NC_040255.1	113795996	T	G
NC_040255.1	113842103	A	C
NC_040255.1	113907553	C	T
NC_040255.1	113940355	T	C
NC_040255.1	113968493	A	G
NC_040255.1	114006228	A	G
NC_040255.1	114064875	A	G
NC_040255.1	114109651	A	G
NC_040255.1	114150285	G	A
NC_040255.1	114181126	T	C
NC_040255.1	114187684	T	C
NC_040255.1	114192041	T	C
NC_040255.1	114236656	T	C
NC_040255.1	114298682	T	C
NC_040255.1	114355403	A	C
NC_040255.1	114424285	T	C
NC_040255.1	114455944	A	G
NC_040255.1	114602784	C	G
NC_040255.1	114665799	C	T
NC_040255.1	114719268	T	C
NC_040255.1	114773728	C	A
NC_040255.1	114818060	G	T
NC_040255.1	114873200	T	G
NC_040255.1	114917115	A	C
NC_040255.1	114948508	G	A
NC_040255.1	114978987	G	A
NC_040255.1	115129638	A	G
NC_040255.1	115187471	C	T
NC_040255.1	115230846	G	A
NC_040255.1	115285938	T	C
NC_040255.1	115338820	T	C
NC_040255.1	115391439	A	G
NC_040255.1	115452556	C	T
NC_040255.1	115488267	A	G
NC_040255.1	115491058	G	C
NC_040255.1	115512222	T	G
NC_040255.1	115515030	T	C
NC_040255.1	115531677	T	C
NC_040255.1	115542680	T	C
NC_040255.1	115575797	G	T

NC_040255.1	115575950	T	C
NC_040255.1	115576481	T	C
NC_040255.1	115623787	A	G
NC_040255.1	115632238	T	C
NC_040255.1	115633634	A	G
NC_040255.1	115683556	T	C
NC_040255.1	115712882	G	T
NC_040255.1	115765735	T	C
NC_040255.1	115830237	T	C
NC_040255.1	115834674	T	C
NC_040255.1	115834877	C	T
NC_040255.1	115863097	A	G
NC_040255.1	115956091	G	C
NC_040255.1	116010450	G	A
NC_040255.1	116077485	A	G
NC_040255.1	116143017	C	T
NC_040255.1	116186204	A	G
NC_040255.1	116234112	A	G
NC_040255.1	116316005	C	T
NC_040255.1	116374174	G	A
NC_040255.1	116435942	T	C
NC_040255.1	116489204	T	G
NC_040255.1	116544143	A	G
NC_040255.1	116544394	C	T
NC_040255.1	116545895	T	C
NC_040255.1	116546573	T	C
NC_040255.1	116546823	T	C
NC_040255.1	116548287	T	C
NC_040255.1	116586601	A	G
NC_040255.1	116616649	A	G
NC_040255.1	116664749	G	T
NC_040255.1	116714556	A	G
NC_040255.1	116770619	T	A
NC_040255.1	116822280	C	A
NC_040255.1	116877935	C	T
NC_040255.1	116937229	C	T
NC_040255.1	117106572	G	A
NC_040255.1	117161089	G	A
NC_040255.1	117213942	A	G
NC_040255.1	117261765	A	G
NC_040255.1	117314724	A	C
NC_040255.1	117452234	T	A
NC_040255.1	117506108	C	T
NC_040255.1	117554970	G	C
NC_040255.1	117593784	T	C
NC_040255.1	117776559	T	A
NC_040255.1	117840016	G	A

NC_040255.1	117895613	A	G
NC_040255.1	117949260	T	C
NC_040255.1	117986946	T	C
NC_040255.1	118034446	T	C
NC_040255.1	118088280	C	T
NC_040255.1	118131747	T	G
NC_040255.1	118193039	A	G
NC_040255.1	118262111	C	G
NC_040255.1	118316257	T	G
NC_040255.1	118365042	G	A
NC_040255.1	118423068	A	G
NC_040255.1	118489510	T	C
NC_040255.1	118558528	T	C
NC_040255.1	118614333	A	G
NC_040255.1	118670233	G	A
NC_040255.1	118721684	G	T
NC_040255.1	118790262	A	G
NC_040255.1	118999418	A	G
NC_040255.1	119051059	A	G
NC_040255.1	119127453	G	A
NC_040255.1	119173800	T	C
NC_040255.1	119230849	C	G
NC_040255.1	119272761	T	C
NC_040255.1	119357038	G	A
NC_040255.1	119413667	A	G
NC_040255.1	119474839	T	G
NC_040255.1	119537919	C	T
NC_040255.1	119586907	A	C
NC_040255.1	119647508	G	A
NC_040255.1	119707038	G	A
NC_040255.1	119755498	A	G
NC_040255.1	119805876	C	T
NC_040255.1	119868712	T	C
NC_040255.1	119924107	T	C
NC_040255.1	119970976	A	G
NC_040255.1	120025570	T	C
NC_040255.1	120079854	A	G
NC_040255.1	120120129	G	C
NC_040255.1	120155100	G	A
NC_040255.1	120252953	T	C
NC_040255.1	120265166	C	T
NC_040255.1	120319343	C	T
NC_040255.1	120376334	G	A
NC_040255.1	120433041	C	G
NC_040255.1	120510719	T	A
NC_040255.1	120546386	G	C
NC_040255.1	120602274	T	C

NC_040255.1	120648809	C	T
NC_040255.1	120711209	T	G
NC_040255.1	120753305	A	G
NC_040255.1	120806490	G	A
NC_040255.1	120859968	T	C
NC_040255.1	120917308	G	C
NC_040255.1	120984423	C	T
NC_040255.1	121037786	G	T
NC_040255.1	121091041	A	G
NC_040255.1	121147493	C	T
NC_040255.1	121206829	T	C
NC_040255.1	121282037	A	G
NC_040255.1	121282349	A	G
NC_040255.1	121283481	T	C
NC_040255.1	121285180	A	G
NC_040255.1	121285410	A	C
NC_040255.1	121336141	T	C
NC_040255.1	121374883	A	C
NC_040255.1	121430152	G	A
NC_040255.1	121475761	G	T
NC_040255.1	121532337	T	C
NC_040255.1	121556898	C	T
NC_040255.1	121564663	A	T
NC_040255.1	121569419	A	G
NC_040255.1	121620420	G	A
NC_040255.1	121676612	G	A
NC_040255.1	121732567	0	G
NC_040255.1	121783796	A	G
NC_040255.1	121829744	G	T
NC_040255.1	121835383	T	C
NC_040255.1	121835571	C	G
NC_040255.1	121836755	C	G
NC_040255.1	121837149	T	C
NC_040255.1	121837925	G	C
NC_040255.1	121839210	A	G
NC_040255.1	121839416	A	G
NC_040255.1	121851307	A	G
NC_040255.1	121900549	A	T
NC_040255.1	121961948	A	C
NC_040255.1	121996738	T	C
NC_040255.1	122062224	T	C
NC_040255.1	122131966	T	C
NC_040255.1	122140163	G	C
NC_040255.1	122142838	T	C
NC_040255.1	122143585	G	A
NC_040255.1	122144139	T	C
NC_040255.1	122144507	T	C

NC_040255.1	122144916	G	A
NC_040255.1	122175130	A	T
NC_040255.1	122195729	A	G
NC_040255.1	122200611	G	A
NC_040255.1	122207038	G	C
NC_040255.1	122211281	A	G
NC_040255.1	122226382	T	C
NC_040255.1	122232770	T	C
NC_040255.1	122234573	A	G
NC_040255.1	122254777	G	A
NC_040255.1	122272232	A	G
NC_040255.1	122277217	C	T
NC_040255.1	122277676	T	C
NC_040255.1	122278646	A	G
NC_040255.1	122316025	C	G
NC_040255.1	122345603	C	T
NC_040255.1	122346185	T	C
NC_040255.1	122346487	A	C
NC_040255.1	122346758	T	C
NC_040255.1	122362281	T	C
NC_040255.1	122384486	A	G
NC_040255.1	122444888	A	G
NC_040255.1	122519363	A	T
NC_040255.1	122559789	A	G
NC_040255.1	122678438	C	T
NC_040255.1	122787304	G	A
NC_040255.1	122835092	G	C
NC_040255.1	123336223	A	G
NC_040255.1	123395156	G	T
NC_040255.1	123446396	A	G
NC_040255.1	123522840	C	A
NC_040255.1	123579079	G	A
NC_040255.1	123630573	T	G
NC_040255.1	123647191	T	C
NC_040255.1	123653435	A	G
NC_040255.1	123658845	A	G
NC_040255.1	123659151	T	A
NC_040255.1	123660497	G	A
NC_040255.1	123663201	T	G
NC_040255.1	123666459	C	G
NC_040255.1	123666638	T	C
NC_040255.1	123668960	C	T
NC_040255.1	123685525	T	C
NC_040255.1	123697264	A	G
NC_040255.1	123709354	T	A
NC_040255.1	123710395	A	G
NC_040255.1	123710767	T	C

NC_040255.1	123718561	A	G
NC_040255.1	123719650	T	C
NC_040255.1	123720602	T	C
NC_040255.1	123780233	T	C
NC_040255.1	123804366	G	A
NC_040255.1	123811986	A	G
NC_040255.1	123812354	A	G
NC_040255.1	123833715	T	C
NC_040255.1	123846629	T	C
NC_040255.1	123849876	C	G
NC_040255.1	123850299	C	T
NC_040255.1	123863086	C	G
NC_040255.1	123871856	G	C
NC_040255.1	123875053	A	G
NC_040255.1	123876802	G	A
NC_040255.1	123881009	T	C
NC_040255.1	123939202	G	T
NC_040255.1	123983114	G	C
NC_040255.1	124025017	A	G
NC_040255.1	124047586	A	G
NC_040255.1	124108128	A	G
NC_040255.1	124168295	T	C
NC_040255.1	124215090	T	C
NC_040255.1	124283837	A	G
NC_040255.1	124327898	G	A
NC_040255.1	124372342	C	T
NC_040255.1	124433731	G	C
NC_040255.1	124481003	A	T
NC_040255.1	124547202	G	A
NC_040255.1	124581948	T	C
NC_040255.1	124618479	C	A
NC_040255.1	124651761	T	C
NC_040255.1	124661096	A	G
NC_040255.1	124662473	A	G
NC_040255.1	124671202	T	C
NC_040255.1	124680619	A	G
NC_040255.1	124680793	A	T
NC_040255.1	124689637	T	C
NC_040255.1	124694103	T	C
NC_040255.1	124711222	T	C
NC_040255.1	124747391	T	C
NC_040255.1	124802366	C	T
NC_040255.1	124861556	T	C
NC_040255.1	124917454	A	G
NC_040255.1	124918168	C	A
NC_040255.1	124970224	A	G
NC_040255.1	125026684	C	T

NC_040255.1	125090861	G	A
NC_040255.1	125151288	T	C
NC_040255.1	125223614	A	G
NC_040255.1	125276321	T	C
NC_040255.1	125334817	G	A
NC_040255.1	125392486	G	A
NC_040255.1	125451746	A	G
NC_040255.1	125498632	A	G
NC_040255.1	125561683	G	A
NC_040255.1	125605635	A	T
NC_040255.1	125688202	T	C
NC_040255.1	125761275	G	A
NC_040255.1	125804867	C	T
NC_040255.1	125877425	A	C
NC_040255.1	125937429	T	G
NC_040255.1	125999448	A	C
NC_040255.1	126058424	C	T
NC_040255.1	126109498	T	C
NC_040255.1	126161003	T	G
NC_040255.1	126380159	C	T
NC_040255.1	126516484	G	T
NC_040255.1	126562117	C	T
NC_040255.1	126617862	C	T
NC_040255.1	126646656	T	C
NC_040255.1	126705280	T	C
NC_040255.1	126763166	T	C
NC_040255.1	126809588	G	C
NC_040255.1	126863554	C	T
NC_040255.1	126913088	A	C
NC_040255.1	126972090	A	C
NC_040255.1	127033028	A	G
NC_040255.1	127076785	A	G
NC_040255.1	127080948	T	C
NC_040255.1	127090467	A	G
NC_040255.1	127138035	A	G
NC_040255.1	127195496	T	C
NC_040255.1	127248450	T	G
NC_040255.1	127304433	A	G
NC_040255.1	127354487	G	C
NC_040255.1	127404887	A	G
NC_040255.1	127461052	A	G
NC_040255.1	127517953	G	T
NC_040255.1	127571516	A	G
NC_040255.1	127592932	A	G
NC_040255.1	127600223	A	G
NC_040255.1	127601196	A	G
NC_040255.1	127601920	G	A

NC_040255.1	127656203	C	T
NC_040255.1	127721392	G	A
NC_040255.1	127773408	T	C
NC_040255.1	127831272	A	G
NC_040255.1	127888732	A	C
NC_040255.1	127942667	A	G
NC_040255.1	128010958	G	C
NC_040255.1	128069370	C	T
NC_040255.1	128122572	A	G
NC_040255.1	128177278	T	C
NC_040255.1	128225487	A	G
NC_040255.1	128265148	C	T
NC_040255.1	128304091	A	G
NC_040255.1	128304252	A	G
NC_040255.1	128304659	G	A
NC_040255.1	128360718	T	C
NC_040255.1	128416463	A	G
NC_040255.1	128532498	T	C
NC_040255.1	128591853	G	C
NC_040255.1	128648826	A	G
NC_040255.1	128695924	C	G
NC_040255.1	128746268	A	G
NC_040255.1	128816134	T	C
NC_040255.1	128886429	T	C
NC_040255.1	128912729	G	A
NC_040255.1	128913686	A	G
NC_040255.1	128913874	A	G
NC_040255.1	128914109	T	C
NC_040255.1	128980213	A	G
NC_040255.1	129029979	A	G
NC_040255.1	129069975	A	G
NC_040255.1	129164266	A	G
NC_040255.1	129227520	A	G
NC_040255.1	129284123	A	G
NC_040255.1	129342866	C	T
NC_040255.1	129403405	T	C
NC_040255.1	129460639	T	C
NC_040255.1	129517899	T	C
NC_040255.1	129574310	A	G
NC_040255.1	129604933	A	G
NC_040255.1	129631708	T	C
NC_040255.1	129637775	T	C
NC_040255.1	129702471	G	T
NC_040255.1	129770850	T	C
NC_040255.1	129786530	T	C
NC_040255.1	129840908	G	A
NC_040255.1	129896820	T	C

NC_040255.1	129955858	A	G
NC_040255.1	130016121	G	A
NC_040256.1	45671	A	T
NC_040256.1	102361	A	G
NC_040256.1	158588	G	C
NC_040256.1	214424	A	G
NC_040256.1	215070	T	G
NC_040256.1	258500	A	G
NC_040256.1	301709	A	G
NC_040256.1	316416	A	T
NC_040256.1	317351	A	G
NC_040256.1	352194	T	C
NC_040256.1	352879	T	C
NC_040256.1	353560	G	A
NC_040256.1	353767	G	A
NC_040256.1	378311	A	G
NC_040256.1	439178	T	C
NC_040256.1	544548	G	A
NC_040256.1	716841	G	C
NC_040256.1	760737	G	A
NC_040256.1	809248	T	C
NC_040256.1	856081	A	T
NC_040256.1	933024	T	C
NC_040256.1	933527	T	G
NC_040256.1	934991	T	C
NC_040256.1	981120	T	C
NC_040256.1	1047145	A	C
NC_040256.1	1106915	A	C
NC_040256.1	1163723	C	T
NC_040256.1	1216704	G	A
NC_040256.1	1250868	T	C
NC_040256.1	1309587	T	C
NC_040256.1	1354826	A	G
NC_040256.1	1377502	T	C
NC_040256.1	1436349	A	G
NC_040256.1	1471033	T	C
NC_040256.1	1532992	T	C
NC_040256.1	1533269	T	C
NC_040256.1	1587722	T	C
NC_040256.1	1619775	G	A
NC_040256.1	1678534	T	C
NC_040256.1	1742380	T	C
NC_040256.1	1798241	A	G
NC_040256.1	1857227	C	G
NC_040256.1	1908151	A	G
NC_040256.1	1963703	A	G
NC_040256.1	2014914	C	T

NC_040256.1	2076221	A	G
NC_040256.1	2138224	A	G
NC_040256.1	2189737	G	T
NC_040256.1	2209215	T	C
NC_040256.1	2272205	T	C
NC_040256.1	2322228	T	A
NC_040256.1	2366422	C	T
NC_040256.1	2368867	C	T
NC_040256.1	2369810	T	C
NC_040256.1	2420224	C	T
NC_040256.1	2422819	C	T
NC_040256.1	2452652	T	C
NC_040256.1	2473230	T	C
NC_040256.1	2534201	T	C
NC_040256.1	2558807	C	G
NC_040256.1	2561652	T	G
NC_040256.1	2584451	G	A
NC_040256.1	2628859	T	C
NC_040256.1	2629387	T	C
NC_040256.1	2631652	G	A
NC_040256.1	2676593	T	A
NC_040256.1	2680474	G	A
NC_040256.1	2728780	T	C
NC_040256.1	2768764	A	G
NC_040256.1	2771751	A	G
NC_040256.1	2850148	G	C
NC_040256.1	2901238	A	G
NC_040256.1	2966650	T	C
NC_040256.1	2975528	A	G
NC_040256.1	2975865	C	T
NC_040256.1	2976110	A	G
NC_040256.1	2977126	G	C
NC_040256.1	2998532	G	C
NC_040256.1	2999219	A	G
NC_040256.1	3037672	A	G
NC_040256.1	3093658	T	G
NC_040256.1	3148899	C	T
NC_040256.1	3202519	C	A
NC_040256.1	3260198	A	T
NC_040256.1	3305107	C	A
NC_040256.1	3371942	G	A
NC_040256.1	3423181	C	T
NC_040256.1	3474171	T	C
NC_040256.1	3485228	A	G
NC_040256.1	3488114	T	C
NC_040256.1	3491264	A	G
NC_040256.1	3492941	C	G

NC_040256.1	3554458	T	C
NC_040256.1	3561775	G	A
NC_040256.1	3563525	T	G
NC_040256.1	3648826	A	G
NC_040256.1	3716686	C	T
NC_040256.1	3716943	A	G
NC_040256.1	3725963	G	A
NC_040256.1	3726192	T	C
NC_040256.1	3737831	G	C
NC_040256.1	3741520	T	C
NC_040256.1	3743375	A	G
NC_040256.1	3745952	T	A
NC_040256.1	3749984	A	G
NC_040256.1	3813295	A	G
NC_040256.1	3836750	C	G
NC_040256.1	3836973	G	C
NC_040256.1	3838320	A	G
NC_040256.1	3839744	A	G
NC_040256.1	3844250	G	T
NC_040256.1	3844782	A	G
NC_040256.1	3850666	A	G
NC_040256.1	3850842	A	G
NC_040256.1	3868510	G	C
NC_040256.1	3869000	A	G
NC_040256.1	3925485	C	T
NC_040256.1	3978542	T	C
NC_040256.1	3979642	T	C
NC_040256.1	3979858	T	C
NC_040256.1	3980904	T	C
NC_040256.1	3981482	C	A
NC_040256.1	4030530	A	G
NC_040256.1	4064354	C	G
NC_040256.1	4118084	G	A
NC_040256.1	4127764	A	T
NC_040256.1	4157197	C	G
NC_040256.1	4157869	T	C
NC_040256.1	4222086	T	C
NC_040256.1	4225803	C	T
NC_040256.1	4251640	T	C
NC_040256.1	4255884	A	G
NC_040256.1	4264265	T	C
NC_040256.1	4326187	C	T
NC_040256.1	4331498	A	G
NC_040256.1	4333657	G	A
NC_040256.1	4370511	A	G
NC_040256.1	4380131	T	C
NC_040256.1	4381867	T	G

NC_040256.1	4383112	A	G
NC_040256.1	4383277	T	C
NC_040256.1	4383750	A	G
NC_040256.1	4395656	G	A
NC_040256.1	4397660	A	G
NC_040256.1	4458796	G	A
NC_040256.1	4474469	T	C
NC_040256.1	4476812	T	C
NC_040256.1	4478816	G	T
NC_040256.1	4480516	T	C
NC_040256.1	4480701	A	G
NC_040256.1	4481643	C	T
NC_040256.1	4483700	C	G
NC_040256.1	4484669	T	C
NC_040256.1	4509419	T	C
NC_040256.1	4565260	C	G
NC_040256.1	4578165	A	G
NC_040256.1	4578617	C	T
NC_040256.1	4578785	A	G
NC_040256.1	4579214	A	G
NC_040256.1	4633642	T	G
NC_040256.1	4634969	G	C
NC_040256.1	4641276	T	C
NC_040256.1	4648282	A	G
NC_040256.1	4660067	A	G
NC_040256.1	4664767	G	A
NC_040256.1	4686928	T	C
NC_040256.1	4756393	A	C
NC_040256.1	4776981	T	C
NC_040256.1	4826472	G	T
NC_040256.1	4869311	T	C
NC_040256.1	4869867	G	A
NC_040256.1	4930021	T	C
NC_040256.1	4946839	G	C
NC_040256.1	4947374	A	G
NC_040256.1	4963702	T	C
NC_040256.1	4963922	G	T
NC_040256.1	4964815	G	A
NC_040256.1	4965804	T	C
NC_040256.1	4980625	T	C
NC_040256.1	5003315	A	G
NC_040256.1	5005186	T	C
NC_040256.1	5019262	T	C
NC_040256.1	5019450	T	C
NC_040256.1	5020738	A	G
NC_040256.1	5062417	A	G
NC_040256.1	5071361	A	G

NC_040256.1	5071850	C	T
NC_040256.1	5073864	T	C
NC_040256.1	5075261	G	A
NC_040256.1	5076034	T	C
NC_040256.1	5076400	T	C
NC_040256.1	5076588	G	A
NC_040256.1	5076753	G	C
NC_040256.1	5085755	G	A
NC_040256.1	5095830	C	T
NC_040256.1	5140385	C	T
NC_040256.1	5176511	A	T
NC_040256.1	5184451	T	G
NC_040256.1	5206448	C	G
NC_040256.1	5266762	C	T
NC_040256.1	5269399	T	A
NC_040256.1	5317409	A	G
NC_040256.1	5319166	C	G
NC_040256.1	5320714	G	C
NC_040256.1	5324736	C	G
NC_040256.1	5324924	A	G
NC_040256.1	5350479	G	A
NC_040256.1	5365394	G	C
NC_040256.1	5380302	T	C
NC_040256.1	5429171	C	T
NC_040256.1	5472404	A	G
NC_040256.1	5508391	T	C
NC_040256.1	5525137	C	G
NC_040256.1	5550684	T	C
NC_040256.1	5595208	A	G
NC_040256.1	5609167	A	G
NC_040256.1	5646699	A	G
NC_040256.1	5675540	T	C
NC_040256.1	5681112	C	G
NC_040256.1	5754187	T	C
NC_040256.1	5850998	G	A
NC_040256.1	5924953	A	G
NC_040256.1	5925150	A	G
NC_040256.1	5940409	T	C
NC_040256.1	5949848	T	C
NC_040256.1	5955166	T	C
NC_040256.1	5956368	T	C
NC_040256.1	5959572	G	T
NC_040256.1	5968679	G	A
NC_040256.1	5969248	T	C
NC_040256.1	5970968	C	T
NC_040256.1	5972817	A	G
NC_040256.1	5977865	G	A

NC_040256.1	5979971	A	G
NC_040256.1	5982111	A	G
NC_040256.1	5982317	G	A
NC_040256.1	5982582	G	A
NC_040256.1	5985593	C	T
NC_040256.1	5990163	A	G
NC_040256.1	5997014	T	C
NC_040256.1	6017261	C	T
NC_040256.1	6017456	A	G
NC_040256.1	6019639	A	G
NC_040256.1	6021950	A	G
NC_040256.1	6022124	T	C
NC_040256.1	6023433	A	G
NC_040256.1	6034850	T	C
NC_040256.1	6039277	G	A
NC_040256.1	6043793	C	T
NC_040256.1	6072434	A	G
NC_040256.1	6078463	T	C
NC_040256.1	6134671	T	C
NC_040256.1	6188915	A	G
NC_040256.1	6241330	C	T
NC_040256.1	6289360	A	C
NC_040256.1	6337910	G	C
NC_040256.1	6409912	A	T
NC_040256.1	6448922	T	C
NC_040256.1	6449558	G	C
NC_040256.1	6532348	T	A
NC_040256.1	6545876	A	G
NC_040256.1	6546542	A	G
NC_040256.1	6600481	A	G
NC_040256.1	6600895	A	G
NC_040256.1	6604846	G	C
NC_040256.1	6620773	A	G
NC_040256.1	6628691	A	G
NC_040256.1	6638474	G	A
NC_040256.1	6644790	G	A
NC_040256.1	6647767	T	G
NC_040256.1	6648025	A	G
NC_040256.1	6691960	C	A
NC_040256.1	6736135	C	A
NC_040256.1	6771062	T	C
NC_040256.1	6812285	A	G
NC_040256.1	6812730	G	A
NC_040256.1	6836172	T	C
NC_040256.1	6836335	A	G
NC_040256.1	6837739	0	C
NC_040256.1	6839038	A	G

NC_040256.1	6891590	G	A
NC_040256.1	6939659	T	G
NC_040256.1	6979835	T	G
NC_040256.1	6980066	G	A
NC_040256.1	7011186	T	C
NC_040256.1	7067952	A	G
NC_040256.1	7127199	A	C
NC_040256.1	7161582	G	T
NC_040256.1	7217130	G	A
NC_040256.1	7333061	A	G
NC_040256.1	7616889	A	G
NC_040256.1	7656228	A	G
NC_040256.1	7828845	C	T
NC_040256.1	7855078	A	G
NC_040256.1	8030338	G	T
NC_040256.1	8050327	T	C
NC_040256.1	8223817	T	C
NC_040256.1	8317807	A	C
NC_040256.1	8443534	G	A
NC_040256.1	8487384	A	C
NC_040256.1	8819604	G	A
NC_040256.1	8842298	C	T
NC_040256.1	8950649	G	A
NC_040256.1	8995394	G	C
NC_040256.1	9093487	A	G
NC_040256.1	9237965	A	G
NC_040256.1	9384635	T	C
NC_040256.1	9406905	A	G
NC_040256.1	9476853	T	C
NC_040256.1	9520411	G	A
NC_040256.1	9570792	A	G
NC_040256.1	9610810	A	G
NC_040256.1	9611120	T	C
NC_040256.1	9611319	A	G
NC_040256.1	9611567	A	T
NC_040256.1	9611764	G	A
NC_040256.1	9632909	A	G
NC_040256.1	9683243	C	T
NC_040256.1	9691487	T	C
NC_040256.1	9738212	T	C
NC_040256.1	9794349	C	G
NC_040256.1	9845872	A	G
NC_040256.1	9870937	T	C
NC_040256.1	9885636	C	G
NC_040256.1	9901356	T	C
NC_040256.1	9901521	G	C
NC_040256.1	9902232	G	T

NC_040256.1	9929629	T	C
NC_040256.1	9949913	T	C
NC_040256.1	9957657	T	C
NC_040256.1	9965895	T	C
NC_040256.1	9966232	A	G
NC_040256.1	9982174	T	C
NC_040256.1	9987487	C	T
NC_040256.1	9990352	C	T
NC_040256.1	10035793	A	G
NC_040256.1	10048359	C	G
NC_040256.1	10059044	C	T
NC_040256.1	10060321	C	G
NC_040256.1	10062820	G	C
NC_040256.1	10101645	G	A
NC_040256.1	10104415	T	C
NC_040256.1	10112305	T	C
NC_040256.1	10118028	C	T
NC_040256.1	10144938	G	A
NC_040256.1	10148642	A	G
NC_040256.1	10149175	A	G
NC_040256.1	10149551	G	A
NC_040256.1	10149719	T	C
NC_040256.1	10149978	A	G
NC_040256.1	10192831	T	C
NC_040256.1	10220644	T	C
NC_040256.1	10222055	C	T
NC_040256.1	10224708	T	C
NC_040256.1	10277511	A	G
NC_040256.1	10339184	T	C
NC_040256.1	10393122	T	C
NC_040256.1	10450029	C	T
NC_040256.1	10514309	G	A
NC_040256.1	10644656	T	C
NC_040256.1	10657431	C	T
NC_040256.1	10688463	A	G
NC_040256.1	10738869	T	C
NC_040256.1	10783284	T	C
NC_040256.1	10822118	A	C
NC_040256.1	10826998	C	T
NC_040256.1	10834594	G	T
NC_040256.1	10834968	T	C
NC_040256.1	10837434	T	C
NC_040256.1	10853794	A	G
NC_040256.1	10866581	A	G
NC_040256.1	10869612	C	T
NC_040256.1	10925041	A	G
NC_040256.1	10997993	A	G

NC_040256.1	10998864	A	G
NC_040256.1	10999659	A	G
NC_040256.1	11001657	G	A
NC_040256.1	11015594	C	T
NC_040256.1	11045815	A	G
NC_040256.1	11046230	T	C
NC_040256.1	11058460	G	C
NC_040256.1	11058710	A	C
NC_040256.1	11062430	A	T
NC_040256.1	11068133	T	C
NC_040256.1	11072066	A	G
NC_040256.1	11073496	A	G
NC_040256.1	11082809	T	C
NC_040256.1	11109482	A	G
NC_040256.1	11122458	A	G
NC_040256.1	11123531	A	T
NC_040256.1	11138686	G	C
NC_040256.1	11164733	C	T
NC_040256.1	11165046	G	A
NC_040256.1	11172961	T	C
NC_040256.1	11181521	A	G
NC_040256.1	11188980	A	G
NC_040256.1	11191870	A	G
NC_040256.1	11197027	T	G
NC_040256.1	11197567	A	G
NC_040256.1	11206021	C	T
NC_040256.1	11208632	C	T
NC_040256.1	11222118	G	C
NC_040256.1	11224893	T	C
NC_040256.1	11270247	G	A
NC_040256.1	11270715	A	G
NC_040256.1	11271669	A	G
NC_040256.1	11320449	C	G
NC_040256.1	11320798	T	C
NC_040256.1	11380724	A	G
NC_040256.1	11457735	A	G
NC_040256.1	11465430	A	G
NC_040256.1	11488716	T	C
NC_040256.1	11489658	A	C
NC_040256.1	11490181	C	A
NC_040256.1	11490876	G	C
NC_040256.1	11491807	T	C
NC_040256.1	11497414	C	T
NC_040256.1	11500094	A	G
NC_040256.1	11543829	C	T
NC_040256.1	11605170	A	G
NC_040256.1	11700010	A	G

NC_040256.1	11802823	A	C
NC_040256.1	11836307	A	G
NC_040256.1	11840668	T	C
NC_040256.1	11842320	G	A
NC_040256.1	11842479	A	G
NC_040256.1	11869241	T	C
NC_040256.1	11915222	T	A
NC_040256.1	11950831	C	T
NC_040256.1	11991657	C	T
NC_040256.1	12200726	G	A
NC_040256.1	12248747	G	A
NC_040256.1	12288810	T	A
NC_040256.1	12367002	T	C
NC_040256.1	12393358	T	C
NC_040256.1	12498984	C	T
NC_040256.1	12514558	C	T
NC_040256.1	12567432	C	T
NC_040256.1	12597874	A	G
NC_040256.1	12648173	T	C
NC_040256.1	12702583	G	A
NC_040256.1	12723650	T	C
NC_040256.1	12723821	A	G
NC_040256.1	12774803	G	A
NC_040256.1	12824468	T	G
NC_040256.1	12830240	C	G
NC_040256.1	12861767	T	C
NC_040256.1	12867993	C	T
NC_040256.1	12889454	T	C
NC_040256.1	12897166	T	C
NC_040256.1	12898794	C	T
NC_040256.1	12927681	G	A
NC_040256.1	12997962	A	G
NC_040256.1	12999907	C	T
NC_040256.1	13001529	A	G
NC_040256.1	13001889	T	C
NC_040256.1	13002268	C	T
NC_040256.1	13019784	A	G
NC_040256.1	13020855	A	G
NC_040256.1	13026068	C	T
NC_040256.1	13034877	A	G
NC_040256.1	13082890	A	G
NC_040256.1	13088184	G	A
NC_040256.1	13103450	T	C
NC_040256.1	13110155	T	C
NC_040256.1	13149575	T	C
NC_040256.1	13155559	T	G
NC_040256.1	13157696	G	A

NC_040256.1	13161536	A	G
NC_040256.1	13165650	C	G
NC_040256.1	13167370	A	G
NC_040256.1	13171468	G	A
NC_040256.1	13171671	A	C
NC_040256.1	13175093	T	C
NC_040256.1	13179447	A	G
NC_040256.1	13179604	C	A
NC_040256.1	13180378	C	T
NC_040256.1	13183247	G	A
NC_040256.1	13192984	T	C
NC_040256.1	13238007	A	T
NC_040256.1	13257654	A	G
NC_040256.1	13260236	C	T
NC_040256.1	13313812	A	G
NC_040256.1	13319431	T	C
NC_040256.1	13338184	A	G
NC_040256.1	13350996	A	G
NC_040256.1	13352513	A	G
NC_040256.1	13384361	T	C
NC_040256.1	13389101	A	G
NC_040256.1	13395918	T	C
NC_040256.1	13400996	C	T
NC_040256.1	13402255	T	C
NC_040256.1	13426345	C	G
NC_040256.1	13475106	C	T
NC_040256.1	13482717	T	A
NC_040256.1	13503823	T	C
NC_040256.1	13545836	C	G
NC_040256.1	13582235	T	C
NC_040256.1	13610169	T	C
NC_040256.1	13610702	T	C
NC_040256.1	13612320	C	G
NC_040256.1	13622484	A	G
NC_040256.1	13624037	A	G
NC_040256.1	13632761	T	A
NC_040256.1	13665651	T	C
NC_040256.1	13675582	G	C
NC_040256.1	13676050	A	G
NC_040256.1	13677099	A	G
NC_040256.1	13680344	A	G
NC_040256.1	13681402	T	A
NC_040256.1	13749709	A	G
NC_040256.1	13753185	A	G
NC_040256.1	13805085	G	T
NC_040256.1	13879196	A	C
NC_040256.1	13880744	G	A

NC_040256.1	13891244	G	A
NC_040256.1	13927920	G	A
NC_040256.1	13928088	G	A
NC_040256.1	13935078	A	G
NC_040256.1	13939268	A	G
NC_040256.1	13941553	A	G
NC_040256.1	13954179	G	A
NC_040256.1	13958115	A	G
NC_040256.1	13960828	T	A
NC_040256.1	14019065	T	C
NC_040256.1	14032193	G	T
NC_040256.1	14047109	C	T
NC_040256.1	14048522	T	C
NC_040256.1	14064910	G	A
NC_040256.1	14069900	T	C
NC_040256.1	14073786	A	C
NC_040256.1	14077673	C	T
NC_040256.1	14078196	T	C
NC_040256.1	14078416	T	C
NC_040256.1	14085700	A	C
NC_040256.1	14085894	C	T
NC_040256.1	14090947	C	G
NC_040256.1	14108981	A	C
NC_040256.1	14109698	A	C
NC_040256.1	14110222	C	T
NC_040256.1	14115915	T	C
NC_040256.1	14119296	T	C
NC_040256.1	14121463	C	T
NC_040256.1	14122890	T	C
NC_040256.1	14135542	T	C
NC_040256.1	14138582	T	C
NC_040256.1	14138797	T	G
NC_040256.1	14142684	G	A
NC_040256.1	14160383	C	G
NC_040256.1	14171935	G	T
NC_040256.1	14172571	C	T
NC_040256.1	14197470	T	C
NC_040256.1	14207839	G	A
NC_040256.1	14208369	C	T
NC_040256.1	14226111	G	C
NC_040256.1	14287669	T	C
NC_040256.1	14318078	T	G
NC_040256.1	14318403	A	G
NC_040256.1	14370313	T	C
NC_040256.1	14372138	C	T
NC_040256.1	14372355	T	C
NC_040256.1	14372512	A	G

NC_040256.1	14373283	T	C
NC_040256.1	14373973	A	C
NC_040256.1	14374303	T	C
NC_040256.1	14400353	A	G
NC_040256.1	14408903	T	G
NC_040256.1	14427444	A	G
NC_040256.1	14496216	A	G
NC_040256.1	14496537	A	G
NC_040256.1	14517903	C	A
NC_040256.1	14583196	C	A
NC_040256.1	14593830	A	G
NC_040256.1	14651341	T	C
NC_040256.1	14661418	A	G
NC_040256.1	14661592	G	A
NC_040256.1	14673034	A	G
NC_040256.1	14701552	T	C
NC_040256.1	14702767	G	A
NC_040256.1	14707782	A	C
NC_040256.1	14727709	T	C
NC_040256.1	14738446	G	A
NC_040256.1	14771367	A	G
NC_040256.1	14773256	T	C
NC_040256.1	14775452	G	A
NC_040256.1	14786029	G	C
NC_040256.1	14799008	G	A
NC_040256.1	14801332	T	C
NC_040256.1	14803688	A	G
NC_040256.1	14837427	T	G
NC_040256.1	14899634	G	A
NC_040256.1	14941607	A	G
NC_040256.1	14949595	T	C
NC_040256.1	14952243	G	C
NC_040256.1	14952429	T	C
NC_040256.1	14958936	C	T
NC_040256.1	14961058	T	C
NC_040256.1	14961674	A	C
NC_040256.1	14969060	G	A
NC_040256.1	14969451	T	A
NC_040256.1	14970713	T	C
NC_040256.1	14971151	A	G
NC_040256.1	14972524	A	G
NC_040256.1	14972717	C	T
NC_040256.1	14972978	T	C
NC_040256.1	14973351	A	G
NC_040256.1	14982863	C	T
NC_040256.1	14983226	G	A
NC_040256.1	14983722	A	G

NC_040256.1	14984378	G	A
NC_040256.1	14987337	C	A
NC_040256.1	14988546	A	C
NC_040256.1	14988806	T	A
NC_040256.1	15025168	A	G
NC_040256.1	15026542	A	G
NC_040256.1	15088497	G	C
NC_040256.1	15090617	T	G
NC_040256.1	15090839	G	A
NC_040256.1	15094590	C	T
NC_040256.1	15104594	T	G
NC_040256.1	15117023	A	C
NC_040256.1	15117486	G	A
NC_040256.1	15123699	T	C
NC_040256.1	15160442	T	C
NC_040256.1	15212196	T	C
NC_040256.1	15267421	T	C
NC_040256.1	15267627	C	T
NC_040256.1	15267897	G	T
NC_040256.1	15293443	C	A
NC_040256.1	15348686	T	C
NC_040256.1	15350537	A	G
NC_040256.1	15373938	A	G
NC_040256.1	15374128	A	G
NC_040256.1	15374279	A	G
NC_040256.1	15421189	G	C
NC_040256.1	15422017	A	G
NC_040256.1	15429242	T	G
NC_040256.1	15429568	C	T
NC_040256.1	15433601	G	A
NC_040256.1	15436169	T	C
NC_040256.1	15436457	T	C
NC_040256.1	15437134	C	T
NC_040256.1	15441591	G	C
NC_040256.1	15445282	A	G
NC_040256.1	15453604	G	C
NC_040256.1	15461896	A	G
NC_040256.1	15463805	C	G
NC_040256.1	15467715	C	G
NC_040256.1	15468441	A	G
NC_040256.1	15483885	A	G
NC_040256.1	15536965	T	C
NC_040256.1	15586312	T	C
NC_040256.1	15586471	C	G
NC_040256.1	15586769	G	T
NC_040256.1	15623954	C	T
NC_040256.1	15677383	C	T

NC_040256.1	15812226	T	G
NC_040256.1	15842335	A	G
NC_040256.1	15909301	C	T
NC_040256.1	15956536	A	T
NC_040256.1	15958305	T	C
NC_040256.1	15959457	T	C
NC_040256.1	15960274	A	G
NC_040256.1	15964022	A	G
NC_040256.1	15969013	T	G
NC_040256.1	15979447	T	C
NC_040256.1	16178579	C	A
NC_040256.1	16186775	C	T
NC_040256.1	16244037	T	C
NC_040256.1	16258813	A	G
NC_040256.1	16272903	G	A
NC_040256.1	16405350	C	G
NC_040256.1	16433392	A	C
NC_040256.1	16492880	C	T
NC_040256.1	16531923	A	G
NC_040256.1	16535773	T	A
NC_040256.1	16538693	T	C
NC_040256.1	16542418	T	C
NC_040256.1	16553766	T	C
NC_040256.1	16564804	C	T
NC_040256.1	16576166	A	G
NC_040256.1	16576316	G	C
NC_040256.1	16577513	C	T
NC_040256.1	16579938	C	G
NC_040256.1	16593147	T	A
NC_040256.1	16604048	A	G
NC_040256.1	16613877	A	G
NC_040256.1	16658844	G	A
NC_040256.1	16726155	A	G
NC_040256.1	16736121	A	G
NC_040256.1	16736688	T	C
NC_040256.1	16758908	T	C
NC_040256.1	16787422	T	C
NC_040256.1	16838390	G	T
NC_040256.1	16873728	G	A
NC_040256.1	16905009	A	G
NC_040256.1	16911734	A	G
NC_040256.1	16912090	G	C
NC_040256.1	16969869	T	C
NC_040256.1	16974482	T	C
NC_040256.1	16979599	T	G
NC_040256.1	16980280	G	A
NC_040256.1	16981834	G	A

NC_040256.1	16982379	A	G
NC_040256.1	16983158	G	T
NC_040256.1	16983364	A	G
NC_040256.1	17025547	C	T
NC_040256.1	17025971	G	T
NC_040256.1	17028633	T	C
NC_040256.1	17031979	A	G
NC_040256.1	17036597	T	C
NC_040256.1	17050173	A	C
NC_040256.1	17052839	C	T
NC_040256.1	17054334	T	C
NC_040256.1	17055491	A	G
NC_040256.1	17055650	G	T
NC_040256.1	17057999	T	C
NC_040256.1	17058177	G	A
NC_040256.1	17084266	A	G
NC_040256.1	17098696	C	G
NC_040256.1	17111596	A	G
NC_040256.1	17114473	A	G
NC_040256.1	17120175	C	G
NC_040256.1	17122965	C	G
NC_040256.1	17125234	A	G
NC_040256.1	17134129	G	C
NC_040256.1	17135314	G	C
NC_040256.1	17145821	T	C
NC_040256.1	17163504	T	C
NC_040256.1	17197364	A	C
NC_040256.1	17254243	T	G
NC_040256.1	17310458	G	A
NC_040256.1	17367457	C	T
NC_040256.1	17479795	T	C
NC_040256.1	17497490	C	T
NC_040256.1	17512683	T	C
NC_040256.1	17512965	C	T
NC_040256.1	17514430	T	C
NC_040256.1	17524713	T	C
NC_040256.1	17525672	A	G
NC_040256.1	17528999	A	G
NC_040256.1	17530288	T	C
NC_040256.1	17585402	T	C
NC_040256.1	17593882	A	G
NC_040256.1	17649958	A	G
NC_040256.1	17708654	A	G
NC_040256.1	17764353	G	A
NC_040256.1	17824444	A	G
NC_040256.1	17825045	T	C
NC_040256.1	17841029	T	C

NC_040256.1	17841337	A	G
NC_040256.1	17841771	C	G
NC_040256.1	17842846	A	G
NC_040256.1	17843110	A	G
NC_040256.1	17843495	G	T
NC_040256.1	17844326	T	C
NC_040256.1	17894255	A	G
NC_040256.1	17951945	T	C
NC_040256.1	17968978	T	A
NC_040256.1	17971622	T	C
NC_040256.1	18016892	C	T
NC_040256.1	18060035	T	C
NC_040256.1	18060437	A	G
NC_040256.1	18060588	C	G
NC_040256.1	18063093	G	A
NC_040256.1	18063706	C	G
NC_040256.1	18078391	A	G
NC_040256.1	18086035	G	A
NC_040256.1	18091422	T	C
NC_040256.1	18091899	G	C
NC_040256.1	18092204	C	T
NC_040256.1	18095381	A	G
NC_040256.1	18102360	A	G
NC_040256.1	18102531	A	G
NC_040256.1	18109058	A	G
NC_040256.1	18113308	T	C
NC_040256.1	18149191	A	G
NC_040256.1	18156789	G	A
NC_040256.1	18165846	A	G
NC_040256.1	18186008	G	A
NC_040256.1	18213396	G	A
NC_040256.1	18215090	A	G
NC_040256.1	18221444	A	G
NC_040256.1	18228321	C	T
NC_040256.1	18237458	T	C
NC_040256.1	18240495	A	G
NC_040256.1	18252349	A	G
NC_040256.1	18256844	G	A
NC_040256.1	18261327	T	C
NC_040256.1	18263971	G	C
NC_040256.1	18277425	C	T
NC_040256.1	18277668	A	G
NC_040256.1	18293664	T	C
NC_040256.1	18299557	G	A
NC_040256.1	18305436	A	T
NC_040256.1	18306265	C	T
NC_040256.1	18307139	G	A

NC_040256.1	18311482	T	A
NC_040256.1	18316613	G	C
NC_040256.1	18316980	A	C
NC_040256.1	18338143	A	G
NC_040256.1	18338932	C	G
NC_040256.1	18339180	A	T
NC_040256.1	18348998	G	C
NC_040256.1	18379902	G	C
NC_040256.1	18401361	T	C
NC_040256.1	18401528	G	A
NC_040256.1	18401738	A	C
NC_040256.1	18436662	C	G
NC_040256.1	18445104	T	G
NC_040256.1	18447157	T	C
NC_040256.1	18450996	C	T
NC_040256.1	18468601	T	C
NC_040256.1	18510923	C	T
NC_040256.1	18522076	C	T
NC_040256.1	18537459	A	G
NC_040256.1	18547841	T	C
NC_040256.1	18549098	T	C
NC_040256.1	18550020	C	G
NC_040256.1	18550388	T	C
NC_040256.1	18563720	A	G
NC_040256.1	18633558	G	A
NC_040256.1	18646281	T	C
NC_040256.1	18647287	T	C
NC_040256.1	18673377	C	T
NC_040256.1	18685639	T	C
NC_040256.1	18686647	T	C
NC_040256.1	18689984	T	C
NC_040256.1	18695753	A	C
NC_040256.1	18738186	C	T
NC_040256.1	18768631	T	C
NC_040256.1	18769935	G	A
NC_040256.1	18772148	C	T
NC_040256.1	18772832	A	G
NC_040256.1	18773221	A	G
NC_040256.1	18778855	T	C
NC_040256.1	18779088	T	C
NC_040256.1	18779238	T	C
NC_040256.1	18787219	T	C
NC_040256.1	18787481	T	C
NC_040256.1	18797086	A	G
NC_040256.1	18803216	G	A
NC_040256.1	18803674	C	T
NC_040256.1	18804012	T	A

NC_040256.1	18804260	C	G
NC_040256.1	18805283	A	G
NC_040256.1	18805824	A	G
NC_040256.1	18806555	C	T
NC_040256.1	18806774	G	A
NC_040256.1	18809296	A	G
NC_040256.1	18813366	A	G
NC_040256.1	18813536	A	G
NC_040256.1	18818090	A	C
NC_040256.1	18836351	C	T
NC_040256.1	18839972	A	G
NC_040256.1	18840269	C	G
NC_040256.1	18841258	A	G
NC_040256.1	18844106	C	T
NC_040256.1	18844338	T	C
NC_040256.1	18844685	T	C
NC_040256.1	18845199	G	A
NC_040256.1	18850583	A	G
NC_040256.1	18893761	T	C
NC_040256.1	18895102	T	C
NC_040256.1	18903935	A	G
NC_040256.1	18904888	T	C
NC_040256.1	18906282	T	C
NC_040256.1	18966431	T	C
NC_040256.1	19029533	A	G
NC_040256.1	19030475	A	G
NC_040256.1	19048696	T	C
NC_040256.1	19111173	A	C
NC_040256.1	19112892	A	G
NC_040256.1	19113068	G	C
NC_040256.1	19113273	A	G
NC_040256.1	19124183	A	G
NC_040256.1	19124341	T	C
NC_040256.1	19143307	G	A
NC_040256.1	19151999	T	C
NC_040256.1	19152437	G	T
NC_040256.1	19152924	A	G
NC_040256.1	19201830	G	A
NC_040256.1	19228450	G	C
NC_040256.1	19236633	T	G
NC_040256.1	19237857	A	G
NC_040256.1	19241434	T	C
NC_040256.1	19311852	T	C
NC_040256.1	19312945	A	G
NC_040256.1	19338388	T	C
NC_040256.1	19355617	A	G
NC_040256.1	19368802	T	C

NC_040256.1	19423580	T	C
NC_040256.1	19424287	T	C
NC_040256.1	19425816	A	G
NC_040256.1	19426808	A	G
NC_040256.1	19434696	T	C
NC_040256.1	19434958	T	C
NC_040256.1	19435192	A	G
NC_040256.1	19435610	G	A
NC_040256.1	19436207	A	G
NC_040256.1	19436438	T	C
NC_040256.1	19437510	A	G
NC_040256.1	19494494	T	C
NC_040256.1	19540490	T	C
NC_040256.1	19577085	T	C
NC_040256.1	19578126	C	T
NC_040256.1	19600828	A	C
NC_040256.1	19618204	T	C
NC_040256.1	19636398	C	T
NC_040256.1	19636625	A	G
NC_040256.1	19637825	C	T
NC_040256.1	19638100	A	C
NC_040256.1	19639626	T	C
NC_040256.1	19640007	T	C
NC_040256.1	19652582	A	G
NC_040256.1	19657449	A	G
NC_040256.1	19709541	T	C
NC_040256.1	19717789	A	G
NC_040256.1	19728630	A	G
NC_040256.1	19751852	T	G
NC_040256.1	19752240	C	T
NC_040256.1	19767198	T	C
NC_040256.1	19768834	A	G
NC_040256.1	19769097	A	G
NC_040256.1	19769264	A	G
NC_040256.1	19821062	A	G
NC_040256.1	19861122	A	G
NC_040256.1	19861818	T	C
NC_040256.1	19862109	T	C
NC_040256.1	19862536	T	C
NC_040256.1	19865518	T	C
NC_040256.1	19867406	T	C
NC_040256.1	19867939	A	G
NC_040256.1	19870427	T	C
NC_040256.1	19873495	T	G
NC_040256.1	19882501	G	C
NC_040256.1	19885519	G	A
NC_040256.1	19886912	T	C

NC_040256.1	19887875	C	T
NC_040256.1	19916121	C	T
NC_040256.1	19917907	G	C
NC_040256.1	19920540	T	C
NC_040256.1	19928290	A	G
NC_040256.1	19930578	T	C
NC_040256.1	19952297	C	T
NC_040256.1	19952454	C	T
NC_040256.1	19980369	G	T
NC_040256.1	20057138	A	G
NC_040256.1	20106348	G	T
NC_040256.1	20171703	G	A
NC_040256.1	20184327	G	A
NC_040256.1	20188705	T	C
NC_040256.1	20191219	G	A
NC_040256.1	20191915	G	A
NC_040256.1	20206976	T	C
NC_040256.1	20226644	G	C
NC_040256.1	20271372	A	G
NC_040256.1	20274307	C	T
NC_040256.1	20325529	T	C
NC_040256.1	20383477	A	G
NC_040256.1	20401578	A	G
NC_040256.1	20401838	C	G
NC_040256.1	20455226	C	G
NC_040256.1	20455382	A	G
NC_040256.1	20504986	C	T
NC_040256.1	20549713	G	T
NC_040256.1	20550006	T	C
NC_040256.1	20552195	T	C
NC_040256.1	20607528	C	T
NC_040256.1	20660687	T	C
NC_040256.1	20697103	C	G
NC_040256.1	20723519	T	C
NC_040256.1	20729462	G	A
NC_040256.1	20786889	C	T
NC_040256.1	20837190	T	C
NC_040256.1	20881791	T	G
NC_040256.1	20918551	A	G
NC_040256.1	20934955	C	A
NC_040256.1	21004604	A	G
NC_040256.1	21016059	G	A
NC_040256.1	21036961	C	T
NC_040256.1	21055846	G	C
NC_040256.1	21056714	G	A
NC_040256.1	21058380	G	A
NC_040256.1	21111845	C	T

NC_040256.1	21212952	T	C
NC_040256.1	21270828	G	T
NC_040256.1	21323455	G	A
NC_040256.1	21324105	A	G
NC_040256.1	21370417	A	G
NC_040256.1	21586599	T	G
NC_040256.1	21617834	C	G
NC_040256.1	21618736	A	G
NC_040256.1	21618976	G	C
NC_040256.1	21665699	A	G
NC_040256.1	21692673	A	T
NC_040256.1	21715337	A	G
NC_040256.1	21727295	C	T
NC_040256.1	21731043	C	T
NC_040256.1	21731209	T	C
NC_040256.1	21731854	G	C
NC_040256.1	21732882	C	A
NC_040256.1	21733542	A	G
NC_040256.1	21734064	G	A
NC_040256.1	21753560	C	A
NC_040256.1	21753861	A	G
NC_040256.1	21754134	A	G
NC_040256.1	21754366	G	C
NC_040256.1	21806365	T	A
NC_040256.1	21859405	G	A
NC_040256.1	21919182	C	T
NC_040256.1	21919391	C	T
NC_040256.1	21921419	C	A
NC_040256.1	21922009	T	C
NC_040256.1	21973548	G	A
NC_040256.1	22035620	C	G
NC_040256.1	22101611	G	A
NC_040256.1	22172952	T	C
NC_040256.1	22224352	A	G
NC_040256.1	22265585	C	T
NC_040256.1	22319751	C	T
NC_040256.1	22375655	C	T
NC_040256.1	22425687	G	A
NC_040256.1	22468821	G	A
NC_040256.1	22502647	C	T
NC_040256.1	22556131	C	T
NC_040256.1	22608323	A	G
NC_040256.1	22650131	T	C
NC_040256.1	22651941	A	G
NC_040256.1	22706259	T	C
NC_040256.1	22763515	A	G
NC_040256.1	22814697	G	T

NC_040256.1	22871048	T	G
NC_040256.1	22939671	A	G
NC_040256.1	22988250	T	G
NC_040256.1	23042205	T	C
NC_040256.1	23090115	T	A
NC_040256.1	23141652	G	A
NC_040256.1	23200393	T	C
NC_040256.1	23242302	G	C
NC_040256.1	23283699	G	A
NC_040256.1	23354287	A	G
NC_040256.1	23410692	T	C
NC_040256.1	23471824	T	C
NC_040256.1	23526525	T	C
NC_040256.1	23584274	G	A
NC_040256.1	23629425	T	C
NC_040256.1	23672540	0	0
NC_040256.1	23729576	G	T
NC_040256.1	23790653	C	A
NC_040256.1	23848445	A	G
NC_040256.1	23904698	T	C
NC_040256.1	23959504	T	C
NC_040256.1	24013564	C	G
NC_040256.1	24067643	A	G
NC_040256.1	24121694	C	T
NC_040256.1	24159591	A	G
NC_040256.1	24159782	A	G
NC_040256.1	24160250	A	G
NC_040256.1	24221041	A	G
NC_040256.1	24275137	A	G
NC_040256.1	24329681	G	A
NC_040256.1	24360435	T	C
NC_040256.1	24366798	A	G
NC_040256.1	24367910	T	G
NC_040256.1	24388310	G	A
NC_040256.1	24439598	T	C
NC_040256.1	24441180	A	G
NC_040256.1	24452652	T	C
NC_040256.1	24474154	A	G
NC_040256.1	24485877	A	G
NC_040256.1	24537973	C	G
NC_040256.1	24594196	G	T
NC_040256.1	24640883	G	A
NC_040256.1	24682923	C	G
NC_040256.1	24736462	T	C
NC_040256.1	24791670	A	G
NC_040256.1	24849203	A	G
NC_040256.1	24902771	C	T

NC_040256.1	24938003	G	A
NC_040256.1	24946743	T	C
NC_040256.1	24947136	G	A
NC_040256.1	25014921	A	T
NC_040256.1	25082186	G	A
NC_040256.1	25130795	A	G
NC_040256.1	25178912	C	T
NC_040256.1	25232255	A	G
NC_040256.1	25276094	A	G
NC_040256.1	25334035	C	G
NC_040256.1	25389897	T	C
NC_040256.1	25444963	T	C
NC_040256.1	25500977	T	C
NC_040256.1	25560996	T	G
NC_040256.1	25616004	T	C
NC_040256.1	25669927	C	T
NC_040256.1	25695666	A	G
NC_040256.1	25734164	C	T
NC_040256.1	25766844	A	G
NC_040256.1	25829849	A	C
NC_040256.1	25906635	T	C
NC_040256.1	25956189	C	G
NC_040256.1	25956379	A	G
NC_040256.1	25997293	T	C
NC_040256.1	25997502	T	A
NC_040256.1	25997931	T	C
NC_040256.1	26015960	T	G
NC_040256.1	26068268	G	A
NC_040256.1	26125432	T	G
NC_040256.1	26187290	G	T
NC_040256.1	26380423	G	C
NC_040256.1	26492997	T	A
NC_040256.1	26548965	T	C
NC_040256.1	26594664	G	A
NC_040256.1	26638148	A	G
NC_040256.1	26723664	T	A
NC_040256.1	26773478	C	T
NC_040256.1	26833255	T	C
NC_040256.1	26884459	T	G
NC_040256.1	26956444	T	C
NC_040256.1	27107068	A	G
NC_040256.1	27237193	C	T
NC_040256.1	27291488	A	G
NC_040256.1	27342554	A	G
NC_040256.1	27392932	G	A
NC_040256.1	27453583	T	A
NC_040256.1	27519487	G	A

NC_040256.1	27575322	G	A
NC_040256.1	27687601	T	C
NC_040256.1	27739390	A	C
NC_040256.1	27777530	T	G
NC_040256.1	27868480	A	G
NC_040256.1	27922391	T	C
NC_040256.1	27975604	A	G
NC_040256.1	28017310	T	C
NC_040256.1	28018215	G	C
NC_040256.1	28074564	C	G
NC_040256.1	28115149	T	C
NC_040256.1	28115308	A	G
NC_040256.1	28115947	C	T
NC_040256.1	28119037	T	C
NC_040256.1	28173524	A	G
NC_040256.1	28229243	T	C
NC_040256.1	28282193	A	G
NC_040256.1	28340197	G	A
NC_040256.1	28397121	C	A
NC_040256.1	28451020	G	A
NC_040256.1	28508133	G	A
NC_040256.1	28559801	A	T
NC_040256.1	28618970	C	G
NC_040256.1	28672288	C	T
NC_040256.1	28730106	T	A
NC_040256.1	28783125	A	G
NC_040256.1	28851059	G	A
NC_040256.1	28901646	A	G
NC_040256.1	28953975	C	G
NC_040256.1	29010132	G	A
NC_040256.1	29084095	C	T
NC_040256.1	29407806	G	A
NC_040256.1	29411915	T	G
NC_040256.1	29413076	G	A
NC_040256.1	29466869	T	G
NC_040256.1	29498467	A	G
NC_040256.1	29551844	T	C
NC_040256.1	29607673	A	G
NC_040256.1	29658658	A	G
NC_040256.1	29698355	C	T
NC_040256.1	29745937	C	T
NC_040256.1	29802340	T	C
NC_040256.1	29858204	C	A
NC_040256.1	29894195	G	A
NC_040256.1	29896660	T	C
NC_040256.1	29897544	G	A
NC_040256.1	29897756	A	G

NC_040256.1	29898198	T	C
NC_040256.1	29952631	A	G
NC_040256.1	30007844	G	C
NC_040256.1	30048925	T	C
NC_040256.1	30091088	A	G
NC_040256.1	30147707	T	C
NC_040256.1	30198045	G	T
NC_040256.1	30245237	A	G
NC_040256.1	30278914	T	C
NC_040256.1	30279281	0	G
NC_040256.1	30279466	G	A
NC_040256.1	30279822	T	C
NC_040256.1	30333973	G	C
NC_040256.1	30390177	A	G
NC_040256.1	30445843	C	T
NC_040256.1	30492909	G	C
NC_040256.1	30557804	T	C
NC_040256.1	30622282	T	C
NC_040256.1	30682434	G	C
NC_040256.1	30715049	A	G
NC_040256.1	30761783	G	A
NC_040256.1	30832710	G	C
NC_040256.1	30884232	A	G
NC_040256.1	30937238	C	T
NC_040256.1	30938131	T	C
NC_040256.1	30941389	G	A
NC_040256.1	30966476	T	C
NC_040256.1	31023051	A	G
NC_040256.1	31057480	G	C
NC_040256.1	31057829	A	C
NC_040256.1	31070802	T	C
NC_040256.1	31071623	C	T
NC_040256.1	31072558	G	T
NC_040256.1	31120121	C	G
NC_040256.1	31155691	A	G
NC_040256.1	31212912	C	G
NC_040256.1	31263905	T	G
NC_040256.1	31402985	A	G
NC_040256.1	31430452	G	C
NC_040256.1	31510445	C	G
NC_040256.1	31540174	A	G
NC_040256.1	31618440	C	T
NC_040256.1	31734941	G	A
NC_040256.1	31851017	A	G
NC_040256.1	32078576	A	G
NC_040256.1	32653773	G	A
NC_040256.1	32712559	A	G

NC_040256.1	32779020	T	C
NC_040256.1	32857429	G	A
NC_040256.1	32908671	C	T
NC_040256.1	32983754	C	T
NC_040256.1	33015804	C	T
NC_040256.1	33097378	A	G
NC_040256.1	33141152	T	C
NC_040256.1	33205190	G	A
NC_040256.1	33264838	C	T
NC_040256.1	33315235	G	T
NC_040256.1	33364506	C	A
NC_040256.1	33443203	C	G
NC_040256.1	33553230	C	T
NC_040256.1	33768596	G	A
NC_040256.1	33824189	A	C
NC_040256.1	33899948	G	A
NC_040256.1	33949967	C	T
NC_040256.1	33999442	A	G
NC_040256.1	34051392	T	C
NC_040256.1	34087120	C	T
NC_040256.1	34145278	T	C
NC_040256.1	34193970	A	G
NC_040256.1	34254601	A	G
NC_040256.1	34299970	A	G
NC_040256.1	34351254	T	C
NC_040256.1	34394591	T	C
NC_040256.1	34396478	T	C
NC_040256.1	34414260	G	T
NC_040256.1	34534530	T	C
NC_040256.1	34575258	A	G
NC_040256.1	34626002	G	A
NC_040256.1	34689440	T	C
NC_040256.1	34757559	T	G
NC_040256.1	34804024	C	T
NC_040256.1	34861229	A	T
NC_040256.1	34919662	T	C
NC_040256.1	35114756	A	G
NC_040256.1	35191944	C	G
NC_040256.1	35247475	A	G
NC_040256.1	35305494	G	A
NC_040256.1	35375795	G	A
NC_040256.1	35571520	C	T
NC_040256.1	35612635	A	G
NC_040256.1	35674782	G	A
NC_040256.1	35788507	A	G
NC_040256.1	35815042	C	T
NC_040256.1	35866936	G	A

NC_040256.1	35942508	A	C
NC_040256.1	35994214	A	G
NC_040256.1	36040014	C	G
NC_040256.1	36104343	T	A
NC_040256.1	36174943	C	T
NC_040256.1	36239737	G	A
NC_040256.1	36535339	C	T
NC_040256.1	36734847	C	T
NC_040256.1	36811353	T	C
NC_040256.1	36874514	T	C
NC_040256.1	36930501	G	A
NC_040256.1	36969030	A	G
NC_040256.1	37082888	T	C
NC_040256.1	37122317	A	G
NC_040256.1	37202442	A	G
NC_040256.1	37259040	A	G
NC_040256.1	37321405	T	C
NC_040256.1	37371706	G	A
NC_040256.1	37424203	T	C
NC_040256.1	37481986	A	G
NC_040256.1	37546968	C	G
NC_040256.1	37603306	A	G
NC_040256.1	37659161	A	G
NC_040256.1	37725846	A	G
NC_040256.1	37776513	C	G
NC_040256.1	37840916	T	C
NC_040256.1	37894516	A	G
NC_040256.1	37971999	G	C
NC_040256.1	38027689	T	C
NC_040256.1	38084882	T	A
NC_040256.1	38135676	C	T
NC_040256.1	38182805	G	C
NC_040256.1	38232886	C	T
NC_040256.1	38247743	A	G
NC_040256.1	38249376	T	C
NC_040256.1	38261310	C	T
NC_040256.1	38266641	T	C
NC_040256.1	38268351	T	C
NC_040256.1	38306051	G	A
NC_040256.1	38358081	C	T
NC_040256.1	38387819	A	G
NC_040256.1	38451780	T	C
NC_040256.1	38506521	A	G
NC_040256.1	38565977	A	G
NC_040256.1	38620700	T	G
NC_040256.1	38671147	G	A
NC_040256.1	38706133	G	T

NC_040256.1	38798111	A	G
NC_040256.1	38854894	C	G
NC_040256.1	38909627	C	T
NC_040256.1	38944296	A	G
NC_040256.1	38995126	A	G
NC_040256.1	39000037	A	G
NC_040256.1	39012554	C	A
NC_040256.1	39016330	G	C
NC_040256.1	39065546	G	A
NC_040256.1	39120130	A	G
NC_040256.1	39133054	A	G
NC_040256.1	39187274	T	C
NC_040256.1	39197626	A	G
NC_040256.1	39200649	C	G
NC_040256.1	39202672	T	G
NC_040256.1	39211600	A	G
NC_040256.1	39224197	G	A
NC_040256.1	39291402	A	G
NC_040256.1	39295892	A	G
NC_040256.1	39372543	A	G
NC_040256.1	39428491	T	C
NC_040256.1	39468158	T	C
NC_040256.1	39472985	G	T
NC_040256.1	39521985	T	A
NC_040256.1	39555630	A	G
NC_040256.1	39563783	T	C
NC_040256.1	39622460	G	A
NC_040256.1	39671732	A	G
NC_040256.1	39726589	C	G
NC_040256.1	39777060	C	G
NC_040256.1	39812639	T	C
NC_040256.1	39869797	C	A
NC_040256.1	39921698	G	A
NC_040256.1	39969767	T	G
NC_040256.1	39982917	T	C
NC_040256.1	40031972	C	T
NC_040256.1	40071641	T	C
NC_040256.1	40217718	T	G
NC_040256.1	40292645	A	G
NC_040256.1	40545539	A	G
NC_040256.1	40704335	T	G
NC_040256.1	40761646	G	A
NC_040256.1	40791996	T	C
NC_040256.1	40860145	A	G
NC_040256.1	40905877	T	C
NC_040256.1	40954352	A	G
NC_040256.1	40991044	C	T

NC_040256.1	41060402	G	C
NC_040256.1	41114994	C	T
NC_040256.1	41155490	T	C
NC_040256.1	41229033	A	G
NC_040256.1	41260217	T	C
NC_040256.1	41519606	A	T
NC_040256.1	41606012	C	A
NC_040256.1	41758796	C	G
NC_040256.1	41835078	A	T
NC_040256.1	41916497	T	A
NC_040256.1	42008051	T	G
NC_040256.1	42586222	G	A
NC_040256.1	43491031	C	T
NC_040256.1	43559672	A	G
NC_040256.1	43643451	G	C
NC_040256.1	43912286	C	G
NC_040256.1	44144707	G	A
NC_040256.1	44330771	A	T
NC_040256.1	44498085	A	G
NC_040256.1	44771039	A	C
NC_040256.1	44899304	T	C
NC_040256.1	45141666	A	G
NC_040256.1	45282632	C	T
NC_040256.1	45334795	C	T
NC_040256.1	45399732	A	G
NC_040256.1	45439922	T	C
NC_040256.1	45496253	A	G
NC_040256.1	45618440	G	T
NC_040256.1	45678249	T	G
NC_040256.1	45728661	A	G
NC_040256.1	45785177	A	G
NC_040256.1	45845173	G	A
NC_040256.1	45945266	A	T
NC_040256.1	46004459	G	A
NC_040256.1	46056803	G	T
NC_040256.1	46181309	G	A
NC_040256.1	46249487	A	G
NC_040256.1	46320030	A	C
NC_040256.1	46568322	C	T
NC_040256.1	46620163	A	T
NC_040256.1	46672323	G	C
NC_040256.1	46744006	A	G
NC_040256.1	46974742	T	C
NC_040256.1	47542268	C	G
NC_040256.1	47627194	A	T
NC_040256.1	47779718	T	C
NC_040256.1	47850995	A	C

NC_040256.1	47967079	A	G
NC_040256.1	48314096	A	C
NC_040256.1	48375998	A	G
NC_040256.1	48452428	A	G
NC_040256.1	48569531	A	G
NC_040256.1	48630517	T	C
NC_040256.1	48722531	T	C
NC_040256.1	48770623	T	C
NC_040256.1	48965250	T	C
NC_040256.1	49019013	T	C
NC_040256.1	49439466	G	A
NC_040256.1	49504415	A	G
NC_040256.1	49695894	C	T
NC_040256.1	49767692	T	C
NC_040256.1	49878896	G	A
NC_040256.1	50040125	C	T
NC_040256.1	50506786	A	G
NC_040256.1	50667561	T	C
NC_040256.1	50756612	A	G
NC_040256.1	50856481	C	T
NC_040256.1	50993177	A	G
NC_040256.1	51132668	G	A
NC_040256.1	51263827	A	C
NC_040256.1	51464260	T	C
NC_040256.1	51622280	C	G
NC_040256.1	51778224	T	G
NC_040256.1	51903869	A	T
NC_040256.1	51959642	T	C
NC_040256.1	52126054	C	T
NC_040256.1	52311225	G	A
NC_040256.1	52373880	A	G
NC_040256.1	52483269	G	A
NC_040256.1	52539024	G	A
NC_040256.1	52649410	A	G
NC_040256.1	52731271	T	G
NC_040256.1	52849543	T	C
NC_040256.1	53102109	C	T
NC_040256.1	53159897	T	C
NC_040256.1	53215766	A	G
NC_040256.1	53274192	T	C
NC_040256.1	53337367	G	C
NC_040256.1	53394940	C	T
NC_040256.1	53449587	T	C
NC_040256.1	53508066	A	G
NC_040256.1	53626229	A	G
NC_040256.1	53696984	G	T
NC_040256.1	53826992	G	A

NC_040256.1	54064134	A	G
NC_040256.1	54139810	A	C
NC_040256.1	54255597	G	T
NC_040256.1	54547661	A	T
NC_040256.1	54607949	G	A
NC_040256.1	54734497	C	A
NC_040256.1	54789183	G	A
NC_040256.1	54858376	A	G
NC_040256.1	54940208	T	C
NC_040256.1	55113327	C	A
NC_040256.1	55187587	C	T
NC_040256.1	55390723	C	A
NC_040256.1	55531642	A	G
NC_040256.1	55643530	T	C
NC_040256.1	55760734	A	T
NC_040256.1	55961828	C	T
NC_040256.1	56020990	T	A
NC_040256.1	56089836	C	T
NC_040256.1	56173745	T	C
NC_040256.1	56259604	A	T
NC_040256.1	56330322	C	T
NC_040256.1	56391669	G	A
NC_040256.1	56451323	T	C
NC_040256.1	56512381	A	G
NC_040256.1	56571341	A	G
NC_040256.1	56618709	A	G
NC_040256.1	56776657	T	A
NC_040256.1	56849227	A	G
NC_040256.1	56992525	T	C
NC_040256.1	57096297	T	A
NC_040256.1	57199992	G	A
NC_040256.1	57404170	T	C
NC_040256.1	57635648	G	A
NC_040256.1	57772263	T	C
NC_040256.1	57961257	A	T
NC_040256.1	58113658	G	C
NC_040256.1	58265178	G	C
NC_040256.1	58394477	A	G
NC_040256.1	58542635	C	T
NC_040256.1	58841717	G	A
NC_040256.1	58954078	C	A
NC_040256.1	59071133	A	C
NC_040256.1	59836394	C	A
NC_040256.1	60103241	A	G
NC_040256.1	60188625	C	T
NC_040256.1	60592683	C	T
NC_040256.1	60698905	A	C

NC_040256.1	60944438	G	A
NC_040256.1	61054407	C	T
NC_040256.1	61120489	A	G
NC_040256.1	61174087	C	T
NC_040256.1	61249929	A	G
NC_040256.1	61294158	C	T
NC_040256.1	61342085	G	A
NC_040256.1	61402663	T	C
NC_040256.1	61480064	A	G
NC_040256.1	61531727	A	G
NC_040256.1	61589241	C	T
NC_040256.1	61707817	T	C
NC_040256.1	61863258	C	A
NC_040256.1	61897543	T	A
NC_040256.1	61958229	A	G
NC_040256.1	62273253	T	G
NC_040256.1	62293221	T	C
NC_040256.1	62426801	A	G
NC_040256.1	62513865	A	T
NC_040256.1	62561662	A	G
NC_040256.1	62611319	T	C
NC_040256.1	62641547	T	C
NC_040256.1	62709467	T	A
NC_040256.1	62794137	C	T
NC_040256.1	62847613	T	C
NC_040256.1	62907227	T	C
NC_040256.1	62972315	T	C
NC_040256.1	63029349	T	C
NC_040256.1	63076961	G	A
NC_040256.1	63129456	G	T
NC_040256.1	63185486	C	T
NC_040256.1	63235194	A	G
NC_040256.1	63307649	G	A
NC_040256.1	63361479	T	C
NC_040256.1	63431663	T	C
NC_040256.1	63493212	C	T
NC_040256.1	63562805	A	T
NC_040256.1	63611134	A	T
NC_040256.1	63660736	C	T
NC_040256.1	63718583	A	G
NC_040256.1	63789757	A	G
NC_040256.1	63843371	T	C
NC_040256.1	63917020	C	T
NC_040256.1	63979464	C	G
NC_040256.1	64018888	G	A
NC_040256.1	64029104	C	T
NC_040256.1	64054184	T	G

NC_040256.1	64097602	A	G
NC_040256.1	64197453	A	G
NC_040256.1	64234022	G	A
NC_040256.1	64284823	A	G
NC_040256.1	64339654	A	G
NC_040256.1	64384233	A	G
NC_040256.1	64448048	A	G
NC_040256.1	64514125	G	C
NC_040256.1	64578820	C	T
NC_040256.1	64601765	A	G
NC_040256.1	64685645	A	G
NC_040256.1	64701921	T	G
NC_040256.1	64728066	C	A
NC_040256.1	64814334	T	C
NC_040256.1	64908632	G	A
NC_040256.1	64964608	A	G
NC_040256.1	65018817	C	T
NC_040256.1	65092624	A	T
NC_040256.1	65194515	A	G
NC_040256.1	65254041	T	C
NC_040256.1	65287584	G	A
NC_040256.1	65330094	A	G
NC_040256.1	65424259	C	T
NC_040256.1	65532526	A	G
NC_040256.1	65695136	A	C
NC_040256.1	65747756	T	C
NC_040256.1	65822789	G	A
NC_040256.1	66032710	C	T
NC_040256.1	66100692	T	C
NC_040256.1	66150763	G	T
NC_040256.1	66371062	T	G
NC_040256.1	66604126	G	A
NC_040256.1	66733130	G	T
NC_040256.1	66830421	G	A
NC_040256.1	66866377	G	T
NC_040256.1	67127966	T	C
NC_040256.1	67281713	G	A
NC_040256.1	67344396	T	C
NC_040256.1	67482561	G	C
NC_040256.1	67526643	T	A
NC_040256.1	67603070	C	G
NC_040256.1	67671640	T	G
NC_040256.1	67710018	G	T
NC_040256.1	67784995	A	G
NC_040256.1	67814790	C	T
NC_040256.1	67892981	A	T
NC_040256.1	67974137	T	G

NC_040256.1	68029294	A	G
NC_040256.1	68059092	G	A
NC_040256.1	68144617	C	T
NC_040256.1	68205197	T	C
NC_040256.1	68361119	T	G
NC_040256.1	68416992	A	G
NC_040256.1	68451394	T	C
NC_040256.1	68477562	A	C
NC_040256.1	68621140	C	G
NC_040256.1	68656245	G	C
NC_040256.1	68755487	T	G
NC_040256.1	68795747	T	G
NC_040256.1	68932916	G	A
NC_040256.1	68992366	A	C
NC_040256.1	69406721	G	T
NC_040256.1	69485154	G	A
NC_040256.1	69604784	A	G
NC_040256.1	69659052	G	A
NC_040256.1	69736224	A	G
NC_040256.1	69862129	A	G
NC_040256.1	70019250	G	C
NC_040256.1	70145009	T	C
NC_040256.1	70186899	T	C
NC_040256.1	70243473	C	A
NC_040256.1	70349794	A	G
NC_040256.1	70442010	T	C
NC_040256.1	70520652	T	A
NC_040256.1	70683045	A	G
NC_040256.1	71192824	A	C
NC_040256.1	71270757	C	G
NC_040256.1	71351163	A	C
NC_040256.1	71721084	A	G
NC_040256.1	71771977	A	G
NC_040256.1	71818434	A	G
NC_040256.1	71878173	G	T
NC_040256.1	71932245	A	G
NC_040256.1	72065299	T	C
NC_040256.1	72125130	C	T
NC_040256.1	72175329	G	A
NC_040256.1	72233193	A	G
NC_040256.1	72349304	T	C
NC_040256.1	72428724	C	G
NC_040256.1	72481739	T	C
NC_040256.1	72527725	G	A
NC_040256.1	72598807	T	C
NC_040256.1	72654002	G	A
NC_040256.1	72729130	C	T

NC_040256.1	72791622	G	C
NC_040256.1	72835252	T	G
NC_040256.1	72884866	T	C
NC_040256.1	72914881	T	G
NC_040256.1	73018019	C	T
NC_040256.1	73096473	A	G
NC_040256.1	73161354	G	A
NC_040256.1	73219992	T	C
NC_040256.1	73276764	A	G
NC_040256.1	73332779	T	C
NC_040256.1	73389692	G	A
NC_040256.1	73445023	T	C
NC_040256.1	73504425	C	T
NC_040256.1	73554485	G	C
NC_040256.1	73607492	A	G
NC_040256.1	73657027	C	T
NC_040256.1	73728591	T	C
NC_040256.1	73791620	C	T
NC_040256.1	73902606	A	G
NC_040256.1	73940430	T	C
NC_040256.1	74027204	T	C
NC_040256.1	74162626	G	C
NC_040256.1	74249795	C	A
NC_040256.1	74295424	T	C
NC_040256.1	74361341	A	T
NC_040256.1	74403614	T	C
NC_040256.1	74523154	C	T
NC_040256.1	74567845	T	G
NC_040256.1	74618337	A	G
NC_040256.1	74681855	C	G
NC_040256.1	74728658	C	T
NC_040256.1	74740775	G	A
NC_040256.1	74794835	G	A
NC_040256.1	74841529	C	T
NC_040256.1	74892580	A	G
NC_040256.1	74971202	C	T
NC_040256.1	75085073	G	T
NC_040256.1	75166536	A	C
NC_040256.1	75422816	A	T
NC_040256.1	75673798	T	C
NC_040256.1	75716477	T	C
NC_040256.1	75907785	C	A
NC_040256.1	75958310	C	T
NC_040256.1	76096127	C	T
NC_040256.1	76168812	C	G
NC_040256.1	76275235	A	G
NC_040256.1	76383475	C	A

NC_040256.1	76467784	A	G
NC_040256.1	76548781	G	A
NC_040256.1	76655324	C	T
NC_040256.1	76816103	C	T
NC_040256.1	77079079	T	A
NC_040256.1	77115435	T	C
NC_040256.1	77230185	G	T
NC_040256.1	77338228	T	C
NC_040256.1	77447699	T	C
NC_040256.1	77586246	A	G
NC_040256.1	77829586	G	A
NC_040256.1	77966414	A	T
NC_040256.1	78012461	C	G
NC_040256.1	78504019	T	C
NC_040256.1	78590609	A	G
NC_040256.1	78637660	C	T
NC_040256.1	78703904	G	A
NC_040256.1	78785576	G	C
NC_040256.1	78827218	T	C
NC_040256.1	78924438	C	G
NC_040256.1	78952997	T	C
NC_040256.1	79024678	G	T
NC_040256.1	79053424	G	C
NC_040256.1	79240706	C	T
NC_040256.1	79408046	C	A
NC_040256.1	80660763	A	G
NC_040256.1	80965088	T	C
NC_040256.1	81214989	G	C
NC_040256.1	81447836	G	A
NC_040256.1	81675182	A	G
NC_040256.1	81747343	G	A
NC_040256.1	81874173	A	C
NC_040256.1	81955982	A	G
NC_040256.1	82049045	C	T
NC_040256.1	82245440	G	T
NC_040256.1	82274377	A	G
NC_040256.1	82356570	G	T
NC_040256.1	82532732	T	C
NC_040256.1	82626076	T	C
NC_040256.1	82674074	C	T
NC_040256.1	82815477	G	A
NC_040256.1	82867759	C	T
NC_040256.1	82928359	C	T
NC_040256.1	83018805	C	T
NC_040256.1	83173853	C	T
NC_040256.1	83224870	A	G
NC_040256.1	83341711	T	C

NC_040256.1	83530763	A	G
NC_040256.1	83571825	C	T
NC_040256.1	83634078	A	G
NC_040256.1	83769400	T	C
NC_040256.1	83844360	G	A
NC_040256.1	83877343	A	G
NC_040256.1	83979850	G	A
NC_040256.1	84280539	A	G
NC_040256.1	84464387	A	G
NC_040256.1	84537394	C	T
NC_040256.1	84615653	A	G
NC_040256.1	84658436	T	A
NC_040256.1	84697195	C	A
NC_040256.1	84764707	T	C
NC_040256.1	84799248	A	C
NC_040256.1	84856098	A	G
NC_040256.1	84941664	C	T
NC_040256.1	85489331	T	C
NC_040256.1	85826662	T	A
NC_040256.1	85903256	T	C
NC_040256.1	85972330	C	G
NC_040256.1	86146904	T	C
NC_040256.1	86217726	A	G
NC_040256.1	86417315	C	A
NC_040256.1	86579657	G	A
NC_040256.1	86838179	A	G
NC_040256.1	87023652	C	T
NC_040256.1	87118503	A	G
NC_040256.1	87183998	T	C
NC_040256.1	87324806	T	C
NC_040256.1	87396198	T	C
NC_040256.1	87535398	A	G
NC_040256.1	87560438	A	G
NC_040256.1	87631070	C	T
NC_040256.1	87674394	T	A
NC_040256.1	87845802	T	C
NC_040256.1	87896221	C	G
NC_040256.1	87966959	A	G
NC_040256.1	88060025	C	A
NC_040256.1	88205188	G	A
NC_040256.1	88240970	G	A
NC_040256.1	88288826	T	A
NC_040256.1	88744178	C	T
NC_040256.1	88818711	C	T
NC_040256.1	89756172	G	A
NC_040256.1	89791690	A	G
NC_040256.1	89829975	A	C

NC_040256.1	89870988	C	T
NC_040256.1	90434355	G	A
NC_040256.1	90497816	A	C
NC_040256.1	90544077	C	G
NC_040256.1	90590301	C	T
NC_040256.1	90691882	C	G
NC_040256.1	91113794	A	G
NC_040256.1	91171396	G	C
NC_040256.1	91210646	T	C
NC_040256.1	91466580	A	G
NC_040256.1	91711198	T	G
NC_040256.1	91763619	A	G
NC_040256.1	91800054	T	G
NC_040256.1	91832016	A	G
NC_040256.1	91873554	T	C
NC_040256.1	91957964	A	G
NC_040256.1	92031845	A	G
NC_040256.1	92100670	A	G
NC_040256.1	92200217	G	A
NC_040256.1	92314042	T	C
NC_040256.1	92380465	G	A
NC_040256.1	92448748	C	G
NC_040256.1	92571161	A	G
NC_040256.1	92633822	C	A
NC_040256.1	92709446	C	T
NC_040256.1	92792375	A	C
NC_040256.1	92986791	G	T
NC_040256.1	93051867	A	G
NC_040256.1	93111776	G	A
NC_040256.1	93147990	A	G
NC_040256.1	93248321	T	C
NC_040256.1	93300530	A	G
NC_040256.1	93335965	T	C
NC_040256.1	93395980	T	C
NC_040256.1	93456554	A	G
NC_040256.1	93493845	A	G
NC_040256.1	93541310	C	T
NC_040256.1	93611416	T	C
NC_040256.1	93649773	A	G
NC_040256.1	93697383	C	T
NC_040256.1	93766562	G	A
NC_040256.1	93841226	T	C
NC_040256.1	93910480	A	G
NC_040256.1	93972164	A	C
NC_040256.1	94035553	T	C
NC_040256.1	94101952	A	T
NC_040256.1	94327831	T	C

NC_040256.1	94393520	A		G
NC_040256.1	94467634	G		C
NC_040256.1	94606626	T		C
NC_040256.1	94689206	A		G
NC_040256.1	94834893	G		T
NC_040256.1	94904240	G		A
NC_040256.1	94959780	G		T
NC_040256.1	95032009		0	A
NC_040256.1	95068194	A		C
NC_040256.1	95112140	A		G
NC_040256.1	95167145	A		G
NC_040256.1	95596089	A		G
NC_040256.1	95729877	T		G
NC_040256.1	95773275	A		C
NC_040256.1	95852312	A		G
NC_040256.1	95878038	G		A
NC_040256.1	96010392	C		T
NC_040256.1	96069555	G		A
NC_040256.1	96124227	T		C
NC_040256.1	96162059	A		G
NC_040256.1	96214704	A		G
NC_040256.1	96268362	C		A
NC_040256.1	96411398	A		G
NC_040256.1	96432635	G		A
NC_040256.1	96506875	T		C
NC_040256.1	96626602	T		C
NC_040256.1	96698460	C		G
NC_040256.1	96734782	A		T
NC_040256.1	96860529	T		C
NC_040256.1	96965308	G		A
NC_040256.1	97009630	T		C
NC_040256.1	97045393	C		T
NC_040256.1	97093922	A		G
NC_040256.1	97194647	G		T
NC_040256.1	97346491	C		T
NC_040256.1	97378440	T		C
NC_040256.1	97441168	T		C
NC_040256.1	97470222	T		G
NC_040256.1	97570149	C		T
NC_040256.1	97878047	G		T
NC_040256.1	98149960	A		G
NC_040256.1	98198522	G		T
NC_040256.1	98231635	C		T
NC_040256.1	98305258	A		G
NC_040256.1	98326922	G		A
NC_040256.1	98385611	C		T
NC_040256.1	98420184	G		A

NC_040256.1	98486016	C	T
NC_040256.1	98548974	A	G
NC_040256.1	98621470	A	C
NC_040256.1	98707064	C	A
NC_040256.1	98748493	G	A
NC_040256.1	98806218	C	T
NC_040256.1	98880419	T	G
NC_040256.1	98944498	C	T
NC_040256.1	98998687	A	G
NC_040256.1	99070854	A	G
NC_040256.1	99141088	C	T
NC_040256.1	99166638	C	T
NC_040256.1	99354017	C	G
NC_040256.1	99517936	C	A
NC_040256.1	99560187	T	C
NC_040256.1	99625241	A	G
NC_040256.1	99706159	T	C
NC_040256.1	99907183	G	A
NC_040256.1	99947435	C	A
NC_040256.1	100025955	G	A
NC_040256.1	100084697	G	C
NC_040256.1	100149902	C	T
NC_040256.1	100375544	T	A
NC_040256.1	100439131	C	T
NC_040256.1	100584463	A	G
NC_040256.1	100648366	G	C
NC_040256.1	100719493	A	T
NC_040256.1	100771719	A	G
NC_040256.1	100866524	A	G
NC_040256.1	100884311	C	G
NC_040256.1	100945753	G	A
NC_040256.1	101069905	T	C
NC_040256.1	101142754	G	A
NC_040256.1	101306207	T	C
NC_040256.1	101355199	C	T
NC_040256.1	101420919	T	C
NC_040256.1	101532477	A	T
NC_040256.1	101620028	G	A
NC_040256.1	101685820	T	C
NC_040256.1	101767678	G	A
NC_040256.1	101802669	A	T
NC_040256.1	101950425	A	G
NC_040256.1	102025577	T	A
NC_040256.1	102097808	G	A
NC_040256.1	102226939	C	G
NC_040256.1	102280777	G	A
NC_040256.1	102425087	C	T

NC_040256.1	102471970	C	T
NC_040256.1	102525401	A	C
NC_040256.1	102601592	A	G
NC_040256.1	102916298	C	T
NC_040256.1	103009382	A	G
NC_040256.1	103354717	G	T
NC_040256.1	103436797	G	T
NC_040256.1	103606320	A	G
NC_040256.1	103666519	T	C
NC_040256.1	104031241	T	C
NC_040256.1	104212431	A	G
NC_040256.1	104256634	C	T
NC_040256.1	104312698	T	C
NC_040256.1	104343442	A	C
NC_040256.1	104411292	A	G
NC_040256.1	104462365	T	C
NC_040256.1	104493248	A	G
NC_040256.1	104541256	G	T
NC_040256.1	104702071	A	G
NC_040256.1	104762429	T	C
NC_040256.1	104818020	T	C
NC_040256.1	104912419	T	C
NC_040256.1	105026081	C	T
NC_040256.1	105128111	T	C
NC_040256.1	105171012	A	G
NC_040256.1	105320490	A	G
NC_040256.1	105560131	G	A
NC_040256.1	105787954	C	T
NC_040256.1	105972324	T	C
NC_040256.1	106212014	T	C
NC_040256.1	106424555	C	T
NC_040256.1	106556623	C	T
NC_040256.1	106612976	C	T
NC_040256.1	106891605	G	A
NC_040256.1	107051324	A	G
NC_040256.1	107156068	A	G
NC_040256.1	107243664	T	C
NC_040256.1	107518382	G	C
NC_040256.1	107592236	T	A
NC_040256.1	107652870	T	A
NC_040256.1	107694336	C	T
NC_040256.1	107764763	G	A
NC_040256.1	107820260	T	G
NC_040256.1	107858303	A	G
NC_040256.1	107943688	C	A
NC_040256.1	108009732	T	C
NC_040256.1	108107831	A	C

NC_040256.1	108143847	C	A
NC_040256.1	108196275	G	C
NC_040256.1	108243835	A	G
NC_040256.1	108299079	C	T
NC_040256.1	108343582	A	G
NC_040256.1	108388239	A	G
NC_040256.1	108454136	T	C
NC_040256.1	108505811	A	C
NC_040256.1	108581407	T	C
NC_040256.1	108632551	A	C
NC_040256.1	108688491	A	G
NC_040256.1	108730586	A	G
NC_040256.1	108785577	G	A
NC_040256.1	108863445	T	C
NC_040256.1	108867796	T	C
NC_040256.1	108901576	C	T
NC_040256.1	108956932	T	C
NC_040256.1	109029648	G	A
NC_040256.1	109046129	C	T
NC_040256.1	109087973	G	A
NC_040256.1	109148736	T	G
NC_040256.1	109210469	A	G
NC_040256.1	109268370	G	A
NC_040256.1	109323263	A	C
NC_040256.1	109375936	A	G
NC_040256.1	109433721	C	T
NC_040256.1	109486785	G	A
NC_040256.1	109554875	C	T
NC_040256.1	109585081	T	G
NC_040256.1	109633234	C	T
NC_040256.1	109666096	C	T
NC_040256.1	109739943	T	G
NC_040256.1	109858588	T	A
NC_040256.1	109910793	T	C
NC_040256.1	109990267	C	T
NC_040256.1	110098706	A	T
NC_040256.1	110163305	C	T
NC_040256.1	110252373	T	C
NC_040256.1	110297375	A	G
NC_040256.1	110338309	T	C
NC_040256.1	110393243	A	G
NC_040256.1	110463786	T	C
NC_040256.1	110529053	A	G
NC_040256.1	110579809	A	G
NC_040256.1	110624895	A	G
NC_040256.1	110679714	C	T
NC_040256.1	110731184	T	C

NC_040256.1	110799079	G	A
NC_040256.1	110900854	T	G
NC_040256.1	110948110	A	G
NC_040256.1	110979999	T	C
NC_040256.1	111033466	T	A
NC_040256.1	111094712	A	G
NC_040256.1	111130314	T	C
NC_040256.1	111182639	A	G
NC_040256.1	111219923	A	T
NC_040256.1	111265650	A	G
NC_040256.1	111323550	A	C
NC_040256.1	111513739	T	G
NC_040256.1	111555356	G	T
NC_040256.1	111609728	C	T
NC_040256.1	111681937	G	A
NC_040256.1	111720121	T	A
NC_040256.1	111770598	G	C
NC_040256.1	111846066	A	G
NC_040256.1	111952285	G	A
NC_040256.1	112040245	G	A
NC_040256.1	112374775	G	A
NC_040256.1	112434322	A	C
NC_040256.1	112465585	T	G
NC_040256.1	112566814	A	G
NC_040256.1	112637501	A	G
NC_040256.1	112685222	G	A
NC_040256.1	112735939	G	A
NC_040256.1	112788760	A	G
NC_040256.1	112842938	T	G
NC_040256.1	112897269	A	G
NC_040256.1	112948533	A	G
NC_040256.1	113005735	0	G
NC_040256.1	113054108	T	C
NC_040256.1	113111108	T	C
NC_040256.1	113171912	C	T
NC_040256.1	113226959	G	A
NC_040256.1	113295893	A	C
NC_040256.1	113403263	A	G
NC_040256.1	113462530	T	C
NC_040256.1	113518244	A	C
NC_040256.1	113572822	C	A
NC_040256.1	113645371	T	G
NC_040256.1	113688560	T	C
NC_040256.1	113741594	G	A
NC_040256.1	113796098	G	A
NC_040256.1	113854028	T	C
NC_040256.1	113904532	A	G

NC_040256.1	113958329	T	C
NC_040256.1	114015223	T	C
NC_040256.1	114067856	T	G
NC_040256.1	114124515	T	C
NC_040256.1	114172885	G	A
NC_040256.1	114227515	A	C
NC_040256.1	114281363	A	C
NC_040256.1	114329478	G	T
NC_040256.1	114383796	A	G
NC_040256.1	114419411	G	A
NC_040256.1	114464097	C	A
NC_040256.1	114541459	T	C
NC_040256.1	114597118	C	G
NC_040256.1	114659019	G	A
NC_040256.1	114720374	T	A
NC_040256.1	114769401	A	G
NC_040256.1	114820195	T	C
NC_040256.1	114873273	A	C
NC_040256.1	114919937	G	A
NC_040256.1	114972339	A	C
NC_040256.1	115006694	A	G
NC_040256.1	115056216	G	A
NC_040256.1	115116255	T	C
NC_040256.1	115152230	G	C
NC_040256.1	115155033	C	G
NC_040256.1	115226072	A	G
NC_040256.1	115282486	C	T
NC_040256.1	115329886	C	T
NC_040256.1	115367551	A	C
NC_040256.1	115423127	T	C
NC_040256.1	115469069	T	C
NC_040256.1	115525244	A	G
NC_040256.1	115562905	T	A
NC_040256.1	115625137	T	C
NC_040256.1	115678480	G	A
NC_040256.1	115740744	A	G
NC_040256.1	115798165	C	T
NC_040256.1	115847011	A	C
NC_040256.1	115872816	A	G
NC_040256.1	115873091	G	T
NC_040256.1	115873609	A	G
NC_040256.1	115937450	T	A
NC_040256.1	115989321	T	C
NC_040256.1	116057104	A	T
NC_040256.1	116110717	A	G
NC_040256.1	116169311	G	A
NC_040256.1	116223432	C	T

NC_040256.1	116301816	G	A
NC_040256.1	116359881	T	C
NC_040256.1	116385054	C	T
NC_040256.1	116446578	A	G
NC_040256.1	116507310	A	G
NC_040256.1	116566713	C	G
NC_040256.1	116619770	T	G
NC_040256.1	116645115	C	T
NC_040256.1	116683817	A	G
NC_040256.1	116739503	T	C
NC_040256.1	116793290	A	G
NC_040256.1	116844389	T	C
NC_040256.1	116901987	A	G
NC_040256.1	116950621	C	T
NC_040256.1	117005772	G	A
NC_040256.1	117071779	T	C
NC_040256.1	117099990	G	T
NC_040256.1	117161409	C	T
NC_040256.1	117216674	C	T
NC_040256.1	117268112	T	C
NC_040256.1	117324613	T	C
NC_040256.1	117381106	G	A
NC_040256.1	117438888	A	G
NC_040256.1	117504441	A	G
NC_040256.1	117522616	T	C
NC_040256.1	117523084	A	G
NC_040256.1	117574816	A	G
NC_040257.1	127635	A	C
NC_040257.1	153111	T	G
NC_040257.1	194522	A	G
NC_040257.1	250843	G	T
NC_040257.1	308591	A	G
NC_040257.1	329440	A	G
NC_040257.1	362652	A	G
NC_040257.1	406974	C	T
NC_040257.1	418226	A	G
NC_040257.1	454783	G	A
NC_040257.1	464089	T	C
NC_040257.1	519794	A	G
NC_040257.1	578789	A	G
NC_040257.1	627399	A	T
NC_040257.1	653002	A	C
NC_040257.1	716892	A	G
NC_040257.1	776184	A	G
NC_040257.1	821749	A	G
NC_040257.1	833509	G	C
NC_040257.1	878529	A	G

NC_040257.1	973851	G	T
NC_040257.1	1005544	A	C
NC_040257.1	1036888	T	G
NC_040257.1	1076428	A	G
NC_040257.1	1095313	A	G
NC_040257.1	1128362	T	G
NC_040257.1	1145216	T	C
NC_040257.1	1219288	G	A
NC_040257.1	1248580	A	T
NC_040257.1	1269298	A	T
NC_040257.1	1323626	G	A
NC_040257.1	1363760	A	C
NC_040257.1	1405234	T	G
NC_040257.1	1502765	T	C
NC_040257.1	1623441	G	C
NC_040257.1	1673256	T	C
NC_040257.1	1675013	T	G
NC_040257.1	1698316	C	G
NC_040257.1	1731137	T	C
NC_040257.1	1783788	C	T
NC_040257.1	1791960	A	G
NC_040257.1	1893175	C	T
NC_040257.1	1942307	G	A
NC_040257.1	2208168	C	T
NC_040257.1	2271157	C	T
NC_040257.1	2303122	A	G
NC_040257.1	2348155	G	A
NC_040257.1	2348885	C	T
NC_040257.1	2373705	A	G
NC_040257.1	2413318	C	A
NC_040257.1	2479693	C	T
NC_040257.1	2543734	A	G
NC_040257.1	2647000	C	A
NC_040257.1	2681498	G	A
NC_040257.1	2694968	T	C
NC_040257.1	2718321	T	A
NC_040257.1	2742471	T	C
NC_040257.1	2763130	G	A
NC_040257.1	2808890	T	C
NC_040257.1	2849499	G	A
NC_040257.1	2927166	A	G
NC_040257.1	2980499	C	T
NC_040257.1	3022136	G	T
NC_040257.1	3066101	T	C
NC_040257.1	3138884	C	G
NC_040257.1	3174502	T	C
NC_040257.1	3529523	T	A

NC_040257.1	3557735	C	T
NC_040257.1	3595736	T	C
NC_040257.1	3643799	A	G
NC_040257.1	3670286	T	C
NC_040257.1	3680023	T	C
NC_040257.1	3837008	C	A
NC_040257.1	3899385	G	A
NC_040257.1	4205707	A	G
NC_040257.1	4314190	T	G
NC_040257.1	4395883	T	A
NC_040257.1	4458401	C	T
NC_040257.1	4583862	A	G
NC_040257.1	4752668	A	G
NC_040257.1	4801126	T	C
NC_040257.1	4812060	C	G
NC_040257.1	4977745	G	A
NC_040257.1	5072817	G	T
NC_040257.1	5126156	G	T
NC_040257.1	5181626	C	A
NC_040257.1	5242045	A	T
NC_040257.1	5295974	C	T
NC_040257.1	5324161	G	A
NC_040257.1	5338152	T	C
NC_040257.1	5372606	A	G
NC_040257.1	5517507	G	A
NC_040257.1	5541488	C	T
NC_040257.1	5705622	A	G
NC_040257.1	5732575	T	C
NC_040257.1	5766467	G	A
NC_040257.1	5851385	T	C
NC_040257.1	5981277	C	T
NC_040257.1	6011206	T	C
NC_040257.1	6060295	A	G
NC_040257.1	6107663	A	T
NC_040257.1	6149150	T	C
NC_040257.1	6492113	A	G
NC_040257.1	6530716	C	T
NC_040257.1	6538316	A	G
NC_040257.1	6629860	G	A
NC_040257.1	6684094	T	C
NC_040257.1	6927024	T	C
NC_040257.1	6978320	G	T
NC_040257.1	7024622	C	T
NC_040257.1	7156357	C	A
NC_040257.1	7190352	T	C
NC_040257.1	7254221	C	A
NC_040257.1	7284810	A	G

NC_040257.1	7324367	C		G
NC_040257.1	7346877	C		T
NC_040257.1	7402429	C		T
NC_040257.1	7466168	T		C
NC_040257.1	7471032	T		C
NC_040257.1	7550235	A		G
NC_040257.1	7601421	C		T
NC_040257.1	7649028	A		G
NC_040257.1	7711011	A		G
NC_040257.1	7746091	A		G
NC_040257.1	7754031	T		C
NC_040257.1	7785165	T		C
NC_040257.1	7805097	A		G
NC_040257.1	7852857	G		C
NC_040257.1	7860866	A		G
NC_040257.1	7917493	T		C
NC_040257.1	7963594	G		A
NC_040257.1	7997495	A		G
NC_040257.1	8222147	T		C
NC_040257.1	8295065		0	T
NC_040257.1	8320815	C		G
NC_040257.1	8539880	G		T
NC_040257.1	8590856	A		G
NC_040257.1	8652377	G		C
NC_040257.1	8711230	A		C
NC_040257.1	8768374	G		A
NC_040257.1	8793295	G		C
NC_040257.1	8840381	T		A
NC_040257.1	8894283	A		C
NC_040257.1	8926528	A		G
NC_040257.1	8961365	C		G
NC_040257.1	8979414	G		T
NC_040257.1	9041029	A		G
NC_040257.1	9199972	C		A
NC_040257.1	9417530	C		G
NC_040257.1	9567216	G		C
NC_040257.1	10075954	G		C
NC_040257.1	10253924	C		T
NC_040257.1	10290941	G		C
NC_040257.1	10298027	G		C
NC_040257.1	10792025	C		T
NC_040257.1	10887956	T		C
NC_040257.1	10993604	C		T
NC_040257.1	11021877	T		C
NC_040257.1	11190221	C		T
NC_040257.1	11254546	G		A
NC_040257.1	11263552	C		T

NC_040257.1	11395253	A	G
NC_040257.1	11456182	T	C
NC_040257.1	11511837	A	C
NC_040257.1	11597978	C	A
NC_040257.1	11644852	A	G
NC_040257.1	11655720	T	C
NC_040257.1	11972013	A	C
NC_040257.1	12141355	T	C
NC_040257.1	12171888	A	G
NC_040257.1	12369600	C	A
NC_040257.1	12829367	G	A
NC_040257.1	12880804	T	C
NC_040257.1	13862107	C	T
NC_040257.1	13926013	C	A
NC_040257.1	13986843	T	G
NC_040257.1	14042018	T	G
NC_040257.1	14069076	T	G
NC_040257.1	14304242	T	C
NC_040257.1	14379145	C	T
NC_040257.1	14440064	A	T
NC_040257.1	14509497	A	G
NC_040257.1	14579125	A	G
NC_040257.1	14639876	T	C
NC_040257.1	14697830	A	G
NC_040257.1	14752254	T	C
NC_040257.1	14816526	G	T
NC_040257.1	15189766	A	G
NC_040257.1	15253240	G	A
NC_040257.1	15313566	C	T
NC_040257.1	15440093	T	C
NC_040257.1	15502683	C	T
NC_040257.1	15565321	T	C
NC_040257.1	15620514	A	G
NC_040257.1	15675819	C	T
NC_040257.1	15734124	A	G
NC_040257.1	15799254	A	G
NC_040257.1	15854678	C	T
NC_040257.1	15929662	C	T
NC_040257.1	15999447	A	C
NC_040257.1	16065721	T	C
NC_040257.1	16131559	T	G
NC_040257.1	16183923	C	T
NC_040257.1	16249122	G	C
NC_040257.1	16327144	C	G
NC_040257.1	16434057	G	A
NC_040257.1	16499243	C	A
NC_040257.1	16564708	T	A

NC_040257.1	16623147	C	T
NC_040257.1	16681502	A	G
NC_040257.1	16954007	G	A
NC_040257.1	17007963	C	T
NC_040257.1	17071266	T	C
NC_040257.1	17138761	C	T
NC_040257.1	17193363	A	G
NC_040257.1	17255603	G	A
NC_040257.1	17324678	A	G
NC_040257.1	17380525	A	T
NC_040257.1	17436285	A	G
NC_040257.1	17497518	C	A
NC_040257.1	17557690	G	C
NC_040257.1	17614953	G	A
NC_040257.1	17673783	T	C
NC_040257.1	17726952	T	G
NC_040257.1	17784724	T	C
NC_040257.1	17805423	G	A
NC_040257.1	17911780	G	A
NC_040257.1	17967349	A	G
NC_040257.1	18028830	A	G
NC_040257.1	18099569	A	G
NC_040257.1	18171198	C	T
NC_040257.1	18303755	T	C
NC_040257.1	18359210	T	C
NC_040257.1	18417443	G	T
NC_040257.1	18471962	G	A
NC_040257.1	18530230	T	A
NC_040257.1	18590275	T	G
NC_040257.1	18659201	A	G
NC_040257.1	18715773	A	C
NC_040257.1	18776932	C	T
NC_040257.1	18805099	A	G
NC_040257.1	19053060	A	G
NC_040257.1	19356272	T	G
NC_040257.1	19601696	T	G
NC_040257.1	20313797	A	G
NC_040257.1	20488863	T	C
NC_040257.1	21437955	C	A
NC_040257.1	21728096	A	G
NC_040257.1	21932654	A	C
NC_040257.1	22041857	T	C
NC_040257.1	22106752	A	G
NC_040257.1	22162747	C	T
NC_040257.1	22216087	T	C
NC_040257.1	22285333	T	C
NC_040257.1	22341172	A	C

NC_040257.1	22403826	T	G
NC_040257.1	22463807	C	T
NC_040257.1	22540137	T	C
NC_040257.1	22766590	A	G
NC_040257.1	22818422	T	A
NC_040257.1	22890935	C	T
NC_040257.1	22961188	T	G
NC_040257.1	23023089	T	C
NC_040257.1	23078427	T	C
NC_040257.1	23142646	T	C
NC_040257.1	23198865	A	G
NC_040257.1	23264044	C	G
NC_040257.1	23334837	G	A
NC_040257.1	23387033	C	T
NC_040257.1	24319487	G	A
NC_040257.1	24690121	C	T
NC_040257.1	25228635	T	C
NC_040257.1	25740870	T	C
NC_040257.1	26159262	G	A
NC_040257.1	26267440	G	A
NC_040257.1	26419413	C	T
NC_040257.1	26754098	A	T
NC_040257.1	26951795	G	A
NC_040257.1	27086849	T	C
NC_040257.1	27154503	A	G
NC_040257.1	27453675	T	C
NC_040257.1	27613174	G	T
NC_040257.1	27908608	G	A
NC_040257.1	28423282	C	T
NC_040257.1	28493762	A	G
NC_040257.1	28576550	C	T
NC_040257.1	28686443	G	A
NC_040257.1	28967157	A	T
NC_040257.1	29682760	G	A
NC_040257.1	29804574	A	G
NC_040257.1	30155992	C	T
NC_040257.1	30214470	T	C
NC_040257.1	30327690	G	A
NC_040257.1	30399036	T	C
NC_040257.1	30975494	A	G
NC_040257.1	31689463	T	C
NC_040257.1	32230031	C	T
NC_040257.1	32535142	T	A
NC_040257.1	33304896	T	C
NC_040257.1	33476026	T	C
NC_040257.1	33589882	G	T
NC_040257.1	33912741	A	G

NC_040257.1	34052546	T	A
NC_040257.1	34174875	C	A
NC_040257.1	34298939	G	T
NC_040257.1	34476703	T	C
NC_040257.1	34592521	T	C
NC_040257.1	34776208	G	C
NC_040257.1	34965221	A	C
NC_040257.1	35126707	T	C
NC_040257.1	35290362	A	G
NC_040257.1	35376482	T	C
NC_040257.1	35501099	G	T
NC_040257.1	35607602	G	A
NC_040257.1	35904096	C	T
NC_040257.1	36397589	G	A
NC_040257.1	36755285	C	A
NC_040257.1	36852140	T	C
NC_040257.1	36992126	G	A
NC_040257.1	37102021	C	T
NC_040257.1	37168778	T	C
NC_040257.1	38122776	G	A
NC_040257.1	38349629	G	T
NC_040257.1	38793336	C	T
NC_040257.1	39273128	G	A
NC_040257.1	39508756	G	A
NC_040257.1	39775556	C	G
NC_040257.1	39887805	G	C
NC_040257.1	40052625	T	A
NC_040257.1	40249058	T	C
NC_040257.1	40668815	G	T
NC_040257.1	40768243	G	A
NC_040257.1	40836778	C	T
NC_040257.1	41293587	C	T
NC_040257.1	41834370	A	G
NC_040257.1	41913851	A	G
NC_040257.1	41981769	A	G
NC_040257.1	42051353	A	G
NC_040257.1	42120859	C	A
NC_040257.1	42180435	A	G
NC_040257.1	42230064	C	T
NC_040257.1	42501317	C	T
NC_040257.1	42560842	A	G
NC_040257.1	42691280	G	T
NC_040257.1	42988335	T	C
NC_040257.1	43124314	T	C
NC_040257.1	43251800	C	G
NC_040257.1	43318652	T	C
NC_040257.1	43576531	G	A

NC_040257.1	43893651	A	G
NC_040257.1	44102255	G	T
NC_040257.1	44286302	G	A
NC_040257.1	44386854	T	A
NC_040257.1	44504481	T	C
NC_040257.1	44584833	A	G
NC_040257.1	44854998	G	A
NC_040257.1	45104504	A	G
NC_040257.1	45211758	T	C
NC_040257.1	45362091	A	G
NC_040257.1	45517759	T	C
NC_040257.1	45673116	T	C
NC_040257.1	46381892	T	C
NC_040257.1	46693282	A	G
NC_040257.1	47142262	A	G
NC_040257.1	47294348	T	A
NC_040257.1	47569125	A	G
NC_040257.1	47684139	G	T
NC_040257.1	47763165	A	G
NC_040257.1	47891464	A	T
NC_040257.1	48068026	T	C
NC_040257.1	48129939	G	T
NC_040257.1	48200355	C	T
NC_040257.1	48302768	T	C
NC_040257.1	48651062	C	T
NC_040257.1	48739520	T	C
NC_040257.1	48828167	A	G
NC_040257.1	48970688	C	G
NC_040257.1	49073468	G	A
NC_040257.1	49545847	G	A
NC_040257.1	49741714	T	C
NC_040257.1	50054149	T	A
NC_040257.1	50918436	T	C
NC_040257.1	51636963	C	G
NC_040257.1	51832907	T	C
NC_040257.1	52398749	G	A
NC_040257.1	52979034	T	C
NC_040257.1	53497195	A	T
NC_040257.1	53874879	C	G
NC_040257.1	54259651	C	T
NC_040257.1	54320063	A	T
NC_040257.1	54376236	T	C
NC_040257.1	54437568	C	A
NC_040257.1	54555251	C	T
NC_040257.1	54613705	G	A
NC_040257.1	54673528	A	G
NC_040257.1	54729090	T	A

NC_040257.1	54783298	A	C
NC_040257.1	54811301	G	A
NC_040257.1	54871340	T	C
NC_040257.1	54926754	T	C
NC_040257.1	54998067	A	T
NC_040257.1	55073761	T	C
NC_040257.1	55128527	T	C
NC_040257.1	55206288	T	C
NC_040257.1	55268154	A	G
NC_040257.1	55323818	G	A
NC_040257.1	55381990	C	T
NC_040257.1	55451058	A	G
NC_040257.1	55511880	A	C
NC_040257.1	55545577	T	C
NC_040257.1	55799731	C	A
NC_040257.1	56078113	G	A
NC_040257.1	56490517	G	A
NC_040257.1	56776680	A	T
NC_040257.1	57535237	G	A
NC_040257.1	58513099	G	C
NC_040257.1	59028288	T	A
NC_040257.1	59230345	A	C
NC_040257.1	59365922	A	G
NC_040257.1	59439763	T	C
NC_040257.1	59883417	A	G
NC_040257.1	59913265	C	G
NC_040257.1	60058260	T	C
NC_040257.1	60099233	A	G
NC_040257.1	60336050	C	T
NC_040257.1	60448926	A	G
NC_040257.1	60474274	A	G
NC_040257.1	60531843	A	G
NC_040257.1	60583915	C	A
NC_040257.1	60635080	T	C
NC_040257.1	60673926	T	C
NC_040257.1	60778017	A	C
NC_040257.1	60867875	C	T
NC_040257.1	61183545	T	C
NC_040257.1	61257255	G	C
NC_040257.1	61302130	C	T
NC_040257.1	61382275	A	T
NC_040257.1	61553423	G	A
NC_040257.1	61617972	A	G
NC_040257.1	61805591	T	C
NC_040257.1	61978261	C	T
NC_040257.1	62109135	A	G
NC_040257.1	62135466	G	A

NC_040257.1	62159761	T	A
NC_040257.1	62189921	T	C
NC_040257.1	62243665	T	G
NC_040257.1	62269394	G	A
NC_040257.1	62501303	T	C
NC_040257.1	62567025	C	T
NC_040257.1	62596644	T	C
NC_040257.1	62917269	G	A
NC_040257.1	62986810	A	G
NC_040257.1	63002581	T	C
NC_040257.1	63093947	G	T
NC_040257.1	63126660	G	A
NC_040257.1	63198260	T	C
NC_040257.1	63272296	C	G
NC_040257.1	63374946	A	C
NC_040257.1	63440266	C	G
NC_040257.1	63482263	T	C
NC_040257.1	63503140	C	A
NC_040257.1	63545132	A	G
NC_040257.1	63600074	T	C
NC_040257.1	63647107	T	C
NC_040257.1	63701847	A	G
NC_040257.1	63761411	C	T
NC_040257.1	63807539	C	G
NC_040257.1	63932214	G	C
NC_040257.1	63972898	A	G
NC_040257.1	64082530	T	C
NC_040257.1	64135910	A	G
NC_040257.1	64205632	T	C
NC_040257.1	64287359	T	C
NC_040257.1	64379584	A	G
NC_040257.1	64417902	G	A
NC_040257.1	64477150	C	T
NC_040257.1	64510198	T	C
NC_040257.1	64546809	A	G
NC_040257.1	64626948	T	C
NC_040257.1	64648282	A	T
NC_040257.1	64724390	T	C
NC_040257.1	64763228	G	A
NC_040257.1	64777765	A	G
NC_040257.1	64917309	C	T
NC_040257.1	64976212	G	A
NC_040257.1	65019461	C	T
NC_040257.1	65287091	A	G
NC_040257.1	65335856	G	A
NC_040257.1	65390248	C	G
NC_040257.1	65437610	A	G

NC_040257.1	65461721	T	G
NC_040257.1	65640948	T	C
NC_040257.1	65683495	G	A
NC_040257.1	65747925	C	T
NC_040257.1	65801562	A	G
NC_040257.1	65922888	G	A
NC_040257.1	65943982	T	C
NC_040257.1	66039456	T	C
NC_040257.1	66074753	A	G
NC_040257.1	66121965	C	A
NC_040257.1	66166158	G	C
NC_040257.1	66244574	A	G
NC_040257.1	66303004	G	A
NC_040257.1	66357616	A	G
NC_040257.1	66392278	T	A
NC_040257.1	66463217	T	A
NC_040257.1	66493980	C	A
NC_040257.1	66630611	T	A
NC_040257.1	66772623	A	T
NC_040257.1	66799284	C	T
NC_040257.1	66809102	A	T
NC_040257.1	67258027	A	G
NC_040257.1	67285880	G	T
NC_040257.1	67306665	C	T
NC_040257.1	67400059	C	T
NC_040257.1	67484634	T	G
NC_040257.1	67505349	T	C
NC_040257.1	67586054	T	C
NC_040257.1	67654472	T	C
NC_040257.1	67687289	T	C
NC_040257.1	67782234	T	C
NC_040257.1	68077570	T	C
NC_040257.1	68106373	A	G
NC_040257.1	68127790	G	A
NC_040257.1	68168204	A	G
NC_040257.1	68211931	T	G
NC_040257.1	68258686	G	A
NC_040257.1	68265180	T	C
NC_040257.1	68392052	G	C
NC_040257.1	68521257	G	C
NC_040257.1	68542085	T	C
NC_040257.1	68596201	C	G
NC_040257.1	68824324	G	T
NC_040257.1	68882925	T	C
NC_040257.1	68921595	T	C
NC_040257.1	69001479	T	C
NC_040257.1	69032924	T	C

NC_040257.1	69076327	G	A
NC_040257.1	69113390	G	A
NC_040257.1	69203435	C	T
NC_040257.1	69365181	G	T
NC_040257.1	69409779	A	G
NC_040257.1	69461863	G	C
NC_040257.1	69811618	C	T
NC_040257.1	69870326	C	T
NC_040257.1	69906088	G	C
NC_040257.1	69928244	C	A
NC_040257.1	70000132	A	G
NC_040257.1	70187521	A	C
NC_040257.1	70209114	A	C
NC_040257.1	70243522	A	C
NC_040257.1	70294144	A	G
NC_040257.1	70499266	C	T
NC_040257.1	70566509	G	C
NC_040257.1	71063967	T	A
NC_040257.1	71175244	C	A
NC_040257.1	71255816	G	A
NC_040257.1	71329132	C	T
NC_040257.1	71358211	T	G
NC_040257.1	71420726	A	C
NC_040257.1	71442952	T	C
NC_040257.1	71481206	T	C
NC_040257.1	71551113	A	T
NC_040257.1	71602051	C	G
NC_040257.1	71663472	G	C
NC_040257.1	71734102	T	C
NC_040257.1	71797444	C	T
NC_040257.1	71840958	T	C
NC_040257.1	71854974	T	C
NC_040257.1	71894379	A	G
NC_040257.1	71935319	G	T
NC_040257.1	71948056	A	G
NC_040257.1	72009027	A	C
NC_040257.1	72053075	C	T
NC_040257.1	72076573	A	G
NC_040257.1	72110708	C	T
NC_040257.1	72131596	T	C
NC_040257.1	72161878	G	A
NC_040257.1	72199915	A	C
NC_040257.1	72228668	T	G
NC_040257.1	72297221	C	T
NC_040257.1	72395270	G	A
NC_040257.1	72423416	T	C
NC_040257.1	72453082	A	G

NC_040257.1	72474187	A	G
NC_040257.1	72501863	G	T
NC_040257.1	72524249	C	T
NC_040257.1	72553203	G	A
NC_040257.1	72639616	G	C
NC_040257.1	72664869	C	T
NC_040257.1	72791938	G	A
NC_040257.1	72824513	C	G
NC_040257.1	72848301	A	G
NC_040257.1	72891619	A	G
NC_040257.1	72904516	A	T
NC_040257.1	73050143	T	C
NC_040257.1	73076037	A	T
NC_040257.1	73151611	T	C
NC_040257.1	73180210	C	T
NC_040257.1	73232887	T	C
NC_040257.1	73238160	A	G
NC_040257.1	73263476	C	G
NC_040257.1	73489542	A	C
NC_040257.1	73566212	T	C
NC_040257.1	73620909	C	T
NC_040257.1	73668512	T	C
NC_040257.1	73708793	G	A
NC_040257.1	73757790	A	C
NC_040257.1	73816767	A	G
NC_040257.1	73853752	A	G
NC_040257.1	73896349	A	G
NC_040257.1	73908456	A	G
NC_040257.1	73966056	T	G
NC_040257.1	73988717	T	C
NC_040257.1	74020017	C	T
NC_040257.1	74090262	T	C
NC_040257.1	74164787	C	A
NC_040257.1	74234492	T	C
NC_040257.1	74291572	T	A
NC_040257.1	74344113	A	C
NC_040257.1	74373696	A	G
NC_040257.1	74443309	G	A
NC_040257.1	74454343	A	G
NC_040257.1	74544258	G	A
NC_040257.1	74618235	C	T
NC_040257.1	74649883	G	A
NC_040257.1	74670701	A	G
NC_040257.1	74713395	G	C
NC_040257.1	74769549	T	C
NC_040257.1	74817022	A	G
NC_040257.1	74838611	C	T

NC_040257.1	74853887	G	A
NC_040257.1	74900317	C	T
NC_040257.1	74966137	A	G
NC_040257.1	75099510	C	A
NC_040257.1	75140534	C	T
NC_040257.1	75206956	G	A
NC_040257.1	75241755	G	T
NC_040257.1	75256155	T	C
NC_040257.1	75303523	A	G
NC_040257.1	75333166	G	T
NC_040257.1	75376312	G	A
NC_040257.1	75411924	C	T
NC_040257.1	75424114	G	A
NC_040257.1	75498302	G	C
NC_040257.1	75548546	T	C
NC_040257.1	75584549	A	G
NC_040257.1	75605357	C	T
NC_040257.1	75636804	T	C
NC_040257.1	75675157	A	G
NC_040257.1	75716562	C	T
NC_040257.1	75730061	T	C
NC_040257.1	75780592	G	C
NC_040257.1	75855834	A	G
NC_040257.1	75906928	T	C
NC_040257.1	75941226	T	C
NC_040257.1	75957357	A	G
NC_040257.1	75985188	G	A
NC_040257.1	76023188	T	C
NC_040257.1	76053118	T	C
NC_040257.1	76066781	T	C
NC_040257.1	76107925	G	T
NC_040257.1	76145986	A	G
NC_040257.1	76177367	G	A
NC_040257.1	76251777	A	G
NC_040257.1	76310099	A	G
NC_040257.1	76355323	A	G
NC_040257.1	76381074	G	T
NC_040257.1	76462247	T	C
NC_040257.1	76492282	T	C
NC_040257.1	76521290	G	A
NC_040257.1	76546819	G	A
NC_040257.1	76574508	A	G
NC_040257.1	76600935	A	G
NC_040257.1	76618009	G	T
NC_040257.1	76883445	T	C
NC_040257.1	76888185	G	C
NC_040257.1	76921879	A	G

NC_040257.1	76934065	G	A
NC_040257.1	76986510	T	C
NC_040257.1	77027919	G	A
NC_040257.1	77057342	G	A
NC_040257.1	77066470	G	C
NC_040257.1	77111024	A	C
NC_040257.1	77180540	A	G
NC_040257.1	77224384	G	C
NC_040257.1	77253616	A	G
NC_040257.1	77264132	A	G
NC_040257.1	77302180	A	G
NC_040257.1	77320196	G	A
NC_040257.1	77394275	C	T
NC_040257.1	77453216	C	T
NC_040257.1	77488927	A	T
NC_040257.1	77502488	G	A
NC_040257.1	77548831	A	G
NC_040257.1	77555135	A	G
NC_040257.1	77589333	A	G
NC_040257.1	77642494	G	A
NC_040257.1	77667994	C	T
NC_040257.1	77674775	A	G
NC_040257.1	77715430	C	T
NC_040257.1	77732216	G	A
NC_040257.1	77787314	T	C
NC_040257.1	77826408	C	T
NC_040257.1	77947531	G	A
NC_040257.1	78024031	A	G
NC_040257.1	78076995	A	G
NC_040257.1	78134553	G	A
NC_040257.1	78156444	C	T
NC_040257.1	78349385	A	G
NC_040257.1	78387682	A	G
NC_040257.1	78461816	G	A
NC_040257.1	78594871	G	A
NC_040257.1	78636795	T	G
NC_040257.1	78661671	G	C
NC_040257.1	78695328	G	A
NC_040257.1	78716784	G	T
NC_040257.1	78833321	T	G
NC_040257.1	78863713	T	C
NC_040257.1	78891742	G	C
NC_040257.1	78915958	A	C
NC_040257.1	78952522	A	C
NC_040257.1	78981015	T	G
NC_040257.1	79002090	A	G
NC_040257.1	79009950	G	C

NC_040257.1	79083150	G	C
NC_040257.1	79112387	A	G
NC_040257.1	79187943	C	T
NC_040257.1	79230343	T	C
NC_040257.1	79255263	T	C
NC_040257.1	79319781	G	T
NC_040257.1	79343692	G	A
NC_040257.1	79372507	C	T
NC_040257.1	79399517	A	G
NC_040257.1	79413980	T	C
NC_040257.1	79453186	T	C
NC_040257.1	79472376	C	T
NC_040257.1	79497962	C	T
NC_040257.1	79534564	T	C
NC_040257.1	79590590	A	G
NC_040257.1	79642206	A	G
NC_040257.1	79694356	C	T
NC_040257.1	79772255	C	T
NC_040257.1	79883901	T	C
NC_040257.1	79949987	C	A
NC_040257.1	79974728	T	C
NC_040257.1	79990170	G	A
NC_040257.1	80031196	T	G
NC_040257.1	80045492	T	C
NC_040257.1	80104435	G	A
NC_040257.1	80150844	C	A
NC_040257.1	80157861	A	G
NC_040257.1	80180720	C	T
NC_040257.1	80197584	G	T
NC_040257.1	80229655	T	G
NC_040257.1	80248541	G	A
NC_040257.1	80274284	A	G
NC_040257.1	80296576	C	A
NC_040257.1	80328983	A	G
NC_040257.1	80392290	T	C
NC_040257.1	80429231	A	T
NC_040257.1	80472546	G	A
NC_040257.1	80495600	T	C
NC_040257.1	80518463	G	C
NC_040257.1	80587354	A	G
NC_040257.1	80608372	G	A
NC_040257.1	80634954	A	T
NC_040257.1	80717714	C	T
NC_040257.1	80841451	A	G
NC_040257.1	80903033	T	C
NC_040257.1	80939082	G	A
NC_040257.1	81024773	T	G

NC_040257.1	81079158	A	G
NC_040257.1	81103515	C	T
NC_040257.1	81173951	T	A
NC_040257.1	81313452	A	C
NC_040257.1	81514534	A	G
NC_040257.1	81675806	G	A
NC_040257.1	81789071	A	T
NC_040257.1	81859372	C	T
NC_040257.1	82048785	T	A
NC_040257.1	82285496	C	G
NC_040257.1	82455025	C	T
NC_040257.1	82483368	C	T
NC_040257.1	82532296	T	G
NC_040257.1	82637152	G	A
NC_040257.1	82676244	T	C
NC_040257.1	82681642	G	A
NC_040257.1	82723928	T	C
NC_040257.1	82808960	G	A
NC_040257.1	82886241	A	G
NC_040257.1	82980311	A	G
NC_040257.1	83005033	C	T
NC_040257.1	83058247	G	A
NC_040257.1	83155905	G	A
NC_040257.1	83187016	G	A
NC_040257.1	83260657	G	T
NC_040257.1	83283510	A	G
NC_040257.1	83356243	C	T
NC_040257.1	83414204	G	A
NC_040257.1	83446972	G	A
NC_040257.1	83479549	T	C
NC_040257.1	83699033	G	C
NC_040257.1	83873053	A	G
NC_040257.1	83965315	C	T
NC_040257.1	84015248	T	C
NC_040257.1	84075447	A	G
NC_040257.1	84178274	C	T
NC_040257.1	84202963	T	A
NC_040257.1	84206063	T	C
NC_040257.1	84261599	G	A
NC_040257.1	84305381	A	G
NC_040257.1	84394325	G	A
NC_040257.1	84432476	T	A
NC_040257.1	84441179	C	G
NC_040257.1	84497250	A	G
NC_040257.1	84532927	C	A
NC_040257.1	84545745	T	A
NC_040257.1	84595076	G	T

NC_040257.1	84623444	G	A
NC_040257.1	84678281	C	T
NC_040257.1	84736334	G	C
NC_040257.1	84756992	G	A
NC_040257.1	84802049	T	C
NC_040257.1	84852855	G	C
NC_040257.1	84918271	A	G
NC_040257.1	84962276	G	A
NC_040257.1	84972608	A	G
NC_040257.1	85025537	A	G
NC_040257.1	85085529	C	A
NC_040257.1	85148677	C	A
NC_040257.1	85180600	A	G
NC_040257.1	85205762	T	C
NC_040257.1	85246239	G	T
NC_040257.1	85271043	A	C
NC_040257.1	85298677	C	T
NC_040257.1	85321383	T	C
NC_040257.1	85356089	T	C
NC_040257.1	85374831	G	C
NC_040257.1	85444811	T	C
NC_040257.1	85504044	A	G
NC_040257.1	85545607	G	A
NC_040257.1	85558316	C	T
NC_040257.1	85562658	G	A
NC_040257.1	85616610	T	C
NC_040257.1	85664948	T	G
NC_040257.1	85731708	A	G
NC_040257.1	85740299	C	T
NC_040257.1	85865331	G	A
NC_040257.1	85954671	C	A
NC_040257.1	86026614	A	G
NC_040257.1	86109526	C	T
NC_040257.1	86162092	G	A
NC_040257.1	86194000	C	G
NC_040257.1	86313593	A	G
NC_040257.1	86350692	A	G
NC_040257.1	86380904	T	C
NC_040257.1	86540671	C	G
NC_040257.1	86601893	T	A
NC_040257.1	86638290	A	G
NC_040257.1	86852314	C	A
NC_040257.1	86911907	G	T
NC_040257.1	86973639	A	C
NC_040257.1	87037409	C	T
NC_040257.1	87179809	T	C
NC_040257.1	87202928	G	A

NC_040257.1	87215675	A	G
NC_040257.1	87344351	A	C
NC_040257.1	87378144	A	C
NC_040257.1	87401789	C	T
NC_040257.1	87462835	T	A
NC_040257.1	87519024	C	T
NC_040257.1	87546207	G	A
NC_040257.1	87559133	T	A
NC_040257.1	87695042	T	C
NC_040257.1	87770392	G	A
NC_040257.1	87824486	G	A
NC_040257.1	87850017	T	C
NC_040257.1	87891389	C	T
NC_040257.1	87905552	A	G
NC_040257.1	88046489	C	G
NC_040257.1	88108540	C	T
NC_040257.1	88165987	C	G
NC_040257.1	88221893	A	G
NC_040257.1	88263681	A	G
NC_040257.1	88335840	T	C
NC_040257.1	88403182	A	G
NC_040257.1	88435082	A	C
NC_040257.1	88451280	A	T
NC_040257.1	88482213	T	G
NC_040257.1	88556327	T	G
NC_040257.1	88595599	C	T
NC_040257.1	88730708	C	G
NC_040257.1	88767268	A	C
NC_040257.1	88826775	C	T
NC_040257.1	88856333	C	T
NC_040257.1	88883470	C	T
NC_040257.1	88934950	T	G
NC_040257.1	88977292	T	G
NC_040257.1	88983084	C	T
NC_040257.1	89012027	C	T
NC_040257.1	89048288	G	A
NC_040257.1	89301662	T	C
NC_040257.1	89362914	C	T
NC_040257.1	89438130	T	C
NC_040257.1	89506861	G	A
NC_040257.1	89611278	A	T
NC_040257.1	89644871	C	T
NC_040257.1	89686560	T	C
NC_040257.1	89728683	G	C
NC_040257.1	89735902	A	G
NC_040257.1	89967823	A	G
NC_040257.1	90000261	A	C

NC_040257.1	90082678	G	T
NC_040257.1	90113942	T	C
NC_040257.1	90161048	A	G
NC_040257.1	90164513	C	A
NC_040257.1	90230597	A	G
NC_040257.1	90496974	A	G
NC_040257.1	90529405	A	C
NC_040257.1	90607137	C	T
NC_040257.1	90655065	C	T
NC_040257.1	90700571	T	C
NC_040257.1	90795844	A	G
NC_040257.1	90860823	A	G
NC_040257.1	90904129	G	A
NC_040257.1	90924056	C	A
NC_040257.1	91198226	A	G
NC_040257.1	91230792	T	G
NC_040257.1	91296579	A	T
NC_040257.1	91396831	T	C
NC_040257.1	91453280	G	A
NC_040257.1	91583260	A	G
NC_040257.1	91645232	C	G
NC_040257.1	91694607	T	C
NC_040257.1	91724219	A	G
NC_040257.1	91739041	T	C
NC_040257.1	91790538	G	C
NC_040257.1	91848889	C	T
NC_040257.1	91900398	T	C
NC_040257.1	91962202	A	G
NC_040257.1	92201135	C	G
NC_040257.1	92245323	G	A
NC_040257.1	92260119	T	C
NC_040257.1	92299716	T	C
NC_040257.1	92384918	T	A
NC_040257.1	92405907	A	C
NC_040257.1	92433557	G	A
NC_040257.1	92442862	A	C
NC_040257.1	92468365	C	T
NC_040257.1	92494941	A	G
NC_040257.1	92535738	C	T
NC_040257.1	92565955	T	C
NC_040257.1	92598399	A	G
NC_040257.1	92660862	A	C
NC_040257.1	92717986	C	T
NC_040257.1	92757127	G	T
NC_040257.1	92787102	T	A
NC_040257.1	92819412	A	C
NC_040257.1	92847208	T	C

NC_040257.1	92908215	G	A
NC_040257.1	92963651	T	G
NC_040257.1	93028804	G	A
NC_040257.1	93063750	G	T
NC_040257.1	93083459	A	G
NC_040257.1	93125392	G	T
NC_040257.1	93166014	G	A
NC_040257.1	93192459	A	T
NC_040257.1	93219963	A	T
NC_040257.1	93255099	G	C
NC_040257.1	93317740	T	C
NC_040257.1	93321504	C	T
NC_040257.1	93348251	C	T
NC_040257.1	93526070	A	C
NC_040257.1	93569318	T	C
NC_040257.1	93603269	C	T
NC_040257.1	93994276	G	A
NC_040257.1	94159312	G	A
NC_040257.1	94162118	A	G
NC_040257.1	94190608	A	G
NC_040257.1	94402803	C	T
NC_040257.1	94429898	C	A
NC_040257.1	94452774	G	A
NC_040257.1	94541896	G	A
NC_040257.1	94599717	A	G
NC_040257.1	94634950	T	G
NC_040257.1	94672978	G	A
NC_040257.1	94690509	T	C
NC_040257.1	94742865	T	A
NC_040257.1	94771220	A	T
NC_040257.1	94825456	T	A
NC_040257.1	94833360	C	T
NC_040257.1	94993566	A	G
NC_040257.1	95070394	A	G
NC_040257.1	95098848	G	A
NC_040257.1	95124204	C	T
NC_040257.1	95182065	T	G
NC_040257.1	95204290	G	A
NC_040257.1	95234959	G	C
NC_040257.1	95279431	C	T
NC_040257.1	95281134	T	C
NC_040257.1	95330780	T	C
NC_040257.1	95335925	G	A
NC_040257.1	95374437	G	T
NC_040257.1	95402065	A	G
NC_040257.1	95460661	T	C
NC_040257.1	95511064	G	C

NC_040257.1	95559488	T	C
NC_040257.1	95619447	G	T
NC_040257.1	95670357	C	T
NC_040257.1	95704181	C	T
NC_040257.1	95727794	C	A
NC_040257.1	95762160	A	G
NC_040257.1	95905973	A	G
NC_040257.1	96115248	A	G
NC_040257.1	96165370	T	C
NC_040257.1	96195005	G	A
NC_040257.1	96217535	T	C
NC_040257.1	96284137	A	G
NC_040257.1	96334696	A	T
NC_040257.1	96366436	G	T
NC_040257.1	96383388	A	G
NC_040257.1	96625295	T	C
NC_040257.1	96658077	T	C
NC_040257.1	96662497	T	C
NC_040257.1	96756967	T	C
NC_040257.1	97127628	C	G
NC_040257.1	97185828	T	G
NC_040257.1	97268298	A	T
NC_040257.1	97300048	T	A
NC_040257.1	97325411	T	C
NC_040257.1	97369390	T	G
NC_040257.1	97431644	C	T
NC_040257.1	97479009	G	A
NC_040257.1	97511113	G	A
NC_040257.1	97565377	T	C
NC_040257.1	97618011	C	A
NC_040257.1	97650175	A	G
NC_040257.1	97664088	G	A
NC_040257.1	97692825	C	T
NC_040257.1	97737317	C	T
NC_040257.1	97788378	G	T
NC_040257.1	97814459	G	A
NC_040257.1	97846995	A	T
NC_040257.1	97886267	T	G
NC_040257.1	97927349	T	A
NC_040257.1	97987501	G	A
NC_040257.1	98050232	A	G
NC_040257.1	98074013	A	G
NC_040257.1	98078849	A	G
NC_040257.1	98112976	C	T
NC_040257.1	98143747	A	G
NC_040257.1	98178676	T	G
NC_040257.1	98195822	G	C

NC_040257.1	98244837	G	A
NC_040257.1	98286299	T	A
NC_040257.1	98298427	G	A
NC_040257.1	98334567	A	G
NC_040257.1	98348145	G	T
NC_040257.1	98350000	G	A
NC_040257.1	98384896	T	C
NC_040257.1	98429214	C	T
NC_040257.1	98473628	T	C
NC_040257.1	98503887	C	T
NC_040257.1	98524661	A	C
NC_040257.1	98554018	A	C
NC_040257.1	98617770	G	C
NC_040257.1	98639160	C	T
NC_040257.1	98670422	T	A
NC_040257.1	98709900	G	A
NC_040257.1	98725509	C	T
NC_040257.1	98795158	C	T
NC_040257.1	98842152	G	A
NC_040257.1	98860391	T	C
NC_040257.1	98916226	C	T
NC_040257.1	98925489	C	T
NC_040257.1	98947161	G	A
NC_040257.1	98999079	C	T
NC_040257.1	99039095	T	C
NC_040257.1	99061524	T	C
NC_040257.1	99114860	G	A
NC_040257.1	99174685	C	T
NC_040257.1	99211470	T	C
NC_040257.1	99233319	A	G
NC_040257.1	99255673	C	G
NC_040257.1	99276863	T	C
NC_040257.1	99281650	T	C
NC_040257.1	99325470	A	G
NC_040257.1	99360787	T	C
NC_040257.1	99398596	T	C
NC_040257.1	99421085	A	G
NC_040257.1	99463431	G	C
NC_040257.1	99507782	T	G
NC_040257.1	99543234	A	G
NC_040257.1	99557627	C	G
NC_040257.1	99587767	A	C
NC_040257.1	99596557	C	T
NC_040257.1	99679641	T	C
NC_040257.1	99712593	T	C
NC_040257.1	99767272	G	C
NC_040257.1	99800949	G	A

NC_040257.1	99841094	A	G
NC_040257.1	99863290	T	A
NC_040257.1	99900379	C	T
NC_040257.1	99980053	A	G
NC_040257.1	100020718	T	C
NC_040257.1	100037328	T	C
NC_040257.1	100097795	T	C
NC_040257.1	100137219	G	A
NC_040257.1	100151375	G	A
NC_040257.1	100185900	T	G
NC_040257.1	100219159	C	G
NC_040257.1	100259796	G	T
NC_040257.1	100281348	G	A
NC_040257.1	100338109	C	T
NC_040257.1	100351906	T	C
NC_040257.1	100386897	C	A
NC_040257.1	100412505	C	T
NC_040257.1	100445539	A	G
NC_040257.1	100479543	G	A
NC_040257.1	100513340	G	A
NC_040257.1	100551194	C	T
NC_040257.1	100571231	T	A
NC_040257.1	100613025	C	T
NC_040257.1	100658794	C	G
NC_040257.1	100671876	T	C
NC_040257.1	100686503	A	G
NC_040257.1	100716777	G	A
NC_040257.1	100767571	A	C
NC_040257.1	100803447	G	T
NC_040257.1	100818250	G	T
NC_040257.1	100841235	A	G
NC_040257.1	100865948	G	A
NC_040257.1	100880090	T	C
NC_040257.1	100942202	A	G
NC_040257.1	100994150	A	G
NC_040257.1	101024662	T	A
NC_040257.1	101027495	T	C
NC_040257.1	101080565	G	A
NC_040257.1	101101645	T	C
NC_040257.1	101160800	T	C
NC_040257.1	101220053	A	G
NC_040257.1	101258496	C	T
NC_040257.1	101292312	C	G
NC_040257.1	101314642	G	A
NC_040257.1	101348942	T	C
NC_040257.1	101370663	G	A
NC_040257.1	101420679	A	T

NC_040257.1	101426865	G	A
NC_040257.1	101462316	G	C
NC_040257.1	101491327	C	T
NC_040257.1	101533142	C	T
NC_040257.1	101553186	T	A
NC_040257.1	101582920	C	T
NC_040257.1	101606190	T	C
NC_040257.1	101637785	T	C
NC_040257.1	101655014	G	A
NC_040257.1	101708183	T	C
NC_040257.1	101765222	C	T
NC_040257.1	101821762	T	C
NC_040257.1	101862739	T	C
NC_040257.1	101895076	A	G
NC_040257.1	101911960	G	A
NC_040257.1	101955311	A	G
NC_040257.1	101970330	G	A
NC_040257.1	102020811	G	T
NC_040257.1	102029704	C	T
NC_040257.1	102078475	C	A
NC_040257.1	102083970	A	G
NC_040257.1	102130762	C	T
NC_040257.1	102137491	C	T
NC_040257.1	102173003	T	C
NC_040257.1	102208554	G	A
NC_040257.1	102247737	G	C
NC_040257.1	102281803	A	G
NC_040257.1	102293990	C	T
NC_040257.1	102317350	A	C
NC_040257.1	102346704	G	A
NC_040257.1	102412722	G	T
NC_040257.1	102434159	T	A
NC_040257.1	102467709	T	C
NC_040257.1	102518263	C	T
NC_040257.1	102615457	G	C
NC_040257.1	102677877	G	A
NC_040257.1	102714348	A	T
NC_040257.1	102754837	A	G
NC_040257.1	102839984	C	A
NC_040257.1	103032102	C	G
NC_040257.1	103076783	G	A
NC_040257.1	103170565	G	A
NC_040257.1	103217232	T	C
NC_040257.1	103254236	A	G
NC_040257.1	103320773	T	G
NC_040257.1	103371253	A	G
NC_040257.1	103397035	T	C

NC_040257.1	103428550	G	A
NC_040257.1	103473443	A	T
NC_040257.1	103483304	T	C
NC_040257.1	103533742	A	G
NC_040257.1	103535129	A	G
NC_040257.1	103589981	A	G
NC_040257.1	103622315	G	A
NC_040257.1	103659563	C	G
NC_040257.1	103664708	A	G
NC_040257.1	103763686	A	G
NC_040257.1	103806604	T	C
NC_040257.1	103846927	T	G
NC_040257.1	103895186	A	G
NC_040257.1	103958142	T	C
NC_040257.1	104009833	T	A
NC_040257.1	104083284	C	T
NC_040257.1	104106481	C	T
NC_040257.1	104137401	G	A
NC_040257.1	104181497	G	A
NC_040257.1	104196449	T	A
NC_040257.1	104222668	A	C
NC_040257.1	104254544	T	C
NC_040257.1	104279689	T	A
NC_040257.1	104324756	G	T
NC_040257.1	104384691	G	A
NC_040257.1	104425931	G	A
NC_040257.1	104466669	G	A
NC_040257.1	104470810	G	C
NC_040257.1	104629356	T	A
NC_040257.1	104649210	T	A
NC_040257.1	104659093	T	C
NC_040257.1	104714694	G	A
NC_040257.1	104745971	T	C
NC_040257.1	104759062	C	T
NC_040257.1	104790908	A	G
NC_040257.1	104823054	T	G
NC_040257.1	104862255	A	G
NC_040257.1	104881027	T	G
NC_040257.1	104955159	G	C
NC_040257.1	105021935	C	G
NC_040257.1	105055714	A	G
NC_040257.1	105090020	A	G
NC_040257.1	105166578	A	T
NC_040257.1	105189188	T	C
NC_040257.1	105270668	T	C
NC_040257.1	105394372	G	A
NC_040257.1	105416464	G	A

NC_040257.1	105528328	G	A
NC_040257.1	105566119	C	T
NC_040257.1	105587064	T	G
NC_040257.1	105630079	C	A
NC_040257.1	105683461	C	T
NC_040257.1	105721018	G	A
NC_040257.1	105754058	T	C
NC_040257.1	105795473	G	A
NC_040257.1	105811718	A	T
NC_040257.1	105840570	T	C
NC_040257.1	105853030	C	G
NC_040257.1	105926442	T	C
NC_040257.1	105957440	A	C
NC_040257.1	106148263	G	T
NC_040257.1	106195338	A	T
NC_040257.1	106204048	T	C
NC_040257.1	106241392	A	G
NC_040257.1	106252290	A	C
NC_040257.1	106277752	C	G
NC_040257.1	106291502	A	G
NC_040257.1	106364912	G	A
NC_040257.1	106420769	T	C
NC_040257.1	106455219	C	A
NC_040257.1	106475285	C	G
NC_040257.1	106533966	T	G
NC_040257.1	106558879	C	T
NC_040257.1	106570793	A	G
NC_040257.1	106606684	T	C
NC_040257.1	106625761	T	C
NC_040257.1	106654082	A	G
NC_040257.1	106681144	A	C
NC_040257.1	106710380	C	T
NC_040257.1	106771925	T	C
NC_040257.1	106814331	T	C
NC_040257.1	106839265	C	T
NC_040257.1	106859995	A	G
NC_040257.1	107069364	C	T
NC_040257.1	107114903	T	C
NC_040257.1	107119691	G	A
NC_040257.1	107174834	C	T
NC_040257.1	107215982	C	T
NC_040257.1	107224029	T	C
NC_040257.1	107252171	T	C
NC_040257.1	107274793	C	T
NC_040257.1	107292536	A	G
NC_040257.1	107348863	G	A
NC_040257.1	107372581	G	A

NC_040257.1	107403097	A	G
NC_040257.1	107441924	G	A
NC_040257.1	107510311	T	C
NC_040257.1	107534705	C	G
NC_040257.1	107571272	C	A
NC_040257.1	107605456	T	C
NC_040257.1	107682563	C	T
NC_040257.1	107731670	C	T
NC_040257.1	107774798	G	T
NC_040257.1	107810500	G	C
NC_040257.1	107825575	A	G
NC_040257.1	107880916	G	T
NC_040257.1	107910956	T	C
NC_040257.1	107968704	A	G
NC_040257.1	107983665	A	G
NC_040257.1	108011350	T	A
NC_040257.1	108054598	C	T
NC_040257.1	108057509	G	A
NC_040257.1	108060152	G	A
NC_040257.1	108096884	G	C
NC_040257.1	108150658	A	G
NC_040257.1	108209964	C	T
NC_040257.1	108263492	G	A
NC_040257.1	108298401	T	C
NC_040257.1	108331821	C	A
NC_040257.1	108345997	T	C
NC_040257.1	108403340	C	T
NC_040257.1	108408807	A	G
NC_040257.1	108461750	A	G
NC_040257.1	108494952	G	A
NC_040257.1	108511810	G	A
NC_040257.1	108536452	T	A
NC_040257.1	108575986	G	T
NC_040257.1	108602887	G	A
NC_040257.1	108623267	A	G
NC_040257.1	108630760	G	A
NC_040257.1	108655779	G	A
NC_040257.1	108680960	C	T
NC_040257.1	108688359	T	C
NC_040257.1	108714040	C	T
NC_040257.1	108745896	A	G
NC_040257.1	108775006	A	G
NC_040257.1	108801289	C	T
NC_040257.1	108835239	C	T
NC_040257.1	108851441	T	G
NC_040257.1	108873085	A	T
NC_040257.1	108905219	T	C

NC_040257.1	108945973	T	C
NC_040257.1	108995005	T	C
NC_040257.1	109018058	T	C
NC_040257.1	109039820	C	T
NC_040257.1	109070878	A	G
NC_040257.1	109145604	T	C
NC_040257.1	109177712	T	C
NC_040257.1	109190283	C	T
NC_040257.1	109224516	T	G
NC_040257.1	109270614	G	C
NC_040257.1	109276580	T	C
NC_040257.1	109328204	A	G
NC_040257.1	109357744	A	G
NC_040257.1	109383733	T	C
NC_040257.1	109434782	G	T
NC_040257.1	109489751	C	T
NC_040257.1	109526252	C	T
NC_040257.1	109573617	A	G
NC_040257.1	109655859	T	C
NC_040257.1	109716422	T	C
NC_040257.1	109775582	C	G
NC_040257.1	109776256	T	C
NC_040257.1	109793458	A	C
NC_040257.1	109798787	T	C
NC_040257.1	109840293	A	G
NC_040257.1	109887509	A	C
NC_040257.1	109925042	T	C
NC_040257.1	109933967	G	A
NC_040257.1	109953280	G	A
NC_040257.1	110009060	C	T
NC_040257.1	110067237	T	G
NC_040257.1	110121139	A	G
NC_040257.1	110156494	A	G
NC_040257.1	110179440	G	A
NC_040257.1	110214863	C	T
NC_040257.1	110235242	A	T
NC_040257.1	110292900	T	C
NC_040257.1	110365141	T	C
NC_040257.1	110391079	A	G
NC_040257.1	110440071	C	T
NC_040257.1	110504704	A	G
NC_040257.1	110553970	T	C
NC_040257.1	110576927	A	G
NC_040257.1	110605234	A	T
NC_040257.1	110626088	G	C
NC_040257.1	110670950	T	C
NC_040257.1	110691099	G	T

NC_040257.1	110734350		0	G
NC_040257.1	110842060	A		G
NC_040257.1	110886851	T		C
NC_040257.1	110893441	T		C
NC_040257.1	110935961	T		C
NC_040257.1	110941298	A		C
NC_040257.1	110968853	G		A
NC_040257.1	111001284	A		G
NC_040257.1	111046800	G		A
NC_040257.1	111070401	A		G
NC_040257.1	111097771	A		G
NC_040257.1	111136110	C		T
NC_040257.1	111143341	T		G
NC_040257.1	111162405	A		G
NC_040257.1	111215135	G		T
NC_040257.1	111285586	C		T
NC_040257.1	111364005	C		T
NC_040257.1	111421851	A		G
NC_040257.1	111477309	C		T
NC_040257.1	111540818	A		G
NC_040257.1	111587477	C		A
NC_040257.1	111599546	C		T
NC_040257.1	111657084	C		A
NC_040257.1	111721192	T		G
NC_040257.1	111775901	G		A
NC_040257.1	111825030	G		A
NC_040257.1	111864481	T		G
NC_040257.1	111881957	C		T
NC_040257.1	111942158	G		A
NC_040257.1	111961028	C		A
NC_040257.1	111992943	T		C
NC_040257.1	112015156	G		A
NC_040257.1	112044008	C		T
NC_040257.1	112048364	C		T
NC_040257.1	112101228	T		C
NC_040257.1	112154261	G		T
NC_040257.1	112201619	C		T
NC_040257.1	112209954	G		C
NC_040257.1	112271527	G		A
NC_040257.1	112317942	T		C
NC_040257.1	112359922	T		C
NC_040257.1	112362071	G		A
NC_040257.1	112376839	A		G
NC_040257.1	112411812	A		G
NC_040257.1	112473163	A		C
NC_040257.1	112516928	T		C
NC_040257.1	112546871	A		T

NC_040257.1	112585221	T	G
NC_040257.1	112705955	A	G
NC_040257.1	112737162	G	T
NC_040257.1	112740547	G	A
NC_040257.1	112790008	C	T
NC_040257.1	112841992	G	T
NC_040257.1	112888137	G	A
NC_040257.1	112911652	T	C
NC_040257.1	112912901	C	T
NC_040257.1	112975251	A	G
NC_040257.1	113018581	T	C
NC_040257.1	113068598	T	C
NC_040257.1	113126248	T	C
NC_040257.1	113158081	A	G
NC_040257.1	113163186	C	T
NC_040257.1	113196128	A	G
NC_040257.1	113217297	A	G
NC_040257.1	113272835	A	G
NC_040257.1	113326402	T	C
NC_040257.1	113385324	C	T
NC_040257.1	113439695	G	A
NC_040257.1	113482857	C	G
NC_040257.1	113483031	T	C
NC_040257.1	113546564	T	C
NC_040257.1	113567308	0	C
NC_040257.1	113609396	T	C
NC_040257.1	113611873	T	A
NC_040257.1	113646475	C	T
NC_040257.1	113663864	G	C
NC_040257.1	113701849	A	G
NC_040257.1	113753801	T	C
NC_040257.1	113805120	T	G
NC_040257.1	113859785	T	C
NC_040257.1	113908661	T	A
NC_040257.1	113914301	T	A
NC_040257.1	113957016	G	A
NC_040257.1	113967784	A	T
NC_040257.1	114011198	G	C
NC_040257.1	114033911	T	C
NC_040257.1	114057644	C	T
NC_040257.1	114087562	A	G
NC_040257.1	114136637	T	G
NC_040257.1	114175753	A	G
NC_040257.1	114192192	T	C
NC_040257.1	114247578	T	C
NC_040257.1	114305575	A	G
NC_040257.1	114361005	A	G

NC_040257.1	114422813	G	T
NC_040257.1	114447577	G	A
NC_040257.1	114488467	T	C
NC_040257.1	114513804	C	T
NC_040257.1	114536199	G	T
NC_040257.1	114566207	A	G
NC_040257.1	114577235	A	G
NC_040257.1	114611268	A	C
NC_040257.1	114611544	C	A
NC_040257.1	114661232	C	T
NC_040257.1	114715419	A	C
NC_040257.1	114771584	A	T
NC_040257.1	114836096	A	C
NC_040257.1	115011930	T	G
NC_040257.1	115045129	A	G
NC_040257.1	115057085	A	G
NC_040257.1	115094018	A	G
NC_040257.1	115111913	A	G
NC_040257.1	115154567	G	T
NC_040257.1	115188675	G	A
NC_040257.1	115190240	T	C
NC_040257.1	115245477	C	G
NC_040257.1	115294144	T	C
NC_040257.1	115311790	T	C
NC_040257.1	115350424	C	T
NC_040257.1	115380380	A	C
NC_040257.1	115397050	T	C
NC_040257.1	115401913	T	C
NC_040257.1	115445731	C	T
NC_040257.1	115446611	G	A
NC_040257.1	115481361	G	T
NC_040257.1	115503584	T	C
NC_040257.1	115561444	A	G
NC_040257.1	115622019	G	C
NC_040257.1	115675273	A	G
NC_040257.1	115737839	T	C
NC_040257.1	115792760	T	C
NC_040257.1	115846163	G	A
NC_040257.1	115905668	G	A
NC_040257.1	115945595	C	T
NC_040257.1	115958833	G	A
NC_040257.1	115959306	C	T
NC_040257.1	115970744	0	T
NC_040257.1	115971323	C	G
NC_040257.1	116023737	T	C
NC_040257.1	116083341	A	T
NC_040257.1	116126416	C	T

NC_040257.1	116149818	G	A
NC_040257.1	116182007	G	A
NC_040257.1	116202226	G	C
NC_040257.1	116232313	A	G
NC_040257.1	116256241	A	C
NC_040257.1	116328637	A	G
NC_040257.1	116382867	A	G
NC_040257.1	116415771	C	T
NC_040257.1	116570781	G	C
NC_040257.1	116615958	G	T
NC_040257.1	116685897	C	A
NC_040257.1	116689446	T	C
NC_040257.1	116862541	T	A
NC_040257.1	116922258	A	G
NC_040257.1	116978297	C	A
NC_040257.1	117007181	A	G
NC_040257.1	117076697	G	C
NC_040257.1	117278041	A	G
NC_040257.1	117327229	C	G
NC_040257.1	117377815	T	C
NC_040257.1	117380186	G	A
NC_040257.1	117380599	A	G
NC_040257.1	117381323	A	G
NC_040257.1	117415396	T	G
NC_040257.1	117415566	G	C
NC_040257.1	117470450	G	A
NC_040257.1	117530939	G	T
NC_040257.1	117627021	G	A
NC_040257.1	117695166	C	T
NC_040257.1	117749655	C	T
NC_040257.1	117791892	A	G
NC_040257.1	117804421	G	A
NC_040257.1	117850633	G	A
NC_040257.1	117880029	G	A
NC_040257.1	117901345	A	G
NC_040257.1	117935866	T	C
NC_040257.1	117959071	C	A
NC_040257.1	118012585	G	A
NC_040257.1	118064094	C	T
NC_040257.1	118067653	C	A
NC_040257.1	118117865	C	T
NC_040257.1	118153431	A	G
NC_040257.1	118303035	C	T
NC_040257.1	118346784	T	A
NC_040257.1	118379556	A	T
NC_040257.1	118423431	G	A
NC_040257.1	118441201	C	T

NC_040257.1	118499640	A	T
NC_040257.1	118555600	A	G
NC_040257.1	118584115	0	T
NC_040257.1	118607250	A	C
NC_040257.1	118667240	C	T
NC_040257.1	118694571	G	C
NC_040257.1	118707133	C	T
NC_040257.1	118738002	T	C
NC_040257.1	118763092	G	C
NC_040257.1	118840349	T	C
NC_040257.1	118903994	G	A
NC_040257.1	118963617	C	A
NC_040257.1	119021351	T	C
NC_040257.1	119046652	C	G
NC_040257.1	119063899	A	G
NC_040257.1	119090259	G	T
NC_040257.1	119117406	T	C
NC_040257.1	119168636	G	A
NC_040257.1	119208297	T	C
NC_040257.1	119340996	T	C
NC_040257.1	119364101	C	A
NC_040257.1	119386307	G	A
NC_040257.1	119414837	T	G
NC_040257.1	119441563	C	G
NC_040257.1	119498747	A	G
NC_040257.1	119554056	G	A
NC_040257.1	119589132	T	C
NC_040257.1	119617847	T	C
NC_040257.1	119636579	A	T
NC_040257.1	119695283	G	A
NC_040257.1	119750814	C	A
NC_040257.1	119804889	A	G
NC_040257.1	119859924	C	T
NC_040257.1	119884854	C	G
NC_040257.1	119885168	A	G
NC_040257.1	119935503	C	A
NC_040257.1	119935966	T	C
NC_040257.1	119936798	C	G
NC_040257.1	119937015	A	G
NC_040257.1	119939125	T	C
NC_040257.1	119939377	T	C
NC_040257.1	119940468	A	G
NC_040257.1	120411087	G	A
NC_040257.1	120466945	A	G
NC_040257.1	120512340	A	G
NC_040257.1	120537292	G	T
NC_040257.1	120555770	T	C

NC_040257.1	120585407	A	G
NC_040257.1	120605916	T	C
NC_040257.1	120660809	C	T
NC_040257.1	120705071	A	G
NC_040257.1	120725250	G	A
NC_040257.1	120770496	G	A
NC_040257.1	120834113	C	T
NC_040257.1	120894848	T	C
NC_040257.1	120945439	C	T
NC_040257.1	120947880	A	G
NC_040257.1	121003919	T	C
NC_040257.1	121030334	A	G
NC_040257.1	121044626	T	C
NC_040257.1	121076069	A	G
NC_040257.1	121095648	A	C
NC_040257.1	121149401	T	C
NC_040257.1	121170287	T	A
NC_040257.1	121180947	C	T
NC_040257.1	121276194	C	T
NC_040257.1	121337351	C	T
NC_040257.1	121384659	T	C
NC_040257.1	121392421	A	G
NC_040257.1	121437447	A	G
NC_040257.1	121486019	A	C
NC_040257.1	121496780	C	T
NC_040257.1	121549644	T	A
NC_040257.1	121599471	C	T
NC_040257.1	121657761	T	C
NC_040257.1	121722934	C	T
NC_040257.1	121777991	G	A
NC_040257.1	121802107	G	A
NC_040257.1	121854606	T	C
NC_040257.1	121911947	G	C
NC_040257.1	121952160	A	G
NC_040257.1	121954877	C	G
NC_040257.1	121980268	T	C
NC_040257.1	122002697	G	T
NC_040257.1	122011828	T	C
NC_040257.1	122066257	T	C
NC_040257.1	122098139	C	T
NC_040257.1	122121802	T	G
NC_040257.1	122172947	C	T
NC_040257.1	122173108	A	G
NC_040257.1	122181136	A	G
NC_040257.1	122188886	A	G
NC_040257.1	122189897	T	C
NC_040257.1	122258561	T	C

NC_040257.1	122288134	T	G
NC_040257.1	122324930	C	T
NC_040257.1	122487180	T	A
NC_040257.1	122631318	T	C
NC_040257.1	122680201	C	T
NC_040257.1	122724138	A	G
NC_040257.1	122787715	A	G
NC_040257.1	122803342	A	G
NC_040257.1	122848424	T	C
NC_040257.1	122892953	A	G
NC_040257.1	123068649	G	A
NC_040257.1	123115035	T	C
NC_040257.1	123138511	T	C
NC_040257.1	123175013	T	C
NC_040257.1	123216392	T	G
NC_040257.1	123275657	G	A
NC_040257.1	123314523	C	T
NC_040257.1	123354741	C	T
NC_040257.1	123411896	T	C
NC_040257.1	123447442	G	T
NC_040257.1	123454889	T	C
NC_040257.1	123494604	A	T
NC_040257.1	123503905	T	G
NC_040257.1	123543906	G	A
NC_040257.1	123545524	A	C
NC_040257.1	123614403	C	T
NC_040257.1	123640958	T	C
NC_040257.1	123687994	T	A
NC_040257.1	123690181	C	T
NC_040257.1	123727429	T	C
NC_040257.1	123743330	T	C
NC_040257.1	123799401	T	C
NC_040257.1	123833722	C	T
NC_040257.1	123857590	G	A
NC_040257.1	123907390	T	C
NC_040257.1	123966154	T	C
NC_040257.1	123998114	A	G
NC_040257.1	124022865	T	C
NC_040257.1	124074886	A	G
NC_040257.1	124130700	G	A
NC_040257.1	124187128	A	G
NC_040257.1	124232073	G	A
NC_040257.1	124420402	C	T
NC_040257.1	124469374	G	C
NC_040257.1	124517737	G	A
NC_040257.1	124521784	A	G
NC_040257.1	124558249	A	T

NC_040257.1	124577507	C	T
NC_040257.1	124630909	T	C
NC_040257.1	124633189	G	T
NC_040257.1	124655041	C	G
NC_040257.1	124674791	T	C
NC_040257.1	124695386	C	G
NC_040257.1	124728458	C	G
NC_040257.1	124778569	A	G
NC_040257.1	124824088	T	C
NC_040257.1	124885104	A	G
NC_040257.1	124917513	G	C
NC_040257.1	124949406	G	A
NC_040257.1	125005835	A	G
NC_040257.1	125040963	G	T
NC_040257.1	125100971	A	G
NC_040257.1	125150708	G	C
NC_040257.1	125204334	A	G
NC_040257.1	125230256	A	G
NC_040257.1	125284990	A	G
NC_040257.1	125338532	T	C
NC_040257.1	125364446	A	G
NC_040257.1	125371557	G	A
NC_040257.1	125372819	G	C
NC_040257.1	125373105	G	A
NC_040257.1	125386576	T	G
NC_040257.1	125387528	A	G
NC_040257.1	125388142	A	G
NC_040257.1	125419466	T	G
NC_040257.1	125502645	A	G
NC_040257.1	125529016	G	C
NC_040257.1	125543472	T	C
NC_040257.1	125543819	G	A
NC_040257.1	125544441	T	C
NC_040257.1	125588336	C	T
NC_040257.1	125595297	G	C
NC_040257.1	125622345	T	C
NC_040257.1	125643763	T	C
NC_040257.1	125690694	G	C
NC_040257.1	125706796	T	C
NC_040257.1	125760757	A	G
NC_040257.1	125763719	G	A
NC_040257.1	125803001	A	G
NC_040257.1	125829647	T	G
NC_040257.1	125852094	A	G
NC_040257.1	125855201	C	G
NC_040257.1	125889753	A	G
NC_040257.1	125924679	C	T

NC_040257.1	125932368	G	T
NC_040257.1	125956148	A	C
NC_040257.1	125999861	T	C
NC_040257.1	126057553	A	C
NC_040257.1	126116213	T	G
NC_040257.1	126130727	C	A
NC_040257.1	126131393	A	G
NC_040257.1	126131727	G	A
NC_040257.1	126131895	A	G
NC_040257.1	126132188	T	C
NC_040257.1	126186890	T	C
NC_040257.1	126242355	A	G
NC_040257.1	126292986	T	C
NC_040257.1	126351056	T	C
NC_040257.1	126390863	T	C
NC_040257.1	126408338	G	A
NC_040257.1	126451919	T	G
NC_040257.1	126460239	T	C
NC_040257.1	126485276	T	C
NC_040257.1	126524293	A	G
NC_040257.1	126560986	G	C
NC_040257.1	126561678	T	C
NC_040257.1	126594473	G	T
NC_040257.1	126623702	G	T
NC_040257.1	126662240	C	T
NC_040257.1	126711513	A	G
NC_040257.1	126718483	C	T
NC_040257.1	126768719	C	T
NC_040257.1	126832052	G	A
NC_040257.1	126883011	A	G
NC_040257.1	126922649	A	G
NC_040257.1	126938079	A	G
NC_040257.1	126992916	A	T
NC_040257.1	127048467	G	A
NC_040257.1	127098394	C	T
NC_040257.1	127159825	T	C
NC_040257.1	127214845	T	C
NC_040257.1	127239809	T	C
NC_040257.1	127252576	0	G
NC_040257.1	127291217	C	T
NC_040257.1	127305416	T	C
NC_040257.1	127332182	C	T
NC_040257.1	127365969	G	A
NC_040257.1	127415842	C	A
NC_040257.1	127419058	T	C
NC_040257.1	127440761	T	C
NC_040257.1	127479455	G	C

NC_040257.1	127480464	T		C
NC_040257.1	127489368	C		G
NC_040257.1	127538803	T		C
NC_040257.1	127559830	T		G
NC_040257.1	127575380	A		G
NC_040257.1	127575610	A		C
NC_040257.1	127576916	A		G
NC_040257.1	127643034	T		C
NC_040257.1	127644677	T		C
NC_040257.1	127658762	T		C
NC_040257.1	127674050	T		C
NC_040257.1	127679144	G		A
NC_040257.1	127705649	A		G
NC_040257.1	127728809	C		T
NC_040257.1	127790594	A		G
NC_040257.1	127790836	A		G
NC_040257.1	127857958	T		C
NC_040257.1	127884130	A		C
NC_040257.1	127909033	A		G
NC_040257.1	127954945	A		G
NC_040257.1	127963223	T		C
NC_040257.1	127965124	C		T
NC_040257.1	128023335	C		T
NC_040257.1	128077106	T		C
NC_040257.1	128077632	A		G
NC_040257.1	128077851	T		C
NC_040257.1	128103261	T		C
NC_040257.1	128111677	A		G
NC_040257.1	128170105	A		G
NC_040257.1	128238678	G		A
NC_040257.1	128239011	G		A
NC_040257.1	128260753	G		C
NC_040257.1	128277994		0	G
NC_040257.1	128280035	T		G
NC_040257.1	128281236	T		C
NC_040257.1	128334510	C		T
NC_040257.1	128389666		0	T
NC_040257.1	128389910	T		G
NC_040257.1	128410010	A		G
NC_040257.1	128415606	A		C
NC_040257.1	128435514	C		T
NC_040257.1	128495160	G		A
NC_040257.1	128557647	T		C
NC_040257.1	128589829	A		G
NC_040257.1	128591717	T		C
NC_040257.1	128609539	A		G
NC_040257.1	128609782	A		G

NC_040257.1	128661162	A	G
NC_040257.1	128674444	G	A
NC_040257.1	128675542	T	C
NC_040257.1	128675740	A	G
NC_040257.1	128676142	A	G
NC_040257.1	128678997	A	G
NC_040257.1	128684639	A	G
NC_040257.1	128692767	T	C
NC_040257.1	128730715	T	C
NC_040257.1	128736148	T	C
NC_040257.1	128736361	C	T
NC_040257.1	128761189	A	G
NC_040257.1	128782732	G	A
NC_040257.1	128783109	A	G
NC_040257.1	128783910	G	A
NC_040257.1	128785918	T	G
NC_040257.1	128786945	A	G
NC_040257.1	128796772	T	C
NC_040257.1	128848031	C	T
NC_040257.1	128848481	G	T
NC_040257.1	128852282	A	G
NC_040257.1	128856727	C	G
NC_040257.1	128911667	C	T
NC_040257.1	128965874	A	G
NC_040257.1	129022306	T	C
NC_040257.1	129068259	T	C
NC_040257.1	129105925	G	A
NC_040257.1	129119028	C	G
NC_040257.1	129119237	A	G
NC_040257.1	129144521	C	T
NC_040257.1	129188786	T	C
NC_040257.1	129197341	T	C
NC_040257.1	129236260	A	G
NC_040257.1	129290617	A	G
NC_040257.1	129352235	C	G
NC_040257.1	129374267	A	G
NC_040257.1	129374666	T	C
NC_040257.1	129375592	C	A
NC_040257.1	129375829	G	A
NC_040257.1	129376405	G	A
NC_040257.1	129377867	C	A
NC_040257.1	129386257	T	C
NC_040257.1	129386972	A	C
NC_040257.1	129387356	A	G
NC_040257.1	129388309	T	C
NC_040257.1	129390253	T	C
NC_040257.1	129412854	G	A

NC_040257.1	129413055	C	A
NC_040257.1	129413774	A	C
NC_040257.1	129454995	A	G
NC_040257.1	129465657	A	G
NC_040257.1	129470226	T	C
NC_040257.1	129475642	A	C
NC_040257.1	129482113	A	G
NC_040257.1	129482877	G	T
NC_040257.1	129495368	T	C
NC_040257.1	129535085	T	G
NC_040257.1	129535584	T	C
NC_040257.1	129553136	A	G
NC_040257.1	129554913	T	C
NC_040257.1	129601956	G	C
NC_040257.1	129632445	T	C
NC_040257.1	129639480	G	A
NC_040257.1	129649587	C	G
NC_040257.1	129665978	T	C
NC_040257.1	129699575	G	A
NC_040257.1	129739169	C	T
NC_040257.1	129748326	T	G
NC_040258.1	38601	G	A
NC_040258.1	109232	T	C
NC_040258.1	177956	A	G
NC_040258.1	210646	A	G
NC_040258.1	227914	G	A
NC_040258.1	280421	A	T
NC_040258.1	329376	A	G
NC_040258.1	365141	C	T
NC_040258.1	401326	G	A
NC_040258.1	469327	G	A
NC_040258.1	524114	A	G
NC_040258.1	578548	A	G
NC_040258.1	633968	A	G
NC_040258.1	680344	C	T
NC_040258.1	688391	T	C
NC_040258.1	743102	G	T
NC_040258.1	775992	T	C
NC_040258.1	809128	A	G
NC_040258.1	813130	A	G
NC_040258.1	876962	A	C
NC_040258.1	988497	A	G
NC_040258.1	1042405	G	A
NC_040258.1	1046350	T	C
NC_040258.1	1047007	C	T
NC_040258.1	1047190	T	C
NC_040258.1	1049287	A	G

NC_040258.1	1050793	T	C
NC_040258.1	1050994	G	A
NC_040258.1	1106788	C	A
NC_040258.1	1159320	G	A
NC_040258.1	1215332	T	G
NC_040258.1	1290125	G	T
NC_040258.1	1377152	G	A
NC_040258.1	1421257	C	T
NC_040258.1	1498004	C	G
NC_040258.1	1536180	G	T
NC_040258.1	1553836	G	A
NC_040258.1	1602372	T	C
NC_040258.1	1646789	G	A
NC_040258.1	1700876	C	G
NC_040258.1	1706649	G	A
NC_040258.1	1766860	A	G
NC_040258.1	1828836	A	G
NC_040258.1	1885864	G	A
NC_040258.1	1939757	C	T
NC_040258.1	1993850	C	G
NC_040258.1	2039042	C	T
NC_040258.1	2069032	A	G
NC_040258.1	2092167	T	C
NC_040258.1	2143711	A	G
NC_040258.1	2195421	G	T
NC_040258.1	2220199	C	T
NC_040258.1	2275133	C	T
NC_040258.1	2331593	G	A
NC_040258.1	2379177	A	C
NC_040258.1	2411457	C	T
NC_040258.1	2419600	A	G
NC_040258.1	2453630	C	T
NC_040258.1	2473987	C	T
NC_040258.1	2528617	G	A
NC_040258.1	2528873	A	G
NC_040258.1	2582173	G	A
NC_040258.1	2618153	T	G
NC_040258.1	2639538	C	G
NC_040258.1	2664671	C	G
NC_040258.1	2711207	A	G
NC_040258.1	2717146	C	T
NC_040258.1	3108169	A	T
NC_040258.1	3164397	C	T
NC_040258.1	3225670	T	C
NC_040258.1	3290900	G	A
NC_040258.1	3310185	T	C
NC_040258.1	3476597	C	G

NC_040258.1	3506174	G	C
NC_040258.1	3520784	C	T
NC_040258.1	3551410	A	C
NC_040258.1	3602321	C	T
NC_040258.1	3643806	G	A
NC_040258.1	3675696	A	G
NC_040258.1	3854298	A	G
NC_040258.1	3885023	G	A
NC_040258.1	3930813	T	C
NC_040258.1	3940737	A	G
NC_040258.1	3993373	A	G
NC_040258.1	4047200	C	T
NC_040258.1	4096570	T	C
NC_040258.1	4142322	G	A
NC_040258.1	4196736	A	G
NC_040258.1	4255661	C	T
NC_040258.1	4309012	C	T
NC_040258.1	4363481	G	A
NC_040258.1	4411519	C	T
NC_040258.1	4427023	C	A
NC_040258.1	4496005	A	G
NC_040258.1	4541747	C	T
NC_040258.1	4566140	C	T
NC_040258.1	4566437	A	T
NC_040258.1	4629130	T	C
NC_040258.1	4678985	T	G
NC_040258.1	4686126	C	T
NC_040258.1	4737754	A	G
NC_040258.1	4803822	C	T
NC_040258.1	4889900	A	T
NC_040258.1	4911072	G	C
NC_040258.1	4973020	C	G
NC_040258.1	5260208	T	G
NC_040258.1	5312699	G	A
NC_040258.1	5373387	G	T
NC_040258.1	5425467	A	C
NC_040258.1	5460514	C	T
NC_040258.1	5509079	C	G
NC_040258.1	5511686	G	A
NC_040258.1	5559473	G	A
NC_040258.1	5620644	C	T
NC_040258.1	5675451	G	C
NC_040258.1	5713247	A	G
NC_040258.1	5731386	T	C
NC_040258.1	5785828	A	G
NC_040258.1	5837394	A	G
NC_040258.1	5896185	A	T

NC_040258.1	5945513	G	A
NC_040258.1	5961357	C	T
NC_040258.1	6000863	A	G
NC_040258.1	6015714	G	C
NC_040258.1	6050360	G	C
NC_040258.1	6102610	T	C
NC_040258.1	6134873	C	T
NC_040258.1	6175647	G	A
NC_040258.1	6181461	G	A
NC_040258.1	6207692	T	C
NC_040258.1	6234694	T	C
NC_040258.1	6287378	A	C
NC_040258.1	6324452	T	C
NC_040258.1	6352254	G	A
NC_040258.1	6408353	T	C
NC_040258.1	6450628	T	C
NC_040258.1	6481830	C	G
NC_040258.1	6555335	C	T
NC_040258.1	6589840	G	A
NC_040258.1	6661666	A	G
NC_040258.1	6750532	T	G
NC_040258.1	6808415	G	C
NC_040258.1	6891851	T	C
NC_040258.1	6922927	A	G
NC_040258.1	6967901	A	G
NC_040258.1	6973464	C	T
NC_040258.1	7037318	C	T
NC_040258.1	7041215	T	G
NC_040258.1	7215204	G	A
NC_040258.1	7269493	T	G
NC_040258.1	7327540	G	A
NC_040258.1	7394301	A	G
NC_040258.1	7489498	A	G
NC_040258.1	7520501	C	A
NC_040258.1	7530417	T	C
NC_040258.1	7558679	G	C
NC_040258.1	7567997	T	C
NC_040258.1	7613024	T	C
NC_040258.1	7676659	T	C
NC_040258.1	7725518	C	T
NC_040258.1	7761333	T	A
NC_040258.1	7800215	C	T
NC_040258.1	7864348	T	C
NC_040258.1	7945393	A	G
NC_040258.1	7978804	T	G
NC_040258.1	8000455	C	G
NC_040258.1	8145735	T	C

NC_040258.1	8231032	G	C
NC_040258.1	8335212	T	C
NC_040258.1	8463657	A	G
NC_040258.1	8491599	C	A
NC_040258.1	8529459	G	A
NC_040258.1	8542448	T	G
NC_040258.1	8651372	C	T
NC_040258.1	8707425	T	C
NC_040258.1	8747578	A	G
NC_040258.1	8780887	T	C
NC_040258.1	8824882	T	C
NC_040258.1	8835772	A	G
NC_040258.1	8886867	T	C
NC_040258.1	8916248	A	G
NC_040258.1	8972126	T	C
NC_040258.1	8997773	A	G
NC_040258.1	9118191	C	T
NC_040258.1	9150984	G	A
NC_040258.1	9187711	G	A
NC_040258.1	9203779	A	T
NC_040258.1	9260537	A	G
NC_040258.1	9312335	G	T
NC_040258.1	9389024	C	T
NC_040258.1	9450584	C	T
NC_040258.1	9478788	T	C
NC_040258.1	9519348	G	T
NC_040258.1	9540634	C	T
NC_040258.1	9593287	C	T
NC_040258.1	9619720	T	A
NC_040258.1	9647365	C	T
NC_040258.1	9686786	C	A
NC_040258.1	9750361	C	T
NC_040258.1	9806258	C	T
NC_040258.1	9831392	G	T
NC_040258.1	9849965	T	C
NC_040258.1	9896223	G	C
NC_040258.1	9904540	T	C
NC_040258.1	9949025	A	C
NC_040258.1	9961466	C	T
NC_040258.1	10000408	T	C
NC_040258.1	10019029	A	G
NC_040258.1	10052801	T	C
NC_040258.1	10109215	T	C
NC_040258.1	10148434	C	T
NC_040258.1	10202973	T	C
NC_040258.1	10442141	A	G
NC_040258.1	10457399	G	A

NC_040258.1	10486835	C	T
NC_040258.1	10550675	T	C
NC_040258.1	10602531	C	T
NC_040258.1	10628884	T	C
NC_040258.1	10653713	G	A
NC_040258.1	10677048	A	C
NC_040258.1	10794096	A	G
NC_040258.1	10835250	A	G
NC_040258.1	10899227	C	T
NC_040258.1	10927797	T	C
NC_040258.1	10980815	G	A
NC_040258.1	11053164	A	T
NC_040258.1	11124139	G	C
NC_040258.1	11152428	T	C
NC_040258.1	11231155	T	C
NC_040258.1	11251552	T	C
NC_040258.1	11316366	C	T
NC_040258.1	11386433	C	T
NC_040258.1	11462414	C	T
NC_040258.1	11521253	C	T
NC_040258.1	11592882	A	G
NC_040258.1	11609210	T	C
NC_040258.1	11693240	C	T
NC_040258.1	11760446	A	G
NC_040258.1	11822654	C	T
NC_040258.1	11871270	A	T
NC_040258.1	11879622	C	A
NC_040258.1	11911607	T	G
NC_040258.1	11953858	T	C
NC_040258.1	12011752	T	C
NC_040258.1	12260674	G	A
NC_040258.1	12324256	A	G
NC_040258.1	12349980	A	G
NC_040258.1	12389230	A	G
NC_040258.1	12420991	T	C
NC_040258.1	12443651	C	T
NC_040258.1	12472252	C	G
NC_040258.1	12504822	A	G
NC_040258.1	12538208	A	G
NC_040258.1	12563454	C	A
NC_040258.1	12580130	A	G
NC_040258.1	12650452	C	A
NC_040258.1	12677128	G	A
NC_040258.1	12784403	A	C
NC_040258.1	12830185	C	G
NC_040258.1	12853583	G	A
NC_040258.1	12868984	A	G

NC_040258.1	12929615	C	T
NC_040258.1	12936886	A	G
NC_040258.1	12998953	G	C
NC_040258.1	13034869	C	T
NC_040258.1	13062716	G	C
NC_040258.1	13094328	T	G
NC_040258.1	13127075	C	G
NC_040258.1	13138969	A	G
NC_040258.1	13184252	T	G
NC_040258.1	13211570	T	C
NC_040258.1	13256574	C	T
NC_040258.1	13309355	G	A
NC_040258.1	13362026	T	G
NC_040258.1	13401030	T	G
NC_040258.1	13423786	T	C
NC_040258.1	13475009	C	T
NC_040258.1	13511989	A	G
NC_040258.1	13549237	C	T
NC_040258.1	13560847	C	T
NC_040258.1	13603169	A	C
NC_040258.1	13611106	A	G
NC_040258.1	13654400	T	C
NC_040258.1	13706638	G	A
NC_040258.1	13765102	T	C
NC_040258.1	13815222	A	G
NC_040258.1	13821707	G	A
NC_040258.1	13874696	G	A
NC_040258.1	13896542	T	C
NC_040258.1	13955203	G	A
NC_040258.1	14027445	C	T
NC_040258.1	14039069	T	A
NC_040258.1	14155532	T	C
NC_040258.1	14182007	C	T
NC_040258.1	14212851	T	C
NC_040258.1	14276086	G	C
NC_040258.1	14294067	C	A
NC_040258.1	14333444	T	C
NC_040258.1	14388015	G	A
NC_040258.1	14429060	A	G
NC_040258.1	14458132	A	C
NC_040258.1	14489824	A	G
NC_040258.1	14532783	T	C
NC_040258.1	14584284	G	A
NC_040258.1	14588513	A	C
NC_040258.1	14640080	T	C
NC_040258.1	14681244	G	A
NC_040258.1	14712033	T	C

NC_040258.1	14724338	G	A
NC_040258.1	14757759	T	C
NC_040258.1	14808930	A	T
NC_040258.1	14864335	G	C
NC_040258.1	14905132	T	C
NC_040258.1	14933298	T	C
NC_040258.1	14961962	C	T
NC_040258.1	15017603	G	A
NC_040258.1	15068951	C	T
NC_040258.1	15119698	C	T
NC_040258.1	15122254	C	T
NC_040258.1	15177881	A	G
NC_040258.1	15224641	G	A
NC_040258.1	15255242	C	A
NC_040258.1	15304361	C	A
NC_040258.1	15360793	A	G
NC_040258.1	15418732	A	T
NC_040258.1	15445028	G	T
NC_040258.1	15485207	C	T
NC_040258.1	15533485	T	C
NC_040258.1	15616305	C	T
NC_040258.1	15629675	G	C
NC_040258.1	15667533	G	A
NC_040258.1	15687146	T	C
NC_040258.1	15753873	A	T
NC_040258.1	15804178	T	A
NC_040258.1	15839590	G	A
NC_040258.1	15907807	C	A
NC_040258.1	15962975	A	G
NC_040258.1	16015542	T	C
NC_040258.1	16053551	A	C
NC_040258.1	16094984	G	A
NC_040258.1	16098756	T	C
NC_040258.1	16137756	A	G
NC_040258.1	16154699	T	C
NC_040258.1	16187850	A	G
NC_040258.1	16238020	T	G
NC_040258.1	16300225	A	G
NC_040258.1	16330744	T	C
NC_040258.1	16374148	T	C
NC_040258.1	16400293	G	T
NC_040258.1	16426335	A	G
NC_040258.1	16533522	T	C
NC_040258.1	16571118	G	A
NC_040258.1	16578862	A	G
NC_040258.1	16605145	G	A
NC_040258.1	16634778	T	C

NC_040258.1	16711489	T	C
NC_040258.1	16805698	A	G
NC_040258.1	16860056	A	C
NC_040258.1	16947336	T	C
NC_040258.1	16987676	T	C
NC_040258.1	17057033	A	G
NC_040258.1	17174601	T	C
NC_040258.1	17232872	A	G
NC_040258.1	17264696	T	C
NC_040258.1	17297752	T	C
NC_040258.1	17309169	T	A
NC_040258.1	17319165	T	A
NC_040258.1	17352092	A	G
NC_040258.1	17422719	T	A
NC_040258.1	17471950	G	A
NC_040258.1	17482592	C	T
NC_040258.1	17533965	T	C
NC_040258.1	17537684	A	G
NC_040258.1	17570424	G	A
NC_040258.1	17587987	T	C
NC_040258.1	17625618	T	C
NC_040258.1	17645740	A	G
NC_040258.1	17703529	T	A
NC_040258.1	17745281	A	G
NC_040258.1	17756835	A	G
NC_040258.1	17789141	A	G
NC_040258.1	17810055	T	C
NC_040258.1	17865923	T	C
NC_040258.1	17922297	A	G
NC_040258.1	17968537	C	G
NC_040258.1	18003951	G	A
NC_040258.1	18075742	C	T
NC_040258.1	18110842	T	C
NC_040258.1	18147649	G	A
NC_040258.1	18200655	G	A
NC_040258.1	18230357	G	A
NC_040258.1	18258400	G	C
NC_040258.1	18290434	A	G
NC_040258.1	18309419	A	G
NC_040258.1	18611359	C	G
NC_040258.1	18682625	G	A
NC_040258.1	18725937	C	T
NC_040258.1	18775420	A	G
NC_040258.1	18825666	C	T
NC_040258.1	18875468	A	G
NC_040258.1	18939071	T	C
NC_040258.1	18973304	G	T

NC_040258.1	19021568	G	A
NC_040258.1	19069020	T	C
NC_040258.1	19104870	T	A
NC_040258.1	19141362	C	T
NC_040258.1	19179275	G	A
NC_040258.1	19218349	C	T
NC_040258.1	19254784	C	G
NC_040258.1	19279848	T	C
NC_040258.1	19308896	A	G
NC_040258.1	19330025	T	C
NC_040258.1	19377249	G	T
NC_040258.1	19432788	C	T
NC_040258.1	19476030	T	A
NC_040258.1	19536822	A	G
NC_040258.1	19593636	T	C
NC_040258.1	19641766	C	T
NC_040258.1	19699533	T	C
NC_040258.1	19730731	G	C
NC_040258.1	19753441	C	T
NC_040258.1	19823081	C	A
NC_040258.1	19869098	G	T
NC_040258.1	19918584	A	G
NC_040258.1	19961054	C	T
NC_040258.1	20003443	C	G
NC_040258.1	20009578	A	G
NC_040258.1	20088885	C	T
NC_040258.1	20132709	T	C
NC_040258.1	20148734	T	C
NC_040258.1	20212368	T	C
NC_040258.1	20215653	G	A
NC_040258.1	20218756	A	C
NC_040258.1	20279734	T	A
NC_040258.1	20334036	C	T
NC_040258.1	20389619	C	G
NC_040258.1	20446577	T	G
NC_040258.1	20488508	A	G
NC_040258.1	20532313	T	C
NC_040258.1	20586860	T	C
NC_040258.1	20589281	G	A
NC_040258.1	20659845	T	C
NC_040258.1	20709161	G	T
NC_040258.1	20751269	G	A
NC_040258.1	20785983	C	T
NC_040258.1	20811262	G	A
NC_040258.1	20867587	T	C
NC_040258.1	20925981	C	T
NC_040258.1	20964316	A	C

NC_040258.1	21012310	A	G
NC_040258.1	21020970	T	C
NC_040258.1	21059922	G	C
NC_040258.1	21093449	G	C
NC_040258.1	21116548	T	A
NC_040258.1	21158733	C	T
NC_040258.1	21168667	C	T
NC_040258.1	21239802	C	T
NC_040258.1	21304110	T	C
NC_040258.1	21360467	C	T
NC_040258.1	21418557	A	G
NC_040258.1	21471323	C	A
NC_040258.1	21522397	C	T
NC_040258.1	21569044	G	A
NC_040258.1	21597357	C	T
NC_040258.1	21626098	A	G
NC_040258.1	21633505	T	A
NC_040258.1	21690984	C	T
NC_040258.1	21744061	C	T
NC_040258.1	21798543	C	T
NC_040258.1	21851223	A	G
NC_040258.1	21905599	G	C
NC_040258.1	21972629	A	T
NC_040258.1	22009121	C	G
NC_040258.1	22027217	C	T
NC_040258.1	22040636	G	C
NC_040258.1	22050604	C	T
NC_040258.1	22053001	T	C
NC_040258.1	22118246	T	G
NC_040258.1	22157779	C	T
NC_040258.1	22217962	T	C
NC_040258.1	22279533	T	C
NC_040258.1	22286554	T	G
NC_040258.1	22295400	T	C
NC_040258.1	22299821	T	C
NC_040258.1	22305487	T	C
NC_040258.1	22309291	T	A
NC_040258.1	22317722	T	C
NC_040258.1	22328628	G	T
NC_040258.1	22346739	G	C
NC_040258.1	22375145	G	A
NC_040258.1	22406042	G	A
NC_040258.1	22446526	T	C
NC_040258.1	22453714	A	G
NC_040258.1	22504439	A	G
NC_040258.1	22529899	G	A
NC_040258.1	22565472	C	T

NC_040258.1	22594083	A	G
NC_040258.1	22617878	T	C
NC_040258.1	22689923	A	G
NC_040258.1	22733295	C	A
NC_040258.1	22803687	T	C
NC_040258.1	22878890	T	C
NC_040258.1	22883820	T	C
NC_040258.1	22913224	T	C
NC_040258.1	22922122	A	T
NC_040258.1	22937036	G	T
NC_040258.1	22995432	T	C
NC_040258.1	23078926	A	G
NC_040258.1	23141821	A	C
NC_040258.1	23172684	G	T
NC_040258.1	23177385	T	G
NC_040258.1	23182229	T	C
NC_040258.1	23238838	A	G
NC_040258.1	23245703	T	C
NC_040258.1	23264109	C	G
NC_040258.1	23268712	T	C
NC_040258.1	23273843	A	G
NC_040258.1	23349792	C	T
NC_040258.1	23484105	C	G
NC_040258.1	23513775	T	C
NC_040258.1	23539623	C	A
NC_040258.1	23595050	A	G
NC_040258.1	23656849	G	A
NC_040258.1	23718274	A	C
NC_040258.1	23777061	A	T
NC_040258.1	23832368	A	C
NC_040258.1	23907125	G	A
NC_040258.1	23930111	C	T
NC_040258.1	23982605	T	C
NC_040258.1	24020541	C	T
NC_040258.1	24575736	A	G
NC_040258.1	26074121	A	G
NC_040258.1	26074777	T	C
NC_040258.1	26521436	T	C
NC_040258.1	26570802	C	T
NC_040258.1	26697606	T	C
NC_040258.1	27015301	T	C
NC_040258.1	27397066	C	T
NC_040258.1	27540526	T	C
NC_040258.1	28000195	T	G
NC_040258.1	28072917	A	C
NC_040258.1	28096919	A	G
NC_040258.1	28154652	T	C

NC_040258.1	28210079	G	A
NC_040258.1	28236947	A	G
NC_040258.1	28280282	A	C
NC_040258.1	28353407	T	G
NC_040258.1	28527515	C	A
NC_040258.1	28617056	G	A
NC_040258.1	28662106	A	G
NC_040258.1	28691683	T	C
NC_040258.1	28733093	A	G
NC_040258.1	28797615	C	T
NC_040258.1	28823272	T	C
NC_040258.1	28856934	A	G
NC_040258.1	28874442	G	A
NC_040258.1	28970262	T	A
NC_040258.1	28990942	A	T
NC_040258.1	29095091	T	G
NC_040258.1	29134272	T	C
NC_040258.1	29179653	T	G
NC_040258.1	29352958	A	C
NC_040258.1	29406516	A	G
NC_040258.1	29456619	T	C
NC_040258.1	29483959	C	T
NC_040258.1	29517739	A	G
NC_040258.1	29543792	G	A
NC_040258.1	29581874	A	C
NC_040258.1	29595056	G	C
NC_040258.1	29676886	G	A
NC_040258.1	29712946	C	A
NC_040258.1	29738995	A	G
NC_040258.1	29791464	C	T
NC_040258.1	29849211	T	C
NC_040258.1	29903551	T	C
NC_040258.1	29935092	C	G
NC_040258.1	30063104	T	C
NC_040258.1	30169402	T	C
NC_040258.1	30213079	G	C
NC_040258.1	30298797	A	G
NC_040258.1	30330004	A	G
NC_040258.1	30369289	T	C
NC_040258.1	30438105	A	T
NC_040258.1	30467023	T	C
NC_040258.1	30484951	C	T
NC_040258.1	30509086	T	C
NC_040258.1	30561227	T	C
NC_040258.1	30615996	C	T
NC_040258.1	30697489	A	G
NC_040258.1	30796093	G	C

NC_040258.1	30861300	G	T
NC_040258.1	30914344	C	T
NC_040258.1	30946714	G	C
NC_040258.1	30976678	G	A
NC_040258.1	31029091	G	A
NC_040258.1	31055051	A	G
NC_040258.1	31074369	T	C
NC_040258.1	31108918	A	G
NC_040258.1	31165279	A	G
NC_040258.1	31222436	G	A
NC_040258.1	31260107	T	G
NC_040258.1	31526377	T	C
NC_040258.1	31588450	A	T
NC_040258.1	31615029	C	A
NC_040258.1	31627357	G	A
NC_040258.1	31654970	T	G
NC_040258.1	31752425	T	G
NC_040258.1	31780062	T	C
NC_040258.1	31804169	T	G
NC_040258.1	31981888	G	A
NC_040258.1	32013733	T	A
NC_040258.1	32053773	A	C
NC_040258.1	32153615	T	C
NC_040258.1	32213966	T	C
NC_040258.1	32256599	A	G
NC_040258.1	32339255	A	G
NC_040258.1	32392772	A	G
NC_040258.1	32424921	G	A
NC_040258.1	32458231	C	T
NC_040258.1	32496578	G	C
NC_040258.1	32540136	T	C
NC_040258.1	32570073	T	G
NC_040258.1	32595844	A	T
NC_040258.1	32639070	A	C
NC_040258.1	32714960	C	G
NC_040258.1	32759136	T	A
NC_040258.1	32800495	A	G
NC_040258.1	32842346	T	C
NC_040258.1	32860153	C	T
NC_040258.1	32949695	G	A
NC_040258.1	33012495	A	C
NC_040258.1	33057161	C	T
NC_040258.1	33106334	G	A
NC_040258.1	33152210	C	G
NC_040258.1	33217535	C	T
NC_040258.1	33268772	C	T
NC_040258.1	33344533	G	A

NC_040258.1	33407022	G	A
NC_040258.1	33505340	T	G
NC_040258.1	33556490	G	T
NC_040258.1	33620856	A	G
NC_040258.1	33671293	C	T
NC_040258.1	33702955	G	A
NC_040258.1	33747794	G	T
NC_040258.1	33790223	A	G
NC_040258.1	33832963	A	G
NC_040258.1	33902652	A	G
NC_040258.1	33951376	T	C
NC_040258.1	34003929	A	C
NC_040258.1	34038715	G	A
NC_040258.1	34076911	G	A
NC_040258.1	34121374	T	C
NC_040258.1	34173063	G	A
NC_040258.1	34234684	G	C
NC_040258.1	34530587	C	T
NC_040258.1	34659478	A	G
NC_040258.1	34685493	C	G
NC_040258.1	34700818	A	T
NC_040258.1	34726815	T	C
NC_040258.1	34791541	G	A
NC_040258.1	34835114	C	T
NC_040258.1	34912329	A	G
NC_040258.1	34959180	A	G
NC_040258.1	35015040	A	G
NC_040258.1	35085463	A	G
NC_040258.1	35114601	A	G
NC_040258.1	35130695	T	A
NC_040258.1	35158215	G	A
NC_040258.1	35188560	C	T
NC_040258.1	35235046	C	T
NC_040258.1	35285123	A	G
NC_040258.1	35343878	C	T
NC_040258.1	35382202	A	G
NC_040258.1	35410205	A	G
NC_040258.1	35551283	G	T
NC_040258.1	35625081	T	C
NC_040258.1	35680185	G	A
NC_040258.1	35728142	A	G
NC_040258.1	35756877	C	G
NC_040258.1	35784374	C	A
NC_040258.1	35811213	T	C
NC_040258.1	35854196	A	G
NC_040258.1	35888036	A	G
NC_040258.1	35942868	A	G

NC_040258.1	35977458	A		G
NC_040258.1	36008161	C		G
NC_040258.1	36038662	A		G
NC_040258.1	36071807	T		C
NC_040258.1	36104718		0	G
NC_040258.1	36216158	A		G
NC_040258.1	36450693	T		G
NC_040258.1	36476304	T		C
NC_040258.1	36508795	G		C
NC_040258.1	36537663	C		T
NC_040258.1	36563720	C		T
NC_040258.1	36616197	T		C
NC_040258.1	36759008		0	G
NC_040258.1	36929705	T		A
NC_040258.1	36975384	A		C
NC_040258.1	37022710	C		T
NC_040258.1	37047918	T		C
NC_040258.1	37078137	A		G
NC_040258.1	37161462	A		G
NC_040258.1	37214286	T		G
NC_040258.1	37245752	A		G
NC_040258.1	37401062	A		G
NC_040258.1	37466467	C		T
NC_040258.1	37549734	G		A
NC_040258.1	37681502	G		A
NC_040258.1	37802043	C		T
NC_040258.1	37839686	G		A
NC_040258.1	37906324	G		A
NC_040258.1	37941272	A		C
NC_040258.1	38042815	C		T
NC_040258.1	38115768	C		T
NC_040258.1	38180531	C		T
NC_040258.1	38271440	C		A
NC_040258.1	38346272	C		A
NC_040258.1	38400563	A		G
NC_040258.1	38454597	G		C
NC_040258.1	38682120	A		G
NC_040258.1	38747748	G		A
NC_040258.1	38863812	C		T
NC_040258.1	38907282	A		C
NC_040258.1	38972158	G		A
NC_040258.1	39030349	G		A
NC_040258.1	39176046	A		G
NC_040258.1	39431090	T		G
NC_040258.1	39481816	A		C
NC_040258.1	39512581	T		C
NC_040258.1	39638507	G		A

NC_040258.1	39663253	A	T
NC_040258.1	39845796	C	T
NC_040258.1	40112581	C	T
NC_040258.1	40570854	T	G
NC_040258.1	40717460	T	C
NC_040258.1	40890841	G	A
NC_040258.1	41037728	C	A
NC_040258.1	41390228	C	T
NC_040258.1	41499323	T	G
NC_040258.1	41531898	C	A
NC_040258.1	41692608	C	T
NC_040258.1	41944643	G	A
NC_040258.1	42247712	T	C
NC_040258.1	42337595	G	A
NC_040258.1	42405394	C	A
NC_040258.1	42516273	T	C
NC_040258.1	42840163	A	G
NC_040258.1	43365901	G	A
NC_040258.1	43441481	G	T
NC_040258.1	43603463	G	A
NC_040258.1	43695171	G	A
NC_040258.1	43729134	C	T
NC_040258.1	43759102	T	C
NC_040258.1	43872173	G	A
NC_040258.1	43923222	C	T
NC_040258.1	44102545	T	C
NC_040258.1	44129491	C	A
NC_040258.1	44168924	T	C
NC_040258.1	44212035	T	C
NC_040258.1	44582227	A	G
NC_040258.1	44762130	T	C
NC_040258.1	44811642	A	G
NC_040258.1	45108964	A	G
NC_040258.1	45197285	T	A
NC_040258.1	45263743	C	T
NC_040258.1	45311916	G	C
NC_040258.1	45357859	G	A
NC_040258.1	45407945	G	A
NC_040258.1	45451094	T	C
NC_040258.1	45511846	T	G
NC_040258.1	45566534	G	T
NC_040258.1	45685218	G	A
NC_040258.1	45846453	A	G
NC_040258.1	45943219	A	G
NC_040258.1	46065537	T	C
NC_040258.1	46094559	T	C
NC_040258.1	46277971	A	C

NC_040258.1	46350011	A	C
NC_040258.1	46511905	G	A
NC_040258.1	46540403	G	A
NC_040258.1	46571497	A	G
NC_040258.1	46623726	C	T
NC_040258.1	46681137	G	C
NC_040258.1	46715856	A	G
NC_040258.1	46739182	C	T
NC_040258.1	46801454	T	C
NC_040258.1	46828544	G	A
NC_040258.1	46849150	G	A
NC_040258.1	46905923	G	T
NC_040258.1	46931606	T	C
NC_040258.1	47014321	G	A
NC_040258.1	47050100	A	T
NC_040258.1	47073111	A	T
NC_040258.1	47101835	C	T
NC_040258.1	47115368	A	G
NC_040258.1	47160250	C	T
NC_040258.1	47215810	T	C
NC_040258.1	47227708	A	G
NC_040258.1	47292921	C	T
NC_040258.1	47327313	C	T
NC_040258.1	47378213	C	T
NC_040258.1	47424019	C	A
NC_040258.1	47483846	C	T
NC_040258.1	47531845	A	G
NC_040258.1	47621088	G	T
NC_040258.1	47672357	G	A
NC_040258.1	47721721	C	T
NC_040258.1	47821425	T	C
NC_040258.1	47841449	G	C
NC_040258.1	47919908	G	C
NC_040258.1	47975893	T	C
NC_040258.1	48016490	T	C
NC_040258.1	48281637	T	C
NC_040258.1	48314018	G	A
NC_040258.1	48341929	C	T
NC_040258.1	48412498	C	T
NC_040258.1	48458217	A	G
NC_040258.1	48492845	T	C
NC_040258.1	48536125	T	C
NC_040258.1	48725267	A	G
NC_040258.1	48765835	C	T
NC_040258.1	48849562	G	A
NC_040258.1	49103513	G	A
NC_040258.1	49160554	G	A

NC_040258.1	49262207	A	C
NC_040258.1	49325621	T	C
NC_040258.1	49487782	C	T
NC_040258.1	49531449	T	G
NC_040258.1	49576792	A	G
NC_040258.1	49611822	T	C
NC_040258.1	49637771	T	C
NC_040258.1	49758340	A	G
NC_040258.1	50125806	A	C
NC_040258.1	50160531	T	C
NC_040258.1	50174573	C	A
NC_040258.1	50268813	C	G
NC_040258.1	50307516	C	T
NC_040258.1	50336831	T	C
NC_040258.1	50354587	G	A
NC_040258.1	50408047	T	G
NC_040258.1	50479960	A	T
NC_040258.1	50518337	T	A
NC_040258.1	50544145	A	G
NC_040258.1	50577361	C	T
NC_040258.1	50650307	T	C
NC_040258.1	50700085	G	T
NC_040258.1	50741099	T	C
NC_040258.1	50763392	T	C
NC_040258.1	50810585	G	A
NC_040258.1	50902620	A	T
NC_040258.1	50945361	C	T
NC_040258.1	50989448	T	C
NC_040258.1	51022227	T	C
NC_040258.1	51063916	A	G
NC_040258.1	51198790	A	G
NC_040258.1	51219686	A	G
NC_040258.1	51282905	A	G
NC_040258.1	51320106	T	C
NC_040258.1	51340664	T	C
NC_040258.1	51385631	G	A
NC_040258.1	51456184	T	G
NC_040258.1	51513646	C	T
NC_040258.1	51580753	T	A
NC_040258.1	51833858	G	A
NC_040258.1	52046454	T	C
NC_040258.1	52149762	C	G
NC_040258.1	52270574	C	T
NC_040258.1	52313892	A	G
NC_040258.1	52345131	T	C
NC_040258.1	52384173	G	A
NC_040258.1	52447694	C	A

NC_040258.1	52526080	T	A
NC_040258.1	52595354	C	T
NC_040258.1	52649678	T	C
NC_040258.1	52700988	C	T
NC_040258.1	52768348	C	A
NC_040258.1	52813691	A	G
NC_040258.1	52843761	T	C
NC_040258.1	52897607	G	A
NC_040258.1	52925658	G	A
NC_040258.1	52950411	G	A
NC_040258.1	52975917	G	C
NC_040258.1	52989367	G	A
NC_040258.1	53055620	C	G
NC_040258.1	53149673	A	G
NC_040258.1	53188098	T	C
NC_040258.1	53326825	G	A
NC_040258.1	53450294	T	C
NC_040258.1	53533941	G	T
NC_040258.1	53619486	G	A
NC_040258.1	53683502	C	T
NC_040258.1	53727913	T	A
NC_040258.1	53792058	A	C
NC_040258.1	53890962	C	T
NC_040258.1	53948796	T	A
NC_040258.1	54002092	T	C
NC_040258.1	54057789	A	G
NC_040258.1	54093960	G	A
NC_040258.1	54117517	C	T
NC_040258.1	54176243	A	T
NC_040258.1	54333808	A	C
NC_040258.1	54378965	C	G
NC_040258.1	54409711	T	C
NC_040258.1	54424626	C	T
NC_040258.1	54460099	T	A
NC_040258.1	54496913	A	C
NC_040258.1	54572901	T	A
NC_040258.1	54602717	G	A
NC_040258.1	54615115	T	G
NC_040258.1	54641687	C	T
NC_040258.1	54689940	T	C
NC_040258.1	54747536	C	G
NC_040258.1	54787499	G	A
NC_040258.1	54849951	G	A
NC_040258.1	55022219	G	A
NC_040258.1	55055848	C	T
NC_040258.1	55080301	G	A
NC_040258.1	55127872	T	C

NC_040258.1	55161972	C	T
NC_040258.1	55192066	C	G
NC_040258.1	55343830	C	T
NC_040258.1	55374585	A	G
NC_040258.1	55412424	T	C
NC_040258.1	55480620	T	C
NC_040258.1	55603349	A	G
NC_040258.1	55651850	A	T
NC_040258.1	55667351	C	G
NC_040258.1	55754513	A	G
NC_040258.1	55798428	G	T
NC_040258.1	55891207	C	A
NC_040258.1	55956109	G	T
NC_040258.1	55997954	A	G
NC_040258.1	56014102	T	C
NC_040258.1	56076693	G	A
NC_040258.1	56142817	T	A
NC_040258.1	56169670	G	C
NC_040258.1	56181360	A	T
NC_040258.1	56476568	T	C
NC_040258.1	56510809	A	C
NC_040258.1	56517019	G	A
NC_040258.1	56558317	A	G
NC_040258.1	56596998	T	C
NC_040258.1	56651706	G	A
NC_040258.1	56699355	A	G
NC_040258.1	56787135	T	C
NC_040258.1	56954578	T	C
NC_040258.1	57032937	G	A
NC_040258.1	57089365	T	G
NC_040258.1	57208627	C	T
NC_040258.1	57305086	G	A
NC_040258.1	57458817	C	T
NC_040258.1	57509504	C	A
NC_040258.1	57700738	T	C
NC_040258.1	57755569	T	C
NC_040258.1	57894847	T	C
NC_040258.1	58351484	G	A
NC_040258.1	58412537	G	A
NC_040258.1	58441583	C	G
NC_040258.1	58526926	C	T
NC_040258.1	58623976	T	C
NC_040258.1	58730858	A	C
NC_040258.1	58914469	T	A
NC_040258.1	58956751	C	T
NC_040258.1	58986310	T	C
NC_040258.1	59090064	G	A

NC_040258.1	59137211	T	C
NC_040258.1	59178411	C	T
NC_040258.1	59277547	G	A
NC_040258.1	59328910	A	G
NC_040258.1	59379669	T	C
NC_040258.1	59416411	T	C
NC_040258.1	59453978	G	C
NC_040258.1	59503905	C	G
NC_040258.1	59570416	C	T
NC_040258.1	59625553	C	T
NC_040258.1	59726003	T	C
NC_040258.1	59804569	T	C
NC_040258.1	59872987	G	A
NC_040258.1	59911856	C	T
NC_040258.1	60006025	T	C
NC_040258.1	60115416	A	G
NC_040258.1	60169635	C	T
NC_040258.1	60212241	G	T
NC_040258.1	60254111	A	G
NC_040258.1	60315355	T	C
NC_040258.1	60445474	T	A
NC_040258.1	60477810	G	A
NC_040258.1	60561799	A	G
NC_040258.1	60629807	A	C
NC_040258.1	60648871	T	C
NC_040258.1	60680491	G	A
NC_040258.1	60702325	C	T
NC_040258.1	60754690	A	G
NC_040258.1	60796884	C	G
NC_040258.1	60855012	C	T
NC_040258.1	60881716	T	C
NC_040258.1	60956325	G	A
NC_040258.1	60996550	G	T
NC_040258.1	61034721	G	A
NC_040258.1	61091317	G	T
NC_040258.1	61136325	T	C
NC_040258.1	61181719	T	C
NC_040258.1	61228098	G	A
NC_040258.1	61276146	C	A
NC_040258.1	61303664	C	A
NC_040258.1	61368760	A	C
NC_040258.1	61424612	G	A
NC_040258.1	61524554	T	C
NC_040258.1	61578052	C	A
NC_040258.1	61613960	G	A
NC_040258.1	61632105	C	T
NC_040258.1	61715663	T	C

NC_040258.1	61752120	C	T
NC_040258.1	61805816	A	G
NC_040258.1	61859442	G	A
NC_040258.1	61898590	T	C
NC_040258.1	61925808	C	T
NC_040258.1	61968006	G	T
NC_040258.1	61983950	C	T
NC_040258.1	62036084	C	T
NC_040258.1	62085228	C	T
NC_040258.1	62120163	C	T
NC_040258.1	62156994	G	A
NC_040258.1	62199882	C	G
NC_040258.1	62255566	C	T
NC_040258.1	62268351	C	G
NC_040258.1	62357309	T	C
NC_040258.1	62432711	T	C
NC_040258.1	62458490	T	G
NC_040258.1	62526372	C	T
NC_040258.1	62577816	C	T
NC_040258.1	62622060	C	T
NC_040258.1	62688392	A	G
NC_040258.1	62743094	T	C
NC_040258.1	62827118	C	T
NC_040258.1	62859160	C	T
NC_040258.1	62887091	A	G
NC_040258.1	62925088	T	C
NC_040258.1	62969589	T	C
NC_040258.1	63002780	T	G
NC_040258.1	63046499	A	G
NC_040258.1	63085548	A	G
NC_040258.1	63159970	T	C
NC_040258.1	63349746	T	C
NC_040258.1	63390663	C	G
NC_040258.1	63406900	A	C
NC_040258.1	63471185	G	A
NC_040258.1	63523656	A	G
NC_040258.1	63619142	T	C
NC_040258.1	63794275	C	T
NC_040258.1	63827270	A	C
NC_040258.1	63840031	A	G
NC_040258.1	63867012	A	G
NC_040258.1	63882719	T	C
NC_040258.1	63926251	T	C
NC_040258.1	63983906	G	T
NC_040258.1	64033197	G	A
NC_040258.1	64089289	T	G
NC_040258.1	64261596	C	A

NC_040258.1	64289748	G	A
NC_040258.1	64318189	T	C
NC_040258.1	64340684	A	G
NC_040258.1	64421248	T	C
NC_040258.1	64447636	C	T
NC_040258.1	64467470	T	C
NC_040258.1	64531690	A	T
NC_040258.1	64610755	G	A
NC_040258.1	64662732	G	C
NC_040258.1	64689313	C	G
NC_040258.1	64710389	G	A
NC_040258.1	64769973	T	C
NC_040258.1	64804613	G	A
NC_040258.1	64886661	T	G
NC_040258.1	64906716	G	T
NC_040258.1	65026646	G	A
NC_040258.1	65060323	T	C
NC_040258.1	65321727	G	A
NC_040258.1	65390500	T	C
NC_040258.1	65574063	A	C
NC_040258.1	65607833	T	C
NC_040258.1	65669081	C	T
NC_040258.1	65786830	T	C
NC_040258.1	65808711	T	C
NC_040258.1	66109698	G	A
NC_040258.1	66493413	G	A
NC_040258.1	66550545	T	C
NC_040258.1	66604027	G	T
NC_040258.1	66731533	G	T
NC_040258.1	66847209	A	G
NC_040258.1	66876049	G	T
NC_040258.1	66886914	A	G
NC_040258.1	66964502	G	A
NC_040258.1	67027229	C	G
NC_040258.1	67109563	G	A
NC_040258.1	67171806	G	A
NC_040258.1	67209709	G	C
NC_040258.1	67298695	A	G
NC_040258.1	67317987	C	T
NC_040258.1	67343399	G	A
NC_040258.1	67368828	T	C
NC_040258.1	67395632	A	T
NC_040258.1	67418117	T	C
NC_040258.1	67454213	T	C
NC_040258.1	67470265	G	A
NC_040258.1	67520905	T	C
NC_040258.1	67573261	C	T

NC_040258.1	67625072	A	G
NC_040258.1	67728577	A	C
NC_040258.1	67781006	C	T
NC_040258.1	67807654	G	A
NC_040258.1	67870347	A	T
NC_040258.1	67982970	C	T
NC_040258.1	68023275	C	T
NC_040258.1	68101819	C	T
NC_040258.1	68195117	G	A
NC_040258.1	68236498	A	C
NC_040258.1	68288617	A	G
NC_040258.1	68364840	A	G
NC_040258.1	68378629	T	A
NC_040258.1	68459619	A	C
NC_040258.1	68550260	T	C
NC_040258.1	68592710	T	C
NC_040258.1	68632185	G	A
NC_040258.1	68661618	C	G
NC_040258.1	68686638	G	A
NC_040258.1	68738731	T	C
NC_040258.1	68785959	C	G
NC_040258.1	68846543	T	C
NC_040258.1	68881334	T	C
NC_040258.1	68899511	C	T
NC_040258.1	68956813	C	A
NC_040258.1	69022359	G	T
NC_040258.1	69063923	G	A
NC_040258.1	69110008	C	T
NC_040258.1	69175299	C	T
NC_040258.1	69249662	A	G
NC_040258.1	69305802	G	A
NC_040258.1	69343824	T	C
NC_040258.1	69357771	T	C
NC_040258.1	69410287	G	T
NC_040258.1	69455955	A	G
NC_040258.1	69545712	T	C
NC_040258.1	69618154	G	A
NC_040258.1	69647130	A	G
NC_040258.1	69681594	C	T
NC_040258.1	69720219	C	G
NC_040258.1	69768173	C	T
NC_040258.1	69854845	C	A
NC_040258.1	69939389	A	G
NC_040258.1	69983913	T	C
NC_040258.1	69997609	C	T
NC_040258.1	70052903	G	A
NC_040258.1	70282124	T	G

NC_040258.1	70314261	A		G
NC_040258.1	70375316	T		G
NC_040258.1	70399285	T		C
NC_040258.1	70482465	A		C
NC_040258.1	70691997	C		T
NC_040258.1	70740701	C		T
NC_040258.1	70900113	A		G
NC_040258.1	71035656	C		A
NC_040258.1	71225974	C		T
NC_040258.1	71245215	T		C
NC_040258.1	71288140	T		C
NC_040258.1	71324904	T		C
NC_040258.1	71356101	G		A
NC_040258.1	71388496	A		G
NC_040258.1	71407022	A		G
NC_040258.1	71445092	A		T
NC_040258.1	71480372	C		G
NC_040258.1	71505695	G		T
NC_040258.1	71563939	T		C
NC_040258.1	71616970	C		A
NC_040258.1	71728795	A		C
NC_040258.1	71761736	A		G
NC_040258.1	71818590	A		G
NC_040258.1	71860045	A		G
NC_040258.1	71890391	A		T
NC_040258.1	71923443	A		T
NC_040258.1	71969807	G		A
NC_040258.1	72013441	T		C
NC_040258.1	72044522	A		G
NC_040258.1	72061247	G		A
NC_040258.1	72134453	G		C
NC_040258.1	72301524	T		C
NC_040258.1	72545338	A		G
NC_040258.1	72843348	T		G
NC_040258.1	72966696	C		T
NC_040258.1	72981135	T		G
NC_040258.1	73006636	T		C
NC_040258.1	73034092	T		C
NC_040258.1	73063855	T		C
NC_040258.1	73090566	A		G
NC_040258.1	73160651	C		T
NC_040258.1	73442325	A		T
NC_040258.1	73510822	C		G
NC_040258.1	73615139	A		G
NC_040258.1	73721355	T		G
NC_040258.1	74138468		0	A
NC_040258.1	74263714	T		C

NC_040258.1	74293134	G	T
NC_040258.1	74324201	T	C
NC_040258.1	74388647	G	A
NC_040258.1	74433984	G	A
NC_040258.1	74468986	G	A
NC_040258.1	74491225	G	A
NC_040258.1	74527316	C	T
NC_040258.1	74591279	C	T
NC_040258.1	74613012	T	C
NC_040258.1	74668948	C	T
NC_040258.1	74690595	G	A
NC_040258.1	74741015	A	G
NC_040258.1	74812784	A	G
NC_040258.1	74951198	A	T
NC_040258.1	75007537	A	G
NC_040258.1	75016527	G	C
NC_040258.1	75172760	G	A
NC_040258.1	75209653	G	A
NC_040258.1	75222346	A	T
NC_040258.1	75311235	A	G
NC_040258.1	75368896	G	A
NC_040258.1	75396833	G	C
NC_040258.1	75430062	T	C
NC_040258.1	75545617	A	G
NC_040258.1	75581908	T	C
NC_040258.1	75596034	A	G
NC_040258.1	75629926	C	A
NC_040258.1	75665837	T	C
NC_040258.1	75676716	T	C
NC_040258.1	75711226	G	T
NC_040258.1	75733218	T	C
NC_040258.1	75862287	C	G
NC_040258.1	75906636	T	C
NC_040258.1	76032725	G	A
NC_040258.1	76056573	T	C
NC_040258.1	76103994	T	C
NC_040258.1	76158991	A	T
NC_040258.1	76202704	T	C
NC_040258.1	76229966	T	C
NC_040258.1	76256007	G	A
NC_040258.1	76326193	G	A
NC_040258.1	76353943	G	C
NC_040258.1	76375574	G	T
NC_040258.1	76458544	A	G
NC_040258.1	76491019	T	G
NC_040258.1	76529454	G	T
NC_040258.1	76585621	A	G

NC_040258.1	76622186	T	A
NC_040258.1	76635018	G	C
NC_040258.1	76713608	C	T
NC_040258.1	76784979	A	G
NC_040258.1	76795330	C	A
NC_040258.1	76854785	A	G
NC_040258.1	76897435	C	A
NC_040258.1	76926719	T	C
NC_040258.1	76974095	T	C
NC_040258.1	77186956	T	C
NC_040258.1	77225415	G	A
NC_040258.1	77248262	A	G
NC_040258.1	77328766	T	G
NC_040258.1	77861333	T	C
NC_040258.1	77970680	A	C
NC_040258.1	78004056	T	G
NC_040258.1	78139750	C	A
NC_040258.1	78312131	C	G
NC_040258.1	78431596	A	T
NC_040258.1	78670379	C	T
NC_040258.1	78756203	T	C
NC_040258.1	78818051	T	C
NC_040258.1	78851394	C	G
NC_040258.1	78885001	G	A
NC_040258.1	78934207	C	G
NC_040258.1	78971236	T	C
NC_040258.1	78988546	T	C
NC_040258.1	79063646	G	A
NC_040258.1	79094086	G	A
NC_040258.1	79106176	G	A
NC_040258.1	79155801	0	A
NC_040258.1	79189766	C	T
NC_040258.1	79212230	T	C
NC_040258.1	79270511	T	C
NC_040258.1	79298486	C	T
NC_040258.1	79313222	C	T
NC_040258.1	79361029	C	T
NC_040258.1	79522882	A	G
NC_040258.1	79572427	A	G
NC_040258.1	79601990	T	G
NC_040258.1	79707153	G	A
NC_040258.1	79982993	A	G
NC_040258.1	80011836	T	C
NC_040258.1	80200706	G	A
NC_040258.1	80261241	G	A
NC_040258.1	80294979	C	T
NC_040258.1	80309087	T	G

NC_040258.1	80343080	A	G
NC_040258.1	80369035	T	C
NC_040258.1	80388603	G	A
NC_040258.1	80420961	T	G
NC_040258.1	80483957	C	T
NC_040258.1	80621754	A	G
NC_040258.1	80658440	C	T
NC_040258.1	80745395	C	T
NC_040258.1	80796840	A	T
NC_040258.1	80864734	T	C
NC_040258.1	80931230	A	T
NC_040258.1	81009008	A	C
NC_040258.1	81060870	A	G
NC_040258.1	81134630	C	G
NC_040258.1	81213877	G	A
NC_040258.1	81253064	T	G
NC_040258.1	81286607	A	T
NC_040258.1	81404663	T	A
NC_040258.1	81431328	T	C
NC_040258.1	81472077	A	G
NC_040258.1	81616827	A	T
NC_040258.1	81792030	G	A
NC_040258.1	82096412	C	T
NC_040258.1	82227350	T	A
NC_040258.1	82528603	A	C
NC_040258.1	82596939	G	A
NC_040258.1	82697366	G	C
NC_040258.1	82755876	A	G
NC_040258.1	82787344	G	C
NC_040258.1	82849752	T	G
NC_040258.1	82870556	C	T
NC_040258.1	82904979	T	G
NC_040258.1	82932348	C	T
NC_040258.1	82941748	T	C
NC_040258.1	82968053	C	T
NC_040258.1	82990172	C	T
NC_040258.1	83037404	G	T
NC_040258.1	83055741	C	T
NC_040258.1	83088308	A	C
NC_040258.1	83141056	T	G
NC_040258.1	83266676	C	A
NC_040258.1	83338380	G	A
NC_040258.1	83367942	G	A
NC_040258.1	83392534	G	T
NC_040258.1	83429866	A	G
NC_040258.1	83455498	A	G
NC_040258.1	83474119	G	T

NC_040258.1	83516575	A	G
NC_040258.1	83554196	G	A
NC_040258.1	83584748	A	G
NC_040258.1	83597753	A	G
NC_040258.1	83635842	C	T
NC_040258.1	83666219	G	A
NC_040258.1	83688878	A	C
NC_040258.1	83750047	T	C
NC_040258.1	83787972	G	A
NC_040258.1	83803453	A	G
NC_040258.1	83862490	A	T
NC_040258.1	83928322	A	G
NC_040258.1	83977408	T	C
NC_040258.1	84033806	T	C
NC_040258.1	84105240	A	G
NC_040258.1	84158574	C	T
NC_040258.1	84197980	T	C
NC_040258.1	84212563	A	G
NC_040258.1	84266542	A	G
NC_040258.1	84302395	A	G
NC_040258.1	84348540	T	C
NC_040258.1	84381259	T	C
NC_040258.1	84393146	A	G
NC_040258.1	84421515	G	C
NC_040258.1	84675692	A	G
NC_040258.1	84702570	T	C
NC_040258.1	84717191	C	A
NC_040258.1	84789137	T	C
NC_040258.1	84819422	C	T
NC_040258.1	84835748	T	C
NC_040258.1	84870794	A	G
NC_040258.1	84878799	T	G
NC_040258.1	84918830	A	C
NC_040258.1	84933828	G	A
NC_040258.1	84969839	T	G
NC_040258.1	85003770	T	G
NC_040258.1	85013120	T	G
NC_040258.1	85052102	A	G
NC_040258.1	85065941	C	A
NC_040258.1	85103060	A	C
NC_040258.1	85133251	G	T
NC_040258.1	85153239	A	T
NC_040258.1	85184475	C	A
NC_040258.1	85204005	A	G
NC_040258.1	85231893	C	T
NC_040258.1	85264701	G	T
NC_040258.1	85291812	A	G

NC_040258.1	85325844	A	G
NC_040258.1	85381509	T	C
NC_040258.1	85426331	C	T
NC_040258.1	85457717	A	G
NC_040258.1	85490886	G	C
NC_040258.1	85606595	A	G
NC_040258.1	85646781	T	C
NC_040258.1	85681985	G	T
NC_040258.1	85734743	T	G
NC_040258.1	85792263	C	T
NC_040258.1	85833431	T	G
NC_040258.1	85846956	G	A
NC_040258.1	85865064	C	T
NC_040258.1	85927367	C	T
NC_040258.1	85999745	A	C
NC_040258.1	86028177	C	T
NC_040258.1	86052300	T	G
NC_040258.1	86101833	G	A
NC_040258.1	86162178	A	G
NC_040258.1	86183854	A	G
NC_040258.1	86264255	A	G
NC_040258.1	86309986	A	G
NC_040258.1	86341744	A	G
NC_040258.1	86383866	G	A
NC_040258.1	86433261	T	C
NC_040258.1	86537384	G	C
NC_040258.1	86579672	C	T
NC_040258.1	86612316	C	T
NC_040258.1	86666158	T	C
NC_040258.1	86689097	G	T
NC_040258.1	86765785	T	C
NC_040258.1	86874651	G	A
NC_040258.1	86926037	C	T
NC_040258.1	86954910	T	C
NC_040258.1	87103362	G	A
NC_040258.1	87165193	G	T
NC_040258.1	87257925	C	T
NC_040258.1	87614120	C	T
NC_040258.1	87692987	C	T
NC_040258.1	87734149	A	G
NC_040258.1	87773071	A	G
NC_040258.1	87785549	T	C
NC_040258.1	87885001	C	T
NC_040258.1	87985492	A	G
NC_040258.1	88028809	G	A
NC_040258.1	88061111	G	A
NC_040258.1	88084503	C	T

NC_040258.1	88127102	T	C
NC_040258.1	88165523	A	G
NC_040258.1	88209048	T	C
NC_040258.1	88257108	T	G
NC_040258.1	88309060	A	C
NC_040258.1	88373899	T	C
NC_040258.1	88430656	A	G
NC_040258.1	88455939	A	G
NC_040258.1	88497357	T	C
NC_040258.1	88635885	A	G
NC_040258.1	88660753	A	C
NC_040258.1	88698251	T	C
NC_040258.1	88770752	A	G
NC_040258.1	88889052	C	T
NC_040258.1	88942475	C	A
NC_040258.1	89048759	A	G
NC_040258.1	89096554	G	A
NC_040258.1	89130872	T	C
NC_040258.1	89304540	A	C
NC_040258.1	89397157	G	A
NC_040258.1	89475600	A	G
NC_040258.1	89526752	T	C
NC_040258.1	89586747	A	G
NC_040258.1	89642006	T	C
NC_040258.1	89672856	G	A
NC_040258.1	89714776	G	T
NC_040258.1	89798231	A	G
NC_040258.1	89835260	A	G
NC_040258.1	89882750	G	A
NC_040258.1	89916740	G	T
NC_040258.1	89958090	T	C
NC_040258.1	89990099	T	C
NC_040258.1	90044638	T	C
NC_040258.1	90119516	C	T
NC_040258.1	90242855	A	G
NC_040258.1	90304358	G	T
NC_040258.1	90361314	A	G
NC_040258.1	90384289	C	A
NC_040258.1	90480841	T	C
NC_040258.1	90541279	C	T
NC_040258.1	90564822	C	A
NC_040258.1	90631739	T	G
NC_040258.1	90695374	T	C
NC_040258.1	90937789	C	T
NC_040258.1	90956576	T	C
NC_040258.1	91007222	A	G
NC_040258.1	91025647	A	G

NC_040258.1	91135996	T	C
NC_040258.1	91166463	T	A
NC_040258.1	91213901	A	T
NC_040258.1	91254102	G	A
NC_040258.1	91288785	A	G
NC_040258.1	91327330	C	T
NC_040258.1	91356820	T	A
NC_040258.1	91445496	A	G
NC_040258.1	91516471	G	A
NC_040258.1	91570529	G	A
NC_040258.1	91666166	A	C
NC_040258.1	91728783	T	C
NC_040258.1	91810337	A	G
NC_040258.1	91845632	C	T
NC_040258.1	91916179	C	T
NC_040258.1	91960268	G	A
NC_040258.1	91999500	G	T
NC_040258.1	92039493	G	A
NC_040258.1	92073121	A	C
NC_040258.1	92118475	A	C
NC_040258.1	92147766	A	G
NC_040258.1	92225694	A	C
NC_040258.1	92255892	A	C
NC_040258.1	92336778	A	C
NC_040258.1	92378772	A	G
NC_040258.1	92422148	G	A
NC_040258.1	92460800	T	C
NC_040258.1	92515464	A	T
NC_040258.1	92553411	C	G
NC_040258.1	92577978	T	A
NC_040258.1	92627851	T	C
NC_040258.1	92680748	T	C
NC_040258.1	92763596	G	A
NC_040258.1	92824288	C	G
NC_040258.1	92841506	T	C
NC_040258.1	92875826	T	C
NC_040258.1	93093520	C	T
NC_040258.1	93130089	G	A
NC_040258.1	93350249	G	A
NC_040258.1	93390715	A	G
NC_040258.1	93420511	T	C
NC_040258.1	93445602	T	G
NC_040258.1	93473138	A	G
NC_040258.1	93522762	C	T
NC_040258.1	93554198	A	G
NC_040258.1	93761884	C	T
NC_040258.1	93839360	C	T

NC_040258.1	93906702	T	C
NC_040258.1	94083532	G	T
NC_040258.1	94322233	A	G
NC_040258.1	94398584	A	G
NC_040258.1	94456747	G	A
NC_040258.1	94568314	G	A
NC_040258.1	94731218	G	A
NC_040258.1	94906020	A	G
NC_040258.1	94966328	A	G
NC_040258.1	95130319	T	C
NC_040258.1	95326277	G	A
NC_040258.1	95369763	A	G
NC_040258.1	95428859	T	C
NC_040258.1	95462607	C	T
NC_040258.1	95498809	G	A
NC_040258.1	95821301	G	A
NC_040258.1	95859675	T	C
NC_040258.1	95888467	T	C
NC_040258.1	95956845	T	C
NC_040258.1	95999815	A	G
NC_040258.1	96097430	G	A
NC_040258.1	96123709	T	C
NC_040258.1	96178753	A	T
NC_040258.1	96214310	A	G
NC_040258.1	96263530	A	G
NC_040258.1	96302728	T	C
NC_040258.1	96378304	A	G
NC_040258.1	96477369	T	C
NC_040258.1	96582577	T	C
NC_040258.1	96632790	A	G
NC_040258.1	96666438	G	T
NC_040258.1	96690868	T	C
NC_040258.1	96739229	C	T
NC_040258.1	96863099	T	C
NC_040258.1	96879164	A	G
NC_040258.1	96936798	T	A
NC_040258.1	97047360	A	G
NC_040258.1	97108346	A	G
NC_040258.1	97151853	T	C
NC_040258.1	97208046	A	G
NC_040258.1	97293476	G	C
NC_040258.1	97395614	G	A
NC_040258.1	97528316	T	G
NC_040258.1	97622204	G	T
NC_040258.1	97663787	T	A
NC_040258.1	97709711	G	A
NC_040258.1	98088676	A	G

NC_040258.1	98109653	T	C
NC_040258.1	98166353	G	A
NC_040258.1	98272954	A	G
NC_040258.1	98311824	A	T
NC_040258.1	98355637	G	C
NC_040258.1	98475609	A	G
NC_040258.1	98874532	A	T
NC_040258.1	98941476	A	G
NC_040258.1	99004113	T	C
NC_040258.1	99040454	T	C
NC_040258.1	99091849	C	T
NC_040258.1	99125622	G	A
NC_040258.1	99259670	G	A
NC_040258.1	99289869	T	A
NC_040258.1	99339149	A	T
NC_040258.1	99694346	C	T
NC_040258.1	99744021	C	T
NC_040258.1	99782222	T	C
NC_040258.1	99904612	T	C
NC_040258.1	99962942	G	A
NC_040258.1	100010756	T	G
NC_040258.1	100075238	T	A
NC_040258.1	100113737	G	A
NC_040258.1	100126496	A	C
NC_040258.1	100163747	A	G
NC_040258.1	100172210	G	T
NC_040258.1	100231898	T	G
NC_040258.1	100258259	T	A
NC_040258.1	100316802	A	T
NC_040258.1	100370547	G	C
NC_040258.1	100463597	A	G
NC_040258.1	100514641	G	A
NC_040258.1	100560792	T	C
NC_040258.1	100594022	C	T
NC_040258.1	100660719	G	A
NC_040258.1	100714440	G	A
NC_040258.1	100744617	T	G
NC_040258.1	100910689	G	A
NC_040258.1	100968669	T	C
NC_040258.1	101053089	C	T
NC_040258.1	101083940	A	G
NC_040258.1	101201102	T	G
NC_040258.1	101236701	T	C
NC_040258.1	101279694	G	A
NC_040258.1	101283329	C	A
NC_040258.1	101317954	A	G
NC_040258.1	101336485	C	G

NC_040258.1	101396000	G	C
NC_040258.1	101440651	A	C
NC_040258.1	101444390	A	G
NC_040258.1	101485161	A	G
NC_040258.1	101527064	A	G
NC_040258.1	101557695	A	G
NC_040258.1	101620949	T	C
NC_040258.1	101673059	T	C
NC_040258.1	101817758	A	C
NC_040258.1	102001677	T	C
NC_040258.1	102021833	C	A
NC_040258.1	102081961	C	A
NC_040258.1	102116964	C	G
NC_040258.1	102246452	A	C
NC_040258.1	102286562	T	C
NC_040258.1	102336468	T	G
NC_040258.1	102343471	T	C
NC_040258.1	102402905	G	A
NC_040258.1	102453008	C	T
NC_040258.1	102500816	G	A
NC_040258.1	102543343	C	T
NC_040258.1	102604451	A	G
NC_040258.1	102628648	G	C
NC_040258.1	102704426	T	G
NC_040258.1	102763172	A	G
NC_040258.1	102821273	G	A
NC_040258.1	102907580	G	A
NC_040258.1	102955795	A	G
NC_040258.1	102998553	T	C
NC_040258.1	103051703	G	T
NC_040258.1	103071275	T	G
NC_040258.1	103269983	T	C
NC_040258.1	103328344	T	C
NC_040258.1	103543991	C	T
NC_040258.1	103604931	C	G
NC_040258.1	103630277	A	C
NC_040258.1	103704691	G	C
NC_040258.1	103744825	G	A
NC_040258.1	103837330	G	A
NC_040258.1	103882724	G	A
NC_040258.1	103917624	A	G
NC_040258.1	103938726	A	G
NC_040258.1	104020944	T	C
NC_040258.1	104057696	T	C
NC_040258.1	104098789	G	C
NC_040258.1	104201834	C	T
NC_040258.1	104249234	A	C

NC_040258.1	104287619	T	C
NC_040258.1	104335361	A	C
NC_040258.1	104387802	G	A
NC_040258.1	104446063	A	C
NC_040258.1	104511597	T	C
NC_040258.1	104536867	C	G
NC_040258.1	104562236	C	T
NC_040258.1	104598314	A	G
NC_040258.1	104637255	T	C
NC_040258.1	104687007	C	A
NC_040258.1	104738997	T	G
NC_040258.1	104793531	T	C
NC_040258.1	104851802	C	T
NC_040258.1	104918044	G	C
NC_040258.1	104983207	A	T
NC_040258.1	105012509	A	G
NC_040258.1	105027608	T	G
NC_040258.1	105058150	A	C
NC_040258.1	105078592	C	G
NC_040258.1	105134192	T	C
NC_040258.1	105136133	G	T
NC_040258.1	105177990	T	C
NC_040258.1	105193610	T	C
NC_040258.1	105261810	T	C
NC_040258.1	105322154	C	T
NC_040258.1	105376160	A	G
NC_040258.1	105388062	A	G
NC_040258.1	105421416	C	T
NC_040258.1	105464334	G	C
NC_040258.1	105527107	C	A
NC_040258.1	105583897	C	T
NC_040258.1	105629667	T	C
NC_040258.1	105680702	G	A
NC_040258.1	105791898	T	C
NC_040258.1	105957035	A	G
NC_040258.1	105993323	C	T
NC_040258.1	106270446	T	C
NC_040258.1	106328761	T	C
NC_040258.1	106348026	G	A
NC_040258.1	106359695	C	T
NC_040258.1	106410430	A	G
NC_040258.1	106465220	A	G
NC_040258.1	106483728	C	T
NC_040258.1	106522535	T	C
NC_040258.1	106569108	A	G
NC_040258.1	106599501	C	T
NC_040258.1	106649467	A	G

NC_040258.1	106662401	A	T
NC_040258.1	106727558	G	A
NC_040258.1	106756863	A	C
NC_040258.1	106824155	G	A
NC_040258.1	106871828	A	G
NC_040258.1	106886534	A	C
NC_040258.1	106931911	C	T
NC_040258.1	106944568	T	C
NC_040258.1	107008466	A	G
NC_040258.1	107087534	A	G
NC_040258.1	107122181	T	C
NC_040258.1	107176895	G	C
NC_040258.1	107231107	T	G
NC_040258.1	107269002	C	T
NC_040258.1	107284359	A	G
NC_040258.1	107347008	A	G
NC_040258.1	107393149	T	C
NC_040258.1	107417837	T	C
NC_040258.1	107435201	A	G
NC_040258.1	107436726	A	G
NC_040258.1	107438281	C	T
NC_040258.1	107452616	T	C
NC_040258.1	107453974	C	T
NC_040258.1	107466733	T	C
NC_040258.1	107466884	A	G
NC_040258.1	107467258	A	G
NC_040258.1	107482855	C	T
NC_040258.1	107483019	T	C
NC_040258.1	107489679	T	C
NC_040258.1	107541853	C	T
NC_040258.1	107604769	C	T
NC_040258.1	107658211	C	A
NC_040259.1	156326	A	G
NC_040259.1	238029	A	G
NC_040259.1	242760	C	G
NC_040259.1	250533	G	C
NC_040259.1	263212	A	G
NC_040259.1	263521	G	C
NC_040259.1	264202	A	G
NC_040259.1	264673	A	G
NC_040259.1	283330	T	A
NC_040259.1	351238	C	A
NC_040259.1	411026	A	G
NC_040259.1	442526	A	G
NC_040259.1	477627	T	G
NC_040259.1	479137	T	G
NC_040259.1	538864	T	C

NC_040259.1	573235	G	T
NC_040259.1	592574	T	C
NC_040259.1	615030	T	C
NC_040259.1	615587	A	G
NC_040259.1	651291	C	A
NC_040259.1	695665	C	T
NC_040259.1	763848	G	T
NC_040259.1	881133	C	T
NC_040259.1	935718	G	A
NC_040259.1	981702	G	A
NC_040259.1	1022207	C	A
NC_040259.1	1110949	T	C
NC_040259.1	1164514	C	T
NC_040259.1	1211145	G	A
NC_040259.1	1248629	T	G
NC_040259.1	1340375	C	T
NC_040259.1	1434221	C	T
NC_040259.1	1490499	C	T
NC_040259.1	1547397	C	T
NC_040259.1	1602574	G	A
NC_040259.1	1670988	T	A
NC_040259.1	1704427	A	G
NC_040259.1	1887880	T	G
NC_040259.1	1931570	T	G
NC_040259.1	1986665	C	T
NC_040259.1	2034667	G	A
NC_040259.1	2097142	G	C
NC_040259.1	2157708	A	G
NC_040259.1	2208005	G	A
NC_040259.1	2259152	A	G
NC_040259.1	2301138	A	G
NC_040259.1	2312093	A	G
NC_040259.1	2324069	G	A
NC_040259.1	2359259	T	A
NC_040259.1	2398967	G	C
NC_040259.1	2435661	C	T
NC_040259.1	2477650	T	A
NC_040259.1	2482389	G	A
NC_040259.1	2483315	T	C
NC_040259.1	2483684	C	T
NC_040259.1	2530614	C	T
NC_040259.1	2587211	G	A
NC_040259.1	2627834	A	G
NC_040259.1	2794303	T	C
NC_040259.1	2922197	C	G
NC_040259.1	2978651	A	T
NC_040259.1	3025313	A	G

NC_040259.1	3071781	T	C
NC_040259.1	3127701	T	A
NC_040259.1	3182383	C	A
NC_040259.1	3225697	G	A
NC_040259.1	3273191	C	T
NC_040259.1	3314514	C	T
NC_040259.1	3370864	T	C
NC_040259.1	3404093	G	T
NC_040259.1	3466153	A	C
NC_040259.1	3522734	C	T
NC_040259.1	3571281	C	T
NC_040259.1	3625739	G	C
NC_040259.1	3685916	A	G
NC_040259.1	3744980	G	A
NC_040259.1	3783437	T	C
NC_040259.1	3835779	T	C
NC_040259.1	3894745	A	T
NC_040259.1	3944268	C	G
NC_040259.1	3982435	A	G
NC_040259.1	4018708	G	A
NC_040259.1	4069597	A	C
NC_040259.1	4109482	T	C
NC_040259.1	4159809	C	A
NC_040259.1	4206211	C	T
NC_040259.1	4243906	A	C
NC_040259.1	4296620	C	T
NC_040259.1	4352503	A	G
NC_040259.1	4416566	T	C
NC_040259.1	4655968	A	C
NC_040259.1	4704563	T	C
NC_040259.1	4762139	A	G
NC_040259.1	4783050	T	A
NC_040259.1	4834092	A	G
NC_040259.1	4966344	G	A
NC_040259.1	5150869	A	G
NC_040259.1	5200946	C	T
NC_040259.1	5253586	C	T
NC_040259.1	5253792	G	A
NC_040259.1	5255240	A	C
NC_040259.1	5255876	A	G
NC_040259.1	5256080	A	G
NC_040259.1	5256304	G	A
NC_040259.1	5309481	G	A
NC_040259.1	5361502	C	T
NC_040259.1	5440451	T	C
NC_040259.1	5496645	C	T
NC_040259.1	5556820	G	C

NC_040259.1	5641495	T	C
NC_040259.1	5706085	G	A
NC_040259.1	5778527	A	G
NC_040259.1	5832066	T	A
NC_040259.1	5895799	T	G
NC_040259.1	5945372	A	G
NC_040259.1	5987515	G	A
NC_040259.1	6025500	A	G
NC_040259.1	6072838	T	G
NC_040259.1	6135038	A	T
NC_040259.1	6187351	T	C
NC_040259.1	6245427	T	G
NC_040259.1	6307215	A	T
NC_040259.1	6357907	G	A
NC_040259.1	6413382	T	C
NC_040259.1	6463319	G	A
NC_040259.1	6508892	T	A
NC_040259.1	6560086	A	G
NC_040259.1	6620975	G	A
NC_040259.1	6652189	T	G
NC_040259.1	6687229	C	A
NC_040259.1	6694433	T	C
NC_040259.1	6737978	A	G
NC_040259.1	6738806	T	G
NC_040259.1	6761406	A	G
NC_040259.1	6771120	A	G
NC_040259.1	6828076	C	T
NC_040259.1	6861063	G	C
NC_040259.1	6916923	G	T
NC_040259.1	6975582	G	A
NC_040259.1	7029740	G	C
NC_040259.1	7084879	T	C
NC_040259.1	7141251	C	T
NC_040259.1	7191766	G	T
NC_040259.1	7252324	A	G
NC_040259.1	7316226	G	T
NC_040259.1	7316558	G	T
NC_040259.1	7319689	A	G
NC_040259.1	7321641	T	C
NC_040259.1	7368824	C	T
NC_040259.1	7425157	T	C
NC_040259.1	7487340	G	A
NC_040259.1	7552557	T	C
NC_040259.1	7599419	T	C
NC_040259.1	7661406	T	G
NC_040259.1	7860960	C	T
NC_040259.1	7906671	G	A

NC_040259.1	7938062	G	A
NC_040259.1	7977418	T	C
NC_040259.1	8040327	G	C
NC_040259.1	8044603	T	C
NC_040259.1	8066385	G	A
NC_040259.1	8077652	T	G
NC_040259.1	8142962	G	C
NC_040259.1	8190831	G	A
NC_040259.1	8272919	G	C
NC_040259.1	8328544	T	C
NC_040259.1	8376732	G	T
NC_040259.1	8433025	A	G
NC_040259.1	8485470	A	G
NC_040259.1	8543468	T	C
NC_040259.1	8598722	C	A
NC_040259.1	8653920	A	G
NC_040259.1	8709415	A	G
NC_040259.1	8749214	A	G
NC_040259.1	8799303	C	A
NC_040259.1	8848693	G	T
NC_040259.1	8906684	C	T
NC_040259.1	8963281	A	G
NC_040259.1	9022586	C	G
NC_040259.1	9086986	G	A
NC_040259.1	9146124	A	G
NC_040259.1	9201201	T	C
NC_040259.1	9243355	A	G
NC_040259.1	9293957	T	C
NC_040259.1	9352943	C	G
NC_040259.1	9403920	G	T
NC_040259.1	9464151	A	C
NC_040259.1	9518372	T	C
NC_040259.1	9576636	T	C
NC_040259.1	9634846	C	T
NC_040259.1	9679724	A	G
NC_040259.1	9737107	C	A
NC_040259.1	9795907	C	T
NC_040259.1	9855623	T	C
NC_040259.1	9904662	G	A
NC_040259.1	9905456	A	C
NC_040259.1	9906263	A	T
NC_040259.1	9906456	A	G
NC_040259.1	9906890	T	C
NC_040259.1	9909133	T	C
NC_040259.1	9909530	T	G
NC_040259.1	9980180	T	C
NC_040259.1	10119469	G	A

NC_040259.1	10177454	A	G
NC_040259.1	10234928	A	G
NC_040259.1	10281940	G	A
NC_040259.1	10331964	C	A
NC_040259.1	10410598	A	G
NC_040259.1	10467217	G	C
NC_040259.1	10503017	C	G
NC_040259.1	10503681	T	C
NC_040259.1	10561546	G	A
NC_040259.1	10618178	T	C
NC_040259.1	10652898	A	C
NC_040259.1	10700020	T	C
NC_040259.1	10700933	T	C
NC_040259.1	10753829	C	T
NC_040259.1	10806880	C	G
NC_040259.1	10863774	T	C
NC_040259.1	10925633	G	A
NC_040259.1	10991443	G	A
NC_040259.1	11042712	C	T
NC_040259.1	11094032	C	T
NC_040259.1	11163136	G	A
NC_040259.1	11218372	G	T
NC_040259.1	11270878	C	T
NC_040259.1	11325249	T	C
NC_040259.1	11380030	T	C
NC_040259.1	11420164	A	G
NC_040259.1	11470137	A	G
NC_040259.1	11525517	A	G
NC_040259.1	11525721	T	A
NC_040259.1	11535386	A	G
NC_040259.1	11565951	C	G
NC_040259.1	11568248	G	T
NC_040259.1	11568944	G	A
NC_040259.1	11569156	T	C
NC_040259.1	11613423	C	T
NC_040259.1	11665536	G	A
NC_040259.1	11718576	G	A
NC_040259.1	11770376	A	G
NC_040259.1	11822821	A	G
NC_040259.1	11873762	C	T
NC_040259.1	11908577	A	G
NC_040259.1	12016730	T	G
NC_040259.1	12032835	C	T
NC_040259.1	12033117	A	G
NC_040259.1	12033651	T	C
NC_040259.1	12034425	G	A
NC_040259.1	12034642	T	A

NC_040259.1	12092611	A	G
NC_040259.1	12141198	C	T
NC_040259.1	12187642	T	C
NC_040259.1	12226740	T	C
NC_040259.1	12290988	G	A
NC_040259.1	12338043	A	G
NC_040259.1	12356986	A	G
NC_040259.1	12357750	A	G
NC_040259.1	12358839	A	C
NC_040259.1	12360208	T	C
NC_040259.1	12400457	T	C
NC_040259.1	12433491	A	G
NC_040259.1	12433740	A	G
NC_040259.1	12457149	C	T
NC_040259.1	12713096	T	C
NC_040259.1	12723394	C	T
NC_040259.1	12777305	A	T
NC_040259.1	12797111	A	C
NC_040259.1	12837643	T	C
NC_040259.1	12873231	T	C
NC_040259.1	12926517	G	A
NC_040259.1	12959969	T	C
NC_040259.1	13016041	G	A
NC_040259.1	13071344	C	T
NC_040259.1	13127777	T	C
NC_040259.1	13185150	A	G
NC_040259.1	13228971	T	A
NC_040259.1	13284532	T	C
NC_040259.1	13340824	A	G
NC_040259.1	13386681	A	G
NC_040259.1	13454650	C	T
NC_040259.1	13514801	G	T
NC_040259.1	13571393	G	T
NC_040259.1	13627789	T	C
NC_040259.1	13666606	A	G
NC_040259.1	13706551	C	T
NC_040259.1	13714405	A	G
NC_040259.1	13770303	T	C
NC_040259.1	13824488	A	G
NC_040259.1	13879789	A	G
NC_040259.1	13940927	G	C
NC_040259.1	13999005	T	A
NC_040259.1	13999393	A	G
NC_040259.1	14000043	G	A
NC_040259.1	14034249	C	A
NC_040259.1	14069027	A	C
NC_040259.1	14070695	T	C

NC_040259.1	14102281	A	G
NC_040259.1	14104016	A	G
NC_040259.1	14148227	A	T
NC_040259.1	14259036	C	T
NC_040259.1	14315674	A	T
NC_040259.1	14369578	G	A
NC_040259.1	14423069	G	T
NC_040259.1	14473483	G	A
NC_040259.1	14528774	G	A
NC_040259.1	14579631	G	A
NC_040259.1	14635913	A	G
NC_040259.1	14693238	T	G
NC_040259.1	14755158	G	A
NC_040259.1	14812149	T	C
NC_040259.1	14857231	A	G
NC_040259.1	14859619	A	G
NC_040259.1	14862753	G	A
NC_040259.1	14865449	C	G
NC_040259.1	14993845	A	C
NC_040259.1	15045489	C	T
NC_040259.1	15098505	G	A
NC_040259.1	15143716	G	A
NC_040259.1	15189152	C	T
NC_040259.1	15189312	T	C
NC_040259.1	15189923	C	T
NC_040259.1	15247779	G	A
NC_040259.1	15302722	T	C
NC_040259.1	15360318	T	C
NC_040259.1	15419623	G	A
NC_040259.1	15464279	C	A
NC_040259.1	15509305	C	T
NC_040259.1	15564930	T	C
NC_040259.1	15623831	C	T
NC_040259.1	15687506	C	T
NC_040259.1	15741128	C	A
NC_040259.1	15785336	T	G
NC_040259.1	15837061	A	G
NC_040259.1	15901593	T	G
NC_040259.1	15955375	G	A
NC_040259.1	16011621	T	C
NC_040259.1	16052260	T	G
NC_040259.1	16097308	C	T
NC_040259.1	16111004	A	G
NC_040259.1	16165498	C	T
NC_040259.1	16218186	T	C
NC_040259.1	16238513	G	A
NC_040259.1	16291481	T	C

NC_040259.1	16340530	G		T
NC_040259.1	16386606	A		G
NC_040259.1	16439076	C		A
NC_040259.1	16489426	T		C
NC_040259.1	16543022	A		G
NC_040259.1	16592930	A		G
NC_040259.1	16657852	G		A
NC_040259.1	16709400	G		A
NC_040259.1	16764223	A		G
NC_040259.1	16805103	G		A
NC_040259.1	16863046	A		G
NC_040259.1	16918683	G		A
NC_040259.1	16975461	A		G
NC_040259.1	17022425	T		C
NC_040259.1	17067289	A		G
NC_040259.1	17070416	T		C
NC_040259.1	17070965	G		A
NC_040259.1	17071395	G		A
NC_040259.1	17071953	A		G
NC_040259.1	17124733	G		A
NC_040259.1	17146869	T		C
NC_040259.1	17147075	A		G
NC_040259.1	17202858	T		C
NC_040259.1	17262405	C		A
NC_040259.1	17327450	C		A
NC_040259.1	17384200	A		G
NC_040259.1	17442417		0	A
NC_040259.1	17490612	C		T
NC_040259.1	17547255	G		A
NC_040259.1	17557324	G		C
NC_040259.1	17563799	T		G
NC_040259.1	17570341	C		T
NC_040259.1	17576550	T		C
NC_040259.1	17576820	T		C
NC_040259.1	17577120	T		C
NC_040259.1	17630850	T		G
NC_040259.1	17672817	T		G
NC_040259.1	17733084	G		A
NC_040259.1	17789489	G		A
NC_040259.1	17850563	G		A
NC_040259.1	17903528	G		A
NC_040259.1	17962202	G		A
NC_040259.1	18017626	A		G
NC_040259.1	18069948	T		C
NC_040259.1	18128363	A		C
NC_040259.1	18184009	C		T
NC_040259.1	18240995	C		T

NC_040259.1	18299360	A	G
NC_040259.1	18342098	A	T
NC_040259.1	18438558	G	A
NC_040259.1	18495020	C	T
NC_040259.1	18551228	G	A
NC_040259.1	18608120	C	T
NC_040259.1	18659422	G	C
NC_040259.1	18703261	G	A
NC_040259.1	18754422	C	A
NC_040259.1	18803179	C	G
NC_040259.1	18852177	G	C
NC_040259.1	18914549	A	G
NC_040259.1	18962746	G	A
NC_040259.1	19012590	A	G
NC_040259.1	19066119	T	C
NC_040259.1	19123315	A	G
NC_040259.1	19150243	C	T
NC_040259.1	19400538	T	G
NC_040259.1	19453014	G	A
NC_040259.1	19485653	G	A
NC_040259.1	19540495	A	G
NC_040259.1	19596726	A	G
NC_040259.1	19812503	T	A
NC_040259.1	19868933	A	C
NC_040259.1	19930850	C	T
NC_040259.1	19986795	A	C
NC_040259.1	20046478	A	G
NC_040259.1	20106098	A	C
NC_040259.1	20136894	T	C
NC_040259.1	20403649	G	A
NC_040259.1	20456256	A	G
NC_040259.1	20571436	C	T
NC_040259.1	20625306	C	T
NC_040259.1	20682115	C	T
NC_040259.1	20733941	T	C
NC_040259.1	20790228	C	G
NC_040259.1	20846243	A	G
NC_040259.1	20900742	T	A
NC_040259.1	20955775	G	A
NC_040259.1	21013869	G	A
NC_040259.1	21034556	T	C
NC_040259.1	21093371	C	A
NC_040259.1	21145954	C	T
NC_040259.1	21200836	T	A
NC_040259.1	21256052	A	G
NC_040259.1	21312093	G	A
NC_040259.1	21367746	G	A

NC_040259.1	21415277	A	G
NC_040259.1	21416964	A	G
NC_040259.1	21480510	T	C
NC_040259.1	21482266	A	C
NC_040259.1	21526986	C	T
NC_040259.1	21531780	T	C
NC_040259.1	21582579	A	G
NC_040259.1	21641733	A	G
NC_040259.1	21705135	T	C
NC_040259.1	21757710	G	A
NC_040259.1	21786649	A	T
NC_040259.1	21787700	A	G
NC_040259.1	21787928	G	A
NC_040259.1	21840521	G	A
NC_040259.1	21861694	T	C
NC_040259.1	21863224	C	A
NC_040259.1	21863392	0	G
NC_040259.1	21864132	A	G
NC_040259.1	21923450	A	G
NC_040259.1	21975774	C	G
NC_040259.1	22021112	A	G
NC_040259.1	22074039	A	C
NC_040259.1	22131958	T	C
NC_040259.1	22182757	T	C
NC_040259.1	22237397	T	C
NC_040259.1	22285058	G	A
NC_040259.1	22338561	A	C
NC_040259.1	22376707	T	C
NC_040259.1	22431101	T	C
NC_040259.1	22473942	G	A
NC_040259.1	22495385	T	C
NC_040259.1	22527984	G	C
NC_040259.1	22598061	G	A
NC_040259.1	22598807	C	T
NC_040259.1	22617339	T	C
NC_040259.1	22617934	C	T
NC_040259.1	22618668	G	A
NC_040259.1	22624394	A	G
NC_040259.1	22625081	T	C
NC_040259.1	22680152	C	T
NC_040259.1	22730571	C	T
NC_040259.1	22775528	C	T
NC_040259.1	22802982	A	C
NC_040259.1	22855899	A	G
NC_040259.1	22856412	T	C
NC_040259.1	22856669	T	C
NC_040259.1	22876086	A	G

NC_040259.1	22877070	A	G
NC_040259.1	22925084	A	G
NC_040259.1	22976186	T	C
NC_040259.1	23028401	G	C
NC_040259.1	23070440	A	G
NC_040259.1	23112309	G	A
NC_040259.1	23169722	A	G
NC_040259.1	23196990	A	G
NC_040259.1	23202170	T	C
NC_040259.1	23237401	A	C
NC_040259.1	23238805	C	T
NC_040259.1	23285556	C	T
NC_040259.1	23289731	A	G
NC_040259.1	23290013	A	G
NC_040259.1	23301914	T	C
NC_040259.1	23306620	C	T
NC_040259.1	23341857	A	G
NC_040259.1	23375709	A	G
NC_040259.1	23375948	C	T
NC_040259.1	23440412	C	A
NC_040259.1	23503507	C	T
NC_040259.1	23506116	A	G
NC_040259.1	23533131	T	C
NC_040259.1	23537966	G	C
NC_040259.1	23579815	A	G
NC_040259.1	23586980	C	T
NC_040259.1	23732747	C	T
NC_040259.1	23809403	A	G
NC_040259.1	23820703	G	A
NC_040259.1	23821495	A	G
NC_040259.1	23866602	T	C
NC_040259.1	23866975	C	A
NC_040259.1	23918930	T	C
NC_040259.1	23964786	A	C
NC_040259.1	23965023	A	G
NC_040259.1	23982877	G	C
NC_040259.1	24009845	T	C
NC_040259.1	24049694	T	C
NC_040259.1	24087166	G	A
NC_040259.1	24087383	C	G
NC_040259.1	24087554	T	C
NC_040259.1	24088854	T	A
NC_040259.1	24107766	C	G
NC_040259.1	24152633	A	C
NC_040259.1	24202289	G	A
NC_040259.1	24240591	A	G
NC_040259.1	24293520	G	A

NC_040259.1	24358290	T	C
NC_040259.1	24420273	T	C
NC_040259.1	24469824	T	G
NC_040259.1	24516283	G	A
NC_040259.1	24554778	C	A
NC_040259.1	24614220	G	A
NC_040259.1	24665387	A	G
NC_040259.1	24690442	G	A
NC_040259.1	24763785	T	C
NC_040259.1	24817343	T	C
NC_040259.1	24875346	C	T
NC_040259.1	24937066	G	C
NC_040259.1	24998520	T	C
NC_040259.1	25259088	A	T
NC_040259.1	25314057	A	G
NC_040259.1	25370974	T	G
NC_040259.1	25425414	G	T
NC_040259.1	25476705	A	G
NC_040259.1	25533864	C	T
NC_040259.1	25587180	G	A
NC_040259.1	25641414	G	A
NC_040259.1	25697121	C	G
NC_040259.1	25739306	T	A
NC_040259.1	25790864	C	T
NC_040259.1	25841010	G	A
NC_040259.1	25895065	G	A
NC_040259.1	25948796	A	G
NC_040259.1	26005696	G	A
NC_040259.1	26061170	C	T
NC_040259.1	26116758	G	A
NC_040259.1	26172304	C	T
NC_040259.1	26208096	A	G
NC_040259.1	26209841	G	A
NC_040259.1	26266311	T	G
NC_040259.1	26325514	T	C
NC_040259.1	26379963	T	C
NC_040259.1	26382047	G	T
NC_040259.1	26382760	A	G
NC_040259.1	26436312	A	G
NC_040259.1	26494598	A	C
NC_040259.1	26550625	A	T
NC_040259.1	26607623	T	C
NC_040259.1	26661003	G	T
NC_040259.1	26704854	G	A
NC_040259.1	26868000	G	A
NC_040259.1	26920522	C	T
NC_040259.1	26973213	C	T

NC_040259.1	27005230	C	A
NC_040259.1	27053908	G	T
NC_040259.1	27121184	T	C
NC_040259.1	27168296	G	C
NC_040259.1	27223460	A	G
NC_040259.1	27279055	T	G
NC_040259.1	27340587	C	T
NC_040259.1	27402038	C	T
NC_040259.1	27468483	C	T
NC_040259.1	27520803	T	A
NC_040259.1	27565571	C	T
NC_040259.1	27622075	T	G
NC_040259.1	27682965	C	T
NC_040259.1	27739625	G	A
NC_040259.1	27791193	T	C
NC_040259.1	27847928	G	A
NC_040259.1	27891247	A	G
NC_040259.1	27946405	T	C
NC_040259.1	27998294	A	G
NC_040259.1	28048906	T	C
NC_040259.1	28100687	C	G
NC_040259.1	28153320	A	C
NC_040259.1	28191767	G	A
NC_040259.1	28243701	C	T
NC_040259.1	28292241	A	G
NC_040259.1	28333888	A	T
NC_040259.1	28335462	T	C
NC_040259.1	28340119	A	G
NC_040259.1	28381576	G	C
NC_040259.1	28431769	C	T
NC_040259.1	28487563	C	T
NC_040259.1	28537776	C	T
NC_040259.1	28567077	G	C
NC_040259.1	28856934	G	A
NC_040259.1	28910416	A	T
NC_040259.1	28976953	C	T
NC_040259.1	29027334	C	T
NC_040259.1	29083064	T	C
NC_040259.1	29139675	G	A
NC_040259.1	29181924	G	A
NC_040259.1	29228748	G	T
NC_040259.1	29230860	C	G
NC_040259.1	29231392	G	C
NC_040259.1	29300048	C	T
NC_040259.1	29342829	T	C
NC_040259.1	29363002	C	T
NC_040259.1	29367131	A	G

NC_040259.1	29367661	G	T
NC_040259.1	29368881	C	T
NC_040259.1	29422857	C	T
NC_040259.1	29478779	T	C
NC_040259.1	29523305	A	G
NC_040259.1	29593650	G	T
NC_040259.1	29631054	A	G
NC_040259.1	29685960	T	C
NC_040259.1	29744780	G	A
NC_040259.1	29794763	C	T
NC_040259.1	29841255	G	A
NC_040259.1	29884819	G	A
NC_040259.1	29885899	C	T
NC_040259.1	29887158	A	G
NC_040259.1	29942820	T	C
NC_040259.1	29997756	A	G
NC_040259.1	30038684	T	C
NC_040259.1	30087886	A	T
NC_040259.1	30137005	T	C
NC_040259.1	30182798	A	T
NC_040259.1	30215633	A	G
NC_040259.1	30273121	G	C
NC_040259.1	30329390	G	A
NC_040259.1	30381702	G	A
NC_040259.1	30434889	A	G
NC_040259.1	30504720	G	A
NC_040259.1	30562712	C	T
NC_040259.1	30614455	G	T
NC_040259.1	30672173	A	G
NC_040259.1	30704561	A	G
NC_040259.1	30916846	T	C
NC_040259.1	30949284	G	A
NC_040259.1	31010414	C	T
NC_040259.1	31046292	C	G
NC_040259.1	31089399	T	A
NC_040259.1	31093154	C	T
NC_040259.1	31131002	C	G
NC_040259.1	31131639	A	G
NC_040259.1	31175511	C	A
NC_040259.1	31226183	C	G
NC_040259.1	31284972	G	A
NC_040259.1	31339791	T	C
NC_040259.1	31397136	A	G
NC_040259.1	31450391	G	C
NC_040259.1	31489708	T	C
NC_040259.1	31524667	A	G
NC_040259.1	31583257	G	C

NC_040259.1	31624842	C	T
NC_040259.1	31662910	A	G
NC_040259.1	31719857	G	A
NC_040259.1	31780979	T	C
NC_040259.1	31845794	C	T
NC_040259.1	31847314	A	T
NC_040259.1	31848585	C	T
NC_040259.1	31906024	T	C
NC_040259.1	31965516	A	G
NC_040259.1	32021031	A	C
NC_040259.1	32077914	T	C
NC_040259.1	32135006	G	A
NC_040259.1	32196080	A	G
NC_040259.1	32235413	G	A
NC_040259.1	32270278	A	G
NC_040259.1	32288810	T	G
NC_040259.1	32298492	G	A
NC_040259.1	32355051	T	A
NC_040259.1	32409619	A	G
NC_040259.1	32436969	C	T
NC_040259.1	32499176	G	A
NC_040259.1	32539382	G	A
NC_040259.1	32540241	C	T
NC_040259.1	32557552	T	C
NC_040259.1	32562774	G	T
NC_040259.1	32564200	C	G
NC_040259.1	32619050	T	C
NC_040259.1	32648085	A	G
NC_040259.1	32668326	T	C
NC_040259.1	32723480	C	G
NC_040259.1	32755625	A	C
NC_040259.1	32775004	T	C
NC_040259.1	32832702	A	G
NC_040259.1	32886161	A	G
NC_040259.1	32943625	T	C
NC_040259.1	33000722	G	A
NC_040259.1	33057490	T	A
NC_040259.1	33115209	T	C
NC_040259.1	33168355	C	T
NC_040259.1	33212210	G	C
NC_040259.1	33253938	G	A
NC_040259.1	33295617	T	C
NC_040259.1	33448077	A	G
NC_040259.1	33450185	C	T
NC_040259.1	33451567	C	G
NC_040259.1	33507419	A	C
NC_040259.1	33562631	T	C

NC_040259.1	33619294	A	G
NC_040259.1	33675702	G	C
NC_040259.1	33732508	T	A
NC_040259.1	33788897	T	C
NC_040259.1	33843678	A	G
NC_040259.1	33844486	C	A
NC_040259.1	33844845	A	G
NC_040259.1	33869819	C	T
NC_040259.1	33917162	C	T
NC_040259.1	33971025	T	C
NC_040259.1	34027258	C	T
NC_040259.1	34083807	A	T
NC_040259.1	34142122	T	C
NC_040259.1	34191183	T	G
NC_040259.1	34248051	A	G
NC_040259.1	34248633	T	C
NC_040259.1	34251435	C	G
NC_040259.1	34268545	T	G
NC_040259.1	34269758	G	A
NC_040259.1	34326417	C	T
NC_040259.1	34381444	T	C
NC_040259.1	34436588	G	C
NC_040259.1	34478633	T	C
NC_040259.1	34524146	C	A
NC_040259.1	34745184	C	A
NC_040259.1	34799102	T	C
NC_040259.1	34860451	A	T
NC_040259.1	34916527	G	T
NC_040259.1	34972302	C	T
NC_040259.1	35026587	G	T
NC_040259.1	35081164	A	G
NC_040259.1	35139135	G	C
NC_040259.1	35189630	T	C
NC_040259.1	35240022	T	C
NC_040259.1	35284452	T	C
NC_040259.1	35331086	C	G
NC_040259.1	35385980	A	C
NC_040259.1	35447053	G	A
NC_040259.1	35502165	A	C
NC_040259.1	35554453	T	C
NC_040259.1	35613096	G	A
NC_040259.1	35667797	A	C
NC_040259.1	35717145	C	T
NC_040259.1	35771816	C	T
NC_040259.1	35826439	A	G
NC_040259.1	35885031	A	G
NC_040259.1	35940077	A	G

NC_040259.1	35994760	G	A
NC_040259.1	36053386	G	T
NC_040259.1	36122088	G	A
NC_040259.1	36170019	A	G
NC_040259.1	36218151	G	A
NC_040259.1	36274371	T	G
NC_040259.1	36333448	A	G
NC_040259.1	36372298	T	C
NC_040259.1	36408315	G	C
NC_040259.1	36480093	T	C
NC_040259.1	36536733	A	C
NC_040259.1	36579701	G	A
NC_040259.1	36619684	T	C
NC_040259.1	37321318	A	G
NC_040259.1	37774864	A	G
NC_040259.1	37816822	T	G
NC_040259.1	37858469	G	A
NC_040259.1	37918482	A	G
NC_040259.1	37963920	A	G
NC_040259.1	37997064	C	G
NC_040259.1	38050809	G	A
NC_040259.1	38101516	C	T
NC_040259.1	38156094	A	G
NC_040259.1	38214617	C	T
NC_040259.1	38266831	A	T
NC_040259.1	38310321	C	T
NC_040259.1	38359749	G	A
NC_040259.1	38389528	A	G
NC_040259.1	38447335	T	C
NC_040259.1	38518947	T	C
NC_040259.1	38570983	T	C
NC_040259.1	38624036	A	G
NC_040259.1	38661147	A	G
NC_040259.1	38716244	A	T
NC_040259.1	38779117	G	A
NC_040259.1	38845843	A	G
NC_040259.1	38893265	A	G
NC_040259.1	38925468	C	A
NC_040259.1	39056802	G	A
NC_040259.1	39154797	C	T
NC_040259.1	39202503	A	C
NC_040259.1	39416661	T	C
NC_040259.1	39661667	A	C
NC_040259.1	39703749	C	A
NC_040259.1	39759159	C	T
NC_040259.1	39808053	A	T
NC_040259.1	39842709	T	C

NC_040259.1	40079488	T		A
NC_040259.1	40124648	C		T
NC_040259.1	40181451	T		A
NC_040259.1	40229299	T		C
NC_040259.1	40283856	A		G
NC_040259.1	40339633	G		A
NC_040259.1	40395571	A		C
NC_040259.1	40448557	G		T
NC_040259.1	40496441	T		C
NC_040259.1	40550584	G		A
NC_040259.1	40605051	A		G
NC_040259.1	40651439	G		A
NC_040259.1	40706535	A		G
NC_040259.1	40762839	C		A
NC_040259.1	40817996	G		A
NC_040259.1	40874024	T		A
NC_040259.1	40915331	A		G
NC_040259.1	40978149	C		T
NC_040259.1	41030424	C		A
NC_040259.1	41079221	G		A
NC_040259.1	41114397	G		A
NC_040259.1	41161294	T		C
NC_040259.1	41215754	A		G
NC_040259.1	41272672	A		T
NC_040259.1	41330377	T		C
NC_040259.1	41388104		0	A
NC_040259.1	41444936	A		G
NC_040259.1	41472114	C		G
NC_040259.1	41542895	T		C
NC_040259.1	41544144	C		T
NC_040259.1	41544827	A		G
NC_040259.1	41602623	G		A
NC_040259.1	41657320	C		T
NC_040259.1	41710399	A		G
NC_040259.1	41761335	G		A
NC_040259.1	41813447	G		A
NC_040259.1	41873532	C		A
NC_040259.1	41929621	T		C
NC_040259.1	41977537	C		T
NC_040259.1	42027838	C		T
NC_040259.1	42079985	G		A
NC_040259.1	42141698	A		C
NC_040259.1	42199228	T		G
NC_040259.1	42202153	G		A
NC_040259.1	42256016	A		T
NC_040259.1	42314984	A		G
NC_040259.1	42318506	G		A

NC_040259.1	42366259	A	C
NC_040259.1	42368605	T	C
NC_040259.1	42428911	A	G
NC_040259.1	42485200	A	G
NC_040259.1	42487863	A	G
NC_040259.1	42489957	T	G
NC_040259.1	42544355	C	T
NC_040259.1	42599190	A	G
NC_040259.1	42656633	C	G
NC_040259.1	42713391	T	C
NC_040259.1	42750383	C	T
NC_040259.1	42799650	C	G
NC_040259.1	42839764	C	T
NC_040259.1	42873785	C	T
NC_040259.1	42874485	G	A
NC_040259.1	42907301	C	T
NC_040259.1	42909938	A	C
NC_040259.1	42912233	G	A
NC_040259.1	42969999	0	A
NC_040259.1	43025745	G	A
NC_040259.1	43082280	G	A
NC_040259.1	43139543	C	T
NC_040259.1	43195768	T	C
NC_040259.1	43246207	A	G
NC_040259.1	43302732	T	C
NC_040259.1	43355930	T	C
NC_040259.1	43400699	G	T
NC_040259.1	43460692	T	C
NC_040259.1	43502448	G	A
NC_040259.1	43558301	A	G
NC_040259.1	43618022	A	G
NC_040259.1	43760325	G	C
NC_040259.1	43817377	G	A
NC_040259.1	43872309	T	C
NC_040259.1	43927384	G	A
NC_040259.1	43984616	A	C
NC_040259.1	44041155	A	G
NC_040259.1	44091808	C	T
NC_040259.1	44142856	G	A
NC_040259.1	44198063	G	A
NC_040259.1	44262116	G	A
NC_040259.1	44323403	G	A
NC_040259.1	44356428	T	C
NC_040259.1	44422744	C	A
NC_040259.1	44501987	T	C
NC_040259.1	44556359	T	C
NC_040259.1	44610677	C	T

NC_040259.1	44673260	A	T
NC_040259.1	44736440	T	A
NC_040259.1	44739483	T	C
NC_040259.1	44801060	T	C
NC_040259.1	44856594	A	G
NC_040259.1	44914014	C	T
NC_040259.1	44961061	T	G
NC_040259.1	45029703	G	A
NC_040259.1	45087357	A	C
NC_040259.1	45140944	A	G
NC_040259.1	45143452	G	A
NC_040259.1	45143839	G	A
NC_040259.1	45203105	A	G
NC_040259.1	45255890	A	G
NC_040259.1	45309120	C	T
NC_040259.1	45360948	T	C
NC_040259.1	45415901	G	A
NC_040259.1	45452299	G	A
NC_040259.1	45524834	G	A
NC_040259.1	45583395	A	C
NC_040259.1	45662806	C	G
NC_040259.1	45708018	A	G
NC_040259.1	45772539	A	G
NC_040259.1	45840929	G	A
NC_040259.1	45885586	T	C
NC_040259.1	45921196	C	T
NC_040259.1	45982057	C	T
NC_040259.1	46072775	T	C
NC_040259.1	46115946	G	C
NC_040259.1	46166072	C	T
NC_040259.1	46231279	A	G
NC_040259.1	46273556	A	G
NC_040259.1	46445477	T	C
NC_040259.1	46490783	C	T
NC_040259.1	46550312	C	T
NC_040259.1	46575354	A	C
NC_040259.1	46633069	T	C
NC_040259.1	46697801	A	T
NC_040259.1	46747615	C	T
NC_040259.1	46789812	A	G
NC_040259.1	46855439	C	T
NC_040259.1	46926409	0	T
NC_040259.1	47424566	T	C
NC_040259.1	47454925	G	A
NC_040259.1	47924549	A	G
NC_040259.1	47991249	T	C
NC_040259.1	48105385	T	G

NC_040259.1	48213435	A	G
NC_040259.1	48268076	C	A
NC_040259.1	48318242	G	A
NC_040259.1	48352807	G	T
NC_040259.1	48423536	G	A
NC_040259.1	48526680	C	A
NC_040259.1	48591074	A	G
NC_040259.1	48712574	C	T
NC_040259.1	48746799	T	C
NC_040259.1	48876062	C	T
NC_040259.1	48940095	C	G
NC_040259.1	48992287	G	C
NC_040259.1	49039010	T	C
NC_040259.1	49094414	G	A
NC_040259.1	49158899	G	A
NC_040259.1	49225938	C	A
NC_040259.1	49259560	G	A
NC_040259.1	49304290	A	G
NC_040259.1	49336733	A	G
NC_040259.1	49379937	A	G
NC_040259.1	49524156	C	T
NC_040259.1	49582715	A	G
NC_040259.1	49628871	A	G
NC_040259.1	49699457	A	C
NC_040259.1	49733431	G	A
NC_040259.1	49792049	A	G
NC_040259.1	49836190	C	T
NC_040259.1	49930904	A	G
NC_040259.1	49961241	C	T
NC_040259.1	50009446	A	G
NC_040259.1	50064986	A	G
NC_040259.1	50104621	T	A
NC_040259.1	50236030	A	G
NC_040259.1	50317144	C	T
NC_040259.1	50366901	A	G
NC_040259.1	50439184	C	T
NC_040259.1	50503750	A	T
NC_040259.1	50576769	A	G
NC_040259.1	50683749	T	C
NC_040259.1	50774620	A	G
NC_040259.1	50832206	T	C
NC_040259.1	50903617	A	G
NC_040259.1	51027334	T	C
NC_040259.1	51081626	T	A
NC_040259.1	51199651	C	T
NC_040259.1	51249848	T	C
NC_040259.1	51651536	C	A

NC_040259.1	51692261	G	A
NC_040259.1	51762202	A	G
NC_040259.1	51818826	C	T
NC_040259.1	51878982	A	G
NC_040259.1	51947366	G	T
NC_040259.1	52005983	A	G
NC_040259.1	52063566	T	G
NC_040259.1	52106931	C	T
NC_040259.1	52156020	A	G
NC_040259.1	52204434	A	T
NC_040259.1	52247038	A	G
NC_040259.1	52305811	G	A
NC_040259.1	52378409	G	A
NC_040259.1	52435079	T	G
NC_040259.1	52484790	A	G
NC_040259.1	52550033	T	C
NC_040259.1	52601987	A	G
NC_040259.1	52638019	T	C
NC_040259.1	52713980	T	C
NC_040259.1	52769427	C	G
NC_040259.1	52825979	A	G
NC_040259.1	52881128	G	C
NC_040259.1	52919824	A	G
NC_040259.1	52987785	A	T
NC_040259.1	53041890	A	C
NC_040259.1	53097446	G	A
NC_040259.1	53145835	C	A
NC_040259.1	53174183	C	T
NC_040259.1	53220102	A	G
NC_040259.1	53260629	G	A
NC_040259.1	53265621	T	C
NC_040259.1	53305410	C	T
NC_040259.1	53354331	C	A
NC_040259.1	53401201	C	G
NC_040259.1	53412970	G	A
NC_040259.1	53476955	C	G
NC_040259.1	53492093	T	C
NC_040259.1	53548534	T	C
NC_040259.1	53615128	G	C
NC_040259.1	53688879	T	C
NC_040259.1	53770807	A	G
NC_040259.1	53834880	A	T
NC_040259.1	53857098	A	G
NC_040259.1	53905744	G	A
NC_040259.1	53933915	A	G
NC_040259.1	54078484	A	C
NC_040259.1	54133552	C	T

NC_040259.1	54201973	T	C
NC_040259.1	54243191	A	G
NC_040259.1	54282656	T	C
NC_040259.1	54364327	C	T
NC_040259.1	54468338	A	G
NC_040259.1	54542911	G	A
NC_040259.1	54593839	G	A
NC_040259.1	54622274	C	T
NC_040259.1	54689765	T	C
NC_040259.1	54696173	T	G
NC_040259.1	54722265	T	C
NC_040259.1	54787269	T	C
NC_040259.1	54834713	A	G
NC_040259.1	54880842	T	C
NC_040259.1	54935380	C	G
NC_040259.1	54987307	A	C
NC_040259.1	55049892	T	C
NC_040259.1	55107490	G	A
NC_040259.1	55176345	C	T
NC_040259.1	55222967	G	A
NC_040259.1	55275989	T	A
NC_040259.1	55331157	G	A
NC_040259.1	55514047	T	G
NC_040259.1	55548630	A	T
NC_040259.1	55593987	G	A
NC_040259.1	55644146	G	A
NC_040259.1	55711392	A	G
NC_040259.1	55760330	T	C
NC_040259.1	55804414	A	G
NC_040259.1	55938981	T	C
NC_040259.1	56133174	T	C
NC_040259.1	56205984	G	A
NC_040259.1	56248417	C	T
NC_040259.1	56555496	T	C
NC_040259.1	56653667	A	G
NC_040259.1	56739982	C	T
NC_040259.1	56787592	G	T
NC_040259.1	56860242	G	A
NC_040259.1	56920671	A	T
NC_040259.1	56975074	A	G
NC_040259.1	57050654	T	C
NC_040259.1	57081744	T	C
NC_040259.1	57231030	A	G
NC_040259.1	57298309	C	G
NC_040259.1	57367503	G	A
NC_040259.1	57412673	G	A
NC_040259.1	57458192	T	C

NC_040259.1	57505996	G	C
NC_040259.1	57565984	T	C
NC_040259.1	57621515	A	G
NC_040259.1	57667595	T	C
NC_040259.1	57714739	C	T
NC_040259.1	57728586	T	C
NC_040259.1	57782361	A	G
NC_040259.1	57846338	A	G
NC_040259.1	57899619	C	A
NC_040259.1	57955188	G	A
NC_040259.1	57980314	A	G
NC_040259.1	58032013	A	G
NC_040259.1	58095592	C	A
NC_040259.1	58150880	T	C
NC_040259.1	58185747	T	C
NC_040259.1	58231867	C	T
NC_040259.1	58321572	G	A
NC_040259.1	58360414	G	A
NC_040259.1	58393923	T	C
NC_040259.1	58479682	G	T
NC_040259.1	58491782	A	G
NC_040259.1	58528184	C	T
NC_040259.1	58590526	T	C
NC_040259.1	58645656	G	T
NC_040259.1	58722671	G	A
NC_040259.1	58788538	C	T
NC_040259.1	58857621	A	G
NC_040259.1	58924210	T	G
NC_040259.1	58989770	G	C
NC_040259.1	59003340	G	A
NC_040259.1	59046167	T	A
NC_040259.1	59102437	T	C
NC_040259.1	59153723	T	C
NC_040259.1	59166041	C	A
NC_040259.1	59168687	G	A
NC_040259.1	59187239	C	T
NC_040259.1	59188132	T	C
NC_040259.1	59227869	T	C
NC_040259.1	59264665	C	T
NC_040259.1	59321541	C	T
NC_040259.1	59371074	C	T
NC_040259.1	59429449	A	G
NC_040259.1	59486362	T	A
NC_040259.1	59542496	T	C
NC_040259.1	59594703	T	A
NC_040259.1	59652545	T	C
NC_040259.1	59704088	C	T

NC_040259.1	59761834	A	G
NC_040259.1	59813741	C	T
NC_040259.1	59868842	T	C
NC_040259.1	59923137	G	C
NC_040259.1	59980015	T	C
NC_040259.1	60037331	A	G
NC_040259.1	60097030	T	C
NC_040259.1	60148760	T	C
NC_040259.1	60185824	G	A
NC_040259.1	60361383	T	C
NC_040259.1	60405970	A	T
NC_040259.1	60462877	C	T
NC_040259.1	60515948	C	A
NC_040259.1	60574885	A	G
NC_040259.1	60631445	A	C
NC_040259.1	60688954	C	T
NC_040259.1	60746400	C	A
NC_040259.1	60787193	G	A
NC_040259.1	60834964	G	A
NC_040259.1	60836649	A	G
NC_040259.1	60872587	A	G
NC_040259.1	60901565	C	T
NC_040259.1	60902828	A	G
NC_040259.1	60961209	T	C
NC_040259.1	61022209	A	G
NC_040259.1	61022898	C	T
NC_040259.1	61078746	C	T
NC_040259.1	61132389	A	G
NC_040259.1	61188524	G	A
NC_040259.1	61241067	C	T
NC_040259.1	61295960	A	C
NC_040259.1	61351473	A	G
NC_040259.1	61405580	G	A
NC_040259.1	61467276	C	T
NC_040259.1	61508416	T	C
NC_040259.1	61575564	A	G
NC_040259.1	61576732	T	C
NC_040259.1	61627804	T	C
NC_040259.1	61682648	C	T
NC_040259.1	61737535	C	T
NC_040259.1	61779365	A	T
NC_040259.1	61836228	T	G
NC_040259.1	61837000	T	G
NC_040259.1	61837955	A	C
NC_040259.1	61884454	C	T
NC_040259.1	61941775	G	A
NC_040259.1	61998750	C	A

NC_040259.1	62048209	G	T
NC_040259.1	62098062	G	A
NC_040259.1	62158105	C	A
NC_040259.1	62204632	A	G
NC_040259.1	62248937	T	C
NC_040259.1	62255330	T	C
NC_040259.1	62256697	A	G
NC_040259.1	62259336	A	G
NC_040259.1	62260488	A	G
NC_040259.1	62316490	A	C
NC_040259.1	62371861	A	G
NC_040259.1	62427078	T	C
NC_040259.1	62472372	T	G
NC_040259.1	62538329	A	T
NC_040259.1	62580646	G	T
NC_040259.1	62615023	A	G
NC_040259.1	62615206	C	T
NC_040259.1	62616432	T	C
NC_040259.1	62663458	G	A
NC_040259.1	62727352	A	G
NC_040259.1	62985945	T	C
NC_040259.1	63014938	C	T
NC_040259.1	63072249	T	C
NC_040259.1	63129230	A	G
NC_040259.1	63179846	C	T
NC_040259.1	63225951	G	A
NC_040259.1	63275757	G	A
NC_040259.1	63311017	A	G
NC_040259.1	63315862	G	C
NC_040259.1	63377941	A	G
NC_040259.1	63378185	A	G
NC_040259.1	63432255	T	C
NC_040259.1	63486640	T	C
NC_040259.1	63543385	G	T
NC_040259.1	63599168	G	A
NC_040259.1	63658004	T	C
NC_040259.1	63705661	A	T
NC_040259.1	63763949	G	A
NC_040259.1	63819580	A	G
NC_040259.1	63871626	C	T
NC_040259.1	63923186	T	C
NC_040259.1	63989099	T	C
NC_040259.1	64077479	G	A
NC_040259.1	64100063	G	A
NC_040259.1	64108334	G	A
NC_040259.1	64140446	T	C
NC_040259.1	64203496	T	C

NC_040259.1	64222119	A	G
NC_040259.1	64222410	A	G
NC_040259.1	64235852	G	A
NC_040259.1	64246480	G	T
NC_040259.1	64295711	A	G
NC_040259.1	64348328	T	C
NC_040259.1	64403868	G	A
NC_040259.1	64459335	G	A
NC_040259.1	64514617	T	C
NC_040259.1	64567198	C	T
NC_040259.1	64620843	G	C
NC_040259.1	64676198	T	C
NC_040259.1	64732279	G	T
NC_040259.1	64782402	G	A
NC_040259.1	64837981	C	T
NC_040259.1	64896526	T	A
NC_040259.1	64948513	G	T
NC_040259.1	65004016	A	G
NC_040259.1	65048046	C	T
NC_040259.1	65049814	T	C
NC_040259.1	65104153	C	T
NC_040259.1	65157854	C	T
NC_040259.1	65204033	G	A
NC_040259.1	65261286	G	A
NC_040259.1	65317070	C	T
NC_040259.1	65371272	A	G
NC_040259.1	65414120	A	G
NC_040259.1	65442351	A	G
NC_040259.1	65452276	C	T
NC_040259.1	65453109	A	G
NC_040259.1	65454315	T	C
NC_040259.1	65492381	A	G
NC_040259.1	65493116	A	G
NC_040259.1	65548141	A	G
NC_040259.1	65605447	A	G
NC_040259.1	65661242	T	C
NC_040259.1	65715548	T	C
NC_040259.1	65764287	A	G
NC_040259.1	65820466	A	G
NC_040259.1	65876744	G	A
NC_040259.1	65932849	T	C
NC_040259.1	65989139	C	T
NC_040259.1	66039255	A	T
NC_040259.1	66095312	G	A
NC_040259.1	66150527	T	A
NC_040259.1	66206649	C	T
NC_040259.1	66274384	A	C

NC_040259.1	66274535	C	T
NC_040259.1	66274686	G	C
NC_040259.1	66299509	A	G
NC_040259.1	66487741	T	C
NC_040259.1	66540995	A	G
NC_040259.1	66595375	C	G
NC_040259.1	66649035	C	T
NC_040259.1	66676457	T	G
NC_040259.1	66708094	G	T
NC_040259.1	66708983	C	T
NC_040259.1	66709138	T	C
NC_040259.1	66765258	A	T
NC_040259.1	66821715	C	G
NC_040259.1	66873568	C	A
NC_040259.1	66932157	C	G
NC_040259.1	66987932	C	T
NC_040259.1	67044537	A	G
NC_040259.1	67094784	T	A
NC_040259.1	67150233	G	A
NC_040259.1	67205548	G	A
NC_040259.1	67261765	C	T
NC_040259.1	67298925	A	G
NC_040259.1	67358329	A	C
NC_040259.1	67415124	C	T
NC_040259.1	67471388	G	A
NC_040259.1	67527730	C	T
NC_040259.1	67582556	C	T
NC_040259.1	67626262	G	C
NC_040259.1	67667693	C	G
NC_040259.1	67669024	T	C
NC_040259.1	67705179	T	C
NC_040259.1	67720536	C	A
NC_040259.1	67728881	A	G
NC_040259.1	67729094	A	G
NC_040259.1	67729711	G	A
NC_040259.1	67730065	C	T
NC_040259.1	67783665	C	T
NC_040259.1	67836715	G	A
NC_040259.1	67867357	G	A
NC_040259.1	67868735	T	C
NC_040259.1	67923770	C	T
NC_040259.1	67980463	C	T
NC_040259.1	68016566	T	C
NC_040259.1	68072807	G	A
NC_040259.1	68129012	C	T
NC_040259.1	68183310	A	G
NC_040259.1	68237864	G	T

NC_040259.1	68278428	A	T
NC_040259.1	68330286	C	T
NC_040259.1	68363496	C	G
NC_040259.1	68363836	T	G
NC_040259.1	68364388	A	C
NC_040259.1	68364567	G	T
NC_040259.1	68406490	A	G
NC_040259.1	68449843	C	G
NC_040259.1	68501497	G	A
NC_040259.1	68541233	G	A
NC_040259.1	68557240	C	A
NC_040259.1	68557435	A	C
NC_040259.1	68610952	A	G
NC_040259.1	68663730	T	A
NC_040259.1	68720241	A	G
NC_040259.1	68777683	A	G
NC_040259.1	68833374	A	G
NC_040259.1	68890356	G	A
NC_040259.1	68947151	A	G
NC_040259.1	69004557	G	A
NC_040259.1	69057941	T	C
NC_040259.1	69112211	G	A
NC_040259.1	69167969	C	T
NC_040259.1	69209760	T	C
NC_040259.1	69214231	T	C
NC_040259.1	69217348	C	A
NC_040259.1	69218858	G	C
NC_040259.1	69249398	G	T
NC_040259.1	69372723	G	A
NC_040259.1	69413188	T	C
NC_040259.1	69446213	C	T
NC_040259.1	69446481	G	A
NC_040259.1	69459824	C	G
NC_040259.1	69510873	G	T
NC_040259.1	69560406	C	T
NC_040259.1	69596819	C	T
NC_040259.1	69633843	T	C
NC_040259.1	69661682	T	C
NC_040259.1	69674315	G	C
NC_040259.1	69674604	G	A
NC_040259.1	69680647	G	A
NC_040259.1	69684198	T	C
NC_040259.1	69741018	C	T
NC_040259.1	69741392	C	A
NC_040259.1	69751578	A	G
NC_040259.1	69751900	T	C
NC_040259.1	69752439	T	C

NC_040259.1	69759388	T	C
NC_040259.1	69760070	C	G
NC_040259.1	69760624	C	T
NC_040259.1	69760822	T	C
NC_040259.1	69820816	C	T
NC_040259.1	69875459	T	C
NC_040259.1	69931966	C	T
NC_040259.1	69985668	T	C
NC_040259.1	70020491	A	G
NC_040259.1	70072470	T	C
NC_040259.1	70103052	A	G
NC_040259.1	70124686	T	G
NC_040259.1	70150794	T	C
NC_040259.1	70176051	A	C
NC_040259.1	70226864	C	T
NC_040259.1	70279488	G	A
NC_040259.1	70336992	A	T
NC_040259.1	70385763	C	T
NC_040259.1	70414398	G	C
NC_040259.1	70414681	G	A
NC_040259.1	70414890	A	G
NC_040259.1	70450994	T	C
NC_040259.1	70452058	A	G
NC_040259.1	70452586	A	G
NC_040259.1	70503520	A	T
NC_040259.1	70550761	A	G
NC_040259.1	70579424	A	G
NC_040259.1	70579986	G	T
NC_040259.1	70636320	T	C
NC_040259.1	70694555	A	G
NC_040259.1	70750193	A	G
NC_040259.1	70805873	G	A
NC_040259.1	70862107	G	A
NC_040259.1	70917408	A	T
NC_040259.1	70972586	T	C
NC_040259.1	71026507	C	A
NC_040259.1	71079876	C	T
NC_040259.1	71131039	T	C
NC_040259.1	71185129	C	A
NC_040259.1	71232409	T	C
NC_040259.1	71287994	T	C
NC_040259.1	71338183	C	T
NC_040259.1	71385720	G	A
NC_040259.1	71429136	T	C
NC_040259.1	71481269	A	G
NC_040259.1	71534297	A	C
NC_040259.1	71586386	T	G

NC_040259.1	71638534	G	A
NC_040259.1	71695786	T	C
NC_040259.1	71750029	T	A
NC_040259.1	71804446	A	C
NC_040259.1	71865169	G	C
NC_040259.1	71897711	T	C
NC_040259.1	71957177	T	C
NC_040259.1	72018073	T	A
NC_040259.1	72080486	A	C
NC_040259.1	72137100	G	A
NC_040259.1	72193497	C	T
NC_040259.1	72237901	C	T
NC_040259.1	72291074	G	C
NC_040259.1	72338226	C	G
NC_040259.1	72381152	C	T
NC_040259.1	72636218	A	G
NC_040259.1	72681946	G	A
NC_040259.1	72726032	A	G
NC_040259.1	72781248	C	T
NC_040259.1	72835788	A	C
NC_040259.1	72891957	C	T
NC_040259.1	72941829	G	A
NC_040259.1	72989374	T	C
NC_040259.1	73036715	C	A
NC_040259.1	73092507	G	T
NC_040259.1	73147908	T	A
NC_040259.1	73207808	G	A
NC_040259.1	73266343	T	G
NC_040259.1	73321880	G	A
NC_040259.1	73374773	C	A
NC_040259.1	73424772	T	C
NC_040259.1	73481265	G	C
NC_040259.1	73539163	C	A
NC_040259.1	73592983	T	C
NC_040259.1	73644287	T	C
NC_040259.1	73698377	C	T
NC_040259.1	73750323	G	A
NC_040259.1	73806981	G	A
NC_040259.1	73858190	T	C
NC_040259.1	73859441	C	T
NC_040259.1	73866662	C	T
NC_040259.1	73866979	G	T
NC_040259.1	73882822	T	C
NC_040259.1	73884648	G	A
NC_040259.1	73887798	C	T
NC_040259.1	73943710	A	T
NC_040259.1	74000316	T	C

NC_040259.1	74056915	A	T
NC_040259.1	74111457	G	A
NC_040259.1	74167494	C	T
NC_040259.1	74219468	C	G
NC_040259.1	74274780	T	G
NC_040259.1	74333422	C	G
NC_040259.1	74386638	G	A
NC_040259.1	74440344	C	T
NC_040259.1	74496955	T	C
NC_040259.1	74538098	G	A
NC_040259.1	74589547	A	G
NC_040259.1	74602494	G	A
NC_040259.1	74604237	T	C
NC_040259.1	74604393	G	C
NC_040259.1	74659884	G	A
NC_040259.1	74715818	A	C
NC_040259.1	74770796	T	A
NC_040259.1	74805094	T	C
NC_040259.1	74857553	G	A
NC_040259.1	74897057	G	C
NC_040259.1	74942552	A	G
NC_040259.1	74943741	T	C
NC_040259.1	74945090	A	G
NC_040259.1	74946924	C	G
NC_040259.1	74957255	A	G
NC_040259.1	75012791	G	A
NC_040259.1	75014944	T	A
NC_040259.1	75016420	T	C
NC_040259.1	75069756	A	G
NC_040259.1	75126678	A	G
NC_040259.1	75178904	T	C
NC_040259.1	75232600	A	C
NC_040259.1	75276531	C	T
NC_040259.1	75308014	T	C
NC_040259.1	75365881	G	T
NC_040259.1	75424266	A	G
NC_040259.1	75478172	T	C
NC_040259.1	75515176	A	G
NC_040259.1	75551958	A	G
NC_040259.1	75607180	T	C
NC_040259.1	75662746	T	C
NC_040259.1	75715462	G	A
NC_040259.1	75754433	G	A
NC_040259.1	75790257	T	C
NC_040259.1	75825755	A	G
NC_040259.1	75861905	T	G
NC_040259.1	75910832	G	C

NC_040259.1	75962055	A	G
NC_040259.1	76071202	G	A
NC_040259.1	76124405	C	G
NC_040259.1	76174097	T	C
NC_040259.1	76226744	C	T
NC_040259.1	76282863	T	C
NC_040259.1	76343042	T	G
NC_040259.1	76400011	G	A
NC_040259.1	76573723	C	G
NC_040259.1	76654247	A	G
NC_040259.1	76702502	G	T
NC_040259.1	76764808	T	C
NC_040259.1	76813668	C	T
NC_040259.1	76866609	G	A
NC_040259.1	76925473	G	A
NC_040259.1	76973452	C	G
NC_040259.1	76974200	C	T
NC_040259.1	77048730	A	G
NC_040259.1	77098179	T	C
NC_040259.1	77105374	A	G
NC_040259.1	77109919	G	A
NC_040259.1	77115047	C	T
NC_040259.1	77130877	C	T
NC_040259.1	77181965	C	T
NC_040259.1	77227257	C	T
NC_040259.1	77275185	G	A
NC_040259.1	77333072	G	C
NC_040259.1	77386402	A	G
NC_040259.1	77442239	C	T
NC_040259.1	77504504	T	C
NC_040259.1	77553497	C	T
NC_040259.1	77582409	C	A
NC_040259.1	77582562	A	G
NC_040259.1	77614441	G	C
NC_040259.1	77649874	G	A
NC_040259.1	77709918	A	T
NC_040259.1	77765474	C	T
NC_040259.1	77818540	T	G
NC_040259.1	77876324	C	A
NC_040259.1	77926896	T	C
NC_040259.1	77977291	A	G
NC_040259.1	78038287	C	T
NC_040259.1	78090977	T	C
NC_040259.1	78146885	A	G
NC_040259.1	78202313	G	A
NC_040259.1	78253105	T	A
NC_040259.1	78303147	T	A

NC_040259.1	78358393	A	G
NC_040259.1	78401487	T	C
NC_040259.1	78424153	A	G
NC_040259.1	78475541	C	T
NC_040259.1	78509389	T	C
NC_040259.1	78566659	T	C
NC_040259.1	78566998	A	G
NC_040259.1	78569494	T	G
NC_040259.1	78569718	A	T
NC_040259.1	78570094	G	C
NC_040259.1	78571193	A	G
NC_040259.1	78628471	T	C
NC_040259.1	78690479	A	C
NC_040259.1	78691295	C	T
NC_040259.1	78744377	C	T
NC_040259.1	78794991	A	C
NC_040259.1	78850528	C	T
NC_040259.1	78906805	A	G
NC_040259.1	78957216	T	A
NC_040259.1	79000445	G	A
NC_040259.1	79047034	A	G
NC_040259.1	79100668	C	T
NC_040259.1	79148510	A	C
NC_040259.1	79194940	A	T
NC_040259.1	79250507	C	A
NC_040259.1	79306243	T	G
NC_040259.1	79358017	C	T
NC_040259.1	79416287	T	C
NC_040259.1	79475435	C	T
NC_040259.1	79527436	G	A
NC_040259.1	79576395	T	C
NC_040259.1	79619830	T	C
NC_040259.1	79620058	G	A
NC_040259.1	79650965	T	G
NC_040259.1	79651863	T	C
NC_040259.1	79707932	A	T
NC_040259.1	79759477	C	T
NC_040259.1	79814969	C	A
NC_040259.1	79852818	G	A
NC_040259.1	79908322	T	C
NC_040259.1	79964403	T	A
NC_040259.1	80020084	G	A
NC_040259.1	80076822	C	T
NC_040259.1	80133433	A	G
NC_040259.1	80186208	G	A
NC_040259.1	80244012	G	A
NC_040259.1	80299784	A	G

NC_040259.1	80355445	T	C
NC_040259.1	80417555	C	A
NC_040259.1	80478508	C	T
NC_040259.1	80480037	G	C
NC_040259.1	80529193	T	C
NC_040259.1	80530351	A	G
NC_040259.1	80530504	T	A
NC_040259.1	80531338	C	T
NC_040259.1	80531925	T	C
NC_040259.1	80586161	A	G
NC_040259.1	80603087	G	A
NC_040259.1	80608740	T	C
NC_040259.1	80609170	T	A
NC_040259.1	80620522	A	C
NC_040259.1	80633009	A	G
NC_040259.1	80651582	C	A
NC_040259.1	80651745	A	G
NC_040259.1	80651959	G	A
NC_040259.1	80652601	T	C
NC_040259.1	80653721	T	A
NC_040259.1	80654322	G	A
NC_040259.1	80659484	G	A
NC_040259.1	80704655	G	C
NC_040259.1	80730799	A	G
NC_040259.1	80747175	C	T
NC_040259.1	80751662	C	T
NC_040259.1	80769335	T	C
NC_040259.1	81430508	T	C
NC_040259.1	81474724	A	G
NC_040259.1	81520835	C	T
NC_040259.1	81560043	C	A
NC_040259.1	81622621	G	A
NC_040259.1	81668585	A	G
NC_040259.1	81713127	A	G
NC_040259.1	81764557	C	G
NC_040259.1	81816245	A	G
NC_040259.1	81871751	A	C
NC_040259.1	81927719	C	G
NC_040259.1	81983476	C	G
NC_040259.1	82052715	T	C
NC_040259.1	82092837	T	C
NC_040259.1	82093358	G	A
NC_040259.1	82094606	G	A
NC_040259.1	82094780	A	C
NC_040259.1	82094972	T	C
NC_040259.1	82149237	T	C
NC_040259.1	82185869	C	A

NC_040259.1	82234574	T		C
NC_040259.1	82290650	G		T
NC_040259.1	82345912	T		A
NC_040259.1	82399202	C		G
NC_040259.1	82451766	C		A
NC_040259.1	82499114	G		T
NC_040259.1	82528184	C		G
NC_040259.1	82533823	T		C
NC_040259.1	82534928	A		G
NC_040259.1	82535770	A		G
NC_040259.1	82536022	T		C
NC_040259.1	82544597	G		A
NC_040259.1	82545825	T		C
NC_040259.1	82546194	T		C
NC_040259.1	82546359	A		G
NC_040259.1	82580562	G		A
NC_040259.1	82581444	T		A
NC_040259.1	82581704	T		C
NC_040259.1	82615183	A		C
NC_040259.1	82615636	T		C
NC_040259.1	82671803	G		A
NC_040259.1	82725278	T		C
NC_040259.1	82727995	A		G
NC_040259.1	82738570	G		C
NC_040259.1	82798694	A		G
NC_040259.1	82849029		0	C
NC_040259.1	82898702	T		C
NC_040259.1	82925501	T		C
NC_040259.1	82928143	G		C
NC_040259.1	82983481	G		T
NC_040259.1	83038637	C		T
NC_040259.1	83095629	G		C
NC_040259.1	83146810	T		C
NC_040259.1	83210667	G		C
NC_040259.1	83210904	T		C
NC_040259.1	83211160	T		C
NC_040259.1	83211379	T		C
NC_040259.1	83212945	T		C
NC_040259.1	83213466	A		G
NC_040259.1	83214856	C		T
NC_040259.1	83249690	T		C
NC_040259.1	83253145	G		A
NC_040259.1	83260970	A		G
NC_040259.1	83271404	C		T
NC_040259.1	83290207	A		G
NC_040259.1	83302146	T		C
NC_040259.1	83350656	A		G

NC_040259.1	83372463	A	G
NC_040259.1	83405999	A	G
NC_040259.1	83443727	A	G
NC_040259.1	83444042	C	T
NC_040259.1	83464568	A	T
NC_040259.1	83478593	A	G
NC_040259.1	83482797	T	G
NC_040259.1	83491176	G	A
NC_040259.1	83545163	G	A
NC_040259.1	83564597	T	C
NC_040259.1	83571995	T	C
NC_040259.1	83628823	T	G
NC_040259.1	83684551	A	G
NC_040259.1	83732772	A	G
NC_040259.1	83802492	A	G
NC_040259.1	83844859	C	T
NC_040259.1	83910267	G	A
NC_040259.1	84113911	C	G
NC_040259.1	84161481	T	A
NC_040259.1	84216067	A	C
NC_040259.1	84261263	T	C
NC_040259.1	84315082	A	G
NC_040259.1	84419642	G	T
NC_040259.1	84486417	T	C
NC_040259.1	84657429	C	T
NC_040259.1	84745649	A	G
NC_040259.1	84851178	A	T
NC_040259.1	84900616	A	C
NC_040259.1	84954201	A	T
NC_040259.1	85012516	A	G
NC_040259.1	85045298	A	G
NC_040259.1	85097977	T	C
NC_040259.1	85122729	G	A
NC_040259.1	85172730	T	C
NC_040259.1	85217605	A	G
NC_040259.1	85272376	T	G
NC_040259.1	85309103	A	G
NC_040259.1	85364775	G	A
NC_040259.1	85417108	C	A
NC_040259.1	85460065	A	C
NC_040259.1	85521168	A	C
NC_040259.1	85581026	C	G
NC_040259.1	85635790	A	G
NC_040259.1	85688522	A	G
NC_040259.1	85746276	A	C
NC_040259.1	85809151	T	C
NC_040259.1	85864085	A	G

NC_040259.1	85918621	G	A
NC_040259.1	85965319	G	A
NC_040259.1	85965667	T	G
NC_040259.1	86029657	A	G
NC_040259.1	86087142	T	C
NC_040259.1	86143180	C	T
NC_040259.1	86184920	G	C
NC_040259.1	86218768	C	T
NC_040259.1	86273418	A	G
NC_040259.1	86333735	T	C
NC_040259.1	86334054	C	T
NC_040259.1	86334216	A	G
NC_040259.1	86350277	A	G
NC_040259.1	86351864	T	A
NC_040259.1	86352221	A	G
NC_040259.1	86381117	T	C
NC_040259.1	86686107	T	C
NC_040259.1	86724313	C	T
NC_040259.1	86765105	T	C
NC_040259.1	86783643	A	C
NC_040259.1	86827353	C	T
NC_040259.1	86882508	G	A
NC_040259.1	86935283	T	C
NC_040259.1	86990521	T	G
NC_040259.1	87040988	G	A
NC_040259.1	87095412	T	C
NC_040259.1	87161919	C	T
NC_040259.1	87217326	T	A
NC_040259.1	87271214	G	T
NC_040259.1	87327247	A	G
NC_040259.1	87383128	T	A
NC_040259.1	87429977	T	C
NC_040259.1	87484837	T	C
NC_040259.1	87544848	T	C
NC_040259.1	87597298	G	A
NC_040259.1	87654977	G	A
NC_040259.1	87716333	A	C
NC_040259.1	87769818	T	A
NC_040259.1	87826026	C	T
NC_040259.1	87881102	G	T
NC_040259.1	87938561	G	A
NC_040259.1	87994003	T	C
NC_040259.1	88052847	G	T
NC_040259.1	88107505	C	A
NC_040259.1	88162182	T	C
NC_040259.1	88217511	A	G
NC_040259.1	88269960	T	C

NC_040259.1	88326858	T	C
NC_040259.1	88382887	T	G
NC_040259.1	88437578	C	T
NC_040259.1	88489993	G	A
NC_040259.1	88545969	C	T
NC_040259.1	88552387	T	C
NC_040259.1	88563917	G	C
NC_040259.1	88564382	G	C
NC_040259.1	88564613	G	A
NC_040259.1	88566688	T	C
NC_040259.1	88569547	T	C
NC_040259.1	88571299	A	G
NC_040259.1	88571808	A	C
NC_040259.1	88572219	G	T
NC_040259.1	88572677	T	C
NC_040259.1	88629962	C	T
NC_040259.1	88689686	C	T
NC_040259.1	88745542	T	A
NC_040259.1	88794676	A	G
NC_040259.1	88843745	T	C
NC_040259.1	88897589	G	A
NC_040259.1	88962532	T	C
NC_040259.1	89024916	C	T
NC_040259.1	89082614	A	G
NC_040259.1	89134191	A	G
NC_040259.1	89185922	T	C
NC_040259.1	89224056	G	A
NC_040259.1	89279366	A	G
NC_040259.1	89329197	G	A
NC_040259.1	89363507	A	G
NC_040259.1	89366499	A	G
NC_040259.1	89373809	T	C
NC_040259.1	89383930	C	T
NC_040259.1	89386336	A	G
NC_040259.1	89437595	C	G
NC_040259.1	89493828	A	C
NC_040259.1	89536885	T	C
NC_040259.1	89592749	T	C
NC_040259.1	89648769	C	T
NC_040259.1	89674934	T	C
NC_040259.1	89675625	G	T
NC_040259.1	89675816	T	C
NC_040259.1	89676143	T	C
NC_040259.1	89679601	G	T
NC_040259.1	89679805	A	G
NC_040259.1	89680138	A	G
NC_040259.1	89736558	C	T

NC_040259.1	89764610	T	C
NC_040259.1	89767510	G	A
NC_040259.1	89768148	A	G
NC_040259.1	89769679	A	G
NC_040259.1	89771074	A	G
NC_040259.1	89796283	A	G
NC_040259.1	89797385	T	C
NC_040259.1	89835379	A	G
NC_040259.1	89875158	T	C
NC_040259.1	89922698	C	A
NC_040259.1	89977809	A	T
NC_040259.1	90046141	T	C
NC_040259.1	90055399	C	A
NC_040259.1	90055705	C	T
NC_040259.1	90082207	C	G
NC_040259.1	90082428	A	G
NC_040259.1	90085254	A	G
NC_040259.1	90145349	G	A
NC_040259.1	90199056	T	C
NC_040259.1	90246559	T	C
NC_040259.1	90287797	T	C
NC_040259.1	90310730	C	T
NC_040259.1	90352030	T	G
NC_040259.1	90410375	T	C
NC_040259.1	90458485	G	C
NC_040259.1	90505989	G	A
NC_040259.1	90534169	C	A
NC_040259.1	90590119	G	C
NC_040259.1	90643297	T	C
NC_040259.1	90699447	A	G
NC_040259.1	90757703	C	A
NC_040259.1	90813926	A	G
NC_040259.1	90814157	A	G
NC_040259.1	90850914	T	C
NC_040259.1	90907909	T	C
NC_040259.1	90950637	A	G
NC_040259.1	90978666	T	C
NC_040259.1	90980245	T	C
NC_040259.1	90993538	A	G
NC_040259.1	90994684	G	A
NC_040259.1	90995027	T	C
NC_040259.1	91017486	G	A
NC_040259.1	91047871	C	G
NC_040259.1	91096178	G	T
NC_040259.1	91127529	A	G
NC_040259.1	91180898	T	C
NC_040259.1	91239749	G	A

NC_040259.1	91282304	T		A
NC_040259.1	91329369	T		C
NC_040259.1	91342585	A		G
NC_040259.1	91398065	A		G
NC_040259.1	91450221	A		G
NC_040259.1	91501894	C		T
NC_040259.1	91509155	T		C
NC_040259.1	91567152	T		C
NC_040259.1	91613241	T		C
NC_040259.1	91650295	A		G
NC_040259.1	91704006	T		C
NC_040259.1	91760207	G		A
NC_040259.1	91807789	T		C
NC_040259.1	91865255	C		T
NC_040259.1	91914994	A		G
NC_040259.1	91968754	G		T
NC_040259.1	92024738	A		G
NC_040259.1	92083141	A		G
NC_040259.1	92140036	C		T
NC_040259.1	92161986	G		C
NC_040259.1	92218736	A		G
NC_040259.1	92270136	T		C
NC_040259.1	92323939	G		T
NC_040259.1	92383902	A		G
NC_040259.1	92433132	A		G
NC_040259.1	92500977	T		C
NC_040259.1	92557464	G		C
NC_040259.1	92609434	T		C
NC_040259.1	92661865	C		T
NC_040259.1	92696335	T		C
NC_040259.1	92735350	C		A
NC_040259.1	92791982	T		G
NC_040259.1	92847571	G		A
NC_040259.1	92904975	A		C
NC_040259.1	93018001	T		C
NC_040259.1	93075350	T		A
NC_040259.1	93127130	A		G
NC_040259.1	93172545	G		A
NC_040259.1	93209009	A		G
NC_040259.1	93263967	C		T
NC_040259.1	93319542	G		A
NC_040259.1	93375034	C		A
NC_040259.1	93427517	G		T
NC_040259.1	93480585	C		T
NC_040259.1	93536711		0	A
NC_040259.1	93593462	A		T
NC_040259.1	93646026	T		C

NC_040259.1	93683339	G	T
NC_040259.1	93685412	G	C
NC_040259.1	93685772	G	A
NC_040259.1	93688148	T	A
NC_040259.1	93691692	A	G
NC_040259.1	93693190	G	C
NC_040259.1	93693694	A	G
NC_040259.1	93746730	C	T
NC_040259.1	93797546	C	T
NC_040259.1	93851797	C	G
NC_040259.1	93903598	C	T
NC_040259.1	93957493	T	C
NC_040259.1	94016625	A	C
NC_040259.1	94076306	C	A
NC_040259.1	94128473	A	C
NC_040259.1	94186068	C	G
NC_040259.1	94226321	C	G
NC_040259.1	94272404	C	T
NC_040259.1	94309300	G	A
NC_040259.1	94373417	C	G
NC_040259.1	94430589	G	T
NC_040259.1	94483422	A	G
NC_040259.1	94534531	T	C
NC_040259.1	94587406	C	T
NC_040259.1	94636638	A	G
NC_040259.1	94680314	G	A
NC_040259.1	94724099	C	T
NC_040259.1	94782273	G	A
NC_040259.1	94849723	A	C
NC_040259.1	94909898	C	G
NC_040259.1	94977226	C	T
NC_040259.1	95031688	T	A
NC_040259.1	95086176	T	G
NC_040259.1	95133965	A	G
NC_040259.1	95158697	T	C
NC_040259.1	95163179	T	C
NC_040259.1	95190664	A	G
NC_040259.1	95278439	T	C
NC_040259.1	95307481	A	G
NC_040259.1	95363743	A	C
NC_040259.1	95419738	T	C
NC_040259.1	95462016	A	G
NC_040259.1	95518139	A	G
NC_040259.1	95572268	G	A
NC_040259.1	95624757	C	A
NC_040259.1	95678626	G	T
NC_040259.1	95728878	T	C

NC_040259.1	95784799	T	C
NC_040259.1	95850730	A	G
NC_040259.1	95886263	G	C
NC_040259.1	95936864	A	G
NC_040259.1	95989670	T	C
NC_040259.1	96046818	C	G
NC_040259.1	96104425	T	C
NC_040259.1	96106222	G	C
NC_040259.1	96158721	T	C
NC_040259.1	96213349	A	G
NC_040259.1	96271426	G	A
NC_040259.1	96324370	T	C
NC_040259.1	96383097	A	G
NC_040259.1	96446857	G	A
NC_040259.1	96483387	C	G
NC_040259.1	96508323	C	T
NC_040259.1	96539356	A	T
NC_040259.1	96593888	A	G
NC_040259.1	96619363	A	C
NC_040259.1	96621269	A	G
NC_040259.1	96672141	T	C
NC_040259.1	96707220	T	C
NC_040259.1	96751994	G	A
NC_040259.1	96804376	A	G
NC_040259.1	96857753	G	A
NC_040259.1	96901443	G	T
NC_040259.1	96998727	T	G
NC_040259.1	97026141	A	G
NC_040259.1	97027479	A	G
NC_040259.1	97039081	T	C
NC_040259.1	97066217	T	G
NC_040259.1	97066962	T	G
NC_040259.1	97067645	T	C
NC_040259.1	97122765	A	G
NC_040259.1	97134358	T	C
NC_040259.1	97191598	C	T
NC_040259.1	97225608	T	G
NC_040259.1	97281121	T	C
NC_040259.1	97337054	A	T
NC_040259.1	97387323	A	G
NC_040259.1	97436172	C	G
NC_040259.1	97475572	A	C
NC_040259.1	97476058	G	C
NC_040259.1	97528046	T	G
NC_040259.1	97576532	A	G
NC_040259.1	97626757	A	G
NC_040259.1	97682333	G	A

NC_040259.1	97736579	G	A
NC_040259.1	97788198	C	T
NC_040259.1	97820739	G	A
NC_040259.1	97822015	T	C
NC_040259.1	97826830	G	A
NC_040259.1	97828606	A	G
NC_040259.1	97829385	G	A
NC_040259.1	97844230	C	G
NC_040259.1	97898142	T	G
NC_040259.1	97964901	A	G
NC_040259.1	98014803	A	C
NC_040259.1	98014960	A	G
NC_040259.1	98067087	G	A
NC_040259.1	98117259	G	A
NC_040259.1	98161530	T	G
NC_040259.1	98162563	0	T
NC_040259.1	98217015	A	G
NC_040259.1	98235448	T	C
NC_040259.1	98289596	A	G
NC_040259.1	98345858	G	A
NC_040259.1	98399540	T	C
NC_040259.1	98455923	T	A
NC_040259.1	98511615	C	T
NC_040259.1	98564595	A	G
NC_040259.1	98603304	T	C
NC_040259.1	98603799	A	G
NC_040259.1	98639185	A	C
NC_040259.1	98643192	C	T
NC_040259.1	98649850	A	G
NC_040259.1	98650030	C	G
NC_040259.1	98650790	C	T
NC_040259.1	98683855	T	C
NC_040259.1	98717547	A	G
NC_040259.1	98731141	T	A
NC_040259.1	98738873	G	A
NC_040259.1	98747922	C	T
NC_040259.1	98748092	T	C
NC_040259.1	98748455	G	A
NC_040259.1	98751873	G	C
NC_040259.1	98752511	C	A
NC_040259.1	98753251	C	T
NC_040260.1	59030	A	G
NC_040260.1	122725	G	C
NC_040260.1	160412	C	T
NC_040260.1	209137	T	C
NC_040260.1	263076	A	C
NC_040260.1	279005	T	A

NC_040260.1	284573	A	G
NC_040260.1	314910	T	C
NC_040260.1	315269	G	A
NC_040260.1	389236	A	G
NC_040260.1	432916	A	C
NC_040260.1	433304	0	G
NC_040260.1	439741	C	T
NC_040260.1	487301	C	G
NC_040260.1	542643	C	T
NC_040260.1	595526	A	G
NC_040260.1	654830	T	C
NC_040260.1	709455	T	C
NC_040260.1	759277	G	A
NC_040260.1	817865	A	G
NC_040260.1	881397	C	T
NC_040260.1	923296	C	T
NC_040260.1	962752	T	C
NC_040260.1	1018632	A	G
NC_040260.1	1076641	T	C
NC_040260.1	1135043	A	G
NC_040260.1	1193639	G	A
NC_040260.1	1236687	G	A
NC_040260.1	1281737	A	G
NC_040260.1	1357502	A	T
NC_040260.1	1465150	G	T
NC_040260.1	1514390	G	A
NC_040260.1	1593300	A	G
NC_040260.1	1660430	C	T
NC_040260.1	1718775	C	T
NC_040260.1	1792050	C	T
NC_040260.1	1847337	A	G
NC_040260.1	2015081	C	G
NC_040260.1	2167740	T	C
NC_040260.1	2244244	T	C
NC_040260.1	2308301	C	T
NC_040260.1	2402528	A	C
NC_040260.1	2492288	T	C
NC_040260.1	2540787	G	C
NC_040260.1	2595479	A	T
NC_040260.1	2658221	C	G
NC_040260.1	2717119	C	G
NC_040260.1	2758988	A	C
NC_040260.1	2803335	G	A
NC_040260.1	2865641	G	A
NC_040260.1	2973471	C	A
NC_040260.1	3068108	T	C
NC_040260.1	3133208	A	G

NC_040260.1	3223186	G	A
NC_040260.1	3256448	T	C
NC_040260.1	3311149	A	G
NC_040260.1	3375481	T	C
NC_040260.1	3424319	G	A
NC_040260.1	3482780	G	A
NC_040260.1	3517373	T	C
NC_040260.1	3572756	G	A
NC_040260.1	3634812	C	T
NC_040260.1	3698135	C	T
NC_040260.1	3716717	C	G
NC_040260.1	3752638	C	T
NC_040260.1	3803040	G	T
NC_040260.1	3866440	C	T
NC_040260.1	3925148	T	C
NC_040260.1	3993334	A	G
NC_040260.1	4176652	C	T
NC_040260.1	4412080	C	T
NC_040260.1	4484527	G	A
NC_040260.1	4728706	G	A
NC_040260.1	4784575	A	C
NC_040260.1	4818976	G	A
NC_040260.1	4863784	A	G
NC_040260.1	4931752	C	G
NC_040260.1	4966005	G	A
NC_040260.1	5014017	T	C
NC_040260.1	5065110	C	T
NC_040260.1	5148601	T	C
NC_040260.1	5205570	C	T
NC_040260.1	5276325	C	G
NC_040260.1	5342143	A	G
NC_040260.1	5443767	T	C
NC_040260.1	5512146	T	C
NC_040260.1	5559199	T	G
NC_040260.1	5637016	T	C
NC_040260.1	5687001	T	C
NC_040260.1	5736836	C	A
NC_040260.1	5767012	C	A
NC_040260.1	5827941	A	G
NC_040260.1	5896756	G	C
NC_040260.1	5914334	A	G
NC_040260.1	5963580	G	C
NC_040260.1	6065880	C	T
NC_040260.1	6089686	T	C
NC_040260.1	6573689	C	T
NC_040260.1	6610798	A	G
NC_040260.1	6736753	T	C

NC_040260.1	6838415	C	T
NC_040260.1	6902418	G	A
NC_040260.1	7070808	T	C
NC_040260.1	7147760	A	G
NC_040260.1	7233238	A	G
NC_040260.1	7300975	A	T
NC_040260.1	7396394	A	G
NC_040260.1	7422753	T	C
NC_040260.1	7522810	T	A
NC_040260.1	7631635	C	T
NC_040260.1	7662981	T	C
NC_040260.1	7791977	G	A
NC_040260.1	7871793	A	G
NC_040260.1	7934001	A	G
NC_040260.1	7971914	A	G
NC_040260.1	8105485	G	T
NC_040260.1	8263383	A	G
NC_040260.1	8314918	A	T
NC_040260.1	8384450	T	G
NC_040260.1	8521367	C	T
NC_040260.1	8595145	A	G
NC_040260.1	8755495	G	C
NC_040260.1	8803237	T	C
NC_040260.1	8876233	A	G
NC_040260.1	8952137	G	T
NC_040260.1	9007794	T	C
NC_040260.1	9031031	C	T
NC_040260.1	9084259	A	C
NC_040260.1	9140852	A	G
NC_040260.1	9186836	T	G
NC_040260.1	9254500	C	A
NC_040260.1	9332889	T	A
NC_040260.1	9442284	T	C
NC_040260.1	9523237	A	G
NC_040260.1	9676895	C	G
NC_040260.1	9752985	T	C
NC_040260.1	9916335	G	C
NC_040260.1	9965413	A	G
NC_040260.1	10262588	C	T
NC_040260.1	10280466	T	A
NC_040260.1	10373107	C	A
NC_040260.1	10432115	A	G
NC_040260.1	10457060	C	T
NC_040260.1	10546854	T	G
NC_040260.1	10634194	C	G
NC_040260.1	10694911	T	C
NC_040260.1	10750421	C	T

NC_040260.1	10822320	A	G
NC_040260.1	10914375	G	A
NC_040260.1	10977275	A	G
NC_040260.1	11037685	G	T
NC_040260.1	11091784	A	G
NC_040260.1	11138551	G	C
NC_040260.1	11176130	A	G
NC_040260.1	11353651	A	G
NC_040260.1	11689670	G	A
NC_040260.1	11876250	C	T
NC_040260.1	11941786	T	C
NC_040260.1	11983295	A	G
NC_040260.1	12016080	T	G
NC_040260.1	12155178	G	A
NC_040260.1	12210026	T	C
NC_040260.1	12263821	T	C
NC_040260.1	12312390	G	T
NC_040260.1	12361500	C	T
NC_040260.1	12416852	T	G
NC_040260.1	12473706	C	T
NC_040260.1	12526967	C	T
NC_040260.1	12673155	T	G
NC_040260.1	12703915	C	T
NC_040260.1	12773097	T	C
NC_040260.1	12886403	C	T
NC_040260.1	12987377	G	A
NC_040260.1	13038930	G	A
NC_040260.1	13128906	G	A
NC_040260.1	13151043	G	A
NC_040260.1	13278997	A	T
NC_040260.1	13355820	A	G
NC_040260.1	13404516	A	G
NC_040260.1	13454509	C	G
NC_040260.1	13513934	G	T
NC_040260.1	13537775	A	G
NC_040260.1	13868522	G	A
NC_040260.1	13909372	T	C
NC_040260.1	13963650	T	C
NC_040260.1	14017516	T	C
NC_040260.1	14025308	T	C
NC_040260.1	14025935	G	T
NC_040260.1	14051935	C	T
NC_040260.1	14052984	T	C
NC_040260.1	14113495	C	T
NC_040260.1	14179092	A	G
NC_040260.1	14238177	C	T
NC_040260.1	14454997	C	A

NC_040260.1	14473398	C	T
NC_040260.1	14474056	T	G
NC_040260.1	14534775	C	G
NC_040260.1	14554786	A	G
NC_040260.1	14558459	A	G
NC_040260.1	14565443	T	C
NC_040260.1	14599395	T	C
NC_040260.1	14614559	T	C
NC_040260.1	14629510	T	C
NC_040260.1	14630144	T	G
NC_040260.1	14634825	G	C
NC_040260.1	14638510	A	C
NC_040260.1	14639154	C	A
NC_040260.1	14639645	A	G
NC_040260.1	14642299	A	G
NC_040260.1	14644961	T	C
NC_040260.1	14650514	A	G
NC_040260.1	14651217	G	A
NC_040260.1	14657552	C	T
NC_040260.1	14658467	T	C
NC_040260.1	14659002	A	G
NC_040260.1	14660751	A	G
NC_040260.1	14662549	T	G
NC_040260.1	14663948	C	G
NC_040260.1	14681097	T	C
NC_040260.1	14706887	A	G
NC_040260.1	14715761	A	G
NC_040260.1	14718641	T	C
NC_040260.1	14756502	T	C
NC_040260.1	14758826	A	G
NC_040260.1	14762391	T	C
NC_040260.1	14771773	A	G
NC_040260.1	14773308	T	G
NC_040260.1	14776508	G	A
NC_040260.1	14785486	A	G
NC_040260.1	14786343	A	G
NC_040260.1	14789707	T	C
NC_040260.1	14805909	A	G
NC_040260.1	14821582	T	C
NC_040260.1	14852936	A	G
NC_040260.1	14855463	G	C
NC_040260.1	14875107	T	C
NC_040260.1	14879386	T	C
NC_040260.1	14920114	T	C
NC_040260.1	14921551	A	G
NC_040260.1	14935252	T	C
NC_040260.1	14935851	G	C

NC_040260.1	14940185	A	G
NC_040260.1	14946829	T	C
NC_040260.1	14957875	A	G
NC_040260.1	14959401	T	C
NC_040260.1	14962286	A	G
NC_040260.1	14964605	G	C
NC_040260.1	14972387	A	G
NC_040260.1	14980189	T	C
NC_040260.1	14982823	T	C
NC_040260.1	14985774	C	T
NC_040260.1	15038534	T	C
NC_040260.1	15046337	C	G
NC_040260.1	15085239	T	C
NC_040260.1	15088964	T	C
NC_040260.1	15090275	T	G
NC_040260.1	15091117	A	G
NC_040260.1	15096430	A	G
NC_040260.1	15141984	T	C
NC_040260.1	15145171	A	G
NC_040260.1	15212531	T	G
NC_040260.1	15215811	A	G
NC_040260.1	15231472	T	C
NC_040260.1	15241403	G	A
NC_040260.1	15249693	A	G
NC_040260.1	15252307	T	C
NC_040260.1	15262214	A	G
NC_040260.1	15283230	T	C
NC_040260.1	15285148	T	C
NC_040260.1	15307093	A	C
NC_040260.1	15307641	A	G
NC_040260.1	15359838	T	C
NC_040260.1	15362127	C	G
NC_040260.1	15364310	T	C
NC_040260.1	15371825	C	T
NC_040260.1	15383823	T	C
NC_040260.1	15384481	T	C
NC_040260.1	15393261	G	A
NC_040260.1	15407720	T	C
NC_040260.1	15409864	T	C
NC_040260.1	15458133	C	T
NC_040260.1	15512785	T	C
NC_040260.1	15639058	C	T
NC_040260.1	15644925	T	C
NC_040260.1	15689624	A	C
NC_040260.1	15733524	C	T
NC_040260.1	15770678	T	C
NC_040260.1	15771970	C	T

NC_040260.1	15828337	C	T
NC_040260.1	15893324	G	A
NC_040260.1	15904108	T	G
NC_040260.1	15911521	A	G
NC_040260.1	15923655	A	G
NC_040260.1	15983570	A	G
NC_040260.1	16028306	A	G
NC_040260.1	16099947	A	G
NC_040260.1	16155713	A	G
NC_040260.1	16209492	T	C
NC_040260.1	16264843	T	C
NC_040260.1	16336915	A	G
NC_040260.1	16396710	C	T
NC_040260.1	16450100	T	C
NC_040260.1	16500909	T	C
NC_040260.1	16560446	G	A
NC_040260.1	16617572	G	A
NC_040260.1	16673755	C	A
NC_040260.1	16724103	C	T
NC_040260.1	16759528	A	G
NC_040260.1	16769959	T	C
NC_040260.1	16806272	C	T
NC_040260.1	16866629	T	C
NC_040260.1	16903528	T	C
NC_040260.1	16938936	C	T
NC_040260.1	16984116	A	G
NC_040260.1	17025459	T	C
NC_040260.1	17078460	G	A
NC_040260.1	17096371	T	G
NC_040260.1	17116511	T	G
NC_040260.1	17140340	C	A
NC_040260.1	17200988	A	G
NC_040260.1	17261567	C	G
NC_040260.1	17280069	A	G
NC_040260.1	17284300	C	T
NC_040260.1	17339092	G	A
NC_040260.1	17385269	T	G
NC_040260.1	17390673	T	C
NC_040260.1	17393780	G	A
NC_040260.1	17394912	G	A
NC_040260.1	17465762	A	C
NC_040260.1	17519954	T	G
NC_040260.1	17570328	C	T
NC_040260.1	17619019	T	C
NC_040260.1	17678168	T	C
NC_040260.1	17746782	G	A
NC_040260.1	17815077	A	G

NC_040260.1	17872830	A	C
NC_040260.1	17919176	T	C
NC_040260.1	17960447	T	C
NC_040260.1	18013796	C	G
NC_040260.1	18073735	C	G
NC_040260.1	18123950	G	A
NC_040260.1	18175341	A	G
NC_040260.1	18254613	A	G
NC_040260.1	18311584	T	G
NC_040260.1	18340855	T	C
NC_040260.1	18401131	T	C
NC_040260.1	18432454	G	T
NC_040260.1	18499620	G	C
NC_040260.1	18552821	G	A
NC_040260.1	18621115	A	G
NC_040260.1	18672711	A	G
NC_040260.1	18738729	C	T
NC_040260.1	18791899	A	C
NC_040260.1	18860764	T	C
NC_040260.1	18911603	A	G
NC_040260.1	19077846	A	G
NC_040260.1	19130970	G	A
NC_040260.1	19155001	T	C
NC_040260.1	19196488	T	A
NC_040260.1	19322453	C	T
NC_040260.1	19391889	G	A
NC_040260.1	19442803	C	T
NC_040260.1	19497510	G	A
NC_040260.1	19559435	G	A
NC_040260.1	19602810	T	A
NC_040260.1	19672753	T	C
NC_040260.1	19717091	A	G
NC_040260.1	19753991	A	G
NC_040260.1	19822156	A	T
NC_040260.1	19869329	A	T
NC_040260.1	19927129	C	T
NC_040260.1	19983059	G	C
NC_040260.1	20040299	C	T
NC_040260.1	20093132	C	T
NC_040260.1	20146082	C	T
NC_040260.1	20201466	A	G
NC_040260.1	20252651	C	T
NC_040260.1	20304304	A	C
NC_040260.1	20361166	C	T
NC_040260.1	20413029	G	T
NC_040260.1	20480303	T	C
NC_040260.1	20535887	C	A

NC_040260.1	20578820	A	G
NC_040260.1	20639321	T	G
NC_040260.1	20687714	C	A
NC_040260.1	20727822	T	C
NC_040260.1	20785749	T	G
NC_040260.1	20840784	C	T
NC_040260.1	20897662	G	A
NC_040260.1	20975762	T	G
NC_040260.1	21036172	G	A
NC_040260.1	21093316	A	G
NC_040260.1	21155805	A	G
NC_040260.1	21211368	G	A
NC_040260.1	21292855	T	C
NC_040260.1	21376178	A	G
NC_040260.1	21431917	C	T
NC_040260.1	21491060	A	G
NC_040260.1	21547420	T	C
NC_040260.1	21604419	G	A
NC_040260.1	21630326	T	C
NC_040260.1	21805596	G	T
NC_040260.1	21869681	T	C
NC_040260.1	21924789	C	T
NC_040260.1	21983226	C	T
NC_040260.1	22078018	T	A
NC_040260.1	22132198	A	G
NC_040260.1	22211825	C	T
NC_040260.1	22265457	G	A
NC_040260.1	22322939	A	G
NC_040260.1	22377015	T	C
NC_040260.1	22452070	A	G
NC_040260.1	22505878	T	C
NC_040260.1	22559604	C	T
NC_040260.1	22604064	A	G
NC_040260.1	22642740	A	G
NC_040260.1	22690418	A	G
NC_040260.1	22744536	C	A
NC_040260.1	22794376	A	C
NC_040260.1	22817635	T	C
NC_040260.1	22842783	G	C
NC_040260.1	22867781	T	C
NC_040260.1	22877068	T	C
NC_040260.1	22906704	G	A
NC_040260.1	22967916	C	A
NC_040260.1	23025053	G	A
NC_040260.1	23049527	T	C
NC_040260.1	23050453	A	G
NC_040260.1	23110140	G	A

NC_040260.1	23187170	G	C
NC_040260.1	23253985	A	G
NC_040260.1	23310687	A	G
NC_040260.1	23311154	T	C
NC_040260.1	23336247	G	A
NC_040260.1	23388602	A	G
NC_040260.1	23450463	G	C
NC_040260.1	23479556	C	G
NC_040260.1	23549446	A	C
NC_040260.1	23609770	A	C
NC_040260.1	23670565	T	C
NC_040260.1	23729398	G	C
NC_040260.1	23788042	A	G
NC_040260.1	23844165	T	C
NC_040260.1	23849135	T	C
NC_040260.1	23908254	C	T
NC_040260.1	23944189	T	C
NC_040260.1	23979852	C	T
NC_040260.1	24048683	C	A
NC_040260.1	24124267	G	A
NC_040260.1	24171879	A	G
NC_040260.1	24230823	A	G
NC_040260.1	24291477	C	T
NC_040260.1	24371508	T	C
NC_040260.1	24426372	T	G
NC_040260.1	24477161	A	G
NC_040260.1	24533686	A	G
NC_040260.1	24568258	T	G
NC_040260.1	24634879	G	A
NC_040260.1	24682220	T	C
NC_040260.1	24732870	A	G
NC_040260.1	24801952	A	G
NC_040260.1	24854075	G	A
NC_040260.1	24909354	A	C
NC_040260.1	24951411	G	A
NC_040260.1	25006746	A	T
NC_040260.1	25068124	A	C
NC_040260.1	25126790	A	G
NC_040260.1	25185915	G	A
NC_040260.1	25240852	C	T
NC_040260.1	25299078	G	A
NC_040260.1	25351272	A	T
NC_040260.1	25414158	A	G
NC_040260.1	25425949	A	C
NC_040260.1	25485721	A	G
NC_040260.1	25543719	G	A
NC_040260.1	25599948	A	G

NC_040260.1	25657911	C	T
NC_040260.1	25719719	C	A
NC_040260.1	25780575	T	G
NC_040260.1	25844612	A	G
NC_040260.1	25898281	A	C
NC_040260.1	25955663	C	T
NC_040260.1	26024366	A	G
NC_040260.1	26080545	G	T
NC_040260.1	26135702	A	G
NC_040260.1	26201278	T	C
NC_040260.1	26257153	A	G
NC_040260.1	26320189	G	T
NC_040260.1	26365060	C	T
NC_040260.1	26419948	T	C
NC_040260.1	26473910	G	C
NC_040260.1	26539227	C	T
NC_040260.1	26593936	G	A
NC_040260.1	26651006	A	T
NC_040260.1	26702828	C	T
NC_040260.1	26775260	A	G
NC_040260.1	26878121	C	T
NC_040260.1	26933521	G	A
NC_040260.1	26988224	C	T
NC_040260.1	27045252	C	G
NC_040260.1	27103549	T	G
NC_040260.1	27154118	A	G
NC_040260.1	27216945	A	G
NC_040260.1	27277641	G	T
NC_040260.1	27332624	T	C
NC_040260.1	27385327	A	G
NC_040260.1	27441634	C	T
NC_040260.1	27506518	A	G
NC_040260.1	27559879	T	G
NC_040260.1	27613064	C	T
NC_040260.1	27673239	G	A
NC_040260.1	27727254	A	T
NC_040260.1	27780680	T	C
NC_040260.1	27837755	A	G
NC_040260.1	27894694	G	A
NC_040260.1	27948043	A	G
NC_040260.1	28011980	C	A
NC_040260.1	28061829	C	T
NC_040260.1	28122579	A	G
NC_040260.1	28176759	G	A
NC_040260.1	28232249	A	G
NC_040260.1	28285266	G	A
NC_040260.1	28328524	G	A

NC_040260.1	28404511	G	A
NC_040260.1	28517581	G	A
NC_040260.1	28581700	A	G
NC_040260.1	28692449	T	C
NC_040260.1	28734134	A	G
NC_040260.1	28791325	C	A
NC_040260.1	28815602	C	G
NC_040260.1	28867656	C	T
NC_040260.1	28940234	T	C
NC_040260.1	29004978	G	C
NC_040260.1	29060655	A	G
NC_040260.1	29084750	G	A
NC_040260.1	29536481	G	A
NC_040260.1	29594323	G	C
NC_040260.1	29616491	T	C
NC_040260.1	29674358	T	G
NC_040260.1	29726904	T	C
NC_040260.1	29800185	T	C
NC_040260.1	29853636	A	G
NC_040260.1	29910437	A	G
NC_040260.1	29967131	G	A
NC_040260.1	30023211	G	A
NC_040260.1	30101851	C	G
NC_040260.1	30103187	T	C
NC_040260.1	30106612	T	C
NC_040260.1	30109393	C	T
NC_040260.1	30172141	A	G
NC_040260.1	30222299	T	C
NC_040260.1	30273517	C	T
NC_040260.1	30332617	G	T
NC_040260.1	30393163	T	C
NC_040260.1	30457098	T	C
NC_040260.1	30478785	A	G
NC_040260.1	30498038	C	T
NC_040260.1	30554137	G	A
NC_040260.1	30610537	G	T
NC_040260.1	30665860	C	T
NC_040260.1	30719464	T	C
NC_040260.1	30778581	C	T
NC_040260.1	30847428	G	A
NC_040260.1	30878142	A	G
NC_040260.1	30920163	A	G
NC_040260.1	30937633	C	T
NC_040260.1	30938143	C	A
NC_040260.1	30938651	T	C
NC_040260.1	30942338	C	T
NC_040260.1	30942887	C	A

NC_040260.1	31003006	G	C
NC_040260.1	31003542	T	C
NC_040260.1	31049581	A	T
NC_040260.1	31115386	G	A
NC_040260.1	31166852	A	T
NC_040260.1	31190491	C	A
NC_040260.1	31250717	C	A
NC_040260.1	31302318	T	C
NC_040260.1	31362665	A	T
NC_040260.1	31400692	C	T
NC_040260.1	31444306	T	C
NC_040260.1	31473551	A	G
NC_040260.1	31519856	A	G
NC_040260.1	31547063	T	C
NC_040260.1	31600962	A	G
NC_040260.1	31646950	T	C
NC_040260.1	31680374	T	C
NC_040260.1	31682906	T	C
NC_040260.1	31733674	C	T
NC_040260.1	31756218	C	T
NC_040260.1	31794172	C	T
NC_040260.1	31840283	T	C
NC_040260.1	31873889	T	G
NC_040260.1	31874449	T	C
NC_040260.1	31878642	A	C
NC_040260.1	31879966	T	C
NC_040260.1	31901193	T	C
NC_040260.1	31903470	A	G
NC_040260.1	31974553	G	A
NC_040260.1	32006002	T	C
NC_040260.1	32060091	A	G
NC_040260.1	32097509	A	G
NC_040260.1	32115688	A	G
NC_040260.1	32175016	A	G
NC_040260.1	32234377	T	C
NC_040260.1	32275999	T	A
NC_040260.1	32328296	C	T
NC_040260.1	32386758	G	C
NC_040260.1	32443284	T	C
NC_040260.1	32504276	C	T
NC_040260.1	32562061	T	C
NC_040260.1	32619932	T	C
NC_040260.1	32676712	G	A
NC_040260.1	32726895	T	C
NC_040260.1	32791036	C	T
NC_040260.1	32859372	T	A
NC_040260.1	32919849	G	A

NC_040260.1	32971955	G	T
NC_040260.1	33027843	C	T
NC_040260.1	33082498	C	T
NC_040260.1	33147097	A	G
NC_040260.1	33235316	A	G
NC_040260.1	33292163	G	A
NC_040260.1	33349006	G	A
NC_040260.1	33390646	T	C
NC_040260.1	33390828	T	C
NC_040260.1	33431122	A	C
NC_040260.1	33431728	C	T
NC_040260.1	33477707	A	G
NC_040260.1	33532774	A	T
NC_040260.1	33587840	T	C
NC_040260.1	33643257	A	G
NC_040260.1	33701056	T	C
NC_040260.1	33754962	A	C
NC_040260.1	33809885	A	G
NC_040260.1	33865897	T	C
NC_040260.1	33914169	C	A
NC_040260.1	33972092	A	G
NC_040260.1	34035626	G	A
NC_040260.1	34097803	A	G
NC_040260.1	34127152	G	A
NC_040260.1	34215915	C	G
NC_040260.1	34305598	C	T
NC_040260.1	34350438	A	G
NC_040260.1	34399899	T	C
NC_040260.1	34440995	C	G
NC_040260.1	34441319	A	G
NC_040260.1	34441620	T	C
NC_040260.1	34441797	G	C
NC_040260.1	34441986	C	T
NC_040260.1	34458336	T	C
NC_040260.1	34461819	A	T
NC_040260.1	34462027	C	A
NC_040260.1	34522169	G	A
NC_040260.1	34563486	T	A
NC_040260.1	34608862	C	T
NC_040260.1	34667228	C	T
NC_040260.1	34721978	G	A
NC_040260.1	34742782	A	G
NC_040260.1	34742953	T	C
NC_040260.1	34752506	A	G
NC_040260.1	34757783	T	C
NC_040260.1	34759176	T	C
NC_040260.1	34778672	A	G

NC_040260.1	34787331	G	A
NC_040260.1	34796550	C	A
NC_040260.1	34814891	T	A
NC_040260.1	34817926	G	A
NC_040260.1	34837134	G	A
NC_040260.1	34859629	G	A
NC_040260.1	34867796	T	C
NC_040260.1	34873596	T	C
NC_040260.1	34874742	G	T
NC_040260.1	34878540	C	T
NC_040260.1	34929651	C	A
NC_040260.1	35046276	A	G
NC_040260.1	35102664	G	A
NC_040260.1	35158377	A	C
NC_040260.1	35218684	A	G
NC_040260.1	35275254	A	G
NC_040260.1	35337433	A	G
NC_040260.1	35337586	A	G
NC_040260.1	35380772	G	A
NC_040260.1	35438095	T	C
NC_040260.1	35498656	A	G
NC_040260.1	35664284	A	G
NC_040260.1	35716462	G	A
NC_040260.1	35764626	C	G
NC_040260.1	35821448	G	T
NC_040260.1	35872924	G	A
NC_040260.1	35932005	G	A
NC_040260.1	35987655	T	C
NC_040260.1	36052984	A	G
NC_040260.1	36120781	G	T
NC_040260.1	36214789	T	C
NC_040260.1	36267132	T	C
NC_040260.1	36323877	T	C
NC_040260.1	36380066	C	T
NC_040260.1	36629497	T	C
NC_040260.1	36687239	T	G
NC_040260.1	36748234	T	C
NC_040260.1	36766641	G	A
NC_040260.1	36785605	G	A
NC_040260.1	36841507	T	G
NC_040260.1	36899256	C	T
NC_040260.1	36922530	T	A
NC_040260.1	36984855	G	C
NC_040260.1	37017447	C	T
NC_040260.1	37076735	C	A
NC_040260.1	37132411	T	C
NC_040260.1	37185083	T	A

NC_040260.1	37235222	T	C
NC_040260.1	37240816	G	A
NC_040260.1	37242607	T	C
NC_040260.1	37266956	C	A
NC_040260.1	37325959	C	G
NC_040260.1	37402371	T	C
NC_040260.1	37459249	C	G
NC_040260.1	37511624	A	G
NC_040260.1	37567964	T	C
NC_040260.1	37593101	G	A
NC_040260.1	37612413	C	G
NC_040260.1	37661916	C	A
NC_040260.1	37716840	G	C
NC_040260.1	37755625	G	A
NC_040260.1	37810611	C	T
NC_040260.1	37863992	A	G
NC_040260.1	37915786	G	A
NC_040260.1	37958304	G	A
NC_040260.1	38026495	T	C
NC_040260.1	38061576	G	A
NC_040260.1	38067794	G	A
NC_040260.1	38069075	G	C
NC_040260.1	38069388	A	G
NC_040260.1	38070544	G	A
NC_040260.1	38110182	T	C
NC_040260.1	38151936	A	G
NC_040260.1	38218624	A	T
NC_040260.1	38271473	A	G
NC_040260.1	38328318	C	T
NC_040260.1	38396901	T	G
NC_040260.1	38461420	C	T
NC_040260.1	38504558	C	T
NC_040260.1	38560807	G	T
NC_040260.1	38622004	A	G
NC_040260.1	38682215	T	C
NC_040260.1	38700695	T	C
NC_040260.1	38776741	T	C
NC_040260.1	38799896	T	C
NC_040260.1	38800155	C	T
NC_040260.1	38818927	G	T
NC_040260.1	38876479	G	C
NC_040260.1	38913027	T	C
NC_040260.1	38913237	A	G
NC_040260.1	38941099	A	G
NC_040260.1	38982634	C	T
NC_040260.1	39035075	T	C
NC_040260.1	39065317	G	A

NC_040260.1	39067772	C	T
NC_040260.1	39069892	T	C
NC_040260.1	39074754	T	C
NC_040260.1	39075012	G	C
NC_040260.1	39121165	G	A
NC_040260.1	39168770	C	T
NC_040260.1	39228294	G	A
NC_040260.1	39228591	T	C
NC_040260.1	39233923	G	C
NC_040260.1	39289548	C	A
NC_040260.1	39345650	A	C
NC_040260.1	39397229	T	C
NC_040260.1	39453836	C	T
NC_040260.1	39521379	G	A
NC_040260.1	39578401	A	C
NC_040260.1	39614157	T	A
NC_040260.1	39615915	A	C
NC_040260.1	39617230	C	A
NC_040260.1	39617657	C	T
NC_040260.1	39624227	C	T
NC_040260.1	39633258	T	G
NC_040260.1	39633507	C	G
NC_040260.1	39694081	G	A
NC_040260.1	39752184	A	G
NC_040260.1	39808267	C	T
NC_040260.1	39865891	G	A
NC_040260.1	39923429	A	C
NC_040260.1	39975495	A	G
NC_040260.1	40037006	C	T
NC_040260.1	40103449	T	C
NC_040260.1	40175719	T	C
NC_040260.1	40177438	T	G
NC_040260.1	40233243	G	A
NC_040260.1	40288928	T	C
NC_040260.1	40336387	A	G
NC_040260.1	40353756	A	G
NC_040260.1	40356937	A	G
NC_040260.1	40397422	A	G
NC_040260.1	40454635	C	A
NC_040260.1	40475232	G	A
NC_040260.1	40494397	A	G
NC_040260.1	40497523	A	G
NC_040260.1	40560193	C	T
NC_040260.1	40612702	C	T
NC_040260.1	40679912	A	G
NC_040260.1	40680201	G	A
NC_040260.1	40688368	A	G

NC_040260.1	40740627	G	C
NC_040260.1	40802674	A	G
NC_040260.1	40863071	G	A
NC_040260.1	40923658	C	T
NC_040260.1	40984362	G	A
NC_040260.1	41040877	A	G
NC_040260.1	41099497	T	A
NC_040260.1	41155773	C	T
NC_040260.1	41213546	G	T
NC_040260.1	41275021	T	G
NC_040260.1	41330478	G	C
NC_040260.1	41382772	G	A
NC_040260.1	41440304	T	A
NC_040260.1	41498888	C	T
NC_040260.1	41556896	A	G
NC_040260.1	41614965	C	G
NC_040260.1	41672634	A	C
NC_040260.1	41716682	A	C
NC_040260.1	41758097	T	A
NC_040260.1	41817118	A	G
NC_040260.1	41874121	A	T
NC_040260.1	41909479	T	G
NC_040260.1	41973118	T	C
NC_040260.1	41973784	A	G
NC_040260.1	42040319	A	G
NC_040260.1	42084204	A	G
NC_040260.1	42084666	A	G
NC_040260.1	42085092	A	G
NC_040260.1	42142624	A	G
NC_040260.1	42167202	G	A
NC_040260.1	42168655	T	C
NC_040260.1	42180789	A	G
NC_040260.1	42186214	C	T
NC_040260.1	42189077	T	C
NC_040260.1	42200946	T	C
NC_040260.1	42205038	T	C
NC_040260.1	42212780	A	G
NC_040260.1	42213538	T	C
NC_040260.1	42270221	G	A
NC_040260.1	42326762	T	C
NC_040260.1	42371121	G	C
NC_040260.1	42728975	A	C
NC_040260.1	42785181	C	T
NC_040260.1	42839999	T	A
NC_040260.1	42913045	G	A
NC_040260.1	42965370	T	C
NC_040260.1	42980112	T	C

NC_040260.1	43030480	C	G
NC_040260.1	43086066	A	G
NC_040260.1	43127706	C	T
NC_040260.1	43130710	C	A
NC_040260.1	43181520	G	T
NC_040260.1	43240104	G	T
NC_040260.1	43287873	T	C
NC_040260.1	43346214	C	A
NC_040260.1	43406768	C	T
NC_040260.1	43595971	G	A
NC_040260.1	43652705	C	A
NC_040260.1	43711876	C	A
NC_040260.1	43863201	T	A
NC_040260.1	43921919	C	T
NC_040260.1	43972095	A	T
NC_040260.1	44028062	T	C
NC_040260.1	44072056	C	T
NC_040260.1	44111496	T	C
NC_040260.1	44163370	G	A
NC_040260.1	44221217	T	C
NC_040260.1	44322906	T	C
NC_040260.1	44368835	T	C
NC_040260.1	44403683	G	T
NC_040260.1	44463157	T	C
NC_040260.1	44467450	G	C
NC_040260.1	44479064	G	A
NC_040260.1	44479673	A	G
NC_040260.1	44480018	T	C
NC_040260.1	44494734	T	C
NC_040260.1	44496350	A	G
NC_040260.1	44497564	A	G
NC_040260.1	44552196	A	G
NC_040260.1	44605140	C	G
NC_040260.1	44662936	C	T
NC_040260.1	44720266	G	A
NC_040260.1	44776695	A	C
NC_040260.1	44842919	T	C
NC_040260.1	44899806	T	C
NC_040260.1	44958330	G	A
NC_040260.1	45016021	C	T
NC_040260.1	45071344	A	G
NC_040260.1	45126239	G	A
NC_040260.1	45179085	T	C
NC_040260.1	45235535	G	A
NC_040260.1	45291499	T	C
NC_040260.1	45349772	T	C
NC_040260.1	45406289	C	T

NC_040260.1	45462229	T	A
NC_040260.1	45523832	C	T
NC_040260.1	45580630	G	C
NC_040260.1	45628347	A	G
NC_040260.1	45628542	C	T
NC_040260.1	45629132	T	C
NC_040260.1	45629689	C	T
NC_040260.1	45631694	C	T
NC_040260.1	45632468	A	T
NC_040260.1	45632919	T	G
NC_040260.1	45686287	A	G
NC_040260.1	45741562	G	A
NC_040260.1	45801489	A	C
NC_040260.1	45882367	A	G
NC_040260.1	45957929	T	C
NC_040260.1	46082427	T	A
NC_040260.1	46137753	T	C
NC_040260.1	46197167	C	T
NC_040260.1	46253376	G	A
NC_040260.1	46310899	T	G
NC_040260.1	46369385	C	T
NC_040260.1	46431925	C	A
NC_040260.1	46447235	C	G
NC_040260.1	46492809	G	A
NC_040260.1	46529007	C	T
NC_040260.1	46585654	C	T
NC_040260.1	46634226	C	G
NC_040260.1	46690972	G	A
NC_040260.1	46742706	G	T
NC_040260.1	46813947	A	G
NC_040260.1	46861157	T	C
NC_040260.1	46879854	T	A
NC_040260.1	46922166	T	A
NC_040260.1	46922842	A	G
NC_040260.1	46924509	C	G
NC_040260.1	46926113	A	C
NC_040260.1	46944663	C	A
NC_040260.1	46946683	G	A
NC_040260.1	46948169	C	T
NC_040260.1	47006122	G	A
NC_040260.1	47063192	T	C
NC_040260.1	47107159	G	A
NC_040260.1	47107889	C	A
NC_040260.1	47166524	T	C
NC_040260.1	47188742	C	T
NC_040260.1	47190136	G	A
NC_040260.1	47232651	T	C

NC_040260.1	47278251	A	T
NC_040260.1	47278995	C	T
NC_040260.1	47280073	C	T
NC_040260.1	47281966	A	G
NC_040260.1	47282133	G	A
NC_040260.1	47282550	A	G
NC_040260.1	47282876	C	T
NC_040260.1	47305518	A	C
NC_040260.1	47306238	G	A
NC_040260.1	47363434	A	T
NC_040260.1	47418238	T	C
NC_040260.1	47430947	A	G
NC_040260.1	47461214	A	G
NC_040260.1	47461402	T	C
NC_040260.1	47479985	G	A
NC_040260.1	47506608	C	A
NC_040260.1	47572341	T	C
NC_040260.1	47573893	G	A
NC_040260.1	47616324	T	C
NC_040260.1	47650935	G	A
NC_040260.1	47691112	G	C
NC_040260.1	47729640	T	A
NC_040260.1	47731464	G	A
NC_040260.1	47732136	T	G
NC_040260.1	47743612	G	A
NC_040260.1	47789459	A	G
NC_040260.1	47825838	T	C
NC_040260.1	47861896	G	C
NC_040260.1	47913071	A	G
NC_040260.1	47940753	C	T
NC_040260.1	47948778	T	C
NC_040260.1	47999503	C	G
NC_040260.1	48055617	C	T
NC_040260.1	48113856	T	C
NC_040260.1	48169958	C	T
NC_040260.1	48227067	T	C
NC_040260.1	48246830	G	T
NC_040260.1	48301407	C	T
NC_040260.1	48359159	T	G
NC_040260.1	48412560	C	T
NC_040260.1	48412716	T	G
NC_040260.1	48473446	A	G
NC_040260.1	48522628	G	A
NC_040260.1	48573433	T	G
NC_040260.1	48585542	C	T
NC_040260.1	48644322	T	C
NC_040260.1	48653832	T	C

NC_040260.1	48703738	A	T
NC_040260.1	48747358	T	C
NC_040260.1	48747881	A	G
NC_040260.1	48748153	C	T
NC_040260.1	48750323	T	G
NC_040260.1	48750876	A	G
NC_040260.1	48751286	G	A
NC_040260.1	48752517	T	A
NC_040260.1	48753032	C	A
NC_040260.1	48753258	G	A
NC_040260.1	48766237	A	C
NC_040260.1	48784449	C	T
NC_040260.1	48788205	G	C
NC_040260.1	48790645	G	T
NC_040260.1	48791215	G	C
NC_040260.1	48791957	C	T
NC_040260.1	48812757	G	A
NC_040260.1	48825751	A	G
NC_040260.1	48826033	C	T
NC_040260.1	48826691	T	C
NC_040260.1	48826874	A	G
NC_040260.1	48884243	T	C
NC_040260.1	48942568	G	A
NC_040260.1	49000365	A	C
NC_040260.1	49062560	G	A
NC_040260.1	49083725	A	C
NC_040260.1	49083890	G	A
NC_040260.1	49089546	G	A
NC_040260.1	49091862	G	A
NC_040260.1	49147863	G	A
NC_040260.1	49203778	G	A
NC_040260.1	49262366	T	C
NC_040260.1	49310903	A	C
NC_040260.1	49346388	A	G
NC_040260.1	49346910	C	T
NC_040260.1	49348864	C	A
NC_040260.1	49350182	G	A
NC_040260.1	49385924	C	A
NC_040260.1	49407171	T	C
NC_040260.1	49410394	G	A
NC_040260.1	49410922	T	C
NC_040260.1	49453460	C	T
NC_040260.1	49512562	T	A
NC_040260.1	49578988	A	G
NC_040260.1	49596172	A	G
NC_040260.1	49603941	A	G
NC_040260.1	49604957	A	G

NC_040260.1	49606090	T	C
NC_040260.1	49607766	G	A
NC_040260.1	49608381	C	T
NC_040260.1	49669104	A	T
NC_040260.1	49709127	A	G
NC_040260.1	49709288	G	C
NC_040260.1	49764712	A	G
NC_040260.1	49832778	T	C
NC_040260.1	49885990	C	T
NC_040260.1	49936778	G	T
NC_040260.1	49992637	C	T
NC_040260.1	50041715	C	T
NC_040260.1	50099226	G	A
NC_040260.1	50154429	A	T
NC_040260.1	50208013	T	C
NC_040260.1	50267210	A	T
NC_040260.1	50324120	C	A
NC_040260.1	50379749	C	T
NC_040260.1	50440532	G	A
NC_040260.1	50479581	C	T
NC_040260.1	50530631	G	T
NC_040260.1	50705675	C	T
NC_040260.1	50731978	G	C
NC_040260.1	50778924	T	G
NC_040260.1	50835925	A	G
NC_040260.1	50893591	A	G
NC_040260.1	50894075	T	G
NC_040260.1	50894420	A	G
NC_040260.1	50896899	T	C
NC_040260.1	50902264	T	C
NC_040260.1	50904141	T	C
NC_040260.1	50904306	T	A
NC_040260.1	50906072	G	A
NC_040260.1	50906671	T	C
NC_040260.1	50907209	T	C
NC_040260.1	50907411	T	C
NC_040260.1	50907927	T	G
NC_040260.1	50908306	T	C
NC_040260.1	50908541	T	A
NC_040260.1	50968482	C	T
NC_040260.1	51019888	G	T
NC_040260.1	51059159	T	C
NC_040260.1	51116220	G	A
NC_040260.1	51163387	C	T
NC_040260.1	51212103	G	A
NC_040260.1	51227363	A	G
NC_040260.1	51228785	G	A

NC_040260.1	51230363	T	G
NC_040260.1	51261896	T	C
NC_040260.1	51262729	T	C
NC_040260.1	51262884	T	C
NC_040260.1	51263652	G	A
NC_040260.1	51264718	A	G
NC_040260.1	51265125	A	G
NC_040260.1	51265370	C	G
NC_040260.1	51271024	T	C
NC_040260.1	51273432	T	C
NC_040260.1	51276208	A	G
NC_040260.1	51313369	C	T
NC_040260.1	51372144	T	C
NC_040260.1	51427906	T	G
NC_040260.1	51488779	A	T
NC_040260.1	51545270	T	C
NC_040260.1	51545450	A	G
NC_040260.1	51545830	C	T
NC_040260.1	51602678	G	A
NC_040260.1	51654512	A	C
NC_040260.1	51704299	G	A
NC_040260.1	51764054	C	T
NC_040260.1	51813483	G	T
NC_040260.1	51869127	G	A
NC_040260.1	51915762	G	A
NC_040260.1	51971804	A	G
NC_040260.1	52025807	G	A
NC_040260.1	52082260	A	G
NC_040260.1	52140947	G	A
NC_040260.1	52203448	T	C
NC_040260.1	52204262	C	T
NC_040260.1	52204525	C	T
NC_040260.1	52204675	A	G
NC_040260.1	52221342	A	G
NC_040260.1	52281487	T	C
NC_040260.1	52341688	G	T
NC_040260.1	52376182	A	G
NC_040260.1	52381419	G	A
NC_040260.1	52436667	A	G
NC_040260.1	52493951	G	C
NC_040260.1	52551357	T	C
NC_040260.1	52607580	G	T
NC_040260.1	52681293	T	C
NC_040260.1	52738819	G	C
NC_040260.1	52795989	G	T
NC_040260.1	52853851	A	T
NC_040260.1	52913188	G	A

NC_040260.1	52968331	A	G
NC_040260.1	53031924	A	C
NC_040260.1	53054431	T	C
NC_040260.1	53079467	T	C
NC_040260.1	53136484	A	C
NC_040260.1	53189512	C	T
NC_040260.1	53243401	A	G
NC_040260.1	53316120	T	A
NC_040260.1	53345157	A	G
NC_040260.1	53403992	G	C
NC_040260.1	53464238	A	G
NC_040260.1	53520409	T	C
NC_040260.1	53580285	C	T
NC_040260.1	53641651	T	C
NC_040260.1	53705318	C	T
NC_040260.1	53768814	A	G
NC_040260.1	53825121	A	G
NC_040260.1	53887404	T	C
NC_040260.1	53944354	C	A
NC_040260.1	53997900	T	A
NC_040260.1	54020193	T	C
NC_040260.1	54042120	C	T
NC_040260.1	54050717	T	C
NC_040260.1	54052308	A	G
NC_040260.1	54078934	T	C
NC_040260.1	54134723	T	G
NC_040260.1	54185812	T	C
NC_040260.1	54245081	T	A
NC_040260.1	54285713	T	C
NC_040260.1	54326385	G	C
NC_040260.1	54327985	T	A
NC_040260.1	54328257	C	A
NC_040260.1	54328579	G	T
NC_040260.1	54389745	C	A
NC_040260.1	54428274	T	C
NC_040260.1	54488233	G	A
NC_040260.1	54532725	A	G
NC_040260.1	54535436	T	C
NC_040260.1	54536970	G	C
NC_040260.1	54537177	A	G
NC_040260.1	54537617	G	A
NC_040260.1	54594894	T	C
NC_040260.1	54635458	C	G
NC_040260.1	54666726	A	G
NC_040260.1	54691459	C	T
NC_040260.1	54826608	T	C
NC_040260.1	54884024	G	A

NC_040260.1	54930138	C	T
NC_040260.1	55012808	C	A
NC_040260.1	55060580	A	C
NC_040260.1	55130150	T	C
NC_040260.1	55190290	C	T
NC_040260.1	55224125	T	G
NC_040260.1	55266482	G	A
NC_040260.1	55267274	A	G
NC_040260.1	55267441	C	T
NC_040260.1	55268665	A	G
NC_040260.1	55311884	C	T
NC_040260.1	55369830	A	G
NC_040260.1	55429606	G	A
NC_040260.1	55479699	T	C
NC_040260.1	55538078	G	A
NC_040260.1	55597194	C	A
NC_040260.1	55653961	A	G
NC_040260.1	55709205	A	G
NC_040260.1	55763663	C	T
NC_040260.1	55779401	T	A
NC_040260.1	55779897	T	C
NC_040260.1	55806648	C	T
NC_040260.1	55864757	C	G
NC_040260.1	55922470	A	G
NC_040260.1	55951871	A	G
NC_040260.1	55971515	A	G
NC_040260.1	56028383	A	T
NC_040260.1	56083971	T	C
NC_040260.1	56139388	C	T
NC_040260.1	56196099	T	C
NC_040260.1	56246353	G	C
NC_040260.1	56449502	A	G
NC_040260.1	56506808	C	T
NC_040260.1	56565393	G	A
NC_040260.1	56585004	T	C
NC_040260.1	56585573	C	G
NC_040260.1	56585918	T	A
NC_040260.1	56645297	G	A
NC_040260.1	56702465	C	G
NC_040260.1	56757749	C	T
NC_040260.1	56817347	G	A
NC_040260.1	56871723	G	A
NC_040260.1	56928811	T	C
NC_040260.1	56979344	T	C
NC_040260.1	57034457	A	G
NC_040260.1	57093553	C	G
NC_040260.1	57153185	A	G

NC_040260.1	57211507	T	A
NC_040260.1	57282675	G	A
NC_040260.1	57338340	C	T
NC_040260.1	57388238	A	G
NC_040260.1	57442797	G	T
NC_040260.1	57477123	T	A
NC_040260.1	57532467	C	T
NC_040260.1	57585795	T	C
NC_040260.1	57609042	A	G
NC_040260.1	57610057	A	C
NC_040260.1	57610423	T	C
NC_040260.1	57610690	T	C
NC_040260.1	57611296	T	C
NC_040260.1	57611473	A	G
NC_040260.1	57666465	T	G
NC_040260.1	57726194	C	A
NC_040260.1	57780250	T	C
NC_040260.1	57780820	T	C
NC_040260.1	57781309	T	C
NC_040260.1	57782200	A	G
NC_040260.1	57784261	G	A
NC_040260.1	57785332	T	C
NC_040260.1	57795421	T	C
NC_040260.1	57796365	C	T
NC_040260.1	57796560	A	G
NC_040260.1	57797079	G	A
NC_040260.1	57798188	T	C
NC_040260.1	57850323	T	G
NC_040260.1	57908294	T	C
NC_040260.1	57930341	C	G
NC_040260.1	57978535	T	C
NC_040260.1	58023821	G	A
NC_040260.1	58080446	T	C
NC_040260.1	58122507	C	A
NC_040260.1	58181340	G	A
NC_040260.1	58237829	A	G
NC_040260.1	58296164	G	A
NC_040260.1	58351815	G	A
NC_040260.1	58427106	G	C
NC_040260.1	58526520	G	A
NC_040260.1	58581024	C	T
NC_040260.1	58636875	C	A
NC_040260.1	58692505	A	T
NC_040260.1	58744580	A	T
NC_040260.1	58976990	A	G
NC_040260.1	59020631	T	A
NC_040260.1	59084832	T	A

NC_040260.1	59143250	C	T
NC_040260.1	59203896	A	G
NC_040260.1	59263202	A	G
NC_040260.1	59317594	G	A
NC_040260.1	59373617	T	C
NC_040260.1	59431071	T	C
NC_040260.1	59476269	G	A
NC_040260.1	59606247	T	C
NC_040260.1	59659224	G	T
NC_040260.1	59708224	G	T
NC_040260.1	59760181	C	G
NC_040260.1	59806062	C	T
NC_040260.1	59857930	C	T
NC_040260.1	59879893	T	C
NC_040260.1	59923944	T	C
NC_040260.1	59980545	C	T
NC_040260.1	60038128	G	A
NC_040260.1	60095087	T	G
NC_040260.1	60188636	A	G
NC_040260.1	60240567	A	C
NC_040260.1	60280065	C	T
NC_040260.1	60335462	A	C
NC_040260.1	60377774	A	G
NC_040260.1	60429143	T	C
NC_040260.1	60462648	A	G
NC_040260.1	60520532	G	A
NC_040260.1	60576274	C	T
NC_040260.1	60645560	G	A
NC_040260.1	60693990	C	T
NC_040260.1	60748543	A	G
NC_040260.1	60799702	C	T
NC_040260.1	60854185	C	T
NC_040260.1	60892263	C	A
NC_040260.1	60949873	T	A
NC_040260.1	61006304	G	A
NC_040260.1	61052922	T	C
NC_040260.1	61053657	C	G
NC_040260.1	61108747	T	C
NC_040260.1	61168232	G	C
NC_040260.1	61221273	C	G
NC_040260.1	61283469	A	G
NC_040260.1	61340153	C	A
NC_040260.1	61389480	G	A
NC_040260.1	61441825	A	G
NC_040260.1	61496790	A	G
NC_040260.1	61555096	C	T
NC_040260.1	61611540	T	C

NC_040260.1	61667214	T	C
NC_040260.1	61731968	A	G
NC_040260.1	61755855	C	T
NC_040260.1	61763819	T	C
NC_040260.1	61783110	A	C
NC_040260.1	61784412	A	C
NC_040260.1	61839955	A	T
NC_040260.1	61913265	G	A
NC_040260.1	61914289	A	C
NC_040260.1	61915937	T	C
NC_040260.1	61918458	A	G
NC_040260.1	61922426	T	C
NC_040260.1	61982253	G	A
NC_040260.1	62043397	T	C
NC_040260.1	62077049	T	C
NC_040260.1	62186861	G	T
NC_040260.1	62219498	C	G
NC_040260.1	62272385	A	C
NC_040260.1	62319717	G	A
NC_040260.1	62321211	C	T
NC_040260.1	62322221	T	C
NC_040260.1	62369511	T	C
NC_040260.1	62433198	A	C
NC_040260.1	62474886	G	A
NC_040260.1	62530461	G	A
NC_040260.1	62590357	A	G
NC_040260.1	62645184	C	A
NC_040260.1	62714649	C	T
NC_040260.1	62771784	A	G
NC_040260.1	62796859	T	C
NC_040260.1	62829807	C	T
NC_040260.1	62899742	C	T
NC_040260.1	62952159	A	C
NC_040260.1	62997808	A	C
NC_040260.1	63051854	G	A
NC_040260.1	63128521	A	T
NC_040260.1	63153124	G	C
NC_040260.1	63217946	A	G
NC_040260.1	63247499	C	T
NC_040260.1	63252671	A	G
NC_040260.1	63280843	G	A
NC_040260.1	63337023	T	A
NC_040260.1	63412050	G	A
NC_040260.1	63465743	T	C
NC_040260.1	63521861	C	T
NC_040260.1	63572264	C	T
NC_040260.1	63637391	T	C

NC_040260.1	63693907	G	A
NC_040260.1	63775530	T	C
NC_040260.1	63848973	T	C
NC_040260.1	63905721	T	C
NC_040260.1	63959313	T	A
NC_040260.1	63999727	C	T
NC_040260.1	64056354	T	C
NC_040260.1	64119122	A	G
NC_040260.1	64220444	C	T
NC_040260.1	64272527	G	A
NC_040260.1	64329520	0	G
NC_040260.1	64378741	G	A
NC_040260.1	64435782	T	G
NC_040260.1	64498936	T	A
NC_040260.1	64555664	C	T
NC_040260.1	64613463	T	C
NC_040260.1	64669998	A	T
NC_040260.1	64727908	T	C
NC_040260.1	64789658	A	G
NC_040260.1	64840107	C	T
NC_040260.1	64895894	T	C
NC_040260.1	64950846	A	G
NC_040260.1	65017173	A	G
NC_040260.1	65078247	C	T
NC_040260.1	65134633	G	C
NC_040260.1	65194922	G	A
NC_040260.1	65233728	T	G
NC_040260.1	65365878	C	T
NC_040260.1	65413460	A	T
NC_040260.1	65473922	C	T
NC_040260.1	65523383	G	A
NC_040260.1	65581675	G	A
NC_040260.1	65644401	G	A
NC_040260.1	65688351	T	G
NC_040260.1	65730277	A	G
NC_040260.1	65795115	A	G
NC_040260.1	65823566	G	A
NC_040260.1	65882745	C	A
NC_040260.1	65942278	C	T
NC_040260.1	65999999	G	C
NC_040260.1	66050546	A	G
NC_040260.1	66123565	A	G
NC_040260.1	66183981	T	C
NC_040260.1	66247218	C	T
NC_040260.1	66302974	G	A
NC_040260.1	66357011	G	A
NC_040260.1	66384494	C	T

NC_040260.1	66566002	G	A
NC_040260.1	66679044	T	C
NC_040260.1	66733077	G	A
NC_040260.1	66766200	C	T
NC_040260.1	66832640	A	T
NC_040260.1	66845898	A	G
NC_040260.1	66906675	C	T
NC_040260.1	66965561	A	T
NC_040260.1	67031078	C	T
NC_040260.1	67082759	G	T
NC_040260.1	67138030	A	C
NC_040260.1	67193929	G	A
NC_040260.1	67250792	A	G
NC_040260.1	67296374	T	C
NC_040260.1	67355025	G	A
NC_040260.1	67411994	T	A
NC_040260.1	67465454	T	C
NC_040260.1	67521927	C	T
NC_040260.1	67580680	C	A
NC_040260.1	67634615	T	G
NC_040260.1	67692361	C	T
NC_040260.1	67745645	C	A
NC_040260.1	67805874	C	G
NC_040260.1	67861336	G	A
NC_040260.1	67917562	A	G
NC_040260.1	67988987	G	A
NC_040260.1	68045107	T	C
NC_040260.1	68093329	G	T
NC_040260.1	68130089	G	A
NC_040260.1	68191798	C	T
NC_040260.1	68257471	C	G
NC_040260.1	68315325	A	G
NC_040260.1	68315733	G	C
NC_040260.1	68318361	A	T
NC_040260.1	68375163	C	T
NC_040260.1	68428405	C	A
NC_040260.1	68484914	T	C
NC_040260.1	68543162	A	G
NC_040260.1	68604878	T	C
NC_040260.1	68663857	T	C
NC_040260.1	68725062	C	T
NC_040260.1	68780907	G	A
NC_040260.1	68834728	G	T
NC_040260.1	68881598	A	T
NC_040260.1	69114567	C	A
NC_040260.1	69176950	T	C
NC_040260.1	69211043	A	T

NC_040260.1	69375982	C	T
NC_040260.1	69439227	A	G
NC_040260.1	69493500	A	C
NC_040260.1	69526064	T	C
NC_040260.1	69576767	T	A
NC_040260.1	69635307	C	T
NC_040260.1	69692596	T	C
NC_040260.1	69742839	A	C
NC_040260.1	69794133	A	G
NC_040260.1	69847491	G	A
NC_040260.1	69915306	T	G
NC_040260.1	70068122	A	G
NC_040260.1	70122064	C	T
NC_040260.1	70203923	T	C
NC_040260.1	70269792	C	T
NC_040260.1	70329113	G	A
NC_040260.1	70388302	G	A
NC_040260.1	70417783	A	C
NC_040260.1	70702358	T	G
NC_040260.1	70759698	C	A
NC_040260.1	70825920	C	A
NC_040260.1	70917573	G	A
NC_040260.1	70940476	A	G
NC_040260.1	71137840	T	G
NC_040260.1	71163343	T	C
NC_040260.1	71219439	G	T
NC_040260.1	71282472	A	G
NC_040260.1	71350129	C	T
NC_040260.1	71371588	G	C
NC_040260.1	71442450	A	G
NC_040260.1	71501098	G	A
NC_040260.1	71564935	A	T
NC_040260.1	71630959	C	G
NC_040260.1	71694675	A	G
NC_040260.1	71719175	A	G
NC_040260.1	71835115	T	A
NC_040260.1	71886012	T	C
NC_040260.1	71948261	T	C
NC_040260.1	72014919	A	G
NC_040260.1	72065492	C	T
NC_040260.1	72120069	A	G
NC_040260.1	72181694	G	T
NC_040260.1	72198165	C	T
NC_040260.1	72198917	G	A
NC_040260.1	72259894	T	A
NC_040260.1	72315558	G	T
NC_040260.1	72373345	A	T

NC_040260.1	72411362	A	G
NC_040260.1	72465975	G	A
NC_040260.1	72510696	C	T
NC_040260.1	72570511	T	A
NC_040260.1	72640943	C	T
NC_040260.1	72690633	C	T
NC_040260.1	72734423	G	A
NC_040260.1	72768988	T	C
NC_040260.1	72843448	C	A
NC_040260.1	72890447	A	G
NC_040260.1	72941735	G	A
NC_040260.1	73061445	C	T
NC_040260.1	73120125	C	T
NC_040260.1	73264796	T	C
NC_040260.1	73315277	A	G
NC_040260.1	73365965	G	C
NC_040260.1	73420366	C	T
NC_040260.1	73459346	T	C
NC_040260.1	73540290	0	C
NC_040260.1	73598200	T	C
NC_040260.1	73656345	G	A
NC_040260.1	73708081	A	G
NC_040260.1	73767024	C	T
NC_040260.1	73823314	G	A
NC_040260.1	73883653	G	T
NC_040260.1	73952266	C	G
NC_040260.1	74013521	T	C
NC_040260.1	74070223	C	A
NC_040260.1	74111863	A	G
NC_040260.1	74169428	C	T
NC_040260.1	74226544	T	C
NC_040260.1	74283341	C	A
NC_040260.1	74343648	A	T
NC_040260.1	74399419	C	T
NC_040260.1	74454753	T	C
NC_040260.1	74529309	T	C
NC_040260.1	74535700	C	G
NC_040260.1	74591419	T	C
NC_040260.1	74668871	T	C
NC_040260.1	74747715	T	C
NC_040260.1	74788250	C	T
NC_040260.1	74833830	G	C
NC_040260.1	74895908	C	T
NC_040260.1	74950758	C	T
NC_040260.1	75009905	A	G
NC_040260.1	75051233	C	G
NC_040260.1	75107175	G	A

NC_040260.1	75126429	C	G
NC_040260.1	75178642	C	A
NC_040260.1	75223752	A	G
NC_040260.1	75245738	A	G
NC_040260.1	75636020	G	A
NC_040260.1	75656281	A	G
NC_040260.1	75718705	A	G
NC_040260.1	75789108	C	T
NC_040260.1	75846440	T	C
NC_040260.1	75908324	C	T
NC_040260.1	75974722	C	T
NC_040260.1	76037151	C	T
NC_040260.1	76106300	C	T
NC_040260.1	76177227	T	C
NC_040260.1	76242389	G	C
NC_040260.1	76316839	A	T
NC_040260.1	76376605	C	G
NC_040260.1	76424082	A	G
NC_040260.1	76590057	A	G
NC_040260.1	76624283	A	G
NC_040260.1	76648532	G	A
NC_040260.1	76707439	G	C
NC_040260.1	76760972	T	G
NC_040260.1	76895547	C	T
NC_040260.1	76958210	G	A
NC_040260.1	77019349	G	A
NC_040260.1	77037015	G	A
NC_040260.1	77038513	C	T
NC_040260.1	77093825	C	T
NC_040260.1	77144473	T	C
NC_040260.1	77201781	C	T
NC_040260.1	77266197	G	A
NC_040260.1	77322665	A	G
NC_040260.1	77381161	C	G
NC_040260.1	77438649	T	C
NC_040260.1	77469699	C	T
NC_040260.1	77529182	T	C
NC_040260.1	77588337	G	A
NC_040260.1	77796658	C	T
NC_040260.1	77850705	T	G
NC_040260.1	77914489	T	C
NC_040260.1	77925626	T	C
NC_040260.1	77991916	A	G
NC_040260.1	78031805	C	A
NC_040260.1	78095237	A	G
NC_040260.1	78164265	T	C
NC_040260.1	78217750	T	C

NC_040260.1	78304752	C	T
NC_040260.1	78366495	C	T
NC_040260.1	78436366	C	T
NC_040260.1	78489296	T	C
NC_040260.1	78506222	T	C
NC_040260.1	78550072	G	C
NC_040260.1	78553237	C	T
NC_040260.1	78618259	A	G
NC_040260.1	78689152	G	A
NC_040260.1	78752081	G	A
NC_040260.1	78835203	G	A
NC_040260.1	78900041	T	C
NC_040260.1	78952972	C	G
NC_040260.1	79007610	T	C
NC_040260.1	79065445	A	G
NC_040260.1	79112852	C	A
NC_040260.1	79180409	A	G
NC_040260.1	79235513	T	C
NC_040260.1	79290898	G	A
NC_040260.1	79351547	T	C
NC_040260.1	79403737	C	T
NC_040260.1	79653260	C	G
NC_040260.1	79654575	A	G
NC_040260.1	79711912	A	C
NC_040260.1	79780978	C	T
NC_040260.1	79842595	A	G
NC_040260.1	79901384	C	A
NC_040260.1	79964555	G	C
NC_040260.1	80019047	T	C
NC_040260.1	80081757	T	C
NC_040260.1	80143991	T	C
NC_040260.1	80171908	G	A
NC_040260.1	80236201	G	C
NC_040260.1	80305766	G	A
NC_040260.1	80360079	T	C
NC_040260.1	80417599	C	A
NC_040260.1	80474969	A	G
NC_040260.1	80530836	A	T
NC_040260.1	80583763	A	T
NC_040260.1	80641367	G	A
NC_040260.1	80703679	A	C
NC_040260.1	80753612	C	G
NC_040260.1	80818281	G	A
NC_040260.1	80850955	A	G
NC_040260.1	80921100	C	T
NC_040260.1	80986140	G	A
NC_040260.1	81043649	G	A

NC_040260.1	81095700	T	C
NC_040260.1	81164899	A	G
NC_040260.1	81225932	A	G
NC_040260.1	81274064	A	G
NC_040260.1	81330981	T	G
NC_040260.1	81372364	C	T
NC_040260.1	81401445	C	T
NC_040260.1	81464455	G	A
NC_040260.1	81530656	C	T
NC_040260.1	81584096	T	C
NC_040260.1	81652285	T	A
NC_040260.1	81724451	A	G
NC_040260.1	81765627	T	C
NC_040260.1	81825401	T	C
NC_040260.1	81841734	G	C
NC_040260.1	81868970	T	C
NC_040260.1	81900714	T	C
NC_040260.1	81902424	G	A
NC_040260.1	81902898	T	C
NC_040260.1	81953721	G	C
NC_040260.1	81996538	T	C
NC_040260.1	82001110	A	G
NC_040260.1	82025132	A	G
NC_040260.1	82086027	A	G
NC_040260.1	82086622	T	C
NC_040260.1	82130394	G	C
NC_040260.1	82196416	A	G
NC_040260.1	82239715	C	T
NC_040260.1	82243515	T	C
NC_040260.1	82280114	G	A
NC_040260.1	82347173	C	T
NC_040260.1	82419130	C	T
NC_040260.1	82447623	T	C
NC_040260.1	82502764	C	G
NC_040260.1	82557803	C	T
NC_040260.1	82600288	T	A
NC_040260.1	82603178	A	G
NC_040260.1	82605752	C	T
NC_040260.1	82677914	G	A
NC_040260.1	82738136	C	T
NC_040260.1	82793988	T	C
NC_040260.1	82815082	A	T
NC_040260.1	82852957	G	A
NC_040260.1	82887983	T	C
NC_040260.1	82926537	G	C
NC_040260.1	82937702	T	C
NC_040260.1	82952026	A	G

NC_040260.1	82953613	A	G
NC_040260.1	82994022	A	G
NC_040260.1	83050967	G	A
NC_040260.1	83122456	T	C
NC_040260.1	83196308	A	G
NC_040260.1	83252562	G	A
NC_040260.1	83316986	G	C
NC_040260.1	83377491	T	G
NC_040260.1	83445421	A	G
NC_040260.1	83502372	T	C
NC_040260.1	83544824	G	A
NC_040260.1	83548797	G	A
NC_040260.1	83609630	A	G
NC_040260.1	83641992	G	A
NC_040260.1	83699090	C	T
NC_040260.1	83720632	A	G
NC_040260.1	83778949	A	G
NC_040260.1	83831051	A	G
NC_040260.1	83862403	T	C
NC_040260.1	83915073	G	A
NC_040260.1	83983423	A	T
NC_040260.1	83991169	C	T
NC_040260.1	83992154	G	A
NC_040260.1	84051521	G	A
NC_040260.1	84108999	T	C
NC_040260.1	84165298	A	C
NC_040260.1	84225238	C	T
NC_040260.1	84250352	T	G
NC_040260.1	84307004	T	C
NC_040260.1	84358049	A	G
NC_040260.1	84416378	G	C
NC_040260.1	84442695	A	G
NC_040260.1	84487808	G	A
NC_040260.1	84522450	G	A
NC_040260.1	84586053	T	C
NC_040260.1	84652125	C	A
NC_040260.1	84680716	C	T
NC_040260.1	84737876	T	G
NC_040260.1	84792106	C	T
NC_040260.1	84852429	T	C
NC_040260.1	84871245	C	G
NC_040260.1	84895576	G	A
NC_040260.1	84921702	C	T
NC_040260.1	84922175	T	G
NC_040260.1	84963212	C	T
NC_040260.1	84994147	C	G
NC_040260.1	85031463	C	A

NC_040260.1	85044931	C	T
NC_040260.1	85081583	A	C
NC_040260.1	85121410	A	G
NC_040260.1	85184138	T	C
NC_040260.1	85237026	T	C
NC_040260.1	85260854	T	C
NC_040260.1	85283125	A	G
NC_040260.1	85312202	T	C
NC_040260.1	85374084	A	G
NC_040260.1	85424829	C	T
NC_040260.1	85486552	A	G
NC_040260.1	85543153	A	G
NC_040260.1	85581630	T	C
NC_040260.1	85616408	T	C
NC_040260.1	85652462	A	G
NC_040260.1	85690852	T	C
NC_040260.1	85724580	A	G
NC_040260.1	85785461	C	T
NC_040260.1	85848445	C	G
NC_040260.1	85889994	T	C
NC_040260.1	85935217	C	T
NC_040260.1	85952880	T	A
NC_040260.1	85995866	C	T
NC_040260.1	86043564	G	C
NC_040260.1	86100440	A	T
NC_040260.1	86118326	G	A
NC_040260.1	86126063	A	C
NC_040260.1	86178979	A	G
NC_040260.1	86211397	T	A
NC_040260.1	86264877	T	G
NC_040260.1	86325472	G	A
NC_040260.1	86386144	T	A
NC_040260.1	86444212	T	C
NC_040260.1	86500411	G	A
NC_040260.1	86560377	G	A
NC_040260.1	86608897	T	G
NC_040260.1	86609522	T	C
NC_040260.1	86610818	G	C
NC_040260.1	86669840	A	G
NC_040260.1	86725079	T	G
NC_040260.1	86748446	A	G
NC_040260.1	86791908	A	G
NC_040260.1	86832692	G	A
NC_040260.1	86864913	T	C
NC_040260.1	86949541	T	C
NC_040260.1	87017641	G	A
NC_040260.1	87066425	A	G

NC_040260.1	87124847	G	A
NC_040260.1	87179765	A	G
NC_040260.1	87227021	G	A
NC_040260.1	87266071	C	G
NC_040260.1	87336239	T	A
NC_040260.1	87338374	C	T
NC_040260.1	87386779	G	A
NC_040260.1	87455921	T	C
NC_040260.1	87507217	T	C
NC_040260.1	87550778	A	G
NC_040260.1	87673252	T	C
NC_040260.1	87742371	G	C
NC_040260.1	87801030	A	C
NC_040260.1	87851908	A	G
NC_040260.1	88055296	C	A
NC_040260.1	88113984	T	G
NC_040260.1	88177927	C	A
NC_040260.1	88231955	T	C
NC_040260.1	88271980	C	A
NC_040260.1	88330937	T	C
NC_040260.1	88388641	T	C
NC_040260.1	88435454	T	C
NC_040260.1	88497061	C	T
NC_040260.1	88518617	C	T
NC_040260.1	88589497	A	G
NC_040260.1	88606677	C	A
NC_040260.1	88642052	A	G
NC_040260.1	88693173	T	C
NC_040260.1	88747229	T	C
NC_040260.1	88762453	A	G
NC_040260.1	88829093	A	G
NC_040260.1	88900230	T	A
NC_040260.1	88970145	G	A
NC_040260.1	89027866	G	A
NC_040260.1	89044003	T	G
NC_040260.1	89113910	G	A
NC_040260.1	89125955	G	A
NC_040260.1	89185943	T	C
NC_040260.1	89241460	A	G
NC_040260.1	89299591	T	C
NC_040260.1	89361515	T	C
NC_040260.1	89418923	T	A
NC_040260.1	89476192	A	G
NC_040260.1	89536118	C	T
NC_040260.1	89602343	C	T
NC_040260.1	89658083	A	T
NC_040260.1	89732151	A	G

NC_040260.1	89783657	A	G
NC_040260.1	89842853	G	A
NC_040260.1	89913515	C	A
NC_040260.1	89971509	C	T
NC_040260.1	90030345	T	C
NC_040260.1	90086378	G	A
NC_040260.1	90124507	G	A
NC_040260.1	90145420	A	G
NC_040260.1	90193989	T	C
NC_040260.1	90247647	A	C
NC_040260.1	90262803	C	G
NC_040260.1	90364479	C	T
NC_040260.1	90376797	A	G
NC_040260.1	90431411	C	T
NC_040260.1	90471121	A	C
NC_040260.1	90525936	C	T
NC_040260.1	90574505	C	T
NC_040260.1	90629002	A	G
NC_040260.1	90685053	G	A
NC_040260.1	90737234	T	C
NC_040260.1	90798168	A	T
NC_040260.1	90842014	T	C
NC_040260.1	90871152	T	C
NC_040260.1	90875111	G	A
NC_040260.1	90883000	C	T
NC_040260.1	90928973	C	T
NC_040260.1	90970923	G	C
NC_040260.1	90972245	C	T
NC_040260.1	90972963	G	T
NC_040260.1	90996976	C	G
NC_040260.1	91023600	A	G
NC_040260.1	91101357	C	T
NC_040260.1	91115061	C	A
NC_040260.1	91163376	T	G
NC_040260.1	91209847	C	A
NC_040260.1	91314859	G	A
NC_040260.1	91370337	A	C
NC_040260.1	91422464	G	A
NC_040260.1	91494073	A	G
NC_040260.1	91529831	G	C
NC_040260.1	91587470	T	A
NC_040260.1	91633741	T	C
NC_040260.1	91667663	C	T
NC_040260.1	91672272	T	C
NC_040260.1	91715927	T	C
NC_040260.1	91717127	C	A
NC_040260.1	91718953	C	T

NC_040260.1	91747927	G	A
NC_040260.1	91804728	A	T
NC_040260.1	91871482	T	C
NC_040260.1	91966782	A	C
NC_040260.1	92017230	A	G
NC_040260.1	92072842	G	C
NC_040260.1	92128382	A	G
NC_040260.1	92182720	G	A
NC_040260.1	92235959	T	C
NC_040260.1	92322643	G	A
NC_040260.1	92375898	T	A
NC_040260.1	92432116	A	G
NC_040260.1	92476161	A	G
NC_040260.1	92547377	C	T
NC_040260.1	92587355	A	G
NC_040260.1	92588433	C	T
NC_040260.1	92589028	T	C
NC_040260.1	92783528	T	C
NC_040260.1	92833332	T	C
NC_040260.1	93022353	T	C
NC_040260.1	93101778	G	A
NC_040260.1	93164247	C	T
NC_040260.1	93215205	G	C
NC_040260.1	93268245	T	C
NC_040260.1	93325195	G	A
NC_040260.1	93383740	A	G
NC_040260.1	93443878	A	C
NC_040260.1	93484084	T	C
NC_040260.1	93559971	A	G
NC_040260.1	93665682	T	G
NC_040260.1	93725652	T	A
NC_040260.1	93776560	T	A
NC_040260.1	93838061	A	C
NC_040260.1	93891175	C	T
NC_040260.1	93919260	C	T
NC_040260.1	93984741	C	A
NC_040260.1	94036016	C	T
NC_040260.1	94053503	C	T
NC_040260.1	94246148	T	C
NC_040260.1	94289491	A	C
NC_040260.1	94340850	T	G
NC_040260.1	94380088	A	C
NC_040260.1	94534795	T	A
NC_040260.1	94564783	T	A
NC_040260.1	94628282	T	A
NC_040260.1	94744256	T	C
NC_040260.1	94751542	C	G

NC_040260.1	94803602	C	T
NC_040260.1	94875451	C	T
NC_040260.1	94929496	G	A
NC_040260.1	94967474	A	G
NC_040260.1	95005060	C	T
NC_040260.1	95056332	C	T
NC_040260.1	95064147	C	G
NC_040260.1	95133439	G	T
NC_040260.1	95180776	G	A
NC_040260.1	95181794	A	C
NC_040260.1	95220004	T	C
NC_040260.1	95253047	G	A
NC_040260.1	95303075	T	G
NC_040260.1	95337756	G	A
NC_040260.1	95407038	G	A
NC_040260.1	95465343	T	A
NC_040260.1	95518879	G	A
NC_040260.1	95583443	A	G
NC_040260.1	95606620	G	A
NC_040260.1	95662436	A	C
NC_040260.1	95726947	C	T
NC_040260.1	95790578	T	C
NC_040260.1	96032911	T	G
NC_040260.1	96165081	A	G
NC_040260.1	96227570	A	T
NC_040260.1	96294592	A	G
NC_040260.1	96374019	G	A
NC_040260.1	96430181	G	T
NC_040260.1	96503458	C	T
NC_040260.1	96571386	C	T
NC_040260.1	96629527	T	C
NC_040260.1	96673983	T	C
NC_040260.1	96674571	C	G
NC_040260.1	96677363	T	A
NC_040260.1	96679040	T	C
NC_040260.1	96755457	C	T
NC_040260.1	96776724	G	A
NC_040260.1	96901950	G	T
NC_040260.1	96965750	C	G
NC_040260.1	97028870	G	A
NC_040260.1	97079230	G	A
NC_040260.1	97152664	C	T
NC_040260.1	97205105	C	T
NC_040260.1	97260894	C	T
NC_040260.1	97307337	G	T
NC_040260.1	97389414	T	G
NC_040260.1	97415373	0	T

NC_040260.1	97498229	A	T
NC_040260.1	97575903	A	G
NC_040260.1	97603707	T	C
NC_040260.1	97664771	G	A
NC_040260.1	97729698	C	G
NC_040260.1	97748404	A	G
NC_040260.1	97811781	T	C
NC_040260.1	97878387	T	C
NC_040260.1	97938248	G	C
NC_040260.1	98002737	A	G
NC_040260.1	98039349	A	G
NC_040260.1	98093776	A	G
NC_040260.1	98148481	A	G
NC_040260.1	98193368	A	G
NC_040260.1	98222410	T	C
NC_040260.1	98298803	T	C
NC_040260.1	98359948	A	G
NC_040260.1	98424521	A	T
NC_040260.1	98459205	C	T
NC_040260.1	98504965	G	A
NC_040260.1	98562935	T	C
NC_040260.1	98639654	G	A
NC_040260.1	98693513	G	T
NC_040260.1	98717476	C	T
NC_040260.1	98750963	T	C
NC_040260.1	98833722	T	G
NC_040260.1	98897834	G	A
NC_040260.1	98955323	A	G
NC_040260.1	99013055	T	G
NC_040260.1	99069749	T	C
NC_040260.1	99121195	T	C
NC_040260.1	99163015	T	C
NC_040260.1	99233403	G	A
NC_040260.1	99459300	C	T
NC_040260.1	99517451	T	G
NC_040260.1	99581615	C	A
NC_040260.1	99619880	T	C
NC_040260.1	99673192	A	G
NC_040260.1	99716475	G	T
NC_040260.1	99751431	A	G
NC_040260.1	99803587	A	G
NC_040260.1	99862326	T	C
NC_040260.1	99916815	C	T
NC_040260.1	99983036	T	C
NC_040260.1	100044282	C	T
NC_040260.1	100104658	T	C
NC_040260.1	100172750	G	T

NC_040260.1	100211019	T	C
NC_040260.1	100296209	T	C
NC_040260.1	100340639	A	G
NC_040260.1	100408360	T	C
NC_040260.1	100456022	G	T
NC_040260.1	100535552	A	C
NC_040260.1	100596449	C	T
NC_040260.1	100662150	C	T
NC_040260.1	100726644	G	T
NC_040260.1	100792299	G	T
NC_040260.1	100878859	T	A
NC_040260.1	100934988	T	C
NC_040260.1	100973882	G	A
NC_040260.1	101026723	C	T
NC_040260.1	101084710	G	A
NC_040260.1	101143019	C	A
NC_040260.1	101197502	C	A
NC_040260.1	101262839	T	A
NC_040260.1	101324241	C	T
NC_040260.1	101399971	T	C
NC_040260.1	101467466	T	C
NC_040260.1	101530513	G	T
NC_040260.1	101591720	C	G
NC_040260.1	101648991	C	T
NC_040260.1	101710523	C	T
NC_040260.1	101779592	G	A
NC_040260.1	101856266	A	C
NC_040260.1	101948503	A	G
NC_040260.1	102005110	G	A
NC_040260.1	102063536	T	A
NC_040260.1	102117567	A	G
NC_040260.1	102199788	T	C
NC_040260.1	102264678	T	C
NC_040260.1	102322038	T	C
NC_040260.1	102395467	C	T
NC_040260.1	102448581	C	A
NC_040260.1	102533045	A	C
NC_040260.1	102587321	C	T
NC_040260.1	102660922	T	C
NC_040260.1	102715039	G	A
NC_040260.1	102862160	T	G
NC_040260.1	102887361	A	G
NC_040260.1	102988969	A	G
NC_040260.1	103045267	C	G
NC_040260.1	103092383	C	T
NC_040260.1	103148998	A	G
NC_040260.1	103190207	G	C

NC_040260.1	103190554	G	A
NC_040260.1	103191607	A	G
NC_040260.1	103221962	C	T
NC_040260.1	103280262	A	G
NC_040260.1	103289816	T	C
NC_040260.1	103313811	G	C
NC_040260.1	103317562	C	T
NC_040260.1	103318508	T	G
NC_040260.1	103334818	A	G
NC_040260.1	103347677	T	C
NC_040260.1	103408047	A	G
NC_040260.1	103442369	G	A
NC_040260.1	103495824	G	C
NC_040260.1	103535901	C	T
NC_040260.1	103598698	A	G
NC_040260.1	103617075	T	C
NC_040260.1	103661723	T	C
NC_040260.1	103724200	A	G
NC_040260.1	103746338	G	A
NC_040260.1	103811451	T	C
NC_040260.1	103885578	T	C
NC_040260.1	103958189	C	T
NC_040260.1	104009720	G	T
NC_040260.1	104061671	A	G
NC_040260.1	104105814	G	A
NC_040260.1	104137001	T	C
NC_040260.1	104176667	C	T
NC_040260.1	104225365	C	A
NC_040260.1	104247488	G	A
NC_040260.1	104308915	A	T
NC_040260.1	104365649	T	C
NC_040260.1	104396797	T	C
NC_040260.1	104440459	A	G
NC_040260.1	104494854	A	G
NC_040260.1	104554339	G	T
NC_040260.1	104610084	A	G
NC_040260.1	104667115	A	T
NC_040261.1	16141	G	C
NC_040261.1	74195	T	G
NC_040261.1	125304	C	T
NC_040261.1	166604	G	A
NC_040261.1	180426	G	A
NC_040261.1	228369	G	A
NC_040261.1	254670	C	A
NC_040261.1	271783	G	A
NC_040261.1	304755	T	C
NC_040261.1	406153	T	G

NC_040261.1	460564	T	A
NC_040261.1	495733	A	G
NC_040261.1	513041	A	G
NC_040261.1	574410	G	T
NC_040261.1	616635	A	T
NC_040261.1	646463	C	T
NC_040261.1	664650	A	G
NC_040261.1	727158	A	G
NC_040261.1	781575	T	G
NC_040261.1	839499	A	G
NC_040261.1	997113	T	G
NC_040261.1	1057684	C	A
NC_040261.1	1063501	G	A
NC_040261.1	1065503	C	T
NC_040261.1	1066295	T	C
NC_040261.1	1066615	G	A
NC_040261.1	1066897	A	C
NC_040261.1	1067634	G	A
NC_040261.1	1067895	C	T
NC_040261.1	1068270	T	G
NC_040261.1	1068738	A	T
NC_040261.1	1123318	T	G
NC_040261.1	1150321	G	C
NC_040261.1	1179641	C	T
NC_040261.1	1238134	G	C
NC_040261.1	1286542	A	C
NC_040261.1	1345294	G	A
NC_040261.1	1394106	C	T
NC_040261.1	1394352	T	G
NC_040261.1	1431237	T	G
NC_040261.1	1621131	A	T
NC_040261.1	1645259	A	G
NC_040261.1	1672213	T	A
NC_040261.1	1684473	A	G
NC_040261.1	2037581	T	A
NC_040261.1	2193467	A	C
NC_040261.1	2223861	C	G
NC_040261.1	2228807	A	C
NC_040261.1	2294911	C	A
NC_040261.1	2514869	T	C
NC_040261.1	2568407	C	T
NC_040261.1	2625930	T	C
NC_040261.1	2660389	C	T
NC_040261.1	2682315	T	C
NC_040261.1	2682481	T	C
NC_040261.1	2732590	A	G
NC_040261.1	2788570	T	C

NC_040261.1	2845812	C	T
NC_040261.1	2904761	T	A
NC_040261.1	2930693	A	G
NC_040261.1	2987221	T	C
NC_040261.1	3040818	C	T
NC_040261.1	3083117	T	C
NC_040261.1	3094889	G	C
NC_040261.1	3123387	T	C
NC_040261.1	3137402	G	A
NC_040261.1	3188074	G	A
NC_040261.1	3194583	T	C
NC_040261.1	3249212	T	C
NC_040261.1	3302691	G	A
NC_040261.1	3366183	G	A
NC_040261.1	3405217	C	A
NC_040261.1	3460322	C	G
NC_040261.1	3513770	A	C
NC_040261.1	3567105	T	C
NC_040261.1	3618311	A	G
NC_040261.1	3675951	C	T
NC_040261.1	3731720	A	T
NC_040261.1	3768946	T	C
NC_040261.1	3797969	A	G
NC_040261.1	3825127	C	T
NC_040261.1	3861550	G	A
NC_040261.1	3869928	A	T
NC_040261.1	3933766	C	T
NC_040261.1	3971316	A	G
NC_040261.1	4011312	A	G
NC_040261.1	4118338	G	A
NC_040261.1	4189187	T	C
NC_040261.1	4250586	C	T
NC_040261.1	4292215	G	C
NC_040261.1	4334538	C	A
NC_040261.1	4340639	C	G
NC_040261.1	4392093	T	A
NC_040261.1	4432989	T	C
NC_040261.1	4446203	G	A
NC_040261.1	4474623	A	G
NC_040261.1	4493598	T	A
NC_040261.1	4557364	A	G
NC_040261.1	4784680	G	A
NC_040261.1	4906991	A	G
NC_040261.1	4989813	T	G
NC_040261.1	5045646	T	C
NC_040261.1	5101741	G	A
NC_040261.1	5143635	A	G

NC_040261.1	5253517	C	A
NC_040261.1	5327953	A	G
NC_040261.1	5381996	G	A
NC_040261.1	5420370	G	C
NC_040261.1	5444483	A	G
NC_040261.1	5591874	A	T
NC_040261.1	5619800	T	G
NC_040261.1	5651893	A	G
NC_040261.1	5669485	C	A
NC_040261.1	5955611	G	C
NC_040261.1	6142166	G	A
NC_040261.1	6199404	A	C
NC_040261.1	6218422	T	A
NC_040261.1	6359328	T	A
NC_040261.1	6398485	C	T
NC_040261.1	6422048	T	G
NC_040261.1	6479379	A	T
NC_040261.1	6510264	T	A
NC_040261.1	6523996	A	G
NC_040261.1	6634290	T	C
NC_040261.1	6688521	G	A
NC_040261.1	6714229	T	C
NC_040261.1	6737532	C	G
NC_040261.1	6840898	C	A
NC_040261.1	6942456	G	A
NC_040261.1	6987207	C	G
NC_040261.1	6998010	T	C
NC_040261.1	7013783	A	G
NC_040261.1	7083069	A	T
NC_040261.1	7129294	A	G
NC_040261.1	7248871	A	G
NC_040261.1	7280924	A	G
NC_040261.1	7285613	T	A
NC_040261.1	7639282	C	T
NC_040261.1	8012843	A	G
NC_040261.1	8156200	T	C
NC_040261.1	8230077	T	G
NC_040261.1	8284615	T	G
NC_040261.1	8406211	G	C
NC_040261.1	8471305	A	G
NC_040261.1	8503997	T	C
NC_040261.1	8576242	C	T
NC_040261.1	8608936	G	A
NC_040261.1	8656332	C	A
NC_040261.1	8730298	A	G
NC_040261.1	8769823	C	A
NC_040261.1	8792538	C	T

NC_040261.1	8861162	G	A
NC_040261.1	8899502	A	C
NC_040261.1	9330682	T	C
NC_040261.1	9570381	C	T
NC_040261.1	9615788	T	C
NC_040261.1	9648976	C	G
NC_040261.1	9727194	C	T
NC_040261.1	10049671	A	G
NC_040261.1	10132840	T	C
NC_040261.1	10289764	T	C
NC_040261.1	10317069	T	C
NC_040261.1	10408717	G	A
NC_040261.1	10544890	A	G
NC_040261.1	10571688	C	A
NC_040261.1	10616146	C	T
NC_040261.1	10650810	G	A
NC_040261.1	10685055	T	G
NC_040261.1	10706174	A	G
NC_040261.1	10732633	A	T
NC_040261.1	10781119	C	T
NC_040261.1	10788222	C	T
NC_040261.1	10844492	G	A
NC_040261.1	10881669	G	A
NC_040261.1	10922915	T	C
NC_040261.1	10954117	G	A
NC_040261.1	11040728	A	G
NC_040261.1	11067871	A	C
NC_040261.1	11126337	G	T
NC_040261.1	11182868	A	G
NC_040261.1	11236398	A	T
NC_040261.1	11269699	G	A
NC_040261.1	11293304	A	G
NC_040261.1	11355459	C	G
NC_040261.1	11358846	G	A
NC_040261.1	11553839	T	G
NC_040261.1	11623299	A	G
NC_040261.1	11682036	T	C
NC_040261.1	11715218	T	A
NC_040261.1	11736135	A	G
NC_040261.1	11766586	T	C
NC_040261.1	11785336	T	C
NC_040261.1	11836934	C	T
NC_040261.1	11891474	C	A
NC_040261.1	11927178	G	A
NC_040261.1	11960426	C	T
NC_040261.1	12001071	C	T
NC_040261.1	12033373	A	G

NC_040261.1	12092250	A	G
NC_040261.1	12185920	A	G
NC_040261.1	12236894	G	A
NC_040261.1	12260957	C	T
NC_040261.1	12330759	A	G
NC_040261.1	12389976	A	G
NC_040261.1	12457817	A	G
NC_040261.1	12517061	T	C
NC_040261.1	12567205	T	C
NC_040261.1	12593663	T	C
NC_040261.1	12630133	T	C
NC_040261.1	12683026	G	A
NC_040261.1	12712865	T	C
NC_040261.1	12750468	C	A
NC_040261.1	12764548	C	T
NC_040261.1	12814537	A	G
NC_040261.1	12824670	A	G
NC_040261.1	12863485	T	C
NC_040261.1	12882779	T	C
NC_040261.1	12972129	A	G
NC_040261.1	13025380	A	G
NC_040261.1	13029393	T	C
NC_040261.1	13090153	C	T
NC_040261.1	13107841	T	C
NC_040261.1	13169207	A	G
NC_040261.1	13202403	A	G
NC_040261.1	13236005	G	A
NC_040261.1	13253475	T	C
NC_040261.1	13318934	C	T
NC_040261.1	13387817	A	C
NC_040261.1	13426075	G	A
NC_040261.1	13468527	T	C
NC_040261.1	13497992	C	G
NC_040261.1	13557873	C	T
NC_040261.1	13581400	G	A
NC_040261.1	13633958	C	T
NC_040261.1	13679221	C	T
NC_040261.1	13706812	A	G
NC_040261.1	13745104	A	C
NC_040261.1	13807292	C	T
NC_040261.1	13856663	G	A
NC_040261.1	13863231	A	C
NC_040261.1	13909263	T	C
NC_040261.1	13955506	C	T
NC_040261.1	14006618	T	C
NC_040261.1	14046023	T	C
NC_040261.1	14090823	T	C

NC_040261.1	14104962	C	T
NC_040261.1	14155016	C	A
NC_040261.1	14186941	A	G
NC_040261.1	14230367	T	G
NC_040261.1	14334707	A	C
NC_040261.1	14365086	T	C
NC_040261.1	14447218	T	C
NC_040261.1	14473445	C	T
NC_040261.1	14484910	A	G
NC_040261.1	14515486	A	G
NC_040261.1	14567195	T	C
NC_040261.1	14623835	A	T
NC_040261.1	14657577	A	G
NC_040261.1	14703310	G	A
NC_040261.1	14714911	A	G
NC_040261.1	14751585	T	C
NC_040261.1	14759176	C	T
NC_040261.1	14813999	G	A
NC_040261.1	14837655	T	C
NC_040261.1	14893539	C	T
NC_040261.1	14902318	C	T
NC_040261.1	14948927	C	G
NC_040261.1	15068803	T	A
NC_040261.1	15173171	G	A
NC_040261.1	15251116	T	C
NC_040261.1	15297546	T	C
NC_040261.1	15344788	G	C
NC_040261.1	15388790	A	G
NC_040261.1	15394359	T	C
NC_040261.1	15427579	C	T
NC_040261.1	15542370	A	C
NC_040261.1	15585947	C	T
NC_040261.1	15612798	T	C
NC_040261.1	15659887	A	G
NC_040261.1	15666490	T	C
NC_040261.1	15719981	A	G
NC_040261.1	15754758	A	G
NC_040261.1	15777685	T	C
NC_040261.1	15815232	A	G
NC_040261.1	15823550	G	A
NC_040261.1	15851833	C	T
NC_040261.1	15919003	C	G
NC_040261.1	15991684	T	C
NC_040261.1	16029871	A	G
NC_040261.1	16041016	C	T
NC_040261.1	16101158	T	C
NC_040261.1	16156337	T	C

NC_040261.1	16206184	G	C
NC_040261.1	16257382	G	A
NC_040261.1	16314729	G	C
NC_040261.1	16369966	C	T
NC_040261.1	16392841	G	A
NC_040261.1	16509274	G	A
NC_040261.1	16547595	T	C
NC_040261.1	16555359	T	C
NC_040261.1	16617071	A	G
NC_040261.1	16645047	A	G
NC_040261.1	16666825	T	C
NC_040261.1	16726006	G	A
NC_040261.1	16780612	A	C
NC_040261.1	16818310	C	A
NC_040261.1	16866365	C	T
NC_040261.1	16922841	A	G
NC_040261.1	16932450	T	C
NC_040261.1	17048082	G	C
NC_040261.1	17112570	A	G
NC_040261.1	17172599	T	A
NC_040261.1	17209708	G	A
NC_040261.1	17226676	A	G
NC_040261.1	17255855	A	C
NC_040261.1	17286250	T	G
NC_040261.1	17337186	A	G
NC_040261.1	17397531	G	C
NC_040261.1	17440278	A	G
NC_040261.1	17465869	C	A
NC_040261.1	17500983	G	A
NC_040261.1	17531451	A	G
NC_040261.1	17567040	A	T
NC_040261.1	17620417	G	C
NC_040261.1	17651274	A	G
NC_040261.1	17707291	C	T
NC_040261.1	17751713	A	G
NC_040261.1	17818807	T	G
NC_040261.1	17871594	A	G
NC_040261.1	17878330	G	A
NC_040261.1	17943194	G	A
NC_040261.1	17988096	C	G
NC_040261.1	18037157	A	G
NC_040261.1	18154944	G	C
NC_040261.1	18194676	A	G
NC_040261.1	18256870	A	G
NC_040261.1	18320721	G	A
NC_040261.1	18370947	C	T
NC_040261.1	18409821	T	C

NC_040261.1	18442570	G	A
NC_040261.1	18503220	A	G
NC_040261.1	18545312	T	C
NC_040261.1	18574690	G	A
NC_040261.1	18599839	G	T
NC_040261.1	18602933	A	G
NC_040261.1	18673305	G	C
NC_040261.1	18726409	T	G
NC_040261.1	18755937	A	G
NC_040261.1	18776628	G	T
NC_040261.1	18834291	A	G
NC_040261.1	18892643	T	C
NC_040261.1	18940431	T	C
NC_040261.1	19025170	G	C
NC_040261.1	19048572	C	T
NC_040261.1	19106880	A	G
NC_040261.1	19157897	T	C
NC_040261.1	19202108	T	G
NC_040261.1	19233425	A	G
NC_040261.1	19341668	T	A
NC_040261.1	19380258	T	C
NC_040261.1	19429068	T	G
NC_040261.1	19444595	T	C
NC_040261.1	19520777	A	T
NC_040261.1	19588536	G	A
NC_040261.1	19641359	T	C
NC_040261.1	19677141	C	T
NC_040261.1	19682801	A	T
NC_040261.1	19750605	T	C
NC_040261.1	19783747	A	G
NC_040261.1	19858368	G	A
NC_040261.1	19921827	A	G
NC_040261.1	20036339	T	C
NC_040261.1	20071605	A	C
NC_040261.1	20130900	G	T
NC_040261.1	20171154	A	G
NC_040261.1	20224557	A	G
NC_040261.1	20283165	A	G
NC_040261.1	20328646	T	C
NC_040261.1	20376217	A	G
NC_040261.1	20413316	T	C
NC_040261.1	20454111	C	T
NC_040261.1	20495239	T	C
NC_040261.1	20532132	C	T
NC_040261.1	20552826	T	C
NC_040261.1	20601396	A	G
NC_040261.1	20645787	G	C

NC_040261.1	20675677	G	A
NC_040261.1	20702878	G	A
NC_040261.1	20763711	T	C
NC_040261.1	20795920	A	G
NC_040261.1	20815945	G	A
NC_040261.1	20869410	T	C
NC_040261.1	20925350	G	A
NC_040261.1	20962879	G	T
NC_040261.1	20980914	C	T
NC_040261.1	21047025	A	T
NC_040261.1	21092899	A	C
NC_040261.1	21103412	G	A
NC_040261.1	21113868	T	C
NC_040261.1	21124763	G	A
NC_040261.1	21153094	T	G
NC_040261.1	21204257	G	T
NC_040261.1	21244014	T	C
NC_040261.1	21269184	A	G
NC_040261.1	21309687	C	T
NC_040261.1	21329071	A	G
NC_040261.1	21384387	C	T
NC_040261.1	21434005	G	C
NC_040261.1	21477536	A	G
NC_040261.1	21572054	T	C
NC_040261.1	21621177	C	A
NC_040261.1	21672740	T	C
NC_040261.1	21712166	T	C
NC_040261.1	21725095	A	T
NC_040261.1	21765252	T	C
NC_040261.1	21781980	C	G
NC_040261.1	21834365	T	C
NC_040261.1	21887875	A	T
NC_040261.1	21948925	C	T
NC_040261.1	22005241	T	C
NC_040261.1	22059077	A	G
NC_040261.1	22114816	G	A
NC_040261.1	22150308	G	A
NC_040261.1	22170120	C	G
NC_040261.1	22211072	C	T
NC_040261.1	22225573	T	G
NC_040261.1	22285711	T	G
NC_040261.1	22327942	G	A
NC_040261.1	22359919	G	A
NC_040261.1	22378506	A	G
NC_040261.1	22435936	C	T
NC_040261.1	22492634	T	C
NC_040261.1	22548573	G	A

NC_040261.1	22607572	G	A
NC_040261.1	22665554	A	G
NC_040261.1	22705119	A	G
NC_040261.1	22735592	A	G
NC_040261.1	22750771	G	A
NC_040261.1	22800462	G	A
NC_040261.1	22804450	T	C
NC_040261.1	22865319	G	A
NC_040261.1	22914830	A	G
NC_040261.1	22971114	C	G
NC_040261.1	23014979	T	C
NC_040261.1	23035813	G	A
NC_040261.1	23067582	T	C
NC_040261.1	23109143	C	T
NC_040261.1	23123459	T	C
NC_040261.1	23187306	T	C
NC_040261.1	23239043	A	G
NC_040261.1	23297433	C	T
NC_040261.1	23347489	C	T
NC_040261.1	23388900	A	G
NC_040261.1	23425237	C	G
NC_040261.1	23439907	G	A
NC_040261.1	23465386	C	T
NC_040261.1	23495638	G	A
NC_040261.1	23553648	T	C
NC_040261.1	23603241	T	C
NC_040261.1	23631842	A	T
NC_040261.1	23680743	A	T
NC_040261.1	23736163	G	A
NC_040261.1	23790205	G	C
NC_040261.1	23836628	T	A
NC_040261.1	23895978	A	G
NC_040261.1	23944519	C	G
NC_040261.1	23948646	T	G
NC_040261.1	24004885	G	T
NC_040261.1	24037207	T	G
NC_040261.1	24082335	T	C
NC_040261.1	24188413	G	A
NC_040261.1	24238545	T	C
NC_040261.1	24296657	T	C
NC_040261.1	24339399	A	G
NC_040261.1	24392911	A	G
NC_040261.1	24447337	C	G
NC_040261.1	24497393	A	G
NC_040261.1	24545529	T	C
NC_040261.1	24557356	G	A
NC_040261.1	24599634	A	G

NC_040261.1	24617043	G	T
NC_040261.1	24672938	C	G
NC_040261.1	24730708	T	C
NC_040261.1	24785090	A	C
NC_040261.1	24828085	T	C
NC_040261.1	24843938	C	T
NC_040261.1	24894540	C	T
NC_040261.1	24954345	A	G
NC_040261.1	25010343	T	G
NC_040261.1	25057130	G	A
NC_040261.1	25114610	A	G
NC_040261.1	25143772	G	A
NC_040261.1	25165460	G	A
NC_040261.1	25211330	G	A
NC_040261.1	25261709	C	T
NC_040261.1	25317558	G	T
NC_040261.1	25385370	C	T
NC_040261.1	25439621	G	T
NC_040261.1	25475492	T	G
NC_040261.1	25501523	T	C
NC_040261.1	25549130	C	A
NC_040261.1	25556947	A	T
NC_040261.1	25591648	G	A
NC_040261.1	25619168	A	G
NC_040261.1	25638381	G	T
NC_040261.1	25688004	T	C
NC_040261.1	25713428	C	T
NC_040261.1	25765826	T	C
NC_040261.1	25796126	G	A
NC_040261.1	25830099	T	C
NC_040261.1	25833672	G	A
NC_040261.1	25868540	G	T
NC_040261.1	25894927	C	T
NC_040261.1	25915174	G	A
NC_040261.1	25983475	A	G
NC_040261.1	26028571	A	C
NC_040261.1	26064210	T	C
NC_040261.1	26090698	T	G
NC_040261.1	26124955	T	C
NC_040261.1	26147512	G	A
NC_040261.1	26205817	G	A
NC_040261.1	26243300	A	G
NC_040261.1	26265110	C	T
NC_040261.1	26302248	T	A
NC_040261.1	26330253	A	G
NC_040261.1	26369328	G	T
NC_040261.1	26506369	T	C

NC_040261.1	26547677	A	G
NC_040261.1	26612910	C	T
NC_040261.1	26645553	A	G
NC_040261.1	26676164	G	A
NC_040261.1	26681385	G	A
NC_040261.1	26713477	C	T
NC_040261.1	26734749	G	C
NC_040261.1	26789103	C	T
NC_040261.1	26822231	G	C
NC_040261.1	26851676	T	C
NC_040261.1	26898872	T	C
NC_040261.1	26944660	A	G
NC_040261.1	26975234	C	T
NC_040261.1	26995493	A	G
NC_040261.1	27024364	T	C
NC_040261.1	27134964	A	G
NC_040261.1	27206891	G	C
NC_040261.1	27244770	G	C
NC_040261.1	27284695	A	G
NC_040261.1	27329853	T	C
NC_040261.1	27375829	T	C
NC_040261.1	27383244	T	C
NC_040261.1	27433472	C	T
NC_040261.1	27463559	T	C
NC_040261.1	27486583	C	T
NC_040261.1	27545014	G	A
NC_040261.1	27602096	C	G
NC_040261.1	27637091	G	T
NC_040261.1	27646348	C	T
NC_040261.1	27680369	T	C
NC_040261.1	27691360	A	G
NC_040261.1	27754335	A	G
NC_040261.1	27778698	T	C
NC_040261.1	27826599	T	G
NC_040261.1	27907474	C	T
NC_040261.1	27936792	T	C
NC_040261.1	27955306	C	G
NC_040261.1	28008916	G	A
NC_040261.1	28047007	C	T
NC_040261.1	28078520	C	T
NC_040261.1	28087886	G	A
NC_040261.1	28162467	G	T
NC_040261.1	28164580	C	T
NC_040261.1	28223309	G	A
NC_040261.1	28277215	C	T
NC_040261.1	28318185	A	C
NC_040261.1	28356714	G	A

NC_040261.1	28405290	A	C
NC_040261.1	28460522	G	A
NC_040261.1	28506109	T	C
NC_040261.1	28533087	T	C
NC_040261.1	28557981	T	C
NC_040261.1	28609480	C	T
NC_040261.1	28645756	C	T
NC_040261.1	28692926	A	G
NC_040261.1	28699396	A	C
NC_040261.1	28745344	A	G
NC_040261.1	28801757	A	C
NC_040261.1	28853620	G	C
NC_040261.1	28910116	G	T
NC_040261.1	28952764	G	A
NC_040261.1	29005162	T	C
NC_040261.1	29063108	A	G
NC_040261.1	29118738	C	T
NC_040261.1	29169851	T	C
NC_040261.1	29215821	A	C
NC_040261.1	29252597	G	A
NC_040261.1	29355784	A	G
NC_040261.1	29410163	G	A
NC_040261.1	29465052	T	C
NC_040261.1	29511053	C	G
NC_040261.1	29544498	T	C
NC_040261.1	29556399	A	G
NC_040261.1	29592657	C	A
NC_040261.1	29617612	T	C
NC_040261.1	29659568	A	G
NC_040261.1	29705717	C	G
NC_040261.1	29739395	C	T
NC_040261.1	29799115	C	T
NC_040261.1	29836165	A	G
NC_040261.1	29844074	G	T
NC_040261.1	29872820	A	C
NC_040261.1	29898714	A	G
NC_040261.1	29931325	G	A
NC_040261.1	29951201	T	C
NC_040261.1	30003646	C	T
NC_040261.1	30030228	T	A
NC_040261.1	30095720	C	T
NC_040261.1	30143731	G	A
NC_040261.1	30182683	G	T
NC_040261.1	30206703	C	G
NC_040261.1	30253122	C	T
NC_040261.1	30287041	T	C
NC_040261.1	30337691	G	A

NC_040261.1	30379508	A	G
NC_040261.1	30410086	T	A
NC_040261.1	30435973	G	A
NC_040261.1	30465873	A	G
NC_040261.1	30575427	C	G
NC_040261.1	30609828	T	C
NC_040261.1	30636114	T	G
NC_040261.1	30664997	T	C
NC_040261.1	30715930	C	T
NC_040261.1	30775441	T	C
NC_040261.1	30822870	A	T
NC_040261.1	30839798	C	T
NC_040261.1	30925559	C	G
NC_040261.1	30951595	C	T
NC_040261.1	30986479	T	G
NC_040261.1	31020176	T	C
NC_040261.1	31059107	G	A
NC_040261.1	31111874	C	G
NC_040261.1	31138487	C	T
NC_040261.1	31159315	C	A
NC_040261.1	31187136	C	G
NC_040261.1	31333019	G	A
NC_040261.1	31374295	A	C
NC_040261.1	31404959	G	C
NC_040261.1	31429956	T	A
NC_040261.1	31463940	C	G
NC_040261.1	31484271	A	G
NC_040261.1	31517480	G	C
NC_040261.1	31561151	C	G
NC_040261.1	31582289	T	C
NC_040261.1	31625049	C	A
NC_040261.1	31645746	C	G
NC_040261.1	31799009	A	T
NC_040261.1	31852299	A	G
NC_040261.1	31910144	T	G
NC_040261.1	31941835	T	C
NC_040261.1	31982839	G	A
NC_040261.1	32001652	G	A
NC_040261.1	32053528	G	A
NC_040261.1	32100936	G	A
NC_040261.1	32149845	T	C
NC_040261.1	32166549	A	G
NC_040261.1	32208227	C	T
NC_040261.1	32236441	A	G
NC_040261.1	32273090	A	G
NC_040261.1	32287361	G	A
NC_040261.1	32325408	G	A

NC_040261.1	32363410	G	T
NC_040261.1	32407929	G	A
NC_040261.1	32464670	T	C
NC_040261.1	32489730	A	G
NC_040261.1	32562980	G	A
NC_040261.1	32617350	T	C
NC_040261.1	32652643	G	A
NC_040261.1	32702419	T	C
NC_040261.1	32746084	A	T
NC_040261.1	32780890	G	C
NC_040261.1	32805513	T	C
NC_040261.1	32821549	A	G
NC_040261.1	32865815	G	A
NC_040261.1	32897160	T	C
NC_040261.1	33007205	G	C
NC_040261.1	33049293	T	C
NC_040261.1	33088056	T	C
NC_040261.1	33127145	G	A
NC_040261.1	33175388	A	G
NC_040261.1	33215225	C	A
NC_040261.1	33247835	G	A
NC_040261.1	33261223	C	T
NC_040261.1	33307427	C	G
NC_040261.1	33338401	T	C
NC_040261.1	33399609	A	G
NC_040261.1	33468042	T	C
NC_040261.1	33509089	T	G
NC_040261.1	33529035	T	C
NC_040261.1	33588179	A	T
NC_040261.1	33650440	G	A
NC_040261.1	33687337	C	T
NC_040261.1	33693866	T	C
NC_040261.1	33749652	A	G
NC_040261.1	33806807	T	C
NC_040261.1	33858227	A	G
NC_040261.1	33889151	T	C
NC_040261.1	33906324	A	C
NC_040261.1	33948457	A	G
NC_040261.1	34020917	T	C
NC_040261.1	34072807	G	A
NC_040261.1	34129554	G	T
NC_040261.1	34184716	G	A
NC_040261.1	34226506	T	C
NC_040261.1	34285146	C	A
NC_040261.1	34304252	G	C
NC_040261.1	34386340	G	A
NC_040261.1	34428253	C	G

NC_040261.1	34527232	T	C
NC_040261.1	34589335	G	C
NC_040261.1	34641723	T	C
NC_040261.1	34699949	A	T
NC_040261.1	34738432	C	T
NC_040261.1	34780159	A	G
NC_040261.1	34801615	G	A
NC_040261.1	34845759	C	T
NC_040261.1	34892375	C	T
NC_040261.1	34932058	C	T
NC_040261.1	34940461	T	C
NC_040261.1	34979355	A	G
NC_040261.1	34997047	T	C
NC_040261.1	35054026	C	G
NC_040261.1	35107825	T	G
NC_040261.1	35155794	T	C
NC_040261.1	35206553	G	A
NC_040261.1	35279353	C	T
NC_040261.1	35318458	A	G
NC_040261.1	35352474	G	A
NC_040261.1	35370286	T	C
NC_040261.1	35423862	C	G
NC_040261.1	35466878	A	G
NC_040261.1	35487606	G	T
NC_040261.1	35540406	T	A
NC_040261.1	35583819	T	C
NC_040261.1	35595647	A	G
NC_040261.1	35819234	T	C
NC_040261.1	35852154	C	A
NC_040261.1	35870126	C	T
NC_040261.1	35924935	G	T
NC_040261.1	35952671	G	A
NC_040261.1	35996380	A	G
NC_040261.1	36033081	A	C
NC_040261.1	36053324	T	C
NC_040261.1	36108582	G	A
NC_040261.1	36167442	G	C
NC_040261.1	36220458	G	A
NC_040261.1	36275325	A	G
NC_040261.1	36319137	G	A
NC_040261.1	36384814	A	G
NC_040261.1	36473400	A	G
NC_040261.1	36501365	T	C
NC_040261.1	36546748	G	C
NC_040261.1	36570806	C	T
NC_040261.1	36618675	A	G
NC_040261.1	36641643	A	C

NC_040261.1	36677380	A	G
NC_040261.1	36735697	A	G
NC_040261.1	36796929	C	G
NC_040261.1	36845909	C	A
NC_040261.1	36878234	A	G
NC_040261.1	36888753	G	A
NC_040261.1	36931961	T	G
NC_040261.1	36967919	A	C
NC_040261.1	36986122	A	G
NC_040261.1	37041372	A	G
NC_040261.1	37100566	G	T
NC_040261.1	37152586	T	G
NC_040261.1	37198176	G	C
NC_040261.1	37263386	T	C
NC_040261.1	37314226	T	C
NC_040261.1	37366778	C	G
NC_040261.1	37406027	T	C
NC_040261.1	37453304	C	T
NC_040261.1	37469700	G	A
NC_040261.1	37533672	G	T
NC_040261.1	37566639	C	T
NC_040261.1	37592186	A	G
NC_040261.1	37654528	C	A
NC_040261.1	37694889	A	C
NC_040261.1	37726853	A	G
NC_040261.1	37773001	T	A
NC_040261.1	37789821	T	C
NC_040261.1	37822225	T	C
NC_040261.1	37868065	T	A
NC_040261.1	37924364	G	A
NC_040261.1	37973450	G	A
NC_040261.1	38028777	C	G
NC_040261.1	38069709	A	T
NC_040261.1	38116614	T	C
NC_040261.1	38189440	T	C
NC_040261.1	38227548	T	C
NC_040261.1	38240828	A	G
NC_040261.1	38285230	C	G
NC_040261.1	38339687	G	A
NC_040261.1	38409905	G	A
NC_040261.1	38488604	T	C
NC_040261.1	38620777	A	G
NC_040261.1	38636803	C	T
NC_040261.1	38666709	C	T
NC_040261.1	38694857	C	T
NC_040261.1	38740625	T	C
NC_040261.1	38776489	G	A

NC_040261.1	38962575	A	G
NC_040261.1	39039007	T	A
NC_040261.1	39110050	T	A
NC_040261.1	39130753	T	A
NC_040261.1	39230917	G	T
NC_040261.1	39269168	T	C
NC_040261.1	39302159	T	A
NC_040261.1	39326585	A	G
NC_040261.1	39483272	G	C
NC_040261.1	39553054	C	T
NC_040261.1	39582920	A	G
NC_040261.1	39741947	G	A
NC_040261.1	39784825	T	A
NC_040261.1	39831066	G	A
NC_040261.1	39856742	A	T
NC_040261.1	39924045	T	C
NC_040261.1	40041356	T	C
NC_040261.1	40354783	T	C
NC_040261.1	41001305	T	C
NC_040261.1	41291413	A	C
NC_040261.1	41409385	G	A
NC_040261.1	41818814	T	A
NC_040261.1	41914057	G	A
NC_040261.1	41952617	G	A
NC_040261.1	41973974	C	T
NC_040261.1	42025501	T	C
NC_040261.1	42103162	G	A
NC_040261.1	42188105	A	G
NC_040261.1	42308813	T	A
NC_040261.1	42354038	C	T
NC_040261.1	42403946	C	T
NC_040261.1	42491524	A	G
NC_040261.1	42559789	C	G
NC_040261.1	42601410	A	G
NC_040261.1	42679841	G	A
NC_040261.1	42736738	C	T
NC_040261.1	42861529	C	T
NC_040261.1	42889905	C	T
NC_040261.1	42931271	T	C
NC_040261.1	42988236	A	G
NC_040261.1	43083044	A	G
NC_040261.1	43228860	T	A
NC_040261.1	43298045	A	G
NC_040261.1	43326454	G	A
NC_040261.1	43403190	C	T
NC_040261.1	43496866	G	T
NC_040261.1	43579020	T	C

NC_040261.1	43641146	T	A
NC_040261.1	43679228	C	T
NC_040261.1	43752310	C	G
NC_040261.1	43773812	T	G
NC_040261.1	43857780	A	G
NC_040261.1	43983412	T	C
NC_040261.1	44066960	C	T
NC_040261.1	44096242	C	T
NC_040261.1	44104777	G	A
NC_040261.1	44146035	C	A
NC_040261.1	44219978	A	T
NC_040261.1	44272047	A	G
NC_040261.1	44333699	T	A
NC_040261.1	44363418	A	T
NC_040261.1	44383930	A	G
NC_040261.1	44461881	G	A
NC_040261.1	44534408	G	A
NC_040261.1	44575393	G	A
NC_040261.1	44634371	A	T
NC_040261.1	44655522	G	A
NC_040261.1	44734411	C	T
NC_040261.1	44770618	A	C
NC_040261.1	44837611	T	C
NC_040261.1	45210986	A	G
NC_040261.1	45247223	A	T
NC_040261.1	45265240	A	T
NC_040261.1	45312757	A	T
NC_040261.1	45430796	G	T
NC_040261.1	45481613	T	A
NC_040261.1	45543669	C	T
NC_040261.1	45634803	G	A
NC_040261.1	45641757	G	A
NC_040261.1	45762571	C	T
NC_040261.1	45802934	A	G
NC_040261.1	45816636	C	G
NC_040261.1	45865915	A	G
NC_040261.1	46006873	G	A
NC_040261.1	46089410	G	A
NC_040261.1	46148288	C	T
NC_040261.1	46178903	G	T
NC_040261.1	46205971	G	T
NC_040261.1	46269849	T	C
NC_040261.1	46296619	T	C
NC_040261.1	46420804	C	T
NC_040261.1	46529435	G	A
NC_040261.1	46654168	T	C
NC_040261.1	46676497	C	T

NC_040261.1	46758006	C	G
NC_040261.1	46794626	T	C
NC_040261.1	47075335	A	G
NC_040261.1	47123574	G	A
NC_040261.1	47162353	A	G
NC_040261.1	47382792	T	C
NC_040261.1	47534884	0	C
NC_040261.1	47646207	A	G
NC_040261.1	48515898	A	C
NC_040261.1	48723552	G	A
NC_040261.1	48842591	A	C
NC_040261.1	48947027	G	A
NC_040261.1	49290114	A	G
NC_040261.1	49677192	A	G
NC_040261.1	49843675	C	T
NC_040261.1	50024807	0	G
NC_040261.1	50105916	G	C
NC_040261.1	50536670	A	G
NC_040261.1	50890265	G	A
NC_040261.1	50996334	C	T
NC_040261.1	51314536	G	C
NC_040261.1	51500332	T	G
NC_040261.1	51976111	C	T
NC_040261.1	52237803	T	C
NC_040261.1	52501103	T	C
NC_040261.1	52620912	G	A
NC_040261.1	52790658	G	A
NC_040261.1	52976531	T	C
NC_040261.1	53091167	G	T
NC_040261.1	53566111	T	C
NC_040261.1	53998653	A	G
NC_040261.1	54832792	T	C
NC_040261.1	54966452	A	T
NC_040261.1	55184272	G	A
NC_040261.1	55433423	G	A
NC_040261.1	56212855	A	T
NC_040261.1	56311952	A	G
NC_040261.1	56692445	A	G
NC_040261.1	57026999	T	C
NC_040261.1	57154699	T	C
NC_040261.1	57458225	C	G
NC_040261.1	57531007	A	G
NC_040261.1	57633824	A	G
NC_040261.1	57964080	T	A
NC_040261.1	58159953	T	C
NC_040261.1	58258541	G	T
NC_040261.1	58598222	T	C

NC_040261.1	58669936	C		A
NC_040261.1	59107186	C		T
NC_040261.1	59238265	C		T
NC_040261.1	59646669	A		G
NC_040261.1	59739942	A		G
NC_040261.1	59946992	G		A
NC_040261.1	60792735	T		C
NC_040261.1	60862422	G		A
NC_040261.1	61121068	T		C
NC_040261.1	61184609	G		T
NC_040261.1	61258721	C		A
NC_040261.1	61463738	G		C
NC_040261.1	61520952	C		T
NC_040261.1	61555935	C		T
NC_040261.1	61607663	T		C
NC_040261.1	61830025	A		G
NC_040261.1	62069519	A		G
NC_040261.1	62414382	T		C
NC_040261.1	62476877	G		A
NC_040261.1	62519037	A		G
NC_040261.1	62540479	G		A
NC_040261.1	62598953	C		T
NC_040261.1	62679047	G		T
NC_040261.1	62743269	C		T
NC_040261.1	62801909	C		T
NC_040261.1	62881569	C		T
NC_040261.1	62916563	G		C
NC_040261.1	63016661	T		C
NC_040261.1	63083227	G		C
NC_040261.1	63187991	T		A
NC_040261.1	63269303	T		A
NC_040261.1	63433450	C		G
NC_040261.1	63500517	A		C
NC_040261.1	63559545	T		C
NC_040261.1	63645932	T		C
NC_040261.1	64205265	A		G
NC_040261.1	64310182	T		G
NC_040261.1	64419803		0	C
NC_040261.1	64489873	T		C
NC_040261.1	64556725	A		G
NC_040261.1	64612129	T		C
NC_040261.1	64797320	A		G
NC_040261.1	65166001	G		A
NC_040261.1	65234771	G		A
NC_040261.1	65416114	C		T
NC_040261.1	65563723	C		T
NC_040261.1	65987834	C		A

NC_040261.1	66240541	A	T
NC_040261.1	66465437	C	T
NC_040261.1	66593117	T	G
NC_040261.1	67345017	C	T
NC_040261.1	67450086	G	T
NC_040261.1	67964066	A	G
NC_040261.1	68175032	A	G
NC_040261.1	68232173	A	G
NC_040261.1	68332963	A	T
NC_040261.1	68406798	C	T
NC_040261.1	68445966	T	C
NC_040261.1	68496903	C	A
NC_040261.1	68611373	G	A
NC_040261.1	68685335	T	C
NC_040261.1	68785024	A	C
NC_040261.1	68843997	A	G
NC_040261.1	68892486	A	C
NC_040261.1	68920491	T	C
NC_040261.1	69048262	T	C
NC_040261.1	69376662	T	G
NC_040261.1	69576153	G	A
NC_040261.1	69648670	C	T
NC_040261.1	69715072	T	C
NC_040261.1	69784028	G	A
NC_040261.1	69845137	G	A
NC_040261.1	70376715	G	A
NC_040261.1	70499406	C	T
NC_040261.1	70566109	T	C
NC_040261.1	70766635	C	T
NC_040261.1	71083437	G	A
NC_040261.1	71852796	T	G
NC_040261.1	72093860	T	C
NC_040261.1	72238607	T	C
NC_040261.1	72503802	C	T
NC_040261.1	72624360	T	C
NC_040261.1	72661157	G	A
NC_040261.1	72697562	G	C
NC_040261.1	72905762	G	A
NC_040261.1	73284588	A	T
NC_040261.1	73475494	T	C
NC_040261.1	73547430	T	C
NC_040261.1	73645847	A	G
NC_040261.1	73687250	G	T
NC_040261.1	73714758	T	C
NC_040261.1	73764619	T	C
NC_040261.1	74187607	C	T
NC_040261.1	74309083	A	G

NC_040261.1	74526425	T	C
NC_040261.1	74604885	T	G
NC_040261.1	74660026	C	G
NC_040261.1	74690057	T	A
NC_040261.1	74777666	T	C
NC_040261.1	75386314	A	C
NC_040261.1	75541126	G	A
NC_040261.1	75660313	G	A
NC_040261.1	76052213	T	C
NC_040261.1	76158870	G	A
NC_040261.1	76290910	T	C
NC_040261.1	76407879	A	C
NC_040261.1	76553909	A	C
NC_040261.1	76898730	T	C
NC_040261.1	77053062	C	T
NC_040261.1	77395677	T	C
NC_040261.1	77566538	G	T
NC_040261.1	77925802	G	A
NC_040261.1	77979832	C	T
NC_040261.1	78049684	G	A
NC_040261.1	78116706	A	C
NC_040261.1	78164025	T	C
NC_040261.1	78200310	C	A
NC_040261.1	78251172	G	A
NC_040261.1	78309054	A	G
NC_040261.1	78363680	T	A
NC_040261.1	78385247	A	T
NC_040261.1	79042849	C	T
NC_040261.1	79223818	A	C
NC_040261.1	80403165	A	G
NC_040261.1	80433450	C	T
NC_040261.1	80482394	A	G
NC_040261.1	81170737	0	T
NC_040261.1	81317669	T	G
NC_040261.1	81587900	T	C
NC_040261.1	81933652	A	G
NC_040261.1	81980705	A	G
NC_040261.1	82016633	T	C
NC_040261.1	82113514	T	C
NC_040261.1	82144799	A	G
NC_040261.1	82165166	C	T
NC_040261.1	82196971	T	C
NC_040261.1	82227494	A	G
NC_040261.1	82241921	C	T
NC_040261.1	82277274	A	G
NC_040261.1	82311334	G	T
NC_040261.1	82494572	T	C

NC_040261.1	82520418	A	G
NC_040261.1	82611136	G	A
NC_040261.1	82739723	A	C
NC_040261.1	82799856	A	G
NC_040261.1	82813414	C	T
NC_040261.1	82887495	G	A
NC_040261.1	82966219	A	G
NC_040261.1	82994345	A	G
NC_040261.1	83031008	G	A
NC_040261.1	83059572	C	T
NC_040261.1	83109660	A	G
NC_040261.1	83148279	A	C
NC_040261.1	83190731	T	C
NC_040261.1	83287128	G	A
NC_040261.1	83339539	T	C
NC_040261.1	83393943	T	C
NC_040261.1	83440243	T	C
NC_040261.1	83452561	C	T
NC_040261.1	83507196	G	T
NC_040261.1	83534707	G	A
NC_040261.1	83560540	T	C
NC_040261.1	83576484	G	A
NC_040261.1	83661940	T	C
NC_040261.1	83688233	T	C
NC_040261.1	83813354	C	T
NC_040261.1	83867465	A	G
NC_040261.1	83902440	C	T
NC_040261.1	83918593	G	A
NC_040261.1	83954196	G	A
NC_040261.1	83993695	A	G
NC_040261.1	84040093	C	A
NC_040261.1	84090224	T	A
NC_040261.1	84148881	G	T
NC_040261.1	84208376	A	C
NC_040261.1	84260122	G	A
NC_040261.1	84313456	G	A
NC_040261.1	84376661	T	A
NC_040261.1	84407553	A	G
NC_040261.1	84431711	G	A
NC_040261.1	84487458	G	T
NC_040261.1	84542244	T	C
NC_040261.1	84596096	G	C
NC_040261.1	84653202	A	G
NC_040261.1	84711676	T	A
NC_040261.1	84752456	A	G
NC_040261.1	84752800	C	T
NC_040261.1	84797087	T	C

NC_040261.1	84853281	C	T
NC_040261.1	84909866	C	T
NC_040261.1	84965010	G	T
NC_040261.1	85018088	A	T
NC_040261.1	85066590	A	G
NC_040261.1	85115285	T	G
NC_040261.1	85154060	T	G
NC_040261.1	85157992	G	T
NC_040261.1	85201087	T	C
NC_040261.1	85256007	C	A
NC_040261.1	85306746	G	A
NC_040261.1	85366653	C	T
NC_040261.1	85403870	C	T
NC_040261.1	85452587	T	G
NC_040261.1	85455286	T	C
NC_040261.1	85511622	G	A
NC_040261.1	85565698	T	C
NC_040261.1	85624008	T	C
NC_040261.1	85752627	A	C
NC_040261.1	85803761	A	G
NC_040261.1	85849967	A	C
NC_040261.1	85929611	A	G
NC_040261.1	85984459	G	A
NC_040261.1	86038406	A	G
NC_040261.1	86082048	C	T
NC_040261.1	86127082	A	G
NC_040261.1	86185126	A	C
NC_040261.1	86237332	T	C
NC_040261.1	86264552	G	A
NC_040261.1	86283340	A	G
NC_040261.1	86331410	T	C
NC_040261.1	86397548	T	C
NC_040261.1	86440425	T	G
NC_040261.1	86471317	G	A
NC_040261.1	86497272	A	G
NC_040261.1	86529113	C	T
NC_040261.1	86578204	T	C
NC_040261.1	86615719	G	A
NC_040261.1	86646907	C	T
NC_040261.1	86698001	T	C
NC_040261.1	86729975	C	G
NC_040261.1	86755595	G	A
NC_040261.1	86813759	T	A
NC_040261.1	86907226	C	G
NC_040261.1	86917770	G	A
NC_040261.1	86979980	C	A
NC_040261.1	87034293	G	C

NC_040261.1	87084465	A	C
NC_040261.1	87112959	A	G
NC_040261.1	87138549	A	G
NC_040261.1	87148742	A	G
NC_040261.1	87247406	C	T
NC_040261.1	87276952	A	G
NC_040261.1	87303816	C	G
NC_040261.1	87359341	A	T
NC_040261.1	87406827	A	G
NC_040261.1	87437936	T	C
NC_040261.1	87446287	T	A
NC_040261.1	87487724	C	T
NC_040261.1	87508375	T	C
NC_040261.1	87549888	A	G
NC_040261.1	87583334	G	C
NC_040261.1	87605439	C	T
NC_040261.1	87642272	A	G
NC_040261.1	87667359	A	C
NC_040261.1	87725312	A	G
NC_040261.1	87793973	A	G
NC_040261.1	87847697	A	G
NC_040261.1	87893906	A	T
NC_040261.1	87937900	T	C
NC_040261.1	87991674	T	C
NC_040261.1	88020982	A	G
NC_040261.1	88038047	C	A
NC_040261.1	88104990	G	A
NC_040261.1	88165264	T	C
NC_040261.1	88255841	G	A
NC_040261.1	88301940	C	G
NC_040261.1	88312836	C	T
NC_040261.1	88374779	G	A
NC_040261.1	88419396	T	C
NC_040261.1	88473917	A	G
NC_040261.1	88526842	A	C
NC_040261.1	88581807	T	C
NC_040261.1	88633668	T	G
NC_040261.1	88667275	G	C
NC_040261.1	88707102	T	C
NC_040261.1	88717125	G	A
NC_040261.1	88752191	A	G
NC_040261.1	88755723	A	G
NC_040261.1	88779107	G	A
NC_040261.1	88822418	C	T
NC_040261.1	88830015	T	C
NC_040261.1	88832487	T	C
NC_040261.1	88835653	C	T

NC_040261.1	88836785	C	T
NC_040261.1	88838090	C	T
NC_040261.1	88840393	C	T
NC_040261.1	88843884	C	T
NC_040261.1	88858959	G	A
NC_040261.1	88877274	A	G
NC_040261.1	88880886	T	C
NC_040261.1	88885233	T	C
NC_040261.1	88940280	A	G
NC_040261.1	88956213	G	A
NC_040261.1	88972765	T	C
NC_040261.1	88977616	A	G
NC_040261.1	89029813	C	T
NC_040261.1	89080402	C	T
NC_040261.1	89134475	A	G
NC_040261.1	89174600	C	G
NC_040261.1	89183442	G	A
NC_040261.1	89236621	G	A
NC_040261.1	89293903	T	C
NC_040261.1	89341614	A	G
NC_040261.1	89382723	A	G
NC_040261.1	89392514	T	C
NC_040261.1	89451102	A	G
NC_040261.1	89484036	T	C
NC_040261.1	89512577	A	G
NC_040261.1	89568045	G	A
NC_040261.1	89612338	C	T
NC_040261.1	89650868	T	C
NC_040261.1	89669407	A	G
NC_040261.1	89726830	C	T
NC_040261.1	89780969	T	C
NC_040261.1	89835115	A	C
NC_040261.1	89869244	C	A
NC_040261.1	89903700	T	C
NC_040261.1	89921606	T	C
NC_040261.1	90033797	G	T
NC_040261.1	90077047	T	C
NC_040261.1	90145315	A	C
NC_040261.1	90208648	A	G
NC_040261.1	90255537	T	C
NC_040261.1	90315216	C	T
NC_040261.1	90358147	G	T
NC_040261.1	90393885	C	A
NC_040261.1	90423861	C	T
NC_040261.1	90445680	C	T
NC_040261.1	90511280	A	G
NC_040261.1	90560786	C	A

NC_040261.1	90602230	G	C
NC_040261.1	90612304	A	T
NC_040261.1	90697244	C	T
NC_040261.1	90725723	G	A
NC_040261.1	90751270	T	A
NC_040261.1	90779287	G	A
NC_040261.1	90946698	C	G
NC_040261.1	91000798	T	C
NC_040261.1	91039583	C	T
NC_040261.1	91054890	A	G
NC_040261.1	91106555	A	G
NC_040261.1	91149416	A	G
NC_040261.1	91165668	G	A
NC_040261.1	91217917	T	A
NC_040261.1	91263141	A	G
NC_040261.1	91314549	G	A
NC_040261.1	91366638	G	A
NC_040261.1	91415944	T	C
NC_040261.1	91491424	T	C
NC_040261.1	91521692	A	G
NC_040261.1	91545891	C	T
NC_040261.1	91570992	C	T
NC_040261.1	91599388	A	G
NC_040261.1	91656847	T	C
NC_040261.1	91721120	C	A
NC_040261.1	91769676	T	C
NC_040261.1	91777562	T	C
NC_040261.1	91835384	A	G
NC_040261.1	91891470	T	C
NC_040261.1	91929559	G	T
NC_040261.1	91946358	C	T
NC_040261.1	92000423	C	A
NC_040261.1	92044679	T	C
NC_040261.1	92048000	G	C
NC_040261.1	92109597	A	T
NC_040261.1	92173425	A	G
NC_040261.1	92207624	A	G
NC_040261.1	92228672	A	G
NC_040261.1	92281469	G	A
NC_040261.1	92330033	G	A
NC_040261.1	92332044	T	C
NC_040261.1	92372391	A	G
NC_040261.1	92411537	A	G
NC_040261.1	92437235	A	G
NC_040261.1	92464625	G	A
NC_040261.1	92532970	A	G
NC_040261.1	92583134	T	G

NC_040261.1	92635431	T	C
NC_040261.1	92691640	C	T
NC_040261.1	92747668	A	T
NC_040261.1	92783180	T	C
NC_040261.1	92803947	C	G
NC_040261.1	92845470	T	C
NC_040261.1	92888216	C	A
NC_040261.1	92942103	T	C
NC_040261.1	92974302	A	C
NC_040261.1	93018260	T	C
NC_040261.1	93058518	T	C
NC_040261.1	93101197	A	C
NC_040261.1	93118024	C	A
NC_040261.1	93175304	G	A
NC_040261.1	93228812	T	C
NC_040261.1	93267668	T	G
NC_040261.1	93284911	C	T
NC_040261.1	93340825	A	G
NC_040261.1	93386795	C	A
NC_040261.1	93443217	A	G
NC_040261.1	93499385	G	C
NC_040261.1	93556779	A	G
NC_040261.1	93622954	T	C
NC_040261.1	93684196	C	T
NC_040261.1	93703650	C	G
NC_040261.1	93704504	C	T
NC_040261.1	93704958	G	A
NC_040261.1	93706272	T	C
NC_040261.1	93708020	C	T
NC_040261.1	93760821	T	C
NC_040261.1	93812889	G	T
NC_040261.1	93851670	G	T
NC_040261.1	93887708	G	A
NC_040261.1	93904711	A	G
NC_040261.1	93955580	A	G
NC_040261.1	94027838	C	T
NC_040261.1	94069820	T	C
NC_040261.1	94101048	C	T
NC_040261.1	94129138	A	G
NC_040261.1	94159275	T	C
NC_040261.1	94203462	G	A
NC_040261.1	94215220	G	A
NC_040261.1	94267416	T	C
NC_040261.1	94323190	A	G
NC_040261.1	94332644	T	G
NC_040261.1	94393086	T	C
NC_040261.1	94412193	G	T

NC_040261.1	94457967	T	C
NC_040261.1	94470862	T	G
NC_040261.1	94530392	T	C
NC_040261.1	94584543	C	G
NC_040261.1	94637486	C	A
NC_040261.1	94682025	C	T
NC_040261.1	94711916	C	G
NC_040261.1	94739233	G	A
NC_040261.1	94739492	A	G
NC_040261.1	94740289	A	G
NC_040261.1	94740557	A	G
NC_040261.1	94741246	G	T
NC_040261.1	94799896	T	A
NC_040261.1	94852842	A	G
NC_040261.1	94907519	A	G
NC_040261.1	94949775	A	T
NC_040261.1	94950042	C	A
NC_040261.1	95004601	A	G
NC_040261.1	95060994	C	T
NC_040261.1	95116177	A	G
NC_040261.1	95172081	T	C
NC_040261.1	95222527	A	G
NC_040261.1	95257035	T	C
NC_040261.1	95291577	C	T
NC_040261.1	95343612	T	C
NC_040261.1	95398986	G	C
NC_040261.1	95409996	T	C
NC_040261.1	95463704	A	G
NC_040261.1	95493897	G	A
NC_040261.1	95524635	C	T
NC_040261.1	95567648	G	A
NC_040261.1	95659121	A	G
NC_040261.1	95714122	T	C
NC_040261.1	95740622	G	A
NC_040261.1	95772773	A	G
NC_040261.1	95817304	G	A
NC_040261.1	95872928	T	C
NC_040261.1	95906983	C	T
NC_040261.1	95920881	A	G
NC_040261.1	95922297	T	C
NC_040261.1	95966507	A	G
NC_040261.1	95998049	G	A
NC_040261.1	96040053	A	C
NC_040261.1	96058975	A	G
NC_040261.1	96114738	C	T
NC_040261.1	96168787	T	A
NC_040261.1	96226406	A	G

NC_040261.1	96268076	G	A
NC_040261.1	96303575	A	G
NC_040261.1	96303728	C	A
NC_040261.1	96322225	T	C
NC_040261.1	96355959	C	A
NC_040261.1	96379054	A	G
NC_040261.1	96428660	G	T
NC_040261.1	96464272	A	G
NC_040261.1	96495412	A	G
NC_040261.1	96496053	T	C
NC_040261.1	96497416	C	T
NC_040261.1	96542977	C	T
NC_040261.1	96574732	A	G
NC_040261.1	96585596	T	C
NC_040261.1	96612925	A	G
NC_040261.1	96618640	T	C
NC_040261.1	96621421	A	G
NC_040261.1	96637606	A	G
NC_040261.1	96673075	G	C
NC_040261.1	96735045	G	A
NC_040261.1	96736404	G	T
NC_040261.1	96738770	T	C
NC_040261.1	96739595	A	G
NC_040261.1	96793206	A	G
NC_040261.1	96830555	A	G
NC_040261.1	96839918	C	T
NC_040261.1	96842744	G	C
NC_040261.1	96855494	A	G
NC_040261.1	96908522	T	C
NC_040261.1	96962730	G	C
NC_040261.1	96993005	G	C
NC_040261.1	97004676	G	A
NC_040261.1	97059715	A	G
NC_040261.1	97114081	T	G
NC_040261.1	97154725	T	C
NC_040261.1	97178497	T	C
NC_040261.1	97182630	C	T
NC_040261.1	97195377	G	T
NC_040261.1	97196202	T	A
NC_040261.1	97196396	G	A
NC_040261.1	97196712	G	A
NC_040261.1	97196939	G	A
NC_040262.1	10239	G	C
NC_040262.1	60972	C	T
NC_040262.1	114696	G	A
NC_040262.1	171425	A	G
NC_040262.1	211299	T	C

NC_040262.1	232356	G	A
NC_040262.1	233591	G	A
NC_040262.1	233864	C	G
NC_040262.1	235372	G	C
NC_040262.1	235648	G	A
NC_040262.1	238050	T	A
NC_040262.1	246502	A	G
NC_040262.1	247156	A	G
NC_040262.1	255505	C	T
NC_040262.1	255757	T	G
NC_040262.1	267083	A	G
NC_040262.1	272322	A	G
NC_040262.1	327464	C	T
NC_040262.1	368662	A	G
NC_040262.1	416135	C	T
NC_040262.1	416550	T	C
NC_040262.1	416711	T	C
NC_040262.1	471352	T	C
NC_040262.1	527434	C	T
NC_040262.1	579091	G	A
NC_040262.1	634410	C	T
NC_040262.1	680925	A	G
NC_040262.1	727058	T	C
NC_040262.1	781887	G	A
NC_040262.1	836608	A	G
NC_040262.1	875619	A	C
NC_040262.1	909286	A	G
NC_040262.1	965139	T	C
NC_040262.1	1017003	C	T
NC_040262.1	1068703	T	C
NC_040262.1	1110406	A	G
NC_040262.1	1110706	T	C
NC_040262.1	1165491	T	C
NC_040262.1	1220870	G	C
NC_040262.1	1275657	A	G
NC_040262.1	1331673	G	A
NC_040262.1	1379409	G	A
NC_040262.1	1402057	A	C
NC_040262.1	1427020	T	C
NC_040262.1	1430363	G	A
NC_040262.1	1430690	G	C
NC_040262.1	1431470	T	C
NC_040262.1	1431958	T	C
NC_040262.1	1472206	G	C
NC_040262.1	1480205	G	T
NC_040262.1	1501213	T	C
NC_040262.1	1544110	A	G

NC_040262.1	1596187	T	C
NC_040262.1	1651065	A	G
NC_040262.1	1651232	G	A
NC_040262.1	1651722	G	A
NC_040262.1	1652050	T	C
NC_040262.1	1653343	A	G
NC_040262.1	1653809	T	C
NC_040262.1	1654235	T	G
NC_040262.1	1655801	T	C
NC_040262.1	1656006	T	C
NC_040262.1	1656181	T	C
NC_040262.1	1667755	G	C
NC_040262.1	1713134	A	G
NC_040262.1	1755468	T	C
NC_040262.1	1798987	T	C
NC_040262.1	1826135	G	A
NC_040262.1	1862385	A	C
NC_040262.1	1895522	G	A
NC_040262.1	1951075	A	G
NC_040262.1	1986292	T	C
NC_040262.1	1999997	C	T
NC_040262.1	2000988	C	T
NC_040262.1	2002155	A	G
NC_040262.1	2019958	T	C
NC_040262.1	2051257	T	A
NC_040262.1	2051966	T	C
NC_040262.1	2052180	A	G
NC_040262.1	2111749	A	G
NC_040262.1	2114218	T	C
NC_040262.1	2114927	T	C
NC_040262.1	2168535	G	A
NC_040262.1	2222266	C	G
NC_040262.1	2277192	A	G
NC_040262.1	2329127	A	G
NC_040262.1	2386544	T	G
NC_040262.1	2440570	A	G
NC_040262.1	2495140	A	G
NC_040262.1	2550958	C	T
NC_040262.1	2598389	T	C
NC_040262.1	2650705	G	A
NC_040262.1	2716591	C	T
NC_040262.1	2718369	A	G
NC_040262.1	2718589	C	A
NC_040262.1	2718964	A	G
NC_040262.1	2719136	T	C
NC_040262.1	2719302	T	C
NC_040262.1	2720329	C	G

NC_040262.1	2762095	T	C
NC_040262.1	2817384	A	G
NC_040262.1	2869343	C	T
NC_040262.1	2917500	A	G
NC_040262.1	2978985	T	C
NC_040262.1	3034843	T	C
NC_040262.1	3090200	G	A
NC_040262.1	3160418	A	G
NC_040262.1	3215532	T	C
NC_040262.1	3272459	C	G
NC_040262.1	3327505	A	G
NC_040262.1	3390427	T	A
NC_040262.1	3442887	C	T
NC_040262.1	3499706	T	G
NC_040262.1	3552335	T	C
NC_040262.1	3597260	G	T
NC_040262.1	3648551	T	C
NC_040262.1	3704874	G	A
NC_040262.1	3760275	T	C
NC_040262.1	3811257	G	A
NC_040262.1	3868891	A	G
NC_040262.1	3923329	G	A
NC_040262.1	3972859	G	C
NC_040262.1	4021343	A	G
NC_040262.1	4076699	A	G
NC_040262.1	4132111	T	C
NC_040262.1	4188891	T	C
NC_040262.1	4245265	T	C
NC_040262.1	4300160	C	A
NC_040262.1	4353789	T	C
NC_040262.1	4397735	A	G
NC_040262.1	4431164	G	A
NC_040262.1	4485065	T	C
NC_040262.1	4544398	T	C
NC_040262.1	4595863	T	A
NC_040262.1	4634927	T	C
NC_040262.1	4686372	A	G
NC_040262.1	4738430	A	C
NC_040262.1	4790568	G	T
NC_040262.1	4830600	C	G
NC_040262.1	4885243	A	T
NC_040262.1	4944443	T	C
NC_040262.1	5011007	C	T
NC_040262.1	5075584	C	T
NC_040262.1	5132695	A	G
NC_040262.1	5194756	A	G
NC_040262.1	5250810	A	G

NC_040262.1	5315332	A	G
NC_040262.1	5325219	G	C
NC_040262.1	5368657	A	G
NC_040262.1	5424019	A	G
NC_040262.1	5475821	A	G
NC_040262.1	5539834	T	C
NC_040262.1	5591116	A	C
NC_040262.1	5639323	T	C
NC_040262.1	5706961	T	C
NC_040262.1	5774595	C	T
NC_040262.1	5833200	T	C
NC_040262.1	5892378	T	G
NC_040262.1	5940203	G	A
NC_040262.1	5991827	T	C
NC_040262.1	6047601	A	G
NC_040262.1	6136154	A	G
NC_040262.1	6138466	A	G
NC_040262.1	6203726	A	C
NC_040262.1	6210708	T	C
NC_040262.1	6267576	A	G
NC_040262.1	6307885	C	T
NC_040262.1	6349512	T	C
NC_040262.1	6402095	A	C
NC_040262.1	6447300	T	C
NC_040262.1	6524430	A	G
NC_040262.1	6539475	G	C
NC_040262.1	6578324	G	C
NC_040262.1	6583229	C	T
NC_040262.1	6583690	G	A
NC_040262.1	6584208	C	A
NC_040262.1	6586007	C	A
NC_040262.1	6622647	T	C
NC_040262.1	6676274	A	C
NC_040262.1	6706970	A	G
NC_040262.1	6722437	T	C
NC_040262.1	6726892	A	G
NC_040262.1	6728779	T	C
NC_040262.1	6777015	T	C
NC_040262.1	6787809	C	T
NC_040262.1	6847636	G	C
NC_040262.1	6889632	T	C
NC_040262.1	6902321	G	C
NC_040262.1	6934984	T	C
NC_040262.1	6955050	A	G
NC_040262.1	7024567	G	T
NC_040262.1	7026759	C	G
NC_040262.1	7045963	G	A

NC_040262.1	7048399	T	C
NC_040262.1	7053719	A	G
NC_040262.1	7056956	G	T
NC_040262.1	7112348	T	C
NC_040262.1	7165851	T	A
NC_040262.1	7219722	A	G
NC_040262.1	7220900	T	C
NC_040262.1	7224476	C	T
NC_040262.1	7225308	T	C
NC_040262.1	7238640	C	T
NC_040262.1	7241332	T	C
NC_040262.1	7243332	A	G
NC_040262.1	7268799	T	C
NC_040262.1	7272927	A	G
NC_040262.1	7277255	A	G
NC_040262.1	7301046	G	T
NC_040262.1	7324289	T	G
NC_040262.1	7336360	T	C
NC_040262.1	7341303	T	C
NC_040262.1	7348785	A	G
NC_040262.1	7385780	G	A
NC_040262.1	7442825	C	A
NC_040262.1	7482316	T	C
NC_040262.1	7483825	A	G
NC_040262.1	7494964	A	G
NC_040262.1	7499572	A	G
NC_040262.1	7502800	T	C
NC_040262.1	7537129	T	G
NC_040262.1	7550009	C	T
NC_040262.1	7558303	A	G
NC_040262.1	7571467	G	A
NC_040262.1	7576659	G	A
NC_040262.1	7617195	C	T
NC_040262.1	7657777	A	G
NC_040262.1	7673434	T	C
NC_040262.1	7682321	A	G
NC_040262.1	7718619	G	A
NC_040262.1	7725733	C	T
NC_040262.1	7780083	G	A
NC_040262.1	7826986	A	G
NC_040262.1	7827597	T	C
NC_040262.1	7828255	A	G
NC_040262.1	7847656	C	T
NC_040262.1	7856955	T	C
NC_040262.1	7858184	A	G
NC_040262.1	7927707	C	T
NC_040262.1	7955383	A	G

NC_040262.1	7998526	T	C
NC_040262.1	8044498	A	C
NC_040262.1	8084904	T	G
NC_040262.1	8147188	C	A
NC_040262.1	8206528	T	G
NC_040262.1	8219363	G	C
NC_040262.1	8227845	C	G
NC_040262.1	8280088	C	T
NC_040262.1	8336143	T	C
NC_040262.1	8393291	A	G
NC_040262.1	8447523	A	G
NC_040262.1	8502232	T	C
NC_040262.1	8558735	G	A
NC_040262.1	8614822	A	G
NC_040262.1	8670851	A	G
NC_040262.1	8725624	A	G
NC_040262.1	8784070	C	T
NC_040262.1	8840476	C	T
NC_040262.1	8904673	C	T
NC_040262.1	8959651	A	G
NC_040262.1	9015286	T	G
NC_040262.1	9070815	A	G
NC_040262.1	9132478	G	A
NC_040262.1	9189510	G	A
NC_040262.1	9236570	A	G
NC_040262.1	9244167	A	G
NC_040262.1	9254695	C	G
NC_040262.1	9285774	T	C
NC_040262.1	9286709	T	C
NC_040262.1	9288850	G	A
NC_040262.1	9300980	T	C
NC_040262.1	9303238	G	T
NC_040262.1	9335538	T	A
NC_040262.1	9389271	A	G
NC_040262.1	9435032	T	C
NC_040262.1	9483427	T	C
NC_040262.1	9487039	T	C
NC_040262.1	9507728	G	A
NC_040262.1	9521196	T	C
NC_040262.1	9539627	A	G
NC_040262.1	9543152	G	A
NC_040262.1	9559735	T	C
NC_040262.1	9571515	T	C
NC_040262.1	9627491	T	C
NC_040262.1	9659469	G	C
NC_040262.1	9693943	A	G
NC_040262.1	9725804	C	A

NC_040262.1	9759169	G	A
NC_040262.1	9786341	A	G
NC_040262.1	9786688	T	C
NC_040262.1	9832137	T	C
NC_040262.1	9866705	A	G
NC_040262.1	9903567	A	G
NC_040262.1	9912584	C	T
NC_040262.1	9940837	A	C
NC_040262.1	9942551	G	A
NC_040262.1	9956629	G	C
NC_040262.1	10009518	A	G
NC_040262.1	10063892	G	C
NC_040262.1	10118921	C	A
NC_040262.1	10176077	T	C
NC_040262.1	10223419	T	C
NC_040262.1	10274693	G	A
NC_040262.1	10326901	G	A
NC_040262.1	10378507	T	C
NC_040262.1	10429719	C	T
NC_040262.1	10464700	T	C
NC_040262.1	10505453	A	G
NC_040262.1	10508760	T	C
NC_040262.1	10509438	A	G
NC_040262.1	10515475	A	T
NC_040262.1	10516091	A	C
NC_040262.1	10571141	G	A
NC_040262.1	10632008	A	G
NC_040262.1	10634427	A	G
NC_040262.1	10677094	A	G
NC_040262.1	10717329	C	T
NC_040262.1	10722025	A	G
NC_040262.1	10760594	T	C
NC_040262.1	10763511	C	T
NC_040262.1	10810845	T	A
NC_040262.1	10834823	A	G
NC_040262.1	10835565	A	C
NC_040262.1	10859382	A	G
NC_040262.1	10872366	C	G
NC_040262.1	10903988	A	C
NC_040262.1	10932882	C	G
NC_040262.1	10935677	T	C
NC_040262.1	10941239	T	C
NC_040262.1	10945101	G	A
NC_040262.1	10946192	T	C
NC_040262.1	10953344	T	C
NC_040262.1	10993097	A	G
NC_040262.1	11020554	C	T

NC_040262.1	11052898	T	C
NC_040262.1	11054034	G	A
NC_040262.1	11058579	G	A
NC_040262.1	11112293	C	T
NC_040262.1	11169580	C	T
NC_040262.1	11228893	C	T
NC_040262.1	11285984	C	G
NC_040262.1	11340785	T	C
NC_040262.1	11395401	T	C
NC_040262.1	11432729	T	C
NC_040262.1	11433808	T	C
NC_040262.1	11446111	A	G
NC_040262.1	11502859	T	C
NC_040262.1	11544265	T	G
NC_040262.1	11544754	A	G
NC_040262.1	11545876	C	T
NC_040262.1	11579777	A	G
NC_040262.1	11613477	A	C
NC_040262.1	11632859	A	G
NC_040262.1	11687970	G	A
NC_040262.1	11744854	T	C
NC_040262.1	11791123	T	C
NC_040262.1	11795591	A	G
NC_040262.1	11843017	C	T
NC_040262.1	11885503	A	G
NC_040262.1	11886482	A	G
NC_040262.1	11887760	A	G
NC_040262.1	11888345	T	C
NC_040262.1	11892633	A	G
NC_040262.1	11893866	A	G
NC_040262.1	11894224	C	A
NC_040262.1	11945202	C	G
NC_040262.1	11956419	C	G
NC_040262.1	11956846	T	C
NC_040262.1	11967331	A	G
NC_040262.1	11978700	A	G
NC_040262.1	11979564	T	G
NC_040262.1	11992802	T	C
NC_040262.1	12042994	A	G
NC_040262.1	12044099	C	G
NC_040262.1	12055368	A	G
NC_040262.1	12061452	A	T
NC_040262.1	12062972	C	A
NC_040262.1	12087367	A	G
NC_040262.1	12119002	C	T
NC_040262.1	12135973	A	T
NC_040262.1	12137055	C	T

NC_040262.1	12139737	G	A
NC_040262.1	12140491	A	G
NC_040262.1	12141622	C	T
NC_040262.1	12142005	T	C
NC_040262.1	12143450	G	C
NC_040262.1	12147952	T	C
NC_040262.1	12152063	G	T
NC_040262.1	12157378	G	A
NC_040262.1	12190284	A	C
NC_040262.1	12212262	T	C
NC_040262.1	12232212	T	C
NC_040262.1	12283113	G	T
NC_040262.1	12291470	C	T
NC_040262.1	12293405	T	C
NC_040262.1	12293950	G	A
NC_040262.1	12295106	G	T
NC_040262.1	12315070	T	C
NC_040262.1	12315755	A	G
NC_040262.1	12318490	A	G
NC_040262.1	12322936	A	C
NC_040262.1	12326503	A	G
NC_040262.1	12336570	T	C
NC_040262.1	12365944	A	G
NC_040262.1	12417586	T	C
NC_040262.1	12463450	A	G
NC_040262.1	12464634	A	G
NC_040262.1	12466152	T	C
NC_040262.1	12471494	T	C
NC_040262.1	12475611	T	C
NC_040262.1	12522343	A	G
NC_040262.1	12648749	T	C
NC_040262.1	12708323	G	C
NC_040262.1	12738837	T	C
NC_040262.1	12752130	C	A
NC_040262.1	12790804	T	C
NC_040262.1	12792897	T	C
NC_040262.1	12795932	G	C
NC_040262.1	12798052	A	G
NC_040262.1	12853957	G	A
NC_040262.1	12897643	C	G
NC_040262.1	12901197	T	G
NC_040262.1	12926340	C	A
NC_040262.1	12934538	G	A
NC_040262.1	12949621	C	T
NC_040262.1	13005307	T	C
NC_040262.1	13057794	G	A
NC_040262.1	13058549	T	C

NC_040262.1	13059617	T	C
NC_040262.1	13061011	A	G
NC_040262.1	13061769	A	G
NC_040262.1	13062561	T	C
NC_040262.1	13111918	T	C
NC_040262.1	13169730	T	C
NC_040262.1	13224671	C	G
NC_040262.1	13280580	C	T
NC_040262.1	13335656	T	A
NC_040262.1	13400621	G	A
NC_040262.1	13451418	G	A
NC_040262.1	13507599	A	C
NC_040262.1	13560485	G	T
NC_040262.1	13602121	G	A
NC_040262.1	13647457	C	T
NC_040262.1	13692695	C	T
NC_040262.1	13723646	G	T
NC_040262.1	13729604	T	C
NC_040262.1	13774920	A	T
NC_040262.1	13827031	A	C
NC_040262.1	13887837	G	A
NC_040262.1	13938977	G	A
NC_040262.1	13987165	C	T
NC_040262.1	14082068	A	C
NC_040262.1	14136624	A	G
NC_040262.1	14151560	T	C
NC_040262.1	14170854	A	G
NC_040262.1	14228417	G	A
NC_040262.1	14279263	G	A
NC_040262.1	14319378	G	A
NC_040262.1	14356212	G	A
NC_040262.1	14407132	T	C
NC_040262.1	14442225	G	A
NC_040262.1	14581416	A	G
NC_040262.1	14600774	T	C
NC_040262.1	14663594	T	A
NC_040262.1	14715582	A	G
NC_040262.1	14766305	G	C
NC_040262.1	14804529	A	G
NC_040262.1	14832554	A	G
NC_040262.1	14836779	A	G
NC_040262.1	14866893	T	C
NC_040262.1	14899582	T	C
NC_040262.1	14900434	G	A
NC_040262.1	14904875	A	G
NC_040262.1	14911511	A	G
NC_040262.1	14930061	A	G

NC_040262.1	14938976	G	A
NC_040262.1	14957926	G	A
NC_040262.1	14976263	A	T
NC_040262.1	14996573	T	A
NC_040262.1	14998026	T	C
NC_040262.1	15032533	C	T
NC_040262.1	15071921	T	C
NC_040262.1	15075025	T	C
NC_040262.1	15105516	T	C
NC_040262.1	15118626	A	G
NC_040262.1	15132922	G	A
NC_040262.1	15165428	T	C
NC_040262.1	15172295	A	G
NC_040262.1	15201693	G	A
NC_040262.1	15205366	T	C
NC_040262.1	15213906	T	C
NC_040262.1	15214658	T	C
NC_040262.1	15261060	A	C
NC_040262.1	15311070	T	C
NC_040262.1	15452983	T	C
NC_040262.1	15509241	G	A
NC_040262.1	15556827	A	G
NC_040262.1	15608922	T	C
NC_040262.1	15661504	T	C
NC_040262.1	15716275	C	G
NC_040262.1	15768042	T	C
NC_040262.1	15837445	T	C
NC_040262.1	15884787	G	C
NC_040262.1	15930737	A	G
NC_040262.1	15959875	G	C
NC_040262.1	16007624	A	C
NC_040262.1	16032409	A	T
NC_040262.1	16088374	C	T
NC_040262.1	16142850	T	C
NC_040262.1	16191166	C	T
NC_040262.1	16240372	T	C
NC_040262.1	16294847	C	T
NC_040262.1	16353278	C	T
NC_040262.1	16404479	T	C
NC_040262.1	16459219	C	T
NC_040262.1	16522165	G	A
NC_040262.1	16574861	A	G
NC_040262.1	16616771	G	A
NC_040262.1	16664321	G	A
NC_040262.1	16707071	G	C
NC_040262.1	16761579	G	T
NC_040262.1	16805774	A	T

NC_040262.1	16840888	G	C
NC_040262.1	16844572	G	A
NC_040262.1	16942886	A	T
NC_040262.1	16998013	G	A
NC_040262.1	17054810	G	A
NC_040262.1	17107831	G	A
NC_040262.1	17164702	T	G
NC_040262.1	17219809	A	G
NC_040262.1	17270991	T	C
NC_040262.1	17325240	A	G
NC_040262.1	17380922	A	C
NC_040262.1	17435503	A	G
NC_040262.1	17484871	C	T
NC_040262.1	17542036	C	T
NC_040262.1	17591193	T	C
NC_040262.1	17626211	C	T
NC_040262.1	17631038	G	A
NC_040262.1	17686325	0	C
NC_040262.1	17742700	T	C
NC_040262.1	17799401	T	G
NC_040262.1	17857023	G	T
NC_040262.1	17897993	T	C
NC_040262.1	17898511	G	C
NC_040262.1	17942342	C	G
NC_040262.1	17981731	T	C
NC_040262.1	18020198	C	T
NC_040262.1	18023032	T	C
NC_040262.1	18078724	A	T
NC_040262.1	18136424	A	G
NC_040262.1	18165318	T	C
NC_040262.1	18171176	A	G
NC_040262.1	18175766	C	G
NC_040262.1	18238304	G	A
NC_040262.1	18239878	C	T
NC_040262.1	18245167	T	C
NC_040262.1	18279874	A	G
NC_040262.1	18283340	A	C
NC_040262.1	18283811	T	C
NC_040262.1	18340679	C	G
NC_040262.1	18396993	A	C
NC_040262.1	18431859	G	A
NC_040262.1	18449886	A	G
NC_040262.1	18450733	A	C
NC_040262.1	18454276	A	T
NC_040262.1	18499667	G	A
NC_040262.1	18526667	T	A
NC_040262.1	18536207	A	G

NC_040262.1	18539701	C	T
NC_040262.1	18557920	A	G
NC_040262.1	18584628	A	G
NC_040262.1	18587394	T	C
NC_040262.1	18603337	T	C
NC_040262.1	18613176	C	T
NC_040262.1	18633787	T	C
NC_040262.1	18634733	T	G
NC_040262.1	18635227	C	T
NC_040262.1	18636266	T	C
NC_040262.1	18642943	G	A
NC_040262.1	18690214	T	C
NC_040262.1	18732524	G	C
NC_040262.1	18735265	A	C
NC_040262.1	18788553	A	C
NC_040262.1	18843598	A	C
NC_040262.1	18867854	A	G
NC_040262.1	18871126	T	C
NC_040262.1	18880861	A	G
NC_040262.1	18884133	T	C
NC_040262.1	18890062	A	G
NC_040262.1	18905340	G	C
NC_040262.1	18906603	0	G
NC_040262.1	18907281	A	G
NC_040262.1	18910219	A	G
NC_040262.1	18933224	G	A
NC_040262.1	18991299	G	A
NC_040262.1	18992292	C	T
NC_040262.1	19036284	G	A
NC_040262.1	19041555	T	C
NC_040262.1	19055474	A	G
NC_040262.1	19066435	C	T
NC_040262.1	19078438	A	G
NC_040262.1	19147241	A	G
NC_040262.1	19153595	T	C
NC_040262.1	19190936	T	C
NC_040262.1	19192205	A	G
NC_040262.1	19242431	C	T
NC_040262.1	19292176	T	C
NC_040262.1	19339250	T	C
NC_040262.1	19369256	A	C
NC_040262.1	19381417	G	A
NC_040262.1	19394606	A	T
NC_040262.1	19396745	G	A
NC_040262.1	19452318	G	A
NC_040262.1	19507821	G	A
NC_040262.1	19556974	G	C

NC_040262.1	19607870	T	G
NC_040262.1	19609050	G	C
NC_040262.1	19633546	A	C
NC_040262.1	19752866	A	G
NC_040262.1	19774074	T	A
NC_040262.1	19774476	C	T
NC_040262.1	19906954	G	T
NC_040262.1	19943387	T	C
NC_040262.1	19973251	G	A
NC_040262.1	19992364	G	C
NC_040262.1	19996741	T	A
NC_040262.1	19997470	G	A
NC_040262.1	20009394	T	C
NC_040262.1	20019552	A	T
NC_040262.1	20040123	G	A
NC_040262.1	20053779	G	A
NC_040262.1	20103834	T	C
NC_040262.1	20149494	G	A
NC_040262.1	20150757	T	C
NC_040262.1	20176980	G	A
NC_040262.1	20263612	A	G
NC_040262.1	20266982	T	C
NC_040262.1	20313100	A	G
NC_040262.1	20325381	T	G
NC_040262.1	20335756	A	G
NC_040262.1	20351021	G	A
NC_040262.1	20382140	T	C
NC_040262.1	20383474	T	A
NC_040262.1	20406998	T	A
NC_040262.1	20475226	A	C
NC_040262.1	20479832	G	C
NC_040262.1	20480842	G	A
NC_040262.1	20486166	T	A
NC_040262.1	20528574	T	C
NC_040262.1	20580513	G	C
NC_040262.1	20626593	C	A
NC_040262.1	20630336	A	G
NC_040262.1	20676452	C	A
NC_040262.1	20715850	A	G
NC_040262.1	20721511	C	T
NC_040262.1	20731032	C	T
NC_040262.1	20772830	A	G
NC_040262.1	20819561	A	G
NC_040262.1	20820382	G	A
NC_040262.1	20829810	T	C
NC_040262.1	20844624	T	C
NC_040262.1	20845142	A	C

NC_040262.1	20856829	C	G
NC_040262.1	20878545	C	T
NC_040262.1	20880846	G	A
NC_040262.1	20884364	T	C
NC_040262.1	20889157	T	C
NC_040262.1	20892946	T	C
NC_040262.1	20894312	C	T
NC_040262.1	20898235	T	C
NC_040262.1	20948838	G	A
NC_040262.1	20949912	A	G
NC_040262.1	20951249	G	C
NC_040262.1	20962979	G	A
NC_040262.1	20999085	T	C
NC_040262.1	21006422	C	G
NC_040262.1	21009819	T	C
NC_040262.1	21042746	G	A
NC_040262.1	21083978	T	G
NC_040262.1	21090725	A	G
NC_040262.1	21091277	C	T
NC_040262.1	21092782	G	A
NC_040262.1	21094185	C	T
NC_040262.1	21096950	A	G
NC_040262.1	21099769	T	C
NC_040262.1	21109087	A	G
NC_040262.1	21120254	A	G
NC_040262.1	21135184	G	C
NC_040262.1	21165324	T	G
NC_040262.1	21207868	A	G
NC_040262.1	21280769	T	C
NC_040262.1	21288036	A	C
NC_040262.1	21290514	G	A
NC_040262.1	21336827	T	C
NC_040262.1	21390501	G	T
NC_040262.1	21438673	G	C
NC_040262.1	21447398	T	C
NC_040262.1	21524276	A	G
NC_040262.1	21580487	C	G
NC_040262.1	21620611	C	T
NC_040262.1	21837628	C	T
NC_040262.1	21937700	T	C
NC_040262.1	22006835	A	G
NC_040262.1	22054962	C	G
NC_040262.1	22119777	C	T
NC_040262.1	22174203	T	C
NC_040262.1	22177832	C	T
NC_040262.1	22233867	G	A
NC_040262.1	22275788	A	G

NC_040262.1	22313480	G	A
NC_040262.1	22365413	T	G
NC_040262.1	22406089	C	T
NC_040262.1	22474236	T	C
NC_040262.1	22509142	A	G
NC_040262.1	22530449	A	G
NC_040262.1	22534825	A	G
NC_040262.1	22556423	G	C
NC_040262.1	22591136	G	A
NC_040262.1	22642047	T	C
NC_040262.1	22665511	T	C
NC_040262.1	22722754	T	C
NC_040262.1	22728359	G	A
NC_040262.1	22733403	T	C
NC_040262.1	22749005	C	T
NC_040262.1	22762071	G	T
NC_040262.1	22791122	C	T
NC_040262.1	22792929	T	C
NC_040262.1	22836215	T	C
NC_040262.1	22838256	A	G
NC_040262.1	22844629	A	G
NC_040262.1	22844994	T	C
NC_040262.1	22849461	T	C
NC_040262.1	22864636	A	G
NC_040262.1	22906897	A	G
NC_040262.1	22924868	C	T
NC_040262.1	22974497	G	T
NC_040262.1	23013573	C	T
NC_040262.1	23048692	C	A
NC_040262.1	23061237	C	T
NC_040262.1	23063129	A	G
NC_040262.1	23091427	C	T
NC_040262.1	23093547	G	A
NC_040262.1	23137050	T	C
NC_040262.1	23140416	A	G
NC_040262.1	23143253	C	G
NC_040262.1	23146409	G	T
NC_040262.1	23161768	A	C
NC_040262.1	23173423	A	T
NC_040262.1	23201201	T	A
NC_040262.1	23254883	A	G
NC_040262.1	23288142	T	C
NC_040262.1	23327711	A	G
NC_040262.1	23341443	G	A
NC_040262.1	23343880	G	A
NC_040262.1	23398552	G	T
NC_040262.1	23439304	A	G

NC_040262.1	23439995	A	G
NC_040262.1	23448444	T	C
NC_040262.1	23457331	C	G
NC_040262.1	23467056	A	G
NC_040262.1	23476667	T	C
NC_040262.1	23479391	A	G
NC_040262.1	23517346	C	T
NC_040262.1	23555706	A	C
NC_040262.1	23624280	T	C
NC_040262.1	23673160	G	A
NC_040262.1	23732931	G	A
NC_040262.1	23776621	T	G
NC_040262.1	23917066	C	G
NC_040262.1	23923542	C	T
NC_040262.1	23929513	T	C
NC_040262.1	23943140	T	C
NC_040262.1	23962499	A	G
NC_040262.1	23966893	A	G
NC_040262.1	23971985	T	C
NC_040262.1	23978978	A	G
NC_040262.1	23980332	G	A
NC_040262.1	23981444	G	A
NC_040262.1	24025523	C	A
NC_040262.1	24080614	T	C
NC_040262.1	24132054	T	C
NC_040262.1	24169856	A	G
NC_040262.1	24171181	C	A
NC_040262.1	24181662	T	G
NC_040262.1	24218906	A	G
NC_040262.1	24271751	A	G
NC_040262.1	24272664	A	G
NC_040262.1	24274543	G	A
NC_040262.1	24305550	T	C
NC_040262.1	24313146	A	G
NC_040262.1	24324114	C	A
NC_040262.1	24345314	G	C
NC_040262.1	24399338	T	A
NC_040262.1	24441743	C	T
NC_040262.1	24477584	T	A
NC_040262.1	24528727	T	C
NC_040262.1	24554015	A	G
NC_040262.1	24563603	C	T
NC_040262.1	24564424	A	T
NC_040262.1	24601156	C	G
NC_040262.1	24609106	T	C
NC_040262.1	24652977	C	A
NC_040262.1	24686472	A	G

NC_040262.1	24691394	C	A
NC_040262.1	24702512	G	C
NC_040262.1	24705391	T	C
NC_040262.1	24714715	C	A
NC_040262.1	24766364	A	G
NC_040262.1	24807931	C	T
NC_040262.1	24874835	T	C
NC_040262.1	24876340	C	G
NC_040262.1	24881104	T	C
NC_040262.1	24904360	T	C
NC_040262.1	24904897	T	C
NC_040262.1	24952978	G	C
NC_040262.1	24953237	T	C
NC_040262.1	24953390	T	G
NC_040262.1	25007019	T	C
NC_040262.1	25062764	T	C
NC_040262.1	25118551	C	T
NC_040262.1	25173214	T	C
NC_040262.1	25214356	T	C
NC_040262.1	25267645	C	A
NC_040262.1	25318580	G	T
NC_040262.1	25379285	A	T
NC_040262.1	25382237	A	G
NC_040262.1	25382451	T	C
NC_040262.1	25434438	T	C
NC_040262.1	25434656	C	T
NC_040262.1	25435211	T	C
NC_040262.1	25436043	G	A
NC_040262.1	25437121	T	C
NC_040262.1	25437411	T	C
NC_040262.1	25438242	G	T
NC_040262.1	25447882	A	C
NC_040262.1	25448968	A	G
NC_040262.1	25456631	C	T
NC_040262.1	25460601	G	C
NC_040262.1	25506650	C	T
NC_040262.1	25533325	A	G
NC_040262.1	25571980	T	C
NC_040262.1	25599587	T	C
NC_040262.1	25642997	A	G
NC_040262.1	25707167	A	G
NC_040262.1	25761316	G	T
NC_040262.1	25802271	C	G
NC_040262.1	25848916	C	T
NC_040262.1	25900218	A	G
NC_040262.1	25935919	T	C
NC_040262.1	25982725	T	C

NC_040262.1	26021811	T	C
NC_040262.1	26024309	G	A
NC_040262.1	26025091	A	G
NC_040262.1	26025893	C	A
NC_040262.1	26026928	T	G
NC_040262.1	26027146	A	G
NC_040262.1	26029130	T	A
NC_040262.1	26030075	A	C
NC_040262.1	26048137	A	C
NC_040262.1	26049573	C	A
NC_040262.1	26050239	C	T
NC_040262.1	26050597	A	G
NC_040262.1	26120769	C	A
NC_040262.1	26165035	C	G
NC_040262.1	26226473	A	C
NC_040262.1	26233051	A	G
NC_040262.1	26233637	T	C
NC_040262.1	26287659	A	G
NC_040262.1	26342896	C	T
NC_040262.1	26394388	G	A
NC_040262.1	26417377	T	C
NC_040262.1	26417651	C	T
NC_040262.1	26417858	G	A
NC_040262.1	26422333	A	G
NC_040262.1	26487377	A	G
NC_040262.1	26514367	T	C
NC_040262.1	26515003	G	T
NC_040262.1	26515182	T	C
NC_040262.1	26525316	G	A
NC_040262.1	26578835	A	C
NC_040262.1	26633867	C	T
NC_040262.1	26663174	A	C
NC_040262.1	26663375	T	C
NC_040262.1	26663573	T	C
NC_040262.1	26663854	T	C
NC_040262.1	26732758	T	C
NC_040262.1	26760006	A	G
NC_040262.1	26760180	A	G
NC_040262.1	26766113	C	A
NC_040262.1	26775593	T	C
NC_040262.1	26780857	A	T
NC_040262.1	26782354	T	C
NC_040262.1	26782530	C	T
NC_040262.1	26782685	C	A
NC_040262.1	26799545	G	A
NC_040262.1	26808460	A	G
NC_040262.1	26817712	T	G

NC_040262.1	26818345	G	A
NC_040262.1	26818519	T	C
NC_040262.1	26839543	G	C
NC_040262.1	26840268	T	C
NC_040262.1	26851947	A	G
NC_040262.1	26852474	T	C
NC_040262.1	26852679	C	G
NC_040262.1	26855764	A	G
NC_040262.1	26858716	T	C
NC_040262.1	26905972	T	C
NC_040262.1	26937750	G	C
NC_040262.1	26978643	A	G
NC_040262.1	27006130	A	G
NC_040262.1	27009577	G	A
NC_040262.1	27011910	G	C
NC_040262.1	27023726	G	C
NC_040262.1	27025471	A	G
NC_040262.1	27025646	T	C
NC_040262.1	27077752	C	T
NC_040262.1	27080828	G	A
NC_040262.1	27094625	T	C
NC_040262.1	27124704	A	G
NC_040262.1	27132569	C	G
NC_040262.1	27134836	A	G
NC_040262.1	27138169	G	A
NC_040262.1	27138489	A	T
NC_040262.1	27139387	T	G
NC_040262.1	27146852	C	G
NC_040262.1	27152948	A	G
NC_040262.1	27176063	G	A
NC_040262.1	27176727	T	C
NC_040262.1	27200394	T	C
NC_040262.1	27223995	T	G
NC_040262.1	27226540	G	A
NC_040262.1	27259468	C	T
NC_040262.1	27269859	T	C
NC_040262.1	27283074	A	C
NC_040262.1	27285634	T	C
NC_040262.1	27310197	G	A
NC_040262.1	27342272	C	T
NC_040262.1	27391644	T	C
NC_040262.1	27434192	T	C
NC_040262.1	27435140	G	A
NC_040262.1	27489929	T	C
NC_040262.1	27523953	A	T
NC_040262.1	27524961	G	T
NC_040262.1	27536736	C	T

NC_040262.1	27543922	A	G
NC_040262.1	27547550	C	T
NC_040262.1	27597765	C	T
NC_040262.1	27688847	T	C
NC_040262.1	27728005	T	C
NC_040262.1	27746183	A	G
NC_040262.1	27783602	G	A
NC_040262.1	27837418	G	A
NC_040262.1	27895015	G	A
NC_040262.1	27957987	A	C
NC_040262.1	28027531	A	C
NC_040262.1	28088783	T	C
NC_040262.1	28090629	T	C
NC_040262.1	28105350	T	C
NC_040262.1	28111781	A	G
NC_040262.1	28126360	A	G
NC_040262.1	28172126	A	T
NC_040262.1	28188713	A	G
NC_040262.1	28243055	G	A
NC_040262.1	28294114	T	C
NC_040262.1	28328838	A	G
NC_040262.1	28345673	T	C
NC_040262.1	28346822	T	C
NC_040262.1	28348804	G	A
NC_040262.1	28359738	T	C
NC_040262.1	28361254	A	T
NC_040262.1	28363344	A	T
NC_040262.1	28379957	T	C
NC_040262.1	28380297	G	A
NC_040262.1	28411046	T	C
NC_040262.1	28443485	G	C
NC_040262.1	28476301	A	C
NC_040262.1	28479513	G	A
NC_040262.1	28488315	A	G
NC_040262.1	28489244	C	T
NC_040262.1	28492276	G	C
NC_040262.1	28495877	A	G
NC_040262.1	28525950	C	T
NC_040262.1	28526544	T	C
NC_040262.1	28580256	A	G
NC_040262.1	28634440	T	C
NC_040262.1	28652882	G	C
NC_040262.1	28666525	A	G
NC_040262.1	28668129	T	C
NC_040262.1	28668850	A	G
NC_040262.1	28670258	A	T
NC_040262.1	28683526	G	A

NC_040262.1	28730149	C	T
NC_040262.1	28776871	A	G
NC_040262.1	28789709	A	G
NC_040262.1	28801926	A	G
NC_040262.1	28802813	A	C
NC_040262.1	28822531	C	T
NC_040262.1	28822900	T	C
NC_040262.1	28824821	G	A
NC_040262.1	28846028	C	T
NC_040262.1	28849301	T	C
NC_040262.1	28857069	C	G
NC_040262.1	28867547	T	C
NC_040262.1	28889355	G	C
NC_040262.1	28909145	A	G
NC_040262.1	28913921	A	G
NC_040262.1	28921215	T	C
NC_040262.1	28926307	G	A
NC_040262.1	28943104	T	C
NC_040262.1	28943967	A	G
NC_040262.1	28953142	A	G
NC_040262.1	28958509	A	G
NC_040262.1	28959153	C	T
NC_040262.1	28959838	A	G
NC_040262.1	28961677	A	T
NC_040262.1	29015731	G	A
NC_040262.1	29029209	A	C
NC_040262.1	29032579	C	T
NC_040262.1	29048052	C	T
NC_040262.1	29102203	A	G
NC_040262.1	29155671	T	C
NC_040262.1	29186813	C	A
NC_040262.1	29188939	A	G
NC_040262.1	29189482	C	A
NC_040262.1	29189897	T	C
NC_040262.1	29190629	A	G
NC_040262.1	29191152	T	C
NC_040262.1	29219143	T	C
NC_040262.1	29221230	A	G
NC_040262.1	29242006	G	A
NC_040262.1	29282620	T	G
NC_040262.1	29323124	A	G
NC_040262.1	29372552	T	C
NC_040262.1	29418387	A	G
NC_040262.1	29444254	G	C
NC_040262.1	29445164	C	A
NC_040262.1	29511268	C	T
NC_040262.1	29513753	C	T

NC_040262.1	29516143	T	C
NC_040262.1	29516869	C	T
NC_040262.1	29571816	G	T
NC_040262.1	29624738	C	T
NC_040262.1	29671920	A	G
NC_040262.1	29703565	G	A
NC_040262.1	29742581	G	A
NC_040262.1	29772268	T	C
NC_040262.1	29773101	A	G
NC_040262.1	29812403	T	C
NC_040262.1	29822924	A	G
NC_040262.1	29851647	G	T
NC_040262.1	29853990	A	G
NC_040262.1	29856651	T	C
NC_040262.1	29872274	A	G
NC_040262.1	29877519	C	G
NC_040262.1	29923167	C	G
NC_040262.1	29967705	T	G
NC_040262.1	30033549	A	G
NC_040262.1	30087062	C	G
NC_040262.1	30157734	C	G
NC_040262.1	30158624	T	C
NC_040262.1	30160873	G	A
NC_040262.1	30209852	T	C
NC_040262.1	30211181	G	A
NC_040262.1	30261959	T	C
NC_040262.1	30296627	T	C
NC_040262.1	30343585	G	C
NC_040262.1	30374213	T	C
NC_040262.1	30432389	T	C
NC_040262.1	30490135	A	G
NC_040262.1	30537718	T	C
NC_040262.1	30596440	G	C
NC_040262.1	30651559	T	C
NC_040262.1	30690910	C	T
NC_040262.1	30737700	T	A
NC_040262.1	30827366	T	C
NC_040262.1	30885391	T	C
NC_040262.1	30939939	C	T
NC_040262.1	31007748	G	A
NC_040262.1	31061173	G	C
NC_040262.1	31114311	T	C
NC_040262.1	31174580	C	A
NC_040262.1	31227781	T	C
NC_040262.1	31273924	G	C
NC_040262.1	31336464	G	C
NC_040262.1	31381963	C	A

NC_040262.1	31419985	A	G
NC_040262.1	31420866	T	C
NC_040262.1	31523494	G	A
NC_040262.1	31597160	G	A
NC_040262.1	31644731	A	G
NC_040262.1	31700874	A	G
NC_040262.1	31756514	C	A
NC_040262.1	31806248	G	A
NC_040262.1	31849414	T	G
NC_040262.1	31896819	A	G
NC_040262.1	32227615	T	G
NC_040262.1	32280860	G	T
NC_040262.1	32334887	T	C
NC_040262.1	32388326	T	C
NC_040262.1	32445446	A	G
NC_040262.1	32502129	T	C
NC_040262.1	32558595	T	G
NC_040262.1	32614001	T	C
NC_040262.1	32667263	T	C
NC_040262.1	32726417	T	C
NC_040262.1	32789104	G	A
NC_040262.1	32850192	G	C
NC_040262.1	32904545	G	A
NC_040262.1	32950586	A	G
NC_040262.1	33006414	A	G
NC_040262.1	33063151	C	A
NC_040262.1	33114965	A	G
NC_040262.1	33170779	T	C
NC_040262.1	33226484	G	A
NC_040262.1	33279873	C	G
NC_040262.1	33336660	C	T
NC_040262.1	33391942	A	G
NC_040262.1	33443229	G	A
NC_040262.1	33496539	T	C
NC_040262.1	33551455	A	G
NC_040262.1	33612227	A	G
NC_040262.1	33666146	A	G
NC_040262.1	33723566	G	A
NC_040262.1	33760720	A	G
NC_040262.1	33799356	A	G
NC_040262.1	33839765	G	T
NC_040262.1	33909971	T	G
NC_040262.1	33961479	G	A
NC_040262.1	33980205	T	C
NC_040262.1	33991110	G	A
NC_040262.1	34023187	C	T
NC_040262.1	34079980	C	T

NC_040262.1	34136133	C	G
NC_040262.1	34187309	G	A
NC_040262.1	34243224	T	C
NC_040262.1	34293104	C	A
NC_040262.1	34301349	C	T
NC_040262.1	34341997	C	T
NC_040262.1	34387761	A	T
NC_040262.1	34389173	T	A
NC_040262.1	34422815	T	C
NC_040262.1	34468823	A	G
NC_040262.1	34483070	T	C
NC_040262.1	34486594	G	C
NC_040262.1	34533605	A	G
NC_040262.1	34590012	G	A
NC_040262.1	34641225	T	C
NC_040262.1	34697531	C	G
NC_040262.1	34754731	C	T
NC_040262.1	34808189	T	C
NC_040262.1	34865764	C	A
NC_040262.1	34920067	T	C
NC_040262.1	34974454	G	C
NC_040262.1	35025838	C	G
NC_040262.1	35062258	A	G
NC_040262.1	35065738	T	C
NC_040262.1	35066973	T	C
NC_040262.1	35114072	A	G
NC_040262.1	35130682	C	T
NC_040262.1	35132743	A	G
NC_040262.1	35185445	A	G
NC_040262.1	35243173	A	G
NC_040262.1	35298731	C	G
NC_040262.1	35343841	A	G
NC_040262.1	35346079	C	T
NC_040262.1	35348137	T	C
NC_040262.1	35362529	C	A
NC_040262.1	35402632	G	A
NC_040262.1	35450700	T	C
NC_040262.1	35470783	A	G
NC_040262.1	35491075	A	G
NC_040262.1	35518317	C	T
NC_040262.1	35536571	T	C
NC_040262.1	35538006	A	G
NC_040262.1	35546721	T	C
NC_040262.1	35571915	T	C
NC_040262.1	35589674	A	G
NC_040262.1	35593023	C	G
NC_040262.1	35595153	T	G

NC_040262.1	35604440	C	G
NC_040262.1	35607789	T	A
NC_040262.1	35609187	A	G
NC_040262.1	35630853	A	C
NC_040262.1	35640780	C	T
NC_040262.1	35686236	G	C
NC_040262.1	35726815	T	G
NC_040262.1	35760952	T	C
NC_040262.1	35816431	G	A
NC_040262.1	35831609	T	G
NC_040262.1	35838059	T	C
NC_040262.1	35853769	C	T
NC_040262.1	35884871	T	C
NC_040262.1	35890989	A	G
NC_040262.1	35908340	T	C
NC_040262.1	35938984	T	C
NC_040262.1	35950900	C	A
NC_040262.1	35956134	A	G
NC_040262.1	35963296	A	G
NC_040262.1	35969171	T	C
NC_040262.1	36008782	T	C
NC_040262.1	36016851	C	T
NC_040262.1	36061724	T	C
NC_040262.1	36078828	G	A
NC_040262.1	36080011	G	A
NC_040262.1	36106863	T	C
NC_040262.1	36120685	T	C
NC_040262.1	36132574	C	T
NC_040262.1	36180874	G	A
NC_040262.1	36183450	G	A
NC_040262.1	36201659	G	T
NC_040262.1	36206504	C	T
NC_040262.1	36207665	A	G
NC_040262.1	36217040	A	G
NC_040262.1	36218605	T	C
NC_040262.1	36231044	C	G
NC_040262.1	36233000	G	A
NC_040262.1	36236606	T	C
NC_040262.1	36242497	G	T
NC_040262.1	36247575	A	G
NC_040262.1	36247990	G	A
NC_040262.1	36269058	G	C
NC_040262.1	36276090	A	C
NC_040262.1	36280540	G	A
NC_040262.1	36281403	G	A
NC_040262.1	36286363	A	G
NC_040262.1	36298999	A	G

NC_040262.1	36299649	A	G
NC_040262.1	36300255	G	A
NC_040262.1	36302354	T	G
NC_040262.1	36317968	C	T
NC_040262.1	36328057	T	C
NC_040262.1	36339279	G	A
NC_040262.1	36383713	A	G
NC_040262.1	36397804	G	A
NC_040262.1	36405223	A	G
NC_040262.1	36430784	T	G
NC_040262.1	36438186	T	C
NC_040262.1	36493808	A	G
NC_040262.1	36526554	A	G
NC_040262.1	36530505	A	C
NC_040262.1	36532109	T	C
NC_040262.1	36533884	A	G
NC_040262.1	36535553	T	C
NC_040262.1	36536923	T	C
NC_040262.1	36540896	A	G
NC_040262.1	36543429	C	T
NC_040262.1	36562348	G	T
NC_040262.1	36631945	A	G
NC_040262.1	36633858	A	G
NC_040262.1	36696973	A	G
NC_040262.1	36703869	A	G
NC_040262.1	36727550	T	C
NC_040262.1	36741905	A	G
NC_040262.1	36743814	T	C
NC_040262.1	36747809	A	G
NC_040262.1	36750144	G	A
NC_040262.1	36752319	A	C
NC_040262.1	36790174	T	C
NC_040262.1	36795093	T	C
NC_040262.1	36810618	C	A
NC_040262.1	36873823	T	C
NC_040262.1	36876149	G	A
NC_040262.1	36877756	T	C
NC_040262.1	36904582	T	C
NC_040262.1	36905348	G	A
NC_040262.1	36909510	G	A
NC_040262.1	36910485	A	T
NC_040262.1	36912005	A	C
NC_040262.1	36914750	T	C
NC_040262.1	36921514	G	T
NC_040262.1	36925542	T	C
NC_040262.1	36932473	T	C
NC_040262.1	36933103	T	C

NC_040262.1	36934874	T	C
NC_040262.1	36948563	T	C
NC_040262.1	36953139	A	T
NC_040262.1	36957370	A	C
NC_040262.1	36962266	T	C
NC_040262.1	37027617	C	G
NC_040262.1	37033822	T	C
NC_040262.1	37065789	A	G
NC_040262.1	37178703	A	G
NC_040262.1	37208171	T	C
NC_040262.1	37209727	A	G
NC_040262.1	37247780	A	G
NC_040262.1	37260236	C	T
NC_040262.1	37290597	A	G
NC_040262.1	37293031	A	G
NC_040262.1	37293877	A	C
NC_040262.1	37345756	C	T
NC_040262.1	37409337	A	G
NC_040262.1	37463905	A	G
NC_040262.1	37520595	A	G
NC_040262.1	37577841	C	T
NC_040262.1	37637516	A	G
NC_040262.1	37677205	A	G
NC_040262.1	37708876	T	C
NC_040262.1	37754291	T	G
NC_040262.1	37813502	C	T
NC_040262.1	37863441	T	G
NC_040262.1	37908377	A	G
NC_040262.1	37945210	A	G
NC_040262.1	37947386	A	T
NC_040262.1	38004864	T	C
NC_040262.1	38057179	C	G
NC_040262.1	38069234	A	G
NC_040262.1	38119998	A	G
NC_040262.1	38123344	G	C
NC_040262.1	38130981	A	C
NC_040262.1	38161462	A	G
NC_040262.1	38170357	T	C
NC_040262.1	38224969	A	G
NC_040262.1	38277300	A	G
NC_040262.1	38301413	A	G
NC_040262.1	38313627	A	G
NC_040262.1	38314730	T	C
NC_040262.1	38325858	A	G
NC_040262.1	38333758	T	C
NC_040262.1	38339577	A	G
NC_040262.1	38346952	T	C

NC_040262.1	38409051	G	A
NC_040262.1	38456776	A	G
NC_040262.1	38514302	A	G
NC_040262.1	38571443	G	A
NC_040262.1	38590193	C	T
NC_040262.1	38642646	T	C
NC_040262.1	38643245	T	C
NC_040262.1	38644750	G	C
NC_040262.1	38646134	G	A
NC_040262.1	38657378	0	G
NC_040262.1	38706522	C	A
NC_040262.1	38735700	T	C
NC_040262.1	38745616	T	C
NC_040262.1	38748264	A	G
NC_040262.1	38750529	G	A
NC_040262.1	38814005	A	G
NC_040262.1	38867553	T	C
NC_040262.1	38901535	G	A
NC_040262.1	38913757	T	G
NC_040262.1	38940476	A	G
NC_040262.1	38962499	A	G
NC_040262.1	38965700	T	C
NC_040262.1	38967123	G	A
NC_040262.1	38995521	C	G
NC_040262.1	39004063	A	G
NC_040262.1	39007576	T	C
NC_040262.1	39067475	G	T
NC_040262.1	39124436	T	C
NC_040262.1	39125317	A	G
NC_040262.1	39125848	C	T
NC_040262.1	39164963	T	C
NC_040262.1	39257048	T	C
NC_040262.1	39438049	C	T
NC_040262.1	39456648	T	C
NC_040262.1	39509153	C	A
NC_040262.1	39531561	A	G
NC_040262.1	39589430	A	C
NC_040262.1	39645293	T	C
NC_040262.1	39709541	T	C
NC_040262.1	39757922	C	T
NC_040262.1	39779571	A	G
NC_040262.1	39792192	C	T
NC_040262.1	39793732	A	C
NC_040262.1	39845364	A	G
NC_040262.1	39899554	C	T
NC_040262.1	39956084	C	T
NC_040262.1	40002781	A	G

NC_040262.1	40010537	A	G
NC_040262.1	40011345	T	C
NC_040262.1	40012566	C	A
NC_040262.1	40019399	A	G
NC_040262.1	40022838	A	G
NC_040262.1	40056304	A	C
NC_040262.1	40059519	G	A
NC_040262.1	40066766	T	A
NC_040262.1	40084622	G	T
NC_040262.1	40137225	C	T
NC_040262.1	40193272	A	G
NC_040262.1	40255679	G	A
NC_040262.1	40299876	T	C
NC_040262.1	40303529	T	C
NC_040262.1	40304691	T	C
NC_040262.1	40307509	G	T
NC_040262.1	40308803	T	C
NC_040262.1	40319491	A	G
NC_040262.1	40369098	T	C
NC_040262.1	40407393	A	G
NC_040262.1	40410087	T	C
NC_040262.1	40412343	T	A
NC_040262.1	40452957	A	G
NC_040262.1	40486246	T	G
NC_040262.1	40507662	T	C
NC_040262.1	40523564	G	T
NC_040262.1	40527444	G	C
NC_040262.1	40553588	A	G
NC_040262.1	40561435	A	G
NC_040262.1	40562932	A	C
NC_040262.1	40597129	A	G
NC_040262.1	40608548	T	C
NC_040262.1	40621802	T	C
NC_040262.1	40623249	A	G
NC_040262.1	40633294	A	G
NC_040262.1	40635573	T	C
NC_040262.1	40651151	T	G
NC_040262.1	40688958	T	C
NC_040262.1	40689907	G	C
NC_040262.1	40691412	T	C
NC_040262.1	40737871	A	G
NC_040262.1	40772135	T	C
NC_040262.1	40780445	A	G
NC_040262.1	40783820	C	T
NC_040262.1	40784157	G	C
NC_040262.1	40813805	T	G
NC_040262.1	40815156	C	A

NC_040262.1	40863307	G	A
NC_040262.1	40884360	T	G
NC_040262.1	40885855	T	C
NC_040262.1	40940949	G	C
NC_040262.1	40999461	C	T
NC_040262.1	41056975	G	C
NC_040262.1	41129935	A	G
NC_040262.1	41201720	G	A
NC_040262.1	41256072	G	A
NC_040262.1	41283633	T	G
NC_040262.1	41337877	G	A
NC_040262.1	41348085	A	C
NC_040262.1	41402406	T	C
NC_040262.1	41446353	G	T
NC_040262.1	41486847	C	T
NC_040262.1	41529887	C	A
NC_040262.1	41538520	A	G
NC_040262.1	41590797	T	C
NC_040262.1	41643984	A	C
NC_040262.1	41683815	T	C
NC_040262.1	41716324	T	C
NC_040262.1	41766012	G	T
NC_040262.1	41784249	T	C
NC_040262.1	41784649	T	C
NC_040262.1	41810286	T	C
NC_040262.1	41811617	T	C
NC_040262.1	41854227	C	T
NC_040262.1	41855012	T	C
NC_040262.1	41880121	T	C
NC_040262.1	41917099	C	T
NC_040262.1	41970473	T	C
NC_040262.1	42013547	A	G
NC_040262.1	42068918	C	T
NC_040262.1	42136984	A	G
NC_040262.1	42191238	C	T
NC_040262.1	42227827	A	G
NC_040262.1	42273430	T	G
NC_040262.1	42324373	A	G
NC_040262.1	42375081	C	A
NC_040262.1	42415033	G	A
NC_040262.1	42415463	C	T
NC_040262.1	42461307	A	T
NC_040262.1	42494745	A	G
NC_040262.1	42498719	G	A
NC_040262.1	42500940	A	G
NC_040262.1	42515637	T	G
NC_040262.1	42549904	A	G

NC_040262.1	42552759	C	T
NC_040262.1	42553724	C	G
NC_040262.1	42563277	A	G
NC_040262.1	42563693	A	G
NC_040262.1	42572118	C	T
NC_040262.1	42574415	A	G
NC_040262.1	42575407	T	C
NC_040262.1	42631764	T	G
NC_040262.1	42684505	A	T
NC_040262.1	42733431	T	A
NC_040262.1	42776654	A	G
NC_040262.1	42781403	G	A
NC_040262.1	42854546	T	C
NC_040262.1	42855056	C	T
NC_040262.1	42896624	A	G
NC_040262.1	42948161	A	C
NC_040262.1	42990013	T	C
NC_040262.1	43029811	A	G
NC_040262.1	43051540	T	C
NC_040262.1	43071166	A	T
NC_040262.1	43072258	G	A
NC_040262.1	43096251	T	C
NC_040262.1	43119165	T	C
NC_040262.1	43121972	T	C
NC_040262.1	43142694	C	T
NC_040262.1	43143651	T	C
NC_040262.1	43199064	A	G
NC_040262.1	43201195	A	G
NC_040262.1	43202287	T	C
NC_040262.1	43214679	G	C
NC_040262.1	43218532	A	G
NC_040262.1	43221751	C	T
NC_040262.1	43223587	A	G
NC_040262.1	43234159	T	C
NC_040262.1	43236004	C	G
NC_040262.1	43243635	C	T
NC_040262.1	43246608	A	G
NC_040262.1	43251119	A	G
NC_040262.1	43252880	A	T
NC_040262.1	43260600	G	A
NC_040262.1	43296319	C	T
NC_040262.1	43299578	A	G
NC_040262.1	43311286	C	T
NC_040262.1	43318797	A	G
NC_040262.1	43326654	C	T
NC_040262.1	43327360	T	C
NC_040262.1	43358942	C	G

NC_040262.1	43371033	T	C
NC_040262.1	43411016	G	T
NC_040262.1	43450157	G	A
NC_040262.1	43482865	G	A
NC_040262.1	43484898	A	G
NC_040262.1	43490957	G	A
NC_040262.1	43493013	T	C
NC_040262.1	43514513	A	G
NC_040262.1	43521810	T	C
NC_040262.1	43543619	C	G
NC_040262.1	43544744	T	C
NC_040262.1	43563423	T	C
NC_040262.1	43599609	T	C
NC_040262.1	43661510	G	A
NC_040262.1	43716523	T	C
NC_040262.1	43771890	T	C
NC_040262.1	43830126	A	G
NC_040262.1	43884698	A	G
NC_040262.1	43940221	A	G
NC_040262.1	43996480	T	C
NC_040262.1	44083736	G	A
NC_040262.1	44127958	T	G
NC_040262.1	44179185	C	G
NC_040262.1	44403023	A	G
NC_040262.1	44463552	A	G
NC_040262.1	44520242	C	G
NC_040262.1	44571332	C	T
NC_040262.1	44623375	A	T
NC_040262.1	44678927	A	G
NC_040262.1	44731145	G	A
NC_040262.1	44778470	C	A
NC_040262.1	44820579	A	G
NC_040262.1	44870143	T	C
NC_040262.1	44911701	T	C
NC_040262.1	44927991	C	T
NC_040262.1	44937466	A	G
NC_040262.1	44950065	A	G
NC_040262.1	45003533	C	T
NC_040262.1	45055959	T	C
NC_040262.1	45105536	A	G
NC_040262.1	45117650	A	G
NC_040262.1	45170721	C	T
NC_040262.1	45229425	T	C
NC_040262.1	45270121	G	T
NC_040262.1	45324404	C	T
NC_040262.1	45413418	G	A
NC_040262.1	45468606	T	G

NC_040262.1	45495824	G	A
NC_040262.1	45557086	G	C
NC_040262.1	45573409	C	T
NC_040262.1	45587908	T	C
NC_040262.1	45593687	A	G
NC_040262.1	45596963	T	A
NC_040262.1	45640611	G	A
NC_040262.1	45662926	T	C
NC_040262.1	45724582	G	A
NC_040262.1	45737278	C	T
NC_040262.1	45796540	A	G
NC_040262.1	45829365	G	A
NC_040262.1	45844233	T	C
NC_040262.1	45860785	A	G
NC_040262.1	45861992	G	C
NC_040262.1	45862930	T	A
NC_040262.1	45863762	T	C
NC_040262.1	45865331	T	G
NC_040262.1	45867361	C	T
NC_040262.1	45910396	C	T
NC_040262.1	45941220	G	C
NC_040262.1	45947563	C	G
NC_040262.1	45948145	T	A
NC_040262.1	45949388	G	A
NC_040262.1	45950578	T	C
NC_040262.1	46006772	C	T
NC_040262.1	46057611	A	G
NC_040262.1	46083303	A	T
NC_040262.1	46221357	T	A
NC_040262.1	46277656	G	A
NC_040262.1	46337114	C	G
NC_040262.1	46396821	T	C
NC_040262.1	46486149	A	G
NC_040262.1	46531620	C	T
NC_040262.1	46584639	T	C
NC_040262.1	46630858	A	G
NC_040262.1	46709686	T	C
NC_040262.1	46764509	G	C
NC_040262.1	46806858	A	C
NC_040262.1	46847352	T	C
NC_040262.1	46899880	T	C
NC_040262.1	46973149	C	T
NC_040262.1	47029512	T	G
NC_040262.1	47099979	C	T
NC_040262.1	47159690	A	C
NC_040262.1	47223620	T	C
NC_040262.1	47265890	C	T

NC_040262.1	47325183	C	A
NC_040262.1	47384254	C	G
NC_040262.1	47439267	T	C
NC_040262.1	47493396	C	T
NC_040262.1	47549797	A	G
NC_040262.1	47614340	C	T
NC_040262.1	47674314	A	G
NC_040262.1	47705836	T	C
NC_040262.1	47745437	C	G
NC_040262.1	47810131	T	C
NC_040262.1	47859876	C	T
NC_040262.1	47943265	A	G
NC_040262.1	48015968	G	T
NC_040262.1	48078916	C	T
NC_040262.1	48127239	C	T
NC_040262.1	48192111	C	T
NC_040262.1	48248938	C	G
NC_040262.1	48305366	T	G
NC_040262.1	48357186	T	C
NC_040262.1	48499942	A	G
NC_040262.1	48594877	T	C
NC_040262.1	48649075	C	T
NC_040262.1	48683581	A	T
NC_040262.1	48685402	A	G
NC_040262.1	48694451	G	A
NC_040262.1	48695884	T	C
NC_040262.1	48753862	A	G
NC_040262.1	48793635	C	G
NC_040262.1	48842605	T	C
NC_040262.1	48899808	A	C
NC_040262.1	48923661	A	G
NC_040262.1	48978849	A	G
NC_040262.1	48998985	C	G
NC_040262.1	49001782	A	G
NC_040262.1	49056601	G	A
NC_040262.1	49116068	A	T
NC_040262.1	49126978	C	T
NC_040262.1	49136505	T	C
NC_040262.1	49152309	A	G
NC_040262.1	49176754	0	G
NC_040262.1	49234784	A	G
NC_040262.1	49284898	T	G
NC_040262.1	49353364	A	G
NC_040262.1	49406867	C	A
NC_040262.1	49440183	T	C
NC_040262.1	49522766	T	A
NC_040262.1	49625132	C	A

NC_040262.1	49646417	G	A
NC_040262.1	49696708	C	T
NC_040262.1	49750955	G	A
NC_040262.1	49808206	C	T
NC_040262.1	49873370	C	T
NC_040262.1	49924927	T	C
NC_040262.1	49946913	A	C
NC_040262.1	49973411	T	C
NC_040262.1	49976700	G	T
NC_040262.1	50031832	A	T
NC_040262.1	50090359	T	G
NC_040262.1	50091378	T	C
NC_040262.1	50136547	C	T
NC_040262.1	50189152	A	G
NC_040262.1	50215378	A	G
NC_040262.1	50283868	G	A
NC_040262.1	50346626	C	T
NC_040262.1	50394592	A	G
NC_040262.1	50572805	C	T
NC_040262.1	50621998	A	C
NC_040262.1	50689658	T	C
NC_040262.1	50740928	0	G
NC_040262.1	50796025	G	A
NC_040262.1	50852124	A	C
NC_040262.1	50908645	G	A
NC_040262.1	50965693	A	G
NC_040262.1	51010438	G	A
NC_040262.1	51046895	T	C
NC_040262.1	51096709	T	C
NC_040262.1	51154794	A	G
NC_040262.1	51193695	T	G
NC_040262.1	51239975	T	C
NC_040262.1	51291696	T	C
NC_040262.1	51352985	C	T
NC_040262.1	51416514	C	T
NC_040262.1	51420051	C	A
NC_040262.1	51425044	C	T
NC_040262.1	51477719	A	T
NC_040262.1	51541619	G	A
NC_040262.1	51595764	C	G
NC_040262.1	51625051	A	G
NC_040262.1	51682023	G	A
NC_040262.1	51738965	A	T
NC_040262.1	51796159	C	T
NC_040262.1	51850100	T	C
NC_040262.1	51867148	T	C
NC_040262.1	51923223	C	T

NC_040262.1	51978897	G	C
NC_040262.1	52030743	A	G
NC_040262.1	52086526	C	T
NC_040262.1	52141571	G	A
NC_040262.1	52195877	T	C
NC_040262.1	52249187	T	G
NC_040262.1	52249494	G	A
NC_040262.1	52252320	T	G
NC_040262.1	52325120	G	A
NC_040262.1	52327918	C	T
NC_040262.1	52328612	A	G
NC_040262.1	52382892	A	C
NC_040262.1	52435668	C	T
NC_040262.1	52492622	T	G
NC_040262.1	52544058	C	T
NC_040262.1	52601774	T	C
NC_040262.1	52658663	A	G
NC_040262.1	52723384	G	C
NC_040262.1	52723565	G	T
NC_040262.1	52725300	G	A
NC_040262.1	52760697	G	A
NC_040262.1	52767232	T	C
NC_040262.1	52801691	C	T
NC_040262.1	52813148	C	T
NC_040262.1	52863893	T	C
NC_040262.1	52886010	A	G
NC_040262.1	52939131	T	A
NC_040262.1	53008115	A	G
NC_040262.1	53066123	G	T
NC_040262.1	53126940	G	A
NC_040262.1	53179623	T	G
NC_040262.1	53225252	A	G
NC_040262.1	53279390	C	T
NC_040262.1	53280135	T	C
NC_040262.1	53315765	T	C
NC_040262.1	53326505	G	A
NC_040262.1	53362032	A	C
NC_040262.1	53406941	T	C
NC_040262.1	53407980	A	G
NC_040262.1	53474439	C	T
NC_040262.1	53530940	A	G
NC_040262.1	53580484	G	C
NC_040262.1	53583359	G	A
NC_040262.1	53649356	A	G
NC_040262.1	53710534	C	A
NC_040262.1	53729443	A	G
NC_040262.1	53777416	T	G

NC_040262.1	53779008	T	C
NC_040262.1	53779898	T	C
NC_040262.1	53781066	G	A
NC_040262.1	53782260	A	G
NC_040262.1	53847259	C	G
NC_040262.1	53905458	A	G
NC_040262.1	53958520	G	A
NC_040262.1	53998737	G	C
NC_040262.1	53999272	A	G
NC_040262.1	54053394	G	A
NC_040262.1	54111532	C	G
NC_040262.1	54165609	G	A
NC_040262.1	54219673	T	A
NC_040262.1	54247979	T	C
NC_040262.1	54248347	T	C
NC_040262.1	54248567	A	T
NC_040262.1	54248775	A	T
NC_040262.1	54309934	C	G
NC_040262.1	54365869	T	C
NC_040262.1	54376830	A	G
NC_040262.1	54404716	C	A
NC_040262.1	54447384	A	G
NC_040262.1	54447568	A	T
NC_040262.1	54462225	A	G
NC_040262.1	54482733	A	G
NC_040262.1	54491882	A	G
NC_040262.1	54492793	T	G
NC_040262.1	54496492	T	C
NC_040262.1	54497351	A	G
NC_040262.1	54505597	A	G
NC_040262.1	54517958	T	C
NC_040262.1	54548402	A	G
NC_040262.1	54554365	A	G
NC_040262.1	54616806	A	G
NC_040262.1	54617893	T	C
NC_040262.1	54649346	A	C
NC_040262.1	54662164	A	G
NC_040262.1	54662738	A	G
NC_040262.1	54663193	T	C
NC_040262.1	54687238	C	T
NC_040262.1	54696063	T	C
NC_040262.1	54822463	C	T
NC_040262.1	54888268	A	G
NC_040262.1	54941525	C	T
NC_040262.1	55002950	T	C
NC_040262.1	55100590	C	T
NC_040262.1	55176642	G	A

NC_040262.1	55233469	T	A
NC_040262.1	55290586	C	A
NC_040262.1	55344507	G	A
NC_040262.1	55397205	C	T
NC_040262.1	55455031	C	T
NC_040262.1	55519620	G	A
NC_040262.1	55573527	T	G
NC_040262.1	55640203	T	C
NC_040262.1	55692730	T	A
NC_040262.1	55741822	A	G
NC_040262.1	55785020	C	A
NC_040262.1	55827262	G	A
NC_040262.1	55880342	T	C
NC_040262.1	55937511	C	T
NC_040262.1	55990720	G	T
NC_040262.1	56040723	A	G
NC_040262.1	56093709	G	A
NC_040262.1	56144395	G	C
NC_040262.1	56198733	A	G
NC_040262.1	56249152	G	C
NC_040262.1	56295504	T	C
NC_040262.1	56348629	A	G
NC_040262.1	56405992	T	C
NC_040262.1	56463980	T	C
NC_040262.1	56521562	A	G
NC_040262.1	56531751	T	C
NC_040262.1	56589953	A	G
NC_040262.1	56647981	A	G
NC_040262.1	56703568	A	G
NC_040262.1	56760876	C	T
NC_040262.1	56825359	G	A
NC_040262.1	56889019	A	G
NC_040262.1	56944428	C	T
NC_040262.1	56999564	G	A
NC_040262.1	57024892	G	A
NC_040262.1	57045971	A	G
NC_040262.1	57058475	T	C
NC_040262.1	57114232	A	G
NC_040262.1	57170430	T	C
NC_040262.1	57226709	T	C
NC_040262.1	57284360	T	C
NC_040262.1	57337299	T	C
NC_040262.1	57387265	C	T
NC_040262.1	57442687	T	C
NC_040262.1	57500314	A	G
NC_040262.1	57548244	T	C
NC_040262.1	57603873	G	A

NC_040262.1	57661581	G	C
NC_040262.1	57714632	A	G
NC_040262.1	57767402	T	C
NC_040262.1	57798549	C	T
NC_040262.1	57854755	C	T
NC_040262.1	57916106	G	A
NC_040262.1	57967233	C	G
NC_040262.1	58012935	T	G
NC_040262.1	58064390	G	A
NC_040262.1	58109875	C	T
NC_040262.1	58165888	T	C
NC_040262.1	58234100	G	A
NC_040262.1	58288838	T	C
NC_040262.1	58407843	G	A
NC_040262.1	58462611	A	C
NC_040262.1	58463083	A	G
NC_040262.1	58509389	C	T
NC_040262.1	58546760	G	C
NC_040262.1	58550955	T	C
NC_040262.1	58551452	G	C
NC_040262.1	58551758	G	A
NC_040262.1	58560678	C	G
NC_040262.1	58560837	G	A
NC_040262.1	58561400	C	T
NC_040262.1	58562126	C	T
NC_040262.1	58562314	A	G
NC_040262.1	58562469	T	C
NC_040262.1	58563078	A	G
NC_040262.1	58563331	A	C
NC_040262.1	58563816	G	A
NC_040262.1	58564022	A	G
NC_040262.1	58564838	G	T
NC_040262.1	58567766	G	C
NC_040262.1	58569083	A	G
NC_040262.1	58598905	G	T
NC_040262.1	58655237	G	A
NC_040262.1	58683713	G	C
NC_040262.1	58744774	T	C
NC_040262.1	58788715	T	C
NC_040262.1	58834809	G	T
NC_040262.1	58853001	A	G
NC_040262.1	58854244	A	G
NC_040262.1	58854419	A	G
NC_040262.1	58909694	A	T
NC_040262.1	58963698	T	G
NC_040262.1	59016930	T	C
NC_040262.1	59066033	A	G

NC_040262.1	59121532	T	C
NC_040262.1	59175718	G	A
NC_040262.1	59228065	T	C
NC_040262.1	59284429	A	G
NC_040262.1	59342011	C	T
NC_040262.1	59393584	A	G
NC_040262.1	59436248	A	T
NC_040262.1	59518568	C	A
NC_040262.1	59575609	T	C
NC_040262.1	59630872	C	T
NC_040262.1	59664925	G	A
NC_040262.1	59680345	G	A
NC_040262.1	59741122	G	A
NC_040262.1	59792869	C	T
NC_040262.1	59849555	A	G
NC_040262.1	59900473	G	A
NC_040262.1	59950054	T	G
NC_040262.1	60003347	C	G
NC_040262.1	60029212	C	T
NC_040262.1	60029362	T	C
NC_040262.1	60051154	C	T
NC_040262.1	60051985	T	C
NC_040262.1	60052147	G	C
NC_040262.1	60105769	C	T
NC_040262.1	60155437	T	C
NC_040262.1	60197313	T	C
NC_040262.1	60252614	A	C
NC_040262.1	60302748	G	A
NC_040262.1	60356057	G	A
NC_040262.1	60408162	A	G
NC_040262.1	60462298	G	A
NC_040262.1	60518444	A	C
NC_040262.1	60576817	G	A
NC_040262.1	60628640	C	T
NC_040262.1	60682081	T	A
NC_040262.1	60735375	C	T
NC_040262.1	60795989	A	G
NC_040262.1	60848153	C	T
NC_040262.1	60901087	T	C
NC_040262.1	60946265	C	T
NC_040263.1	173991	A	C
NC_040263.1	205535	G	A
NC_040263.1	246038	C	T
NC_040263.1	363793	A	G
NC_040263.1	402666	G	A
NC_040263.1	547361	A	T
NC_040263.1	548067	G	A

NC_040263.1	580619	A	C
NC_040263.1	580808	A	G
NC_040263.1	581340	A	C
NC_040263.1	586751	A	G
NC_040263.1	708021	A	G
NC_040263.1	708346	T	C
NC_040263.1	723336	C	T
NC_040263.1	864034	G	T
NC_040263.1	919618	G	A
NC_040263.1	961301	G	C
NC_040263.1	1168293	C	T
NC_040263.1	1192378	C	A
NC_040263.1	1203638	T	C
NC_040263.1	1204612	C	A
NC_040263.1	1208990	C	T
NC_040263.1	1209597	A	G
NC_040263.1	1225140	T	A
NC_040263.1	1487438	A	G
NC_040263.1	1503221	G	A
NC_040263.1	1515313	G	C
NC_040263.1	1515519	T	C
NC_040263.1	1520322	C	T
NC_040263.1	1540197	T	C
NC_040263.1	1544347	C	G
NC_040263.1	1590974	T	C
NC_040263.1	1601501	G	A
NC_040263.1	1633748	T	C
NC_040263.1	1684711	A	G
NC_040263.1	1723402	A	G
NC_040263.1	1761116	T	C
NC_040263.1	1774780	G	T
NC_040263.1	1775017	C	T
NC_040263.1	1775968	G	A
NC_040263.1	1827215	T	C
NC_040263.1	1849895	A	G
NC_040263.1	1899005	T	G
NC_040263.1	1900304	C	T
NC_040263.1	1900518	A	G
NC_040263.1	1902348	T	C
NC_040263.1	1920034	T	C
NC_040263.1	1989745	G	A
NC_040263.1	1989938	A	G
NC_040263.1	1990124	T	C
NC_040263.1	1991843	G	A
NC_040263.1	2052403	G	C
NC_040263.1	2339503	A	T
NC_040263.1	2374720	G	A

NC_040263.1	2392170	T	C
NC_040263.1	2407987	T	C
NC_040263.1	2460748	G	C
NC_040263.1	2486814	A	G
NC_040263.1	2536790	T	C
NC_040263.1	2576487	A	G
NC_040263.1	2577550	A	T
NC_040263.1	2578992	T	C
NC_040263.1	2586823	C	T
NC_040263.1	2593831	C	T
NC_040263.1	2626487	A	T
NC_040263.1	2663453	G	A
NC_040263.1	2677111	T	C
NC_040263.1	2709919	A	G
NC_040263.1	2710839	T	C
NC_040263.1	2711179	T	C
NC_040263.1	2724850	G	A
NC_040263.1	2750242	T	C
NC_040263.1	2814840	C	G
NC_040263.1	2917836	G	A
NC_040263.1	2926938	A	C
NC_040263.1	2986970	A	G
NC_040263.1	2995802	G	A
NC_040263.1	3011501	T	C
NC_040263.1	3022531	T	A
NC_040263.1	3024101	C	A
NC_040263.1	3026356	G	T
NC_040263.1	3026782	C	T
NC_040263.1	3029466	C	T
NC_040263.1	3029641	A	G
NC_040263.1	3029844	A	G
NC_040263.1	3057941	G	A
NC_040263.1	3058638	T	C
NC_040263.1	3059240	C	G
NC_040263.1	3109357	A	G
NC_040263.1	3152674	T	C
NC_040263.1	3153370	T	C
NC_040263.1	3177901	C	T
NC_040263.1	3178258	T	G
NC_040263.1	3178507	C	T
NC_040263.1	3241210	A	G
NC_040263.1	3257780	T	C
NC_040263.1	3279211	A	G
NC_040263.1	3289830	A	G
NC_040263.1	3365534	T	C
NC_040263.1	3377668	A	T
NC_040263.1	3452589	G	A

NC_040263.1	3588827	C	T
NC_040263.1	3600507	G	T
NC_040263.1	3653591	G	C
NC_040263.1	3708899	C	G
NC_040263.1	3759100	A	G
NC_040263.1	3814206	A	G
NC_040263.1	3849616	T	C
NC_040263.1	3849908	G	A
NC_040263.1	3852737	G	A
NC_040263.1	3855828	G	C
NC_040263.1	3856123	T	C
NC_040263.1	3856598	G	A
NC_040263.1	3856962	T	C
NC_040263.1	3857258	T	C
NC_040263.1	3864130	A	G
NC_040263.1	3872912	T	C
NC_040263.1	3876398	A	G
NC_040263.1	3886084	A	G
NC_040263.1	3895843	G	C
NC_040263.1	3943451	G	A
NC_040263.1	3968648	T	C
NC_040263.1	3969486	C	T
NC_040263.1	3993012	G	C
NC_040263.1	4044853	T	C
NC_040263.1	4045509	A	G
NC_040263.1	4094176	T	A
NC_040263.1	4128374	T	C
NC_040263.1	4167334	T	C
NC_040263.1	4238268	T	C
NC_040263.1	4297559	T	C
NC_040263.1	4300377	T	C
NC_040263.1	4330711	T	C
NC_040263.1	4367178	G	T
NC_040263.1	4448644	T	C
NC_040263.1	4475712	G	A
NC_040263.1	4476519	A	G
NC_040263.1	4476795	G	A
NC_040263.1	4477874	T	A
NC_040263.1	4478526	T	A
NC_040263.1	4478708	T	A
NC_040263.1	4479459	A	T
NC_040263.1	4482608	A	G
NC_040263.1	4482897	T	G
NC_040263.1	4483397	T	C
NC_040263.1	4494227	T	C
NC_040263.1	4504684	C	T
NC_040263.1	4506291	G	A

NC_040263.1	4507108	T	C
NC_040263.1	4507768	C	T
NC_040263.1	4509573	C	G
NC_040263.1	4510527	C	T
NC_040263.1	4510730	A	C
NC_040263.1	4563096	T	C
NC_040263.1	4599424	G	A
NC_040263.1	4652312	G	C
NC_040263.1	4811858	C	T
NC_040263.1	4919224	A	G
NC_040263.1	4943708	G	A
NC_040263.1	5079578	A	G
NC_040263.1	5082591	A	G
NC_040263.1	5083661	A	G
NC_040263.1	5093024	C	A
NC_040263.1	5262193	A	C
NC_040263.1	5421478	G	A
NC_040263.1	5453599	A	G
NC_040263.1	5610263	C	G
NC_040263.1	5842020	T	G
NC_040263.1	5873891	C	T
NC_040263.1	5956503	T	A
NC_040263.1	6004974	C	T
NC_040263.1	6061752	T	C
NC_040263.1	6122168	G	A
NC_040263.1	6168483	G	C
NC_040263.1	6224342	C	G
NC_040263.1	6275918	G	A
NC_040263.1	6322281	A	G
NC_040263.1	6368739	G	T
NC_040263.1	6513051	T	C
NC_040263.1	6804528	G	C
NC_040263.1	6811914	C	G
NC_040263.1	12380426	T	C
NC_040263.1	12382333	C	T
NC_040263.1	12383500	A	G
NC_040263.1	12418304	C	A
NC_040263.1	12418611	C	T
NC_040263.1	12423741	T	G
NC_040263.1	12424732	G	A
NC_040263.1	12425815	C	T
NC_040263.1	12454038	T	C
NC_040263.1	12506126	T	C
NC_040263.1	12508204	C	A
NC_040263.1	12511099	A	G
NC_040263.1	12522319	A	G
NC_040263.1	12583493	T	C

NC_040263.1	12583832	G	A
NC_040263.1	12839372	C	T
NC_040263.1	13022386	A	C
NC_040263.1	13328917	C	A
NC_040263.1	13350240	G	A
NC_040263.1	13567087	T	C
NC_040263.1	13587902	T	C
NC_040263.1	16228859	C	G
NC_040263.1	16230873	A	C
NC_040263.1	16231086	A	G
NC_040263.1	16231309	A	G
NC_040263.1	16251668	T	C
NC_040263.1	16311625	A	G
NC_040263.1	16569461	G	A
NC_040263.1	16606665	C	T
NC_040263.1	16648090	T	G
NC_040263.1	16733433	C	T
NC_040263.1	16779587	A	C
NC_040263.1	16836952	T	C
NC_040263.1	16903591	G	C
NC_040263.1	20807492	C	T
NC_040263.1	20838707	T	C
NC_040263.1	20871113	G	A
NC_040263.1	20872359	T	C
NC_040263.1	20917966	T	C
NC_040263.1	20960002	T	G
NC_040263.1	21023999	T	C
NC_040263.1	21078362	G	C
NC_040263.1	21124832	T	C
NC_040263.1	21165024	A	G
NC_040263.1	21218846	A	G
NC_040263.1	21249854	T	G
NC_040263.1	21301864	A	G
NC_040263.1	21355101	A	G
NC_040263.1	21396199	C	T
NC_040263.1	21406401	G	A
NC_040263.1	21438215	A	G
NC_040263.1	21492726	C	A
NC_040263.1	21532737	A	G
NC_040263.1	21568640	G	A
NC_040263.1	21621347	T	A
NC_040263.1	21675794	G	A
NC_040263.1	21715806	G	A
NC_040263.1	21767058	G	C
NC_040263.1	21807402	A	T
NC_040263.1	21910919	T	G
NC_040263.1	21964555	T	C

NC_040263.1	22013247	A		C
NC_040263.1	22069336		0	C
NC_040263.1	22122962	C		G
NC_040263.1	22173156	G		T
NC_040263.1	22231543	T		C
NC_040263.1	22285851	G		A
NC_040263.1	22341314	A		G
NC_040263.1	22377050	G		A
NC_040263.1	22910474	G		C
NC_040263.1	22913566	T		C
NC_040263.1	22966880	G		A
NC_040263.1	23023568	T		C
NC_040263.1	23075731	C		T
NC_040263.1	23097720	C		A
NC_040263.1	23152616	G		A
NC_040263.1	23205715		0	A
NC_040263.1	23260554	G		A
NC_040263.1	23317230	A		C
NC_040263.1	23357189	T		C
NC_040263.1	23815647	T		C
NC_040263.1	23865564	A		G
NC_040263.1	23918314	T		C
NC_040263.1	23964589	T		C
NC_040263.1	23972946	A		G
NC_040263.1	23973926	G		A
NC_040263.1	24732690	C		T
NC_040263.1	24760650	C		T
NC_040263.1	25504278	A		C
NC_040263.1	25505864	A		G
NC_040263.1	25515418	T		C
NC_040263.1	25563716	C		T
NC_040263.1	25608386	C		T
NC_040263.1	25629754	C		T
NC_040263.1	25659882	A		G
NC_040263.1	25695605	G		T
NC_040263.1	25699816	A		G
NC_040263.1	25706668	T		G
NC_040263.1	25707676	G		A
NC_040263.1	25763100	C		T
NC_040263.1	25804745	A		G
NC_040263.1	26089579	A		G
NC_040263.1	26147512	A		G
NC_040263.1	26200481	A		G
NC_040263.1	26225480	G		A
NC_040263.1	26225868	A		T
NC_040263.1	26226436	T		C
NC_040263.1	26226718	T		A

NC_040263.1	26227197	T	C
NC_040263.1	26303142	C	A
NC_040263.1	26331051	A	G
NC_040263.1	26355895	C	G
NC_040263.1	26356162	A	C
NC_040263.1	26415702	T	C
NC_040263.1	26418558	T	G
NC_040263.1	27359398	T	C
NC_040263.1	27359638	A	G
NC_040263.1	27396087	T	C
NC_040263.1	28324536	A	G
NC_040263.1	28324693	T	G
NC_040263.1	28531824	T	C
NC_040263.1	28538165	A	G
NC_040263.1	28540503	C	T
NC_040263.1	28610610	C	T
NC_040263.1	28614120	G	A
NC_040263.1	28614729	C	A
NC_040263.1	28687423	G	A
NC_040263.1	28743093	T	C
NC_040263.1	28801257	T	C
NC_040263.1	28858130	A	G
NC_040263.1	28908691	G	A
NC_040263.1	28991531	G	A
NC_040263.1	28991756	C	T
NC_040263.1	28992030	C	A
NC_040263.1	28992324	A	G
NC_040263.1	29072946	A	C
NC_040263.1	29128068	T	C
NC_040263.1	29187266	A	T
NC_040263.1	29232862	A	G
NC_040263.1	29233747	A	G
NC_040263.1	29233904	T	C
NC_040263.1	29424742	G	C
NC_040263.1	29457314	A	G
NC_040263.1	29487383	G	A
NC_040263.1	29538888	A	G
NC_040263.1	29588144	C	T
NC_040263.1	29602929	A	G
NC_040263.1	29654069	T	C
NC_040263.1	29747800	C	T
NC_040263.1	29773409	A	G
NC_040263.1	29774344	T	C
NC_040263.1	29775065	G	A
NC_040263.1	29775324	A	G
NC_040263.1	29775529	C	T
NC_040263.1	29810115	A	G

NC_040263.1	29858061	C	T
NC_040263.1	29926386	A	G
NC_040263.1	29979155	G	A
NC_040263.1	29995547	C	T
NC_040263.1	29996874	A	G
NC_040263.1	29997384	A	G
NC_040263.1	30033036	C	G
NC_040263.1	30186814	G	C
NC_040263.1	30259747	G	T
NC_040263.1	30318300	C	A
NC_040263.1	30364294	T	C
NC_040263.1	30693982	A	G
NC_040263.1	30753496	A	G
NC_040263.1	30816417	G	A
NC_040263.1	30848462	C	A
NC_040263.1	30878761	C	T
NC_040263.1	30881353	C	T
NC_040263.1	30917189	C	T
NC_040263.1	30954668	G	A
NC_040263.1	30958833	T	C
NC_040263.1	31011387	G	C
NC_040263.1	31068437	C	T
NC_040263.1	31089091	T	A
NC_040263.1	31089348	T	C
NC_040263.1	31089500	C	A
NC_040263.1	31089678	A	G
NC_040263.1	31107558	T	C
NC_040263.1	31111039	C	A
NC_040263.1	31184942	C	A
NC_040263.1	31185463	A	G
NC_040263.1	31193405	C	T
NC_040263.1	31204304	A	G
NC_040263.1	31242436	G	C
NC_040263.1	31312137	G	C
NC_040263.1	31372643	G	A
NC_040263.1	31495647	A	G
NC_040263.1	31502253	G	A
NC_040263.1	31545890	C	T
NC_040263.1	31604334	T	C
NC_040263.1	31639191	G	C
NC_040263.1	31696644	G	A
NC_040263.1	31700442	A	G
NC_040263.1	31772538	T	C
NC_040263.1	31852791	T	C
NC_040263.1	31853642	G	A
NC_040263.1	31854038	G	A
NC_040263.1	31854277	T	C

NC_040263.1	31861129	G	A
NC_040263.1	31889992	C	T
NC_040263.1	31932036	A	G
NC_040263.1	31933882	T	C
NC_040263.1	31966258	C	T
NC_040263.1	32019620	A	G
NC_040263.1	32061784	A	G
NC_040263.1	32078298	T	C
NC_040263.1	32102852	T	A
NC_040263.1	32247651	C	A
NC_040263.1	32298001	C	T
NC_040263.1	32352709	T	C
NC_040263.1	32403997	C	A
NC_040263.1	32426657	T	C
NC_040263.1	32426917	C	T
NC_040263.1	32428022	A	G
NC_040263.1	32428265	G	C
NC_040263.1	32533035	A	G
NC_040263.1	32538008	A	C
NC_040263.1	32550189	T	C
NC_040263.1	32606953	G	A
NC_040263.1	32644605	G	A
NC_040263.1	32645826	T	C
NC_040263.1	32647202	T	C
NC_040263.1	32647987	A	G
NC_040263.1	32705153	C	T
NC_040263.1	32758560	T	C
NC_040263.1	32783749	G	A
NC_040263.1	32837015	C	T
NC_040263.1	32894038	T	A
NC_040263.1	32941681	G	A
NC_040263.1	33097213	T	C
NC_040263.1	33113562	T	C
NC_040263.1	33113749	G	A
NC_040263.1	33130064	T	C
NC_040263.1	33142459	T	C
NC_040263.1	33147622	G	A
NC_040263.1	33165522	A	G
NC_040263.1	33176659	C	A
NC_040263.1	33176969	A	C
NC_040263.1	33233807	T	C
NC_040263.1	33269825	G	A
NC_040263.1	33288394	A	C
NC_040263.1	33344990	G	A
NC_040263.1	33382157	T	C
NC_040263.1	33382522	G	A
NC_040263.1	33383132	C	T

NC_040263.1	33419923	C	T
NC_040263.1	33470695	C	G
NC_040263.1	33470853	C	G
NC_040263.1	33524692	A	G
NC_040263.1	33560496	G	T
NC_040263.1	33613866	C	T
NC_040263.1	33675886	T	C
NC_040263.1	33731168	G	A
NC_040263.1	33784713	C	T
NC_040263.1	33842067	A	G
NC_040263.1	33903669	A	G
NC_040263.1	34018242	G	C
NC_040263.1	34074354	T	G
NC_040263.1	34128193	G	C
NC_040263.1	34176038	T	C
NC_040263.1	34226733	T	C
NC_040263.1	34254154	A	G
NC_040263.1	34308111	C	T
NC_040263.1	34327937	A	G
NC_040263.1	34387546	T	C
NC_040263.1	34402537	G	A
NC_040263.1	34403002	T	C
NC_040263.1	34403503	C	T
NC_040263.1	34403712	T	C
NC_040263.1	34440594	A	C
NC_040263.1	34557921	A	G
NC_040263.1	34558767	A	G
NC_040263.1	34615131	G	C
NC_040263.1	34673477	T	A
NC_040263.1	34729773	T	C
NC_040263.1	34797467	C	T
NC_040263.1	34851382	A	G
NC_040263.1	34907108	G	T
NC_040263.1	34957238	G	A
NC_040263.1	34974303	G	A
NC_040263.1	35027250	C	T
NC_040263.1	35089219	T	C
NC_040263.1	35141817	T	C
NC_040263.1	35142214	G	C
NC_040263.1	35234196	G	C
NC_040263.1	35241345	A	C
NC_040263.1	35242873	C	G
NC_040263.1	35257874	T	G
NC_040263.1	35361159	C	T
NC_040263.1	35395927	A	C
NC_040263.1	35641738	T	C
NC_040263.1	35697915	C	A

NC_040263.1	35744411	A	C
NC_040263.1	36074441	G	A
NC_040263.1	36271047	T	C
NC_040263.1	36313627	C	T
NC_040263.1	36371545	T	C
NC_040263.1	36399959	T	G
NC_040263.1	36461822	C	T
NC_040263.1	36648104	C	A
NC_040263.1	36702159	T	G
NC_040263.1	36756955	G	A
NC_040263.1	36812693	T	C
NC_040263.1	36846106	A	T
NC_040263.1	36886410	A	G
NC_040263.1	36955754	A	G
NC_040263.1	36992041	G	C
NC_040263.1	37044785	C	A
NC_040263.1	37070135	A	G
NC_040263.1	37070401	T	C
NC_040263.1	37070654	T	C
NC_040263.1	37101760	C	T
NC_040263.1	37102450	T	C
NC_040263.1	37102749	T	C
NC_040263.1	37754302	A	C
NC_040263.1	37819992	C	G
NC_040263.1	37883883	A	C
NC_040263.1	37936309	C	T
NC_040263.1	37958269	T	C
NC_040263.1	38001684	G	A
NC_040263.1	38045524	G	A
NC_040263.1	38136743	A	G
NC_040263.1	38138715	G	A
NC_040263.1	38211058	A	G
NC_040263.1	38256902	T	C
NC_040263.1	38310965	A	G
NC_040263.1	38343327	G	A
NC_040263.1	38357402	A	G
NC_040263.1	38413535	C	T
NC_040263.1	38472250	C	G
NC_040263.1	38479910	G	T
NC_040263.1	38493187	A	G
NC_040263.1	38608990	G	A
NC_040263.1	38665676	G	A
NC_040263.1	38734202	G	A
NC_040263.1	38847852	A	G
NC_040263.1	38915896	T	G
NC_040263.1	38966343	A	G
NC_040263.1	39024300	C	T

NC_040263.1	39074987	G	A
NC_040263.1	39115265	C	T
NC_040263.1	39412198	C	T
NC_040263.1	39553578	C	G
NC_040263.1	39576138	A	G
NC_040263.1	40006640	A	G
NC_040263.1	40034418	G	A
NC_040263.1	40076550	C	T
NC_040263.1	40116018	C	T
NC_040263.1	40174221	C	T
NC_040263.1	40202659	G	A
NC_040263.1	40333819	G	A
NC_040263.1	40362528	T	A
NC_040263.1	40373925	C	T
NC_040263.1	40374771	A	G
NC_040263.1	40397819	A	G
NC_040263.1	40397988	C	A
NC_040263.1	40398181	G	A
NC_040263.1	40423190	T	C
NC_040263.1	40463844	A	G
NC_040263.1	40490267	G	A
NC_040263.1	40490707	T	G
NC_040263.1	40497322	G	A
NC_040263.1	40506405	A	G
NC_040263.1	40537622	C	G
NC_040263.1	40541276	C	G
NC_040263.1	40573227	T	C
NC_040263.1	40645828	A	G
NC_040263.1	40665121	G	A
NC_040263.1	40666612	C	G
NC_040263.1	40686591	C	T
NC_040263.1	40751605	C	T
NC_040263.1	40807009	T	C
NC_040263.1	40807174	T	C
NC_040263.1	40824548	A	G
NC_040263.1	40869240	G	A
NC_040263.1	40923983	G	A
NC_040263.1	40973663	T	C
NC_040263.1	41013829	G	A
NC_040263.1	41397925	T	C
NC_040263.1	41421937	G	T
NC_040263.1	41509744	T	C
NC_040263.1	41509973	A	T
NC_040263.1	41562607	G	C
NC_040263.1	41586390	T	A
NC_040263.1	41587348	A	G
NC_040263.1	41587518	A	G

NC_040263.1	41587690	G	A
NC_040263.1	41587987	T	C
NC_040263.1	41588338	C	A
NC_040263.1	41829085	C	T
NC_040263.1	41868945	G	C
NC_040263.1	41942526	A	G
NC_040263.1	41965163	T	C
NC_040263.1	41969047	G	A
NC_040263.1	42015591	C	T
NC_040263.1	42056657	T	C
NC_040263.1	42111226	C	G
NC_040263.1	42131781	A	G
NC_040263.1	42144869	A	C
NC_040263.1	42166167	G	A
NC_040263.1	42190989	A	G
NC_040263.1	42283337	T	C
NC_040263.1	42316586	T	C
NC_040263.1	42320237	C	T
NC_040263.1	42326860	C	G
NC_040263.1	42332980	A	G
NC_040263.1	42336937	T	C
NC_040263.1	42337292	A	G
NC_040263.1	42337608	A	G
NC_040263.1	42342421	A	G
NC_040263.1	42355236	G	C
NC_040263.1	42372537	T	C
NC_040263.1	42376395	T	C
NC_040263.1	42376577	A	G
NC_040263.1	42394181	T	G
NC_040263.1	42407846	A	G
NC_040263.1	42463189	T	C
NC_040263.1	42489899	G	T
NC_040263.1	42497362	C	T
NC_040263.1	42558462	A	G
NC_040263.1	42614593	A	G
NC_040263.1	42676270	G	A
NC_040263.1	42742513	C	T
NC_040263.1	42796375	T	G
NC_040263.1	42815682	A	T
NC_040263.1	42868155	A	C
NC_040263.1	42921205	A	T
NC_040263.1	42970832	G	A
NC_040263.1	43029840	G	A
NC_040263.1	43082683	A	C
NC_040263.1	43138753	T	C
NC_040263.1	43174688	T	C
NC_040263.1	43175488	T	C

NC_040263.1	43180823	G	A
NC_040263.1	43181398	T	A
NC_040263.1	43182151	C	T
NC_040263.1	43183165	G	A
NC_040263.1	43216314	G	T
NC_040263.1	43216546	A	G
NC_040263.1	43240224	G	C
NC_040263.1	43318170	G	T
NC_040263.1	43381722	T	C
NC_040263.1	43404522	T	C
NC_040263.1	43405963	T	C
NC_040263.1	43455272	A	T
NC_040263.1	43455562	G	A
NC_040263.1	43462668	C	T
NC_040263.1	44037536	C	A
NC_040263.1	44038143	G	A
NC_040263.1	44046140	G	A
NC_040263.1	44188913	A	G
NC_040263.1	44189298	G	T
NC_040263.1	44475988	C	A
NC_040263.1	44526314	T	G
NC_040263.1	44569165	C	T
NC_040263.1	44573238	A	G
NC_040263.1	44628249	A	G
NC_040263.1	44665780	G	A
NC_040263.1	44683598	A	G
NC_040263.1	44686580	C	T
NC_040263.1	44726976	A	G
NC_040263.1	44729511	A	G
NC_040263.1	44764801	C	T
NC_040263.1	44794281	A	G
NC_040263.1	44794518	G	A
NC_040263.1	44794785	G	A
NC_040263.1	44795679	T	C
NC_040263.1	44795838	G	A
NC_040263.1	44796132	T	C
NC_040263.1	44810889	T	A
NC_040263.1	44818210	G	A
NC_040263.1	44818421	T	C
NC_040263.1	44857945	C	A
NC_040263.1	44891955	A	G
NC_040263.1	44924460	A	G
NC_040263.1	44924833	C	A
NC_040263.1	44924997	G	A
NC_040263.1	44925286	T	C
NC_040263.1	44962082	G	C
NC_040263.1	45020356	A	G

NC_040263.1	45020716	T	C
NC_040263.1	45047617	G	A
NC_040263.1	45069170	T	C
NC_040263.1	45106298	A	G
NC_040263.1	45238402	C	T
NC_040263.1	45242984	G	T
NC_040263.1	45249681	A	G
NC_040263.1	45275547	A	G
NC_040263.1	45284266	A	G
NC_040263.1	45330757	G	A
NC_040263.1	45376783	A	C
NC_040263.1	45416208	T	C
NC_040263.1	45428771	A	G
NC_040263.1	45429206	C	T
NC_040263.1	45433200	T	A
NC_040263.1	45434001	T	C
NC_040263.1	45434538	C	G
NC_040263.1	45435366	A	G
NC_040263.1	45435724	T	C
NC_040263.1	45439640	T	C
NC_040263.1	45551043	C	T
NC_040263.1	45584508	G	A
NC_040263.1	45781332	C	T
NC_040263.1	45797332	T	A
NC_040263.1	45802714	T	C
NC_040263.1	45810523	G	A
NC_040263.1	45818227	G	A
NC_040263.1	45842414	G	A
NC_040263.1	45852985	A	G
NC_040263.1	45857181	A	G
NC_040263.1	45858773	T	C
NC_040263.1	45894261	C	T
NC_040263.1	45910627	T	C
NC_040263.1	45911378	T	C
NC_040263.1	45932592	G	A
NC_040263.1	45944648	A	G
NC_040263.1	45944880	T	C
NC_040263.1	45950521	A	G
NC_040263.1	45974096	A	G
NC_040263.1	46001275	T	C
NC_040263.1	46001446	T	C
NC_040263.1	46067416	T	C
NC_040263.1	46305473	A	G
NC_040263.1	46343714	T	C
NC_040263.1	46367120	C	T
NC_040263.1	46367396	A	G
NC_040263.1	46423313	A	G

NC_040263.1	46478551	T	C
NC_040263.1	46516883	G	C
NC_040263.1	46519296	C	T
NC_040263.1	46520356	C	T
NC_040263.1	46520560	T	C
NC_040263.1	46526316	A	G
NC_040263.1	46541997	C	T
NC_040263.1	46595314	T	C
NC_040263.1	46599091	C	G
NC_040263.1	46609171	C	T
NC_040263.1	46628931	T	C
NC_040263.1	46636799	T	C
NC_040263.1	46660764	C	A
NC_040263.1	46661701	C	T
NC_040263.1	46707159	T	C
NC_040263.1	46748213	G	A
NC_040263.1	46783493	A	G
NC_040263.1	46783871	A	G
NC_040263.1	46818601	A	G
NC_040263.1	46822355	G	A
NC_040263.1	46885031	A	G
NC_040263.1	46941724	A	G
NC_040263.1	46948055	A	G
NC_040263.1	46976675	G	C
NC_040263.1	46983991	G	A
NC_040263.1	47040596	A	G
NC_040263.1	47155528	T	C
NC_040263.1	47180201	T	C
NC_040263.1	47182250	T	C
NC_040263.1	47184010	C	T
NC_040263.1	47184904	C	T
NC_040263.1	47189920	A	G
NC_040263.1	47193788	T	C
NC_040263.1	47195432	A	G
NC_040263.1	47195872	C	T
NC_040263.1	47287212	A	G
NC_040263.1	47288192	T	G
NC_040263.1	47290526	T	C
NC_040263.1	47316304	T	C
NC_040263.1	47344209	C	G
NC_040263.1	47509509	C	T
NC_040263.1	47520214	A	G
NC_040263.1	47582782	T	C
NC_040263.1	47605976	A	G
NC_040263.1	47608056	G	A
NC_040263.1	47609851	C	G
NC_040263.1	47611159	C	T

NC_040263.1	47612867	A	G
NC_040263.1	47641694	G	A
NC_040263.1	47737038	C	T
NC_040263.1	47798992	G	A
NC_040263.1	48010065	C	T
NC_040263.1	48012580	G	A
NC_040263.1	48028697	T	C
NC_040263.1	48117820	T	C
NC_040263.1	48175653	T	C
NC_040263.1	48232354	T	C
NC_040263.1	48286475	G	A
NC_040263.1	48329476	A	G
NC_040263.1	48385667	G	T
NC_040263.1	48440624	G	A
NC_040263.1	48496173	A	G
NC_040263.1	48552169	T	C
NC_040263.1	48584571	T	C
NC_040263.1	48593839	T	C
NC_040263.1	48609230	G	C
NC_040263.1	48903139	C	T
NC_040263.1	48907159	C	T
NC_040263.1	48907986	T	C
NC_040263.1	48908348	T	C
NC_040263.1	48909970	C	A
NC_040263.1	48910220	G	A
NC_040263.1	48910505	T	C
NC_040263.1	48910661	T	C
NC_040263.1	48910853	A	G
NC_040263.1	48911110	G	T
NC_040263.1	48911900	A	G
NC_040263.1	48912616	T	C
NC_040263.1	48914169	T	C
NC_040263.1	48914336	G	A
NC_040263.1	48968090	T	C
NC_040263.1	49010071	C	T
NC_040263.1	49010808	T	A
NC_040263.1	49084590	T	C
NC_040263.1	49089338	T	G
NC_040263.1	49103811	T	C
NC_040263.1	49167104	A	G
NC_040263.1	49176749	G	A
NC_040263.1	49178653	A	G
NC_040263.1	49196439	C	T
NC_040263.1	49196733	T	C
NC_040263.1	49197274	C	T
NC_040263.1	49262439	A	G
NC_040263.1	50178974	A	G

NC_040263.1	50234482	T	C
NC_040263.1	50259811	A	G
NC_040263.1	50363488	T	G
NC_040263.1	50413541	T	C
NC_040263.1	50419757	G	C
NC_040263.1	50425918	C	G
NC_040263.1	50426086	C	T
NC_040263.1	50428474	A	G
NC_040263.1	50428787	T	C
NC_040263.1	50430412	A	G
NC_040263.1	50437121	A	G
NC_040263.1	50437896	C	T
NC_040263.1	50438184	C	T
NC_040263.1	50440305	A	G
NC_040263.1	50452414	T	C
NC_040263.1	50452922	T	C
NC_040263.1	50462218	T	G
NC_040263.1	50463330	G	T
NC_040263.1	50463556	C	G
NC_040263.1	50464691	T	C
NC_040263.1	50464965	T	C
NC_040263.1	50476588	T	C
NC_040263.1	50480013	T	C
NC_040263.1	50482122	T	C
NC_040263.1	50486504	G	A
NC_040263.1	50500318	C	T
NC_040263.1	50550872	C	T
NC_040263.1	50552473	C	T
NC_040263.1	50555046	C	G
NC_040263.1	50555266	C	T
NC_040263.1	50555453	A	G
NC_040263.1	50559080	A	C
NC_040263.1	50573263	G	T
NC_040263.1	50574355	G	A
NC_040263.1	50593519	A	C
NC_040263.1	50600091	C	T
NC_040263.1	50601233	A	C
NC_040263.1	50601614	G	A
NC_040263.1	50661772	A	G
NC_040263.1	50685256	A	G
NC_040263.1	50733201	0	A
NC_040263.1	50737562	T	C
NC_040263.1	50743398	C	G
NC_040263.1	50743989	C	T
NC_040263.1	50744223	A	G
NC_040263.1	50744517	T	G
NC_040263.1	50746559	A	G

NC_040263.1	50746785	C	G
NC_040263.1	50747741	T	C
NC_040263.1	50761513	T	C
NC_040263.1	50765350	G	A
NC_040263.1	50772430	A	G
NC_040263.1	50774448	C	T
NC_040263.1	50774646	T	C
NC_040263.1	50786894	G	A
NC_040263.1	50848100	A	C
NC_040263.1	50850745	G	A
NC_040263.1	50864529	C	T
NC_040263.1	50865100	C	T
NC_040263.1	50865528	T	C
NC_040263.1	50867030	A	C
NC_040263.1	50867247	C	T
NC_040263.1	50867493	T	C
NC_040263.1	50868266	A	T
NC_040263.1	50868666	T	G
NC_040263.1	50868977	A	G
NC_040263.1	50869492	A	G
NC_040263.1	50921768	A	G
NC_040263.1	50968195	C	A
NC_040263.1	51021765	G	A
NC_040263.1	51062597	A	G
NC_040263.1	51107471	T	C
NC_040263.1	52169577	A	G
NC_040263.1	52170259	T	C
NC_040263.1	52170488	T	C
NC_040263.1	52220491	G	A
NC_040263.1	52284494	C	T
NC_040263.1	53047379	A	G
NC_040263.1	53055820	T	G
NC_040263.1	53108558	C	T
NC_040263.1	53115607	A	G
NC_040263.1	53140698	C	T
NC_040263.1	53166382	A	G
NC_040263.1	53203719	T	C
NC_040263.1	53245205	T	C
NC_040263.1	53250151	A	G
NC_040263.1	53254296	A	G
NC_040263.1	53318135	G	A
NC_040263.1	53371675	C	T
NC_040263.1	53378034	A	G
NC_040263.1	53379051	C	A
NC_040263.1	53392598	A	G
NC_040263.1	53409356	C	G
NC_040263.1	53410207	A	G

NC_040263.1	53410767	C	T
NC_040263.1	53412451	T	C
NC_040263.1	53413525	C	G
NC_040263.1	53443436	A	T
NC_040263.1	53498995	A	C
NC_040263.1	53553680	A	G
NC_040263.1	53609664	T	C
NC_040263.1	53642272	C	G
NC_040263.1	53698239	A	G
NC_040263.1	53746695	T	C
NC_040263.1	53794806	T	C
NC_040263.1	53794961	T	C
NC_040263.1	53999146	C	T
NC_040263.1	54009701	G	A
NC_040263.1	54014666	A	G
NC_040263.1	54018977	T	G
NC_040263.1	54045594	T	C
NC_040263.1	54047762	T	G
NC_040263.1	54048232	A	G
NC_040263.1	54058162	T	C
NC_040263.1	54063884	T	C
NC_040263.1	54099149	G	A
NC_040263.1	54108231	T	C
NC_040263.1	54108415	G	A
NC_040263.1	54116564	A	G
NC_040263.1	54117324	C	T
NC_040263.1	54120954	A	G
NC_040263.1	54121319	A	G
NC_040263.1	54127898	A	G
NC_040263.1	54129177	T	G
NC_040263.1	54135648	A	G
NC_040263.1	54136052	A	G
NC_040263.1	54200927	G	T
NC_040263.1	54201256	G	A
NC_040263.1	54262040	T	C
NC_040263.1	54275626	T	C
NC_040263.1	54275982	T	C
NC_040263.1	54278439	A	G
NC_040263.1	54324053	T	A
NC_040263.1	54357214	T	C
NC_040263.1	54357822	A	G
NC_040263.1	54358574	T	C
NC_040263.1	54416955	T	C
NC_040263.1	54475432	T	C
NC_040263.1	54479352	A	G
NC_040263.1	54495944	A	G
NC_040263.1	54534845	A	T

NC_040263.1	54556764	T	C
NC_040263.1	54558726	A	G
NC_040263.1	54559108	A	G
NC_040263.1	54578681	A	C
NC_040263.1	54579052	A	G
NC_040263.1	54580143	A	G
NC_040263.1	54581546	G	A
NC_040263.1	54581741	A	G
NC_040263.1	54584143	T	A
NC_040263.1	54584556	T	G
NC_040263.1	54638723	A	C
NC_040263.1	54682421	T	C
NC_040263.1	54687947	A	C
NC_040263.1	54702331	G	A
NC_040263.1	54703529	A	G
NC_040263.1	54707094	A	C
NC_040263.1	54723325	T	G
NC_040263.1	54739909	A	G
NC_040263.1	54740339	T	C
NC_040263.1	54762937	G	A
NC_040263.1	54768025	A	G
NC_040263.1	54768805	A	G
NC_040263.1	54771012	G	C
NC_040263.1	54774021	T	C
NC_040263.1	54779466	G	T
NC_040263.1	54820226	A	G
NC_040263.1	54830747	A	G
NC_040263.1	54831855	A	G
NC_040263.1	54833127	A	T
NC_040263.1	54834197	C	T
NC_040263.1	54836003	T	C
NC_040263.1	54836189	T	C
NC_040263.1	54837838	A	G
NC_040263.1	54841291	C	G
NC_040263.1	54851043	A	G
NC_040263.1	54868235	A	G
NC_040263.1	54877010	C	G
NC_040263.1	54879566	T	C
NC_040263.1	54879833	G	A
NC_040263.1	54889130	G	A
NC_040263.1	54892246	T	C
NC_040263.1	54892913	T	G
NC_040263.1	54894088	G	T
NC_040263.1	54894613	A	G
NC_040263.1	54915264	A	G
NC_040263.1	54939669	C	T
NC_040263.1	54940716	T	G

NC_040263.1	54941903	C	A
NC_040263.1	54957024	A	G
NC_040263.1	54960905	A	C
NC_040263.1	54962281	A	G
NC_040263.1	54962537	A	G
NC_040263.1	54963188	A	G
NC_040263.1	54963738	A	C
NC_040263.1	54965726	G	A
NC_040263.1	54967154	A	G
NC_040263.1	54967566	G	A
NC_040263.1	54967962	A	G
NC_040263.1	54979584	C	T
NC_040263.1	54979821	A	G
NC_040263.1	54981264	G	A
NC_040263.1	54983292	T	C
NC_040263.1	54983913	A	G
NC_040263.1	54991531	T	C
NC_040263.1	54991873	A	G
NC_040263.1	54998247	T	C
NC_040263.1	54998577	C	T
NC_040263.1	55000274	A	G
NC_040263.1	55000426	A	C
NC_040263.1	55009141	T	C
NC_040263.1	55009529	A	G
NC_040263.1	55012196	A	G
NC_040263.1	55020130	G	T
NC_040263.1	55020882	T	C
NC_040263.1	55023085	A	G
NC_040263.1	55035573	G	A
NC_040263.1	55035881	A	G
NC_040263.1	55036132	T	G
NC_040263.1	55040472	T	C
NC_040263.1	55050309	G	A
NC_040263.1	55050750	G	A
NC_040263.1	55051809	T	C
NC_040263.1	55053277	C	T
NC_040263.1	55053460	C	T
NC_040263.1	55063793	T	G
NC_040263.1	55083724	G	A
NC_040263.1	55084208	A	C
NC_040263.1	55085099	T	C
NC_040263.1	55086016	G	T
NC_040263.1	55086300	T	C
NC_040263.1	55086618	G	A
NC_040263.1	55086770	T	C
NC_040263.1	55087360	A	G
NC_040263.1	55146491	G	A

NC_040263.1	55152499	A	G
NC_040263.1	55155175	G	A
NC_040263.1	55156492	C	A
NC_040263.1	55156664	C	A
NC_040263.1	55156922	A	G
NC_040263.1	55157109	G	C
NC_040263.1	55166946	T	G
NC_040263.1	55167169	G	A
NC_040263.1	55171075	G	A
NC_040263.1	55171420	A	G
NC_040263.1	55171611	A	G
NC_040263.1	55174321	T	C
NC_040263.1	55178142	A	T
NC_040263.1	55178546	G	A
NC_040263.1	55179087	T	C
NC_040263.1	55189323	T	C
NC_040263.1	55190059	C	G
NC_040263.1	55193554	T	C
NC_040263.1	55195674	A	G
NC_040263.1	55196382	T	C
NC_040263.1	55196864	T	C
NC_040263.1	55198237	A	G
NC_040263.1	55199207	G	A
NC_040263.1	55200419	T	C
NC_040263.1	55201107	G	A
NC_040263.1	55203010	C	A
NC_040263.1	55228046	T	G
NC_040263.1	55261115	T	C
NC_040263.1	55261844	C	T
NC_040263.1	55262055	C	T
NC_040263.1	55263382	G	C
NC_040263.1	55266899	A	G
NC_040263.1	55275672	T	C
NC_040263.1	55289195	A	G
NC_040263.1	55298150	A	G
NC_040263.1	55311350	A	G
NC_040263.1	55311842	C	A
NC_040263.1	55370571	C	T
NC_040263.1	55395866	G	A
NC_040263.1	55397701	G	A
NC_040263.1	55406558	T	C
NC_040263.1	55410215	A	G
NC_040263.1	55460628	C	T
NC_040263.1	55487837	A	T
NC_040263.1	55537909	G	C
NC_040263.1	55538874	T	A
NC_040263.1	55569263	A	G

NC_040263.1	55569536	A	G
NC_040263.1	55570788	G	A
NC_040263.1	55572565	T	C
NC_040263.1	55580993	A	G
NC_040263.1	55584741	T	C
NC_040263.1	55587811	T	C
NC_040263.1	55589430	A	G
NC_040263.1	55590804	C	G
NC_040263.1	55593885	C	A
NC_040263.1	55632663	C	T
NC_040263.1	55633632	C	T
NC_040263.1	55635083	G	A
NC_040263.1	55635292	A	G
NC_040263.1	55636673	C	T
NC_040263.1	55636871	T	G
NC_040263.1	55637327	C	T
NC_040263.1	55638028	G	A
NC_040263.1	55638422	A	C
NC_040263.1	55639648	T	C
NC_040263.1	55644513	T	C
NC_040263.1	55655366	A	G
NC_040263.1	55656712	C	T
NC_040263.1	55657086	A	G
NC_040263.1	55663392	T	C
NC_040263.1	55714932	T	A
NC_040263.1	55716141	A	G
NC_040263.1	55717674	A	T
NC_040263.1	55762733	G	A
NC_040263.1	55774519	T	C
NC_040263.1	55778904	G	A
NC_040263.1	55779211	G	A
NC_040263.1	55779449	T	C
NC_040263.1	55780039	T	C
NC_040263.1	55780344	T	G
NC_040263.1	55782115	A	G
NC_040263.1	55804957	A	G
NC_040263.1	55807773	G	T
NC_040263.1	55808019	G	A
NC_040263.1	55810736	C	T
NC_040263.1	55814397	T	C
NC_040263.1	55814984	C	T
NC_040263.1	55843223	G	A
NC_040263.1	55888204	T	C
NC_040263.1	55945611	C	T
NC_040263.1	56003331	A	G
NC_040263.1	56032227	T	C
NC_040263.1	56032832	A	G

NC_040263.1	56088134	T	C
NC_040263.1	56191423	A	G
NC_040263.1	56240030	T	A
NC_040263.1	56266972	C	T
NC_040263.1	57674567	G	T
NC_040263.1	57682392	T	A
NC_040263.1	57713136	A	G
NC_040263.1	57714336	A	G
NC_040263.1	57714603	A	G
NC_040263.1	57769689	G	T
NC_040263.1	57804381	C	G
NC_040263.1	57889451	C	A
NC_040263.1	57914674	G	A
NC_040263.1	57987572	T	G
NC_040263.1	58020263	A	G
NC_040263.1	58089475	C	T
NC_040263.1	58115389	G	A
NC_040263.1	58332287	A	G
NC_040263.1	58352230	T	A
NC_040263.1	58427358	G	A
NC_040263.1	58484771	G	A
NC_040263.1	58660666	G	A
NC_040263.1	58756213	C	T
NC_040263.1	58761979	T	G
NC_040263.1	58782526	G	A
NC_040263.1	58806384	C	T
NC_040263.1	58816284	T	C
NC_040263.1	58871528	A	G
NC_040263.1	58896302	G	A
NC_040263.1	59000620	T	C
NC_040263.1	59004748	T	C
NC_040263.1	59058087	G	C
NC_040263.1	59108652	C	T
NC_040263.1	59157736	T	C
NC_040263.1	59178489	A	G
NC_040263.1	59222105	A	G
NC_040263.1	59277421	T	G
NC_040263.1	59294279	A	G
NC_040263.1	59307290	T	C
NC_040263.1	59307587	C	T
NC_040263.1	59317064	C	T
NC_040263.1	59317530	G	A
NC_040263.1	59318684	T	C
NC_040263.1	59334690	A	G
NC_040263.1	59338729	T	C
NC_040263.1	59357457	A	G
NC_040263.1	59358229	A	G

NC_040263.1	59358528	G	C
NC_040263.1	59365396	G	A
NC_040263.1	59397728	C	T
NC_040263.1	59546604	C	G
NC_040263.1	59602681	A	G
NC_040263.1	59658320	T	C
NC_040263.1	59702961	G	T
NC_040263.1	59763132	G	A
NC_040263.1	59812533	C	A
NC_040263.1	59812718	A	G
NC_040263.1	59814692	G	A
NC_040263.1	59871235	G	T
NC_040263.1	59925311	C	G
NC_040263.1	59979955	G	T
NC_040263.1	60034919	A	G
NC_040263.1	60090967	G	A
NC_040263.1	60144455	A	G
NC_040263.1	60207201	T	A
NC_040263.1	60212805	C	T
NC_040263.1	60213213	C	T
NC_040263.1	60278761	A	G
NC_040263.1	60278977	C	T
NC_040263.1	60325012	C	T
NC_040263.1	60359300	A	G
NC_040263.1	60418248	C	T
NC_040263.1	60432256	T	C
NC_040263.1	60487403	T	G
NC_040263.1	60501041	G	A
NC_040263.1	60535863	A	G
NC_040263.1	60648295	A	G
NC_040263.1	60700260	A	G
NC_040263.1	60749963	T	C
NC_040263.1	60799924	T	C
NC_040263.1	60849785	T	C
NC_040263.1	61365459	A	G
NC_040263.1	61422359	G	A
NC_040263.1	61481699	G	A
NC_040263.1	61526606	G	A
NC_040263.1	61842735	A	C
NC_040263.1	61882033	T	C
NC_040263.1	61972369	T	C
NC_040263.1	62013881	A	T
NC_040263.1	62044243	A	G
NC_040263.1	62099685	G	A
NC_040263.1	62121882	G	A
NC_040263.1	62166641	T	C
NC_040263.1	62210268	A	C

NC_040263.1	62235506	A	T
NC_040263.1	62236078	C	T
NC_040263.1	62236446	T	C
NC_040263.1	62286092	G	A
NC_040263.1	62343644	G	A
NC_040263.1	62398272	T	C
NC_040263.1	62451446	C	T
NC_040263.1	62497600	T	G
NC_040263.1	62554148	G	C
NC_040263.1	62602686	C	G
NC_040263.1	62631807	G	C
NC_040263.1	62698326	T	C
NC_040263.1	62747317	G	A
NC_040263.1	62783633	C	T
NC_040263.1	63659017	T	C
NC_040263.1	63689197	T	C
NC_040263.1	63756975	G	C
NC_040263.1	63782357	T	G
NC_040263.1	63832834	C	A
NC_040263.1	63899877	T	C
NC_040263.1	63946843	A	G
NC_040263.1	64004626	G	A
NC_040263.1	64041301	A	C
NC_040263.1	64073669	A	C
NC_040263.1	64123625	C	T
NC_040263.1	64179437	T	C
NC_040263.1	64183761	A	G
NC_040263.1	64201604	A	G
NC_040263.1	64219636	T	C
NC_040263.1	64223387	T	C
NC_040263.1	64252947	A	G
NC_040263.1	64461665	A	G
NC_040263.1	64514818	G	A
NC_040263.1	64569941	C	T
NC_040263.1	64615355	G	A
NC_040263.1	64615551	A	G
NC_040263.1	64616732	G	A
NC_040263.1	64617841	C	A
NC_040263.1	64621240	C	T
NC_040263.1	64621496	A	G
NC_040263.1	64714151	C	T
NC_040263.1	64719607	A	G
NC_040263.1	64750976	G	C
NC_040263.1	64751324	G	T
NC_040263.1	64753670	T	G
NC_040263.1	64753869	C	T
NC_040263.1	64754385	A	G

NC_040263.1	64754571	A	G
NC_040263.1	64757401	T	A
NC_040263.1	64759712	A	G
NC_040263.1	64788529	A	G
NC_040263.1	64788961	G	A
NC_040263.1	64789717	C	T
NC_040263.1	64789889	T	C
NC_040263.1	64791576	A	G
NC_040263.1	64792428	C	T
NC_040263.1	64796126	T	C
NC_040263.1	64812419	A	G
NC_040263.1	64874138	T	A
NC_040263.1	64934534	T	C
NC_040263.1	64993898	C	T
NC_040263.1	64994051	C	T
NC_040263.1	65016733	G	C
NC_040263.1	65020764	A	G
NC_040263.1	65089905	C	T
NC_040263.1	65090057	T	C
NC_040263.1	65090572	T	G
NC_040263.1	65119260	A	C
NC_040263.1	65166790	C	T
NC_040263.1	65187879	A	G
NC_040263.1	65198959	G	A
NC_040263.1	65332123	A	C
NC_040263.1	65355429	C	T
NC_040263.1	65356143	T	A
NC_040263.1	65359093	C	T
NC_040263.1	65359947	C	A
NC_040263.1	65381925	G	T
NC_040263.1	65438775	A	G
NC_040263.1	65441300	T	C
NC_040263.1	65500646	C	T
NC_040263.1	65510870	A	G
NC_040263.1	65511029	A	G
NC_040263.1	65511265	G	A
NC_040263.1	65549626	A	T
NC_040263.1	65591777	C	A
NC_040263.1	65619860	C	T
NC_040263.1	65621070	A	G
NC_040263.1	65630096	C	T
NC_040263.1	65630893	T	A
NC_040263.1	65632297	A	G
NC_040263.1	65632884	G	T
NC_040263.1	65696909	T	C
NC_040263.1	65699530	G	A
NC_040263.1	65758039	C	T

NC_040263.1	65807643	T	C
NC_040263.1	65858914	T	C
NC_040263.1	65917631	C	T
NC_040263.1	65973658	A	G
NC_040263.1	66017531	G	A
NC_040263.1	66058896	C	T
NC_040263.1	66197874	A	G
NC_040263.1	66251423	A	G
NC_040263.1	66306534	A	G
NC_040263.1	66360934	G	A
NC_040263.1	66402314	C	G
NC_040263.1	66438267	A	G
NC_040263.1	66465610	A	G
NC_040263.1	66477063	T	C
NC_040263.1	66477321	C	G
NC_040263.1	66477583	T	C
NC_040263.1	66479196	C	T
NC_040263.1	66479380	C	T
NC_040263.1	66479653	T	C
NC_040263.1	66479982	G	C
NC_040263.1	66482191	A	G
NC_040263.1	66550300	G	A
NC_040263.1	66551632	G	A
NC_040263.1	66552426	C	T
NC_040263.1	66587142	T	C
NC_040263.1	66587579	T	C
NC_040263.1	66587970	A	G
NC_040263.1	67075412	A	G
NC_040263.1	67103003	A	G
NC_040263.1	67154689	G	A
NC_040263.1	67208091	T	G
NC_040263.1	67272238	A	G
NC_040263.1	67337323	A	G
NC_040263.1	67338370	T	C
NC_040263.1	67360589	A	G
NC_040263.1	67389589	A	C
NC_040263.1	67392933	T	C
NC_040263.1	67407347	T	C
NC_040263.1	67407708	G	C
NC_040263.1	67408637	T	C
NC_040263.1	67410354	A	G
NC_040263.1	67410673	T	C
NC_040263.1	67736299	T	C
NC_040263.1	68051040	T	C
NC_040263.1	68062066	A	G
NC_040263.1	68062498	C	T
NC_040263.1	68118419	A	G

NC_040263.1	68161931	A	G
NC_040263.1	68210827	C	T
NC_040263.1	68210981	T	C
NC_040263.1	68276266	T	G
NC_040263.1	68276785	T	C
NC_040263.1	68278650	A	C
NC_040263.1	68279931	T	C
NC_040263.1	68280495	T	G
NC_040263.1	68282081	A	G
NC_040263.1	68307674	C	G
NC_040263.1	68423045	G	T
NC_040263.1	68464790	G	A
NC_040263.1	68465485	T	C
NC_040263.1	68494519	C	G
NC_040263.1	68516771	C	T
NC_040263.1	68519619	T	C
NC_040263.1	68524733	C	T
NC_040263.1	68557467	C	T
NC_040263.1	68635837	T	C
NC_040263.1	68688968	T	C
NC_040263.1	68721532	C	T
NC_040263.1	68733013	T	C
NC_040263.1	68736879	G	A
NC_040263.1	68739503	A	G
NC_040263.1	68753093	G	A
NC_040263.1	68753502	A	G
NC_040263.1	68753686	T	C
NC_040263.1	68814489	T	C
NC_040263.1	68830897	T	C
NC_040263.1	68867246	G	A
NC_040263.1	68867406	C	T
NC_040263.1	68870648	C	T
NC_040263.1	68871135	A	G
NC_040263.1	68929133	T	C
NC_040263.1	68984149	A	G
NC_040263.1	69035438	G	T
NC_040263.1	69088474	A	G
NC_040263.1	69133215	A	G
NC_040263.1	69169375	A	G
NC_040263.1	69190628	A	G
NC_040263.1	69249049	T	C
NC_040263.1	69267636	G	A
NC_040263.1	69268361	A	G
NC_040263.1	69269061	C	T
NC_040263.1	69402485	T	C
NC_040263.1	69449071	T	C
NC_040263.1	69484428	A	G

NC_040263.1	69528647	T	C
NC_040263.1	69588139	T	C
NC_040263.1	69658583	C	T
NC_040263.1	69729900	C	T
NC_040263.1	70034161	C	T
NC_040263.1	70087935	G	A
NC_040263.1	70153902	T	C
NC_040263.1	70251834	A	C
NC_040263.1	70288010	G	A
NC_040263.1	70326046	G	A
NC_040263.1	70397125	C	A
NC_040263.1	70398162	G	A
NC_040263.1	70399177	T	C
NC_040263.1	70424273	T	C
NC_040263.1	70437689	C	T
NC_040263.1	70464256	A	C
NC_040263.1	70496548	A	G
NC_040263.1	70497553	C	T
NC_040263.1	70499019	T	C
NC_040263.1	70553643	A	T
NC_040263.1	70611025	C	A
NC_040263.1	70670185	A	G
NC_040263.1	70734895	C	T
NC_040263.1	70775402	T	C
NC_040263.1	70776552	C	T
NC_040263.1	70802180	T	C
NC_040263.1	70815732	T	A
NC_040263.1	70825121	C	T
NC_040263.1	70835943	A	G
NC_040263.1	70888642	G	A
NC_040263.1	70918383	A	T
NC_040263.1	70923154	T	A
NC_040263.1	70930841	G	A
NC_040263.1	70931917	A	G
NC_040263.1	70943564	A	G
NC_040263.1	71449615	A	T
NC_040263.1	71505944	A	G
NC_040263.1	71550126	T	C
NC_040263.1	71594145	G	C
NC_040263.1	71681928	G	A
NC_040263.1	71756447	G	C
NC_040263.1	71785817	T	C
NC_040263.1	71833723	G	A
NC_040263.1	71863406	A	G
NC_040263.1	71874066	T	G
NC_040263.1	71875751	T	C
NC_040263.1	71904490	G	A

NC_040263.1	71966316	C	A
NC_040263.1	71988810	C	T
NC_040263.1	72005947	A	G
NC_040263.1	72201292	G	A
NC_040263.1	72224596	T	C
NC_040263.1	72259416	T	C
NC_040263.1	72404538	A	C
NC_040263.1	72406853	T	C
NC_040263.1	72408624	A	G
NC_040263.1	72460085	C	T
NC_040263.1	72460593	A	G
NC_040263.1	72476336	A	G
NC_040263.1	72535806	C	T
NC_040263.1	72749107	T	C
NC_040263.1	72934698	T	C
NC_040263.1	72990538	T	C
NC_040263.1	73020968	G	A
NC_040263.1	73056354	A	G
NC_040263.1	73088679	A	G
NC_040263.1	73387617	A	G
NC_040263.1	73439193	T	C
NC_040263.1	73486100	C	T
NC_040263.1	73519358	C	T
NC_040263.1	73522952	C	T
NC_040263.1	74121305	A	G
NC_040263.1	74133590	G	A
NC_040263.1	74139660	A	G
NC_040263.1	74145438	A	G
NC_040263.1	74145659	T	C
NC_040263.1	74146278	T	A
NC_040263.1	74168634	T	A
NC_040263.1	74223599	T	C
NC_040263.1	74279646	A	G
NC_040263.1	74334630	A	G
NC_040263.1	74385893	T	C
NC_040263.1	74400610	G	C
NC_040263.1	74442937	T	C
NC_040263.1	74472909	A	C
NC_040263.1	74722957	A	G
NC_040263.1	74723296	T	C
NC_040263.1	74724536	C	A
NC_040263.1	74725909	A	G
NC_040263.1	74767217	T	A
NC_040263.1	74769821	T	G
NC_040263.1	74770406	A	C
NC_040263.1	74770692	A	G
NC_040263.1	75321594	C	T

NC_040263.1	75321893	C	T
NC_040263.1	75325347	T	A
NC_040263.1	75326347	T	G
NC_040263.1	75326682	C	G
NC_040263.1	75326858	T	C
NC_040263.1	75328965	A	G
NC_040263.1	75421128	T	C
NC_040263.1	75448883	T	C
NC_040263.1	75504803	A	G
NC_040263.1	75505191	C	T
NC_040263.1	75505391	C	T
NC_040263.1	75506234	C	T
NC_040263.1	75556793	A	C
NC_040263.1	75640167	T	C
NC_040263.1	75689261	C	T
NC_040263.1	75739474	T	C
NC_040263.1	75795103	G	A
NC_040263.1	75839511	C	G
NC_040263.1	75902176	A	G
NC_040263.1	75948525	C	T
NC_040263.1	75980697	G	A
NC_040263.1	75987367	T	C
NC_040263.1	76044489	C	T
NC_040263.1	76097701	A	G
NC_040263.1	76159013	G	A
NC_040263.1	76217666	G	A
NC_040263.1	76252201	C	G
NC_040263.1	76303558	A	G
NC_040263.1	76358639	T	A
NC_040263.1	76425439	T	C
NC_040263.1	76483762	A	G
NC_040263.1	76566799	A	G
NC_040263.1	76572518	C	A
NC_040263.1	76643458	G	A
NC_040263.1	76700695	C	T
NC_040263.1	76701235	T	C
NC_040263.1	76701561	C	T
NC_040263.1	76701800	G	A
NC_040263.1	76777870	G	T
NC_040263.1	76823213	T	C
NC_040263.1	76917891	G	A
NC_040263.1	76973997	T	A
NC_040263.1	76994247	A	G
NC_040263.1	76995770	G	T
NC_040263.1	77014066	G	A
NC_040263.1	77074839	T	C
NC_040263.1	77106034	T	C

NC_040263.1	77141965	C	T
NC_040263.1	77167037	T	C
NC_040263.1	77171020	T	C
NC_040263.1	77183968	T	C
NC_040263.1	77185378	T	A
NC_040263.1	77206396	T	C
NC_040263.1	78631716	A	C
NC_040263.1	78655675	T	C
NC_040263.1	78716412	C	T
NC_040263.1	78777363	A	G
NC_040263.1	78837917	C	T
NC_040263.1	78850645	C	T
NC_040263.1	78853372	T	C
NC_040263.1	78974440	A	G
NC_040263.1	78982814	G	A
NC_040263.1	79087892	T	C
NC_040263.1	79093665	T	C
NC_040263.1	79126339	T	C
NC_040263.1	79226241	T	C
NC_040263.1	79226498	G	A
NC_040263.1	79263812	T	C
NC_040263.1	79305912	C	A
NC_040263.1	79340578	C	T
NC_040263.1	79389981	G	A
NC_040263.1	79421437	A	C
NC_040263.1	79488657	G	A
NC_040263.1	79524240	G	A
NC_040263.1	79542605	T	C
NC_040263.1	79559502	T	C
NC_040263.1	79561357	T	C
NC_040263.1	79595132	G	C
NC_040263.1	79595520	C	T
NC_040263.1	79595859	T	A
NC_040263.1	79618358	T	C
NC_040263.1	79618618	T	C
NC_040263.1	79619409	T	C
NC_040263.1	79659642	C	T
NC_040263.1	79660585	C	T
NC_040263.1	79715378	G	A
NC_040263.1	79785712	T	C
NC_040263.1	79809643	A	C
NC_040263.1	79907289	A	T
NC_040263.1	79924186	A	G
NC_040263.1	80011963	A	G
NC_040263.1	80075445	A	G
NC_040263.1	80131515	T	A
NC_040263.1	80178697	C	T

NC_040263.1	80226447	T	C
NC_040263.1	80269619	G	C
NC_040263.1	80367604	G	A
NC_040263.1	80377926	C	T
NC_040263.1	80392707	A	G
NC_040263.1	80395045	A	G
NC_040263.1	80580955	A	G
NC_040263.1	80633950	T	C
NC_040263.1	80689109	C	T
NC_040263.1	80713762	C	T
NC_040263.1	80714290	A	G
NC_040263.1	80715769	T	A
NC_040263.1	80912945	C	G
NC_040263.1	81039785	C	T
NC_040263.1	81104960	C	T
NC_040263.1	81168716	A	G
NC_040263.1	82367414	T	C
NC_040263.1	82379425	A	G
NC_040263.1	82385191	A	G
NC_040263.1	82443040	G	T
NC_040263.1	82492155	T	C
NC_040263.1	82493172	C	T
NC_040263.1	82493666	A	G
NC_040263.1	82741553	A	G
NC_040263.1	82742134	G	T
NC_040263.1	82831729	T	C
NC_040263.1	82832383	A	G
NC_040263.1	82832547	A	G
NC_040263.1	82832746	T	C
NC_040263.1	82832932	T	C
NC_040263.1	82833517	A	G
NC_040263.1	82836182	A	G
NC_040263.1	82889675	T	C
NC_040263.1	82894707	G	A
NC_040263.1	82905772	A	G
NC_040263.1	82963854	C	T
NC_040263.1	83009966	A	G
NC_040263.1	83033759	A	G
NC_040263.1	83045491	T	C
NC_040263.1	83046078	C	T
NC_040263.1	83046419	G	C
NC_040263.1	83055733	A	G
NC_040263.1	83056994	G	A
NC_040263.1	83058037	A	G
NC_040263.1	83066227	A	G
NC_040263.1	83098813	A	G
NC_040263.1	83101029	C	T

NC_040263.1	83101927	G	C
NC_040263.1	83102094	T	C
NC_040263.1	83132261	A	G
NC_040263.1	83132415	T	C
NC_040263.1	83132678	A	G
NC_040263.1	83133147	A	G
NC_040263.1	83133480	T	C
NC_040263.1	83133808	C	G
NC_040263.1	83134459	T	C
NC_040263.1	83134997	C	G
NC_040263.1	83153785	A	T
NC_040263.1	83158178	A	G
NC_040263.1	83195557	T	C
NC_040263.1	83223254	A	G
NC_040263.1	83240881	T	C
NC_040263.1	83301357	C	T
NC_040263.1	83369656	G	A
NC_040263.1	83406803	C	G
NC_040263.1	83435040	A	G
NC_040263.1	83435691	A	C
NC_040263.1	83444077	C	A
NC_040263.1	83458781	G	A
NC_040263.1	83459022	T	C
NC_040263.1	83475850	A	G
NC_040263.1	83495957	C	T
NC_040263.1	83523124	A	G
NC_040263.1	83524074	C	A
NC_040263.1	83527663	T	C
NC_040263.1	83575717	T	C
NC_040263.1	83578690	T	C
NC_040263.1	83586890	T	C
NC_040263.1	83598326	G	A
NC_040263.1	83602121	A	G
NC_040263.1	83698161	T	G
NC_040263.1	83755601	C	T
NC_040263.1	83807190	T	C
NC_040263.1	83854661	C	T
NC_040263.1	83907322	A	C
NC_040263.1	83974631	C	T
NC_040263.1	83981544	A	G
NC_040263.1	83982021	A	G
NC_040263.1	83982323	A	G
NC_040263.1	83987359	T	C
NC_040263.1	83987725	G	T
NC_040263.1	83993734	A	G
NC_040263.1	83994423	T	C
NC_040263.1	83998756	T	C

NC_040263.1	83999134	T	G
NC_040263.1	83999309	A	G
NC_040263.1	84013015	A	C
NC_040263.1	84015985	G	A
NC_040263.1	84071981	T	C
NC_040263.1	84081593	G	A
NC_040263.1	84117871	C	T
NC_040263.1	84132972	G	A
NC_040263.1	84133748	G	C
NC_040263.1	84140999	T	C
NC_040263.1	84141470	A	G
NC_040263.1	84143697	A	G
NC_040263.1	84161626	A	G
NC_040263.1	84161861	A	G
NC_040263.1	84179144	C	T
NC_040263.1	84200589	A	C
NC_040263.1	84200923	T	C
NC_040263.1	84201378	T	C
NC_040263.1	84202490	T	G
NC_040263.1	84203199	A	G
NC_040263.1	84203426	G	A
NC_040263.1	84204084	A	C
NC_040263.1	84204239	A	G
NC_040263.1	84204500	T	C
NC_040263.1	84204810	T	C
NC_040263.1	84205060	A	G
NC_040263.1	84227412	A	G
NC_040263.1	84227583	T	C
NC_040263.1	84229106	C	G
NC_040263.1	84279725	G	A
NC_040263.1	84322191	T	C
NC_040263.1	84322371	C	G
NC_040263.1	84331734	A	G
NC_040263.1	84332585	T	C
NC_040263.1	84332904	A	G
NC_040263.1	84342945	A	G
NC_040263.1	84344056	G	A
NC_040263.1	84414746	T	C
NC_040263.1	84433386	T	C
NC_040263.1	84433851	C	G
NC_040263.1	84434053	A	G
NC_040263.1	84447704	C	A
NC_040263.1	84494191	G	A
NC_040263.1	84494392	T	C
NC_040264.1	213267	T	C
NC_040264.1	292516	G	T
NC_040264.1	295422	G	C

NC_040264.1	341352	A	T
NC_040264.1	623080	A	T
NC_040264.1	680127	C	T
NC_040264.1	729727	T	C
NC_040264.1	785767	A	G
NC_040264.1	842102	T	C
NC_040264.1	899847	C	T
NC_040264.1	952251	C	T
NC_040264.1	1002379	G	A
NC_040264.1	1059997	G	T
NC_040264.1	1102937	G	A
NC_040264.1	1159002	A	G
NC_040264.1	1218654	T	C
NC_040264.1	1270044	G	A
NC_040264.1	1377032	A	G
NC_040264.1	1434188	T	C
NC_040264.1	1436666	T	C
NC_040264.1	1681932	G	A
NC_040264.1	1737164	G	T
NC_040264.1	1793536	T	C
NC_040264.1	1853074	T	G
NC_040264.1	1912600	G	A
NC_040264.1	1957811	G	A
NC_040264.1	2004756	G	A
NC_040264.1	2069388	T	G
NC_040264.1	2203320	C	A
NC_040264.1	2244388	A	G
NC_040264.1	2244651	T	C
NC_040264.1	2294647	A	G
NC_040264.1	2338792	A	G
NC_040264.1	2402111	A	G
NC_040264.1	2465853	A	G
NC_040264.1	2540688	A	G
NC_040264.1	2587185	T	C
NC_040264.1	2643268	G	A
NC_040264.1	2680740	T	A
NC_040264.1	2713462	C	T
NC_040264.1	3018973	G	A
NC_040264.1	3024816	T	C
NC_040264.1	3025077	A	C
NC_040264.1	3028662	A	G
NC_040264.1	3030033	T	C
NC_040264.1	3032950	G	A
NC_040264.1	3033111	A	G
NC_040264.1	3033769	A	G
NC_040264.1	3033937	T	C
NC_040264.1	3034457	A	G

NC_040264.1	3223748	G	C
NC_040264.1	3276850	T	C
NC_040264.1	3297476	A	C
NC_040264.1	3309285	A	G
NC_040264.1	3440341	C	T
NC_040264.1	3447030	C	T
NC_040264.1	3451579	C	T
NC_040264.1	3512041	T	A
NC_040264.1	3567376	C	G
NC_040264.1	3623551	A	G
NC_040264.1	3678161	C	T
NC_040264.1	3680362	A	G
NC_040264.1	3703352	A	G
NC_040264.1	3725705	C	T
NC_040264.1	5358256	G	A
NC_040264.1	5360273	A	G
NC_040264.1	5367036	T	C
NC_040264.1	5367507	T	C
NC_040264.1	5369191	G	A
NC_040264.1	6543580	C	T
NC_040264.1	6581257	A	G
NC_040264.1	6616423	T	C
NC_040264.1	6807569	T	G
NC_040264.1	6839877	T	A
NC_040264.1	6940070	G	A
NC_040264.1	6998207	C	G
NC_040264.1	7102160	T	C
NC_040264.1	7162387	A	G
NC_040264.1	7223777	A	C
NC_040264.1	7279407	A	G
NC_040264.1	7339557	C	G
NC_040264.1	7423913	C	G
NC_040264.1	7474046	A	G
NC_040264.1	7478971	A	G
NC_040264.1	7483323	T	C
NC_040264.1	7537482	T	C
NC_040264.1	7587490	A	G
NC_040264.1	7664379	C	G
NC_040264.1	7720617	C	A
NC_040264.1	7773921	G	C
NC_040264.1	7829087	G	A
NC_040264.1	7879727	G	A
NC_040264.1	7933119	A	G
NC_040264.1	7979739	C	T
NC_040264.1	8019344	A	G
NC_040264.1	8020226	C	T
NC_040264.1	8020565	A	G

NC_040264.1	8022400	G	A
NC_040264.1	8022599	C	T
NC_040264.1	8065329	C	A
NC_040264.1	8121242	C	T
NC_040264.1	8172213	T	G
NC_040264.1	8240479	A	G
NC_040264.1	8297106	A	G
NC_040264.1	8338511	A	G
NC_040264.1	8401427	G	T
NC_040264.1	8463386	T	C
NC_040264.1	8551755	A	G
NC_040264.1	8608014	T	G
NC_040264.1	8656269	C	A
NC_040264.1	8712690	C	G
NC_040264.1	8775481	T	A
NC_040264.1	8834251	C	T
NC_040264.1	8888811	T	G
NC_040264.1	8922033	T	C
NC_040264.1	8972888	A	G
NC_040264.1	9029460	T	C
NC_040264.1	9089016	C	T
NC_040264.1	9130639	C	G
NC_040264.1	9178056	G	A
NC_040264.1	9231651	A	G
NC_040264.1	9261761	A	G
NC_040264.1	9319607	G	T
NC_040264.1	9371615	G	T
NC_040264.1	9572898	C	T
NC_040264.1	9630206	T	C
NC_040264.1	9663298	G	A
NC_040264.1	9820881	G	A
NC_040264.1	9871506	C	T
NC_040264.1	9933605	A	C
NC_040264.1	9987057	T	C
NC_040264.1	10042379	T	C
NC_040264.1	10099230	A	G
NC_040264.1	10151790	A	T
NC_040264.1	10195012	A	C
NC_040264.1	10196505	A	C
NC_040264.1	10197857	A	G
NC_040264.1	10486871	T	C
NC_040264.1	10487528	C	T
NC_040264.1	10487900	A	G
NC_040264.1	10536609	T	C
NC_040264.1	10586205	G	T
NC_040264.1	10586625	T	C
NC_040264.1	10617593	C	T

NC_040264.1	10669179	A	G
NC_040264.1	10722703	T	C
NC_040264.1	10781691	G	C
NC_040264.1	10973133	A	G
NC_040264.1	11064092	A	G
NC_040264.1	11073022	A	G
NC_040264.1	11076117	T	C
NC_040264.1	11156573	A	G
NC_040264.1	11200259	T	G
NC_040264.1	11283520	A	G
NC_040264.1	11313166	A	G
NC_040264.1	11317226	A	G
NC_040264.1	11319742	G	C
NC_040264.1	11329494	A	G
NC_040264.1	11365611	T	C
NC_040264.1	11383715	T	C
NC_040264.1	11425921	A	G
NC_040264.1	11516236	A	C
NC_040264.1	11566565	C	T
NC_040264.1	11589701	A	G
NC_040264.1	11854954	A	G
NC_040264.1	11865949	A	G
NC_040264.1	11929813	A	G
NC_040264.1	11946557	A	G
NC_040264.1	12388245	C	T
NC_040264.1	12391435	T	C
NC_040264.1	12448851	0	G
NC_040264.1	12511107	A	C
NC_040264.1	12567283	T	G
NC_040264.1	12674261	T	C
NC_040264.1	12721030	G	A
NC_040264.1	12751518	A	G
NC_040264.1	12794181	T	C
NC_040264.1	12797405	T	C
NC_040264.1	12815330	G	A
NC_040264.1	12815767	C	T
NC_040264.1	12830893	C	T
NC_040264.1	12845396	G	A
NC_040264.1	12901669	T	A
NC_040264.1	12945672	A	G
NC_040264.1	12977455	T	G
NC_040264.1	13036060	A	G
NC_040264.1	15706639	C	T
NC_040264.1	15758304	A	G
NC_040264.1	15802585	G	A
NC_040264.1	15855968	C	T
NC_040264.1	15904689	G	A

NC_040264.1	15933686	A	G
NC_040264.1	15934195	T	A
NC_040264.1	15985626	A	G
NC_040264.1	16042149	T	C
NC_040264.1	16095065	A	G
NC_040264.1	16151971	T	C
NC_040264.1	16206045	A	C
NC_040264.1	16257351	T	A
NC_040264.1	16258076	A	G
NC_040264.1	16259094	C	T
NC_040264.1	16260633	A	G
NC_040264.1	16366376	A	T
NC_040264.1	16367166	C	T
NC_040264.1	16378360	G	A
NC_040264.1	16381505	T	G
NC_040264.1	16437859	A	G
NC_040264.1	16487384	G	T
NC_040264.1	16504057	A	G
NC_040264.1	16602087	A	C
NC_040264.1	16613469	G	T
NC_040264.1	16614590	A	G
NC_040264.1	16615548	G	T
NC_040264.1	16631106	T	G
NC_040264.1	16686407	C	T
NC_040264.1	16741572	A	C
NC_040264.1	16770291	A	G
NC_040264.1	16778917	A	C
NC_040264.1	16827077	G	C
NC_040264.1	16855498	T	C
NC_040264.1	16870049	T	C
NC_040264.1	16925200	T	C
NC_040264.1	16980040	T	C
NC_040264.1	17063640	C	T
NC_040264.1	17119660	G	C
NC_040264.1	17175187	G	T
NC_040264.1	17226070	A	G
NC_040264.1	17285157	G	A
NC_040264.1	17322306	G	A
NC_040264.1	17372792	A	G
NC_040264.1	17422951	G	A
NC_040264.1	17444656	T	C
NC_040264.1	17447120	A	G
NC_040264.1	17451189	A	G
NC_040264.1	17451538	A	G
NC_040264.1	17452075	A	G
NC_040264.1	17544983	A	G
NC_040264.1	17593334	A	G

NC_040264.1	17622608	A	G
NC_040264.1	17705693	C	T
NC_040264.1	17738442	A	G
NC_040264.1	17899857	C	T
NC_040264.1	17915031	G	T
NC_040264.1	17917548	C	T
NC_040264.1	17930660	C	G
NC_040264.1	17945558	C	T
NC_040264.1	17954143	G	A
NC_040264.1	17954307	A	G
NC_040264.1	18014502	T	C
NC_040264.1	18069981	A	G
NC_040264.1	18097943	T	C
NC_040264.1	18098428	C	T
NC_040264.1	18099136	A	G
NC_040264.1	18139527	G	A
NC_040264.1	18143984	G	T
NC_040264.1	18145985	T	C
NC_040264.1	18163937	A	G
NC_040264.1	18198608	A	G
NC_040264.1	18203302	C	A
NC_040264.1	18205068	C	T
NC_040264.1	18209614	C	A
NC_040264.1	18274243	T	C
NC_040264.1	18274975	C	T
NC_040264.1	18275310	A	G
NC_040264.1	18330383	A	G
NC_040264.1	18389021	C	T
NC_040264.1	18390390	T	C
NC_040264.1	18390776	T	C
NC_040264.1	18407873	T	A
NC_040264.1	18408560	A	G
NC_040264.1	18408726	G	A
NC_040264.1	18463982	T	C
NC_040264.1	18519979	G	A
NC_040264.1	18566922	A	G
NC_040264.1	18719782	G	A
NC_040264.1	18756483	A	G
NC_040264.1	18803985	A	G
NC_040264.1	18853241	G	A
NC_040264.1	18909919	A	G
NC_040264.1	18964676	C	T
NC_040264.1	19019818	T	G
NC_040264.1	19075132	T	C
NC_040264.1	19105131	A	G
NC_040264.1	19130372	T	C
NC_040264.1	19185643	A	G

NC_040264.1	19240972	C	T
NC_040264.1	19294381	A	G
NC_040264.1	19343341	T	G
NC_040264.1	19343795	G	C
NC_040264.1	19921479	T	C
NC_040264.1	19977701	T	C
NC_040264.1	19984337	A	G
NC_040264.1	20042256	G	C
NC_040264.1	20065515	T	C
NC_040264.1	20066233	G	T
NC_040264.1	20066605	G	A
NC_040264.1	20250152	A	G
NC_040264.1	20292478	C	T
NC_040264.1	20353024	A	G
NC_040264.1	20406402	C	G
NC_040264.1	20444422	A	G
NC_040264.1	20493238	C	T
NC_040264.1	20547192	A	G
NC_040264.1	20605244	A	G
NC_040264.1	20605709	C	T
NC_040264.1	20606145	G	T
NC_040264.1	20607642	C	T
NC_040264.1	21768581	C	G
NC_040264.1	21837073	T	C
NC_040264.1	21884693	A	T
NC_040264.1	21924189	A	G
NC_040264.1	22039137	A	G
NC_040264.1	22063030	A	G
NC_040264.1	22187889	T	C
NC_040264.1	22244961	A	G
NC_040264.1	22299126	T	C
NC_040264.1	22350314	G	A
NC_040264.1	22403307	T	C
NC_040264.1	22471890	T	C
NC_040264.1	22525071	G	A
NC_040264.1	22565360	C	T
NC_040264.1	22924446	T	G
NC_040264.1	22925514	G	A
NC_040264.1	22926356	C	T
NC_040264.1	22926585	C	T
NC_040264.1	22926742	T	C
NC_040264.1	22929001	A	G
NC_040264.1	22929490	C	T
NC_040264.1	22972662	T	C
NC_040264.1	22991323	T	C
NC_040264.1	22993974	T	A
NC_040264.1	22996003	T	C

NC_040264.1	23049243	A	C
NC_040264.1	23102857	C	G
NC_040264.1	23157738	T	A
NC_040264.1	23214424	C	T
NC_040264.1	23263143	C	T
NC_040264.1	23281735	T	C
NC_040264.1	23310352	A	G
NC_040264.1	23568974	A	G
NC_040264.1	23569189	A	G
NC_040264.1	23573413	A	G
NC_040264.1	23574047	T	C
NC_040264.1	23628748	G	A
NC_040264.1	23683872	T	C
NC_040264.1	23737518	T	C
NC_040264.1	23772978	T	G
NC_040264.1	23802467	A	G
NC_040264.1	23802625	T	C
NC_040264.1	23814945	T	C
NC_040264.1	23815849	C	T
NC_040264.1	23816629	T	C
NC_040264.1	23818755	A	G
NC_040264.1	23869499	G	A
NC_040264.1	23923783	G	A
NC_040264.1	23973135	T	A
NC_040264.1	24005515	A	C
NC_040264.1	24281879	C	G
NC_040264.1	24295394	A	G
NC_040264.1	24327316	C	A
NC_040264.1	24353445	C	T
NC_040264.1	24472868	T	C
NC_040264.1	24473724	A	G
NC_040264.1	24528801	G	C
NC_040264.1	24585739	A	C
NC_040264.1	24626619	A	G
NC_040264.1	24654381	C	G
NC_040264.1	24798838	C	A
NC_040264.1	24837363	A	G
NC_040264.1	24861735	A	G
NC_040264.1	24900096	C	T
NC_040264.1	24959589	G	C
NC_040264.1	25038733	T	C
NC_040264.1	25039407	G	C
NC_040264.1	25039895	T	C
NC_040264.1	25061883	A	C
NC_040264.1	25065295	A	G
NC_040264.1	25097740	A	C
NC_040264.1	25136314	T	C

NC_040264.1	25202248	T	C
NC_040264.1	25203061	T	A
NC_040264.1	25203315	T	C
NC_040264.1	26014012	A	G
NC_040264.1	26068359	T	C
NC_040264.1	26125060	T	C
NC_040264.1	26166445	T	C
NC_040264.1	26211831	T	C
NC_040264.1	26251606	A	G
NC_040264.1	26252097	G	A
NC_040264.1	26252301	T	C
NC_040264.1	26288372	A	G
NC_040264.1	26334314	C	T
NC_040264.1	26352426	T	C
NC_040264.1	26358143	T	C
NC_040264.1	26359499	C	T
NC_040264.1	26360238	C	A
NC_040264.1	26360793	T	A
NC_040264.1	26412108	G	A
NC_040264.1	26413284	T	C
NC_040264.1	26413526	T	C
NC_040264.1	26429214	G	A
NC_040264.1	26442326	T	C
NC_040264.1	26442903	A	G
NC_040264.1	26443191	C	T
NC_040264.1	26487618	A	G
NC_040264.1	26535654	0	A
NC_040264.1	26557875	T	C
NC_040264.1	26572311	A	G
NC_040264.1	26652121	A	G
NC_040264.1	26652285	C	T
NC_040264.1	26652575	T	C
NC_040264.1	26652770	G	A
NC_040264.1	26709644	A	G
NC_040264.1	26748227	T	C
NC_040264.1	26783155	A	G
NC_040264.1	26824336	C	G
NC_040264.1	26824615	C	T
NC_040264.1	26825067	T	C
NC_040264.1	26825440	A	G
NC_040264.1	26827211	G	A
NC_040264.1	26933599	A	G
NC_040264.1	26933995	T	C
NC_040264.1	26934264	G	C
NC_040264.1	26934824	G	C
NC_040264.1	26986352	A	G
NC_040264.1	27043508	G	A

NC_040264.1	27102248	A	G
NC_040264.1	27153696	A	G
NC_040264.1	27191848	C	T
NC_040264.1	27233026	T	C
NC_040264.1	27234704	C	A
NC_040264.1	27236251	A	G
NC_040264.1	27236452	C	T
NC_040264.1	27478774	T	C
NC_040264.1	27521753	A	G
NC_040264.1	27575577	T	C
NC_040264.1	27630741	G	C
NC_040264.1	27683795	T	C
NC_040264.1	27728818	C	T
NC_040264.1	27753903	A	G
NC_040264.1	27754248	A	C
NC_040264.1	27755649	T	C
NC_040264.1	27755819	A	G
NC_040264.1	28819274	G	A
NC_040264.1	28858038	A	G
NC_040264.1	28877505	A	G
NC_040264.1	28877658	G	C
NC_040264.1	28883291	A	T
NC_040264.1	28886544	G	A
NC_040264.1	28891429	A	G
NC_040264.1	28893385	A	G
NC_040264.1	28966205	T	C
NC_040264.1	28993349	A	G
NC_040264.1	28996393	A	G
NC_040264.1	29008174	T	C
NC_040264.1	29123692	G	A
NC_040264.1	29124997	C	T
NC_040264.1	29126432	T	C
NC_040264.1	29128183	T	G
NC_040264.1	29128375	T	A
NC_040264.1	29183843	C	T
NC_040264.1	29205301	G	A
NC_040264.1	29205505	C	T
NC_040264.1	29271760	G	A
NC_040264.1	29296706	A	G
NC_040264.1	29324591	T	C
NC_040264.1	29381824	T	C
NC_040264.1	29434365	T	C
NC_040264.1	29489730	C	G
NC_040264.1	29545207	T	C
NC_040264.1	29596327	C	T
NC_040264.1	29661099	G	C
NC_040264.1	30267881	C	G

NC_040264.1	30268851	T	C
NC_040264.1	30280659	A	G
NC_040264.1	30338809	A	G
NC_040264.1	30641435	A	G
NC_040264.1	30654827	A	G
NC_040264.1	30659032	A	G
NC_040264.1	30660172	T	C
NC_040264.1	30661490	T	G
NC_040264.1	30661741	G	A
NC_040264.1	30663809	C	T
NC_040264.1	30664293	G	C
NC_040264.1	30681843	G	A
NC_040264.1	30683494	A	G
NC_040264.1	30707545	T	C
NC_040264.1	30713582	A	G
NC_040264.1	30716831	C	T
NC_040264.1	30717409	T	C
NC_040264.1	30736497	A	C
NC_040264.1	30778208	A	T
NC_040264.1	30820493	A	G
NC_040264.1	30852927	A	G
NC_040264.1	30909725	C	T
NC_040264.1	30958452	G	C
NC_040264.1	31009354	A	G
NC_040264.1	31062726	T	C
NC_040264.1	31085161	T	C
NC_040264.1	31841951	T	C
NC_040264.1	31887483	T	A
NC_040264.1	31916130	G	C
NC_040264.1	31917220	C	T
NC_040264.1	31918596	C	T
NC_040264.1	31918833	G	A
NC_040264.1	31982942	T	C
NC_040264.1	32038671	C	T
NC_040264.1	32093036	A	G
NC_040264.1	32132955	T	G
NC_040264.1	32188550	C	A
NC_040264.1	32246690	A	G
NC_040264.1	32298751	A	G
NC_040264.1	32315233	T	C
NC_040264.1	32333988	G	C
NC_040264.1	32380686	G	A
NC_040264.1	32414035	T	C
NC_040264.1	32454959	G	A
NC_040264.1	32504946	T	C
NC_040264.1	32508466	C	T
NC_040264.1	32532080	G	A

NC_040264.1	32595293	G	T
NC_040264.1	32692433	T	C
NC_040264.1	32748224	T	A
NC_040264.1	32793821	A	C
NC_040264.1	32817881	C	T
NC_040264.1	33026046	T	C
NC_040264.1	33072865	T	A
NC_040264.1	33103115	G	A
NC_040264.1	33103338	T	C
NC_040264.1	33170334	T	C
NC_040264.1	33213667	C	T
NC_040264.1	33238991	T	C
NC_040264.1	33245226	G	A
NC_040264.1	33250266	C	T
NC_040264.1	33272550	C	T
NC_040264.1	33330187	T	C
NC_040264.1	33380688	C	T
NC_040264.1	33402628	G	A
NC_040264.1	33511277	A	G
NC_040264.1	33523146	C	T
NC_040264.1	33543272	A	G
NC_040264.1	33596573	T	C
NC_040264.1	33637042	A	C
NC_040264.1	33675828	C	T
NC_040264.1	33795035	G	A
NC_040264.1	33856610	T	G
NC_040264.1	33915519	T	G
NC_040264.1	33965533	G	A
NC_040264.1	34012470	T	C
NC_040264.1	34049342	T	C
NC_040264.1	34049620	T	C
NC_040264.1	34090682	A	G
NC_040264.1	34129972	T	C
NC_040264.1	34183663	G	A
NC_040264.1	34236343	C	T
NC_040264.1	34279301	G	A
NC_040264.1	34378023	T	C
NC_040264.1	34403382	G	A
NC_040264.1	34419773	C	A
NC_040264.1	34485981	T	C
NC_040264.1	34496459	C	T
NC_040264.1	34549402	C	T
NC_040264.1	34593583	G	A
NC_040264.1	34835593	G	A
NC_040264.1	34836450	G	T
NC_040264.1	34891285	C	G
NC_040264.1	34948529	A	G

NC_040264.1	35003532	A	G
NC_040264.1	35034222	G	C
NC_040264.1	35325243	A	T
NC_040264.1	35363821	A	G
NC_040264.1	35425572	C	T
NC_040264.1	35494631	G	T
NC_040264.1	35628775	A	C
NC_040264.1	35668244	C	A
NC_040264.1	35703499	T	C
NC_040264.1	35759741	C	G
NC_040264.1	35801841	T	A
NC_040264.1	35814785	G	A
NC_040264.1	35889691	C	G
NC_040264.1	36173593	C	A
NC_040264.1	36175341	A	G
NC_040264.1	36176409	A	G
NC_040264.1	36204957	C	T
NC_040264.1	36379471	T	G
NC_040264.1	36440873	G	A
NC_040264.1	36441758	T	C
NC_040264.1	36443495	A	C
NC_040264.1	36464782	A	G
NC_040264.1	36625290	G	A
NC_040264.1	37176644	A	G
NC_040264.1	37219049	A	G
NC_040264.1	37220423	A	C
NC_040264.1	37277093	C	T
NC_040264.1	37297744	G	A
NC_040264.1	37464054	T	C
NC_040264.1	37522880	C	T
NC_040264.1	37580020	A	G
NC_040264.1	37635892	G	A
NC_040264.1	37696652	G	C
NC_040264.1	37760459	G	A
NC_040264.1	37814069	T	A
NC_040264.1	37840629	A	G
NC_040264.1	37896547	A	G
NC_040264.1	37922074	G	C
NC_040264.1	37997608	A	G
NC_040264.1	38060014	T	C
NC_040264.1	38166376	C	G
NC_040264.1	38168407	T	G
NC_040264.1	38191110	A	T
NC_040264.1	38306190	T	C
NC_040264.1	38328845	G	C
NC_040264.1	38741297	G	C
NC_040264.1	38796300	T	C

NC_040264.1	38851874	T	G
NC_040264.1	38906895	A	G
NC_040264.1	38975885	T	C
NC_040264.1	38998872	T	C
NC_040264.1	39200331	T	A
NC_040264.1	39252699	A	C
NC_040264.1	39321869	C	A
NC_040264.1	39354290	T	C
NC_040264.1	39394290	A	G
NC_040264.1	39444476	T	A
NC_040264.1	39588074	T	C
NC_040264.1	39597099	C	G
NC_040264.1	39638035	T	A
NC_040264.1	39697411	A	G
NC_040264.1	39725863	C	T
NC_040264.1	39726860	G	C
NC_040264.1	39728351	T	C
NC_040264.1	39789302	A	T
NC_040264.1	39867010	T	C
NC_040264.1	39895973	A	G
NC_040264.1	39911655	C	T
NC_040264.1	39933168	G	A
NC_040264.1	39943047	T	C
NC_040264.1	39996472	C	G
NC_040264.1	40037097	G	A
NC_040264.1	40249879	T	G
NC_040264.1	40306040	T	C
NC_040264.1	40356870	C	T
NC_040264.1	40410933	G	A
NC_040264.1	40465991	T	C
NC_040264.1	40522737	T	C
NC_040264.1	40577591	G	C
NC_040264.1	40633291	G	A
NC_040264.1	40674412	T	C
NC_040264.1	40702971	G	A
NC_040264.1	40745319	A	C
NC_040264.1	40805000	G	T
NC_040264.1	40844741	A	G
NC_040264.1	40906436	G	A
NC_040264.1	40907052	A	G
NC_040264.1	40935979	G	T
NC_040264.1	40951542	G	A
NC_040264.1	41007248	T	A
NC_040264.1	41061678	C	T
NC_040264.1	41119278	T	C
NC_040264.1	41170296	A	C
NC_040264.1	41216426	A	C

NC_040264.1	41271284	G	C
NC_040264.1	41327958	A	G
NC_040264.1	41387881	T	G
NC_040264.1	41443343	G	A
NC_040264.1	41507947	G	T
NC_040264.1	41818401	T	C
NC_040264.1	41871192	A	G
NC_040264.1	41901713	T	C
NC_040264.1	41965890	C	T
NC_040264.1	42010919	G	A
NC_040264.1	42237644	C	T
NC_040264.1	42239952	T	G
NC_040264.1	42245994	T	C
NC_040264.1	42968742	T	C
NC_040264.1	43272374	G	A
NC_040264.1	43275253	G	T
NC_040264.1	43299319	G	A
NC_040264.1	43438782	C	T
NC_040264.1	43449098	A	G
NC_040264.1	43449416	A	G
NC_040264.1	43454352	C	A
NC_040264.1	43457881	T	G
NC_040264.1	43506453	G	C
NC_040264.1	43520147	C	T
NC_040264.1	43542712	T	C
NC_040264.1	43583558	G	A
NC_040264.1	43922439	C	T
NC_040264.1	43976374	C	G
NC_040264.1	44016465	A	G
NC_040264.1	44085403	C	G
NC_040264.1	44090159	C	T
NC_040264.1	44130910	T	C
NC_040264.1	44184467	T	C
NC_040264.1	44187061	G	A
NC_040264.1	44238551	C	G
NC_040264.1	44296934	T	C
NC_040264.1	44298946	G	A
NC_040264.1	44362193	A	G
NC_040264.1	44369338	T	C
NC_040264.1	44414682	C	T
NC_040264.1	44450441	T	C
NC_040264.1	44451660	A	G
NC_040264.1	44452312	T	C
NC_040264.1	44453342	T	C
NC_040264.1	44455738	A	G
NC_040264.1	44476254	A	G
NC_040264.1	44505655	A	C

NC_040264.1	44537254	T	C
NC_040264.1	44634816	G	C
NC_040264.1	44704466	C	T
NC_040264.1	44781675	T	C
NC_040264.1	44876960	A	G
NC_040264.1	44912449	T	C
NC_040264.1	45055053	A	C
NC_040264.1	45102368	A	C
NC_040264.1	45147027	A	G
NC_040264.1	45230583	G	A
NC_040264.1	45291339	A	C
NC_040264.1	45332857	T	C
NC_040264.1	45371478	T	C
NC_040264.1	45415690	T	C
NC_040264.1	45451899	C	G
NC_040264.1	46154811	G	C
NC_040264.1	46159770	T	C
NC_040264.1	46161003	G	A
NC_040264.1	46162332	G	A
NC_040264.1	46647124	C	A
NC_040264.1	46698739	C	A
NC_040264.1	46722195	A	G
NC_040264.1	47630305	A	G
NC_040264.1	47682410	G	A
NC_040264.1	47739758	A	C
NC_040264.1	47781743	T	C
NC_040264.1	47811832	C	A
NC_040264.1	47878853	G	A
NC_040264.1	47900903	A	C
NC_040264.1	47902915	T	C
NC_040264.1	47910639	T	C
NC_040264.1	47915728	G	A
NC_040264.1	47936287	G	A
NC_040264.1	47959929	A	T
NC_040264.1	48032199	T	C
NC_040264.1	48044669	G	C
NC_040264.1	48051742	A	G
NC_040264.1	48079469	G	A
NC_040264.1	48088747	A	G
NC_040264.1	48141166	C	T
NC_040264.1	48197009	G	C
NC_040264.1	48251705	A	G
NC_040264.1	48308224	C	T
NC_040264.1	48354803	G	A
NC_040264.1	48382474	A	G
NC_040264.1	48439897	G	T
NC_040264.1	48461545	T	C

NC_040264.1	48527002	T	C
NC_040264.1	48661550	C	T
NC_040264.1	48702596	C	G
NC_040264.1	48747025	A	G
NC_040264.1	48835117	A	G
NC_040264.1	48876884	C	T
NC_040264.1	48918825	G	A
NC_040264.1	48950404	C	T
NC_040264.1	49060681	G	A
NC_040264.1	49063020	T	C
NC_040264.1	49064428	G	T
NC_040264.1	49068868	T	C
NC_040264.1	49114126	T	C
NC_040264.1	49203372	A	G
NC_040264.1	49621595	G	C
NC_040264.1	49626139	G	A
NC_040264.1	49679866	T	C
NC_040264.1	49680507	C	T
NC_040264.1	49835531	C	A
NC_040264.1	49902624	T	G
NC_040264.1	49924228	T	C
NC_040264.1	49973345	A	C
NC_040264.1	50015745	A	G
NC_040264.1	50040017	A	G
NC_040264.1	50192953	G	A
NC_040264.1	50212409	C	T
NC_040264.1	50214718	A	G
NC_040264.1	50215978	A	G
NC_040264.1	50267044	T	G
NC_040264.1	50313681	C	A
NC_040264.1	51168354	A	G
NC_040264.1	51170421	T	C
NC_040264.1	52690235	G	A
NC_040264.1	52704241	T	G
NC_040264.1	52748757	A	G
NC_040264.1	52976914	A	G
NC_040264.1	53012971	A	G
NC_040264.1	53022322	A	G
NC_040264.1	53047667	T	C
NC_040264.1	53232115	G	C
NC_040264.1	53288810	T	G
NC_040264.1	53336262	A	G
NC_040264.1	53406071	A	G
NC_040264.1	53443231	A	G
NC_040264.1	53477280	C	G
NC_040264.1	53478983	T	C
NC_040264.1	53480189	A	G

NC_040264.1	53501955	A	G
NC_040264.1	53553680	T	C
NC_040264.1	53556057	A	G
NC_040264.1	53570765	A	G
NC_040264.1	53575666	C	T
NC_040264.1	53579557	A	G
NC_040264.1	53582910	G	C
NC_040264.1	53599923	T	C
NC_040264.1	53601118	T	C
NC_040264.1	53641915	T	G
NC_040264.1	53684025	C	T
NC_040264.1	53737148	C	T
NC_040264.1	53782268	A	C
NC_040264.1	53935149	G	T
NC_040264.1	53977821	A	G
NC_040264.1	54015512	G	C
NC_040264.1	54062738	A	G
NC_040264.1	54110914	T	C
NC_040264.1	54128424	A	C
NC_040264.1	54129344	T	C
NC_040264.1	54132393	T	C
NC_040264.1	54134676	A	G
NC_040264.1	54147767	G	A
NC_040264.1	54188462	T	C
NC_040264.1	54232391	A	G
NC_040264.1	54235989	G	A
NC_040264.1	54237405	T	C
NC_040264.1	54290605	T	C
NC_040264.1	54332691	G	T
NC_040264.1	54379009	C	T
NC_040264.1	54422422	T	C
NC_040264.1	54741832	A	G
NC_040264.1	54745294	T	C
NC_040264.1	54748274	A	G
NC_040264.1	54798995	G	C
NC_040264.1	54893873	G	A
NC_040264.1	54944039	C	T
NC_040264.1	54998778	T	C
NC_040264.1	55030651	G	A
NC_040264.1	55120009	T	C
NC_040264.1	55149987	A	G
NC_040264.1	55308283	A	G
NC_040264.1	55344765	G	A
NC_040264.1	55510804	C	T
NC_040264.1	55550162	T	C
NC_040264.1	55904268	A	G
NC_040264.1	55913042	T	C

NC_040264.1	55959552	A	C
NC_040264.1	55980922	A	G
NC_040264.1	56034901	A	G
NC_040264.1	56073515	A	G
NC_040264.1	56073908	A	C
NC_040264.1	56074283	A	C
NC_040264.1	56078847	A	G
NC_040264.1	56079452	T	C
NC_040264.1	56079999	T	C
NC_040264.1	56083094	A	G
NC_040264.1	56086114	C	T
NC_040264.1	56086551	A	G
NC_040264.1	56087798	C	G
NC_040264.1	56088213	C	T
NC_040264.1	56089054	G	A
NC_040264.1	56094771	T	C
NC_040264.1	56095802	T	C
NC_040264.1	56130273	T	C
NC_040264.1	56150619	T	C
NC_040264.1	56156667	G	T
NC_040264.1	56162536	G	A
NC_040264.1	56186507	A	G
NC_040264.1	56187799	G	A
NC_040264.1	56191155	C	A
NC_040264.1	56191559	A	G
NC_040264.1	56200235	A	C
NC_040264.1	56206014	G	C
NC_040264.1	56207220	T	C
NC_040264.1	56208947	A	G
NC_040264.1	56255757	C	T
NC_040264.1	56258428	A	G
NC_040264.1	56299134	T	C
NC_040264.1	56327290	0	T
NC_040264.1	56329281	T	C
NC_040264.1	56334810	C	T
NC_040264.1	56335601	A	G
NC_040264.1	56353104	T	C
NC_040264.1	56354724	A	G
NC_040264.1	56358724	A	G
NC_040264.1	56368188	T	C
NC_040264.1	56385025	G	A
NC_040264.1	56387457	C	G
NC_040264.1	56398249	T	C
NC_040264.1	56400627	A	G
NC_040264.1	56401246	G	A
NC_040264.1	56408095	T	C
NC_040264.1	56424158	T	C

NC_040264.1	56425426	C	T
NC_040264.1	56427723	C	T
NC_040264.1	56435839	A	C
NC_040264.1	56436943	A	G
NC_040264.1	56438743	C	G
NC_040264.1	56490035	C	T
NC_040264.1	56508829	A	G
NC_040264.1	56517337	T	C
NC_040264.1	56519307	A	G
NC_040264.1	56520419	A	G
NC_040264.1	56576211	A	G
NC_040264.1	56597558	A	C
NC_040264.1	56622393	T	C
NC_040264.1	56640606	T	C
NC_040264.1	56644233	C	G
NC_040264.1	56644764	C	T
NC_040264.1	56809223	T	C
NC_040264.1	56826009	T	C
NC_040264.1	56826397	A	G
NC_040264.1	56831929	T	G
NC_040264.1	56853826	A	G
NC_040264.1	56855835	A	G
NC_040264.1	56878900	A	G
NC_040264.1	56904223	A	G
NC_040264.1	56908311	T	C
NC_040264.1	56926796	T	C
NC_040264.1	56935814	G	C
NC_040264.1	56967786	A	G
NC_040264.1	56971461	T	C
NC_040264.1	57027068	C	T
NC_040264.1	57050451	T	G
NC_040264.1	57105896	A	G
NC_040264.1	57157030	A	G
NC_040264.1	57164444	G	A
NC_040264.1	57203521	C	T
NC_040264.1	57220284	C	G
NC_040264.1	57221882	C	T
NC_040264.1	57268910	T	C
NC_040264.1	57274994	A	T
NC_040264.1	57276258	T	C
NC_040264.1	57280315	A	G
NC_040264.1	57288276	T	C
NC_040264.1	57290738	A	C
NC_040264.1	57291408	T	C
NC_040264.1	57292772	T	G
NC_040264.1	57309651	T	C
NC_040264.1	57360106	T	C

NC_040264.1	57369317	C	T
NC_040264.1	57370385	C	T
NC_040264.1	57371409	C	T
NC_040264.1	57378248	A	G
NC_040264.1	57379097	C	T
NC_040264.1	57387040	G	T
NC_040264.1	57412534	T	C
NC_040264.1	57467256	T	C
NC_040264.1	57512638	C	G
NC_040264.1	57525818	G	A
NC_040264.1	57528588	C	A
NC_040264.1	57529421	A	G
NC_040264.1	57574905	G	C
NC_040264.1	57628508	T	G
NC_040264.1	57654111	C	T
NC_040264.1	58896657	T	C
NC_040264.1	58914863	C	T
NC_040264.1	58958438	T	C
NC_040264.1	58963755	T	G
NC_040264.1	58964487	T	C
NC_040264.1	58965304	A	G
NC_040264.1	58966477	G	A
NC_040264.1	58989279	T	C
NC_040264.1	59055355	T	C
NC_040264.1	59055844	C	A
NC_040264.1	59095277	C	G
NC_040264.1	59153681	T	C
NC_040264.1	59200323	C	A
NC_040264.1	59242357	T	C
NC_040264.1	59425092	G	A
NC_040264.1	59448640	G	C
NC_040264.1	59552979	C	T
NC_040264.1	59560706	G	A
NC_040264.1	59561314	G	A
NC_040264.1	59561888	C	A
NC_040264.1	59700671	T	C
NC_040264.1	59704779	T	A
NC_040264.1	59712502	C	A
NC_040264.1	59712949	G	A
NC_040264.1	59739050	C	T
NC_040264.1	59818023	A	G
NC_040264.1	59868696	T	C
NC_040264.1	59881611	G	A
NC_040264.1	60017832	A	G
NC_040264.1	60045791	A	G
NC_040264.1	60070406	G	A
NC_040264.1	60207323	T	C

NC_040264.1	60271398	C	G
NC_040264.1	60315650	C	T
NC_040264.1	60341060	A	G
NC_040264.1	60342440	G	A
NC_040264.1	60343600	T	G
NC_040264.1	60344083	T	C
NC_040264.1	60345362	T	G
NC_040264.1	60391208	A	G
NC_040264.1	60477042	A	G
NC_040264.1	60814320	T	C
NC_040264.1	60867906	A	G
NC_040264.1	60869620	T	A
NC_040264.1	60893300	G	T
NC_040264.1	60936465	T	C
NC_040264.1	60984669	A	G
NC_040264.1	61086567	C	G
NC_040264.1	61117120	G	A
NC_040264.1	61150346	T	A
NC_040264.1	61208297	C	G
NC_040264.1	61250320	A	G
NC_040264.1	61284681	C	T
NC_040264.1	61689991	G	A
NC_040264.1	61768232	A	G
NC_040264.1	61784123	T	C
NC_040264.1	61785757	T	G
NC_040264.1	61786607	G	C
NC_040264.1	61789578	A	G
NC_040264.1	61822485	T	C
NC_040264.1	61827615	T	C
NC_040264.1	61856479	C	G
NC_040264.1	61863187	T	C
NC_040264.1	61864598	T	C
NC_040264.1	61869320	G	C
NC_040264.1	62003573	G	A
NC_040264.1	62018734	G	A
NC_040264.1	62019916	C	T
NC_040264.1	62022645	A	G
NC_040264.1	62023617	T	C
NC_040264.1	62047883	A	G
NC_040264.1	62054251	C	G
NC_040264.1	62089725	G	A
NC_040264.1	62132346	T	C
NC_040264.1	62135784	T	C
NC_040264.1	62137705	T	C
NC_040264.1	62139641	A	G
NC_040264.1	62146268	0	C
NC_040264.1	62192086	T	C

NC_040264.1	62224724	A	G
NC_040264.1	62271130	A	G
NC_040264.1	62273313	T	C
NC_040264.1	62274337	T	A
NC_040264.1	62275815	C	A
NC_040264.1	62276444	C	G
NC_040264.1	62277282	A	T
NC_040264.1	62327700	T	C
NC_040264.1	62446131	T	G
NC_040264.1	62474816	C	T
NC_040264.1	62521160	T	C
NC_040264.1	62553777	A	G
NC_040264.1	62564267	C	T
NC_040264.1	62565261	A	G
NC_040264.1	62590120	G	A
NC_040264.1	62633627	G	C
NC_040264.1	62635463	T	C
NC_040264.1	62710466	A	C
NC_040264.1	62720521	C	T
NC_040264.1	62787274	T	C
NC_040264.1	62841329	C	T
NC_040264.1	62896587	G	A
NC_040264.1	62911399	T	C
NC_040264.1	62965035	C	T
NC_040264.1	63001601	T	G
NC_040264.1	63004872	G	A
NC_040264.1	63024093	0	G
NC_040264.1	63043525	G	C
NC_040264.1	63046226	T	C
NC_040264.1	63047043	G	C
NC_040264.1	63047691	A	G
NC_040264.1	63068702	T	C
NC_040264.1	63134682	T	G
NC_040264.1	63209287	A	G
NC_040264.1	63256954	T	C
NC_040264.1	63318607	T	A
NC_040264.1	63340320	C	T
NC_040264.1	63413934	T	G
NC_040264.1	63454980	A	C
NC_040264.1	63484989	A	G
NC_040264.1	63541178	A	G
NC_040264.1	63544082	T	C
NC_040264.1	63611152	T	A
NC_040264.1	63663621	T	C
NC_040264.1	63680651	G	A
NC_040264.1	63682830	G	A
NC_040264.1	63697240	A	G

NC_040264.1	63709984	A	G
NC_040264.1	63719940	A	G
NC_040264.1	63720460	A	C
NC_040264.1	63723419	G	A
NC_040264.1	63733085	T	G
NC_040264.1	63753224	T	C
NC_040264.1	63766038	A	G
NC_040264.1	63825663	C	G
NC_040264.1	63850965	T	C
NC_040264.1	63875263	T	C
NC_040264.1	63929843	T	C
NC_040264.1	63972129	C	T
NC_040264.1	64010534	G	A
NC_040264.1	64011403	A	T
NC_040264.1	64020395	T	C
NC_040264.1	64021241	G	A
NC_040264.1	64025096	A	C
NC_040264.1	64026997	C	G
NC_040264.1	64045346	G	C
NC_040264.1	64052501	T	G
NC_040264.1	64054131	A	G
NC_040264.1	64101732	G	A
NC_040264.1	64102553	A	G
NC_040264.1	64117677	T	C
NC_040264.1	64119419	C	T
NC_040264.1	64140640	A	G
NC_040264.1	64200477	A	G
NC_040264.1	64202938	A	G
NC_040264.1	64205175	A	G
NC_040264.1	64212429	T	C
NC_040264.1	64269388	G	C
NC_040264.1	64337123	A	G
NC_040264.1	64441003	T	C
NC_040264.1	64505453	T	C
NC_040264.1	64563699	A	G
NC_040264.1	64616555	C	T
NC_040264.1	64671424	C	T
NC_040264.1	64730171	G	C
NC_040264.1	65386590	T	C
NC_040264.1	65450710	C	T
NC_040264.1	65464216	C	G
NC_040264.1	65464610	G	A
NC_040264.1	65496319	T	C
NC_040264.1	65502123	A	G
NC_040264.1	65511447	A	G
NC_040264.1	65566115	C	T
NC_040264.1	65568504	A	C

NC_040264.1	65574809	A	G
NC_040264.1	65583644	G	C
NC_040264.1	65604663	T	C
NC_040264.1	65624233	A	G
NC_040264.1	65643370	A	G
NC_040264.1	65699935	A	G
NC_040264.1	65753720	A	G
NC_040264.1	65795488	T	G
NC_040264.1	65833141	T	C
NC_040264.1	65869187	A	G
NC_040264.1	65926803	G	A
NC_040264.1	65952102	T	C
NC_040264.1	66011474	A	G
NC_040264.1	66119028	C	G
NC_040264.1	66175876	A	G
NC_040264.1	66231044	T	G
NC_040264.1	66450509	G	A
NC_040264.1	66495486	G	A
NC_040264.1	66589842	A	G
NC_040264.1	66632285	T	C
NC_040264.1	66686878	C	T
NC_040264.1	66689182	G	A
NC_040264.1	66710545	T	C
NC_040264.1	66727214	A	G
NC_040264.1	66782582	G	A
NC_040264.1	66813727	T	C
NC_040264.1	66848039	C	G
NC_040264.1	66853870	T	C
NC_040264.1	66855451	C	T
NC_040264.1	66856394	C	T
NC_040264.1	66877135	A	G
NC_040264.1	66884958	G	A
NC_040264.1	66948211	C	T
NC_040264.1	66988812	C	T
NC_040264.1	67019254	A	G
NC_040264.1	67030784	T	C
NC_040264.1	67079077	A	G
NC_040264.1	67086177	A	G
NC_040264.1	67091339	A	G
NC_040264.1	67095611	T	C
NC_040264.1	67148536	T	A
NC_040264.1	67196801	A	C
NC_040264.1	67249655	A	G
NC_040264.1	67304770	A	G
NC_040264.1	67338404	G	A
NC_040264.1	67339907	T	C
NC_040264.1	67341312	G	C

NC_040264.1	67351133	A	G
NC_040264.1	67356375	T	C
NC_040264.1	67406562	A	G
NC_040264.1	67451132	T	C
NC_040264.1	67494662	A	C
NC_040264.1	67533638	C	G
NC_040264.1	67534199	T	C
NC_040264.1	67535585	A	C
NC_040264.1	67543266	C	T
NC_040264.1	67593172	T	C
NC_040264.1	67621193	A	G
NC_040264.1	67634231	C	G
NC_040264.1	67683125	T	C
NC_040264.1	67703957	C	A
NC_040264.1	67763674	A	G
NC_040264.1	67801858	C	A
NC_040264.1	67841894	A	G
NC_040264.1	67873777	A	G
NC_040264.1	67886958	A	G
NC_040264.1	67952969	T	C
NC_040264.1	68037424	G	A
NC_040264.1	68093044	A	G
NC_040264.1	68127602	T	C
NC_040264.1	68131545	C	G
NC_040264.1	68177187	G	A
NC_040264.1	68301081	C	T
NC_040264.1	68338769	C	T
NC_040264.1	68361927	C	T
NC_040264.1	68395447	A	G
NC_040264.1	68425194	A	G
NC_040264.1	68477411	A	G
NC_040264.1	68530229	T	G
NC_040264.1	68580430	T	C
NC_040264.1	68618449	T	C
NC_040264.1	68618764	T	C
NC_040264.1	68626609	C	G
NC_040264.1	68628288	A	C
NC_040264.1	68629027	C	T
NC_040264.1	68630012	A	G
NC_040264.1	68636609	C	G
NC_040264.1	68688501	C	A
NC_040264.1	68719695	C	A
NC_040264.1	68772993	G	C
NC_040264.1	68831435	A	T
NC_040264.1	68888944	G	A
NC_040264.1	68936444	C	T
NC_040264.1	68976885	G	A

NC_040264.1	68998393	G	A
NC_040264.1	69053521	G	A
NC_040264.1	69099791	T	C
NC_040264.1	69134772	C	T
NC_040264.1	69248039	A	G
NC_040264.1	69248772	A	G
NC_040264.1	69251056	A	G
NC_040264.1	69255267	T	C
NC_040264.1	69256681	G	C
NC_040264.1	69257700	A	G
NC_040264.1	69413899	G	A
NC_040264.1	69463409	T	C
NC_040264.1	69510148	T	C
NC_040264.1	69551121	C	G
NC_040264.1	69608148	T	C
NC_040264.1	69670207	T	A
NC_040264.1	69694658	A	G
NC_040264.1	69696623	T	C
NC_040264.1	69761987	T	C
NC_040264.1	69805923	T	C
NC_040264.1	69835538	T	C
NC_040264.1	69885278	A	G
NC_040264.1	69894243	C	G
NC_040264.1	69921192	C	T
NC_040264.1	69973259	A	G
NC_040264.1	70037248	G	A
NC_040264.1	70116054	A	C
NC_040264.1	70117095	T	C
NC_040264.1	70177939	T	C
NC_040264.1	70228140	C	T
NC_040264.1	70248500	G	A
NC_040264.1	70250368	C	T
NC_040264.1	70252863	A	G
NC_040264.1	70263357	C	T
NC_040264.1	70270347	T	C
NC_040264.1	70270798	T	C
NC_040264.1	70271544	G	T
NC_040264.1	70272108	T	G
NC_040264.1	70275211	A	G
NC_040264.1	70366855	A	G
NC_040264.1	70381201	A	G
NC_040264.1	70431152	T	A
NC_040264.1	70481155	C	A
NC_040264.1	70527302	G	A
NC_040264.1	70563480	C	G
NC_040264.1	70602956	C	T
NC_040264.1	70629133	T	G

NC_040264.1	72244273	G	A
NC_040264.1	72557032	G	A
NC_040264.1	72609552	G	T
NC_040264.1	72661916	C	G
NC_040264.1	72681437	T	C
NC_040264.1	72684137	A	G
NC_040264.1	72685953	G	A
NC_040264.1	72690747	A	G
NC_040264.1	72691340	G	C
NC_040264.1	72692485	T	C
NC_040264.1	72695271	T	C
NC_040264.1	72750654	T	C
NC_040264.1	72799375	C	T
NC_040264.1	72840507	A	G
NC_040264.1	72841335	T	C
NC_040264.1	72844104	A	C
NC_040264.1	72881475	C	T
NC_040264.1	72888190	A	G
NC_040264.1	72899115	C	T
NC_040264.1	72950742	T	C
NC_040264.1	73003075	A	C
NC_040264.1	73045913	T	C
NC_040264.1	73084347	T	C
NC_040264.1	73711273	G	T
NC_040264.1	73767358	T	C
NC_040264.1	73819864	C	G
NC_040264.1	73877336	G	A
NC_040264.1	73966033	C	A
NC_040264.1	74010401	T	C
NC_040264.1	74068143	C	G
NC_040264.1	74088680	A	G
NC_040264.1	74140396	C	T
NC_040264.1	74341253	A	T
NC_040264.1	74398161	T	C
NC_040264.1	74450244	T	C
NC_040264.1	74485076	G	A
NC_040264.1	74517121	T	C
NC_040264.1	74579983	A	C
NC_040264.1	75240493	C	G
NC_040264.1	75244516	A	G
NC_040264.1	75245652	A	G
NC_040264.1	75283121	T	C
NC_040264.1	75341717	G	T
NC_040264.1	75381570	C	T
NC_040264.1	75390734	A	G
NC_040264.1	75404002	T	G
NC_040264.1	75543115	G	T

NC_040264.1	75582404	G	A
NC_040264.1	75650828	A	G
NC_040264.1	75713355	A	G
NC_040264.1	75759288	C	G
NC_040264.1	75796295	A	G
NC_040264.1	75805590	C	A
NC_040264.1	75841057	A	G
NC_040264.1	75869631	A	G
NC_040264.1	75874255	T	G
NC_040264.1	75898299	T	C
NC_040264.1	75950866	T	C
NC_040264.1	75976000	T	C
NC_040264.1	75976636	T	C
NC_040264.1	76026626	C	T
NC_040264.1	76043134	A	G
NC_040264.1	76091421	C	T
NC_040264.1	76145423	G	A
NC_040264.1	76180044	G	A
NC_040264.1	76240988	T	C
NC_040264.1	76275485	T	C
NC_040264.1	76281021	A	T
NC_040264.1	76347627	A	G
NC_040264.1	76410215	T	G
NC_040264.1	76453821	G	C
NC_040264.1	76507672	T	C
NC_040264.1	76558836	G	A
NC_040264.1	76593024	G	A
NC_040264.1	76612573	C	A
NC_040264.1	76866862	G	A
NC_040264.1	76875244	T	C
NC_040264.1	76880767	C	T
NC_040264.1	76899744	A	G
NC_040264.1	76932653	C	T
NC_040264.1	76966420	A	G
NC_040264.1	76977557	G	A
NC_040264.1	77035388	A	T
NC_040264.1	77259909	A	T
NC_040264.1	77418922	A	G
NC_040264.1	77496822	G	A
NC_040264.1	77679595	C	T
NC_040264.1	77742818	C	A
NC_040264.1	77775429	A	G
NC_040264.1	77810844	G	A
NC_040264.1	77841162	A	G
NC_040264.1	77918377	T	C
NC_040264.1	77922013	T	C
NC_040264.1	77967456	T	C

NC_040264.1	77986859	T	C
NC_040264.1	78002854	A	G
NC_040264.1	78003854	C	T
NC_040264.1	78007037	A	G
NC_040264.1	78013057	T	C
NC_040264.1	78030013	A	G
NC_040264.1	78030197	A	G
NC_040264.1	78058541	A	G
NC_040264.1	78059942	C	T
NC_040264.1	78061321	T	G
NC_040264.1	78061571	C	T
NC_040264.1	78073433	A	G
NC_040264.1	78079371	A	G
NC_040264.1	78082673	G	A
NC_040264.1	78083322	T	C
NC_040264.1	78112996	C	T
NC_040264.1	78115411	C	G
NC_040264.1	78156383	T	C
NC_040264.1	78158382	C	G
NC_040264.1	78158689	T	C
NC_040264.1	78161526	A	G
NC_040264.1	78161699	C	A
NC_040264.1	78162209	T	C
NC_040264.1	78162464	A	G
NC_040264.1	78168057	G	T
NC_040264.1	78173003	G	A
NC_040264.1	78173347	G	C
NC_040264.1	78175218	G	A
NC_040264.1	78176927	G	T
NC_040264.1	78177621	C	T
NC_040264.1	78179001	G	A
NC_040264.1	78211281	G	T
NC_040264.1	78265841	T	C
NC_040264.1	78269984	T	C
NC_040264.1	78318850	T	G
NC_040264.1	78348512	C	G
NC_040264.1	78448255	T	C
NC_040264.1	78498204	C	T
NC_040264.1	78614779	G	A
NC_040264.1	78622052	T	C
NC_040264.1	78622207	A	G
NC_040264.1	78676415	A	C
NC_040264.1	78703799	C	G
NC_040264.1	78739664	A	T
NC_040264.1	78755612	G	A
NC_040264.1	78881438	G	C
NC_040264.1	78938684	T	C

NC_040264.1	78994334	A	G
NC_040264.1	79051264	A	G
NC_040264.1	79098076	C	T
NC_040264.1	79155409	G	A
NC_040264.1	79209015	A	G
NC_040264.1	79269969	G	A
NC_040264.1	79270219	C	G
NC_040264.1	79271873	G	A
NC_040264.1	79272248	T	A
NC_040264.1	79273350	G	T
NC_040264.1	79410777	G	A
NC_040264.1	79467590	A	G
NC_040264.1	79521983	G	A
NC_040264.1	79534150	G	A
NC_040264.1	79537690	T	C
NC_040264.1	79539770	A	G
NC_040264.1	79596766	A	G
NC_040264.1	79662128	C	G
NC_040264.1	80231836	T	C
NC_040264.1	80240411	A	G
NC_040264.1	80298131	T	C
NC_040264.1	80354425	G	A
NC_040264.1	80402226	A	G
NC_040264.1	80485067	T	C
NC_040264.1	80506159	T	C
NC_040264.1	80531740	T	C
NC_040264.1	80551460	T	C
NC_040264.1	80564799	C	T
NC_040264.1	80567292	A	T
NC_040264.1	80599814	C	T
NC_040264.1	80623889	T	C
NC_040264.1	80661205	T	C
NC_040264.1	80681664	G	T
NC_040264.1	80718036	T	C
NC_040264.1	80721825	A	G
NC_040264.1	80724620	G	A
NC_040264.1	80728316	C	T
NC_040264.1	80730896	G	A
NC_040264.1	80736049	T	G
NC_040264.1	80736321	G	A
NC_040264.1	80737274	T	C
NC_040264.1	80737567	G	A
NC_040264.1	80745133	A	G
NC_040264.1	80746517	T	C
NC_040264.1	80749037	A	G
NC_040264.1	80753936	T	C
NC_040264.1	80754276	T	C

NC_040264.1	80754569	C		T
NC_040264.1	80845512	A		G
NC_040264.1	80846723	C		T
NC_040264.1	80847792	C		A
NC_040264.1	80847968	T		A
NC_040264.1	80849494	T		C
NC_040264.1	80853092	A		G
NC_040264.1	80853484	A		C
NC_040264.1	80854803	T		C
NC_040264.1	80907712	T		C
NC_040264.1	80972766	T		G
NC_040264.1	80974653	T		C
NC_040264.1	80974917	T		C
NC_040264.1	80975171	T		C
NC_040264.1	80989557	C		T
NC_040264.1	81034211	A		G
NC_040264.1	81162574	A		G
NC_040264.1	81163654	G		C
NC_040264.1	81163905	A		G
NC_040264.1	81173833	A		G
NC_040264.1	81233825	T		A
NC_040264.1	81321512	C		G
NC_040264.1	81384689	A		G
NC_040264.1	81391127	T		C
NC_040264.1	81392126	G		A
NC_040264.1	81392673	C		A
NC_040264.1	81405234	T		C
NC_040264.1	81435974	T		C
NC_040264.1	81451550	T		G
NC_040264.1	81532707	T		C
NC_040264.1	81557945	A		G
NC_040264.1	81595950		0	G
NC_040264.1	81625190	G		C
NC_040264.1	81626010		0	G
NC_040264.1	81935292	A		C
NC_040264.1	81977713	A		G
NC_040264.1	81993620	T		C
NC_040264.1	81997035	A		G
NC_040264.1	82010394	T		C
NC_040264.1	82010568	A		G
NC_040264.1	82012375	G		A
NC_040264.1	82029148	T		C
NC_040264.1	82073762	C		T
NC_040264.1	82110778	A		G
NC_040264.1	82121817	G		T
NC_040264.1	82122146	T		C
NC_040264.1	82122389	G		C

NC_040264.1	82178744	C	T
NC_040264.1	82209636	G	A
NC_040264.1	82218821	C	T
NC_040264.1	82219508	T	C
NC_040264.1	82222413	A	G
NC_040264.1	82223258	T	C
NC_040264.1	82246409	C	G
NC_040264.1	82246570	T	C
NC_040264.1	82271164	G	C
NC_040264.1	82331024	G	A
NC_040264.1	82668540	C	T
NC_040264.1	82671779	G	A
NC_040264.1	82710111	T	C
NC_040264.1	82758149	G	T
NC_040264.1	82787965	C	G
NC_040264.1	82788658	A	G
NC_040264.1	82852438	T	A
NC_040264.1	82853212	T	C
NC_040264.1	82854877	A	G
NC_040264.1	82910825	A	G
NC_040264.1	82946463	T	C
NC_040264.1	82994698	A	G
NC_040264.1	82995028	G	C
NC_040264.1	82995247	T	C
NC_040264.1	83256625	A	G
NC_040264.1	83305643	T	C
NC_040264.1	84021807	G	T
NC_040264.1	84075174	C	T
NC_040264.1	84128231	A	G
NC_040264.1	84184538	A	G
NC_040264.1	84239216	G	T
NC_040264.1	84280855	T	C
NC_040264.1	84309734	T	C
NC_040264.1	84311679	G	A
NC_040264.1	84353812	A	C
NC_040264.1	84386077	C	T
NC_040264.1	84443944	T	C
NC_040264.1	84506678	C	T
NC_040264.1	84506997	T	G
NC_040264.1	84507155	A	G
NC_040264.1	84508064	A	G
NC_040264.1	84508511	T	C
NC_040264.1	84508683	C	T
NC_040264.1	84569944	G	A
NC_040264.1	84573541	A	G
NC_040264.1	84578494	T	C
NC_040264.1	84578707	G	C

NC_040264.1	84578878	A	G
NC_040264.1	84579037	A	G
NC_040264.1	84880090	A	C
NC_040264.1	84894820	C	T
NC_040264.1	84945992	T	C
NC_040264.1	84976104	G	A
NC_040264.1	85059850	A	G
NC_040264.1	85071821	C	T
NC_040264.1	85105301	T	C
NC_040264.1	85397470	T	C
NC_040264.1	85425916	A	G
NC_040264.1	85472377	C	A
NC_040264.1	86749154	T	C
NC_040264.1	86755783	0	G
NC_040265.1	829531	G	A
NC_040265.1	948962	T	C
NC_040265.1	1019007	A	G
NC_040265.1	1019438	T	C
NC_040265.1	1026535	T	C
NC_040265.1	1036745	T	C
NC_040265.1	1037395	T	C
NC_040265.1	1037557	A	C
NC_040265.1	1038798	T	C
NC_040265.1	1039134	T	C
NC_040265.1	1103591	A	G
NC_040265.1	1103860	G	T
NC_040265.1	1104912	C	G
NC_040265.1	1137080	C	A
NC_040265.1	1143751	A	G
NC_040265.1	1152174	A	G
NC_040265.1	1155326	G	A
NC_040265.1	1171077	T	C
NC_040265.1	1174426	G	A
NC_040265.1	1182033	A	G
NC_040265.1	1238515	G	C
NC_040265.1	1252087	G	A
NC_040265.1	1288669	T	C
NC_040265.1	1289544	T	G
NC_040265.1	1326430	A	G
NC_040265.1	1341020	T	C
NC_040265.1	1402996	G	A
NC_040265.1	1404726	T	C
NC_040265.1	1463087	A	C
NC_040265.1	1479111	A	C
NC_040265.1	1493411	T	C
NC_040265.1	1513508	T	C
NC_040265.1	1599349	C	G

NC_040265.1	1599877	A	G
NC_040265.1	1600622	C	T
NC_040265.1	1607087	A	G
NC_040265.1	1615749	A	G
NC_040265.1	1631094	C	T
NC_040265.1	1635595	A	G
NC_040265.1	1658962	T	C
NC_040265.1	1669092	C	G
NC_040265.1	1669948	A	C
NC_040265.1	1786353	A	G
NC_040265.1	1796240	C	T
NC_040265.1	1811849	G	A
NC_040265.1	1903061	T	C
NC_040265.1	1990100	G	A
NC_040265.1	1990358	A	G
NC_040265.1	1993262	C	T
NC_040265.1	1993430	G	C
NC_040265.1	1994739	T	G
NC_040265.1	2041714	T	A
NC_040265.1	2043706	G	A
NC_040265.1	2121593	G	C
NC_040265.1	2122486	C	T
NC_040265.1	2291790	A	G
NC_040265.1	2344472	C	T
NC_040265.1	2366331	A	G
NC_040265.1	2385817	G	T
NC_040265.1	2460500	A	G
NC_040265.1	2461713	G	A
NC_040265.1	2466257	G	A
NC_040265.1	2467736	A	T
NC_040265.1	2490841	T	C
NC_040265.1	3075768	T	C
NC_040265.1	3315962	G	A
NC_040265.1	3346988	A	G
NC_040265.1	4290984	T	G
NC_040265.1	4291449	G	A
NC_040265.1	4409881	G	A
NC_040265.1	4429834	T	C
NC_040265.1	4484610	A	G
NC_040265.1	4875703	A	T
NC_040265.1	4929007	C	T
NC_040265.1	5172297	C	G
NC_040265.1	5663229	A	G
NC_040265.1	6725887	A	G
NC_040265.1	7526393	T	G
NC_040265.1	7580287	T	A
NC_040265.1	7582232	A	G

NC_040265.1	7582386	A	G
NC_040265.1	7583342	T	C
NC_040265.1	7584303	T	C
NC_040265.1	7604573	T	C
NC_040265.1	7636495	T	C
NC_040265.1	7876053	A	G
NC_040265.1	7942125	A	G
NC_040265.1	7945595	A	G
NC_040265.1	7983755	A	G
NC_040265.1	7992277	A	G
NC_040265.1	8004979	T	C
NC_040265.1	8027335	T	C
NC_040265.1	8033098	A	G
NC_040265.1	8049857	C	G
NC_040265.1	8178317	T	C
NC_040265.1	8179721	T	C
NC_040265.1	8187853	G	A
NC_040265.1	8241764	G	C
NC_040265.1	8395745	T	C
NC_040265.1	8423271	G	A
NC_040265.1	8512088	A	G
NC_040265.1	8811121	A	G
NC_040265.1	8956584	T	C
NC_040265.1	8958165	T	C
NC_040265.1	9046261	G	A
NC_040265.1	9048834	T	C
NC_040265.1	9193405	T	C
NC_040265.1	9234502	C	T
NC_040265.1	9653672	T	C
NC_040265.1	9821725	G	A
NC_040265.1	9934911	G	A
NC_040265.1	10166046	C	T
NC_040265.1	10251395	A	G
NC_040265.1	10837390	T	C
NC_040265.1	10911460	G	A
NC_040265.1	10911685	T	C
NC_040265.1	10967846	A	C
NC_040265.1	10973946	T	C
NC_040265.1	10974585	T	C
NC_040265.1	11005472	T	C
NC_040265.1	11007697	A	G
NC_040265.1	11053244	T	C
NC_040265.1	11068586	T	C
NC_040265.1	11072092	T	C
NC_040265.1	11074110	A	G
NC_040265.1	11074527	T	C
NC_040265.1	11083613	A	G

NC_040265.1	11096795	A	T
NC_040265.1	11107819	A	G
NC_040265.1	11108151	G	A
NC_040265.1	11108991	A	G
NC_040265.1	11110133	A	G
NC_040265.1	11110308	C	T
NC_040265.1	11123028	C	T
NC_040265.1	11148128	A	G
NC_040265.1	11148295	G	C
NC_040265.1	11163260	C	T
NC_040265.1	11165122	A	G
NC_040265.1	11187931	G	A
NC_040265.1	11248368	T	C
NC_040265.1	11367011	G	A
NC_040265.1	11373015	T	C
NC_040265.1	11449927	T	C
NC_040265.1	11518677	T	C
NC_040265.1	11524686	A	T
NC_040265.1	11583543	T	C
NC_040265.1	11596352	G	A
NC_040265.1	11607693	A	G
NC_040265.1	11671955	T	C
NC_040265.1	11723152	T	C
NC_040265.1	11789365	T	C
NC_040265.1	11792952	A	G
NC_040265.1	11815716	T	C
NC_040265.1	11863088	G	A
NC_040265.1	11864139	A	G
NC_040265.1	11864577	A	G
NC_040265.1	11864955	T	C
NC_040265.1	11865197	A	G
NC_040265.1	11873963	T	C
NC_040265.1	11912201	T	C
NC_040265.1	11934923	T	G
NC_040265.1	11935781	T	C
NC_040265.1	11936741	T	C
NC_040265.1	12227885	A	C
NC_040265.1	12316992	T	C
NC_040265.1	12317373	A	G
NC_040265.1	12321349	T	G
NC_040265.1	12325576	G	T
NC_040265.1	12326313	A	G
NC_040265.1	12326642	G	C
NC_040265.1	12326824	C	T
NC_040265.1	12327293	G	A
NC_040265.1	12327881	A	G
NC_040265.1	12329320	A	G

NC_040265.1	12329572	C	G
NC_040265.1	12371731	T	C
NC_040265.1	12430584	C	T
NC_040265.1	12441572	G	A
NC_040265.1	12441828	A	G
NC_040265.1	12523448	C	T
NC_040265.1	12539596	T	C
NC_040265.1	12541455	A	G
NC_040265.1	12541626	T	C
NC_040265.1	13019501	T	C
NC_040265.1	13019660	A	G
NC_040265.1	13021350	A	G
NC_040265.1	13035381	A	G
NC_040265.1	13035579	A	G
NC_040265.1	13036269	G	A
NC_040265.1	13036450	T	C
NC_040265.1	13036625	C	T
NC_040265.1	13063224	A	C
NC_040265.1	13063986	A	G
NC_040265.1	13064490	T	C
NC_040265.1	13074410	G	C
NC_040265.1	13076776	C	A
NC_040265.1	13076988	C	A
NC_040265.1	13679931	T	C
NC_040265.1	13698017	T	C
NC_040265.1	13698985	C	T
NC_040265.1	13699744	C	T
NC_040265.1	13700582	C	A
NC_040265.1	13701096	T	C
NC_040265.1	13703701	T	C
NC_040265.1	13710298	C	T
NC_040265.1	13739068	T	C
NC_040265.1	13768802	A	G
NC_040265.1	13771458	T	C
NC_040265.1	13771794	A	G
NC_040265.1	13772334	T	C
NC_040265.1	13813735	T	C
NC_040265.1	13921690	A	G
NC_040265.1	13997382	G	T
NC_040265.1	14003635	T	C
NC_040265.1	14080470	G	A
NC_040265.1	14081212	A	G
NC_040265.1	14083221	T	G
NC_040265.1	14211469	T	C
NC_040265.1	14235944	C	T
NC_040265.1	14448268	T	C
NC_040265.1	14481867	A	G

NC_040265.1	14483028	G	T
NC_040265.1	14484177	A	G
NC_040265.1	14485202	A	G
NC_040265.1	14485554	A	G
NC_040265.1	14485708	A	G
NC_040265.1	14485872	T	C
NC_040265.1	14486087	G	A
NC_040265.1	14486539	C	T
NC_040265.1	14489896	T	C
NC_040265.1	14490052	C	T
NC_040265.1	14491018	T	C
NC_040265.1	14491433	C	T
NC_040265.1	14492173	A	G
NC_040265.1	14492927	T	C
NC_040265.1	14493114	G	C
NC_040265.1	14562232	A	G
NC_040265.1	14616209	G	A
NC_040265.1	14655230	A	G
NC_040265.1	14656887	A	G
NC_040265.1	14657249	C	T
NC_040265.1	14666461	T	C
NC_040265.1	14674052	A	T
NC_040265.1	14674322	T	C
NC_040265.1	14692898	C	T
NC_040265.1	14693605	A	G
NC_040265.1	14693769	C	T
NC_040265.1	14704074	A	C
NC_040265.1	14704460	G	A
NC_040265.1	14707748	G	A
NC_040265.1	14712313	T	G
NC_040265.1	14720924	T	C
NC_040265.1	14722858	A	G
NC_040265.1	14724314	G	A
NC_040265.1	14726842	T	C
NC_040265.1	14727359	T	C
NC_040265.1	14727825	A	G
NC_040265.1	14728252	T	C
NC_040265.1	14728628	C	T
NC_040265.1	14735329	C	A
NC_040265.1	14788308	G	A
NC_040265.1	14788769	C	T
NC_040265.1	14790827	T	C
NC_040265.1	14791339	A	G
NC_040265.1	14791923	G	C
NC_040265.1	14792254	C	T
NC_040265.1	14793121	G	T
NC_040265.1	14817045	T	C

NC_040265.1	14817550	G	A
NC_040265.1	14821779	G	A
NC_040265.1	14826965	C	G
NC_040265.1	14828025	C	A
NC_040265.1	14828364	A	G
NC_040265.1	14828515	T	C
NC_040265.1	14829410	C	T
NC_040265.1	14830010	T	C
NC_040265.1	14830917	A	G
NC_040265.1	14837493	A	G
NC_040265.1	14846653	T	C
NC_040265.1	14846893	A	G
NC_040265.1	14983666	A	G
NC_040265.1	14990110	T	C
NC_040265.1	14999107	A	G
NC_040265.1	15026942	T	C
NC_040265.1	15053223	C	T
NC_040265.1	15055175	T	C
NC_040265.1	15056613	A	C
NC_040265.1	15057283	A	G
NC_040265.1	15057545	T	C
NC_040265.1	15057928	A	G
NC_040265.1	15113518	A	G
NC_040265.1	15113788	A	G
NC_040265.1	15119540	A	G
NC_040265.1	15119908	C	T
NC_040265.1	15120189	A	G
NC_040265.1	15121486	A	C
NC_040265.1	15122686	T	C
NC_040265.1	15124720	A	G
NC_040265.1	15140120	T	C
NC_040265.1	15261682	G	T
NC_040265.1	15289620	A	T
NC_040265.1	15323055	T	C
NC_040265.1	15351153	G	A
NC_040265.1	15356640	T	C
NC_040265.1	15357782	A	G
NC_040265.1	15358734	T	C
NC_040265.1	15359012	T	C
NC_040265.1	15375593	A	G
NC_040265.1	15388640	T	C
NC_040265.1	15389349	G	A
NC_040265.1	15416589	C	T
NC_040265.1	15425680	A	G
NC_040265.1	15445637	A	G
NC_040265.1	15454693	A	C
NC_040265.1	15460121	G	A

NC_040265.1	15460594	A	G
NC_040265.1	15460750	T	C
NC_040265.1	15477116	T	G
NC_040265.1	15478924	C	T
NC_040265.1	15516597	C	G
NC_040265.1	15517576	A	G
NC_040265.1	15518367	T	C
NC_040265.1	15518517	A	G
NC_040265.1	15518732	T	C
NC_040265.1	15519423	C	G
NC_040265.1	15520541	T	C
NC_040265.1	15522491	A	G
NC_040265.1	15545749	T	C
NC_040265.1	15547112	G	C
NC_040265.1	15547469	T	C
NC_040265.1	15572969	A	G
NC_040265.1	15668126	T	A
NC_040265.1	15761280	C	T
NC_040265.1	15811542	G	A
NC_040265.1	15837933	C	T
NC_040265.1	15855337	A	G
NC_040265.1	15867175	A	G
NC_040265.1	16097122	A	T
NC_040265.1	16130139	G	C
NC_040265.1	16130885	A	G
NC_040265.1	16158467	A	G
NC_040265.1	16269955	C	A
NC_040265.1	16270304	A	G
NC_040265.1	16356001	T	C
NC_040265.1	16356297	G	T
NC_040265.1	16357149	C	T
NC_040265.1	16764287	G	A
NC_040265.1	17445898	G	A
NC_040265.1	17908309	G	C
NC_040265.1	17914084	T	C
NC_040265.1	17954638	T	C
NC_040265.1	18009371	A	G
NC_040265.1	18082126	T	C
NC_040265.1	18113491	T	C
NC_040265.1	18188072	T	C
NC_040265.1	18192011	A	T
NC_040265.1	18367984	T	C
NC_040265.1	18371809	C	A
NC_040265.1	19121156	A	G
NC_040265.1	19226278	C	T
NC_040265.1	19238392	T	C
NC_040265.1	19355611	G	C

NC_040265.1	19496927	A	G
NC_040265.1	19497662	G	A
NC_040265.1	19498389	A	G
NC_040265.1	19499075	T	C
NC_040265.1	19846490	T	C
NC_040265.1	19910172	A	C
NC_040265.1	20114251	C	T
NC_040265.1	20327027	T	C
NC_040265.1	20418132	C	T
NC_040265.1	20452361	C	T
NC_040265.1	20475704	A	G
NC_040265.1	20476328	A	G
NC_040265.1	20523099	T	C
NC_040265.1	20581133	A	G
NC_040265.1	20582295	T	A
NC_040265.1	20922198	A	C
NC_040265.1	20923843	A	G
NC_040265.1	20925682	A	G
NC_040265.1	22342043	C	G
NC_040265.1	23042404	A	G
NC_040265.1	23131012	T	C
NC_040265.1	23169512	T	C
NC_040265.1	23245642	C	T
NC_040265.1	23247577	A	G
NC_040265.1	23251513	G	A
NC_040265.1	23317139	A	C
NC_040265.1	23328527	A	G
NC_040265.1	23329263	G	A
NC_040265.1	23330402	C	A
NC_040265.1	23430193	A	G
NC_040265.1	23480344	C	T
NC_040265.1	23490557	A	G
NC_040265.1	23529433	G	C
NC_040265.1	23766234	C	A
NC_040265.1	24111896	C	G
NC_040265.1	24723886	G	T
NC_040265.1	25092937	C	G
NC_040265.1	25095626	C	G
NC_040265.1	25241263	A	T
NC_040265.1	25241649	G	C
NC_040265.1	25249103	C	T
NC_040265.1	25300672	A	G
NC_040265.1	25317124	A	G
NC_040265.1	25332200	A	G
NC_040265.1	25407582	A	G
NC_040265.1	25448064	T	C
NC_040265.1	25504532	T	C

NC_040265.1	25606125	C	A
NC_040265.1	25688135	A	G
NC_040265.1	25725522	A	G
NC_040265.1	26251135	T	C
NC_040265.1	26401002	A	G
NC_040265.1	26496853	T	C
NC_040265.1	26567079	A	G
NC_040265.1	26663934	G	A
NC_040265.1	26733396	A	C
NC_040265.1	26857118	G	A
NC_040265.1	26966439	A	G
NC_040265.1	27038465	C	T
NC_040265.1	27070404	T	C
NC_040265.1	27206539	G	A
NC_040265.1	27238013	C	T
NC_040265.1	27287432	A	C
NC_040265.1	27323378	T	C
NC_040265.1	27459381	A	C
NC_040265.1	27506516	A	G
NC_040265.1	27509419	T	C
NC_040265.1	27527958	T	C
NC_040265.1	27562405	A	G
NC_040265.1	27693640	C	T
NC_040265.1	27804483	A	G
NC_040265.1	27858395	A	G
NC_040265.1	27898371	A	C
NC_040265.1	27902480	C	T
NC_040265.1	28031330	T	C
NC_040265.1	30963172	C	G
NC_040265.1	31220725	T	C
NC_040265.1	35499557	A	G
NC_040265.1	35511072	A	G
NC_040265.1	36917123	T	C
NC_040265.1	36948092	G	A
NC_040265.1	37020365	A	G
NC_040265.1	37162276	A	G
NC_040265.1	37242641	T	C
NC_040265.1	37283199	A	G
NC_040265.1	37374323	T	C
NC_040265.1	37376302	A	T
NC_040265.1	37393053	T	C
NC_040265.1	37424173	A	G
NC_040265.1	37426745	G	A
NC_040265.1	37438732	C	T
NC_040265.1	37571180	T	C
NC_040265.1	37582360	A	G
NC_040265.1	37602325	T	G

NC_040265.1	37606609	C	A
NC_040265.1	37617691	A	G
NC_040265.1	37638459	A	G
NC_040265.1	37644932	A	C
NC_040265.1	37674860	A	G
NC_040265.1	37679427	A	G
NC_040265.1	37687063	T	C
NC_040265.1	37689208	A	C
NC_040265.1	37755757	T	C
NC_040265.1	37758842	C	G
NC_040265.1	37773053	C	T
NC_040265.1	37904507	A	G
NC_040265.1	37958927	C	T
NC_040265.1	37966178	A	G
NC_040265.1	37976712	C	G
NC_040265.1	37995472	G	A
NC_040265.1	38015372	G	A
NC_040265.1	38039259	C	A
NC_040265.1	38124619	T	C
NC_040265.1	38143970	A	G
NC_040265.1	38150653	C	T
NC_040265.1	38153298	T	C
NC_040265.1	38187237	T	A
NC_040265.1	38239758	T	C
NC_040265.1	38253183	C	T
NC_040265.1	38282768	T	C
NC_040265.1	38323397	C	A
NC_040265.1	38361631	T	C
NC_040265.1	38447990	C	T
NC_040265.1	38495510	T	C
NC_040265.1	38668049	A	G
NC_040265.1	38682373	T	C
NC_040265.1	38747163	A	C
NC_040265.1	38780917	G	A
NC_040265.1	38792873	G	A
NC_040265.1	38961053	G	A
NC_040265.1	39108107	T	C
NC_040265.1	39225125	A	G
NC_040265.1	39227528	A	C
NC_040265.1	39247500	G	C
NC_040265.1	39253036	A	T
NC_040265.1	39254614	G	A
NC_040265.1	39258837	T	C
NC_040265.1	39440518	C	T
NC_040265.1	39498934	C	T
NC_040265.1	39534122	C	T
NC_040265.1	39535502	A	G

NC_040265.1	39538314	T	C
NC_040265.1	39540996	A	G
NC_040265.1	39542998	A	G
NC_040265.1	39551071	T	C
NC_040265.1	39735250	A	G
NC_040265.1	41059141	G	A
NC_040265.1	41205733	T	C
NC_040265.1	41209128	A	G
NC_040265.1	41784047	A	T
NC_040265.1	41824164	T	C
NC_040265.1	41846778	A	G
NC_040265.1	41851259	G	A
NC_040265.1	41856110	G	A
NC_040265.1	41858713	T	G
NC_040265.1	41908811	A	G
NC_040265.1	42036564	T	C
NC_040265.1	42038548	G	A
NC_040265.1	42047755	C	T
NC_040265.1	42052947	A	G
NC_040265.1	42058404	T	A
NC_040265.1	42061029	C	T
NC_040265.1	42128433	T	C
NC_040265.1	42168496	C	A
NC_040265.1	42233773	G	A
NC_040265.1	42251647	G	A
NC_040265.1	42354278	T	C
NC_040265.1	42426081	T	C
NC_040265.1	42439612	A	G
NC_040265.1	42499642	C	T
NC_040265.1	42576382	G	A
NC_040265.1	42643632	T	C
NC_040265.1	42767124	T	C
NC_040265.1	42887638	G	T
NC_040265.1	42925694	C	T
NC_040265.1	43080881	G	C
NC_040265.1	43088242	T	C
NC_040265.1	43103884	C	G
NC_040265.1	43110719	T	C
NC_040265.1	43217577	A	G
NC_040265.1	43342120	A	G
NC_040265.1	43557635	C	T
NC_040265.1	43757875	T	C
NC_040265.1	43859162	A	G
NC_040265.1	43866782	T	G
NC_040265.1	44637825	G	A
NC_040265.1	45762754	A	G
NC_040265.1	45770107	C	T

NC_040265.1	45799869	A	G
NC_040265.1	45802809	C	T
NC_040265.1	45820232	A	G
NC_040265.1	46003458	A	G
NC_040265.1	46009198	G	A
NC_040265.1	46071745	C	G
NC_040265.1	46167424	T	C
NC_040265.1	46351947	T	C
NC_040265.1	46443886	A	G
NC_040265.1	46507522	A	G
NC_040265.1	46553254	G	A
NC_040265.1	46555160	G	A
NC_040265.1	46570084	T	C
NC_040265.1	46709057	C	T
NC_040265.1	47025422	G	T
NC_040265.1	47121690	G	C
NC_040265.1	47132784	A	G
NC_040265.1	47133705	C	T
NC_040265.1	47135263	G	A
NC_040265.1	47509691	T	C
NC_040265.1	47561807	A	G
NC_040265.1	47593139	A	C
NC_040265.1	47662653	C	T
NC_040265.1	47663137	G	A
NC_040265.1	47672850	A	C
NC_040265.1	47674238	T	C
NC_040265.1	47690731	T	G
NC_040265.1	47696428	T	C
NC_040265.1	47697129	A	G
NC_040265.1	47769113	T	C
NC_040265.1	47800843	T	C
NC_040265.1	47801617	T	C
NC_040265.1	47978434	T	C
NC_040265.1	48059290	C	T
NC_040265.1	48166382	C	A
NC_040265.1	48185944	T	C
NC_040265.1	48233391	A	G
NC_040265.1	48260071	C	A
NC_040265.1	48412864	T	C
NC_040265.1	48455450	T	G
NC_040265.1	48475907	T	C
NC_040265.1	48497623	T	C
NC_040265.1	48563645	T	C
NC_040265.1	48567851	A	G
NC_040265.1	48677819	G	A
NC_040265.1	48685077	T	C
NC_040265.1	48692552	A	G

NC_040265.1	48756110	T	C
NC_040265.1	48761432	C	T
NC_040265.1	48766093	T	C
NC_040265.1	48827613	T	C
NC_040265.1	48885836	T	C
NC_040265.1	48896727	G	C
NC_040265.1	48901297	G	A
NC_040265.1	48913424	T	C
NC_040265.1	48920782	A	G
NC_040265.1	48928016	C	T
NC_040265.1	48929549	A	G
NC_040265.1	49068342	C	T
NC_040265.1	49073117	G	A
NC_040265.1	49093180	A	T
NC_040265.1	49155525	A	G
NC_040265.1	49156769	C	T
NC_040265.1	49157615	T	G
NC_040265.1	49159584	C	A
NC_040265.1	49165250	A	G
NC_040265.1	49171688	T	C
NC_040265.1	49183009	C	T
NC_040265.1	49184957	G	A
NC_040265.1	49195974	T	C
NC_040265.1	49196434	A	G
NC_040265.1	49237616	G	C
NC_040265.1	49245521	T	A
NC_040265.1	49263145	G	A
NC_040265.1	49273673	T	C
NC_040265.1	49288214	T	C
NC_040265.1	49289383	A	C
NC_040265.1	49294302	A	G
NC_040265.1	49299052	G	C
NC_040265.1	49304947	T	C
NC_040265.1	49318115	A	G
NC_040265.1	49320323	A	G
NC_040265.1	49360379	A	G
NC_040265.1	49405015	A	G
NC_040265.1	49429628	G	A
NC_040265.1	49498076	T	C
NC_040265.1	49499853	A	G
NC_040265.1	49506766	T	C
NC_040265.1	49525959	G	C
NC_040265.1	49583686	G	A
NC_040265.1	49602502	G	A
NC_040265.1	49603870	A	C
NC_040265.1	49630300	G	A
NC_040265.1	49635299	G	C

NC_040265.1	49682765	A	G
NC_040265.1	49683835	T	C
NC_040265.1	49708677	C	T
NC_040265.1	49788440	A	C
NC_040265.1	49796212	G	A
NC_040265.1	49871275	G	A
NC_040265.1	49872969	T	C
NC_040265.1	49896331	G	A
NC_040265.1	49897164	A	C
NC_040265.1	49936487	T	C
NC_040265.1	49968981	T	G
NC_040265.1	49984410	G	A
NC_040265.1	50014264	C	A
NC_040265.1	50028597	T	C
NC_040265.1	50239860	C	T
NC_040265.1	50242030	A	G
NC_040265.1	50259567	A	C
NC_040265.1	50260234	G	A
NC_040265.1	50369348	A	C
NC_040265.1	50470652	C	T
NC_040265.1	50553836	A	T
NC_040265.1	50554502	T	C
NC_040265.1	50556246	G	A
NC_040265.1	50561510	A	G
NC_040265.1	50570755	C	A
NC_040265.1	50719269	A	G
NC_040265.1	50748476	A	G
NC_040265.1	50885971	T	C
NC_040265.1	50908544	A	G
NC_040265.1	50915221	C	T
NC_040265.1	50963458	T	G
NC_040265.1	51021512	A	G
NC_040265.1	51028557	G	A
NC_040265.1	51089753	G	C
NC_040265.1	51092202	A	G
NC_040265.1	51098790	T	C
NC_040265.1	51104999	A	G
NC_040265.1	51121056	T	C
NC_040265.1	51129024	A	G
NC_040265.1	51131985	T	C
NC_040265.1	51140070	C	T
NC_040265.1	51159017	A	G
NC_040265.1	51162255	A	G
NC_040265.1	51171418	A	G
NC_040265.1	51189962	G	A
NC_040265.1	51355337	G	A
NC_040265.1	51442687	T	A

NC_040265.1	51461025	T	C
NC_040265.1	51467921	A	G
NC_040265.1	51494747	T	C
NC_040265.1	51501979	G	T
NC_040265.1	51531085	A	G
NC_040265.1	51543799	A	G
NC_040265.1	51545930	G	A
NC_040265.1	51627889	A	G
NC_040265.1	51682660	A	G
NC_040265.1	51726324	C	T
NC_040265.1	51734144	C	T
NC_040265.1	51795898	T	C
NC_040265.1	51824143	A	G
NC_040265.1	51914817	A	G
NC_040265.1	51918286	T	C
NC_040265.1	51982090	C	A
NC_040265.1	52003520	A	G
NC_040265.1	52009806	T	A
NC_040265.1	52010754	T	C
NC_040265.1	52011447	A	G
NC_040265.1	52038344	C	T
NC_040265.1	52076930	G	A
NC_040265.1	52082015	T	C
NC_040265.1	52084180	A	G
NC_040265.1	52105253	G	C
NC_040265.1	52477708	T	C
NC_040265.1	52478719	T	C
NC_040265.1	52567261	C	T
NC_040265.1	52574279	A	G
NC_040265.1	52626736	A	G
NC_040265.1	52634707	T	G
NC_040265.1	52652270	A	C
NC_040265.1	52689376	G	A
NC_040265.1	52705803	A	G
NC_040265.1	52750995	C	G
NC_040265.1	52783427	C	G
NC_040265.1	52793892	C	T
NC_040265.1	52798958	G	C
NC_040265.1	52815714	A	G
NC_040265.1	52834951	T	C
NC_040265.1	52869458	T	G
NC_040265.1	52931977	T	C
NC_040265.1	52951363	T	G
NC_040265.1	52953959	A	G
NC_040265.1	52955693	T	G
NC_040265.1	52964571	A	G
NC_040265.1	52965030	T	C

NC_040265.1	52966012	A	G
NC_040265.1	53008591	A	C
NC_040265.1	53011093	G	A
NC_040265.1	53022913	T	C
NC_040265.1	53023703	T	C
NC_040265.1	53076171	A	G
NC_040265.1	53079194	A	C
NC_040265.1	53081406	T	C
NC_040265.1	53082741	A	G
NC_040265.1	53085374	A	G
NC_040265.1	53093033	A	G
NC_040265.1	53183668	G	A
NC_040265.1	53256952	T	C
NC_040265.1	53259239	G	A
NC_040265.1	53280333	T	C
NC_040265.1	53283726	C	T
NC_040265.1	53324691	A	G
NC_040265.1	53325918	G	A
NC_040265.1	53614303	A	G
NC_040265.1	53626139	G	A
NC_040265.1	53626714	G	A
NC_040265.1	53637132	A	G
NC_040265.1	53671783	G	A
NC_040265.1	53672448	A	C
NC_040265.1	53720006	T	C
NC_040265.1	53741860	A	G
NC_040265.1	53765010	T	C
NC_040265.1	53793783	A	G
NC_040265.1	53854955	C	T
NC_040265.1	53880669	T	C
NC_040265.1	53994396	A	C
NC_040265.1	54000312	C	T
NC_040265.1	54060848	A	G
NC_040265.1	54064503	A	G
NC_040265.1	54066890	A	G
NC_040265.1	54087332	A	G
NC_040265.1	54103316	T	C
NC_040265.1	54106327	G	A
NC_040265.1	54114265	A	C
NC_040265.1	54126822	T	G
NC_040265.1	54128699	C	T
NC_040265.1	54139236	T	C
NC_040265.1	54145519	T	C
NC_040265.1	54149897	A	T
NC_040265.1	54153574	T	C
NC_040265.1	54170669	T	C
NC_040265.1	54171890	T	C

NC_040265.1	54194193	T	C
NC_040265.1	54211595	A	G
NC_040265.1	54231705	T	C
NC_040265.1	54299783	T	C
NC_040265.1	54325553	G	T
NC_040265.1	54332204	A	G
NC_040265.1	54333977	G	A
NC_040265.1	54338921	A	G
NC_040265.1	54340133	A	G
NC_040265.1	54341291	T	A
NC_040265.1	54412565	T	C
NC_040265.1	54492797	T	C
NC_040265.1	54515993	T	C
NC_040265.1	54839592	A	G
NC_040265.1	54847516	T	C
NC_040265.1	54855641	A	G
NC_040265.1	55080163	T	C
NC_040265.1	55130948	A	G
NC_040265.1	55133226	T	C
NC_040265.1	55192638	C	T
NC_040265.1	55204291	C	T
NC_040265.1	55215336	A	G
NC_040265.1	55331705	C	A
NC_040265.1	55335099	T	C
NC_040265.1	55350776	T	C
NC_040265.1	55356313	T	C
NC_040265.1	55442783	T	G
NC_040265.1	55495034	T	C
NC_040265.1	55573801	G	T
NC_040265.1	55575387	G	A
NC_040265.1	55631731	T	C
NC_040265.1	55666884	T	G
NC_040265.1	55668374	G	A
NC_040265.1	55677174	T	G
NC_040265.1	55742546	A	G
NC_040265.1	55743300	A	G
NC_040265.1	55791048	A	G
NC_040265.1	55826633	A	G
NC_040265.1	55889418	T	C
NC_040265.1	55915485	G	C
NC_040265.1	55928758	G	T
NC_040265.1	55932687	G	A
NC_040265.1	56016226	G	A
NC_040265.1	56056785	T	C
NC_040265.1	56075012	G	A
NC_040265.1	56131351	T	G
NC_040265.1	56133723	T	G

NC_040265.1	56239634	A	G
NC_040265.1	56243543	G	T
NC_040265.1	56246611	G	C
NC_040265.1	56323369	T	C
NC_040265.1	56324758	G	C
NC_040265.1	56388920	T	C
NC_040265.1	56404923	A	C
NC_040265.1	56425963	T	C
NC_040265.1	56434560	G	C
NC_040265.1	56472894	A	G
NC_040265.1	56497883	A	G
NC_040265.1	56503759	T	C
NC_040265.1	56515355	A	G
NC_040265.1	56515921	A	G
NC_040265.1	56565949	G	T
NC_040265.1	56567320	G	C
NC_040265.1	56572095	T	C
NC_040265.1	56596655	A	C
NC_040265.1	56603825	T	C
NC_040265.1	56608819	A	G
NC_040265.1	56609753	A	G
NC_040265.1	56681919	C	T
NC_040265.1	56741998	G	A
NC_040265.1	56742924	G	A
NC_040265.1	56752524	A	G
NC_040265.1	56759972	A	G
NC_040265.1	56760740	G	A
NC_040265.1	56798630	G	C
NC_040265.1	56799607	G	A
NC_040265.1	56801057	T	C
NC_040265.1	56830858	A	G
NC_040265.1	56862638	A	G
NC_040265.1	56886321	G	A
NC_040265.1	56886928	A	G
NC_040265.1	56988149	A	G
NC_040265.1	57052883	A	G
NC_040265.1	57059428	A	G
NC_040265.1	57075696	G	A
NC_040265.1	57175497	A	T
NC_040265.1	57175910	A	C
NC_040265.1	57237562	A	G
NC_040265.1	57238473	C	T
NC_040265.1	57257158	A	G
NC_040265.1	57282977	C	T
NC_040265.1	57301070	A	G
NC_040265.1	57306886	C	T
NC_040265.1	57307295	A	G

NC_040265.1	57336075	G	T
NC_040265.1	57336889	A	G
NC_040265.1	57408754	G	A
NC_040265.1	57462020	A	G
NC_040265.1	57462641	A	G
NC_040265.1	57463790	T	C
NC_040265.1	57465248	T	C
NC_040265.1	57533681	A	G
NC_040265.1	57570098	A	G
NC_040265.1	57587499	C	T
NC_040265.1	57705305	A	G
NC_040265.1	57747369	A	G
NC_040265.1	57748975	T	C
NC_040265.1	57835350	A	G
NC_040265.1	57836874	C	T
NC_040265.1	57850224	G	A
NC_040265.1	57859171	A	G
NC_040265.1	57876517	A	C
NC_040265.1	57879849	C	G
NC_040265.1	57899816	G	T
NC_040265.1	57913097	T	G
NC_040265.1	57927942	A	G
NC_040265.1	57929954	T	C
NC_040265.1	57968828	C	T
NC_040265.1	57969264	A	G
NC_040265.1	58065504	G	A
NC_040265.1	58067661	A	G
NC_040265.1	58068687	G	A
NC_040265.1	58083346	A	G
NC_040265.1	58092792	A	G
NC_040265.1	58107953	T	G
NC_040265.1	58315827	G	A
NC_040265.1	58338438	A	G
NC_040265.1	58346345	A	G
NC_040265.1	58361031	T	A
NC_040265.1	58383659	G	T
NC_040265.1	58384059	T	A
NC_040265.1	58400251	G	A
NC_040265.1	58465379	C	T
NC_040265.1	58471538	A	G
NC_040265.1	58473084	G	C
NC_040265.1	58504205	A	G
NC_040265.1	58513877	C	T
NC_040265.1	58536936	G	A
NC_040265.1	58579411	A	G
NC_040265.1	58586110	T	C
NC_040265.1	58588975	A	G

NC_040265.1	58591580	A	G
NC_040265.1	58596676	T	C
NC_040265.1	58598327	A	G
NC_040265.1	58613399	T	C
NC_040265.1	58620292	C	G
NC_040265.1	58623771	A	G
NC_040265.1	58728990	G	A
NC_040265.1	58767739	A	G
NC_040265.1	58806649	G	A
NC_040265.1	58811036	C	T
NC_040265.1	58812982	G	A
NC_040265.1	58814586	G	A
NC_040265.1	58820715	G	A
NC_040265.1	58826324	A	C
NC_040265.1	58873810	G	A
NC_040265.1	58892233	A	G
NC_040265.1	58920037	C	A
NC_040265.1	58921859	G	A
NC_040265.1	58947178	T	C
NC_040265.1	58968532	A	G
NC_040265.1	58988703	A	C
NC_040265.1	58999482	T	G
NC_040265.1	59005106	A	G
NC_040265.1	59014833	T	C
NC_040265.1	59020331	T	C
NC_040265.1	59022386	C	T
NC_040265.1	59024364	A	G
NC_040265.1	59024993	T	G
NC_040265.1	59030801	T	C
NC_040265.1	59074009	C	G
NC_040265.1	59089323	A	T
NC_040265.1	59137773	C	T
NC_040265.1	59213543	C	T
NC_040265.1	59266818	T	C
NC_040265.1	59281190	G	C
NC_040265.1	59311973	C	T
NC_040265.1	59329426	A	T
NC_040265.1	59348999	T	G
NC_040265.1	59361439	T	C
NC_040265.1	59449438	C	G
NC_040265.1	59450009	A	C
NC_040265.1	59485160	G	A
NC_040265.1	59491759	0	G
NC_040265.1	59492922	T	C
NC_040265.1	59500448	C	G
NC_040265.1	59502883	A	T
NC_040265.1	59525457	T	C

NC_040265.1	59559225	G	C
NC_040265.1	59654566	A	G
NC_040265.1	59655349	T	C
NC_040265.1	59659551	A	G
NC_040265.1	59661438	T	C
NC_040265.1	59674033	G	C
NC_040265.1	59685100	C	T
NC_040265.1	59686384	G	A
NC_040265.1	59710139	A	G
NC_040265.1	59730144	T	C
NC_040265.1	59776009	A	G
NC_040265.1	59791305	A	T
NC_040265.1	59854235	G	C
NC_040265.1	59914414	T	G
NC_040265.1	59936422	C	T
NC_040265.1	59944777	C	T
NC_040265.1	59953546	A	G
NC_040265.1	59983571	T	C
NC_040265.1	59987897	T	C
NC_040265.1	60013014	G	A
NC_040265.1	60013873	A	C
NC_040265.1	60017261	C	T
NC_040265.1	60019555	A	G
NC_040265.1	60024242	T	C
NC_040265.1	60143531	C	G
NC_040265.1	60146977	T	C
NC_040265.1	60150506	T	C
NC_040265.1	60151816	T	C
NC_040265.1	60152500	G	A
NC_040265.1	60223195	C	A
NC_040265.1	60226103	T	C
NC_040265.1	60254009	T	C
NC_040265.1	60258355	G	T
NC_040265.1	60282059	A	G
NC_040265.1	60286524	T	G
NC_040265.1	60288154	A	G
NC_040265.1	60299149	A	G
NC_040265.1	60301665	A	G
NC_040265.1	60307381	A	G
NC_040265.1	60361127	G	C
NC_040265.1	60367158	C	T
NC_040265.1	60368006	A	G
NC_040265.1	60389016	G	C
NC_040265.1	60408711	G	A
NC_040265.1	60436287	T	C
NC_040265.1	60489502	A	C
NC_040265.1	60489727	A	G

NC_040265.1	60596183	T	C
NC_040265.1	60664220	G	A
NC_040265.1	61091462	T	C
NC_040265.1	61107291	T	C
NC_040265.1	61132826	A	C
NC_040265.1	61135048	T	C
NC_040265.1	61136982	A	G
NC_040265.1	61137777	A	T
NC_040265.1	61150041	T	C
NC_040265.1	61150350	T	G
NC_040265.1	61171543	T	G
NC_040265.1	61172227	T	C
NC_040265.1	61194539	C	G
NC_040265.1	61196000	T	G
NC_040265.1	61219904	C	T
NC_040265.1	61236724	T	C
NC_040265.1	61269075	C	T
NC_040265.1	61509805	A	G
NC_040265.1	61563343	A	G
NC_040265.1	61632936	A	G
NC_040265.1	61677728	G	A
NC_040265.1	61677943	T	A
NC_040265.1	61678434	C	G
NC_040265.1	61678862	T	C
NC_040265.1	61679481	T	C
NC_040265.1	61679701	A	G
NC_040265.1	61721075	A	G
NC_040265.1	61761236	C	T
NC_040265.1	61939129	A	C
NC_040265.1	62220680	C	T
NC_040265.1	62227363	T	C
NC_040265.1	62228350	A	T
NC_040265.1	62228527	G	A
NC_040265.1	62229092	C	T
NC_040265.1	62261780	G	A
NC_040265.1	62262404	G	A
NC_040265.1	62262569	T	C
NC_040265.1	62263365	G	C
NC_040265.1	62315178	T	A
NC_040265.1	62996383	C	G
NC_040265.1	63789436	G	A
NC_040265.1	63844752	C	T
NC_040265.1	64212085	C	G
NC_040265.1	64432538	C	T
NC_040265.1	64481910	T	C
NC_040265.1	64534705	T	C
NC_040265.1	64535928	A	G

NC_040265.1	64576319	T	C
NC_040265.1	64602853	C	T
NC_040265.1	64768292	A	G
NC_040265.1	64914525	C	T
NC_040265.1	65038425	T	C
NC_040265.1	65064035	G	A
NC_040265.1	65134596	A	G
NC_040265.1	65155623	A	G
NC_040265.1	65194979	C	T
NC_040265.1	65209879	A	G
NC_040265.1	65210053	G	A
NC_040265.1	65506396	C	T
NC_040265.1	65641085	T	G
NC_040265.1	65642243	A	G
NC_040265.1	65708206	C	T
NC_040265.1	65816552	G	A
NC_040265.1	65869586	A	C
NC_040265.1	65869816	A	G
NC_040265.1	65985014	C	T
NC_040265.1	65995608	G	A
NC_040265.1	65999120	A	G
NC_040265.1	66078740	A	G
NC_040265.1	66130123	G	C
NC_040265.1	66135861	T	C
NC_040265.1	66136068	A	G
NC_040265.1	66151132	G	A
NC_040265.1	66182477	G	T
NC_040265.1	66210708	A	G
NC_040265.1	66211053	0	0
NC_040265.1	66217904	T	C
NC_040265.1	66261462	A	G
NC_040265.1	66456411	T	G
NC_040265.1	66497760	C	T
NC_040265.1	66554448	T	G
NC_040265.1	66565195	T	G
NC_040265.1	66580024	A	G
NC_040265.1	66581925	A	C
NC_040265.1	66582083	C	T
NC_040265.1	66583079	A	T
NC_040265.1	66583957	G	A
NC_040265.1	66584166	A	C
NC_040265.1	66585114	A	G
NC_040265.1	66585869	G	A
NC_040265.1	66586555	0	G
NC_040265.1	66587496	T	A
NC_040265.1	66611626	A	C
NC_040265.1	66611876	T	C

NC_040265.1	66613203	C		T
NC_040265.1	66617073	C		T
NC_040265.1	66619136	G		C
NC_040265.1	66619324	T		G
NC_040265.1	66636185	T		A
NC_040265.1	66645155		0	T
NC_040265.1	66646522	C		G
NC_040265.1	66669953	G		T
NC_040265.1	66677465	A		C
NC_040265.1	66700232	T		C
NC_040265.1	66703189	A		G
NC_040265.1	66703883	A		C
NC_040265.1	66705774	C		G
NC_040265.1	66712959	G		C
NC_040265.1	66713149	A		G
NC_040265.1	66714658	A		G
NC_040265.1	66715507	A		G
NC_040265.1	66716350	T		C
NC_040265.1	66749184	C		T
NC_040265.1	66749448	T		C
NC_040265.1	66762951	A		G
NC_040265.1	66763714	A		G
NC_040265.1	66764279	A		T
NC_040265.1	66765310	T		C
NC_040265.1	66765957	A		G
NC_040265.1	66767059	A		G
NC_040265.1	66779174	C		A
NC_040265.1	66782739	G		A
NC_040265.1	66791392	A		G
NC_040265.1	66792643	G		T
NC_040265.1	66793032	C		T
NC_040265.1	66793188	A		G
NC_040265.1	66808957	A		C
NC_040265.1	66821699	A		G
NC_040265.1	66846388	G		T
NC_040265.1	66861123	A		G
NC_040265.1	66872271	A		G
NC_040265.1	66894778	C		G
NC_040265.1	66908543	T		C
NC_040265.1	66915762	T		C
NC_040265.1	66923902	T		G
NC_040265.1	66925184	A		G
NC_040265.1	66937996	G		C
NC_040265.1	66971156	A		G
NC_040265.1	66976452	A		G
NC_040265.1	66996585	A		G
NC_040265.1	67068440	T		G

NC_040265.1	67909791	T	C	
NC_040265.1	67958519	A	G	
NC_040265.1	67971445	G	A	
NC_040265.1	67972008	T	C	
NC_040265.1	67973465	A	G	
NC_040265.1	67978031	A	C	
NC_040265.1	67981934	G	A	
NC_040265.1	67986388	C	A	
NC_040265.1	68117263	A	C	
NC_040265.1	68127908	G	A	
NC_040265.1	68128393	A	G	
NC_040265.1	68129892	T	C	
NC_040265.1	68131154	T	C	
NC_040265.1	68131383	A	C	
NC_040265.1	68143725	C	G	
NC_040265.1	68152673	C	G	
NC_040265.1	68153001	A	T	
NC_040265.1	68154185	T	A	
NC_040265.1	68160976	G	A	
NC_040265.1	68162770	C	T	
NC_040265.1	68170580	G	T	
NC_040265.1	68172743	C	T	
NC_040265.1	68205551	T	C	
NC_040265.1	68414423	T	C	
NC_040265.1	68422964	C	T	
NC_040265.1	68423629	C	T	
NC_040265.1	68423783	A	G	
NC_040265.1	68485530		0	0
NC_040265.1	68485728	T	C	
NC_040265.1	68497977	G	C	
NC_040265.1	68524852	T	C	
NC_040265.1	68525108	T	C	
NC_040265.1	68582231	T	C	
NC_040265.1	68582382	A	G	
NC_040265.1	68679624	A	G	
NC_040265.1	68680389	C	T	
NC_040265.1	68680764	C	T	
NC_040265.1	68680942	G	T	
NC_040265.1	68681118	C	T	
NC_040265.1	68716179	A	G	
NC_040265.1	68737112	A	G	
NC_040265.1	68767957	A	C	
NC_040265.1	68777751	T	G	
NC_040265.1	68777954	A	G	
NC_040265.1	68811869	G	C	
NC_040265.1	68812127	A	G	
NC_040265.1	69132511	A	G	

NC_040265.1	69212694	A	G
NC_040265.1	69213981	C	T
NC_040265.1	69214505	T	C
NC_040265.1	69214826	A	G
NC_040265.1	69215042	T	C
NC_040265.1	69216677	A	T
NC_040265.1	69220721	T	C
NC_040265.1	69522207	G	A
NC_040265.1	69581815	G	T
NC_040265.1	69602227	A	G
NC_040265.1	69604161	C	T
NC_040265.1	69604577	T	C
NC_040265.1	69605400	G	T
NC_040265.1	69606231	T	C
NC_040265.1	69609091	T	C
NC_040265.1	69611399	C	T
NC_040265.1	69630871	C	T
NC_040265.1	69706129	T	C
NC_040265.1	69709456	T	C
NC_040265.1	69714510	G	C
NC_040265.1	69722140	C	T
NC_040265.1	69790214	T	G
NC_040265.1	69795445	A	G
NC_040265.1	69847623	G	A
NC_040265.1	69860840	A	G
NC_040265.1	69917297	A	C
NC_040265.1	69923102	A	G
NC_040265.1	70148003	T	A
NC_040265.1	70156465	T	C
NC_040265.1	70161312	G	A
NC_040265.1	70196543	G	C
NC_040265.1	70198473	C	T
NC_040265.1	70210090	T	C
NC_040265.1	70210396	C	T
NC_040265.1	70231736	T	G
NC_040265.1	70234096	A	G
NC_040265.1	70257740	T	C
NC_040265.1	70290862	A	C
NC_040265.1	70291337	A	G
NC_040265.1	70293574	C	A
NC_040265.1	70299991	A	T
NC_040265.1	70301586	A	C
NC_040265.1	70302561	T	G
NC_040265.1	70338009	T	C
NC_040265.1	70356539	G	A
NC_040265.1	70381950	T	C
NC_040265.1	70401493	T	C

NC_040265.1	70405221	T	C
NC_040265.1	70412340	G	A
NC_040265.1	70523471	T	C
NC_040265.1	70556962	T	C
NC_040265.1	70621969	A	C
NC_040265.1	70642809	C	T
NC_040265.1	70644728	T	C
NC_040265.1	70645006	T	C
NC_040265.1	70716015	C	A
NC_040265.1	70729885	C	T
NC_040265.1	70730291	T	C
NC_040265.1	70732248	T	C
NC_040265.1	70732467	G	C
NC_040265.1	70732827	T	C
NC_040265.1	70750896	G	C
NC_040265.1	70774952	T	C
NC_040265.1	70797360	G	T
NC_040265.1	70856159	A	G
NC_040265.1	70879952	T	G
NC_040265.1	70880248	T	C
NC_040265.1	70880440	G	A
NC_040265.1	70881143	A	G
NC_040265.1	70882513	A	C
NC_040265.1	70882766	T	C
NC_040265.1	70884722	A	T
NC_040265.1	70885406	G	T
NC_040265.1	70905453	C	T
NC_040265.1	70906040	A	G
NC_040265.1	70930741	G	T
NC_040265.1	70943607	T	C
NC_040265.1	70943809	T	C
NC_040265.1	70949933	A	G
NC_040265.1	70958103	A	C
NC_040265.1	70959095	T	C
NC_040265.1	70977400	C	T
NC_040265.1	70980789	T	C
NC_040265.1	70981354	C	G
NC_040265.1	70982570	G	T
NC_040265.1	70987402	T	C
NC_040265.1	70991311	T	C
NC_040265.1	70991467	T	C
NC_040265.1	70996470	A	G
NC_040265.1	71042014	A	G
NC_040265.1	71048942	A	G
NC_040265.1	71054603	G	C
NC_040265.1	71064217	T	C
NC_040265.1	71068124	T	C

NC_040265.1	71068666	A	G
NC_040265.1	71074468	T	C
NC_040266.1	58556	T	C
NC_040266.1	113633	C	T
NC_040266.1	168019	A	G
NC_040266.1	228510	A	C
NC_040266.1	282912	C	T
NC_040266.1	345897	C	T
NC_040266.1	419029	G	T
NC_040266.1	542662	T	C
NC_040266.1	556242	C	T
NC_040266.1	628326	A	G
NC_040266.1	690094	A	G
NC_040266.1	743173	G	T
NC_040266.1	812266	C	G
NC_040266.1	865914	A	G
NC_040266.1	917348	T	C
NC_040266.1	971158	T	A
NC_040266.1	1024483	A	G
NC_040266.1	1073705	C	T
NC_040266.1	1117902	C	T
NC_040266.1	1167975	C	G
NC_040266.1	1183941	A	G
NC_040266.1	1231098	T	G
NC_040266.1	1258410	C	G
NC_040266.1	1258562	T	G
NC_040266.1	1259407	T	G
NC_040266.1	1297405	C	G
NC_040266.1	1297868	C	T
NC_040266.1	1321732	A	G
NC_040266.1	1323510	G	A
NC_040266.1	1360403	T	G
NC_040266.1	1384700	T	A
NC_040266.1	1390251	A	G
NC_040266.1	1391294	C	T
NC_040266.1	1391468	C	G
NC_040266.1	1391741	C	T
NC_040266.1	1392179	C	T
NC_040266.1	1452170	G	T
NC_040266.1	1503240	G	A
NC_040266.1	1512551	C	G
NC_040266.1	1520970	A	G
NC_040266.1	1521488	A	G
NC_040266.1	1608654	A	G
NC_040266.1	1614112	A	G
NC_040266.1	1616067	A	G
NC_040266.1	1653802	A	G

NC_040266.1	1673950	C	A
NC_040266.1	1700267	A	G
NC_040266.1	1728390	G	C
NC_040266.1	1890266	A	G
NC_040266.1	1913515	G	C
NC_040266.1	1989993	G	A
NC_040266.1	2035041	G	A
NC_040266.1	2086940	G	A
NC_040266.1	2115466	A	T
NC_040266.1	2171314	C	G
NC_040266.1	2205432	A	G
NC_040266.1	2285828	A	G
NC_040266.1	2300295	A	G
NC_040266.1	2458777	C	T
NC_040266.1	2501513	G	A
NC_040266.1	2574454	T	C
NC_040266.1	2628447	G	A
NC_040266.1	2714301	G	A
NC_040266.1	2810890	A	G
NC_040266.1	2864547	C	T
NC_040266.1	2913009	A	T
NC_040266.1	3016310	A	T
NC_040266.1	3075691	C	T
NC_040266.1	3172290	T	G
NC_040266.1	3210699	A	G
NC_040266.1	3247833	A	C
NC_040266.1	3255166	G	A
NC_040266.1	3272370	A	G
NC_040266.1	3281206	G	T
NC_040266.1	3336820	G	T
NC_040266.1	3377138	T	C
NC_040266.1	3470203	G	A
NC_040266.1	3517009	C	T
NC_040266.1	3581698	T	C
NC_040266.1	3634289	A	C
NC_040266.1	3664329	A	G
NC_040266.1	3735567	A	G
NC_040266.1	3786283	A	G
NC_040266.1	3842595	C	A
NC_040266.1	3892175	A	C
NC_040266.1	4041803	A	G
NC_040266.1	4094640	T	C
NC_040266.1	4152740	T	C
NC_040266.1	4206846	G	C
NC_040266.1	4271315	A	G
NC_040266.1	4332542	A	C
NC_040266.1	4391902	T	C

NC_040266.1	4443963	C	A
NC_040266.1	4492796	G	A
NC_040266.1	4524267	T	C
NC_040266.1	4577920	T	C
NC_040266.1	4630409	A	G
NC_040266.1	4674448	C	T
NC_040266.1	4674734	G	T
NC_040266.1	4727176	T	C
NC_040266.1	4780982	A	G
NC_040266.1	4831838	C	T
NC_040266.1	4889315	T	A
NC_040266.1	4952510	G	A
NC_040266.1	5003033	A	G
NC_040266.1	5073222	A	G
NC_040266.1	5127955	A	G
NC_040266.1	5160419	A	G
NC_040266.1	5218315	G	A
NC_040266.1	5272682	G	A
NC_040266.1	5443509	C	G
NC_040266.1	5482470	G	T
NC_040266.1	5514304	A	G
NC_040266.1	5570283	A	G
NC_040266.1	5603792	A	G
NC_040266.1	5627724	G	A
NC_040266.1	5649542	G	A
NC_040266.1	5679787	A	G
NC_040266.1	5738838	A	G
NC_040266.1	5789059	C	T
NC_040266.1	5828270	A	G
NC_040266.1	5875178	A	C
NC_040266.1	5916009	G	T
NC_040266.1	5918247	C	T
NC_040266.1	5972004	G	A
NC_040266.1	6008363	C	T
NC_040266.1	6010760	T	C
NC_040266.1	6027802	A	G
NC_040266.1	6065123	G	A
NC_040266.1	6096471	T	C
NC_040266.1	6146484	G	A
NC_040266.1	6180361	A	G
NC_040266.1	6250596	C	T
NC_040266.1	6286429	T	C
NC_040266.1	6309663	C	T
NC_040266.1	6376802	A	G
NC_040266.1	6442545	G	A
NC_040266.1	6655464	A	G
NC_040266.1	6660219	G	A

NC_040266.1	6691914	A	C
NC_040266.1	6710961	C	T
NC_040266.1	6712089	A	C
NC_040266.1	6714503	T	C
NC_040266.1	6757761	C	T
NC_040266.1	6758477	C	A
NC_040266.1	6813841	A	G
NC_040266.1	6864950	A	C
NC_040266.1	6868658	T	G
NC_040266.1	6887352	T	C
NC_040266.1	6924155	C	T
NC_040266.1	6974840	T	C
NC_040266.1	7003769	T	C
NC_040266.1	7036226	C	A
NC_040266.1	7036377	T	C
NC_040266.1	7061544	A	T
NC_040266.1	7103269	A	T
NC_040266.1	7138027	T	C
NC_040266.1	7170110	C	T
NC_040266.1	7170799	C	T
NC_040266.1	7214582	T	C
NC_040266.1	7250233	G	A
NC_040266.1	7300325	T	C
NC_040266.1	7367680	0	G
NC_040266.1	7457608	C	T
NC_040266.1	7475724	A	G
NC_040266.1	7669902	G	A
NC_040266.1	7736853	T	C
NC_040266.1	7782249	C	A
NC_040266.1	7828119	T	C
NC_040266.1	7871723	A	G
NC_040266.1	7932186	A	G
NC_040266.1	7985277	C	T
NC_040266.1	8030089	A	G
NC_040266.1	8070739	C	T
NC_040266.1	8118499	C	T
NC_040266.1	8247767	G	A
NC_040266.1	8292083	T	C
NC_040266.1	8331201	A	T
NC_040266.1	8367567	A	G
NC_040266.1	8367860	T	C
NC_040266.1	8368355	T	C
NC_040266.1	8368688	C	G
NC_040266.1	8406327	C	T
NC_040266.1	8452882	T	C
NC_040266.1	8453038	T	C
NC_040266.1	8476485	A	G

NC_040266.1	8514501	T	C
NC_040266.1	8516773	C	T
NC_040266.1	8571904	C	T
NC_040266.1	8626318	T	A
NC_040266.1	8682376	C	A
NC_040266.1	8739180	T	G
NC_040266.1	8790554	C	T
NC_040266.1	8846862	T	G
NC_040266.1	8910045	G	T
NC_040266.1	8967453	C	G
NC_040266.1	9024413	A	G
NC_040266.1	9076938	T	C
NC_040266.1	9136480	G	A
NC_040266.1	9179737	C	A
NC_040266.1	9236332	A	G
NC_040266.1	9291901	A	G
NC_040266.1	9321708	A	C
NC_040266.1	9379256	A	T
NC_040266.1	9443653	A	G
NC_040266.1	9495827	G	A
NC_040266.1	9555552	G	C
NC_040266.1	9613010	A	T
NC_040266.1	9656338	T	A
NC_040266.1	9687343	G	C
NC_040266.1	9727758	G	A
NC_040266.1	9754792	T	A
NC_040266.1	9872698	A	G
NC_040266.1	9920305	C	A
NC_040266.1	9974550	A	T
NC_040266.1	10034295	C	T
NC_040266.1	10094284	T	G
NC_040266.1	10149515	C	T
NC_040266.1	10210234	G	A
NC_040266.1	10264909	T	C
NC_040266.1	10313767	A	C
NC_040266.1	10361916	T	C
NC_040266.1	10426993	C	G
NC_040266.1	10480352	G	A
NC_040266.1	10542495	G	A
NC_040266.1	10614689	T	C
NC_040266.1	10721854	T	G
NC_040266.1	10777264	A	C
NC_040266.1	10837280	G	A
NC_040266.1	11011054	A	G
NC_040266.1	11065767	C	G
NC_040266.1	11114120	A	G
NC_040266.1	11161909	G	T

NC_040266.1	11209860	C	A
NC_040266.1	11257351	T	G
NC_040266.1	11310893	T	C
NC_040266.1	11348958	G	T
NC_040266.1	11400393	G	A
NC_040266.1	11584373	G	T
NC_040266.1	11666865	C	G
NC_040266.1	11712252	A	G
NC_040266.1	11780853	G	A
NC_040266.1	11834678	G	A
NC_040266.1	11920109	A	T
NC_040266.1	12101352	C	T
NC_040266.1	12153999	T	C
NC_040266.1	12209510	T	A
NC_040266.1	12265238	T	C
NC_040266.1	12320015	T	A
NC_040266.1	12347214	A	G
NC_040266.1	12438270	G	A
NC_040266.1	12488257	G	A
NC_040266.1	12544957	C	T
NC_040266.1	12607289	G	A
NC_040266.1	12633071	C	A
NC_040266.1	12687175	A	G
NC_040266.1	12716755	C	A
NC_040266.1	12894826	T	G
NC_040266.1	12969191	C	G
NC_040266.1	13086862	C	A
NC_040266.1	13157730	G	A
NC_040266.1	13234505	A	C
NC_040266.1	13290072	A	G
NC_040266.1	13375574	A	G
NC_040266.1	13521136	C	T
NC_040266.1	13601521	T	A
NC_040266.1	13674532	C	A
NC_040266.1	13702507	T	C
NC_040266.1	13871991	T	G
NC_040266.1	13908117	A	G
NC_040266.1	13967605	C	T
NC_040266.1	14021681	G	T
NC_040266.1	14086501	A	T
NC_040266.1	14417815	G	T
NC_040266.1	14501467	A	G
NC_040266.1	14554390	T	C
NC_040266.1	14588560	T	C
NC_040266.1	14643736	T	G
NC_040266.1	14688636	A	G
NC_040266.1	14739235	T	C

NC_040266.1	14772132	C	T
NC_040266.1	14846772	C	T
NC_040266.1	14947596	A	C
NC_040266.1	15004649	A	C
NC_040266.1	15047858	G	A
NC_040266.1	15095062	C	T
NC_040266.1	15152256	T	C
NC_040266.1	15196506	A	G
NC_040266.1	15225842	C	T
NC_040266.1	15227022	T	G
NC_040266.1	15228444	T	A
NC_040266.1	15257228	G	A
NC_040266.1	15261364	T	C
NC_040266.1	15309025	T	C
NC_040266.1	15364089	C	G
NC_040266.1	15420104	A	G
NC_040266.1	15473904	A	G
NC_040266.1	15530215	G	A
NC_040266.1	15585556	T	C
NC_040266.1	15638053	T	G
NC_040266.1	15666916	G	A
NC_040266.1	15668018	C	T
NC_040266.1	15668556	G	A
NC_040266.1	15722372	A	G
NC_040266.1	15773912	G	A
NC_040266.1	15818570	C	T
NC_040266.1	15830319	T	C
NC_040266.1	15890606	T	C
NC_040266.1	15891519	C	G
NC_040266.1	15950121	T	G
NC_040266.1	15995681	C	G
NC_040266.1	16168343	T	G
NC_040266.1	16222251	A	G
NC_040266.1	16250888	T	C
NC_040266.1	16355513	T	C
NC_040266.1	16423053	C	T
NC_040266.1	16489470	T	C
NC_040266.1	16544566	T	A
NC_040266.1	16599851	A	G
NC_040266.1	16656562	C	T
NC_040266.1	16722099	A	C
NC_040266.1	16786135	T	C
NC_040266.1	16789381	G	A
NC_040266.1	16790626	G	A
NC_040266.1	16791552	C	T
NC_040266.1	16851559	C	T
NC_040266.1	16883259	C	T

NC_040266.1	16943625	C	G
NC_040266.1	16986093	G	A
NC_040266.1	17029170	A	G
NC_040266.1	17064970	G	A
NC_040266.1	17126572	A	C
NC_040266.1	17144026	A	G
NC_040266.1	17181645	T	C
NC_040266.1	17233009	T	C
NC_040266.1	17260500	A	C
NC_040266.1	17307071	C	A
NC_040266.1	17368117	C	T
NC_040266.1	17418447	T	A
NC_040266.1	17487614	A	C
NC_040266.1	17490642	T	G
NC_040266.1	17492217	T	C
NC_040266.1	17532380	C	T
NC_040266.1	17593796	A	G
NC_040266.1	17646943	A	T
NC_040266.1	17697562	T	C
NC_040266.1	17736751	G	A
NC_040266.1	17803421	C	A
NC_040266.1	17852261	C	T
NC_040266.1	17884222	C	T
NC_040266.1	18245122	C	A
NC_040266.1	18361634	A	G
NC_040266.1	18419381	A	C
NC_040266.1	18486218	G	A
NC_040266.1	18530511	G	A
NC_040266.1	18580955	A	G
NC_040266.1	18585931	T	C
NC_040266.1	18617945	G	C
NC_040266.1	18663705	G	A
NC_040266.1	18708092	T	A
NC_040266.1	18725471	T	C
NC_040266.1	18796567	T	C
NC_040266.1	18831752	C	G
NC_040266.1	18892799	T	G
NC_040266.1	18901585	G	A
NC_040266.1	18945929	G	T
NC_040266.1	18985898	C	T
NC_040266.1	19040891	C	T
NC_040266.1	19042969	C	T
NC_040266.1	19099207	C	G
NC_040266.1	19143941	G	A
NC_040266.1	19175390	T	C
NC_040266.1	19207518	C	A
NC_040266.1	19207698	A	G

NC_040266.1	19269755	T	G
NC_040266.1	19325739	T	C
NC_040266.1	19373950	C	T
NC_040266.1	19392685	T	C
NC_040266.1	19392955	A	G
NC_040266.1	19393826	A	G
NC_040266.1	19395558	T	C
NC_040266.1	19461830	C	T
NC_040266.1	19464160	T	C
NC_040266.1	19464323	C	A
NC_040266.1	19470069	A	G
NC_040266.1	19470764	T	G
NC_040266.1	19471384	C	T
NC_040266.1	19513600	T	C
NC_040266.1	19514027	A	G
NC_040266.1	19541880	G	A
NC_040266.1	19555513	A	G
NC_040266.1	19561995	G	A
NC_040266.1	19597355	G	T
NC_040266.1	19601331	T	A
NC_040266.1	19607909	T	C
NC_040266.1	19640249	C	T
NC_040266.1	19775682	G	T
NC_040266.1	19806147	T	C
NC_040266.1	20031635	C	T
NC_040266.1	20054073	C	A
NC_040266.1	20112159	T	C
NC_040266.1	20149615	C	T
NC_040266.1	20188600	G	A
NC_040266.1	20244366	T	G
NC_040266.1	20297726	G	A
NC_040266.1	20353675	A	G
NC_040266.1	20409689	C	T
NC_040266.1	20465149	T	C
NC_040266.1	20493643	A	G
NC_040266.1	20543417	A	G
NC_040266.1	20599875	T	G
NC_040266.1	20653936	A	G
NC_040266.1	20773298	T	C
NC_040266.1	20830374	G	T
NC_040266.1	20880881	A	G
NC_040266.1	20934679	G	C
NC_040266.1	21023319	A	G
NC_040266.1	21061633	G	T
NC_040266.1	21117109	G	A
NC_040266.1	21195725	G	A
NC_040266.1	21249975	A	G

NC_040266.1	21308874	G	T
NC_040266.1	21355561	A	G
NC_040266.1	21397460	C	T
NC_040266.1	21489471	A	G
NC_040266.1	21490509	C	G
NC_040266.1	21491163	T	C
NC_040266.1	21492940	C	T
NC_040266.1	21493137	0	T
NC_040266.1	21494705	C	G
NC_040266.1	21553333	A	G
NC_040266.1	21586135	G	A
NC_040266.1	21719708	T	C
NC_040266.1	21774419	T	C
NC_040266.1	21822562	C	T
NC_040266.1	21882100	C	T
NC_040266.1	21938749	T	G
NC_040266.1	22013908	C	T
NC_040266.1	22070950	A	G
NC_040266.1	22128146	T	C
NC_040266.1	22175633	T	C
NC_040266.1	22211658	A	C
NC_040266.1	22249140	G	A
NC_040266.1	22250845	T	C
NC_040266.1	22251017	G	A
NC_040266.1	22251302	C	G
NC_040266.1	22261593	A	G
NC_040266.1	22285033	C	T
NC_040266.1	22335256	A	G
NC_040266.1	22363384	C	A
NC_040266.1	22418090	C	G
NC_040266.1	22466217	G	C
NC_040266.1	22493486	A	G
NC_040266.1	22543171	T	C
NC_040266.1	22600457	G	A
NC_040266.1	22600896	C	T
NC_040266.1	22614225	A	G
NC_040266.1	22656378	T	C
NC_040266.1	22716514	G	A
NC_040266.1	22764067	C	G
NC_040266.1	22828077	T	A
NC_040266.1	22939722	C	T
NC_040266.1	23161080	T	G
NC_040266.1	23320720	T	C
NC_040266.1	23387936	G	C
NC_040266.1	23453924	T	A
NC_040266.1	23575274	A	T
NC_040266.1	23648238	T	C

NC_040266.1	23706916	T	C
NC_040266.1	23777056	A	T
NC_040266.1	23834219	C	T
NC_040266.1	23895765	T	C
NC_040266.1	23948935	G	C
NC_040266.1	23973354	A	G
NC_040266.1	24016789	C	G
NC_040266.1	24062661	G	T
NC_040266.1	24089810	0	C
NC_040266.1	24152960	A	C
NC_040266.1	24169979	T	C
NC_040266.1	24212949	T	C
NC_040266.1	24255025	A	G
NC_040266.1	24302269	C	A
NC_040266.1	24335816	G	A
NC_040266.1	24391293	G	A
NC_040266.1	24446044	T	C
NC_040266.1	24499474	C	G
NC_040266.1	24564657	A	C
NC_040266.1	24566498	A	G
NC_040266.1	24567059	A	G
NC_040266.1	24570579	C	T
NC_040266.1	24624552	T	C
NC_040266.1	24667362	A	G
NC_040266.1	24710820	C	T
NC_040266.1	24730352	T	C
NC_040266.1	24736071	T	C
NC_040266.1	24738878	A	G
NC_040266.1	24757499	G	C
NC_040266.1	24762338	G	C
NC_040266.1	24808557	C	G
NC_040266.1	24846410	A	G
NC_040266.1	24848932	T	C
NC_040266.1	24882961	A	C
NC_040266.1	24885689	G	T
NC_040266.1	24891753	A	G
NC_040266.1	24903187	C	T
NC_040266.1	24984925	T	C
NC_040266.1	25023355	G	A
NC_040266.1	25059882	C	T
NC_040266.1	25069057	T	A
NC_040266.1	25163047	T	C
NC_040266.1	25235623	C	T
NC_040266.1	25295684	C	A
NC_040266.1	25352890	G	A
NC_040266.1	25395760	A	G
NC_040266.1	25459791	A	T

NC_040266.1	25492438	T	C
NC_040266.1	25544949	T	A
NC_040266.1	25598919	A	T
NC_040266.1	25667736	A	G
NC_040266.1	25727917	G	A
NC_040266.1	25784621	C	A
NC_040266.1	25840748	A	C
NC_040266.1	25895124	T	C
NC_040266.1	25937308	G	A
NC_040266.1	25984277	G	A
NC_040266.1	26035296	G	A
NC_040266.1	26082788	G	A
NC_040266.1	26122110	G	A
NC_040266.1	26185440	G	C
NC_040266.1	26242067	T	C
NC_040266.1	26296408	A	G
NC_040266.1	26349984	A	G
NC_040266.1	26351537	A	G
NC_040266.1	26409156	A	G
NC_040266.1	26440299	T	G
NC_040266.1	26501692	T	C
NC_040266.1	26563887	T	G
NC_040266.1	26613732	G	A
NC_040266.1	26668099	A	G
NC_040266.1	26697399	T	C
NC_040266.1	26744814	A	G
NC_040266.1	26784602	C	T
NC_040266.1	26842868	T	C
NC_040266.1	26882803	C	T
NC_040266.1	26937023	T	A
NC_040266.1	27000603	T	C
NC_040266.1	27058321	C	T
NC_040266.1	27134509	T	C
NC_040266.1	27190554	A	G
NC_040266.1	27246138	G	A
NC_040266.1	27309405	A	G
NC_040266.1	27361134	T	C
NC_040266.1	27416817	A	G
NC_040266.1	27469819	G	A
NC_040266.1	27532084	A	G
NC_040266.1	27609185	G	A
NC_040266.1	27651284	C	T
NC_040266.1	27721070	C	T
NC_040266.1	27748341	A	G
NC_040266.1	27801849	G	A
NC_040266.1	27857420	A	G
NC_040266.1	27895886	T	C

NC_040266.1	27948205	T	C
NC_040266.1	27996743	A	G
NC_040266.1	28055667	T	C
NC_040266.1	28118419	T	C
NC_040266.1	28172456	T	C
NC_040266.1	28228707	T	G
NC_040266.1	28270034	A	G
NC_040266.1	28330409	C	T
NC_040266.1	28374431	T	C
NC_040266.1	28431943	T	C
NC_040266.1	28486313	T	C
NC_040266.1	28540551	C	T
NC_040266.1	28586504	T	C
NC_040266.1	28621878	A	G
NC_040266.1	28675915	A	G
NC_040266.1	28727365	T	C
NC_040266.1	28781929	A	G
NC_040266.1	28836916	G	A
NC_040266.1	28892678	G	A
NC_040266.1	28955742	T	G
NC_040266.1	29011820	T	C
NC_040266.1	29081656	G	T
NC_040266.1	29131603	G	A
NC_040266.1	29170889	T	C
NC_040266.1	29240123	G	A
NC_040266.1	29373884	G	A
NC_040266.1	29429959	A	C
NC_040266.1	29488550	A	G
NC_040266.1	29547515	A	G
NC_040266.1	29602310	T	C
NC_040266.1	29660502	C	T
NC_040266.1	29720404	T	C
NC_040266.1	29778810	G	C
NC_040266.1	29832495	G	T
NC_040266.1	29897330	T	G
NC_040266.1	29951958	G	T
NC_040266.1	30010443	C	T
NC_040266.1	30070347	C	G
NC_040266.1	30115217	G	A
NC_040266.1	30154150	T	C
NC_040266.1	30191721	C	T
NC_040266.1	30214105	G	A
NC_040266.1	30220379	T	C
NC_040266.1	30225267	T	C
NC_040266.1	30234564	T	C
NC_040266.1	30290498	T	C
NC_040266.1	30342498	C	T

NC_040266.1	30377716	G	A
NC_040266.1	30416754	G	A
NC_040266.1	30524275	A	G
NC_040266.1	30588482	T	C
NC_040266.1	30654991	C	T
NC_040266.1	30671778	A	G
NC_040266.1	30696861	A	G
NC_040266.1	30705824	A	G
NC_040266.1	30755322	A	C
NC_040266.1	30777909	C	T
NC_040266.1	30786760	T	C
NC_040266.1	30843463	A	G
NC_040266.1	30904165	T	C
NC_040266.1	30945195	T	C
NC_040266.1	30976282	T	C
NC_040266.1	30987304	T	C
NC_040266.1	31043746	T	G
NC_040266.1	31100719	G	C
NC_040266.1	31158229	C	A
NC_040266.1	31215203	A	G
NC_040266.1	31266610	A	G
NC_040266.1	31323914	T	G
NC_040266.1	31339342	G	C
NC_040266.1	31387636	G	A
NC_040266.1	31417386	T	C
NC_040266.1	31480208	T	C
NC_040266.1	31490248	C	T
NC_040266.1	31541551	C	T
NC_040266.1	31579265	A	G
NC_040266.1	31616346	G	A
NC_040266.1	31678100	T	C
NC_040266.1	31721890	A	G
NC_040266.1	31732053	C	T
NC_040266.1	31734345	G	A
NC_040266.1	31766357	A	G
NC_040266.1	31829600	T	C
NC_040266.1	31837607	G	A
NC_040266.1	31883172	T	C
NC_040266.1	31899639	G	A
NC_040266.1	31936712	T	C
NC_040266.1	31962492	C	T
NC_040266.1	31963797	A	G
NC_040266.1	31990001	T	A
NC_040266.1	31990995	C	A
NC_040266.1	32007315	T	G
NC_040266.1	32016279	A	G
NC_040266.1	32019789	T	G

NC_040266.1	32037460	C	T
NC_040266.1	32078129	G	A
NC_040266.1	32096138	T	C
NC_040266.1	32096729	T	C
NC_040266.1	32106666	T	C
NC_040266.1	32109136	C	T
NC_040266.1	32137114	A	G
NC_040266.1	32180135	A	G
NC_040266.1	32180735	A	G
NC_040266.1	32210653	C	T
NC_040266.1	32251839	A	G
NC_040266.1	32292991	G	T
NC_040266.1	32304911	G	A
NC_040266.1	32306127	T	C
NC_040266.1	32307641	T	C
NC_040266.1	32308544	A	G
NC_040266.1	32330352	T	C
NC_040266.1	32374295	G	A
NC_040266.1	32405131	T	G
NC_040266.1	32411358	A	G
NC_040266.1	32443547	A	G
NC_040266.1	32444740	T	C
NC_040266.1	32447360	C	A
NC_040266.1	32448759	G	A
NC_040266.1	32452015	T	C
NC_040266.1	32452519	C	G
NC_040266.1	32480504	A	G
NC_040266.1	32486644	T	C
NC_040266.1	32503641	T	C
NC_040266.1	32511404	T	C
NC_040266.1	32519864	A	G
NC_040266.1	32536670	C	G
NC_040266.1	32542145	T	C
NC_040266.1	32569684	C	T
NC_040266.1	32574360	A	G
NC_040266.1	32577498	G	C
NC_040266.1	32582885	A	C
NC_040266.1	32583463	T	A
NC_040266.1	32628960	A	G
NC_040266.1	32668383	T	C
NC_040266.1	32670776	T	C
NC_040266.1	32672710	A	C
NC_040266.1	32675151	T	C
NC_040266.1	32675960	G	T
NC_040266.1	32676671	A	G
NC_040266.1	32679774	T	C
NC_040266.1	32682655	T	C

NC_040266.1	32684801	T	C
NC_040266.1	32708631	G	C
NC_040266.1	32741439	C	A
NC_040266.1	32774200	C	T
NC_040266.1	32827255	A	T
NC_040266.1	32886872	T	C
NC_040266.1	32955651	T	C
NC_040266.1	33008672	A	G
NC_040266.1	33011645	G	T
NC_040266.1	33061321	T	C
NC_040266.1	33079334	C	T
NC_040266.1	33137033	G	C
NC_040266.1	33189003	C	A
NC_040266.1	33252985	T	C
NC_040266.1	33323093	G	C
NC_040266.1	33382438	T	C
NC_040266.1	33440018	C	G
NC_040266.1	33483564	G	A
NC_040266.1	33499485	T	C
NC_040266.1	33583690	T	G
NC_040266.1	33640834	A	C
NC_040266.1	33702201	T	C
NC_040266.1	33714198	C	T
NC_040266.1	33715158	T	C
NC_040266.1	33717113	G	A
NC_040266.1	33772292	G	T
NC_040266.1	33810819	A	G
NC_040266.1	33880794	G	A
NC_040266.1	33881956	A	G
NC_040266.1	33937443	A	G
NC_040266.1	33994392	T	C
NC_040266.1	34055197	A	G
NC_040266.1	34120290	C	T
NC_040266.1	34175660	T	C
NC_040266.1	34231624	C	T
NC_040266.1	34291629	T	C
NC_040266.1	34346770	G	A
NC_040266.1	34404392	A	G
NC_040266.1	34412738	C	G
NC_040266.1	34455250	G	A
NC_040266.1	34486464	C	T
NC_040266.1	34521988	T	C
NC_040266.1	34544694	A	C
NC_040266.1	34594841	C	A
NC_040266.1	34659920	G	T
NC_040266.1	34712645	G	C
NC_040266.1	34766939	A	C

NC_040266.1	34828754	A	G
NC_040266.1	34887272	T	C
NC_040266.1	34940771	G	A
NC_040266.1	34987346	T	G
NC_040266.1	35017442	G	A
NC_040266.1	35048656	T	C
NC_040266.1	35123058	A	G
NC_040266.1	35180003	C	T
NC_040266.1	35233959	T	C
NC_040266.1	35295177	G	T
NC_040266.1	35350940	C	G
NC_040266.1	35401562	A	G
NC_040266.1	35460793	C	T
NC_040266.1	35522104	T	C
NC_040266.1	35577349	A	C
NC_040266.1	35639354	T	C
NC_040266.1	35696852	T	C
NC_040266.1	35749975	C	T
NC_040266.1	35807149	T	C
NC_040266.1	35863729	C	G
NC_040266.1	35929482	A	G
NC_040266.1	35982600	G	T
NC_040266.1	36033961	T	C
NC_040266.1	36082133	A	C
NC_040266.1	36124180	A	G
NC_040266.1	36179205	A	T
NC_040266.1	36230435	A	G
NC_040266.1	36275780	A	G
NC_040266.1	36307658	T	A
NC_040266.1	36419186	C	T
NC_040266.1	36463869	G	C
NC_040266.1	36489123	C	G
NC_040266.1	36523294	A	G
NC_040266.1	36575180	T	C
NC_040266.1	36576141	C	T
NC_040266.1	36631400	A	G
NC_040266.1	36674279	A	C
NC_040266.1	36734202	C	T
NC_040266.1	36805260	C	T
NC_040266.1	36868989	A	G
NC_040266.1	36919106	C	T
NC_040266.1	36929294	G	A
NC_040266.1	36982486	T	C
NC_040266.1	37029785	A	G
NC_040266.1	37030304	A	G
NC_040266.1	37031350	C	T
NC_040266.1	37085307	T	C

NC_040266.1	37125283	A	G
NC_040266.1	37125998	T	C
NC_040266.1	37127361	C	T
NC_040266.1	37132533	T	G
NC_040266.1	37133746	A	C
NC_040266.1	37157338	G	A
NC_040266.1	37210002	C	G
NC_040266.1	37210948	C	G
NC_040266.1	37215572	A	G
NC_040266.1	37279693	A	G
NC_040266.1	37302761	T	C
NC_040266.1	37345067	C	T
NC_040266.1	37361781	C	A
NC_040266.1	37368730	G	C
NC_040266.1	37422284	A	G
NC_040266.1	37477860	T	C
NC_040266.1	37530506	T	A
NC_040266.1	37574652	T	C
NC_040266.1	37636127	G	A
NC_040266.1	37646114	C	T
NC_040266.1	37652973	A	G
NC_040266.1	37702299	G	A
NC_040266.1	37775906	A	G
NC_040266.1	37796952	T	C
NC_040266.1	37822724	C	T
NC_040266.1	37837797	A	G
NC_040266.1	37894733	G	A
NC_040266.1	37950083	G	A
NC_040266.1	37986992	A	G
NC_040266.1	38017308	G	C
NC_040266.1	38019241	T	C
NC_040266.1	38066137	G	T
NC_040266.1	38093112	C	A
NC_040266.1	38154267	C	T
NC_040266.1	38194631	G	A
NC_040266.1	38228442	A	G
NC_040266.1	38264935	C	T
NC_040266.1	38305329	C	T
NC_040266.1	38306963	G	T
NC_040266.1	38361960	C	A
NC_040266.1	38420424	T	C
NC_040266.1	38476008	C	T
NC_040266.1	38535708	T	C
NC_040266.1	38571106	C	T
NC_040266.1	38573007	A	G
NC_040266.1	38586543	C	T
NC_040266.1	38612700	T	C

NC_040266.1	38671823	C	T
NC_040266.1	38710532	A	G
NC_040266.1	38711494	G	C
NC_040266.1	38770044	A	G
NC_040266.1	38824247	T	C
NC_040266.1	38933670	A	T
NC_040266.1	38985935	G	A
NC_040266.1	39039727	G	T
NC_040266.1	39091514	C	T
NC_040266.1	39147068	C	G
NC_040266.1	39203021	T	C
NC_040266.1	39248891	C	T
NC_040266.1	39308667	G	A
NC_040266.1	39364667	G	A
NC_040266.1	39420404	A	G
NC_040266.1	39422955	A	G
NC_040266.1	39424834	G	A
NC_040266.1	39475646	A	G
NC_040266.1	39536013	C	T
NC_040266.1	39577587	A	T
NC_040266.1	39635422	G	T
NC_040266.1	39691956	G	A
NC_040266.1	39749496	T	G
NC_040266.1	39805138	A	G
NC_040266.1	39860292	C	A
NC_040266.1	39906268	T	C
NC_040266.1	39967232	T	C
NC_040266.1	40033698	G	A
NC_040266.1	40094006	G	A
NC_040266.1	40147458	C	T
NC_040266.1	40201203	C	T
NC_040266.1	40250104	T	C
NC_040266.1	40303108	0	T
NC_040266.1	40333703	A	G
NC_040266.1	40377063	A	C
NC_040266.1	40410784	C	G
NC_040266.1	40439307	T	C
NC_040266.1	40493367	T	G
NC_040266.1	40520620	G	A
NC_040266.1	40576879	T	C
NC_040266.1	40647201	T	C
NC_040266.1	40810167	G	A
NC_040266.1	40877222	A	G
NC_040266.1	40933656	T	C
NC_040266.1	40986191	A	G
NC_040266.1	41043823	T	C
NC_040266.1	41109261	T	C

NC_040266.1	41168714	G	T
NC_040266.1	41236072	T	G
NC_040266.1	41277484	G	A
NC_040266.1	41286541	G	A
NC_040266.1	41332692	T	C
NC_040266.1	41383018	T	C
NC_040266.1	41428351	T	C
NC_040266.1	41460855	G	A
NC_040266.1	41473675	C	T
NC_040266.1	41474642	G	T
NC_040266.1	41527996	C	T
NC_040266.1	41581445	G	T
NC_040266.1	41628712	T	C
NC_040266.1	41685452	G	A
NC_040266.1	41741998	C	T
NC_040266.1	41787145	A	G
NC_040266.1	41846153	G	A
NC_040266.1	41888680	A	G
NC_040266.1	41943570	C	A
NC_040266.1	42001502	C	G
NC_040266.1	42003618	G	T
NC_040266.1	42004200	C	T
NC_040266.1	42022518	A	G
NC_040266.1	42079707	A	G
NC_040266.1	42113082	C	T
NC_040266.1	42162135	A	G
NC_040266.1	42212761	T	C
NC_040266.1	42246507	T	G
NC_040266.1	42283403	G	A
NC_040266.1	42322376	A	G
NC_040266.1	42350710	A	G
NC_040266.1	42401428	C	T
NC_040266.1	42404318	A	T
NC_040266.1	42430994	C	T
NC_040266.1	42493822	C	A
NC_040266.1	42557700	T	C
NC_040266.1	42617181	C	G
NC_040266.1	42683499	G	A
NC_040266.1	42734092	C	T
NC_040266.1	42736826	C	A
NC_040266.1	42795597	A	G
NC_040266.1	42798723	A	G
NC_040266.1	42802682	T	C
NC_040266.1	42872643	A	G
NC_040266.1	42940499	G	C
NC_040266.1	42993703	C	T
NC_040266.1	43030488	A	G

NC_040266.1	43060008	A	C
NC_040266.1	43123284	G	A
NC_040266.1	43182113	A	G
NC_040266.1	43216809	T	C
NC_040266.1	43267457	A	T
NC_040266.1	43321925	A	G
NC_040266.1	43371962	T	C
NC_040266.1	43431730	A	G
NC_040266.1	43481911	T	C
NC_040266.1	43522646	A	G
NC_040266.1	43578107	A	G
NC_040266.1	43635829	A	C
NC_040266.1	43714779	C	G
NC_040266.1	43764572	A	G
NC_040266.1	43765432	A	G
NC_040266.1	43770688	A	C
NC_040266.1	43807972	A	G
NC_040266.1	43839644	C	T
NC_040266.1	43865005	A	G
NC_040266.1	43931524	T	C
NC_040266.1	43982430	A	G
NC_040266.1	44041588	G	A
NC_040266.1	44094081	T	C
NC_040266.1	44139971	G	A
NC_040266.1	44200634	G	A
NC_040266.1	44255763	T	C
NC_040266.1	44313049	T	G
NC_040266.1	44372135	T	C
NC_040266.1	44413597	T	C
NC_040266.1	44446254	T	C
NC_040266.1	44504511	C	T
NC_040266.1	44556834	C	T
NC_040266.1	44602904	C	T
NC_040266.1	44662397	G	A
NC_040266.1	44714300	A	G
NC_040266.1	44826046	A	G
NC_040266.1	44874316	C	G
NC_040266.1	44877490	A	G
NC_040266.1	44929354	C	G
NC_040266.1	44970070	A	G
NC_040266.1	45033570	C	A
NC_040266.1	45090434	T	C
NC_040266.1	45137638	C	T
NC_040266.1	45138884	T	C
NC_040266.1	45185905	A	T
NC_040266.1	45219644	C	T
NC_040266.1	45233822	A	G

NC_040266.1	45271787	T	G
NC_040266.1	45330979	T	C
NC_040266.1	45386598	A	G
NC_040266.1	45427295	A	C
NC_040266.1	45482328	A	C
NC_040266.1	45646480	G	A
NC_040266.1	45701930	G	A
NC_040266.1	45761153	C	T
NC_040266.1	45815806	C	T
NC_040266.1	45871940	A	T
NC_040266.1	45925855	T	C
NC_040266.1	45973617	G	A
NC_040266.1	46005025	C	T
NC_040266.1	46034275	T	G
NC_040266.1	46038448	G	C
NC_040266.1	46090082	A	G
NC_040266.1	46124872	A	G
NC_040266.1	46178095	T	C
NC_040266.1	46228766	A	G
NC_040266.1	46268898	C	G
NC_040266.1	46325665	A	T
NC_040266.1	46373239	G	T
NC_040266.1	46426855	G	C
NC_040266.1	46464164	T	C
NC_040266.1	46508351	C	G
NC_040266.1	46558586	C	A
NC_040266.1	46606605	T	C
NC_040266.1	46660536	T	C
NC_040266.1	46717315	G	A
NC_040266.1	46753880	C	A
NC_040266.1	46806804	C	T
NC_040266.1	46815659	G	C
NC_040266.1	46827417	G	T
NC_040266.1	46868203	T	C
NC_040266.1	46896362	T	C
NC_040266.1	46950739	A	G
NC_040266.1	47007858	G	C
NC_040266.1	47049732	C	T
NC_040266.1	47066967	T	C
NC_040266.1	47087952	T	C
NC_040266.1	47088391	A	T
NC_040266.1	47108544	T	G
NC_040266.1	47152712	A	G
NC_040266.1	47206774	C	G
NC_040266.1	47264235	T	G
NC_040266.1	47298991	T	C
NC_040266.1	47361915	A	T

NC_040266.1	47406120	T	C
NC_040266.1	47437244	C	T
NC_040266.1	47475357	G	A
NC_040266.1	47500012	A	G
NC_040266.1	47550935	T	C
NC_040266.1	47576348	T	C
NC_040266.1	47577025	A	G
NC_040266.1	47579339	A	G
NC_040266.1	47582783	G	T
NC_040266.1	47631640	T	G
NC_040266.1	47680314	G	A
NC_040266.1	47680812	C	T
NC_040266.1	47746539	T	G
NC_040266.1	47785729	C	T
NC_040266.1	47826283	T	G
NC_040266.1	47872344	C	T
NC_040266.1	47934959	A	T
NC_040266.1	47976728	A	G
NC_040266.1	48032332	G	T
NC_040266.1	48084653	T	G
NC_040266.1	48107372	T	C
NC_040266.1	48164368	T	C
NC_040266.1	48167250	A	G
NC_040266.1	48222503	G	T
NC_040266.1	48278249	C	T
NC_040266.1	48336887	A	G
NC_040266.1	48396263	C	G
NC_040266.1	48437238	T	C
NC_040266.1	48467692	C	A
NC_040266.1	48529122	A	G
NC_040266.1	48582236	T	C
NC_040266.1	48634412	G	A
NC_040266.1	48683899	T	C
NC_040266.1	48753187	G	T
NC_040266.1	48796531	A	G
NC_040266.1	48856027	C	T
NC_040266.1	48871566	T	C
NC_040266.1	48892985	T	C
NC_040266.1	48893495	T	A
NC_040266.1	48968934	G	A
NC_040266.1	49010337	C	T
NC_040266.1	49022677	T	C
NC_040266.1	49077510	T	C
NC_040266.1	49147947	C	T
NC_040266.1	49191345	A	G
NC_040266.1	49464823	C	T
NC_040266.1	49560191	T	A

NC_040266.1	49570092	C	T
NC_040266.1	49606505	T	C
NC_040266.1	49615673	C	G
NC_040266.1	49617179	T	C
NC_040266.1	49625353	T	C
NC_040266.1	49630205	A	C
NC_040266.1	49630833	T	C
NC_040266.1	49634586	T	A
NC_040266.1	49638281	C	T
NC_040266.1	49640247	A	G
NC_040266.1	49648032	A	G
NC_040266.1	49650880	A	T
NC_040266.1	49651464	T	C
NC_040266.1	49676781	A	T
NC_040266.1	49683382	T	C
NC_040266.1	49689347	T	C
NC_040266.1	49690997	T	C
NC_040266.1	49697197	G	C
NC_040266.1	49698726	T	C
NC_040266.1	49699088	G	A
NC_040266.1	49700425	C	G
NC_040266.1	49707210	T	C
NC_040266.1	49737917	C	T
NC_040266.1	49749100	T	C
NC_040266.1	49751386	T	C
NC_040266.1	49756230	A	G
NC_040266.1	49776657	C	G
NC_040266.1	49784201	T	C
NC_040266.1	49825614	A	G
NC_040266.1	49835773	G	A
NC_040266.1	49838802	T	C
NC_040266.1	49894702	T	C
NC_040266.1	49947440	T	A
NC_040266.1	49975865	G	A
NC_040266.1	49982023	G	A
NC_040266.1	50000511	A	G
NC_040266.1	50007020	T	C
NC_040266.1	50008889	A	G
NC_040266.1	50017661	G	A
NC_040266.1	50072233	T	G
NC_040266.1	50103657	G	A
NC_040266.1	50131434	C	T
NC_040266.1	50192139	T	A
NC_040266.1	50258089	C	T
NC_040266.1	50279543	A	G
NC_040266.1	50561711	A	G
NC_040266.1	50701364	C	G

NC_040266.1	50706833	T	C
NC_040266.1	50898536	A	G
NC_040266.1	51031729	A	C
NC_040266.1	51380472	A	G
NC_040266.1	51382618	T	C
NC_040266.1	51383761	T	C
NC_040266.1	51443348	G	A
NC_040266.1	51498601	G	T
NC_040266.1	51499078	A	G
NC_040266.1	51558796	C	T
NC_040266.1	51564819	G	A
NC_040266.1	51587611	T	A
NC_040266.1	51642679	T	C
NC_040266.1	51706412	C	T
NC_040266.1	51777580	T	A
NC_040266.1	51844838	A	G
NC_040266.1	51971053	T	C
NC_040266.1	52104167	A	G
NC_040266.1	52157004	G	A
NC_040266.1	52229617	T	C
NC_040266.1	52264283	T	C
NC_040266.1	52288416	T	C
NC_040266.1	52367259	G	A
NC_040266.1	52401725	T	C
NC_040266.1	52429085	A	G
NC_040266.1	52480093	A	G
NC_040266.1	52635161	A	G
NC_040266.1	52700476	T	G
NC_040266.1	52755389	C	G
NC_040266.1	52795671	C	T
NC_040266.1	52838088	A	T
NC_040266.1	52911331	G	A
NC_040266.1	52942911	T	C
NC_040266.1	52983888	T	C
NC_040266.1	53034995	T	C
NC_040266.1	53143380	A	G
NC_040266.1	53493884	A	G
NC_040266.1	53551725	A	G
NC_040266.1	53562652	A	G
NC_040266.1	53601217	A	G
NC_040266.1	53651802	T	C
NC_040266.1	53714381	A	C
NC_040266.1	53751048	A	G
NC_040266.1	53822415	A	G
NC_040266.1	53879785	T	A
NC_040266.1	53931165	T	C
NC_040266.1	54025016	G	C

NC_040266.1	54060513	A	G
NC_040266.1	54087954	A	G
NC_040266.1	54095800	G	A
NC_040266.1	54143267	C	T
NC_040266.1	54199450	T	C
NC_040266.1	54240244	G	A
NC_040266.1	54283452	T	G
NC_040266.1	54304645	C	T
NC_040266.1	54315219	C	A
NC_040266.1	54315778	C	T
NC_040266.1	54347057	T	C
NC_040266.1	54395988	C	G
NC_040266.1	54415151	A	G
NC_040266.1	54478552	G	A
NC_040266.1	54485427	T	C
NC_040266.1	54518916	G	A
NC_040266.1	54558050	A	C
NC_040266.1	54611216	A	G
NC_040266.1	54642611	C	T
NC_040266.1	54645817	A	C
NC_040266.1	54657807	A	G
NC_040266.1	54659369	T	C
NC_040266.1	54660291	A	G
NC_040266.1	54687585	C	G
NC_040266.1	54732052	A	G
NC_040266.1	54733118	T	C
NC_040266.1	54743291	A	G
NC_040266.1	54796260	G	C
NC_040266.1	54831305	T	C
NC_040266.1	54883526	G	A
NC_040266.1	54884179	A	G
NC_040266.1	54937779	A	G
NC_040266.1	54990191	C	G
NC_040266.1	55024533	C	T
NC_040266.1	55080038	T	C
NC_040266.1	55142184	A	G
NC_040266.1	55144160	A	G
NC_040266.1	55199913	T	C
NC_040266.1	55264909	A	G
NC_040266.1	55277492	C	A
NC_040266.1	55328345	A	G
NC_040266.1	55382384	T	C
NC_040266.1	55441460	G	A
NC_040266.1	55501187	T	A
NC_040266.1	55565674	T	C
NC_040266.1	55614571	C	A
NC_040266.1	55666348	G	T

NC_040266.1	55707959	A	G
NC_040266.1	55758943	A	G
NC_040266.1	55770303	T	C
NC_040266.1	55787809	A	G
NC_040266.1	55788214	T	C
NC_040266.1	55796752	G	T
NC_040266.1	55797221	T	C
NC_040266.1	55798395	T	C
NC_040266.1	55799049	C	T
NC_040266.1	55839817	G	C
NC_040266.1	55842665	T	C
NC_040266.1	55852795	A	G
NC_040266.1	55853724	C	A
NC_040266.1	55873299	T	C
NC_040266.1	55877725	G	A
NC_040266.1	55878165	C	T
NC_040266.1	55881817	A	C
NC_040266.1	55882993	T	C
NC_040266.1	55884111	A	C
NC_040266.1	55884966	G	A
NC_040266.1	55937357	G	A
NC_040266.1	55990033	C	T
NC_040266.1	56157851	G	A
NC_040266.1	56204757	G	A
NC_040266.1	56273485	T	C
NC_040266.1	56329208	A	T
NC_040266.1	56389991	T	C
NC_040266.1	56433757	A	T
NC_040266.1	56480415	T	C
NC_040266.1	56523116	A	G
NC_040266.1	56574963	C	G
NC_040266.1	56625874	G	A
NC_040266.1	56683892	T	C
NC_040266.1	56734788	C	T
NC_040266.1	56745202	A	G
NC_040266.1	56795241	T	G
NC_040266.1	56816749	C	T
NC_040266.1	56859819	A	G
NC_040266.1	56916530	G	A
NC_040266.1	56942143	A	C
NC_040266.1	57012164	G	A
NC_040266.1	57053402	A	G
NC_040266.1	57116251	T	C
NC_040266.1	57172934	A	G
NC_040266.1	57233383	T	C
NC_040266.1	57254400	C	T
NC_040266.1	57256577	G	C

NC_040266.1	57257171	G	C
NC_040266.1	57259985	C	A
NC_040266.1	57263153	A	G
NC_040266.1	57313177	C	T
NC_040266.1	57342755	T	C
NC_040266.1	57369786	T	G
NC_040266.1	57373247	A	G
NC_040266.1	57382866	T	A
NC_040266.1	57456133	T	G
NC_040266.1	57495450	T	C
NC_040266.1	57524838	A	G
NC_040266.1	57533046	T	C
NC_040266.1	57573182	G	C
NC_040266.1	57585786	G	T
NC_040266.1	57638980	C	A
NC_040266.1	57658896	A	G
NC_040266.1	57714827	A	G
NC_040266.1	57767355	T	C
NC_040266.1	57809029	T	C
NC_040266.1	57850420	T	C
NC_040266.1	57887822	C	T
NC_040266.1	57937900	C	T
NC_040266.1	57994329	A	G
NC_040266.1	58025171	C	T
NC_040266.1	58131356	C	T
NC_040266.1	58191351	C	T
NC_040266.1	58216394	G	C
NC_040266.1	58216883	T	A
NC_040266.1	58280899	C	T
NC_040266.1	58337919	A	G
NC_040266.1	58395282	A	G
NC_040266.1	58421425	A	T
NC_040266.1	58468524	C	T
NC_040266.1	58524409	C	T
NC_040266.1	58578472	G	A
NC_040266.1	58635627	G	T
NC_040266.1	58686807	T	G
NC_040266.1	58871784	T	C
NC_040266.1	58921835	T	G
NC_040266.1	58980467	A	G
NC_040266.1	59037829	A	G
NC_040266.1	59078216	C	G
NC_040266.1	59130268	C	A
NC_040266.1	59192798	G	A
NC_040266.1	59240424	T	C
NC_040266.1	59292745	T	A
NC_040266.1	59347782	T	C

NC_040266.1	59358466	A	C
NC_040266.1	59359362	C	G
NC_040266.1	59405346	G	A
NC_040266.1	59510863	A	G
NC_040266.1	59542475	T	C
NC_040266.1	59554432	T	C
NC_040266.1	59609887	A	G
NC_040266.1	59657403	G	A
NC_040266.1	59716328	G	A
NC_040266.1	59754342	G	A
NC_040266.1	59810692	T	C
NC_040266.1	59826164	A	G
NC_040266.1	59887821	G	T
NC_040266.1	59968429	C	T
NC_040266.1	60095305	G	A
NC_040266.1	60170013	G	A
NC_040266.1	60207178	T	C
NC_040266.1	60260509	A	G
NC_040266.1	60303832	G	T
NC_040266.1	60334497	G	T
NC_040266.1	60386088	A	G
NC_040266.1	60415803	C	T
NC_040266.1	60476456	G	T
NC_040266.1	60532633	T	C
NC_040266.1	60564497	C	T
NC_040266.1	60612295	A	G
NC_040266.1	60780457	C	T
NC_040266.1	60846011	A	G
NC_040266.1	60915098	A	G
NC_040266.1	60941829	C	T
NC_040266.1	61005094	G	A
NC_040266.1	61055514	C	T
NC_040266.1	61132921	C	T
NC_040266.1	61190693	A	T
NC_040266.1	61254428	C	G
NC_040266.1	61320272	G	A
NC_040266.1	61343001	T	C
NC_040266.1	61369554	T	C
NC_040266.1	61430841	A	C
NC_040266.1	61489712	A	G
NC_040266.1	61543374	T	C
NC_040266.1	61588779	A	G
NC_040266.1	61650243	A	G
NC_040266.1	61703488	A	G
NC_040266.1	61742080	A	C
NC_040266.1	61763632	G	T
NC_040266.1	61764771	G	A

NC_040266.1	61819180	A	T
NC_040266.1	61874779	T	C
NC_040266.1	61933360	A	T
NC_040266.1	61990122	T	C
NC_040266.1	62046577	T	C
NC_040266.1	62107471	G	A
NC_040266.1	62179994	T	G
NC_040266.1	62306884	T	C
NC_040266.1	62360553	G	A
NC_040266.1	62405639	T	C
NC_040266.1	62458417	G	C
NC_040266.1	62475684	G	C
NC_040266.1	62476364	G	A
NC_040266.1	62527536	T	C
NC_040266.1	62582075	A	C
NC_040266.1	62615664	T	C
NC_040266.1	62668564	G	T
NC_040266.1	62726786	G	A
NC_040266.1	62771824	T	C
NC_040266.1	62829857	C	T
NC_040266.1	62885726	A	G
NC_040266.1	62940730	T	C
NC_040266.1	62999108	A	C
NC_040266.1	63057467	C	A
NC_040266.1	63117355	C	T
NC_040266.1	63186892	C	T
NC_040266.1	63286667	G	A
NC_040266.1	63346774	C	A
NC_040266.1	63411196	T	C
NC_040266.1	63443405	G	A
NC_040266.1	63499521	T	C
NC_040266.1	63567213	A	T
NC_040266.1	63621547	T	C
NC_040266.1	63683011	C	T
NC_040266.1	63742392	A	G
NC_040266.1	63796693	A	G
NC_040266.1	63853293	C	T
NC_040266.1	63906289	A	G
NC_040266.1	63961776	T	C
NC_040266.1	64025535	A	G
NC_040266.1	64073844	C	T
NC_040266.1	64232899	G	A
NC_040266.1	64291700	T	C
NC_040266.1	64341191	T	C
NC_040266.1	64396335	T	C
NC_040266.1	64442868	A	G
NC_040266.1	64466543	G	A

NC_040266.1	64513301	A	G
NC_040266.1	64560487	C	T
NC_040266.1	64561291	A	G
NC_040266.1	64563646	A	C
NC_040266.1	64591331	T	G
NC_040266.1	64660771	T	G
NC_040266.1	65829472	A	G
NC_040266.1	65887063	A	C
NC_040266.1	65943512	C	T
NC_040266.1	66004145	A	G
NC_040266.1	66050354	C	T
NC_040266.1	66119891	A	G
NC_040266.1	66167159	G	A
NC_040266.1	66209543	A	G
NC_040266.1	66265764	C	T
NC_040266.1	66315883	T	C
NC_040266.1	66371350	T	C
NC_040266.1	66416265	C	T
NC_040266.1	66476553	G	C
NC_040266.1	66535385	T	C
NC_040266.1	66591282	G	A
NC_040266.1	66662622	C	T
NC_040266.1	66721145	T	C
NC_040266.1	66763852	G	A
NC_040266.1	66766034	T	C
NC_040266.1	66822121	T	C
NC_040266.1	66871713	C	T
NC_040266.1	66903519	T	C
NC_040266.1	66931863	T	G
NC_040266.1	66989076	A	G
NC_040266.1	67038921	A	C
NC_040266.1	67089090	A	C
NC_040266.1	67143010	A	G
NC_040266.1	67196005	C	T
NC_040266.1	67253586	T	C
NC_040266.1	67305418	C	T
NC_040266.1	67362995	A	G
NC_040266.1	67401265	A	G
NC_040266.1	67459826	T	C
NC_040266.1	67513164	A	C
NC_040266.1	67571353	G	T
NC_040266.1	67601764	A	G
NC_040266.1	67602637	C	T
NC_040266.1	67623940	C	A
NC_040266.1	67682489	G	C
NC_040266.1	67743178	T	C
NC_040266.1	67797990	C	T

NC_040266.1	67853030	A	G
NC_040266.1	67899780	G	A
NC_040266.1	67976425	G	T
NC_040266.1	68028842	C	T
NC_040266.1	68087510	C	T
NC_040266.1	68117546	T	G
NC_040266.1	68172283	G	A
NC_040266.1	68173112	G	C
NC_040266.1	68221030	T	G
NC_040266.1	68273109	A	G
NC_040266.1	68322410	C	T
NC_040266.1	68358885	T	C
NC_040266.1	68415339	A	G
NC_040266.1	68476940	G	A
NC_040266.1	68514440	T	C
NC_040266.1	68569879	G	A
NC_040266.1	68613352	C	T
NC_040266.1	68634865	C	A
NC_040266.1	68637122	T	C
NC_040266.1	68674764	G	T
NC_040266.1	68724771	G	A
NC_040266.1	68740563	G	C
NC_040266.1	68763568	A	G
NC_040266.1	68772858	C	A
NC_040266.1	68829232	T	C
NC_040266.1	68884891	A	G
NC_040266.1	68937048	A	G
NC_040266.1	68961913	A	G
NC_040266.1	69015037	G	A
NC_040266.1	69074832	T	C
NC_040266.1	69139421	T	C
NC_040266.1	69195281	G	A
NC_040266.1	69247787	A	G
NC_040266.1	69266600	C	T
NC_040266.1	69302248	A	G
NC_040266.1	69345692	C	T
NC_040266.1	69398420	A	G
NC_040266.1	69451270	T	C
NC_040266.1	69521892	T	G
NC_040266.1	69577542	G	C
NC_040266.1	69624337	T	A
NC_040266.1	69663273	G	C
NC_040266.1	69704501	T	C
NC_040266.1	69720547	T	C
NC_040266.1	69728527	C	T
NC_040266.1	69770309	A	G
NC_040266.1	69829974	T	C

NC_040266.1	69886101	G	A
NC_040266.1	69941829	T	C
NC_040266.1	69973715	T	C
NC_040266.1	70017627	T	C
NC_040266.1	70062692	T	G
NC_040266.1	70122821	C	T
NC_040266.1	70187849	T	C
NC_040266.1	70230122	A	G
NC_040266.1	70263445	T	C
NC_040266.1	70319959	C	T
NC_040266.1	70376202	T	C
NC_040266.1	70413176	A	G
NC_040266.1	70446142	T	A
NC_040266.1	70490220	A	G
NC_040266.1	70540205	A	G
NC_040266.1	70594406	T	C
NC_040266.1	70656957	A	C
NC_040266.1	70690083	T	G
NC_040266.1	70753315	T	C
NC_040266.1	70802794	T	C
NC_040266.1	70804820	G	A
NC_040266.1	70805941	T	C
NC_040266.1	70809806	C	T
NC_040266.1	70810658	A	C
NC_040266.1	70815994	T	C
NC_040266.1	70942815	T	C
NC_040266.1	70990799	C	T
NC_040266.1	71033891	T	A
NC_040266.1	71084440	A	G
NC_040266.1	71095882	T	C
NC_040266.1	71137493	C	T
NC_040266.1	71176231	C	A
NC_040266.1	71211449	A	G
NC_040266.1	71277635	A	G
NC_040266.1	71289078	T	C
NC_040266.1	71354823	G	C
NC_040266.1	71410303	A	C
NC_040266.1	71447018	A	G
NC_040266.1	71504286	G	C
NC_040266.1	71557328	T	C
NC_040266.1	71598327	A	G
NC_040266.1	71649372	C	G
NC_040266.1	71702732	C	T
NC_040266.1	71757256	C	T
NC_040266.1	71813276	T	G
NC_040266.1	71862262	T	C
NC_040266.1	71889554	G	A

NC_040266.1	71941801	A	G
NC_040266.1	72000700	A	G
NC_040266.1	72033665	C	A
NC_040266.1	72076796	T	C
NC_040266.1	72106012	T	C
NC_040266.1	72124007	A	G
NC_040266.1	72163427	A	C
NC_040266.1	72164933	A	G
NC_040266.1	72367483	T	C
NC_040266.1	72417071	G	A
NC_040266.1	72472660	T	C
NC_040266.1	72532204	A	T
NC_040266.1	72572366	C	T
NC_040266.1	72654619	A	G
NC_040266.1	72699080	T	C
NC_040266.1	72755625	C	T
NC_040266.1	72822208	T	C
NC_040266.1	72878869	C	T
NC_040266.1	72931583	A	G
NC_040266.1	72984365	T	C
NC_040266.1	73033974	G	A
NC_040266.1	73097035	A	G
NC_040266.1	73158612	G	A
NC_040266.1	73209371	T	C
NC_040266.1	73276309	T	A
NC_040266.1	73340125	C	T
NC_040266.1	73395182	G	T
NC_040266.1	73449017	C	A
NC_040266.1	73492386	C	T
NC_040266.1	73532408	G	A
NC_040266.1	73604547	T	C
NC_040266.1	73963356	T	C
NC_040266.1	74031752	T	C
NC_040266.1	74093321	G	A
NC_040266.1	74139007	A	G
NC_040266.1	74175957	A	G
NC_040266.1	74296556	T	C
NC_040266.1	74357537	A	G
NC_040266.1	74414033	A	G
NC_040266.1	74468014	C	T
NC_040266.1	74560827	C	T
NC_040266.1	74595414	A	G
NC_040266.1	74643404	G	A
NC_040266.1	74700728	T	C
NC_040266.1	74749566	A	G
NC_040266.1	74804551	C	A
NC_040266.1	74837365	A	G

NC_040266.1	74935428	A	G
NC_040266.1	74986531	G	A
NC_040266.1	75114966	A	G
NC_040266.1	75195210	T	C
NC_040266.1	75255822	T	C
NC_040266.1	75327488	G	T
NC_040266.1	75426339	C	T
NC_040266.1	75499277	G	A
NC_040266.1	75557912	G	T
NC_040266.1	75621011	A	G
NC_040266.1	75672741	C	T
NC_040266.1	75719437	G	C
NC_040266.1	75746787	A	G
NC_040266.1	75821934	A	G
NC_040266.1	75883637	T	C
NC_040266.1	75932159	G	A
NC_040266.1	76008054	C	A
NC_040266.1	76061196	A	G
NC_040266.1	76108064	T	C
NC_040266.1	76163711	A	C
NC_040266.1	76206372	A	C
NC_040266.1	76240122	G	T
NC_040266.1	76241556	G	A
NC_040266.1	76296665	T	G
NC_040266.1	76358854	G	A
NC_040266.1	76430442	G	T
NC_040266.1	76481765	A	G
NC_040266.1	76536801	T	C
NC_040266.1	76588492	G	T
NC_040266.1	76641982	G	T
NC_040266.1	76697817	A	G
NC_040266.1	76753625	A	G
NC_040266.1	76817790	A	G
NC_040266.1	76870643	A	C
NC_040266.1	76909329	G	A
NC_040266.1	76954389	G	C
NC_040266.1	76983153	C	A
NC_040266.1	77103159	G	A
NC_040266.1	77154359	C	T
NC_040266.1	77208840	C	A
NC_040266.1	77256056	G	C
NC_040266.1	77296031	C	T
NC_040266.1	77353278	C	G
NC_040266.1	77392642	A	G
NC_040266.1	77446192	G	T
NC_040266.1	77508361	C	T
NC_040266.1	77555986	T	C

NC_040266.1	77783597	A	G
NC_040266.1	77835973	G	A
NC_040266.1	77965804	C	A
NC_040266.1	78014548	A	G
NC_040266.1	78066610	T	C
NC_040266.1	78114210	T	C
NC_040266.1	78219621	G	A
NC_040266.1	78257368	C	T
NC_040266.1	78323351	G	A
NC_040266.1	78384504	T	C
NC_040266.1	78453378	C	T
NC_040266.1	78505447	G	A
NC_040266.1	78544438	C	T
NC_040266.1	78597427	T	C
NC_040266.1	78644528	A	G
NC_040266.1	78712660	C	T
NC_040266.1	78771478	T	C
NC_040266.1	78827328	A	G
NC_040266.1	78863221	C	T
NC_040266.1	78923802	C	G
NC_040266.1	78983677	G	A
NC_040266.1	79049875	A	C
NC_040266.1	79101390	T	C
NC_040266.1	79151970	T	C
NC_040266.1	79231335	T	C
NC_040266.1	79307164	C	A
NC_040266.1	79365510	T	A
NC_040266.1	79439655	A	G
NC_040266.1	79528464	G	A
NC_040266.1	79598288	A	G
NC_040266.1	79655213	T	C
NC_040266.1	79709268	A	G
NC_040266.1	79759245	A	G
NC_040266.1	79816501	T	C
NC_040266.1	79876556	T	C
NC_040266.1	79922307	A	C
NC_040266.1	79953212	C	T
NC_040266.1	80010924	T	C
NC_040266.1	80067516	A	C
NC_040266.1	80118557	T	C
NC_040266.1	80164678	G	A
NC_040266.1	80203382	C	G
NC_040266.1	80263335	A	T
NC_040266.1	80317720	A	G
NC_040266.1	80379539	T	C
NC_040266.1	80394215	T	C
NC_040266.1	80450572	A	G

NC_040266.1	80509780	A	C
NC_040266.1	80575367	T	C
NC_040266.1	80631455	C	T
NC_040266.1	80686584	A	G
NC_040266.1	80746469	T	G
NC_040266.1	80806975	G	C
NC_040266.1	80861985	T	C
NC_040266.1	80949520	T	C
NC_040266.1	80958258	A	G
NC_040266.1	81021778	A	G
NC_040266.1	81078063	G	C
NC_040266.1	81136027	G	A
NC_040266.1	81163923	T	C
NC_040266.1	81218973	A	G
NC_040266.1	81267101	C	T
NC_040266.1	81324088	C	G
NC_040266.1	81364708	A	G
NC_040266.1	81426763	T	C
NC_040266.1	81468337	A	G
NC_040266.1	81476497	T	C
NC_040266.1	81489863	T	C
NC_040266.1	81500205	G	A
NC_040266.1	81544797	C	A
NC_040266.1	81602059	G	A
NC_040266.1	81652064	A	G
NC_040266.1	81684894	T	G
NC_040266.1	81766867	T	C
NC_040266.1	81822897	A	G
NC_040266.1	81883461	T	C
NC_040266.1	81940164	A	G
NC_040266.1	81940626	A	G
NC_040266.1	81941375	A	G
NC_040266.1	81996288	T	C
NC_040266.1	82052554	G	A
NC_040266.1	82099213	C	G
NC_040266.1	82155238	A	C
NC_040266.1	82165820	G	A
NC_040266.1	82167061	T	C
NC_040266.1	82188736	T	C
NC_040266.1	82194798	T	C
NC_040266.1	82198024	A	G
NC_040266.1	82205754	T	C
NC_040266.1	82207786	T	G
NC_040266.1	82214210	C	T
NC_040266.1	82269500	T	C
NC_040266.1	82334775	A	G
NC_040266.1	82374875	G	T

NC_040266.1	82406766	T	C
NC_040266.1	82426434	G	C
NC_040266.1	82483391	G	A
NC_040266.1	82537026	C	G
NC_040266.1	82566737	T	C
NC_040266.1	82626688	0	G
NC_040266.1	82640368	A	G
NC_040266.1	82644199	C	T
NC_040266.1	82645219	A	G
NC_040266.1	82663565	A	G
NC_040266.1	82674111	C	T
NC_040266.1	82729913	G	C
NC_040266.1	82776559	T	G
NC_040266.1	82808077	A	G
NC_040266.1	82846928	C	T
NC_040266.1	82899798	T	C
NC_040266.1	82902522	A	C
NC_040266.1	82907609	T	C
NC_040266.1	82908101	T	G
NC_040266.1	82921006	T	C
NC_040266.1	82922145	A	C
NC_040266.1	82966461	C	T
NC_040266.1	83000973	G	T
NC_040266.1	83017910	C	T
NC_040266.1	83062159	A	G
NC_040266.1	83063577	A	G
NC_040266.1	83114398	T	A
NC_040266.1	83166460	A	T
NC_040266.1	83218793	T	C
NC_040266.1	83272926	T	G
NC_040266.1	83324817	A	C
NC_040266.1	83346108	A	G
NC_040266.1	83348141	A	G
NC_040266.1	83354075	A	G
NC_040266.1	83375962	C	G
NC_040266.1	83393509	G	A
NC_040266.1	83394154	A	C
NC_040266.1	83395211	A	G
NC_040266.1	83417029	T	C
NC_040266.1	83422536	C	G
NC_040266.1	83426683	T	C
NC_040266.1	83428437	T	C
NC_040266.1	83453602	T	C
NC_040266.1	83500761	T	C
NC_040266.1	83523889	T	C
NC_040266.1	83568695	T	C
NC_040266.1	83598674	A	G

NC_040266.1	83653206	T	C
NC_040266.1	83663574	A	G
NC_040266.1	83667219	T	G
NC_040266.1	83688850	C	T
NC_040266.1	83725306	T	C
NC_040266.1	83739092	T	G
NC_040266.1	83753511	C	T
NC_040266.1	83781327	A	G
NC_040266.1	83848350	A	G
NC_040266.1	83904315	A	T
NC_040266.1	83963162	A	G
NC_040266.1	84018502	A	G
NC_040266.1	84059081	C	T
NC_040266.1	84099496	G	A
NC_040266.1	84107202	T	C
NC_040266.1	84134554	T	C
NC_040266.1	84591992	A	G
NC_040266.1	84763633	G	A
NC_040266.1	84771920	C	T
NC_040266.1	84838898	A	T
NC_040266.1	84887707	A	T
NC_040266.1	85243337	A	G
NC_040266.1	85280723	A	G
NC_040266.1	85333138	C	G
NC_040266.1	85354196	C	T
NC_040266.1	85655195	A	G
NC_040266.1	85777956	C	G
NC_040266.1	85827248	A	C
NC_040266.1	85951490	A	G
NC_040266.1	85958377	A	T
NC_040266.1	85981013	G	A
NC_040266.1	86163509	A	G
NC_040266.1	86211685	C	T
NC_040266.1	86236663	G	A
NC_040266.1	86458163	A	G
NC_040266.1	86554405	T	C
NC_040266.1	86604901	C	T
NC_040266.1	86655347	C	T
NC_040266.1	86694183	T	G
NC_040266.1	86750504	C	G
NC_040266.1	86750714	A	G
NC_040266.1	86805708	A	G
NC_040266.1	86814779	T	C
NC_040266.1	86817881	T	C
NC_040266.1	86818043	T	C
NC_040266.1	86833702	A	G
NC_040266.1	86871729	A	G

NC_040266.1	86941282	T	C
NC_040266.1	86969487	T	C
NC_040266.1	86970447	C	A
NC_040266.1	87007120	0	G
NC_040266.1	87022200	T	G
NC_040266.1	87022771	C	T
NC_040266.1	87045826	G	C
NC_040266.1	87059431	A	G
NC_040266.1	87114841	T	C
NC_040266.1	87151503	T	C
NC_040266.1	87151665	A	G
NC_040266.1	87177936	A	G
NC_040266.1	87189002	C	T
NC_040266.1	87189775	T	C
NC_040266.1	87198352	A	G
NC_040266.1	87198556	C	T
NC_040266.1	87217039	C	T
NC_040266.1	87225920	C	T
NC_040266.1	87226504	C	T
NC_040266.1	87232061	T	C
NC_040266.1	87239326	G	A
NC_040266.1	87240049	A	G
NC_040266.1	87240649	A	G
NC_040266.1	87240949	C	T
NC_040266.1	87279022	A	G
NC_040266.1	87284467	A	G
NC_040266.1	87295360	A	G
NC_040266.1	87295779	A	G
NC_040266.1	87296033	T	C
NC_040266.1	87297464	G	C
NC_040266.1	87297653	A	G
NC_040266.1	87342632	C	T
NC_040266.1	87391274	A	T
NC_040266.1	87446781	G	A
NC_040266.1	87523901	G	A
NC_040266.1	87548784	T	C
NC_040266.1	87646628	A	G
NC_040266.1	87700378	G	A
NC_040266.1	87748960	C	T
NC_040266.1	87925841	C	T
NC_040266.1	88012869	T	C
NC_040266.1	88196057	A	G
NC_040266.1	88256481	C	T
NC_040266.1	88302142	T	C
NC_040266.1	88341181	A	G
NC_040266.1	88379698	T	C
NC_040266.1	88379947	T	C

NC_040266.1	88380572	G	C
NC_040266.1	88382392	A	G
NC_040266.1	88383977	A	C
NC_040266.1	88424525	G	C
NC_040266.1	88425058	A	G
NC_040266.1	88425319	G	A
NC_040266.1	88475844	G	C
NC_040266.1	88530742	A	G
NC_040266.1	88566685	G	A
NC_040266.1	88668048	G	A
NC_040266.1	88682852	G	A
NC_040266.1	88734825	T	G
NC_040266.1	88743385	G	A
NC_040266.1	88769329	C	G
NC_040266.1	88831087	T	C
NC_040266.1	88870556	T	A
NC_040266.1	88934197	A	G
NC_040266.1	89016439	A	C
NC_040266.1	89077057	G	A
NC_040266.1	89111771	T	C
NC_040266.1	89111946	G	T
NC_040266.1	89112291	T	C
NC_040266.1	89113206	G	T
NC_040266.1	89113848	C	T
NC_040266.1	89123132	T	C
NC_040266.1	89164702	T	C
NC_040266.1	89183081	G	A
NC_040266.1	89243225	C	T
NC_040266.1	89281300	C	G
NC_040266.1	89320915	T	C
NC_040266.1	89325493	C	G
NC_040266.1	89326618	A	C
NC_040266.1	89346026	T	C
NC_040266.1	89354408	T	G
NC_040266.1	89378278	A	C
NC_040266.1	89435932	C	G
NC_040266.1	89500213	C	A
NC_040266.1	89534762	T	C
NC_040266.1	89535278	G	A
NC_040266.1	89535432	T	C
NC_040266.1	89535635	A	G
NC_040266.1	89547210	C	T
NC_040266.1	89588735	G	C
NC_040266.1	89645721	C	G
NC_040266.1	89670122	C	T
NC_040266.1	89723923	T	G
NC_040266.1	89762348	C	T

NC_040266.1	89817837	A	G
NC_040266.1	89846234	T	C
NC_040266.1	89882564	G	A
NC_040266.1	89893200	C	T
NC_040266.1	89897006	G	C
NC_040266.1	89917568	C	G
NC_040266.1	89930011	C	G
NC_040266.1	89955769	T	C
NC_040266.1	89960253	T	C
NC_040266.1	90004747	T	C
NC_040266.1	90010085	T	C
NC_040266.1	90010311	T	C
NC_040266.1	90010866	T	C
NC_040266.1	90015396	T	C
NC_040266.1	90031612	G	C
NC_040266.1	90087489	A	G
NC_040266.1	90141553	A	G
NC_040266.1	90196490	T	C
NC_040266.1	90242439	T	C
NC_040267.1	322109	C	T
NC_040267.1	367908	A	G
NC_040267.1	407629	T	C
NC_040267.1	472701	A	G
NC_040267.1	515778	T	C
NC_040267.1	623586	C	T
NC_040267.1	657151	A	G
NC_040267.1	711402	T	C
NC_040267.1	770673	G	T
NC_040267.1	828066	A	C
NC_040267.1	887373	C	A
NC_040267.1	928441	G	A
NC_040267.1	961073	A	G
NC_040267.1	985437	A	G
NC_040267.1	1032154	G	T
NC_040267.1	1032615	C	T
NC_040267.1	1075006	C	G
NC_040267.1	1088647	A	G
NC_040267.1	1240575	A	G
NC_040267.1	1289998	G	C
NC_040267.1	1294854	T	C
NC_040267.1	1349481	A	G
NC_040267.1	1350606	T	G
NC_040267.1	1409603	G	A
NC_040267.1	1462423	G	A
NC_040267.1	1500724	T	C
NC_040267.1	1524036	T	A
NC_040267.1	1578704	T	C

NC_040267.1	1633489	G	T
NC_040267.1	1688499	C	A
NC_040267.1	1737526	A	G
NC_040267.1	1781718	T	C
NC_040267.1	1810221	A	G
NC_040267.1	1859379	G	A
NC_040267.1	1882825	T	C
NC_040267.1	1939439	A	T
NC_040267.1	1975234	A	G
NC_040267.1	2008894	G	C
NC_040267.1	2096566	G	A
NC_040267.1	2150463	G	A
NC_040267.1	2196644	T	C
NC_040267.1	2221774	A	T
NC_040267.1	2303004	A	G
NC_040267.1	2304249	T	C
NC_040267.1	2333801	T	C
NC_040267.1	2358577	A	G
NC_040267.1	2390035	T	C
NC_040267.1	2400058	A	G
NC_040267.1	2445932	A	G
NC_040267.1	2467360	T	C
NC_040267.1	2519861	T	C
NC_040267.1	2551901	T	G
NC_040267.1	2591910	C	T
NC_040267.1	2654034	C	G
NC_040267.1	2699312	0	C
NC_040267.1	2752101	A	G
NC_040267.1	2799166	T	C
NC_040267.1	2825611	A	G
NC_040267.1	2850572	A	G
NC_040267.1	2904594	A	T
NC_040267.1	2963375	A	G
NC_040267.1	3022880	T	G
NC_040267.1	3063242	T	A
NC_040267.1	3098399	G	A
NC_040267.1	3163414	T	C
NC_040267.1	3212100	C	T
NC_040267.1	3260577	C	T
NC_040267.1	3265027	C	T
NC_040267.1	3312113	G	A
NC_040267.1	3317466	G	A
NC_040267.1	3374733	G	A
NC_040267.1	3425158	G	A
NC_040267.1	3445193	A	C
NC_040267.1	3498229	G	A
NC_040267.1	3551700	C	T

NC_040267.1	3608549	A	G
NC_040267.1	3664526	T	C
NC_040267.1	3717481	A	G
NC_040267.1	3771118	G	T
NC_040267.1	3828021	A	C
NC_040267.1	3869687	T	G
NC_040267.1	3884906	T	C
NC_040267.1	3928149	C	T
NC_040267.1	3962710	G	A
NC_040267.1	3965633	A	G
NC_040267.1	4012054	C	T
NC_040267.1	4079108	A	G
NC_040267.1	4131175	A	G
NC_040267.1	4174122	A	G
NC_040267.1	4207996	G	A
NC_040267.1	4241314	A	G
NC_040267.1	4274667	A	T
NC_040267.1	4320448	T	C
NC_040267.1	4373825	T	C
NC_040267.1	4378007	T	C
NC_040267.1	4421689	G	C
NC_040267.1	4460134	G	A
NC_040267.1	4513011	C	T
NC_040267.1	4570453	A	G
NC_040267.1	4623492	C	T
NC_040267.1	4651990	A	G
NC_040267.1	4679791	A	G
NC_040267.1	4710903	A	G
NC_040267.1	4725649	G	T
NC_040267.1	4730513	A	C
NC_040267.1	4791283	A	G
NC_040267.1	4845860	G	T
NC_040267.1	4900407	A	C
NC_040267.1	4948079	T	C
NC_040267.1	4991331	T	C
NC_040267.1	5001457	T	C
NC_040267.1	5057201	T	C
NC_040267.1	5113731	A	G
NC_040267.1	5128850	A	G
NC_040267.1	5182883	G	C
NC_040267.1	5183306	T	C
NC_040267.1	5227345	A	G
NC_040267.1	5272215	C	T
NC_040267.1	5283125	T	C
NC_040267.1	5316708	T	C
NC_040267.1	5337002	C	T
NC_040267.1	5387635	C	T

NC_040267.1	5396880	A	G
NC_040267.1	5439994	A	G
NC_040267.1	5475995	T	C
NC_040267.1	5500930	A	G
NC_040267.1	5551362	G	A
NC_040267.1	5593007	A	G
NC_040267.1	5615774	T	G
NC_040267.1	5681583	C	T
NC_040267.1	5858347	T	C
NC_040267.1	5913122	C	A
NC_040267.1	5967592	A	G
NC_040267.1	6022310	G	T
NC_040267.1	6059788	C	T
NC_040267.1	6061163	C	A
NC_040267.1	6109672	G	A
NC_040267.1	6145623	C	T
NC_040267.1	6222174	G	T
NC_040267.1	6278453	C	T
NC_040267.1	6355917	A	C
NC_040267.1	6423403	A	G
NC_040267.1	6451944	A	G
NC_040267.1	6475752	T	C
NC_040267.1	6520160	T	C
NC_040267.1	6531877	C	T
NC_040267.1	6557899	T	C
NC_040267.1	6589829	G	C
NC_040267.1	6639355	C	T
NC_040267.1	6705014	A	T
NC_040267.1	6775148	T	C
NC_040267.1	6807677	G	A
NC_040267.1	6830072	T	A
NC_040267.1	6888900	A	G
NC_040267.1	6945482	0	C
NC_040267.1	6995005	C	T
NC_040267.1	7024031	G	A
NC_040267.1	7061900	G	A
NC_040267.1	7103751	C	T
NC_040267.1	7166688	T	C
NC_040267.1	7216507	G	A
NC_040267.1	7241134	T	C
NC_040267.1	7243080	G	A
NC_040267.1	7243633	G	A
NC_040267.1	7305306	G	C
NC_040267.1	7405923	T	C
NC_040267.1	7410033	A	G
NC_040267.1	7422059	G	A
NC_040267.1	7478705	A	G

NC_040267.1	7528216	C	G
NC_040267.1	7540329	A	G
NC_040267.1	7595890	G	T
NC_040267.1	7645401	A	G
NC_040267.1	7649363	T	C
NC_040267.1	7704417	C	T
NC_040267.1	7770449	A	G
NC_040267.1	7825538	T	C
NC_040267.1	7879398	C	A
NC_040267.1	7940096	A	G
NC_040267.1	7994880	A	G
NC_040267.1	8052847	C	T
NC_040267.1	8080159	C	T
NC_040267.1	8108416	A	G
NC_040267.1	8171374	G	A
NC_040267.1	8200861	A	G
NC_040267.1	8265311	C	A
NC_040267.1	8320603	A	G
NC_040267.1	8375222	G	T
NC_040267.1	8418367	C	T
NC_040267.1	8471908	C	T
NC_040267.1	8536705	C	A
NC_040267.1	8544549	G	C
NC_040267.1	8596197	A	T
NC_040267.1	8651409	C	A
NC_040267.1	8707136	A	G
NC_040267.1	8763676	A	G
NC_040267.1	8818277	G	A
NC_040267.1	8872092	T	C
NC_040267.1	8919705	C	T
NC_040267.1	8965154	A	G
NC_040267.1	8965715	A	G
NC_040267.1	9014438	C	A
NC_040267.1	9094610	T	C
NC_040267.1	9131276	0	G
NC_040267.1	9178377	T	C
NC_040267.1	9183032	G	A
NC_040267.1	9194953	A	G
NC_040267.1	9250569	T	C
NC_040267.1	9291291	A	G
NC_040267.1	9319499	C	A
NC_040267.1	9348269	G	A
NC_040267.1	9404722	G	A
NC_040267.1	9447104	A	G
NC_040267.1	9482178	T	G
NC_040267.1	9497381	C	T
NC_040267.1	9553789	G	A

NC_040267.1	9592407	G	A
NC_040267.1	9592895	G	C
NC_040267.1	9611101	A	G
NC_040267.1	9665346	G	A
NC_040267.1	9697783	A	G
NC_040267.1	9759400	G	A
NC_040267.1	9818458	T	C
NC_040267.1	9845065	G	T
NC_040267.1	9846334	G	C
NC_040267.1	9847151	C	T
NC_040267.1	9848807	A	G
NC_040267.1	9854437	C	G
NC_040267.1	9895876	G	C
NC_040267.1	9907074	C	T
NC_040267.1	9960547	A	C
NC_040267.1	10018218	A	G
NC_040267.1	10070210	T	A
NC_040267.1	10128061	T	G
NC_040267.1	10193243	T	C
NC_040267.1	10251597	A	T
NC_040267.1	10293695	C	T
NC_040267.1	10320006	T	C
NC_040267.1	10339689	C	T
NC_040267.1	10372269	T	C
NC_040267.1	10394570	G	A
NC_040267.1	10421760	G	A
NC_040267.1	10456247	T	A
NC_040267.1	10489361	C	T
NC_040267.1	10490474	C	T
NC_040267.1	10539656	C	T
NC_040267.1	10582461	T	C
NC_040267.1	10596972	T	C
NC_040267.1	10655168	T	C
NC_040267.1	10711320	A	C
NC_040267.1	10757485	T	G
NC_040267.1	10780946	T	C
NC_040267.1	10801804	G	A
NC_040267.1	10845611	A	G
NC_040267.1	10901173	G	T
NC_040267.1	10957387	A	G
NC_040267.1	10992109	T	C
NC_040267.1	11013586	T	C
NC_040267.1	11071920	A	G
NC_040267.1	11129450	T	A
NC_040267.1	11185593	G	A
NC_040267.1	11227853	T	C
NC_040267.1	11291790	A	C

NC_040267.1	11345842	G	C
NC_040267.1	11395069	A	G
NC_040267.1	11423185	G	T
NC_040267.1	11449331	C	T
NC_040267.1	11450787	T	C
NC_040267.1	11492446	T	G
NC_040267.1	11524671	T	C
NC_040267.1	11547657	A	G
NC_040267.1	11599184	G	A
NC_040267.1	11654786	T	G
NC_040267.1	11706096	G	A
NC_040267.1	11759988	G	A
NC_040267.1	11800943	C	T
NC_040267.1	11837989	C	T
NC_040267.1	11839851	T	C
NC_040267.1	11847020	G	A
NC_040267.1	11889609	T	C
NC_040267.1	11923609	A	G
NC_040267.1	11933483	G	C
NC_040267.1	11989631	G	A
NC_040267.1	12047335	A	G
NC_040267.1	12098117	T	C
NC_040267.1	12151056	C	G
NC_040267.1	12206271	A	T
NC_040267.1	12257305	C	T
NC_040267.1	12283616	A	T
NC_040267.1	12316574	A	G
NC_040267.1	12350887	T	C
NC_040267.1	12368994	A	G
NC_040267.1	12426716	G	C
NC_040267.1	12480051	G	C
NC_040267.1	12532133	T	C
NC_040267.1	12588048	C	T
NC_040267.1	12647721	G	A
NC_040267.1	12704504	G	C
NC_040267.1	12754231	A	C
NC_040267.1	12808084	A	G
NC_040267.1	12850713	A	G
NC_040267.1	12870739	T	C
NC_040267.1	12873712	T	C
NC_040267.1	12919175	T	C
NC_040267.1	12968873	C	T
NC_040267.1	12978352	T	C
NC_040267.1	13039087	C	G
NC_040267.1	13090379	G	A
NC_040267.1	13151036	G	C
NC_040267.1	13208419	G	A

NC_040267.1	13267891	0	T
NC_040267.1	13325212	C	T
NC_040267.1	13382747	C	T
NC_040267.1	13428699	T	C
NC_040267.1	13436242	C	T
NC_040267.1	13492411	A	G
NC_040267.1	13548977	A	G
NC_040267.1	13596153	G	T
NC_040267.1	13626045	C	A
NC_040267.1	13647364	T	C
NC_040267.1	13699534	T	C
NC_040267.1	13728875	A	G
NC_040267.1	13749560	A	G
NC_040267.1	13785028	C	T
NC_040267.1	13821888	A	G
NC_040267.1	13841040	G	C
NC_040267.1	13895056	C	T
NC_040267.1	13910849	C	T
NC_040267.1	13963601	C	T
NC_040267.1	14017261	A	G
NC_040267.1	14060924	T	C
NC_040267.1	14096608	C	G
NC_040267.1	14149351	T	C
NC_040267.1	14152434	T	G
NC_040267.1	14205052	A	G
NC_040267.1	14251112	A	G
NC_040267.1	14287270	T	C
NC_040267.1	14298431	C	T
NC_040267.1	14334993	G	A
NC_040267.1	14336986	G	T
NC_040267.1	14386983	T	C
NC_040267.1	14404873	C	A
NC_040267.1	14409008	T	C
NC_040267.1	14439999	T	C
NC_040267.1	14487337	A	G
NC_040267.1	14545087	A	G
NC_040267.1	14600140	G	A
NC_040267.1	14658010	T	C
NC_040267.1	14713537	C	G
NC_040267.1	14767739	T	C
NC_040267.1	14769130	A	G
NC_040267.1	14824292	G	A
NC_040267.1	14881002	G	A
NC_040267.1	14903011	T	C
NC_040267.1	14956946	T	C
NC_040267.1	14986032	T	G
NC_040267.1	15013739	C	G

NC_040267.1	15068798	C	A
NC_040267.1	15131737	A	G
NC_040267.1	15167063	G	A
NC_040267.1	15187610	A	G
NC_040267.1	15189292	G	A
NC_040267.1	15191173	G	A
NC_040267.1	15230073	G	A
NC_040267.1	15272041	A	G
NC_040267.1	15293709	G	T
NC_040267.1	15295026	G	T
NC_040267.1	15298905	A	G
NC_040267.1	15350271	C	T
NC_040267.1	15402060	T	C
NC_040267.1	15403744	G	C
NC_040267.1	15450146	T	G
NC_040267.1	16137681	A	C
NC_040267.1	16171452	C	G
NC_040267.1	16218469	T	C
NC_040267.1	16220630	C	T
NC_040267.1	16252128	G	A
NC_040267.1	16270288	A	G
NC_040267.1	16332022	C	G
NC_040267.1	16365359	C	T
NC_040267.1	16382436	G	C
NC_040267.1	16411177	T	C
NC_040267.1	16437996	T	G
NC_040267.1	16493566	G	T
NC_040267.1	16525133	T	C
NC_040267.1	16539144	C	T
NC_040267.1	16588875	G	A
NC_040267.1	16653730	T	G
NC_040267.1	16680082	T	C
NC_040267.1	16716465	C	T
NC_040267.1	16768654	T	C
NC_040267.1	16815240	C	T
NC_040267.1	16865947	G	A
NC_040267.1	16965671	A	G
NC_040267.1	17017562	C	T
NC_040267.1	17102529	G	A
NC_040267.1	17127172	G	A
NC_040267.1	17167657	T	C
NC_040267.1	17176079	0	C
NC_040267.1	17239696	G	A
NC_040267.1	17396807	T	C
NC_040267.1	17448324	G	T
NC_040267.1	17487046	G	A
NC_040267.1	17503989	T	C

NC_040267.1	17532555	T	C
NC_040267.1	17547000	G	A
NC_040267.1	17567562	T	C
NC_040267.1	17647676	T	C
NC_040267.1	17700024	C	T
NC_040267.1	17739850	A	C
NC_040267.1	17770333	A	G
NC_040267.1	17786529	T	C
NC_040267.1	17840989	C	T
NC_040267.1	17852400	A	C
NC_040267.1	17916825	A	G
NC_040267.1	17974341	T	G
NC_040267.1	18001835	T	A
NC_040267.1	18020095	C	A
NC_040267.1	18021890	T	C
NC_040267.1	18022751	T	C
NC_040267.1	18023684	C	A
NC_040267.1	18024657	A	G
NC_040267.1	18078698	G	A
NC_040267.1	18137671	A	G
NC_040267.1	18189654	A	G
NC_040267.1	18243079	A	G
NC_040267.1	18295252	A	G
NC_040267.1	18357275	T	C
NC_040267.1	18417389	T	C
NC_040267.1	18470865	T	G
NC_040267.1	18504031	A	G
NC_040267.1	18537062	A	T
NC_040267.1	18554035	A	G
NC_040267.1	18611120	A	G
NC_040267.1	18664229	C	T
NC_040267.1	18710777	G	A
NC_040267.1	18714846	A	G
NC_040267.1	18772102	G	A
NC_040267.1	18811441	T	C
NC_040267.1	18861974	G	A
NC_040267.1	18916565	C	T
NC_040267.1	18971388	C	T
NC_040267.1	19030690	A	G
NC_040267.1	19084276	A	C
NC_040267.1	19139855	G	C
NC_040267.1	19181879	A	G
NC_040267.1	19205728	C	T
NC_040267.1	19247330	A	G
NC_040267.1	19285857	C	T
NC_040267.1	19331630	T	G
NC_040267.1	19366442	C	T

NC_040267.1	19376956	A	G
NC_040267.1	19400372	G	C
NC_040267.1	19431774	C	T
NC_040267.1	19487086	G	A
NC_040267.1	19551488	T	G
NC_040267.1	19588615	G	T
NC_040267.1	19721681	G	A
NC_040267.1	19794535	C	T
NC_040267.1	19835802	G	A
NC_040267.1	19866981	A	G
NC_040267.1	19894870	C	T
NC_040267.1	19959159	G	A
NC_040267.1	20002504	T	C
NC_040267.1	20029408	G	A
NC_040267.1	20059904	A	G
NC_040267.1	20109135	T	C
NC_040267.1	20146312	G	C
NC_040267.1	20315377	A	G
NC_040267.1	20374635	C	T
NC_040267.1	20405614	A	G
NC_040267.1	20511472	G	T
NC_040267.1	20570691	A	G
NC_040267.1	20624398	A	G
NC_040267.1	20667241	C	T
NC_040267.1	20730337	A	G
NC_040267.1	20781888	T	C
NC_040267.1	20834290	T	C
NC_040267.1	20891268	A	G
NC_040267.1	20949111	C	A
NC_040267.1	21014014	C	T
NC_040267.1	21072595	C	G
NC_040267.1	21118352	A	G
NC_040267.1	21174079	A	G
NC_040267.1	21207227	A	G
NC_040267.1	21253183	T	G
NC_040267.1	21281219	A	G
NC_040267.1	21300460	G	A
NC_040267.1	21335151	C	T
NC_040267.1	21358187	C	A
NC_040267.1	21418478	T	C
NC_040267.1	21443808	C	G
NC_040267.1	21482817	T	C
NC_040267.1	21499200	A	G
NC_040267.1	21538706	C	G
NC_040267.1	21569151	T	C
NC_040267.1	21580563	C	T
NC_040267.1	21609331	A	G

NC_040267.1	21666869	A	G
NC_040267.1	21697482	C	T
NC_040267.1	21699723	A	G
NC_040267.1	21773185	C	T
NC_040267.1	21822726	G	A
NC_040267.1	21865119	T	G
NC_040267.1	21886510	A	G
NC_040267.1	21933443	T	C
NC_040267.1	21960286	T	C
NC_040267.1	21985600	C	A
NC_040267.1	22000231	G	A
NC_040267.1	22046467	T	C
NC_040267.1	22083463	T	C
NC_040267.1	22101011	G	A
NC_040267.1	22102349	T	C
NC_040267.1	22160261	T	C
NC_040267.1	22195020	T	A
NC_040267.1	22226335	G	A
NC_040267.1	22265791	G	T
NC_040267.1	22272274	C	T
NC_040267.1	22324453	C	G
NC_040267.1	22361842	G	A
NC_040267.1	22380043	C	T
NC_040267.1	22426308	A	G
NC_040267.1	22469389	C	A
NC_040267.1	22581487	A	T
NC_040267.1	22616082	C	T
NC_040267.1	22640036	T	C
NC_040267.1	22675193	G	C
NC_040267.1	22694711	T	A
NC_040267.1	22743785	A	G
NC_040267.1	22774847	A	T
NC_040267.1	22817762	A	G
NC_040267.1	22842971	C	A
NC_040267.1	22934432	C	T
NC_040267.1	22980298	C	T
NC_040267.1	23141832	A	G
NC_040267.1	23195342	T	C
NC_040267.1	23227904	G	A
NC_040267.1	23295638	T	C
NC_040267.1	23339291	A	G
NC_040267.1	23367647	T	A
NC_040267.1	23396807	A	G
NC_040267.1	23463163	A	G
NC_040267.1	23552550	C	T
NC_040267.1	23605429	A	G
NC_040267.1	23634874	G	T

NC_040267.1	23661694	T	C
NC_040267.1	23700177	C	T
NC_040267.1	23750331	C	T
NC_040267.1	23808510	A	C
NC_040267.1	23854307	G	A
NC_040267.1	23861272	T	G
NC_040267.1	23915669	C	T
NC_040267.1	23970846	C	G
NC_040267.1	24014629	A	G
NC_040267.1	24036000	G	A
NC_040267.1	24091112	C	T
NC_040267.1	24142226	C	A
NC_040267.1	24187599	G	A
NC_040267.1	24240680	T	C
NC_040267.1	24305082	C	T
NC_040267.1	24365400	A	T
NC_040267.1	24420241	T	C
NC_040267.1	24477993	T	C
NC_040267.1	24527305	T	G
NC_040267.1	24569556	T	C
NC_040267.1	24612048	G	A
NC_040267.1	24637885	C	T
NC_040267.1	24643199	A	G
NC_040267.1	24693647	T	C
NC_040267.1	24725102	T	C
NC_040267.1	24737228	C	T
NC_040267.1	24792556	T	C
NC_040267.1	24842002	A	G
NC_040267.1	24873831	G	A
NC_040267.1	24921040	A	T
NC_040267.1	24946097	A	G
NC_040267.1	24968761	T	G
NC_040267.1	25025657	T	C
NC_040267.1	25078885	T	C
NC_040267.1	25131420	C	T
NC_040267.1	25179316	C	T
NC_040267.1	25229515	T	C
NC_040267.1	25273809	C	A
NC_040267.1	25330204	T	G
NC_040267.1	25384646	T	C
NC_040267.1	25439965	C	T
NC_040267.1	25489897	C	T
NC_040267.1	25534178	C	T
NC_040267.1	25544464	T	C
NC_040267.1	25581270	T	G
NC_040267.1	25590020	A	G
NC_040267.1	25641768	A	G

NC_040267.1	25708002	A	C
NC_040267.1	25755774	T	C
NC_040267.1	25789256	C	T
NC_040267.1	25831377	A	G
NC_040267.1	25848172	T	C
NC_040267.1	25889054	C	T
NC_040267.1	25924854	A	C
NC_040267.1	25990778	T	C
NC_040267.1	26051368	G	A
NC_040267.1	26107159	T	C
NC_040267.1	26160333	G	A
NC_040267.1	26225967	C	T
NC_040267.1	26294818	T	G
NC_040267.1	26383422	G	A
NC_040267.1	26421484	T	C
NC_040267.1	26480105	T	C
NC_040267.1	26525417	A	C
NC_040267.1	26550456	G	A
NC_040267.1	26568680	A	C
NC_040267.1	26595870	G	A
NC_040267.1	26647783	T	C
NC_040267.1	26689245	C	T
NC_040267.1	26782241	G	A
NC_040267.1	26811494	A	G
NC_040267.1	26838073	T	C
NC_040267.1	26875709	G	A
NC_040267.1	26913560	G	T
NC_040267.1	26932628	C	T
NC_040267.1	26986747	C	T
NC_040267.1	27017073	A	T
NC_040267.1	27066364	G	C
NC_040267.1	27119294	T	G
NC_040267.1	27180130	T	C
NC_040267.1	27225680	T	A
NC_040267.1	27252199	A	G
NC_040267.1	27304231	T	G
NC_040267.1	27324220	G	C
NC_040267.1	27336561	A	G
NC_040267.1	27369111	A	G
NC_040267.1	27412484	C	T
NC_040267.1	27462866	T	C
NC_040267.1	27538304	C	A
NC_040267.1	27633730	G	A
NC_040267.1	27683633	T	C
NC_040267.1	27693758	A	G
NC_040267.1	27731380	T	C
NC_040267.1	27780759	G	A

NC_040267.1	27787130	G	A
NC_040267.1	27818229	A	G
NC_040267.1	27852563	A	G
NC_040267.1	27872113	C	G
NC_040267.1	28175436	C	T
NC_040267.1	28229363	C	T
NC_040267.1	28579706	C	T
NC_040267.1	28684540	A	G
NC_040267.1	28741008	A	G
NC_040267.1	28809877	A	G
NC_040267.1	28867021	C	T
NC_040267.1	28923028	A	G
NC_040267.1	28980791	C	T
NC_040267.1	29025044	A	G
NC_040267.1	29081720	A	C
NC_040267.1	29152952	A	G
NC_040267.1	29201164	A	C
NC_040267.1	29255832	G	A
NC_040267.1	29436481	C	T
NC_040267.1	29482882	T	A
NC_040267.1	29540549	T	G
NC_040267.1	29607767	C	T
NC_040267.1	29645145	C	T
NC_040267.1	29657893	C	T
NC_040267.1	29691345	G	T
NC_040267.1	29710880	G	C
NC_040267.1	29757312	G	A
NC_040267.1	29797388	A	G
NC_040267.1	29863000	T	C
NC_040267.1	29906221	A	G
NC_040267.1	29955879	C	T
NC_040267.1	29964316	C	T
NC_040267.1	30038863	G	A
NC_040267.1	30098726	T	C
NC_040267.1	30135648	C	T
NC_040267.1	30220979	A	G
NC_040267.1	30269567	A	G
NC_040267.1	30369793	C	G
NC_040267.1	30440103	G	A
NC_040267.1	30466436	C	A
NC_040267.1	30501999	C	A
NC_040267.1	30574816	T	C
NC_040267.1	30605005	C	T
NC_040267.1	30608315	C	G
NC_040267.1	30652418	G	T
NC_040267.1	30691779	T	C
NC_040267.1	30730912	C	T

NC_040267.1	30755253	A	C
NC_040267.1	30799329	G	A
NC_040267.1	30828630	A	G
NC_040267.1	31152228	C	A
NC_040267.1	31210252	G	T
NC_040267.1	31232227	A	G
NC_040267.1	31316429	A	G
NC_040267.1	31459124	C	T
NC_040267.1	31571523	T	A
NC_040267.1	31699819	A	C
NC_040267.1	31724260	C	T
NC_040267.1	31799553	G	A
NC_040267.1	31824763	T	A
NC_040267.1	31848510	C	T
NC_040267.1	31922831	C	T
NC_040267.1	31979828	T	C
NC_040267.1	32020949	G	C
NC_040267.1	32042035	G	C
NC_040267.1	32087751	A	G
NC_040267.1	32151227	T	C
NC_040267.1	32183192	A	C
NC_040267.1	32209169	G	A
NC_040267.1	32262478	C	A
NC_040267.1	32308065	A	G
NC_040267.1	32333638	A	G
NC_040267.1	32372096	C	T
NC_040267.1	32420568	A	G
NC_040267.1	32426173	C	T
NC_040267.1	32539568	C	T
NC_040267.1	32594497	C	G
NC_040267.1	32661672	T	C
NC_040267.1	32708938	C	A
NC_040267.1	32738962	T	C
NC_040267.1	32784012	C	T
NC_040267.1	32793875	T	A
NC_040267.1	32831483	T	C
NC_040267.1	32855840	C	T
NC_040267.1	32884188	A	G
NC_040267.1	32910480	A	G
NC_040267.1	32920571	T	C
NC_040267.1	32976764	T	G
NC_040267.1	33025965	G	A
NC_040267.1	33078298	A	G
NC_040267.1	33143936	C	A
NC_040267.1	33206550	G	C
NC_040267.1	33242658	T	C
NC_040267.1	33303104	T	C

NC_040267.1	33461652	T	A
NC_040267.1	33548962	C	T
NC_040267.1	33583510	C	T
NC_040267.1	33609245	G	T
NC_040267.1	33636930	C	T
NC_040267.1	33664462	C	T
NC_040267.1	33704383	A	G
NC_040267.1	33737366	T	C
NC_040267.1	33749743	G	T
NC_040267.1	33806098	C	T
NC_040267.1	33831141	A	G
NC_040267.1	33882320	T	G
NC_040267.1	33936548	C	A
NC_040267.1	33992879	G	A
NC_040267.1	34035247	G	T
NC_040267.1	34072272	T	C
NC_040267.1	34111649	C	T
NC_040267.1	34137072	G	A
NC_040267.1	34233955	A	G
NC_040267.1	34297480	A	T
NC_040267.1	34344665	A	G
NC_040267.1	34385331	G	T
NC_040267.1	34491099	C	T
NC_040267.1	34524684	T	C
NC_040267.1	34570625	C	T
NC_040267.1	34596677	C	G
NC_040267.1	34686044	A	G
NC_040267.1	34796765	A	G
NC_040267.1	34841960	T	C
NC_040267.1	34883400	C	T
NC_040267.1	35050474	A	T
NC_040267.1	35137650	T	G
NC_040267.1	35188010	C	T
NC_040267.1	35203660	A	G
NC_040267.1	35290821	C	T
NC_040267.1	35382625	T	A
NC_040267.1	35412708	A	G
NC_040267.1	35433419	A	T
NC_040267.1	35461286	G	A
NC_040267.1	35531029	G	A
NC_040267.1	35712973	A	G
NC_040267.1	35776539	G	A
NC_040267.1	35853008	G	A
NC_040267.1	35906488	T	C
NC_040267.1	35934427	C	T
NC_040267.1	35969558	T	C
NC_040267.1	35993983	C	T

NC_040267.1	36054424	C	T
NC_040267.1	36088080	A	G
NC_040267.1	36199096	T	C
NC_040267.1	36266093	A	G
NC_040267.1	36290543	G	A
NC_040267.1	36338188	G	T
NC_040267.1	36399071	T	C
NC_040267.1	36637127	G	A
NC_040267.1	36703382	A	C
NC_040267.1	36745899	C	G
NC_040267.1	36804155	G	A
NC_040267.1	36872956	T	C
NC_040267.1	36929278	G	A
NC_040267.1	36983653	T	C
NC_040267.1	37131355	T	A
NC_040267.1	37173569	G	A
NC_040267.1	37279316	C	A
NC_040267.1	37306069	T	C
NC_040267.1	37332842	C	T
NC_040267.1	37361965	G	A
NC_040267.1	37386129	A	T
NC_040267.1	37422081	T	C
NC_040267.1	37761414	T	C
NC_040267.1	37802448	T	C
NC_040267.1	37907194	A	G
NC_040267.1	37932710	C	T
NC_040267.1	37990320	T	C
NC_040267.1	38184939	C	G
NC_040267.1	38474159	C	T
NC_040267.1	38575700	A	G
NC_040267.1	38643491	T	C
NC_040267.1	38737731	T	C
NC_040267.1	38768088	T	C
NC_040267.1	38801251	A	G
NC_040267.1	38859612	C	T
NC_040267.1	38938478	T	C
NC_040267.1	39025308	C	A
NC_040267.1	39158893	T	C
NC_040267.1	39219707	T	C
NC_040267.1	39278407	C	T
NC_040267.1	39306839	A	G
NC_040267.1	39358112	C	T
NC_040267.1	39552217	T	G
NC_040267.1	39599094	A	G
NC_040267.1	39628105	T	C
NC_040267.1	39907585	C	T
NC_040267.1	40135042	C	T

NC_040267.1	40252943	A	G
NC_040267.1	40360055	C	T
NC_040267.1	40603572	C	T
NC_040267.1	40636751	G	A
NC_040267.1	40866284	C	G
NC_040267.1	41025617	T	C
NC_040267.1	41225830	G	A
NC_040267.1	41495777	G	A
NC_040267.1	41573508	G	T
NC_040267.1	41737055	T	C
NC_040267.1	41917592	G	T
NC_040267.1	42014893	A	G
NC_040267.1	42609837	A	G
NC_040267.1	42860488	T	C
NC_040267.1	43104512	G	A
NC_040267.1	43325069	G	A
NC_040267.1	43425381	C	T
NC_040267.1	43493945	G	C
NC_040267.1	43540124	C	G
NC_040267.1	43648083	C	T
NC_040267.1	43725485	G	C
NC_040267.1	43766950	G	A
NC_040267.1	43901856	T	G
NC_040267.1	43942654	A	G
NC_040267.1	44013847	A	C
NC_040267.1	44128844	A	G
NC_040267.1	44228575	A	G
NC_040267.1	44275194	T	C
NC_040267.1	44394816	T	G
NC_040267.1	44460460	C	A
NC_040267.1	44499402	T	A
NC_040267.1	44550030	T	C
NC_040267.1	44645022	G	A
NC_040267.1	44714990	C	T
NC_040267.1	44743274	T	C
NC_040267.1	44833853	A	G
NC_040267.1	45243072	G	C
NC_040267.1	45462759	A	C
NC_040267.1	45602314	G	A
NC_040267.1	46428262	C	A
NC_040267.1	47133909	G	T
NC_040267.1	47253112	C	T
NC_040267.1	47624662	A	T
NC_040267.1	47698485	A	C
NC_040267.1	47750645	T	C
NC_040267.1	48693080	T	C
NC_040267.1	48763808	T	C

NC_040267.1	48816556	G	A
NC_040267.1	48883397	C	A
NC_040267.1	48953461	T	A
NC_040267.1	49013925	G	A
NC_040267.1	49085273	T	C
NC_040267.1	49173474	T	C
NC_040267.1	49233863	T	C
NC_040267.1	49347907	T	C
NC_040267.1	49478339	A	C
NC_040267.1	49532738	G	A
NC_040267.1	49602765	A	G
NC_040267.1	49920887	T	C
NC_040267.1	49950591	A	G
NC_040267.1	50229273	A	T
NC_040267.1	50316777	A	T
NC_040267.1	50523015	G	A
NC_040267.1	51378197	A	T
NC_040267.1	51786455	G	A
NC_040267.1	52185176	T	C
NC_040267.1	52484262	C	T
NC_040267.1	52563488	T	C
NC_040267.1	52628969	C	G
NC_040267.1	52676357	A	G
NC_040267.1	53524025	A	G
NC_040267.1	53686306	A	T
NC_040267.1	54476223	T	C
NC_040267.1	54800511	A	C
NC_040267.1	54908344	T	C
NC_040267.1	55464246	G	A
NC_040267.1	55521821	G	A
NC_040267.1	55588216	T	C
NC_040267.1	55638345	A	G
NC_040267.1	55734349	A	G
NC_040267.1	56023613	T	C
NC_040267.1	56104100	G	C
NC_040267.1	56150517	C	G
NC_040267.1	56292156	G	A
NC_040267.1	56488035	T	A
NC_040267.1	56970523	G	C
NC_040267.1	57046606	T	C
NC_040267.1	57113708	G	A
NC_040267.1	57151208	T	C
NC_040267.1	57513830	T	C
NC_040267.1	57624318	G	A
NC_040267.1	57790668	A	G
NC_040267.1	57873733	C	T
NC_040267.1	57920417	C	T

NC_040267.1	57994222	A	G
NC_040267.1	58134274	A	G
NC_040267.1	58268244	A	G
NC_040267.1	58453920	G	A
NC_040267.1	59171423	C	T
NC_040267.1	59340489	C	A
NC_040267.1	59444136	T	A
NC_040267.1	59711944	A	G
NC_040267.1	59899018	A	G
NC_040267.1	59972764	C	T
NC_040267.1	60015287	A	C
NC_040267.1	60301249	T	C
NC_040267.1	60335609	T	C
NC_040267.1	60371551	A	C
NC_040267.1	60434270	A	G
NC_040267.1	60644308	T	G
NC_040267.1	60689468	A	G
NC_040267.1	60728778	T	G
NC_040267.1	60890495	C	T
NC_040267.1	60992548	C	T
NC_040267.1	61098953	A	G
NC_040267.1	61145400	A	G
NC_040267.1	61158312	G	T
NC_040267.1	61228665	C	T
NC_040267.1	61289619	A	G
NC_040267.1	61325749	C	A
NC_040267.1	61340368	C	T
NC_040267.1	61371988	A	G
NC_040267.1	61398933	C	T
NC_040267.1	61426360	T	C
NC_040267.1	61464237	G	A
NC_040267.1	61496143	G	A
NC_040267.1	61506119	T	C
NC_040267.1	61622931	A	C
NC_040267.1	61648100	G	T
NC_040267.1	61671221	T	C
NC_040267.1	61834255	T	C
NC_040267.1	61859382	A	G
NC_040267.1	61892821	C	T
NC_040267.1	61957187	T	C
NC_040267.1	62003929	A	T
NC_040267.1	62053141	T	C
NC_040267.1	62108136	G	A
NC_040267.1	62144130	A	C
NC_040267.1	62188201	T	G
NC_040267.1	62196142	T	C
NC_040267.1	62248059	T	C

NC_040267.1	62299672	T	C
NC_040267.1	62347665	A	T
NC_040267.1	62377584	T	C
NC_040267.1	62409777	C	G
NC_040267.1	62438678	T	C
NC_040267.1	62515122	T	C
NC_040267.1	62540321	C	T
NC_040267.1	62567598	A	G
NC_040267.1	62622017	G	T
NC_040267.1	62687113	T	C
NC_040267.1	62763593	A	C
NC_040267.1	62819308	G	A
NC_040267.1	62878210	T	C
NC_040267.1	62908106	C	T
NC_040267.1	62950108	C	T
NC_040267.1	62954875	G	C
NC_040267.1	63006869	C	T
NC_040267.1	63009557	T	C
NC_040267.1	63078982	A	C
NC_040267.1	63121783	T	C
NC_040267.1	63157485	A	G
NC_040267.1	63173289	G	T
NC_040267.1	63335975	T	C
NC_040267.1	63395781	C	T
NC_040267.1	63444625	A	G
NC_040267.1	63475349	G	A
NC_040267.1	63520486	C	T
NC_040267.1	63554543	A	C
NC_040267.1	63579069	T	A
NC_040267.1	63622815	A	G
NC_040267.1	63657787	G	C
NC_040267.1	63713363	T	C
NC_040267.1	63773079	C	T
NC_040267.1	63823283	A	G
NC_040267.1	63842405	A	C
NC_040267.1	63842808	G	A
NC_040267.1	63873960	G	T
NC_040267.1	63912722	G	A
NC_040267.1	63959991	G	A
NC_040267.1	63989240	C	T
NC_040267.1	64031147	G	C
NC_040267.1	64076036	G	A
NC_040267.1	64125622	G	A
NC_040267.1	64154085	T	C
NC_040267.1	64188711	T	G
NC_040267.1	64239470	G	C
NC_040267.1	64294576	G	A

NC_040267.1	64349853	A	G
NC_040267.1	64412919	A	G
NC_040267.1	64448559	T	C
NC_040267.1	64472676	T	C
NC_040267.1	64512232	A	G
NC_040267.1	64554865	A	G
NC_040267.1	64583324	G	A
NC_040267.1	64667604	A	G
NC_040267.1	64720623	A	G
NC_040267.1	64755736	T	C
NC_040267.1	64809763	T	C
NC_040267.1	64878933	C	G
NC_040267.1	64909483	A	G
NC_040267.1	64916163	G	A
NC_040267.1	64953347	G	A
NC_040267.1	64955620	T	G
NC_040267.1	65035933	C	T
NC_040267.1	65071941	C	T
NC_040267.1	65078904	T	C
NC_040267.1	65125757	G	C
NC_040267.1	65135210	T	G
NC_040267.1	65163207	T	C
NC_040267.1	65196241	G	A
NC_040267.1	65256629	T	C
NC_040267.1	65312950	G	A
NC_040267.1	65371963	A	G
NC_040267.1	65415289	A	G
NC_040267.1	65427759	G	A
NC_040267.1	65651858	G	A
NC_040267.1	65697585	G	A
NC_040267.1	65738908	G	C
NC_040267.1	65753125	T	A
NC_040267.1	65812291	T	C
NC_040267.1	65846461	G	A
NC_040267.1	66232256	A	G
NC_040267.1	66386782	A	G
NC_040267.1	66442387	A	G
NC_040267.1	66476467	T	C
NC_040267.1	66481369	G	C
NC_040267.1	66598241	A	G
NC_040267.1	66622996	T	C
NC_040267.1	66688308	A	G
NC_040267.1	66695396	G	C
NC_040267.1	66862449	A	G
NC_040267.1	66921071	G	A
NC_040267.1	66978361	T	A
NC_040267.1	67003521	A	G

NC_040267.1	67033090	A	G
NC_040267.1	67082026	T	C
NC_040267.1	67144827	A	G
NC_040267.1	67207655	C	T
NC_040267.1	67246406	G	A
NC_040267.1	67257858	A	G
NC_040267.1	67314203	G	A
NC_040267.1	67351014	C	T
NC_040267.1	67370437	T	G
NC_040267.1	67399928	T	C
NC_040267.1	67432486	G	T
NC_040267.1	67619248	T	G
NC_040267.1	67649034	G	A
NC_040267.1	67668586	A	G
NC_040267.1	67698825	A	G
NC_040267.1	67723609	G	T
NC_040267.1	67787425	A	C
NC_040267.1	67789527	A	G
NC_040267.1	67846139	A	G
NC_040267.1	67855298	A	G
NC_040267.1	67884065	C	A
NC_040267.1	67904785	G	C
NC_040267.1	67937291	A	G
NC_040267.1	67952299	A	G
NC_040267.1	67995371	C	T
NC_040267.1	68007865	A	G
NC_040267.1	68055651	G	A
NC_040267.1	68059700	A	G
NC_040267.1	68091265	C	T
NC_040267.1	68115857	A	G
NC_040267.1	68170075	G	A
NC_040267.1	68227329	C	T
NC_040267.1	68253662	T	A
NC_040267.1	68295388	G	A
NC_040267.1	68309519	C	T
NC_040267.1	68373626	C	T
NC_040267.1	68423978	G	C
NC_040267.1	68476990	C	A
NC_040267.1	68526594	G	A
NC_040267.1	68565470	T	G
NC_040267.1	68566316	T	G
NC_040267.1	68567435	A	G
NC_040267.1	68567769	C	T
NC_040267.1	68614899	G	A
NC_040267.1	68645481	T	A
NC_040267.1	68662504	T	C
NC_040267.1	68713132	T	C

NC_040267.1	68766039	C	T
NC_040267.1	68782269	T	A
NC_040267.1	68793414	T	C
NC_040267.1	68848048	G	C
NC_040267.1	68905658	C	T
NC_040267.1	68925698	T	C
NC_040267.1	68926051	C	T
NC_040267.1	68939608	A	G
NC_040267.1	68991855	A	G
NC_040267.1	69044328	G	A
NC_040267.1	69088013	A	T
NC_040267.1	69090806	T	C
NC_040267.1	69143685	A	G
NC_040267.1	69191216	C	T
NC_040267.1	69231017	G	A
NC_040267.1	69248409	T	C
NC_040267.1	69301159	G	A
NC_040267.1	69355629	T	G
NC_040267.1	69398361	T	C
NC_040267.1	69459840	A	G
NC_040267.1	69513130	G	A
NC_040267.1	69567825	G	A
NC_040267.1	69623017	C	T
NC_040267.1	69661599	G	A
NC_040267.1	69669767	T	C
NC_040267.1	69725787	T	C
NC_040267.1	69781073	G	A
NC_040267.1	69830761	T	G
NC_040267.1	69869931	G	A
NC_040267.1	69888603	G	C
NC_040267.1	69948235	C	G
NC_040267.1	69993877	A	G
NC_040267.1	70054452	C	T
NC_040267.1	70110468	A	G
NC_040267.1	70168677	G	A
NC_040267.1	70169325	T	C
NC_040267.1	70225379	A	G
NC_040267.1	70280774	T	C
NC_040267.1	70312799	T	C
NC_040267.1	70351118	T	G
NC_040267.1	70408942	C	T
NC_040267.1	70434924	C	G
NC_040267.1	70468547	C	T
NC_040267.1	70513786	A	T
NC_040267.1	70557312	C	A
NC_040267.1	70639889	T	A
NC_040267.1	70669499	A	G

NC_040267.1	70696409	C		T
NC_040267.1	70759541	C		T
NC_040267.1	70825598	G		A
NC_040267.1	70859343	T		C
NC_040267.1	70888353	C		T
NC_040267.1	70941234	A		G
NC_040267.1	71014620	G		A
NC_040267.1	71040572	A		G
NC_040267.1	71077562	T		G
NC_040267.1	71090264	T		C
NC_040267.1	71155823	T		C
NC_040267.1	71236272	T		C
NC_040267.1	71265172	T		G
NC_040267.1	71332765	G		A
NC_040267.1	71360001	A		G
NC_040267.1	71415577	A		G
NC_040267.1	71456481	A		G
NC_040267.1	71517925	C		G
NC_040267.1	71543388	C		T
NC_040267.1	71577181	G		T
NC_040267.1	71639710	A		T
NC_040267.1	71698299	G		A
NC_040267.1	71831357	T		A
NC_040267.1	71849236	C		G
NC_040267.1	71902016	C		T
NC_040267.1	71956318	T		G
NC_040267.1	72013465	A		G
NC_040267.1	72067991	G		A
NC_040267.1	72120413	T		C
NC_040267.1	72171223	G		A
NC_040267.1	72210774	C		T
NC_040267.1	72243138		0	A
NC_040267.1	72257535	C		T
NC_040267.1	72362549	A		G
NC_040267.1	72392992	C		A
NC_040267.1	72420795	A		G
NC_040267.1	72472845	T		G
NC_040267.1	72520915	G		A
NC_040267.1	72531409	C		T
NC_040267.1	72574895	G		A
NC_040267.1	72594860	T		C
NC_040267.1	72595063	A		G
NC_040267.1	72605845	A		G
NC_040267.1	72662372	G		A
NC_040267.1	72704035	A		C
NC_040267.1	72737508	G		C
NC_040267.1	72777360	T		C

NC_040267.1	72814152	T	C
NC_040267.1	72875994	G	T
NC_040267.1	72933541	T	C
NC_040267.1	72984624	A	G
NC_040267.1	73036166	T	C
NC_040267.1	73094843	A	G
NC_040267.1	73124252	C	T
NC_040267.1	73146014	C	T
NC_040267.1	73207431	A	G
NC_040267.1	73258380	T	A
NC_040267.1	73295204	G	A
NC_040267.1	73333937	A	G
NC_040267.1	73347052	A	G
NC_040267.1	73398178	A	T
NC_040267.1	73455031	T	C
NC_040267.1	73503736	T	C
NC_040267.1	73553808	C	T
NC_040267.1	73720756	A	G
NC_040267.1	73766349	G	A
NC_040267.1	73767434	G	A
NC_040267.1	73856591	C	T
NC_040267.1	73954521	T	C
NC_040267.1	74004852	A	G
NC_040267.1	74034938	A	G
NC_040267.1	74058755	T	C
NC_040267.1	74060702	T	C
NC_040267.1	74118458	A	G
NC_040267.1	74163218	G	A
NC_040267.1	74200847	T	G
NC_040267.1	74244057	A	G
NC_040267.1	74275654	A	G
NC_040267.1	74323250	T	A
NC_040267.1	74372075	T	C
NC_040267.1	74425147	G	C
NC_040267.1	74485478	G	A
NC_040267.1	74543114	A	G
NC_040267.1	74603324	G	A
NC_040267.1	74655141	T	C
NC_040267.1	74683238	T	C
NC_040267.1	74708200	T	C
NC_040267.1	74764696	A	G
NC_040267.1	74821268	A	G
NC_040267.1	74870015	T	G
NC_040267.1	74936910	A	G
NC_040267.1	75038170	G	A
NC_040267.1	75094178	A	G
NC_040267.1	75156038	T	C

NC_040267.1	75192500	A	T	
NC_040267.1	75227626	A	G	
NC_040267.1	75245899	G	A	
NC_040267.1	75277325	G	C	
NC_040267.1	75302397	T	C	
NC_040267.1	75302548	T	C	
NC_040267.1	75362361	T	C	
NC_040267.1	75413589	T	C	
NC_040267.1	75466263	T	C	
NC_040267.1	75499839	A	G	
NC_040267.1	75518112	A	G	
NC_040267.1	75570319	T	C	
NC_040267.1	75611977	G	A	
NC_040267.1	75644811	A	G	
NC_040267.1	75663852	T	C	
NC_040267.1	75711801	A	T	
NC_040267.1	75768732	C	T	
NC_040267.1	75769052	C	T	
NC_040267.1	75820821	C	G	
NC_040267.1	75879830	G	C	
NC_040267.1	75939618	C	G	
NC_040267.1	75979911	G	A	
NC_040267.1	75996808	A	G	
NC_040267.1	76050037	A	G	
NC_040267.1	76106700	T	C	
NC_040267.1	76163098	G	C	
NC_040267.1	76214216	A	G	
NC_040267.1	76265744	A	G	
NC_040267.1	76289821	G	A	
NC_040267.1	76411026	A	G	
NC_040267.1	76450825	T	C	
NC_040267.1	76494298	G	A	
NC_040267.1	76494790	G	A	
NC_040267.1	76522841	A	G	
NC_040267.1	76553165		0	0
NC_040267.1	76563605	G	A	
NC_040267.1	76612463	T	C	
NC_040267.1	76650369	T	C	
NC_040267.1	76689870	A	C	
NC_040267.1	76712636	T	C	
NC_040267.1	76770566	T	C	
NC_040267.1	76829027	C	T	
NC_040267.1	76871057	C	T	
NC_040267.1	76884116	A	G	
NC_040267.1	76920804	C	T	
NC_040267.1	76936643	G	A	
NC_040267.1	76989129	A	G	

NC_040267.1	77031898	C		T
NC_040267.1	77058516	T		C
NC_040267.1	77086885	G		A
NC_040267.1	77132141	G		A
NC_040267.1	77189031	G		T
NC_040267.1	77248372	G		A
NC_040267.1	77297047	T		G
NC_040267.1	77337582	A		G
NC_040267.1	77337821	C		G
NC_040267.1	77356208	G		C
NC_040267.1	77358206	A		G
NC_040267.1	77408508	C		T
NC_040267.1	77468674	G		A
NC_040267.1	77526842	G		A
NC_040267.1	77527580	A		G
NC_040267.1	77573693	C		G
NC_040267.1	77597047	A		C
NC_040267.1	77607575	C		T
NC_040267.1	77628865	T		C
NC_040267.1	77639440	G		C
NC_040267.1	77669372	C		T
NC_040267.1	77676480	A		C
NC_040267.1	77677939	T		C
NC_040267.1	77684365	A		G
NC_040267.1	77730909	A		G
NC_040267.1	77768716	A		G
NC_040267.1	77772023	A		G
NC_040267.1	77795512		0	A
NC_040267.1	77798675	G		A
NC_040267.1	77862343	A		G
NC_040267.1	77882779	T		C
NC_040267.1	77895660	T		C
NC_040267.1	77911208	C		A
NC_040267.1	77936245	T		C
NC_040267.1	77946429	G		A
NC_040267.1	77975834	A		G
NC_040267.1	77975992	A		G
NC_040267.1	77976188	C		T
NC_040267.1	77986210	C		T
NC_040267.1	77988229	A		G
NC_040267.1	78039786	A		G
NC_040267.1	78094133	A		G
NC_040267.1	78117512	C		T
NC_040267.1	78121728	A		G
NC_040267.1	78134167	T		C
NC_040267.1	78134711	A		G
NC_040267.1	78136028	C		A

NC_040267.1	78136253	A	T
NC_040267.1	78136471	C	A
NC_040267.1	78147563	T	C
NC_040267.1	78167990	T	C
NC_040267.1	78174717	T	C
NC_040267.1	78179780	C	G
NC_040267.1	78235818	T	C
NC_040267.1	78287245	A	T
NC_040267.1	78293170	T	C
NC_040267.1	78297309	A	G
NC_040267.1	78308553	C	G
NC_040267.1	78319378	C	T
NC_040267.1	78326371	A	G
NC_040268.1	601579	T	C
NC_040268.1	616634	C	G
NC_040268.1	630521	T	G
NC_040268.1	778206	G	C
NC_040268.1	835808	A	G
NC_040268.1	894028	A	C
NC_040268.1	960493	G	A
NC_040268.1	1022376	A	G
NC_040268.1	1081221	A	G
NC_040268.1	1125094	0	G
NC_040268.1	1152319	G	T
NC_040268.1	2046338	G	A
NC_040268.1	2088886	G	A
NC_040268.1	2138750	A	G
NC_040268.1	2152524	T	C
NC_040268.1	2213386	G	A
NC_040268.1	2305594	A	G
NC_040268.1	2336346	G	A
NC_040268.1	2454786	C	T
NC_040268.1	3016257	C	T
NC_040268.1	3068802	A	G
NC_040268.1	3143631	C	T
NC_040268.1	3330170	A	G
NC_040268.1	3331323	C	T
NC_040268.1	3353167	T	C
NC_040268.1	3355441	C	G
NC_040268.1	3376108	A	G
NC_040268.1	3404622	A	G
NC_040268.1	3542795	T	C
NC_040268.1	3599033	C	T
NC_040268.1	3645594	T	G
NC_040268.1	3669613	C	T
NC_040268.1	3720584	G	T
NC_040268.1	3779970	G	A

NC_040268.1	3830714	C	T
NC_040268.1	4573247	C	T
NC_040268.1	4650632	G	A
NC_040268.1	4679760	A	G
NC_040268.1	4691000	T	C
NC_040268.1	4691809	G	A
NC_040268.1	4692048	A	C
NC_040268.1	4727626	A	G
NC_040268.1	4728434	C	T
NC_040268.1	4786811	T	C
NC_040268.1	4844298	T	C
NC_040268.1	4902426	A	C
NC_040268.1	4974634	A	G
NC_040268.1	5019585	T	A
NC_040268.1	5041756	C	T
NC_040268.1	5043562	A	G
NC_040268.1	5044312	C	T
NC_040268.1	5107401	G	A
NC_040268.1	5166033	G	T
NC_040268.1	5456307	A	G
NC_040268.1	5458964	G	A
NC_040268.1	5469493	A	G
NC_040268.1	5516218	A	G
NC_040268.1	5548793	T	C
NC_040268.1	5601301	A	G
NC_040268.1	5652315	T	C
NC_040268.1	5662273	G	A
NC_040268.1	5663480	A	G
NC_040268.1	5754012	T	G
NC_040268.1	5776842	G	A
NC_040268.1	5801741	G	A
NC_040268.1	5802573	T	C
NC_040268.1	6015393	A	T
NC_040268.1	6042631	C	T
NC_040268.1	6104118	G	A
NC_040268.1	6750140	T	C
NC_040268.1	6806027	T	C
NC_040268.1	6870806	G	C
NC_040268.1	6914103	A	C
NC_040268.1	6943250	A	G
NC_040268.1	6971551	C	T
NC_040268.1	7023493	A	G
NC_040268.1	7085072	C	T
NC_040268.1	7132130	G	C
NC_040268.1	7254069	T	C
NC_040268.1	7296428	T	C
NC_040268.1	7345038	G	A

NC_040268.1	7567745		0	C
NC_040268.1	7618018	T		C
NC_040268.1	7713964	G		T
NC_040268.1	7786030	A		G
NC_040268.1	7836061	G		A
NC_040268.1	7875136	G		A
NC_040268.1	7928867	G		A
NC_040268.1	7958648	G		A
NC_040268.1	8008909	A		G
NC_040268.1	8060862	A		G
NC_040268.1	8119068	A		G
NC_040268.1	8171677	C		A
NC_040268.1	8228692	C		T
NC_040268.1	8274732	A		G
NC_040268.1	8388488	G		A
NC_040268.1	8432760	A		G
NC_040268.1	8468740	A		G
NC_040268.1	8493428	T		C
NC_040268.1	8552838	C		A
NC_040268.1	8610191	T		C
NC_040268.1	8651793	T		A
NC_040268.1	8698814	G		T
NC_040268.1	9084803	G		A
NC_040268.1	9140081	A		T
NC_040268.1	9196475	T		C
NC_040268.1	9251136	C		A
NC_040268.1	9310443	G		A
NC_040268.1	9371150	C		T
NC_040268.1	9409372	C		T
NC_040268.1	9557295	T		G
NC_040268.1	9842860	C		G
NC_040268.1	9971183	C		A
NC_040268.1	10002564	C		A
NC_040268.1	10052464	A		G
NC_040268.1	10110303	G		A
NC_040268.1	10179580	G		A
NC_040268.1	10210722	A		G
NC_040268.1	10261630	T		C
NC_040268.1	10282885	C		G
NC_040268.1	10334867	T		C
NC_040268.1	10382435	A		G
NC_040268.1	10424872	C		T
NC_040268.1	10514647	A		G
NC_040268.1	10557665	A		G
NC_040268.1	10581565	T		C
NC_040268.1	10717009	A		G
NC_040268.1	10816225	G		A

NC_040268.1	10839167	G	T
NC_040268.1	10840782	A	C
NC_040268.1	10847221	T	C
NC_040268.1	10891515	C	T
NC_040268.1	10946206	A	G
NC_040268.1	10989176	C	T
NC_040268.1	11037605	C	T
NC_040268.1	11088770	G	A
NC_040268.1	11142399	C	A
NC_040268.1	11196318	T	C
NC_040268.1	11242335	C	G
NC_040268.1	11272606	G	A
NC_040268.1	11323553	C	T
NC_040268.1	11378887	C	T
NC_040268.1	11426022	T	C
NC_040268.1	11480115	T	A
NC_040268.1	11542526	A	G
NC_040268.1	11602515	A	G
NC_040268.1	11655480	A	G
NC_040268.1	11716328	G	A
NC_040268.1	11781574	G	C
NC_040268.1	11855972	G	A
NC_040268.1	11873546	G	C
NC_040268.1	11928019	A	G
NC_040268.1	11928366	A	C
NC_040268.1	12541522	C	T
NC_040268.1	12584073	G	A
NC_040268.1	12611366	A	G
NC_040268.1	12661492	C	T
NC_040268.1	12715919	T	A
NC_040268.1	12763938	C	T
NC_040268.1	12809451	G	A
NC_040268.1	12871543	T	C
NC_040268.1	13069838	G	A
NC_040268.1	13122511	C	T
NC_040268.1	13176922	A	C
NC_040268.1	13227744	A	G
NC_040268.1	13269045	T	G
NC_040268.1	13332594	C	T
NC_040268.1	13364466	C	T
NC_040268.1	13414517	C	T
NC_040268.1	13415345	T	C
NC_040268.1	13450378	C	T
NC_040268.1	13676340	T	C
NC_040268.1	13731013	G	A
NC_040268.1	13786290	G	A
NC_040268.1	13834528	C	T

NC_040268.1	13859194	T	C
NC_040268.1	13866344	C	A
NC_040268.1	13888184	G	C
NC_040268.1	13935363	A	G
NC_040268.1	13966813	G	C
NC_040268.1	14037413	C	T
NC_040268.1	14071877	T	C
NC_040268.1	14106510	G	A
NC_040268.1	14403840	T	C
NC_040268.1	14415449	T	C
NC_040268.1	14424036	T	C
NC_040268.1	14439718	T	C
NC_040268.1	14440416	G	A
NC_040268.1	14440786	T	C
NC_040268.1	14440982	A	C
NC_040268.1	14441458	A	G
NC_040268.1	14441691	G	T
NC_040268.1	14446615	C	T
NC_040268.1	14498233	G	A
NC_040268.1	14562803	A	C
NC_040268.1	14756626	G	A
NC_040268.1	14760469	C	T
NC_040268.1	14788054	G	A
NC_040268.1	14843143	G	A
NC_040268.1	14869430	T	G
NC_040268.1	15629461	G	A
NC_040268.1	15675397	T	C
NC_040268.1	15732682	C	T
NC_040268.1	15766046	T	C
NC_040268.1	15797826	C	G
NC_040268.1	15816351	T	C
NC_040268.1	15818622	T	C
NC_040268.1	15871947	C	T
NC_040268.1	15914098	A	G
NC_040268.1	15920264	A	G
NC_040268.1	15920713	A	G
NC_040268.1	16064108	A	C
NC_040268.1	16089697	A	G
NC_040268.1	16350133	C	G
NC_040268.1	16406672	C	T
NC_040268.1	16461240	G	A
NC_040268.1	16509796	A	G
NC_040268.1	16566609	C	T
NC_040268.1	16597893	C	T
NC_040268.1	16632341	G	A
NC_040268.1	16685690	T	C
NC_040268.1	16739993	A	G

NC_040268.1	16764646	G	T
NC_040268.1	16808294	T	C
NC_040268.1	16842025	G	C
NC_040268.1	16899231	A	G
NC_040268.1	16952893	G	A
NC_040268.1	17001151	A	C
NC_040268.1	17053918	A	G
NC_040268.1	17104956	G	A
NC_040268.1	17152717	C	T
NC_040268.1	17196950	C	G
NC_040268.1	17234683	T	C
NC_040268.1	17443337	C	T
NC_040268.1	17498502	T	C
NC_040268.1	17539845	A	G
NC_040268.1	17540924	C	T
NC_040268.1	17542155	G	A
NC_040268.1	18083898	C	T
NC_040268.1	18180534	T	C
NC_040268.1	18237617	T	C
NC_040268.1	18292764	A	G
NC_040268.1	18347360	T	G
NC_040268.1	18403382	A	C
NC_040268.1	18454336	G	T
NC_040268.1	18455615	T	C
NC_040268.1	18456216	T	C
NC_040268.1	18457354	G	A
NC_040268.1	18458467	A	C
NC_040268.1	18461052	G	A
NC_040268.1	18551417	C	T
NC_040268.1	18610600	G	A
NC_040268.1	18668853	T	G
NC_040268.1	18698214	A	G
NC_040268.1	18698371	G	T
NC_040268.1	18702844	A	G
NC_040268.1	18704463	A	G
NC_040268.1	18761157	G	A
NC_040268.1	18780220	T	C
NC_040268.1	18802519	C	G
NC_040268.1	18829602	C	T
NC_040268.1	18846848	C	A
NC_040268.1	18889618	A	C
NC_040268.1	19086083	T	C
NC_040268.1	19086910	T	C
NC_040268.1	19141969	A	G
NC_040268.1	19198684	A	T
NC_040268.1	19254793	G	A
NC_040268.1	19305309	A	G

NC_040268.1	19354884	A	G
NC_040268.1	19522600	A	G
NC_040268.1	19534328	T	C
NC_040268.1	19576310	C	T
NC_040268.1	19672952	C	T
NC_040268.1	19673484	T	G
NC_040268.1	19673912	G	A
NC_040268.1	19674084	T	C
NC_040268.1	19710941	A	G
NC_040268.1	19771588	T	C
NC_040268.1	19819895	A	G
NC_040268.1	19820833	C	T
NC_040268.1	19821374	C	T
NC_040268.1	19823671	T	A
NC_040268.1	19840317	A	G
NC_040268.1	19870541	T	C
NC_040268.1	19913330	A	C
NC_040268.1	19978744	C	G
NC_040268.1	20025725	T	C
NC_040268.1	20076077	A	T
NC_040268.1	20085526	A	G
NC_040268.1	20087394	A	G
NC_040268.1	20103561	C	A
NC_040268.1	20923548	C	T
NC_040268.1	20978406	A	G
NC_040268.1	21007709	C	T
NC_040268.1	21890118	C	T
NC_040268.1	21890959	G	T
NC_040268.1	21892027	C	T
NC_040268.1	21904029	T	A
NC_040268.1	21963910	C	T
NC_040268.1	22009694	A	T
NC_040268.1	22070127	A	T
NC_040268.1	22127087	T	C
NC_040268.1	22190153	G	A
NC_040268.1	22293859	T	A
NC_040268.1	22437870	C	T
NC_040268.1	22803668	G	A
NC_040268.1	22892560	A	C
NC_040268.1	22953529	T	C
NC_040268.1	23234988	A	G
NC_040268.1	23473543	C	T
NC_040268.1	23671387	A	G
NC_040268.1	24235668	A	C
NC_040268.1	25491814	C	T
NC_040268.1	25532244	G	A
NC_040268.1	25564662	C	G

NC_040268.1	25634733	G	A
NC_040268.1	26375376	T	C
NC_040268.1	26892830	C	T
NC_040268.1	27517389	A	T
NC_040268.1	27806226	C	T
NC_040268.1	27820052	G	A
NC_040268.1	27831288	C	G
NC_040268.1	27942968	C	T
NC_040268.1	28098039	T	A
NC_040268.1	28264340	T	C
NC_040268.1	28289626	T	C
NC_040268.1	28508797	G	C
NC_040268.1	28965052	A	G
NC_040268.1	29012098	C	T
NC_040268.1	29071717	0	A
NC_040268.1	29119871	A	G
NC_040268.1	29510720	A	T
NC_040268.1	29540255	G	A
NC_040268.1	29695420	G	A
NC_040268.1	29747376	T	C
NC_040268.1	29773943	T	C
NC_040268.1	29861155	G	T
NC_040268.1	29889978	A	C
NC_040268.1	29946445	A	G
NC_040268.1	29959543	A	G
NC_040268.1	30161254	G	A
NC_040268.1	30199463	G	A
NC_040268.1	30268473	T	C
NC_040268.1	30516428	A	G
NC_040268.1	30627106	A	T
NC_040268.1	30643577	G	A
NC_040268.1	30749237	A	G
NC_040268.1	30817449	A	G
NC_040268.1	30914529	G	A
NC_040268.1	30959917	G	A
NC_040268.1	31031787	A	C
NC_040268.1	31080519	C	T
NC_040268.1	31164775	C	A
NC_040268.1	31408919	C	G
NC_040268.1	31458703	C	T
NC_040268.1	31532532	T	C
NC_040268.1	31596334	T	G
NC_040268.1	31666399	C	T
NC_040268.1	31719567	A	T
NC_040268.1	31796298	T	C
NC_040268.1	31817373	C	T
NC_040268.1	31918016	T	G

NC_040268.1	31992170	G	A
NC_040268.1	32158744	C	T
NC_040268.1	32207118	A	G
NC_040268.1	32296887	G	A
NC_040268.1	32362975	T	C
NC_040268.1	32834388	T	C
NC_040268.1	32895737	A	G
NC_040268.1	32937557	A	C
NC_040268.1	32974077	T	A
NC_040268.1	33011785	C	T
NC_040268.1	33070072	A	G
NC_040268.1	33180595	T	C
NC_040268.1	33227410	G	A
NC_040268.1	33278761	A	T
NC_040268.1	33322530	T	C
NC_040268.1	36054232	T	C
NC_040268.1	36074217	G	A
NC_040268.1	36111755	C	T
NC_040268.1	36199937	A	G
NC_040268.1	36823692	C	T
NC_040268.1	36852218	C	T
NC_040268.1	37701740	C	T
NC_040268.1	37755864	T	C
NC_040268.1	37814515	G	A
NC_040268.1	37880013	A	G
NC_040268.1	37927897	G	A
NC_040268.1	37940296	G	T
NC_040268.1	37980222	T	C
NC_040268.1	38043499	A	G
NC_040268.1	38144757	G	A
NC_040268.1	38152471	T	C
NC_040268.1	38206303	T	C
NC_040268.1	38276653	T	C
NC_040268.1	38281776	A	G
NC_040268.1	38505046	C	T
NC_040268.1	38535277	A	G
NC_040268.1	38541182	G	A
NC_040268.1	38599101	A	G
NC_040268.1	38777045	T	C
NC_040268.1	38826689	A	G
NC_040268.1	38880237	T	C
NC_040268.1	38936130	A	G
NC_040268.1	38988683	G	A
NC_040268.1	39031880	C	G
NC_040268.1	39075434	C	T
NC_040268.1	40262139	T	C
NC_040268.1	40314319	C	T

NC_040268.1	40355065	G	A
NC_040268.1	40389757	T	C
NC_040268.1	40447209	C	T
NC_040268.1	40501431	T	C
NC_040268.1	40542275	T	C
NC_040268.1	40594374	C	T
NC_040268.1	40631874	T	C
NC_040268.1	40651958	T	C
NC_040268.1	40707569	A	G
NC_040268.1	40762164	G	C
NC_040268.1	40841351	A	G
NC_040268.1	41074291	C	T
NC_040268.1	41229704	T	C
NC_040268.1	41279477	T	A
NC_040268.1	41335012	A	G
NC_040268.1	41390342	T	C
NC_040268.1	41444184	A	G
NC_040268.1	41488991	G	A
NC_040268.1	41521602	G	A
NC_040268.1	41764045	C	G
NC_040268.1	41807896	C	T
NC_040268.1	42402014	A	T
NC_040268.1	42487695	T	C
NC_040268.1	42562230	T	G
NC_040268.1	42827477	C	T
NC_040268.1	42890644	A	G
NC_040268.1	42943648	T	C
NC_040268.1	43180391	C	T
NC_040268.1	43198693	T	C
NC_040268.1	43348675	T	C
NC_040268.1	43388952	G	A
NC_040268.1	43457748	C	T
NC_040268.1	46973340	C	T
NC_040268.1	47033495	T	C
NC_040268.1	47085394	C	T
NC_040268.1	47139468	T	C
NC_040268.1	47197780	G	A
NC_040268.1	47229215	G	C
NC_040268.1	47341078	T	G
NC_040268.1	47404112	C	T
NC_040268.1	47459005	G	A
NC_040268.1	47529767	C	T
NC_040268.1	47618304	G	A
NC_040268.1	47637216	A	G
NC_040268.1	47784999	C	T
NC_040268.1	48009164	G	T
NC_040268.1	48094947	A	G

NC_040268.1	48397260	A	G
NC_040268.1	48401521	T	C
NC_040268.1	49430200	A	G
NC_040268.1	49472410	T	C
NC_040268.1	49512895	C	G
NC_040268.1	49682981	C	T
NC_040268.1	49722034	A	G
NC_040268.1	49777462	C	G
NC_040268.1	49830872	A	T
NC_040268.1	49867502	C	T
NC_040268.1	49984486	C	T
NC_040268.1	50028505	T	C
NC_040268.1	50092605	C	T
NC_040268.1	50144028	T	G
NC_040268.1	50219478	T	C
NC_040268.1	51322027	C	G
NC_040268.1	51362936	A	T
NC_040268.1	51403407	C	T
NC_040268.1	51465267	T	C
NC_040268.1	51474462	G	A
NC_040268.1	51513758	C	G
NC_040268.1	51556287	C	T
NC_040268.1	51556447	A	G
NC_040268.1	51557514	G	A
NC_040268.1	51605812	G	A
NC_040268.1	51916661	C	T
NC_040268.1	51918199	T	C
NC_040268.1	51935318	A	G
NC_040268.1	51936178	A	G
NC_040268.1	51973813	A	G
NC_040268.1	52006449	A	C
NC_040268.1	52007623	G	A
NC_040268.1	52007777	T	C
NC_040268.1	52008464	A	C
NC_040268.1	52054101	A	G
NC_040268.1	52054395	G	A
NC_040268.1	52079828	T	C
NC_040268.1	52080536	G	C
NC_040268.1	52082717	C	T
NC_040268.1	52083005	C	T
NC_040268.1	52095842	C	T
NC_040268.1	52097703	G	A
NC_040268.1	52106222	G	A
NC_040268.1	52159091	G	A
NC_040268.1	52167110	T	C
NC_040268.1	52171204	G	A
NC_040268.1	52171411	A	G

NC_040268.1	52173331	A	G
NC_040268.1	52174422	T	C
NC_040268.1	52202437	A	G
NC_040268.1	52267045	T	C
NC_040268.1	52290833	T	C
NC_040268.1	52291049	A	T
NC_040268.1	52334598	T	G
NC_040268.1	52347206	C	T
NC_040268.1	52348810	G	A
NC_040268.1	52350681	C	A
NC_040268.1	52378876	C	T
NC_040268.1	52379047	A	G
NC_040268.1	52381667	T	C
NC_040268.1	52384261	A	G
NC_040268.1	52402793	A	C
NC_040268.1	52402979	G	T
NC_040268.1	52428962	T	C
NC_040268.1	52438220	T	C
NC_040268.1	52440250	C	G
NC_040268.1	52440562	A	G
NC_040268.1	52441727	T	C
NC_040268.1	52446514	T	C
NC_040268.1	52462914	A	G
NC_040268.1	52497467	C	T
NC_040268.1	52498451	C	T
NC_040268.1	52499218	T	C
NC_040268.1	52500874	T	C
NC_040268.1	52555158	C	T
NC_040268.1	52580315	G	A
NC_040268.1	52728422	A	C
NC_040268.1	52785500	A	C
NC_040268.1	52838328	C	T
NC_040268.1	52895259	A	G
NC_040268.1	52895841	T	C
NC_040268.1	52896149	T	C
NC_040268.1	52897782	T	C
NC_040268.1	52900754	A	G
NC_040268.1	52903040	G	A
NC_040268.1	52903955	T	C
NC_040268.1	52904166	A	G
NC_040268.1	52904457	A	G
NC_040268.1	52925525	C	T
NC_040268.1	52936166	C	G
NC_040268.1	52940782	G	C
NC_040268.1	52943282	A	G
NC_040268.1	52949277	A	C
NC_040268.1	52963439	A	G

NC_040268.1	52975739	G	A
NC_040268.1	53009490	T	C
NC_040268.1	53035517	A	G
NC_040268.1	53058813	T	C
NC_040268.1	53059498	A	G
NC_040268.1	53059751	G	A
NC_040268.1	53062891	T	C
NC_040268.1	53063087	G	C
NC_040268.1	53124843	C	T
NC_040268.1	53125307	T	G
NC_040268.1	53126609	A	G
NC_040268.1	53135171	G	A
NC_040268.1	53137093	T	C
NC_040268.1	53161770	T	C
NC_040268.1	53203380	G	A
NC_040268.1	53239633	C	G
NC_040268.1	53239844	G	C
NC_040268.1	53944045	A	G
NC_040268.1	53984135	G	A
NC_040268.1	54040817	A	G
NC_040268.1	54101132	C	T
NC_040268.1	54181160	G	A
NC_040268.1	54216806	C	G
NC_040268.1	54250692	C	T
NC_040268.1	54284489	T	C
NC_040268.1	54485513	T	C
NC_040268.1	54538208	A	G
NC_040268.1	54580893	G	A
NC_040268.1	54606963	G	A
NC_040268.1	54649239	A	C
NC_040268.1	54828500	C	G
NC_040268.1	55244409	C	T
NC_040268.1	55297026	C	G
NC_040268.1	55352703	G	A
NC_040268.1	55404901	G	A
NC_040268.1	55463847	A	C
NC_040268.1	55519673	T	C
NC_040268.1	55569843	G	T
NC_040268.1	55683336	G	A
NC_040268.1	55717669	T	G
NC_040268.1	55764328	G	A
NC_040268.1	55815442	G	A
NC_040268.1	55855251	T	C
NC_040268.1	56080934	G	C
NC_040268.1	56138614	C	T
NC_040268.1	56184636	T	C
NC_040268.1	56234638	T	C

NC_040268.1	56322963	A	C
NC_040268.1	56323668	G	A
NC_040268.1	56324055	C	T
NC_040268.1	56381003	T	C
NC_040268.1	56431667	A	G
NC_040268.1	56483745	T	C
NC_040268.1	56537717	T	C
NC_040268.1	56581819	A	G
NC_040268.1	56610741	A	G
NC_040268.1	58177903	T	C
NC_040268.1	58178852	T	C
NC_040268.1	58180431	G	A
NC_040268.1	58180892	A	G
NC_040268.1	58236729	T	C
NC_040268.1	58296496	G	A
NC_040268.1	58351765	G	A
NC_040268.1	58384574	A	G
NC_040268.1	59712729	G	A
NC_040268.1	59727698	A	G
NC_040268.1	59761216	T	C
NC_040268.1	59803135	G	T
NC_040268.1	59804516	T	C
NC_040268.1	59825927	A	G
NC_040268.1	59830059	G	C
NC_040268.1	59841386	T	C
NC_040268.1	59850598	T	A
NC_040268.1	59854450	A	G
NC_040268.1	59857959	T	C
NC_040268.1	59858209	A	G
NC_040268.1	59860499	T	C
NC_040268.1	59860765	A	G
NC_040268.1	59884577	A	T
NC_040268.1	59886259	T	C
NC_040268.1	59939570	C	A
NC_040268.1	60001001	T	C
NC_040268.1	60001280	C	G
NC_040268.1	60001487	A	G
NC_040268.1	60010523	G	C
NC_040268.1	60010873	G	C
NC_040268.1	60243145	C	T
NC_040268.1	60301500	G	A
NC_040268.1	60350292	T	C
NC_040268.1	60400167	G	A
NC_040268.1	60423197	A	G
NC_040268.1	60434178	C	T
NC_040268.1	60439752	A	G
NC_040268.1	60440512	G	A

NC_040268.1	60440747	A	G
NC_040268.1	60448200	T	C
NC_040268.1	60450748	A	G
NC_040268.1	60695844	G	A
NC_040268.1	60697421	T	C
NC_040268.1	60729807	G	A
NC_040268.1	60754723	C	T
NC_040268.1	60755476	T	C
NC_040268.1	60769406	T	C
NC_040268.1	60770980	G	A
NC_040268.1	60826207	A	G
NC_040268.1	60845347	T	A
NC_040268.1	60862649	C	T
NC_040268.1	60887679	T	C
NC_040268.1	60892477	C	T
NC_040268.1	60933330	T	C
NC_040268.1	60956922	A	G
NC_040268.1	60972941	T	C
NC_040268.1	60987920	T	C
NC_040268.1	61049129	T	C
NC_040268.1	61079399	C	T
NC_040268.1	61131664	A	G
NC_040268.1	61149264	A	G
NC_040268.1	61165895	T	A
NC_040268.1	61199663	G	C
NC_040268.1	61199867	C	G
NC_040268.1	61201306	G	A
NC_040268.1	61237969	G	A
NC_040268.1	61241992	G	A
NC_040268.1	61278161	A	T
NC_040268.1	61278633	G	A
NC_040268.1	61278867	T	C
NC_040268.1	61279333	A	G
NC_040268.1	61279845	A	G
NC_040268.1	61281885	A	G
NC_040268.1	61282415	T	C
NC_040268.1	61323404	A	G
NC_040268.1	61336239	G	A
NC_040268.1	61345688	T	A
NC_040268.1	61376233	A	G
NC_040268.1	61376486	C	T
NC_040268.1	61377658	T	A
NC_040268.1	61378474	C	T
NC_040268.1	61434082	T	C
NC_040268.1	61482745	G	A
NC_040268.1	61501991	G	T
NC_040268.1	61524587	T	C

NC_040268.1	61526251	A	G
NC_040268.1	61535516	T	C
NC_040268.1	61536880	G	A
NC_040268.1	61537716	A	G
NC_040268.1	61538780	C	T
NC_040268.1	61539756	T	C
NC_040268.1	61573072	A	G
NC_040268.1	61576723	C	T
NC_040268.1	61584615	C	T
NC_040268.1	61624807	G	A
NC_040268.1	61647542	G	A
NC_040268.1	61650057	T	C
NC_040268.1	61650872	T	C
NC_040268.1	61653322	T	C
NC_040268.1	61676456	C	G
NC_040268.1	61721226	G	A
NC_040268.1	61767353	A	G
NC_040268.1	61768321	A	G
NC_040268.1	61768893	G	A
NC_040268.1	61784742	A	C
NC_040268.1	61786084	G	A
NC_040268.1	61838785	A	T
NC_040268.1	61840006	A	G
NC_040268.1	61853427	T	C
NC_040268.1	61865577	A	G
NC_040268.1	61868402	T	C
NC_040268.1	61936909	A	G
NC_040268.1	61951730	A	G
NC_040268.1	61951963	A	G
NC_040268.1	61952241	T	C
NC_040268.1	62017398	A	G
NC_040268.1	62017604	T	C
NC_040268.1	62046322	T	C
NC_040268.1	62052296	G	C
NC_040268.1	62096304	A	C
NC_040268.1	62107964	A	G
NC_040268.1	62132032	T	C
NC_040268.1	62132741	A	G
NC_040268.1	62137912	C	T
NC_040268.1	62167306	A	G
NC_040268.1	62205181	A	G
NC_040268.1	62206093	T	C
NC_040268.1	62206395	A	G
NC_040268.1	62208127	A	G
NC_040268.1	62208316	A	G
NC_040268.1	62208945	G	C
NC_040268.1	62209135	T	C

NC_040268.1	62214004	T	C
NC_040268.1	62262108	C	G
NC_040268.1	62308280	C	G
NC_040268.1	62362257	A	G
NC_040268.1	62406526	T	C
NC_040268.1	62413840	A	G
NC_040268.1	62454081	A	G
NC_040268.1	62455590	C	T
NC_040268.1	62461431	C	T
NC_040268.1	62461611	T	C
NC_040268.1	62461838	T	C
NC_040268.1	62465841	A	G
NC_040268.1	62470415	A	G
NC_040268.1	62470598	T	C
NC_040268.1	62477624	T	C
NC_040268.1	62477956	G	A
NC_040268.1	62478727	T	A
NC_040268.1	62481320	G	T
NC_040268.1	62481501	A	G
NC_040268.1	62483189	T	C
NC_040268.1	62483423	T	G
NC_040268.1	62484948	T	C
NC_040268.1	62485507	C	T
NC_040268.1	62485731	G	A
NC_040268.1	62486042	G	A
NC_040268.1	62486584	G	C
NC_040268.1	62501441	T	C
NC_040268.1	62540402	A	G
NC_040268.1	62568645	C	A
NC_040268.1	62571414	A	G
NC_040268.1	62579001	A	G
NC_040268.1	62584325	T	C
NC_040268.1	62584575	T	G
NC_040268.1	62584731	G	A
NC_040268.1	62640313	G	A
NC_040268.1	62683453	A	G
NC_040268.1	62737563	T	C
NC_040268.1	62739867	A	G
NC_040268.1	62745233	C	A
NC_040268.1	62749199	A	G
NC_040268.1	62749560	A	T
NC_040268.1	62751790	A	C
NC_040268.1	62812750	A	G
NC_040268.1	62843076	A	G
NC_040268.1	62903231	T	C
NC_040268.1	62903577	T	C
NC_040268.1	62904155	T	C

NC_040268.1	62904404	A	G
NC_040268.1	62904694	G	T
NC_040268.1	62913096	T	C
NC_040268.1	62923916	T	C
NC_040268.1	62953294	A	G
NC_040268.1	62972624	G	A
NC_040268.1	63002756	A	G
NC_040268.1	63052786	T	C
NC_040268.1	63110934	A	G
NC_040268.1	63143993	G	A
NC_040268.1	63199252	A	T
NC_040268.1	63241657	A	C
NC_040268.1	63242991	T	C
NC_040268.1	63250582	A	G
NC_040268.1	63250775	T	C
NC_040268.1	63266558	A	C
NC_040268.1	63269694	A	G
NC_040268.1	63336268	T	C
NC_040268.1	63345561	A	G
NC_040268.1	63345822	G	A
NC_040268.1	63371998	G	T
NC_040268.1	63373494	C	T
NC_040268.1	63383408	G	T
NC_040268.1	63384694	T	A
NC_040268.1	63385257	C	T
NC_040268.1	63385697	G	C
NC_040268.1	63385897	C	T
NC_040268.1	63386410	T	A
NC_040268.1	63423964	A	C
NC_040268.1	63483864	A	G
NC_040268.1	63487273	T	C
NC_040268.1	63547105	C	T
NC_040268.1	63654680	G	T
NC_040268.1	63688944	A	G
NC_040268.1	63978165	A	G
NC_040268.1	64033240	A	G
NC_040268.1	64064409	A	G
NC_040268.1	64071275	T	C
NC_040268.1	64075676	A	G
NC_040268.1	64076981	T	C
NC_040268.1	64077321	G	A
NC_040268.1	64077604	G	A
NC_040268.1	64088729	A	G
NC_040268.1	64125947	T	C
NC_040268.1	64126140	A	G
NC_040268.1	64149535	G	A
NC_040268.1	64151462	T	C

NC_040268.1	64206579	A	G
NC_040268.1	64253456	0	0
NC_040268.1	64281544	C	T
NC_040268.1	64282994	G	C
NC_040268.1	64283891	T	C
NC_040268.1	64312249	C	G
NC_040268.1	64368062	T	C
NC_040268.1	64390735	G	C
NC_040268.1	64400179	G	T
NC_040268.1	64412206	A	G
NC_040268.1	64412828	G	T
NC_040268.1	64413125	C	G
NC_040268.1	64525961	T	G
NC_040268.1	64578174	A	G
NC_040268.1	64616485	T	G
NC_040268.1	64618272	C	T
NC_040268.1	64628334	T	C
NC_040268.1	64660790	C	T
NC_040268.1	64681187	T	C
NC_040268.1	64697063	A	G
NC_040268.1	64697605	T	C
NC_040268.1	64697761	G	A
NC_040268.1	64697965	G	T
NC_040268.1	64698557	T	C
NC_040268.1	64698762	T	C
NC_040268.1	64699219	A	G
NC_040268.1	64707042	T	C
NC_040268.1	64739899	G	A
NC_040268.1	64748742	G	T
NC_040268.1	64784355	T	C
NC_040268.1	64840114	C	G
NC_040268.1	64891012	T	C
NC_040268.1	64947592	C	A
NC_040268.1	64993110	T	C
NC_040268.1	65106946	G	A
NC_040268.1	65117859	A	G
NC_040268.1	65136355	A	G
NC_040268.1	65146895	T	C
NC_040268.1	65199654	A	G
NC_040268.1	65254013	G	C
NC_040268.1	65305978	C	G
NC_040268.1	65810752	C	A
NC_040268.1	65865083	A	T
NC_040268.1	65934789	C	A
NC_040268.1	65969682	T	A
NC_040268.1	66024410	A	G
NC_040268.1	66050446	A	G

NC_040268.1	66106177	T	A
NC_040268.1	66122227	C	A
NC_040268.1	66144057	T	C
NC_040268.1	66145961	C	T
NC_040268.1	66146114	T	C
NC_040268.1	66147136	G	T
NC_040268.1	66147393	T	A
NC_040268.1	66148049	T	G
NC_040268.1	66150629	T	C
NC_040268.1	66193857	G	T
NC_040268.1	66245455	C	T
NC_040268.1	66300230	C	T
NC_040268.1	66342818	C	T
NC_040268.1	66393385	T	C
NC_040268.1	66451247	A	G
NC_040268.1	66506975	A	G
NC_040268.1	66561069	A	C
NC_040268.1	66617630	A	G
NC_040268.1	66675408	T	G
NC_040268.1	66770985	A	G
NC_040268.1	66810810	A	G
NC_040268.1	66837025	G	T
NC_040268.1	66857365	A	G
NC_040268.1	66859102	T	C
NC_040268.1	66860619	A	G
NC_040268.1	66862384	T	C
NC_040268.1	66862558	G	A
NC_040268.1	66862908	A	G
NC_040268.1	66863155	C	T
NC_040268.1	66863383	G	C
NC_040268.1	66877170	C	G
NC_040268.1	66913593	T	C
NC_040268.1	66914452	C	A
NC_040268.1	66915016	T	C
NC_040268.1	66953408	T	C
NC_040268.1	66993524	C	G
NC_040268.1	67035942	C	T
NC_040268.1	67102140	C	T
NC_040268.1	67119883	G	C
NC_040268.1	67161713	A	G
NC_040268.1	67162780	G	C
NC_040268.1	67163535	C	T
NC_040268.1	67176528	G	T
NC_040268.1	67210623	T	C
NC_040268.1	67220764	A	G
NC_040268.1	67249857	C	T
NC_040268.1	67597855	G	C

NC_040268.1	67653054	A	G
NC_040268.1	67708667	A	T
NC_040268.1	67764347	G	T
NC_040268.1	67815650	T	C
NC_040268.1	67830992	C	T
NC_040268.1	67858100	A	G
NC_040268.1	67858343	0	C
NC_040268.1	67877043	A	G
NC_040268.1	67878324	C	T
NC_040268.1	69110863	T	C
NC_040268.1	69119545	G	C
NC_040268.1	69122029	T	C
NC_040268.1	69289657	T	C
NC_040268.1	69290090	T	G
NC_040268.1	69291965	G	A
NC_040268.1	69292528	A	G
NC_040268.1	69312145	A	G
NC_040268.1	69330014	T	C
NC_040268.1	69341603	T	C
NC_040268.1	69341790	A	G
NC_040268.1	69342604	A	C
NC_040268.1	69342853	T	C
NC_040268.1	69343381	C	T
NC_040268.1	69643629	A	G
NC_040268.1	69664157	T	C
NC_040268.1	69720470	G	A
NC_040268.1	69762782	A	G
NC_040268.1	70010829	T	C
NC_040268.1	70011354	T	C
NC_040268.1	70068156	A	G
NC_040268.1	70107561	A	C
NC_040268.1	70107722	A	G
NC_040268.1	70144840	C	T
NC_040268.1	70196276	A	C
NC_040268.1	70217727	T	C
NC_040268.1	70226503	A	G
NC_040268.1	70252490	T	C
NC_040268.1	70267089	A	G
NC_040268.1	70312356	T	C
NC_040268.1	70315151	G	A
NC_040268.1	70343273	G	A
NC_040268.1	70393527	T	C
NC_040268.1	70400905	G	A
NC_040268.1	70403548	C	T
NC_040268.1	70408479	C	G
NC_040268.1	70433577	G	C
NC_040268.1	70443613	A	C

NC_040268.1	70505885	G	A
NC_040268.1	70506063	C	T
NC_040268.1	70538310	T	G
NC_040268.1	70590038	C	T
NC_040268.1	70642973	T	C
NC_040268.1	70696593	A	G
NC_040268.1	70747541	T	C
NC_040268.1	70783591	T	C
NC_040268.1	70957008	A	G
NC_040268.1	70959479	C	T
NC_040268.1	70959675	A	G
NC_040268.1	70972167	G	A
NC_040268.1	71022469	C	G
NC_040268.1	71042951	C	G
NC_040268.1	71088580	A	T
NC_040268.1	71121609	T	C
NC_040268.1	71165631	A	G
NC_040268.1	71190428	A	G
NC_040268.1	71202632	A	G
NC_040268.1	71206256	T	C
NC_040268.1	71206519	T	C
NC_040268.1	71206832	A	G
NC_040268.1	71215404	T	C
NC_040268.1	71221079	C	T
NC_040268.1	71221491	A	G
NC_040268.1	71223152	A	G
NC_040268.1	71223509	A	G
NC_040268.1	71223709	T	C
NC_040268.1	71224343	T	C
NC_040268.1	71224883	T	G
NC_040268.1	71225062	A	C
NC_040268.1	71228624	A	G
NC_040268.1	71228982	A	G
NC_040268.1	71235813	G	A
NC_040268.1	71243725	C	T
NC_040268.1	71295300	T	C
NC_040268.1	71314850	T	C
NC_040268.1	71329925	C	G
NC_040268.1	71372568	A	T
NC_040268.1	71381077	G	T
NC_040268.1	71401575	G	A
NC_040268.1	71426268	C	T
NC_040268.1	71459020	T	C
NC_040268.1	71511067	T	C
NC_040268.1	71528590	A	G
NC_040268.1	71592495	A	G
NC_040268.1	71647136	A	G

NC_040268.1	71672582	G	C
NC_040268.1	71673395	T	C
NC_040268.1	71673676	C	T
NC_040268.1	71674092	T	G
NC_040268.1	71684402	C	T
NC_040268.1	71703054	A	G
NC_040268.1	71712377	A	G
NC_040268.1	71712643	A	G
NC_040268.1	71716413	A	G
NC_040268.1	71731385	A	G
NC_040268.1	71740086	A	G
NC_040268.1	71758012	A	G
NC_040268.1	71762825	C	T
NC_040268.1	71764003	A	G
NC_040268.1	71764252	A	G
NC_040268.1	71767986	T	C
NC_040268.1	71769475	C	T
NC_040268.1	71773568	T	C
NC_040268.1	71831541	A	G
NC_040268.1	71847429	C	G
NC_040268.1	71940610	C	T
NC_040268.1	71941082	G	A
NC_040268.1	71952062	C	A
NC_040268.1	71991557	A	G
NC_040268.1	72029678	G	A
NC_040268.1	72093084	G	A
NC_040268.1	72093766	T	G
NC_040268.1	72113216	A	G
NC_040268.1	72137512	T	C
NC_040268.1	72137699	C	T
NC_040268.1	72137899	G	A
NC_040268.1	72138093	G	A
NC_040268.1	72140913	G	T
NC_040268.1	72141874	G	A
NC_040268.1	72154726	G	A
NC_040268.1	72156589	G	A
NC_040268.1	72157054	A	G
NC_040268.1	72158424	A	G
NC_040268.1	72179897	G	C
NC_040268.1	72181680	C	T
NC_040268.1	72221708	T	A
NC_040268.1	72275833	T	C
NC_040268.1	72318834	G	C
NC_040268.1	72367137	T	C
NC_040268.1	72376854	T	C
NC_040268.1	72414552	A	G
NC_040268.1	72469597	T	G

NC_040268.1	72469820	A	G
NC_040268.1	72470120	T	C
NC_040268.1	72470443	C	T
NC_040268.1	72502883	G	A
NC_040268.1	72513242	T	C
NC_040268.1	72517549	C	T
NC_040268.1	72541378	C	T
NC_040268.1	72545482	T	C
NC_040268.1	72552274	T	C
NC_040268.1	72600007	C	T
NC_040268.1	72600164	C	G
NC_040268.1	72634440	C	T
NC_040268.1	72721120	G	T
NC_040268.1	72814646	A	G
NC_040268.1	72833472	C	T
NC_040268.1	72853378	A	G
NC_040268.1	72918400	A	G
NC_040268.1	72963998	G	A
NC_040268.1	72974579	A	G
NC_040268.1	73031742	G	C
NC_040268.1	73033310	A	G
NC_040268.1	73047033	C	T
NC_040268.1	73055808	G	A
NC_040268.1	73058188	C	T
NC_040268.1	73118990	C	T
NC_040268.1	73146754	A	G
NC_040268.1	73193638	C	T
NC_040268.1	73210224	G	T
NC_040268.1	73210532	A	G
NC_040268.1	73211151	A	C
NC_040268.1	73212948	T	C
NC_040268.1	73215255	T	C
NC_040268.1	73217690	A	G
NC_040268.1	73220503	T	C
NC_040268.1	73235681	T	C
NC_040268.1	73250383	T	C
NC_040268.1	73317022	T	G
NC_040268.1	73323608	A	G
NC_040268.1	73323828	C	G
NC_040268.1	73383263	A	G
NC_040268.1	73395634	A	G
NC_040268.1	73406300	G	A
NC_040268.1	73407052	G	A
NC_040268.1	73407387	T	C
NC_040268.1	73409359	G	A
NC_040268.1	73462884	T	C
NC_040268.1	73508063	T	C

NC_040268.1	73509489	A	G
NC_040268.1	73513490	T	C
NC_040268.1	73514416	G	A
NC_040268.1	73515425	G	A
NC_040268.1	73539697	C	T
NC_040268.1	73542887	C	T
NC_040268.1	73584900	G	A
NC_040268.1	73589410	A	G
NC_040268.1	73616372	A	G
NC_040268.1	73624561	G	A
NC_040268.1	73633367	G	T
NC_040268.1	73633557	A	G
NC_040268.1	73633740	A	G
NC_040268.1	73634923	A	G
NC_040268.1	73769710	A	G
NC_040268.1	73809212	A	G
NC_040268.1	73809564	A	G
NC_040268.1	73809925	T	C
NC_040268.1	73810099	T	C
NC_040268.1	73835428	T	C
NC_040268.1	73835664	A	G
NC_040268.1	73836408	A	G
NC_040268.1	73902325	G	A
NC_040268.1	73950116	A	G
NC_040268.1	74218433	A	G
NC_040268.1	74263686	T	C
NC_040268.1	74295530	A	G
NC_040268.1	74332733	G	A
NC_040268.1	74371583	C	A
NC_040268.1	74407976	G	T
NC_040268.1	74410742	G	A
NC_040268.1	74411142	C	G
NC_040268.1	74470650	A	G
NC_040268.1	74534125	C	T
NC_040268.1	74534306	G	T
NC_040268.1	74534975	A	T
NC_040268.1	74535129	T	C
NC_040268.1	74563420	T	C
NC_040268.1	74636928	G	T
NC_040268.1	74690467	G	A
NC_040268.1	74723398	C	T
NC_040268.1	74751247	G	A
NC_040268.1	74777412	T	C
NC_040268.1	74788284	T	C
NC_040268.1	74843901	G	A
NC_040268.1	74894852	G	A
NC_040268.1	74941677	T	C

NC_040268.1	74998678	A	G
NC_040268.1	75246680	T	C
NC_040268.1	75310116	A	G
NC_040268.1	75312466	C	T
NC_040268.1	75313133	T	C
NC_040268.1	75348771	C	A
NC_040268.1	75356860	G	A
NC_040268.1	75371389	A	G
NC_040268.1	75371593	A	G
NC_040268.1	75410148	G	A
NC_040268.1	75415196	T	C
NC_040268.1	75417116	A	G
NC_040268.1	75434482	G	A
NC_040268.1	75477165	C	T
NC_040268.1	75490150	A	G
NC_040268.1	75519564	G	C
NC_040268.1	76602176	A	G
NC_040268.1	76602767	C	G
NC_040268.1	76645912	A	C
NC_040268.1	76646122	A	G
NC_040268.1	76646578	A	G
NC_040268.1	76646902	T	C
NC_040268.1	76648569	T	C
NC_040268.1	76649230	T	C
NC_040268.1	76704947	T	C
NC_040268.1	76757639	C	T
NC_040268.1	76815964	A	G
NC_040268.1	76816185	A	G
NC_040268.1	76816434	T	C
NC_040268.1	76816700	T	C
NC_040268.1	76873409	C	G
NC_040268.1	76908482	T	G
NC_040268.1	76963150	A	G
NC_040268.1	77022915	C	T
NC_040268.1	77083676	A	G
NC_040268.1	77139134	C	T
NC_040268.1	77193289	C	T
NC_040268.1	77225686	G	A
NC_040268.1	77226190	A	G
NC_040268.1	77281060	G	A
NC_040268.1	77333320	A	G
NC_040268.1	77360805	C	G
NC_040268.1	77381599	A	G
NC_040268.1	77383134	C	T
NC_040268.1	77383459	T	A
NC_040268.1	77383778	G	A
NC_040268.1	77384788	C	T

NC_040268.1	77385584	T	C
NC_040268.1	77418630	G	A
NC_040268.1	77420346	C	T
NC_040268.1	77443808	C	T
NC_040268.1	77453682	A	C
NC_040268.1	77453833	G	A
NC_040268.1	77453995	T	G
NC_040268.1	77509513	C	T
NC_040268.1	77562454	C	T
NC_040268.1	77613495	G	A
NC_040268.1	77636172	T	C
NC_040268.1	77644655	G	A
NC_040268.1	77646913	T	G
NC_040268.1	77712220	T	C
NC_040268.1	77712411	A	C
NC_040268.1	77758160	T	C
NC_040268.1	77782526	A	G
NC_040268.1	77786869	T	C
NC_040268.1	77791377	T	C
NC_040268.1	77798415	A	G
NC_040268.1	77798630	C	A
NC_040268.1	77852040	T	C
NC_040268.1	77894710	C	G
NC_040268.1	77934814	A	C
NC_040268.1	77937319	A	G
NC_040268.1	77950909	C	G
NC_040268.1	78006311	A	G
NC_040268.1	78056686	G	A
NC_040268.1	78097093	T	C
NC_040268.1	78131113	G	A
NC_040268.1	78151925	A	G
NC_040268.1	78183991	A	G
NC_040268.1	78214657	G	C
NC_040268.1	78215216	T	G
NC_040268.1	78215450	T	A
NC_040268.1	78215672	G	A
NC_040268.1	78215828	G	A
NC_040268.1	78216177	A	G
NC_040268.1	78270826	G	A
NC_040268.1	78323280	T	C
NC_040268.1	78382248	C	T
NC_040268.1	78405212	A	C
NC_040268.1	78436063	T	C
NC_040268.1	78498093	A	G
NC_040268.1	78498322	A	G
NC_040268.1	78499185	C	A
NC_040268.1	78504900	T	G

NC_040268.1	78536719	T		C
NC_040268.1	78543443	C		G
NC_040268.1	78571687	C		G
NC_040268.1	78643595	A		G
NC_040268.1	78661340	T		C
NC_040268.1	78661508	C		T
NC_040268.1	78680464	A		G
NC_040268.1	78680724	A		G
NC_040268.1	78702666	A		G
NC_040268.1	78703587	C		G
NC_040268.1	78703763	T		C
NC_040268.1	78714290	A		G
NC_040268.1	78720614	A		G
NC_040268.1	78735328	T		C
NC_040268.1	78736413	T		C
NC_040268.1	78738423	T		C
NC_040268.1	78773506	A		C
NC_040268.1	78799898	A		G
NC_040268.1	78840213	C		G
NC_040268.1	78850039	T		C
NC_040268.1	78858674	T		C
NC_040268.1	78859171	A		G
NC_040268.1	78866636	A		G
NC_040268.1	78866801	G		A
NC_040268.1	78921720	C		T
NC_040268.1	78992843	C		T
NC_040268.1	78996579	T		C
NC_040268.1	78996948	T		G
NC_040268.1	79026190	C		T
NC_040268.1	79058461	C		G
NC_040268.1	79142452	C		T
NC_040268.1	79163526	A		G
NC_040268.1	79166048	A		G
NC_040268.1	79174498	G		A
NC_040268.1	79184669		0	C
NC_040268.1	79190023	A		G
NC_040268.1	79206903	A		G
NC_040268.1	79211963	T		C
NC_040268.1	79224595	A		G
NC_040268.1	79226052	A		G
NC_040268.1	79272756	A		G
NC_040268.1	79281561	T		C
NC_040268.1	79284885	T		C
NC_040268.1	79292198	A		G
NC_040268.1	79316138	A		G
NC_040268.1	79328300	T		C
NC_040268.1	79368580	A		G

NC_040268.1	79374863	A	G
NC_040268.1	79376202	A	G
NC_040268.1	79399768	T	C
NC_040268.1	79444599	T	G
NC_040268.1	79485892	T	C
NC_040268.1	79494431	A	G
NC_040268.1	79539209	C	T
NC_040268.1	79539827	G	A
NC_040268.1	79541305	A	G
NC_040268.1	79541873	C	T
NC_040268.1	79555167	C	G
NC_040268.1	79556336	T	C
NC_040268.1	79557950	C	T
NC_040268.1	79593386	A	G
NC_040268.1	79622948	C	G
NC_040268.1	79627834	A	G
NC_040268.1	79681948	C	G
NC_040268.1	79684322	A	G
NC_040268.1	79689750	T	C
NC_040268.1	79766799	A	G
NC_040268.1	79794515	A	G
NC_040268.1	79807879	T	C
NC_040268.1	80362685	C	T
NC_040268.1	80363422	T	C
NC_040268.1	80614883	T	C
NC_040268.1	80642697	C	T
NC_040268.1	80691970	C	T
NC_040268.1	80692150	A	G
NC_040268.1	80694271	T	C
NC_040268.1	80711412	C	G
NC_040268.1	80753261	C	T
NC_040268.1	80792892	T	A
NC_040268.1	80795597	T	C
NC_040268.1	80807274	C	T
NC_040268.1	80807666	A	G
NC_040268.1	80810322	A	G
NC_040268.1	80826310	T	C
NC_040268.1	80830884	T	C
NC_040268.1	80845582	T	C
NC_040268.1	80888411	T	C
NC_040268.1	80888595	A	G
NC_040268.1	80906008	T	C
NC_040268.1	80921217	C	T
NC_040268.1	80927483	G	A
NC_040268.1	80927964	T	C
NC_040268.1	80931488	A	G
NC_040268.1	80932947	T	C

NC_040268.1	80936652	C	T
NC_040268.1	80937362	A	G
NC_040268.1	80944710	G	A
NC_040268.1	80945082	G	C
NC_040268.1	80978064	T	G
NC_040268.1	80986208	A	G
NC_040268.1	81015387	T	G
NC_040268.1	81060798	G	C
NC_040268.1	81061019	T	C
NC_040268.1	81061181	0	T
NC_040268.1	81061822	A	G
NC_040268.1	81080209	A	G
NC_040268.1	81080472	T	G
NC_040268.1	81134638	A	G
NC_040268.1	81134953	T	C
NC_040268.1	81177277	T	C
NC_040268.1	81223705	A	G
NC_040268.1	81290656	T	C
NC_040268.1	81295130	T	C
NC_040268.1	81311292	G	C
NC_040268.1	81335654	A	G
NC_040268.1	81380217	G	A
NC_040268.1	81421010	C	A
NC_040268.1	81425655	A	G
NC_040268.1	81431607	G	A
NC_040268.1	81452068	T	C
NC_040268.1	81503403	A	G
NC_040268.1	81518544	G	A
NC_040268.1	81518788	C	T
NC_040268.1	81519584	C	T
NC_040268.1	81519927	A	G
NC_040268.1	81536523	T	C
NC_040268.1	81557603	A	G
NC_040268.1	81576499	G	A
NC_040268.1	81577606	T	C
NC_040268.1	81578083	T	C
NC_040268.1	81581472	A	G
NC_040268.1	81589366	G	T
NC_040268.1	81591752	A	G
NC_040268.1	81591971	C	T
NC_040268.1	81593672	T	C
NC_040268.1	81607729	A	G
NC_040268.1	81626207	A	G
NC_040268.1	81626876	C	G
NC_040268.1	81628087	G	C
NC_040268.1	81628966	C	G
NC_040268.1	81629211	C	T

NC_040268.1	81629728	G	A
NC_040268.1	81657185	T	C
NC_040268.1	81657387	T	C
NC_040268.1	81674461	T	C
NC_040268.1	81675002	A	G
NC_040268.1	81675386	A	G
NC_040268.1	81677577	T	C
NC_040268.1	81697181	A	G
NC_040268.1	81697404	G	A
NC_040268.1	81698684	A	G
NC_040268.1	81699349	A	G
NC_040268.1	81706759	T	C
NC_040268.1	81718656	A	G
NC_040268.1	81722590	A	T
NC_040268.1	81729837	0	C
NC_040268.1	81765991	G	A
NC_040268.1	81776851	T	C
NC_040268.1	81785626	T	C
NC_040268.1	81794836	C	G
NC_040268.1	81800394	T	C
NC_040268.1	81813961	A	G
NC_040268.1	81828276	T	C
NC_040268.1	81832466	T	C
NC_040268.1	81874022	A	G
NC_040268.1	81885104	G	A
NC_040268.1	81889745	T	C
NC_040268.1	81909889	A	G
NC_040268.1	81910255	A	G
NC_040268.1	81951237	T	C
NC_040268.1	81951459	T	C
NC_040268.1	81958613	A	G
NC_040268.1	81959019	T	C
NC_040268.1	81969466	A	G
NC_040268.1	81972074	A	G
NC_040268.1	81972243	G	C
NC_040268.1	81972587	G	A
NC_040268.1	81997271	T	C
NC_040268.1	81997692	C	G
NC_040268.1	81997922	G	A
NC_040268.1	81998082	A	G
NC_040268.1	82021276	G	A
NC_040268.1	82021465	A	G
NC_040268.1	82023319	A	G
NC_040268.1	82048467	T	C
NC_040268.1	82049133	A	G
NC_040268.1	82053183	A	G
NC_040268.1	82054227	T	C

NC_040268.1	82054523	T	C
NC_040268.1	82075037	C	T
NC_040268.1	82107966	A	G
NC_040268.1	82108629	T	C
NC_040268.1	82161936	G	T
NC_040268.1	82193064	A	G
NC_040268.1	82193226	C	A
NC_040268.1	82193684	C	G
NC_040268.1	82247833	G	C
NC_040268.1	82250371	G	C
NC_040268.1	82251048	A	G
NC_040268.1	82251360	T	C
NC_040268.1	82251984	A	G
NC_040268.1	82253799	A	G
NC_040268.1	82254119	T	G
NC_040268.1	82294514	T	C
NC_040268.1	82294923	T	C
NC_040268.1	82295548	T	C
NC_040268.1	82303706	T	G
NC_040268.1	82318161	T	C
NC_040268.1	82318322	A	G
NC_040268.1	82318754	T	C
NC_040268.1	82318963	G	C
NC_040268.1	82319119	T	C
NC_040268.1	82319393	A	G
NC_040268.1	82353689	G	A
NC_040268.1	82369977	A	C
NC_040268.1	82376754	G	C
NC_040268.1	82379598	G	C
NC_040268.1	82404946	G	C
NC_040268.1	82421980	T	C
NC_040268.1	82431170	T	C
NC_040268.1	82433938	T	G
NC_040268.1	82447401	T	C
NC_040268.1	82448040	A	G
NC_040268.1	82464449	T	A
NC_040268.1	82464605	C	T
NC_040268.1	82466280	T	C
NC_040268.1	82471325	C	G
NC_040268.1	82521798	T	C
NC_040268.1	82522870	A	G
NC_040268.1	82565912	A	G
NC_040268.1	82566105	T	C
NC_040268.1	82574325	T	C
NC_040268.1	82584106	0	G
NC_040269.1	425044	T	C
NC_040269.1	425217	T	C

NC_040269.1	425542	T	G
NC_040269.1	476166	T	G
NC_040269.1	476522	G	A
NC_040269.1	478278	C	G
NC_040269.1	478494	T	C
NC_040269.1	479396	G	C
NC_040269.1	521382	A	G
NC_040269.1	594826	A	G
NC_040269.1	661329	A	T
NC_040269.1	723336	A	T
NC_040269.1	783863	C	T
NC_040269.1	842603	G	T
NC_040269.1	917547	A	G
NC_040269.1	968132	G	C
NC_040269.1	1029704	T	C
NC_040269.1	1117027	T	C
NC_040269.1	1126222	G	A
NC_040269.1	1137836	A	G
NC_040269.1	1168667	T	C
NC_040269.1	1333526	G	A
NC_040269.1	1394268	T	C
NC_040269.1	1461405	G	A
NC_040269.1	1527465	T	C
NC_040269.1	1599526	A	G
NC_040269.1	1623572	C	T
NC_040269.1	1624112	T	C
NC_040269.1	1661095	A	G
NC_040269.1	1692895	A	C
NC_040269.1	1754222	T	C
NC_040269.1	1810952	T	A
NC_040269.1	1863177	C	A
NC_040269.1	1908521	T	C
NC_040269.1	2255789	T	G
NC_040269.1	2362956	T	C
NC_040269.1	2415972	A	C
NC_040269.1	2469954	T	C
NC_040269.1	2525029	A	G
NC_040269.1	2575682	C	A
NC_040269.1	2595944	A	G
NC_040269.1	2655617	C	A
NC_040269.1	2717478	A	G
NC_040269.1	2783151	T	C
NC_040269.1	2844810	T	C
NC_040269.1	2881931	C	A
NC_040269.1	2930174	T	C
NC_040269.1	2974569	T	C
NC_040269.1	3039447	T	C

NC_040269.1	3097540	G	A
NC_040269.1	3156430	T	A
NC_040269.1	3233216	T	C
NC_040269.1	3292808	C	A
NC_040269.1	3364848	G	A
NC_040269.1	3431365	G	A
NC_040269.1	3479271	G	T
NC_040269.1	3522033	G	A
NC_040269.1	3524201	C	G
NC_040269.1	3579167	A	G
NC_040269.1	3635278	C	G
NC_040269.1	3691024	A	C
NC_040269.1	3751773	A	G
NC_040269.1	3809769	C	G
NC_040269.1	3952405	A	G
NC_040269.1	3982856	A	G
NC_040269.1	4030220	G	A
NC_040269.1	4079858	C	T
NC_040269.1	4136432	G	T
NC_040269.1	4191891	A	G
NC_040269.1	4225189	T	G
NC_040269.1	4313396	A	G
NC_040269.1	4357213	G	A
NC_040269.1	4423993	G	A
NC_040269.1	4489322	A	G
NC_040269.1	4542999	A	C
NC_040269.1	4592645	G	A
NC_040269.1	4652719	C	T
NC_040269.1	4705954	G	A
NC_040269.1	4761831	A	G
NC_040269.1	4822482	G	A
NC_040269.1	4875744	C	T
NC_040269.1	4947112	T	A
NC_040269.1	4999579	T	G
NC_040269.1	5061639	T	C
NC_040269.1	5202182	A	G
NC_040269.1	5240796	T	C
NC_040269.1	5297366	C	T
NC_040269.1	5337152	G	A
NC_040269.1	5338263	G	A
NC_040269.1	5380556	T	C
NC_040269.1	5698917	T	C
NC_040269.1	5699207	T	C
NC_040269.1	5699985	A	G
NC_040269.1	5700196	C	A
NC_040269.1	5701223	G	A
NC_040269.1	5747085	C	T

NC_040269.1	5776296	T	C
NC_040269.1	5778794	G	A
NC_040269.1	5781001	G	A
NC_040269.1	5781290	A	G
NC_040269.1	5832877	A	G
NC_040269.1	5873459	G	A
NC_040269.1	5921515	A	G
NC_040269.1	5959446	A	G
NC_040269.1	6054278	T	C
NC_040269.1	6104474	C	T
NC_040269.1	6154354	T	G
NC_040269.1	6209536	G	A
NC_040269.1	6266145	A	G
NC_040269.1	6308068	T	C
NC_040269.1	6361553	G	A
NC_040269.1	6408915	A	G
NC_040269.1	6442948	A	G
NC_040269.1	6540534	A	C
NC_040269.1	6541137	C	A
NC_040269.1	6542399	T	C
NC_040269.1	6542570	T	G
NC_040269.1	6545200	T	C
NC_040269.1	6591728	A	G
NC_040269.1	6591994	T	C
NC_040269.1	6593944	C	A
NC_040269.1	6594110	G	A
NC_040269.1	6639353	C	T
NC_040269.1	6687907	A	G
NC_040269.1	6735741	A	G
NC_040269.1	6776551	A	G
NC_040269.1	7012248	G	A
NC_040269.1	7060296	A	G
NC_040269.1	7109115	C	T
NC_040269.1	7164858	T	C
NC_040269.1	7214682	G	A
NC_040269.1	7269997	G	A
NC_040269.1	7325620	C	T
NC_040269.1	7379226	G	A
NC_040269.1	7379665	T	C
NC_040269.1	7379875	G	A
NC_040269.1	7380424	A	G
NC_040269.1	7380890	G	A
NC_040269.1	7381627	G	A
NC_040269.1	7383440	T	C
NC_040269.1	7396913	A	G
NC_040269.1	7575978	C	G
NC_040269.1	7629450	A	C

NC_040269.1	7682991	C	T
NC_040269.1	7739277	G	A
NC_040269.1	7794824	T	C
NC_040269.1	7851494	T	C
NC_040269.1	7874944	G	A
NC_040269.1	7875642	A	G
NC_040269.1	7876204	A	G
NC_040269.1	7877506	A	G
NC_040269.1	7877726	T	C
NC_040269.1	7878495	A	G
NC_040269.1	7878657	C	G
NC_040269.1	7940647	C	A
NC_040269.1	7941538	A	G
NC_040269.1	7991911	A	G
NC_040269.1	8025088	A	G
NC_040269.1	8082786	C	A
NC_040269.1	8145635	T	C
NC_040269.1	8201565	A	G
NC_040269.1	8277123	G	A
NC_040269.1	8361365	A	G
NC_040269.1	8436760	C	G
NC_040269.1	8525946	C	T
NC_040269.1	8596037	A	C
NC_040269.1	8689383	A	T
NC_040269.1	8767671	G	A
NC_040269.1	8838168	T	C
NC_040269.1	8909797	T	C
NC_040269.1	8964975	G	A
NC_040269.1	9014861	T	C
NC_040269.1	9096650	C	T
NC_040269.1	9184802	A	G
NC_040269.1	9252384	A	G
NC_040269.1	9307844	G	A
NC_040269.1	9386428	T	C
NC_040269.1	9457767	T	C
NC_040269.1	9522450	C	T
NC_040269.1	9581237	A	G
NC_040269.1	9636648	A	C
NC_040269.1	9692917	T	C
NC_040269.1	9756515	G	A
NC_040269.1	9819974	G	A
NC_040269.1	9888565	C	T
NC_040269.1	9962631	A	G
NC_040269.1	10018451	C	T
NC_040269.1	10084067	A	G
NC_040269.1	10143428	C	T
NC_040269.1	10212790	G	A

NC_040269.1	10283738	A	T
NC_040269.1	10343123	T	G
NC_040269.1	10409354	G	C
NC_040269.1	10486474	G	A
NC_040269.1	10550113	C	T
NC_040269.1	10613130	G	T
NC_040269.1	10677216	C	T
NC_040269.1	10740460	G	A
NC_040269.1	10802601	C	T
NC_040269.1	10859477	T	C
NC_040269.1	10915035	A	C
NC_040269.1	10974476	C	G
NC_040269.1	11033544	C	A
NC_040269.1	11107550	T	C
NC_040269.1	11167085	T	C
NC_040269.1	11224152	T	C
NC_040269.1	11290160	T	C
NC_040269.1	11350614	T	C
NC_040269.1	11420819	A	G
NC_040269.1	11485981	T	C
NC_040269.1	11547069	T	A
NC_040269.1	11618069	A	C
NC_040269.1	11674211	G	A
NC_040269.1	11738246	C	A
NC_040269.1	11802127	C	T
NC_040269.1	11870038	C	A
NC_040269.1	11936454	A	G
NC_040269.1	11976759	C	A
NC_040269.1	12037377	C	T
NC_040269.1	12105500	A	G
NC_040269.1	12177733	A	G
NC_040269.1	12511821	C	T
NC_040269.1	12573889	G	A
NC_040269.1	12626298	C	T
NC_040269.1	12684313	T	A
NC_040269.1	12753316	A	G
NC_040269.1	12809675	A	G
NC_040269.1	12868391	G	A
NC_040269.1	12930166	T	C
NC_040269.1	13002624	A	G
NC_040269.1	13067084	C	T
NC_040269.1	13127936	A	G
NC_040269.1	13198527	T	G
NC_040269.1	13256433	T	C
NC_040269.1	13300662	A	G
NC_040269.1	13364487	A	G
NC_040269.1	13427219	A	G

NC_040269.1	13495290	C	T
NC_040269.1	13548061	A	G
NC_040269.1	13600322	C	A
NC_040269.1	13664272	T	C
NC_040269.1	13726052	A	G
NC_040269.1	13788661	T	A
NC_040269.1	13843231	C	T
NC_040269.1	13908726	G	A
NC_040269.1	13972471	A	C
NC_040269.1	14028325	C	T
NC_040269.1	14071194	G	A
NC_040269.1	14095308	G	A
NC_040269.1	14095476	G	A
NC_040269.1	14095626	A	G
NC_040269.1	14117953	A	G
NC_040269.1	14150891	A	C
NC_040269.1	14151540	A	G
NC_040269.1	14192663	T	A
NC_040269.1	14231627	T	C
NC_040269.1	14243160	G	C
NC_040269.1	14413939	T	C
NC_040269.1	14550634	G	A
NC_040269.1	14605749	T	C
NC_040269.1	14666271	C	T
NC_040269.1	14700014	T	C
NC_040269.1	14700411	C	G
NC_040269.1	14701382	G	A
NC_040269.1	14701868	C	T
NC_040269.1	14702629	T	C
NC_040269.1	14702962	T	G
NC_040269.1	14703290	T	C
NC_040269.1	14722674	A	G
NC_040269.1	14771688	T	G
NC_040269.1	15033304	A	G
NC_040269.1	15081248	G	T
NC_040269.1	15127568	T	C
NC_040269.1	15191181	T	C
NC_040269.1	15244522	G	A
NC_040269.1	15303786	T	C
NC_040269.1	15361754	T	C
NC_040269.1	15402967	T	C
NC_040269.1	15548013	A	G
NC_040269.1	15745709	A	G
NC_040269.1	15806752	A	G
NC_040269.1	15862883	A	C
NC_040269.1	15920869	C	G
NC_040269.1	15977911	A	G

NC_040269.1	16039635	G	A
NC_040269.1	16087689	A	G
NC_040269.1	16087879	T	C
NC_040269.1	16088297	T	C
NC_040269.1	16096145	C	G
NC_040269.1	16096304	A	C
NC_040269.1	16151370	C	G
NC_040269.1	16209216	T	C
NC_040269.1	16263631	G	A
NC_040269.1	16324076	A	C
NC_040269.1	16371047	T	C
NC_040269.1	16428685	T	C
NC_040269.1	16488071	C	T
NC_040269.1	16544133	C	G
NC_040269.1	16600978	T	A
NC_040269.1	16679485	C	A
NC_040269.1	16721839	C	A
NC_040269.1	16729651	G	A
NC_040269.1	16731349	A	G
NC_040269.1	16741228	G	A
NC_040269.1	16773358	G	A
NC_040269.1	16785278	G	A
NC_040269.1	16785431	A	G
NC_040269.1	16840406	A	G
NC_040269.1	16845125	T	C
NC_040269.1	16850184	T	C
NC_040269.1	16851126	G	C
NC_040269.1	16851441	A	G
NC_040269.1	16851966	G	T
NC_040269.1	16880594	A	G
NC_040269.1	16890530	A	T
NC_040269.1	17076490	T	C
NC_040269.1	17078896	T	C
NC_040269.1	17139032	C	T
NC_040269.1	17520487	G	T
NC_040269.1	17562190	C	T
NC_040269.1	17809281	0	C
NC_040269.1	17841833	A	C
NC_040269.1	17843817	C	T
NC_040269.1	17860040	T	C
NC_040269.1	17877345	T	C
NC_040269.1	17879878	G	A
NC_040269.1	17896572	A	T
NC_040269.1	17897157	C	A
NC_040269.1	17897611	A	G
NC_040269.1	17912149	G	A
NC_040269.1	18010450	T	G

NC_040269.1	18066433	C	T
NC_040269.1	18119907	A	G
NC_040269.1	18120989	T	C
NC_040269.1	18121633	C	A
NC_040269.1	18122223	A	G
NC_040269.1	18122990	T	C
NC_040269.1	18129463	T	C
NC_040269.1	18129958	A	G
NC_040269.1	18174840	A	G
NC_040269.1	18216888	C	G
NC_040269.1	18227843	T	C
NC_040269.1	18232183	C	T
NC_040269.1	18361932	C	T
NC_040269.1	18372662	A	G
NC_040269.1	18379723	A	G
NC_040269.1	18441074	T	C
NC_040269.1	18451056	A	G
NC_040269.1	18476222	A	G
NC_040269.1	18487240	T	C
NC_040269.1	18496640	A	G
NC_040269.1	18513654	A	G
NC_040269.1	18530828	C	T
NC_040269.1	18571009	C	T
NC_040269.1	18612217	A	G
NC_040269.1	18619650	A	G
NC_040269.1	18624826	C	T
NC_040269.1	18674989	A	G
NC_040269.1	18675329	C	T
NC_040269.1	18675685	G	A
NC_040269.1	18676353	T	C
NC_040269.1	18676936	A	C
NC_040269.1	18677198	T	C
NC_040269.1	18724085	C	T
NC_040269.1	18830426	T	G
NC_040269.1	18889293	A	G
NC_040269.1	18904079	T	C
NC_040269.1	18910291	A	G
NC_040269.1	18921182	T	C
NC_040269.1	19012450	G	A
NC_040269.1	19031970	T	C
NC_040269.1	19040418	C	T
NC_040269.1	19084541	C	T
NC_040269.1	19091482	G	A
NC_040269.1	19112832	G	A
NC_040269.1	19115322	A	G
NC_040269.1	19135331	G	A
NC_040269.1	19146163	C	T

NC_040269.1	19150040	T	G
NC_040269.1	19191328	A	G
NC_040269.1	19198746	T	C
NC_040269.1	19199164	G	A
NC_040269.1	19200097	0	C
NC_040269.1	19200418	A	G
NC_040269.1	19200955	G	A
NC_040269.1	19206791	T	C
NC_040269.1	19207033	T	C
NC_040269.1	19260047	A	G
NC_040269.1	19278824	C	T
NC_040269.1	19307443	C	T
NC_040269.1	19309741	A	G
NC_040269.1	19310064	C	T
NC_040269.1	19339525	A	G
NC_040269.1	19405437	C	T
NC_040269.1	19405599	A	G
NC_040269.1	19407000	A	G
NC_040269.1	19407170	C	T
NC_040269.1	19426192	G	A
NC_040269.1	19500634	C	G
NC_040269.1	19519891	G	C
NC_040269.1	19520287	C	T
NC_040269.1	19533856	A	G
NC_040269.1	19538306	T	C
NC_040269.1	19540943	A	G
NC_040269.1	19542189	A	G
NC_040269.1	19574621	G	A
NC_040269.1	19649914	A	G
NC_040269.1	19703770	T	C
NC_040269.1	19705014	C	A
NC_040269.1	19705694	G	A
NC_040269.1	19706187	T	C
NC_040269.1	19719599	C	G
NC_040269.1	19730844	C	T
NC_040269.1	19733932	C	T
NC_040269.1	19761572	A	G
NC_040269.1	19776003	A	G
NC_040269.1	19831944	C	T
NC_040269.1	19840538	G	A
NC_040269.1	19841419	T	C
NC_040269.1	19860476	A	G
NC_040269.1	19861914	T	C
NC_040269.1	19863404	T	C
NC_040269.1	19894553	T	C
NC_040269.1	19912600	T	C
NC_040269.1	19913750	A	G

NC_040269.1	19913990	T	G
NC_040269.1	19914983	A	G
NC_040269.1	19920514	A	G
NC_040269.1	19925559	G	T
NC_040269.1	19926232	C	T
NC_040269.1	19929131	C	T
NC_040269.1	19940781	G	A
NC_040269.1	19951783	T	G
NC_040269.1	20000136	A	G
NC_040269.1	20000591	T	C
NC_040269.1	20076552	T	C
NC_040269.1	20133753	A	G
NC_040269.1	20198681	T	C
NC_040269.1	20260580	C	T
NC_040269.1	20268120	A	G
NC_040269.1	20279201	A	G
NC_040269.1	20337996	A	G
NC_040269.1	20396011	A	G
NC_040269.1	20462094	T	G
NC_040269.1	20503027	A	C
NC_040269.1	20515167	G	A
NC_040269.1	20571692	T	C
NC_040269.1	20625608	C	T
NC_040269.1	20684436	A	G
NC_040269.1	20920382	A	G
NC_040269.1	20956588	A	G
NC_040269.1	21036711	G	C
NC_040269.1	21093584	C	T
NC_040269.1	21142216	A	G
NC_040269.1	21201967	A	G
NC_040269.1	21202831	T	C
NC_040269.1	21203202	A	G
NC_040269.1	21207775	T	C
NC_040269.1	21208032	A	T
NC_040269.1	21212012	T	C
NC_040269.1	21279649	G	C
NC_040269.1	21372704	G	C
NC_040269.1	21440345	A	T
NC_040269.1	21507047	T	C
NC_040269.1	21557335	A	G
NC_040269.1	21589794	T	C
NC_040269.1	21647890	A	G
NC_040269.1	21690431	C	T
NC_040269.1	21731867	A	G
NC_040269.1	21746445	G	A
NC_040269.1	21802720	T	C
NC_040269.1	21859419	A	G

NC_040269.1	21901945	C	T
NC_040269.1	21946557	G	A
NC_040269.1	21947593	T	C
NC_040269.1	21948242	G	T
NC_040269.1	21948438	G	A
NC_040269.1	21948846	A	G
NC_040269.1	21956341	A	C
NC_040269.1	21962951	G	C
NC_040269.1	21964622	C	G
NC_040269.1	22021739	T	A
NC_040269.1	22055596	C	G
NC_040269.1	22055961	T	C
NC_040269.1	22075618	A	G
NC_040269.1	22148323	G	C
NC_040269.1	22149309	A	G
NC_040269.1	22150002	T	G
NC_040269.1	23057517	T	C
NC_040269.1	23097109	T	C
NC_040269.1	23149958	T	C
NC_040269.1	23167407	G	A
NC_040269.1	23217290	A	G
NC_040269.1	23374288	A	G
NC_040269.1	23374527	G	C
NC_040269.1	23496348	T	C
NC_040269.1	23496846	T	C
NC_040269.1	23497179	G	T
NC_040269.1	23875988	C	G
NC_040269.1	23882842	G	A
NC_040269.1	23956550	C	T
NC_040269.1	24020893	T	C
NC_040269.1	24063831	T	C
NC_040269.1	24064176	T	C
NC_040269.1	24065299	T	C
NC_040269.1	24065535	G	C
NC_040269.1	24106771	T	C
NC_040269.1	24203676	A	G
NC_040269.1	24237591	A	G
NC_040269.1	24300993	G	A
NC_040269.1	24364368	A	T
NC_040269.1	24366448	G	A
NC_040269.1	24457228	0	C
NC_040269.1	24507544	T	C
NC_040269.1	24551868	C	A
NC_040269.1	24611138	C	T
NC_040269.1	24666274	A	G
NC_040269.1	24706920	A	G
NC_040269.1	24708520	A	C

NC_040269.1	24709623	T	C
NC_040269.1	24740434	T	C
NC_040269.1	24861145	G	A
NC_040269.1	24879618	T	G
NC_040269.1	24906861	C	T
NC_040269.1	24963083	G	T
NC_040269.1	25006298	A	G
NC_040269.1	25007619	G	A
NC_040269.1	25021102	G	A
NC_040269.1	25022570	A	T
NC_040269.1	25040646	A	G
NC_040269.1	25040952	C	T
NC_040269.1	25092985	T	C
NC_040269.1	25252863	A	G
NC_040269.1	25264009	C	T
NC_040269.1	25279391	T	C
NC_040269.1	25283074	A	G
NC_040269.1	25292952	G	A
NC_040269.1	25298097	G	A
NC_040269.1	25298322	0	A
NC_040269.1	25300526	T	C
NC_040269.1	25321129	C	T
NC_040269.1	25351070	C	T
NC_040269.1	25368209	T	C
NC_040269.1	25416668	C	G
NC_040269.1	25553555	A	C
NC_040269.1	25589869	A	G
NC_040269.1	25895241	0	G
NC_040269.1	25938119	A	G
NC_040269.1	25982706	T	C
NC_040269.1	25983092	T	C
NC_040269.1	26042622	T	C
NC_040269.1	26100187	T	C
NC_040269.1	26163343	G	C
NC_040269.1	26218412	A	G
NC_040269.1	26276890	T	G
NC_040269.1	26340281	T	C
NC_040269.1	26352201	A	G
NC_040269.1	26352642	T	C
NC_040269.1	26387169	T	C
NC_040269.1	26448777	A	G
NC_040269.1	26696767	C	T
NC_040269.1	26739335	A	G
NC_040269.1	26893542	C	T
NC_040269.1	26950235	A	G
NC_040269.1	27008947	T	C
NC_040269.1	27049194	A	G

NC_040269.1	27087288	A		G
NC_040269.1	27432728	C		A
NC_040269.1	27432902	T		C
NC_040269.1	27491915	T		C
NC_040269.1	27551170	A		C
NC_040269.1	27591542	G		A
NC_040269.1	27993584	T		C
NC_040269.1	28058112	T		C
NC_040269.1	28110141	A		G
NC_040269.1	28161201	G		A
NC_040269.1	28180950	A		G
NC_040269.1	28181137	T		G
NC_040269.1	28261043	A		G
NC_040269.1	28268323	A		G
NC_040269.1	28306981	A		G
NC_040269.1	28311163	G		A
NC_040269.1	28316819	A		G
NC_040269.1	28319698	A		G
NC_040269.1	28333954	T		C
NC_040269.1	28344035	A		G
NC_040269.1	28373199	C		G
NC_040269.1	28434157	A		C
NC_040269.1	28501977	G		A
NC_040269.1	28502467	T		C
NC_040269.1	28558715	T		C
NC_040269.1	28617116	A		G
NC_040269.1	28617321	T		C
NC_040269.1	28618127	A		G
NC_040269.1	28690935	G		C
NC_040269.1	28775550	T		C
NC_040269.1	28799192	T		C
NC_040269.1	28814663	A		G
NC_040269.1	28874951	C		G
NC_040269.1	28888225	G		T
NC_040269.1	28942912	A		G
NC_040269.1	28961969	T		C
NC_040269.1	29079079	G		C
NC_040269.1	29104578	T		C
NC_040269.1	29110058	G		A
NC_040269.1	29167272	G		A
NC_040269.1	29331468	A		G
NC_040269.1	29394480	T		A
NC_040269.1	29450534	T		G
NC_040269.1	29501311		0	C
NC_040269.1	29535123	C		G
NC_040269.1	29587270	A		G
NC_040269.1	29598330	C		T

NC_040269.1	29666241	A	G
NC_040269.1	29723244	C	T
NC_040269.1	29776964	C	T
NC_040269.1	29834682	A	G
NC_040269.1	29887829	C	T
NC_040269.1	29942075	A	G
NC_040269.1	29994471	T	G
NC_040269.1	30018110	G	A
NC_040269.1	30036199	A	G
NC_040269.1	30084364	T	C
NC_040269.1	30106263	G	C
NC_040269.1	30129606	C	T
NC_040269.1	30129759	G	T
NC_040269.1	30152729	T	C
NC_040269.1	30157709	C	A
NC_040269.1	30210901	T	C
NC_040269.1	30211174	A	G
NC_040269.1	30211568	T	C
NC_040269.1	30214618	A	G
NC_040269.1	30278751	A	G
NC_040269.1	30280202	T	C
NC_040269.1	30336440	C	T
NC_040269.1	30391663	T	A
NC_040269.1	30448018	G	A
NC_040269.1	30507195	A	G
NC_040269.1	30540035	A	G
NC_040269.1	30595029	T	A
NC_040269.1	30616495	T	C
NC_040269.1	30617305	A	G
NC_040269.1	30751284	A	G
NC_040269.1	30751464	G	C
NC_040269.1	31016684	A	G
NC_040269.1	31018174	T	C
NC_040269.1	31050303	A	G
NC_040269.1	31059594	A	G
NC_040269.1	31065134	T	C
NC_040269.1	31065363	G	C
NC_040269.1	31078615	T	C
NC_040269.1	31080578	C	T
NC_040269.1	31080919	G	A
NC_040269.1	31089712	T	C
NC_040269.1	31090214	A	G
NC_040269.1	31133732	T	C
NC_040269.1	31186474	C	T
NC_040269.1	31221829	T	A
NC_040269.1	31222783	A	G
NC_040269.1	31231878	A	C

NC_040269.1	31265616	C	T
NC_040269.1	31276782	A	G
NC_040269.1	31297505	C	T
NC_040269.1	31298467	C	A
NC_040269.1	31298834	G	A
NC_040269.1	31309360	T	C
NC_040269.1	31316573	T	C
NC_040269.1	31328197	G	T
NC_040269.1	31329340	A	G
NC_040269.1	31388837	T	C
NC_040269.1	31389162	G	A
NC_040269.1	31389421	T	G
NC_040269.1	31526134	T	C
NC_040269.1	31549060	T	C
NC_040269.1	31549769	C	G
NC_040269.1	31550023	G	A
NC_040269.1	31552611	C	T
NC_040269.1	31609511	A	G
NC_040269.1	31644309	C	G
NC_040269.1	31651794	G	A
NC_040269.1	31652009	C	A
NC_040269.1	31693727	A	G
NC_040269.1	31694133	A	G
NC_040269.1	31699197	T	C
NC_040269.1	31699979	T	C
NC_040269.1	31744278	A	G
NC_040269.1	31783200	T	C
NC_040269.1	31809535	T	C
NC_040269.1	31904609	A	C
NC_040269.1	31953093	G	C
NC_040269.1	31956056	T	C
NC_040269.1	31956274	A	G
NC_040269.1	31957591	A	G
NC_040269.1	31958823	A	G
NC_040269.1	31959712	G	C
NC_040269.1	31972696	A	C
NC_040269.1	31995145	T	C
NC_040269.1	32037663	A	G
NC_040269.1	32097964	T	C
NC_040269.1	32130679	T	G
NC_040269.1	32160760	T	C
NC_040269.1	32233629	T	G
NC_040269.1	32236716	T	C
NC_040269.1	32239912	T	C
NC_040269.1	32246330	C	T
NC_040269.1	32252491	T	C
NC_040269.1	32273084	T	C

NC_040269.1	32388754	C	T
NC_040269.1	32408080	G	C
NC_040269.1	32413732	C	T
NC_040269.1	32413896	A	G
NC_040269.1	32414050	C	G
NC_040269.1	32414866	G	A
NC_040269.1	32415223	T	C
NC_040269.1	32457146	T	G
NC_040269.1	32457816	T	C
NC_040269.1	32477729	T	C
NC_040269.1	32480520	A	G
NC_040269.1	32490969	T	C
NC_040269.1	32496900	G	T
NC_040269.1	32540794	T	C
NC_040269.1	32544062	C	T
NC_040269.1	32565427	T	C
NC_040269.1	32588351	G	A
NC_040269.1	32647238	T	C
NC_040269.1	32819224	T	C
NC_040269.1	33046365	T	C
NC_040269.1	33046518	A	G
NC_040269.1	33047630	T	C
NC_040269.1	33105178	A	C
NC_040269.1	33162974	A	G
NC_040269.1	33228454	G	A
NC_040269.1	33291494	C	G
NC_040269.1	33311670	G	A
NC_040269.1	33335997	C	A
NC_040269.1	33394322	T	C
NC_040269.1	33443254	T	C
NC_040269.1	33504887	C	T
NC_040269.1	33568723	G	A
NC_040269.1	33635377	G	A
NC_040269.1	33719757	C	G
NC_040269.1	33971064	C	T
NC_040269.1	34039992	A	T
NC_040269.1	34103825	C	G
NC_040269.1	34184961	T	C
NC_040269.1	34249366	G	A
NC_040269.1	34308760	C	A
NC_040269.1	34370320	A	G
NC_040269.1	34427051	A	C
NC_040269.1	34487795	A	G
NC_040269.1	34545074	G	T
NC_040269.1	34602988	C	T
NC_040269.1	34664777	G	A
NC_040269.1	34741595	G	A

NC_040269.1	34802220	A	C
NC_040269.1	34868149	T	G
NC_040269.1	34949961	C	T
NC_040269.1	35006659	T	C
NC_040269.1	35060706	T	A
NC_040269.1	35121257	T	G
NC_040269.1	35181315	C	T
NC_040269.1	35234808	T	C
NC_040269.1	35299832	G	C
NC_040269.1	35362899	G	A
NC_040269.1	35423858	A	T
NC_040269.1	35475769	A	T
NC_040269.1	35553979	G	C
NC_040269.1	35624058	T	C
NC_040269.1	35676502	A	G
NC_040269.1	35745752	T	C
NC_040269.1	35771196	G	A
NC_040269.1	35890844	G	A
NC_040269.1	35937144	A	T
NC_040269.1	36019054	C	A
NC_040269.1	36079858	G	A
NC_040269.1	36127671	C	T
NC_040269.1	36211073	G	A
NC_040269.1	36291799	T	C
NC_040269.1	36361875	A	G
NC_040269.1	36415880	A	C
NC_040269.1	36495405	C	T
NC_040269.1	36565037	A	T
NC_040269.1	36650281	G	A
NC_040269.1	36710080	A	C
NC_040269.1	36771292	T	G
NC_040269.1	36833748	T	G
NC_040269.1	36895195	0	A
NC_040269.1	36938040	T	C
NC_040269.1	37007730	A	C
NC_040269.1	37059276	G	A
NC_040269.1	37292987	C	T
NC_040269.1	37365683	C	T
NC_040269.1	37423847	G	A
NC_040269.1	37481117	C	T
NC_040269.1	37557718	C	G
NC_040269.1	37619600	C	T
NC_040269.1	37699849	A	G
NC_040269.1	37771999	C	G
NC_040269.1	37908909	G	C
NC_040269.1	37959369	C	T
NC_040269.1	38080433	T	C

NC_040269.1	38143051	T	G
NC_040269.1	38193057	T	C
NC_040269.1	38334078	A	C
NC_040269.1	38393968	A	G
NC_040269.1	38461356	G	A
NC_040269.1	38521244	T	G
NC_040269.1	38583472	G	A
NC_040269.1	38645863	G	A
NC_040269.1	38704900	T	C
NC_040269.1	38781957	C	T
NC_040269.1	38875084	G	T
NC_040269.1	38898324	T	C
NC_040269.1	38959524	C	G
NC_040269.1	38982075	T	C
NC_040269.1	39053757	T	A
NC_040269.1	39110555	A	C
NC_040269.1	39180567	A	G
NC_040269.1	39268772	A	G
NC_040269.1	39305986	C	G
NC_040269.1	40054038	A	G
NC_040269.1	40099637	G	A
NC_040269.1	40125859	A	G
NC_040269.1	40127075	G	A
NC_040269.1	40182989	A	C
NC_040269.1	40228213	G	T
NC_040269.1	40392898	G	A
NC_040269.1	40403908	G	T
NC_040269.1	40407283	C	T
NC_040269.1	40408157	G	A
NC_040269.1	40408375	C	T
NC_040269.1	40408547	G	A
NC_040269.1	40466208	G	A
NC_040269.1	40511685	A	G
NC_040269.1	40559605	T	C
NC_040269.1	40563842	G	A
NC_040269.1	40564085	T	C
NC_040269.1	40583955	T	C
NC_040269.1	40587536	T	C
NC_040269.1	40595025	C	T
NC_040269.1	40632369	C	T
NC_040269.1	40633162	G	A
NC_040269.1	40693190	G	T
NC_040269.1	40695045	C	T
NC_040269.1	40704550	C	A
NC_040269.1	40708307	A	G
NC_040269.1	40760652	G	T
NC_040269.1	40788130	C	T

NC_040269.1	40842162	A	G
NC_040269.1	40911252	C	T
NC_040269.1	40966028	G	A
NC_040269.1	41009193	A	G
NC_040269.1	41068202	G	A
NC_040269.1	41107140	C	T
NC_040269.1	41137740	T	C
NC_040269.1	41351170	C	G
NC_040269.1	41363383	T	C
NC_040269.1	41364930	T	G
NC_040269.1	41418959	A	G
NC_040269.1	41665814	C	T
NC_040269.1	41725158	C	T
NC_040269.1	41778510	C	A
NC_040269.1	41836056	A	G
NC_040269.1	41910011	A	T
NC_040269.1	41967240	A	G
NC_040269.1	42018143	T	C
NC_040269.1	42054810	T	A
NC_040269.1	42056331	A	C
NC_040269.1	42288399	G	A
NC_040269.1	42349320	T	A
NC_040269.1	42486977	T	G
NC_040269.1	42542726	A	C
NC_040269.1	42599669	C	A
NC_040269.1	42638103	C	T
NC_040269.1	42697477	G	A
NC_040269.1	42751517	G	C
NC_040269.1	42806042	A	G
NC_040269.1	42861691	A	G
NC_040269.1	42920253	A	G
NC_040269.1	42975186	C	A
NC_040269.1	43034618	T	C
NC_040269.1	43035218	T	C
NC_040269.1	43141196	A	G
NC_040269.1	43162821	A	G
NC_040269.1	43647119	A	G
NC_040269.1	43747171	T	C
NC_040269.1	43779464	A	G
NC_040269.1	43866779	C	G
NC_040269.1	43868866	A	G
NC_040269.1	43887054	C	T
NC_040269.1	43891586	C	T
NC_040269.1	43948305	A	G
NC_040269.1	44018171	T	C
NC_040269.1	44074938	C	T
NC_040269.1	44108095	A	G

NC_040269.1	44108974	T	C
NC_040269.1	44110532	G	A
NC_040269.1	44176828	C	T
NC_040269.1	44231707	T	C
NC_040269.1	44304499	C	T
NC_040269.1	44383802	G	A
NC_040269.1	44670846	T	C
NC_040269.1	44792844	C	T
NC_040269.1	44802380	A	T
NC_040269.1	44861099	T	A
NC_040269.1	44938638	G	A
NC_040269.1	45373606	C	T
NC_040269.1	45742547	A	G
NC_040269.1	45761172	T	C
NC_040269.1	45761653	T	C
NC_040269.1	45763469	G	A
NC_040269.1	45822507	A	T
NC_040269.1	45869569	G	T
NC_040269.1	46312257	C	G
NC_040269.1	46440005	A	G
NC_040269.1	46500512	A	T
NC_040269.1	46565472	T	C
NC_040269.1	46619496	G	A
NC_040269.1	46679895	C	T
NC_040269.1	46719868	C	T
NC_040269.1	46766855	G	A
NC_040269.1	46813637	G	A
NC_040269.1	46867762	A	G
NC_040269.1	46923438	C	T
NC_040269.1	46958637	A	G
NC_040269.1	48295894	G	A
NC_040269.1	48355043	A	G
NC_040269.1	48361834	G	A
NC_040269.1	48403375	C	A
NC_040269.1	48475448	G	C
NC_040269.1	48484655	T	C
NC_040269.1	48503049	C	T
NC_040269.1	51046400	A	G
NC_040269.1	51047834	G	T
NC_040269.1	54287365	T	C
NC_040269.1	54331777	T	C
NC_040269.1	54332260	C	T
NC_040269.1	54763863	A	C
NC_040269.1	54985202	C	G
NC_040269.1	55010617	A	G
NC_040269.1	55015133	T	A
NC_040269.1	55043828	A	G

NC_040269.1	55045416	G	C
NC_040269.1	55119176	A	G
NC_040269.1	55170501	T	C
NC_040269.1	55199148	A	G
NC_040269.1	55269966	T	C
NC_040269.1	55274069	T	C
NC_040269.1	55324568	A	G
NC_040269.1	55357916	A	G
NC_040269.1	55364728	T	C
NC_040269.1	55428272	C	T
NC_040269.1	55430878	C	T
NC_040269.1	55432649	A	G
NC_040269.1	55438335	A	G
NC_040269.1	55441566	C	G
NC_040269.1	55442109	T	C
NC_040269.1	55481824	A	G
NC_040269.1	55485710	G	T
NC_040269.1	55513044	T	A
NC_040269.1	55555189	A	G
NC_040269.1	55595758	T	C
NC_040269.1	55598244	G	A
NC_040269.1	55600449	G	C
NC_040269.1	55601855	A	G
NC_040269.1	55603910	T	C
NC_040269.1	55618524	A	G
NC_040269.1	55639742	A	G
NC_040269.1	55645583	0	C
NC_040269.1	55655806	G	A
NC_040269.1	55717566	G	A
NC_040269.1	55721909	A	G
NC_040269.1	55739894	G	A
NC_040269.1	55743743	A	G
NC_040269.1	55763346	T	G
NC_040269.1	55805172	A	G
NC_040269.1	55831509	A	G
NC_040269.1	55859451	T	C
NC_040269.1	56317690	A	G
NC_040269.1	56372619	A	G
NC_040269.1	56426255	A	G
NC_040269.1	56479634	A	G
NC_040269.1	56524019	C	T
NC_040269.1	56540011	C	T
NC_040269.1	56557983	G	T
NC_040269.1	56603648	G	A
NC_040269.1	56668394	G	A
NC_040269.1	56734384	T	C
NC_040269.1	56828146	A	G

NC_040269.1	56873561	A	G
NC_040269.1	56873846	T	C
NC_040269.1	56924599	C	T
NC_040269.1	56961058	T	C
NC_040269.1	56977418	G	C
NC_040269.1	56977607	T	A
NC_040269.1	56978468	G	A
NC_040269.1	56979356	T	C
NC_040269.1	57009240	C	A
NC_040269.1	57045526	A	G
NC_040269.1	57055614	G	A
NC_040269.1	57056989	C	A
NC_040269.1	57058334	G	A
NC_040269.1	57075218	C	T
NC_040269.1	57131833	T	C
NC_040269.1	57359534	C	T
NC_040269.1	57421266	G	C
NC_040269.1	57487309	T	C
NC_040269.1	57531254	A	G
NC_040269.1	57591040	A	G
NC_040269.1	57643252	C	T
NC_040269.1	57684887	T	C
NC_040269.1	57685380	C	G
NC_040269.1	57685557	A	G
NC_040269.1	57688866	T	C
NC_040269.1	57746411	T	C
NC_040269.1	57830755	T	C
NC_040269.1	57852494	G	A
NC_040269.1	57923420	C	T
NC_040269.1	57932170	T	C
NC_040269.1	57933418	A	G
NC_040269.1	57933694	A	G
NC_040269.1	57942138	C	T
NC_040269.1	58003938	A	G
NC_040269.1	58005163	A	G
NC_040269.1	58060585	A	G
NC_040269.1	58096475	A	G
NC_040269.1	58156047	A	C
NC_040269.1	58157148	C	T
NC_040269.1	58165100	T	C
NC_040269.1	58165519	C	A
NC_040269.1	58165691	C	T
NC_040269.1	58189883	T	C
NC_040269.1	58190213	G	C
NC_040269.1	58190469	C	G
NC_040269.1	58190779	G	A
NC_040269.1	58203707	T	C

NC_040269.1	58204379	A	T
NC_040269.1	58206204	T	C
NC_040269.1	58254031	C	T
NC_040269.1	58255282	G	A
NC_040269.1	58434164	A	C
NC_040269.1	58484896	G	C
NC_040269.1	58520971	T	C
NC_040269.1	58523224	G	A
NC_040269.1	58545331	T	C
NC_040269.1	58564960	T	G
NC_040269.1	58565338	T	C
NC_040269.1	58596017	T	C
NC_040269.1	58644283	A	G
NC_040269.1	58705554	G	A
NC_040269.1	58896022	T	C
NC_040269.1	58896583	A	G
NC_040269.1	58909442	C	G
NC_040269.1	58969679	A	G
NC_040269.1	59025356	T	C
NC_040269.1	59046824	G	A
NC_040269.1	59129697	A	G
NC_040269.1	59179417	A	G
NC_040269.1	59230515	A	C
NC_040269.1	59280217	A	G
NC_040269.1	59314992	T	C
NC_040269.1	59322870	A	G
NC_040269.1	59333117	G	A
NC_040269.1	59333389	C	T
NC_040269.1	59333669	T	A
NC_040269.1	59334424	T	G
NC_040269.1	59334942	T	C
NC_040269.1	59335860	G	A
NC_040269.1	59367409	T	G
NC_040269.1	59425231	T	A
NC_040269.1	59427602	A	C
NC_040269.1	59428059	A	G
NC_040269.1	59501667	G	A
NC_040269.1	59552335	T	G
NC_040269.1	59561286	A	G
NC_040269.1	59561498	G	A
NC_040269.1	59632152	A	G
NC_040269.1	59763475	G	C
NC_040269.1	59940150	A	G
NC_040269.1	60260188	C	T
NC_040269.1	60263559	C	T
NC_040269.1	60264445	A	G
NC_040269.1	60264644	A	G

NC_040269.1	60271097	T	C
NC_040269.1	60274443	C	T
NC_040269.1	60277082	A	G
NC_040269.1	60277276	A	G
NC_040269.1	60288374	T	C
NC_040269.1	60297317	A	G
NC_040269.1	60333149	C	G
NC_040269.1	60382073	A	G
NC_040269.1	60453142	T	C
NC_040269.1	60483904	G	T
NC_040269.1	60577877	C	T
NC_040269.1	60605287	C	T
NC_040269.1	60676241	G	C
NC_040269.1	60819318	C	A
NC_040269.1	60855614	A	G
NC_040269.1	61226718	G	A
NC_040269.1	61243809	T	C
NC_040269.1	61632456	A	G
NC_040269.1	61680509	C	T
NC_040269.1	61737126	T	A
NC_040269.1	61773683	C	T
NC_040269.1	61837852	T	C
NC_040269.1	61872257	C	T
NC_040269.1	61910491	G	A
NC_040269.1	62142015	A	G
NC_040269.1	62188503	T	C
NC_040269.1	62208737	A	T
NC_040269.1	64707435	T	G
NC_040269.1	64709276	A	G
NC_040269.1	64709705	T	C
NC_040269.1	64767772	G	C
NC_040269.1	64803153	A	G
NC_040269.1	64932425	T	C
NC_040269.1	64933131	T	C
NC_040269.1	64992764	T	C
NC_040269.1	65009420	C	A
NC_040269.1	65032440	T	C
NC_040269.1	65038342	C	G
NC_040269.1	65038503	T	C
NC_040269.1	65042402	A	G
NC_040269.1	65098722	T	C
NC_040269.1	65155255	T	G
NC_040269.1	65173623	G	C
NC_040269.1	65174565	T	A
NC_040269.1	65180335	G	A
NC_040269.1	65180542	C	T
NC_040269.1	65181206	T	C

NC_040269.1	65199307	T	C
NC_040269.1	65254334	T	C
NC_040269.1	65300497	C	T
NC_040269.1	65346096	C	T
NC_040269.1	65389947	C	T
NC_040269.1	65392490	A	G
NC_040269.1	65395136	C	T
NC_040269.1	65397371	T	C
NC_040269.1	65452957	C	A
NC_040269.1	65509187	G	C
NC_040269.1	65561169	A	G
NC_040269.1	65612436	G	T
NC_040269.1	65672901	C	T
NC_040269.1	65694911	T	C
NC_040269.1	65696373	T	C
NC_040269.1	65696615	T	C
NC_040269.1	65697736	C	G
NC_040269.1	65698602	A	G
NC_040269.1	65705772	T	C
NC_040269.1	65706489	A	G
NC_040269.1	65706639	C	T
NC_040269.1	65706807	C	A
NC_040269.1	65709692	T	C
NC_040269.1	65710885	G	A
NC_040269.1	65730216	T	C
NC_040269.1	65730426	A	G
NC_040269.1	65730807	T	C
NC_040269.1	65741035	C	A
NC_040269.1	65741214	G	C
NC_040269.1	65748597	A	G
NC_040269.1	65806163	T	C
NC_040269.1	65860126	A	G
NC_040269.1	65919831	T	C
NC_040269.1	65920551	T	C
NC_040269.1	65925271	C	G
NC_040269.1	65925853	C	G
NC_040269.1	65965165	G	C
NC_040269.1	66123977	A	C
NC_040269.1	66130948	A	G
NC_040269.1	66303154	A	C
NC_040269.1	66305889	T	C
NC_040269.1	66306124	A	G
NC_040269.1	66306456	T	C
NC_040269.1	66325601	T	C
NC_040269.1	66329297	C	G
NC_040269.1	66329940	A	T
NC_040269.1	66330529	G	A

NC_040269.1	66330740	A	G
NC_040269.1	67003950	T	C
NC_040269.1	67194466	A	G
NC_040269.1	67232976	A	G
NC_040269.1	67233274	G	T
NC_040269.1	67233476	C	T
NC_040269.1	67294476	A	G
NC_040269.1	67326210	G	A
NC_040269.1	67327271	A	G
NC_040269.1	67328877	G	A
NC_040269.1	67331113	T	C
NC_040269.1	67376332	T	C
NC_040269.1	67382986	T	C
NC_040269.1	67394330	A	G
NC_040269.1	67395790	T	C
NC_040269.1	67415960	C	T
NC_040269.1	67421504	A	G
NC_040269.1	67422830	T	C
NC_040269.1	67424519	T	G
NC_040269.1	67458795	A	G
NC_040269.1	67515925	A	G
NC_040269.1	67558029	T	C
NC_040269.1	67560089	C	G
NC_040269.1	67591256	G	A
NC_040269.1	67640571	T	C
NC_040269.1	67710184	A	C
NC_040269.1	67721538	C	T
NC_040269.1	67768516	T	C
NC_040269.1	67771396	C	A
NC_040269.1	67843020	T	C
NC_040269.1	67901859	A	G
NC_040269.1	67958482	G	A
NC_040269.1	67959607	T	C
NC_040269.1	67959882	T	C
NC_040269.1	67960886	C	T
NC_040269.1	67961085	T	C
NC_040269.1	67961396	A	G
NC_040269.1	67961906	A	G
NC_040269.1	68018118	G	A
NC_040269.1	68068122	T	C
NC_040269.1	68090865	A	G
NC_040269.1	68116703	A	G
NC_040269.1	68148789	A	G
NC_040269.1	68161829	T	C
NC_040269.1	68219050	G	A
NC_040269.1	68275030	T	C
NC_040269.1	68289423	G	A

NC_040269.1	68342763	A	G
NC_040269.1	68501561	C	T
NC_040269.1	68696662	A	G
NC_040269.1	68704350	C	A
NC_040269.1	68704900	C	G
NC_040269.1	68758307	G	A
NC_040269.1	68809759	C	T
NC_040269.1	68832479	A	G
NC_040269.1	68841636	C	T
NC_040269.1	68863048	A	C
NC_040269.1	68871386	T	C
NC_040269.1	68876245	G	T
NC_040269.1	68894553	T	C
NC_040269.1	68894726	G	A
NC_040269.1	68895731	G	A
NC_040269.1	68897245	A	G
NC_040269.1	68897840	G	A
NC_040269.1	68899984	0	G
NC_040269.1	68968157	T	G
NC_040269.1	69000960	G	C
NC_040269.1	69016190	A	G
NC_040269.1	69023654	A	G
NC_040269.1	69034900	T	C
NC_040269.1	69044282	T	C
NC_040269.1	69096758	A	C
NC_040269.1	69181128	T	C
NC_040269.1	69220856	G	A
NC_040269.1	69246482	T	C
NC_040269.1	69247079	A	G
NC_040269.1	69298664	G	A
NC_040269.1	69359909	T	C
NC_040269.1	69390617	A	G
NC_040269.1	69390840	A	G
NC_040269.1	69770994	A	G
NC_040269.1	69781968	T	C
NC_040269.1	69782122	T	C
NC_040269.1	69866627	A	G
NC_040269.1	69889154	T	C
NC_040269.1	69890457	0	C
NC_040269.1	69927940	A	G
NC_040269.1	69960265	T	A
NC_040269.1	70014194	T	C
NC_040269.1	70030281	T	C
NC_040269.1	70036301	A	G
NC_040269.1	70053432	A	G
NC_040269.1	70053854	A	G
NC_040269.1	70054080	A	G

NC_040269.1	70056809	C	T
NC_040269.1	70058064	A	G
NC_040269.1	70107923	T	C
NC_040269.1	70145349	G	A
NC_040269.1	70215615	G	A
NC_040269.1	70221672	G	C
NC_040269.1	70246427	C	T
NC_040269.1	70263039	G	C
NC_040269.1	70263216	A	G
NC_040269.1	70263603	A	G
NC_040269.1	70264294	T	C
NC_040269.1	70264612	C	T
NC_040269.1	70267450	G	C
NC_040269.1	70319188	G	A
NC_040269.1	70333527	T	C
NC_040269.1	70391870	T	C
NC_040269.1	70440659	T	C
NC_040269.1	70457426	T	C
NC_040269.1	70463925	T	C
NC_040269.1	70475410	C	T
NC_040269.1	70475691	G	A
NC_040269.1	70539313	T	C
NC_040269.1	70539852	A	G
NC_040269.1	70540462	T	C
NC_040269.1	70625036	A	G
NC_040269.1	70675120	G	A
NC_040269.1	70676126	T	C
NC_040269.1	70676351	T	C
NC_040270.1	158966	T	C
NC_040270.1	161378	C	G
NC_040270.1	316681	T	A
NC_040270.1	319773	A	G
NC_040270.1	320719	G	C
NC_040270.1	321031	T	C
NC_040270.1	321697	C	T
NC_040270.1	322387	A	G
NC_040270.1	325018	G	C
NC_040270.1	335320	A	G
NC_040270.1	335499	C	T
NC_040270.1	335687	T	C
NC_040270.1	798908	G	C
NC_040270.1	799963	T	G
NC_040270.1	800198	T	C
NC_040270.1	801764	G	C
NC_040270.1	801950	C	T
NC_040270.1	802252	T	C
NC_040270.1	802552	T	C

NC_040270.1	802965	G	T
NC_040270.1	803565	G	C
NC_040270.1	805026	T	C
NC_040270.1	808280	A	G
NC_040270.1	1973844	A	G
NC_040270.1	1975175	C	T
NC_040270.1	2028124	T	C
NC_040270.1	2320421	G	C
NC_040270.1	2321153	G	C
NC_040270.1	2324828	A	C
NC_040270.1	2325917	G	C
NC_040270.1	4070472	G	A
NC_040270.1	4874128	G	A
NC_040270.1	5520762	A	C
NC_040270.1	5538757	G	A
NC_040270.1	5540332	G	A
NC_040270.1	6580943	A	G
NC_040270.1	6591812	T	C
NC_040270.1	6611488	C	T
NC_040270.1	6681310	G	A
NC_040270.1	7088312	C	A
NC_040270.1	7239574	T	C
NC_040270.1	7358473	G	A
NC_040270.1	7359187	A	G
NC_040270.1	7401919	T	C
NC_040270.1	7860364	A	G
NC_040270.1	7861365	A	G
NC_040270.1	7861641	T	C
NC_040270.1	7912290	A	G
NC_040270.1	8098557	C	T
NC_040270.1	8099465	A	G
NC_040270.1	8099812	C	T
NC_040270.1	8100141	C	G
NC_040270.1	8100384	C	T
NC_040270.1	8100563	T	C
NC_040270.1	8270329	T	C
NC_040270.1	8346805	A	C
NC_040270.1	8347934	C	G
NC_040270.1	8348250	T	C
NC_040270.1	8472282	A	G
NC_040270.1	8473065	A	G
NC_040270.1	8658192	0	C
NC_040270.1	8659723	C	T
NC_040270.1	8684056	A	G
NC_040270.1	8685091	A	G
NC_040270.1	8685301	G	A
NC_040270.1	8687931	C	G

NC_040270.1	8688352	T	C
NC_040270.1	10198617	C	G
NC_040270.1	10199045	A	G
NC_040270.1	10200454	C	T
NC_040270.1	10244930	A	G
NC_040270.1	10249782	C	T
NC_040270.1	10250977	C	T
NC_040270.1	10284111	T	C
NC_040270.1	10292743	A	G
NC_040270.1	10970283	T	C
NC_040270.1	11031494	A	G
NC_040270.1	11070217	A	G
NC_040270.1	11079635	A	G
NC_040270.1	11166850	G	A
NC_040270.1	11168854	G	C
NC_040270.1	11265138	A	G
NC_040270.1	11265312	A	G
NC_040270.1	11265711	T	C
NC_040270.1	11296535	C	T
NC_040270.1	11296687	A	G
NC_040270.1	11297771	T	C
NC_040270.1	11298936	A	G
NC_040270.1	11352051	T	C
NC_040270.1	11439021	G	A
NC_040270.1	11461968	A	G
NC_040270.1	11462502	G	A
NC_040270.1	11463343	T	G
NC_040270.1	11464121	C	T
NC_040270.1	11472343	T	C
NC_040270.1	11508915	G	A
NC_040270.1	11518533	A	C
NC_040270.1	12016613	C	T
NC_040270.1	12016811	G	A
NC_040270.1	12017903	T	C
NC_040270.1	12046945	C	G
NC_040270.1	12073915	A	G
NC_040270.1	12094904	C	A
NC_040270.1	12105741	T	C
NC_040270.1	12105942	G	A
NC_040270.1	12160259	G	A
NC_040270.1	12160975	T	C
NC_040270.1	12193660	T	A
NC_040270.1	12193846	A	G
NC_040270.1	12219184	A	G
NC_040270.1	12329479	A	G
NC_040270.1	12329770	G	A
NC_040270.1	12344687	A	G

NC_040270.1	12361971	T	C
NC_040270.1	12366884	G	A
NC_040270.1	12455879	A	G
NC_040270.1	12497726	A	G
NC_040270.1	12570229	T	C
NC_040270.1	12631704	T	C
NC_040270.1	12827665	A	G
NC_040270.1	12828936	T	C
NC_040270.1	13011918	T	G
NC_040270.1	13013054	G	T
NC_040270.1	13013428	T	G
NC_040270.1	13014058	A	G
NC_040270.1	13014211	G	A
NC_040270.1	13014928	T	C
NC_040270.1	13015154	C	G
NC_040270.1	13024379	G	C
NC_040270.1	13033214	A	G
NC_040270.1	13051857	G	T
NC_040270.1	13055720	A	G
NC_040270.1	13057264	A	G
NC_040270.1	13061002	C	T
NC_040270.1	13062113	T	C
NC_040270.1	13062408	C	T
NC_040270.1	13062629	G	A
NC_040270.1	13066467	T	G
NC_040270.1	13066891	A	C
NC_040270.1	13069375	G	T
NC_040270.1	13070420	A	C
NC_040270.1	13089639	A	G
NC_040270.1	13089795	T	C
NC_040270.1	13092134	G	A
NC_040270.1	13093862	T	C
NC_040270.1	13095117	A	G
NC_040270.1	13095345	A	G
NC_040270.1	13095498	A	G
NC_040270.1	13153810	G	A
NC_040270.1	13217558	A	G
NC_040270.1	13218284	G	A
NC_040270.1	13218550	A	G
NC_040270.1	13224082	C	T
NC_040270.1	13247785	G	C
NC_040270.1	13269978	T	C
NC_040270.1	13351532	C	T
NC_040270.1	13743415	C	G
NC_040270.1	14441585	T	C
NC_040270.1	14452921	C	A
NC_040270.1	14454283	C	T

NC_040270.1	14454516	C	A
NC_040270.1	15186036	C	T
NC_040270.1	15270312	G	A
NC_040270.1	15383653	G	A
NC_040270.1	15423191	G	A
NC_040270.1	15423450	G	A
NC_040270.1	15423616	A	C
NC_040270.1	15464993	A	G
NC_040270.1	15483658	C	T
NC_040270.1	15495243	G	A
NC_040270.1	15495449	C	T
NC_040270.1	15626665	T	C
NC_040270.1	15627399	T	C
NC_040270.1	15671957	A	G
NC_040270.1	15683969	T	C
NC_040270.1	15685912	A	G
NC_040270.1	15686120	C	G
NC_040270.1	15692016	T	C
NC_040270.1	15692551	G	A
NC_040270.1	15693093	C	T
NC_040270.1	15701807	A	G
NC_040270.1	15703629	A	G
NC_040270.1	15721027	T	C
NC_040270.1	15722297	A	G
NC_040270.1	15729325	A	G
NC_040270.1	15735309	T	C
NC_040270.1	15748613	T	C
NC_040270.1	15773558	T	C
NC_040270.1	15805168	C	T
NC_040270.1	15868504	T	C
NC_040270.1	15882670	C	G
NC_040270.1	15883309	T	C
NC_040270.1	15884533	A	G
NC_040270.1	15965211	C	T
NC_040270.1	16018362	T	C
NC_040270.1	16018914	T	C
NC_040270.1	16251517	A	G
NC_040270.1	16302608	C	G
NC_040270.1	16315241	T	C
NC_040270.1	16315392	C	T
NC_040270.1	16315635	G	A
NC_040270.1	16315794	A	G
NC_040270.1	16316164	A	G
NC_040270.1	17196088	T	A
NC_040270.1	17196312	G	A
NC_040270.1	17196493	C	G
NC_040270.1	17196852	A	G

NC_040270.1	17197710	C	T
NC_040270.1	17295806	G	A
NC_040270.1	17296262	G	A
NC_040270.1	17319583	G	A
NC_040270.1	17365540	T	C
NC_040270.1	17366042	A	C
NC_040270.1	17377140	C	T
NC_040270.1	17381564	A	G
NC_040270.1	17388725	G	A
NC_040270.1	17388879	G	A
NC_040270.1	17391328	G	C
NC_040270.1	17392822	A	G
NC_040270.1	17408940	A	G
NC_040270.1	17409387	C	T
NC_040270.1	17409693	T	C
NC_040270.1	17427086	A	G
NC_040270.1	17436022	C	A
NC_040270.1	17436182	T	C
NC_040270.1	17436337	T	C
NC_040270.1	17437662	C	T
NC_040270.1	17441284	G	A
NC_040270.1	17449568	T	A
NC_040270.1	17450234	T	C
NC_040270.1	17450750	G	A
NC_040270.1	17451642	T	C
NC_040270.1	17453806	T	C
NC_040270.1	17454024	G	A
NC_040270.1	17470263	A	C
NC_040270.1	17483350	A	G
NC_040270.1	17483647	A	G
NC_040270.1	17494061	T	C
NC_040270.1	17497416	C	T
NC_040270.1	17529773	A	C
NC_040270.1	17530707	C	T
NC_040270.1	17548964	A	G
NC_040270.1	17560317	A	G
NC_040270.1	17650023	G	A
NC_040270.1	17653272	C	T
NC_040270.1	17655743	T	C
NC_040270.1	17665655	A	G
NC_040270.1	17673877	T	C
NC_040270.1	17714230	A	C
NC_040270.1	17714400	G	A
NC_040270.1	17715419	C	G
NC_040270.1	17737410	C	T
NC_040270.1	17757248	T	C
NC_040270.1	17762298	T	C

NC_040270.1	17773430	T	C
NC_040270.1	17814743	A	G
NC_040270.1	17814918	C	G
NC_040270.1	17816318	G	C
NC_040270.1	17816573	A	G
NC_040270.1	17817628	T	C
NC_040270.1	17819310	T	C
NC_040270.1	17819594	A	G
NC_040270.1	17822101	G	A
NC_040270.1	17823548	G	C
NC_040270.1	17827924	A	G
NC_040270.1	17828104	T	C
NC_040270.1	17830425	T	C
NC_040270.1	17836896	G	A
NC_040270.1	17862321	A	G
NC_040270.1	17868808	C	G
NC_040270.1	17884674	T	C
NC_040270.1	17887147	A	G
NC_040270.1	17888039	T	C
NC_040270.1	17888222	C	T
NC_040270.1	17894655	G	A
NC_040270.1	17909733	T	A
NC_040270.1	17920124	T	G
NC_040270.1	17925980	T	C
NC_040270.1	17932905	A	G
NC_040270.1	17954752	A	G
NC_040270.1	17955898	A	G
NC_040270.1	17960498	A	T
NC_040270.1	17962773	A	G
NC_040270.1	17963014	A	C
NC_040270.1	17963165	A	G
NC_040270.1	17971075	C	T
NC_040270.1	17980254	C	T
NC_040270.1	17988753	A	G
NC_040270.1	17996352	G	C
NC_040270.1	18015501	A	G
NC_040270.1	18021164	A	G
NC_040270.1	18079443	T	G
NC_040270.1	18108884	T	C
NC_040270.1	18110008	A	G
NC_040270.1	18110479	T	G
NC_040270.1	18110925	C	G
NC_040270.1	18111491	A	G
NC_040270.1	18111921	T	C
NC_040270.1	18126452	C	G
NC_040270.1	18150197	G	T
NC_040270.1	18152659	A	G

NC_040270.1	18167725	G	C
NC_040270.1	18223135	G	T
NC_040270.1	18223624	T	C
NC_040270.1	18243665	G	C
NC_040270.1	18330480	G	A
NC_040270.1	18505922	A	G
NC_040270.1	18572455	G	A
NC_040270.1	18572727	T	A
NC_040270.1	18573635	T	G
NC_040270.1	18574452	G	A
NC_040270.1	18574609	C	T
NC_040270.1	18575162	A	G
NC_040270.1	18575523	A	G
NC_040270.1	18653977	G	A
NC_040270.1	18751007	C	G
NC_040270.1	18951019	C	G
NC_040270.1	19861323	C	A
NC_040270.1	19902941	A	G
NC_040270.1	20119143	T	C
NC_040270.1	20147219	A	G
NC_040270.1	20264396	T	C
NC_040270.1	22679867	T	A
NC_040270.1	22682648	A	G
NC_040270.1	22697017	A	G
NC_040270.1	22725107	T	C
NC_040270.1	23015390	T	C
NC_040270.1	23187329	A	G
NC_040270.1	23188053	A	G
NC_040270.1	23222201	A	G
NC_040270.1	23492177	A	G
NC_040270.1	23514370	G	A
NC_040270.1	23948763	T	C
NC_040270.1	23949648	T	C
NC_040270.1	23950472	T	C
NC_040270.1	23951064	G	C
NC_040270.1	23951318	C	T
NC_040270.1	23991332	A	C
NC_040270.1	24618855	G	A
NC_040270.1	24619557	T	G
NC_040270.1	24619888	T	C
NC_040270.1	24620077	G	A
NC_040270.1	24620510	T	C
NC_040270.1	24621335	G	C
NC_040270.1	24621596	G	A
NC_040270.1	24698520	T	A
NC_040270.1	24718866	A	G
NC_040270.1	24741830	A	G

NC_040270.1	24751322	T	C
NC_040270.1	25010222	G	A
NC_040270.1	25064961	C	T
NC_040270.1	25123969	C	T
NC_040270.1	25193774	A	C
NC_040270.1	25259188	T	C
NC_040270.1	25323823	C	A
NC_040270.1	25384657	G	A
NC_040270.1	25443982	T	A
NC_040270.1	25500945	A	G
NC_040270.1	25558980	T	C
NC_040270.1	25612293	T	A
NC_040270.1	26025426	C	T
NC_040270.1	26083992	A	G
NC_040270.1	26143732	A	C
NC_040270.1	26200242	A	G
NC_040270.1	27229517	G	T
NC_040270.1	27230318	G	A
NC_040270.1	27230546	A	G
NC_040270.1	27231354	C	T
NC_040270.1	28639304	A	G
NC_040270.1	28715000	A	G
NC_040270.1	29430963	A	G
NC_040270.1	29690953	A	C
NC_040270.1	29698061	A	G
NC_040270.1	29699129	T	C
NC_040270.1	29972854	T	C
NC_040270.1	29973570	A	G
NC_040270.1	30047340	G	A
NC_040270.1	30052337	T	C
NC_040270.1	30158650	T	C
NC_040270.1	30248962	C	G
NC_040270.1	30581260	C	T
NC_040270.1	30584228	A	G
NC_040270.1	30584660	G	A
NC_040270.1	31207733	A	T
NC_040270.1	31207915	C	T
NC_040270.1	31208133	T	A
NC_040270.1	31208935	C	G
NC_040270.1	31234906	T	A
NC_040270.1	31243804	A	T
NC_040270.1	31279228	A	G
NC_040270.1	31284208	A	G
NC_040270.1	31284591	T	A
NC_040270.1	31388265	A	T
NC_040270.1	31390767	C	T
NC_040270.1	31392295	G	A

NC_040270.1	31668115	T	C
NC_040270.1	31715808	G	A
NC_040270.1	32005508	A	G
NC_040270.1	32006479	T	A
NC_040270.1	32008609	A	G
NC_040270.1	33092256	C	T
NC_040270.1	33093312	A	G
NC_040270.1	33093623	A	G
NC_040270.1	33382132	A	G
NC_040270.1	33923373	A	C
NC_040270.1	33930047	T	C
NC_040270.1	33938794	T	C
NC_040270.1	33939926	A	G
NC_040270.1	33995796	T	C
NC_040270.1	34026137	G	A
NC_040270.1	34049821	T	C
NC_040270.1	34051902	C	T
NC_040270.1	34055140	G	A
NC_040270.1	34055747	A	G
NC_040270.1	34056042	T	C
NC_040270.1	34119795	T	C
NC_040270.1	34131356	G	T
NC_040270.1	34131906	C	T
NC_040270.1	34662051	T	G
NC_040270.1	34662830	C	T
NC_040270.1	35716033	A	G
NC_040270.1	35717674	A	C
NC_040270.1	36188848	C	T
NC_040270.1	36190578	C	G
NC_040270.1	36190796	T	A
NC_040270.1	36190970	C	A
NC_040270.1	36194577	A	G
NC_040270.1	36195087	C	T
NC_040270.1	36195321	T	C
NC_040270.1	36803639	G	A
NC_040270.1	36809910	C	G
NC_040270.1	36810108	G	A
NC_040270.1	37037811	C	A
NC_040270.1	37203238	A	G
NC_040270.1	37820528	T	C
NC_040270.1	37836086	A	G
NC_040270.1	37853852	T	C
NC_040270.1	37854589	A	G
NC_040270.1	38586169	A	G
NC_040270.1	38642396	T	C
NC_040270.1	38712064	G	A
NC_040270.1	38729339	C	G

NC_040270.1	38747270	A	G
NC_040270.1	38747772	T	A
NC_040270.1	39119396	C	T
NC_040270.1	39160739	A	G
NC_040270.1	39160931	T	C
NC_040270.1	39161942	C	A
NC_040270.1	39162098	C	A
NC_040270.1	39163502	A	C
NC_040270.1	39164562	A	G
NC_040270.1	39166186	G	A
NC_040270.1	39287508	A	G
NC_040270.1	39289222	A	G
NC_040270.1	39289543	C	T
NC_040270.1	39290004	T	C
NC_040270.1	39291613	A	G
NC_040270.1	39293411	A	G
NC_040270.1	39294042	A	G
NC_040270.1	39430732	T	C
NC_040270.1	39430955	T	C
NC_040270.1	39613014	A	T
NC_040270.1	40240937	C	G
NC_040270.1	40625407	T	C
NC_040270.1	40651717	T	C
NC_040270.1	40743234	T	C
NC_040270.1	40744092	T	G
NC_040270.1	40745588	T	C
NC_040270.1	40794379	T	C
NC_040270.1	40808738	A	G
NC_040270.1	40885194	G	A
NC_040270.1	40886832	T	C
NC_040270.1	40887069	A	G
NC_040270.1	40968759	T	C
NC_040270.1	41626663	A	G
NC_040270.1	41904746	G	T
NC_040270.1	41957750	A	G
NC_040270.1	42019975	T	G
NC_040270.1	42089204	C	A
NC_040270.1	42143373	G	A
NC_040270.1	42207645	T	G
NC_040270.1	42271542	C	A
NC_040270.1	42330381	G	T
NC_040270.1	42395598	G	A
NC_040270.1	42455755	A	C
NC_040270.1	42529021	G	C
NC_040270.1	42576191	C	A
NC_040270.1	42682834	A	G
NC_040270.1	42749408	A	G

NC_040270.1	42804841	G	A
NC_040270.1	42878545	G	A
NC_040270.1	42935513	T	G
NC_040270.1	43002063	A	G
NC_040270.1	43062408	G	A
NC_040270.1	43128046	G	T
NC_040270.1	43201890	C	T
NC_040270.1	43259802	T	C
NC_040270.1	43320577	C	T
NC_040270.1	43397283	A	G
NC_040270.1	43460848	T	C
NC_040270.1	43539022	T	C
NC_040270.1	44347364	C	T
NC_040270.1	44410517	G	A
NC_040270.1	44494493	A	G
NC_040270.1	44594067	A	G
NC_040270.1	44835843	T	C
NC_040270.1	44841262	G	A
NC_040270.1	44849039	A	G
NC_040270.1	44866645	T	G
NC_040270.1	44866924	A	G
NC_040270.1	44867167	A	G
NC_040270.1	44873605	C	T
NC_040270.1	44921809	G	C
NC_040270.1	44967226	A	G
NC_040270.1	45009014	A	G
NC_040270.1	45009528	A	G
NC_040270.1	45010772	T	C
NC_040270.1	45015587	A	G
NC_040270.1	45029773	T	G
NC_040270.1	45103033	A	G
NC_040270.1	45130494	A	G
NC_040270.1	45157498	A	G
NC_040270.1	45163596	A	G
NC_040270.1	45181866	A	G
NC_040270.1	45186810	G	A
NC_040270.1	45323144	T	C
NC_040270.1	45323424	C	T
NC_040270.1	45324312	A	T
NC_040270.1	45338839	T	G
NC_040270.1	45523304	A	G
NC_040270.1	45555065	C	T
NC_040270.1	45629650	A	G
NC_040270.1	45632140	A	G
NC_040270.1	45632738	A	C
NC_040270.1	45691374	T	C
NC_040270.1	45751343	T	C

NC_040270.1	45788239	C	A
NC_040270.1	45813461	T	C
NC_040270.1	45829578	T	C
NC_040270.1	45829884	A	G
NC_040270.1	45831947	A	C
NC_040270.1	45832644	T	G
NC_040270.1	45866506	T	C
NC_040270.1	45877348	A	G
NC_040270.1	45877505	C	G
NC_040270.1	45878794	C	T
NC_040270.1	45879001	A	C
NC_040270.1	45879196	T	C
NC_040270.1	45879380	T	C
NC_040270.1	45881560	T	C
NC_040270.1	45881859	C	T
NC_040270.1	45882773	A	G
NC_040270.1	45885139	A	G
NC_040270.1	45971795	T	C
NC_040270.1	45973439	T	C
NC_040270.1	45976508	A	G
NC_040270.1	45976694	T	G
NC_040270.1	45978520	G	T
NC_040270.1	45981656	G	A
NC_040270.1	45982915	C	T
NC_040270.1	45992148	C	G
NC_040270.1	45995795	A	G
NC_040270.1	45996307	G	A
NC_040270.1	46031196	G	A
NC_040270.1	46328535	T	C
NC_040270.1	46328711	T	C
NC_040270.1	46332622	T	C
NC_040270.1	46332797	G	C
NC_040270.1	46357890	T	C
NC_040270.1	46361918	G	A
NC_040270.1	46369831	A	G
NC_040270.1	46377550	C	A
NC_040270.1	46379643	C	T
NC_040270.1	46379815	G	A
NC_040270.1	46380217	T	C
NC_040270.1	46390409	T	C
NC_040270.1	46419559	T	C
NC_040270.1	46934052	A	G
NC_040270.1	47027485	G	A
NC_040270.1	47027918	A	G
NC_040270.1	47553292	T	C
NC_040270.1	47553470	C	G
NC_040270.1	47553639	A	G

NC_040270.1	47553921	T	C
NC_040270.1	47554191	C	T
NC_040270.1	47570906	A	C
NC_040270.1	48056203	T	C
NC_040270.1	48059450	T	C
NC_040270.1	48065299	C	G
NC_040270.1	48066839	C	T
NC_040270.1	48067932	A	T
NC_040270.1	48068342	T	C
NC_040270.1	48069675	A	G
NC_040270.1	48070184	C	T
NC_040270.1	48070337	G	A
NC_040270.1	48364096	A	G
NC_040270.1	48623867	C	T
NC_040270.1	49015278	C	T
NC_040270.1	49039184	C	T
NC_040270.1	49046503	T	C
NC_040270.1	49053728	T	C
NC_040270.1	49121542	T	C
NC_040270.1	49142696	A	G
NC_040270.1	49143530	G	A
NC_040270.1	49144153	T	C
NC_040270.1	49147068	T	C
NC_040270.1	49148522	C	T
NC_040270.1	49206752	T	C
NC_040270.1	49497289	G	A
NC_040270.1	49676049	C	A
NC_040270.1	49678033	A	G
NC_040270.1	49712598	C	T
NC_040270.1	49731758	C	T
NC_040270.1	49731963	G	A
NC_040270.1	49832075	C	G
NC_040270.1	49857966	A	C
NC_040270.1	49940823	A	G
NC_040270.1	50027038	C	T
NC_040270.1	50028520	T	C
NC_040270.1	50028733	G	A
NC_040270.1	50029062	C	T
NC_040270.1	50034985	T	C
NC_040270.1	50062611	A	T
NC_040270.1	50067173	T	G
NC_040270.1	50075726	C	T
NC_040270.1	50107241	A	G
NC_040270.1	50114440	G	A
NC_040270.1	50134192	T	C
NC_040270.1	50143093	C	T
NC_040270.1	50143464	G	A

NC_040270.1	50177896	C	T
NC_040270.1	50188751	G	A
NC_040270.1	50207446	G	A
NC_040270.1	50207872	C	T
NC_040270.1	50249896	T	A
NC_040270.1	50250138	C	A
NC_040270.1	50251644	G	C
NC_040270.1	50251844	C	T
NC_040270.1	50258568	T	C
NC_040270.1	50309686	A	G
NC_040270.1	50315067	A	G
NC_040270.1	50319118	A	G
NC_040270.1	50324905	A	G
NC_040270.1	50330378	C	A
NC_040270.1	50332799	T	C
NC_040270.1	50339451	T	C
NC_040270.1	50343302	A	G
NC_040270.1	50380991	G	A
NC_040270.1	50413662	T	C
NC_040270.1	50414726	C	T
NC_040270.1	50419500	A	G
NC_040270.1	50431576	T	C
NC_040270.1	50442479	C	T
NC_040270.1	50443033	G	A
NC_040270.1	50449033	A	T
NC_040270.1	50453802	T	C
NC_040270.1	50469892	G	A
NC_040270.1	50531075	A	G
NC_040270.1	50556810	A	G
NC_040270.1	50602992	G	A
NC_040270.1	50603805	T	C
NC_040270.1	50605052	T	C
NC_040270.1	50608614	T	G
NC_040270.1	50608861	A	G
NC_040270.1	50628812	A	G
NC_040270.1	50630424	A	G
NC_040270.1	50634320	T	C
NC_040270.1	50634826	A	G
NC_040270.1	50635008	C	T
NC_040270.1	50646756	G	T
NC_040270.1	50651266	A	G
NC_040270.1	50783679	T	C
NC_040270.1	50785335	A	G
NC_040270.1	50909538	C	T
NC_040270.1	50923770	G	T
NC_040270.1	50927375	A	G
NC_040270.1	50927589	A	G

NC_040270.1	50935261	T	C
NC_040270.1	50935725	T	G
NC_040270.1	50936239	T	C
NC_040270.1	50949346	T	G
NC_040270.1	50949504	C	G
NC_040270.1	50955968	G	T
NC_040270.1	50962651	G	A
NC_040270.1	50965469	A	G
NC_040270.1	51005681	T	G
NC_040270.1	51077519	G	A
NC_040270.1	51077672	T	C
NC_040270.1	51078056	A	G
NC_040270.1	51078317	A	G
NC_040270.1	51080427	A	G
NC_040270.1	51088601	A	G
NC_040270.1	51119098	A	G
NC_040270.1	51126738	A	G
NC_040270.1	51143696	T	C
NC_040270.1	51210438	G	T
NC_040270.1	51338255	T	C
NC_040270.1	51338423	A	C
NC_040270.1	51360738	A	G
NC_040270.1	51366556	G	A
NC_040270.1	51367227	A	G
NC_040270.1	51369598	A	G
NC_040270.1	51370760	A	G
NC_040270.1	51371266	A	G
NC_040270.1	51380803	A	G
NC_040270.1	51381578	G	A
NC_040270.1	51381789	C	T
NC_040270.1	51458006	T	C
NC_040270.1	51696583	A	G
NC_040270.1	51737693	C	A
NC_040270.1	51766995	T	C
NC_040270.1	51775523	A	G
NC_040270.1	51775756	T	C
NC_040270.1	51955506	A	G
NC_040270.1	51960604	C	T
NC_040270.1	51968810	T	C
NC_040270.1	51970257	C	T
NC_040270.1	51971576	T	A
NC_040270.1	51971796	G	A
NC_040270.1	51976555	T	C
NC_040270.1	51983955	C	T
NC_040270.1	51984823	A	G
NC_040270.1	51991684	A	G
NC_040270.1	52013025	A	G

NC_040270.1	52017004	C	T
NC_040270.1	52018498	G	A
NC_040270.1	52021403	G	A
NC_040270.1	52022168	A	G
NC_040270.1	52022586	T	C
NC_040270.1	52023977	T	C
NC_040270.1	52024164	A	G
NC_040270.1	52025170	T	C
NC_040270.1	52025361	A	G
NC_040270.1	52054481	G	C
NC_040270.1	52058921	A	C
NC_040270.1	52059214	T	C
NC_040270.1	52072605	C	T
NC_040270.1	52072924	A	G
NC_040270.1	52078265	A	G
NC_040270.1	52079263	T	C
NC_040270.1	52089653	A	G
NC_040270.1	52120599	A	G
NC_040270.1	52140922	C	G
NC_040270.1	52141113	C	T
NC_040270.1	52150245	C	T
NC_040270.1	52153240	A	G
NC_040270.1	52173480	T	C
NC_040270.1	52173662	A	G
NC_040270.1	52175420	T	C
NC_040270.1	52214175	T	C
NC_040270.1	52235580	A	G
NC_040270.1	52237182	G	T
NC_040270.1	52239093	A	G
NC_040270.1	52330208	G	A
NC_040270.1	52333630	A	C
NC_040270.1	52339536	T	C
NC_040270.1	52361672	T	C
NC_040270.1	52362948	G	A
NC_040270.1	52364419	A	C
NC_040270.1	52364929	T	C
NC_040270.1	52378493	T	C
NC_040270.1	52387358	T	C
NC_040270.1	52402944	T	C
NC_040270.1	52403249	A	G
NC_040270.1	52405294	A	G
NC_040270.1	52405735	T	C
NC_040270.1	52412225	T	C
NC_040270.1	52414441	G	A
NC_040270.1	52414655	C	G
NC_040270.1	52414836	T	C
NC_040270.1	52422288	C	T

NC_040270.1	52423086	T	C
NC_040270.1	52454295	T	C
NC_040270.1	52459826	G	A
NC_040270.1	52460358	C	T
NC_040270.1	52460897	A	G
NC_040270.1	52461447	G	C
NC_040270.1	52461748	T	C
NC_040270.1	52462938	A	G
NC_040270.1	52464964	A	G
NC_040270.1	52466834	A	G
NC_040270.1	52468242	A	G
NC_040270.1	52468791	T	C
NC_040270.1	52469065	C	T
NC_040270.1	52480862	A	G
NC_040270.1	52493559	T	G
NC_040270.1	52494027	G	A
NC_040270.1	52495244	T	C
NC_040270.1	52497160	T	C
NC_040270.1	52497927	T	C
NC_040270.1	52508366	T	G
NC_040270.1	52508520	T	C
NC_040270.1	52510192	A	G
NC_040270.1	52510534	T	C
NC_040270.1	52510932	A	G
NC_040270.1	52518074	T	C
NC_040270.1	52518269	T	C
NC_040270.1	52519343	A	G
NC_040270.1	52522365	A	G
NC_040270.1	52522617	T	C
NC_040270.1	52523565	T	C
NC_040270.1	52523880	T	C
NC_040270.1	52524135	T	C
NC_040270.1	52524811	T	C
NC_040270.1	52524965	C	T
NC_040270.1	52527510	T	C
NC_040270.1	52527777	T	C
NC_040270.1	52536618	T	C
NC_040270.1	52595771	T	A
NC_040270.1	52597675	T	C
NC_040270.1	52598295	A	G
NC_040270.1	52599366	C	G
NC_040270.1	52674637	G	C
NC_040270.1	52675625	G	C
NC_040270.1	52683540	T	C
NC_040270.1	52735093	T	A
NC_040270.1	52735755	G	C
NC_040270.1	52735916	T	C

NC_040270.1	52768449	A	G
NC_040270.1	52776904	A	G
NC_040270.1	52784380	A	G
NC_040270.1	52794625	G	A
NC_040270.1	52794862	C	T
NC_040270.1	52795154	T	A
NC_040270.1	52795379	C	A
NC_040270.1	52795568	G	T
NC_040270.1	52796282	G	A
NC_040270.1	52817075	T	C
NC_040270.1	52817462	G	A
NC_040270.1	52836047	T	C
NC_040270.1	52836723	G	A
NC_040270.1	52840442	A	C
NC_040270.1	52845586	G	A
NC_040270.1	52846144	G	C
NC_040270.1	52846766	C	A
NC_040270.1	52849636	G	C
NC_040270.1	52852198	A	G
NC_040270.1	52853197	T	C
NC_040270.1	52856477	G	A
NC_040270.1	52860451	A	G
NC_040270.1	52861963	T	C
NC_040270.1	52863949	A	C
NC_040270.1	52864950	A	G
NC_040270.1	52869202	A	G
NC_040270.1	52871549	T	C
NC_040270.1	52871863	A	G
NC_040270.1	52872091	A	G
NC_040270.1	52873309	T	C
NC_040270.1	52874646	A	C
NC_040270.1	52874888	T	C
NC_040270.1	52885100	T	C
NC_040270.1	52887229	T	C
NC_040270.1	52887916	G	C
NC_040270.1	52888172	A	T
NC_040270.1	52896881	T	C
NC_040270.1	52897098	G	T
NC_040270.1	52925415	G	A
NC_040270.1	52941541	C	T
NC_040270.1	52946592	A	G
NC_040270.1	52947703	A	G
NC_040270.1	52952055	A	G
NC_040270.1	52952239	T	C
NC_040270.1	52953831	T	C
NC_040270.1	52956846	G	T
NC_040270.1	52958255	A	G

NC_040270.1	52962834	C	T
NC_040270.1	52965297	C	G
NC_040270.1	52978315	A	G
NC_040270.1	52979254	T	G
NC_040270.1	53010574	C	T
NC_040270.1	53014719	A	G
NC_040270.1	53014875	C	A
NC_040270.1	53015508	G	A
NC_040270.1	53015687	G	A
NC_040270.1	53015906	T	G
NC_040270.1	53130491	A	G
NC_040270.1	53132538	G	A
NC_040270.1	53180198	T	C
NC_040270.1	53185779	G	T
NC_040270.1	53194121	C	T
NC_040270.1	53194891	G	T
NC_040270.1	53195140	C	G
NC_040270.1	53195716	A	G
NC_040270.1	53196036	C	G
NC_040270.1	53196708	A	C
NC_040270.1	53197393	A	G
NC_040270.1	53197695	A	G
NC_040270.1	53200043	T	C
NC_040270.1	53204129	T	C
NC_040270.1	53213563	T	C
NC_040270.1	53220347	A	G
NC_040270.1	53221175	T	C
NC_040270.1	53223881	A	T
NC_040270.1	53224451	A	G
NC_040270.1	53244211	A	G
NC_040270.1	53256891	T	C
NC_040270.1	53258460	A	G
NC_040270.1	53286158	A	G
NC_040270.1	53287681	T	C
NC_040270.1	53288309	C	G
NC_040270.1	53327050	A	G
NC_040270.1	53337313	C	G
NC_040270.1	53356372	A	G
NC_040270.1	53356604	A	G
NC_040270.1	53359760	A	G
NC_040270.1	53361700	A	G
NC_040270.1	53369763	G	C
NC_040270.1	53374390	T	C
NC_040270.1	53391420	C	T
NC_040270.1	53391576	G	A
NC_040270.1	53393941	T	G
NC_040270.1	53394281	A	G

NC_040270.1	53394962	T	C
NC_040270.1	53405608	A	G
NC_040270.1	53407013	T	C
NC_040270.1	53412042	G	C
NC_040270.1	53416519	T	C
NC_040270.1	53425296	G	A
NC_040270.1	53425487	T	C
NC_040270.1	53648172	T	C
NC_040270.1	53659441	G	C
NC_040270.1	53662363	C	T
NC_040270.1	53662887	C	T
NC_040270.1	53663097	T	G
NC_040270.1	53663512	T	C
NC_040270.1	53663779	C	A
NC_040270.1	53771024	C	A
NC_040270.1	53787347	A	G
NC_040270.1	53823336	A	G
NC_040270.1	53823922	T	C
NC_040270.1	53828242	C	G
NC_040270.1	53837462	G	A
NC_040270.1	53837856	A	G
NC_040270.1	53852935	T	C
NC_040270.1	53863911	T	C
NC_040270.1	53864940	T	C
NC_040270.1	53865091	A	G
NC_040270.1	53865869	T	C
NC_040270.1	53867861	A	G
NC_040270.1	53869135	G	A
NC_040270.1	53869948	C	G
NC_040270.1	53894025	T	A
NC_040270.1	53894694	G	C
NC_040270.1	53971142	T	C
NC_040270.1	53979772	A	T
NC_040270.1	54154575	G	C
NC_040270.1	54198427	T	C
NC_040270.1	54209950	A	G
NC_040270.1	54213029	A	G
NC_040270.1	54213876	A	G
NC_040270.1	54214158	T	C
NC_040270.1	54214898	A	G
NC_040270.1	54215319	A	G
NC_040270.1	54215490	G	A
NC_040270.1	54241944	T	C
NC_040270.1	54254929	T	C
NC_040270.1	54255090	A	C
NC_040270.1	54267411	A	G
NC_040270.1	54303902	C	A

NC_040270.1	54314509	A	G
NC_040270.1	54320477	T	C
NC_040270.1	54409065	T	C
NC_040270.1	54411052	C	T
NC_040270.1	54412696	G	C
NC_040270.1	54436466	T	C
NC_040270.1	54452280	C	T
NC_040270.1	54463641	T	G
NC_040270.1	54466888	T	C
NC_040270.1	54469963	T	C
NC_040270.1	54470764	T	G
NC_040270.1	54474869	A	G
NC_040270.1	54478268	T	C
NC_040270.1	54478642	A	G
NC_040270.1	54483424	T	C
NC_040270.1	54484393	C	T
NC_040270.1	54575054	T	C
NC_040270.1	54626596	A	G
NC_040270.1	54729227	A	G
NC_040270.1	54731239	T	C
NC_040270.1	54738126	C	T
NC_040270.1	54738291	A	G
NC_040270.1	54875248	C	T
NC_040270.1	54875959	T	C
NC_040270.1	54882005	T	C
NC_040270.1	54947559	C	T
NC_040270.1	54976917	A	G
NC_040270.1	54990990	C	T
NC_040270.1	55087939	A	G
NC_040270.1	55183201	C	T
NC_040270.1	55183410	T	G
NC_040270.1	55188419	A	G
NC_040270.1	55191325	A	G
NC_040270.1	55269482	T	C
NC_040270.1	55270100	A	G
NC_040270.1	55270432	T	C
NC_040270.1	55270745	C	T
NC_040270.1	55271456	G	A
NC_040270.1	55285734	A	G
NC_040270.1	55303792	A	G
NC_040270.1	55304059	T	C
NC_040270.1	55304377	C	G
NC_040270.1	55324998	A	G
NC_040270.1	55329369	G	A
NC_040270.1	55340440	A	G
NC_040270.1	55353084	A	G
NC_040270.1	55356209	A	G

NC_040270.1	55368799	A	G
NC_040270.1	55369054	C	T
NC_040270.1	55440840	G	C
NC_040270.1	55484004	C	T
NC_040270.1	55508237	A	T
NC_040270.1	55514012	C	T
NC_040270.1	55514358	T	C
NC_040270.1	55515005	G	A
NC_040270.1	55642088	T	A
NC_040270.1	55674326	A	G
NC_040270.1	55937727	C	G
NC_040270.1	56064347	G	A
NC_040270.1	56069723	T	C
NC_040270.1	56181586	A	G
NC_040270.1	56194970	A	G
NC_040270.1	56231896	T	C
NC_040270.1	56265814	C	T
NC_040270.1	56283503	A	G
NC_040270.1	56284134	A	G
NC_040270.1	56284688	C	A
NC_040270.1	56284844	A	G
NC_040270.1	56286663	C	T
NC_040270.1	56287509	T	C
NC_040270.1	56287678	T	A
NC_040270.1	56320546	A	G
NC_040270.1	56898965	A	G
NC_040270.1	56899159	G	A
NC_040270.1	56977784	C	G
NC_040270.1	56992701	G	A
NC_040270.1	57095425	0	A
NC_040270.1	57202028	C	G
NC_040270.1	57202642	A	G
NC_040270.1	57485909	C	G
NC_040270.1	57793292	A	G
NC_040270.1	57793464	T	C
NC_040270.1	57793677	T	C
NC_040270.1	57897863	C	G
NC_040270.1	57898445	A	C
NC_040270.1	57972551	T	G
NC_040270.1	57973416	G	C
NC_040270.1	57974133	G	A
NC_040270.1	57984848	A	G
NC_040270.1	58007938	A	G
NC_040270.1	58008517	A	G
NC_040270.1	58039890	G	C
NC_040270.1	58069841	A	G
NC_040270.1	58088052	G	A

NC_040270.1	58096961	T	C
NC_040270.1	58131983	A	G
NC_040270.1	58135738	A	G
NC_040270.1	58137410	T	C
NC_040270.1	58143663	T	C
NC_040270.1	58153949	A	G
NC_040270.1	58217633	A	G
NC_040270.1	58218364	T	A
NC_040270.1	58224218	A	G
NC_040270.1	58249012	G	A
NC_040270.1	58259380	A	G
NC_040270.1	58259686	T	C
NC_040270.1	58260228	A	G
NC_040270.1	58320918	T	C
NC_040270.1	58428960	A	G
NC_040270.1	58440630	T	C
NC_040270.1	58457291	T	C
NC_040270.1	58478774	A	G
NC_040270.1	58547823	A	G
NC_040270.1	58841720	A	G
NC_040270.1	58842007	A	G
NC_040270.1	58842914	C	T
NC_040270.1	59078568	G	A
NC_040270.1	59079140	C	T
NC_040270.1	59144649	C	T
NC_040270.1	59146558	C	T
NC_040270.1	59146725	A	G
NC_040270.1	59150888	C	G
NC_040270.1	59151110	A	G
NC_040270.1	59278340	C	T
NC_040270.1	59278719	A	G
NC_040270.1	59307953	A	G
NC_040270.1	59338229	A	C
NC_040270.1	59382670	A	G
NC_040270.1	59437338	T	G
NC_040270.1	59452713	A	G
NC_040270.1	59486597	A	G
NC_040270.1	59497359	T	C
NC_040270.1	59651687	C	G
NC_040270.1	59652563	A	G
NC_040270.1	59671640	T	C
NC_040270.1	59673587	C	G
NC_040270.1	59720672	T	C
NC_040270.1	59722273	A	T
NC_040270.1	59723827	A	G
NC_040270.1	59741683	T	C
NC_040270.1	59741897	A	G

NC_040270.1	59742364	G	A
NC_040270.1	59743122	C	T
NC_040270.1	59936150	C	G
NC_040270.1	59945316	A	G
NC_040270.1	60085465	A	G
NC_040270.1	60201475	C	T
NC_040270.1	60296441	C	G
NC_040270.1	60442542	A	G
NC_040270.1	60443464	T	C
NC_040270.1	60717928	T	C
NC_040270.1	60891815	A	G
NC_040270.1	60904812	T	G
NC_040270.1	60905019	G	A
NC_040270.1	60905181	A	G
NC_040270.1	60911153	C	G
NC_040270.1	60928141	G	A
NC_040270.1	60941313	C	T
NC_040270.1	60962961	A	C
NC_040270.1	61088087	C	G
NC_040270.1	61127560	A	G
NC_040270.1	61245244	G	A
NC_040270.1	61304186	0	G
NC_040270.1	61320499	T	C
NC_040270.1	61342173	T	C
NC_040270.1	61360285	T	C
NC_040270.1	61452925	T	C
NC_040270.1	61495153	G	C
NC_040270.1	61495422	T	C
NC_040270.1	61495704	A	G
NC_040270.1	61495880	T	G
NC_040270.1	61787913	T	G
NC_040270.1	61876947	G	A
NC_040270.1	62208726	A	G
NC_040270.1	62219810	C	T
NC_040270.1	62221613	A	G
NC_040270.1	62229178	C	T
NC_040270.1	62239091	T	C
NC_040270.1	62239330	A	G
NC_040270.1	62239573	T	C
NC_040270.1	62252918	A	G
NC_040270.1	62386236	T	C
NC_040270.1	62412403	A	G
NC_040270.1	62421885	A	G
NC_040270.1	62451749	G	T
NC_040270.1	62466628	T	C
NC_040270.1	62479843	C	T
NC_040270.1	62481710	A	G

NC_040270.1	62487785	G	T
NC_040270.1	62488841	T	C
NC_040270.1	62489845	A	G
NC_040270.1	62491786	T	C
NC_040270.1	62506710	T	C
NC_040270.1	62562288	G	C
NC_040270.1	62562579	G	A
NC_040270.1	62565119	G	A
NC_040270.1	62565895	T	C
NC_040270.1	62572212	A	G
NC_040270.1	62622017	A	G
NC_040270.1	62623251	C	T
NC_040270.1	62625338	A	C
NC_040271.1	201315	C	T
NC_040271.1	264289	A	G
NC_040271.1	288174	G	A
NC_040271.1	438472	A	G
NC_040271.1	475935	C	T
NC_040271.1	521252	A	C
NC_040271.1	569930	A	G
NC_040271.1	613960	A	G
NC_040271.1	702670	C	T
NC_040271.1	740044	C	T
NC_040271.1	898383	C	G
NC_040271.1	934956	C	T
NC_040271.1	1158652	T	C
NC_040271.1	1259565	G	A
NC_040271.1	1302978	G	A
NC_040271.1	1470553	T	C
NC_040271.1	4013016	C	T
NC_040271.1	4029065	C	T
NC_040271.1	4109486	T	C
NC_040271.1	4224953	C	T
NC_040271.1	4240932	A	G
NC_040271.1	4261296	G	A
NC_040271.1	4261731	G	A
NC_040271.1	4262034	T	C
NC_040271.1	4265598	G	T
NC_040271.1	4276864	T	C
NC_040271.1	4304423	C	T
NC_040271.1	4305216	C	T
NC_040271.1	4306015	C	G
NC_040271.1	4382574	T	C
NC_040271.1	4428051	A	T
NC_040271.1	4469583	A	G
NC_040271.1	4520635	C	G
NC_040271.1	4577342	G	A

NC_040271.1	4635420	G	A
NC_040271.1	4684705	A	G
NC_040271.1	4729237	T	C
NC_040271.1	4758224	G	A
NC_040271.1	4775692	G	A
NC_040271.1	4777318	T	C
NC_040271.1	4777492	A	G
NC_040271.1	4778262	T	C
NC_040271.1	4778622	A	G
NC_040271.1	4780621	A	G
NC_040271.1	4781959	T	C
NC_040271.1	4785768	A	G
NC_040271.1	4790315	A	G
NC_040271.1	4792679	T	C
NC_040271.1	4792893	G	A
NC_040271.1	4795827	C	T
NC_040271.1	4801996	A	T
NC_040271.1	4826887	G	A
NC_040271.1	4869829	A	C
NC_040271.1	4919676	T	C
NC_040271.1	4941776	A	G
NC_040271.1	5227038	C	T
NC_040271.1	5280812	A	G
NC_040271.1	5333116	C	T
NC_040271.1	5559139	G	C
NC_040271.1	5592076	A	C
NC_040271.1	5824989	A	G
NC_040271.1	5825430	G	A
NC_040271.1	5875751	C	T
NC_040271.1	5920544	A	T
NC_040271.1	5967200	A	G
NC_040271.1	5967350	T	C
NC_040271.1	6208658	C	T
NC_040271.1	6268195	C	T
NC_040271.1	6379389	C	T
NC_040271.1	6434528	T	G
NC_040271.1	6647555	T	C
NC_040271.1	6705932	T	A
NC_040271.1	6760635	C	A
NC_040271.1	6800286	G	A
NC_040271.1	6833350	C	G
NC_040271.1	7030462	A	G
NC_040271.1	7032475	T	C
NC_040271.1	7032769	A	G
NC_040271.1	7033323	G	A
NC_040271.1	7033820	C	T
NC_040271.1	7034646	A	G

NC_040271.1	7034796	T	C
NC_040271.1	7079996	T	A
NC_040271.1	7104991	G	A
NC_040271.1	7644642	A	G
NC_040271.1	7677413	A	G
NC_040271.1	7786847	A	C
NC_040271.1	7799720	C	G
NC_040271.1	7843972	C	T
NC_040271.1	7891366	C	T
NC_040271.1	7944975	A	G
NC_040271.1	7992677	G	A
NC_040271.1	8062939	C	T
NC_040271.1	8168655	G	A
NC_040271.1	8221328	A	T
NC_040271.1	8260256	A	G
NC_040271.1	8292060	C	G
NC_040271.1	8397233	C	G
NC_040271.1	8410751	T	C
NC_040271.1	8415923	C	G
NC_040271.1	8553506	0	C
NC_040271.1	8609536	A	G
NC_040271.1	8861979	A	G
NC_040271.1	8867419	T	C
NC_040271.1	8867861	T	C
NC_040271.1	8868796	A	G
NC_040271.1	8892693	C	T
NC_040271.1	8967992	C	A
NC_040271.1	8991274	T	G
NC_040271.1	8992175	T	C
NC_040271.1	9022340	A	G
NC_040271.1	9053706	T	C
NC_040271.1	9053989	G	C
NC_040271.1	9055757	A	G
NC_040271.1	9064482	A	G
NC_040271.1	9068408	A	G
NC_040271.1	9083793	T	C
NC_040271.1	9084961	G	A
NC_040271.1	9090593	A	C
NC_040271.1	9091182	C	G
NC_040271.1	9093911	T	C
NC_040271.1	9132984	G	A
NC_040271.1	9143711	A	G
NC_040271.1	9148412	T	C
NC_040271.1	9154676	T	C
NC_040271.1	9154906	T	C
NC_040271.1	9157576	T	C
NC_040271.1	9164169	G	A

NC_040271.1	9164686	G	T
NC_040271.1	9165343	C	T
NC_040271.1	9168811	T	C
NC_040271.1	9174790	G	C
NC_040271.1	9187115	A	G
NC_040271.1	9188204	A	G
NC_040271.1	9192240	A	G
NC_040271.1	9278021	C	G
NC_040271.1	9286706	C	G
NC_040271.1	9288299	G	A
NC_040271.1	9296169	T	C
NC_040271.1	9297473	C	T
NC_040271.1	9297723	T	C
NC_040271.1	9322180	T	C
NC_040271.1	9322813	T	C
NC_040271.1	9323416	A	G
NC_040271.1	9330938	G	A
NC_040271.1	9332439	C	G
NC_040271.1	9334106	A	C
NC_040271.1	9335889	A	G
NC_040271.1	9336339	C	T
NC_040271.1	9336980	T	C
NC_040271.1	9411053	T	C
NC_040271.1	9413600	A	G
NC_040271.1	9461862	C	T
NC_040271.1	9507642	T	C
NC_040271.1	9546611	T	C
NC_040271.1	9546951	0	A
NC_040271.1	9592213	C	T
NC_040271.1	9592445	A	C
NC_040271.1	9616718	C	A
NC_040271.1	9836801	A	G
NC_040271.1	9854529	G	A
NC_040271.1	9893800	G	A
NC_040271.1	9939838	T	C
NC_040271.1	9940384	A	G
NC_040271.1	10010606	G	A
NC_040271.1	10043241	C	G
NC_040271.1	10045341	C	T
NC_040271.1	10099687	G	T
NC_040271.1	10156642	0	G
NC_040271.1	10176864	T	C
NC_040271.1	10263168	T	C
NC_040271.1	10268750	T	A
NC_040271.1	10269852	T	C
NC_040271.1	10270328	A	G
NC_040271.1	10320318	G	C

NC_040271.1	10320470	G	A
NC_040271.1	10322541	0	G
NC_040271.1	10322771	A	G
NC_040271.1	10328774	G	T
NC_040271.1	10329059	A	G
NC_040271.1	10329213	C	T
NC_040271.1	10374996	T	C
NC_040271.1	10422754	A	G
NC_040271.1	10469180	A	T
NC_040271.1	10511261	A	G
NC_040271.1	10556915	T	C
NC_040271.1	10560650	A	G
NC_040271.1	10609575	G	C
NC_040271.1	10652793	T	C
NC_040271.1	10654216	T	A
NC_040271.1	10680177	G	A
NC_040271.1	10785398	C	T
NC_040271.1	10875866	G	C
NC_040271.1	10897790	T	C
NC_040271.1	10900872	T	C
NC_040271.1	10901926	A	G
NC_040271.1	10904930	A	G
NC_040271.1	10909690	A	G
NC_040271.1	10922826	G	A
NC_040271.1	10948008	T	C
NC_040271.1	10950951	A	G
NC_040271.1	10951508	T	G
NC_040271.1	10993641	A	G
NC_040271.1	11035327	C	A
NC_040271.1	11065881	T	C
NC_040271.1	11066133	T	C
NC_040271.1	11068202	T	C
NC_040271.1	11098099	T	C
NC_040271.1	11098446	T	C
NC_040271.1	11112645	A	G
NC_040271.1	11147216	A	G
NC_040271.1	11147642	A	G
NC_040271.1	11255262	T	C
NC_040271.1	11310236	C	T
NC_040271.1	11367002	A	G
NC_040271.1	11408495	T	C
NC_040271.1	11409616	A	G
NC_040271.1	11410943	A	G
NC_040271.1	11412411	A	G
NC_040271.1	11459652	T	G
NC_040271.1	11463893	C	T
NC_040271.1	11464362	G	A

NC_040271.1	11477089	T	C
NC_040271.1	11495158	C	T
NC_040271.1	11500371	C	T
NC_040271.1	11562342	C	T
NC_040271.1	11562696	A	G
NC_040271.1	11564190	A	G
NC_040271.1	11568136	G	T
NC_040271.1	11586826	A	G
NC_040271.1	11587886	T	C
NC_040271.1	11645712	G	T
NC_040271.1	11703924	A	C
NC_040271.1	11713767	A	G
NC_040271.1	11717280	G	A
NC_040271.1	11730831	0	G
NC_040271.1	11739147	C	T
NC_040271.1	11788758	T	A
NC_040271.1	11790116	A	G
NC_040271.1	11802480	T	C
NC_040271.1	11802651	A	G
NC_040271.1	11819031	T	C
NC_040271.1	11878272	G	A
NC_040271.1	11923957	A	G
NC_040271.1	11997702	C	T
NC_040271.1	12053173	G	A
NC_040271.1	12062647	T	C
NC_040271.1	12068549	A	G
NC_040271.1	12068704	C	G
NC_040271.1	12069043	C	T
NC_040271.1	12069435	A	C
NC_040271.1	12069622	A	C
NC_040271.1	12118809	C	A
NC_040271.1	12167574	A	G
NC_040271.1	12212919	T	G
NC_040271.1	12237043	A	G
NC_040271.1	12273010	A	G
NC_040271.1	12291455	C	T
NC_040271.1	12344242	T	C
NC_040271.1	12401179	A	C
NC_040271.1	12454018	C	T
NC_040271.1	12472196	T	C
NC_040271.1	12476786	A	G
NC_040271.1	12477073	C	T
NC_040271.1	12477694	T	C
NC_040271.1	12478842	A	G
NC_040271.1	12479054	A	G
NC_040271.1	12480638	C	G
NC_040271.1	12512484	G	A

NC_040271.1	12553418	T	C
NC_040271.1	12701945	T	C
NC_040271.1	12750712	G	A
NC_040271.1	12792646	T	C
NC_040271.1	12836994	T	C
NC_040271.1	12838119	G	A
NC_040271.1	12881418	C	T
NC_040271.1	12938279	T	C
NC_040271.1	12974364	A	G
NC_040271.1	12975958	G	A
NC_040271.1	13150111	A	G
NC_040271.1	13155841	A	G
NC_040271.1	13168589	T	C
NC_040271.1	13208865	C	G
NC_040271.1	13329702	A	G
NC_040271.1	13386428	A	G
NC_040271.1	13441691	G	A
NC_040271.1	13499001	T	C
NC_040271.1	13558010	A	G
NC_040271.1	13615861	A	G
NC_040271.1	13643051	T	C
NC_040271.1	13643486	A	G
NC_040271.1	13699824	T	C
NC_040271.1	13754889	A	T
NC_040271.1	13810915	A	G
NC_040271.1	13867179	C	A
NC_040271.1	13922629	T	C
NC_040271.1	13960360	T	A
NC_040271.1	14007807	G	T
NC_040271.1	14018055	A	G
NC_040271.1	14056939	G	A
NC_040271.1	14105292	C	G
NC_040271.1	14160755	C	T
NC_040271.1	14163210	C	T
NC_040271.1	14186414	C	T
NC_040271.1	14221419	A	G
NC_040271.1	14231271	T	C
NC_040271.1	14284958	C	T
NC_040271.1	14320615	T	C
NC_040271.1	14376651	G	A
NC_040271.1	14433283	A	G
NC_040271.1	14464730	C	A
NC_040271.1	14507258	G	C
NC_040271.1	14522840	G	A
NC_040271.1	14523436	T	C
NC_040271.1	14526408	G	A
NC_040271.1	14526857	T	C

NC_040271.1	14527952	A	G
NC_040271.1	14542680	T	C
NC_040271.1	14548954	C	T
NC_040271.1	14632432	0	C
NC_040271.1	14669904	A	G
NC_040271.1	14736121	T	G
NC_040271.1	14754005	T	C
NC_040271.1	14800081	G	A
NC_040271.1	14850179	T	C
NC_040271.1	14907889	A	G
NC_040271.1	14964667	C	A
NC_040271.1	15024844	T	C
NC_040271.1	15082115	T	G
NC_040271.1	15136052	A	G
NC_040271.1	15190223	T	C
NC_040271.1	15364583	C	T
NC_040271.1	15365154	G	C
NC_040271.1	15417905	G	A
NC_040271.1	15462046	G	C
NC_040271.1	15493076	A	G
NC_040271.1	15495814	G	T
NC_040271.1	15528883	A	G
NC_040271.1	16041669	G	A
NC_040271.1	16084983	C	G
NC_040271.1	16703122	T	C
NC_040271.1	16732281	A	C
NC_040271.1	16764951	A	G
NC_040271.1	16765322	T	C
NC_040271.1	16779211	G	A
NC_040271.1	16795264	C	T
NC_040271.1	16798490	T	C
NC_040271.1	16800340	C	T
NC_040271.1	16802589	C	T
NC_040271.1	16803837	A	G
NC_040271.1	16816325	C	A
NC_040271.1	16828487	A	G
NC_040271.1	16838914	G	A
NC_040271.1	16868557	T	C
NC_040271.1	16868813	T	C
NC_040271.1	16868998	G	A
NC_040271.1	16869437	T	C
NC_040271.1	16869646	T	C
NC_040271.1	16871729	C	T
NC_040271.1	16901182	T	C
NC_040271.1	17121598	T	C
NC_040271.1	17138844	A	C
NC_040271.1	17163323	A	G

NC_040271.1	17172210	T	C
NC_040271.1	17221352	G	A
NC_040271.1	17224422	C	A
NC_040271.1	17253855	T	A
NC_040271.1	17254472	T	C
NC_040271.1	17306931	T	C
NC_040271.1	17337518	G	A
NC_040271.1	17341298	T	C
NC_040271.1	17344613	C	T
NC_040271.1	17344781	G	T
NC_040271.1	17347956	G	C
NC_040271.1	17352511	A	G
NC_040271.1	17353897	A	G
NC_040271.1	17358005	G	A
NC_040271.1	17359136	T	C
NC_040271.1	17359716	T	C
NC_040271.1	17359922	C	A
NC_040271.1	17367514	A	G
NC_040271.1	17401033	G	A
NC_040271.1	17431990	G	A
NC_040271.1	17434150	A	G
NC_040271.1	17440788	A	C
NC_040271.1	17450441	T	G
NC_040271.1	17452057	T	G
NC_040271.1	17452587	A	G
NC_040271.1	17510559	G	A
NC_040271.1	17561403	T	C
NC_040271.1	17565545	T	A
NC_040271.1	17566971	G	A
NC_040271.1	17567258	A	G
NC_040271.1	17585124	G	A
NC_040271.1	17585313	A	G
NC_040271.1	17585562	A	G
NC_040271.1	17585753	T	C
NC_040271.1	17586907	A	G
NC_040271.1	17587252	C	G
NC_040271.1	17587607	A	G
NC_040271.1	17588835	T	A
NC_040271.1	17589358	C	T
NC_040271.1	17590322	T	C
NC_040271.1	17619089	C	G
NC_040271.1	17637787	C	T
NC_040271.1	17655790	T	C
NC_040271.1	17676990	G	A
NC_040271.1	17689691	G	A
NC_040271.1	17698938	T	C
NC_040271.1	17729266	C	T

NC_040271.1	17785359	A	G
NC_040271.1	17841906	T	C
NC_040271.1	17895882	T	C
NC_040271.1	18011165	G	T
NC_040271.1	18048278	G	A
NC_040271.1	18078481	G	A
NC_040271.1	18111921	A	G
NC_040271.1	18117931	T	C
NC_040271.1	18131102	T	C
NC_040271.1	18147062	A	G
NC_040271.1	18182144	C	T
NC_040271.1	18215373	T	C
NC_040271.1	18216367	A	G
NC_040271.1	18216727	T	C
NC_040271.1	18217952	G	C
NC_040271.1	18218113	G	A
NC_040271.1	18221410	G	T
NC_040271.1	18223117	A	G
NC_040271.1	18241084	G	A
NC_040271.1	18252982	A	C
NC_040271.1	18282293	C	T
NC_040271.1	18300984	A	G
NC_040271.1	18312930	T	C
NC_040271.1	18313963	C	T
NC_040271.1	18320147	A	G
NC_040271.1	18324258	T	C
NC_040271.1	18324455	T	C
NC_040271.1	18336997	C	T
NC_040271.1	18337151	C	T
NC_040271.1	18358729	A	G
NC_040271.1	18359217	T	C
NC_040271.1	18362923	A	G
NC_040271.1	18363585	T	A
NC_040271.1	18363848	G	T
NC_040271.1	18365927	A	G
NC_040271.1	18368191	T	C
NC_040271.1	18368657	G	C
NC_040271.1	18380709	T	C
NC_040271.1	18390082	A	C
NC_040271.1	18391425	G	A
NC_040271.1	18392670	G	C
NC_040271.1	18394068	A	G
NC_040271.1	18399209	A	G
NC_040271.1	18399818	T	C
NC_040271.1	18414565	T	C
NC_040271.1	18449337	T	C
NC_040271.1	18477832	A	G

NC_040271.1	18492063	T	C
NC_040271.1	18492810	T	C
NC_040271.1	18493608	T	C
NC_040271.1	18497698	A	C
NC_040271.1	18498846	T	C
NC_040271.1	18499869	T	C
NC_040271.1	18502692	T	C
NC_040271.1	18507181	G	A
NC_040271.1	18510890	C	G
NC_040271.1	18511070	T	C
NC_040271.1	18567358	A	G
NC_040271.1	18592263	A	G
NC_040271.1	18592598	C	G
NC_040271.1	18602722	C	T
NC_040271.1	18608072	C	T
NC_040271.1	18608879	A	C
NC_040271.1	18610785	A	T
NC_040271.1	18610954	C	T
NC_040271.1	18639771	A	G
NC_040271.1	18639949	G	T
NC_040271.1	18640576	C	T
NC_040271.1	18641756	C	T
NC_040271.1	18653374	C	T
NC_040271.1	18654295	T	C
NC_040271.1	18656349	C	T
NC_040271.1	18705406	G	A
NC_040271.1	18716836	T	C
NC_040271.1	18717588	T	C
NC_040271.1	18718633	A	G
NC_040271.1	18719993	T	G
NC_040271.1	18721845	A	G
NC_040271.1	18722789	T	C
NC_040271.1	18750945	A	G
NC_040271.1	18763905	T	C
NC_040271.1	18764864	G	A
NC_040271.1	18765155	A	G
NC_040271.1	18766130	T	G
NC_040271.1	18771163	G	A
NC_040271.1	18771887	T	C
NC_040271.1	18775147	T	C
NC_040271.1	18782545	G	A
NC_040271.1	18783302	C	T
NC_040271.1	18818649	G	C
NC_040271.1	18851870	A	C
NC_040271.1	18852446	A	G
NC_040271.1	18853011	A	T
NC_040271.1	18853356	G	A

NC_040271.1	18853522	T	G
NC_040271.1	18855543	G	A
NC_040271.1	18856320	G	A
NC_040271.1	18861375	G	A
NC_040271.1	18861803	T	C
NC_040271.1	18862789	A	G
NC_040271.1	18872714	G	A
NC_040271.1	18887799	T	C
NC_040271.1	18893285	G	A
NC_040271.1	18899951	A	G
NC_040271.1	18900297	T	C
NC_040271.1	18901326	A	G
NC_040271.1	18901936	T	C
NC_040271.1	18902114	G	A
NC_040271.1	18993321	A	G
NC_040271.1	19243341	T	C
NC_040271.1	19243512	G	C
NC_040271.1	19243760	T	C
NC_040271.1	19244071	T	C
NC_040271.1	19244348	A	G
NC_040271.1	19244503	T	C
NC_040271.1	19347122	A	G
NC_040271.1	19376665	T	C
NC_040271.1	19424634	T	C
NC_040271.1	19425716	A	C
NC_040271.1	19425904	A	G
NC_040271.1	19426091	A	C
NC_040271.1	19437416	A	G
NC_040271.1	19439312	A	G
NC_040271.1	19453764	T	C
NC_040271.1	19455121	G	T
NC_040271.1	19458326	A	G
NC_040271.1	19458797	A	G
NC_040271.1	19460917	T	C
NC_040271.1	19466177	T	C
NC_040271.1	19469907	G	T
NC_040271.1	19478050	T	C
NC_040271.1	19478206	T	C
NC_040271.1	19478812	T	C
NC_040271.1	19493069	G	A
NC_040271.1	19501160	T	C
NC_040271.1	19505356	C	T
NC_040271.1	19541367	T	G
NC_040271.1	19566725	T	C
NC_040271.1	19604461	T	C
NC_040271.1	19930818	C	A
NC_040271.1	19988236	C	T

NC_040271.1	20044924	C	A
NC_040271.1	20102820	C	T
NC_040271.1	20163299	C	T
NC_040271.1	20220778	T	C
NC_040271.1	20276784	C	T
NC_040271.1	20332768	G	T
NC_040271.1	20388825	C	G
NC_040271.1	20448161	C	T
NC_040271.1	20506755	T	C
NC_040271.1	20508170	A	T
NC_040271.1	20509132	G	C
NC_040271.1	20509494	G	T
NC_040271.1	20509718	T	C
NC_040271.1	20510193	A	G
NC_040271.1	20856752	G	A
NC_040271.1	20856958	G	A
NC_040271.1	20857879	T	C
NC_040271.1	20858361	C	T
NC_040271.1	20913185	C	G
NC_040271.1	20968416	T	C
NC_040271.1	21014641	T	C
NC_040271.1	21060128	T	C
NC_040271.1	21065794	T	C
NC_040271.1	21128545	A	G
NC_040271.1	21189251	G	T
NC_040271.1	21239411	T	C
NC_040271.1	21290699	T	C
NC_040271.1	21346183	G	A
NC_040271.1	21401306	T	C
NC_040271.1	21459417	C	T
NC_040271.1	21496824	C	T
NC_040271.1	21537093	C	T
NC_040271.1	21573354	A	G
NC_040271.1	21581863	C	A
NC_040271.1	21582661	G	A
NC_040271.1	21583665	T	C
NC_040271.1	21584745	C	G
NC_040271.1	21586441	T	C
NC_040271.1	21587117	C	G
NC_040271.1	21625331	C	T
NC_040271.1	21627611	G	C
NC_040271.1	21627868	G	A
NC_040271.1	21637189	T	G
NC_040271.1	21650805	G	A
NC_040271.1	21743067	A	G
NC_040271.1	21764967	A	C
NC_040271.1	21802469	G	A

NC_040271.1	21846055	A	G
NC_040271.1	21900948	T	C
NC_040271.1	21901141	G	A
NC_040271.1	21939844	A	G
NC_040271.1	22091011	G	A
NC_040271.1	22125610	A	G
NC_040271.1	22266555	T	C
NC_040271.1	22294671	A	G
NC_040271.1	22352612	A	G
NC_040271.1	22352849	A	T
NC_040271.1	22394353	T	C
NC_040271.1	22480057	G	A
NC_040271.1	22507730	C	T
NC_040271.1	22572757	A	G
NC_040271.1	22573064	A	C
NC_040271.1	22624045	A	C
NC_040271.1	22676882	A	G
NC_040271.1	22717329	C	T
NC_040271.1	22717507	T	C
NC_040271.1	22773073	A	C
NC_040271.1	23751033	G	T
NC_040271.1	23782721	T	C
NC_040271.1	23783745	T	C
NC_040271.1	23809464	G	A
NC_040271.1	23860696	T	C
NC_040271.1	23953451	A	C
NC_040271.1	23990723	G	C
NC_040271.1	24026712	A	G
NC_040271.1	24113192	A	T
NC_040271.1	24183001	T	C
NC_040271.1	24856096	T	C
NC_040271.1	24910341	A	G
NC_040271.1	24954007	A	T
NC_040271.1	24956895	T	G
NC_040271.1	24957674	T	C
NC_040271.1	24958085	T	C
NC_040271.1	24958669	G	C
NC_040271.1	24978789	A	G
NC_040271.1	25637970	T	C
NC_040271.1	25638227	T	C
NC_040271.1	25638693	C	T
NC_040271.1	25648110	A	G
NC_040271.1	25691097	A	G
NC_040271.1	25743574	T	C
NC_040271.1	25802172	A	G
NC_040271.1	25857370	C	T
NC_040271.1	25930538	C	T

NC_040271.1	25992279	G	A
NC_040271.1	26061865	G	A
NC_040271.1	26278876	A	G
NC_040271.1	26372004	C	A
NC_040271.1	26383367	C	G
NC_040271.1	26441807	C	T
NC_040271.1	26444216	T	C
NC_040271.1	26466787	T	C
NC_040271.1	26467348	T	C
NC_040271.1	26467539	A	G
NC_040271.1	26525835	C	A
NC_040271.1	26649886	T	C
NC_040271.1	26661306	C	T
NC_040271.1	26715486	G	T
NC_040271.1	26778456	T	C
NC_040271.1	26855536	T	C
NC_040271.1	26906517	A	G
NC_040271.1	26922279	T	C
NC_040271.1	26922450	A	G
NC_040271.1	27013295	C	T
NC_040271.1	27072866	T	G
NC_040271.1	27396743	A	G
NC_040271.1	27397574	A	C
NC_040271.1	27453242	C	T
NC_040271.1	27672779	G	A
NC_040271.1	28070612	G	A
NC_040271.1	28232295	A	T
NC_040271.1	28233199	T	G
NC_040271.1	28357719	C	T
NC_040271.1	28536787	G	A
NC_040271.1	28796755	T	C
NC_040271.1	28844604	A	G
NC_040271.1	28845475	G	C
NC_040271.1	28918794	A	G
NC_040271.1	28976534	T	A
NC_040271.1	29015336	G	A
NC_040271.1	29019561	A	G
NC_040271.1	29021946	A	G
NC_040271.1	29035036	T	C
NC_040271.1	29039458	A	G
NC_040271.1	29041070	T	G
NC_040271.1	29044873	G	C
NC_040271.1	29045258	A	G
NC_040271.1	29047206	0	A
NC_040271.1	29050227	T	C
NC_040271.1	29050404	A	G
NC_040271.1	29050850	T	C

NC_040271.1	29051007	T	C
NC_040271.1	29052201	C	G
NC_040271.1	29053181	T	C
NC_040271.1	29053441	C	T
NC_040271.1	29053996	C	T
NC_040271.1	29054193	A	G
NC_040271.1	29061083	G	A
NC_040271.1	29062292	A	T
NC_040271.1	29066569	C	T
NC_040271.1	29067079	C	T
NC_040271.1	29067257	T	C
NC_040271.1	29087299	A	G
NC_040271.1	29092062	T	C
NC_040271.1	29108444	T	G
NC_040271.1	29115799	A	G
NC_040271.1	29118782	A	G
NC_040271.1	29120055	T	C
NC_040271.1	29122362	A	G
NC_040271.1	29129809	T	C
NC_040271.1	29144959	T	C
NC_040271.1	29146925	T	C
NC_040271.1	29150316	C	T
NC_040271.1	29151449	A	G
NC_040271.1	29176168	T	C
NC_040271.1	29216503	A	G
NC_040271.1	29222910	C	T
NC_040271.1	29224835	A	G
NC_040271.1	29232581	C	T
NC_040271.1	29233308	G	A
NC_040271.1	29241658	A	G
NC_040271.1	29245220	A	G
NC_040271.1	29249401	G	A
NC_040271.1	29290364	C	G
NC_040271.1	29291003	0	T
NC_040271.1	29293795	A	G
NC_040271.1	29295710	T	C
NC_040271.1	29298547	T	C
NC_040271.1	29305232	T	C
NC_040271.1	29313219	C	T
NC_040271.1	29319772	A	G
NC_040271.1	29363366	A	G
NC_040271.1	29364687	A	G
NC_040271.1	29370396	T	C
NC_040271.1	29383696	T	C
NC_040271.1	29387065	A	G
NC_040271.1	29389485	T	C
NC_040271.1	29393540	G	A

NC_040271.1	29394780	A	T
NC_040271.1	29406114	G	A
NC_040271.1	29408487	G	T
NC_040271.1	29412310	T	C
NC_040271.1	29419207	T	C
NC_040271.1	29421784	T	C
NC_040271.1	29422210	C	T
NC_040271.1	29422399	A	G
NC_040271.1	29422563	A	G
NC_040271.1	29424990	A	G
NC_040271.1	29426220	G	A
NC_040271.1	29434268	T	C
NC_040271.1	29464246	T	C
NC_040271.1	29464815	A	G
NC_040271.1	29464981	T	C
NC_040271.1	29465981	A	G
NC_040271.1	29466138	T	C
NC_040271.1	29473288	A	G
NC_040271.1	29475238	A	G
NC_040271.1	29477516	A	G
NC_040271.1	29480424	C	T
NC_040271.1	29481168	A	C
NC_040271.1	29481688	T	C
NC_040271.1	29484043	T	C
NC_040271.1	29485523	A	C
NC_040271.1	29489927	T	C
NC_040271.1	29491532	T	C
NC_040271.1	29494605	A	G
NC_040271.1	29495079	A	G
NC_040271.1	29499325	A	C
NC_040271.1	29502818	T	C
NC_040271.1	29503073	T	C
NC_040271.1	29509026	T	G
NC_040271.1	29540337	A	G
NC_040271.1	29541831	T	C
NC_040271.1	29542020	T	C
NC_040271.1	29544255	G	C
NC_040271.1	29548591	A	G
NC_040271.1	29552793	T	C
NC_040271.1	29553052	T	C
NC_040271.1	29553250	A	G
NC_040271.1	29570540	C	G
NC_040271.1	29579989	T	C
NC_040271.1	29585110	C	T
NC_040271.1	29585896	G	C
NC_040271.1	29597195	G	C
NC_040271.1	29626520	C	A

NC_040271.1	29687067	A	G
NC_040271.1	29699202	A	T
NC_040271.1	29732706	G	A
NC_040271.1	29732861	G	A
NC_040271.1	29733632	T	C
NC_040271.1	29735269	T	C
NC_040271.1	29737754	T	C
NC_040271.1	29739706	A	G
NC_040271.1	29750858	T	C
NC_040271.1	29888283	A	G
NC_040271.1	29941949	G	C
NC_040271.1	29958456	A	G
NC_040271.1	29958638	T	C
NC_040271.1	29967940	A	G
NC_040271.1	30070157	T	G
NC_040271.1	30099763	G	A
NC_040271.1	30102349	G	C
NC_040271.1	30102524	T	C
NC_040271.1	30107906	C	T
NC_040271.1	30109951	A	G
NC_040271.1	30123774	A	G
NC_040271.1	30130797	T	C
NC_040271.1	30135254	C	T
NC_040271.1	30137373	T	C
NC_040271.1	30138778	A	C
NC_040271.1	30140763	A	G
NC_040271.1	30141545	T	C
NC_040271.1	30150478	T	A
NC_040271.1	30150673	A	C
NC_040271.1	30170772	A	C
NC_040271.1	30171042	A	G
NC_040271.1	30172939	A	G
NC_040271.1	30173851	C	T
NC_040271.1	30178415	T	C
NC_040271.1	30179968	A	G
NC_040271.1	30208526	T	C
NC_040271.1	30211944	T	C
NC_040271.1	30250661	T	C
NC_040271.1	30252623	C	T
NC_040271.1	30281947	C	G
NC_040271.1	30793841	T	C
NC_040271.1	30813200	T	C
NC_040271.1	30822390	G	A
NC_040271.1	30856498	C	T
NC_040271.1	30858936	T	C
NC_040271.1	30885475	G	T
NC_040271.1	30889548	T	C

NC_040271.1	30920611		0	C
NC_040271.1	30948512	T		C
NC_040271.1	31321231	G		C
NC_040271.1	32088599	A		C
NC_040271.1	32443565	T		C
NC_040271.1	32446643	G		A
NC_040271.1	32483970	A		G
NC_040271.1	32497177	C		T
NC_040271.1	32497841	G		A
NC_040271.1	32498258	A		C
NC_040271.1	32698580	C		T
NC_040271.1	32758046	A		G
NC_040271.1	32812208	T		C
NC_040271.1	32826619	A		G
NC_040271.1	32827911	A		T
NC_040271.1	32848657	C		G
NC_040271.1	32856030	G		A
NC_040271.1	32863117	T		C
NC_040271.1	32908206	T		C
NC_040271.1	32941374	C		G
NC_040271.1	32942229	C		T
NC_040271.1	32949056	A		T
NC_040271.1	32949342	T		C
NC_040271.1	32956878	G		A
NC_040271.1	32981344	C		T
NC_040271.1	32982948	C		T
NC_040271.1	32988845	C		T
NC_040271.1	32989085	C		T
NC_040271.1	32996984	A		G
NC_040271.1	33292166	A		G
NC_040271.1	33316116	T		C
NC_040271.1	33400186	C		A
NC_040271.1	33403036	A		G
NC_040271.1	33426511	G		A
NC_040271.1	33607599	A		G
NC_040271.1	33626015	C		T
NC_040271.1	33626881	C		T
NC_040271.1	33637535	A		G
NC_040271.1	33637758	A		G
NC_040271.1	33638090	C		T
NC_040271.1	33638819	T		G
NC_040271.1	33673254	C		T
NC_040271.1	33673546	G		A
NC_040271.1	33674857	A		G
NC_040271.1	33675847	T		C
NC_040271.1	33676006	C		T
NC_040271.1	33704594	G		A

NC_040271.1	34040342	C	A
NC_040271.1	34048321	A	G
NC_040271.1	34101975	G	C
NC_040271.1	34107726	C	T
NC_040271.1	34108115	A	G
NC_040271.1	34164655	T	C
NC_040271.1	34164864	G	A
NC_040271.1	34165715	G	A
NC_040271.1	34185204	G	A
NC_040271.1	34193686	T	G
NC_040271.1	34196262	T	G
NC_040271.1	34223191	G	A
NC_040271.1	34236748	G	A
NC_040271.1	34278334	C	T
NC_040271.1	34314303	G	A
NC_040271.1	34358752	C	T
NC_040271.1	34415738	C	A
NC_040271.1	34426705	T	A
NC_040271.1	34429029	T	C
NC_040271.1	34478571	A	G
NC_040271.1	34497054	A	G
NC_040271.1	34497232	A	G
NC_040271.1	34536529	A	G
NC_040271.1	34564674	C	T
NC_040271.1	34601812	C	T
NC_040271.1	34641765	G	A
NC_040271.1	34732327	T	C
NC_040271.1	34787991	A	G
NC_040271.1	34843795	A	G
NC_040271.1	34899020	A	C
NC_040271.1	34954479	G	A
NC_040271.1	35012426	G	T
NC_040271.1	35065093	T	C
NC_040271.1	35125810	C	T
NC_040271.1	35148826	C	T
NC_040271.1	35256045	T	C
NC_040271.1	35292120	A	G
NC_040271.1	35322752	A	C
NC_040271.1	35488990	G	A
NC_040271.1	35529638	T	C
NC_040271.1	35572071	T	C
NC_040271.1	35592155	G	A
NC_040271.1	35592519	T	C
NC_040271.1	35599588	G	A
NC_040271.1	35725021	A	C
NC_040271.1	35747075	T	C
NC_040271.1	35765545	C	T

NC_040271.1	35765703	T	C
NC_040271.1	35774914	T	C
NC_040271.1	35790566	T	C
NC_040271.1	35805286	A	G
NC_040271.1	35831515	C	A
NC_040271.1	35892722	G	A
NC_040271.1	35914847	C	T
NC_040271.1	35971149	T	C
NC_040271.1	36028929	A	C
NC_040271.1	36076222	C	G
NC_040271.1	36095291	A	G
NC_040271.1	37098710	C	T
NC_040271.1	37123390	T	C
NC_040271.1	38138541	A	T
NC_040271.1	38451383	C	T
NC_040271.1	38512641	T	C
NC_040271.1	38517428	T	C
NC_040271.1	38882414	A	G
NC_040271.1	39723784	C	T
NC_040271.1	39725213	T	C
NC_040271.1	39728117	A	G
NC_040271.1	40045289	T	C
NC_040271.1	40101758	A	G
NC_040271.1	40157695	A	C
NC_040271.1	40197508	G	A
NC_040271.1	40241082	C	T
NC_040271.1	40297231	C	T
NC_040271.1	40352948	A	G
NC_040271.1	40411256	C	T
NC_040271.1	40467373	T	C
NC_040271.1	40521256	C	A
NC_040271.1	40581283	T	C
NC_040271.1	40628271	T	C
NC_040271.1	40697818	G	T
NC_040271.1	40704068	G	A
NC_040271.1	40738798	C	G
NC_040271.1	40770936	T	C
NC_040271.1	40929507	T	G
NC_040271.1	40978757	G	A
NC_040271.1	41017536	T	C
NC_040271.1	41042388	A	G
NC_040271.1	41042630	G	A
NC_040271.1	42523774	A	G
NC_040271.1	42524788	T	C
NC_040271.1	42524994	G	A
NC_040271.1	42525243	A	G
NC_040271.1	42587818	G	A

NC_040271.1	42643526	A	G
NC_040271.1	42693358	T	C
NC_040271.1	42715531	T	G
NC_040271.1	42722304	T	C
NC_040271.1	42764141	T	A
NC_040271.1	42779719	A	G
NC_040271.1	42786972	C	G
NC_040271.1	42855466	T	G
NC_040271.1	42911630	A	T
NC_040271.1	42950294	A	G
NC_040271.1	42989691	C	T
NC_040271.1	43011032	T	C
NC_040271.1	43034616	A	G
NC_040271.1	43088502	C	A
NC_040271.1	43123550	C	T
NC_040271.1	43143078	C	T
NC_040271.1	43144912	C	A
NC_040271.1	43148325	T	G
NC_040271.1	43158057	T	G
NC_040271.1	43181226	G	A
NC_040271.1	43195027	T	C
NC_040271.1	43248094	A	G
NC_040271.1	43305692	G	A
NC_040271.1	43374663	A	G
NC_040271.1	43375079	A	G
NC_040271.1	43375538	A	G
NC_040271.1	43385711	G	A
NC_040271.1	43451485	C	T
NC_040271.1	43745467	T	G
NC_040271.1	43746855	G	A
NC_040271.1	43750644	T	C
NC_040271.1	43811039	G	A
NC_040271.1	43811998	A	G
NC_040271.1	43869462	T	C
NC_040271.1	43925986	T	G
NC_040271.1	43980503	C	A
NC_040271.1	44039490	T	G
NC_040271.1	44093083	T	C
NC_040271.1	44149915	A	G
NC_040271.1	44150638	T	C
NC_040271.1	44163461	A	G
NC_040271.1	44167214	G	C
NC_040271.1	44167652	C	G
NC_040271.1	44168404	G	A
NC_040271.1	44169779	T	C
NC_040271.1	44170530	T	C
NC_040271.1	44180713	A	G

NC_040271.1	44230304	C	A
NC_040271.1	44333127	G	C
NC_040271.1	44726942	A	T
NC_040271.1	44784368	A	G
NC_040271.1	44827854	T	C
NC_040271.1	44843122	A	G
NC_040271.1	44843370	T	C
NC_040271.1	44847094	A	G
NC_040271.1	44847258	T	C
NC_040271.1	44883205	G	A
NC_040271.1	44926622	A	C
NC_040271.1	44983166	T	C
NC_040271.1	45018799	T	C
NC_040271.1	45055644	G	C
NC_040271.1	46086056	T	C
NC_040271.1	46219767	C	G
NC_040271.1	46259474	T	G
NC_040271.1	46316257	C	T
NC_040271.1	46375775	T	A
NC_040271.1	46414793	T	C
NC_040271.1	46450850	A	G
NC_040271.1	46476058	C	G
NC_040271.1	46530217	T	C
NC_040271.1	46577747	A	G
NC_040271.1	46621637	C	G
NC_040271.1	46621882	T	C
NC_040271.1	46622302	C	A
NC_040271.1	46658550	T	C
NC_040271.1	46674432	T	C
NC_040271.1	46698615	A	G
NC_040271.1	46700248	T	C
NC_040271.1	46739617	A	G
NC_040271.1	46769571	A	G
NC_040271.1	46822486	A	G
NC_040271.1	46879999	G	A
NC_040271.1	46936070	A	G
NC_040271.1	47066443	T	C
NC_040271.1	47109616	A	G
NC_040271.1	47153832	T	G
NC_040271.1	47215440	G	C
NC_040271.1	47269728	A	T
NC_040271.1	47309546	T	C
NC_040271.1	47676869	C	T
NC_040271.1	47677437	A	G
NC_040271.1	47792473	A	G
NC_040271.1	47793461	C	T
NC_040271.1	47823722	C	T

NC_040271.1	47824209	T	C
NC_040271.1	47824745	T	C
NC_040271.1	47824919	C	A
NC_040271.1	47825336	T	C
NC_040271.1	47825702	T	G
NC_040271.1	47827979	G	C
NC_040271.1	47828267	A	G
NC_040271.1	47829029	T	C
NC_040271.1	47829289	T	C
NC_040271.1	47877042	C	T
NC_040271.1	47927863	A	G
NC_040271.1	48127281	T	C
NC_040271.1	48188174	T	C
NC_040271.1	48318022	A	G
NC_040271.1	48347029	A	G
NC_040271.1	48596083	C	T
NC_040271.1	48642551	G	A
NC_040271.1	48687086	T	G
NC_040271.1	48741881	T	C
NC_040271.1	48776784	A	G
NC_040271.1	48816227	C	T
NC_040271.1	48848860	G	A
NC_040271.1	48889280	A	G
NC_040271.1	48917223	A	G
NC_040271.1	48967660	A	C
NC_040271.1	49010867	A	G
NC_040271.1	49035032	T	C
NC_040271.1	49067609	C	T
NC_040271.1	49144085	A	T
NC_040271.1	49531532	C	T
NC_040271.1	49575172	T	C
NC_040271.1	49624871	T	C
NC_040271.1	49676791	C	T
NC_040271.1	49725524	T	C
NC_040271.1	50665559	A	G
NC_040271.1	50671162	C	T
NC_040271.1	50680922	T	C
NC_040271.1	50683619	C	G
NC_040271.1	50875855	A	C
NC_040271.1	50923254	C	T
NC_040271.1	50984183	T	C
NC_040271.1	51001047	A	G
NC_040271.1	51052665	T	C
NC_040271.1	51110086	T	C
NC_040271.1	51153466	T	C
NC_040271.1	51287289	C	T
NC_040271.1	51295540	G	A

NC_040271.1	51307593	A	G
NC_040271.1	51307923	T	C
NC_040271.1	51308427	A	G
NC_040271.1	51308706	A	G
NC_040271.1	51308865	G	A
NC_040271.1	51309159	G	A
NC_040271.1	51310440	A	G
NC_040271.1	51312073	T	C
NC_040271.1	51313021	A	G
NC_040271.1	51313432	A	G
NC_040271.1	51321833	A	G
NC_040271.1	51347522	G	C
NC_040271.1	51413933	T	C
NC_040271.1	51414282	A	G
NC_040271.1	51415367	T	G
NC_040271.1	51416487	T	C
NC_040271.1	51417490	T	C
NC_040271.1	51417648	T	C
NC_040271.1	51418555	A	G
NC_040271.1	51419014	C	A
NC_040271.1	51419292	G	A
NC_040271.1	51420743	T	C
NC_040271.1	51438589	G	A
NC_040271.1	51443130	A	G
NC_040271.1	51443283	A	G
NC_040271.1	51455530	A	G
NC_040271.1	51476935	T	C
NC_040271.1	51484130	G	C
NC_040271.1	51499137	A	G
NC_040271.1	51499657	C	T
NC_040271.1	51500450	G	A
NC_040271.1	51511905	C	A
NC_040271.1	51542748	G	T
NC_040271.1	51543881	T	C
NC_040271.1	51588416	A	G
NC_040271.1	51588623	T	C
NC_040271.1	51589045	T	C
NC_040271.1	51589911	A	G
NC_040271.1	51590355	T	C
NC_040271.1	51590573	A	G
NC_040271.1	51591034	C	G
NC_040271.1	51998461	A	G
NC_040271.1	52034667	T	C
NC_040271.1	52116834	A	G
NC_040271.1	52144214	T	C
NC_040271.1	52250092	G	C
NC_040271.1	52306311	A	G

NC_040271.1	52348246	G	T
NC_040271.1	52451193	A	G
NC_040271.1	52457848	A	C
NC_040271.1	52582650	G	A
NC_040271.1	52639990	A	T
NC_040271.1	52701004	C	T
NC_040271.1	52759785	G	A
NC_040271.1	52832412	C	T
NC_040271.1	52924044	T	C
NC_040271.1	52966571	G	A
NC_040271.1	52974920	T	C
NC_040271.1	52975152	C	A
NC_040271.1	52975391	C	T
NC_040271.1	52975674	A	T
NC_040271.1	52976616	C	T
NC_040271.1	52976828	G	T
NC_040271.1	52977343	T	C
NC_040271.1	52977577	C	G
NC_040271.1	52978861	A	C
NC_040271.1	52979012	T	C
NC_040271.1	52979847	A	G
NC_040271.1	53012098	A	G
NC_040271.1	53041097	C	T
NC_040271.1	53101754	G	A
NC_040271.1	53131367	C	G
NC_040271.1	53477984	T	C
NC_040271.1	53506791	T	C
NC_040271.1	53513975	A	G
NC_040271.1	53518220	T	C
NC_040271.1	53518615	A	G
NC_040271.1	53519037	G	A
NC_040271.1	53520051	G	A
NC_040271.1	53520232	T	G
NC_040271.1	53537158	C	T
NC_040271.1	53717302	A	C
NC_040271.1	53835676	A	G
NC_040271.1	53891026	C	T
NC_040271.1	53929006	T	C
NC_040271.1	53961270	T	C
NC_040271.1	53962688	C	T
NC_040271.1	53963194	A	G
NC_040271.1	53964371	G	A
NC_040271.1	53968560	A	G
NC_040271.1	54033023	T	C
NC_040271.1	54055565	G	A
NC_040271.1	54056287	C	T
NC_040271.1	54058480	G	T

NC_040271.1	54082934	C	G
NC_040271.1	54130082	A	C
NC_040271.1	54229942	T	G
NC_040271.1	54285588	T	C
NC_040271.1	54310255	T	G
NC_040271.1	54316926	G	A
NC_040271.1	54334846	T	G
NC_040271.1	54371875	C	T
NC_040271.1	54407589	T	C
NC_040271.1	54407778	A	C
NC_040271.1	54429538	C	G
NC_040271.1	54649344	C	T
NC_040271.1	54707068	A	G
NC_040271.1	54763168	T	A
NC_040271.1	54820483	A	G
NC_040271.1	54877554	G	A
NC_040271.1	54933974	C	G
NC_040271.1	54990120	T	C
NC_040271.1	55028898	T	C
NC_040271.1	55063949	G	A
NC_040271.1	55162367	T	C
NC_040271.1	55538566	G	A
NC_040271.1	55577324	T	C
NC_040271.1	55635433	G	A
NC_040271.1	55675757	T	C
NC_040271.1	55720126	T	C
NC_040271.1	55722906	A	G
NC_040271.1	55766552	A	G
NC_040271.1	55767319	T	C
NC_040271.1	55767558	T	C
NC_040271.1	55771066	A	G
NC_040271.1	55809566	A	G
NC_040272.1	114438	A	G
NC_040272.1	115079	C	T
NC_040272.1	115833	T	C
NC_040272.1	115996	T	C
NC_040272.1	166205	T	C
NC_040272.1	174436	G	A
NC_040272.1	202190	G	A
NC_040272.1	354393	T	C
NC_040272.1	616177	C	G
NC_040272.1	642667	A	G
NC_040272.1	682455	G	A
NC_040272.1	695926	C	A
NC_040272.1	705696	G	A
NC_040272.1	713750	T	C
NC_040272.1	742140	T	A

NC_040272.1	757807	C	T
NC_040272.1	758001	T	C
NC_040272.1	759650	T	G
NC_040272.1	768137	C	G
NC_040272.1	779492	C	T
NC_040272.1	1210609	G	A
NC_040272.1	1243364	A	T
NC_040272.1	1297496	A	C
NC_040272.1	1333295	C	G
NC_040272.1	1540718	A	G
NC_040272.1	1541989	A	C
NC_040272.1	1542192	T	A
NC_040272.1	1697983	C	G
NC_040272.1	1702776	A	C
NC_040272.1	1783348	G	C
NC_040272.1	1783808	C	T
NC_040272.1	1785384	T	C
NC_040272.1	1786222	T	C
NC_040272.1	1786534	A	C
NC_040272.1	1825297	T	C
NC_040272.1	2051848	T	C
NC_040272.1	2283311	C	T
NC_040272.1	2338591	C	A
NC_040272.1	2400293	G	C
NC_040272.1	2470530	G	T
NC_040272.1	2521061	A	G
NC_040272.1	2668339	C	T
NC_040272.1	2731993	A	C
NC_040272.1	2780216	C	T
NC_040272.1	2814826	A	G
NC_040272.1	2870121	T	C
NC_040272.1	2925027	G	A
NC_040272.1	2954214	A	G
NC_040272.1	3011507	C	T
NC_040272.1	3063107	T	C
NC_040272.1	3121778	C	T
NC_040272.1	3122279	G	A
NC_040272.1	5794318	G	A
NC_040272.1	5815080	C	T
NC_040272.1	5889467	G	T
NC_040272.1	5939623	C	T
NC_040272.1	6688689	A	G
NC_040272.1	6870908	T	C
NC_040272.1	6888654	C	T
NC_040272.1	7060487	G	A
NC_040272.1	7113264	G	A
NC_040272.1	7180896	T	G

NC_040272.1	7232813	A	T
NC_040272.1	7233039	A	C
NC_040272.1	7296041	T	C
NC_040272.1	7354083	T	G
NC_040272.1	7379387	A	G
NC_040272.1	7379657	G	C
NC_040272.1	7523796	T	C
NC_040272.1	7591238	G	A
NC_040272.1	7658672	A	G
NC_040272.1	7712810	T	A
NC_040272.1	7763032	G	A
NC_040272.1	7814856	T	C
NC_040272.1	7882917	A	T
NC_040272.1	7941446	C	T
NC_040272.1	8022235	C	T
NC_040272.1	8080788	G	T
NC_040272.1	8123615	A	G
NC_040272.1	8159345	T	C
NC_040272.1	8159549	A	C
NC_040272.1	8335902	T	C
NC_040272.1	8342691	G	A
NC_040272.1	8360514	A	G
NC_040272.1	8374692	T	G
NC_040272.1	8377623	T	C
NC_040272.1	8468782	C	G
NC_040272.1	8533244	T	C
NC_040272.1	9236737	G	A
NC_040272.1	9238902	A	G
NC_040272.1	9241649	C	T
NC_040272.1	9301652	T	C
NC_040272.1	9366407	T	C
NC_040272.1	9422469	C	T
NC_040272.1	9465522	C	A
NC_040272.1	9505339	T	C
NC_040272.1	9585565	C	T
NC_040272.1	9586429	T	C
NC_040272.1	9587386	C	G
NC_040272.1	9587840	A	C
NC_040272.1	9588286	T	C
NC_040272.1	9589065	A	T
NC_040272.1	9591145	A	G
NC_040272.1	9709409	T	C
NC_040272.1	9726268	C	T
NC_040272.1	9876506	G	A
NC_040272.1	9931703	A	C
NC_040272.1	9993008	T	C
NC_040272.1	10048282	G	A

NC_040272.1	10098798	T	C
NC_040272.1	10126469	C	T
NC_040272.1	10178829	C	T
NC_040272.1	10234046	C	A
NC_040272.1	10251925	C	T
NC_040272.1	10488604	A	T
NC_040272.1	10536192	T	C
NC_040272.1	10568254	A	G
NC_040272.1	10582295	T	C
NC_040272.1	10583779	G	C
NC_040272.1	10608214	G	A
NC_040272.1	10612383	A	G
NC_040272.1	10707902	G	C
NC_040272.1	10765127	T	C
NC_040272.1	10796509	A	C
NC_040272.1	10798760	A	G
NC_040272.1	10799044	G	A
NC_040272.1	10804301	C	A
NC_040272.1	10827155	T	C
NC_040272.1	10834074	G	A
NC_040272.1	10834811	C	T
NC_040272.1	10854241	G	A
NC_040272.1	10854396	G	A
NC_040272.1	10856638	T	C
NC_040272.1	10857972	G	A
NC_040272.1	10858536	T	C
NC_040272.1	10890750	T	A
NC_040272.1	10892552	T	G
NC_040272.1	10898075	G	A
NC_040272.1	10898651	G	A
NC_040272.1	10976304	T	C
NC_040272.1	11039262	T	C
NC_040272.1	11096382	A	G
NC_040272.1	11158733	T	C
NC_040272.1	11221085	A	G
NC_040272.1	11274643	A	G
NC_040272.1	11346696	T	G
NC_040272.1	11409775	A	G
NC_040272.1	11457941	C	T
NC_040272.1	11504726	T	C
NC_040272.1	11570650	G	T
NC_040272.1	11637205	C	T
NC_040272.1	11702521	C	A
NC_040272.1	11771431	A	C
NC_040272.1	11840288	A	G
NC_040272.1	11889415	A	T
NC_040272.1	11946785	T	C

NC_040272.1	12009561	G	A
NC_040272.1	12067143	A	G
NC_040272.1	12126304	T	C
NC_040272.1	12171782	T	C
NC_040272.1	12241925	G	T
NC_040272.1	12301500	G	A
NC_040272.1	12363216	C	A
NC_040272.1	12417914	T	C
NC_040272.1	12470735	G	A
NC_040272.1	12549363	G	A
NC_040272.1	12600582	C	G
NC_040272.1	12631828	G	A
NC_040272.1	12863963	A	G
NC_040272.1	13311109	A	G
NC_040272.1	13370454	T	C
NC_040272.1	13431289	A	C
NC_040272.1	13502691	C	T
NC_040272.1	13563125	G	C
NC_040272.1	13620114	G	A
NC_040272.1	13681130	G	A
NC_040272.1	13742871	A	G
NC_040272.1	13799385	A	G
NC_040272.1	13862983	A	T
NC_040272.1	13893130	C	A
NC_040272.1	13894675	A	G
NC_040272.1	13896401	C	T
NC_040272.1	14029420	A	G
NC_040272.1	14048842	C	T
NC_040272.1	14057389	C	A
NC_040272.1	14057601	T	C
NC_040272.1	14061720	T	C
NC_040272.1	14113825	G	A
NC_040272.1	14114427	A	G
NC_040272.1	14133237	A	G
NC_040272.1	14133408	T	C
NC_040272.1	14133801	G	A
NC_040272.1	14136374	C	A
NC_040272.1	14136604	C	T
NC_040272.1	14224150	G	A
NC_040272.1	14280785	G	A
NC_040272.1	14318532	T	C
NC_040272.1	14318797	T	C
NC_040272.1	14358095	T	C
NC_040272.1	14358374	C	T
NC_040272.1	14358543	T	C
NC_040272.1	14358723	T	C
NC_040272.1	14358992	C	T

NC_040272.1	14431299	G	A
NC_040272.1	14432731	G	A
NC_040272.1	14452913	C	T
NC_040272.1	14562327	C	G
NC_040272.1	18455245	A	G
NC_040272.1	18522657	G	T
NC_040272.1	18592556	A	G
NC_040272.1	18655874	G	C
NC_040272.1	18687860	T	C
NC_040272.1	18750290	A	G
NC_040272.1	18816446	C	G
NC_040272.1	18873291	C	T
NC_040272.1	18930935	T	C
NC_040272.1	18966701	T	A
NC_040272.1	19026935	A	G
NC_040272.1	19083534	T	C
NC_040272.1	19121455	C	G
NC_040272.1	19168908	T	C
NC_040272.1	19231510	C	A
NC_040272.1	19301109	T	C
NC_040272.1	19344417	G	A
NC_040272.1	19345013	A	G
NC_040272.1	19406070	G	C
NC_040272.1	19458015	C	T
NC_040272.1	19487223	A	G
NC_040272.1	19489646	T	C
NC_040272.1	19502810	C	G
NC_040272.1	19502987	G	A
NC_040272.1	19503935	C	T
NC_040272.1	19575085	T	C
NC_040272.1	19577072	C	A
NC_040272.1	19628755	A	G
NC_040272.1	19649458	T	C
NC_040272.1	19721737	A	G
NC_040272.1	19781001	G	A
NC_040272.1	19831935	T	C
NC_040272.1	19832212	C	A
NC_040272.1	19832386	T	G
NC_040272.1	19833505	T	C
NC_040272.1	19833988	C	T
NC_040272.1	19834598	T	C
NC_040272.1	19950109	G	A
NC_040272.1	19987706	G	A
NC_040272.1	20009996	G	A
NC_040272.1	20014783	G	A
NC_040272.1	20016377	A	G
NC_040272.1	20017041	T	C

NC_040272.1	20017525	T	G
NC_040272.1	20018059	C	T
NC_040272.1	20073461	C	T
NC_040272.1	20204054	T	C
NC_040272.1	20257735	T	G
NC_040272.1	20285422	T	C
NC_040272.1	20302005	A	G
NC_040272.1	20362192	A	C
NC_040272.1	20504007	T	A
NC_040272.1	20571759	A	G
NC_040272.1	20629889	G	T
NC_040272.1	20745876	T	C
NC_040272.1	20804290	C	T
NC_040272.1	20856475	C	G
NC_040272.1	20912248	A	T
NC_040272.1	20938976	T	A
NC_040272.1	20990563	G	T
NC_040272.1	21023576	T	C
NC_040272.1	21968320	C	T
NC_040272.1	22031682	T	G
NC_040272.1	22098359	A	G
NC_040272.1	22156238	A	G
NC_040272.1	22218307	G	A
NC_040272.1	22265276	G	A
NC_040272.1	22326479	G	A
NC_040272.1	22394193	T	C
NC_040272.1	22456586	T	C
NC_040272.1	22483819	G	T
NC_040272.1	23613389	G	T
NC_040272.1	23665140	A	C
NC_040272.1	23731657	A	G
NC_040272.1	23782742	T	C
NC_040272.1	23825553	T	A
NC_040272.1	23883530	A	G
NC_040272.1	23941415	C	T
NC_040272.1	24020421	C	T
NC_040272.1	24072676	G	A
NC_040272.1	24118757	T	C
NC_040272.1	24189402	C	T
NC_040272.1	24264300	T	C
NC_040272.1	24322361	C	T
NC_040272.1	24358610	T	C
NC_040272.1	24529251	G	C
NC_040272.1	24593562	A	G
NC_040272.1	24643857	G	C
NC_040272.1	24686442	G	A
NC_040272.1	24730748	T	C

NC_040272.1	24772709	A	G
NC_040272.1	24805041	G	A
NC_040272.1	24858195	T	G
NC_040272.1	24920628	G	T
NC_040272.1	24978789	C	T
NC_040272.1	25010738	G	A
NC_040272.1	25193485	C	T
NC_040272.1	25194531	C	A
NC_040272.1	25194835	C	G
NC_040272.1	25195071	A	G
NC_040272.1	25195285	T	C
NC_040272.1	25238838	T	A
NC_040272.1	25262672	T	C
NC_040272.1	25296961	T	C
NC_040272.1	25303177	T	C
NC_040272.1	25306748	T	C
NC_040272.1	25306986	A	G
NC_040272.1	25363508	A	G
NC_040272.1	25415496	G	C
NC_040272.1	25468977	C	T
NC_040272.1	25531757	C	T
NC_040272.1	25588113	C	G
NC_040272.1	25659704	A	G
NC_040272.1	26129957	T	C
NC_040272.1	26139479	T	C
NC_040272.1	26144484	A	G
NC_040272.1	26185095	T	C
NC_040272.1	26200783	T	C
NC_040272.1	26229756	A	G
NC_040272.1	26322521	A	T
NC_040272.1	26332447	G	A
NC_040272.1	26422329	G	A
NC_040272.1	26469775	G	C
NC_040272.1	26492975	C	T
NC_040272.1	26494745	A	G
NC_040272.1	26497761	T	G
NC_040272.1	26497958	T	C
NC_040272.1	26553180	A	G
NC_040272.1	26553333	T	C
NC_040272.1	26554531	C	T
NC_040272.1	26554943	A	G
NC_040272.1	26621766	G	A
NC_040272.1	26689203	G	C
NC_040272.1	26689510	A	G
NC_040272.1	26689730	T	C
NC_040272.1	26767316	G	C
NC_040272.1	26788699	A	G

NC_040272.1	26808003	A	G
NC_040272.1	26814433	G	A
NC_040272.1	26823045	T	G
NC_040272.1	26825623	G	A
NC_040272.1	26899913	C	T
NC_040272.1	26900088	G	A
NC_040272.1	27072118	A	G
NC_040272.1	27072445	T	A
NC_040272.1	27271401	G	A
NC_040272.1	27402066	A	G
NC_040272.1	27405725	T	G
NC_040272.1	27415315	C	T
NC_040272.1	27459140	T	C
NC_040272.1	27582327	G	A
NC_040272.1	29089311	C	A
NC_040272.1	29108223	A	C
NC_040272.1	29123021	A	G
NC_040272.1	29163875	T	C
NC_040272.1	29225089	T	C
NC_040272.1	29230850	T	C
NC_040272.1	29241030	G	T
NC_040272.1	29241897	A	G
NC_040272.1	29270482	A	G
NC_040272.1	29270690	C	A
NC_040272.1	29322361	T	C
NC_040272.1	29324995	C	T
NC_040272.1	29329525	T	C
NC_040272.1	29332465	A	G
NC_040272.1	29333658	C	T
NC_040272.1	29333825	G	A
NC_040272.1	29344298	A	G
NC_040272.1	29377234	G	C
NC_040272.1	29422247	C	A
NC_040272.1	29425894	C	G
NC_040272.1	29428056	C	A
NC_040272.1	29428565	C	G
NC_040272.1	29441190	A	G
NC_040272.1	29443958	A	G
NC_040272.1	29516057	T	C
NC_040272.1	29595249	T	C
NC_040272.1	29671080	G	A
NC_040272.1	29728270	A	G
NC_040272.1	29786386	T	G
NC_040272.1	29843533	G	A
NC_040272.1	29887552	C	T
NC_040272.1	29887900	A	G
NC_040272.1	29901286	A	G

NC_040272.1	29912012	A	G
NC_040272.1	29941433	T	G
NC_040272.1	30006544	G	A
NC_040272.1	30008443	T	C
NC_040272.1	30014467	A	G
NC_040272.1	30025547	C	T
NC_040272.1	30035421	G	T
NC_040272.1	30036427	T	A
NC_040272.1	30042169	A	G
NC_040272.1	30046379	G	T
NC_040272.1	30047592	G	A
NC_040272.1	30059059	C	A
NC_040272.1	30097596	T	C
NC_040272.1	30211881	T	C
NC_040272.1	30256505	A	T
NC_040272.1	30256720	T	C
NC_040272.1	30260199	C	A
NC_040272.1	30260718	G	A
NC_040272.1	30263051	C	T
NC_040272.1	30263207	G	A
NC_040272.1	30263765	C	T
NC_040272.1	30272509	A	G
NC_040272.1	30329859	A	G
NC_040272.1	30352596	T	C
NC_040272.1	30390061	G	A
NC_040272.1	30392748	C	T
NC_040272.1	30427437	T	C
NC_040272.1	30556341	C	T
NC_040272.1	30558969	A	G
NC_040272.1	30559158	T	C
NC_040272.1	30605911	T	C
NC_040272.1	30618855	T	C
NC_040272.1	30645704	T	C
NC_040272.1	30645926	G	C
NC_040272.1	30652754	C	T
NC_040272.1	30682134	C	T
NC_040272.1	30736204	C	G
NC_040272.1	30746965	T	C
NC_040272.1	30747496	T	G
NC_040272.1	30757029	G	A
NC_040272.1	30758404	T	C
NC_040272.1	32882133	T	C
NC_040272.1	32883522	C	T
NC_040272.1	32883844	G	A
NC_040272.1	32940467	T	C
NC_040272.1	32990823	T	C
NC_040272.1	33124422	G	C

NC_040272.1	33203128	T	C
NC_040272.1	33247012	C	G
NC_040272.1	33282941	C	T
NC_040272.1	33315584	T	A
NC_040272.1	33353321	C	A
NC_040272.1	33353513	A	G
NC_040272.1	33357761	A	G
NC_040272.1	33361209	T	C
NC_040272.1	33404932	T	G
NC_040272.1	33407935	T	C
NC_040272.1	33408094	C	T
NC_040272.1	33411823	A	G
NC_040272.1	33471945	T	G
NC_040272.1	33528881	A	G
NC_040272.1	33678151	G	A
NC_040272.1	33753621	T	G
NC_040272.1	34070279	C	T
NC_040272.1	34070520	C	G
NC_040272.1	34073575	C	T
NC_040272.1	34099820	T	C
NC_040272.1	34194403	C	T
NC_040272.1	34202978	C	T
NC_040272.1	34204672	T	C
NC_040272.1	34214220	T	G
NC_040272.1	34217203	A	G
NC_040272.1	34220579	T	C
NC_040272.1	34225024	A	G
NC_040272.1	34225735	T	C
NC_040272.1	34238934	G	A
NC_040272.1	34239504	G	T
NC_040272.1	34239716	A	G
NC_040272.1	35055836	C	G
NC_040272.1	35120053	A	C
NC_040272.1	35185884	G	A
NC_040272.1	35256688	A	C
NC_040272.1	35325531	T	A
NC_040272.1	35387550	C	T
NC_040272.1	35451911	T	C
NC_040272.1	35511222	A	G
NC_040272.1	35554841	A	G
NC_040272.1	35634334	T	C
NC_040272.1	35694449	G	T
NC_040272.1	35757614	C	T
NC_040272.1	35832508	G	A
NC_040272.1	35895168	T	C
NC_040272.1	35952227	G	A
NC_040272.1	36003011	T	C

NC_040272.1	36048525	C	T
NC_040272.1	36048701	C	A
NC_040272.1	36104066	G	A
NC_040272.1	36155490	G	A
NC_040272.1	36217415	A	C
NC_040272.1	36283017	C	T
NC_040272.1	36342489	A	T
NC_040272.1	36401395	C	T
NC_040272.1	36457236	T	C
NC_040272.1	36516211	C	T
NC_040272.1	36586698	T	C
NC_040272.1	37079787	A	G
NC_040272.1	37089194	T	C
NC_040272.1	37103329	T	C
NC_040272.1	37148121	C	G
NC_040272.1	37169919	T	G
NC_040272.1	37240646	C	A
NC_040272.1	37297077	C	T
NC_040272.1	37338599	A	C
NC_040272.1	37359414	A	T
NC_040272.1	37400255	G	A
NC_040272.1	37401716	A	T
NC_040272.1	37401897	A	G
NC_040272.1	37413401	T	C
NC_040272.1	37418201	C	G
NC_040272.1	37431217	G	A
NC_040272.1	37431462	A	G
NC_040272.1	37431676	C	T
NC_040272.1	37433244	A	G
NC_040272.1	37437514	T	C
NC_040272.1	37488682	A	G
NC_040272.1	37542621	T	C
NC_040272.1	37544523	A	G
NC_040272.1	37558738	T	C
NC_040272.1	37559201	A	C
NC_040272.1	37584712	A	G
NC_040272.1	37585552	T	C
NC_040272.1	37586281	T	G
NC_040272.1	37598205	T	C
NC_040272.1	37968962	T	C
NC_040272.1	37999348	T	C
NC_040272.1	37999658	C	G
NC_040272.1	37999915	C	T
NC_040272.1	38000203	G	A
NC_040272.1	38094598	A	G
NC_040272.1	38102442	A	G
NC_040272.1	38112047	T	C

NC_040272.1	38135673	A	G
NC_040272.1	38142672	C	T
NC_040272.1	38148706	T	C
NC_040272.1	38165787	C	T
NC_040272.1	38166278	T	C
NC_040272.1	38191169	C	T
NC_040272.1	38226391	A	G
NC_040272.1	38226574	T	C
NC_040272.1	38226816	T	C
NC_040272.1	38300021	T	C
NC_040272.1	38306363	T	C
NC_040272.1	38309499	T	C
NC_040272.1	38310023	T	C
NC_040272.1	38314184	T	C
NC_040272.1	38322984	T	C
NC_040272.1	38326860	A	G
NC_040272.1	38327107	T	C
NC_040272.1	38392967	T	C
NC_040272.1	38463806	G	A
NC_040272.1	38498230	C	G
NC_040272.1	38554555	T	C
NC_040272.1	38603983	G	T
NC_040272.1	38669167	T	C
NC_040272.1	38705321	T	C
NC_040272.1	38834343	T	C
NC_040272.1	38838066	A	T
NC_040272.1	39038154	G	A
NC_040272.1	39330917	C	T
NC_040272.1	39402536	T	C
NC_040272.1	39525771	C	G
NC_040272.1	39587452	C	T
NC_040272.1	39649755	G	A
NC_040272.1	39690343	T	A
NC_040272.1	40304077	G	T
NC_040272.1	40639915	A	G
NC_040272.1	41274314	G	A
NC_040272.1	41286282	A	G
NC_040272.1	41424548	A	G
NC_040272.1	41504129	T	G
NC_040272.1	41618217	G	A
NC_040272.1	41619958	G	A
NC_040272.1	41670251	G	A
NC_040272.1	41704244	A	G
NC_040272.1	41704438	A	G
NC_040272.1	41712006	G	A
NC_040272.1	41715429	A	G
NC_040272.1	41730331	G	A

NC_040272.1	41734047	T	C
NC_040272.1	41741886	G	A
NC_040272.1	41744127	A	G
NC_040272.1	41744505	T	C
NC_040272.1	41752753	T	C
NC_040272.1	41773637	C	G
NC_040272.1	41782989	C	T
NC_040272.1	41813156	T	C
NC_040272.1	41861412	T	C
NC_040272.1	41862054	G	T
NC_040272.1	41862206	G	A
NC_040272.1	41867783	A	C
NC_040272.1	41869540	T	C
NC_040272.1	41890238	A	G
NC_040272.1	41928999	T	G
NC_040272.1	42048037	A	G
NC_040272.1	42074375	A	G
NC_040272.1	42075833	G	A
NC_040272.1	42106889	C	T
NC_040272.1	42108058	G	A
NC_040272.1	42114267	G	A
NC_040272.1	42125202	G	A
NC_040272.1	42125735	C	T
NC_040272.1	42125944	G	A
NC_040272.1	42128710	C	T
NC_040272.1	42128877	T	C
NC_040272.1	42129058	G	C
NC_040272.1	42139210	T	C
NC_040272.1	42239910	A	C
NC_040272.1	42273809	A	G
NC_040272.1	42319585	A	G
NC_040272.1	42359237	A	G
NC_040272.1	42489372	A	G
NC_040272.1	42490867	C	A
NC_040272.1	42491023	T	A
NC_040272.1	42562043	A	G
NC_040272.1	42576694	A	G
NC_040272.1	42634811	A	G
NC_040272.1	42634982	A	G
NC_040272.1	42740733	A	C
NC_040272.1	42741762	T	C
NC_040272.1	42742056	A	G
NC_040272.1	42742211	C	G
NC_040272.1	42742922	C	A
NC_040272.1	42743973	T	C
NC_040272.1	42745551	A	G
NC_040272.1	42748839	G	A

NC_040272.1	42749218	T	A
NC_040272.1	42753722	T	G
NC_040272.1	42756077	G	A
NC_040272.1	42756835	A	G
NC_040272.1	42757035	C	T
NC_040272.1	42757323	T	C
NC_040272.1	42757724	C	T
NC_040272.1	42757923	T	C
NC_040272.1	42791475	A	C
NC_040272.1	42794262	G	T
NC_040272.1	42802267	A	G
NC_040272.1	42806016	A	G
NC_040272.1	42810612	T	C
NC_040272.1	42814503	T	C
NC_040272.1	42814749	A	G
NC_040272.1	42820667	A	G
NC_040272.1	42821022	T	C
NC_040272.1	42821177	G	C
NC_040272.1	42822046	T	C
NC_040272.1	42822330	T	C
NC_040272.1	42842823	G	C
NC_040272.1	42846979	C	G
NC_040272.1	42847286	G	T
NC_040272.1	42847580	T	C
NC_040272.1	42847866	C	T
NC_040272.1	42849005	G	T
NC_040272.1	42849524	T	C
NC_040272.1	42862290	A	C
NC_040272.1	42878287	A	G
NC_040272.1	42879652	C	G
NC_040272.1	42880105	T	C
NC_040272.1	42888342	A	G
NC_040272.1	42890670	T	C
NC_040272.1	42891663	T	C
NC_040272.1	42892117	T	C
NC_040272.1	42895457	C	G
NC_040272.1	42896488	T	C
NC_040272.1	42906963	G	A
NC_040272.1	42907376	A	C
NC_040272.1	42959476	T	C
NC_040272.1	42960020	T	G
NC_040272.1	42960335	G	C
NC_040272.1	42962491	A	G
NC_040272.1	42963063	T	G
NC_040272.1	42963933	T	C
NC_040272.1	42966502	T	C
NC_040272.1	43005235	T	G

NC_040272.1	43008414	T	C
NC_040272.1	43023219	C	T
NC_040272.1	43023398	T	C
NC_040272.1	43051980	A	G
NC_040272.1	43054444	G	C
NC_040272.1	43079802	G	C
NC_040272.1	43080162	T	C
NC_040272.1	43080699	T	C
NC_040272.1	43081152	T	C
NC_040272.1	43143119	C	T
NC_040272.1	43143339	T	C
NC_040272.1	43179433	T	C
NC_040272.1	43268771	C	T
NC_040272.1	43338829	T	C
NC_040272.1	43396137	A	G
NC_040272.1	43552746	C	T
NC_040272.1	43561544	G	A
NC_040272.1	43629441	T	C
NC_040272.1	43684647	G	A
NC_040272.1	43684838	G	A
NC_040272.1	43687967	A	G
NC_040272.1	43693796	A	G
NC_040272.1	43721511	A	G
NC_040272.1	43765185	G	C
NC_040272.1	43804426	C	T
NC_040272.1	43844686	C	T
NC_040272.1	43852727	A	C
NC_040272.1	43910676	T	C
NC_040272.1	43912726	T	C
NC_040272.1	43914360	A	G
NC_040272.1	43919116	T	C
NC_040272.1	43919396	A	G
NC_040272.1	43972449	C	T
NC_040272.1	43973468	C	T
NC_040272.1	43982694	T	C
NC_040272.1	43982913	G	A
NC_040272.1	44004949	T	C
NC_040272.1	44006842	T	C
NC_040272.1	44029155	T	C
NC_040272.1	44059368	T	C
NC_040272.1	44098016	T	C
NC_040272.1	44099234	T	C
NC_040272.1	44126777	A	G
NC_040272.1	44186215	T	C
NC_040272.1	44238634	T	C
NC_040272.1	44239868	C	G
NC_040272.1	44270237	T	G

NC_040272.1	44281640	T	C
NC_040272.1	44281808	G	T
NC_040272.1	44311296	A	G
NC_040272.1	44329692	A	G
NC_040272.1	44331872	A	G
NC_040272.1	44333395	T	C
NC_040272.1	44336731	T	C
NC_040272.1	44336972	C	G
NC_040272.1	44341551	A	G
NC_040272.1	44368059	T	C
NC_040272.1	44369210	G	A
NC_040272.1	44373431	G	A
NC_040272.1	44389478	G	C
NC_040272.1	44393956	A	G
NC_040272.1	44400953	C	A
NC_040272.1	44401261	C	A
NC_040272.1	44405292	A	G
NC_040272.1	44410885	C	T
NC_040272.1	44413863	A	G
NC_040272.1	44431576	T	C
NC_040272.1	44433495	A	C
NC_040272.1	44436293	A	G
NC_040272.1	44436762	A	G
NC_040272.1	44441439	A	G
NC_040272.1	44449986	T	C
NC_040272.1	44451791	T	C
NC_040272.1	44600742	A	G
NC_040272.1	44645292	G	A
NC_040272.1	44649967	C	G
NC_040272.1	44658010	A	G
NC_040272.1	44672868	A	G
NC_040272.1	44680841	A	G
NC_040272.1	44693027	T	C
NC_040272.1	44701690	T	C
NC_040272.1	44710008	A	G
NC_040272.1	44710457	T	C
NC_040272.1	44732045	T	C
NC_040272.1	44772446	A	G
NC_040272.1	44786744	A	G
NC_040272.1	44790118	C	T
NC_040272.1	44793922	G	A
NC_040272.1	44804987	C	A
NC_040272.1	44808673	T	C
NC_040272.1	44828030	T	C
NC_040272.1	44831225	G	A
NC_040272.1	44843068	G	A
NC_040272.1	44843277	A	G

NC_040272.1	44859602	T	C
NC_040272.1	44860391	G	A
NC_040272.1	44877485	T	C
NC_040272.1	44883955	G	T
NC_040272.1	44884175	A	G
NC_040272.1	44885047	T	C
NC_040272.1	44885496	G	A
NC_040272.1	44928945	A	G
NC_040272.1	44934996	C	T
NC_040272.1	44950897	T	C
NC_040272.1	44955091	G	A
NC_040272.1	44956672	T	C
NC_040272.1	44965162	C	G
NC_040272.1	45009515	T	C
NC_040272.1	45015596	T	C
NC_040272.1	45057647	C	A
NC_040272.1	45057884	C	T
NC_040272.1	45077283	A	G
NC_040272.1	45096875	A	C
NC_040272.1	45097695	C	T
NC_040272.1	45119755	A	G
NC_040272.1	45124577	T	C
NC_040272.1	45133401	G	C
NC_040272.1	45136144	G	A
NC_040272.1	45184134	T	G
NC_040272.1	45200765	G	A
NC_040272.1	45202849	T	C
NC_040272.1	45279885	T	C
NC_040272.1	45339150	A	G
NC_040272.1	45346756	G	C
NC_040272.1	45352216	A	G
NC_040272.1	45352418	T	C
NC_040272.1	45386996	T	C
NC_040272.1	45500123	A	G
NC_040272.1	45501061	A	G
NC_040272.1	45501834	T	C
NC_040272.1	45509190	C	A
NC_040272.1	45521153	G	A
NC_040272.1	45523489	T	C
NC_040272.1	45526247	A	G
NC_040272.1	45532185	G	A
NC_040272.1	45532347	T	C
NC_040272.1	45548432	T	C
NC_040272.1	45550007	T	C
NC_040272.1	45550646	C	T
NC_040272.1	45556500	A	G
NC_040272.1	45558414	G	A

NC_040272.1	45566107	T	A
NC_040272.1	45566695	A	G
NC_040272.1	45569853	A	G
NC_040272.1	45573304	A	G
NC_040272.1	45573733	T	C
NC_040272.1	45589141	C	T
NC_040272.1	45592102	A	G
NC_040272.1	45593296	A	G
NC_040272.1	45605809	T	C
NC_040272.1	45606843	T	C
NC_040272.1	45608549	C	T
NC_040272.1	45608702	T	C
NC_040272.1	45612654	A	G
NC_040272.1	45621039	C	T
NC_040272.1	45624385	A	G
NC_040272.1	45624910	T	A
NC_040272.1	45626509	A	G
NC_040272.1	45634304	T	C
NC_040272.1	45677359	T	C
NC_040272.1	45678243	T	C
NC_040272.1	45690815	C	A
NC_040272.1	45706444	T	G
NC_040272.1	45736025	G	A
NC_040272.1	45753523	A	G
NC_040272.1	45754169	G	C
NC_040272.1	45754380	G	A
NC_040272.1	45755611	C	T
NC_040272.1	45758378	A	G
NC_040272.1	45759083	A	G
NC_040272.1	45764076	T	C
NC_040272.1	45764894	G	A
NC_040272.1	45767851	C	G
NC_040272.1	45770815	A	G
NC_040272.1	45773279	C	T
NC_040272.1	45787415	A	G
NC_040272.1	45789503	A	G
NC_040272.1	45793245	A	G
NC_040272.1	45794809	G	T
NC_040272.1	45800290	C	G
NC_040272.1	45812870	G	A
NC_040272.1	45813956	G	A
NC_040272.1	45874916	T	C
NC_040272.1	45876078	T	C
NC_040272.1	45876257	A	G
NC_040272.1	45877604	A	G
NC_040272.1	45879540	C	T
NC_040272.1	45888082	G	C

NC_040272.1	45888621	A	G
NC_040272.1	45899181	T	C
NC_040272.1	45899376	A	G
NC_040272.1	45920449	T	C
NC_040272.1	45923804	C	G
NC_040272.1	45924426	C	G
NC_040272.1	45929717	T	C
NC_040272.1	45933541	T	C
NC_040272.1	45991224	A	G
NC_040272.1	46048302	A	T
NC_040272.1	46080064	T	C
NC_040272.1	46097483	C	T
NC_040272.1	46101509	A	G
NC_040272.1	46104351	T	C
NC_040272.1	46118071	C	A
NC_040272.1	46119748	A	G
NC_040272.1	46150481	A	G
NC_040272.1	46151126	C	G
NC_040272.1	46151281	T	G
NC_040272.1	46162109	T	G
NC_040272.1	46162269	G	A
NC_040272.1	46162460	G	A
NC_040272.1	46164477	A	G
NC_040272.1	46166360	T	C
NC_040272.1	46196230	T	C
NC_040272.1	46198897	T	C
NC_040272.1	46246660	A	G
NC_040272.1	46247660	A	C
NC_040272.1	46261011	G	A
NC_040272.1	46266584	T	C
NC_040272.1	46294583	T	C
NC_040272.1	46294763	A	G
NC_040272.1	46294977	G	A
NC_040272.1	46295336	G	T
NC_040272.1	46300359	C	T
NC_040272.1	46349381	G	A
NC_040272.1	46349690	T	C
NC_040272.1	46359967	C	T
NC_040272.1	46375891	G	A
NC_040272.1	46400347	A	G
NC_040272.1	46401478	T	C
NC_040272.1	46401662	A	C
NC_040272.1	46401841	C	G
NC_040272.1	46402304	C	T
NC_040272.1	46402544	A	T
NC_040272.1	46402723	G	C
NC_040272.1	46402902	C	G

NC_040272.1	46411502	C	T
NC_040272.1	46415664	G	A
NC_040272.1	46431591	A	G
NC_040272.1	46431987	C	G
NC_040272.1	46432431	A	G
NC_040272.1	46434092	A	G
NC_040272.1	46434304	A	G
NC_040272.1	46435068	A	G
NC_040272.1	46436320	C	T
NC_040272.1	46436614	C	T
NC_040272.1	46440329	A	G
NC_040272.1	46440881	C	G
NC_040272.1	46466511	G	T
NC_040272.1	46495304	A	G
NC_040272.1	46496194	T	C
NC_040272.1	46497340	G	T
NC_040272.1	46499340	C	T
NC_040272.1	46499517	T	C
NC_040272.1	46508715	T	C
NC_040272.1	46550810	A	G
NC_040272.1	46592024	T	G
NC_040272.1	46596780	C	T
NC_040272.1	46625565	A	G
NC_040272.1	46626985	T	C
NC_040272.1	46633696	T	C
NC_040272.1	46634119	C	T
NC_040272.1	46642961	A	G
NC_040272.1	46643876	G	C
NC_040272.1	46676311	T	C
NC_040272.1	46719396	C	T
NC_040272.1	46739044	T	C
NC_040272.1	46782033	C	T
NC_040272.1	46789463	T	C
NC_040272.1	46806380	A	G
NC_040272.1	46807280	T	C
NC_040272.1	46868784	G	A
NC_040272.1	46912769	T	C
NC_040272.1	46923795	A	G
NC_040272.1	46923969	C	T
NC_040272.1	46933672	A	G
NC_040272.1	46960217	T	C
NC_040272.1	46963980	T	C
NC_040272.1	46964276	T	C
NC_040272.1	46964684	T	C
NC_040272.1	46979304	C	A
NC_040272.1	46980694	T	C
NC_040272.1	46981961	T	C

NC_040272.1	46985582	T	C
NC_040272.1	47011637	A	C
NC_040272.1	47047023	T	C
NC_040272.1	47048825	A	G
NC_040272.1	47049166	G	C
NC_040272.1	47050341	A	G
NC_040272.1	47051767	G	C
NC_040272.1	47055020	T	C
NC_040272.1	47058931	A	G
NC_040272.1	47069418	T	C
NC_040272.1	47071559	T	C
NC_040272.1	47073190	T	C
NC_040272.1	47074474	A	G
NC_040272.1	47081314	T	C
NC_040272.1	47096381	T	C
NC_040272.1	47097000	A	G
NC_040272.1	47108280	T	C
NC_040272.1	47111492	T	C
NC_040272.1	47113199	T	C
NC_040272.1	47144207	A	G
NC_040272.1	47145203	T	G
NC_040272.1	47146052	T	C
NC_040272.1	47215255	A	G
NC_040272.1	47218638	T	C
NC_040272.1	47277051	T	C
NC_040272.1	47304777	T	C
NC_040272.1	47310072	A	G
NC_040272.1	47319251	T	C
NC_040272.1	47320601	A	G
NC_040272.1	47348588	T	C
NC_040272.1	47375604	G	C
NC_040272.1	47375884	T	C
NC_040272.1	47412167	A	G
NC_040272.1	47412347	T	C
NC_040272.1	47468494	A	G
NC_040272.1	47468665	A	G
NC_040272.1	47591898	T	C
NC_040272.1	47628163	T	C
NC_040272.1	47642032	C	T
NC_040272.1	47700150	T	C
NC_040272.1	47775452	T	C
NC_040272.1	47843970	T	C
NC_040272.1	47884208	C	G
NC_040272.1	47889048	A	G
NC_040272.1	47961019	T	C
NC_040272.1	48018895	T	A
NC_040272.1	48095247	A	G

NC_040272.1	48095825	A		G
NC_040272.1	48103261	C		G
NC_040272.1	48454235	A		G
NC_040272.1	48498809	A		G
NC_040272.1	48500699	A		G
NC_040272.1	48706380	A		G
NC_040272.1	48732650	A		G
NC_040272.1	48763394	C		A
NC_040272.1	48818754	G		C
NC_040272.1	49082335		0	G
NC_040272.1	49088322	A		G
NC_040272.1	49128088	G		A
NC_040272.1	49180900	A		G
NC_040272.1	49181456	A		G
NC_040272.1	49199668	A		G
NC_040272.1	49199825	T		C
NC_040272.1	49244860	C		T
NC_040272.1	49289986	T		C
NC_040272.1	49315347	T		C
NC_040272.1	49380870	G		T
NC_040272.1	49381898	C		A
NC_040272.1	49393517	T		C
NC_040272.1	49393760	T		C
NC_040272.1	49463960	T		C
NC_040272.1	49519148	C		T
NC_040272.1	49578392	T		C
NC_040272.1	49650584	C		T
NC_040272.1	49723460	A		T
NC_040272.1	49758455	T		C
NC_040272.1	49805289	G		A
NC_040272.1	50029176	T		C
NC_040272.1	50120121	A		G
NC_040272.1	50125574	C		T
NC_040272.1	50137216	A		T
NC_040272.1	50159181	C		T
NC_040272.1	50290085	G		T
NC_040272.1	50359247	T		C
NC_040272.1	50361072	A		G
NC_040272.1	50445195	T		G
NC_040272.1	50516901	T		C
NC_040272.1	50570000	T		G
NC_040272.1	50631959	A		G
NC_040272.1	50688875	C		G
NC_040272.1	50746419	A		G
NC_040272.1	50803094	G		A
NC_040272.1	50864493	G		A
NC_040272.1	50925492	A		G

NC_040272.1	50981543	G	A
NC_040272.1	51018960	T	C
NC_040272.1	51023678	A	C
NC_040272.1	51031414	A	G
NC_040272.1	51034420	C	G
NC_040272.1	51038181	A	G
NC_040272.1	51038410	A	C
NC_040272.1	51056175	A	G
NC_040272.1	51096559	C	A
NC_040272.1	51096833	G	T
NC_040272.1	51141186	T	G
NC_040272.1	51219789	A	G
NC_040272.1	51223226	A	G
NC_040272.1	51225957	T	C
NC_040272.1	51239730	A	G
NC_040272.1	51245982	T	C
NC_040272.1	51253857	T	C
NC_040272.1	51322481	A	G
NC_040272.1	51333877	C	T
NC_040272.1	51407421	C	T
NC_040272.1	51443205	T	C
NC_040272.1	51468281	A	G
NC_040272.1	51471938	C	G
NC_040272.1	51486440	T	C
NC_040272.1	51511901	A	G
NC_040272.1	51512112	A	G
NC_040272.1	51512376	A	G
NC_040272.1	51575264	C	T
NC_040272.1	51598677	T	C
NC_040272.1	51852150	A	G
NC_040272.1	51865138	T	C
NC_040272.1	51865409	A	G
NC_040272.1	51866026	A	C
NC_040272.1	51866306	T	G
NC_040272.1	51866518	T	C
NC_040272.1	51867155	C	T
NC_040272.1	51929002	T	C
NC_040272.1	51947374	G	A
NC_040272.1	51985295	T	C
NC_040272.1	52051930	T	A
NC_040272.1	52128359	G	C
NC_040272.1	52132867	G	A
NC_040272.1	52133059	T	C
NC_040272.1	52146193	A	G
NC_040272.1	52215479	C	G
NC_040272.1	52243422	A	G
NC_040272.1	52248805	0	A

NC_040272.1	52257930	A	G
NC_040272.1	52260393	T	C
NC_040272.1	52260669	T	C
NC_040272.1	52264037	C	G
NC_040272.1	52264368	A	G
NC_040272.1	52277340	A	G
NC_040272.1	52292233	A	G
NC_040272.1	52299996	A	G
NC_040272.1	52330029	T	C
NC_040272.1	52330324	C	T
NC_040272.1	52334639	A	G
NC_040272.1	52336271	G	A
NC_040272.1	52350392	A	G
NC_040272.1	52367156	C	A
NC_040272.1	52385635	0	G
NC_040272.1	52397233	G	A
NC_040272.1	52399729	G	A
NC_040272.1	52404132	G	A
NC_040272.1	52407656	C	G
NC_040272.1	52410968	A	G
NC_040272.1	52412895	C	G
NC_040272.1	52413320	C	T
NC_040272.1	52413627	G	A
NC_040272.1	52414081	T	C
NC_040272.1	52416152	A	G
NC_040272.1	52445072	A	C
NC_040272.1	52445804	G	A
NC_040272.1	52446139	A	G
NC_040272.1	52446324	0	T
NC_040272.1	52451572	A	G
NC_040272.1	52455828	C	T
NC_040272.1	52469710	G	A
NC_040272.1	52469861	A	G
NC_040272.1	52470407	G	C
NC_040272.1	52470631	A	G
NC_040272.1	52471012	C	T
NC_040272.1	52471203	C	T
NC_040272.1	52507456	A	G
NC_040272.1	52517022	T	C
NC_040272.1	52577324	T	C
NC_040272.1	52596980	C	T
NC_040272.1	52608356	T	C
NC_040272.1	52610182	T	C
NC_040272.1	52733609	G	T
NC_040272.1	52737117	G	C
NC_040272.1	52737806	A	G
NC_040272.1	52816699	T	C

NC_040272.1	52821015	A	G
NC_040272.1	52867223	C	G
NC_040272.1	52868279	C	T
NC_040272.1	52870424	A	G
NC_040272.1	52873289	T	C
NC_040272.1	52876925	C	T
NC_040272.1	52879567	C	T
NC_040272.1	52880250	A	C
NC_040272.1	52885698	G	A
NC_040272.1	52940270	A	G
NC_040273.1	17027	G	A
NC_040273.1	83421	T	C
NC_040273.1	146917	A	G
NC_040273.1	197353	A	G
NC_040273.1	252877	T	G
NC_040273.1	296142	A	G
NC_040273.1	335374	A	G
NC_040273.1	376756	T	C
NC_040273.1	449689	T	G
NC_040273.1	576565	G	A
NC_040273.1	618408	A	T
NC_040273.1	661150	T	C
NC_040273.1	789390	A	G
NC_040273.1	877558	C	T
NC_040273.1	921808	C	T
NC_040273.1	966348	G	T
NC_040273.1	1052602	T	G
NC_040273.1	1093613	G	C
NC_040273.1	1149002	G	C
NC_040273.1	1215549	G	A
NC_040273.1	1292376	C	T
NC_040273.1	1387470	T	G
NC_040273.1	1436885	G	C
NC_040273.1	1613186	G	T
NC_040273.1	1660539	T	G
NC_040273.1	1755123	G	A
NC_040273.1	2047515	C	T
NC_040273.1	2672614	C	T
NC_040273.1	2777703	C	T
NC_040273.1	3005002	T	C
NC_040273.1	3054564	C	T
NC_040273.1	3110625	G	A
NC_040273.1	3200279	T	A
NC_040273.1	3212581	A	G
NC_040273.1	3466147	A	G
NC_040273.1	3501679	C	T
NC_040273.1	3601625	G	A

NC_040273.1	3655455	G	T
NC_040273.1	3691262	C	T
NC_040273.1	4000112	G	A
NC_040273.1	4039988	T	A
NC_040273.1	4103306	T	C
NC_040273.1	4136072	G	A
NC_040273.1	4186170	C	G
NC_040273.1	4252550	A	G
NC_040273.1	4315542	T	G
NC_040273.1	4568538	G	C
NC_040273.1	4632320	T	C
NC_040273.1	4682388	A	G
NC_040273.1	4731195	G	A
NC_040273.1	4828057	T	C
NC_040273.1	4884116	C	A
NC_040273.1	4985136	A	C
NC_040273.1	5055641	A	T
NC_040273.1	5179985	T	C
NC_040273.1	5265733	G	T
NC_040273.1	5324486	G	A
NC_040273.1	5405049	A	G
NC_040273.1	5428295	A	T
NC_040273.1	5561131	G	A
NC_040273.1	5611626	C	G
NC_040273.1	5622163	A	G
NC_040273.1	6391780	C	T
NC_040273.1	7102818	A	G
NC_040273.1	7332193	A	T
NC_040273.1	7364967	C	T
NC_040273.1	7669303	C	G
NC_040273.1	8294284	C	A
NC_040273.1	8347846	C	A
NC_040273.1	8417124	C	T
NC_040273.1	8617068	T	A
NC_040273.1	8681844	T	C
NC_040273.1	8744037	A	G
NC_040273.1	8793460	C	T
NC_040273.1	8854814	A	G
NC_040273.1	9048776	C	T
NC_040273.1	9171022	G	A
NC_040273.1	9199238	G	T
NC_040273.1	9344868	A	T
NC_040273.1	9528425	A	G
NC_040273.1	9576560	A	G
NC_040273.1	9645373	C	T
NC_040273.1	9696559	A	T
NC_040273.1	9850513	T	C

NC_040273.1	9994248	C	T
NC_040273.1	10071012	T	C
NC_040273.1	10641632	T	A
NC_040273.1	10735450	A	C
NC_040273.1	10783753	A	T
NC_040273.1	10883775	A	G
NC_040273.1	10945983	C	A
NC_040273.1	11019449	C	A
NC_040273.1	11173280	T	G
NC_040273.1	11212743	G	A
NC_040273.1	11253668	G	A
NC_040273.1	11339781	A	T
NC_040273.1	11397035	C	T
NC_040273.1	11457336	A	C
NC_040273.1	11524019	G	A
NC_040273.1	12158916	C	T
NC_040273.1	12277409	A	G
NC_040273.1	12300807	T	C
NC_040273.1	12384427	G	C
NC_040273.1	12456458	T	C
NC_040273.1	12535531	C	T
NC_040273.1	12575460	A	C
NC_040273.1	12705986	G	A
NC_040273.1	12760992	T	C
NC_040273.1	12813980	G	A
NC_040273.1	12879986	C	A
NC_040273.1	12967611	C	T
NC_040273.1	13128568	A	G
NC_040273.1	13191658	G	A
NC_040273.1	13685414	A	G
NC_040273.1	14062919	A	G
NC_040273.1	14373991	T	C
NC_040273.1	14422873	C	A
NC_040273.1	14610242	G	A
NC_040273.1	14716785	C	T
NC_040273.1	14771820	A	G
NC_040273.1	14838074	C	T
NC_040273.1	15036018	C	T
NC_040273.1	15158302	G	T
NC_040273.1	15185039	A	G
NC_040273.1	15308033	G	A
NC_040273.1	15334389	G	A
NC_040273.1	15407895	T	C
NC_040273.1	15463507	T	G
NC_040273.1	15493400	G	A
NC_040273.1	15556479	C	A
NC_040273.1	15593184	T	C

NC_040273.1	15681548	T	C
NC_040273.1	15770646	C	T
NC_040273.1	15845068	G	C
NC_040273.1	15932758	G	T
NC_040273.1	16094813	T	G
NC_040273.1	16151616	C	T
NC_040273.1	16201552	A	T
NC_040273.1	16247522	T	C
NC_040273.1	16324325	0	G
NC_040273.1	16360637	G	A
NC_040273.1	16423717	A	G
NC_040273.1	16485931	G	A
NC_040273.1	16519603	C	A
NC_040273.1	16582603	G	A
NC_040273.1	16641776	G	A
NC_040273.1	16693732	T	C
NC_040273.1	16713612	G	T
NC_040273.1	16923396	C	A
NC_040273.1	16964512	G	C
NC_040273.1	17015808	G	A
NC_040273.1	17091662	G	A
NC_040273.1	17146092	T	C
NC_040273.1	17227128	T	C
NC_040273.1	17281378	A	G
NC_040273.1	17314633	G	A
NC_040273.1	17447722	T	C
NC_040273.1	17503379	T	C
NC_040273.1	17568716	G	A
NC_040273.1	17643567	G	C
NC_040273.1	17694502	G	T
NC_040273.1	17755579	G	C
NC_040273.1	17826974	A	G
NC_040273.1	17848697	G	A
NC_040273.1	18193494	T	C
NC_040273.1	18225506	A	C
NC_040273.1	18282836	T	C
NC_040273.1	18835956	A	T
NC_040273.1	18879773	G	C
NC_040273.1	18999777	T	A
NC_040273.1	19266890	T	G
NC_040273.1	19332940	G	A
NC_040273.1	19375248	G	A
NC_040273.1	19427925	A	G
NC_040273.1	19471623	A	G
NC_040273.1	19514056	A	C
NC_040273.1	19580437	A	G
NC_040273.1	19655072	C	T

NC_040273.1	19705789	C	T
NC_040273.1	19759386	G	A
NC_040273.1	19785916	T	A
NC_040273.1	19787689	G	A
NC_040273.1	19843301	A	G
NC_040273.1	19888690	G	T
NC_040273.1	19916861	G	A
NC_040273.1	19977866	T	A
NC_040273.1	20001454	G	A
NC_040273.1	20004753	G	C
NC_040273.1	20060865	T	C
NC_040273.1	20109236	G	A
NC_040273.1	20158967	A	G
NC_040273.1	20178069	T	C
NC_040273.1	20190524	G	C
NC_040273.1	20242325	A	G
NC_040273.1	20299366	T	C
NC_040273.1	20352362	G	A
NC_040273.1	20403321	A	G
NC_040273.1	20409695	A	G
NC_040273.1	20461638	T	A
NC_040273.1	20517173	A	G
NC_040273.1	20561113	A	G
NC_040273.1	20625758	G	A
NC_040273.1	20673623	C	A
NC_040273.1	20721686	C	G
NC_040273.1	20729664	A	G
NC_040273.1	20735875	G	A
NC_040273.1	20752087	T	A
NC_040273.1	20756447	G	A
NC_040273.1	20760178	A	G
NC_040273.1	20763520	T	C
NC_040273.1	20767587	G	A
NC_040273.1	20788019	G	A
NC_040273.1	20840851	C	A
NC_040273.1	20890095	C	G
NC_040273.1	20937705	A	G
NC_040273.1	20978934	A	G
NC_040273.1	21027068	C	T
NC_040273.1	21069493	A	C
NC_040273.1	21115414	A	G
NC_040273.1	21147937	T	C
NC_040273.1	21204544	G	A
NC_040273.1	21260872	A	G
NC_040273.1	21282575	A	G
NC_040273.1	21339022	T	A
NC_040273.1	21400047	C	A

NC_040273.1	21436356	C	G
NC_040273.1	21493268	T	C
NC_040273.1	21552328	C	T
NC_040273.1	21607769	T	G
NC_040273.1	21665954	C	T
NC_040273.1	21721570	G	C
NC_040273.1	21785435	G	A
NC_040273.1	21844425	A	G
NC_040273.1	21893430	T	C
NC_040273.1	21936305	T	C
NC_040273.1	21964378	A	G
NC_040273.1	21980952	T	C
NC_040273.1	22029755	T	C
NC_040273.1	22089966	A	G
NC_040273.1	22162754	A	G
NC_040273.1	22197162	A	G
NC_040273.1	22435051	T	G
NC_040273.1	22488314	G	A
NC_040273.1	22561174	T	C
NC_040273.1	22598909	G	A
NC_040273.1	22659592	A	G
NC_040273.1	22695072	G	A
NC_040273.1	22746806	C	G
NC_040273.1	22798571	G	A
NC_040273.1	22844522	T	C
NC_040273.1	22900160	T	C
NC_040273.1	22957162	T	C
NC_040273.1	22997718	T	G
NC_040273.1	23062384	T	C
NC_040273.1	23108492	C	T
NC_040273.1	23170755	A	G
NC_040273.1	23208036	G	A
NC_040273.1	23250884	T	C
NC_040273.1	23310190	C	G
NC_040273.1	23367304	A	G
NC_040273.1	23435011	T	C
NC_040273.1	23465620	G	C
NC_040273.1	23472118	T	C
NC_040273.1	23486932	C	T
NC_040273.1	23546311	C	T
NC_040273.1	23599494	T	G
NC_040273.1	23662804	C	T
NC_040273.1	23689907	C	T
NC_040273.1	23726535	T	C
NC_040273.1	23783431	A	G
NC_040273.1	23834361	A	G
NC_040273.1	23888762	T	C

NC_040273.1	23951828	T	A
NC_040273.1	24013926	C	T
NC_040273.1	24050479	T	C
NC_040273.1	24105696	A	G
NC_040273.1	24161319	C	G
NC_040273.1	24216952	T	C
NC_040273.1	24273218	T	C
NC_040273.1	24328505	T	C
NC_040273.1	24383180	T	C
NC_040273.1	24389475	C	T
NC_040273.1	24418065	A	G
NC_040273.1	24420496	T	C
NC_040273.1	24434379	T	C
NC_040273.1	24438457	T	C
NC_040273.1	24440026	C	T
NC_040273.1	24444196	T	C
NC_040273.1	24446989	A	G
NC_040273.1	24456434	A	G
NC_040273.1	24478940	T	C
NC_040273.1	24487375	T	C
NC_040273.1	24488438	T	G
NC_040273.1	24534580	T	C
NC_040273.1	24590376	G	A
NC_040273.1	24640693	T	G
NC_040273.1	24695539	C	T
NC_040273.1	24751209	A	G
NC_040273.1	24807284	T	C
NC_040273.1	24856666	C	T
NC_040273.1	24898025	G	A
NC_040273.1	24933366	G	T
NC_040273.1	24964366	A	G
NC_040273.1	25018416	A	G
NC_040273.1	25072612	A	C
NC_040273.1	25130005	G	A
NC_040273.1	25193013	A	T
NC_040273.1	25251706	G	T
NC_040273.1	25306734	A	G
NC_040273.1	25348339	A	G
NC_040273.1	25380656	C	T
NC_040273.1	25412762	G	A
NC_040273.1	25454376	C	T
NC_040273.1	25456495	C	G
NC_040273.1	25496168	T	A
NC_040273.1	25500511	C	T
NC_040273.1	25555160	A	G
NC_040273.1	25602185	T	C
NC_040273.1	25635884	T	A

NC_040273.1	25665785	T	C
NC_040273.1	25706460	C	A
NC_040273.1	25765418	C	G
NC_040273.1	25818563	A	G
NC_040273.1	25872948	C	G
NC_040273.1	25920446	T	C
NC_040273.1	25957829	T	C
NC_040273.1	26017554	T	C
NC_040273.1	26056314	G	A
NC_040273.1	26102813	A	G
NC_040273.1	26155492	T	C
NC_040273.1	26222391	C	T
NC_040273.1	26270576	G	A
NC_040273.1	26271321	T	C
NC_040273.1	26275271	T	C
NC_040273.1	26279942	A	G
NC_040273.1	26296070	A	G
NC_040273.1	26337346	T	C
NC_040273.1	26388851	T	C
NC_040273.1	26427567	C	G
NC_040273.1	26438720	A	G
NC_040273.1	26497848	T	G
NC_040273.1	26524222	G	A
NC_040273.1	26579902	A	G
NC_040273.1	26626747	A	G
NC_040273.1	26700955	C	T
NC_040273.1	26703106	T	C
NC_040273.1	26712277	C	T
NC_040273.1	26768568	T	C
NC_040273.1	26815857	A	G
NC_040273.1	26873702	T	C
NC_040273.1	26937871	A	G
NC_040273.1	26986931	T	C
NC_040273.1	27043262	A	G
NC_040273.1	27105064	G	A
NC_040273.1	27130622	T	C
NC_040273.1	27181185	A	G
NC_040273.1	27248494	C	T
NC_040273.1	27300127	G	A
NC_040273.1	27367718	G	T
NC_040273.1	27420941	A	C
NC_040273.1	27439389	G	T
NC_040273.1	27503576	A	G
NC_040273.1	27565626	G	C
NC_040273.1	27604305	A	G
NC_040273.1	27759534	C	G
NC_040273.1	27826666	C	T

NC_040273.1	27888051	G	A
NC_040273.1	27956888	T	C
NC_040273.1	28025351	T	C
NC_040273.1	28143790	C	T
NC_040273.1	28205465	C	T
NC_040273.1	28253139	T	C
NC_040273.1	28293505	T	A
NC_040273.1	28364370	A	T
NC_040273.1	28406132	T	C
NC_040273.1	28438264	C	T
NC_040273.1	28486960	T	C
NC_040273.1	28533922	C	T
NC_040273.1	28583387	A	G
NC_040273.1	28638455	C	T
NC_040273.1	28701528	A	C
NC_040273.1	28815570	G	T
NC_040273.1	29022815	T	A
NC_040273.1	29052785	C	T
NC_040273.1	29146389	C	T
NC_040273.1	29263445	T	C
NC_040273.1	29328774	T	G
NC_040273.1	29405109	A	T
NC_040273.1	29485388	C	A
NC_040273.1	29529729	T	C
NC_040273.1	29591541	G	T
NC_040273.1	29659955	C	T
NC_040273.1	29721598	G	A
NC_040273.1	30066513	T	C
NC_040273.1	30094652	A	T
NC_040273.1	30160042	G	A
NC_040273.1	30210877	C	T
NC_040273.1	30272683	G	A
NC_040273.1	30333324	C	T
NC_040273.1	30388274	G	T
NC_040273.1	30452819	C	T
NC_040273.1	30519950	T	C
NC_040273.1	30616219	A	C
NC_040273.1	30678497	A	T
NC_040273.1	30735377	A	G
NC_040273.1	30774907	C	G
NC_040273.1	30805617	G	A
NC_040273.1	30869797	T	G
NC_040273.1	30931267	C	T
NC_040273.1	30984942	A	T
NC_040273.1	31023349	G	C
NC_040273.1	31081794	A	G
NC_040273.1	31132246	T	C

NC_040273.1	31190977	A	C
NC_040273.1	31244167	C	T
NC_040273.1	31294258	T	C
NC_040273.1	31354306	C	T
NC_040273.1	31466677	T	C
NC_040273.1	31554287	C	T
NC_040273.1	31607442	G	A
NC_040273.1	31661291	A	G
NC_040273.1	32085457	G	A
NC_040273.1	32144910	C	G
NC_040273.1	32216336	A	C
NC_040273.1	32269114	C	A
NC_040273.1	32309805	G	C
NC_040273.1	32472034	A	T
NC_040273.1	32514414	T	C
NC_040273.1	32602295	T	C
NC_040273.1	32637441	C	T
NC_040273.1	32694363	T	G
NC_040273.1	32739275	T	A
NC_040273.1	32814671	T	A
NC_040273.1	32898928	C	T
NC_040273.1	32946635	G	C
NC_040273.1	33018659	C	T
NC_040273.1	33065435	G	C
NC_040273.1	33131752	C	G
NC_040273.1	33200127	G	T
NC_040273.1	33248589	C	T
NC_040273.1	33299898	G	A
NC_040273.1	33361763	C	T
NC_040273.1	33390414	T	G
NC_040273.1	33474088	T	G
NC_040273.1	33527998	A	G
NC_040273.1	33585462	A	C
NC_040273.1	33641692	T	C
NC_040273.1	33699311	G	A
NC_040273.1	33754106	T	C
NC_040273.1	33809635	G	A
NC_040273.1	33857591	T	A
NC_040273.1	33865765	A	G
NC_040273.1	33931250	A	C
NC_040273.1	33981042	A	G
NC_040273.1	34022891	C	T
NC_040273.1	34071787	T	G
NC_040273.1	34130233	T	C
NC_040273.1	34187635	C	T
NC_040273.1	34239110	A	G
NC_040273.1	34285230	T	C

NC_040273.1	34343030	C	T
NC_040273.1	34395465	G	C
NC_040273.1	34432768	C	T
NC_040273.1	34465120	A	G
NC_040273.1	34521920	A	G
NC_040273.1	34567930	C	T
NC_040273.1	34606026	T	C
NC_040273.1	34660017	G	T
NC_040273.1	34728641	C	T
NC_040273.1	34798453	T	C
NC_040273.1	34844962	T	C
NC_040273.1	34895803	C	T
NC_040273.1	34953346	C	T
NC_040273.1	35010710	C	G
NC_040273.1	35070708	T	A
NC_040273.1	35128331	T	C
NC_040273.1	35180079	C	T
NC_040273.1	35239025	T	G
NC_040273.1	35294577	A	G
NC_040273.1	35350569	A	G
NC_040273.1	35410515	C	T
NC_040273.1	35462917	T	C
NC_040273.1	35511886	C	T
NC_040273.1	35560903	G	A
NC_040273.1	35623741	C	A
NC_040273.1	35668356	T	C
NC_040273.1	35702928	G	T
NC_040273.1	35757797	T	C
NC_040273.1	35807125	G	A
NC_040273.1	35844316	G	A
NC_040273.1	35915317	C	T
NC_040273.1	35974999	C	G
NC_040273.1	36042136	A	G
NC_040273.1	36118219	C	T
NC_040273.1	36120183	T	C
NC_040273.1	36173301	T	A
NC_040273.1	36224962	G	A
NC_040273.1	36285835	C	T
NC_040273.1	36338620	A	G
NC_040273.1	36397277	G	A
NC_040273.1	36450332	G	A
NC_040273.1	36505001	A	C
NC_040273.1	36514143	C	G
NC_040273.1	36553488	C	T
NC_040273.1	36606022	G	T
NC_040273.1	36660861	A	G
NC_040273.1	36716580	A	G

NC_040273.1	36773493	T	C
NC_040273.1	36825386	A	G
NC_040273.1	36882142	A	T
NC_040273.1	36905946	T	C
NC_040273.1	36955140	G	A
NC_040273.1	37008581	A	C
NC_040273.1	37064213	C	T
NC_040273.1	37120711	C	G
NC_040273.1	37189246	A	G
NC_040273.1	37235153	T	C
NC_040273.1	37290417	A	G
NC_040273.1	37324101	G	A
NC_040273.1	37383113	G	C
NC_040273.1	37436510	T	A
NC_040273.1	37467626	T	C
NC_040273.1	37472379	A	T
NC_040273.1	37528340	A	G
NC_040273.1	37582071	C	T
NC_040273.1	37641955	A	C
NC_040273.1	37709928	A	G
NC_040273.1	37763842	C	G
NC_040273.1	37801501	A	G
NC_040273.1	37858279	A	G
NC_040273.1	37912207	T	C
NC_040273.1	37934270	A	G
NC_040273.1	37935672	A	G
NC_040273.1	37993999	T	C
NC_040273.1	38042994	C	T
NC_040273.1	38081505	C	T
NC_040273.1	38129424	C	A
NC_040273.1	38164910	C	T
NC_040273.1	38290077	C	T
NC_040273.1	38356548	A	G
NC_040273.1	38412552	A	C
NC_040273.1	38477286	T	G
NC_040273.1	38497018	T	A
NC_040273.1	38499200	G	T
NC_040273.1	38575913	T	G
NC_040273.1	38603112	C	G
NC_040273.1	38668378	C	G
NC_040273.1	38729502	C	T
NC_040273.1	38777437	T	A
NC_040273.1	38853460	T	G
NC_040273.1	38916256	C	T
NC_040273.1	38988335	G	A
NC_040273.1	39083811	G	A
NC_040273.1	39100774	A	G

NC_040273.1	39231084	A	G
NC_040273.1	39266590	T	C
NC_040273.1	39313660	T	C
NC_040273.1	39362536	G	T
NC_040273.1	39414308	C	T
NC_040273.1	39460205	T	C
NC_040273.1	39511428	T	C
NC_040273.1	39584614	C	G
NC_040273.1	39635756	C	T
NC_040273.1	39689431	A	G
NC_040273.1	39748543	C	T
NC_040273.1	39808012	A	T
NC_040273.1	39858951	A	G
NC_040273.1	39915819	T	C
NC_040273.1	39967630	C	T
NC_040273.1	40024427	A	T
NC_040273.1	40083017	A	G
NC_040273.1	40150889	A	C
NC_040273.1	40219972	T	C
NC_040273.1	40249368	A	G
NC_040273.1	40299514	G	A
NC_040273.1	40350900	G	A
NC_040273.1	40406712	T	C
NC_040273.1	40449475	C	A
NC_040273.1	40500722	A	G
NC_040273.1	40547604	C	A
NC_040273.1	40597644	T	G
NC_040273.1	40657220	T	C
NC_040273.1	40709807	A	G
NC_040273.1	40769154	T	C
NC_040273.1	40844313	A	G
NC_040273.1	40906850	T	C
NC_040273.1	40971384	A	G
NC_040273.1	41018514	T	C
NC_040273.1	41077690	G	A
NC_040273.1	41137976	T	A
NC_040273.1	41185245	G	A
NC_040273.1	41226314	A	G
NC_040273.1	41286233	T	G
NC_040273.1	41333013	G	A
NC_040273.1	41383088	A	G
NC_040273.1	41429793	G	A
NC_040273.1	41470375	G	C
NC_040273.1	41554447	C	T
NC_040273.1	41624175	C	T
NC_040273.1	41680960	A	G
NC_040273.1	41732590	A	G

NC_040273.1	41782136	C	T
NC_040273.1	41839940	T	C
NC_040273.1	41895991	G	T
NC_040273.1	41957048	C	T
NC_040273.1	42023736	G	A
NC_040273.1	42070603	T	C
NC_040273.1	42095506	G	C
NC_040273.1	42152714	A	C
NC_040273.1	42200181	T	G
NC_040273.1	42256193	G	A
NC_040273.1	42310591	C	T
NC_040273.1	42361171	A	C
NC_040273.1	42413623	T	C
NC_040273.1	42462125	C	T
NC_040273.1	42515542	C	T
NC_040273.1	42562656	G	A
NC_040273.1	42592095	G	A
NC_040273.1	42846915	T	C
NC_040273.1	42902166	A	G
NC_040273.1	42959829	T	C
NC_040273.1	43025634	T	C
NC_040273.1	43081767	A	G
NC_040273.1	43151584	C	T
NC_040273.1	43205034	C	G
NC_040273.1	43265212	C	T
NC_040273.1	43311270	T	C
NC_040273.1	43362114	T	C
NC_040273.1	43462179	A	G
NC_040273.1	43540481	T	C
NC_040273.1	43580255	A	G
NC_040273.1	43640373	G	A
NC_040273.1	43696027	C	T
NC_040273.1	43748173	A	T
NC_040273.1	43798596	C	T
NC_040273.1	43859208	G	T
NC_040273.1	43911439	T	C
NC_040273.1	43973197	G	A
NC_040273.1	44031282	A	G
NC_040273.1	44082602	C	A
NC_040273.1	44137532	T	A
NC_040273.1	44182386	A	C
NC_040273.1	44183271	T	C
NC_040273.1	44199114	A	G
NC_040273.1	44200500	G	A
NC_040273.1	44255460	A	C
NC_040273.1	44313509	A	G
NC_040273.1	44370196	A	G

NC_040273.1	44426325	G	C
NC_040273.1	44480302	A	G
NC_040273.1	44548960	A	G
NC_040273.1	44606305	G	A
NC_040273.1	44662945	C	T
NC_040273.1	44711421	C	T
NC_040273.1	44764537	A	G
NC_040273.1	44764767	G	A
NC_040273.1	44781427	C	G
NC_040273.1	44817580	T	C
NC_040273.1	44868846	G	A
NC_040273.1	44922575	A	G
NC_040273.1	44979266	T	C
NC_040273.1	44981198	T	C
NC_040273.1	44982310	A	G
NC_040273.1	45035738	G	T
NC_040273.1	45068389	T	C
NC_040273.1	45109092	A	G
NC_040273.1	45139112	C	T
NC_040273.1	45162406	A	G
NC_040273.1	45168283	G	C
NC_040273.1	45225100	G	A
NC_040273.1	45254058	G	A
NC_040273.1	45254417	T	C
NC_040273.1	45254885	A	G
NC_040273.1	45255045	T	G
NC_040273.1	45255354	A	G
NC_040273.1	45256110	T	C
NC_040273.1	45256378	C	T
NC_040273.1	45256747	G	C
NC_040273.1	45257272	T	C
NC_040273.1	45258006	A	G
NC_040273.1	45318447	T	G
NC_040273.1	45375409	T	C
NC_040273.1	45376221	A	G
NC_040273.1	45391369	A	G
NC_040273.1	45401270	G	A
NC_040273.1	45439938	G	A
NC_040273.1	45466196	T	C
NC_040273.1	45468914	G	A
NC_040273.1	45509027	G	C
NC_040273.1	45561598	G	A
NC_040273.1	45618226	T	C
NC_040273.1	45644044	T	C
NC_040273.1	45701199	G	A
NC_040273.1	45747156	G	A
NC_040273.1	45790043	T	C

NC_040273.1	45844188	A	G
NC_040273.1	45895971	C	T
NC_040273.1	45955531	A	G
NC_040273.1	46010695	A	G
NC_040273.1	46010976	C	T
NC_040273.1	46037471	T	C
NC_040273.1	46047630	G	A
NC_040273.1	46059520	G	C
NC_040273.1	46092211	C	T
NC_040273.1	46095917	C	T
NC_040273.1	46110354	C	T
NC_040273.1	46164879	C	A
NC_040273.1	46219968	G	T
NC_040273.1	46273564	T	C
NC_040273.1	46336753	T	G
NC_040273.1	46385619	A	C
NC_040273.1	46505580	A	G
NC_040273.1	46558474	C	T
NC_040273.1	46617113	T	C
NC_040273.1	46734762	T	C
NC_040273.1	46791593	A	G
NC_040273.1	46817148	T	C
NC_040273.1	46842249	T	C
NC_040273.1	46885511	A	G
NC_040273.1	46928069	T	C
NC_040273.1	46983675	A	G
NC_040273.1	47047146	C	T
NC_040273.1	47112291	A	G
NC_040273.1	47141747	T	C
NC_040273.1	47145105	G	A
NC_040273.1	47183522	C	T
NC_040273.1	47240944	T	C
NC_040273.1	47295975	C	A
NC_040273.1	47352567	C	T
NC_040273.1	47417294	A	C
NC_040273.1	47451963	T	C
NC_040273.1	47479511	T	G
NC_040273.1	47479668	T	C
NC_040273.1	47525504	C	T
NC_040273.1	47575704	A	G
NC_040273.1	47577231	T	C
NC_040273.1	47577721	A	G
NC_040273.1	47623893	A	G
NC_040273.1	47624620	G	A
NC_040273.1	47625839	A	G
NC_040273.1	47682674	G	A
NC_040273.1	47739054	A	G

NC_040273.1	47770311	T		C
NC_040273.1	47821520	T		C
NC_040273.1	47865136		0	C
NC_040273.1	47916214	A		G
NC_040273.1	47968627	T		C
NC_040273.1	47970716	T		C
NC_040273.1	47971463	T		C
NC_040273.1	48021046	C		T
NC_040273.1	48064683	C		T
NC_040273.1	48077401	A		G
NC_040273.1	48100823		0	A
NC_040273.1	48147864	T		G
NC_040273.1	48197375	C		T
NC_040273.1	48255772	C		A
NC_040273.1	48309605	C		T
NC_040273.1	48362860	G		T
NC_040273.1	48416833	G		A
NC_040273.1	48476163	C		T
NC_040273.1	48537479	C		T
NC_040273.1	48590767	T		C
NC_040273.1	48653198	A		G
NC_040273.1	48666351	C		T
NC_040273.1	48666522	G		A
NC_040273.1	48720421	A		G
NC_040273.1	48779597	C		T
NC_040273.1	48827949	A		G
NC_040273.1	48897037	A		G
NC_040273.1	48898038	C		T
NC_040273.1	48899038	A		G
NC_040273.1	48899757	T		C
NC_040273.1	48900264	A		G
NC_040273.1	48941671	A		G
NC_040273.1	48957762	T		C
NC_040273.1	49015019	T		C
NC_040273.1	49061324	T		G
NC_040273.1	49112960	C		T
NC_040273.1	49169205	A		G
NC_040273.1	49213522	A		C
NC_040273.1	49257180	A		G
NC_040273.1	49288446	G		C
NC_040273.1	49349476	A		G
NC_040273.1	49396654	C		T
NC_040273.1	49440081	G		A
NC_040273.1	49498257	T		C
NC_040273.1	49556999	C		T
NC_040273.1	49622638	G		C
NC_040273.1	49677971	G		A

NC_040273.1	49731473	G		A
NC_040273.1	49793955	C		T
NC_040273.1	49854198	T		C
NC_040273.1	49915395	T		C
NC_040273.1	49983132	T		C
NC_040273.1	49983707	T		C
NC_040273.1	49984497	G		C
NC_040273.1	49985413	T		C
NC_040273.1	49986114	A		G
NC_040273.1	50019337	C		T
NC_040273.1	50020915	A		G
NC_040273.1	50023275	G		A
NC_040273.1	50025696	A		G
NC_040273.1	50075584	A		G
NC_040273.1	50104264	T		C
NC_040273.1	50145754	A		G
NC_040273.1	50201617	C		A
NC_040273.1	50357025	G		A
NC_040273.1	50413194	T		C
NC_040273.1	50450473	C		G
NC_040273.1	50696200	G		A
NC_040273.1	50747675	T		G
NC_040273.1	50806814	T		A
NC_040273.1	50836643	C		T
NC_040273.1	50888475	C		G
NC_040273.1	50923835	A		G
NC_040273.1	50986487		0	C
NC_040273.1	51041543	A		G
NC_040273.1	51090867	A		G
NC_040273.1	51162796	C		T
NC_040273.1	51163010	G		T
NC_040273.1	51166073	G		T
NC_040273.1	51176361	A		G
NC_040273.1	51177199	A		C
NC_040273.1	51232961	G		A
NC_040273.1	51285035	A		G
NC_040273.1	51336501	T		C
NC_040273.1	51384830	A		C
NC_040273.1	51439998	T		C
NC_040273.1	51498982	T		C
NC_040273.1	51553529	A		G
NC_040273.1	51608553	G		C
NC_040273.1	51666300	G		A
NC_040273.1	51716491	A		T
NC_040273.1	51771625	G		A
NC_040273.1	51824417	A		G
NC_040273.1	51877513	T		C

NC_040273.1	51933797	G	A
NC_040273.1	51989759	A	G
NC_040273.1	52047020	A	C
NC_040273.1	52106760	G	A
NC_040273.1	52157500	T	C
NC_040273.1	52214330	G	A
NC_040273.1	52270980	T	C
NC_040273.1	52329137	A	G
NC_040273.1	52374548	A	G
NC_040273.1	52430719	C	T
NC_040273.1	52489454	T	G
NC_040273.1	52546478	G	T
NC_040273.1	52602581	G	A
NC_040273.1	52660554	T	G
NC_040273.1	52716251	G	C
NC_040273.1	52773044	G	T
NC_040273.1	52827696	A	G
NC_040273.1	52828672	T	C
NC_040273.1	52829859	G	A
NC_040273.1	52886244	A	C
NC_040273.1	52940319	T	C
NC_040273.1	52999266	G	C
NC_040273.1	53058162	C	G
NC_040273.1	53114583	T	C
NC_040273.1	53190378	G	C
NC_040273.1	53254194	T	C
NC_040273.1	53327092	A	G
NC_040273.1	53371773	A	T
NC_040273.1	53427542	C	A
NC_040273.1	53480498	T	C
NC_040273.1	53522119	A	G
NC_040273.1	53582433	T	C
NC_040273.1	53645680	G	A
NC_040273.1	53703448	G	A
NC_040273.1	53754878	G	T
NC_040273.1	53786694	C	T
NC_040273.1	53873103	A	G
NC_040273.1	53931680	T	C
NC_040273.1	53969207	A	C
NC_040273.1	54007436	A	G
NC_040273.1	54122594	G	C
NC_040273.1	54179625	T	C
NC_040273.1	54236611	A	G
NC_040273.1	54301322	G	A
NC_040273.1	54351052	T	C
NC_040273.1	54407675	T	C
NC_040273.1	54453837	G	A

NC_040273.1	54476758	A	C
NC_040273.1	54477171	T	C
NC_040273.1	54513959	A	G
NC_040273.1	54543933	A	G
NC_040273.1	54585965	G	A
NC_040273.1	54623909	C	T
NC_040273.1	54624765	T	G
NC_040273.1	54671390	T	C
NC_040273.1	54671894	C	G
NC_040273.1	54672522	A	G
NC_040273.1	54672948	A	G
NC_040273.1	54683884	A	G
NC_040273.1	54684202	A	G
NC_040273.1	54740198	A	G
NC_040273.1	54790911	C	T
NC_040273.1	54846272	A	G
NC_040273.1	54916107	G	C
NC_040273.1	54918916	A	G
NC_040273.1	54950556	A	G
NC_040273.1	54968235	A	G
NC_040273.1	54982433	A	G
NC_040273.1	55000721	C	T
NC_040273.1	55052843	T	C
NC_040273.1	55110316	T	C
NC_040273.1	55159821	A	G
NC_040273.1	55180440	A	G
NC_040273.1	55192674	A	G
NC_040273.1	55209164	C	G
NC_040273.1	55239745	G	T
NC_040273.1	55265408	A	G
NC_040273.1	55267185	C	A
NC_040273.1	55269227	A	G
NC_040273.1	55288738	C	T
NC_040273.1	55300809	T	C
NC_040273.1	55347212	A	G
NC_040273.1	55354574	T	C
NC_040273.1	55391329	C	T
NC_040273.1	55435222	A	G
NC_040273.1	55437102	T	C
NC_040273.1	55437303	T	C
NC_040273.1	55446509	A	G
NC_040273.1	55514168	A	G
NC_040273.1	55565079	A	T
NC_040273.1	55607737	A	G
NC_040273.1	55614361	A	G
NC_040273.1	55618071	T	C
NC_040273.1	55664590	C	A

NC_040274.1	26905	A	C
NC_040274.1	60154	C	A
NC_040274.1	94436	A	G
NC_040274.1	151860	T	C
NC_040274.1	316412	A	G
NC_040274.1	401141	T	G
NC_040274.1	488091	C	T
NC_040274.1	543937	T	C
NC_040274.1	567789	T	C
NC_040274.1	570137	T	C
NC_040274.1	570303	A	G
NC_040274.1	593743	A	G
NC_040274.1	594368	C	T
NC_040274.1	637692	C	T
NC_040274.1	653831	T	C
NC_040274.1	654390	C	G
NC_040274.1	659631	T	C
NC_040274.1	662617	T	C
NC_040274.1	680101	G	A
NC_040274.1	690959	T	C
NC_040274.1	711164	G	C
NC_040274.1	756044	A	G
NC_040274.1	801160	T	C
NC_040274.1	831929	C	T
NC_040274.1	844682	A	G
NC_040274.1	856834	T	C
NC_040274.1	858107	C	T
NC_040274.1	923513	C	A
NC_040274.1	960092	A	T
NC_040274.1	998951	A	G
NC_040274.1	1025316	G	A
NC_040274.1	1028168	A	G
NC_040274.1	1040673	T	C
NC_040274.1	1071703	T	C
NC_040274.1	1128634	T	C
NC_040274.1	1179702	C	T
NC_040274.1	1212605	G	A
NC_040274.1	1214699	T	C
NC_040274.1	1215257	C	T
NC_040274.1	1215998	A	G
NC_040274.1	1217657	G	T
NC_040274.1	1272413	G	C
NC_040274.1	1333174	T	C
NC_040274.1	1391596	T	C
NC_040274.1	1449150	C	T
NC_040274.1	1500495	G	A
NC_040274.1	1556531	T	G

NC_040274.1	1609500	C	G
NC_040274.1	1650746	C	A
NC_040274.1	1707513	G	A
NC_040274.1	1752302	C	G
NC_040274.1	1807167	T	C
NC_040274.1	1864690	G	A
NC_040274.1	1916404	G	A
NC_040274.1	1970958	G	A
NC_040274.1	2018105	T	C
NC_040274.1	2111238	C	T
NC_040274.1	2170872	C	G
NC_040274.1	2227360	C	T
NC_040274.1	2274310	G	A
NC_040274.1	2317009	A	C
NC_040274.1	2363254	G	T
NC_040274.1	2410038	T	G
NC_040274.1	2410251	A	G
NC_040274.1	2466879	C	G
NC_040274.1	2513110	T	C
NC_040274.1	2550054	A	G
NC_040274.1	2583056	A	G
NC_040274.1	2590950	G	A
NC_040274.1	2591164	T	C
NC_040274.1	2622634	C	T
NC_040274.1	2628734	T	C
NC_040274.1	2640729	A	G
NC_040274.1	2641184	A	G
NC_040274.1	2696461	G	A
NC_040274.1	2757126	A	G
NC_040274.1	2817457	A	G
NC_040274.1	2876328	C	T
NC_040274.1	2931640	G	A
NC_040274.1	2990741	C	T
NC_040274.1	3047934	G	A
NC_040274.1	3102865	G	A
NC_040274.1	3159309	T	C
NC_040274.1	3214202	A	G
NC_040274.1	3266946	G	A
NC_040274.1	3292759	C	A
NC_040274.1	3340244	G	A
NC_040274.1	3389047	G	A
NC_040274.1	3437098	A	G
NC_040274.1	3494885	A	G
NC_040274.1	3554290	T	C
NC_040274.1	3613352	A	G
NC_040274.1	3654239	T	C
NC_040274.1	3682781	C	T

NC_040274.1	3732218	T	G
NC_040274.1	3739371	T	C
NC_040274.1	3793213	T	C
NC_040274.1	3844121	A	G
NC_040274.1	3896551	C	A
NC_040274.1	3947721	A	G
NC_040274.1	4004289	C	T
NC_040274.1	4061269	T	C
NC_040274.1	4130219	G	A
NC_040274.1	4176463	A	G
NC_040274.1	4227999	C	G
NC_040274.1	4275787	A	G
NC_040274.1	4303286	G	A
NC_040274.1	4363175	A	T
NC_040274.1	4399388	T	C
NC_040274.1	4448951	T	C
NC_040274.1	4493851	T	C
NC_040274.1	4523689	G	A
NC_040274.1	4558526	A	G
NC_040274.1	4601144	T	A
NC_040274.1	4666880	0	A
NC_040274.1	4727501	T	A
NC_040274.1	4793756	T	A
NC_040274.1	4900522	A	T
NC_040274.1	4973625	C	T
NC_040274.1	5055297	C	A
NC_040274.1	5093247	A	G
NC_040274.1	5150056	G	C
NC_040274.1	5204043	T	C
NC_040274.1	5254151	C	A
NC_040274.1	5305508	T	C
NC_040274.1	5362224	T	C
NC_040274.1	5417308	C	T
NC_040274.1	5466193	T	G
NC_040274.1	5567783	A	G
NC_040274.1	5639436	A	C
NC_040274.1	5739621	C	A
NC_040274.1	5781016	A	G
NC_040274.1	5867884	G	A
NC_040274.1	5935883	C	T
NC_040274.1	6058047	G	T
NC_040274.1	6098420	A	G
NC_040274.1	6129896	G	A
NC_040274.1	6192717	G	A
NC_040274.1	6294971	C	T
NC_040274.1	6327579	A	C
NC_040274.1	6393541	G	A

NC_040274.1	6467678	G	A
NC_040274.1	6505845	G	T
NC_040274.1	6547503	G	A
NC_040274.1	6589215	G	A
NC_040274.1	6667865	G	C
NC_040274.1	6720020	A	G
NC_040274.1	6774083	A	G
NC_040274.1	6836764	A	G
NC_040274.1	6975483	A	G
NC_040274.1	7030377	C	T
NC_040274.1	7083152	A	T
NC_040274.1	7112264	C	A
NC_040274.1	7213611	T	C
NC_040274.1	7313515	C	T
NC_040274.1	7605133	G	A
NC_040274.1	7662860	T	C
NC_040274.1	7769864	C	T
NC_040274.1	7874721	T	C
NC_040274.1	7927109	G	A
NC_040274.1	7992028	G	A
NC_040274.1	8033539	A	G
NC_040274.1	8089963	G	A
NC_040274.1	8144745	G	A
NC_040274.1	8204125	C	G
NC_040274.1	8258290	T	C
NC_040274.1	8406464	A	G
NC_040274.1	8521675	T	C
NC_040274.1	8554474	T	C
NC_040274.1	8615424	T	A
NC_040274.1	8881187	T	C
NC_040274.1	8937836	T	A
NC_040274.1	9030339	T	C
NC_040274.1	9124334	T	C
NC_040274.1	9496561	A	G
NC_040274.1	9562549	G	A
NC_040274.1	9612927	T	C
NC_040274.1	9666685	C	T
NC_040274.1	9706899	C	T
NC_040274.1	9756617	T	C
NC_040274.1	9804040	G	A
NC_040274.1	9862415	A	G
NC_040274.1	9889795	T	C
NC_040274.1	9944946	C	T
NC_040274.1	10020099	G	A
NC_040274.1	10052386	T	C
NC_040274.1	10101757	T	C
NC_040274.1	10149965	A	T

NC_040274.1	10187783	T	C
NC_040274.1	10264415	C	G
NC_040274.1	10339548	T	C
NC_040274.1	10373375	A	G
NC_040274.1	10410267	A	G
NC_040274.1	10455465	T	C
NC_040274.1	10523086	C	T
NC_040274.1	10542826	A	G
NC_040274.1	10598396	C	A
NC_040274.1	10631819	T	A
NC_040274.1	10686253	G	A
NC_040274.1	10713513	T	C
NC_040274.1	10814080	G	A
NC_040274.1	10900895	T	G
NC_040274.1	10913359	T	C
NC_040274.1	10987183	C	T
NC_040274.1	11006749	A	G
NC_040274.1	11039881	T	C
NC_040274.1	11161776	G	A
NC_040274.1	11197973	A	G
NC_040274.1	11296563	A	C
NC_040274.1	11344340	C	T
NC_040274.1	11394844	A	G
NC_040274.1	11470978	A	C
NC_040274.1	11531264	A	T
NC_040274.1	11585107	G	A
NC_040274.1	11657878	T	C
NC_040274.1	11724565	G	A
NC_040274.1	11783846	C	T
NC_040274.1	11794905	T	C
NC_040274.1	11833058	A	G
NC_040274.1	12035468	G	A
NC_040274.1	12108943	C	A
NC_040274.1	12185499	T	C
NC_040274.1	12253923	G	T
NC_040274.1	12308113	A	T
NC_040274.1	12340281	C	T
NC_040274.1	12380862	G	A
NC_040274.1	12422866	C	T
NC_040274.1	12461225	C	A
NC_040274.1	12560399	G	A
NC_040274.1	12595826	G	A
NC_040274.1	12651347	T	G
NC_040274.1	13041249	A	G
NC_040274.1	13147778	A	G
NC_040274.1	13201869	G	C
NC_040274.1	13281097	T	C

NC_040274.1	13494250	G	A
NC_040274.1	13540470	0	T
NC_040274.1	13610104	A	G
NC_040274.1	13782137	G	A
NC_040274.1	13838149	A	G
NC_040274.1	13883842	G	T
NC_040274.1	13948009	G	T
NC_040274.1	13999454	T	C
NC_040274.1	14038121	T	C
NC_040274.1	14076442	T	C
NC_040274.1	14133848	A	G
NC_040274.1	14182917	G	A
NC_040274.1	14212124	G	A
NC_040274.1	14338866	A	C
NC_040274.1	14386322	T	C
NC_040274.1	14606554	C	G
NC_040274.1	14668222	A	G
NC_040274.1	14793250	C	T
NC_040274.1	15010056	T	A
NC_040274.1	15029123	T	C
NC_040274.1	15113195	A	G
NC_040274.1	15137535	A	T
NC_040274.1	15369725	C	T
NC_040274.1	15416102	C	G
NC_040274.1	15480188	T	C
NC_040274.1	15682487	C	G
NC_040274.1	15771695	A	C
NC_040274.1	15894502	T	C
NC_040274.1	15947069	T	C
NC_040274.1	15999431	T	A
NC_040274.1	16058694	G	T
NC_040274.1	16112722	G	A
NC_040274.1	16217839	G	C
NC_040274.1	16275653	C	T
NC_040274.1	16309289	C	G
NC_040274.1	16355491	G	C
NC_040274.1	16457998	C	A
NC_040274.1	16511606	T	C
NC_040274.1	16557852	T	C
NC_040274.1	16619080	G	A
NC_040274.1	16665292	G	A
NC_040274.1	16723537	T	C
NC_040274.1	16772777	C	T
NC_040274.1	16816730	T	A
NC_040274.1	16874446	T	C
NC_040274.1	16996015	A	T
NC_040274.1	17043457	C	T

NC_040274.1	17100237	A	G
NC_040274.1	17129106	C	T
NC_040274.1	17295586	T	G
NC_040274.1	17347476	G	C
NC_040274.1	17447955	G	A
NC_040274.1	17535275	T	A
NC_040274.1	17720781	T	C
NC_040274.1	17741909	C	T
NC_040274.1	17835618	T	C
NC_040274.1	17911379	C	T
NC_040274.1	17984374	T	C
NC_040274.1	18069096	C	G
NC_040274.1	18113602	C	T
NC_040274.1	18183335	T	A
NC_040274.1	18224899	T	C
NC_040274.1	18290278	C	T
NC_040274.1	18681093	A	G
NC_040274.1	18735306	C	T
NC_040274.1	18769654	A	G
NC_040274.1	18816833	T	C
NC_040274.1	18891247	C	T
NC_040274.1	19384354	A	G
NC_040274.1	19445418	G	A
NC_040274.1	19537337	A	G
NC_040274.1	19597244	A	G
NC_040274.1	19726476	A	T
NC_040274.1	19791873	T	C
NC_040274.1	19863395	A	G
NC_040274.1	19929429	A	G
NC_040274.1	19988302	T	C
NC_040274.1	20081797	G	C
NC_040274.1	20119780	G	A
NC_040274.1	20203265	G	A
NC_040274.1	20255502	A	G
NC_040274.1	20283035	G	A
NC_040274.1	20340094	A	C
NC_040274.1	20392274	A	G
NC_040274.1	20455894	T	C
NC_040274.1	20490047	G	A
NC_040274.1	20554139	G	A
NC_040274.1	20611870	G	A
NC_040274.1	20667601	A	T
NC_040274.1	20721760	T	C
NC_040274.1	20781467	C	T
NC_040274.1	20818864	A	T
NC_040274.1	20894404	A	C
NC_040274.1	20946266	C	G

NC_040274.1	21023514	T	C
NC_040274.1	21090603	C	T
NC_040274.1	21130374	C	T
NC_040274.1	21185009	G	A
NC_040274.1	21235864	T	C
NC_040274.1	21271694	A	C
NC_040274.1	21397951	C	T
NC_040274.1	21447349	A	G
NC_040274.1	21500207	A	G
NC_040274.1	21567873	T	C
NC_040274.1	21633393	T	C
NC_040274.1	21679156	G	A
NC_040274.1	21736803	A	G
NC_040274.1	21777721	C	T
NC_040274.1	21904395	C	G
NC_040274.1	21961263	T	C
NC_040274.1	22024117	C	T
NC_040274.1	22053057	T	G
NC_040274.1	22100874	A	G
NC_040274.1	22158151	A	G
NC_040274.1	22220564	T	C
NC_040274.1	22274118	T	C
NC_040274.1	22425653	A	G
NC_040274.1	22480831	T	C
NC_040274.1	22508585	G	A
NC_040274.1	22578265	G	A
NC_040274.1	22645442	G	C
NC_040274.1	22719374	G	A
NC_040274.1	22795467	G	A
NC_040274.1	22929964	A	G
NC_040274.1	22987242	G	A
NC_040274.1	23022867	C	G
NC_040274.1	23081477	A	G
NC_040274.1	23138621	A	G
NC_040274.1	23191942	A	G
NC_040274.1	23249614	T	G
NC_040274.1	23303908	A	G
NC_040274.1	23365564	T	C
NC_040274.1	23414668	C	T
NC_040274.1	23456252	C	G
NC_040274.1	23496885	C	T
NC_040274.1	23543650	C	T
NC_040274.1	23591657	C	T
NC_040274.1	23622554	G	A
NC_040274.1	23676823	C	T
NC_040274.1	23742787	C	G
NC_040274.1	23796618	A	G

NC_040274.1	23858884	C	T
NC_040274.1	24077847	C	T
NC_040274.1	24122893	C	T
NC_040274.1	24163598	A	C
NC_040274.1	24224829	C	T
NC_040274.1	24264673	A	T
NC_040274.1	24307196	G	A
NC_040274.1	24363033	C	T
NC_040274.1	24427801	C	T
NC_040274.1	24482172	A	G
NC_040274.1	24536374	T	C
NC_040274.1	24589504	T	A
NC_040274.1	24695052	G	A
NC_040274.1	24751061	T	A
NC_040274.1	24818792	A	G
NC_040274.1	24897416	G	A
NC_040274.1	24965226	G	A
NC_040274.1	25011945	A	G
NC_040274.1	25104326	C	T
NC_040274.1	25153119	G	A
NC_040274.1	25212805	A	T
NC_040274.1	25256473	C	T
NC_040274.1	25318664	C	T
NC_040274.1	25395969	C	T
NC_040274.1	25450584	G	A
NC_040274.1	25494438	T	C
NC_040274.1	25684635	G	T
NC_040274.1	25736975	T	A
NC_040274.1	25780768	0	G
NC_040274.1	25852671	C	T
NC_040274.1	25906191	T	C
NC_040274.1	25962642	T	C
NC_040274.1	26015997	C	T
NC_040274.1	26071172	G	A
NC_040274.1	26127236	A	G
NC_040274.1	26199902	C	T
NC_040274.1	26258171	G	A
NC_040274.1	26302850	T	G
NC_040274.1	26349808	T	C
NC_040274.1	26420759	C	T
NC_040274.1	26437900	G	A
NC_040274.1	26490922	A	G
NC_040274.1	26537333	A	C
NC_040274.1	26608495	T	C
NC_040274.1	26671025	A	G
NC_040274.1	26720220	C	T
NC_040274.1	26762553	C	A

NC_040274.1	26803440	C	A
NC_040274.1	26866304	C	T
NC_040274.1	26938007	G	C
NC_040274.1	27035204	G	C
NC_040274.1	27069027	G	A
NC_040274.1	27133053	C	A
NC_040274.1	27162684	T	C
NC_040274.1	27212545	G	A
NC_040274.1	27275807	G	A
NC_040274.1	27455168	A	G
NC_040274.1	27478040	A	C
NC_040274.1	27777464	T	C
NC_040274.1	27977306	T	C
NC_040274.1	28016801	A	G
NC_040274.1	28023562	G	A
NC_040274.1	28079132	A	G
NC_040274.1	28133286	C	A
NC_040274.1	28182055	A	C
NC_040274.1	28219993	G	A
NC_040274.1	28271603	C	T
NC_040274.1	28312037	C	T
NC_040274.1	28358923	T	G
NC_040274.1	28389174	G	A
NC_040274.1	28460296	C	G
NC_040274.1	28503338	G	A
NC_040274.1	28557106	A	T
NC_040274.1	28654292	G	A
NC_040274.1	28708932	T	C
NC_040274.1	28740617	G	A
NC_040274.1	28787467	A	G
NC_040274.1	28842673	A	G
NC_040274.1	28890870	C	T
NC_040274.1	28946006	G	A
NC_040274.1	29002633	T	C
NC_040274.1	29057600	C	T
NC_040274.1	29110903	G	A
NC_040274.1	29166758	T	C
NC_040274.1	29224393	A	G
NC_040274.1	29289352	G	A
NC_040274.1	29340608	T	C
NC_040274.1	29397146	T	C
NC_040274.1	29426409	T	C
NC_040274.1	29477496	G	T
NC_040274.1	29540904	A	C
NC_040274.1	29577329	G	T
NC_040274.1	29632187	T	C
NC_040274.1	29683595	G	A

NC_040274.1	29740287	C	T
NC_040274.1	29776934	G	A
NC_040274.1	29807383	C	T
NC_040274.1	29871455	T	G
NC_040274.1	29930311	A	G
NC_040274.1	30034801	T	C
NC_040274.1	30072735	A	T
NC_040274.1	30119588	C	G
NC_040274.1	30162891	C	T
NC_040274.1	30194694	C	A
NC_040274.1	30199346	A	G
NC_040274.1	30269065	C	T
NC_040274.1	30309875	G	A
NC_040274.1	30376300	T	C
NC_040274.1	30438079	C	T
NC_040274.1	30488504	T	G
NC_040274.1	30523454	C	G
NC_040274.1	30575797	T	C
NC_040274.1	30631024	C	T
NC_040274.1	30685073	A	C
NC_040274.1	30812674	C	T
NC_040274.1	30870007	A	T
NC_040274.1	30923384	A	G
NC_040274.1	30949787	C	T
NC_040274.1	31011418	A	T
NC_040274.1	31045907	T	A
NC_040274.1	31109446	A	G
NC_040274.1	31184800	T	G
NC_040274.1	31252081	T	C
NC_040274.1	31310509	G	A
NC_040274.1	31367776	T	C
NC_040274.1	31428628	G	A
NC_040274.1	31477239	G	A
NC_040274.1	31624273	G	T
NC_040274.1	31714009	C	T
NC_040274.1	31942069	T	C
NC_040274.1	31965671	C	T
NC_040274.1	32030571	T	C
NC_040274.1	32059775	T	C
NC_040274.1	32118726	C	T
NC_040274.1	32292276	G	T
NC_040274.1	32343947	C	T
NC_040274.1	32401374	G	C
NC_040274.1	32453246	C	T
NC_040274.1	32518222	G	A
NC_040274.1	32608283	T	A
NC_040274.1	32668003	A	T

NC_040274.1	32725065	G	A
NC_040274.1	32778063	C	T
NC_040274.1	32814045	T	C
NC_040274.1	32874951	C	T
NC_040274.1	32928918	A	G
NC_040274.1	32983021	C	A
NC_040274.1	33020135	G	A
NC_040274.1	33071018	T	C
NC_040274.1	33134385	T	G
NC_040274.1	33197812	A	G
NC_040274.1	33248167	C	T
NC_040274.1	33292110	G	A
NC_040274.1	33365481	A	G
NC_040274.1	33424982	G	A
NC_040274.1	33467411	C	T
NC_040274.1	33514508	T	C
NC_040274.1	33541435	A	T
NC_040274.1	33627178	T	C
NC_040274.1	33681311	T	C
NC_040274.1	33747167	G	T
NC_040274.1	33788259	G	A
NC_040274.1	33843211	A	G
NC_040274.1	33903128	G	A
NC_040274.1	33967665	G	A
NC_040274.1	34023771	A	G
NC_040274.1	34086513	T	G
NC_040274.1	34154036	C	T
NC_040274.1	34205699	G	A
NC_040274.1	34347306	A	G
NC_040274.1	34390515	T	G
NC_040274.1	34456943	T	C
NC_040274.1	34511167	A	G
NC_040274.1	34556205	C	T
NC_040274.1	34687770	A	G
NC_040274.1	34733274	T	C
NC_040274.1	34779543	T	C
NC_040274.1	34819134	T	C
NC_040274.1	34876271	C	T
NC_040274.1	34939798	G	A
NC_040274.1	34986215	C	G
NC_040274.1	35026299	C	T
NC_040274.1	35088187	C	T
NC_040274.1	35116966	A	G
NC_040274.1	35175868	G	A
NC_040274.1	35222073	T	C
NC_040274.1	35275385	A	G
NC_040274.1	35330839	G	T

NC_040274.1	35381385	T	C
NC_040274.1	35434573	G	A
NC_040274.1	35488672	A	G
NC_040274.1	35542864	T	C
NC_040274.1	35597940	G	A
NC_040274.1	35653028	A	G
NC_040274.1	35706963	T	C
NC_040274.1	35765266	G	A
NC_040274.1	35819595	A	G
NC_040274.1	35871997	T	C
NC_040274.1	35931368	A	G
NC_040274.1	35995857	G	A
NC_040274.1	36036727	A	G
NC_040274.1	36086765	A	C
NC_040274.1	36152003	C	G
NC_040274.1	36203312	G	A
NC_040274.1	36247550	T	C
NC_040274.1	36288823	T	C
NC_040274.1	36344071	T	G
NC_040274.1	36388661	T	C
NC_040274.1	36445508	A	G
NC_040274.1	36495833	C	T
NC_040274.1	36549380	A	G
NC_040274.1	36622245	C	T
NC_040274.1	36694628	G	A
NC_040274.1	36745215	T	C
NC_040274.1	36818353	A	G
NC_040274.1	36874022	T	G
NC_040274.1	36925478	A	G
NC_040274.1	36974665	A	G
NC_040274.1	37041456	C	T
NC_040274.1	37098906	T	C
NC_040274.1	37167210	A	C
NC_040274.1	37220415	G	C
NC_040274.1	37266657	G	A
NC_040274.1	37321870	G	A
NC_040274.1	37324763	C	G
NC_040274.1	37331461	G	C
NC_040274.1	37379397	A	G
NC_040274.1	37432500	C	G
NC_040274.1	37486352	G	A
NC_040274.1	37510251	C	T
NC_040274.1	37517184	G	C
NC_040274.1	37525650	A	C
NC_040274.1	37579378	A	G
NC_040274.1	37631186	T	C
NC_040274.1	37660863	G	A

NC_040274.1	37735718	C	T
NC_040274.1	37795733	G	A
NC_040274.1	37857974	T	C
NC_040274.1	37916546	A	G
NC_040274.1	37920763	T	C
NC_040274.1	37964052	G	A
NC_040274.1	38022299	G	A
NC_040274.1	38083543	C	G
NC_040274.1	38137471	T	C
NC_040274.1	38191061	G	C
NC_040274.1	38256656	G	T
NC_040274.1	38313362	A	C
NC_040274.1	38364008	C	T
NC_040274.1	38414643	G	A
NC_040274.1	38468958	A	G
NC_040274.1	38529130	T	C
NC_040274.1	38579811	T	C
NC_040274.1	38639542	T	C
NC_040274.1	38693917	T	A
NC_040274.1	38757755	T	C
NC_040274.1	38814290	T	C
NC_040274.1	38882120	G	A
NC_040274.1	38943894	A	G
NC_040274.1	38962219	A	G
NC_040274.1	39018272	C	T
NC_040274.1	39073322	T	A
NC_040274.1	39136071	G	C
NC_040274.1	39200292	C	T
NC_040274.1	39215485	G	A
NC_040274.1	39217632	G	A
NC_040274.1	39219506	A	G
NC_040274.1	39256154	T	C
NC_040274.1	39312205	G	A
NC_040274.1	39360194	C	T
NC_040274.1	39382747	G	A
NC_040274.1	39417031	G	A
NC_040274.1	39417791	T	C
NC_040274.1	39418557	T	A
NC_040274.1	39443162	C	A
NC_040274.1	39448815	G	C
NC_040274.1	39451071	T	C
NC_040274.1	39455234	A	G
NC_040274.1	39465391	A	G
NC_040274.1	39469119	A	G
NC_040274.1	39538106	A	G
NC_040274.1	39557409	T	C
NC_040274.1	39557830	T	G

NC_040274.1	39614769	T	C
NC_040274.1	39671123	T	C
NC_040274.1	39723392	T	C
NC_040274.1	39770025	A	G
NC_040274.1	39816519	G	A
NC_040274.1	39842198	T	C
NC_040274.1	39862057	A	T
NC_040274.1	39898712	T	C
NC_040274.1	39906173	C	T
NC_040274.1	39906658	C	T
NC_040274.1	39908396	A	G
NC_040274.1	39926035	A	G
NC_040274.1	39941099	A	G
NC_040274.1	39941812	G	T
NC_040274.1	39947340	G	A
NC_040274.1	39955023	C	T
NC_040274.1	39957942	T	G
NC_040274.1	39962895	T	C
NC_040274.1	39986439	C	T
NC_040274.1	40041734	T	C
NC_040274.1	40097876	C	T
NC_040274.1	40155615	A	G
NC_040274.1	40211140	C	A
NC_040274.1	40266773	G	A
NC_040274.1	40311196	A	G
NC_040274.1	40338727	T	C
NC_040274.1	40343715	A	G
NC_040274.1	40344920	A	G
NC_040274.1	40373267	G	A
NC_040274.1	40397122	A	G
NC_040274.1	40436916	A	G
NC_040274.1	40494262	C	T
NC_040274.1	40529617	C	T
NC_040274.1	40624121	C	A
NC_040274.1	40718347	C	T
NC_040274.1	40775896	G	A
NC_040274.1	40830393	G	C
NC_040274.1	40881359	G	T
NC_040274.1	40938683	G	A
NC_040274.1	40989930	G	A
NC_040274.1	41050160	T	G
NC_040274.1	41113482	T	C
NC_040274.1	41161101	C	T
NC_040274.1	41309158	C	A
NC_040274.1	41364884	C	T
NC_040274.1	41421948	T	C
NC_040274.1	41452851	C	T

NC_040274.1	41508193	C	T
NC_040274.1	41600037	G	A
NC_040274.1	41664867	C	T
NC_040274.1	41721069	T	C
NC_040274.1	41763092	C	A
NC_040274.1	41815604	A	T
NC_040274.1	41872023	A	G
NC_040274.1	41928407	A	C
NC_040274.1	41981803	A	G
NC_040274.1	42040303	A	G
NC_040274.1	42095107	C	T
NC_040274.1	42098502	T	C
NC_040274.1	42146689	G	A
NC_040274.1	42173970	G	A
NC_040274.1	42193588	G	A
NC_040274.1	42198865	G	A
NC_040274.1	42212719	C	A
NC_040274.1	42226649	G	C
NC_040274.1	42252978	C	A
NC_040274.1	42305492	T	C
NC_040274.1	42314781	C	T
NC_040274.1	42330421	A	G
NC_040274.1	42337046	G	A
NC_040274.1	42339401	A	G
NC_040274.1	42382343	G	C
NC_040274.1	42444068	T	C
NC_040274.1	42495413	A	G
NC_040274.1	42551091	G	A
NC_040274.1	42598801	G	A
NC_040274.1	42649571	A	G
NC_040274.1	42703695	G	A
NC_040274.1	42758492	A	G
NC_040274.1	42790127	C	T
NC_040274.1	42821251	C	T
NC_040274.1	42875767	T	C
NC_040274.1	42932349	T	C
NC_040274.1	42982929	G	T
NC_040274.1	43026657	T	C
NC_040274.1	43059243	A	C
NC_040274.1	43109768	T	C
NC_040274.1	43168006	A	G
NC_040274.1	43221150	A	G
NC_040274.1	43279790	A	G
NC_040274.1	43338908	A	T
NC_040274.1	43394940	A	G
NC_040274.1	43456471	A	C
NC_040274.1	43513063	A	G

NC_040274.1	43561263	G	A
NC_040274.1	43616089	T	C
NC_040274.1	43670839	A	G
NC_040274.1	43714553	T	C
NC_040274.1	43768320	A	G
NC_040274.1	43820249	T	A
NC_040274.1	43870904	T	G
NC_040274.1	43923193	A	G
NC_040274.1	43965865	C	T
NC_040274.1	44021957	C	T
NC_040274.1	44051532	A	G
NC_040274.1	44054046	T	C
NC_040274.1	44064562	T	C
NC_040274.1	44095088	A	G
NC_040274.1	44151315	A	C
NC_040274.1	44209216	A	G
NC_040274.1	44266435	C	T
NC_040274.1	44319286	G	A
NC_040274.1	44382063	A	C
NC_040274.1	44436639	T	C
NC_040274.1	44489921	A	G
NC_040274.1	44542762	T	C
NC_040274.1	44593170	C	T
NC_040274.1	44649195	G	A
NC_040274.1	44706145	A	G
NC_040274.1	44739001	G	A
NC_040274.1	44795291	A	G
NC_040274.1	44851018	A	G
NC_040274.1	44906052	G	A
NC_040274.1	44960360	A	G
NC_040274.1	44987014	C	G
NC_040274.1	45045389	A	G
NC_040274.1	45097089	T	C
NC_040274.1	45126668	T	C
NC_040274.1	45127321	C	T
NC_040274.1	45149401	C	T
NC_040274.1	45159625	G	A
NC_040274.1	45192800	C	G
NC_040274.1	45193924	A	C
NC_040274.1	45224985	C	T
NC_040274.1	45267790	G	T
NC_040274.1	45332856	G	A
NC_040274.1	45383317	T	C
NC_040274.1	45439672	G	T
NC_040274.1	45492836	G	A
NC_040274.1	45534185	A	C
NC_040274.1	45589295	T	C

NC_040274.1	45648801	A	T
NC_040274.1	45707623	A	G
NC_040274.1	45764523	T	A
NC_040274.1	45816811	A	G
NC_040274.1	45874244	T	C
NC_040274.1	45923331	T	C
NC_040274.1	45948827	T	C
NC_040274.1	45978423	A	G
NC_040274.1	46030195	G	A
NC_040274.1	46074362	G	A
NC_040274.1	46130947	T	C
NC_040274.1	46184667	T	C
NC_040274.1	46241482	G	T
NC_040274.1	46281523	T	C
NC_040274.1	46291105	A	G
NC_040274.1	46328509	T	C
NC_040274.1	46328821	A	C
NC_040274.1	46370793	G	A
NC_040274.1	46399703	A	C
NC_040274.1	46428894	T	C
NC_040274.1	46429057	T	C
NC_040274.1	46439049	T	C
NC_040274.1	46499828	T	C
NC_040274.1	46553597	A	C
NC_040274.1	46607957	A	G
NC_040274.1	46625799	C	T
NC_040274.1	46644843	T	C
NC_040274.1	46645933	G	T
NC_040274.1	46646253	C	T
NC_040274.1	46701286	A	G
NC_040274.1	46725282	G	A
NC_040274.1	46725517	A	G
NC_040274.1	46730630	C	G
NC_040274.1	46732068	C	T
NC_040274.1	46732253	A	G
NC_040274.1	46732886	T	C
NC_040274.1	46733138	T	C
NC_040274.1	46733888	A	G
NC_040274.1	46745049	A	T
NC_040274.1	46756516	G	C
NC_040274.1	46773983	A	G
NC_040274.1	46775553	T	C
NC_040274.1	46776181	G	T
NC_040274.1	46827410	C	T
NC_040274.1	46875111	A	G
NC_040274.1	46875545	C	T
NC_040274.1	46881149	C	T

NC_040274.1	46895036	T	C
NC_040274.1	46950765	C	G
NC_040274.1	47007897	T	C
NC_040274.1	47042562	T	C
NC_040274.1	47077374	A	G
NC_040274.1	47079636	T	C
NC_040274.1	47080608	G	A
NC_040274.1	47128335	G	A
NC_040274.1	47143090	A	G
NC_040274.1	47143674	A	G
NC_040274.1	47200377	G	T
NC_040274.1	47256340	T	C
NC_040274.1	47311206	T	C
NC_040274.1	47355833	A	G
NC_040274.1	47356168	A	G
NC_040274.1	47369179	T	C
NC_040274.1	47370152	A	G
NC_040274.1	47370439	A	G
NC_040274.1	47370672	A	G
NC_040274.1	47370905	T	G
NC_040274.1	47395960	G	A
NC_040274.1	47396389	T	C
NC_040274.1	47396788	T	C
NC_040274.1	47397223	A	G
NC_040274.1	47451764	C	T
NC_040274.1	47506795	T	C
NC_040274.1	47563212	T	C
NC_040274.1	47616665	C	T
NC_040274.1	47671469	T	C
NC_040274.1	47724018	A	G
NC_040274.1	47773855	G	A
NC_040274.1	47828385	A	G
NC_040274.1	47891135	A	G
NC_040274.1	47950522	C	A
NC_040274.1	48172897	T	C
NC_040274.1	48198322	A	G
NC_040274.1	48208074	G	A
NC_040274.1	48212150	C	A
NC_040274.1	48237988	T	C
NC_040274.1	48247892	A	G
NC_040274.1	48260335	A	G
NC_040274.1	48261036	A	G
NC_040274.1	48268776	T	A
NC_040274.1	48272979	T	C
NC_040274.1	48273993	C	G
NC_040274.1	48288089	C	T
NC_040274.1	48303579	C	T

NC_040274.1	48367199	A	T
NC_040274.1	48368435	T	C
NC_040274.1	48368593	T	C
NC_040274.1	48370726	T	C
NC_040274.1	48375576	A	G
NC_040274.1	48431099	T	C
NC_040274.1	48487808	T	C
NC_040274.1	48517731	G	A
NC_040274.1	48518135	C	T
NC_040274.1	48518709	C	T
NC_040274.1	48558972	A	G
NC_040274.1	48565193	T	C
NC_040274.1	48569389	C	T
NC_040274.1	48624190	T	C
NC_040274.1	48656198	T	C
NC_040274.1	48674780	G	A
NC_040274.1	48675632	G	A
NC_040274.1	48679226	G	C
NC_040274.1	48711447	C	T
NC_040274.1	48714353	C	A
NC_040274.1	48769911	C	A
NC_040274.1	48826588	C	T
NC_040274.1	48881405	C	T
NC_040274.1	48923025	T	C
NC_040274.1	48924158	A	G
NC_040274.1	48926290	C	G
NC_040274.1	48928210	A	C
NC_040274.1	48928366	C	T
NC_040274.1	48928727	A	G
NC_040274.1	48932276	G	C
NC_040274.1	48933016	T	C
NC_040274.1	48936077	A	T
NC_040274.1	48945507	G	A
NC_040274.1	48945901	C	G
NC_040274.1	48991740	A	G
NC_040274.1	49015431	T	G
NC_040274.1	49015754	A	G
NC_040274.1	49015986	T	C
NC_040274.1	49067868	G	A
NC_040274.1	49124063	T	C
NC_040274.1	49176253	C	T
NC_040274.1	49231517	A	T
NC_040274.1	49289544	T	C
NC_040274.1	49348473	C	A
NC_040274.1	49394105	A	G
NC_040274.1	49446178	G	T
NC_040274.1	49503598	G	A

NC_040274.1	49563295	G	A
NC_040274.1	49617969	T	C
NC_040274.1	49669757	A	G
NC_040274.1	49707965	A	G
NC_040274.1	49762413	C	T
NC_040274.1	49815577	A	G
NC_040274.1	49871086	T	G
NC_040274.1	49922487	G	A
NC_040274.1	49976847	G	A
NC_040274.1	49977384	T	C
NC_040274.1	49978005	T	C
NC_040274.1	49978452	C	T
NC_040274.1	50034991	A	G
NC_040274.1	50089178	G	A
NC_040274.1	50089843	T	G
NC_040274.1	50090153	G	A
NC_040274.1	50092514	T	C
NC_040274.1	50092679	T	C
NC_040274.1	50149495	T	G
NC_040274.1	50204549	T	C
NC_040274.1	50257674	T	G
NC_040274.1	50310591	T	C
NC_040274.1	50374321	T	C
NC_040274.1	50426392	T	C
NC_040274.1	50485354	C	T
NC_040274.1	50540548	T	C
NC_040274.1	50572456	G	C
NC_040274.1	50674049	A	G
NC_040274.1	50730059	C	T
NC_040274.1	50787341	C	T
NC_040274.1	50823228	C	T
NC_040274.1	50876796	T	C
NC_040274.1	50930874	A	G
NC_040274.1	50973056	C	T
NC_040274.1	51022775	A	G
NC_040274.1	51022943	C	G
NC_040274.1	51023097	A	G
NC_040274.1	51024677	T	C
NC_040274.1	51029153	C	T
NC_040274.1	51042602	A	C
NC_040274.1	51042858	A	G
NC_040274.1	51043040	A	G
NC_040274.1	51099862	G	T
NC_040274.1	51159767	C	T
NC_040274.1	51196802	C	T
NC_040274.1	51197180	T	C
NC_040274.1	51248887	G	T

NC_040274.1	51306273	C	G
NC_040274.1	51364733	A	G
NC_040274.1	51424718	A	G
NC_040274.1	51447682	G	A
NC_040274.1	51450084	T	C
NC_040274.1	51508118	A	C
NC_040274.1	51565211	A	G
NC_040274.1	51607031	T	C
NC_040274.1	51664737	G	A
NC_040274.1	51726924	T	C
NC_040274.1	51787213	T	C
NC_040274.1	51855562	T	C
NC_040274.1	51911368	G	A
NC_040274.1	51971110	C	T
NC_040274.1	52029407	A	T
NC_040274.1	52072051	T	C
NC_040274.1	52126704	T	C
NC_040274.1	52176950	A	C
NC_040274.1	52231777	A	G
NC_040274.1	52253682	C	T
NC_040274.1	52255079	A	C
NC_040274.1	52309754	A	C
NC_040274.1	52339353	C	T
NC_040274.1	52340250	T	C
NC_040274.1	52341354	G	A
NC_040274.1	52398172	C	T
NC_040274.1	52446537	G	A
NC_040274.1	52505544	G	A
NC_040274.1	52558712	T	C
NC_040274.1	52611257	G	A
NC_040274.1	52671980	G	T
NC_040274.1	52729415	C	T
NC_040274.1	52786393	G	T
NC_040274.1	52837565	G	A
NC_040274.1	52905663	T	C
NC_040274.1	52956575	A	G
NC_040274.1	52997216	C	T
NC_040274.1	53056544	A	G
NC_040274.1	53101618	T	C
NC_040274.1	53102026	G	T
NC_040274.1	53102255	C	G
NC_040274.1	53104062	T	C
NC_040274.1	53117462	T	C
NC_040274.1	53173120	G	A
NC_040274.1	53211916	C	G
NC_040274.1	53212131	C	T
NC_040274.1	53212868	T	C

NC_040274.1	53213499	A	G
NC_040274.1	53265568	T	A
NC_040274.1	53303808	G	A
NC_040274.1	53359222	A	G
NC_040274.1	53409184	C	T
NC_040274.1	53467714	G	A
NC_040274.1	53523830	C	G
NC_040274.1	53568259	T	C
NC_040274.1	53636248	T	C
NC_040274.1	53693213	T	C
NC_040274.1	53748656	A	G
NC_040274.1	53802571	T	C
NC_040274.1	53858575	T	C
NC_040274.1	53915088	G	A
NC_040274.1	53966318	A	G
NC_040274.1	53966531	A	G
NC_040274.1	54021718	T	C
NC_040274.1	54021934	T	C
NC_040274.1	54046678	C	T
NC_040274.1	54096109	A	G
NC_040274.1	54141880	C	G
NC_040274.1	54142359	C	T
NC_040274.1	54143406	C	T
NC_040274.1	54202705	C	T
NC_040274.1	54254383	A	T
NC_040274.1	54310392	G	A
NC_040274.1	54363507	T	C
NC_040274.1	54419442	A	G
NC_040274.1	54483649	A	T
NC_040274.1	54538334	C	T
NC_040274.1	54573582	C	T
NC_040274.1	54610136	A	C
NC_040274.1	54615747	C	A
NC_040274.1	54670378	A	G
NC_040274.1	54736224	C	T
NC_040274.1	54792959	T	C
NC_040274.1	54842366	A	G
NC_040274.1	54851013	G	C
NC_040274.1	54888624	A	G
NC_040274.1	54905628	C	G
NC_040274.1	54928711	T	C
NC_040274.1	54976539	A	G
NC_040274.1	55034292	A	G
NC_040274.1	55091807	A	G
NC_040274.1	55145684	C	T
NC_040274.1	55212521	A	C
NC_040274.1	55261462	T	A

NC_040274.1	55281786	T	C
NC_040274.1	55302447	C	T
NC_040274.1	55302686	T	C
NC_040274.1	55304138	G	A
NC_040274.1	55306670	C	T
NC_040274.1	55372567	G	A
NC_040274.1	55426406	T	C
NC_040274.1	55471408	G	A
NC_040274.1	55527158	A	G
NC_040274.1	55595722	A	T
NC_040274.1	55654307	A	G
NC_040274.1	55714794	T	C
NC_040274.1	55759901	A	G
NC_040274.1	55811418	T	C
NC_040274.1	55841930	G	C
NC_040274.1	55869420	C	A
NC_040274.1	55894771	T	C
NC_040274.1	55894990	T	C
NC_040274.1	55920161	C	T
NC_040274.1	55923737	A	G
NC_040274.1	55924064	C	A
NC_040274.1	55982404	A	G
NC_040274.1	56013071	T	G
NC_040274.1	56013227	T	C
NC_040274.1	56013489	G	T
NC_040274.1	56013783	T	A
NC_040274.1	56014791	T	C
NC_040274.1	56015471	G	T
NC_040274.1	56015796	C	T
NC_040274.1	56015947	A	C
NC_040274.1	56081353	A	G
NC_040274.1	56098859	A	G
NC_040274.1	56153863	T	C
NC_040274.1	56211554	A	G
NC_040274.1	56269939	T	C
NC_040274.1	56322138	G	A
NC_040274.1	56377104	C	A
NC_040274.1	56436322	C	A
NC_040274.1	56489628	C	T
NC_040274.1	56518673	T	C
NC_040274.1	56758254	C	G
NC_040274.1	56812729	C	A
NC_040274.1	56867913	T	C
NC_040274.1	56929304	T	C
NC_040274.1	56971724	C	G
NC_040274.1	57028260	T	C
NC_040274.1	57076649	G	A

NC_040274.1	57146256	G	A
NC_040274.1	57200893	G	A
NC_040274.1	57257299	G	C
NC_040274.1	57328395	T	G
NC_040274.1	57383949	C	T
NC_040274.1	57445688	T	A
NC_040274.1	57502958	C	T
NC_040274.1	57555879	A	G
NC_040274.1	57615470	T	C
NC_040274.1	57682889	T	C
NC_040274.1	57734252	G	C
NC_040274.1	57796140	T	G
NC_040274.1	57848987	C	T
NC_040274.1	57914006	A	G
NC_040274.1	57971745	T	C
NC_040274.1	58023995	T	G
NC_040274.1	58078423	T	C
NC_040274.1	58135427	A	G
NC_040274.1	58164052	T	C
NC_040274.1	58231675	G	A
NC_040274.1	58331331	C	T
NC_040274.1	58386589	A	G
NC_040274.1	58439228	C	T
NC_040274.1	58495560	G	A
NC_040274.1	58537918	G	A
NC_040274.1	58602342	A	T
NC_040274.1	58658222	G	A
NC_040274.1	58724395	C	A
NC_040274.1	58781537	G	A
NC_040274.1	58821885	A	G
NC_040274.1	58877534	C	T
NC_040274.1	58914512	T	C
NC_040274.1	58961633	0	C
NC_040274.1	59006839	G	T
NC_040274.1	59079365	A	G
NC_040274.1	59127824	G	A
NC_040274.1	59185998	A	G
NC_040274.1	59234764	T	C
NC_040274.1	59298065	A	G
NC_040274.1	59355617	G	A
NC_040274.1	59396972	G	T
NC_040274.1	59434575	T	C
NC_040274.1	59490455	C	T
NC_040274.1	59550242	C	T
NC_040274.1	59614153	A	G
NC_040274.1	59673371	T	C
NC_040274.1	59728042	T	A

NC_040274.1	59785867	G	C
NC_040274.1	59841663	C	T
NC_040274.1	59919809	G	C
NC_040274.1	59956073	T	C
NC_040274.1	60097322	A	G
NC_040274.1	60146379	T	A
NC_040274.1	60184335	C	T
NC_040274.1	60232616	T	C
NC_040274.1	60234531	T	A
NC_040274.1	60234770	G	A
NC_040274.1	60240009	T	C
NC_040274.1	60288189	C	T
NC_040274.1	60351424	T	C
NC_040274.1	60405091	G	C
NC_040274.1	60454935	A	G
NC_040274.1	60513503	T	C
NC_040274.1	60575992	A	G
NC_040274.1	60632604	A	G
NC_040274.1	60682377	T	C
NC_040274.1	60682654	T	A
NC_040274.1	60683579	T	G
NC_040274.1	60698116	T	C
NC_040274.1	60764622	T	C
NC_040274.1	60820287	G	T
NC_040274.1	60876853	C	T
NC_040274.1	60933750	A	C
NC_040274.1	60989835	T	C
NC_040274.1	61047122	T	C
NC_040274.1	61098561	G	A
NC_040274.1	61158650	G	A
NC_040274.1	61212196	A	G
NC_040274.1	61269427	G	A
NC_040274.1	61332751	G	A
NC_040274.1	61388859	C	T
NC_040274.1	61448561	G	A
NC_040274.1	61505333	C	T
NC_040274.1	61561298	G	A
NC_040274.1	61618636	T	C
NC_040274.1	61678455	T	C
NC_040274.1	61737687	G	A
NC_040274.1	61794829	C	T
NC_040274.1	61852004	G	A
NC_040274.1	61900530	G	T
NC_040274.1	62025159	G	A
NC_040274.1	62071636	G	T
NC_040274.1	62130899	G	A
NC_040274.1	62199845	T	G

NC_040274.1	62256886	G	A
NC_040274.1	62307251	T	C
NC_040274.1	62370108	G	A
NC_040274.1	62427207	A	G
NC_040274.1	62481392	A	C
NC_040274.1	62541750	G	A
NC_040274.1	62604432	T	C
NC_040274.1	62655200	T	C
NC_040274.1	62706383	T	C
NC_040274.1	62714410	A	G
NC_040274.1	62718983	C	A
NC_040274.1	62775808	T	C
NC_040274.1	62816427	G	C
NC_040274.1	62819077	G	A
NC_040274.1	62819987	A	G
NC_040274.1	62821485	A	G
NC_040274.1	62821900	G	A
NC_040274.1	62822093	C	T
NC_040274.1	62822278	T	C
NC_040274.1	62824698	T	G
NC_040274.1	62825423	C	T
NC_040274.1	62826693	G	A
NC_040274.1	62827105	A	G
NC_040274.1	62827748	C	A
NC_040274.1	62882484	T	G
NC_040274.1	62937771	G	A
NC_040274.1	62961976	A	G
NC_040274.1	62969737	A	G
NC_040274.1	62997382	A	G
NC_040274.1	63002456	T	C
NC_040274.1	63060256	C	T
NC_040274.1	63116259	T	C
NC_040274.1	63173197	A	G
NC_040274.1	63229846	G	T
NC_040274.1	63287676	T	G
NC_040274.1	63344524	A	G
NC_040274.1	63404628	C	T
NC_040274.1	63464163	T	C
NC_040274.1	63523031	C	T
NC_040274.1	63588047	A	G
NC_040274.1	63590143	T	C
NC_040274.1	63616660	T	C
NC_040274.1	63616811	A	G
NC_040274.1	63686271	A	G
NC_040274.1	63719844	T	C
NC_040274.1	63720555	G	A
NC_040274.1	63769614	A	G

NC_040274.1	63822082	A	G
NC_040274.1	63860469	C	G
NC_040274.1	63896607	T	C
NC_040274.1	63954186	C	G
NC_040274.1	64007339	G	A
NC_040274.1	64007558	A	G
NC_040274.1	64058886	T	C
NC_040274.1	64059210	T	C
NC_040274.1	64120417	T	C
NC_040274.1	64122203	G	A
NC_040274.1	64122375	G	A
NC_040274.1	64176664	G	A
NC_040274.1	64239213	G	A
NC_040274.1	64303403	G	A
NC_040274.1	64371521	G	C
NC_040274.1	64372221	C	A
NC_040274.1	64383658	A	G
NC_040274.1	64440635	T	C
NC_040274.1	64492389	A	G
NC_040274.1	64544280	A	G
NC_040274.1	64604344	C	T
NC_040274.1	64659292	T	C
NC_040274.1	64714588	C	T
NC_040274.1	64764859	G	T
NC_040274.1	64816202	C	T
NC_040274.1	64873608	C	T
NC_040274.1	64942324	G	A
NC_040274.1	64981084	G	A
NC_040274.1	65052339	T	A
NC_040274.1	65109281	T	C
NC_040274.1	65176438	T	C
NC_040274.1	65225112	C	T
NC_040274.1	65225579	T	C
NC_040274.1	65279357	A	G
NC_040274.1	65331773	C	G
NC_040274.1	65382405	G	A
NC_040274.1	65427053	A	G
NC_040274.1	65462910	G	A
NC_040274.1	65519986	A	T
NC_040274.1	65553439	G	T
NC_040274.1	65666601	A	G
NC_040274.1	65715022	G	A
NC_040274.1	65759992	C	T
NC_040274.1	65818221	G	A
NC_040274.1	65890084	G	A
NC_040274.1	65940607	A	G
NC_040274.1	65984540	T	C

NC_040274.1	66028961	A	G
NC_040274.1	66070015	A	G
NC_040274.1	66131595	C	T
NC_040274.1	66196365	T	C
NC_040274.1	66206688	G	A
NC_040274.1	66221116	G	C
NC_040274.1	66282505	A	G
NC_040274.1	66331139	G	A
NC_040274.1	66383898	C	T
NC_040274.1	66428486	A	C
NC_040274.1	66433318	T	C
NC_040274.1	66433706	A	G
NC_040274.1	66434204	C	G
NC_040274.1	66491372	T	C
NC_040274.1	66544715	T	A
NC_040274.1	66601310	A	T
NC_040274.1	66666827	A	G
NC_040274.1	66724259	T	G
NC_040274.1	66780282	C	T
NC_040274.1	66829605	A	G
NC_040274.1	66890214	A	G
NC_040274.1	66890402	G	A
NC_040274.1	66944173	C	A
NC_040274.1	66989733	A	G
NC_040274.1	67047135	A	T
NC_040274.1	67058263	C	G
NC_040274.1	67061795	G	A
NC_040274.1	67072133	C	T
NC_040274.1	67072828	T	C
NC_040274.1	67075277	G	A
NC_040274.1	67075549	C	T
NC_040274.1	67132224	T	G
NC_040274.1	67188536	T	C
NC_040274.1	67243099	G	A
NC_040274.1	67302428	C	G
NC_040274.1	67354007	T	C
NC_040274.1	67399967	A	G
NC_040274.1	67456953	G	A
NC_040274.1	67517401	T	C
NC_040274.1	67572436	A	G
NC_040274.1	67626020	T	C
NC_040274.1	67683479	A	C
NC_040274.1	67709073	A	G
NC_040274.1	67716653	A	G
NC_040274.1	67720408	C	G
NC_040274.1	67764187	C	T
NC_040274.1	67880232	T	C

NC_040274.1	67937618	A	G
NC_040274.1	67993384	A	G
NC_040274.1	68046802	T	C
NC_040274.1	68105419	T	C
NC_040274.1	68138706	G	A
NC_040274.1	68144787	A	G
NC_040274.1	68199601	T	C
NC_040274.1	68249447	A	G
NC_040274.1	68301293	T	C
NC_040274.1	68301757	T	C
NC_040275.1	23100	G	C
NC_040275.1	99108	A	G
NC_040275.1	103037	G	A
NC_040275.1	104028	T	C
NC_040275.1	105269	A	G
NC_040275.1	150207	C	T
NC_040275.1	190733	T	A
NC_040275.1	199649	T	C
NC_040275.1	244944	A	G
NC_040275.1	257196	C	T
NC_040275.1	257467	A	G
NC_040275.1	260090	T	C
NC_040275.1	262728	T	G
NC_040275.1	265504	A	G
NC_040275.1	269888	T	C
NC_040275.1	276554	T	C
NC_040275.1	276741	T	C
NC_040275.1	277453	T	C
NC_040275.1	291003	A	G
NC_040275.1	316241	A	G
NC_040275.1	329671	G	C
NC_040275.1	331581	A	G
NC_040275.1	356431	C	T
NC_040275.1	358840	A	G
NC_040275.1	361629	A	G
NC_040275.1	361807	T	C
NC_040275.1	392733	A	C
NC_040275.1	433063	T	C
NC_040275.1	433240	A	G
NC_040275.1	443338	A	G
NC_040275.1	444932	T	C
NC_040275.1	445139	A	G
NC_040275.1	445372	T	C
NC_040275.1	445641	A	G
NC_040275.1	448030	T	C
NC_040275.1	452849	T	C
NC_040275.1	468853	A	G

NC_040275.1	480239	T	C
NC_040275.1	487647	G	C
NC_040275.1	506945	T	C
NC_040275.1	509767	G	A
NC_040275.1	510919	A	G
NC_040275.1	512914	T	A
NC_040275.1	514213	T	G
NC_040275.1	514572	T	C
NC_040275.1	523617	C	T
NC_040275.1	525501	T	C
NC_040275.1	526159	T	C
NC_040275.1	526522	C	T
NC_040275.1	537360	A	G
NC_040275.1	542377	G	C
NC_040275.1	546823	A	G
NC_040275.1	549081	T	G
NC_040275.1	550755	A	C
NC_040275.1	551895	T	C
NC_040275.1	552857	A	G
NC_040275.1	554145	A	G
NC_040275.1	556342	C	A
NC_040275.1	558947	A	G
NC_040275.1	565546	A	C
NC_040275.1	567007	A	G
NC_040275.1	569172	A	G
NC_040275.1	569324	T	C
NC_040275.1	570269	G	T
NC_040275.1	591341	G	T
NC_040275.1	592089	C	T
NC_040275.1	597124	T	C
NC_040275.1	600256	A	G
NC_040275.1	619974	T	C
NC_040275.1	620193	T	C
NC_040275.1	637039	C	G
NC_040275.1	637330	C	T
NC_040275.1	643999	C	T
NC_040275.1	646350	T	C
NC_040275.1	693924	T	C
NC_040275.1	694392	C	T
NC_040275.1	756930	A	G
NC_040275.1	758891	T	C
NC_040275.1	819350	C	T
NC_040275.1	828159	A	G
NC_040275.1	841248	T	C
NC_040275.1	841944	T	A
NC_040275.1	846171	C	G
NC_040275.1	898514	T	C

NC_040275.1	926880	A	G
NC_040275.1	928868	G	A
NC_040275.1	929187	A	G
NC_040275.1	953993	A	G
NC_040275.1	954455	T	C
NC_040275.1	1009509	A	G
NC_040275.1	1009674	A	G
NC_040275.1	1022770	C	G
NC_040275.1	1032371	T	C
NC_040275.1	1033873	G	A
NC_040275.1	1044572	G	A
NC_040275.1	1045341	A	T
NC_040275.1	1045994	C	G
NC_040275.1	1046234	G	A
NC_040275.1	1081110	C	G
NC_040275.1	1089301	C	G
NC_040275.1	1089961	T	C
NC_040275.1	1090780	A	G
NC_040275.1	1097719	T	C
NC_040275.1	1099558	A	G
NC_040275.1	1100209	A	G
NC_040275.1	1103343	A	G
NC_040275.1	1112920	T	C
NC_040275.1	1125490	T	C
NC_040275.1	1134799	A	G
NC_040275.1	1143085	A	G
NC_040275.1	1202414	C	T
NC_040275.1	1230537	A	G
NC_040275.1	1234159	C	T
NC_040275.1	1237003	T	C
NC_040275.1	1237604	T	C
NC_040275.1	1241062	T	A
NC_040275.1	1241301	A	G
NC_040275.1	1241674	T	C
NC_040275.1	1243189	T	C
NC_040275.1	1243596	C	T
NC_040275.1	1243983	G	A
NC_040275.1	1255294	G	T
NC_040275.1	1256480	A	G
NC_040275.1	1258552	T	C
NC_040275.1	1279326	T	C
NC_040275.1	1299935	T	C
NC_040275.1	1303698	A	C
NC_040275.1	1304626	A	G
NC_040275.1	1307521	T	C
NC_040275.1	1309183	C	T
NC_040275.1	1309956	A	G

NC_040275.1	1311862	A	G
NC_040275.1	1316894	A	G
NC_040275.1	1320224	A	G
NC_040275.1	1320996	A	G
NC_040275.1	1321923	T	C
NC_040275.1	1323339	T	C
NC_040275.1	1323557	G	A
NC_040275.1	1324522	T	C
NC_040275.1	1324709	T	C
NC_040275.1	1326447	A	G
NC_040275.1	1327260	A	G
NC_040275.1	1328580	G	A
NC_040275.1	1332669	T	A
NC_040275.1	1335885	A	G
NC_040275.1	1361696	G	A
NC_040275.1	1397542	G	A
NC_040275.1	1444590	G	A
NC_040275.1	1449182	A	C
NC_040275.1	1465058	A	G
NC_040275.1	1467088	T	C
NC_040275.1	1471667	T	C
NC_040275.1	1484499	T	C
NC_040275.1	1486489	T	C
NC_040275.1	1489734	A	G
NC_040275.1	1494526	A	G
NC_040275.1	1505484	G	A
NC_040275.1	1511534	C	T
NC_040275.1	1527622	T	C
NC_040275.1	1538735	C	A
NC_040275.1	1539069	T	C
NC_040275.1	1543170	A	G
NC_040275.1	1544269	T	C
NC_040275.1	1544769	T	C
NC_040275.1	1549871	T	G
NC_040275.1	1551354	T	G
NC_040275.1	1580740	C	T
NC_040275.1	1581622	A	G
NC_040275.1	1581985	T	C
NC_040275.1	1582301	G	C
NC_040275.1	1582494	A	G
NC_040275.1	1584461	G	C
NC_040275.1	1585091	A	C
NC_040275.1	1586115	C	G
NC_040275.1	1592691	C	T
NC_040275.1	1592919	A	G
NC_040275.1	1593141	T	C
NC_040275.1	1596963	T	C

NC_040275.1	1597220	T	C
NC_040275.1	1597714	A	G
NC_040275.1	1598425	A	G
NC_040275.1	1606649	A	G
NC_040275.1	1606952	T	C
NC_040275.1	1639303	T	C
NC_040275.1	1640195	G	A
NC_040275.1	1668220	T	C
NC_040275.1	1669126	T	C
NC_040275.1	1670015	A	G
NC_040275.1	1675957	A	G
NC_040275.1	1691305	A	G
NC_040275.1	1695088	T	C
NC_040275.1	1705336	A	G
NC_040275.1	1707781	G	C
NC_040275.1	1708852	T	C
NC_040275.1	1714900	T	C
NC_040275.1	1717550	A	G
NC_040275.1	1719107	T	C
NC_040275.1	1721013	T	C
NC_040275.1	1729420	T	C
NC_040275.1	1731766	G	C
NC_040275.1	1737768	T	C
NC_040275.1	1741554	C	T
NC_040275.1	1741885	T	G
NC_040275.1	1748491	T	C
NC_040275.1	1754076	A	G
NC_040275.1	1777548	T	G
NC_040275.1	1781858	T	C
NC_040275.1	1806193	A	G
NC_040275.1	1816381	T	C
NC_040275.1	1825048	T	C
NC_040275.1	1862793	A	G
NC_040275.1	1865398	G	C
NC_040275.1	1888807	A	G
NC_040275.1	1900289	G	A
NC_040275.1	1913031	T	C
NC_040275.1	1913242	G	A
NC_040275.1	1923157	A	G
NC_040275.1	1923395	A	G
NC_040275.1	1924912	T	C
NC_040275.1	1927764	C	T
NC_040275.1	1929473	C	T
NC_040275.1	1931692	G	A
NC_040275.1	1943175	G	A
NC_040275.1	1944531	A	G
NC_040275.1	1946382	T	C

NC_040275.1	1950011	A	G
NC_040275.1	1952242	C	T
NC_040275.1	1964261	A	C
NC_040275.1	2002668	T	C
NC_040275.1	2041811	A	G
NC_040275.1	2042814	T	G
NC_040275.1	2046231	T	G
NC_040275.1	2072680	T	C
NC_040275.1	2182519	C	T
NC_040275.1	2196168	G	C
NC_040275.1	2215639	A	C
NC_040275.1	2230021	C	T
NC_040275.1	2230245	A	G
NC_040275.1	2230697	T	C
NC_040275.1	2231541	C	T
NC_040275.1	2231982	T	C
NC_040275.1	2232948	C	T
NC_040275.1	2233941	T	C
NC_040275.1	2234243	C	T
NC_040275.1	2241363	C	A
NC_040275.1	2242482	C	T
NC_040275.1	2243083	C	T
NC_040275.1	2271069	C	T
NC_040275.1	2295885	A	C
NC_040275.1	2321214	T	C
NC_040275.1	2321486	A	G
NC_040275.1	2324239	T	C
NC_040275.1	2446055	A	C
NC_040275.1	2475401	T	C
NC_040275.1	2476020	G	C
NC_040275.1	2590169	T	C
NC_040275.1	2608764	A	C
NC_040275.1	2617492	C	G
NC_040275.1	2702946	C	G
NC_040275.1	2703174	A	C
NC_040275.1	2711948	G	A
NC_040275.1	2712933	T	C
NC_040275.1	2766357	G	A
NC_040275.1	2766651	C	T
NC_040275.1	2766999	T	C
NC_040275.1	2767186	G	C
NC_040275.1	2792331	G	C
NC_040275.1	2793019	T	C
NC_040275.1	2808018	A	T
NC_040275.1	2854515	T	G
NC_040275.1	2854745	T	C
NC_040275.1	2892867	T	C

NC_040275.1	2896239	G	C
NC_040275.1	2898136	C	A
NC_040275.1	2922934	A	G
NC_040275.1	2923241	G	T
NC_040275.1	2923556	G	A
NC_040275.1	2940543	G	A
NC_040275.1	2968968	C	G
NC_040275.1	2969321	A	G
NC_040275.1	2969608	G	A
NC_040275.1	2969796	C	A
NC_040275.1	2969950	T	C
NC_040275.1	2970875	A	G
NC_040275.1	2972075	T	C
NC_040275.1	2984608	T	G
NC_040275.1	2996704	C	G
NC_040275.1	3000197	T	G
NC_040275.1	3001303	A	G
NC_040275.1	3004221	C	G
NC_040275.1	3004734	T	C
NC_040275.1	3005171	T	C
NC_040275.1	3005515	A	G
NC_040275.1	3006959	A	G
NC_040275.1	3011473	A	G
NC_040275.1	3016081	C	G
NC_040275.1	3048415	T	C
NC_040275.1	3057946	T	C
NC_040275.1	3109126	A	C
NC_040275.1	3110428	T	C
NC_040275.1	3111057	T	C
NC_040275.1	3131434	A	G
NC_040275.1	3142303	A	G
NC_040275.1	3157427	A	G
NC_040275.1	3204408	A	G
NC_040275.1	3299079	T	G
NC_040275.1	3299309	A	G
NC_040275.1	3300128	A	G
NC_040275.1	3300375	A	G
NC_040275.1	3304657	A	G
NC_040275.1	3305874	C	T
NC_040275.1	3306918	A	G
NC_040275.1	3363676	C	G
NC_040275.1	3406414	A	G
NC_040275.1	3407355	A	G
NC_040275.1	3455114	A	G
NC_040275.1	3455264	A	G
NC_040275.1	3455627	A	G
NC_040275.1	3510826	C	G

NC_040275.1	3511198	C	G
NC_040275.1	3551846	A	G
NC_040275.1	3552926	G	A
NC_040275.1	3553187	T	C
NC_040275.1	3553438	A	G
NC_040275.1	3558506	T	C
NC_040275.1	3558971	T	C
NC_040275.1	3559314	C	T
NC_040275.1	3560313	A	G
NC_040275.1	3569619	T	G
NC_040275.1	3570318	T	C
NC_040275.1	3593161	T	C
NC_040275.1	3615168	T	C
NC_040275.1	3622604	C	G
NC_040275.1	3684822	T	G
NC_040275.1	3709440	T	C
NC_040275.1	3711550	A	G
NC_040275.1	3713184	T	C
NC_040275.1	3713339	T	C
NC_040275.1	3713642	T	C
NC_040275.1	3715718	A	G
NC_040275.1	3758848	A	G
NC_040275.1	3767739	G	A
NC_040275.1	3769538	T	C
NC_040275.1	3770256	C	T
NC_040275.1	3793696	G	A
NC_040275.1	3801331	G	A
NC_040275.1	3801482	G	A
NC_040275.1	3847000	T	C
NC_040275.1	3856744	A	G
NC_040275.1	3860915	T	C
NC_040275.1	3863921	A	T
NC_040275.1	3864284	A	G
NC_040275.1	3870118	A	G
NC_040275.1	3873960	A	G
NC_040275.1	3876506	G	A
NC_040275.1	3878551	C	G
NC_040275.1	3890644	A	G
NC_040275.1	3890922	G	T
NC_040275.1	3928306	T	C
NC_040275.1	3940405	T	C
NC_040275.1	3959481	T	C
NC_040275.1	3975058	G	C
NC_040275.1	4005480	T	C
NC_040275.1	4007686	A	G
NC_040275.1	4008249	A	G
NC_040275.1	4009489	A	G

NC_040275.1	4016143	T	C
NC_040275.1	4019052	T	C
NC_040275.1	4054690	G	T
NC_040275.1	4112218	A	G
NC_040275.1	4126693	T	C
NC_040275.1	4128424	A	G
NC_040275.1	4168167	T	C
NC_040275.1	4176057	T	C
NC_040275.1	4207115	C	T
NC_040275.1	4989164	A	G
NC_040275.1	5026742	A	G
NC_040275.1	5113398	T	C
NC_040275.1	5164849	G	C
NC_040275.1	5226951	A	G
NC_040275.1	5288424	T	C
NC_040275.1	5349179	A	G
NC_040275.1	5408513	C	T
NC_040275.1	5477410	G	A
NC_040275.1	5478498	T	C
NC_040275.1	5530743	G	C
NC_040275.1	5589899	C	T
NC_040275.1	5672612	T	C
NC_040275.1	5727319	T	A
NC_040275.1	5798840	T	C
NC_040275.1	5855589	A	G
NC_040275.1	5920300	G	C
NC_040275.1	5990808	C	T
NC_040275.1	6050929	G	A
NC_040275.1	6120809	G	A
NC_040275.1	6272765	C	T
NC_040275.1	6333372	G	A
NC_040275.1	6405861	C	T
NC_040275.1	6406966	C	T
NC_040275.1	6407117	T	C
NC_040275.1	6407347	A	C
NC_040275.1	6407585	A	T
NC_040275.1	6407793	T	C
NC_040275.1	6408095	T	A
NC_040275.1	6461262	A	G
NC_040275.1	6519467	C	A
NC_040275.1	6579758	G	A
NC_040275.1	6641379	G	A
NC_040275.1	6705343	T	C
NC_040275.1	6768831	T	C
NC_040275.1	6844392	T	G
NC_040275.1	6844888	G	A
NC_040275.1	7759474	C	T

NC_040275.1	7822302	T	C
NC_040275.1	7869475	T	C
NC_040275.1	7877721	G	A
NC_040275.1	7877872	G	A
NC_040275.1	7878042	G	A
NC_040275.1	7879192	G	A
NC_040275.1	7922405	0	G
NC_040275.1	7925771	A	G
NC_040275.1	7958101	A	G
NC_040275.1	7958733	A	G
NC_040275.1	8011188	C	T
NC_040275.1	8120592	T	C
NC_040275.1	8144503	C	A
NC_040275.1	8748254	T	C
NC_040275.1	8749288	T	A
NC_040275.1	8753596	C	T
NC_040275.1	8754946	C	T
NC_040275.1	8757453	A	G
NC_040275.1	8758289	T	C
NC_040275.1	8821509	A	G
NC_040275.1	8876056	C	T
NC_040275.1	8933802	G	A
NC_040275.1	8990050	T	A
NC_040275.1	9043574	C	T
NC_040275.1	9088722	G	C
NC_040275.1	9139653	T	C
NC_040275.1	9185977	T	C
NC_040275.1	9435192	A	G
NC_040275.1	9452672	C	T
NC_040275.1	9452917	A	G
NC_040275.1	9458793	A	G
NC_040275.1	9526389	G	A
NC_040275.1	9567260	C	T
NC_040275.1	9628856	G	C
NC_040275.1	9669096	G	A
NC_040275.1	9694751	A	C
NC_040275.1	9727422	T	C
NC_040275.1	9800579	C	T
NC_040275.1	9849638	C	T
NC_040275.1	9857621	T	C
NC_040275.1	9907270	A	G
NC_040275.1	9958191	T	C
NC_040275.1	9969716	A	G
NC_040275.1	9981793	A	G
NC_040275.1	10046332	A	G
NC_040275.1	10109427	C	G
NC_040275.1	10109612	A	T

NC_040275.1	10110411	A	G
NC_040275.1	10165430	A	G
NC_040275.1	10165951	T	G
NC_040275.1	10166552	T	C
NC_040275.1	10189746	G	A
NC_040275.1	10246237	A	G
NC_040275.1	10246806	A	G
NC_040275.1	10293010	T	G
NC_040275.1	10487936	A	C
NC_040275.1	10496756	A	G
NC_040275.1	10621177	G	A
NC_040275.1	10625334	T	C
NC_040275.1	10625894	A	C
NC_040275.1	10626274	A	G
NC_040275.1	10627760	G	A
NC_040275.1	10635824	C	T
NC_040275.1	10638836	A	G
NC_040275.1	10639244	G	A
NC_040275.1	10694659	C	T
NC_040275.1	10694828	G	A
NC_040275.1	10721036	G	A
NC_040275.1	10793120	A	G
NC_040275.1	10811611	C	T
NC_040275.1	10812836	A	T
NC_040275.1	10813978	T	C
NC_040275.1	10842701	T	C
NC_040275.1	10925185	A	G
NC_040275.1	10977037	T	G
NC_040275.1	11041112	A	C
NC_040275.1	11101981	T	C
NC_040275.1	11164028	A	G
NC_040275.1	11211540	C	T
NC_040275.1	11254679	C	G
NC_040275.1	11275523	A	G
NC_040275.1	11350925	G	C
NC_040275.1	11411252	T	G
NC_040275.1	11463788	T	C
NC_040275.1	11634013	C	T
NC_040275.1	11664390	T	C
NC_040275.1	11724857	T	C
NC_040275.1	11760155	T	C
NC_040275.1	11858051	A	C
NC_040275.1	11919840	A	G
NC_040275.1	11980073	G	A
NC_040275.1	12042242	T	C
NC_040275.1	12102946	G	A
NC_040275.1	12152696	C	T

NC_040275.1	12203054	T	C
NC_040275.1	12204123	A	G
NC_040275.1	13068482	G	A
NC_040275.1	13076211	A	G
NC_040275.1	13092199	T	C
NC_040275.1	13150251	C	T
NC_040275.1	13206983	C	T
NC_040275.1	13268812	C	A
NC_040275.1	13325290	T	G
NC_040275.1	13349988	C	T
NC_040275.1	13350254	A	G
NC_040275.1	13350645	A	G
NC_040275.1	13397618	A	C
NC_040275.1	13405984	A	G
NC_040275.1	13406543	G	A
NC_040275.1	13410535	T	C
NC_040275.1	13419048	T	C
NC_040275.1	13419774	A	G
NC_040275.1	13420716	A	G
NC_040275.1	13420923	A	G
NC_040275.1	13421695	T	C
NC_040275.1	13422617	A	C
NC_040275.1	13422828	C	T
NC_040275.1	13423367	T	C
NC_040275.1	13593127	A	G
NC_040275.1	13603475	G	C
NC_040275.1	13658284	T	C
NC_040275.1	13718418	T	C
NC_040275.1	13777457	G	A
NC_040275.1	13781724	G	C
NC_040275.1	13788237	A	G
NC_040275.1	13813483	C	T
NC_040275.1	13900390	C	G
NC_040275.1	13901565	T	C
NC_040275.1	13901925	T	C
NC_040275.1	13907487	T	G
NC_040275.1	13907659	T	A
NC_040275.1	13922172	T	C
NC_040275.1	13949353	C	T
NC_040275.1	13961640	T	C
NC_040275.1	13968824	A	G
NC_040275.1	13970583	T	C
NC_040275.1	14024040	T	A
NC_040275.1	14100792	A	G
NC_040275.1	14161945	C	T
NC_040275.1	14201298	T	C
NC_040275.1	14248641	C	T

NC_040275.1	14285359	A	G
NC_040275.1	14291984	T	C
NC_040275.1	14326225	G	A
NC_040275.1	14365164	A	G
NC_040275.1	14389127	C	G
NC_040275.1	14409323	A	G
NC_040275.1	14412409	A	G
NC_040275.1	14449941	A	T
NC_040275.1	14483888	A	G
NC_040275.1	14484196	C	T
NC_040275.1	14484467	T	C
NC_040275.1	14485136	T	G
NC_040275.1	14495289	T	C
NC_040275.1	14553835	A	G
NC_040275.1	14592292	C	T
NC_040275.1	14633384	A	G
NC_040275.1	14657573	T	C
NC_040275.1	14660264	T	C
NC_040275.1	14669012	T	C
NC_040275.1	14702649	T	C
NC_040275.1	14708722	G	A
NC_040275.1	14708873	C	G
NC_040275.1	14777449	A	G
NC_040275.1	14833481	C	A
NC_040275.1	15757674	C	A
NC_040275.1	15818074	T	C
NC_040275.1	15878505	A	G
NC_040275.1	15949001	A	G
NC_040275.1	16009845	G	A
NC_040275.1	16057951	C	T
NC_040275.1	16102116	T	C
NC_040275.1	16104485	A	G
NC_040275.1	16107074	T	C
NC_040275.1	16641494	C	T
NC_040275.1	16660746	A	T
NC_040275.1	16660998	A	G
NC_040275.1	16661200	T	C
NC_040275.1	16661560	G	C
NC_040275.1	16662226	G	A
NC_040275.1	16663799	T	C
NC_040275.1	16664127	T	G
NC_040275.1	16671316	C	T
NC_040275.1	16682627	A	G
NC_040275.1	16683234	A	G
NC_040275.1	16687652	A	G
NC_040275.1	16688212	C	T
NC_040275.1	16699830	A	G

NC_040275.1	16730559	T	C
NC_040275.1	16758074	A	C
NC_040275.1	16892417	T	C
NC_040275.1	16946393	G	A
NC_040275.1	16991557	G	C
NC_040275.1	17069354	C	T
NC_040275.1	17071215	G	A
NC_040275.1	17071941	A	G
NC_040275.1	17121059	C	T
NC_040275.1	17171568	G	A
NC_040275.1	17238383	C	T
NC_040275.1	17246220	G	T
NC_040275.1	17254942	A	G
NC_040275.1	17261461	T	C
NC_040275.1	17321832	T	C
NC_040275.1	17337298	G	C
NC_040275.1	17350190	A	G
NC_040275.1	17351372	C	A
NC_040275.1	17351609	T	C
NC_040275.1	17409763	T	C
NC_040275.1	17453474	C	T
NC_040275.1	17499320	C	T
NC_040275.1	17553525	C	T
NC_040275.1	17618526	A	T
NC_040275.1	17662624	C	T
NC_040275.1	17662906	A	G
NC_040275.1	17837587	G	A
NC_040275.1	17880344	C	T
NC_040275.1	18180437	C	A
NC_040275.1	18184645	T	C
NC_040275.1	18188212	A	G
NC_040275.1	18190277	T	C
NC_040275.1	18214380	T	C
NC_040275.1	18230223	G	A
NC_040275.1	18288499	G	A
NC_040275.1	18361643	T	C
NC_040275.1	18366112	T	C
NC_040275.1	18380201	T	C
NC_040275.1	18407926	A	G
NC_040275.1	18460980	T	C
NC_040275.1	18488939	C	T
NC_040275.1	18655903	T	C
NC_040275.1	18659197	A	G
NC_040275.1	18715247	G	A
NC_040275.1	18743956	C	T
NC_040275.1	18754168	T	C
NC_040275.1	18769877	T	C

NC_040275.1	18771948	C	A
NC_040275.1	18784553	G	C
NC_040275.1	18848151	A	G
NC_040275.1	18849675	A	G
NC_040275.1	18850292	A	G
NC_040275.1	18855563	C	G
NC_040275.1	18876088	A	G
NC_040275.1	19267747	C	T
NC_040275.1	19268773	C	T
NC_040275.1	19269578	G	A
NC_040275.1	19270255	G	A
NC_040275.1	19271598	A	G
NC_040275.1	19302276	C	T
NC_040275.1	19302978	C	T
NC_040275.1	19333414	G	A
NC_040275.1	19335452	T	G
NC_040275.1	19399805	A	G
NC_040275.1	19451368	T	C
NC_040275.1	19497518	A	G
NC_040275.1	19520105	T	G
NC_040275.1	19548508	C	T
NC_040275.1	19550392	A	C
NC_040275.1	19593894	G	A
NC_040275.1	19621065	G	A
NC_040275.1	19711348	C	G
NC_040275.1	19723692	A	G
NC_040275.1	19742271	A	G
NC_040275.1	19783382	G	C
NC_040275.1	19837231	A	G
NC_040275.1	19898824	A	G
NC_040275.1	19929833	C	T
NC_040275.1	20023745	G	A
NC_040275.1	20049725	C	T
NC_040275.1	20087334	G	T
NC_040275.1	20126122	G	C
NC_040275.1	20139693	G	A
NC_040275.1	20199219	C	T
NC_040275.1	20251648	T	A
NC_040275.1	20257907	A	C
NC_040275.1	20271290	G	A
NC_040275.1	20273464	G	A
NC_040275.1	20326828	C	T
NC_040275.1	20474639	C	T
NC_040275.1	20483773	A	G
NC_040275.1	20518959	A	G
NC_040275.1	20554843	A	G
NC_040275.1	20586287	T	C

NC_040275.1	20631939	T	C
NC_040275.1	20636009	T	C
NC_040275.1	20683626	T	C
NC_040275.1	20684328	A	G
NC_040275.1	20719910	0	G
NC_040275.1	20727817	A	G
NC_040275.1	20728132	A	C
NC_040275.1	20763161	A	G
NC_040275.1	20784488	G	C
NC_040275.1	20822501	T	C
NC_040275.1	20832168	G	A
NC_040275.1	20890324	T	C
NC_040275.1	20927501	A	C
NC_040275.1	21167278	A	T
NC_040275.1	21224918	A	G
NC_040275.1	21277108	A	G
NC_040275.1	21431570	G	A
NC_040275.1	21433368	A	T
NC_040275.1	21435138	G	A
NC_040275.1	21490485	C	T
NC_040275.1	21552652	C	T
NC_040275.1	21574226	G	A
NC_040275.1	21662339	G	A
NC_040275.1	21691776	G	A
NC_040275.1	21736966	A	G
NC_040275.1	21807954	A	G
NC_040275.1	21879035	T	A
NC_040275.1	21920568	A	G
NC_040275.1	21920792	T	C
NC_040275.1	21966432	A	G
NC_040275.1	21970147	C	T
NC_040275.1	22018895	T	C
NC_040275.1	22037634	T	C
NC_040275.1	22087356	C	T
NC_040275.1	22185679	A	G
NC_040275.1	22246195	T	A
NC_040275.1	22308899	T	A
NC_040275.1	22371602	A	G
NC_040275.1	22434962	G	A
NC_040275.1	22488179	T	A
NC_040275.1	22553599	C	T
NC_040275.1	22553767	T	C
NC_040275.1	22556197	G	C
NC_040275.1	22557454	T	C
NC_040275.1	22601231	T	C
NC_040275.1	22662635	C	G
NC_040275.1	22697971	G	C

NC_040275.1	22698401	A	G
NC_040275.1	22875739	A	G
NC_040275.1	22902998	G	C
NC_040275.1	22908654	A	C
NC_040275.1	22908991	A	G
NC_040275.1	22909681	G	A
NC_040275.1	22910769	T	A
NC_040275.1	22911215	T	A
NC_040275.1	22971252	T	C
NC_040275.1	23021719	C	A
NC_040275.1	23071679	G	T
NC_040275.1	23122502	A	G
NC_040275.1	23123505	T	C
NC_040275.1	23123772	A	G
NC_040275.1	23154197	T	C
NC_040275.1	23195961	G	A
NC_040275.1	23236424	A	G
NC_040275.1	23248816	C	T
NC_040275.1	23266488	C	T
NC_040275.1	23325572	A	G
NC_040275.1	23380644	G	C
NC_040275.1	23426442	G	A
NC_040275.1	23467598	C	T
NC_040275.1	23482519	G	T
NC_040275.1	23483286	G	A
NC_040275.1	23484129	T	G
NC_040275.1	23955918	A	G
NC_040275.1	24013579	A	G
NC_040275.1	24079069	G	A
NC_040275.1	24159792	G	A
NC_040275.1	24229407	A	G
NC_040275.1	24296478	A	G
NC_040275.1	24356659	G	A
NC_040275.1	24433380	T	A
NC_040275.1	25461746	A	G
NC_040275.1	25462331	T	G
NC_040275.1	25462553	C	A
NC_040275.1	25463364	C	T
NC_040275.1	25507224	A	G
NC_040275.1	25550291	G	A
NC_040275.1	25557096	A	G
NC_040275.1	25616188	C	A
NC_040275.1	25677000	C	A
NC_040275.1	25742242	C	G
NC_040275.1	25776606	T	C
NC_040275.1	25819427	G	A
NC_040275.1	25857060	T	C

NC_040275.1	25916247	C	T
NC_040275.1	25973739	A	G
NC_040275.1	26028360	C	T
NC_040275.1	26081637	A	G
NC_040275.1	26127255	G	A
NC_040275.1	26170636	A	G
NC_040275.1	26181101	A	G
NC_040275.1	26203718	C	T
NC_040275.1	26264473	G	A
NC_040275.1	26324857	A	G
NC_040275.1	26359617	A	G
NC_040275.1	26360439	A	G
NC_040275.1	26396933	A	G
NC_040275.1	26406746	T	C
NC_040275.1	26410066	A	G
NC_040275.1	26410263	G	A
NC_040275.1	26439397	T	C
NC_040275.1	26439579	T	C
NC_040275.1	26443839	G	A
NC_040275.1	26462008	G	A
NC_040275.1	26463319	A	G
NC_040275.1	26474692	A	G
NC_040275.1	26484940	C	T
NC_040275.1	26486382	T	C
NC_040275.1	26490401	T	C
NC_040275.1	26491616	G	A
NC_040275.1	26503480	T	C
NC_040275.1	26504009	T	C
NC_040275.1	26509697	C	T
NC_040275.1	26510755	T	C
NC_040275.1	26518763	T	C
NC_040275.1	26550704	A	G
NC_040275.1	26553091	T	C
NC_040275.1	26561988	T	C
NC_040275.1	26586451	T	C
NC_040275.1	26589958	T	C
NC_040275.1	26590522	A	G
NC_040275.1	26591008	T	C
NC_040275.1	26592536	T	C
NC_040275.1	26592930	T	A
NC_040275.1	26593158	G	A
NC_040275.1	26595572	A	G
NC_040275.1	26595808	G	A
NC_040275.1	26597392	C	T
NC_040275.1	26598797	T	C
NC_040275.1	26658102	T	C
NC_040275.1	26663336	C	T

NC_040275.1	26666194	G	T
NC_040275.1	26666347	T	G
NC_040275.1	26671619	A	G
NC_040275.1	26720776	T	C
NC_040275.1	26721663	G	C
NC_040275.1	26724769	T	C
NC_040275.1	26727200	G	T
NC_040275.1	26728322	A	G
NC_040275.1	26731799	A	G
NC_040275.1	26732918	A	C
NC_040275.1	26737474	A	C
NC_040275.1	26748490	T	C
NC_040275.1	26752797	T	G
NC_040275.1	26754508	G	C
NC_040275.1	26760897	T	G
NC_040275.1	26790306	A	G
NC_040275.1	26791705	T	C
NC_040275.1	26798320	A	T
NC_040275.1	26798491	G	A
NC_040275.1	26802787	A	G
NC_040275.1	26803389	A	G
NC_040275.1	26806595	T	C
NC_040275.1	26807626	T	C
NC_040275.1	26812492	T	G
NC_040275.1	26812698	T	C
NC_040275.1	26812914	T	C
NC_040275.1	26813639	A	G
NC_040275.1	26815504	G	C
NC_040275.1	26815777	T	C
NC_040275.1	26841178	G	T
NC_040275.1	26888259	A	G
NC_040275.1	26889054	A	G
NC_040275.1	26890164	C	G
NC_040275.1	26890376	T	C
NC_040275.1	26895429	C	T
NC_040275.1	26910188	T	C
NC_040275.1	26915645	A	G
NC_040275.1	26918882	T	C
NC_040275.1	26923266	T	G
NC_040275.1	26962576	A	G
NC_040275.1	26962756	A	G
NC_040275.1	26965644	A	G
NC_040275.1	26966094	A	G
NC_040275.1	26966477	A	G
NC_040275.1	26967749	G	C
NC_040275.1	26971089	A	G
NC_040275.1	26980284	T	C

NC_040275.1	26981315	A	T
NC_040275.1	27036245	T	C
NC_040275.1	27147696	C	T
NC_040275.1	27149815	G	T
NC_040275.1	27152409	A	G
NC_040275.1	27161296	A	G
NC_040275.1	27161706	T	G
NC_040275.1	27185110	T	C
NC_040275.1	27191782	T	A
NC_040275.1	27199950	A	G
NC_040275.1	27203719	T	C
NC_040275.1	27282671	A	G
NC_040275.1	27290542	A	G
NC_040275.1	27291449	T	G
NC_040275.1	27293792	T	C
NC_040275.1	27309279	G	A
NC_040275.1	27316840	T	C
NC_040275.1	27317008	C	G
NC_040275.1	27317920	C	G
NC_040275.1	27319817	A	G
NC_040275.1	27325719	A	G
NC_040275.1	27325971	A	G
NC_040275.1	27339878	T	C
NC_040275.1	27343407	T	C
NC_040275.1	27360030	T	C
NC_040275.1	27360313	T	C
NC_040275.1	27360955	A	G
NC_040275.1	27361125	A	G
NC_040275.1	27362424	G	A
NC_040275.1	27381625	G	T
NC_040275.1	27391995	A	C
NC_040275.1	27398630	C	T
NC_040275.1	27474402	G	C
NC_040275.1	27487820	T	C
NC_040275.1	27494069	G	A
NC_040275.1	27499709	A	G
NC_040275.1	27499945	T	C
NC_040275.1	27504675	A	T
NC_040275.1	27506031	A	G
NC_040275.1	27509310	G	C
NC_040275.1	27577349	A	G
NC_040275.1	27834248	T	C
NC_040275.1	27845417	C	T
NC_040275.1	27845815	A	G
NC_040275.1	27851401	T	C
NC_040275.1	27851924	C	T
NC_040275.1	27852827	T	C

NC_040275.1	27853515	G	C
NC_040275.1	27857304	G	A
NC_040275.1	27857496	T	C
NC_040275.1	27857657	T	C
NC_040275.1	27859652	T	C
NC_040275.1	27860158	A	C
NC_040275.1	27860429	T	G
NC_040275.1	27868447	T	C
NC_040275.1	27868714	C	T
NC_040275.1	27872439	T	C
NC_040275.1	27885096	T	C
NC_040275.1	27935005	C	T
NC_040275.1	27943509	T	C
NC_040275.1	27944440	A	G
NC_040275.1	27945661	A	G
NC_040275.1	27945823	C	G
NC_040275.1	27954006	T	C
NC_040275.1	27954176	G	C
NC_040275.1	27955388	G	A
NC_040275.1	27955610	T	C
NC_040275.1	27956090	T	C
NC_040275.1	27956315	T	C
NC_040275.1	27956579	A	G
NC_040275.1	27956809	C	T
NC_040275.1	27957821	T	C
NC_040275.1	27958174	A	G
NC_040275.1	27964542	A	G
NC_040275.1	27968057	T	A
NC_040275.1	27982003	T	C
NC_040275.1	27982183	T	C
NC_040275.1	27983123	C	T
NC_040275.1	27985836	G	A
NC_040275.1	28005773	A	G
NC_040275.1	28051415	C	A
NC_040275.1	28070219	A	G
NC_040275.1	28071533	G	A
NC_040275.1	28124532	A	G
NC_040275.1	28135251	T	C
NC_040275.1	28135986	A	C
NC_040275.1	28148315	T	C
NC_040275.1	28191743	A	G
NC_040275.1	28238267	A	G
NC_040275.1	28261468	A	G
NC_040275.1	28261666	T	C
NC_040275.1	28262321	C	T
NC_040275.1	28263375	T	G
NC_040275.1	28263543	C	T

NC_040275.1	28265400	G	C
NC_040275.1	28270840	T	G
NC_040275.1	28272426	A	G
NC_040275.1	28281141	C	A
NC_040275.1	28294717	C	T
NC_040275.1	28339459	G	A
NC_040275.1	28411540	A	G
NC_040275.1	28456412	T	C
NC_040275.1	28493096	T	C
NC_040275.1	28505731	T	C
NC_040275.1	28520122	C	A
NC_040275.1	28597936	G	A
NC_040275.1	28648533	T	C
NC_040275.1	28649602	A	G
NC_040275.1	28650967	A	G
NC_040275.1	28653523	T	C
NC_040275.1	28665350	A	G
NC_040275.1	28727394	A	C
NC_040275.1	28781690	G	T
NC_040275.1	28819526	G	C
NC_040275.1	28842147	G	A
NC_040275.1	28842619	C	A
NC_040275.1	28902102	T	C
NC_040275.1	28963332	G	A
NC_040275.1	28991034	C	T
NC_040275.1	28991499	A	G
NC_040275.1	28992138	T	G
NC_040275.1	29033908	A	G
NC_040275.1	29089891	G	A
NC_040275.1	29090136	G	C
NC_040275.1	29105253	G	A
NC_040275.1	29141771	T	C
NC_040275.1	29202068	A	G
NC_040275.1	29238528	T	C
NC_040275.1	29311432	C	G
NC_040275.1	29311582	G	A
NC_040275.1	29374045	A	T
NC_040275.1	29431109	T	C
NC_040275.1	29486816	G	A
NC_040275.1	29539660	G	T
NC_040275.1	29596307	T	G
NC_040275.1	29653730	T	A
NC_040275.1	29709766	G	A
NC_040275.1	29749400	T	C
NC_040275.1	29797175	T	C
NC_040275.1	29828084	A	C
NC_040275.1	29828328	T	A

NC_040275.1	29830593	C	T
NC_040275.1	29831109	A	G
NC_040275.1	29885063	C	T
NC_040275.1	29948428	A	C
NC_040275.1	30001242	T	G
NC_040275.1	30058007	G	A
NC_040275.1	30119429	T	C
NC_040275.1	30180379	A	G
NC_040275.1	30243967	A	G
NC_040275.1	30291394	A	G
NC_040275.1	30516963	A	G
NC_040275.1	30517182	G	A
NC_040275.1	30518202	T	C
NC_040275.1	30518604	C	T
NC_040275.1	30518802	A	G
NC_040275.1	30519035	T	C
NC_040275.1	30577638	C	T
NC_040275.1	30637177	G	C
NC_040275.1	30693382	C	T
NC_040275.1	30747947	A	G
NC_040275.1	30806236	C	G
NC_040275.1	30866288	G	A
NC_040275.1	30929422	T	C
NC_040275.1	30990303	G	A
NC_040275.1	31050548	C	A
NC_040275.1	31107998	A	C
NC_040275.1	31171811	G	A
NC_040275.1	31232676	A	G
NC_040275.1	31289029	G	C
NC_040275.1	31348521	A	G
NC_040275.1	31407965	C	T
NC_040275.1	31466589	C	T
NC_040275.1	31527082	A	G
NC_040275.1	31589229	G	T
NC_040275.1	31649388	A	G
NC_040275.1	31720216	A	G
NC_040275.1	33319561	C	T
NC_040275.1	33362269	A	C
NC_040275.1	33367485	T	C
NC_040275.1	33368263	C	T
NC_040275.1	33369415	A	G
NC_040275.1	33436818	G	C
NC_040275.1	33463108	G	A
NC_040275.1	33514519	T	C
NC_040275.1	33551889	T	C
NC_040275.1	33553732	T	C
NC_040275.1	33559818	T	C

NC_040275.1	33618354	G	A
NC_040275.1	33655539	T	C
NC_040275.1	33681650	A	G
NC_040275.1	33682886	A	G
NC_040275.1	33729942	T	C
NC_040275.1	33766494	G	C
NC_040275.1	33767184	C	T
NC_040275.1	33767351	A	G
NC_040275.1	33767897	A	G
NC_040275.1	33826360	T	G
NC_040275.1	33881918	A	G
NC_040275.1	33905975	C	A
NC_040275.1	33955083	A	G
NC_040275.1	33957402	A	G
NC_040275.1	33989469	G	T
NC_040275.1	33990013	C	T
NC_040275.1	33994404	T	C
NC_040275.1	34003192	G	C
NC_040275.1	34009014	G	A
NC_040275.1	34158483	T	C
NC_040275.1	34159356	A	G
NC_040275.1	34191411	C	T
NC_040275.1	34192608	G	C
NC_040275.1	34223000	T	C
NC_040275.1	34245867	T	C
NC_040275.1	34246697	C	T
NC_040275.1	34247753	A	G
NC_040275.1	34271644	T	C
NC_040275.1	34288617	C	T
NC_040275.1	34288906	C	T
NC_040275.1	34289578	A	G
NC_040275.1	34296918	T	C
NC_040275.1	34337137	T	C
NC_040275.1	34341964	T	C
NC_040275.1	34345312	T	C
NC_040275.1	34345511	A	G
NC_040275.1	34357131	A	G
NC_040275.1	34357630	A	G
NC_040275.1	34358451	G	A
NC_040275.1	34359045	A	T
NC_040275.1	34382392	C	T
NC_040275.1	34382882	C	A
NC_040275.1	34438960	T	C
NC_040275.1	34439186	G	A
NC_040275.1	34439337	A	G
NC_040275.1	34440034	A	G
NC_040275.1	34444976	T	C

NC_040275.1	34445144	A		G
NC_040275.1	34446472		0	A
NC_040275.1	34502618	A		G
NC_040275.1	34536367	T		C
NC_040275.1	34537515	G		C
NC_040275.1	34537677	G		A
NC_040275.1	34542678	A		G
NC_040275.1	34553306	T		C
NC_040275.1	34554543	C		T
NC_040275.1	34584094	T		C
NC_040275.1	34585294	T		C
NC_040275.1	34596657	T		C
NC_040275.1	34657652	A		C
NC_040275.1	34700212	C		T
NC_040275.1	34741229	C		T
NC_040275.1	34767323	C		T
NC_040275.1	34817341	A		G
NC_040275.1	34876454	A		G
NC_040275.1	34913291	T		C
NC_040275.1	34951864	G		A
NC_040275.1	35011840	A		G
NC_040275.1	35028071	A		G
NC_040275.1	35030748	T		C
NC_040275.1	35038983	A		G
NC_040275.1	35040409	T		C
NC_040275.1	35040642	T		C
NC_040275.1	35061313	T		C
NC_040275.1	35061942	G		A
NC_040275.1	35062279	C		T
NC_040275.1	35063272	G		T
NC_040275.1	35108197	T		C
NC_040275.1	35116622	A		G
NC_040275.1	35138759	T		C
NC_040275.1	35141765	A		G
NC_040275.1	35142934	T		C
NC_040275.1	35168562	T		C
NC_040275.1	35174780	T		C
NC_040275.1	35183569	A		G
NC_040275.1	35190595	A		G
NC_040275.1	35195022	A		C
NC_040275.1	35205768		0	C
NC_040275.1	35208576	A		G
NC_040275.1	35234533	G		A
NC_040275.1	35250537	G		A
NC_040275.1	35265941	T		C
NC_040275.1	35266288	A		G
NC_040275.1	35269046	A		G

NC_040275.1	35271382	C	T
NC_040275.1	35275439	T	G
NC_040275.1	35279121	T	C
NC_040275.1	35282029	T	G
NC_040275.1	35282263	G	A
NC_040275.1	35284084	C	T
NC_040275.1	35291045	G	T
NC_040275.1	35291498	C	G
NC_040275.1	35292227	A	G
NC_040275.1	35292773	C	T
NC_040275.1	35294744	G	A
NC_040275.1	35295182	A	G
NC_040275.1	35295922	T	C
NC_040275.1	35313232	T	A
NC_040275.1	35314587	C	T
NC_040275.1	35368382	T	C
NC_040275.1	35368570	C	T
NC_040275.1	35369392	G	T
NC_040275.1	35384075	C	T
NC_040275.1	35449604	A	G
NC_040275.1	35508917	A	G
NC_040275.1	35569253	T	C
NC_040275.1	35629337	A	G
NC_040275.1	35689337	C	G
NC_040275.1	35731047	G	A
NC_040275.1	35864406	C	G
NC_040275.1	35923813	C	T
NC_040275.1	35985255	A	G
NC_040275.1	36044223	A	G
NC_040275.1	36099308	A	G
NC_040275.1	36143359	A	G
NC_040275.1	36143796	G	A
NC_040275.1	36147468	T	C
NC_040275.1	36148117	T	C
NC_040275.1	36148302	T	C
NC_040275.1	36149193	A	G
NC_040275.1	36149585	A	C
NC_040275.1	36159686	T	C
NC_040275.1	36166359	T	C
NC_040275.1	36168362	T	C
NC_040275.1	36169975	A	G
NC_040275.1	36173056	C	T
NC_040275.1	36235161	C	T
NC_040275.1	36249111	A	G
NC_040275.1	36249935	G	T
NC_040275.1	36252428	A	G
NC_040275.1	36256533	T	C

NC_040275.1	36258697	A	G
NC_040275.1	36275200	A	G
NC_040275.1	36275964	T	C
NC_040275.1	36303081	A	G
NC_040275.1	36303274	G	C
NC_040275.1	36305226	A	G
NC_040275.1	36358562	C	A
NC_040275.1	36413805	G	C
NC_040275.1	36436032	A	C
NC_040275.1	36436494	C	G
NC_040275.1	36438064	A	G
NC_040275.1	36441646	T	C
NC_040275.1	36447326	A	G
NC_040275.1	36458427	A	G
NC_040275.1	36459604	A	G
NC_040275.1	36460342	T	C
NC_040275.1	36461756	A	G
NC_040275.1	36464856	A	G
NC_040275.1	36465695	T	C
NC_040275.1	36467740	A	G
NC_040275.1	36469760	A	G
NC_040275.1	36469935	C	G
NC_040275.1	36483455	T	C
NC_040275.1	36489450	A	G
NC_040275.1	36501370	G	C
NC_040275.1	36513237	A	C
NC_040275.1	36517331	A	G
NC_040275.1	36518272	A	G
NC_040275.1	36528644	A	G
NC_040275.1	36535304	A	G
NC_040275.1	36555783	A	G
NC_040275.1	36568692	C	T
NC_040275.1	36569084	A	G
NC_040275.1	36574875	A	G
NC_040275.1	36575425	A	G
NC_040275.1	36577867	C	T
NC_040275.1	36579975	G	A
NC_040275.1	36581233	A	G
NC_040275.1	36585679	T	C
NC_040275.1	36610078	A	G
NC_040275.1	36616797	T	A
NC_040275.1	36624272	C	A
NC_040275.1	36631924	A	G
NC_040275.1	36635655	T	C
NC_040275.1	36639686	A	G
NC_040275.1	36650563	A	G
NC_040275.1	36651721	T	C

NC_040275.1	36653843	T	C
NC_040275.1	36655746	A	G
NC_040275.1	36663043	T	C
NC_040275.1	36663542	A	G
NC_040275.1	36665410	G	A
NC_040275.1	36673172	T	C
NC_040275.1	36737300	C	T
NC_040275.1	36739597	A	G
NC_040275.1	36740340	T	C
NC_040275.1	36748055	T	C
NC_040275.1	36748777	T	C
NC_040275.1	36759304	G	A
NC_040275.1	36779948	T	G
NC_040275.1	36782369	A	G
NC_040275.1	36782677	T	C
NC_040275.1	36783617	T	C
NC_040275.1	36783810	T	C
NC_040275.1	36783968	C	T
NC_040275.1	36785544	T	A
NC_040275.1	36793281	C	G
NC_040275.1	36796515	T	G
NC_040275.1	36808352	G	C
NC_040275.1	36808545	C	T
NC_040275.1	36859582	A	T
NC_040275.1	36862584	A	G
NC_040275.1	36862753	T	G
NC_040275.1	36872789	C	A
NC_040275.1	36875255	T	C
NC_040275.1	36883132	G	T
NC_040275.1	36907801	A	C
NC_040275.1	36909080	A	G
NC_040275.1	36910350	A	G
NC_040275.1	36914393	A	G
NC_040275.1	36915998	G	A
NC_040275.1	36935020	T	C
NC_040275.1	36987645	T	C
NC_040275.1	37009165	A	G
NC_040275.1	37009642	G	C
NC_040275.1	37014264	T	A
NC_040275.1	37014682	C	T
NC_040275.1	37030744	G	A
NC_040275.1	37052220	A	C
NC_040275.1	37053191	A	G
NC_040275.1	37054117	A	G
NC_040275.1	37064769	G	A
NC_040275.1	37089261	A	G
NC_040275.1	37149880	T	C

NC_040275.1	37151480	A	G
NC_040275.1	37155796	A	G
NC_040275.1	37183275	A	G
NC_040275.1	37229407	G	A
NC_040275.1	37290559	A	C
NC_040275.1	37349390	T	C
NC_040275.1	37410328	T	C
NC_040275.1	37473698	C	T
NC_040275.1	37487611	G	A
NC_040275.1	37488339	A	G
NC_040275.1	37488575	A	G
NC_040275.1	37490596	A	G
NC_040275.1	37512819	C	G
NC_040275.1	37514996	A	G
NC_040275.1	37516253	A	C
NC_040275.1	37518544	C	T
NC_040275.1	37519386	T	C
NC_040275.1	37519924	T	C
NC_040275.1	37522353	C	T
NC_040275.1	37522505	T	C
NC_040275.1	37529177	C	T
NC_040275.1	37532478	C	G
NC_040275.1	37533296	C	T
NC_040275.1	37534173	G	C
NC_040275.1	37535283	C	T
NC_040275.1	37535893	C	T
NC_040275.1	37536287	A	C
NC_040275.1	37560954	T	C
NC_040275.1	37563603	A	G
NC_040275.1	37567655	C	G
NC_040275.1	37569377	C	T
NC_040275.1	37569591	A	G
NC_040275.1	37576033	G	T
NC_040275.1	37576939	A	G
NC_040275.1	37579105	A	G
NC_040275.1	37593045	A	G
NC_040275.1	37594464	T	C
NC_040275.1	37599614	T	C
NC_040275.1	37613998	G	T
NC_040275.1	37642605	A	G
NC_040275.1	37654889	G	A
NC_040275.1	37665114	C	T
NC_040275.1	37686421	T	C
NC_040275.1	37688499	T	C
NC_040275.1	37723837	G	A
NC_040275.1	37811754	T	G
NC_040275.1	37824395	T	C

NC_040275.1	37859612	A	G
NC_040275.1	37933121	A	T
NC_040275.1	37987878	T	C
NC_040275.1	38015688	T	C
NC_040275.1	38015850	T	C
NC_040275.1	38022042	C	T
NC_040275.1	38038584	G	A
NC_040275.1	38064259	C	T
NC_040275.1	38096532	A	G
NC_040275.1	38227109	C	T
NC_040275.1	38399913	A	G
NC_040275.1	38457668	G	A
NC_040275.1	38505289	C	T
NC_040275.1	38533552	C	A
NC_040275.1	38534355	A	C
NC_040275.1	38534518	A	G
NC_040275.1	38535451	A	G
NC_040275.1	38536665	C	T
NC_040275.1	38539242	T	C
NC_040275.1	38553979	G	A
NC_040275.1	38554159	T	C
NC_040275.1	38554366	A	G
NC_040275.1	38610403	G	A
NC_040275.1	38681864	C	T
NC_040275.1	38686017	T	C
NC_040275.1	38686329	A	C
NC_040275.1	38686599	T	A
NC_040275.1	38686773	T	A
NC_040275.1	38702778	C	A
NC_040275.1	38736632	T	C
NC_040275.1	38740328	T	G
NC_040275.1	38747463	T	G
NC_040275.1	38747828	A	G
NC_040275.1	38756069	A	G
NC_040275.1	38763374	C	T
NC_040275.1	38771019	C	T
NC_040275.1	38795935	A	C
NC_040275.1	38937832	C	G
NC_040275.1	38942429	T	C
NC_040275.1	38945205	T	C
NC_040275.1	38946842	A	G
NC_040275.1	38949670	A	G
NC_040275.1	38953050	T	C
NC_040275.1	38954529	T	C
NC_040275.1	39063898	T	C
NC_040275.1	39126084	T	C
NC_040275.1	39150665	G	A

NC_040275.1	39151508	A	G
NC_040275.1	39213955	A	G
NC_040275.1	39252818	G	C
NC_040275.1	39267992	T	G
NC_040275.1	39284797	T	C
NC_040275.1	39284980	T	C
NC_040275.1	39290888	A	G
NC_040275.1	39294033	T	C
NC_040275.1	39326528	A	C
NC_040275.1	39327441	T	C
NC_040275.1	39329638	A	G
NC_040275.1	39331262	T	A
NC_040275.1	39370077	T	C
NC_040275.1	39370399	A	G
NC_040275.1	39370630	C	G
NC_040275.1	39371628	A	G
NC_040275.1	39373360	G	T
NC_040275.1	39374354	G	A
NC_040275.1	39397232	T	C
NC_040275.1	39397861	T	G
NC_040275.1	39403502	T	G
NC_040275.1	39453231	C	T
NC_040275.1	39456674	A	G
NC_040275.1	39464801	C	T
NC_040275.1	39525906	A	C
NC_040275.1	39556155	G	A
NC_040275.1	39556690	T	C
NC_040275.1	39557694	G	C
NC_040275.1	39570693	A	G
NC_040275.1	39633982	G	A
NC_040275.1	39634362	G	A
NC_040275.1	39635277	T	G
NC_040275.1	39654591	G	C
NC_040275.1	39654938	A	G
NC_040275.1	39656074	T	C
NC_040275.1	39730700	T	C
NC_040275.1	39731000	C	T
NC_040275.1	39768925	C	T
NC_040275.1	39801149	T	C
NC_040275.1	39837982	T	C
NC_040275.1	39839958	T	C
NC_040275.1	39861964	G	T
NC_040275.1	39933377	A	G
NC_040275.1	39965062	T	C
NC_040275.1	39992563	T	C
NC_040275.1	40060522	A	G
NC_040275.1	40326140	T	C

NC_040275.1	40357541	C	T
NC_040275.1	40424464	G	A
NC_040275.1	40483791	A	T
NC_040275.1	40552975	A	G
NC_040275.1	40614820	T	C
NC_040275.1	40671781	C	T
NC_040275.1	40722351	G	C
NC_040275.1	40785183	G	T
NC_040275.1	40827752	T	C
NC_040275.1	40887985	A	G
NC_040275.1	40949187	C	T
NC_040275.1	41021138	A	T
NC_040275.1	41237963	A	G
NC_040275.1	41306947	A	G
NC_040275.1	41377945	G	C
NC_040275.1	41434680	A	G
NC_040275.1	41483327	C	T
NC_040275.1	41511202	G	A
NC_040275.1	41538105	A	C
NC_040275.1	41562348	T	C
NC_040275.1	41634895	A	G
NC_040275.1	41635183	T	G
NC_040275.1	41688224	C	G
NC_040275.1	41692307	T	C
NC_040275.1	41697632	A	G
NC_040275.1	41703182	A	G
NC_040275.1	41747864	C	G
NC_040275.1	41780485	A	G
NC_040275.1	41781332	T	C
NC_040275.1	41841698	T	G
NC_040275.1	41902926	A	G
NC_040275.1	41963300	A	C
NC_040275.1	42002412	C	T
NC_040275.1	42029513	G	T
NC_040275.1	42080892	C	G
NC_040275.1	42081054	T	C
NC_040275.1	42082764	A	G
NC_040275.1	42117399	T	C
NC_040275.1	42141968	T	C
NC_040275.1	42213593	T	C
NC_040275.1	42223100	A	G
NC_040275.1	42229848	C	T
NC_040275.1	42230870	T	C
NC_040275.1	42247364	G	A
NC_040275.1	42250357	T	C
NC_040275.1	42250600	A	G
NC_040275.1	42250774	T	C

NC_040275.1	42257030	T	C	
NC_040275.1	42264189	T	C	
NC_040275.1	42266169	T	C	
NC_040275.1	42287943	A	G	
NC_040275.1	42487938	A	G	
NC_040275.1	42510980	T	C	
NC_040275.1	42513357	G	C	
NC_040275.1	42514047	A	G	
NC_040275.1	42521833	T	C	
NC_040275.1	42524018	C	G	
NC_040275.1	42524366	A	C	
NC_040275.1	42551964	G	C	
NC_040275.1	42559776	T	C	
NC_040275.1	42570678	G	T	
NC_040275.1	42593349	C	T	
NC_040275.1	42610301	T	C	
NC_040275.1	42610477	G	C	
NC_040275.1	42624564	G	A	
NC_040275.1	42628671	A	G	
NC_040275.1	42635040	A	G	
NC_040275.1	42644326	T	C	
NC_040275.1	42648424	T	C	
NC_040275.1	42650155	T	C	
NC_040275.1	42661088	T	C	
NC_040275.1	42662128	T	C	
NC_040275.1	42664917	C	T	
NC_040275.1	42665725	G	A	
NC_040275.1	42712118	C	T	
NC_040275.1	42750579	C	T	
NC_040275.1	42786640		0	0
NC_040275.1	42818050	A	G	
NC_040275.1	42827715	T	C	
NC_040275.1	42829094	T	C	
NC_040275.1	42934602	T	C	
NC_040275.1	42963240	T	C	
NC_040275.1	43059953	C	T	
NC_040276.1	100537	T	G	
NC_040276.1	178913	A	G	
NC_040276.1	236681	T	C	
NC_040276.1	242108	A	C	
NC_040276.1	242273	G	A	
NC_040276.1	769139	G	T	
NC_040276.1	810616	A	G	
NC_040276.1	812028	T	C	
NC_040276.1	833887	A	G	
NC_040276.1	952716	G	C	
NC_040276.1	953692	A	G	

NC_040276.1	955027	A	C
NC_040276.1	1022591	T	A
NC_040276.1	1024014	G	A
NC_040276.1	1024249	T	C
NC_040276.1	1055971	G	C
NC_040276.1	1093325	G	A
NC_040276.1	1131498	T	C
NC_040276.1	1955041	A	G
NC_040276.1	1999291	A	G
NC_040276.1	2066567	T	C
NC_040276.1	2123388	C	G
NC_040276.1	2162756	C	T
NC_040276.1	2571332	T	C
NC_040276.1	2700791	T	C
NC_040276.1	2759788	T	C
NC_040276.1	2818155	A	G
NC_040276.1	2869552	C	G
NC_040276.1	2928966	A	C
NC_040276.1	3122329	G	A
NC_040276.1	3154707	C	G
NC_040276.1	3197171	T	C
NC_040276.1	3248513	C	T
NC_040276.1	3272559	T	C
NC_040276.1	3328189	A	C
NC_040276.1	3465116	T	C
NC_040276.1	3465866	C	G
NC_040276.1	3517294	A	G
NC_040276.1	3621128	A	C
NC_040276.1	3624994	A	G
NC_040276.1	3640399	T	C
NC_040276.1	3662969	G	A
NC_040276.1	3687696	T	G
NC_040276.1	3688807	G	T
NC_040276.1	3689246	A	G
NC_040276.1	3689484	G	T
NC_040276.1	3736021	G	A
NC_040276.1	3902012	A	T
NC_040276.1	3930749	G	A
NC_040276.1	3931593	C	G
NC_040276.1	3931777	A	T
NC_040276.1	4053623	A	G
NC_040276.1	4108586	A	G
NC_040276.1	4174715	T	C
NC_040276.1	4232178	C	G
NC_040276.1	4292619	T	C
NC_040276.1	4363478	C	T
NC_040276.1	4475288	C	A

NC_040276.1	4524586	A	G
NC_040276.1	4867073	A	G
NC_040276.1	4867446	C	T
NC_040276.1	4913961	T	C
NC_040276.1	4947912	C	G
NC_040276.1	4983155	G	A
NC_040276.1	4983393	A	G
NC_040276.1	4983888	G	A
NC_040276.1	5050253	G	T
NC_040276.1	5109200	C	T
NC_040276.1	5332704	A	G
NC_040276.1	5504334	T	C
NC_040276.1	5564745	T	A
NC_040276.1	5624887	G	A
NC_040276.1	5676680	A	C
NC_040276.1	5722412	G	A
NC_040276.1	5752946	A	G
NC_040276.1	5805348	T	C
NC_040276.1	5827890	C	T
NC_040276.1	5870022	G	A
NC_040276.1	5926780	T	C
NC_040276.1	6150556	G	C
NC_040276.1	6184865	T	C
NC_040276.1	6698049	G	T
NC_040276.1	6764853	G	A
NC_040276.1	6826572	A	G
NC_040276.1	6827211	C	T
NC_040276.1	6827390	G	A
NC_040276.1	6873411	G	A
NC_040276.1	6920491	C	G
NC_040276.1	6938206	A	G
NC_040276.1	6938367	T	C
NC_040276.1	7064925	C	A
NC_040276.1	7065617	T	C
NC_040276.1	7065987	A	C
NC_040276.1	7068226	A	G
NC_040276.1	7544929	C	T
NC_040276.1	7590984	T	C
NC_040276.1	7592292	A	C
NC_040276.1	7648710	T	C
NC_040276.1	7703485	C	T
NC_040276.1	7724134	C	T
NC_040276.1	7734077	G	A
NC_040276.1	7788060	C	G
NC_040276.1	7834408	A	G
NC_040276.1	7837334	A	G
NC_040276.1	7892873	A	T

NC_040276.1	7961846	A	G
NC_040276.1	8012938	T	C
NC_040276.1	8047909	G	A
NC_040276.1	8065269	G	A
NC_040276.1	8115693	G	A
NC_040276.1	8123044	C	T
NC_040276.1	8123334	T	C
NC_040276.1	8145277	T	C
NC_040276.1	8146664	C	T
NC_040276.1	8146892	T	C
NC_040276.1	8156474	T	C
NC_040276.1	8165235	C	T
NC_040276.1	8165805	G	A
NC_040276.1	8165997	G	A
NC_040276.1	8168078	C	T
NC_040276.1	8211121	A	G
NC_040276.1	8343627	T	C
NC_040276.1	8363351	T	C
NC_040276.1	8373621	A	G
NC_040276.1	8391927	A	G
NC_040276.1	8394669	T	C
NC_040276.1	8408357	T	C
NC_040276.1	8441922	A	G
NC_040276.1	8514021	T	C
NC_040276.1	8553830	A	G
NC_040276.1	8555613	C	A
NC_040276.1	8556895	C	T
NC_040276.1	8557221	T	C
NC_040276.1	8558164	T	C
NC_040276.1	8559198	A	G
NC_040276.1	8559475	T	C
NC_040276.1	8563527	T	C
NC_040276.1	8574403	A	G
NC_040276.1	8623029	T	G
NC_040276.1	8623199	G	C
NC_040276.1	8731052	G	A
NC_040276.1	8794137	A	G
NC_040276.1	8818380	C	G
NC_040276.1	8839563	G	A
NC_040276.1	8845032	C	G
NC_040276.1	8860586	C	T
NC_040276.1	8870711	A	G
NC_040276.1	8884190	T	C
NC_040276.1	8970329	A	G
NC_040276.1	9003703	C	T
NC_040276.1	9011156	T	C
NC_040276.1	9030145	T	C

NC_040276.1	9062049	G	A
NC_040276.1	9074004	T	C
NC_040276.1	9098618	G	T
NC_040276.1	9101804	C	T
NC_040276.1	9142987	A	G
NC_040276.1	9180466	A	G
NC_040276.1	9185489	T	C
NC_040276.1	9336473	T	G
NC_040276.1	9393694	C	T
NC_040276.1	9450681	T	C
NC_040276.1	9500088	G	A
NC_040276.1	9555426	T	C
NC_040276.1	9619028	T	C
NC_040276.1	9678563	A	G
NC_040276.1	9730476	T	C
NC_040276.1	9786838	A	G
NC_040276.1	9838657	C	T
NC_040276.1	9908793	T	C
NC_040276.1	9968352	A	C
NC_040276.1	10028633	A	G
NC_040276.1	10073082	A	G
NC_040276.1	10073508	T	C
NC_040276.1	10073700	T	C
NC_040276.1	10133647	A	T
NC_040276.1	10216154	T	A
NC_040276.1	10217953	A	G
NC_040276.1	11947544	G	A
NC_040276.1	12002591	G	T
NC_040276.1	12054748	T	C
NC_040276.1	12113890	T	C
NC_040276.1	12168255	A	G
NC_040276.1	12223077	T	C
NC_040276.1	12282899	C	T
NC_040276.1	12345863	T	A
NC_040276.1	12410964	T	C
NC_040276.1	12451467	A	G
NC_040276.1	12750555	G	T
NC_040276.1	12750740	A	G
NC_040276.1	12774235	A	G
NC_040276.1	12776840	A	G
NC_040276.1	12871083	C	T
NC_040276.1	12897718	A	G
NC_040276.1	12897874	T	C
NC_040276.1	12898599	A	G
NC_040276.1	12898781	T	A
NC_040276.1	12974517	T	C
NC_040276.1	12995535	A	G

NC_040276.1	12995827	C	T
NC_040276.1	13001668	G	A
NC_040276.1	13227133	A	G
NC_040276.1	13252382	G	C
NC_040276.1	13267095	T	C
NC_040276.1	13280858	T	C
NC_040276.1	13281349	A	G
NC_040276.1	13314257	G	A
NC_040276.1	13314491	G	C
NC_040276.1	13317315	G	A
NC_040276.1	13322159	C	A
NC_040276.1	13341072	T	C
NC_040276.1	13341987	A	T
NC_040276.1	13343136	G	A
NC_040276.1	13351345	T	G
NC_040276.1	13351539	A	G
NC_040276.1	13351720	A	G
NC_040276.1	13385846	C	T
NC_040276.1	13549523	A	G
NC_040276.1	13565096	A	G
NC_040276.1	13568134	T	A
NC_040276.1	13568611	T	G
NC_040276.1	13580706	A	G
NC_040276.1	13732452	T	C
NC_040276.1	13787958	T	C
NC_040276.1	13845338	G	A
NC_040276.1	13909181	C	T
NC_040276.1	13955693	A	T
NC_040276.1	14028705	C	T
NC_040276.1	14079776	A	G
NC_040276.1	14547766	C	A
NC_040276.1	14589233	A	T
NC_040276.1	14641044	A	T
NC_040276.1	14661271	T	C
NC_040276.1	14718876	C	A
NC_040276.1	14770751	T	C
NC_040276.1	14801232	G	T
NC_040276.1	15087383	T	C
NC_040276.1	15088790	T	G
NC_040276.1	15088941	T	G
NC_040276.1	15133203	A	C
NC_040276.1	15186742	A	G
NC_040276.1	15201349	C	T
NC_040276.1	15227412	C	T
NC_040276.1	15283942	G	A
NC_040276.1	15335042	G	A
NC_040276.1	15449724	T	C

NC_040276.1	15450378	T	C
NC_040276.1	15450575	C	T
NC_040276.1	15490790	C	T
NC_040276.1	15496616	A	G
NC_040276.1	15498890	T	C
NC_040276.1	15500015	T	C
NC_040276.1	15502064	A	G
NC_040276.1	15503360	C	T
NC_040276.1	15503711	C	T
NC_040276.1	15513620	G	A
NC_040276.1	15570593	A	G
NC_040276.1	15617560	T	C
NC_040276.1	15623275	A	G
NC_040276.1	15681326	T	C
NC_040276.1	15741526	T	C
NC_040276.1	15796146	C	T
NC_040276.1	15829896	A	G
NC_040276.1	16255958	G	C
NC_040276.1	16345231	T	G
NC_040276.1	16345516	T	C
NC_040276.1	16365383	A	G
NC_040276.1	16421053	A	G
NC_040276.1	16967098	T	C
NC_040276.1	16967297	G	A
NC_040276.1	17029870	A	G
NC_040276.1	17295323	G	A
NC_040276.1	17351407	T	A
NC_040276.1	17406262	T	C
NC_040276.1	17454739	C	T
NC_040276.1	17612707	A	G
NC_040276.1	17669023	C	T
NC_040276.1	17724980	A	G
NC_040276.1	17779342	T	C
NC_040276.1	17798393	G	T
NC_040276.1	17798964	G	C
NC_040276.1	17801140	C	T
NC_040276.1	17802755	T	C
NC_040276.1	17803683	A	G
NC_040276.1	18002641	G	A
NC_040276.1	18009287	T	C
NC_040276.1	18053199	T	C
NC_040276.1	18097290	A	T
NC_040276.1	18257351	C	T
NC_040276.1	18258272	A	G
NC_040276.1	18281726	G	A
NC_040276.1	18282402	T	G
NC_040276.1	18282619	G	C

NC_040276.1	18553992	A	G
NC_040276.1	18719155	A	T
NC_040276.1	18720697	C	T
NC_040276.1	18724884	T	C
NC_040276.1	19112141	C	A
NC_040276.1	19151571	C	T
NC_040276.1	19176403	G	A
NC_040276.1	19181823	T	C
NC_040276.1	19192398	T	C
NC_040276.1	19194014	C	T
NC_040276.1	19197373	A	G
NC_040276.1	19197665	A	G
NC_040276.1	19254646	G	T
NC_040276.1	19310070	C	G
NC_040276.1	19360617	T	G
NC_040276.1	19413698	C	T
NC_040276.1	19471396	C	T
NC_040276.1	19471643	0	C
NC_040276.1	19520296	A	G
NC_040276.1	19568221	A	G
NC_040276.1	19570726	G	A
NC_040276.1	22901526	G	A
NC_040276.1	22902219	G	A
NC_040276.1	23209895	G	T
NC_040276.1	23265943	C	A
NC_040276.1	23322002	G	A
NC_040276.1	23384907	G	A
NC_040276.1	23444099	A	C
NC_040276.1	23498519	C	A
NC_040276.1	23560249	T	C
NC_040276.1	23625733	T	C
NC_040276.1	23836225	A	C
NC_040276.1	23900386	T	C
NC_040276.1	23950067	C	T
NC_040276.1	23997302	T	C
NC_040276.1	24053337	C	A
NC_040276.1	24122307	A	C
NC_040276.1	24179773	C	T
NC_040276.1	24237972	C	A
NC_040276.1	24294461	A	G
NC_040276.1	24352230	T	C
NC_040276.1	24353948	C	G
NC_040276.1	24410699	C	T
NC_040276.1	24465441	A	G
NC_040276.1	24522747	C	T
NC_040276.1	24578113	T	C
NC_040276.1	24633829	G	T

NC_040276.1	24686827	T	C
NC_040276.1	24714843	C	T
NC_040276.1	24814902	G	A
NC_040276.1	24869455	G	A
NC_040276.1	24922445	A	G
NC_040276.1	24991320	G	A
NC_040276.1	25053938	T	C
NC_040276.1	25098900	C	T
NC_040276.1	25143069	T	C
NC_040276.1	25212025	A	G
NC_040276.1	25232672	A	G
NC_040276.1	25347150	G	A
NC_040276.1	25379187	T	C
NC_040276.1	25425123	A	G
NC_040276.1	25481074	G	C
NC_040276.1	25537411	A	G
NC_040276.1	25567176	T	C
NC_040276.1	25581573	G	T
NC_040276.1	25582455	G	C
NC_040276.1	25582695	A	G
NC_040276.1	25593621	T	C
NC_040276.1	25618372	C	T
NC_040276.1	25618550	A	C
NC_040276.1	25619183	T	A
NC_040276.1	25619438	A	G
NC_040276.1	25619976	T	C
NC_040276.1	25620744	C	A
NC_040276.1	25621027	A	C
NC_040276.1	25622864	C	T
NC_040276.1	25623698	A	G
NC_040276.1	25624806	T	C
NC_040276.1	25625158	C	T
NC_040276.1	25626391	A	T
NC_040276.1	25656510	T	C
NC_040276.1	25660204	C	T
NC_040276.1	25693959	G	T
NC_040276.1	25694203	T	C
NC_040276.1	25694511	A	T
NC_040276.1	25694868	T	C
NC_040276.1	25695734	A	G
NC_040276.1	25695907	G	T
NC_040276.1	25702695	A	G
NC_040276.1	25724930	A	G
NC_040276.1	25725736	G	A
NC_040276.1	25745328	T	G
NC_040276.1	25750353	C	T
NC_040276.1	25758911	A	G

NC_040276.1	25770850	T	C
NC_040276.1	25784848	A	G
NC_040276.1	25819822	G	C
NC_040276.1	25858618	G	C
NC_040276.1	25900353	C	T
NC_040276.1	25934949	A	G
NC_040276.1	25935419	T	G
NC_040276.1	25935590	A	G
NC_040276.1	25936470	C	A
NC_040276.1	25936696	G	A
NC_040276.1	25979082	C	T
NC_040276.1	25980601	A	C
NC_040276.1	25982013	C	T
NC_040276.1	25984738	T	G
NC_040276.1	25985009	G	A
NC_040276.1	25985281	A	G
NC_040276.1	25986097	C	T
NC_040276.1	26008176	G	T
NC_040276.1	26028907	A	G
NC_040276.1	26033369	G	A
NC_040276.1	26111790	A	G
NC_040276.1	26132039	T	C
NC_040276.1	26178086	G	A
NC_040276.1	26251409	A	G
NC_040276.1	26402085	T	C
NC_040276.1	26452394	T	C
NC_040276.1	26506446	T	C
NC_040276.1	26542620	G	A
NC_040276.1	26604534	A	G
NC_040276.1	26658182	C	T
NC_040276.1	26676531	T	C
NC_040276.1	26678264	C	T
NC_040276.1	26678869	C	T
NC_040276.1	26689246	A	G
NC_040276.1	26735272	T	A
NC_040276.1	26765219	C	T
NC_040276.1	26860377	A	G
NC_040276.1	27012595	T	G
NC_040276.1	27064984	T	C
NC_040276.1	27119487	T	C
NC_040276.1	27162505	A	G
NC_040276.1	27219445	G	A
NC_040276.1	27279192	T	G
NC_040276.1	27287174	G	A
NC_040276.1	27288477	T	C
NC_040276.1	27288722	T	C
NC_040276.1	27295398	T	C

NC_040276.1	27298737	A	G
NC_040276.1	27348676	T	C
NC_040276.1	27379636	T	C
NC_040276.1	27418667	T	C
NC_040276.1	27419750	C	T
NC_040276.1	27420014	A	G
NC_040276.1	27421091	A	G
NC_040276.1	27475888	T	C
NC_040276.1	27523280	G	A
NC_040276.1	27543824	A	G
NC_040276.1	27545146	C	T
NC_040276.1	27549455	A	T
NC_040276.1	27553153	T	C
NC_040276.1	27555533	G	A
NC_040276.1	27606821	T	C
NC_040276.1	27642610	C	T
NC_040276.1	27676936	T	C
NC_040276.1	27677915	T	C
NC_040276.1	27700995	G	T
NC_040276.1	27760466	G	A
NC_040276.1	27813808	A	G
NC_040276.1	27822232	T	C
NC_040276.1	27841962	A	G
NC_040276.1	27896514	A	G
NC_040276.1	27918059	G	A
NC_040276.1	28000201	G	C
NC_040276.1	28022242	G	A
NC_040276.1	28026580	T	C
NC_040276.1	28088094	T	G
NC_040276.1	28088451	A	G
NC_040276.1	28423091	T	G
NC_040276.1	28490721	A	G
NC_040276.1	28508707	A	G
NC_040276.1	28510444	A	G
NC_040276.1	28513966	A	G
NC_040276.1	28558960	T	C
NC_040276.1	28569925	A	G
NC_040276.1	28570781	A	G
NC_040276.1	28571105	A	G
NC_040276.1	28626524	A	C
NC_040276.1	28690025	T	C
NC_040276.1	28745818	G	C
NC_040276.1	28802026	A	G
NC_040276.1	28858075	C	T
NC_040276.1	28924279	C	T
NC_040276.1	28939705	A	G
NC_040276.1	28940555	A	G

NC_040276.1	28954991	G	A
NC_040276.1	28956172	A	G
NC_040276.1	29004542	G	A
NC_040276.1	29029494	T	C
NC_040276.1	29032356	C	T
NC_040276.1	29036754	T	C
NC_040276.1	29037409	G	A
NC_040276.1	29066163	A	C
NC_040276.1	29131585	G	A
NC_040276.1	29163166	A	G
NC_040276.1	29163763	A	G
NC_040276.1	29210939	T	C
NC_040276.1	29234457	T	C
NC_040276.1	29234773	A	G
NC_040276.1	29304618	A	G
NC_040276.1	29367428	G	A
NC_040276.1	29395873	T	C
NC_040276.1	29435840	G	A
NC_040276.1	29436592	G	C
NC_040276.1	29436915	G	A
NC_040276.1	29471201	C	T
NC_040276.1	29472342	T	C
NC_040276.1	29527459	A	T
NC_040276.1	29591383	C	T
NC_040276.1	29655335	C	T
NC_040276.1	29663699	G	T
NC_040276.1	29664295	T	C
NC_040276.1	29699213	T	A
NC_040276.1	29800468	T	C
NC_040276.1	29854493	G	T
NC_040276.1	29894766	C	A
NC_040276.1	29939832	G	C
NC_040276.1	29980556	T	C
NC_040276.1	30014692	G	A
NC_040276.1	30039407	G	C
NC_040276.1	30125786	T	C
NC_040276.1	30167928	A	G
NC_040276.1	30201602	A	G
NC_040276.1	30250798	C	T
NC_040276.1	30253636	T	C
NC_040276.1	30311135	G	C
NC_040276.1	30342646	C	A
NC_040276.1	30352716	G	A
NC_040276.1	30395412	T	G
NC_040276.1	30434784	G	A
NC_040276.1	30435013	A	G
NC_040276.1	30446561	C	T

NC_040276.1	30449713	A	G
NC_040276.1	30463769	T	C
NC_040276.1	30474028	T	C
NC_040276.1	30474734	A	G
NC_040276.1	30475107	C	T
NC_040276.1	30481446	G	A
NC_040276.1	30482714	A	G
NC_040276.1	30483331	A	G
NC_040276.1	30484113	C	G
NC_040276.1	30539065	C	T
NC_040276.1	30595754	C	A
NC_040276.1	30658971	A	G
NC_040276.1	30725040	A	G
NC_040276.1	30736237	A	G
NC_040276.1	30747126	T	C
NC_040276.1	30750223	T	G
NC_040276.1	30765354	T	C
NC_040276.1	30773445	T	C
NC_040276.1	30773624	A	G
NC_040276.1	30773970	G	T
NC_040276.1	30781257	T	C
NC_040276.1	30781410	A	G
NC_040276.1	30786997	G	A
NC_040276.1	30787199	T	C
NC_040276.1	30787988	G	A
NC_040276.1	30788158	T	C
NC_040276.1	30791573	T	C
NC_040276.1	30792037	G	A
NC_040276.1	30792342	C	T
NC_040276.1	30845549	C	A
NC_040276.1	30885260	G	A
NC_040276.1	30886008	G	A
NC_040276.1	30886854	C	T
NC_040276.1	30965375	G	T
NC_040276.1	31014129	A	G
NC_040276.1	31077264	T	C
NC_040276.1	31097565	C	G
NC_040276.1	31098063	T	C
NC_040276.1	31098271	A	G
NC_040276.1	31099252	T	C
NC_040276.1	31101767	A	G
NC_040276.1	31120811	C	T
NC_040276.1	31178848	C	G
NC_040276.1	31236947	G	A
NC_040276.1	31286085	C	T
NC_040276.1	31343514	A	G
NC_040276.1	31405571	T	C

NC_040276.1	31462387	A	G
NC_040276.1	31520904	C	T
NC_040276.1	31578297	C	T
NC_040276.1	31634081	A	G
NC_040276.1	31662638	C	T
NC_040276.1	31737730	C	G
NC_040276.1	31790679	G	A
NC_040276.1	31845475	T	C
NC_040276.1	31871025	A	G
NC_040276.1	31871272	T	C
NC_040276.1	31871470	T	G
NC_040276.1	31908974	T	G
NC_040276.1	31916708	G	A
NC_040276.1	31916875	G	A
NC_040276.1	31917544	T	C
NC_040276.1	31918237	A	G
NC_040276.1	31918480	A	G
NC_040276.1	31949976	A	G
NC_040276.1	31975801	A	G
NC_040276.1	31975957	A	G
NC_040276.1	31977899	A	C
NC_040276.1	31978055	G	C
NC_040276.1	32041184	T	C
NC_040276.1	32041490	T	C
NC_040276.1	32041807	G	A
NC_040276.1	32041999	C	G
NC_040276.1	32042829	T	G
NC_040276.1	32045067	A	G
NC_040276.1	32084007	A	G
NC_040276.1	32237851	A	G
NC_040276.1	32238052	A	G
NC_040276.1	32238223	A	G
NC_040276.1	32271968	C	G
NC_040276.1	32324969	T	C
NC_040276.1	32381878	T	C
NC_040276.1	32439987	G	A
NC_040276.1	32496795	G	A
NC_040276.1	32554053	T	C
NC_040276.1	32611831	T	G
NC_040276.1	32667010	T	C
NC_040276.1	32737170	G	A
NC_040276.1	32793458	C	T
NC_040276.1	32850059	A	G
NC_040276.1	32907738	T	C
NC_040276.1	32964423	T	C
NC_040276.1	33019126	G	A
NC_040276.1	33086370	T	C

NC_040276.1	33144633	G	T
NC_040276.1	33201118	A	G
NC_040276.1	33235489	G	A
NC_040276.1	33302710	T	C
NC_040276.1	33355948	A	G
NC_040276.1	33408723	T	A
NC_040276.1	33704481	A	C
NC_040276.1	33707353	A	T
NC_040276.1	33707818	A	G
NC_040276.1	33718130	T	C
NC_040276.1	33719961	A	G
NC_040276.1	33772658	A	G
NC_040276.1	33827286	G	T
NC_040276.1	33886302	C	T
NC_040276.1	33959227	A	G
NC_040276.1	34014737	T	A
NC_040276.1	34069986	T	G
NC_040276.1	34126643	A	G
NC_040276.1	34176031	C	T
NC_040276.1	34233121	T	G
NC_040276.1	34290798	A	G
NC_040276.1	34348110	T	C
NC_040276.1	34404484	T	C
NC_040276.1	34453869	G	A
NC_040276.1	34733319	G	C
NC_040276.1	34737023	A	G
NC_040276.1	34748169	T	C
NC_040276.1	34748511	C	T
NC_040276.1	34762973	C	T
NC_040276.1	34822637	A	G
NC_040276.1	34886366	A	C
NC_040276.1	34914843	G	A
NC_040276.1	34915020	C	T
NC_040276.1	34929204	T	C
NC_040276.1	35964989	A	G
NC_040276.1	36022349	G	C
NC_040276.1	36078068	A	G
NC_040276.1	36128281	T	C
NC_040276.1	36148929	A	G
NC_040276.1	36150380	A	G
NC_040276.1	36176455	G	A
NC_040276.1	36230819	T	C
NC_040276.1	36259440	G	A
NC_040276.1	36325464	G	C
NC_040276.1	36382314	A	G
NC_040276.1	36434814	T	C
NC_040276.1	36748993	A	G

NC_040276.1	36759988	T	A
NC_040276.1	36760490	T	C
NC_040276.1	36780363	C	G
NC_040276.1	36822653	A	G
NC_040276.1	36832223	G	A
NC_040276.1	36867907	A	G
NC_040276.1	36916263	T	C
NC_040276.1	36924636	A	G
NC_040276.1	36924823	C	T
NC_040276.1	36982103	T	C
NC_040276.1	37032371	C	T
NC_040276.1	37089005	C	T
NC_040276.1	39699435	C	G
NC_040276.1	39714924	A	C
NC_040276.1	39721132	A	G
NC_040276.1	39721557	A	G
NC_040276.1	40999151	G	T
NC_040276.1	41010912	A	G
NC_040276.1	41011672	T	C
NC_040276.1	41011822	A	G
NC_040276.1	41029658	T	C
NC_040276.1	41044234	A	G
NC_040276.1	41099436	A	G
NC_040276.1	41152695	C	G
NC_040276.1	41183108	T	C
NC_040276.1	41183309	T	C
NC_040276.1	41183465	C	T
NC_040276.1	41183966	C	T
NC_040276.1	41220227	A	G
NC_040276.1	41276884	T	A
NC_040276.1	41304564	A	G
NC_040276.1	41305057	T	C
NC_040276.1	41305569	C	T
NC_040276.1	42261283	C	T
NC_040276.1	42319521	C	T
NC_040276.1	42376005	C	T
NC_040276.1	42432082	C	T
NC_040276.1	42485586	A	G
NC_040276.1	42540470	A	G
NC_040276.1	42597883	T	G
NC_040276.1	42655192	A	G
NC_040276.1	42704959	A	T
NC_040276.1	42775184	A	G
NC_040276.1	42786798	A	G
NC_040276.1	42845885	A	C
NC_040276.1	42903305	A	G
NC_040276.1	42932288	C	G

NC_040276.1	43011090	G	A
NC_040276.1	43011614	A	G
NC_040276.1	43012304	G	T
NC_040276.1	43062546	A	G
NC_040276.1	43087017	T	C
NC_040276.1	43164401	C	T
NC_040276.1	43168766	T	C
NC_040276.1	43170705	T	C
NC_040276.1	43206710	G	A
NC_040276.1	43206936	T	C
NC_040276.1	43207149	A	G
NC_040276.1	43221992	T	C
NC_040276.1	43234387	A	G
NC_040276.1	43235489	A	G
NC_040276.1	43236273	T	C
NC_040276.1	43255893	G	C
NC_040276.1	43307265	G	C
NC_040276.1	43363392	T	C
NC_040276.1	43396727	T	C
NC_040276.1	43406888	G	A
NC_040276.1	43407237	C	T
NC_040276.1	43410812	A	G
NC_040276.1	43411304	A	G
NC_040276.1	43411511	C	T
NC_040276.1	43421605	T	G
NC_040276.1	43435413	C	G
NC_040276.1	43501940	C	A
NC_040276.1	43564329	G	A
NC_040276.1	43564550	G	A
NC_040276.1	43591626	G	A
NC_040276.1	43616595	G	A
NC_040276.1	43643051	A	G
NC_040276.1	43728323	T	C
NC_040276.1	43772537	G	A
NC_040276.1	43806451	A	G
NC_040276.1	44008468	C	T
NC_040276.1	44035176	C	A
NC_040276.1	44056672	A	G
NC_040276.1	44056912	T	G
NC_040276.1	44057139	C	T
NC_040276.1	44065104	A	G
NC_040276.1	44065278	A	G
NC_040276.1	44086805	A	G
NC_040276.1	44108229	C	T
NC_040276.1	44425536	G	C
NC_040276.1	44472620	G	A
NC_040276.1	44476525	G	C

NC_040276.1	44537864	T	C
NC_040276.1	44590500	T	C
NC_040276.1	44653900	G	A
NC_040276.1	44681724	C	T
NC_040276.1	44683185	A	G
NC_040276.1	44683485	T	C
NC_040276.1	44740038	T	C
NC_040276.1	44795459	A	G
NC_040276.1	44855582	A	T
NC_040276.1	44914167	G	A
NC_040276.1	44931754	C	T
NC_040276.1	44946918	T	C
NC_040276.1	44982599	G	A
NC_040276.1	45039993	A	G
NC_040276.1	45094072	C	T
NC_040276.1	45137630	T	C
NC_040276.1	45172924	C	A
NC_040276.1	45225635	A	C
NC_040276.1	45270979	C	T
NC_040276.1	45318367	T	C
NC_040276.1	45376183	G	A
NC_040276.1	45394604	T	C
NC_040276.1	45395163	A	G
NC_040276.1	45395634	A	G
NC_040276.1	45396240	A	G
NC_040276.1	45396796	A	G
NC_040276.1	45396998	G	A
NC_040276.1	45399269	T	C
NC_040276.1	45399705	T	A
NC_040276.1	45400098	T	C
NC_040276.1	45401336	A	G
NC_040276.1	45419799	G	A
NC_040276.1	45420486	T	C
NC_040276.1	45421098	A	G
NC_040276.1	45421428	T	C
NC_040276.1	45421928	T	C
NC_040276.1	45422525	A	C
NC_040276.1	45474266	G	C
NC_040276.1	45501365	T	C
NC_040276.1	45502465	A	G
NC_040276.1	45502639	C	G
NC_040276.1	45509379	T	C
NC_040276.1	45520299	C	A
NC_040276.1	45557623	T	C
NC_040276.1	45608492	A	G
NC_040276.1	45608651	A	C
NC_040276.1	45608885	G	A

NC_040276.1	45666117	A	G
NC_040276.1	45669191	A	G
NC_040276.1	45681104	T	C
NC_040276.1	45718445	G	A
NC_040276.1	45774535	T	A
NC_040276.1	45831414	A	G
NC_040276.1	45894254	T	A
NC_040276.1	45939665	T	C
NC_040276.1	46001078	A	G
NC_040276.1	46025523	G	A
NC_040276.1	46040972	A	G
NC_040276.1	46089840	C	G
NC_040276.1	46147382	A	G
NC_040276.1	46206155	T	C
NC_040276.1	46210564	T	C
NC_040276.1	46212497	T	C
NC_040276.1	46212788	G	C
NC_040276.1	46213558	G	C
NC_040276.1	46221955	T	C
NC_040276.1	46227118	A	G
NC_040276.1	46262107	T	C
NC_040276.1	46353733	T	G
NC_040276.1	46353949	T	C
NC_040276.1	46366652	A	G
NC_040276.1	46375229	G	A
NC_040276.1	46433132	A	G
NC_040276.1	46801573	T	C
NC_040276.1	46804512	T	C
NC_040276.1	46827471	C	T
NC_040276.1	47443040	T	A
NC_040276.1	47443920	A	G
NC_040276.1	47470069	A	G
NC_040276.1	47481129	T	C
NC_040276.1	47481753	A	G
NC_040276.1	47482994	C	T
NC_040276.1	47484226	T	G
NC_040276.1	47486221	A	G
NC_040276.1	47486881	A	G
NC_040276.1	47507818	T	C
NC_040276.1	47567213	C	T
NC_040276.1	47629930	T	C
NC_040277.1	40624	A	C
NC_040277.1	106676	T	G
NC_040277.1	151163	A	C
NC_040277.1	228881	A	C
NC_040277.1	302783	T	C
NC_040277.1	348437	A	G

NC_040277.1	401662	C	T
NC_040277.1	454269	A	G
NC_040277.1	516658	C	T
NC_040277.1	569233	C	T
NC_040277.1	626185	0	G
NC_040277.1	682992	T	A
NC_040277.1	737181	A	G
NC_040277.1	794273	C	T
NC_040277.1	851690	G	C
NC_040277.1	906786	T	C
NC_040277.1	960608	A	G
NC_040277.1	1018200	A	G
NC_040277.1	1067350	T	C
NC_040277.1	1116585	G	A
NC_040277.1	1161844	A	G
NC_040277.1	1186296	T	C
NC_040277.1	1242317	A	G
NC_040277.1	1294441	A	G
NC_040277.1	1330660	T	C
NC_040277.1	1386998	G	A
NC_040277.1	1429935	T	C
NC_040277.1	1454741	G	A
NC_040277.1	1455308	A	G
NC_040277.1	1456268	T	G
NC_040277.1	1487677	G	C
NC_040277.1	1544969	G	A
NC_040277.1	1579733	G	A
NC_040277.1	1662525	C	G
NC_040277.1	1700016	T	C
NC_040277.1	1785593	T	C
NC_040277.1	1838721	A	G
NC_040277.1	1894262	T	C
NC_040277.1	1950139	A	G
NC_040277.1	2005266	G	T
NC_040277.1	2041706	A	C
NC_040277.1	2108356	C	T
NC_040277.1	2162775	T	C
NC_040277.1	2222437	A	G
NC_040277.1	2244799	T	C
NC_040277.1	2305194	T	G
NC_040277.1	2368168	G	A
NC_040277.1	2426181	T	C
NC_040277.1	2470455	T	C
NC_040277.1	2533743	T	C
NC_040277.1	2578911	G	T
NC_040277.1	2618561	G	C
NC_040277.1	2682269	A	G

NC_040277.1	2713365	A		G
NC_040277.1	2764037	G		A
NC_040277.1	2821439	G		A
NC_040277.1	2875099	A		G
NC_040277.1	2934019	G		C
NC_040277.1	2972746	G		A
NC_040277.1	3195989	A		G
NC_040277.1	3234653	T		C
NC_040277.1	3315498		0	C
NC_040277.1	3370883	G		C
NC_040277.1	3441703	T		C
NC_040277.1	3527751	C		G
NC_040277.1	3585992	C		T
NC_040277.1	3641214	A		G
NC_040277.1	3698129	C		G
NC_040277.1	3763957	A		C
NC_040277.1	3833834	T		C
NC_040277.1	3890351	C		T
NC_040277.1	3931049	A		G
NC_040277.1	3980319	A		G
NC_040277.1	4042954	G		T
NC_040277.1	4085777	A		G
NC_040277.1	4149776	T		C
NC_040277.1	4207348	G		T
NC_040277.1	4259656	T		C
NC_040277.1	4316868	A		G
NC_040277.1	4370736	C		T
NC_040277.1	4437506	A		G
NC_040277.1	4502575	T		C
NC_040277.1	4571478	A		G
NC_040277.1	4631758	T		C
NC_040277.1	4683079	A		G
NC_040277.1	4729688	C		T
NC_040277.1	4914249	T		G
NC_040277.1	4975714	G		A
NC_040277.1	5022915	G		C
NC_040277.1	5081384	T		C
NC_040277.1	5126115	C		A
NC_040277.1	5177047	C		T
NC_040277.1	5230616	G		A
NC_040277.1	5409462	A		G
NC_040277.1	5463907	T		C
NC_040277.1	5521123	C		T
NC_040277.1	5588484	T		A
NC_040277.1	5666816	C		T
NC_040277.1	5734313	G		A
NC_040277.1	5803124	C		T

NC_040277.1	5852454	A	G
NC_040277.1	5911966	A	G
NC_040277.1	5970361	C	T
NC_040277.1	6014955	T	A
NC_040277.1	6068492	C	T
NC_040277.1	6126879	A	G
NC_040277.1	6187468	A	G
NC_040277.1	6245179	C	A
NC_040277.1	6293228	T	C
NC_040277.1	6323496	T	C
NC_040277.1	6384220	T	C
NC_040277.1	6427762	T	C
NC_040277.1	6499071	G	A
NC_040277.1	6554968	A	G
NC_040277.1	6601924	C	T
NC_040277.1	6655755	T	C
NC_040277.1	6723391	A	G
NC_040277.1	6793601	C	T
NC_040277.1	7001553	C	T
NC_040277.1	7061245	T	C
NC_040277.1	7116603	C	T
NC_040277.1	7174497	T	G
NC_040277.1	7228725	T	C
NC_040277.1	7289445	T	A
NC_040277.1	7343207	G	A
NC_040277.1	7387378	T	C
NC_040277.1	7400482	T	C
NC_040277.1	7413421	A	G
NC_040277.1	7452916	T	G
NC_040277.1	7485198	A	G
NC_040277.1	7541175	C	T
NC_040277.1	7590622	A	C
NC_040277.1	7646204	T	C
NC_040277.1	7691949	A	G
NC_040277.1	7721959	T	C
NC_040277.1	7775650	G	C
NC_040277.1	7831389	T	C
NC_040277.1	7875570	T	C
NC_040277.1	7930215	T	C
NC_040277.1	7986355	G	A
NC_040277.1	8040253	C	T
NC_040277.1	8085086	G	A
NC_040277.1	8135706	G	A
NC_040277.1	8184739	T	C
NC_040277.1	8251954	T	C
NC_040277.1	8308229	A	T
NC_040277.1	8369286	C	T

NC_040277.1	8436371	A	G
NC_040277.1	8494460	T	C
NC_040277.1	8528079	A	G
NC_040277.1	8581653	C	T
NC_040277.1	8644163	G	A
NC_040277.1	8713775	T	A
NC_040277.1	8890606	T	A
NC_040277.1	8926275	C	T
NC_040277.1	9064505	T	C
NC_040277.1	9129264	G	A
NC_040277.1	9255476	A	G
NC_040277.1	9314605	C	G
NC_040277.1	9359229	C	A
NC_040277.1	9573843	T	C
NC_040277.1	9633789	T	C
NC_040277.1	9692580	A	T
NC_040277.1	9754138	C	A
NC_040277.1	9800975	A	G
NC_040277.1	9827003	T	G
NC_040277.1	10044722	T	G
NC_040277.1	10092705	C	T
NC_040277.1	10166067	A	G
NC_040277.1	10225383	T	C
NC_040277.1	10279440	G	T
NC_040277.1	10302134	C	T
NC_040277.1	10566395	T	C
NC_040277.1	10614165	G	A
NC_040277.1	10702538	A	G
NC_040277.1	10749110	T	C
NC_040277.1	10800976	C	G
NC_040277.1	10850435	G	A
NC_040277.1	10878836	T	C
NC_040277.1	11016278	A	G
NC_040277.1	11068958	A	C
NC_040277.1	11123350	A	G
NC_040277.1	11185528	G	A
NC_040277.1	11235233	A	G
NC_040277.1	11300761	A	G
NC_040277.1	11342917	T	A
NC_040277.1	11399240	G	T
NC_040277.1	11463981	C	T
NC_040277.1	11512826	A	T
NC_040277.1	11536456	A	T
NC_040277.1	11598697	G	A
NC_040277.1	11657587	G	T
NC_040277.1	11707409	A	G
NC_040277.1	11747201	A	G

NC_040277.1	11808247	G	A
NC_040277.1	11856210	G	A
NC_040277.1	11917855	C	T
NC_040277.1	11949068	A	G
NC_040277.1	12012097	T	C
NC_040277.1	12042068	A	G
NC_040277.1	12108242	T	G
NC_040277.1	12167088	C	T
NC_040277.1	12233473	C	T
NC_040277.1	12292681	C	T
NC_040277.1	12342744	T	C
NC_040277.1	12410455	T	C
NC_040277.1	12557221	T	C
NC_040277.1	12618017	G	A
NC_040277.1	12680619	C	T
NC_040277.1	12722964	A	C
NC_040277.1	12789110	A	G
NC_040277.1	12853017	A	G
NC_040277.1	12910096	G	A
NC_040277.1	12960911	0	G
NC_040277.1	12991520	A	G
NC_040277.1	13148219	G	C
NC_040277.1	13193000	G	C
NC_040277.1	13251080	C	T
NC_040277.1	13307024	A	G
NC_040277.1	13364637	A	G
NC_040277.1	13425444	C	T
NC_040277.1	13482242	A	C
NC_040277.1	13536351	C	T
NC_040277.1	13592866	T	C
NC_040277.1	13639302	A	G
NC_040277.1	13700098	A	G
NC_040277.1	13773960	T	C
NC_040277.1	13840142	C	A
NC_040277.1	13898349	A	G
NC_040277.1	13954379	T	C
NC_040277.1	13996811	T	C
NC_040277.1	14124635	G	A
NC_040277.1	14171444	A	G
NC_040277.1	14230169	G	C
NC_040277.1	14287162	G	T
NC_040277.1	14334849	C	T
NC_040277.1	14396030	T	C
NC_040277.1	14450070	G	A
NC_040277.1	14503580	A	G
NC_040277.1	14542645	G	A
NC_040277.1	14567879	T	C

NC_040277.1	14601545	A	G
NC_040277.1	14605277	G	A
NC_040277.1	14613293	T	C
NC_040277.1	14614389	G	C
NC_040277.1	14674417	G	A
NC_040277.1	14739886	C	G
NC_040277.1	14791556	A	G
NC_040277.1	14848601	G	A
NC_040277.1	14904868	G	C
NC_040277.1	14950020	C	G
NC_040277.1	14985110	A	G
NC_040277.1	14987818	G	A
NC_040277.1	14991020	A	T
NC_040277.1	14991727	G	C
NC_040277.1	15046002	A	G
NC_040277.1	15086446	G	C
NC_040277.1	15087797	A	G
NC_040277.1	15124789	C	T
NC_040277.1	15152104	G	A
NC_040277.1	15295274	C	T
NC_040277.1	15350613	T	G
NC_040277.1	15407694	A	G
NC_040277.1	15463730	T	G
NC_040277.1	15515784	T	A
NC_040277.1	15555927	G	A
NC_040277.1	15556575	T	C
NC_040277.1	15564228	G	A
NC_040277.1	15566596	G	T
NC_040277.1	15568459	C	T
NC_040277.1	15622388	T	C
NC_040277.1	15671833	A	G
NC_040277.1	15728067	G	T
NC_040277.1	15771284	T	A
NC_040277.1	15830866	C	T
NC_040277.1	15890085	A	G
NC_040277.1	15946328	C	T
NC_040277.1	16001949	G	A
NC_040277.1	16056140	C	T
NC_040277.1	16111915	A	G
NC_040277.1	16165256	T	C
NC_040277.1	16232341	G	T
NC_040277.1	16303769	A	G
NC_040277.1	16352928	A	G
NC_040277.1	16403628	T	C
NC_040277.1	16471403	T	C
NC_040277.1	16478410	T	C
NC_040277.1	16534796	C	A

NC_040277.1	16593065	G	C
NC_040277.1	16596096	A	G
NC_040277.1	16632370	T	C
NC_040277.1	16666954	C	T
NC_040277.1	16686819	G	T
NC_040277.1	16698187	T	C
NC_040277.1	16764303	G	A
NC_040277.1	16782257	T	G
NC_040277.1	16837944	T	C
NC_040277.1	16887945	T	G
NC_040277.1	16938934	T	A
NC_040277.1	16996296	T	C
NC_040277.1	17049702	A	G
NC_040277.1	17100551	T	C
NC_040277.1	17154532	A	C
NC_040277.1	17156109	C	T
NC_040277.1	17180861	G	A
NC_040277.1	17223208	T	C
NC_040277.1	17229967	G	A
NC_040277.1	17274175	A	C
NC_040277.1	17316632	G	A
NC_040277.1	17369819	A	G
NC_040277.1	17406029	T	C
NC_040277.1	17416016	A	G
NC_040277.1	17427062	G	A
NC_040277.1	17481511	A	G
NC_040277.1	17539553	T	A
NC_040277.1	17590379	G	A
NC_040277.1	17646090	T	C
NC_040277.1	17705214	T	C
NC_040277.1	17762107	G	A
NC_040277.1	17841164	G	A
NC_040277.1	17894913	T	C
NC_040277.1	17951415	A	G
NC_040277.1	18006139	A	G
NC_040277.1	18066125	C	T
NC_040277.1	18112169	C	T
NC_040277.1	18173159	C	T
NC_040277.1	18206376	C	T
NC_040277.1	18265459	C	G
NC_040277.1	18341515	G	C
NC_040277.1	18396314	C	T
NC_040277.1	18451861	A	C
NC_040277.1	18486019	C	T
NC_040277.1	18542561	C	T
NC_040277.1	18602659	C	T
NC_040277.1	18658872	G	A

NC_040277.1	18713480	T	C
NC_040277.1	18772782	C	T
NC_040277.1	18827145	G	C
NC_040277.1	18877876	C	T
NC_040277.1	18924636	A	G
NC_040277.1	18979111	C	G
NC_040277.1	19298356	G	A
NC_040277.1	19349482	C	A
NC_040277.1	19406401	T	C
NC_040277.1	19444885	A	G
NC_040277.1	19499310	C	T
NC_040277.1	19559988	C	T
NC_040277.1	19752655	G	C
NC_040277.1	19819961	C	G
NC_040277.1	19842084	C	T
NC_040277.1	19917437	G	A
NC_040277.1	19987181	A	T
NC_040277.1	20024645	A	G
NC_040277.1	20069706	T	C
NC_040277.1	20169199	G	T
NC_040277.1	20222171	C	T
NC_040277.1	20275172	C	T
NC_040277.1	20332750	G	C
NC_040277.1	20370169	A	G
NC_040277.1	20596244	C	T
NC_040277.1	20673523	G	A
NC_040277.1	20699263	A	G
NC_040277.1	20745794	C	T
NC_040277.1	20747684	A	C
NC_040277.1	20806663	A	G
NC_040277.1	20863736	C	T
NC_040277.1	20917110	G	A
NC_040277.1	20978210	C	T
NC_040277.1	21042529	C	T
NC_040277.1	21089625	A	G
NC_040277.1	21137986	T	A
NC_040277.1	21211535	G	C
NC_040277.1	21245110	A	G
NC_040277.1	21300074	A	G
NC_040277.1	21351469	A	G
NC_040277.1	21393417	G	A
NC_040277.1	21446920	A	G
NC_040277.1	21482647	G	A
NC_040277.1	21519881	T	C
NC_040277.1	21534059	T	G
NC_040277.1	21540293	T	C
NC_040277.1	21590440	C	T

NC_040277.1	21628479	C	A
NC_040277.1	21682651	C	T
NC_040277.1	21744188	A	G
NC_040277.1	21799612	G	A
NC_040277.1	21865941	A	C
NC_040277.1	21923396	T	C
NC_040277.1	21970615	T	G
NC_040277.1	22026534	A	G
NC_040277.1	22076099	A	G
NC_040277.1	22113110	T	C
NC_040277.1	22154006	G	C
NC_040277.1	22204807	G	T
NC_040277.1	22264128	T	C
NC_040277.1	22293852	G	A
NC_040277.1	22483919	C	A
NC_040277.1	22537813	A	G
NC_040277.1	22601879	G	A
NC_040277.1	22656001	T	C
NC_040277.1	22704937	G	A
NC_040277.1	22759893	C	T
NC_040277.1	22841227	T	G
NC_040277.1	22868216	A	G
NC_040277.1	23085881	C	T
NC_040277.1	23127304	G	T
NC_040277.1	23189484	G	A
NC_040277.1	23243741	T	A
NC_040277.1	23299215	C	T
NC_040277.1	23319308	T	G
NC_040277.1	23528047	G	C
NC_040277.1	23586714	A	T
NC_040277.1	23656555	C	G
NC_040277.1	23704953	A	G
NC_040277.1	23753111	T	C
NC_040277.1	23808396	G	A
NC_040277.1	23851581	A	T
NC_040277.1	23910197	C	A
NC_040277.1	23965579	T	C
NC_040277.1	24008340	T	A
NC_040277.1	24074488	G	A
NC_040277.1	24188864	A	G
NC_040277.1	24367496	T	C
NC_040277.1	24426537	C	T
NC_040277.1	24485495	G	A
NC_040277.1	24552656	C	A
NC_040277.1	24606413	A	G
NC_040277.1	24651015	C	T
NC_040277.1	24704517	C	A

NC_040277.1	24753596	A	G
NC_040277.1	24811403	T	C
NC_040277.1	24869969	C	T
NC_040277.1	24911390	G	C
NC_040277.1	24956080	C	T
NC_040277.1	25046163	T	C
NC_040277.1	25090722	G	A
NC_040277.1	25143995	T	C
NC_040277.1	25164035	G	C
NC_040277.1	25212503	A	G
NC_040277.1	25276736	T	C
NC_040277.1	25331442	C	T
NC_040277.1	25372691	T	C
NC_040277.1	25428177	C	T
NC_040277.1	25475155	A	T
NC_040277.1	25523172	T	C
NC_040277.1	25553413	C	A
NC_040277.1	25596276	A	G
NC_040277.1	25598701	T	C
NC_040277.1	25600855	C	T
NC_040277.1	25671679	A	G
NC_040277.1	25723737	G	A
NC_040277.1	25774188	G	A
NC_040277.1	25813883	A	G
NC_040277.1	25931311	G	T
NC_040277.1	25986622	T	C
NC_040277.1	26042512	T	C
NC_040277.1	26090826	C	A
NC_040277.1	26147793	C	T
NC_040277.1	26202577	G	A
NC_040277.1	26259237	G	A
NC_040277.1	26317060	A	G
NC_040277.1	26364964	C	A
NC_040277.1	26483892	T	C
NC_040277.1	26535732	T	C
NC_040277.1	26593469	G	A
NC_040277.1	26634230	A	G
NC_040277.1	26687598	C	A
NC_040277.1	26719586	A	C
NC_040277.1	26774679	A	G
NC_040277.1	26804456	C	T
NC_040277.1	26860218	T	C
NC_040277.1	26902804	T	C
NC_040277.1	26957204	C	T
NC_040277.1	27013829	A	C
NC_040277.1	27061761	G	T
NC_040277.1	27125914	C	T

NC_040277.1	27183664	C	T
NC_040277.1	27220826	G	C
NC_040277.1	27254851	C	T
NC_040277.1	27255516	C	A
NC_040277.1	27257131	A	G
NC_040277.1	27297391	A	G
NC_040277.1	27451850	A	G
NC_040277.1	27466062	T	C
NC_040277.1	27510787	G	A
NC_040277.1	27570165	T	C
NC_040277.1	27571463	T	C
NC_040277.1	27620958	T	C
NC_040277.1	27676081	T	C
NC_040277.1	27731906	A	G
NC_040277.1	27790229	C	G
NC_040277.1	27855058	C	T
NC_040277.1	27914995	A	G
NC_040277.1	27970073	T	C
NC_040277.1	28023077	A	G
NC_040277.1	28048794	T	A
NC_040277.1	28103144	G	T
NC_040277.1	28162034	A	T
NC_040277.1	28215718	T	C
NC_040277.1	28276397	A	G
NC_040277.1	28331882	A	G
NC_040277.1	28386040	T	C
NC_040277.1	28444948	C	A
NC_040277.1	28496607	A	G
NC_040277.1	28549513	C	G
NC_040277.1	28596375	G	A
NC_040277.1	28647426	A	G
NC_040277.1	28698934	T	C
NC_040277.1	28754617	G	T
NC_040277.1	28808803	G	A
NC_040277.1	28864153	A	C
NC_040277.1	28918543	T	C
NC_040277.1	28973002	G	A
NC_040277.1	29022232	C	T
NC_040277.1	29074481	T	C
NC_040277.1	29115498	A	G
NC_040277.1	29157715	A	G
NC_040277.1	29210811	C	T
NC_040277.1	29269362	C	T
NC_040277.1	29325853	C	T
NC_040277.1	29382834	T	C
NC_040277.1	29436958	A	G
NC_040277.1	29494088	T	C

NC_040277.1	29548428	A	G
NC_040277.1	29604126	G	A
NC_040277.1	29652756	T	C
NC_040277.1	29710078	C	T
NC_040277.1	29751972	G	C
NC_040277.1	29805212	A	G
NC_040277.1	29863822	C	A
NC_040277.1	29919366	A	T
NC_040277.1	29969377	G	A
NC_040277.1	30012964	C	G
NC_040277.1	30040122	A	G
NC_040277.1	30098295	C	T
NC_040277.1	30147287	A	G
NC_040277.1	30202498	T	G
NC_040277.1	30233706	G	A
NC_040277.1	30291035	T	G
NC_040277.1	30343530	C	T
NC_040277.1	30396964	G	A
NC_040277.1	30453154	G	A
NC_040277.1	30506582	C	T
NC_040277.1	30558309	T	G
NC_040277.1	30615039	G	A
NC_040277.1	30669420	G	A
NC_040277.1	30717546	A	T
NC_040277.1	30775871	A	G
NC_040277.1	30828760	A	G
NC_040277.1	30900220	C	T
NC_040277.1	31005743	G	A
NC_040277.1	31050442	T	C
NC_040277.1	31079162	T	C
NC_040277.1	31083259	T	C
NC_040277.1	31085597	G	T
NC_040277.1	31087373	G	A
NC_040277.1	31143507	C	A
NC_040277.1	31200029	C	T
NC_040277.1	31250819	C	T
NC_040277.1	31312929	C	T
NC_040277.1	31355955	G	A
NC_040277.1	31405295	C	T
NC_040277.1	31459012	C	T
NC_040277.1	31515639	G	C
NC_040277.1	31546516	T	C
NC_040277.1	31589325	G	T
NC_040277.1	31641600	T	C
NC_040277.1	31645878	G	T
NC_040277.1	31646410	G	C
NC_040277.1	31686788	A	G

NC_040277.1	31730224	T	C
NC_040277.1	31789901	A	C
NC_040277.1	31843818	A	G
NC_040277.1	31902588	A	G
NC_040277.1	31964178	T	C
NC_040277.1	32007102	A	T
NC_040277.1	32090760	G	C
NC_040277.1	32162708	T	G
NC_040277.1	32224857	C	A
NC_040277.1	32279640	A	G
NC_040277.1	32340878	T	C
NC_040277.1	32389265	A	G
NC_040277.1	32711355	C	T
NC_040277.1	32768117	C	G
NC_040277.1	32823042	T	C
NC_040277.1	32874276	A	G
NC_040277.1	32932525	A	G
NC_040277.1	32994932	C	T
NC_040277.1	33048152	C	T
NC_040277.1	33106728	T	C
NC_040277.1	33160735	T	C
NC_040277.1	33216754	A	G
NC_040277.1	33274262	G	A
NC_040277.1	33332385	C	T
NC_040277.1	33387882	A	G
NC_040277.1	33441201	T	C
NC_040277.1	33498604	C	T
NC_040277.1	33556872	T	C
NC_040277.1	33591114	T	G
NC_040277.1	33643504	G	A
NC_040277.1	33701792	T	C
NC_040277.1	33762221	C	T
NC_040277.1	33822664	T	C
NC_040277.1	33879550	A	G
NC_040277.1	33942266	G	A
NC_040277.1	34094097	G	C
NC_040277.1	34158450	T	G
NC_040277.1	34161725	G	A
NC_040277.1	34213268	G	T
NC_040277.1	34244123	T	C
NC_040277.1	34295799	G	A
NC_040277.1	34355807	T	C
NC_040277.1	34411499	C	G
NC_040277.1	34454262	A	C
NC_040277.1	34514092	A	G
NC_040277.1	34563659	G	A
NC_040277.1	34620664	C	T

NC_040277.1	34685349	A	T
NC_040277.1	34739363	G	A
NC_040277.1	34786815	C	T
NC_040277.1	34828250	A	C
NC_040277.1	34866192	A	C
NC_040277.1	34912164	T	C
NC_040277.1	34951016	G	C
NC_040277.1	34968196	G	A
NC_040277.1	35021702	T	C
NC_040277.1	35079002	G	A
NC_040277.1	35128964	C	G
NC_040277.1	35197932	T	G
NC_040277.1	35260470	T	C
NC_040277.1	35316175	A	G
NC_040277.1	35371727	T	C
NC_040277.1	35423973	T	C
NC_040277.1	35477951	G	A
NC_040277.1	35536481	T	G
NC_040277.1	35589647	A	G
NC_040277.1	35636419	T	C
NC_040277.1	35690261	G	C
NC_040277.1	35919679	G	A
NC_040277.1	35956813	A	G
NC_040277.1	35958658	T	G
NC_040277.1	35992883	A	G
NC_040277.1	35994369	A	G
NC_040277.1	36036786	T	C
NC_040277.1	36076447	C	G
NC_040277.1	36080901	C	G
NC_040277.1	36081864	T	G
NC_040277.1	36094385	T	C
NC_040277.1	36095284	T	C
NC_040277.1	36132708	T	G
NC_040277.1	36194601	T	C
NC_040277.1	36241444	C	A
NC_040277.1	36278260	A	G
NC_040277.1	36291706	T	C
NC_040277.1	36354432	T	C
NC_040277.1	36370555	T	C
NC_040277.1	36393867	T	G
NC_040277.1	36397599	T	C
NC_040277.1	36416226	T	C
NC_040277.1	36441340	T	C
NC_040277.1	36450131	G	A
NC_040277.1	36469623	T	C
NC_040277.1	36509762	T	G
NC_040277.1	36527885	C	A

NC_040277.1	36577379	0	C
NC_040277.1	36635228	T	C
NC_040277.1	36701905	C	T
NC_040277.1	36757918	G	T
NC_040277.1	36816602	C	T
NC_040277.1	36877053	A	G
NC_040277.1	36906325	G	C
NC_040277.1	36933957	G	A
NC_040277.1	36934982	G	C
NC_040277.1	36937240	T	G
NC_040277.1	36993467	C	T
NC_040277.1	37036396	T	C
NC_040277.1	37073513	G	A
NC_040277.1	37075602	T	C
NC_040277.1	37130647	G	A
NC_040277.1	37183056	T	C
NC_040277.1	37233693	T	C
NC_040277.1	37285393	A	G
NC_040277.1	37326243	C	T
NC_040277.1	37364880	G	C
NC_040277.1	37399406	T	G
NC_040277.1	37525551	T	C
NC_040277.1	37583149	C	A
NC_040277.1	37646236	C	T
NC_040277.1	37813571	T	C
NC_040277.1	37868246	G	A
NC_040277.1	37988242	C	T
NC_040277.1	38093201	A	G
NC_040277.1	38179278	T	C
NC_040277.1	38237619	G	T
NC_040277.1	38302106	A	G
NC_040277.1	38358670	G	A
NC_040277.1	38412045	G	A
NC_040277.1	38464412	T	G
NC_040277.1	38528888	T	C
NC_040277.1	38575630	A	T
NC_040277.1	38626130	C	A
NC_040277.1	38681929	C	A
NC_040277.1	38745870	T	C
NC_040277.1	38798468	A	G
NC_040277.1	38849928	T	C
NC_040277.1	38901305	T	A
NC_040277.1	38944130	T	A
NC_040277.1	39000006	T	C
NC_040277.1	39056207	A	G
NC_040277.1	39114158	T	A
NC_040277.1	39154352	T	G

NC_040277.1	39198055	A	G
NC_040277.1	39298841	C	T
NC_040277.1	39354217	T	C
NC_040277.1	39409005	A	G
NC_040277.1	39462303	G	A
NC_040277.1	39522309	A	G
NC_040277.1	39583329	C	T
NC_040277.1	39583829	G	A
NC_040277.1	39641133	T	C
NC_040277.1	39697360	T	C
NC_040277.1	39748366	A	G
NC_040277.1	39803298	T	C
NC_040277.1	39863323	C	A
NC_040277.1	39893788	C	G
NC_040277.1	39946648	T	C
NC_040277.1	39978993	A	G
NC_040277.1	39993123	G	A
NC_040277.1	40047581	T	C
NC_040277.1	40074728	A	G
NC_040277.1	40142159	T	C
NC_040277.1	40212837	A	G
NC_040277.1	40268372	T	A
NC_040277.1	40311914	C	G
NC_040277.1	40356438	A	G
NC_040277.1	40401579	A	G
NC_040277.1	40409300	T	C
NC_040277.1	40466923	G	T
NC_040277.1	40500241	T	C
NC_040277.1	40531881	T	C
NC_040277.1	40533643	A	G
NC_040277.1	40587002	T	C
NC_040277.1	40633202	T	C
NC_040277.1	40662961	A	G
NC_040277.1	40687492	T	C
NC_040277.1	40718986	G	A
NC_040277.1	40720718	A	G
NC_040277.1	40770759	C	T
NC_040277.1	40825960	T	C
NC_040277.1	40883076	G	C
NC_040277.1	40927360	G	A
NC_040277.1	40982751	T	C
NC_040277.1	41039286	G	A
NC_040277.1	41041046	G	T
NC_040277.1	41041624	C	A
NC_040277.1	41044267	G	A
NC_040277.1	41044570	G	A
NC_040277.1	41044819	G	A

NC_040277.1	41045053	T	A
NC_040277.1	41046846	A	G
NC_040277.1	41047020	A	G
NC_040277.1	41102256	G	T
NC_040277.1	41154859	T	G
NC_040277.1	41176345	G	A
NC_040277.1	41230211	G	A
NC_040277.1	41283379	T	C
NC_040277.1	41337585	A	G
NC_040277.1	41393880	G	C
NC_040277.1	41451231	G	C
NC_040277.1	41505201	T	C
NC_040277.1	41568703	A	G
NC_040277.1	41602185	C	T
NC_040277.1	41660072	T	C
NC_040277.1	41713117	A	G
NC_040277.1	41771095	T	C
NC_040277.1	41823501	A	C
NC_040277.1	41867415	G	C
NC_040277.1	41900233	G	A
NC_040277.1	42022826	C	T
NC_040277.1	42070116	T	C
NC_040277.1	42115375	T	C
NC_040277.1	42176943	A	G
NC_040277.1	42229661	A	G
NC_040277.1	42295876	G	T
NC_040277.1	42355204	A	G
NC_040277.1	42394497	G	T
NC_040277.1	42439490	T	C
NC_040277.1	42447115	T	G
NC_040277.1	42498399	A	G
NC_040277.1	42551350	C	T
NC_040277.1	42604510	T	C
NC_040277.1	42661315	G	A
NC_040277.1	42716266	A	G
NC_040277.1	42741486	G	A
NC_040277.1	42741727	T	C
NC_040277.1	42806118	G	C
NC_040277.1	42856508	G	A
NC_040277.1	42908623	G	T
NC_040277.1	42963451	A	T
NC_040277.1	43020615	A	G
NC_040277.1	43076551	T	C
NC_040277.1	43132911	T	C
NC_040277.1	43190276	G	A
NC_040277.1	43243906	A	T
NC_040277.1	43299844	C	T

NC_040277.1	43340748	C	T
NC_040277.1	43395229	T	C
NC_040277.1	43442495	A	G
NC_040277.1	43456553	T	C
NC_040277.1	43456762	T	C
NC_040277.1	43502933	C	T
NC_040277.1	43508779	A	G
NC_040277.1	43515744	T	A
NC_040277.1	43570467	T	C
NC_040277.1	43595415	T	C
NC_040277.1	43598706	T	C
NC_040277.1	43623698	A	G
NC_040277.1	43626075	A	T
NC_040277.1	43635503	A	G
NC_040277.1	43636064	G	A
NC_040277.1	43638953	A	G
NC_040277.1	43703017	G	A
NC_040277.1	43751910	T	C
NC_040277.1	43810235	A	G
NC_040277.1	43867776	A	C
NC_040277.1	43922806	C	A
NC_040277.1	43978159	A	G
NC_040277.1	44035091	C	T
NC_040277.1	44091945	T	G
NC_040277.1	44147541	C	G
NC_040277.1	44199288	G	T
NC_040277.1	44255253	A	G
NC_040277.1	44300449	T	G
NC_040277.1	44361784	G	A
NC_040277.1	44420494	G	A
NC_040277.1	44475397	A	C
NC_040277.1	44536992	A	G
NC_040277.1	44594467	A	G
NC_040277.1	44653672	A	T
NC_040277.1	44708618	T	C
NC_040277.1	44750360	A	G
NC_040277.1	44800986	A	C
NC_040277.1	44857659	T	C
NC_040277.1	44911180	C	A
NC_040277.1	44969925	T	C
NC_040277.1	45027657	G	A
NC_040277.1	45074474	A	G
NC_040277.1	45118706	T	C
NC_040277.1	45219032	C	T
NC_040277.1	45271654	C	T
NC_040277.1	45322892	C	T
NC_040277.1	45377412	G	A

NC_040277.1	45447149	G	A
NC_040277.1	45569957	C	T
NC_040277.1	45582891	C	T
NC_040277.1	45583686	A	C
NC_040277.1	45583928	C	T
NC_040277.1	45584490	G	T
NC_040277.1	45585439	C	G
NC_040277.1	45602490	T	C
NC_040277.1	45653906	G	A
NC_040277.1	45708227	T	C
NC_040277.1	45768159	C	T
NC_040277.1	45831923	C	T
NC_040277.1	45880303	C	T
NC_040277.1	45934284	A	G
NC_040277.1	45987172	T	C
NC_040277.1	46026887	T	C
NC_040277.1	46083271	A	G
NC_040277.1	46129465	A	C
NC_040277.1	46190544	A	G
NC_040277.1	46246935	T	C
NC_040277.1	46314728	T	C
NC_040277.1	46374441	T	C
NC_040277.1	46429531	C	A
NC_040277.1	46483648	A	T
NC_040277.1	46539719	C	G
NC_040277.1	46588214	G	A
NC_040277.1	46628662	G	A
NC_040277.1	46713177	A	G
NC_040277.1	46745094	A	G
NC_040277.1	46871755	A	G
NC_040277.1	46921535	A	G
NC_040277.1	46990717	G	A
NC_040277.1	47055661	T	C
NC_040277.1	47110995	G	T
NC_040277.1	47149701	G	A
NC_040277.1	47208157	A	G
NC_040277.1	47260093	C	T
NC_040277.1	47312770	A	G
NC_040277.1	47375072	T	C
NC_040277.1	47408202	G	A
NC_040277.1	47463516	G	A
NC_040277.1	47511986	T	A
NC_040277.1	47549652	G	A
NC_040277.1	47619186	A	G
NC_040277.1	47674174	T	C
NC_040277.1	47674415	G	T
NC_040277.1	47725380	C	A

NC_040277.1	47782475	A	G
NC_040277.1	47827307	T	C
NC_040277.1	47891369	G	C
NC_040277.1	47954531	A	G
NC_040277.1	48038922	A	G
NC_040277.1	48039174	A	G
NC_040277.1	48039893	T	C
NC_040277.1	48072425	A	G
NC_040277.1	48129377	G	C
NC_040277.1	48184086	C	T
NC_040277.1	48239572	T	C
NC_040277.1	48289343	T	C
NC_040277.1	48333777	T	C
NC_040277.1	48383243	T	C
NC_040277.1	48425563	T	C
NC_040277.1	48477215	A	T
NC_040277.1	48527027	G	A
NC_040277.1	48581803	T	C
NC_040277.1	48620416	G	C
NC_040277.1	48695267	A	T
NC_040277.1	48748860	C	T
NC_040277.1	48806590	G	C
NC_040277.1	48861253	T	C
NC_040277.1	48915882	A	C
NC_040277.1	48971537	G	T
NC_040277.1	49013208	T	C
NC_040277.1	49069801	T	C
NC_040277.1	49120949	C	T
NC_040277.1	49278303	C	A

- Position; REF - reference allele; ALT - alternative allele
