# Supplementary material for: Genome-wide association study identifies genetic variants underlying footrot in Portuguese Merino sheep
Source: BMC Genomics. 2024 Jan 23;25:100. doi: 10.1186/s12864-023-09844-x (PMC10804546; doi:10.1186/s12864-023-09844-x)
Supplement: Supplementary file 2 — Additional file 2: Table S1. Index footrot score weighting factors per individual score. Table S2. Details on farm locations, breeds and number of sampled animals per farm. Breed acronyms are as follows: Merino Branco -MB; Merino Preto -MP; and crossbreds –CR. Table S3. Genomic location of the SNPs used for genotyping and respective alleles. Chr - Chromosome; Pos. Table S4. Descriptive statistics for footrot score. Number of analysed animals (N), minimum (Min) and maximum (Max) footrot score, mean and standard deviation (SD). Table S5. Functional annotation of the candidate genes identified in the GWAS. Distribution of KEGG pathways and gene ontology categories are depicted, namely the cellular component, biological process, and molecular function. [file 12864_2023_9844_MOESM2_ESM.zip › Table_S4.pdf]

Table S4: Descriptive statistics for footrot score. Number of analysed animals (N), minimum (Min) and maximum (Max) footrot score, mean and standard deviation (SD).

Breed	N	Index footrot score				Global footrot score				Highest footrot score			
		Min	Max	Mean	SD	Min	Max	Mean	SD	Min	Max	Mean	SD
Merino Branco	334	0	30	2.85	4.38	0	12	1.81	2.07	0	4	0.95	0.95
Merino Preto	138	0	7.5	0.31	1.04	0	4	0.20	0.60	0	3	0.17	0.50
Crossbreds	903	0	39	3.28	5.64	0	14	1.73	2.46	0	5	0.98	1.15
Total	1375	0	39	2.87	5.14	0	14	1.60	2.30	0	5	0.89	1.09
