# Supplementary material for: Genome-wide association study identifies genetic variants underlying footrot in Portuguese Merino sheep
Source: BMC Genomics. 2024 Jan 23;25:100. doi: 10.1186/s12864-023-09844-x (PMC10804546; doi:10.1186/s12864-023-09844-x)
Supplement: Supplementary file 2 — Additional file 2: Table S1. Index footrot score weighting factors per individual score. Table S2. Details on farm locations, breeds and number of sampled animals per farm. Breed acronyms are as follows: Merino Branco -MB; Merino Preto -MP; and crossbreds –CR. Table S3. Genomic location of the SNPs used for genotyping and respective alleles. Chr - Chromosome; Pos. Table S4. Descriptive statistics for footrot score. Number of analysed animals (N), minimum (Min) and maximum (Max) footrot score, mean and standard deviation (SD). Table S5. Functional annotation of the candidate genes identified in the GWAS. Distribution of KEGG pathways and gene ontology categories are depicted, namely the cellular component, biological process, and molecular function. [file 12864_2023_9844_MOESM2_ESM.zip › Table_S5.pdf]

Table S5: Functional annotation of the candidate genes identified in the GWAS. Distribution of KEGG pathways and gene ontology categories are depicted, namely the cellular component, biological process, and molecular function.

Gene ID	Gene name	EC number	KEGG Pathways	Cellular Component	Biological Process	Molecular Function
SMG1	SMG1 nonsense mediated mRNA decay associated PI3K related kinase	2.7.11.1	mRNA surveillance pathway	-	Nonsense-mediated mRNA decay	Kinase, Serine/threonine-protein kinase, Transferase,
HSPG2	Heparan sulfate proteoglycan 2	-	ECM-receptor interaction, Hepatitis B, Proteoglycans in cancer	Basement membrane, Extracellular matrix, Secreted	-	-
RALYL	RALY RNA binding protein like	-	-	-	-	-
PCLO	Piccolo presynaptic cytomatrix protein	-	Insulin secretion	Cell projection	-	-
THBS1	Thrombospondin 1	-	Rap1 signaling pathway, p53 signaling pathway, Phagosome, PI3K-Akt signaling pathway, TGF-beta signaling pathway, Focal adhesion, ECM-receptor interaction, Malaria, Human papillomavirus infection, Proteoglycans in cancer, MicroRNAs in cancer, Bladder cancer	-	Cell adhesion	Heparin-binding
